# SIRT1 plays a critical role in maintaining the viability of Yak Sertoli cells by regulating mitochondrial biogenesis via activating the PGC-1α-NRF-1-TFAM pathway

**DOI:** 10.5713/ab.251005

**Published:** 2026-04-16

**Authors:** Dongju Liu, Xiaodong Dong, Huai Zhang, Wending Zhou, Gan Yang, Xianrong Xiong, Yan Xiong, Jian Li, Daoliang Lan, Shi Yin

**Affiliations:** 1 College of Animal and Veterinary Sciences, Southwest Minzu University, Chengdu, China; 2 Key Laboratory of Animal Science of National Ethnic Affairs Commission of China, Southwest Minzu University, Chengdu, China

**Keywords:** Mitochondria, PGC-1α-NRF-1-TFAM Pathway, Sertoli Cells, *SIRT1*, Yak

## Abstract

**Objective:**

Sertoli cells are somatic cells located within the seminiferous tubules, critical for spermatogenesis through various mechanisms, such as paracrine signaling and the formation of the blood-testis barrier. Sirtuin 1 (SIRT1), a member of the evolutionarily conserved sirtuin family, is an NAD^+^-dependent class III histone deacetylase. The involvement of SIRT1 has been documented in multiple key biological processes; however, its role in Sertoli cells remains unknown.

**Methods:**

This study involved isolation of yak Sertoli cells to examine the impact of SIRT1 on cell viability and its related regulatory mechanisms using RNA interference (RNAi).

**Results:**

The findings revealed that *SIRT1* knockdown significantly impaired the viability and function of yak Sertoli cells. Transcriptome sequencing indicated a significant impact on mitochondrial structure following *SIRT1* knockdown. Subsequent studies demonstrated that knockdown of *SIRT1* in yak Sertoli cells led to significant downregulation of genes related to mitochondrial morphology, a reduction in membrane potential, decreased expression of mitochondrial genes, and a diminished capacity for ATP synthesis. The PGC-1α-NRF-1-TFAM pathway, a key signaling cascade in mitochondrial biogenesis, was inhibited after *SIRT1* knockdown. The overexpression of *PPARGC1A* in *SIRT1*-knockdown yak Sertoli cells partially rescued the reduction in cell viability and the impairment of mitochondrial biogenesis. These findings indicate that SIRT1 regulates mitochondrial biogenesis in yak Sertoli cells through the activation of the PGC-1α-NRF-1-TFAM signaling pathway, thereby maintaining cellular viability.

**Conclusion:**

The present study elucidates the regulatory role and mechanism of SIRT1 in yak Sertoli cells, providing fundamental data and new insights for further research on the function of SIRT1 in reproductive regulation in yaks.

## INTRODUCTION

Sertoli cells (SCs) are the only somatic cells within the seminiferous epithelium of the mammalian testis that directly interact with germ cells [1]. They play a central role in supporting, nourishing, and protecting spermatogenic cells through multiple means. Studies have shown that SC-secreted factors, such as platelet-derived growth factor D (PDGFD), glial cell line-derived neurotrophic factor (GDNF), and stem cell factor (SCF), are critical for regulating the viability and differentiation of spermatogonia, as well as maintaining the self-renewal capacity of spermatogonial stem cells [2,3]. Furthermore, tight junctions formed between adjacent SCs, as well as those between SCs and germ cells, constitute the blood-testis barrier (BTB). This barrier establishes an immune-privileged microenvironment within the seminiferous tubules. The BTB prevents the entry of harmful substances and helps maintain a stable internal environment necessary for normal spermatogenesis [4,5]. SCs facilitate the movement of progressively maturing spermatogenic cells toward the lumen through the contraction of their microtubules and microfilaments, as well as by secreting testicular fluid [6]. Substances secreted in testicular fluid, such as lactic acid, serve as important energy sources for spermatogenic cells [7]. Besides, SCs participate in the hormonal regulation of spermatogenesis by secreting androgen-binding protein (ABP), activins, inhibins, among other factors [8]. Beyond these roles, SCs are also capable of phagocytizing dead spermatogenic cells and the cytoplasmic residues shed during sperm maturation [9]. However, although SCs play a critical role in spermatogenesis, the molecular mechanisms regulating their fate and function remain incompletely understood.

Sirtuin 1 (SIRT1), a member of the evolutionarily conserved sirtuin family, is an NAD^+^-dependent class III histone deacetylase. Current evidence suggests that SIRT1 catalyzes the removal of acetyl groups from lysine residues on histones, including H3 and H4, thereby facilitating chromatin condensation and transcriptional repression of specific genes [10]. SIRT1 plays a multifaceted role in critical cellular processes such as aging regulation, DNA repair, anti-inflammatory responses, and metabolic homeostasis. For instance, elevated SIRT1 expression extends lifespan in model organisms, including yeast, *Caenorhabditis elegans*, and mice, by modulating signaling pathways such as nuclear factor kappa-B (NF-κB), AMP-activated protein kinase (AMPK), and mechanistic target of rapamycin (mTOR) [11]. Inhibition of SIRT1 in human embryonic stem cells leads to activation of p53 and reduction in DNA repair enzyme levels, impairing genomic integrity [12]. Furthermore, SIRT1 protein expression is downregulated by IL-1β/NF-κB signaling during acetaminophen-induced hepatotoxicity, exacerbating inflammatory responses [13]. Moreover, it has been reported that SIRT1-mediated deacetylation of cAMP response element-binding protein (CREB) is essential for maintaining the balance between glucose and lipid metabolism [14].

Studies have demonstrated that SIRT1 is essential for maintaining fertility in both males and females. It has been found that *SIRT1*-knockout female mice exhibit a thin-walled uterus and small ovaries, with follicles present but no corpora lutea [15]. In males, global knockout of *SIRT1* leads to extensive apoptosis of pachytene spermatocytes, aberrant maturation of Leydig and SCs, and a pronounced reduction in intratesticular testosterone levels [16]. Consistent with the detrimental role of excessive apoptosis in reproductive success, a recent study in bovine IVF embryos reported that increased apoptosis in late-developing blastocysts was significantly correlated with lower pregnancy rates [17]. Moreover, germ cell-specific deletion of *SIRT1* results in smaller testes, delayed differentiation of pre-meiotic germ cells, reduced sperm count, an increased frequency of abnormal sperm morphology, and subfertility in mice [18]. Given that systemic *SIRT1* deficiency has a more severe impact on male fertility compared to germ cell-specific knockout, further investigation is necessary to elucidate the functional role of SIRT1 in SCs.

The yak (*Bos grunniens*) is a unique livestock species native to the Tibetan Plateau, constituting a vital means of production for local herders. However, yaks are characterized by late sexual maturity, low sperm motility, an extended sperm maturation cycle, and overall low reproductive efficiency [19]. Therefore, an in-depth investigation into yak reproductive mechanisms is essential for improving livestock productivity in high-altitude regions. In this study, we hypothesize that SIRT1 plays a critical role in regulating the viability and function of yak SCs. To validate this hypothesis, *SIRT1* expression was inhibited in yak SCs and its effects were evaluated on cellular viability and function, and the underlying molecular mechanisms were further elucidated. Importantly, this study fills the research gap regarding SIRT1 in SCs, providing a theoretical foundation for further investigation into the role of SIRT1 in yak reproductive regulation, particularly in spermatogenesis. It also offers a potential molecular target for improving and enhancing the reproductive performance of yaks.

## MATERIALS AND METHODS

### Isolation and culture of yak Sertoli cells

Testicles were collected from male yaks (3 and 4 years old, with mean body weights of 280±15 kg) at a slaughterhouse in Qingbaijiang District, Chengdu, Sichuan Province, for the isolation and primary culture of SCs. Under sterile conditions, the testes were decapsulated, and SCs were isolated using a two-step enzymatic digestion protocol. The resulting cell pellet was resuspended in 1 mL of DMEM/F12 medium (Procell) supplemented with 10% fetal bovine serum (Procell) to generate a single-cell suspension. This suspension was seeded into culture flasks and maintained at 37°C in a 5% CO_2_ incubator for subsequent experiments. Three independent samples from the same group were treated as biological replicates. The third-passage cells were used for subsequent experiments.

### Immunofluorescence staining

A suspension of SCs at a density of 5×10^5^ cells/mL was seeded into 12-well plates and cultured for 48 h under the same conditions. The cells were then fixed with 4% paraformaldehyde (Biosharp) for 30 min and washed three times with PBS. Next, cells on coverslips were permeabilized with 0.1% Triton X-100 (Biofroxx) for 10 min. After washing, blocking was performed using PBS containing 1% bovine serum albumin (Solarbio) for 45 min. The cells were subsequently incubated overnight at 4°C with primary antibodies against WT1 or SOX9. Following three washes with PBS, 500 μL of FITC-conjugated goat anti-rabbit IgG was added to each well and incubated for 1 h at room temperature in the dark. After additional washes, the cells were counterstained with DAPI (Abcam). Imaging was conducted using a fluorescence microscope (Zeiss) for subsequent analysis. Each of the three biological replicates included three technical replicates. The details of antibodies are listed in Supplement 1.

### RNA interference

The cells were seeded in well plates the day before transfection, and when the degree of cell confluence reached 70%–80%. The third-generation yak SCs were transfected with 20 nM of SIRT1-specific small interfering RNA (RNAi group), with a sense strand sequence of 5′-GAGACUGUGACGU AAUUAUTT-3′ and an antisense strand sequence of 5′ - AUAAUUACGUCACAGUCUCTT-3′, along with a non-targeting negative control (NC group) with a sense strand sequence of 5′-UUCUCCGAACGUGUCACGUTT-3′ and an antisense strand sequence of 5′-ACGUGACACGUUCGG AGAATT-3′ (GenePharma) using Lipofectamine RNAiMAX Transfection Reagent (Invitrogen) according to the manufacturer**’**s instructions. Following 4–6 h of transfection, the cells were washed with PBS to remove residual transfection complexes. Fresh complete medium was then added, and the cells were cultured for another 48 h. Subsequently, both cells and culture supernatant were collected for further analysis.

### RNA extraction, cDNA synthesis, and real-time polymerase chain reaction

Cells were initially seeded in 6-well plates. At 48 h post-transfection, the culture medium was aspirated, and the cells were washed three times with PBS. Total RNA was extracted from yak SCs using 1 mL of TRIzol reagent (Invitrogen) per well. RNA purity was determined by measuring the absorbance ratio at 260/280 nm using a UV-Vis spectrophotometer (BioSpec-nano; Shimadzu). Subsequently, cDNA synthesis was conducted according to the manufacturer’s instructions using HiScript III RT SuperMix for quantitative polymerase chain reaction (qPCR) (+gDNA wiper) (Vazyme). Primers for each gene were designed using the NCBI database and synthesized by Tsingke Biotechnology. All primer sequences used are provided in Supplement 2. Quantitative real-time PCR was performed in a 20 μL reaction mixture comprising 10 μL of ChamQ Universal SYBR qPCR Master Mix (Vazyme), 1 μL of cDNA, 0.5 μL each of forward and reverse primers (10 μM), and 8 μL of nuclease-free ddH_2_O then was performed on a LightCycler 96 instrument (Roche). The amplification protocol included an initial denaturation at 95°C for 1 min, followed by 40 cycles of denaturation at 95°C for 10 s and annealing/extension at 60°C for 30 s. *GAPDH* was used as the reference gene, and relative gene expression levels were quantified using the comparative CT (2^−ΔΔCT^) method.

### Western blot

Cells were seeded in 6-well plates at a density of 2×10^5^ cells/mL. At 48 h after transfection, the old medium was aspirated, and the cells were washed twice with ice-cold PBS for 5 min each. A lysis buffer was prepared by combining RIPA Lysis Buffer (Solarbio) with PMSF (Solarbio) at a 100:1 ratio. Then, 100 μL of the lysis buffer was added to each well, and the cells were lysed on ice for 30 min. The lysates were transferred to centrifuge tubes and incubated on ice for another 20 min, followed by centrifugation at 12,000×g for 5 min at 4°C. The resulting supernatant was collected for subsequent analysis. Protein concentration was determined using a BCA Protein Assay Kit (Solarbio) according to the manufacturer’s instructions. Western blot analysis was conducted as described previously [20]. Following separation via 12.5% SDS-PAGE (20 μg protein per lane), the proteins were transferred to a PVDF membrane. The membrane was then blocked with 5% skim milk in TBST buffer for 2 h at room temperature. Subsequently, the membrane was incubated with the specific primary antibody at 4°C overnight, followed by incubation with an HRP-conjugated secondary antibody for 2 h at room temperature, with GAPDH serving as the internal control. The grayscale values of protein bands were quantified using ImageJ software (National Institutes of Health, ver. 1.8.0). More details on the antibodies used are listed in Supplement 1.

### Cell counting kit-8 assay

SCs were seeded into 96-well plates at a density of 1×10^4^ cells per well. At 48 h after transfection, cell viability was evaluated using a CCK-8 assay kit (Beyotime) according to the manufacturer’s instructions. The optical density (OD) at 450 nm was measured with a microplate reader (Thermo Fisher Scientific), and cell viability was calculated as per the manufacturer’s protocol.

### Flow cytometry assay

Cells were seeded in 6-well plates at a density of 2×10^5^ cells/mL. At 48 h post-transfection, the cells were digested with EDTA-free trypsin (Biosharp) for 30 s, followed by termination of digestion through the addition of complete medium. The cell suspension was collected and centrifuged at 250 g for 5 min. After aspiration of the supernatant, the cell pellet was washed twice with PBS, followed by centrifugation at 250 g for 5 min and removal of the supernatant to obtain the final cell pellet. According to the manufacturer’s instructions of the Annexin V-APC/PI Apoptosis Detection Kit (KeyGEN BioTECH), the cells were resuspended in 500 μL of Binding Buffer. Then, 5 μL of Annexin V-APC was added and mixed gently, followed by the addition of 5 μL of propidium iodide (PI) with thorough mixing. The samples were incubated at room temperature for 10 min in the dark. Apoptosis analysis was performed using a flow cytometer (Beckman Coulter), and the flow cytometry data were analyzed with CytExpert software (ver. 2.3).

### *In vitro* blood-testis barrier assay

SCs were seeded into 24-well Transwell inserts at a density of 5×10^4^ cells per well and allowed to adhere for 12 h before transfection. At 48 h after transfection, the culture medium was aspirated and replaced both inside and outside the inserts with Hank’s Balanced Salt Solution (HBSS; Procell, PB180323). The inner chamber contained HBSS supplemented with 10 μg/mL sodium fluorescein (Beyotime). The plate was incubated at 37°C for 1 h. Subsequently, the inserts were carefully removed, and the solution in each lower chamber was mixed thoroughly by pipetting. A 10 μL aliquot of this solution was diluted with 90 μL of HBSS. The diluted sample was transferred to a 96-well plate under light-protected conditions. Fluorescence intensity was measured using a microplate reader with excitation and emission wavelengths set at 460 nm and 515 nm, respectively. The results were normalized to the fluorescence intensity of the NC group, which was assigned a value of 1.

### RNA-sequencing

The cDNA libraries were sequenced on the Illumina/T7 sequencing platform by Verygenome Technology. After the library construction is completed, initial quantification is performed using NanoDrop. The library was then diluted to 1 ng/μL, and the insert size was detected using the Agilent 2100 system. Libraries meeting the expected insert size criteria were then quantified via real-time PCR on a Bio-Rad CFX96 system using the iQ SYBR Green Supermix to determine the effective concentration, ensuring a value greater than 10 nM. Libraries that passed the quality control were sequenced on a BGI T7 platform with a PE150 sequencing strategy. Gene expression levels were estimated using FPKM (Fragments per Kilobase per Million Mapped Fragments), and differential gene expression analysis was conducted with DESeq2. The screening of differentially expressed (DE) genes primarily relies on the fold change value and the q-value (*P*_adj_ value, adjusted p-value) as key indicators. Genes with |log2Foldchange| ≥1 and q<0.05 are typically identified as significantly DE genes. All DE genes were mapped to various terms in the Gene Ontology database (http://www.geneontology.org/), and the number of genes per term was calculated. Hypergeometric tests were then applied to identify GO terms that were significantly enriched in the DE genes compared to the background of identified genes. Gene set enrichment analysis (GSEA) was further performed on GO entries using the ClusterProfiler software (ver. 4.0) with the Gene Ontology database as the reference. The GSEA method calculates an enrichment score (ES) that reflects the overrepresentation of a metabolite set at the top or bottom of a ranked list. Statistical significance was assessed based on the p-value of the ES, the normalized enrichment score (NES) -a size-normalized ES value -and the false discovery rate (FDR) derived from the NES. Pathways meeting the criteria of |NES|>1, p-value≤0.05, and FDR<0.25 were considered significantly enriched.

### Quantification of mitochondrial DNA copy number

Cells were seeded into 6-well plates at a density of 3×10^5^/mL. At 48 h post-transfection, the culture medium was removed by aspiration, and the cells were detached using trypsin, collected by centrifugation at 300×g for 3 min. Approximately 5×10^6^ cells were obtained for subsequent genomic DNA extraction. Genomic DNA was isolated using the MolPure Cell/Tissue DNA Kit (Yeasen Biotechnology) in accordance with the manufacturer’s instructions. Using genomic DNA as the template, the copy number of the mitochondrial gene was detected by real-time PCR as described above. The sequences of all primers used are provided in Supplement 2.

### Mitochondrial membrane potential detection

Cells were seeded into either poly-L-lysine-coated coverslip in 24-well plates or 6-well plates, and transfected upon reaching 70%–80% confluence. At 48 h post-transfection, mitochondrial membrane potential (MMP) was assessed using a JC-1 assay kit (Beyotime) according to the manufacturer’s instructions. For fluorescence microscopy, cells cultured on coverslips were incubated with JC-1 working solution at 37°C for 20 min, washed twice with 1× staining buffer, and visualized under a fluorescence microscope (Zeiss). ImageJ software was used to quantify the fluorescence intensity, and the MMP was expressed as the ratio of red (JC-1 aggregates) to green (JC-1 monomers) fluorescence. For flow cytometry analysis, cells were seeded in 6-well plates were harvested by trypsinization, resuspended in 500 μL of JC-1 working solution mixed with an equal volume of culture medium, and incubated at 37°C with 5% CO_2_ for 20 min. After incubation, cells were centrifuged at 600×g for 4 min, washed twice with 1× staining buffer, and finally resuspended in 500 μL of 1× staining buffer. Samples were then analyzed using a flow cytometer (Beckman Coulter), and data were processed with CytExpert software (ver. 2.3).

### Transmission electron microscopy

The treated cells were collected and centrifuged at 300×g for 5 min. After centrifugation, the cell pellet was fixed with 2.5% glutaraldehyde (Biosharp) and 1% osmium tetroxide (115355; AIKE) for 2 h. The samples were then dehydrated through a graded acetone series. Subsequently, they were infiltrated stepwise with mixtures of acetone (Xilongs) and Epon-812 embedding resin (Jijing) at ratios of 3:1, 1:1, and 1:3, followed by final embedding in pure Epon-812. Ultrathin sections (60–90 nm) were cut using an ultramicrotome (Leica Microsystems) and mounted onto copper grids. The sections were stained with uranyl acetate for 10–15 min and then with lead citrate for 1–2 min. Finally, the samples were examined and imaged using a transmission electron microscope (JEOL).

### Determination of ATP levels

Cells were seeded into 6-well plates. At 48 h after transfection, the culture medium was aspirated, and 150–200 μL of lysis buffer was added to each well to lyse the cells. Complete lysis was achieved by repeated pipetting, followed by incubation for 30 min. The lysates were then centrifuged at 12,000×g for 5 min at 4°C, and the supernatant was carefully collected. ATP levels were quantified using an ATP Content Assay Kit (MCE). A volume of 100 μL ATP detection reagent was added to all test wells, followed by incubation at room temperature for 3–5 min. The relative light units (RLU) were then measured using a chemiluminescence plate reader (Thermo Fisher Scientific).

### *PPARGC1A* overexpression vector construction and plasmid transfection

To construct the peroxisome proliferator-activated receptor gamma coactivator 1-alpha (*PPARGC1A*) overexpression vector, the cloning vector pcDNA3.1(+) underwent double digestion with BamHI and EcoRI. The digested vector and the coding sequence (CDS) of yak *PPARGC1A* were recombined using a gene recombination kit (Vazyme). The following transfection groups were established: non-targeting negative control siRNA along with the empty vector pcDNA3.1 (+) (si-NC+OE-NC group), 20 nM SIRT1-specific small interfering RNA combined with the empty vector pcDNA3.1 (+) (RNAi+OE-NC group), and 20 nM *SIRT1*-specific small interfering RNA together with the overexpression plasmid pcDNA3.1 (+)-PPARGC1A (RNAi+OE-PPARGC1A group). When cell confluence reached 60%–70%, the following mixtures were prepared: Liquid A (50 μL Opti-DMEM+2 μL Lipo3000, incubated for 5 min) and Liquid B (50 μL Opti-DMEM+2 μL P3000+1μg DNA, incubated for 5 min). These mixtures were incubated for 15 min, then added to the 12-well plates. Cells from different groups were handled under consistent passage times and confluence levels. After 48 h, both cells and supernatant were collected for subsequent analysis.

### Statistical analysis

Statistical comparisons were performed using unpaired Student’s t-test or one-way analysis of variance (ANOVA) followed by Tukey’s post hoc test for multiple comparisons. All experiments were independently repeated at least three times. Data were presented as mean±standard error of the mean (SEM). A p-value<0.05 was considered statistically significant.

## RESULTS

### SIRT1 plays a critical role in maintaining the viability and function of yak Sertoli cells

**Figure 1:**
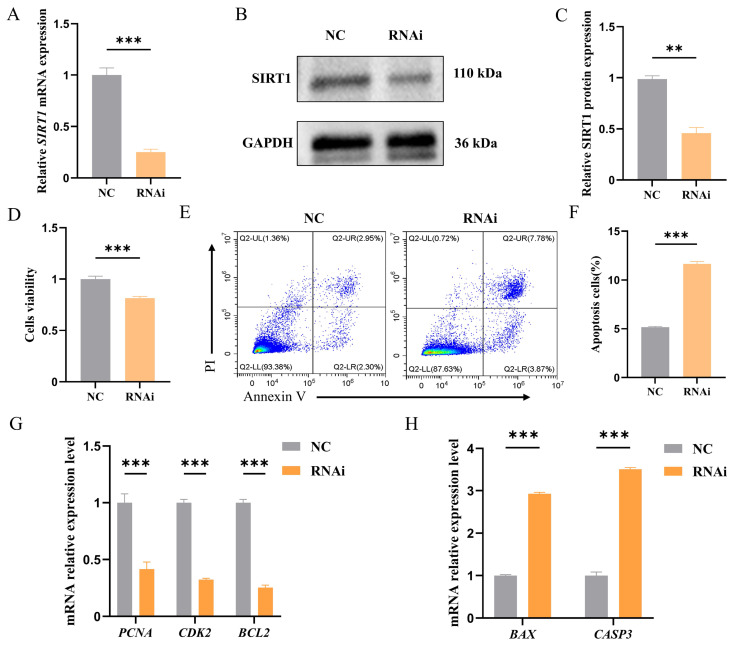
*SIRT1* knockdown impaired the viability of yak SCs. (A) *SIRT1* mRNA abundance in yak SCs after transfection with negative control (NC) and *SIRT1*-specific siRNA (RNAi). (B) Western blot analysis of SIRT1 in yak SCs of NC and RNAi groups. (C) Relative protein abundance of SIRT1 to GAPDH in the NC and RNAi groups by optical density analysis. (D) The cell viability rates were detected in the NC and RNAi groups using the CCK-8 assay. The OD values at 450 nm were compared after the cells were cultured for 48 h. (E) Apoptotic cells were detected using flow cytometry in different groups. (F) Statistical analysis of the proportion of apoptotic cells in (E). (G, H) Relative mRNA levels of proliferation-related genes (G) and apoptosis-related genes (H) in NC and RNAi groups. Data are expressed as mean±SEM from three independent experiments. ** p<0.01, *** p<0.001. Student’s unpaired t-test. RNAi, RNA interference; SCs, Sertoli cells; OD, optical density; SEM, standard error of the mean.

The SCs isolated from yak were successfully identified through immunostaining of the specific markers WT1 and SOX9 (Supplement 3). Subsequently, RNAi was utilized to knock down *SIRT1* expression in these cells. The knockdown efficiency was validated at both the mRNA and protein levels using quantitative RT-PCR (Figure 1A) and Western blot analysis (Figures 1B, 1C, Supplement 4), respectively. Cell viability was detected by CCK-8 assay and flow cytometry. The results showed that the cell viability decreased by 18.62% (Figure 1D) and the proportion of apoptotic cells increased by 225.41% (Figures 1E, 1F) in the RNAi group compared with that of the NC group. Besides, the expression levels of several viability and apoptosis-related genes were detected. Compared with the NC group, the expression levels of proliferation-related genes *PCNA* and *CDK2*, and the anti-apoptosis gene *BCL-2* were significantly downregulated in the RNAi group by 58.48%, 67.62%, and 74.87%, respectively (Figure 1G). Meanwhile, the expression levels of pro-apoptotic genes *BAX* and *CASP3* were significantly upregulated by 192.77% and 250.63%, respectively (Figure 1H).

**Figure 2:**
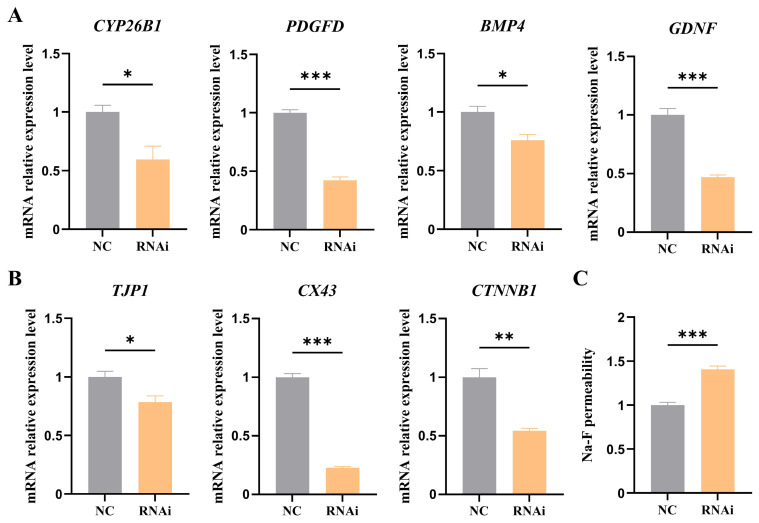
*SIRT1* knockdown impairs the function of yak SCs. (A) Relative mRNA levels of several Sertoli cell secretory factors responsible for spermatogenesis in NC and RNAi groups. (B) Relative mRNA levels of several genes encoding BTB-related proteins in different groups. (C) The permeability of Na-F in the NC and RNAi groups. Data are expressed as mean±SEM from three independent experiments. * p<0.05, ** p<0.01, *** p<0.001 indicated statistical significance at different levels; Student’s unpaired t-test. SCs, Sertoli cells; RNAi, RNA interference; BTB, blood-testis barrier; SEM, standard error of the mean.

Given that SCs support spermatogenesis through paracrine signaling and the formation of BTB, the mRNA expression levels of several spermatogenesis-related factors secreted by SCs were measured, including cytochrome P450 family 26 subfamily B member 1 (*CYP26B1*), *PDGFD*, *BMP4*, and bone morphogenetic protein 4 (*GDNF*), as well as genes associated with BTB assembly, including tight junction protein 1(*TJP1*), connexin 43 (*CX43*), and catenin beta 1 (*CTNNB1*). Compared with the NC group, these genes showed significant downregulation (Figures 2A, 2B). Furthermore, the integrity of the BTB was evaluated *in vitro* using a sodium fluorescein (Na-F) permeability assay. Results revealed that knockdown of *SIRT1* led to a significant increase in Na-F permeability (Figure 2C), indicating that SIRT1 is important for maintaining BTB integrity.

### Dynamic transcriptome changes in yak Sertoli cells after *SIRT1* knockdown

**Figure 3:**
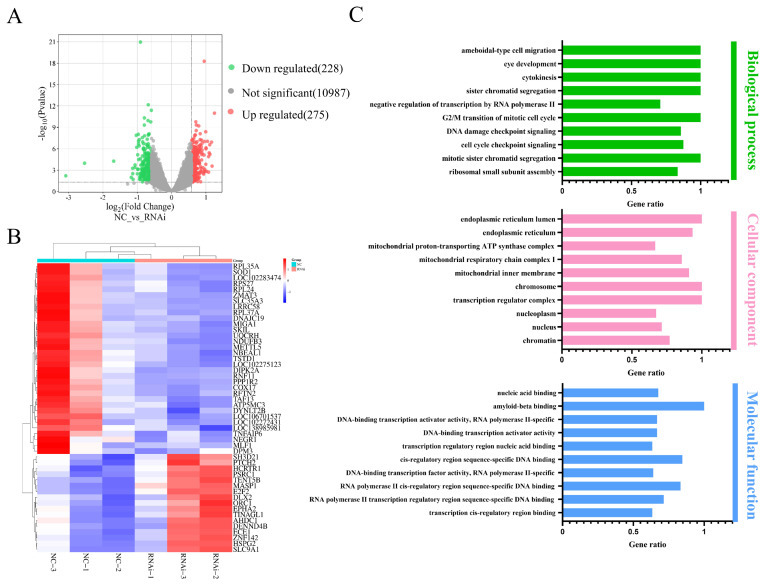
Transcriptome analysis of yak SCs after knockdown of *SIRT1*. (A) Volcano plot of all transcripts in the NC vs. RNAi group. The Y-axis represents the negative logarithm value of the error detection rate. Gray, red, and green dots represent genes with no significant change, significantly up-regulated genes, and significantly down-regulated genes. (B) Top 50 significantly differentially expressed genes clustering heatmap in the NC vs RNAi group, the three biological replicates for the control (NC) and RNAi groups were designated as NC-1, NC-2, and NC-3, and RNAi-1, RNAi-2, and RNAi-3, respectively. (C) GSEA-GO analysis of the Top 10 significantly differentially expressed terms in BP, CC, and MF, respectively. SCs, Sertoli cells; RNAi, RNA interference; GSEA, gene set enrichment analysis; BP, biological process; CC, cellular component; MF, molecular function.

To further explore the mechanism by which *SIRT1* regulates the viability in yak SCs, mRNA from the NC and RNAi groups were collected and sequenced. A total of 11,490 DE transcripts were identified in NC vs RNAi groups (Figures 3A, 3B, Supplement 5), with 275 transcripts up-regulated and 228 transcripts down-regulated. The accuracy of the sequencing data was confirmed by real-time PCR (Supplement 6). To investigate the functional implications of significant metabolic differences, GO analysis was conducted via GSEA. Functional enrichment analysis was performed on the terms corresponding to significantly DE genes in the three major functional categories (biological process [BP], cellular component [CC], and molecular function [MF] of the GO enrichment results). The top 10 most significantly enriched terms in BP, CC, and MF were listed (Figure 3C, Supplements 7–9). It can be observed that SIRT1 broadly influences BPs and MFs such as cell division, metabolism and gene transcription regulation, as well as CCs including chromosomes, the endoplasmic reticulum, and mitochondria.

### *SIRT1* knockdown impaired mitochondrial biogenesis in yak Sertoli cells

**Figure 4:**
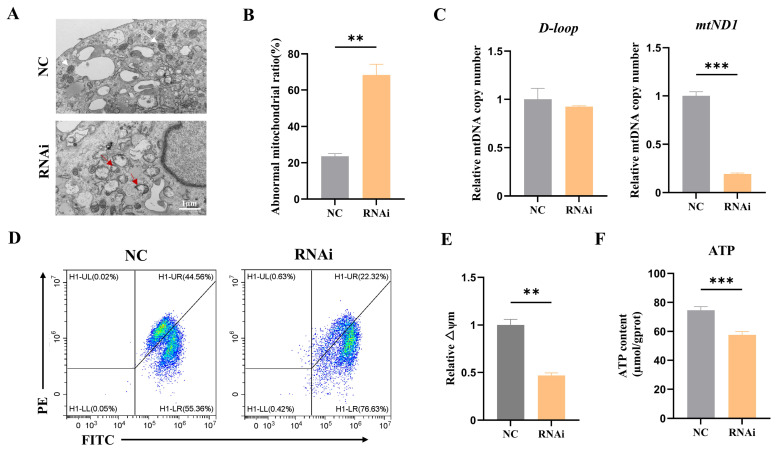
*SIRT1* knockdown impaired mitochondrial biogenesis in yak SCs. (A) Ultrastructure of the mitochondria in yak SCs of NC and RNAi groups as observed by TEM. Normal and abnormal mitochondria were marked by white and red arrowheads, respectively. (B) Rate of abnormal mitochondria in (A) observed by TEM. (C) Relative mRNA levels of mitochondrial genes, *mtND1* and *D-loop* in NC and RNAi groups. (D) Representative flow cytometry scatter plots showing JC-1 staining. The x-axis (FITC channel) detects JC-1 green fluorescent monomers, and the y-axis (PE channel) detects JC-1 red fluorescent aggregates. The upper right quadrant (Q2) represents cells with normal mitochondrial membrane potential, while the lower right quadrant (Q4) represents cells with decreased mitochondrial membrane potential. (E) MMP in (D) was quantified by the red/green fluorescence geometric mean ratio. (F) Intracellular ATP levels in different groups. ** p<0.01, *** p<0.001 indicated statistical significance at different levels; Student’s unpaired t-test. SCs, Sertoli cells; RNAi, RNA interference; MMP, mitochondrial membrane potential.

RNA-Seq results revealed that knockdown of *SIRT1* significantly affected multiple mitochondrial-related components in yak SCs, including mitochondrial inner membrane (GO:0005743), mitochondrial proton-transporting ATP synthase complex (GO:0005747), and mitochondrial proton-transporting ATP synthase complex (GO:0005753) (Figure 3C, Supplement 7). To evaluate the effect of SIRT1 on mitochondrial biogenesis in yak SCs, mitochondrial morphology, MMP, mitochondrial genome copy number, and energy metabolism levels were examined in both the NC and RNAi groups. In the NC group, the mitochondria were predominantly round, oval, or rhabdoid in shape, with well-preserved cristae, uniform matrix density, and intact inner membranes. In contrast, mitochondria in the RNAi group appeared mostly shrunken and vacuolated, exhibiting shortened and disorganized cristae along with reduced matrix electron density (Figures 4A, 4B). The expression levels of two mitochondrial genes, *D-loop* and *mtND1*, were also examined. While *SIRT1* knockdown had no effect on *D-loop* expression, it markedly downregulated *mtND1* expression (Figure 4C). The results show that suppression of *SIRT1* led to a significant reduction in MMP, with the red/green fluorescence ratio in the RNAi group decreasing to approximately 53.2% of that in the NC group (Figures 4D, 4E). Representative JC-1 fluorescence images are provided in Supplement 10. Furthermore, ATP content was significantly decreased in the RNAi group relative to the NC group (Figure 4F). Collectively, these results indicate that *SIRT1* plays an important role in mitochondrial biogenesis in yak SCs.

### SIRT1 is important for mitochondrial biogenesis by regulating the PGC-1α-NRF-1-TFAM pathway in yak Sertoli cells

**Figure 5:**
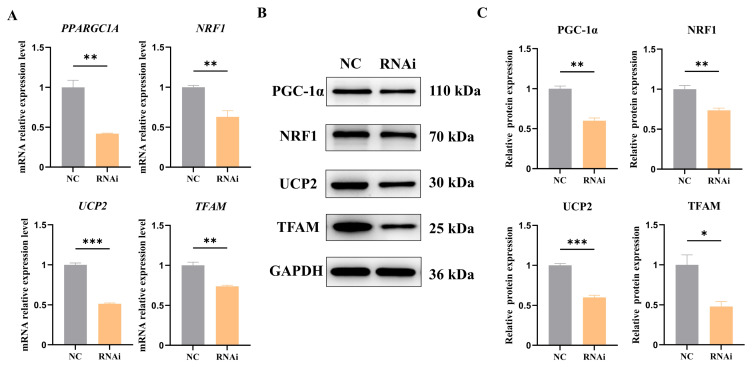
*SIRT1* knockdown inhibited the PGC-1α-NRF-1-TFAM pathway in yak SCs. (A) Relative mRNA levels of *PPARGC1A*, *NRF1*, *UCP2*, and *TFAM*. (B) Relative protein levels of PGC-1α, NRF1, UCP2, and TFAM. (C) Optical density analysis of (B). * p<0.05, ** p<0.01, *** p<0.001 indicated statistical significance at different levels; Student’s unpaired t-test. SCs, Sertoli cells; RNAi, RNA interference.

The PGC-1α–NRF-1–TFAM signaling pathway is the primary pathway regulating mitochondrial biogenesis. In this study, the activity of this pathway was examined in SIRT1-knockdown yak SCs. The results showed that, compared to the NC group, the mRNA levels of key members of the PGC-1α-NRF-1-TFAM signaling pathway, *PPARGC1A*, nuclear respiratory factor 1 (*NRF1*), uncoupling protein 2 (*UCP2*) and mitochondrial transcription factor A (*TFAM*), decreased by 58.0%, 36.9%, 48.6%, and 26.0%, respectively, in the RNAi group (Figure 5A). Correspondingly, the protein expression levels encoded by these four genes decreased by 40.4%, 26.3%, 40.0%, and 52.1%, respectively, in the RNAi group (Figures 5B, 5C, Supplement 4). These findings indicate that inhibition of *SIRT1* suppresses the PGC-1α-NRF-1-TFAM signaling pathway in yak SCs.

**Figure 6:**
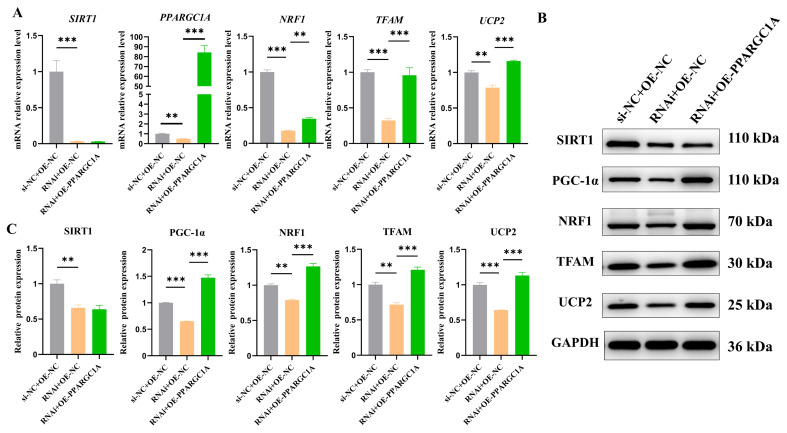
Overexpression of *PPARGC1A* activated the expression of NRF1, TFAM, and UCP2 in *SIRT1*-knockdown yak SCs. (A) Relative mRNA levels of *SIRT1*, *PPARGC1A*, *NRF1*, *UCP2*, and *TFAM* in si-NC+OE-NC, RNAi+OE-NC and RNAi+OE-PPARGC1A groups. (B) Protein levels of SIRT1, PGC-1α, NRF1, UCP2, and TFAM in different groups. (C) Optical density analysis of (B). ** p<0.01, *** p<0.001 indicated statistical significance at different levels; One-way ANOVA. SCs, Sertoli cells; RNAi, RNA interference.

**Figure 7:**
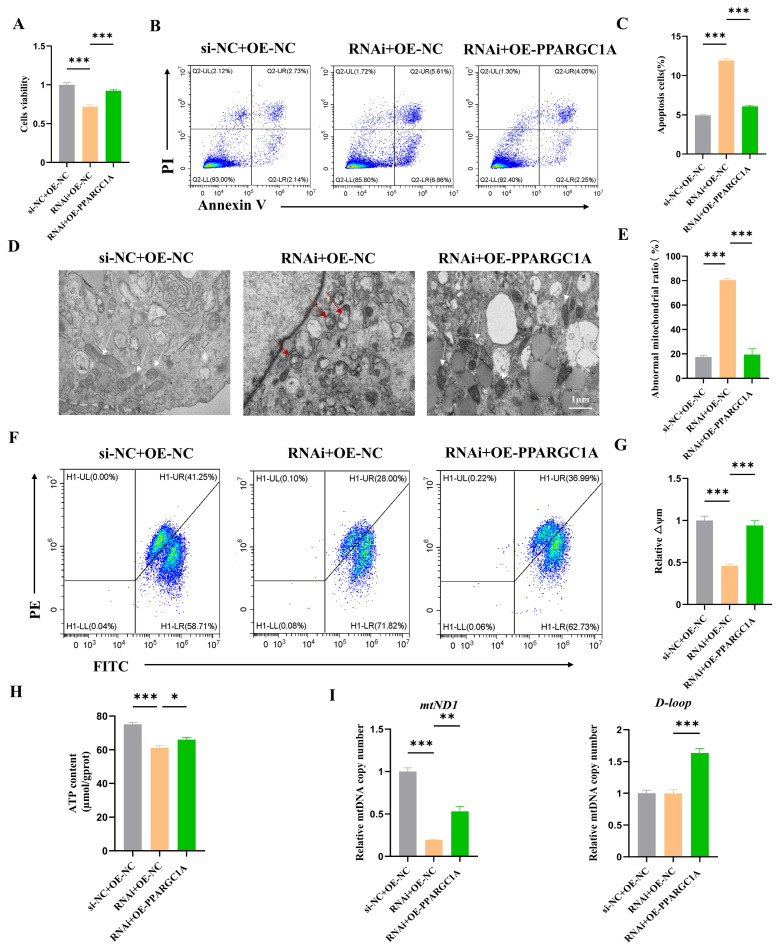
*PPARGC1A* overexpression rescued deficits in cell viability and mitochondrial biogenesis in yak SCs after *SIRT1* knockdown. (A) The cell viability rates were detected in different groups using the CCK-8 assay. The OD values at 450 nm were compared after the cells were cultured for 48 h. (B) Apoptotic cells were detected using flow cytometry in different groups. (C) Statistical analysis of the proportion of apoptotic cells in (B). (D) Ultrastructure of the mitochondria in yak SCs of different groups as observed by TEM. Normal and abnormal mitochondria were marked by white and red arrows, respectively. (E) Rate of abnormal mitochondria in (D) observed by TEM. (F) Representative flow cytometry scatter plots showing JC-1 staining. The x-axis (FITC channel) detects JC-1 green fluorescent monomers, and the y-axis (PE channel) detects JC-1 red fluorescent aggregates. The upper right quadrant (Q2) represents cells with normal mitochondrial membrane potential, while the lower right quadrant (Q4) represents cells with decreased mitochondrial membrane potential. (G) MMP in (F) was quantified by the red/green fluorescence geometric mean ratio. (H) Intracellular ATP levels in different groups. (I) Relative mRNA levels of mitochondrial genes, *D-loop* and *mtND1* in si-NC+OE-NC, RNAi+OE-NC and RNAi+OE-PPARGC1A groups. * p<0.05, ** p<0.01, *** p<0.001 indicated statistical significance at different levels; One-way ANOVA (with Tukey's multiple comparison test as the post-hoc test). RNAi, RNA interference; SCs, Sertoli cells; OD, optical density; MMP, mitochondrial membrane potential; ANOVA, analysis of variance.

To further confirm whether *SIRT1* regulates the viability and mitochondrial biogenesis of yak SCs through the PGC-1α-NRF-1-TFAM pathway, *PPARGC1A* was overexpressed in *SIRT1*-knockdown SCs. The results showed that, compared with the RNAi+OE-NC group, the expression of *SIRT1* did not change significantly in the RNAi+OE-PPARGC1A group, while the expression of *PPARGC1A* and its downstream target genes, including *NRF1*, *UCP2*, and *TFAM*, was significantly upregulated at both the mRNA and protein levels (Figures 6A–6C, Supplement 11). Compared to the RNAi+OE-NC group, the RNAi+OE-PPARGC1A group exhibited a significant restoration of cell viability and a marked reduction in apoptosis (Figures 7A–7C). Moreover, the overexpression of *PPARGC1A* partially rescued the abnormalities in mitochondrial morphology (Figures 7D, 7E), MMP (Figures 7F, 7G, Supplement 12), ATP synthesis capacity (Figure 7H), and genomic copy number (Figure 7I) caused by *SIRT1* deficiency, although the recovery of some indicators did not reach the levels observed in normal cells. These results suggested that SIRT1 is necessary for mitochondrial biogenesis by activating the PGC-1α-NRF-1-TFAM pathway in yak SCs.

## DISCUSSION

It is well-established that systemic knockout of *SIRT1* in mice causes damage to germ cells, SCs, and Leydig cells. Furthermore, specific knockout of *SIRT1* in germ cells and Leydig cells has demonstrated that SIRT1 contributes significantly to the development or function of both cell types [18]. However, although several studies have demonstrated that under certain stress conditions, such as combined treatment with resveratrol and follicle-stimulating hormone [21], triptolide induction [22], and PM2.5 exposure [23], SIRT1 expression is affected alongside damage to SCs, there is currently no direct animal model or cellular experiment confirming whether SIRT1 directly influences the viability and function of SCs under physiological conditions. In the present study, *SIRT1* expression was knocked down in yak SCs. We found that *SIRT1* knockdown led to reduced cell viability, increased apoptosis, downregulation of multiple genes encoding secretory factors regulating spermatogenic cells and components of the BTB, and impaired mitochondrial biogenesis in SCs. These results indicate that SIRT1 directly regulates the viability and function of SCs. This represents the first study to explore SIRT1 function specifically in SCs, providing a novel perspective for understanding the role of SIRT1 in male reproductive regulation.

It is well-established that SCs can support the normal development of spermatogenic cells through paracrine secretion and forming the BTB. Studies have shown that SIRT1 can influence paracrine function under certain drug-induced conditions [24] and the integrity of the BTB is closely linked to SIRT1 under stress conditions [25]. The present study demonstrated that knocking down *SIRT1* in yak SCs resulted in significant downregulation of several key genes encoding paracrine factors (*CYP26B1*, *PDGFD*, *BMP4*, and *GDNF)* and BTB components (*TJP1*, *CX43* and *CTNNB1*), which suggests that SIRT1 may play a role in regulating spermatogenesis in yak testis. Consistent with our findings, several of these genes—including *GDNF*, *TJP1*, and *CX43*—have been reported to be positively regulated by SIRT1 in other cell types [26–28]. Additionally, *CYP26B1* and *BMP4* have been shown to exhibit expression levels that positively correlate with SIRT1 [29,30]. In contrast, *PDGFD* and *CTNNB1* are considered to be negatively regulated by SIRT1 in endothelial cells and hypoxia-exposed HepG2 cells, respectively [30,31]. Together, these results suggest that the regulation of target genes by SIRT1 may be bidirectional and cell-type dependent. The importance of mitochondrial function in reproductive cell biology has been increasingly recognized.

Mitochondria play a crucial role in regulating cell fate, signal transduction, substance synthesis, and energy metabolism, among many other functions. Studies have reported that SIRT1 may play a contradictory role in mitochondrial biogenesis. Gurd and colleagues found that the overexpression of *SIRT1* reduces mitochondrial biogenesis in rat heart and skeletal muscle [32]. However, more studies have shown that *SIRT1* plays a positive role in mitochondrial biogenesis [33], which is consistent with our results in yak SCs. The PGC-1α–NRF-1–TFAM pathway is a key regulatory axis for mitochondrial biogenesis, and PGC-1α is one of the targets regulated by SIRT1 [34]. Consistently, pyrroloquinoline quinone has been reported to promote porcine oocyte maturation by upregulating PGC-1α, NRF1, NRF2 and TFAM, thereby enhancing mitochondrial function [35]. Our results collectively indicate that the regulatory role of SIRT1 in mitochondrial biogenesis in yak SCs partially depends on the PGC-1α-NRF-1-TFAM pathway. Notably, although there is a growing consensus suggesting that SIRT1 regulates PGC-1α through protein deacetylation [36], our results in yak SCs demonstrated that SIRT1 could regulate PGC-1α expression at both the transcriptional and protein levels. It is also noted that in *SIRT1*-knockdown cells, compared to the control group, the mRNA expression of *PPARGC1A* was upregulated more than 80-fold upon *PPARGC1A* overexpression. This increase was far greater than the fold upregulation of its downstream targets, *NRF1*, *TFAM*, and *UCP2* mRNAs. This suggests that the activation of downstream gene transcription by the PGC-1α protein may still depend on post-translational modifications. In *SIRT1*-knockdown cells, although *PPARGC1A* mRNA is highly abundant, the translated protein may exist in a hyperacetylated state, resulting in low transcriptional activity and only weak activation of these downstream genes. More intriguingly, the fold increases in protein levels of the four genes (*NRF1*, *TFAM*, *UCP2*, and PPARGC1A) were similar. It is speculated that under the stress induced by *SIRT1* knockdown, SCs might employ a series of special regulatory mechanisms to maintain basal survival. In the absence of *SIRT1*, the high levels of *PPARGC1A* mRNA might trigger negative feedback inhibition, resulting in extremely low translation efficiency. Conversely, the downstream genes (*NRF1*, *TFAM*, *UCP2*) originally had low mRNA levels. Yet, under the dual pressure of *SIRT1* deficiency and *PPARGC1A* overexpression, cells might prioritize the translation of these mRNAs related to mitochondrial function. This could cause the fold change in downstream protein levels to “catch up”, appearing consistent with the fold change of PGC-1α protein. Another possibility is that the absence of SIRT1 differentially affects the synthesis or degradation of these four proteins, ultimately leading to their similar fold changes in protein levels. Due to species-specific limitations, further experiments are warranted to verify the relationship between SIRT1 and this pathway. A recent study reported that plant essential oil alleviated liver injury via the SIRT1/PGC-1α pathway, improving mitochondrial function [37]. These parallel findings across tissues and species support a conserved role of this axis in maintaining mitochondrial homeostasis and cell viability.

Notably, the viability of SCs with *SIRT1* knockdown was not restored to normal levels after *PPARGC1A* overexpression in the present study. Considering the diverse functions of SIRT1, it is reasonable to infer that SIRT1 may regulate the viability of yak SCs through pathways other than mitochondrial biogenesis. For example, transcriptome sequencing results revealed that after *SIRT1* knockdown, several terms related to cell division and cycle, such as “mitotic sister chromatid segregation”, “G2/M transition of mitotic cell cycle” and “cell cycle checkpoint signaling” were enriched. It has been reported that *SIRT1* deficiency leads to cell cycle defects oligodendrocytes [38] and mouse embryonic fibroblasts [39]. Whether the decreased viability of yak SCs after *SIRT1* deletion is related to the cell cycle requires further experimental verification. In addition, multiple DE genes were enriched in MFs related to gene transcription regulation, such as “transcription coactivator activity” and “chromatin binding”. This suggests that SIRT1 is broadly involved in the transcriptional regulation of yak SCs. Given that SIRT1 functions as a histone demethylase, it is speculated that SIRT1 may participate in gene transcription regulation by altering chromatin structure.

## CONCLUSION

It is worth noting that although knockdown of *SIRT1* resulted in a more than 70% reduction in its mRNA expression in SCs, the decrease at the protein level was not as pronounced. Several possible reasons may account for this. First, the SIRT1 protein may have a relatively long half-life and stable structure. Second, SCs might possess a compensatory mechanism that partially offsets the loss of mRNA by enhancing the translation efficiency of the remaining mRNA or the synthesis efficiency of the protein. Transcriptomic sequencing results indicated that after *SIRT1* knockdown, several genes involved in endoplasmic reticulum regulation, such as FAD-dependent oxidoreductase domain containing 2 (*FOXRED2*), fibroblast growth factor receptor 3 (*FGFR3*) and proprotein convertase subtilisin/kexin type 6 (*PCSK6*), were upregulated in SCs, which may be related to this compensatory mechanism. Third, changes in certain pathways following knockdown *SIRT1*, such as the inhibition of mitochondrial biogenesis, might lead to suppressed degradation of the SIRT1 protein. In the future, it is necessary to completely eliminate SIRT1 expression at the protein level and, through *in vivo* and *in vitro* experiments, determine the extent to which SIRT1 influences the fate of SCs and the process of spermatogenesis.

## Data Availability

The raw data of RNA-Seq have been deposited in the NCBI Sequence Read Archive (SRA) database with the accession number SRR35803251- SRR35803256. Other data presented in this study are included in the published article and SUPPLEMENTARY MATERIAL. Further inquiries can be directed to the corresponding author.

## SUPPLEMENTARY MATERIAL

**Supplement 1**. Antibodies information.

**Supplement 2**. Primer sequences.

**Supplement 3**. Identification of yak SCs.

**Supplement 4**. The original Western Blot images corresponding to Figure 1 and Figure 5.

**Supplement 5**. Details of DE mRNAs in NC vs RNAi group.

**Supplement 6**. Transcriptome data reliability verification.

**Supplement 7**. The GSEA-GO-BP enrichment of DE mRNAs in NC vs RNAi group.

**Supplement 8**. The GSEA-GO-CC enrichment of DE mRNAs in NC vs RNAi group.

**Supplement 9**. The GSEA-GO-MF enrichment of DE mRNAs in NC vs RNAi group.

**Supplement 10**. Representative fluorescence images of JC-1 staining of NC group and RNAi group.

**Supplement 11**. The original Western Blot images corresponding to Figure 6.

**Supplement 12**. Representative fluorescence images of JC-1 staining of si-NC+OE-NC group, RNAi+OE-NC group and RNAi+OE-PPARGC1A group.
