# Supplementary material for: SIRT1 plays a critical role in maintaining the viability of Yak Sertoli cells by regulating mitochondrial biogenesis via activating the PGC-1α-NRF-1-TFAM pathway
Source: Anim Biosci. 2026 Apr 16;39(7):251005. doi: 10.5713/ab.251005 (PMC13353117; doi:10.5713/ab.251005)
Supplement: Supplementary file 1 [file ab-251005-Supplementary-1.pdf]

Supplement 1. Antibodies information

Antibody name	Article number	Manufacturer	Dilution rate	Application
WT1	12609-1-AP	Proteintech	1: 500	Immunofluorescence
SOX9	55152-1-AP	Proteintech	1: 500	Immunofluorescence
SIRT1	13161-1-AP	Proteintech	1： 3000	Western Blot
PGC-1α	66369-1-AP	Proteintech	1： 5000	Western Blot
NRF1	12482-1-AP	Proteintech	1： 3000	Western Blot
TFAM	22586-1-AP	Proteintech	1： 10000	Western Blot
UCP2	11081-1-AP	Proteintech	1： 1000	Western Blot
GAPDH	GB15002-100	Servicebio	1： 5000	Western Blot
Goat anti-rabbit IgG	GB23303	Servicebio	1:10000	Western Blot
Goat anti-mouse IgG	GB23301	Servicebio	1:10000	Western Blot
