# Supplementary material for: SIRT1 plays a critical role in maintaining the viability of Yak Sertoli cells by regulating mitochondrial biogenesis via activating the PGC-1α-NRF-1-TFAM pathway
Source: Anim Biosci. 2026 Apr 16;39(7):251005. doi: 10.5713/ab.251005 (PMC13353117; doi:10.5713/ab.251005)
Supplement: Supplementary file 2 [file ab-251005-Supplementary-2.pdf]

Supplement 2. Primer sequences

Primer name	Primer sequence (5´→3´)	Annealing temperature	Products size	Utilization	GenBank accession numbers
SIRT1	F: GATTGGCACAGATCCTCGAAC<br>R: AGTCTATTGCAAGGCGAGCA	60.2	199	RT-PCR	XM_070364475.1
PCNA	F: CGGTCGCAGCGGTAAGTGTC<br>R: ACATCAGCTCAAGTGGCGTGAAC	63.74	106	RT-PCR	XM_005906528.3
CDK2	F: TGCATCTTTGCTGAGATGGTGA<br>R: CAGGCATAGAAGTAACTCCTGG	60.29	133	RT-PCR	XM_014481481.2
BCL2	F: ACTTCGCCGAGATGTCCAGT<br>R: CACACATGACCCCTCCGAAC	60.67	144	RT-PCR	XM_070361474.1
BAX	F: TGGACATTGGACTTCCTTCG<br>R: GGTGAGCACTCCAGCCACA	57.53	132	RT-PCR	XM_005895371.2
CASP3	F: CCGAGGCACAGAACTGGACTG<br>R: TCGCCAGGAAAAGTAACCAGGTG	62.95	133	RT-PCR	XM_014480600.2
CYP26B1	F: GACAAGAGCTGCAAGCTGCC<br>R: CGAGCAAGTGCGTCTTGAAC	60.11	145	RT-PCR	XM_070379800.1
PDGFD	F: TTCTTGGAGTGACGCTGTCC<br>R: CGATGGTCTCGTCTCTTCGG	59.97	206	RT-PCR	XM_070384346.1
BMP4	F: CTCCTCTGAGCCTTTCCAGC<br>R: AACGACCATCAGCATTCGGT	60.11	123	RT-PCR	XM_005901930.3
GDNF	F: GACTTGGGTTTGGGCTACGA<br>R: CCCGACTTTGTCACTCACCA	59.96	141	RT-PCR	XM_005898534.2
TJP1	F: GGTAACGCCGTCCTCTGAAA<br>R: GCTATCGAGGCTCTTGCTCC	60.04	112	RT-PCR	XM_070358471.1
CX43	F: CGAATCCTGCTACTGGGGAC<br>R: CCAGAAGCGCACATGAGAGA	59.89	138	RT-PCR	XM_014476487.2
CTNNB1	F: TCCGATTGGCGGCTTAAACT<br>R: ATTGCAAAGCGCACTTGGTT	60.04	194	RT-PCR	XM_070359563.1
PPARGC1A	F: AATGCAGTGGCCTCAGTACC<br>R: CTCTGAGCACGGACGTCTTT	60.04	149	RT-PCR	XM_070372208.1
NRF1	F: CCGCTCTGAGTGGATCTTCAT<br>R: CACTGCGTGAGCTTCTATGG	59.59	79	RT-PCR	XM_070369235.1
TFAM	F: ACTAGGTCTCAATACCGGAAGC<br>R: TGAATTGGCTGGCAGAAGTCC	59.31	108	RT-PCR	XM_005906585.3
UCP2	F: AGCAGTTCTACACCAAGGGC<br>R: CGGGCAATGGTTTTGTAGGC	59.96	193	RT-PCR	XM_070383747.1
TGM3	F: ATCTACGAGTCGAACGGTGC<br>R:ACATCCATGCGAGAGTTGCT	59.9	104	RT-PCR	XM_005893928.3
FOSL1	F: TTGAACCCGAAGCATTGCAC<br>R: CTGGCGTACTGGGGTATGTG	59.69	96	RT-PCR	XM_070359333.1
MASP1	F: CTCCACCAGGAACAACCTCAC<br>R: TCCCCAGGACTTCATTCATCC	60.54	140	RT-PCR	XM_005899647.3

Continued Supplement 2. Primer sequences

Primer name	Primer sequence (5′→3′)	Annealing temperature	Products size	Utilization	GenBank accession numbers
ATP5ME	F: CGCCAAGCGCTACAATTACC<br>R: CTTCTTCTCCTCGGCTGCAA	59.97	70	RT-PCR	XM_070372860.1
COX17	F: GCTTCGCCAAGTCTGGAGAG<br>R: CAGACCGGGCATTTTTCGTG	60.74	73	RT-PCR	XM_005905432.3
NREP	F: CGATTCCTGCCGAGTGTAGAG<br>R: GTCTTGGCCTTGTACCCTCC	60.27	104	RT-PCR	XM_070377189.1
KRIT1	F: GAGAGACGGAGAGCAATGGG<br>R: TTCCCGAGAATTGAGACTGGC	59.9	96	RT-PCR	XM_070369871.1
BCAS2	F: CTCGCAGATACCGACCTACG<br>R: GGTTGTCGAGCAGCCAATCT	59.76	115	RT-PCR	XM_005887109.3
KMT2E	F: CGCGGATCCCCAGTTACTAC<br>R: CAGGATCAGGATCACTGTCTTTA	59.31	106	RT-PCR	XM_070369647.1
D-loop	F: TCTTCAGGGCCATCTCACCT<br>R: CAATAGATGCTCCGGGTCGG	60.25	186	RT-PCR	NC_006380.3
mtND1	F: CTGACCATGTGGATTCCGCT<br>R: TAGCTAATGGTCTGCGCCAC	60.11	179	RT-PCR	NC_006380.3
GAPDH	F: GGTGCTGAGTATGTCGTGGAG<br>R: CCCTTCCACAATGCCAAAGTT	60.47	249	RT-PCR	XM_070371805.1
