# Supplementary material for: SIRT1 plays a critical role in maintaining the viability of Yak Sertoli cells by regulating mitochondrial biogenesis via activating the PGC-1α-NRF-1-TFAM pathway
Source: Anim Biosci. 2026 Apr 16;39(7):251005. doi: 10.5713/ab.251005 (PMC13353117; doi:10.5713/ab.251005)
Supplement: Supplementary file 3 [file ab-251005-Supplementary-3.pdf]

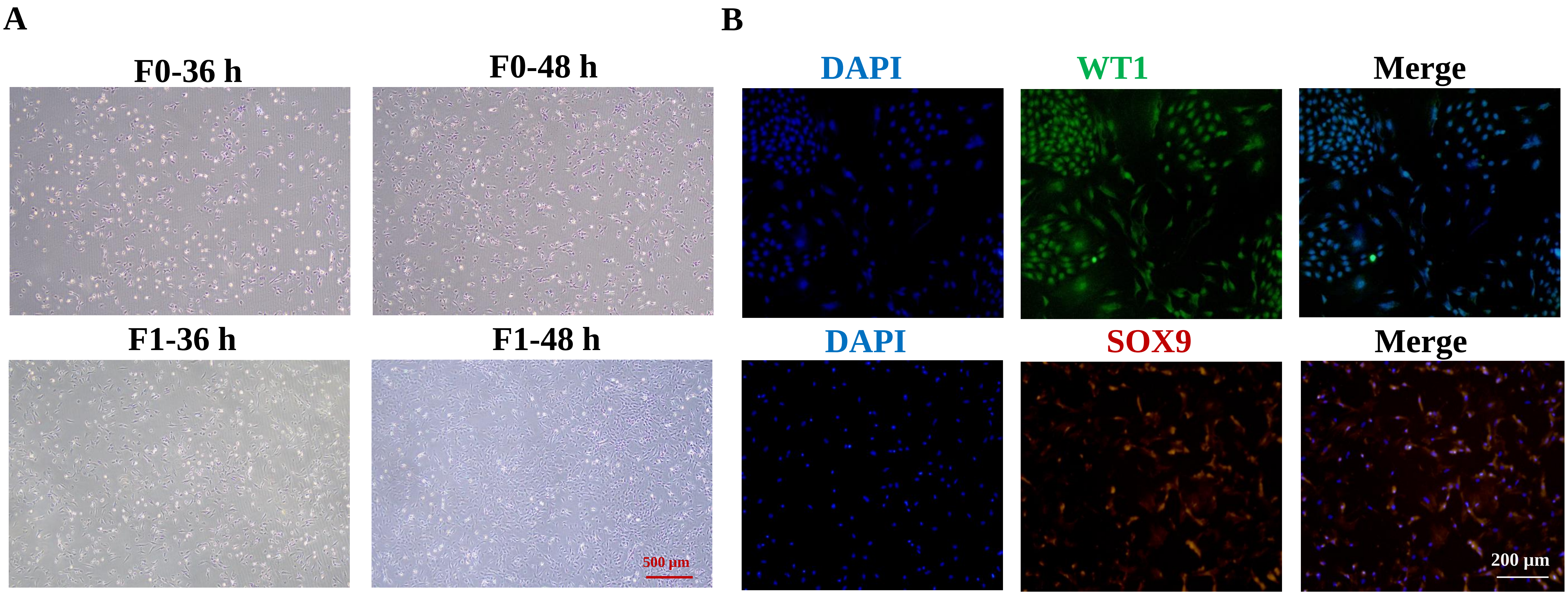

Supplement 3. Identification of yak SCs. (A) Representative photos of F0 and F1 generation yak Sertoli cells cultured for 36 and 48 h, respectively. (B) Yak SCs were identified by immunostaining with SC marker protein SOX9 and WT1. Images represent three independent experiments, and three slides were analyzed in each experiment.
