# Supplementary material for: SIRT1 plays a critical role in maintaining the viability of Yak Sertoli cells by regulating mitochondrial biogenesis via activating the PGC-1α-NRF-1-TFAM pathway
Source: Anim Biosci. 2026 Apr 16;39(7):251005. doi: 10.5713/ab.251005 (PMC13353117; doi:10.5713/ab.251005)
Supplement: Supplementary file 4 [file ab-251005-Supplementary-4.pdf]

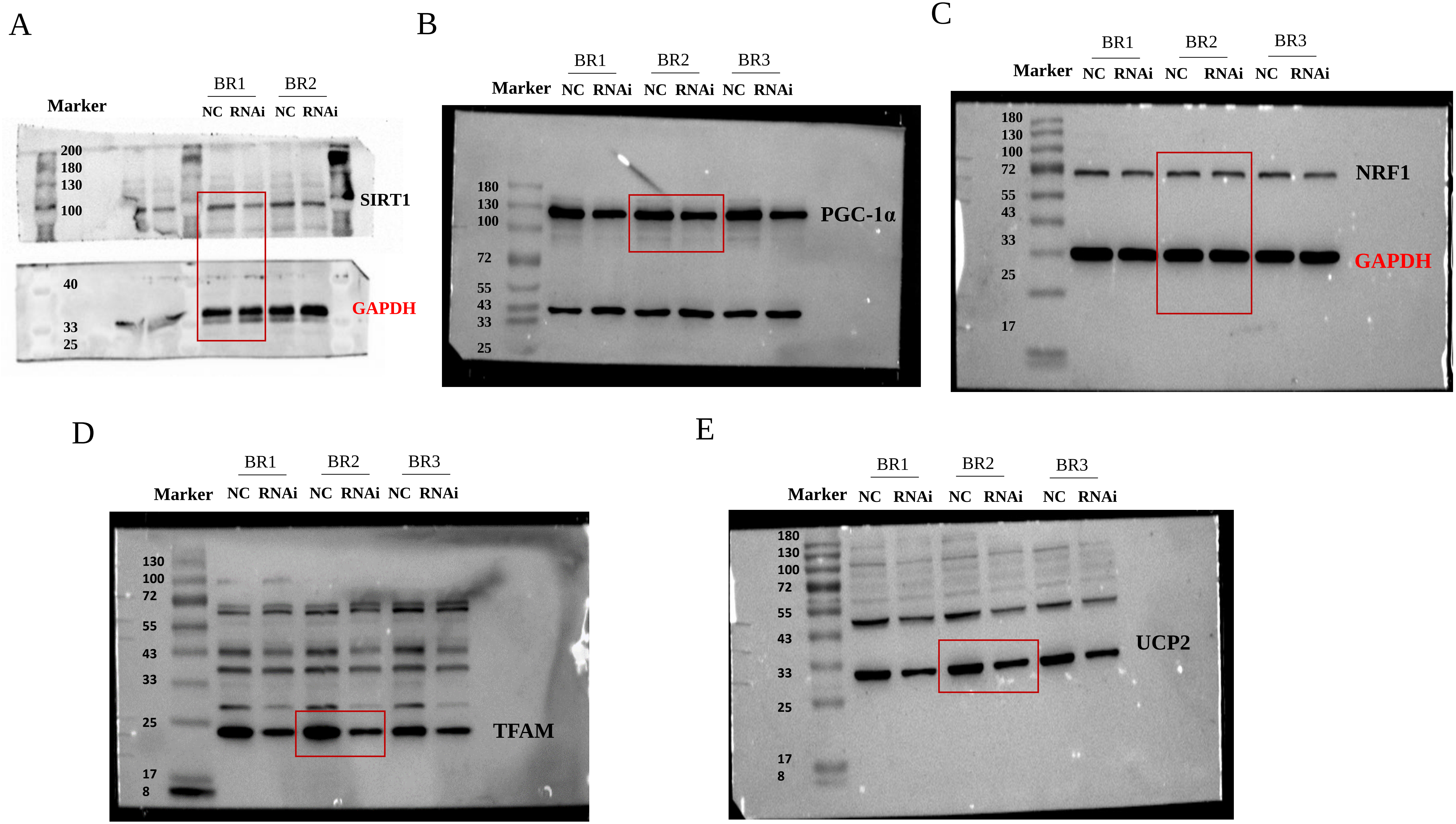

Supplement 4. The original Western Blot images corresponding to Figure 1 and Figure 5. (A) Western blot analysis of SIRT1 in yak SCs of NC and RNAi group. (B) Western blot analysis of PGC-1α in yak SCs of NC and RNAi group. (C) Western blot analysis of NRF1 and GAPDH in yak SCs of NC and RNAi group. (D) Western blot analysis of TFAM in yak SCs of NC and RNAi group. (E) Western blot analysis of UCP2 in yak SCs of NC and RNAi group. BR1, BR2, and BR3 represents three independent biological replications. The size of each membrane was indicated on the images. The bands described in the main text were marked using red rectangles.
