# Supplementary material for: SIRT1 plays a critical role in maintaining the viability of Yak Sertoli cells by regulating mitochondrial biogenesis via activating the PGC-1α-NRF-1-TFAM pathway
Source: Anim Biosci. 2026 Apr 16;39(7):251005. doi: 10.5713/ab.251005 (PMC13353117; doi:10.5713/ab.251005)
Supplement: Supplementary file 6 [file ab-251005-Supplementary-6.pdf]

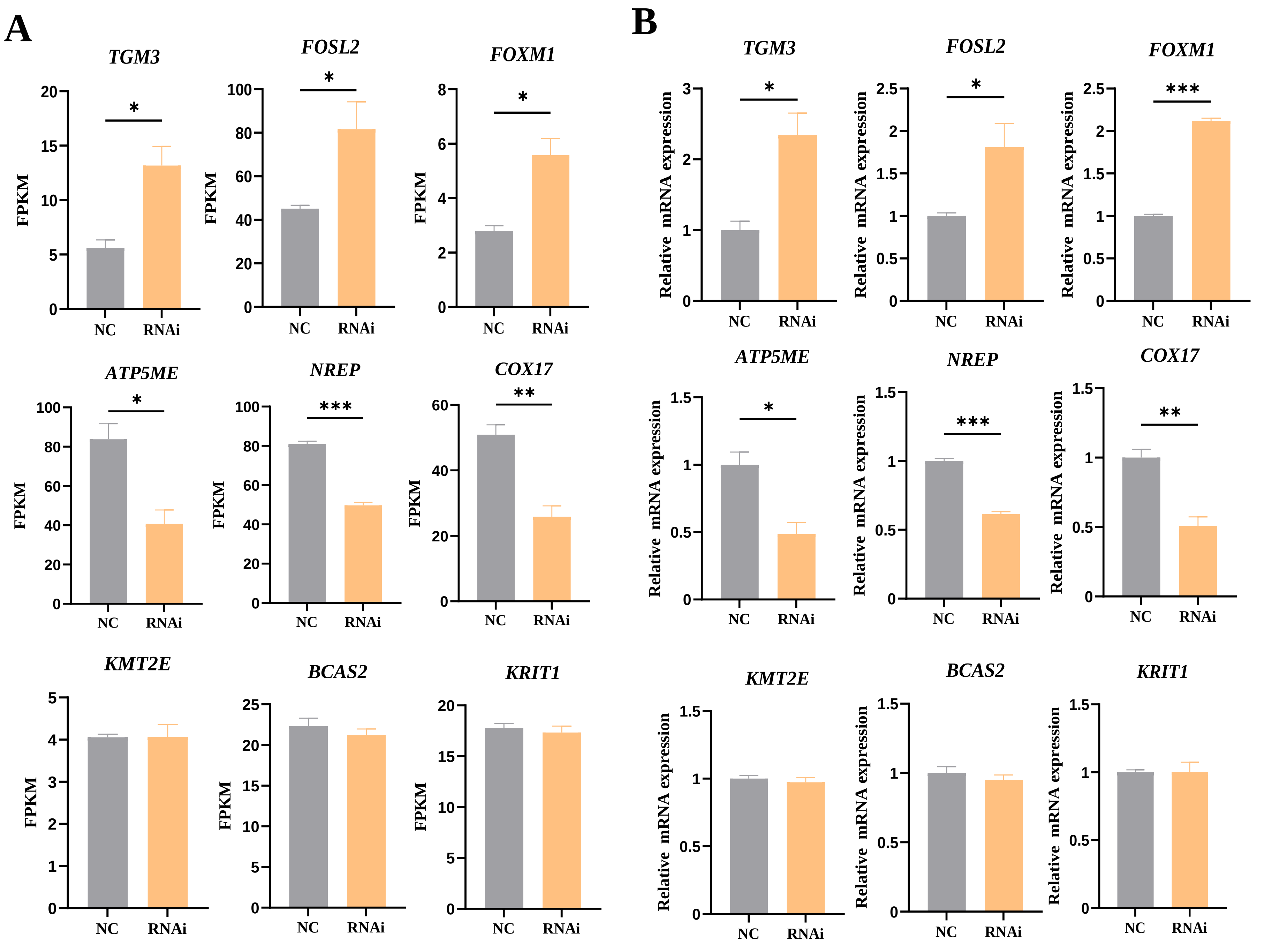

Supplement 6. Transcriptome data reliability verification. (A) Nine genes with different expression tendencies were randomly selected from the RNA-seq data. (B) The mRNA expression levels of gene in (A) were analyzed using real-time PCR. *$P < 0.05$, **$P < 0.01$, ***$P < 0.001$ indicated statistical significance at different levels; Student's unpaired t-test.
