# Supplementary material for: SIRT1 plays a critical role in maintaining the viability of Yak Sertoli cells by regulating mitochondrial biogenesis via activating the PGC-1α-NRF-1-TFAM pathway
Source: Anim Biosci. 2026 Apr 16;39(7):251005. doi: 10.5713/ab.251005 (PMC13353117; doi:10.5713/ab.251005)
Supplement: Supplementary file 7 [file ab-251005-Supplementary-7.pdf]

Supplement 7. The GSEA-GO-BP enrichment of DE mRNAs in NC vs RNAi group.

ID	Description	setSize	enrichmentScore	NES	pvalue	p.adjust	qvalue	rank	leading_edge	core_enrichment
GO:0000028	ribosomal small subunit assembly	6	-0.688128773	-1.98222854	0.003928008	0.178653189	0.174226801	162	tags=100%, list=32%, signal=69%	RPS27/LOC102275123/RPS19/RPS28/LOC102265456
GO:0000070	mitotic sister chromatid segregation	3	0.772	1.65906228	0.040321682	0.607954898	0.592891949	117	tags=100%, list=23%, signal=77%	KIF18B/PLK1/ESPL1
GO:0000075	cell cycle checkpoint signaling	8	0.582962541	1.948404299	0.010707594	0.335498224	0.327185777	160	tags=88%, list=32%, signal=61%	LOC102287159/GTSE1/PLK1/ZNF385A/TICRR/PML/RBM38
GO:0000077	DNA damage checkpoint signaling	7	0.566454211	1.774291326	0.0188543	0.412725217	0.402499361	160	tags=86%, list=32%, signal=59%	LOC102287159/GTSE1/PLK1/ZNF385A/PML/RBM38
GO:0000086	G2/M transition of mitotic cell cycle	3	0.798	1.714937435	0.02752649	0.517292844	0.504476177	104	tags=100%, list=21%, signal=80%	FOXM1/CDC25B/PLK1
GO:0000122	negative regulation of transcription by RNA polymerase II	24	0.339775991	1.82385702	0.015058852	0.365278921	0.356228615	203	tags=71%, list=40%, signal=44%	NCOR2/NACC2/DLX2/HCFC1/GLIS2/RXRA/ERF/E2F8/CIC/SALL2/PLK1/PER1/NFIC/PPP1R13L/FOXC2/USP2/PHF1[illegible]
GO:0000819	sister chromatid segregation	3	0.772	1.65906228	0.040321682	0.607954898	0.592891949	117	tags=100%, list=23%, signal=77%	KIF18B/PLK1/ESPL1
GO:0000910	cytokinesis	3	0.772	1.65906228	0.040321682	0.607954898	0.592891949	117	tags=100%, list=23%, signal=77%	PLK1/INCENP/ESPL1
GO:0001654	eye development	5	0.626506024	1.699553893	0.033376497	0.578848426	0.564506631	191	tags=100%, list=38%, signal=63%	SIPA1L3/RXRA/SALL2/PPP1R13L/FOXC2
GO:0001667	ameboidal-type cell migration	7	0.534274194	1.673494607	0.02774478	0.517292844	0.504476177	238	tags=100%, list=47%, signal=53%	TMEM201/LRP5/PML/SEMA4B/SEMA3F/SCARB1/PLXND1
GO:0001708	cell fate specification	2	0.874251497	1.571920622	0.039316641	0.607954898	0.592891949	65	tags=100%, list=13%, signal=87%	PTCH2/SIX2
GO:0001867	complement activation, lectin pathway	1	0.992031873	1.343189254	0.013407276	0.335498224	0.327185777	5	tags=100%, list=1%, signal=99%	MASP1
GO:0001890	placenta development	3	0.81	1.740725967	0.02130589	0.447299091	0.436216618	98	tags=100%, list=19%, signal=81%	CCNF/RXRA/E2F8
GO:0002062	chondrocyte differentiation	2	0.874251497	1.571920622	0.039316641	0.607954898	0.592891949	65	tags=100%, list=13%, signal=87%	FGFR3/SIX2
GO:0002181	cytoplasmic translation	5	-0.765060241	-2.053046589	0.003191296	0.161812701	0.157803561	123	tags=100%, list=24%, signal=76%	RPL35A/LOC138987848/LOC102269867/RPL22
GO:0002821	positive regulation of adaptive immune response	2	-0.832335329	-1.503660258	0.046194156	0.667515691	0.650977038	87	tags=100%, list=17%, signal=83%	SIRT1
GO:0002920	regulation of humoral immune response	1	0.992031873	1.343189254	0.013407276	0.335498224	0.327185777	5	tags=100%, list=1%, signal=99%	MASP1

GO:0002921	negative regulation of humoral immune response	1	0.992031873	1.343189254	0.013407276	0.335498224	0.327185777	5	tags=100%, list=1%, signal=99%	MASP1
GO:0003002	regionalization	5	0.62248996	1.688659317	0.037050876	0.604711416	0.589728828	193	tags=100%, list=38%, signal=62%	DLX2/LRP5/KAT2A/FOXC2/EMX2
GO:0003179	heart valve morphogenesis	3	0.872	1.87396672	0.005582234	0.220222204	0.214765885	67	tags=100%, list=13%, signal=87%	FGFRL1/LOC138991273/ZFPM1
GO:0006091	generation of precursor metabolites and energy	7	-0.636323819	-1.966533776	0.006448725	0.250855314	0.244640016	126	tags=86%, list=25%, signal=65%	PPP1R2/UQCR10/NDUFS4/UQCRH/NDUFB3/NDUFA5
GO:0006139	nucleobase-containing compound metabolic process	97	0.224967304	2.059276021	0.002792533	0.156643658	0.152762588	168	tags=49%, list=33%, signal=41%	FOXM1/ZNF865/MAMSTR/SP2/FOSL1/ZMIZ2/FOXK1/LOC106701625/E2F2/ZNF142/KLF2/NCOR2/NACC2/SLC2A4<br>G/RAVER1/LOC102274386/MKI67/RECQL4/LOC138991273/ZFPM1/ATN1/RXRA/SETD1A/SOX13/ERF/ZNF575/ZF<br>6/E2F8/CIC/SALL2/CHERP/ZNF385A/PER1/KMT5C/TICRR/LIPE/ZNF574/PHF19/NFIC/KAT2A/PDE7A/PML/BCL9L/Z<br>F358/BCL3/RBM38/ASF1B/PPP1R13L
GO:0006164	purine nucleotide biosynthetic process	3	-0.814	-1.746848987	0.013644217	0.335498224	0.327185777	97	tags=100%, list=19%, signal=81%	ATP5MC3/ATP5ME
GO:0006334	nucleosome assembly	1	-0.988047809	-1.329247561	0.023074547	0.468009166	0.456413572	8	tags=100%, list=2%, signal=99%	
GO:0006351	DNA-templated transcription	67	0.268375624	2.155725215	0.000767113	0.059868189	0.058384869	266	tags=76%, list=53%, signal=41%	FOXM1/ZNF865/MAMSTR/SP2/FOSL1/ZMIZ2/FOXK1/ZNF142/KLF2/NCOR2/NACC2/SLC2A4RG/ZFPM1/ATN1/RX<br>/SETD1A/SOX13/ERF/ZNF575/E2F8/CIC/SALL2/ZNF385A/PER1/KMT5C/ZNF574/PHF19/NFIC/KAT2A/PML/BCL9L/<br>NF358/BCL3/ASF1B/PPP1R13L/ZNF438/SNAPC4/TCF7L1/PHF12/SRCAP/ZNF341/GATA6/MSX1/PPRC1/ZNF414/T<br>22D4/E2F1/MLLT1/ZNF335/VAX2/UHRF1
GO:0006355	regulation of DNA-templated transcription	98	0.292730592	2.667114775	8.88143E-06	0.002125623	0.002072958	234	tags=69%, list=47%, signal=46%	FOXM1/ZNF865/MAMSTR/SP2/FOSL1/ZMIZ2/FOXK1/TFAP4/ZNF142/KLF2/NCOR2/NACC2/SLC2A4RG/DLX2/ABC<br>/LOC138991273/SIX2/ZFPM1/ATN1/HCFC1/GLIS2/RXRA/SETD1A/ETV4/MYBL2/ERF/ZNF575/JUP/E2F8/CIC/SALL<br>PLK1/ZNF385A/PER1/RNF44/SOX12/KMT5C/LRP5/MRTFA/ZNF574/PHF19/NFIC/KAT2A/PML/PSRC1/BCL9L/ZNF3<br>/BCL3/ASF1B/PPP1R13L/ZNF438/KDM6B/CAMTA2/SNAPC4/FOXC2/EMX2/USP2/TCF7L1/PHF12/SRCAP/BCL7A/[illegible]
GO:0006357	regulation of transcription by RNA polymerase II	54	0.394358496	2.936891244	2.15529E-06	0.001105357	0.00107797	231	tags=80%, list=46%, signal=48%	FOXM1/ZNF865/MAMSTR/SP2/FOSL1/ZMIZ2/KLF2/NCOR2/NACC2/DLX2/ABCA2/SIX2/HCFC1/GLIS2/RXRA/ETV4/<br>YBL2/ERF/E2F8/CIC/SALL2/PLK1/PER1/SOX12/LRP5/MRTFA/NFIC/KAT2A/PML/BCL9L/BCL3/PPP1R13L/KDM6B/C<br>MTA2/SNAPC4/FOXC2/USP2/TCF7L1/PHF12/SRCAP/GATA6/PPRC1/AUTS2
GO:0006364	rRNA processing	6	-0.820925553	-2.364763872	0.000128888	0.0159555	0.01556018	96	tags=100%, list=19%, signal=82%	RPL34/RPS19/SIRT1/RPS28/LOC102265456
GO:0006412	translation	30	-0.567114467	-3.248162226	5.34045E-08	4.79305E-05	4.6743E-05	222	tags=97%, list=44%, signal=57%	RPS23/RPL24/RPL27A/RPL37A/RPL23/RPS17/RPS27/LOC102266576/LOC102286668/RPS29/RPL36A/RPLP2/RPL[illegible]<br>A/LOC138987848/RPL34/LOC102275123/RPS19/MRPS18C/RPL36AL/RPL39/LOC102269867/LOC106701537/RPL[illegible]<br>/RPS28/LOC102279476/LOC102285651/LOC102281282/LOC102265456/LOC102270678
GO:0006413	translational initiation	4	-0.753507014	-1.828950275	0.012430243	0.335498224	0.327185777	128	tags=100%, list=25%, signal=75%	MRPL33/RPL34/MRPS18C
GO:0006414	translational elongation	4	-0.753507014	-1.828950275	0.012430243	0.335498224	0.327185777	128	tags=100%, list=25%, signal=75%	RPLP2/MRPL33/MRPS18C
GO:0006417	regulation of translation	8	0.580246574	1.939326869	0.011298349	0.335498224	0.327185777	159	tags=88%, list=32%, signal=61%	LOC138991273/RXRA/ZFP36/ZNF385A/PER1/PML/BCL3
GO:0006468	protein phosphorylation	10	0.529411765	1.983293649	0.011187363	0.335498224	0.327185777	242	tags=100%, list=48%, signal=53%	CDC25B/FGFR3/LOC138991273/PLK1/FAM20C/ULK1/MARK4/DYRK1B/LYN/LOC138990715
GO:0006508	proteolysis	16	0.355849285	1.606524261	0.04138251	0.613897559	0.598687372	212	tags=75%, list=42%, signal=45%	FOXRED2/CCNF/GTSE1/PCSK6/RNPEPL1/RNF44/CAPN15/ECE1/PML/FBXW5/USP2/MMP15
GO:0006518	peptide metabolic process	36	-0.45794608	-2.810801302	1.34635E-05	0.002565099	0.002501545	229	tags=86%, list=46%, signal=51%	SOD1/RPS23/RPL24/CARNMT1/RPL27A/RPL37A/RPL23/RPS17/RPS27/LOC102266576/LOC102286668/RPS29/RP<br>6A/RPLP2/RPL35A/LOC138987848/RPL34/LOC102275123/RPS19/MRPS18C/RPL36AL/RPL39/LOC102269867/LO[illegible]<br>06701537/RPL22/RPS28/LOC102279476/LOC102285651/LOC102281282/LOC102265456/LOC102270678

GO:0006607	NLS-bearing protein import into nucleus	1	-0.978087649	-1.315847888	0.040314726	0.607954898	0.592891949	13	tags=100%, list=3%, signal=98%	
GO:0006725	cellular aromatic compound metabolic process	99	0.214579535	1.956422741	0.00373066	0.173935944	0.169626433	168	tags=48%, list=33%, signal=40%	FOXM1/ZNF865/MAMSTR/SP2/FOSL1/ZMIZ2/FOXK1/LOC106701625/E2F2/ZNF142/KLF2/NCOR2/NACC2/SLC2A<br>G/RAVER1/LOC102274386/MKI67/RECQL4/LOC138991273/ZFPM1/ATN1/RXRA/SETD1A/SOX13/ERF/ZNF575/ZF<br>6/E2F8/CIC/SALL2/CHERP/ZNF385A/PER1/KMT5C/TICRR/LIPE/ZNF574/PHF19/NFIC/KAT2A/PDE7A/PML/BCL9L/<br>F358/BCL3/RBM38/ASF1B/PPP1R13L
GO:0006807	nitrogen compound metabolic process	177	0.151116387	1.602391163	0.021700232	0.44772317	0.43663019	162	tags=41%, list=32%, signal=43%	TGM3/FOXRED2/FOXM1/ZDHHC8/ZNF865/CEMIP/MAMSTR/SP2/FOSL1/ZMIZ2/FOXK1/LOC106701625/E2F2/ZN<br>42/KLF2/NCOR2/CCNF/CDC25B/NACC2/LOC102287159/SLC2A4RG/RAVER1/LOC102274386/MKI67/POM121C/F<br>R3/RECQL4/UROC1/LOC138991273/ZFPM1/ATN1/ABCA3/RXRA/SETD1A/GGT5/SOX13/ERF/ZNF575/GTSE1/PCSK<br>RNPEPL1/ZFP36/E2F8/CIC/SALL2/CHERP/PLK1/INCENP/ZNF385A/PER1/CERS1/RNF44/FAM20C/PIGT/KMT5C/CA
GO:0006874	intracellular calcium ion homeostasis	4	0.709418838	1.742525496	0.028242172	0.517292844	0.504476177	149	tags=100%, list=30%, signal=71%	GRINA/HCRTR1/CHERP/PML
GO:0006915	apoptotic process	12	0.422287398	1.710816423	0.030543988	0.548264589	0.534680552	179	tags=75%, list=36%, signal=49%	E2F2/ZNF385A/MTFP1/ESPL1/PIGT/LRP5/PML/PPP1R13L/RTKN
GO:0006950	response to stress	53	0.238100175	1.763172329	0.032105226	0.570582983	0.556445976	106	tags=38%, list=21%, signal=33%	MASP1/FOXRED2/FOXM1/FOSL1/TFAP4/KLF2/LOC102269246/LOC102287159/MKI67/RECQL4/LOC138991273/<br>RL3/GGT5/GTSE1/SCAP/GAS2L1/VASN/ZFP36/PLK1/ZNF385A
GO:0006977	DNA damage response, signal transduction by p53 class mediator resulting in cell cycle arrest	6	0.544063929	1.600439009	0.034695476	0.579334539	0.5649807	160	tags=83%, list=32%, signal=58%	LOC102287159/GTSE1/ZNF385A/PML/RBM38
GO:0007049	cell cycle	20	0.343118603	1.72935481	0.032415786	0.570639661	0.55650125	109	tags=50%, list=22%, signal=41%	KIF18B/E2F2/CCNF/CDC25B/MKI67/HCFC1/E2F8/PLK1/INCENP/CCNB3
GO:0007059	chromosome segregation	4	0.773547094	1.900041925	0.010709709	0.335498224	0.327185777	117	tags=100%, list=23%, signal=77%	KIF18B/PLK1/INCENP/ESPL1
GO:0007093	mitotic cell cycle checkpoint signaling	8	0.582962541	1.948404299	0.010707594	0.335498224	0.327185777	160	tags=88%, list=32%, signal=61%	LOC102287159/GTSE1/PLK1/ZNF385A/TICRR/PML/RBM38
GO:0007165	signal transduction	73	0.197309334	1.643192853	0.026098191	0.517292844	0.504476177	281	tags=74%, list=56%, signal=38%	ARHGAP33/TNS2/TFAP4/FGFRL1/LOC106701625/E2F2/LOC102287159/FGFR3/LOC138991273/NPRL3/RXRA/CA<br>N2/GTSE1/SCAP/ZFP36/SALL2/HCRTR1/PLK1/ZNF385A/LRRN2/LRP5/TICRR/PDE7A/PML/SEMA4B/BCL9L/BCL3/<br>M38/BCAR1/RTKN/NPHP4/PLPPR2/FOXC2/SEMA3F/ARRB2/EPHA2/ULK1/TCF7L1/IL1RL1/SIPA1/GIT1/PLXNA1/L<br>/PLXND1/LOC138990715/E2F1/SH3BP2/DLG4/ZYX/VAX2/SOD1/INHBA/IL18/HTR2A
GO:0007275	multicellular organism development	12	0.410079911	1.661360132	0.035288546	0.586508711	0.571977121	262	tags=92%, list=52%, signal=45%	FOSL1/KLF2/RECQL4/RXRA/ERF/KAT2A/EPHA2/GATA6/MSX1/PLXNA1/ZNF335
GO:0007346	regulation of mitotic cell cycle	21	0.336448453	1.707504318	0.02148293	0.44772317	0.43663019	160	tags=62%, list=32%, signal=44%	CDC25B/LOC102287159/MKI67/HCFC1/GTSE1/PLK1/ZNF385A/CCNB3/ESPL1/LRP5/TICRR/PML/RBM38
GO:0008219	cell death	12	0.422287398	1.710816423	0.030543988	0.548264589	0.534680552	179	tags=75%, list=36%, signal=49%	E2F2/ZNF385A/MTFP1/ESPL1/PIGT/LRP5/PML/PPP1R13L/RTKN
GO:0008283	cell population proliferation	17	0.438246999	2.04288201	0.003372062	0.163590594	0.159537404	229	tags=88%, list=46%, signal=50%	LOC102287159/MKI67/FGFR3/SIX2/RXRA/E2F8/MTCP1/KAT2A/BCAR1/FOXC2/EMX2/EPHA2/FSCN1/SCARB1/SI<br>1
GO:0008285	negative regulation of cell population proliferation	18	0.348310835	1.664488028	0.031539312	0.563314074	0.549357165	160	tags=61%, list=32%, signal=43%	FOSL1/TNS2/TFAP4/FGFRL1/NACC2/LOC102287159/ETV4/CHERP/TOB2/PML/RBM38
GO:0009141	nucleoside triphosphate metabolic process	3	-0.814	-1.746848987	0.013644217	0.335498224	0.327185777	97	tags=100%, list=19%, signal=81%	ATP5MC3/ATP5ME
GO:0009142	nucleoside triphosphate biosynthetic process	3	-0.814	-1.746848987	0.013644217	0.335498224	0.327185777	97	tags=100%, list=19%, signal=81%	ATP5MC3/ATP5ME

GO:0009144	purine nucleoside triphosphate metabolic process	3	-0.814	-1.746848987	0.013644217	0.335498224	0.327185777	97	tags=100%, list=19%, signal=81%	ATP5MC3/ATP5ME
GO:0009145	purine nucleoside triphosphate biosynthetic process	3	-0.814	-1.746848987	0.013644217	0.335498224	0.327185777	97	tags=100%, list=19%, signal=81%	ATP5MC3/ATP5ME
GO:0009152	purine ribonucleotide biosynthetic process	3	-0.814	-1.746848987	0.013644217	0.335498224	0.327185777	97	tags=100%, list=19%, signal=81%	ATP5MC3/ATP5ME
GO:0009165	nucleotide biosynthetic process	3	-0.814	-1.746848987	0.013644217	0.335498224	0.327185777	97	tags=100%, list=19%, signal=81%	ATP5MC3/ATP5ME
GO:0009199	ribonucleoside triphosphate metabolic process	3	-0.814	-1.746848987	0.013644217	0.335498224	0.327185777	97	tags=100%, list=19%, signal=81%	ATP5MC3/ATP5ME
GO:0009201	ribonucleoside triphosphate biosynthetic process	3	-0.814	-1.746848987	0.013644217	0.335498224	0.327185777	97	tags=100%, list=19%, signal=81%	ATP5MC3/ATP5ME
GO:0009205	purine ribonucleoside triphosphate metabolic process	3	-0.814	-1.746848987	0.013644217	0.335498224	0.327185777	97	tags=100%, list=19%, signal=81%	ATP5MC3/ATP5ME
GO:0009206	purine ribonucleoside triphosphate biosynthetic process	3	-0.814	-1.746848987	0.013644217	0.335498224	0.327185777	97	tags=100%, list=19%, signal=81%	ATP5MC3/ATP5ME
GO:0009260	ribonucleotide biosynthetic process	3	-0.814	-1.746848987	0.013644217	0.335498224	0.327185777	97	tags=100%, list=19%, signal=81%	ATP5MC3/ATP5ME
GO:0009615	response to virus	4	0.689378758	1.693301612	0.03712649	0.604711416	0.589728828	159	tags=100%, list=32%, signal=69%	FOSL1/LOC102285558/PML/BCL3
GO:0009653	anatomical structure morphogenesis	33	0.369329123	2.26999054	0.000942254	0.069034564	0.067324135	286	tags=91%, list=57%, signal=42%	FGFRL1/KLF2/DLX2/FGFR3/SIPA1L3/LOC138991273/SIX2/LRFN3/ZFPM1/LRFN4/NPRL3/RXRA/ETV4/SOX13/FAM<br>C/LRP5/PML/ZNF358/PPP1R13L/FOXC2/EPHA2/FSCN1/GATA6/PLXNA1/PLXND1/DLG4/ZNF335/VAX2/INHBA/SI
GO:0009719	response to endogenous stimulus	24	0.36932419	1.982466492	0.006793659	0.256728779	0.250367958	248	tags=83%, list=49%, signal=44%	FOXM1/FOSL1/TFAP4/KLF2/LOC102287159/ABCA2/LOC138991273/ABCA3/GAS2L1/JUP/ZFP36/HCRTR1/LRP5/K<br>2A/BCAR1/FOXC2/EPHA2/GATA6/LYN/E2F1
GO:0009725	response to hormone	17	0.464429128	2.164929623	0.001512724	0.096976398	0.094573669	122	tags=65%, list=24%, signal=51%	FOXM1/FOSL1/TFAP4/LOC102287159/ABCA2/LOC138991273/ABCA3/GAS2L1/ZFP36/HCRTR1/LRP5
GO:0009887	animal organ morphogenesis	11	0.454860199	1.763807314	0.025329554	0.508006133	0.495419557	122	tags=64%, list=24%, signal=49%	DLX2/FGFR3/LOC138991273/SIX2/RXRA/FAM20C/LRP5
GO:0009888	tissue development	18	0.375019697	1.792122822	0.017825018	0.406364657	0.396296393	214	tags=78%, list=43%, signal=46%	FGFR3/LOC138991273/NPRL3/RXRA/SALL2/FAM20C/LRP5/ZNF358/PPP1R13L/FAM83H/FOXC2/SEMA3F/EPHA2,<br>YRK1B
GO:0009889	regulation of biosynthetic process	123	0.242479982	2.350908414	0.000103022	0.014224957	0.013872514	237	tags=65%, list=47%, signal=46%	FOXM1/ZNF865/MAMSTR/SP2/FOSL1/ZMIZ2/FOXK1/TFAP4/LOC106701625/ZNF142/KLF2/NCOR2/NACC2/SLC2<br>RG/DLX2/POM121C/ABCA2/LOC138991273/SIX2/ZFPM1/ATN1/HCFC1/GLIS2/RXRA/SETD1A/ETV4/MYBL2/ERF/S<br>45A3/ZNF575/GAS2L1/JUP/ZFP36/E2F8/CIC/SALL2/PLK1/ZNF385A/PER1/CERS1/RNF44/TOB2/SOX12/KMT5C/LR<br>/MRTFA/ZNF574/PHF19/NFIC/KAT2A/PML/PSRC1/BCL9L/ZNF358/BCL3/RBM38/ASF1B/PPP1R13L/ZNF438/KDM
GO:0009890	negative regulation of biosynthetic process	52	0.244725145	1.807115244	0.009466388	0.335498224	0.327185777	108	tags=40%, list=21%, signal=35%	FOXK1/TFAP4/LOC106701625/KLF2/NCOR2/NACC2/DLX2/POM121C/ZFPM1/HCFC1/GLIS2/RXRA/ERF/GAS2L1/Z<br>36/E2F8/CIC/SALL2/PLK1/PER1/CERS1
GO:0009891	positive regulation of biosynthetic process	58	0.261770515	1.997666711	0.003200195	0.161812701	0.157803561	160	tags=52%, list=32%, signal=40%	FOXM1/MAMSTR/FOSL1/ZMIZ2/FOXK1/TFAP4/LOC106701625/KLF2/NCOR2/LOC138991273/SIX2/ZFPM1/GLIS2<br>XRA/ETV4/MYBL2/SLC45A3/ZFP36/E2F8/PER1/SOX12/LRP5/MRTFA/NFIC/KAT2A/PML/PSRC1/BCL9L/BCL3/RBM

GO:0009892	negative regulation of metabolic process	59	0.240832051	1.860009652	0.00840679	0.311137905	0.30342902	266	tags=75%, list=53%, signal=40%	FOXK1/TFAP4/LOC106701625/KLF2/NCOR2/NACC2/DLX2/POM121C/LOC138991273/ZFPM1/HCFC1/GLIS2/RXRA<br>RF/GAS2L1/ZFP36/E2F8/CIC/SALL2/PLK1/PER1/CERS1/LRP5/MRTFA/PHF19/NFIC/PML/BCL3/RBM38/PPP1R13L/<br>F438/FOXC2/EPHA2/USP2/PHF12/BCL7A/IL1RL1/GATA6/LYN/TSC22D4/E2F1/MLLT1/DCP1B/UHRF1
GO:0009893	positive regulation of metabolic process	74	0.23513579	1.963675025	0.005426873	0.21647194	0.211108539	160	tags=49%, list=32%, signal=39%	FOXM1/CEMIP/MAMSTR/FOSL1/ZMIZ2/FOXK1/TFAP4/LOC106701625/KLF2/NCOR2/CDC25B/FGFR3/WIZ/LOC13<br>91273/SIX2/ZFPM1/GLIS2/RXRA/ETV4/MYBL2/SLC45A3/ZFP36/E2F8/PLK1/PER1/SOX12/LRP5/PNPLA2/MRTFA/N<br>C/KAT2A/PML/PSRC1/BCL9L/BCL3/RBM38
GO:0009987	cellular process	332	0.159175829	1.738569831	0.013421375	0.335498224	0.327185777	256	tags=57%, list=51%, signal=82%	MASP1/FOXRED2/FOXM1/ARHGAP33/ZNF865/TROAP/STX1A/ISYNA1/SYN1/CEMIP/MAMSTR/KIF18B/SP2/FOSL1<br>MIZ2/FOXK1/TNS2/TFAP4/FGFRL1/CLIC5/LOC106701625/E2F2/ZNF142/KLF2/NCOR2/CCNF/CDC25B/TMEM201/<br>ACC2/LOC102269246/LOC102287159/SLC2A4RG/PTCH2/RAVER1/NACAD/LOC102274386/MKI67/DLX2/POM121<br>FGFR3/RECQL4/UROC1/WIZ/SIPA1L3/LOC138991273/SIX2/LRFN3/ZFPM1/ATN1/HCFC1/MDGA1/LRFN4/ABCA3/
GO:0010033	response to organic substance	43	0.237790831	1.636826619	0.040453478	0.607954898	0.592891949	108	tags=40%, list=21%, signal=34%	FOXRED2/FOXM1/FOSL1/TFAP4/KLF2/LOC102287159/MKI67/ABCA2/LOC138991273/ABCA3/RXRA/GAS2L1/JUP<br>FP36/HCRTR1/PER1/CERS1
GO:0010243	response to organonitrogen compound	13	0.56446477	2.367487956	0.000539088	0.045781071	0.04464678	122	tags=77%, list=24%, signal=60%	FOXRED2/FOXM1/FOSL1/KLF2/LOC102287159/LOC138991273/JUP/HCRTR1/PER1/LRP5
GO:0010468	regulation of gene expression	118	0.230232115	2.223371735	0.000169627	0.018727607	0.018263604	237	tags=64%, list=47%, signal=44%	FOXM1/ZNF865/MAMSTR/SP2/FOSL1/ZMIZ2/FOXK1/TFAP4/ZNF142/KLF2/NCOR2/NACC2/SLC2A4RG/DLX2/POM<br>21C/ABCA2/LOC138991273/SIX2/ZFPM1/ATN1/HCFC1/GLIS2/RXRA/SETD1A/ETV4/MYBL2/ERF/ZNF575/GAS2L1/<br>P/ZFP36/E2F8/CIC/SALL2/PLK1/ZNF385A/PER1/RNF44/TOB2/SOX12/KMT5C/LRP5/MRTFA/ZNF574/PHF19/NFIC<br>AT2A/PML/PSRC1/BCL9L/ZNF358/BCL3/RBM38/ASF1B/PPP1R13L/ZNF438/KDM6B/CAMTA2/SNAPC4/FOXC2/EN
GO:0010556	regulation of macromolecule biosynthetic process	120	0.2396657	2.319238347	7.52E-05	0.011687013	0.011397451	237	tags=65%, list=47%, signal=45%	FOXM1/ZNF865/MAMSTR/SP2/FOSL1/ZMIZ2/FOXK1/TFAP4/LOC106701625/ZNF142/KLF2/NCOR2/NACC2/SLC2<br>RG/DLX2/POM121C/ABCA2/LOC138991273/SIX2/ZFPM1/ATN1/HCFC1/GLIS2/RXRA/SETD1A/ETV4/MYBL2/ERF/<br>F575/GAS2L1/JUP/ZFP36/E2F8/CIC/SALL2/PLK1/ZNF385A/PER1/CERS1/RNF44/TOB2/SOX12/KMT5C/LRP5/MRTF<br>ZNF574/PHF19/NFIC/KAT2A/PML/PSRC1/BCL9L/ZNF358/BCL3/RBM38/ASF1B/PPP1R13L/ZNF438/KDM6B/CAMT
GO:0010557	positive regulation of macromolecule biosynthetic process	55	0.270231908	2.016427159	0.004128687	0.18298748	0.178453704	160	tags=53%, list=32%, signal=40%	FOXM1/MAMSTR/FOSL1/ZMIZ2/FOXK1/TFAP4/LOC106701625/KLF2/NCOR2/LOC138991273/SIX2/ZFPM1/GLIS2<br>XRA/ETV4/MYBL2/ZFP36/E2F8/PER1/SOX12/LRP5/MRTFA/NFIC/KAT2A/PML/PSRC1/BCL9L/BCL3/RBM38
GO:0010558	negative regulation of macromolecule biosynthetic process	51	0.254842541	1.884101104	0.01037358	0.335498224	0.327185777	215	tags=65%, list=43%, signal=41%	FOXK1/TFAP4/LOC106701625/KLF2/NCOR2/NACC2/DLX2/POM121C/ZFPM1/HCFC1/GLIS2/RXRA/ERF/GAS2L1/Z<br>36/E2F8/CIC/SALL2/PLK1/PER1/CERS1/PHF19/NFIC/PML/BCL3/PPP1R13L/ZNF438/FOXC2/EPHA2/USP2/PHF12/B<br>7A/IL1RL1
GO:0010564	regulation of cell cycle process	22	0.453485015	2.352064686	0.00033277	0.033481422	0.032651872	160	tags=73%, list=32%, signal=52%	CCNF/CDC25B/LOC102287159/MKI67/WIZ/GTSE1/E2F8/PLK1/ZNF385A/CCNB3/ESPL1/LRP5/TICRR/PML/PSRC1/<br>M38
GO:0010604	positive regulation of macromolecule metabolic process	67	0.259193564	2.081970387	0.001417633	0.092532753	0.090240122	160	tags=51%, list=32%, signal=40%	FOXM1/CEMIP/MAMSTR/FOSL1/ZMIZ2/FOXK1/TFAP4/LOC106701625/KLF2/NCOR2/CDC25B/FGFR3/WIZ/LOC13<br>91273/SIX2/ZFPM1/GLIS2/RXRA/ETV4/MYBL2/ZFP36/E2F8/PLK1/PER1/SOX12/LRP5/MRTFA/NFIC/KAT2A/PML/<br>RC1/BCL9L/BCL3/RBM38
GO:0010605	negative regulation of macromolecule metabolic process	57	0.268409953	2.041573147	0.002916878	0.158660506	0.154729465	266	tags=77%, list=53%, signal=41%	FOXK1/TFAP4/LOC106701625/KLF2/NCOR2/NACC2/DLX2/POM121C/LOC138991273/ZFPM1/HCFC1/GLIS2/RXRA<br>RF/GAS2L1/ZFP36/E2F8/CIC/SALL2/PLK1/PER1/CERS1/LRP5/MRTFA/PHF19/NFIC/PML/BCL3/RBM38/PPP1R13L/<br>F438/FOXC2/EPHA2/USP2/PHF12/BCL7A/IL1RL1/GATA6/LYN/TSC22D4/E2F1/MLLT1/DCP1B/UHRF1
GO:0010608	post-transcriptional regulation of gene expression	11	0.532694833	2.065625977	0.004730253	0.20709278	0.201961761	160	tags=82%, list=32%, signal=57%	NACC2/LOC138991273/RXRA/ZFP36/ZNF385A/PER1/PML/BCL3/RBM38
GO:0010638	positive regulation of organelle organization	9	0.516086729	1.831316708	0.017839056	0.406364657	0.396296393	152	tags=78%, list=30%, signal=55%	SYNPO2L/LOC106701625/LOC138991273/ESPL1/LRP5/PML/PSRC1
GO:0010701	positive regulation of norepinephrine secretion	1	0.972111554	1.316217582	0.049212598	0.667515691	0.650977038	15	tags=100%, list=3%, signal=97%	STX1A
GO:0010721	negative regulation of cell development	6	0.535211268	1.574397685	0.040626192	0.607954898	0.592891949	237	tags=100%, list=47%, signal=54%	DLX2/ZFPM1/TOB2/SEMA4B/SEMA3F/LYN
GO:0010831	positive regulation of myotube differentiation	2	0.876247505	1.575509482	0.039316641	0.607954898	0.592891949	64	tags=100%, list=13%, signal=88%	MAMSTR/LOC138991273
GO:0010874	regulation of cholesterol efflux	2	-0.876247505	-1.582990055	0.027551693	0.517292844	0.504476177	65	tags=100%, list=13%, signal=87%	SIRT1

GO:0010875	positive regulation of cholesterol efflux	2	-0.876247505	-1.582990055	0.027551693	0.517292844	0.504476177	65	tags=100%, list=13%, signal=87%	SIRT1
GO:0010948	negative regulation of cell cycle process	12	0.550067297	2.228492187	0.001401425	0.092532753	0.090240122	160	tags=83%, list=32%, signal=58%	CCNF/LOC102287159/GTSE1/E2F8/PLK1/ZNF385A/ESPL1/TICRR/PML/RBM38
GO:0012501	programmed cell death	12	0.422287398	1.710816423	0.030543988	0.548264589	0.534680552	179	tags=75%, list=36%, signal=49%	E2F2/ZNF385A/MTFP1/ESPL1/PIGT/LRP5/PML/PPP1R13L/RTKN
GO:0014061	regulation of norepinephrine secretion	1	0.972111554	1.316217582	0.049212598	0.667515691	0.650977038	15	tags=100%, list=3%, signal=97%	STX1A
GO:0014070	response to organic cyclic compound	22	0.349672912	1.813628415	0.013112688	0.335498224	0.327185777	107	tags=50%, list=21%, signal=41%	FOXM1/FOSL1/TFAP4/KLF2/MKI67/ABCA2/LOC138991273/ABCA3/JUP/ZFP36/PER1
GO:0016070	RNA metabolic process	84	0.24038498	2.04726036	0.001236962	0.088813903	0.086613412	168	tags=51%, list=33%, signal=41%	FOXM1/ZNF865/MAMSTR/SP2/FOSL1/ZMIZ2/FOXK1/LOC106701625/E2F2/ZNF142/KLF2/NCOR2/NACC2/SLC2A4<br>G/RAVER1/LOC102274386/ZFPM1/ATN1/RXRA/SETD1A/SOX13/ERF/ZNF575/ZFP36/E2F8/CIC/SALL2/CHERP/ZN<br>85A/PER1/KMT5C/LIPE/ZNF574/PHF19/NFIC/KAT2A/PML/BCL9L/ZNF358/BCL3/RBM38/ASF1B/PPP1R13L
GO:0016072	rRNA metabolic process	6	-0.820925553	-2.364763872	0.000128888	0.0159555	0.01556018	96	tags=100%, list=19%, signal=82%	RPL34/RPS19/SIRT1/RPS28/LOC102265456
GO:0016310	phosphorylation	10	0.529411765	1.983293649	0.011187363	0.335498224	0.327185777	242	tags=100%, list=48%, signal=53%	CDC25B/FGFR3/LOC138991273/PLK1/FAM20C/ULK1/MARK4/DYRK1B/LYN/LOC138990715
GO:0018130	heterocycle biosynthetic process	75	0.232725575	1.935781516	0.005284723	0.21647194	0.211108539	168	tags=51%, list=33%, signal=40%	FOXM1/ZNF865/MAMSTR/SP2/FOSL1/ZMIZ2/FOXK1/E2F2/ZNF142/KLF2/NCOR2/NACC2/SLC2A4RG/LOC138991<br>3/ZFPM1/ATN1/RXRA/SETD1A/SOX13/ERF/ZNF575/E2F8/CIC/SALL2/ZNF385A/PER1/KMT5C/LIPE/ZNF574/PHF1<br>NFIC/KAT2A/PML/BCL9L/ZNF358/BCL3/ASF1B/PPP1R13L
GO:0018149	peptide cross-linking	1	1	1.353977923	0.001831796	0.113381857	0.11057266	1	tags=100%, list=0%, signal=100%	TGM3
GO:0019219	regulation of nucleobase-containing compound metabolic process	107	0.296236533	2.780206296	2.82339E-06	0.001266994	0.001235603	234	tags=69%, list=47%, signal=47%	FOXM1/ZNF865/MAMSTR/SP2/FOSL1/ZMIZ2/FOXK1/TFAP4/LOC106701625/ZNF142/KLF2/NCOR2/NACC2/SLC2<br>RG/DLX2/WIZ/ABCA2/LOC138991273/SIX2/ZFPM1/ATN1/HCFC1/GLIS2/RXRA/SETD1A/ETV4/MYBL2/ERF/ZNF57<br>UP/ZFP36/E2F8/CIC/SALL2/PLK1/ZNF385A/PER1/CERS1/RNF44/SOX12/KMT5C/LRP5/MRTFA/TICRR/ZNF574/PH<br>9/NFIC/KAT2A/PML/PSRC1/BCL9L/ZNF358/BCL3/RBM38/ASF1B/PPP1R13L/ZNF438/KDM6B/CAMTA2/SNAPC4/
GO:0019222	regulation of metabolic process	143	0.228829453	2.266968225	0.000168839	0.018727607	0.018263604	237	tags=63%, list=47%, signal=47%	FOXM1/ZNF865/CEMIP/MAMSTR/SP2/FOSL1/ZMIZ2/FOXK1/TFAP4/LOC106701625/ZNF142/KLF2/NCOR2/CDC2<br>/NACC2/SLC2A4RG/DLX2/POM121C/FGFR3/WIZ/ABCA2/LOC138991273/SIX2/ZFPM1/ATN1/HCFC1/GLIS2/RXRA<br>ETD1A/ETV4/MYBL2/ERF/SLC45A3/ZNF575/GAS2L1/JUP/ZFP36/E2F8/CIC/SALL2/PLK1/ZNF385A/PER1/CERS1/C<br>B3/RNF44/FAM20C/TOB2/SOX12/KMT5C/LRP5/PNPLA2/MRTFA/TICRR/ZNF574/PHF19/NFIC/KAT2A/PML/PSRC1
GO:0019438	aromatic compound biosynthetic process	73	0.233191347	1.942018386	0.0048969	0.211805676	0.206557889	168	tags=51%, list=33%, signal=39%	FOXM1/ZNF865/MAMSTR/SP2/FOSL1/ZMIZ2/FOXK1/E2F2/ZNF142/KLF2/NCOR2/NACC2/SLC2A4RG/ZFPM1/ATN<br>RXRA/SETD1A/SOX13/ERF/ZNF575/E2F8/CIC/SALL2/ZNF385A/PER1/KMT5C/LIPE/ZNF574/PHF19/NFIC/KAT2A/P<br>/BCL9L/ZNF358/BCL3/ASF1B/PPP1R13L
GO:0019731	antibacterial humoral response	2	-0.832335329	-1.503660258	0.046194156	0.667515691	0.650977038	87	tags=100%, list=17%, signal=83%	RPL39
GO:0019827	stem cell population maintenance	6	0.531354163	1.56305148	0.049309665	0.667515691	0.650977038	157	tags=83%, list=31%, signal=58%	SIX2/SETD1A/LRP5/BCL9L/ZNF358
GO:0021700	developmental maturation	5	0.621345447	1.68555454	0.038275671	0.607954898	0.592891949	113	tags=80%, list=22%, signal=63%	KLF2/CDC25B/FGFR3/FAM20C
GO:0022402	cell cycle process	25	0.464675999	2.519997979	7.81305E-05	0.011687013	0.011397451	172	tags=76%, list=34%, signal=53%	FOXM1/KIF18B/CCNF/CDC25B/LOC102287159/POM121C/MYBL2/GTSE1/PLK1/INCENP/ZNF385A/ESPL1/TICRR/<br>APH/CEP72/PML/PSRC1/RBM38/NCAPD3
GO:0022613	ribonucleoprotein complex biogenesis	4	-0.811623246	-1.970012929	0.003329019	0.163590594	0.159537404	99	tags=100%, list=20%, signal=81%	RPL34/RPS28/LOC102265456

GO:0022618	protein-RNA complex assembly	12	-0.45285294	-1.765914824	0.027956898	0.517292844	0.504476177	210	tags=83%, list=42%, signal=50%	RPL24/GEMIN2/RPS17/RPS27/LOC102275123/RPS19/RPS28/LOC102279476/LOC102285651/LOC102265456
GO:0022900	electron transport chain	5	-0.831325301	-2.230869522	0.000504356	0.045265922	0.044144394	90	tags=100%, list=18%, signal=83%	NDUFS4/UQCRH/NDUFB3/NDUFA5
GO:0022904	respiratory electron transport chain	3	-0.828	-1.776893073	0.01050108	0.335498224	0.327185777	90	tags=100%, list=18%, signal=83%	UQCRH/NDUFA5
GO:0030030	cell projection organization	21	0.375099181	1.903660023	0.006542367	0.250855314	0.244640016	276	tags=90%, list=55%, signal=43%	CLIC5/LOC138991273/LRFN3/LRFN4/ETV4/MRTFA/SEMA3F/ULK1/MARK4/FSCN1/MTSS2/DPYSL4/PLXNA1/LYN/ XND1/DLG4/ZNF335/VAX2/SOD1
GO:0030154	cell differentiation	52	0.223917358	1.653465038	0.021628093	0.44772317	0.43663019	227	tags=65%, list=45%, signal=40%	FOXK1/KLF2/FGFR3/SIPA1L3/SIX2/ZFPM1/MDGA1/GLIS2/RXRA/ETV4/MYBL2/ERF/E2F8/ZNF385A/FAM20C/PIG MRTFA/KAT2A/CLN8/PML/BCL9L/BCL3/RBM38/KDM6B/SNAPC4/SNPH/FOXC2/EMX2/EPHA2/NLGN4X/PRRC2A/ MP15/WDR62/GATA6
GO:0030212	hyaluronan metabolic process	2	0.898203593	1.614986941	0.025195391	0.508006133	0.495419557	53	tags=100%, list=11%, signal=90%	CEMIP/MKI67
GO:0030330	DNA damage response, signal transduction by p53 class mediator	8	0.585448066	1.956711535	0.01011684	0.335498224	0.327185777	160	tags=88%, list=32%, signal=61%	TFAP4/LOC102287159/GTSE1/ZNF385A/PML/BCL3/RBM38
GO:0030449	regulation of complement activation	1	0.992031873	1.343189254	0.013407276	0.335498224	0.327185777	5	tags=100%, list=1%, signal=99%	MASP1
GO:0030490	maturation of SSU-rRNA	3	-0.864	-1.854149293	0.005366907	0.21647194	0.211108539	72	tags=100%, list=14%, signal=86%	RPS28/LOC102265456
GO:0030855	epithelial cell differentiation	9	0.558704453	1.982544293	0.006568356	0.250855314	0.244640016	227	tags=100%, list=45%, signal=56%	KLF2/SIX2/RXRA/E2F8/FAM20C/KDM6B/FOXC2/EPHA2/GATA6
GO:0031323	regulation of cellular metabolic process	142	0.234934642	2.352420588	0.00017097	0.018727607	0.018263604	237	tags=63%, list=47%, signal=47%	FOXM1/ZNF865/CEMIP/MAMSTR/SP2/FOSL1/ZMIZ2/FOXK1/TFAP4/LOC106701625/ZNF142/KLF2/NCOR2/CDC2 /NACC2/SLC2A4RG/DLX2/POM121C/FGFR3/WIZ/ABCA2/LOC138991273/SIX2/ZFPM1/ATN1/HCFC1/GLIS2/RXRA ETD1A/ETV4/MYBL2/ERF/SLC45A3/ZNF575/GAS2L1/JUP/ZFP36/E2F8/CIC/SALL2/PLK1/ZNF385A/PER1/CERS1/C B3/RNF44/FAM20C/TOB2/SOX12/KMT5C/LRP5/PNPLA2/MRTFA/TICRR/ZNF574/PHF19/NFIC/KAT2A/PML/PSRC1
GO:0031324	negative regulation of cellular metabolic process	56	0.263225012	1.981300482	0.005378741	0.21647194	0.211108539	266	tags=77%, list=53%, signal=41%	FOXK1/TFAP4/LOC106701625/KLF2/NCOR2/NACC2/DLX2/POM121C/LOC138991273/ZFPM1/HCFC1/GLIS2/RXRA RF/GAS2L1/ZFP36/E2F8/CIC/SALL2/PLK1/PER1/CERS1/LRP5/PHF19/NFIC/PML/BCL3/RBM38/PPP1R13L/ZNF438/ OXC2/EPHA2/USP2/PHF12/BCL7A/IL1RL1/GATA6/LYN/TSC22D4/E2F1/MLLT1/DCP1B/UHRF1
GO:0031325	positive regulation of cellular metabolic process	71	0.258751491	2.132577609	0.001375208	0.092532753	0.090240122	160	tags=51%, list=32%, signal=40%	FOXM1/CEMIP/MAMSTR/FOSL1/ZMIZ2/FOXK1/TFAP4/LOC106701625/KLF2/NCOR2/CDC25B/FGFR3/WIZ/LOC13 91273/SIX2/ZFPM1/GLIS2/RXRA/ETV4/MYBL2/SLC45A3/ZFP36/E2F8/PLK1/PER1/SOX12/LRP5/PNPLA2/MRTFA/N C/KAT2A/PML/PSRC1/BCL9L/BCL3/RBM38
GO:0031326	regulation of cellular biosynthetic process	123	0.242479982	2.350908414	0.000103022	0.014224957	0.013872514	237	tags=65%, list=47%, signal=46%	FOXM1/ZNF865/MAMSTR/SP2/FOSL1/ZMIZ2/FOXK1/TFAP4/LOC106701625/ZNF142/KLF2/NCOR2/NACC2/SLC2 RG/DLX2/POM121C/ABCA2/LOC138991273/SIX2/ZFPM1/ATN1/HCFC1/GLIS2/RXRA/SETD1A/ETV4/MYBL2/ERF/S 45A3/ZNF575/GAS2L1/JUP/ZFP36/E2F8/CIC/SALL2/PLK1/ZNF385A/PER1/CERS1/RNF44/TOB2/SOX12/KMT5C/LR /MRTFA/ZNF574/PHF19/NFIC/KAT2A/PML/PSRC1/BCL9L/ZNF358/BCL3/RBM38/ASF1B/PPP1R13L/ZNF438/KDM
GO:0031327	negative regulation of cellular biosynthetic process	51	0.254842541	1.884101104	0.01037358	0.335498224	0.327185777	215	tags=65%, list=43%, signal=41%	FOXK1/TFAP4/LOC106701625/KLF2/NCOR2/NACC2/DLX2/POM121C/ZFPM1/HCFC1/GLIS2/RXRA/ERF/GAS2L1/Z 36/E2F8/CIC/SALL2/PLK1/PER1/CERS1/PHF19/NFIC/PML/BCL3/PPP1R13L/ZNF438/FOXC2/EPHA2/USP2/PHF12/B 7A/IL1RL1
GO:0031328	positive regulation of cellular biosynthetic process	58	0.261770515	1.997666711	0.003200195	0.161812701	0.157803561	160	tags=52%, list=32%, signal=40%	FOXM1/MAMSTR/FOSL1/ZMIZ2/FOXK1/TFAP4/LOC106701625/KLF2/NCOR2/LOC138991273/SIX2/ZFPM1/GLIS2 XRA/ETV4/MYBL2/SLC45A3/ZFP36/E2F8/PER1/SOX12/LRP5/MRTFA/NFIC/KAT2A/PML/PSRC1/BCL9L/BCL3/RBM
GO:0031348	negative regulation of defense response	3	-0.864	-1.854149293	0.005295823	0.21647194	0.211108539	72	tags=100%, list=14%, signal=86%	TNFAIP6/C1QTNF3
GO:0031424	keratinization	1	1	1.353977923	0.001831796	0.113381857	0.11057266	1	tags=100%, list=0%, signal=100%	TGM3

GO:0031570	DNA integrity checkpoint signaling	8	0.582962541	1.948404299	0.010707594	0.335498224	0.327185777	160	tags=88%, list=32%, signal=61%	LOC102287159/GTSE1/PLK1/ZNF385A/TICRR/PML/RBM38
GO:0031571	mitotic G1 DNA damage checkpoint signaling	6	0.544063929	1.600439009	0.034695476	0.579334539	0.5649807	160	tags=83%, list=32%, signal=58%	LOC102287159/GTSE1/ZNF385A/PML/RBM38
GO:0031629	synaptic vesicle fusion to presynaptic active zone membrane	1	0.972111554	1.316217582	0.049212598	0.667515691	0.650977038	15	tags=100%, list=3%, signal=97%	STX1A
GO:0031960	response to corticosteroid	5	0.650962015	1.765896871	0.021176656	0.44720115	0.436121104	97	tags=80%, list=19%, signal=65%	FOSL1/TFAP4/ABCA3/ZFP36
GO:0032370	positive regulation of lipid transport	2	-0.876247505	-1.582990055	0.027551693	0.517292844	0.504476177	65	tags=100%, list=13%, signal=87%	SIRT1
GO:0032373	positive regulation of sterol transport	2	-0.876247505	-1.582990055	0.027551693	0.517292844	0.504476177	65	tags=100%, list=13%, signal=87%	SIRT1
GO:0032376	positive regulation of cholesterol transport	2	-0.876247505	-1.582990055	0.027551693	0.517292844	0.504476177	65	tags=100%, list=13%, signal=87%	SIRT1
GO:0032386	regulation of intracellular transport	3	0.814	1.749322145	0.017884573	0.406364657	0.396296393	96	tags=100%, list=19%, signal=81%	ABCA2/GTSE1/JUP
GO:0032501	multicellular organismal process	65	0.255552803	2.021040395	0.002156769	0.126931186	0.123786285	281	tags=78%, list=56%, signal=40%	TGM3/CEMIP/SP2/FOSL1/TNS2/CLIC5/KLF2/PTCH2/DLX2/FGFR3/RECQL4/LOC138991273/SIX2/ZFPM1/RXRA/SE<br>1A/ETV4/ERF/PCSK6/HCRTR1/ZNF385A/LRP5/KAT2A/CLN8/PML/BCL9L/ZNF358/BCL3/PPP1R13L/CAMTA2/NPHP<br>PCDH17/FOXC2/EMX2/EPHA2/USP2/NLGN4X/SCARB1/GATA6/MSX1/AUTS2/PLXNA1/LYN/PLXND1/DLG4/ZNF33<br>VAX2/SOD1/INHBA/IL18/HTR2A
GO:0032502	developmental process	109	0.288813278	2.729585331	5.95299E-06	0.001643941	0.00160321	279	tags=79%, list=55%, signal=45%	FOSL1/FOXK1/TNS2/FGFRL1/KLF2/CCNF/CDC25B/LOC102287159/PTCH2/MKI67/DLX2/FGFR3/RECQL4/SIPA1L3/<br>C138991273/SIX2/LRFN3/ZFPM1/MDGA1/LRFN4/GLIS2/NPRL3/RXRA/SETD1A/ETV4/MYBL2/SOX13/ERF/JUP/E2<br>/CIC/SALL2/CHERP/ZNF385A/FAM20C/SOX12/PIGT/LRP5/MRTFA/TESK1/KAT2A/CLN8/PML/BCL9L/ZNF358/BCL3<br>BM38/PPP1R13L/KDM6B/FAM83H/NPHP4/SNAPC4/SNPH/FOXC2/EMX2/SEMA3F/EPHA2/ULK1/USP2/NLGN4X/
GO:0032774	RNA biosynthetic process	69	0.278087504	2.266807678	0.000544273	0.045781071	0.04464678	266	tags=77%, list=53%, signal=42%	FOXM1/ZNF865/MAMSTR/SP2/FOSL1/ZMIZ2/FOXK1/E2F2/ZNF142/KLF2/NCOR2/NACC2/SLC2A4RG/ZFPM1/ATN<br>RXRA/SETD1A/SOX13/ERF/ZNF575/E2F8/CIC/SALL2/ZNF385A/PER1/KMT5C/LIPE/ZNF574/PHF19/NFIC/KAT2A/P<br>/BCL9L/ZNF358/BCL3/ASF1B/PPP1R13L/ZNF438/SNAPC4/TCF7L1/PHF12/SRCAP/ZNF341/GATA6/MSX1/PPRC1/<br>F414/TSC22D4/E2F1/MLLT1/ZNF335/VAX2/UHRF1
GO:0032870	cellular response to hormone stimulus	7	0.564828202	1.769198216	0.0188543	0.412725217	0.402499361	102	tags=71%, list=20%, signal=58%	TFAP4/LOC102287159/GAS2L1/ZFP36/HCRTR1
GO:0032984	protein-containing complex disassembly	3	0.762	1.637571836	0.045322726	0.66683847	0.650316596	122	tags=100%, list=24%, signal=76%	KIF18B/NCKAP5L/LRP5
GO:0033043	regulation of organelle organization	25	0.305871795	1.658782265	0.032878561	0.575424155	0.561167201	152	tags=56%, list=30%, signal=41%	SYNPO2L/LOC106701625/CCNF/MKI67/LOC138991273/NPRL3/ATXN2L/GAS2L1/PLK1/ESPL1/LRP5/KAT2A/PML/<br>RC1
GO:0033108	mitochondrial respiratory chain complex assembly	4	-0.681362725	-1.653838014	0.035531211	0.587820494	0.573256403	164	tags=100%, list=33%, signal=68%	UQCR10/NDUFS4/COX17
GO:0033554	cellular response to stress	40	0.251689401	1.692661274	0.019299133	0.419294166	0.408905555	199	tags=60%, list=40%, signal=39%	FOXRED2/FOXM1/TFAP4/KLF2/LOC102287159/MKI67/RECQL4/LOC138991273/NPRL3/GTSE1/SCAP/GAS2L1/VA<br>/PLK1/ZNF385A/TICRR/CLN8/PML/BCL3/RBM38/KDM6B/POLD1/EPHA2/ULK1
GO:0033605	positive regulation of catecholamine secretion	1	0.972111554	1.316217582	0.049212598	0.667515691	0.650977038	15	tags=100%, list=3%, signal=97%	STX1A
GO:0034248	regulation of amide metabolic process	8	0.580246574	1.939326869	0.011298349	0.335498224	0.327185777	159	tags=88%, list=32%, signal=61%	LOC138991273/RXRA/ZFP36/ZNF385A/PER1/PML/BCL3

GO:0034470	ncRNA processing	7	-0.715868096	-2.212362242	0.000900759	0.067369231	0.065700062	96	tags=86%, list=19%, signal=70%	LOC138987848/RPL34/RPS19/SIRT1/RPS28/LOC102265456
GO:0034654	nucleobase-containing compound biosynthetic process	72	0.240076969	1.986413627	0.005084094	0.21647194	0.211108539	168	tags=51%, list=33%, signal=40%	FOXM1/ZNF865/MAMSTR/SP2/FOSL1/ZMIZ2/FOXK1/E2F2/ZNF142/KLF2/NCOR2/NACC2/SLC2A4RG/ZFPM1/ATN<br>RXRA/SETD1A/SOX13/ERF/ZNF575/E2F8/CIC/SALL2/ZNF385A/PER1/KMT5C/LIPE/ZNF574/PHF19/NFIC/KAT2A/P<br>/BCL9L/ZNF358/BCL3/ASF1B/PPP1R13L
GO:0034660	ncRNA metabolic process	10	-0.506974982	-1.821389684	0.013513236	0.335498224	0.327185777	120	tags=70%, list=24%, signal=54%	POLR2K/LOC138987848/RPL34/RPS19/SIRT1/RPS28/LOC102265456
GO:0034728	nucleosome organization	1	-0.988047809	-1.329247561	0.023074547	0.468009166	0.456413572	8	tags=100%, list=2%, signal=99%	
GO:0035264	multicellular organism growth	6	0.674044266	1.982794077	0.00398113	0.178653189	0.174226801	168	tags=100%, list=33%, signal=67%	SP2/TNS2/KLF2/LOC138991273/KAT2A/PPP1R13L
GO:0035356	intracellular triglyceride homeostasis	2	-0.888223553	-1.604625455	0.022412569	0.459777836	0.448386185	59	tags=100%, list=12%, signal=89%	C1QTNF3
GO:0036503	ERAD pathway	1	0.990039841	1.340492087	0.02017349	0.43366964	0.422924856	6	tags=100%, list=1%, signal=99%	FOXRED2
GO:0040007	growth	13	0.394504909	1.654639351	0.044648322	0.659619242	0.643276235	236	tags=85%, list=47%, signal=46%	SP2/TNS2/KLF2/FGFR3/LOC138991273/KAT2A/PPP1R13L/SEMA3F/ULK1/NLGN4X/PLXNA1
GO:0042127	regulation of cell population proliferation	38	0.263567181	1.729156478	0.014871626	0.363191414	0.354192829	133	tags=47%, list=26%, signal=38%	FOSL1/TNS2/TFAP4/FGFRL1/CDC25B/NACC2/LOC102287159/FGFR3/LOC138991273/SIX2/SETD1A/ETV4/JUP/Z<br>6/CHERP/TOB2/LRP5/LOC102285558
GO:0042254	ribosome biogenesis	3	-0.844	-1.811229171	0.00835804	0.311137905	0.30342902	82	tags=100%, list=16%, signal=84%	RPS28/LOC102265456
GO:0042770	signal transduction in response to DNA damage	9	0.598559286	2.123967848	0.002566436	0.146246087	0.142622631	160	tags=89%, list=32%, signal=62%	TFAP4/LOC102287159/GTSE1/PLK1/ZNF385A/PML/BCL3/RBM38
GO:0043043	peptide biosynthetic process	31	-0.53952513	-3.100115382	1.57559E-07	0.000113127	0.000110324	210	tags=90%, list=42%, signal=56%	RPL24/RPL27A/RPL37A/RPL23/RPS17/RPS27/LOC102266576/LOC102286668/RPS29/RPL36A/RPLP2/RPL35A/LO<br>38987848/RPL34/LOC102275123/RPS19/MRPS18C/RPL36AL/RPL39/LOC102269867/LOC106701537/RPL22/RPS2<br>LOC102279476/LOC102285651/LOC102281282/LOC102265456/LOC102270678
GO:0043603	amide metabolic process	40	-0.399902011	-2.536074505	6.37718E-05	0.010406402	0.010148569	229	tags=80%, list=46%, signal=47%	SOD1/RPS23/MCEE/RPL24/CARNMT1/RPL27A/RPL37A/RPL23/RPS17/RPS27/LOC102266576/LOC102286668/RP<br>9/RPL36A/RPLP2/RPL35A/LOC138987848/RPL34/LOC102275123/RPS19/MRPS18C/RPL36AL/RPL39/LOC102269<br>7/LOC106701537/RPL22/RPS28/LOC102279476/LOC102285651/LOC102281282/LOC102265456/LOC10227067
GO:0043604	amide biosynthetic process	32	-0.51602135	-3.01590654	1.34021E-06	0.000801895	0.000782027	162	tags=75%, list=32%, signal=54%	RPS17/RPS27/LOC102266576/LOC102286668/RPS29/RPL36A/RPLP2/RPL35A/LOC138987848/RPL34/LOC102275<br>3/RPS19/MRPS18C/RPL36AL/RPL39/LOC102269867/LOC106701537/RPL22/RPS28/LOC102279476/LOC102285<br>/LOC102281282/LOC102265456/LOC102270678
GO:0044085	cellular component biogenesis	5	-0.671126174	-1.800973607	0.027081535	0.517292844	0.504476177	99	tags=80%, list=20%, signal=65%	RPL35A/RPL34/RPS28/LOC102265456
GO:0044238	primary metabolic process	190	0.139577547	1.51701404	0.046782462	0.667515691	0.650977038	149	tags=37%, list=30%, signal=42%	TGM3/FOXRED2/FOXM1/ZDHHC8/ZNF865/ISYNA1/MAMSTR/SP2/FOSL1/ZMIZ2/FOXK1/LOC106701625/E2F2/Z<br>142/KLF2/NCOR2/CCNF/CDC25B/NACC2/LOC102287159/SLC2A4RG/RAVER1/LOC102274386/MKI67/POM121C/<br>FR3/RECQL4/UROC1/LOC138991273/ZFPM1/ATN1/ABCA3/RXRA/SETD1A/GGT5/SOX13/ERF/ZNF575/GTSE1/PC<br>6/SCAP/RNPEPL1/ZFP36/E2F8/CIC/SALL2/CHERP/PLK1/INCENP/ZNF385A/PER1/CERS1/RNF44/FAM20C/PIGT/K
GO:0044242	cellular lipid catabolic process	4	0.683366733	1.678534447	0.03958108	0.607954898	0.592891949	162	tags=100%, list=32%, signal=68%	LOC102287159/PNPLA2/LIPE/CPT1C
GO:0044773	mitotic DNA damage checkpoint signaling	7	0.566454211	1.774291326	0.0188543	0.412725217	0.402499361	160	tags=86%, list=32%, signal=59%	LOC102287159/GTSE1/PLK1/ZNF385A/PML/RBM38

GO:0044774	mitotic DNA integrity checkpoint signaling	8	0.582962541	1.948404299	0.010707594	0.335498224	0.327185777	160	tags=88%, list=32%, signal=61%	LOC102287159/GTSE1/PLK1/ZNF385A/TICRR/PML/RBM38
GO:0044819	mitotic G1/S transition checkpoint signaling	6	0.544063929	1.600439009	0.034695476	0.579334539	0.5649807	160	tags=83%, list=32%, signal=58%	LOC102287159/GTSE1/ZNF385A/PML/RBM38
GO:0044839	cell cycle G2/M phase transition	3	0.798	1.714937435	0.02752649	0.517292844	0.504476177	104	tags=100%, list=21%, signal=80%	FOXM1/CDC25B/PLK1
GO:0045165	cell fate commitment	7	0.556451613	1.742960421	0.020693705	0.442204762	0.431248509	227	tags=100%, list=45%, signal=56%	DLX2/ZFPM1/SOX12/PML/KDM6B/FOXC2/GATA6
GO:0045333	cellular respiration	3	-0.828	-1.776893073	0.01050108	0.335498224	0.327185777	90	tags=100%, list=18%, signal=83%	NDUFS4/UQCRH
GO:0045428	regulation of nitric oxide biosynthetic process	2	0.876247505	1.575509482	0.039316641	0.607954898	0.592891949	64	tags=100%, list=13%, signal=88%	KLF2/LOC138991273
GO:0045429	positive regulation of nitric oxide biosynthetic process	2	0.876247505	1.575509482	0.039316641	0.607954898	0.592891949	64	tags=100%, list=13%, signal=88%	KLF2/LOC138991273
GO:0045637	regulation of myeloid cell differentiation	7	0.531997938	1.666364744	0.029277626	0.533536435	0.520317309	115	tags=71%, list=23%, signal=56%	LOC138991273/ZFPM1/GAS2L1/ZFP36/TOB2
GO:0045685	regulation of glial cell differentiation	3	0.834	1.792303033	0.012457606	0.335498224	0.327185777	86	tags=100%, list=17%, signal=83%	DLX2/LOC138991273/SLC45A3
GO:0045786	negative regulation of cell cycle	16	0.419871187	1.895558814	0.009802015	0.335498224	0.327185777	160	tags=69%, list=32%, signal=48%	TFAP4/CCNF/LOC102287159/GTSE1/E2F8/PLK1/ZNF385A/ESPL1/TICRR/PML/RBM38
GO:0045787	positive regulation of cell cycle	11	0.553014061	2.144417667	0.002900821	0.158660506	0.154729465	152	tags=82%, list=30%, signal=58%	FOSL1/CDC25B/WIZ/HCFC1/E2F8/ESPL1/LRP5/LOC102285558/PSRC1
GO:0045892	negative regulation of DNA-templated transcription	32	0.35190405	2.135543358	0.001391752	0.092532753	0.090240122	248	tags=81%, list=49%, signal=44%	FOXK1/TFAP4/NCOR2/NACC2/DLX2/HCFC1/GLIS2/RXRA/ERF/E2F8/CIC/SALL2/PLK1/PER1/NFIC/PML/BCL3/PPP1[illegible]<br>3L/ZNF438/FOXC2/USP2/PHF12/BCL7A/GATA6/TSC22D4/E2F1
GO:0045893	positive regulation of DNA-templated transcription	41	0.342589418	2.337934191	0.000465851	0.042882207	0.041819739	177	tags=63%, list=35%, signal=45%	FOXM1/MAMSTR/FOSL1/ZMIZ2/FOXK1/TFAP4/KLF2/LOC138991273/SIX2/ZFPM1/GLIS2/RXRA/ETV4/MYBL2/E2[illegible]<br>PER1/SOX12/LRP5/MRTFA/NFIC/PML/PSRC1/BCL9L/BCL3/KDM6B/CAMTA2
GO:0045916	negative regulation of complement activation	1	0.992031873	1.343189254	0.013407276	0.335498224	0.327185777	5	tags=100%, list=1%, signal=99%	MASP1
GO:0045921	positive regulation of exocytosis	1	0.972111554	1.316217582	0.049212598	0.667515691	0.650977038	15	tags=100%, list=3%, signal=97%	STX1A
GO:0045934	negative regulation of nucleobase-containing compound metabolic process	39	0.34121558	2.278325865	0.000823733	0.062919216	0.061360303	211	tags=72%, list=42%, signal=45%	FOXK1/TFAP4/LOC106701625/NCOR2/NACC2/DLX2/LOC138991273/HCFC1/GLIS2/RXRA/ERF/ZFP36/E2F8/CIC/S[illegible]<br>L2/PLK1/PER1/CERS1/NFIC/PML/BCL3/RBM38/PPP1R13L/ZNF438/FOXC2/USP2/PHF12/BCL7A
GO:0045935	positive regulation of nucleobase-containing compound metabolic process	45	0.357916623	2.511508334	0.000120052	0.0159555	0.01556018	177	tags=64%, list=35%, signal=46%	FOXM1/MAMSTR/FOSL1/ZMIZ2/FOXK1/TFAP4/LOC106701625/KLF2/WIZ/LOC138991273/SIX2/ZFPM1/GLIS2/R[illegible]<br>A/ETV4/MYBL2/ZFP36/E2F8/PER1/SOX12/LRP5/MRTFA/NFIC/PML/PSRC1/BCL9L/BCL3/KDM6B/CAMTA2
GO:0045944	positive regulation of transcription by RNA polymerase II	30	0.373427845	2.20370414	0.001925477	0.117160365	0.114257549	231	tags=80%, list=46%, signal=46%	FOXM1/MAMSTR/ZMIZ2/KLF2/SIX2/GLIS2/RXRA/ETV4/MYBL2/E2F8/PER1/SOX12/LRP5/MRTFA/NFIC/PML/BCL[illegible]<br>BCL3/KDM6B/CAMTA2/FOXC2/GATA6/PPRC1/AUTS2

GO:0045956	positive regulation of calcium ion-dependent exocytosis	1	0.972111554	1.316217582	0.049212598	0.667515691	0.650977038	15	tags=100%, list=3%, signal=97%	STX1A
GO:0046390	ribose phosphate biosynthetic process	3	-0.814	-1.746848987	0.013644217	0.335498224	0.327185777	97	tags=100%, list=19%, signal=81%	ATP5MC3/ATP5ME
GO:0046483	heterocycle metabolic process	99	0.213350069	1.945213122	0.003977994	0.178653189	0.174226801	168	tags=48%, list=33%, signal=40%	FOXM1/ZNF865/MAMSTR/SP2/FOSL1/ZMIZ2/FOXK1/LOC106701625/E2F2/ZNF142/KLF2/NCOR2/NACC2/SLC2A<br>G/RAVER1/LOC102274386/MKI67/RECQL4/LOC138991273/ZFPM1/ATN1/RXRA/SETD1A/SOX13/ERF/ZNF575/ZF<br>6/E2F8/CIC/SALL2/CHERP/ZNF385A/PER1/KMT5C/TICRR/LIPE/ZNF574/PHF19/NFIC/KAT2A/PDE7A/PML/BCL9L/<br>F358/BCL3/RBM38/ASF1B/PPP1R13L
GO:0048469	cell maturation	3	0.78	1.676254635	0.034695523	0.579334539	0.5649807	113	tags=100%, list=22%, signal=78%	KLF2/CDC25B/FAM20C
GO:0048499	synaptic vesicle membrane organization	1	0.972111554	1.316217582	0.049212598	0.667515691	0.650977038	15	tags=100%, list=3%, signal=97%	STX1A
GO:0048513	animal organ development	36	0.330107167	2.104492397	0.00350889	0.167958881	0.16379746	101	tags=47%, list=20%, signal=41%	FOXK1/TNS2/FGFRL1/CCNF/LOC102287159/PTCH2/MKI67/DLX2/SIPA1L3/LOC138991273/SIX2/ZFPM1/MDGA1/<br>RA/JUP/E2F8/SALL2
GO:0048518	positive regulation of biological process	121	0.262647378	2.544787409	1.35757E-05	0.002565099	0.002501545	232	tags=65%, list=46%, signal=46%	MASP1/FOXM1/STX1A/CEMIP/MAMSTR/SYNPO2L/FOSL1/ZMIZ2/FOXK1/TFAP4/LOC106701625/KLF2/NCOR2/C<br>25B/NACC2/PTCH2/FGFR3/WIZ/LOC138991273/SIX2/ZFPM1/HCFC1/GLIS2/RXRA/SETD1A/ETV4/MYBL2/SLC45A<br>GTSE1/JUP/ZFP36/E2F8/HCRTR1/CHERP/PLK1/ZNF385A/PER1/FAM20C/TOB2/SOX12/ESPL1/LRP5/PNPLA2/MRT<br>/LOC102285558/PLEKHG2/NFIC/KAT2A/PML/SEMA4B/PSRC1/SF3A2/BCL9L/BCL3/RBM38/PPP1R13L/BCAR1/KD
GO:0048519	negative regulation of biological process	111	0.275681674	2.604145645	1.25065E-05	0.002565099	0.002501545	204	tags=60%, list=41%, signal=46%	MASP1/MMP28/FOSL1/FOXK1/TNS2/TFAP4/FGFRL1/LOC106701625/KLF2/NCOR2/CCNF/NACC2/LOC102287159<br>TCH2/DLX2/POM121C/FGFR3/LOC138991273/SIX2/ZFPM1/HCFC1/GLIS2/NPRL3/RXRA/ETV4/ERF/GTSE1/GRINA<br>AS2L1/VASN/ZFP36/E2F8/CIC/SALL2/CHERP/PLK1/ZNF385A/PER1/CERS1/TOB2/ESPL1/LRP5/PNPLA2/MRTFA/TI<br>R/PHF19/NFIC/CLN8/PML/SEMA4B/PSRC1/BCL9L/BCL3/RBM38/CBARP/PPP1R13L/ZNF438/NPHP4/PCDH17/FOX
GO:0048522	positive regulation of cellular process	113	0.283742123	2.699183498	4.27525E-06	0.001439187	0.001403529	232	tags=67%, list=46%, signal=47%	FOXM1/STX1A/CEMIP/MAMSTR/SYNPO2L/FOSL1/ZMIZ2/FOXK1/TFAP4/LOC106701625/KLF2/NCOR2/CDC25B/<br>CC2/PTCH2/FGFR3/WIZ/LOC138991273/SIX2/ZFPM1/HCFC1/GLIS2/RXRA/SETD1A/ETV4/MYBL2/SLC45A3/GTSE1<br>UP/ZFP36/E2F8/HCRTR1/CHERP/PLK1/ZNF385A/PER1/FAM20C/SOX12/ESPL1/LRP5/PNPLA2/MRTFA/LOC10228<br>58/PLEKHG2/NFIC/KAT2A/PML/SEMA4B/PSRC1/SF3A2/BCL9L/BCL3/RBM38/PPP1R13L/BCAR1/KDM6B/CAMTA2
GO:0048523	negative regulation of cellular process	105	0.271343258	2.536063771	2.83482E-05	0.004846186	0.004726114	203	tags=60%, list=40%, signal=45%	MMP28/FOSL1/FOXK1/TNS2/TFAP4/FGFRL1/LOC106701625/KLF2/NCOR2/CCNF/NACC2/LOC102287159/PTCH2<br>LX2/POM121C/LOC138991273/SIX2/ZFPM1/HCFC1/GLIS2/NPRL3/RXRA/ETV4/ERF/GTSE1/GRINA/GAS2L1/VASN<br>FP36/E2F8/CIC/SALL2/CHERP/PLK1/ZNF385A/PER1/CERS1/TOB2/ESPL1/LRP5/MRTFA/TICRR/PHF19/NFIC/CLN8<br>ML/SEMA4B/PSRC1/BCL9L/BCL3/RBM38/CBARP/PPP1R13L/ZNF438/NPHP4/PCDH17/FOXC2/SEMA3F/ARRB2/E
GO:0048545	response to steroid hormone	6	0.679664764	1.999327549	0.003616257	0.170820566	0.166588242	97	tags=83%, list=19%, signal=68%	FOSL1/TFAP4/ABCA2/ABCA3/ZFP36
GO:0048589	developmental growth	12	0.453269286	1.836333604	0.018442807	0.412725217	0.402499361	236	tags=92%, list=47%, signal=50%	SP2/TNS2/KLF2/FGFR3/LOC138991273/KAT2A/PPP1R13L/SEMA3F/ULK1/NLGN4X/PLXNA1
GO:0048713	regulation of oligodendrocyte differentiation	3	0.834	1.792303033	0.012457606	0.335498224	0.327185777	86	tags=100%, list=17%, signal=83%	DLX2/LOC138991273/SLC45A3
GO:0048731	system development	16	0.390134645	1.76130963	0.018812095	0.412725217	0.402499361	251	tags=88%, list=50%, signal=45%	FGFRL1/FGFR3/GLIS2/CHERP/FAM20C/CLN8/EMX2/EPHA2/LRCH4/MARK4/TCOF1/LYN/MBOAT7/DLG4
GO:0048856	anatomical structure development	81	0.317198578	2.690465288	3.96052E-06	0.001439187	0.001403529	251	tags=77%, list=50%, signal=46%	FOSL1/FOXK1/TNS2/FGFRL1/KLF2/CCNF/LOC102287159/PTCH2/MKI67/DLX2/FGFR3/RECQL4/SIPA1L3/LOC1389<br>273/SIX2/ZFPM1/MDGA1/GLIS2/NPRL3/RXRA/ERF/JUP/E2F8/CIC/SALL2/CHERP/ZNF385A/FAM20C/SOX12/LRP<br>MRTFA/KAT2A/CLN8/ZNF358/BCL3/PPP1R13L/KDM6B/FAM83H/NPHP4/SNPH/FOXC2/EMX2/SEMA3F/EPHA2/U<br>1/USP2/NLGN4X/LRCH4/MARK4/DYRK1B/XIRP1/WDR62/TCOF1/GATA6/MSX1/GIT1/PLXNA1/LYN/PLXND1/MBO
GO:0048869	cellular developmental process	62	0.221729222	1.73332125	0.016163715	0.386851574	0.377266775	219	tags=63%, list=44%, signal=41%	FOXK1/KLF2/CDC25B/PTCH2/DLX2/FGFR3/SIPA1L3/LOC138991273/SIX2/ZFPM1/MDGA1/GLIS2/RXRA/ETV4/MY<br>2/ERF/E2F8/ZNF385A/FAM20C/SOX12/PIGT/LRP5/MRTFA/KAT2A/CLN8/PML/BCL9L/BCL3/RBM38/KDM6B/SNA<br>4/SNPH/FOXC2/EMX2/EPHA2/NLGN4X/PRRC2A/MMP15/WDR62
GO:0050433	regulation of catecholamine secretion	1	0.972111554	1.316217582	0.049212598	0.667515691	0.650977038	15	tags=100%, list=3%, signal=97%	STX1A
GO:0050728	negative regulation of inflammatory response	3	-0.864	-1.854149293	0.005295823	0.21647194	0.211108539	72	tags=100%, list=14%, signal=86%	TNFAIP6/C1QTNF3

GO:0050789	regulation of biological process	234	0.290214824	3.196225164	3.55837E-09	4.25819E-06	4.15269E-06	238	tags=62%, list=47%, signal=61%	MASP1/DLGAP3/FOXM1/ARHGAP33/ZNF865/STX1A/CEMIP/MAMSTR/MMP28/SYNPO2L/KIF18B/SP2/FOSL1/ZM<br>2/FOXK1/TNS2/TFAP4/FGFRL1/LOC106701625/E2F2/ZNF142/KLF2/NCOR2/CCNF/CDC25B/NACC2/LOC10228715<br>SLC2A4RG/PTCH2/MKI67/DLX2/POM121C/FGFR3/WIZ/ABCA2/SIPA1L3/LOC138991273/SIX2/ZFPM1/ATN1/HCFC<br>LRFN4/GLIS2/NPRL3/RXRA/SETD1A/CASKIN2/ETV4/ATXN2L/MYBL2/SOX13/ERF/SLC45A3/ZNF575/GTSE1/PCSK6
GO:0050793	regulation of developmental process	50	0.224443976	1.617271679	0.040643224	0.607954898	0.592891949	286	tags=78%, list=57%, signal=37%	MAMSTR/PTCH2/DLX2/FGFR3/LOC138991273/SIX2/ZFPM1/RXRA/ETV4/SOX13/SLC45A3/GAS2L1/VASN/ZFP36/<br>F385A/FAM20C/TOB2/LRP5/PML/SEMA4B/BCL9L/RBM38/PPP1R13L/FOXC2/SEMA3F/EPHA2/GATA6/AMIGO3/P<br>NA1/LYN/PLXND1/LOC138990715/E2F1/ZNF335/SOD1/INHBA/IL18/HTR2A/SIX4
GO:0050794	regulation of cellular process	226	0.29807944	3.258419393	1.1333E-09	2.25029E-06	2.19454E-06	242	tags=64%, list=48%, signal=60%	FOXM1/ARHGAP33/ZNF865/STX1A/CEMIP/MAMSTR/MMP28/SYNPO2L/KIF18B/SP2/FOSL1/ZMIZ2/FOXK1/TNS2<br>FAP4/FGFRL1/LOC106701625/E2F2/ZNF142/KLF2/NCOR2/CCNF/CDC25B/NACC2/LOC102287159/SLC2A4RG/PTC<br>2/MKI67/DLX2/POM121C/FGFR3/WIZ/ABCA2/SIPA1L3/LOC138991273/SIX2/ZFPM1/ATN1/HCFC1/GLIS2/NPRL3/<br>XRA/SETD1A/CASKIN2/ETV4/ATXN2L/MYBL2/SOX13/ERF/SLC45A3/ZNF575/GTSE1/PCSK6/SCAP/GRINA/GAS2L1/
GO:0050821	protein stabilization	5	0.608433735	1.6505283	0.049298855	0.667515691	0.650977038	200	tags=100%, list=40%, signal=61%	WIZ/HCFC1/GTSE1/PML/USP2
GO:0050896	response to stimulus	92	0.18445657	1.645531184	0.032426321	0.570639661	0.55650125	249	tags=64%, list=50%, signal=40%	MASP1/FOXRED2/FOXM1/FOSL1/TNS2/TFAP4/KLF2/LOC102269246/LOC102287159/MKI67/RECQL4/ABCA2/LO<br>38991273/ABCA3/NPRL3/RXRA/GGT5/GTSE1/SCAP/GAS2L1/VASN/JUP/ZFP36/HCRTR1/PLK1/ZNF385A/PER1/CE<br>1/LRP5/TICRR/LOC102285558/KAT2A/CLN8/PML/BCL3/RBM38/TINAGL1/BCAR1/SLC9A1/KDM6B/CAMTA2/NPH<br>/POLD1/FOXC2/EMX2/EPHA2/ULK1/USP2/OSBPL7/MMP15/IL1RL1/SCARB1/GATA6/SIPA1/AUTS2/LYN/CXCL2/E
GO:0051155	positive regulation of striated muscle cell differentiation	2	0.876247505	1.575509482	0.039316641	0.607954898	0.592891949	64	tags=100%, list=13%, signal=88%	MAMSTR/LOC138991273
GO:0051171	regulation of nitrogen compound metabolic process	122	0.267405362	2.595842378	5.84129E-06	0.001643941	0.00160321	237	tags=66%, list=47%, signal=46%	FOXM1/ZNF865/CEMIP/MAMSTR/SP2/FOSL1/ZMIZ2/FOXK1/TFAP4/LOC106701625/ZNF142/KLF2/NCOR2/CDC2<br>/NACC2/SLC2A4RG/DLX2/FGFR3/WIZ/ABCA2/LOC138991273/SIX2/ZFPM1/ATN1/HCFC1/GLIS2/RXRA/SETD1A/E<br>4/MYBL2/ERF/ZNF575/JUP/ZFP36/E2F8/CIC/SALL2/PLK1/ZNF385A/PER1/CERS1/CCNB3/RNF44/SOX12/KMT5C/L<br>5/MRTFA/TICRR/ZNF574/PHF19/NFIC/KAT2A/PML/PSRC1/BCL9L/ZNF358/BCL3/RBM38/ASF1B/PPP1R13L/ZNF43
GO:0051172	negative regulation of nitrogen compound metabolic process	43	0.338337931	2.328939809	0.000719981	0.05743851	0.056015389	266	tags=84%, list=53%, signal=43%	FOXK1/TFAP4/LOC106701625/NCOR2/NACC2/DLX2/LOC138991273/HCFC1/GLIS2/RXRA/ERF/ZFP36/E2F8/CIC/S<br>L2/PLK1/PER1/CERS1/LRP5/MRTFA/NFIC/PML/BCL3/RBM38/PPP1R13L/ZNF438/FOXC2/USP2/PHF12/BCL7A/GA<br>6/LYN/TSC22D4/E2F1/MLLT1/UHRF1
GO:0051173	positive regulation of nitrogen compound metabolic process	57	0.304315761	2.314679017	0.000388993	0.037742808	0.036807677	159	tags=54%, list=32%, signal=42%	FOXM1/CEMIP/MAMSTR/FOSL1/ZMIZ2/FOXK1/TFAP4/LOC106701625/KLF2/CDC25B/FGFR3/WIZ/LOC13899127<br>SIX2/ZFPM1/GLIS2/RXRA/ETV4/MYBL2/ZFP36/E2F8/PLK1/PER1/SOX12/LRP5/MRTFA/NFIC/PML/PSRC1/BCL9L/B<br>3
GO:0051248	negative regulation of protein metabolic process	10	0.429164606	1.607745602	0.049115914	0.667515691	0.650977038	149	tags=70%, list=30%, signal=50%	TFAP4/LOC138991273/ZFP36/PLK1/LRP5/MRTFA/PML
GO:0051252	regulation of RNA metabolic process	102	0.28458852	2.605813101	1.45889E-05	0.0026187	0.002553818	234	tags=69%, list=47%, signal=46%	FOXM1/ZNF865/MAMSTR/SP2/FOSL1/ZMIZ2/FOXK1/TFAP4/ZNF142/KLF2/NCOR2/NACC2/SLC2A4RG/DLX2/ABC<br>/LOC138991273/SIX2/ZFPM1/ATN1/HCFC1/GLIS2/RXRA/SETD1A/ETV4/MYBL2/ERF/ZNF575/JUP/ZFP36/E2F8/C<br>SALL2/PLK1/ZNF385A/PER1/RNF44/SOX12/KMT5C/LRP5/MRTFA/ZNF574/PHF19/NFIC/KAT2A/PML/PSRC1/BCL9<br>ZNF358/BCL3/RBM38/ASF1B/PPP1R13L/ZNF438/KDM6B/CAMTA2/SNAPC4/FOXC2/EMX2/USP2/TCF7L1/PHF12/
GO:0051253	negative regulation of RNA metabolic process	37	0.322747698	2.078025046	0.00335207	0.163590594	0.159537404	211	tags=70%, list=42%, signal=44%	FOXK1/TFAP4/NCOR2/NACC2/DLX2/LOC138991273/HCFC1/GLIS2/RXRA/ERF/ZFP36/E2F8/CIC/SALL2/PLK1/PER1<br>FIC/PML/BCL3/RBM38/PPP1R13L/ZNF438/FOXC2/USP2/PHF12/BCL7A
GO:0051254	positive regulation of RNA metabolic process	42	0.351889698	2.3933603	0.000172148	0.018727607	0.018263604	177	tags=64%, list=35%, signal=45%	FOXM1/MAMSTR/FOSL1/ZMIZ2/FOXK1/TFAP4/KLF2/LOC138991273/SIX2/ZFPM1/GLIS2/RXRA/ETV4/MYBL2/ZF<br>6/E2F8/PER1/SOX12/LRP5/MRTFA/NFIC/PML/PSRC1/BCL9L/BCL3/KDM6B/CAMTA2
GO:0051276	chromosome organization	7	0.66733871	2.09028949	0.002095663	0.125390485	0.122283757	172	tags=100%, list=34%, signal=67%	KIF18B/RECQL4/PLK1/INCENP/ESPL1/NCAPH/NCAPD3
GO:0051384	response to glucocorticoid	5	0.650962015	1.765896871	0.021176656	0.44720115	0.436121104	97	tags=80%, list=19%, signal=65%	FOSL1/TFAP4/ABCA3/ZFP36
GO:0051716	cellular response to stimulus	52	0.267374798	1.974366279	0.003184597	0.161812701	0.157803561	201	tags=62%, list=40%, signal=41%	FOXRED2/FOXM1/FOSL1/TFAP4/KLF2/LOC102287159/MKI67/RECQL4/LOC138991273/NPRL3/GTSE1/SCAP/GAS<br>1/VASN/JUP/ZFP36/HCRTR1/PLK1/ZNF385A/CERS1/TICRR/KAT2A/CLN8/PML/BCL3/RBM38/SLC9A1/KDM6B/PO<br>1/EPHA2/ULK1/OSBPL7
GO:0051726	regulation of cell cycle	37	0.362738055	2.335504694	0.000548353	0.045781071	0.04464678	160	tags=62%, list=32%, signal=46%	FOSL1/TFAP4/E2F2/CCNF/CDC25B/LOC102287159/MKI67/WIZ/HCFC1/MYBL2/GTSE1/GAS2L1/E2F8/PLK1/ZNF3<br>A/CCNB3/ESPL1/LRP5/TICRR/LOC102285558/PML/PSRC1/RBM38
GO:0051952	regulation of amine transport	1	0.972111554	1.316217582	0.049212598	0.667515691	0.650977038	15	tags=100%, list=3%, signal=97%	STX1A

GO:0051954	positive regulation of amine transport	1	0.972111554	1.316217582	0.049212598	0.667515691	0.650977038	15	tags=100%, list=3%, signal=97%	STX1A
GO:0055074	calcium ion homeostasis	4	0.709418838	1.742525496	0.028242172	0.517292844	0.504476177	149	tags=100%, list=30%, signal=71%	GRINA/HCRTR1/CHERP/PML
GO:0060255	regulation of macromolecule metabolic process	134	0.231412355	2.283390248	0.000335747	0.033481422	0.032651872	237	tags=63%, list=47%, signal=46%	FOXM1/ZNF865/CEMIP/MAMSTR/SP2/FOSL1/ZMIZ2/FOXK1/TFAP4/LOC106701625/ZNF142/KLF2/NCOR2/CDC2[illegible]<br>/NACC2/SLC2A4RG/DLX2/POM121C/FGFR3/WIZ/ABCA2/LOC138991273/SIX2/ZFPM1/ATN1/HCFC1/GLIS2/RXRA<br>ETD1A/ETV4/MYBL2/ERF/ZNF575/GAS2L1/JUP/ZFP36/E2F8/CIC/SALL2/PLK1/ZNF385A/PER1/CERS1/CCNB3/RNF<br>/TOB2/SOX12/KMT5C/LRP5/MRTFA/TICRR/ZNF574/PHF19/NFIC/KAT2A/PML/PSRC1/BCL9L/ZNF358/BCL3/RBM3
GO:0060412	ventricular septum morphogenesis	2	0.870259481	1.564742903	0.0412038	0.61378275	0.598575407	67	tags=100%, list=13%, signal=87%	FGFRL1/ZFPM1
GO:0065007	biological regulation	239	0.294694327	3.239035697	1.25364E-09	2.25029E-06	2.19454E-06	238	tags=62%, list=47%, signal=63%	MASP1/DLGAP3/FOXM1/ARHGAP33/ZNF865/STX1A/CEMIP/MAMSTR/MMP28/SYNPO2L/KIF18B/SP2/FOSL1/Z[illegible]<br>2/FOXK1/TNS2/TFAP4/FGFRL1/LOC106701625/E2F2/ZNF142/KLF2/NCOR2/CCNF/CDC25B/NACC2/LOC10228715<br>SLC2A4RG/PTCH2/MKI67/DLX2/POM121C/FGFR3/WIZ/ABCA2/SIPA1L3/LOC138991273/SIX2/ZFPM1/ATN1/HCF[illegible]<br>LRFN4/GLIS2/NPRL3/RXRA/SETD1A/CASKIN2/ETV4/ATXN2L/MYBL2/SOX13/ERF/SLC45A3/ZNF575/GTSE1/PCSK6
GO:0070124	mitochondrial translational initiation	3	-0.752	-1.613796607	0.037225967	0.604711416	0.589728828	128	tags=100%, list=25%, signal=75%	MRPL33/MRPS18C
GO:0070125	mitochondrial translational elongation	3	-0.752	-1.613796607	0.037225967	0.604711416	0.589728828	128	tags=100%, list=25%, signal=75%	MRPL33/MRPS18C
GO:0071417	cellular response to organonitrogen compound	3	0.814	1.749322145	0.017884573	0.406364657	0.396296393	96	tags=100%, list=19%, signal=81%	KLF2/LOC102287159/JUP
GO:0071495	cellular response to endogenous stimulus	13	0.400636354	1.680355964	0.04039617	0.607954898	0.592891949	171	tags=69%, list=34%, signal=47%	TFAP4/KLF2/LOC102287159/GAS2L1/JUP/ZFP36/HCRTR1/KAT2A/BCAR1
GO:0071695	anatomical structure maturation	4	0.781563126	1.919731479	0.008886814	0.325547562	0.317481657	113	tags=100%, list=22%, signal=78%	KLF2/CDC25B/FGFR3/FAM20C
GO:0071826	protein-RNA complex organization	12	-0.45285294	-1.765914824	0.027956898	0.517292844	0.504476177	210	tags=83%, list=42%, signal=50%	RPL24/GEMIN2/RPS17/RPS27/LOC102275123/RPS19/RPS28/LOC102279476/LOC102285651/LOC102265456
GO:0072522	purine-containing compound biosynthetic process	3	-0.814	-1.746848987	0.013644217	0.335498224	0.327185777	97	tags=100%, list=19%, signal=81%	ATP5MC3/ATP5ME
GO:0080090	regulation of primary metabolic process	125	0.267667286	2.603664955	1.23901E-05	0.002565099	0.002501545	237	tags=66%, list=47%, signal=47%	FOXM1/ZNF865/CEMIP/MAMSTR/SP2/FOSL1/ZMIZ2/FOXK1/TFAP4/LOC106701625/ZNF142/KLF2/NCOR2/CDC2[illegible]<br>/NACC2/SLC2A4RG/DLX2/FGFR3/WIZ/ABCA2/LOC138991273/SIX2/ZFPM1/ATN1/HCFC1/GLIS2/RXRA/SETD1A/E[illegible]<br>4/MYBL2/ERF/SLC45A3/ZNF575/JUP/ZFP36/E2F8/CIC/SALL2/PLK1/ZNF385A/PER1/CERS1/CCNB3/RNF44/SOX12[illegible]<br>MT5C/LRP5/PNPLA2/MRTFA/TICRR/ZNF574/PHF19/NFIC/KAT2A/PML/PSRC1/BCL9L/ZNF358/BCL3/RBM38/ASF1
GO:0080164	regulation of nitric oxide metabolic process	2	0.876247505	1.575509482	0.039316641	0.607954898	0.592891949	64	tags=100%, list=13%, signal=88%	KLF2/LOC138991273
GO:0090068	positive regulation of cell cycle process	6	0.622023186	1.829766903	0.010497421	0.335498224	0.327185777	122	tags=83%, list=24%, signal=64%	CDC25B/WIZ/E2F8/ESPL1/LRP5
GO:0090304	nucleic acid metabolic process	92	0.245584484	2.19085135	0.0006225	0.050790333	0.04953193	168	tags=51%, list=33%, signal=42%	FOXM1/ZNF865/MAMSTR/SP2/FOSL1/ZMIZ2/FOXK1/LOC106701625/E2F2/ZNF142/KLF2/NCOR2/NACC2/SLC2A[illegible]<br>G/RAVER1/LOC102274386/MKI67/RECQL4/LOC138991273/ZFPM1/ATN1/RXRA/SETD1A/SOX13/ERF/ZNF575/ZF<br>6/E2F8/CIC/SALL2/CHERP/ZNF385A/PER1/KMT5C/TICRR/LIPE/ZNF574/PHF19/NFIC/KAT2A/PML/BCL9L/ZNF358/<br>L3/RBM38/ASF1B/PPP1R13L
GO:0098727	maintenance of cell number	6	0.531354163	1.56305148	0.049309665	0.667515691	0.650977038	157	tags=83%, list=31%, signal=58%	SIX2/SETD1A/LRP5/BCL9L/ZNF358
GO:0098813	nuclear chromosome segregation	3	0.772	1.65906228	0.040321682	0.607954898	0.592891949	117	tags=100%, list=23%, signal=77%	KIF18B/PLK1/ESPL1

GO:0099500	vesicle fusion to plasma membrane	1	0.972111554	1.316217582	0.049212598	0.667515691	0.650977038	15	tags=100%, list=3%, signal=97%	STX1A
GO:1900180	regulation of protein localization to nucleus	3	0.814	1.749322145	0.017884573	0.406364657	0.396296393	96	tags=100%, list=19%, signal=81%	GLIS2/GTSE1/JUP
GO:1900182	positive regulation of protein localization to nucleus	3	0.814	1.749322145	0.017884573	0.406364657	0.396296393	96	tags=100%, list=19%, signal=81%	GLIS2/GTSE1/JUP
GO:1901293	nucleoside phosphate biosynthetic process	3	-0.814	-1.746848987	0.013644217	0.335498224	0.327185777	97	tags=100%, list=19%, signal=81%	ATP5MC3/ATP5ME
GO:1901360	organic cyclic compound metabolic process	106	0.212109497	1.989538725	0.003077989	0.161812701	0.157803561	168	tags=48%, list=33%, signal=41%	FOXM1/ZNF865/MAMSTR/SP2/FOSL1/ZMIZ2/FOXK1/LOC106701625/E2F2/ZNF142/KLF2/NCOR2/NACC2/SLC2A4<br>G/RAVER1/LOC102274386/MKI67/RECQL4/LOC138991273/ZFPM1/ATN1/RXRA/SETD1A/SOX13/ERF/ZNF575/SC<br>/ZFP36/E2F8/CIC/SALL2/CHERP/ZNF385A/PER1/KMT5C/LRP5/TICRR/LIPE/ZNF574/PHF19/NFIC/KAT2A/CLN8/PD<br>A/PML/BCL9L/ZNF358/BCL3/RBM38/ASF1B/PPP1R13L
GO:1901362	organic cyclic compound biosynthetic process	77	0.218818742	1.84443953	0.009450942	0.335498224	0.327185777	168	tags=49%, list=33%, signal=39%	FOXM1/ZNF865/MAMSTR/SP2/FOSL1/ZMIZ2/FOXK1/E2F2/ZNF142/KLF2/NCOR2/NACC2/SLC2A4RG/LOC138991<br>3/ZFPM1/ATN1/RXRA/SETD1A/SOX13/ERF/ZNF575/E2F8/CIC/SALL2/ZNF385A/PER1/KMT5C/LIPE/ZNF574/PHF1<br>NFIC/KAT2A/PML/BCL9L/ZNF358/BCL3/ASF1B/PPP1R13L
GO:1901566	organonitrogen compound biosynthetic process	41	-0.455427238	-2.892179537	4.40977E-06	0.001439187	0.001403529	141	tags=85%, list=28%, signal=67%	INHBA/RPS23/GCH1/RPL24/RPL27A/RPL37A/RPL23/RPS17/RPS27/LOC102266576/LOC102286668/RPS29/RPL36<br>RPLP2/B4GALT6/RPL35A/NME1/LOC138987848/ATP5MC3/RPL34/LOC102275123/RPS19/MRPS18C/RPL36AL/RP<br>9/LOC102269867/LOC106701537/RPL22/RPS28/LOC102279476/LOC102285651/LOC102281282/ATP5ME/LOC1<br>265456/LOC102270678
GO:1901575	organic substance catabolic process	30	0.288610297	1.703171619	0.033018768	0.575424155	0.561167201	294	tags=87%, list=58%, signal=38%	FOXRED2/CEMIP/CCNF/LOC102287159/UROC1/GTSE1/RNPEPL1/ZFP36/RNF44/LRP5/PNPLA2/LIPE/CLN8/PDE7A<br>ML/CPT1C/FBXW5/USP2/SCARB1/HSPG2/IDUA/DCP1B/UHRF1/SOD1/RNF11/LYPLAL1
GO:1901653	cellular response to peptide	2	0.914171657	1.64369782	0.018309799	0.412725217	0.402499361	45	tags=100%, list=9%, signal=91%	KLF2/LOC102287159
GO:1901654	response to ketone	4	0.738401602	1.813715042	0.019387975	0.419294166	0.408905555	37	tags=75%, list=7%, signal=70%	FOSL1/TFAP4/KLF2
GO:1901698	response to nitrogen compound	14	0.502963731	2.166096106	0.002280163	0.132028783	0.128757582	122	tags=71%, list=24%, signal=56%	FOXRED2/FOXM1/FOSL1/KLF2/LOC102287159/LOC138991273/JUP/HCRTR1/PER1/LRP5
GO:1901699	cellular response to nitrogen compound	3	0.814	1.749322145	0.017884573	0.406364657	0.396296393	96	tags=100%, list=19%, signal=81%	KLF2/LOC102287159/JUP
GO:1901987	regulation of cell cycle phase transition	12	0.465458147	1.885714436	0.012259199	0.335498224	0.327185777	160	tags=75%, list=32%, signal=52%	LOC102287159/GTSE1/PLK1/ZNF385A/CCNB3/ESPL1/TICRR/PML/RBM38
GO:1901988	negative regulation of cell cycle phase transition	9	0.493378288	1.750736553	0.026833361	0.517292844	0.504476177	160	tags=78%, list=32%, signal=54%	LOC102287159/GTSE1/PLK1/ZNF385A/TICRR/PML/RBM38
GO:1901990	regulation of mitotic cell cycle phase transition	12	0.465458147	1.885714436	0.012259199	0.335498224	0.327185777	160	tags=75%, list=32%, signal=52%	LOC102287159/GTSE1/PLK1/ZNF385A/CCNB3/ESPL1/TICRR/PML/RBM38
GO:1901991	negative regulation of mitotic cell cycle phase transition	9	0.493378288	1.750736553	0.026833361	0.517292844	0.504476177	160	tags=78%, list=32%, signal=54%	LOC102287159/GTSE1/PLK1/ZNF385A/TICRR/PML/RBM38
GO:1902105	regulation of leukocyte differentiation	5	0.607028046	1.646715018	0.049911255	0.667515691	0.650977038	115	tags=80%, list=23%, signal=62%	LOC138991273/ZFPM1/SOX13/TOB2
GO:1902600	proton transmembrane transport	3	-0.822	-1.764017036	0.011786908	0.335498224	0.327185777	93	tags=100%, list=18%, signal=82%	ATP5MC3/UQCR10

GO:1902679	negative regulation of RNA biosynthetic process	32	0.35190405	2.135543358	0.001391752	0.092532753	0.090240122	248	tags=81%, list=49%, signal=44%	FOXK1/TFAP4/NCOR2/NACC2/DLX2/HCFC1/GLIS2/RXRA/ERF/E2F8/CIC/SALL2/PLK1/PER1/NFIC/PML/BCL3/PPP1[illegible]<br>3L/ZNF438/FOXC2/USP2/PHF12/BCL7A/GATA6/TSC22D4/E2F1
GO:1902680	positive regulation of RNA biosynthetic process	41	0.342589418	2.337934191	0.000465851	0.042882207	0.041819739	177	tags=63%, list=35%, signal=45%	FOXM1/MAMSTR/FOSL1/ZMIZ2/FOXK1/TFAP4/KLF2/LOC138991273/SIX2/ZFPM1/GLIS2/RXRA/ETV4/MYBL2/E2F[illegible]<br>PER1/SOX12/LRP5/MRTFA/NFIC/PML/PSRC1/BCL9L/BCL3/KDM6B/CAMTA2
GO:1902749	regulation of cell cycle G2/M phase transition	4	0.745490982	1.831128488	0.017556081	0.406364657	0.396296393	131	tags=100%, list=26%, signal=75%	GTSE1/PLK1/CCNB3/TICRR
GO:1902806	regulation of cell cycle G1/S phase transition	6	0.544063929	1.600439009	0.034695476	0.579334539	0.5649807	160	tags=83%, list=32%, signal=58%	LOC102287159/GTSE1/ZNF385A/PML/RBM38
GO:1902807	negative regulation of cell cycle G1/S phase transition	6	0.544063929	1.600439009	0.034695476	0.579334539	0.5649807	160	tags=83%, list=32%, signal=58%	LOC102287159/GTSE1/ZNF385A/PML/RBM38
GO:1903047	mitotic cell cycle process	21	0.4615519	2.342414875	0.000288383	0.0304498	0.029695363	172	tags=76%, list=34%, signal=52%	FOXM1/KIF18B/CDC25B/LOC102287159/POM121C/MYBL2/GTSE1/PLK1/ZNF385A/ESPL1/TICRR/NCAPH/PML/PS[illegible]<br>1/RBM38/NCAPD3
GO:1903307	positive regulation of regulated secretory pathway	1	0.972111554	1.316217582	0.049212598	0.667515691	0.650977038	15	tags=100%, list=3%, signal=97%	STX1A
GO:1903706	regulation of hemopoiesis	8	0.565528501	1.890135444	0.015287407	0.36833418	0.359208175	115	tags=75%, list=23%, signal=59%	LOC138991273/ZFPM1/SOX13/GAS2L1/ZFP36/TOB2
GO:1904407	positive regulation of nitric oxide metabolic process	2	0.876247505	1.575509482	0.039316641	0.607954898	0.592891949	64	tags=100%, list=13%, signal=88%	KLF2/LOC138991273
GO:2000045	regulation of G1/S transition of mitotic cell cycle	6	0.544063929	1.600439009	0.034695476	0.579334539	0.5649807	160	tags=83%, list=32%, signal=58%	LOC102287159/GTSE1/ZNF385A/PML/RBM38
GO:2000134	negative regulation of G1/S transition of mitotic cell cycle	6	0.544063929	1.600439009	0.034695476	0.579334539	0.5649807	160	tags=83%, list=32%, signal=58%	LOC102287159/GTSE1/ZNF385A/PML/RBM38
GO:2001141	regulation of RNA biosynthetic process	98	0.292730592	2.667114775	8.88143E-06	0.002125623	0.002072958	234	tags=69%, list=47%, signal=46%	FOXM1/ZNF865/MAMSTR/SP2/FOSL1/ZMIZ2/FOXK1/TFAP4/ZNF142/KLF2/NCOR2/NACC2/SLC2A4RG/DLX2/ABC[illegible]<br>/LOC138991273/SIX2/ZFPM1/ATN1/HCFC1/GLIS2/RXRA/SETD1A/ETV4/MYBL2/ERF/ZNF575/JUP/E2F8/CIC/SALL[illegible]<br>PLK1/ZNF385A/PER1/RNF44/SOX12/KMT5C/LRP5/MRTFA/ZNF574/PHF19/NFIC/KAT2A/PML/PSRC1/BCL9L/ZNF3[illegible]<br>/BCL3/ASF1B/PPP1R13L/ZNF438/KDM6B/CAMTA2/SNAPC4/FOXC2/EMX2/USP2/TCF7L1/PHF12/SRCAP/BCL7A/[illegible]
GO:2001251	negative regulation of chromosome organization	4	0.709418838	1.742525496	0.028242172	0.517292844	0.504476177	149	tags=100%, list=30%, signal=71%	LOC106701625/PLK1/ESPL1/PML
