# Supplementary material for: SIRT1 plays a critical role in maintaining the viability of Yak Sertoli cells by regulating mitochondrial biogenesis via activating the PGC-1α-NRF-1-TFAM pathway
Source: Anim Biosci. 2026 Apr 16;39(7):251005. doi: 10.5713/ab.251005 (PMC13353117; doi:10.5713/ab.251005)
Supplement: Supplementary file 8 [file ab-251005-Supplementary-8.pdf]

## Supplement 8. The GSEA-GO-CC enrichment of DE mRNAs in NC vs RNAi group.

| ID | Description | setSize | enrichmentScore | NES | pvalue | p.adjust | qvalue | rank | leading_edge | core_enrichment |
|---|---|---|---|---|---|---|---|---|---|---|
| GO:0000785 | chromatin | 13 | 0.45187333 | 1.82553006 | 0.014248737 | 0.179054229 | 0.166475182 | 172 | tags=77%, list=34%, signal=52% | NCOR2/NACC2/RXRA/SETD1A/PLK1/INCENP/KMT5C/KAT2A/ASF1B/NCAPD3 |
| GO:0005634 | nucleus | 136 | 0.25582506 | 2.543829614 | 4.59756E-05 | 0.001304558 | 0.001212909 | 267 | tags=71%, list=53%, signal=46% | FOXM1/ZNF865/CEMIP/MAMSTR/SYNPO2L/KIF18B/SP2/FOSL1/ZMIZ2/FOXK1/TFAP4/LOC106701625/ZNF142/KLF2/NCOR2/CCNF/NACC2/SLC2A4RG/RAVER1/NACAD/MBD6/LOC102274386/MKI67/DLX2/FGFR3/RECQL4/WIZ/LOC138991273/SIX2/ZFPM1/ATN1/HCFC1/GLIS2/RXRA/SETD1A/ETV4/SOX13/ERF/ZNF575/JUP/ZFP36/E2F8/CIC/SALL2/PLK1/PER1/CCNB3/TOB2/ESPL1/MRT |
| GO:0005654 | nucleoplasm | 52 | 0.313305767 | 2.265576678 | 0.000303862 | 0.007260697 | 0.006750613 | 203 | tags=67%, list=40%, signal=45% | ZMIZ2/LOC106701625/E2F2/NCOR2/CDC25B/SLC2A4RG/MBD6/SIX2/ZFPM1/RXRA/SETD1A/ETV4/MYBL2/SOX13/GTSE1/INCENP/ZNF385A/RNF44/SOX12/KMT5C/MRTFA/TICRR/PHF19/KAT2A/PML/BCL9L/BCL3/ASF1B/NCAPD3/ZNF438/KDM6B/SNAPC4/TMEM94/TCF7L1/PHF12 |
| GO:0005667 | transcription regulator complex | 13 | 0.520408163 | 2.102404993 | 0.003026126 | 0.041632161 | 0.038707389 | 248 | tags=100%, list=49%, signal=52% | TFAP4/E2F2/NCOR2/ZFPM1/RXRA/E2F8/SALL2/KAT2A/SNAPC4/TCF7L1/PHF12/GATA6/E2F1 |
| GO:0005694 | chromosome | 2 | 0.886227545 | 1.576398846 | 0.019289095 | 0.21359144 | 0.198586059 | 59 | tags=100%, list=12%, signal=89% | MKI67/RECQL4 |
| GO:0005743 | mitochondrial inner membrane | 11 | -0.584276359 | -2.161942467 | 0.002033891 | 0.031840911 | 0.029604001 | 174 | tags=91%, list=35%, signal=61% | DNAJC19/HIGD1A/UQCRQ/KGD4/MRPL33/UQCRH/MRPS18C/ROMO1/LOC106700981/COX6C |
| GO:0005747 | mitochondrial respiratory chain complex I | 7 | -0.836693548 | -2.551918748 | 8.36964E-06 | 0.000345438 | 0.00032117 | 89 | tags=100%, list=18%, signal=83% | NDUFB3/NDUFA2/NDUFA1/NDUFB1/NDUFC1/NDUFA5 |
| GO:0005753 | mitochondrial proton-transporting ATP synthase complex | 3 | -0.924 | -1.939148207 | 0.001276232 | 0.021459613 | 0.019952016 | 42 | tags=100%, list=8%, signal=92% | ATP5MJ/ATP5ME |
| GO:0005783 | endoplasmic reticulum | 15 | 0.410941397 | 1.764920818 | 0.016498615 | 0.197115036 | 0.183267167 | 270 | tags=93%, list=54%, signal=45% | CEMIP/FGFR3/PCSK6/GRINA/CERS1/LRP5/TMC6/CLN8/CPT1C/SLC9A1/CBLN3/DLG4/ANGEL1/NBEAL2 |
| GO:0005788 | endoplasmic reticulum lumen | 1 | 0.990039841 | 1.326039068 | 0.026189127 | 0.272659695 | 0.253504608 | 6 | tags=100%, list=1%, signal=99% | FOXRED2 |
| GO:0005840 | ribosome | 7 | -0.568548387 | -1.734074908 | 0.018665367 | 0.21359144 | 0.198586059 | 222 | tags=100%, list=44%, signal=57% | RPL24/RPL37A/RPL23/LOC102266576/LOC102279476/LOC102270678 |
| GO:0005856 | cytoskeleton | 11 | 0.4262083 | 1.617721454 | 0.042185874 | 0.375536992 | 0.349154496 | 251 | tags=91%, list=50%, signal=47% | SYNPO2L/GAS2L1/JUP/BCAR1/FAM83H/MARK4/FSCN1/MTSS2/LOC138987895/DLG4 |
| GO:0005886 | plasma membrane | 71 | 0.202431442 | 1.627857769 | 0.019194393 | 0.21359144 | 0.198586059 | 242 | tags=65%, list=48%, signal=39% | GPR146/ARHGAP33/STX1A/CEMIP/TNS2/FGFRL1/CLIC5/FGFR3/SIX2/MDGA1/LRFN4/ABCA3/GGT5/RNPEPL1/VASN/HCRTR1/LRP5/PNPLA2/RELT/ECE1/SEMA4B/SLCO4A1/CBARP/BCAR1/PGAP6/SLC9A1/PCDH17/PLPPR2/ARHGEF39/ARRB2/EPHA2/COMT/OSBPL7/NLGN4X/MMP15/GPR153/IL1RL1/MTSS2/C22H11orf24/ANKRD13B/SCARB1/PLXNA1/LYN/PLXND1/HSPG2/LOC138990 |

| | | | | | | | | | |
|---|---|---|---|---|---|---|---|---|---|
| GO:0005911 | cell-cell junction | 13 | 0.419728092 | 1.695666016 | 0.032397075 | 0.306422338 | 0.284895334 | 254 | tags=92%, list=50%, signal=47% | FGFRL1/SIPA1L3/ATXN2L/VASN/JUP/PPP1R13L/NPHP4/EPHA2/FSCN1/LYN/LOC138990715/ZYX |
| GO:0008021 | synaptic vesicle | 3 | 0.73180361 | 1.560315922 | 0.049163866 | 0.42113953 | 0.391553331 | 18 | tags=67%, list=4%, signal=65% | STX1A/SYN1 |
| GO:0009986 | cell surface | 9 | 0.461177009 | 1.607661207 | 0.035468127 | 0.322050593 | 0.299425661 | 224 | tags=89%, list=45%, signal=50% | FGFR3/LRFN3/LRFN4/PCSK6/VASN/EPHA2/NLGN4X/SCARB1 |
| GO:0015934 | large ribosomal subunit | 16 | -0.626335257 | -2.699023522 | 1.85179E-05 | 0.000646703 | 0.00060127 | 124 | tags=81%, list=25%, signal=63% | RPL36A/RPLP2/RPL35A/LOC138987848/MRPL33/RPL34/RPL36AL/RPL39/LOC102269867/RPL22/LOC102279476/LOC102285651/LOC102281282 |
| GO:0015935 | small ribosomal subunit | 14 | -0.597027793 | -2.440940481 | 0.000273108 | 0.006888378 | 0.006404451 | 162 | tags=86%, list=32%, signal=60% | RPS17/RPS27/LOC102286668/RPS29/KGD4/LOC102275123/RPS19/MRPS18C/LOC106701537/RPS28/LOC102265456/LOC102270678 |
| GO:0016469 | proton-transporting two-sector ATPase complex | 3 | -0.924 | -1.939148207 | 0.001276232 | 0.021459613 | 0.019952016 | 42 | tags=100%, list=8%, signal=92% | ATP5MJ/ATP5ME |
| GO:0017053 | transcription repressor complex | 4 | 0.669738023 | 1.615503914 | 0.031455043 | 0.30384233 | 0.282496579 | 67 | tags=75%, list=13%, signal=66% | TFAP4/NCOR2/ZFPM1 |
| GO:0019866 | organelle inner membrane | 13 | -0.516124238 | -2.050980816 | 0.003527369 | 0.047100751 | 0.043791795 | 174 | tags=85%, list=35%, signal=57% | DNAJC19/HIGD1A/UQCRQ/KGD4/MRPL33/UQCRH/MRPS18C/SIRT1/ROMO1/LOC106700981/COX6C |
| GO:0022625 | cytosolic large ribosomal subunit | 15 | -0.615468785 | -2.592561963 | 5.82829E-05 | 0.001556495 | 0.001447147 | 124 | tags=80%, list=25%, signal=62% | RPL36A/RPLP2/RPL35A/LOC138987848/RPL34/RPL36AL/RPL39/LOC102269867/RPL22/LOC102279476/LOC102285651/LOC102281282 |
| GO:0022627 | cytosolic small ribosomal subunit | 13 | -0.590232354 | -2.345472554 | 0.00039987 | 0.009077051 | 0.008439363 | 162 | tags=85%, list=32%, signal=59% | RPS17/RPS27/LOC102286668/RPS29/LOC102275123/RPS19/MRPS18C/LOC106701537/RPS28/LOC102265456/LOC102270678 |
| GO:0030054 | cell junction | 41 | 0.247726362 | 1.673520675 | 0.027612098 | 0.272659695 | 0.253504608 | 254 | tags=73%, list=50%, signal=39% | DLGAP3/STX1A/SYN1/TNS2/FGFRL1/SIPA1L3/LRFN3/ATN1/ATXN2L/VASN/JUP/CBARP/CPT1C/PPP1R13L/BCAR1/NPHP4/CBLN3/SNPH/EPHA2/NLGN4X/FSCN1/XIRP1/LOC138987895/GIT1/LYN/HSPG2/SCARF2/LOC138990715/DLG4/ZYX |
| GO:0030964 | NADH dehydrogenase complex | 7 | -0.836693548 | -2.551918748 | 8.36964E-06 | 0.000345438 | 0.00032117 | 89 | tags=100%, list=18%, signal=83% | NDUFB3/NDUFA2/NDUFA1/NDUFB1/NDUFC1/NDUFA5 |
| GO:0031966 | mitochondrial membrane | 18 | -0.460716256 | -2.081538897 | 0.002693647 | 0.038665324 | 0.03594898 | 175 | tags=78%, list=35%, signal=53% | TOMM7/DNAJC19/HIGD1A/UQCRQ/COX16/KGD4/MIGA1/MRPL33/UQCRH/MRPS18C/NDUFA2/ROMO1/LOC106700981/COX6C |
| GO:0032991 | protein-containing complex | 138 | -0.261283063 | -2.503304224 | 3.61344E-05 | 0.001093669 | 0.001016836 | 100 | tags=33%, list=20%, signal=37% | CAV2/RPL35A/LOC138987848/MRPL33/C10H15orf48/ATP5MC3/UQCR10/NDUFS4/LOC138989425/RPL34/LOC138989917/LOC102275123/RPS19/LOC138989196/NDUFB3/DIPK2A/MRPS18C/SIRT1/NDUFA2/LOC102277803/RPL36AL/HINT1/RPL39/LOC102269867/ATP5MK/GNG11/KBTBD3/LOC106701537/RPL22/NDUFA1/C1QTNF3/NDUFB1/LOC106700981/COX6C/ATP5MJ/RPS28 |

| | | | | | | | | | | |
|---|---|---|---|---|---|---|---|---|---|---|
| GO:0032993 | protein-DNA complex | 15 | 0.483541111 | 2.076723782 | 0.002268813 | 0.03433471 | 0.031922603 | 172 | tags=80%, list=34%, signal=54% | NCOR2/NACC2/RXRA/SETD1A/JUP/PLK1/INCENP/SOX12/KMT5C/KAT2A/ASF1B/NCAPD3 |
| GO:0042470 | melanosome | 1 | -0.988047809 | -1.307064565 | 0.027626313 | 0.272659695 | 0.253504608 | 8 | tags=100%, list=2%, signal=99% | |
| GO:0043226 | organelle | 242 | 0.194201819 | 2.150381789 | 0.000701269 | 0.014525277 | 0.013504837 | 296 | tags=70%, list=59%, signal=55% | DLGAP3/FOXM1/ZDHHC8/ZNF865/STX1A/SYN1/CEMIP/MAMSTR/SYNPO2L/KIF18B/SP2/FOSL1/ZMIZ2/FOXK1/TFAP4/FGFRL1/CLIC5/LOC106701625/ZNF142/KLF2/NCOR2/CCNF/NACC2/SLC2A4RG/RAVER1/NACAD/MBD6/LOC102274386/MKI67/DLX2/FGFR3/RECQL4/WIZ/ABCA2/LOC138991273/SIX2/ZFPM1/ATN1/HCFC1/MDGA1/ABCA3/GLIS2/NPRL3/RXRA/SETD1A/ETV4/ATXN2L/ |
| GO:0043227 | membrane-bounded organelle | 221 | 0.192961005 | 2.10262061 | 0.001181896 | 0.021459613 | 0.019952016 | 296 | tags=70%, list=59%, signal=51% | FOXM1/ZDHHC8/ZNF865/STX1A/SYN1/CEMIP/MAMSTR/SYNPO2L/KIF18B/SP2/FOSL1/ZMIZ2/FOXK1/TFAP4/FGFRL1/CLIC5/LOC106701625/ZNF142/KLF2/NCOR2/CCNF/NACC2/SLC2A4RG/RAVER1/NACAD/MBD6/LOC102274386/MKI67/DLX2/FGFR3/RECQL4/WIZ/ABCA2/LOC138991273/SIX2/ZFPM1/ATN1/HCFC1/MDGA1/ABCA3/GLIS2/NPRL3/RXRA/SETD1A/ETV4/SOX13/ERF/ZNF5 |
| GO:0043229 | intracellular organelle | 214 | 0.226862239 | 2.494531167 | 2.12378E-05 | 0.000688711 | 0.000640328 | 290 | tags=71%, list=58%, signal=53% | FOXM1/ZDHHC8/ZNF865/STX1A/SYN1/CEMIP/MAMSTR/SYNPO2L/KIF18B/SP2/FOSL1/ZMIZ2/FOXK1/TFAP4/FGFRL1/CLIC5/LOC106701625/ZNF142/KLF2/NCOR2/CCNF/NACC2/SLC2A4RG/RAVER1/NACAD/MBD6/LOC102274386/MKI67/DLX2/FGFR3/RECQL4/WIZ/ABCA2/LOC138991273/SIX2/ZFPM1/ATN1/HCFC1/MDGA1/ABCA3/GLIS2/NPRL3/RXRA/SETD1A/ETV4/ATXN2L/SOX13/E |
| GO:0043231 | intracellular membrane-bounded organelle | 188 | 0.274590863 | 2.979102667 | 1.5926E-07 | 1.44608E-05 | 1.34449E-05 | 290 | tags=75%, list=58%, signal=51% | FOXM1/ZDHHC8/ZNF865/STX1A/SYN1/CEMIP/MAMSTR/SYNPO2L/KIF18B/SP2/FOSL1/ZMIZ2/FOXK1/TFAP4/FGFRL1/CLIC5/LOC106701625/ZNF142/KLF2/NCOR2/CCNF/NACC2/SLC2A4RG/RAVER1/NACAD/MBD6/LOC102274386/MKI67/DLX2/FGFR3/RECQL4/WIZ/ABCA2/LOC138991273/SIX2/ZFPM1/ATN1/HCFC1/MDGA1/ABCA3/GLIS2/NPRL3/RXRA/SETD1A/ETV4/SOX13/ERF/ZNF5 |
| GO:0044391 | ribosomal subunit | 30 | -0.589962854 | -3.310630459 | 7.39061E-09 | 1.67767E-06 | 1.55981E-06 | 162 | tags=83%, list=32%, signal=60% | RPS17/RPS27/LOC102286668/RPS29/KGD4/RPL36A/RPLP2/RPL35A/LOC138987848/MRPL33/RPL34/LOC102275123/RPS19/MRPS18C/RPL36AL/RPL39/LOC102269867/LOC106701537/RPL22/RPS28/LOC102279476/LOC102285651/LOC102281282/LOC102265456/LOC102270678 |
| GO:0045259 | proton-transporting ATP synthase complex | 3 | -0.924 | -1.939148207 | 0.001276232 | 0.021459613 | 0.019952016 | 42 | tags=100%, list=8%, signal=92% | ATP5MJ/ATP5ME |
| GO:0045271 | respiratory chain complex I | 7 | -0.836693548 | -2.551918748 | 8.36964E-06 | 0.000345438 | 0.00032117 | 89 | tags=100%, list=18%, signal=83% | NDUFB3/NDUFA2/NDUFA1/NDUFB1/NDUFC1/NDUFA5 |
| GO:0045277 | respiratory chain complex IV | 3 | -0.822 | -1.725086392 | 0.014592525 | 0.179054229 | 0.166475182 | 93 | tags=100%, list=18%, signal=82% | LOC106700981/COX6C |
| GO:0048188 | Set1C/COMPASS complex | 2 | 0.850299401 | 1.512490785 | 0.034356101 | 0.31831979 | 0.295956958 | 77 | tags=100%, list=15%, signal=85% | HCFC1/SETD1A |
| GO:0048770 | pigment granule | 1 | -0.988047809 | -1.307064565 | 0.027626313 | 0.272659695 | 0.253504608 | 8 | tags=100%, list=2%, signal=99% | |
| GO:0070069 | cytochrome complex | 4 | -0.823647295 | -1.957399405 | 0.002725309 | 0.038665324 | 0.03594898 | 93 | tags=100%, list=18%, signal=82% | UQCR10/LOC106700981/COX6C |
| GO:0070382 | exocytic vesicle | 3 | 0.73180361 | 1.560315922 | 0.049163866 | 0.42113953 | 0.391553331 | 18 | tags=67%, list=4%, signal=65% | STX1A/SYN1 |

| | | | | | | | | | | |
|---|---|---|---|---|---|---|---|---|---|---|
| GO:0070469 | respirasome | 5 | -0.670682731 | -1.756957439 | 0.012359456 | 0.160319807 | 0.149056904 | 170 | tags=100%, list=34%, signal=67% | CYCS/UQCRQ/UQCRH/COX7A2 |
| GO:0098796 | membrane protein complex | 34 | -0.500475717 | -2.994563083 | 6.19523E-07 | 4.43666E-05 | 4.12497E-05 | 93 | tags=59%, list=18%, signal=51% | C10H15orf48/ATP5MC3/UQCR10/NDUFS4/LOC138989425/NDUFB3/DIPK2A/NDUFA2/LOC102277803/ATP5MK/GNG11/NDUFA1/NDUFB1/LOC106700981/COX6C/ATP5MJ/LOC102269855/NDUFC1/ATP5ME/NDUFA5 |
| GO:0098798 | mitochondrial protein-containing complex | 18 | -0.714123581 | -3.226445764 | 1.21587E-08 | 1.84002E-06 | 1.71075E-06 | 94 | tags=83%, list=19%, signal=70% | MRPL33/C10H15orf48/ATP5MC3/UQCR10/NDUFS4/NDUFB3/MRPS18C/NDUFA2/ATP5MK/NDUFA1/NDUFB1/ATP5MJ/NDUFC1/ATP5ME/NDUFA5 |
| GO:0098800 | inner mitochondrial membrane protein complex | 13 | -0.83877551 | -3.333136391 | 8.67762E-10 | 3.93964E-07 | 3.66287E-07 | 93 | tags=100%, list=18%, signal=84% | ATP5MC3/UQCR10/NDUFS4/NDUFB3/NDUFA2/ATP5MK/NDUFA1/NDUFB1/ATP5MJ/NDUFC1/ATP5ME/NDUFA5 |
| GO:0098803 | respiratory chain complex | 11 | -0.835365854 | -3.091025141 | 2.91729E-08 | 3.31112E-06 | 3.07851E-06 | 93 | tags=100%, list=18%, signal=83% | UQCR10/NDUFS4/NDUFB3/NDUFA2/NDUFA1/NDUFB1/LOC106700981/COX6C/NDUFC1/NDUFA5 |
| GO:0110165 | cellular anatomical entity | 408 | 0.273873862 | 2.548389776 | 6.91119E-06 | 0.000345438 | 0.00032117 | 341 | tags=74%, list=68%, signal=125% | GPR146/MASP1/FOXRED2/DLGAP3/FOXM1/ARHGAP33/ZDHHC8/ZNF865/TROAP/STX1A/ISYNA1/SYN1/CEMIP/MAMSTR/MMP28/SYNPO2L/KIF18B/SP2/FOSL1/ZMIZ2/FOXK1/TNS2/TFAP4/FGFRL1/CLIC5/LOC106701625/E2F2/ZNF142/KLF2/NCOR2/PCNX3/CCNF/CDC25B/TMEM201/NACC2/LOC102269246/LOC102287159/SLC2A4RG/PTCH2/C21H15orf39/RAVER1/NACAD/MBD6/LO |
| GO:1902494 | catalytic complex | 45 | -0.323314368 | -2.128937298 | 0.001872499 | 0.030361239 | 0.02822828 | 129 | tags=49%, list=26%, signal=40% | COMMD1/KGD4/PPP1R2/POLR2K/C10H15orf48/UQCR10/NDUFS4/NDUFB3/NDUFA2/HINT1/ATP5MK/GNG11/KBTBD3/NDUFA1/NDUFB1/LOC106700981/COX6C/ATP5MJ/LOC102269855/NDUFC1/ATP5ME/NDUFA5 |
| GO:1902495 | transmembrane transporter complex | 11 | -0.618683278 | -2.289255132 | 0.000735862 | 0.014525277 | 0.013504837 | 90 | tags=73%, list=18%, signal=61% | UQCR10/NDUFS4/NDUFB3/NDUFA2/NDUFA1/NDUFB1/NDUFC1/NDUFA5 |
| GO:1904949 | ATPase complex | 4 | -0.721611977 | -1.714912274 | 0.020128518 | 0.217579693 | 0.202294127 | 42 | tags=75%, list=8%, signal=69% | ATP5MK/ATP5MJ/ATP5ME |
| GO:1990204 | oxidoreductase complex | 9 | -0.761133603 | -2.578595717 | 1.05186E-05 | 0.000397952 | 0.000369995 | 128 | tags=100%, list=25%, signal=76% | UQCR10/NDUFS4/NDUFB3/NDUFA2/NDUFA1/NDUFB1/NDUFC1/NDUFA5 |
| GO:1990351 | transporter complex | 11 | -0.618683278 | -2.289255132 | 0.000735862 | 0.014525277 | 0.013504837 | 90 | tags=73%, list=18%, signal=61% | UQCR10/NDUFS4/NDUFB3/NDUFA2/NDUFA1/NDUFB1/NDUFC1/NDUFA5 |
| GO:1990904 | ribonucleoprotein complex | 35 | -0.496368247 | -3.017901963 | 6.84067E-07 | 4.43666E-05 | 4.12497E-05 | 210 | tags=86%, list=42%, signal=54% | RPL24/RPL27A/GEMIN2/LOC102276651/RPL23/RPS17/RPS27/LOC102286668/RPS29/KGD4/RPL36A/RPLP2/RPL35A/LOC138987848/MRPL33/RPL34/LOC102275123/RPS19/MRPS18C/RPL36AL/RPL39/LOC102269867/LOC106701537/RPL22/RPS28/LOC102279476/LOC102285651/LOC102281282/LOC102265456/LOC102270678 |
