# Supplementary material for: SIRT1 plays a critical role in maintaining the viability of Yak Sertoli cells by regulating mitochondrial biogenesis via activating the PGC-1α-NRF-1-TFAM pathway
Source: Anim Biosci. 2026 Apr 16;39(7):251005. doi: 10.5713/ab.251005 (PMC13353117; doi:10.5713/ab.251005)
Supplement: Supplementary file 9 [file ab-251005-Supplementary-9.pdf]

Supplement 9. The GSEA-GO-MF enrichment of DE mRNAs in NC vs RNAi group.

ID	Description	setSize	enrichmentScore	NES	pvalue	p.adjust	qvalue	rank	leading_edge	core_enrichment
GO:0000976	transcription cis-regulatory region binding	30	0.446994847	2.680044178	3.49415E-05	0.001894644	0.001711975	128	tags=63%, list=25%, signal=50%	FOSL1/FOXK1/TFAP4/E2F2/NACC2/DLX2/LOC138991273/SIX2/ZFPM1/GLIS2/RXRA/ETV4/MYBL2/SOX13/E2F8/SALL2/PER1/SOX12/MRTFA
GO:0000977	RNA polymerase II transcription regulatory region sequence-specific DNA binding	21	0.525936277	2.650280729	3.91225E-05	0.001894644	0.001711975	128	tags=71%, list=25%, signal=56%	FOSL1/FOXK1/TFAP4/NACC2/DLX2/SIX2/ZFPM1/GLIS2/RXRA/ETV4/MYBL2/SOX13/E2F8/PER1/MRTFA
GO:0000978	RNA polymerase II cis-regulatory region sequence-specific DNA binding	12	0.621644246	2.512414755	0.000141745	0.00505807	0.004570404	128	tags=83%, list=25%, signal=64%	FOSL1/TFAP4/NACC2/SIX2/RXRA/ETV4/MYBL2/E2F8/PER1/MRTFA
GO:0000981	DNA-binding transcription factor activity, RNA polymerase II-specific	25	0.48257954	2.635153931	3.7847E-05	0.001894644	0.001711975	116	tags=64%, list=23%, signal=52%	FOXM1/SP2/FOSL1/FOXK1/TFAP4/NACC2/DLX2/SIX2/ZFPM1/GLIS2/RXRA/ETV4/MYBL2/ERF/E2F8/SOX12
GO:0000987	cis-regulatory region sequence-specific DNA binding	13	0.63417081	2.630718142	3.77192E-05	0.001894644	0.001711975	128	tags=85%, list=25%, signal=65%	FOSL1/TFAP4/NACC2/LOC138991273/SIX2/RXRA/ETV4/MYBL2/E2F8/PER1/MRTFA
GO:0001067	transcription regulatory region nucleic acid binding	30	0.446994847	2.680044178	3.49415E-05	0.001894644	0.001711975	128	tags=63%, list=25%, signal=50%	FOSL1/FOXK1/TFAP4/E2F2/NACC2/DLX2/LOC138991273/SIX2/ZFPM1/GLIS2/RXRA/ETV4/MYBL2/SOX13/E2F8/SALL2/PER1/SOX12/MRTFA
GO:0001216	DNA-binding transcription activator activity	12	0.495234329	2.001521037	0.005352144	0.074056192	0.066916168	116	tags=67%, list=23%, signal=53%	FOSL1/TFAP4/DLX2/SIX2/GLIS2/ETV4/MYBL2/SOX12
GO:0001228	DNA-binding transcription activator activity, RNA polymerase II-specific	12	0.495234329	2.001521037	0.005352144	0.074056192	0.066916168	116	tags=67%, list=23%, signal=53%	FOSL1/TFAP4/DLX2/SIX2/GLIS2/ETV4/MYBL2/SOX12
GO:0001540	amyloid-beta binding	1	0.986055777	1.334576848	0.030317525	0.256941023	0.232168414	8	tags=100%, list=2%, signal=99%	DLGAP3
GO:0003676	nucleic acid binding	114	0.178608246	1.7283604	0.011401034	0.138033942	0.124725592	266	tags=68%, list=53%, signal=41%	FOXM1/ZNF865/SP2/FOSL1/FOXK1/TFAP4/LOC106701625/E2F2/ZNF142/KLF2/NCOR2/NACC2/SLC2A4RG/RAVER1/MKI67/DLX2/RECQL4/LOC138991273/SIX2/ZFPM1/GLIS2/RXRA/SETD1A/ETV4/ATXN2L/MYBL2/SOX13/ERF/ZNF575/ZFP36/E2F8/CIC/SALL2/CHERP/ZNF385A/PER1/SOX12/MRTFA/LOC102285558/LIPE/ZNF574/NFIC/PML/SF3A2/ZNF358/BCL3/RBM38/AHDC1/ZNF438/KDM6B/CAMTA2/S
GO:0003677	DNA binding	73	0.348925436	2.882108386	3.41354E-06	0.000727733	0.000657569	266	tags=82%, list=53%, signal=45%	FOXM1/ZNF865/SP2/FOSL1/FOXK1/TFAP4/LOC106701625/E2F2/ZNF142/KLF2/NCOR2/NACC2/SLC2A4RG/MKI67/DLX2/RECQL4/LOC138991273/SIX2/ZFPM1/GLIS2/RXRA/ETV4/MYBL2/SOX13/ERF/ZNF575/ZFP36/E2F8/CIC/SALL2/ZNF385A/PER1/SOX12/MRTFA/ZNF574/NFIC/PML/ZNF358/BCL3/AHDC1/ZNF438/KDM6B/CAMTA2/SNAPC4/POLD1/FOXC2/EMX2/TCF7L1/PHF12/SRCAP/ZNF341/GATA6/MS
GO:0003682	chromatin binding	20	0.444898342	2.196634275	0.00102628	0.023993729	0.021680407	265	tags=95%, list=53%, signal=47%	MBD6/DLX2/HCFC1/RXRA/SETD1A/CIC/PER1/TICRR/KAT2A/NCAPD3/KDM6B/CAMTA2/POLD1/FOXC2/PHF12/GATA6/AUTS2/ORC1/VAX2
GO:0003690	double-stranded DNA binding	31	0.428378367	2.581161832	0.000107755	0.004058774	0.003667453	128	tags=61%, list=25%, signal=49%	FOSL1/FOXK1/TFAP4/E2F2/NACC2/DLX2/LOC138991273/SIX2/ZFPM1/GLIS2/RXRA/ETV4/MYBL2/SOX13/E2F8/SALL2/PER1/SOX12/MRTFA
GO:0003697	single-stranded DNA binding	2	0.886227545	1.573259293	0.029195494	0.250563862	0.226406098	59	tags=100%, list=12%, signal=89%	LOC106701625/RECQL4
GO:0003700	DNA-binding transcription factor activity	39	0.401320003	2.639953162	5.35339E-05	0.0022685	0.002049785	202	tags=74%, list=40%, signal=48%	FOXM1/SP2/FOSL1/FOXK1/TFAP4/E2F2/KLF2/NACC2/SLC2A4RG/DLX2/SIX2/ZFPM1/GLIS2/RXRA/ETV4/MYBL2/SOX13/ERF/E2F8/SALL2/SOX12/MRTFA/NFIC/BCL3/ZNF438/CAMTA2/SNAPC4/FOXC2/TCF7L1

GO:0003712	transcription coregulator activity	23	0.384502671	2.012655182	0.004856448	0.070665764	0.063852623	230	tags=83%, list=46%, signal=47%	ZNF865/ZMIZ2/NCOR2/HCFC1/MYBL2/ERF/JUP/E2F8/TOB2/SOX12/MRTFA/KAT2A/PML/BCL9L/SNAPC4/PHF12/SRCAP/DYRK1B/PPRC1
GO:0003713	transcription coactivator activity	13	0.39144644	1.62382947	0.042461907	0.312925796	0.282755493	230	tags=85%, list=46%, signal=47%	ZMIZ2/HCFC1/JUP/SOX12/MRTFA/KAT2A/PML/BCL9L/SRCAP/DYRK1B/PPRC1
GO:0003727	single-stranded RNA binding	2	0.894211577	1.587432801	0.024776764	0.220009244	0.198797361	55	tags=100%, list=11%, signal=89%	LOC106701625/DLX2
GO:0003735	structural constituent of ribosome	31	-0.569627764	-3.252431062	1.37269E-08	9.30686E-06	8.40955E-06	210	tags=94%, list=42%, signal=58%	RPL24/RPL27A/RPL37A/RPL23/RPS17/RPS27/LOC102266576/LOC102286668/RPS29/RPL36A/RPLP2/RPL35A/LOC138987848/MRPL33/RPL34/LOC102275123/RPS19/MRPS18C/RPL36AL/RPL39/LOC102269867/LOC106701537/RPL22/RPS28/LOC102279476/LOC102285651/LOC102281282/LOC102265456/LOC102270678
GO:0003810	protein-glutamine gamma-glutamyltransferase activity	1	1	1.353449651	0.003809155	0.067593823	0.061076859	1	tags=100%, list=0%, signal=100%	TGM3
GO:0003954	NADH dehydrogenase activity	5	-0.825301205	-2.177315431	0.000355963	0.010055964	0.009086432	93	tags=100%, list=18%, signal=82%	NDUFS4/NDUFA2/NDUFB1/NDUFA5
GO:0003955	NAD(P)H dehydrogenase (quinone) activity	4	-0.823647295	-1.959587468	0.001915137	0.03934737	0.035553748	93	tags=100%, list=18%, signal=82%	NDUFS4/NDUFA2/NDUFA5
GO:0004129	cytochrome-c oxidase activity	3	-0.954	-2.008616477	0.000213484	0.00657919	0.005944867	27	tags=100%, list=5%, signal=95%	LOC106700981/COX6C
GO:0004175	endopeptidase activity	9	0.517613627	1.802064276	0.013707872	0.155271418	0.140301142	139	tags=78%, list=28%, signal=57%	MASP1/MMP28/PCSK6/ESPL1/PIGT/CAPN15/ECE1
GO:0004197	cysteine-type endopeptidase activity	3	0.752	1.616621969	0.024986301	0.220009244	0.198797361	127	tags=100%, list=25%, signal=75%	ESPL1/PIGT/CAPN15
GO:0004672	protein kinase activity	14	0.533742331	2.290198709	0.000688976	0.017300946	0.015632899	242	tags=100%, list=48%, signal=53%	PRAG1/FGFRL1/FGFR3/LOC138991273/PLK1/FAM20C/TESK1/PML/EPHA2/ULK1/MARK4/DYRK1B/LYN/LOC138990715
GO:0004674	protein serine/threonine kinase activity	8	0.527272727	1.744625086	0.019005087	0.19523408	0.17641086	242	tags=100%, list=48%, signal=53%	LOC138991273/PLK1/FAM20C/TESK1/ULK1/MARK4/DYRK1B/LOC138990715
GO:0004713	protein tyrosine kinase activity	7	0.536290323	1.667386808	0.021048053	0.207870715	0.187829151	237	tags=100%, list=47%, signal=54%	PRAG1/FGFRL1/FGFR3/TESK1/EPHA2/DYRK1B/LYN
GO:0004721	phosphoprotein phosphatase activity	3	0.916	1.969183143	0.000894315	0.021655198	0.019567342	45	tags=100%, list=9%, signal=92%	TNS2/CDC25B/LOC102287159
GO:0004725	protein tyrosine phosphatase activity	2	0.914171657	1.622866568	0.017517448	0.185575467	0.167683468	45	tags=100%, list=9%, signal=91%	CDC25B/LOC102287159

GO:0004888	transmembrane signaling receptor activity	13	0.405541368	1.682299183	0.031768026	0.265910143	0.240272789	238	tags=85%, list=47%, signal=46%	GPR146/FGFRL1/PTCH2/FGFR3/HCRTR1/LRP5/EPHA2/GPR153/IL1RL1/PLXNA1/PLXND1
GO:0005007	fibroblast growth factor receptor activity	2	0.888223553	1.57680267	0.028248623	0.245545721	0.221871774	58	tags=100%, list=12%, signal=89%	FGFRL1/FGFR3
GO:0005198	structural molecule activity	39	-0.492223551	-3.093815435	1.78972E-07	6.06714E-05	5.48219E-05	210	tags=85%, list=42%, signal=53%	RPL24/LOC106701310/RPL27A/RPL37A/SNTB1/RPL23/RPS17/RPS27/LOC102266576/LOC102286668/RPS29/KRT10/RPL36A/RPLP2/RPL35A/LOC138987848/MRPL33/RPL34/LOC102275123/RPS19/MRPS18C/RPL36AL/RPL39/LOC102269867/LOC106701537/RPL22/RPS28/MGP/LOC102279476/LOC102285651/LOC102281282/LOC102265456/LOC102270678
GO:0005215	transporter activity	32	-0.341875442	-1.963823449	0.004689309	0.070652253	0.063840415	202	tags=69%, list=40%, signal=44%	SLC35A3/SLC2A12/SLC38A4/LOC102276651/SLC30A1/UQCRQ/SLC2A13/SLC7A11/SLC6A2/C10H15orf48/ATP5MC3/NDUFS4/UQCRH/SLC16A7/NDUFA2/SLC4A5/COX7A2/LOC106700981/COX6C/ATP5ME/SEC61G/NDUFA5
GO:0005484	SNAP receptor activity	1	0.972111554	1.315704044	0.043787257	0.315827237	0.285377196	15	tags=100%, list=3%, signal=97%	STX1A
GO:0005488	binding	262	0.216516469	2.408612694	4.82567E-05	0.002181202	0.001970904	281	tags=66%, list=56%, signal=61%	TGM3/PRAG1/MASP1/FOXRED2/DLGAP3/FOXM1/ARHGAP33/ZDHHC8/ZNF865/STX1A/SYN1/CEMIP/MAMSTR/MMP28/SYNPO2L/KIF18B/SP2/FOSL1/ZMIZ2/FOXK1/TNS2/TFAP4/FGFRL1/LOC106701625/E2F2/ZNF142/KLF2/NCOR2/CDC25B/TMEM201/NACC2/SLC2A4RG/PTCH2/RAVER1/MBD6/MKI67/DLX2/POM121C/FGFR3/RECQL4/WIZ/ABCA2/LOC138991273/SIX2/ZFPM1/HCFC1/ABCA3/GLIS2/RXR
GO:0005515	protein binding	102	0.266017991	2.505340989	0.000106981	0.004058774	0.003667453	252	tags=71%, list=50%, signal=44%	MASP1/FOXRED2/STX1A/CEMIP/MAMSTR/SYNPO2L/KIF18B/TNS2/TFAP4/E2F2/NCOR2/CDC25B/TMEM201/NACC2/PTCH2/MKI67/FGFR3/LOC138991273/SIX2/ZFPM1/RXRA/SETD1A/ATXN2L/PCSK6/GAS2L1/VASN/JUP/ZFP36/CHERP/PLK1/ZNF385A/PER1/TNXB/LRP5/MRTFA/PHF19/ECE1/CEP72/KAT2A/PML/SEMA4B/BCL9L/BCL3/CBARP/PPP1R13L/BCAR1/NCAPD3/SLC9A1/CAMTA2/FAM83H/RTKN/F
GO:0005524	ATP binding	25	0.363791182	1.98650312	0.003987836	0.067593823	0.061076859	253	tags=84%, list=50%, signal=44%	PRAG1/SYN1/KIF18B/MKI67/FGFR3/RECQL4/ABCA2/LOC138991273/ABCA3/PLK1/FAM20C/TESK1/TTLL12/EPHA2/ULK1/MARK4/SRCAP/DYRK1B/LYN/LOC138990715/ORC1
GO:0008134	transcription factor binding	17	0.457587173	2.122106784	0.00428542	0.069433485	0.062739153	248	tags=94%, list=49%, signal=49%	MAMSTR/E2F2/NCOR2/NACC2/LOC138991273/SIX2/ZFPM1/RXRA/GAS2L1/PER1/KAT2A/BCL3/CAMTA2/GATA6/PPRC1/E2F1
GO:0008137	NADH dehydrogenase (ubiquinone) activity	4	-0.823647295	-1.959587468	0.001915137	0.03934737	0.035553748	93	tags=100%, list=18%, signal=82%	NDUFS4/NDUFA2/NDUFA5
GO:0008233	peptidase activity	14	0.447091032	1.918392535	0.00636542	0.086315091	0.077993143	212	tags=86%, list=42%, signal=51%	MASP1/MMP28/GGT5/PCSK6/RNPEPL1/ESPL1/PIGT/CAPN15/ECE1/TINAGL1/USP2/MMP15
GO:0008320	protein transmembrane transporter activity	1	-0.990039841	-1.33187807	0.02362985	0.216500514	0.19562692	7	tags=100%, list=1%, signal=99%	
GO:0008324	monoatomic cation transmembrane transporter activity	15	-0.43653741	-1.860545263	0.009760986	0.120326334	0.108725239	202	tags=80%, list=40%, signal=49%	SLC35A3/SLC2A12/SLC30A1/UQCRQ/SLC2A13/SLC6A2/ATP5MC3/UQCRH/COX7A2/LOC106700981/COX6C/ATP5ME
GO:0009055	electron transfer activity	10	-0.709939148	-2.497832868	2.75785E-05	0.001894644	0.001711975	154	tags=100%, list=31%, signal=71%	UQCRQ/C10H15orf48/NDUFS4/UQCRH/NDUFA2/COX7A2/LOC106700981/COX6C/NDUFA5
GO:0015078	proton transmembrane transporter activity	12	-0.462564954	-1.754589609	0.014875866	0.160092658	0.144657549	202	tags=83%, list=40%, signal=51%	SLC35A3/SLC2A12/UQCRQ/SLC2A13/ATP5MC3/UQCRH/COX7A2/LOC106700981/COX6C/ATP5ME
GO:0015318	inorganic molecular entity transmembrane transporter activity	16	-0.385452144	-1.696224542	0.034957783	0.28904118	0.261173679	149	tags=62%, list=30%, signal=45%	UQCRQ/SLC2A13/SLC6A2/ATP5MC3/UQCRH/SLC4A5/COX7A2/LOC106700981/COX6C/ATP5ME

GO:0015399	primary active transmembrane transporter activity	12	-0.630056308	-2.389913548	0.000178575	0.006053686	0.005470028	93	tags=75%, list=18%, signal=63%	C10H15orf48/NDUFS4/UQCRH/NDUFA2/COX7A2/LOC106700981/COX6C/SEC61G/NDUFA5
GO:0015450	protein-transporting ATPase activity	1	-0.990039841	-1.33187807	0.02362985	0.216500514	0.19562692	7	tags=100%, list=1%, signal=99%	
GO:0015453	oxidoreduction-driven active transmembrane transporter activity	9	-0.750174493	-2.532868179	1.23806E-05	0.001678807	0.001516947	93	tags=89%, list=18%, signal=74%	C10H15orf48/NDUFS4/UQCRH/NDUFA2/COX7A2/LOC106700981/COX6C/NDUFA5
GO:0016301	kinase activity	18	0.350469491	1.668371022	0.038954989	0.295565329	0.267068811	251	tags=83%, list=50%, signal=43%	PRAG1/FGFRL1/FGFR3/LOC138991273/PLK1/FAM20C/TESK1/PML/EPHA2/ULK1/MARK4/DYRK1B/LYN/LOC138990715/DLG4
GO:0016417	S-acyltransferase activity	1	0.978087649	1.323792388	0.037050876	0.295535221	0.267041606	12	tags=100%, list=2%, signal=98%	ZDHHC8
GO:0016491	oxidoreductase activity	17	-0.468474516	-2.118721381	0.002377072	0.046047272	0.041607689	149	tags=71%, list=30%, signal=51%	UQCRQ/ALDH1A1/CYP7B1/C10H15orf48/NDUFS4/UQCRH/NDUFA2/COX7A2/NDUFB1/LOC106700981/COX6C/NDUFA5
GO:0016651	oxidoreductase activity, acting on NAD(P)H	5	-0.825301205	-2.177315431	0.000355963	0.010055964	0.009086432	93	tags=100%, list=18%, signal=82%	NDUFS4/NDUFA2/NDUFB1/NDUFA5
GO:0016655	oxidoreductase activity, acting on NAD(P)H, quinone or similar compound as acceptor	4	-0.823647295	-1.959587468	0.001915137	0.03934737	0.035553748	93	tags=100%, list=18%, signal=82%	NDUFS4/NDUFA2/NDUFA5
GO:0016675	oxidoreductase activity, acting on a heme group of donors	3	-0.954	-2.008616477	0.000213484	0.00657919	0.005944867	27	tags=100%, list=5%, signal=95%	LOC106700981/COX6C
GO:0016746	acyltransferase activity	12	0.454175153	1.835577764	0.013740833	0.155271418	0.140301142	280	tags=100%, list=56%, signal=45%	TGM3/ZDHHC8/CERS1/RNF44/KAT2A/CPT1C/NAA80/SRCAP/MBOAT7/UHRF1/QPCTL/RNF11
GO:0016773	phosphotransferase activity, alcohol group as acceptor	15	0.4723313	2.100574935	0.002674206	0.050364204	0.04550841	242	tags=93%, list=48%, signal=50%	PRAG1/FGFRL1/FGFR3/LOC138991273/PLK1/FAM20C/TESK1/PML/EPHA2/ULK1/MARK4/DYRK1B/LYN/LOC138990715
GO:0017022	myosin binding	1	0.972111554	1.315704044	0.043787257	0.315827237	0.285377196	15	tags=100%, list=3%, signal=97%	STX1A
GO:0019706	protein-cysteine S-palmitoyltransferase activity	1	0.978087649	1.323792388	0.037050876	0.295535221	0.267041606	12	tags=100%, list=2%, signal=98%	ZDHHC8
GO:0019707	protein-cysteine S-acyltransferase activity	1	0.978087649	1.323792388	0.037050876	0.295535221	0.267041606	12	tags=100%, list=2%, signal=98%	ZDHHC8
GO:0019838	growth factor binding	3	0.816	1.754206818	0.009034313	0.113430814	0.102494541	95	tags=100%, list=19%, signal=82%	FGFR3/PCSK6/VASN
GO:0019843	rRNA binding	8	-0.546416827	-1.729020753	0.013062795	0.152699572	0.137977256	179	tags=88%, list=36%, signal=57%	RPL23/LOC102286668/RPLP2/LOC102269867/LOC106701537/LOC102279476/LOC102285651

GO:0019899	enzyme binding	27	0.334488837	1.906493338	0.008045835	0.102925961	0.093002498	252	tags=81%, list=50%, signal=43%	TNS2/TFAP4/NCOR2/CDC25B/NACC2/RXRA/JUP/ZFP36/PER1/KAT2A/PML/BCAR1/CAMTA2/FAM83H/RTKN/FBXW5/ULK1/LOC138987895/GATA6/LYN/DLG4/PPP6R1
GO:0022804	active transmembrane transporter activity	23	-0.392654194	-1.961984311	0.004898659	0.070665764	0.063852623	93	tags=61%, list=18%, signal=52%	UQCRQ/SLC2A13/SLC6A2/C10H15orf48/NDUFS4/UQCRH/SLC16A7/NDUFA2/SLC4A5/COX7A2/LOC106700981/COX6C/SEC61G/NDUFA5
GO:0022857	transmembrane transporter activity	31	-0.361024952	-2.061361542	0.001989816	0.039679264	0.035853643	202	tags=71%, list=40%, signal=45%	SLC35A3/SLC2A12/SLC38A4/LOC102276651/SLC30A1/UQCRQ/SLC2A13/SLC7A11/SLC6A2/C10H15orf48/ATP5MC3/NDUFS4/UQCRH/SLC16A7/NDUFA2/SLC4A5/COX7A2/LOC106700981/COX6C/ATP5ME/SEC61G/NDUFA5
GO:0022890	inorganic cation transmembrane transporter activity	14	-0.42479284	-1.74951928	0.012905097	0.152699572	0.137977256	202	tags=79%, list=40%, signal=48%	SLC35A3/SLC2A12/UQCRQ/SLC2A13/SLC6A2/ATP5MC3/UQCRH/COX7A2/LOC106700981/COX6C/ATP5ME
GO:0030554	adenyl nucleotide binding	27	0.300287563	1.711555588	0.019762649	0.199986213	0.180704823	253	tags=78%, list=50%, signal=41%	PRAG1/SYN1/KIF18B/MKI67/FGFR3/RECQL4/ABCA2/LOC138991273/ABCA3/PLK1/FAM20C/TESK1/TTLL12/EPHA2/ULK1/MARK4/SRCAP/DYRK1B/LYN/LOC138990715/ORC1
GO:0030674	protein-macromolecule adaptor activity	26	0.410026238	2.289993741	0.000516121	0.013458849	0.012161233	230	tags=85%, list=46%, signal=48%	ZNF865/STX1A/ZMIZ2/NCOR2/HCFC1/MYBL2/ERF/GAS2L1/JUP/E2F8/TOB2/SOX12/MRTFA/KAT2A/PML/BCL9L/BCL3/SNAPC4/PHF12/SRCAP/DYRK1B/PPRC1
GO:0030695	GTPase regulator activity	12	0.404205182	1.633620949	0.039648427	0.295565329	0.267068811	267	tags=92%, list=53%, signal=44%	ARHGAP33/FGFR3/SIPA1L3/NPRL3/PLEKHG2/RTKN/ARHGEF39/SIPA1/GIT1/DLG4/DENND4B
GO:0032553	ribonucleotide binding	31	0.268658215	1.618779995	0.039670273	0.295565329	0.267068811	255	tags=74%, list=51%, signal=39%	PRAG1/SYN1/KIF18B/MKI67/FGFR3/RECQL4/ABCA2/LOC138991273/ABCA3/PLK1/FAM20C/TESK1/RTKN/TTLL12/EPHA2/ULK1/MARK4/SRCAP/DYRK1B/LYN/LOC138990715/ORC1/TUBG1
GO:0032555	purine ribonucleotide binding	31	0.268658215	1.618779995	0.039670273	0.295565329	0.267068811	255	tags=74%, list=51%, signal=39%	PRAG1/SYN1/KIF18B/MKI67/FGFR3/RECQL4/ABCA2/LOC138991273/ABCA3/PLK1/FAM20C/TESK1/RTKN/TTLL12/EPHA2/ULK1/MARK4/SRCAP/DYRK1B/LYN/LOC138990715/ORC1/TUBG1
GO:0032559	adenyl ribonucleotide binding	25	0.363791182	1.98650312	0.003987836	0.067593823	0.061076859	253	tags=84%, list=50%, signal=44%	PRAG1/SYN1/KIF18B/MKI67/FGFR3/RECQL4/ABCA2/LOC138991273/ABCA3/PLK1/FAM20C/TESK1/TTLL12/EPHA2/ULK1/MARK4/SRCAP/DYRK1B/LYN/LOC138990715/ORC1
GO:0035639	purine ribonucleoside triphosphate binding	31	0.268658215	1.618779995	0.039670273	0.295565329	0.267068811	255	tags=74%, list=51%, signal=39%	PRAG1/SYN1/KIF18B/MKI67/FGFR3/RECQL4/ABCA2/LOC138991273/ABCA3/PLK1/FAM20C/TESK1/RTKN/TTLL12/EPHA2/ULK1/MARK4/SRCAP/DYRK1B/LYN/LOC138990715/ORC1/TUBG1
GO:0036094	small molecule binding	128	0.173058386	1.729371704	0.017951104	0.187243826	0.169190975	289	tags=70%, list=57%, signal=40%	TGM3/PRAG1/MASP1/FOXRED2/ZDHHC8/ZNF865/SYN1/MMP28/KIF18B/SP2/ZMIZ2/TNS2/LOC106701625/ZNF142/KLF2/SLC2A4RG/RAVER1/MKI67/FGFR3/RECQL4/WIZ/ABCA2/LOC138991273/ZFPM1/ABCA3/GLIS2/RXRA/SETD1A/ZNF575/SCAP/RNPEPL1/ZFP36/SALL2/PLK1/ZNF385A/RNF44/FAM20C/CAPN15/ZNF574/PHF19/ECE1/TESK1/PDE7A/PML/SF3A2/ZNF358/RBM38/ZNF438/KDM6B/RTKN/P
GO:0038023	signaling receptor activity	17	0.439151117	2.036607713	0.006644312	0.086631605	0.07827914	238	tags=88%, list=47%, signal=48%	GPR146/FGFRL1/PTCH2/FGFR3/RXRA/HCRTR1/LRRN2/LRP5/EPHA2/NLGN4X/GPR153/IL1RL1/SCARB1/PLXNA1/PLXND1
GO:0042277	peptide binding	7	0.532381322	1.655233285	0.022259431	0.212561894	0.192068036	102	tags=71%, list=20%, signal=58%	DLGAP3/CEMIP/POM121C/RNPEPL1/HCRTR1
GO:0042826	histone deacetylase binding	5	0.654618474	1.738790804	0.014464982	0.160092658	0.144657549	177	tags=100%, list=35%, signal=65%	TFAP4/NCOR2/NACC2/KAT2A/CAMTA2
GO:0043167	ion binding	116	0.175445187	1.718172711	0.014722625	0.160092658	0.144657549	289	tags=71%, list=57%, signal=39%	TGM3/PRAG1/MASP1/FOXRED2/ZDHHC8/ZNF865/SYN1/MMP28/KIF18B/SP2/ZMIZ2/TNS2/ZNF142/KLF2/SLC2A4RG/MKI67/FGFR3/RECQL4/WIZ/ABCA2/LOC138991273/ZFPM1/ABCA3/GLIS2/RXRA/ZNF575/RNPEPL1/ZFP36/SALL2/PLK1/ZNF385A/RNF44/FAM20C/CAPN15/ZNF574/PHF19/ECE1/TESK1/PDE7A/PML/SF3A2/ZNF358/ZNF438/KDM6B/RTKN/PCDH17/GBGT1/POLD1/ARRB2/TTLL12/EPHA2/C

GO:0043168	anion binding	36	0.275595527	1.777238438	0.021154984	0.207870715	0.187829151	255	tags=75%, list=51%, signal=40%	PRAG1/FOXRED2/SYN1/KIF18B/MKI67/FGFR3/RECQL4/ABCA2/LOC138991273/ABCA3/PLK1/FAM20C/TESK1/RTKN/ARRB2/TTLL12/EPHA2/ULK1/NLGN4X/MARK4/SRCAP/DYRK1B/MTSS2/LYN/LOC138990715/ORC1/TUBG1
GO:0043565	sequence-specific DNA binding	40	0.40362464	2.694939478	2.15054E-05	0.001894644	0.001711975	203	tags=75%, list=40%, signal=49%	FOXM1/FOSL1/FOXK1/TFAP4/LOC106701625/E2F2/KLF2/NACC2/DLX2/LOC138991273/SIX2/ZFPM1/GLIS2/RXRA/ETV4/MYBL2/SOX13/ERF/E2F8/SALL2/PER1/SOX12/MRTFA/KDM6B/CAMTA2/SNAPC4/FOXC2/EMX2/TCF7L1/PHF12
GO:0044877	protein-containing complex binding	35	0.326567451	2.07606878	0.003556372	0.065168119	0.058885026	265	tags=83%, list=53%, signal=42%	TMEM201/MBD6/DLX2/LOC138991273/SIX2/HCFC1/RXRA/SETD1A/CIC/PER1/TNXB/TICRR/KAT2A/NCAPD3/KDM6B/CAMTA2/POLD1/FOXC2/ULK1/PHF12/FSCN1/SCARB1/LOC138987895/GATA6/AUTS2/GIT1/DLG4/ORC1/VAX2
GO:0045182	translation regulator activity	3	-0.725107358	-1.526690343	0.049904031	0.352447217	0.318466512	74	tags=67%, list=15%, signal=57%	LOC138985981/LOC102270678
GO:0046983	protein dimerization activity	10	0.457919544	1.707592827	0.021882455	0.211947208	0.191512614	149	tags=70%, list=30%, signal=50%	MASP1/TFAP4/NACC2/RXRA/JUP/ECE1/PML
GO:0050136	NADH dehydrogenase (quinone) activity	4	-0.823647295	-1.959587468	0.001915137	0.03934737	0.035553748	93	tags=100%, list=18%, signal=82%	NDUFS4/NDUFA2/NDUFA5
GO:0050660	flavin adenine dinucleotide binding	1	0.990039841	1.339969077	0.024832999	0.220009244	0.198797361	6	tags=100%, list=1%, signal=99%	FOXRED2
GO:0060089	molecular transducer activity	17	0.439151117	2.036607713	0.006644312	0.086631605	0.07827914	238	tags=88%, list=47%, signal=48%	GPR146/FGFRL1/PTCH2/FGFR3/RXRA/HCRTR1/LRRN2/LRP5/EPHA2/NLGN4X/GPR153/IL1RL1/SCARB1/PLXNA1/PLXND1
GO:0060090	molecular adaptor activity	26	0.410026238	2.289993741	0.000516121	0.013458849	0.012161233	230	tags=85%, list=46%, signal=48%	ZNF865/STX1A/ZMIZ2/NCOR2/HCFC1/MYBL2/ERF/GAS2L1/JUP/E2F8/TOB2/SOX12/MRTFA/KAT2A/PML/BCL9L/BCL3/SNAPC4/PHF12/SRCAP/DYRK1B/PPRC1
GO:0060589	nucleoside-triphosphatase regulator activity	12	0.404205182	1.633620949	0.039648427	0.295565329	0.267068811	267	tags=92%, list=53%, signal=44%	ARHGAP33/FGFR3/SIPA1L3/NPRL3/PLEKHG2/RTKN/ARHGEF39/SIPA1/GIT1/DLG4/DENND4B
GO:0061629	RNA polymerase II-specific DNA-binding transcription factor binding	8	0.594194561	1.966054156	0.004403599	0.069433485	0.062739153	94	tags=75%, list=19%, signal=62%	MAMSTR/NCOR2/NACC2/ZFPM1/RXRA/GAS2L1
GO:0097159	organic cyclic compound binding	147	0.182802309	1.900974197	0.004548912	0.070094601	0.063336528	266	tags=66%, list=53%, signal=44%	PRAG1/FOXRED2/FOXM1/ZNF865/SYN1/KIF18B/SP2/FOSL1/FOXK1/TFAP4/LOC106701625/E2F2/ZNF142/KLF2/NCOR2/NACC2/SLC2A4RG/RAVER1/MKI67/DLX2/FGFR3/RECQL4/ABCA2/LOC138991273/SIX2/ZFPM1/ABCA3/GLIS2/RXRA/SETD1A/ETV4/ATXN2L/MYBL2/SOX13/ERF/ZNF575/SCAP/ZFP36/E2F8/CIC/SALL2/CHERP/PLK1/ZNF385A/PER1/FAM20C/SOX12/MRTFA/LOC102285558/LIPE/ZNF574/TES
GO:0140096	catalytic activity, acting on a protein	49	0.364709765	2.63011975	3.25812E-05	0.001894644	0.001711975	214	tags=73%, list=43%, signal=47%	TGM3/PRAG1/MASP1/ZDHHC8/MMP28/TNS2/FGFRL1/CDC25B/NACC2/LOC102287159/FGFR3/LOC138991273/SETD1A/GGT5/PCSK6/RNPEPL1/PLK1/RNF44/FAM20C/ESPL1/PIGT/KMT5C/CAPN15/ECE1/TESK1/KAT2A/PML/TINAGL1/KDM6B/EPHA2/ULK1/USP2/MARK4/SRCAP/MMP15/DYRK1B
GO:0140110	transcription regulator activity	56	0.385104363	2.943354769	4.29341E-06	0.000727733	0.000657569	230	tags=79%, list=46%, signal=48%	FOXM1/ZNF865/SP2/FOSL1/ZMIZ2/FOXK1/TFAP4/E2F2/KLF2/NCOR2/NACC2/SLC2A4RG/DLX2/SIX2/ZFPM1/HCFC1/GLIS2/RXRA/ETV4/MYBL2/SOX13/ERF/JUP/E2F8/SALL2/TOB2/SOX12/MRTFA/NFIC/KAT2A/PML/BCL9L/BCL3/ZNF438/CAMTA2/SNAPC4/FOXC2/TCF7L1/PHF12/SRCAP/DYRK1B/ZNF341/GATA6/PPRC1
GO:0140297	DNA-binding transcription factor binding	8	0.594194561	1.966054156	0.004403599	0.069433485	0.062739153	94	tags=75%, list=19%, signal=62%	MAMSTR/NCOR2/NACC2/ZFPM1/RXRA/GAS2L1
GO:0140318	protein transporter activity	1	-0.990039841	-1.33187807	0.02362985	0.216500514	0.19562692	7	tags=100%, list=1%, signal=99%	

GO:1901618	organic hydroxy compound transmembrane transporter activity	3	-0.732	-1.54120258	0.047984645	0.342458834	0.309441145	138	tags=100%, list=27%, signal=73%	SLC6A2/SLC16A7
GO:1990837	sequence-specific double-stranded DNA binding	30	0.446994847	2.680044178	3.49415E-05	0.001894644	0.001711975	128	tags=63%, list=25%, signal=50%	FOSL1/FOXK1/TFAP4/E2F2/NACC2/DLX2/LOC138991273/SIX2/ZFPM1/GLIS2/RXRA/ETV4/MYBL2/SOX13/E2F8/SALL2/PER1/SOX12/MRTFA
