# Supplementary material for: SIRT1 plays a critical role in maintaining the viability of Yak Sertoli cells by regulating mitochondrial biogenesis via activating the PGC-1α-NRF-1-TFAM pathway
Source: Anim Biosci. 2026 Apr 16;39(7):251005. doi: 10.5713/ab.251005 (PMC13353117; doi:10.5713/ab.251005)
Supplement: Supplementary file 10 [file ab-251005-Supplementary-10.pdf]

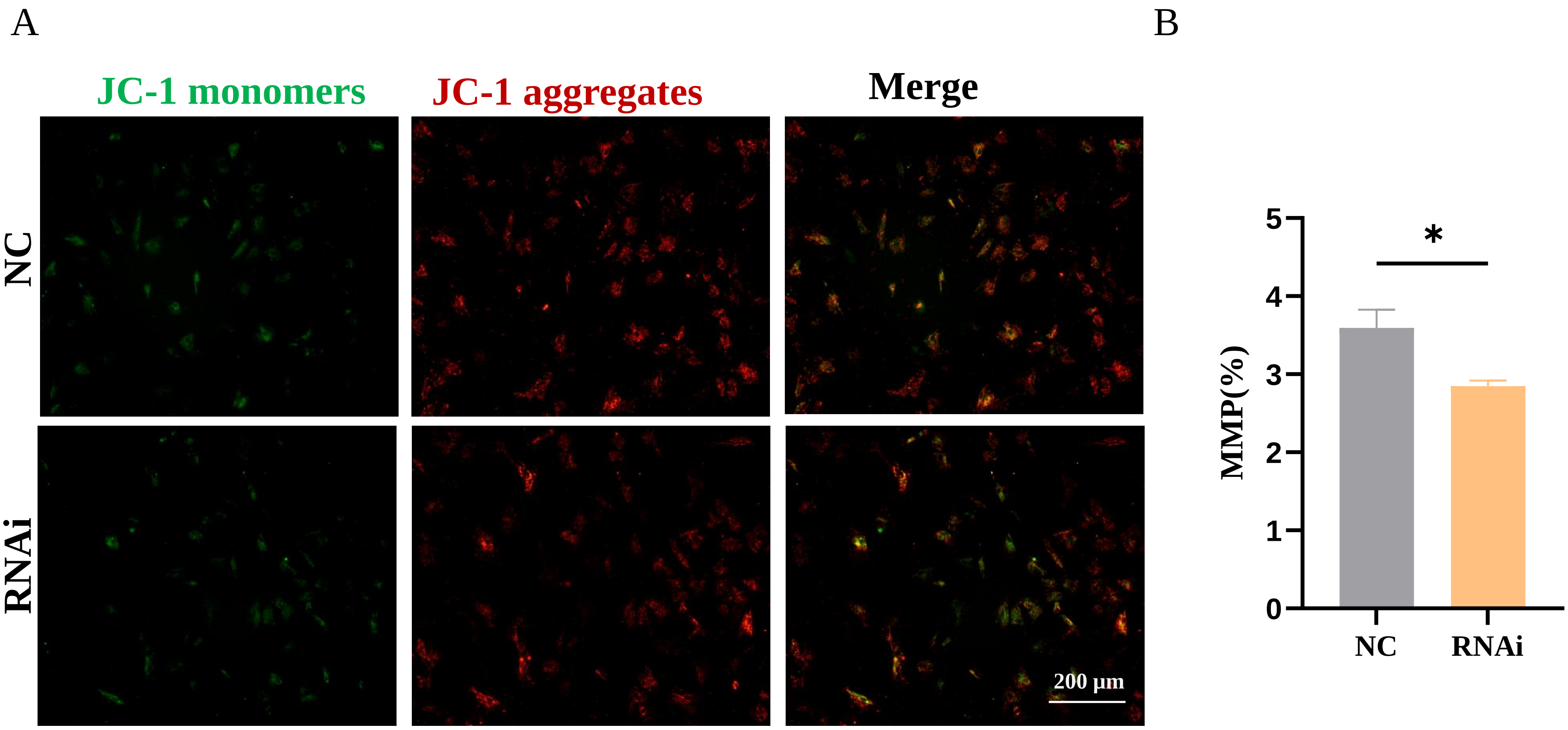

Supplement 10. Representative fluorescence images of JC-1 staining of NC group and RNAi group. (A)MMP was detected using the JC-1 fluorescent probe. JC-1-monomers and JC-1-aggregates produce green and red fluorescence, respectively. (B) MMP in (A) was quantified by measuring the ratio of the fluorescence intensity of JC-1-aggregates to JC-1-monomers.
