# Supplementary material for: SIRT1 plays a critical role in maintaining the viability of Yak Sertoli cells by regulating mitochondrial biogenesis via activating the PGC-1α-NRF-1-TFAM pathway
Source: Anim Biosci. 2026 Apr 16;39(7):251005. doi: 10.5713/ab.251005 (PMC13353117; doi:10.5713/ab.251005)
Supplement: Supplementary file 11 [file ab-251005-Supplementary-11.pdf]

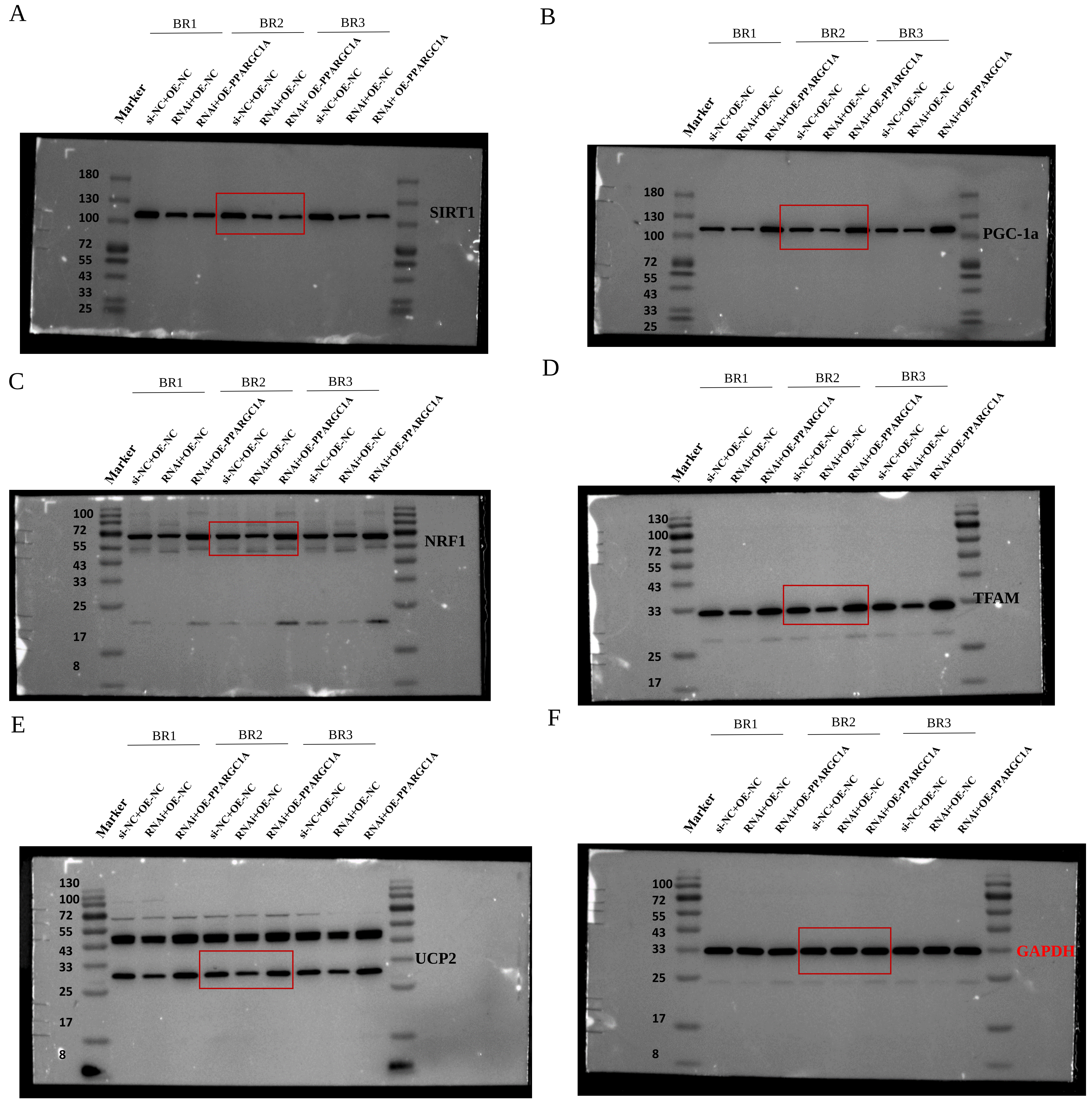

Supplement 11. The original Western Blot images corresponding to Figure 6. (A) Western blot analysis of SIRT1 in yak SCs of si-NC+OE-NC, RNAi+OE-NC and RNAi+OE-PPARGC1A group. (B) Western blot analysis of PGC-1α in yak SCs of si-NC+OE-NC, RNAi+OE-NC and RNAi+OE-PPARGC1A group. (C) Western blot analysis of NRF1 in yak SCs of si-NC+OE-NC, RNAi+OE-NC and RNAi+OE-PPARGC1A group. (D) Western blot analysis of TFAM in yak SCs of si-NC+OE-NC, RNAi+OE-NC and RNAi+OE-PPARGC1A group. (E) Western blot analysis of UCP2 in yak SCs of si-NC+OE-NC, RNAi+OE-NC and RNAi+OE-PPARGC1A group. (F) Western blot analysis of GAPDH in yak SCs of si-NC+OE-NC, RNAi+OE-NC and RNAi+OE-PPARGC1A group. BR1, BR2, and BR3 represents four independent biological replications. The size of each membrane was indicated on the images. The bands described in the main text were marked using red rectangles.
