# Supplementary material for: SIRT1 plays a critical role in maintaining the viability of Yak Sertoli cells by regulating mitochondrial biogenesis via activating the PGC-1α-NRF-1-TFAM pathway
Source: Anim Biosci. 2026 Apr 16;39(7):251005. doi: 10.5713/ab.251005 (PMC13353117; doi:10.5713/ab.251005)
Supplement: Supplementary file 12 [file ab-251005-Supplementary-12.pdf]

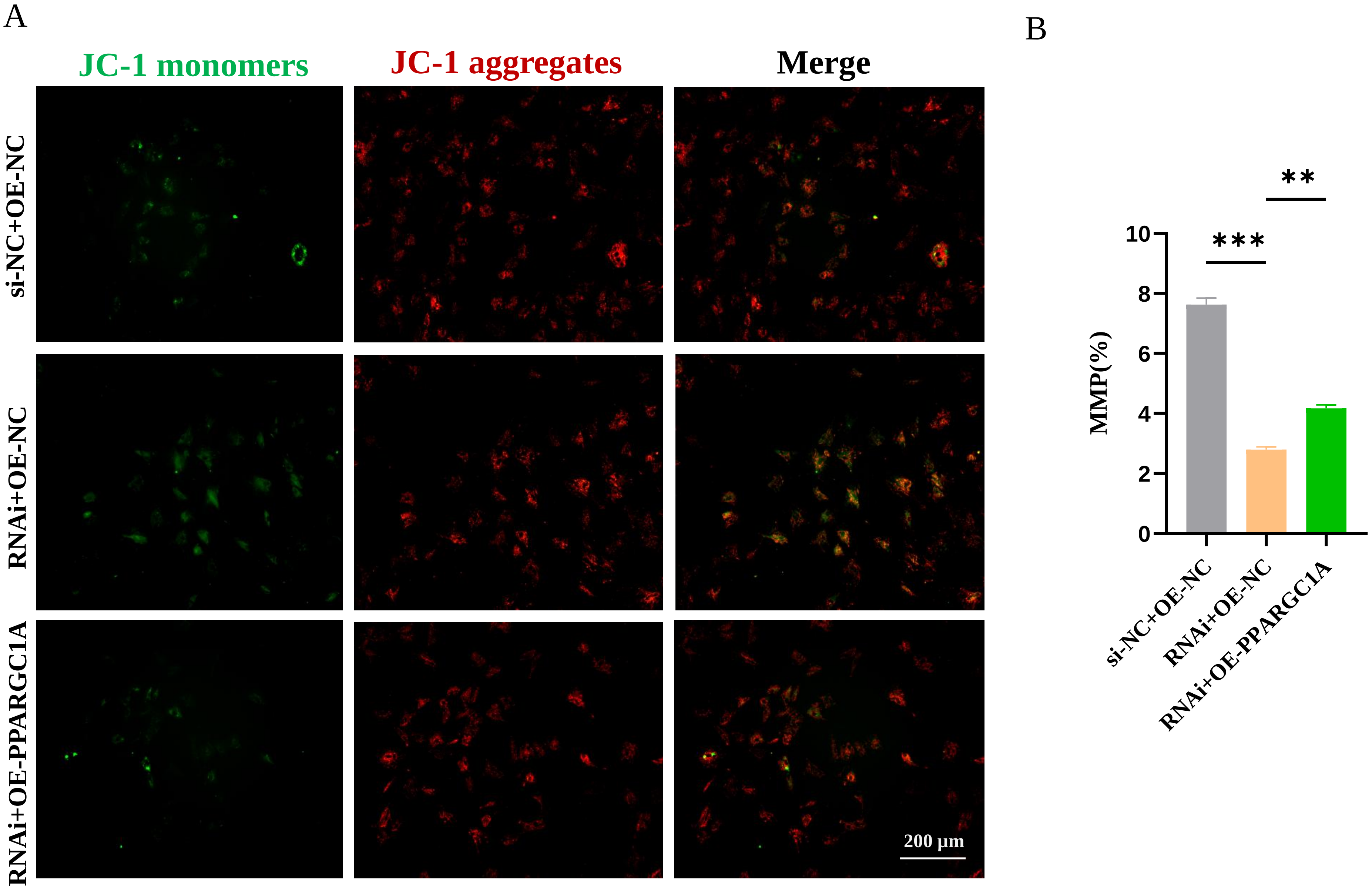

Supplement 12. Representative fluorescence images of JC-1 staining of si-NC+OE-NC group, RNAi+OE-NC group and RNAi+OE-PPARGC1A group. (A)MMP was detected using the JC-1 fluorescent probe. JC-1-monomers and JC-1-aggregates produce green and red fluorescence, respectively. (B) MMP in (A) was quantified by measuring the ratio of the fluorescence intensity of JC-1-aggregates to JC-1-monomers.
