# Supplementary material for: SIRT1 plays a critical role in maintaining the viability of Yak Sertoli cells by regulating mitochondrial biogenesis via activating the PGC-1α-NRF-1-TFAM pathway
Source: Anim Biosci. 2026 Apr 16;39(7):251005. doi: 10.5713/ab.251005 (PMC13353117; doi:10.5713/ab.251005)
Supplement: Supplementary file 5 [file ab-251005-Supplementary-5.pdf]

Supplement 5. Details of DE mRNAs in NC vs RNAi group.

<table>
<tr><th>GeneID</th><th>baseMean</th><th>log2FoldChange</th><th>lfcSE</th><th>stat</th><th>pvalue</th><th>padj</th><th>Up_Down</th><th>Sig</th><th>NR:Seq-id</th><th>NR:Score</th><th>NR:Evalue</th><th>NR:Description</th><th>NT:Seq-id</th><th>NT:Score</th><th>NT:Evalue</th><th>NT:Description</th><th>Uniprot:UniProtKB-AC</th><th>Uniprot:Score</th><th>Uniprot:Evalue</th><th>Uniprot:Description</th><th>COG:gene</th><th>COG:Score</th><th>COG:Eval</th><th>COG:num</th><th>Pfam:pfam_ID</th><th>Pfam:pfam_Name</th><th>Pfam:pfam_Description</th><th>GO:biological_process</th><th>GO:cellular_component</th><th>GO:molecular_function</th><th>KEGG:KO</th><th>KEGG:Description</th></tr>
<tr><td>C1QTNF3</td><td>3418.214</td><td>-0.90486</td><td>0.094574334</td><td>-9.5677</td><td>1.093E-21</td><td>1.26E-17</td><td>DOWN</td><td>yes</td><td>gi|528998337|ref|XP_005221661.1|</td><td>1324</td><td>2e-171</td><td>PREDICTED: complement C1q tumor necrosis factor-related protein 3 isoform X1 [Bos taurus]</td><td>gi|942032682|ref|XM_005887708.2|</td><td>2992</td><td>0</td><td>PREDICTED: Bos mutus C1q and tumor necrosis factor related protein 3 (C1QTNF3), transcript variant X1, mRNA</td><td>Q9BXJ4</td><td>362</td><td>2e-115</td><td>Complement C1q tumor necrosis factor-related protein 3</td><td>.</td><td>.</td><td>.</td><td>.</td><td>pfam00386</td><td>C1q</td><td>C1q domain. C1q is a subunit of the C1 enzyme complex that activates the serum complement system.</td><td>GO:0035356|cellular triglyceride homeostasis;GO:0045444|fat cell differentiation;GO:0010629|negative regulation of gene expression;GO:0045721|negative regulation of gluconeogenesis;GO:0050728|negative regulation of inflammatory response;GO:1900165|negative regulation of interleukin-6 secretion;GO:0071638|negative regulation of monocyte chemotactic protein-1 production;GO:0042347|negative regulation of NF-kappaB import into nucleus;GO:0070165|positive regulation of adiponectin</td><td>GO:0005623|cell;GO:0005581|collagen trimer;GO:0070062|extracellular exosome;GO:0005615|extracellular space;GO:0016020|membrane;</td><td>.</td><td>.</td><td>.</td></tr>
<tr><td>CEMIP</td><td>4180.668</td><td>0.951499</td><td>0.106826897</td><td>8.906924</td><td>5.247E-19</td><td>3.01E-15</td><td>UP</td><td>yes</td><td>gi|594104928|ref|XP_006075432.1|</td><td>7322</td><td>0</td><td>PREDICTED: protein KIAA1199 homolog isoform X1 [Bubalus bubalis]</td><td>gi|983003678|ref|XM_597255.8|</td><td>6996</td><td>0</td><td>PREDICTED: Bos taurus cell migration inducing protein, hyaluronan binding (CEMIP), transcript variant X1, mRNA</td><td>Q8WUJ3</td><td>2618</td><td>0</td><td>Cell migration-inducing and hyaluronan-binding protein</td><td>.</td><td>.</td><td>.</td><td>.</td><td>pfam10162</td><td>G8</td><td>G8 domain. This domain is found in disease proteins PKHD1 and KIAA1199 and is named G8 after its 8 conserved glycines. It is</td><td>GO:0030213|hyaluronan biosynthetic process;GO:0030214|hyaluronan catabolic process;GO:0030335|positive regulation of cell migration;GO:0010800|positive regulation of peptidyl-threonine phosphorylation;GO:1900020|positive regulation of protein kinase C activity;GO:0090314|positive regulation of protein targeting to membrane;GO:0051281|positive regulation of release of sequestered calcium ion into cytosol;GO:0007605|sensory perception of sound;</td><td>GO:0005905|coated pit;GO:0030665|clathrin-coated vesicle membrane;GO:0005737|cytoplasm;GO:0005783|endoplasmic</td><td>GO:0032050|clathrin heavy chain binding;GO:0046923|ER retention</td><td>K19031</td><td>CEMIP, KIAA1199; cell migration-inducing and hyaluronan-binding</td></tr>
<tr><td>NREP</td><td>2029.642</td><td>-0.67876</td><td>0.094484583</td><td>-7.18385</td><td>6.778E-13</td><td>2.6E-09</td><td>DOWN</td><td>yes</td><td>gi|602731729|ref|XP_007451266.1|</td><td>392</td><td>1e-41</td><td>PREDICTED: neuronal regeneration-related protein-like [Lipotes vexillifer]</td><td>gi|555981718|ref|XM_005902710.1|</td><td>1935</td><td>0</td><td>PREDICTED: Bos mutus neuronal regeneration related protein (NREP), mRNA</td><td>Q5NVD3</td><td>119</td><td>2e-30</td><td>Neuronal regeneration-related protein</td><td>.</td><td>.</td><td>.</td><td>.</td><td>pfam11092</td><td>Alveol-reg_P311</td><td>Neuronal protein 3.1 (p311). P311 has several PEST-like motifs and is found in neuron and muscle cells. P311 could have some function in</td><td>.</td><td>GO:0005737|cytoplasm;</td><td>.</td><td>.</td><td>.</td></tr>
<tr><td>INHBA</td><td>23088.23</td><td>-0.58842</td><td>0.084833841</td><td>-6.93613</td><td>4.03E-12</td><td>1.16E-08</td><td>DOWN</td><td>yes</td><td>gi|27805949|ref|NP_776788.1|</td><td>2103</td><td>0</td><td>inhibin beta A chain precursor [Bos taurus]</td><td>gi|982921918|ref|XM_005205620.3|</td><td>5874</td><td>0</td><td>PREDICTED: Bos taurus inhibin beta A (INHBA), transcript variant X1, mRNA</td><td>P07995</td><td>814</td><td>0</td><td>Inhibin beta A chain</td><td>.</td><td>.</td><td>.</td><td>.</td><td>pfam00019</td><td>TGF_beta</td><td>Transforming growth factor beta like domain.</td><td>GO:0032924|activin receptor signaling pathway;GO:0007050|cell cycle arrest;GO:0048468|cell development;GO:0035987|endodermal cell differentiation;GO:0097191|extrinsic apoptotic signaling pathway;GO:0061029|eyelid development in camera-type eye;GO:0000082|G1/S transition of mitotic cell cycle;GO:0097154|GABAergic neuron differentiation;GO:0040007|growth;GO:0001942|hair follicle development;GO:0002244|hematopoietic progenitor cell differentiation;GO:0042541|hemoglobin biosynthetic process;GO:0008584|male</td><td>GO:0043509|activin A complex;GO:0005576|extracellular region;GO:0005615|extracellular space;GO:0043512|inhibin A</td><td>GO:0005125|cytokine activity;GO:0042802|identical protein</td><td>K04667</td><td>INHB; inhibin, beta</td></tr>
<tr><td>TGM3</td><td>355.8312</td><td>1.249695</td><td>0.183669967</td><td>6.804023</td><td>1.017E-11</td><td>2.34E-08</td><td>UP</td><td>yes</td><td>gi|742103668|ref|XP_010834823.1|</td><td>3629</td><td>0</td><td>PREDICTED: protein-glutamine gamma-glutamyltransferase E [Bison bison bison]</td><td>gi|742103667|ref|XM_010836521.1|</td><td>2670</td><td>0</td><td>PREDICTED: Bison bison bison transglutaminase 3 (TGM3), mRNA</td><td>A6QP57</td><td>1392</td><td>0</td><td>Protein-glutamine gamma-glutamyltransferase E</td><td>.</td><td>.</td><td>.</td><td>.</td><td>pfam00868</td><td>Transglut_N</td><td>Transglutaminase family.</td><td>GO:0031424|keratinization;GO:0018149|peptide cross-linking;</td><td>.</td><td>GO:0005509|calcium ion binding;GO:0003810|protein-glutamine</td><td>K05620</td><td>TGM3; transglutaminase 3 [EC:2.3.2.13]</td></tr>
<tr><td>UBTD2</td><td>507.2817</td><td>-0.79041</td><td>0.120174392</td><td>-6.57719</td><td>4.794E-11</td><td>9.18E-08</td><td>DOWN</td><td>yes</td><td>gi|62751565|ref|NP_001015679.1|</td><td>1261</td><td>2e-162</td><td>ubiquitin domain-containing protein 2 [Bos taurus]</td><td>gi|965882897|ref|XM_012154018.2|</td><td>2722</td><td>0</td><td>PREDICTED: Ovis aries musimon ubiquitin domain containing 2 (UBTD2), transcript variant X3, mRNA</td><td>Q5EAE3</td><td>490</td><td>5e-164</td><td>Ubiquitin domain-containing protein 2</td><td>.</td><td>.</td><td>.</td><td>.</td><td>pfam00240</td><td>ubiquitin</td><td>Ubiquitin family. This family contains a number of ubiquitin-like proteins: SUMO (smt3 homologue) Nedd8, Elongin B, Rub1, and</td><td>.</td><td>GO:0005737|cytoplasm;</td><td>.</td><td>.</td><td>.</td></tr>
<tr><td>SLC6A2</td><td>891.567</td><td>-0.68479</td><td>0.106182255</td><td>-6.44918</td><td>1.125E-10</td><td>1.85E-07</td><td>DOWN</td><td>yes</td><td>gi|465980633|ref|XP_004264955.1|</td><td>3199</td><td>0</td><td>PREDICTED: sodium-dependent noradrenaline transporter isoform X1 [Orcinus orca]</td><td>gi|926713747|ref|XM_005691989.2|</td><td>5053</td><td>0</td><td>PREDICTED: Capra hircus solute carrier family 6 (neurotransmitter transporter), member 2 (SLC6A2), transcript variant X1, mRNA</td><td>P51143</td><td>1230</td><td>0</td><td>Sodium-dependent noradrenaline transporter</td><td>VC2012</td><td>228</td><td>2.65e-63</td><td>COG0733</td><td>pfam00209</td><td>SNF</td><td>Sodium:neurotransmitter symporter family.</td><td>GO:0015874|norepinephrine transport;</td><td>GO:0005887|integral component of plasma membrane;GO:0043005|neuron projection;</td><td>GO:0046872|metal ion binding;GO:0005328|neurotransmitter:s</td><td>K05035</td><td>SLC6A2; solute carrier family 6 (neurotransmitter transporter,</td></tr>
<tr><td>INCENP</td><td>767.4576</td><td>0.706031</td><td>0.110491896</td><td>6.389891</td><td>1.66E-10</td><td>2.2E-07</td><td>UP</td><td>yes</td><td>gi|555978840|ref|XP_005901357.1|</td><td>2005</td><td>0</td><td>PREDICTED: inner centromere protein [Bos mutus]</td><td>gi|594037219|ref|XM_006043187.1|</td><td>2830</td><td>0</td><td>PREDICTED: Bubalus bubalis inner centromere protein antigens 135/155kDa (INCENP), transcript variant X1, mRNA</td><td>Q9NQS7</td><td>526</td><td>1e-168</td><td>Inner centromere protein</td><td>.</td><td>.</td><td>.</td><td>.</td><td>pfam03941</td><td>INCENP_ARK-bind</td><td>Inner centromere protein, ARK binding region. This region of the inner centromere protein has been found to be necessary and</td><td>GO:0007059|chromosome segregation;GO:0000910|cytokinesis;GO:0007067|mitotic nuclear division;GO:0016925|protein sumoylation;GO:0007062|sister chromatid cohesion;</td><td>GO:0000801|central element;GO:0010369|chromocenter;GO:0000775|chromosome, centromeric region;GO:0000777|condensed</td><td>.</td><td>K11515</td><td>INCENP; inner centromere protein</td></tr>
<tr><td>DPT</td><td>1782.034</td><td>-0.60613</td><td>0.094945591</td><td>-6.384</td><td>1.725E-10</td><td>2.2E-07</td><td>DOWN</td><td>yes</td><td>gi|587017864|ref|XP_003999210.2|</td><td>902</td><td>2e-114</td><td>PREDICTED: dermatopontin [Felis catus]</td><td>gi|942058289|ref|XM_005897193.2|</td><td>1920</td><td>0</td><td>PREDICTED: Bos mutus dermatopontin (DPT), mRNA</td><td>P19427</td><td>347</td><td>4e-114</td><td>Dermatopontin</td><td>.</td><td>.</td><td>.</td><td>.</td><td>pfam14704</td><td>DERM</td><td>Dermatopontin. Members of this family mediate cell adhesion via cell surface integrin binding. They also induce haemagglutination and</td><td>GO:0007155|cell adhesion;GO:0030199|collagen fibril organization;GO:0008285|negative regulation of cell proliferation;</td><td>GO:0070062|extracellular exosome;GO:0031012|extracellular matrix;GO:0005615|extracellular space;GO:0005578|proteinaceous</td><td>.</td><td>.</td><td>.</td></tr>
<tr><td>MYBL2</td><td>1177.405</td><td>0.722259</td><td>0.115941605</td><td>6.229502</td><td>4.679E-10</td><td>4.97E-07</td><td>UP</td><td>yes</td><td>gi|115495777|ref|NP_001068916.1|</td><td>3289</td><td>0</td><td>myb-related protein B [Bos taurus]</td><td>gi|402743717|ref|NM_001075448.2|</td><td>2655</td><td>0</td><td>Bos taurus v-myb avian myeloblastosis viral oncogene homolog-like 2 (MYBL2), mRNA</td><td>P10244</td><td>1158</td><td>0</td><td>Myb-related protein B</td><td>ECU11g1690</td><td>147</td><td>2.10e-39</td><td>COG5147</td><td>pfam09316</td><td>Cmyb_C</td><td>C-myb, C-terminal. Members of this family are predominantly found in the proto-oncogene c-myb and the viral transforming</td><td>GO:0030154|cell differentiation;GO:0090307|mitotic spindle assembly;GO:0045944|positive regulation of transcription from RNA polymerase II promoter;GO:0051726|regulation of cell cycle;</td><td>GO:0031523|Myb complex;GO:0005654|nucleoplasm;</td><td>GO:0000978|RNA polymerase II core promoter proximal region sequence-</td><td>K09421</td><td>MYBL; myb-related protein</td></tr>
<tr><td>DIPK2A</td><td>511.7345</td><td>-0.79277</td><td>0.127313365</td><td>-6.22689</td><td>4.758E-10</td><td>4.97E-07</td><td>DOWN</td><td>yes</td><td>gi|329663892|ref|NP_001192580.1|</td><td>2040</td><td>0</td><td>deleted in autism protein 1 [Bos taurus]</td><td>gi|329663891|ref|NM_001205651.1|</td><td>3860</td><td>0</td><td>Bos taurus chromosome 1 open reading frame, human C3orf58 (C1H3orf58), mRNA</td><td>Q8NDZ4</td><td>788</td><td>0</td><td>Deleted in autism protein 1</td><td>.</td><td>.</td><td>.</td><td>.</td><td>pfam12260</td><td>PIP49_C</td><td>Protein-kinase domain of FAM69. This is the C-terminal region of a family of FAM69 proteins from Metazoa and Viridiplantae that are</td><td>GO:0060038|cardiac muscle cell proliferation;GO:0014066|regulation of phosphatidylinositol 3-kinase signaling;</td><td>GO:0030126|COPI vesicle coat;GO:0005615|extracellular space;GO:0000139|Golgi membrane;</td><td>.</td><td>.</td><td>.</td></tr>
<tr><td>MKI67</td><td>1210.599</td><td>0.791606</td><td>0.128028966</td><td>6.183026</td><td>6.288E-10</td><td>6.02E-07</td><td>UP</td><td>yes</td><td>gi|942064524|ref|XP_014334735.1|</td><td>3999</td><td>0</td><td>PREDICTED: antigen KI-67 [Bos mutus]</td><td>gi|742199218|ref|XM_010859148.1|</td><td>2842</td><td>0</td><td>PREDICTED: Bison bison bison marker of proliferation Ki-67 (MKI67), transcript variant X4, mRNA</td><td>P46013</td><td>357</td><td>8e-100</td><td>Proliferation marker protein Ki-67</td><td>.</td><td>.</td><td>.</td><td>.</td><td>pfam08065</td><td>K167R</td><td>K167R (NUC007) repeat. This family represents the K167/Chmadrin repeat. The function of this repeat is unknown.</td><td>GO:0031100|organ regeneration;GO:0008283|cell proliferation;GO:0034605|cellular response to heat;GO:0006259|DNA metabolic process;GO:0030212|hyaluronan metabolic process;GO:0007126|meiotic nuclear division;GO:1902275|regulation of chromatin organization;GO:0051983|regulation of chromosome segregation;GO:0007088|regulation of mitotic nuclear division;GO:0014070|response to organic cyclic compound;</td><td>GO:0000775|chromosome, centromeric region;GO:0000793|condensed chromosome;GO:0005737|cytoplasm;GO:0016020|membrane;GO:0005730|nucleolus;G</td><td>GO:0005524|ATP binding;GO:0003677|DNA binding;GO:0044822</td><td>K17582</td><td>MKI67; antigen KI-67</td></tr>
<tr><td>GTSE1</td><td>618.7176</td><td>0.718473</td><td>0.120948782</td><td>5.940311</td><td>2.845E-09</td><td>2.51E-06</td><td>UP</td><td>yes</td><td>gi|942102394|ref|XP_014339042.1|</td><td>3092</td><td>0</td><td>PREDICTED: G2 and S phase-expressed protein 1 [Bos mutus]</td><td>gi|742082647|ref|XM_010859532.1|</td><td>2677</td><td>0</td><td>PREDICTED: Bison bison bison G-2 and S-phase expressed 1 (GTSE1), transcript variant X1, mRNA</td><td>Q9NYZ3</td><td>583</td><td>0</td><td>G2 and S phase-expressed protein 1</td><td>.</td><td>.</td><td>.</td><td>.</td><td>pfam15259</td><td>GTSE1_N</td><td>G-2 and S-phase expressed 1. This family is the N-terminus of GTSE1 proteins. GTSE-1 (G2 and S phase-expressed-1) protein is specifically</td><td>GO:0006977|DNA damage response, signal transduction by p53 class mediator resulting in cell cycle arrest;GO:0007017|microtubule-based process;GO:1904527|negative regulation of microtubule binding;GO:0030335|positive regulation of cell migration;GO:0046827|positive regulation of protein export from nucleus;GO:1900182|positive regulation of protein localization to nucleus;GO:0043161|proteasome-mediated ubiquitin-dependent protein catabolic process;GO:0050821|protein stabilization;GO:1902749|regulation of cell cycle G2/M phase</td><td>GO:0005881|cytoplasmic microtubule;GO:0005829|cytosol;GO:0016020|membrane;GO:0005654|nucleoplasm;</td><td>.</td><td>K10129</td><td>GTSE1, B99; G-2 and S-phase expressed protein 1</td></tr>
<tr><td>TROAP</td><td>299.263</td><td>0.995676</td><td>0.169404102</td><td>5.877518</td><td>4.165E-09</td><td>3.42E-06</td><td>UP</td><td>yes</td><td>gi|555968854|ref|XP_005896458.1|</td><td>3247</td><td>0</td><td>PREDICTED: tastin isoform X1 [Bos mutus]</td><td>gi|742193573|ref|XM_010857761.1|</td><td>2322</td><td>0</td><td>PREDICTED: Bison bison bison trophinin associated protein (TROAP), transcript variant X2, mRNA</td><td>Q12815</td><td>836</td><td>0</td><td>Tastin</td><td>.</td><td>.</td><td>.</td><td>.</td><td>pfam09770</td><td>PAT1</td><td>Topoisomerase II-associated protein PAT1. Members of this family are necessary for accurate chromosome transmission during</td><td>GO:0007155|cell adhesion;</td><td>GO:0005737|cytoplasm;</td><td>.</td><td>.</td><td>.</td></tr>
<tr><td>ZNF142</td><td>588.8506</td><td>0.835522</td><td>0.144750659</td><td>5.772145</td><td>7.827E-09</td><td>6E-06</td><td>UP</td><td>yes</td><td>gi|528900959|ref|XP_877798.4|</td><td>8829</td><td>0</td><td>PREDICTED: zinc finger protein 142 [Bos taurus]</td><td>gi|982977668|ref|XM_872705.6|</td><td>6048</td><td>0</td><td>PREDICTED: Bos taurus zinc finger protein 142 (ZNF142), mRNA</td><td>P52746</td><td>2550</td><td>0</td><td>Zinc finger protein 142</td><td>.</td><td>.</td><td>.</td><td>.</td><td>pfam04388</td><td>Hamartin</td><td>Hamartin protein. This family includes the hamartin protein which is thought to function as a tumor suppressor. The hamartin</td><td>GO:0006355|regulation of transcription, DNA-templated;GO:0006351|transcription, DNA-templated;</td><td>GO:0005634|nucleus;</td><td>GO:0003677|DNA binding;GO:0046872|metal ion binding;</td><td>.</td><td>.</td></tr>
<tr><td>LOC102271913</td><td>2676.565</td><td>-0.65124</td><td>0.113124562</td><td>-5.75685</td><td>8.57E-09</td><td>6.05E-06</td><td>DOWN</td><td>yes</td><td>gi|440902835|gb|ELR53576.1|</td><td>495</td><td>9e-60</td><td>C-C motif chemokine 2, partial [Bos mutus]</td><td>gi|942066099|ref|XM_005899695.2|</td><td>761</td><td>0</td><td>PREDICTED: Bos mutus C-C motif chemokine 2 (LOC102271913), mRNA</td><td>P28291</td><td>177</td><td>1e-54</td><td>C-C motif chemokine 2</td><td>.</td><td>.</td><td>.</td><td>.</td><td>pfam00048</td><td>IL8</td><td>Small cytokines (intecrine/chemokine), interleukin-8 like. Includes a number of secreted growth factors and</td><td>GO:0043615|astrocyte cell migration;GO:0044344|cellular response to fibroblast growth factor stimulus;GO:0071346|cellular response to interferon-gamma;GO:0071347|cellular response to interleukin-1;GO:0071407|cellular response to organic cyclic compound;GO:0071356|cellular response to tumor necrosis factor;GO:0070098|chemokine-mediated signaling pathway;GO:0007010|cytoskeleton organization;GO:0007186|G-protein coupled receptor signaling pathway;GO:0035684|helper T cell</td><td>GO:0005615|extracellular space;GO:0005622|intracellular;</td><td>GO:0048020|CCR chemokine receptor binding;GO:0008009|chemokine activity;</td><td>K14624</td><td>CCL2, MCP1; C-C motif chemokine 2</td></tr>
<tr><td>ALDH1A1</td><td>4070.13</td><td>-0.66541</td><td>0.115756846</td><td>-5.74838</td><td>9.01E-09</td><td>6.05E-06</td><td>DOWN</td><td>yes</td><td>gi|742143647|ref|XP_010843188.1|</td><td>2596</td><td>0</td><td>PREDICTED: retinal dehydrogenase 1 [Bison bison bison]</td><td>gi|742143646|ref|XM_010844886.1|</td><td>2074</td><td>0</td><td>PREDICTED: Bison bison bison aldehyde dehydrogenase 1 family, member A1 (ALDH1A1), mRNA</td><td>P48644</td><td>1004</td><td>0</td><td>Retinal dehydrogenase 1</td><td>SPAC9E9.09c</td><td>523</td><td>1.51e-179</td><td>COG1012</td><td>pfam00171</td><td>Aldedh</td><td>Aldehyde dehydrogenase family. This family of dehydrogenases act on aldehyde substrates. Members use NADP as a cofactor. The family</td><td>GO:0042572|retinol metabolic process;</td><td>GO:0005737|cytoplasm;GO:0070062|extracellular exosome;</td><td>GO:0018479|benzaldehyde dehydrogenase (NAD+)</td><td>K07249</td><td>E1.2.1.36; retinal dehydrogenase [EC:1.2.1.36]</td></tr>
<tr><td>COX17</td><td>227.9621</td><td>-0.96697</td><td>0.168467246</td><td>-5.73979</td><td>9.479E-09</td><td>6.05E-06</td><td>DOWN</td><td>yes</td><td>gi|942083730|ref|XP_014336829.1|</td><td>449</td><td>6e-55</td><td>PREDICTED: cytochrome c oxidase copper chaperone [Bos mutus]</td><td>gi|982909953|ref|XM_002684734.5|</td><td>412</td><td>0</td><td>PREDICTED: Bos taurus COX17 cytochrome c oxidase assembly homolog (S. cerevisiae) (COX17), transcript variant X1, mRNA</td><td>Q6J3Q7</td><td>121</td><td>8e-35</td><td>Cytochrome c oxidase copper chaperone</td><td>.</td><td>.</td><td>.</td><td>.</td><td>pfam05051</td><td>COX17</td><td>Cytochrome C oxidase copper chaperone (COX17). Cox17 is essential for the assembly of functional cytochrome c oxidase</td><td>GO:0006825|copper ion transport;GO:0033617|mitochondrial respiratory chain complex IV assembly;</td><td>GO:0005758|mitochondrial intermembrane space;</td><td>GO:0016531|copper chaperone activity;</td><td>K02260</td><td>COX17; cytochrome c oxidase assembly protein subunit 17</td></tr>
<tr><td>ESPL1</td><td>495.9585</td><td>0.696116</td><td>0.122010111</td><td>5.705394</td><td>1.161E-08</td><td>6.79E-06</td><td>UP</td><td>yes</td><td>gi|555980550|ref|XP_005902199.1|</td><td>10380</td><td>0</td><td>PREDICTED: separin [Bos mutus]</td><td>gi|555980549|ref|XM_005902137.1|</td><td>6565</td><td>0</td><td>PREDICTED: Bos mutus extra spindle pole bodies like 1, separase (ESPL1), mRNA</td><td>Q14674</td><td>3326</td><td>0</td><td>Separin</td><td>SPCC5E4.04</td><td>163</td><td>3.38e-39</td><td>COG5155</td><td>pfam03568</td><td>Peptidase_C50</td><td>Peptidase family C50.</td><td>GO:0006915|apoptotic process;GO:0000910|cytokinesis;GO:0040001|establishment of mitotic spindle localization;GO:0045143|homologous chromosome segregation;GO:0051307|meiotic chromosome separation;GO:0000212|meiotic spindle organization;GO:0000070|mitotic sister chromatid segregation;GO:0045875|negative regulation of sister chromatid cohesion;GO:0045842|positive regulation of mitotic metaphase/anaphase transition;GO:1901970|positive regulation of mitotic sister chromatid separation;</td><td>GO:0005813|centrosome;GO:0005737|cytoplasm;GO:0005829|cytosol;GO:0072686|mitotic spindle;GO:0005634|nucleus;</td><td>GO:0003824|catalytic activity;GO:0004197|cysteine-type</td><td>K02365</td><td>ESP1; separase [EC:3.4.22.49]</td></tr>
<tr><td>CCN4</td><td>636.1333</td><td>-0.67884</td><td>0.119137409</td><td>-5.69793</td><td>1.213E-08</td><td>6.79E-06</td><td>DOWN</td><td>yes</td><td>gi|528975502|ref|XP_005215377.1|</td><td>1541</td><td>0</td><td>PREDICTED: WNT1-inducible-signaling pathway protein 1 [Bos taurus]</td><td>gi|982994280|ref|XM_015466295.1|</td><td>3219</td><td>0</td><td>PREDICTED: Bos taurus WNT1 inducible signaling pathway protein 1 (WISP1), mRNA</td><td>O95388</td><td>533</td><td>2e-176</td><td>WNT1-inducible-signaling pathway protein 1</td><td>.</td><td>.</td><td>.</td><td>.</td><td>pfam00219</td><td>IGFBP</td><td>Insulin-like growth factor binding protein.</td><td>GO:0007155|cell adhesion;GO:0007267|cell-cell signaling;GO:0060548|negative regulation of cell death;GO:0001558|regulation of cell growth;GO:0007165|signal transduction;GO:0016055|Wnt signaling pathway;</td><td>GO:0005737|cytoplasm;GO:0005615|extracellular space;GO:0005578|proteinaceous extracellular matrix;</td><td>GO:0008201|heparin binding;GO:0005178|integrin binding;</td><td>.</td><td>.</td></tr>
<tr><td>ATP5ME</td><td>420.7577</td><td>-1.02779</td><td>0.18063829</td><td>-5.68975</td><td>1.272E-08</td><td>6.79E-06</td><td>DOWN</td><td>yes</td><td>gi|926694687|ref|XP_013820338.1|</td><td>431</td><td>2e-51</td><td>PREDICTED: ATP synthase subunit e, mitochondrial [Capra hircus]</td><td>gi|926694686|ref|XM_013964884.1|</td><td>314</td><td>4e-162</td><td>PREDICTED: Capra hircus ATP synthase, H+ transporting, mitochondrial Fo complex, subunit E (ATP5I), mRNA</td><td>Q00361</td><td>103</td><td>2e-27</td><td>ATP synthase subunit e, mitochondrial</td><td>.</td><td>.</td><td>.</td><td>.</td><td>pfam05680</td><td>ATP-synt_E</td><td>ATP synthase E chain. This family consists of several ATP synthase E chain sequences which are components of the CF(0) subunit.</td><td>GO:0015986|ATP synthesis coupled proton transport;</td><td>GO:0005753|mitochondrial proton-transporting ATP synthase complex;GO:0000276|mitochondrial proton-transporting ATP synthase complex,</td><td>GO:0015078|hydrogen ion transmembrane transporter activity;</td><td>K02129</td><td>ATPeF0E, ATP5I; F-type H+-transporting ATPase subunit e</td></tr>
<tr><td>ECE1</td><td>1807.331</td><td>0.673398</td><td>0.118427766</td><td>5.686154</td><td>1.299E-08</td><td>6.79E-06</td><td>UP</td><td>yes</td><td>gi|555986822|ref|XP_005905267.1|</td><td>3995</td><td>0</td><td>PREDICTED: endothelin-converting enzyme 1 isoform X1 [Bos mutus]</td><td>gi|982915376|ref|XM_005203072.3|</td><td>4267</td><td>0</td><td>PREDICTED: Bos taurus endothelin converting enzyme 1 (ECE1), transcript variant X4, mRNA</td><td>P42891</td><td>1531</td><td>0</td><td>Endothelin-converting enzyme 1</td><td>MA2001</td><td>359</td><td>9.95e-108</td><td>COG3590</td><td>pfam05649</td><td>Peptidase_M13_N</td><td>Peptidase family M13. M13 peptidases are well-studied proteases found in a wide range of organisms including mammals and</td><td>GO:0016486|peptide hormone processing;GO:0016485|protein processing;GO:0006508|proteolysis;</td><td>GO:0016021|integral component of membrane;GO:0016020|membrane;GO:0005886|plasma membrane;</td><td>GO:0004175|endopeptidase activity;GO:0046872|metal ion</td><td>.</td><td>.</td></tr>
<tr><td>LOC138989196</td><td>655.2826</td><td>-0.78439</td><td>0.138615974</td><td>-5.65876</td><td>1.525E-08</td><td>7.62E-06</td><td>DOWN</td><td>yes</td><td>gi|114050967|ref|NP_001040099.1|</td><td>285</td><td>2e-25</td><td>bitter taste receptor Bota-T2R65A [Bos taurus]</td><td>gi|850485237|gb|CP011893.1|</td><td>918</td><td>0</td><td>Ovis canadensis canadensis isolate 43U chromosome 8 sequence</td><td>Q9UKX7</td><td>72.8</td><td>3e-12</td><td>Nuclear pore complex protein Nup50</td><td>.</td><td>.</td><td>.</td><td>.</td><td>pfam06079</td><td>Apyrase</td><td>Apyrase. This family consists of several eukaryotic apyrase proteins (EC:3.6.1.5). The salivary apyrases of blood-feeding</td><td>GO:0031047|gene silencing by RNA;GO:0075733|intracellular transport of virus;GO:0007077|mitotic nuclear envelope disassembly;GO:0006406|mRNA export from nucleus;GO:0006606|protein import into nucleus;GO:0016925|protein sumoylation;GO:1900034|regulation of cellular response to heat;GO:0010827|regulation of glucose transport;GO:0006409|tRNA export from nucleus;GO:0016032|viral process;GO:0019083|viral transcription;</td><td>GO:0005737|cytoplasm;GO:0031965|nuclear membrane;GO:0005643|nuclear pore;GO:0005654|nucleoplasm;</td><td>GO:0008536|Ran GTPase binding;</td><td>K14295</td><td>NUP50, NPAP60; nuclear pore complex protein Nup50</td></tr>
<tr><td>GNG11</td><td>243.7406</td><td>-0.85223</td><td>0.153414583</td><td>-5.55508</td><td>2.775E-08</td><td>1.33E-05</td><td>DOWN</td><td>yes</td><td>gi|66792836|ref|NP_001019694.1|</td><td>367</td><td>4e-40</td><td>guanine nucleotide-binding protein G(I)/G(S)/G(O) subunit gamma-11 precursor [Bos taurus]</td><td>gi|942097318|ref|XM_005909693.2|</td><td>1026</td><td>0</td><td>PREDICTED: Bos mutus guanine nucleotide binding protein (G protein), gamma 11 (GNG11), mRNA</td><td>Q5E9F0</td><td>145</td><td>1e-41</td><td>Guanine nucleotide-binding protein G(I)/G(S)/G(O) subunit gamma-11</td><td>.</td><td>.</td><td>.</td><td>.</td><td>pfam00631</td><td>G-gamma</td><td>GGL domain. G-protein gamma like domains (GGL) are found in the gamma subunit of the heterotrimeric G protein complex</td><td>GO:0007186|G-protein coupled receptor signaling pathway;</td><td>GO:0005834|heterotrimeric G-protein complex;</td><td>GO:0004871|signal transducer activity;</td><td>K04546</td><td>GNG11; guanine nucleotide-binding protein G(I)/G(S)/G(O)</td></tr>
<tr><td>TINAGL1</td><td>898.5466</td><td>0.652261</td><td>0.118005429</td><td>5.527382</td><td>3.25E-08</td><td>1.49E-05</td><td>UP</td><td>yes</td><td>gi|296490232|tpg|DAA32345.1|</td><td>2440</td><td>0</td><td>TPA: tubulointerstitial nephritis antigen-like 1-like [Bos taurus]</td><td>gi|742105181|ref|XM_010836890.1|</td><td>1911</td><td>0</td><td>PREDICTED: Bison bison bison tubulointerstitial nephritis antigen-like 1 (TINAGL1), mRNA</td><td>Q9GZM7</td><td>827</td><td>0</td><td>Tubulointerstitial nephritis antigen-like</td><td>.</td><td>.</td><td>.</td><td>.</td><td>pfam00112</td><td>Peptidase_C1</td><td>Papain family cysteine protease.</td><td>GO:0007155|cell adhesion;GO:0016197|endosomal transport;GO:0006955|immune response;</td><td>GO:0005737|cytoplasm;GO:0070062|extracellular exosome;GO:0031012|extracellular matrix;GO:0005576|extracellular region;GO:0005615|extracellular space;</td><td>GO:0008234|cysteine-type peptidase activity;GO:0005201|extracellular matrix</td><td>.</td><td>.</td></tr>
<tr><td>MAPK6</td><td>3722.887</td><td>-0.58084</td><td>0.105485828</td><td>-5.50632</td><td>3.664E-08</td><td>1.62E-05</td><td>Not_Sig</td><td>no</td><td>gi|359069633|ref|XP_002691011.2|</td><td>3777</td><td>0</td><td>PREDICTED: mitogen-activated protein kinase 6 isoform X1 [Bos taurus]</td><td>gi|942068525|ref|XM_005900456.2|</td><td>4082</td><td>0</td><td>PREDICTED: Bos mutus mitogen-activated protein kinase 6 (MAPK6), transcript variant X1, mRNA</td><td>Q16659</td><td>1424</td><td>0</td><td>Mitogen-activated protein kinase 6</td><td>.</td><td>.</td><td>.</td><td>.</td><td>pfam00069</td><td>Pkinase</td><td>Protein kinase domain.</td><td>GO:0007049|cell cycle;GO:0060999|positive regulation of dendritic spine development;GO:0006468|protein phosphorylation;GO:0007165|signal transduction;</td><td>GO:0005737|cytoplasm;GO:0005829|cytosol;GO:0005654|nucleoplasm;GO:0005634|nucleus;GO:0043234|protein complex;GO:0032156|septin cytoskeleton;</td><td>GO:0005524|ATP binding;GO:0004707|MAP kinase activity;GO:0004674</td><td>K06855</td><td>MAPK4_6; mitogen-activated protein kinase 4/6 [EC:2.7.11.24]</td></tr>
<tr><td>FAU</td><td>2301.62</td><td>-0.7078</td><td>0.129428496</td><td>-5.46865</td><td>4.535E-08</td><td>1.93E-05</td><td>DOWN</td><td>yes</td><td>gi|440907406|gb|ELR57560.1|</td><td>586</td><td>5e-74</td><td>Ubiquitin-like protein FUBI, partial [Bos mutus]</td><td>gi|942050176|ref|XM_005894064.2|</td><td>596</td><td>0</td><td>PREDICTED: Bos mutus Finkel-Biskis-Reilly murine sarcoma virus (FBR-MuSV) ubiquitously expressed (FAU), transcript variant X1, mRNA</td><td>P62865</td><td>142</td><td>8e-42</td><td>Ubiquitin-like protein FUBI</td><td>SPAC1805.12c_1</td><td>53.5</td><td>2.64e-09</td><td>COG5272</td><td>pfam04758</td><td>Ribosomal_S30</td><td>Ribosomal protein S30.</td><td>.</td><td>.</td><td>.</td><td>K02983</td><td>RP-S30e, RPS30; small subunit ribosomal protein S30e</td></tr>
<tr><td>IL1RL1</td><td>14903.2</td><td>0.61342</td><td>0.112560589</td><td>5.449686</td><td>5.046E-08</td><td>2.03E-05</td><td>UP</td><td>yes</td><td>gi|440902584|gb|ELR53360.1|</td><td>2904</td><td>0</td><td>Interleukin-1 receptor-like 1, partial [Bos mutus]</td><td>gi|982938365|ref|XM_005212445.3|</td><td>2816</td><td>0</td><td>PREDICTED: Bos taurus interleukin 1 receptor like 1 (IL1RL1), transcript variant X1, mRNA</td><td>Q01638</td><td>763</td><td>0</td><td>Interleukin-1 receptor-like 1</td><td>.</td><td>.</td><td>.</td><td>.</td><td>pfam01582</td><td>TIR</td><td>TIR domain. The Toll/interleukin-1 receptor (TIR) homology domain is an intracellular signalling domain found in MyD88, interleukin 1</td><td>GO:0006955|immune response;GO:0043124|negative regulation of I-kappaB kinase/NF-kappaB signaling;GO:0032689|negative regulation of interferon-gamma production;GO:0002826|negative regulation of T-helper 1 type immune response;GO:0090197|positive regulation of chemokine secretion;GO:0050729|positive regulation of inflammatory response;GO:0032754|positive regulation of interleukin-5 production;GO:0043032|positive regulation of macrophage activation;GO:0007165|signal transduction;</td><td>GO:0009897|external side of plasma membrane;GO:0016021|integral component of membrane;GO:0005886|plasma</td><td>GO:0004896|cytokine receptor activity;GO:0004908|interleukin-1</td><td>K05171</td><td>IL1RL1, ST2; interleukin 1 receptor-like 1</td></tr>
<tr><td>RPS28</td><td>1695.902</td><td>-0.94086</td><td>0.172718256</td><td>-5.44738</td><td>5.112E-08</td><td>2.03E-05</td><td>DOWN</td><td>yes</td><td>gi|45268967|gb|AAS55896.1|</td><td>321</td><td>3e-36</td><td>40S ribosomal protein S28, partial [Sus scrofa]</td><td>gi|942041151|ref|XM_005890722.2|</td><td>345</td><td>2e-179</td><td>PREDICTED: Bos mutus ribosomal protein S28 (RPS28), mRNA</td><td>P62859</td><td>112</td><td>2e-31</td><td>40S ribosomal protein S28</td><td>SPCC285.15c</td><td>87.8</td><td>1.46e-23</td><td>COG2053</td><td>pfam01200</td><td>Ribosomal_S28e</td><td>Ribosomal protein S28e.</td><td>GO:0030490|maturation of SSU-rRNA;GO:0000028|ribosomal small subunit assembly;GO:0042254|ribosome biogenesis;GO:0006412|translation;</td><td>GO:0022627|cytosolic small ribosomal subunit;</td><td>GO:0003723|RNA binding;GO:0003735|structural constituent of</td><td>K02979</td><td>RP-S28e, RPS28; small subunit ribosomal protein S28e</td></tr>
<tr><td>NCOR2</td><td>3741.106</td><td>0.830365</td><td>0.152728109</td><td>5.436884</td><td>5.422E-08</td><td>2.08E-05</td><td>UP</td><td>yes</td><td>gi|594064726|ref|XP_006056367.1|</td><td>6808</td><td>0</td><td>PREDICTED: nuclear receptor corepressor 2 isoform X4 [Bubalus bubalis]</td><td>gi|594064719|ref|XM_006056302.1|</td><td>8378</td><td>0</td><td>PREDICTED: Bubalus bubalis nuclear receptor corepressor 2 (NCOR2), transcript variant X1, mRNA</td><td>Q9Y618</td><td>2372</td><td>0</td><td>Nuclear receptor corepressor 2</td><td>.</td><td>.</td><td>.</td><td>.</td><td>pfam00249</td><td>Myb_DNA-binding</td><td>Myb-like DNA-binding domain. This family contains the DNA binding domains from Myb proteins, as well as the SANT</td><td>GO:0044255|cellular lipid metabolic process;GO:1903799|negative regulation of production of miRNAs involved in gene silencing by miRNA;GO:0000122|negative regulation of transcription from RNA polymerase II promoter;GO:0072365|regulation of cellular ketone metabolic process by negative regulation of transcription from RNA polymerase II promoter;GO:0006351|transcription, DNA-templated;</td><td>GO:0000118|histone deacetylase complex;GO:0016020|membrane;GO:0016604|nuclear body;GO:0000790|nuclear chromatin;GO:0016363|nuclear</td><td>GO:0003677|DNA binding;GO:0042826|histone deacetylase</td><td>K06065</td><td>NCOR2, SMRT; nuclear receptor co-repressor 2</td></tr>
<tr><td>PRR12</td><td>389.6942</td><td>0.872568</td><td>0.160987711</td><td>5.420088</td><td>5.957E-08</td><td>2.21E-05</td><td>UP</td><td>yes</td><td>gi|594114884|ref|XP_006080154.1|</td><td>3777</td><td>0</td><td>PREDICTED: LOW QUALITY PROTEIN: proline-rich protein 12 [Bubalus bubalis]</td><td>gi|983000295|ref|XM_015467617.1|</td><td>6784</td><td>0</td><td>PREDICTED: Bos taurus proline rich 12 (PRR12), mRNA</td><td>Q9ULL5</td><td>749</td><td>0</td><td>Proline-rich protein 12</td><td>.</td><td>.</td><td>.</td><td>.</td><td>pfam13926</td><td>DUF4211</td><td>Domain of unknown function (DUF4211).</td><td>.</td><td>.</td><td>.</td><td>.</td><td>.</td></tr>
<tr><td>UHRF1</td><td>681.186</td><td>0.590125</td><td>0.109093265</td><td>5.409359</td><td>6.325E-08</td><td>2.27E-05</td><td>UP</td><td>yes</td><td>gi|594087441|ref|XP_006067195.1|</td><td>4235</td><td>0</td><td>PREDICTED: E3 ubiquitin-protein ligase UHRF1 [Bubalus bubalis]</td><td>gi|982930881|ref|XM_010806982.2|</td><td>4117</td><td>0</td><td>PREDICTED: Bos taurus ubiquitin-like with PHD and ring finger domains 1 (UHRF1), transcript variant X1, mRNA</td><td>A7E320</td><td>1475</td><td>0</td><td>E3 ubiquitin-protein ligase UHRF1</td><td>DR1533</td><td>119</td><td>1.91e-28</td><td>COG3440</td><td>pfam02182</td><td>SAD_SRA</td><td>SAD/SRA domain. The domain goes by several names including SAD, SRA and YDG. It adopts a beta barrel, modified PUA-like, fold that</td><td>GO:0007049|cell cycle;GO:0006281|DNA repair;GO:0010390|histone monoubiquitination;GO:0016574|histone ubiquitination;GO:0010216|maintenance of DNA methylation;GO:0000122|negative regulation of transcription from RNA polymerase II promoter;GO:0051865|protein autoubiquitination;GO:0042787|protein ubiquitination involved in ubiquitin-dependent protein catabolic process;GO:0090308|regulation of methylation-dependent chromatin silencing;GO:0006351|transcription, DNA-templated;</td><td>GO:0000791|euchromatin;GO:0000792|heterochromatin;GO:0000790|nuclear chromatin;GO:0005720|nuclear heterochromatin;GO:0016363|nuclear</td><td>GO:0044729|hemi-methylated DNA-binding;GO:0042393|histone</td><td>K10638</td><td>UHRF1, NP95; E3 ubiquitin-protein ligase UHRF1 [EC:2.3.2.27]</td></tr>
<tr><td>GJA1</td><td>10153.44</td><td>-0.52903</td><td>0.098434282</td><td>-5.37448</td><td>7.68E-08</td><td>2.67E-05</td><td>Not_Sig</td><td>no</td><td>gi|27806607|ref|NP_776493.1|</td><td>1876</td><td>0</td><td>gap junction alpha-1 protein [Bos taurus]</td><td>gi|742210206|ref|XM_010861789.1|</td><td>3059</td><td>0</td><td>PREDICTED: Bison bison bison gap junction protein, alpha 1, 43kDa (GJA1), mRNA</td><td>P18246</td><td>727</td><td>0</td><td>Gap junction alpha-1 protein</td><td>.</td><td>.</td><td>.</td><td>.</td><td>pfam00029</td><td>Connexin</td><td>Connexin.</td><td>GO:0007512|adult heart development;GO:0015867|ATP transport;GO:0003294|atrial ventricular junction remodeling;GO:0048514|blood vessel morphogenesis;GO:0061337|cardiac conduction;GO:0007154|cell communication;GO:0010643|cell communication by chemical coupling;GO:0045216|cell-cell junction organization;GO:0007267|cell-cell signaling;GO:0042733|embryonic digit morphogenesis;GO:0035050|embryonic heart tube development;GO:0002070|epithelial cell maturation;GO:0007507|heart</td><td>GO:0016324|apical plasma membrane;GO:0030054|cell junction;GO:0005922|connexon complex;GO:0043292|contractile</td><td>GO:0005243|gap junction channel activity;GO:1903763|gap junction</td><td>K07372</td><td>GJA1, CX43; gap junction protein, alpha 1</td></tr>
<tr><td>LOC102279476</td><td>577.4298</td><td>-0.96352</td><td>0.180128951</td><td>-5.34907</td><td>8.841E-08</td><td>2.9E-05</td><td>DOWN</td><td>yes</td><td>gi|725552974|ref|XP_010347552.1|</td><td>941</td><td>5e-127</td><td>PREDICTED: 60S ribosomal protein L11 isoform X1 [Saimiri boliviensis boliviensis]</td><td>gi|402744964|ref|NM_001075581.2|</td><td>604</td><td>0</td><td>Bos taurus ribosomal protein L11 (RPL11), mRNA</td><td>P62914</td><td>363</td><td>2e-127</td><td>60S ribosomal protein L11</td><td>SPBC17G9.10</td><td>264</td><td>1.64e-90</td><td>COG0094</td><td>pfam00673</td><td>Ribosomal_L5_C</td><td>ribosomal L5P family C-terminus. This region is found associated with pfam00281.</td><td>GO:0043433|negative regulation of sequence-specific DNA binding transcription factor activity;GO:0034504|protein localization to nucleus;GO:0000027|ribosomal large subunit assembly;GO:0006412|translation;</td><td>GO:0022625|cytosolic large ribosomal subunit;GO:0022626|cytosolic ribosome;GO:0005730|nucleolus;</td><td>GO:0042975|peroxisome proliferator activated receptor binding;GO:0019843</td><td>.</td><td>.</td></tr>
<tr><td>FOXM1</td><td>158.6596</td><td>1.044359</td><td>0.195245746</td><td>5.348948</td><td>8.847E-08</td><td>2.9E-05</td><td>UP</td><td>yes</td><td>gi|594091414|ref|XP_006069087.1|</td><td>2985</td><td>0</td><td>PREDICTED: forkhead box protein M1 isoform X1 [Bubalus bubalis]</td><td>gi|982982455|ref|XM_872119.8|</td><td>2400</td><td>0</td><td>PREDICTED: Bos taurus forkhead box M1 (FOXM1), transcript variant X1, mRNA</td><td>P97691</td><td>926</td><td>0</td><td>Forkhead box protein M1</td><td>YCR065w</td><td>87.4</td><td>5.59e-17</td><td>COG5025</td><td>pfam00250</td><td>Fork_head</td><td>Fork head domain.</td><td>GO:0006281|DNA repair;GO:0000086|G2/M transition of mitotic cell cycle;GO:0045944|positive regulation of transcription from RNA polymerase II promoter;GO:0051591|response to cAMP;GO:0034698|response to gonadotropin;GO:0006351|transcription, DNA-templated;</td><td>GO:0005634|nucleus;</td><td>GO:0003677|DNA binding;GO:0000981|RNA polymerase II transcription factor</td><td>.</td><td>.</td></tr>
<tr><td>SYN1</td><td>294.7159</td><td>0.957741</td><td>0.179625576</td><td>5.331876</td><td>9.72E-08</td><td>3.1E-05</td><td>UP</td><td>yes</td><td>gi|926728951|ref|XP_013831960.1|</td><td>2347</td><td>0</td><td>PREDICTED: synapsin-1 [Capra hircus]</td><td>gi|151553960|gb|BC149033.1|</td><td>2969</td><td>0</td><td>Bos taurus synapsin I, mRNA (cDNA clone MGC:152080 IMAGE:8393130), complete cds</td><td>P17599</td><td>907</td><td>0</td><td>Synapsin-1</td><td>.</td><td>.</td><td>.</td><td>.</td><td>pfam02750</td><td>Synapsin_C</td><td>Synapsin, ATP binding domain. Ca dependent ATP binding in this ATP grasp fold. Function unknown.</td><td>GO:0007269|neurotransmitter secretion;</td><td>GO:0030054|cell junction;GO:0005794|Golgi apparatus;GO:0008021|synaptic vesicle;</td><td>GO:0005524|ATP binding;GO:0003824|catalytic activity;</td><td>.</td><td>.</td></tr>
<tr><td>RXRA</td><td>558.3829</td><td>0.739823</td><td>0.139039964</td><td>5.320936</td><td>1.032E-07</td><td>3.19E-05</td><td>UP</td><td>yes</td><td>gi|803226090|ref|XP_011973350.1|</td><td>2140</td><td>0</td><td>PREDICTED: retinoic acid receptor RXR-alpha isoform X1 [Ovis aries musimon]</td><td>gi|982940181|ref|XM_005213506.3|</td><td>4530</td><td>0</td><td>PREDICTED: Bos taurus retinoid X receptor alpha (RXRA), transcript variant X1, mRNA</td><td>P28700</td><td>818</td><td>0</td><td>Retinoic acid receptor RXR-alpha</td><td>.</td><td>.</td><td>.</td><td>.</td><td>pfam00104</td><td>Hormone_recep</td><td>Ligand-binding domain of nuclear hormone receptor. This all helical domain is involved in binding the hormone in these receptors.</td><td>GO:0060978|angiogenesis involved in coronary vascular morphogenesis;GO:0043010|camera-type eye development;GO:0055007|cardiac muscle cell differentiation;GO:0060038|cardiac muscle cell proliferation;GO:0007566|embryo implantation;GO:0007507|heart development;GO:0003007|heart morphogenesis;GO:0001701|in utero embryonic development;GO:0001893|maternal placenta development;GO:0060485|mesenchyme development;GO:0019048|modulation by virus of host morphology or</td><td>GO:0000790|nuclear chromatin;GO:0005654|nucleoplasm;GO:0005634|nucleus;GO:0043235|receptor complex;GO:0090575|RNA polymerase II</td><td>GO:0004886|9-cis retinoic acid receptor activity;GO:0031490</td><td>K08524</td><td>NR2B1, RXRA; retinoid X receptor alpha</td></tr>
<tr><td>ROMO1</td><td>485.1165</td><td>-0.84016</td><td>0.158007591</td><td>-5.31719</td><td>1.054E-07</td><td>3.19E-05</td><td>DOWN</td><td>yes</td><td>gi|528758629|gb|EPY78288.1|</td><td>411</td><td>2e-47</td><td>hypothetical protein CB1_001108037 [Camelus ferus]</td><td>gi|742182392|ref|XM_010854912.1|</td><td>523</td><td>0</td><td>PREDICTED: Bison bison bison reactive oxygen species modulator 1 (ROMO1), transcript variant X2, mRNA</td><td>P60603</td><td>125</td><td>2e-35</td><td>Reactive oxygen species modulator 1</td><td>.</td><td>.</td><td>.</td><td>.</td><td>pfam10247</td><td>Romo1</td><td>Reactive mitochondrial oxygen species modulator 1. This is a family of small, approximately 100 amino acid, proteins found from</td><td>GO:0034614|cellular response to reactive oxygen species;GO:0042742|defense response to bacterium;GO:0008284|positive regulation of cell proliferation;GO:2000379|positive regulation of reactive oxygen species metabolic process;GO:0001302|replicative cell aging;</td><td>GO:0016021|integral component of membrane;GO:0005743|mitochondrial inner membrane;GO:0005739|mitochondrion;</td><td>.</td><td>.</td><td>.</td></tr>
</table>

<table>
<tr><td>NCAPH</td><td>433.7485</td><td>0.674531</td><td>0.127094946</td><td>5.3073</td><td>1.113E-07</td><td>3.28E-05</td><td>UP</td><td>yes</td><td>gi|528967580|ref|XP_005212351.1|</td><td>3595</td><td>0</td><td>PREDICTED: condensin complex subunit 2 isoform X1 [Bos taurus]</td><td>gi|742227091|ref|XM_010829588.1|</td><td>3925</td><td>0</td><td>PREDICTED: Bison bison bison non-SMC condensin I complex, subunit H (NCAPH), transcript variant X1, mRNA</td><td>Q15003</td><td>1067</td><td>0</td><td>Condensin complex subunit 2</td><td>SPCC306.03c</td><td>107</td><td>1.68e-22</td><td>COG5229</td><td>pfam05786</td><td>Cnd2</td><td>Condensin complex subunit 2. This family consists of several Barren protein homologues from several eukaryotic organisms. In</td><td>GO:0051301|cell division;GO:0007076|mitotic chromosome condensation;</td><td>GO:0000796|condensin complex;GO:0005829|cytosol;GO:0016020|membrane;GO:0005634|nucleus;</td><td>.</td><td>K06676</td><td>BRRN1, BRN1, CAPH; condensin complex subunit 2</td></tr>
<tr><td>LOC138984351</td><td>103.9952</td><td>1.163964</td><td>0.219592853</td><td>5.300554</td><td>1.155E-07</td><td>3.32E-05</td><td>UP</td><td>yes</td><td>gi|742092912|ref|XP_010832253.1|</td><td>174</td><td>4e-11</td><td>PREDICTED: uncharacterized protein LOC104984283 isoform X3 [Bison bison bison]</td><td>gi|983003376|ref|XR_001498895.1|</td><td>654</td><td>0</td><td>PREDICTED: Bos taurus uncharacterized LOC101909328 (LOC101909328), transcript variant X10, ncRNA</td><td>.</td><td>.</td><td>.</td><td>.</td><td>.</td><td>.</td><td>.</td><td>.</td><td>pfam05722</td><td>Ustilago_mating</td><td>Ustilago B locus mating-type protein. This family consists of several Ustilago mating-type proteins. The b locus of the</td><td>.</td><td>.</td><td>.</td><td>.</td><td>.</td></tr>
<tr><td>EPHA2</td><td>3099.357</td><td>0.623621</td><td>0.118230688</td><td>5.274612</td><td>1.33E-07</td><td>3.73E-05</td><td>UP</td><td>yes</td><td>gi|329664532|ref|NP_001192660.1|</td><td>5031</td><td>0</td><td>ephrin type-A receptor 2 precursor [Bos taurus]</td><td>gi|942085404|ref|XM_005906023.2|</td><td>3649</td><td>0</td><td>PREDICTED: Bos mutus EPH receptor A2 (EPHA2), mRNA</td><td>P29317</td><td>1840</td><td>0</td><td>Ephrin type-A receptor 2</td><td>YAR019c</td><td>111</td><td>6.84e-24</td><td>COG0515</td><td>pfam07714</td><td>Pkinase_Tyr</td><td>Protein tyrosine kinase.</td><td>GO:0090630|activation of GTPase activity;GO:0048320|axial mesoderm formation;GO:0002043|blood vessel endothelial cell proliferation involved in sprouting angiogenesis;GO:0046849|bone remodeling;GO:0060444|branching involved in mammary gland duct morphogenesis;GO:0060326|cell chemotaxis;GO:0016477|cell migration;GO:0050830|defense response to Gram-positive bacterium;GO:0048013|ephrin receptor signaling pathway;GO:0006954|inflammatory response;GO:0008630|intrinsic apoptotic signaling</td><td>GO:0009986|cell surface;GO:0005913|cell-cell adherens junction;GO:0005925|focal adhesion;GO:0005887|integral component of plasma</td><td>GO:0005524|ATP binding;GO:0098641|cadherin binding involved in cell-cell</td><td>K05103</td><td>EPHA2, ECK; Eph receptor A2 [EC:2.7.10.1]</td></tr>
<tr><td>SCAP</td><td>1211.452</td><td>0.717665</td><td>0.137025527</td><td>5.237455</td><td>1.628E-07</td><td>4.43E-05</td><td>UP</td><td>yes</td><td>gi|296474783|tpg|DAA16898.1|</td><td>5874</td><td>0</td><td>TPA: sterol regulatory element-binding protein cleavage-activating protein [Bos taurus]</td><td>gi|156120426|ref|NM_001101889.1|</td><td>3936</td><td>0</td><td>Bos taurus SREBF chaperone (SCAP), mRNA</td><td>A6QM06</td><td>2264</td><td>0</td><td>Sterol regulatory element-binding protein cleavage-activating protein</td><td>.</td><td>.</td><td>.</td><td>.</td><td>pfam12349</td><td>Sterol-sensing</td><td>Sterol-sensing domain of SREBP cleavage-activation. Sterol regulatory element-binding proteins (SREBPs) are membrane-</td><td>GO:0008203|cholesterol metabolic process;GO:0032933|SREBP signaling pathway;</td><td>GO:0005789|endoplasmic reticulum membrane;GO:0012507|ER to Golgi transport vesicle membrane;GO:0000139|Golgi</td><td>GO:0015485|cholesterol binding;</td><td>.</td><td>.</td></tr>
<tr><td>IQGAP3</td><td>804.9045</td><td>0.556522</td><td>0.106325284</td><td>5.234148</td><td>1.657E-07</td><td>4.43E-05</td><td>Not_Sig</td><td>no</td><td>gi|742111227|ref|XP_010836529.1|</td><td>7579</td><td>0</td><td>PREDICTED: ras GTPase-activating-like protein IQGAP3 isoform X1 [Bison bison bison]</td><td>gi|742111226|ref|XM_010838227.1|</td><td>4821</td><td>0</td><td>PREDICTED: Bison bison bison IQ motif containing GTPase activating protein 3 (IQGAP3), transcript variant X1, mRNA</td><td>Q86VI3</td><td>2650</td><td>0</td><td>Ras GTPase-activating-like protein IQGAP3</td><td>SPAC4F8.13c</td><td>254</td><td>1.42e-67</td><td>COG5261</td><td>pfam00616</td><td>RasGAP</td><td>GTPase-activator protein for Ras-like GTPase. All alpha-helical domain that accelerates the GTPase activity of Ras, thereby</td><td>GO:0000187|activation of MAPK activity;GO:0071310|cellular response to organic substance;GO:0070371|ERK1 and ERK2 cascade;GO:0000082|G1/S transition of mitotic cell cycle;GO:0010629|negative regulation of gene expression;GO:0010628|positive regulation of gene expression;GO:0033601|positive regulation of mammary gland epithelial cell proliferation;GO:0007265|Ras protein signal transduction;GO:0008361|regulation of cell size;GO:0043087|regulation of GTPase activity;</td><td>GO:0005911|cell-cell junction;GO:0005829|cytosol;GO:0016328|lateral plasma membrane;</td><td>GO:0005516|calmodulin binding;GO:0070856|myosin VI light</td><td>K05767</td><td>IQGAP2_3; Ras GTPase-activating-like protein IQGAP2/3</td></tr>
<tr><td>FOSL1</td><td>908.4479</td><td>0.888257</td><td>0.169907903</td><td>5.227876</td><td>1.715E-07</td><td>4.48E-05</td><td>UP</td><td>yes</td><td>gi|555964041|ref|XP_005894093.1|</td><td>610</td><td>6e-74</td><td>PREDICTED: LOW QUALITY PROTEIN: fos-related antigen 1 [Bos mutus]</td><td>gi|965802927|ref|XM_012117284.2|</td><td>904</td><td>0</td><td>PREDICTED: Ovis aries musimon FOS-like antigen 1 (FOSL1), transcript variant X4, mRNA</td><td>P15407</td><td>225</td><td>1e-69</td><td>Fos-related antigen 1</td><td>.</td><td>.</td><td>.</td><td>.</td><td>pfam00170</td><td>bZIP_1</td><td>bZIP transcription factor. The Pfam entry includes the basic region and the leucine zipper region.</td><td>GO:0006968|cellular defense response;GO:0031668|cellular response to extracellular stimulus;GO:0006935|chemotaxis;GO:0007565|female pregnancy;GO:0001701|in utero embryonic development;GO:0007612|learning;GO:0008285|negative regulation of cell proliferation;GO:0060674|placenta blood vessel development;GO:0043065|positive regulation of apoptotic process;GO:0045787|positive regulation of cell cycle;GO:0008284|positive regulation of cell proliferation;GO:2000144|positive regulation of DNA-templated transcription,</td><td>GO:0005829|cytosol;GO:0043005|neuron projection;GO:0005634|nucleus;GO:0042734|presynaptic membrane;</td><td>GO:0000978|RNA polymerase II core promoter proximal region sequence-</td><td>K04502</td><td>FOSL1; fos-like antigen 1</td></tr>
<tr><td>RPL36AL</td><td>1219.333</td><td>-0.81757</td><td>0.156939806</td><td>-5.20947</td><td>1.894E-07</td><td>4.84E-05</td><td>DOWN</td><td>yes</td><td>gi|744573224|ref|XP_010981272.1|</td><td>592</td><td>7e-75</td><td>PREDICTED: 60S ribosomal protein L36a-like, partial [Camelus dromedarius]</td><td>gi|742134229|ref|XM_010842234.1|</td><td>533</td><td>0</td><td>PREDICTED: Bison bison bison ribosomal protein L36a-like (RPL36AL), mRNA</td><td>P83883</td><td>214</td><td>7e-70</td><td>60S ribosomal protein L36a</td><td>YNL162w</td><td>139</td><td>6.01e-42</td><td>COG1631</td><td>pfam00935</td><td>Ribosomal_L44</td><td>Ribosomal protein L44.</td><td>GO:0032526|response to retinoic acid;GO:0006412|translation;</td><td>GO:0022625|cytosolic large ribosomal subunit;</td><td>GO:0003735|structural constituent of ribosome;</td><td>K02929</td><td>RP-L44e, RPL44; large subunit ribosomal protein L44e</td></tr>
<tr><td>PPP1R2</td><td>924.5734</td><td>-0.67598</td><td>0.129981924</td><td>-5.20055</td><td>1.987E-07</td><td>4.96E-05</td><td>DOWN</td><td>yes</td><td>gi|555971781|ref|XP_005897899.1|</td><td>972</td><td>4e-120</td><td>PREDICTED: protein phosphatase inhibitor 2 isoform X2 [Bos mutus]</td><td>gi|982910651|ref|XM_015471887.1|</td><td>3276</td><td>0</td><td>PREDICTED: Bos taurus protein phosphatase 1 regulatory inhibitor subunit 2 (PPP1R2), transcript variant X1, mRNA</td><td>Q3SZX2</td><td>377</td><td>7e-121</td><td>Protein phosphatase inhibitor 2</td><td>.</td><td>.</td><td>.</td><td>.</td><td>pfam04979</td><td>IPP-2</td><td>Protein phosphatase inhibitor 2 (IPP-2). Protein phosphotase inhibitor 2 (IPP-2) is a phosphoprotein conserved among</td><td>GO:0005977|glycogen metabolic process;GO:0043666|regulation of phosphoprotein phosphatase activity;GO:0009966|regulation of signal transduction;</td><td>GO:0000164|protein phosphatase type 1 complex;</td><td>GO:0004864|protein phosphatase inhibitor activity;</td><td>K16833</td><td>PPP1R2, IPP2; protein phosphatase inhibitor 2</td></tr>
<tr><td>C24H18orf32</td><td>202.7238</td><td>-0.96111</td><td>0.185177885</td><td>-5.19017</td><td>2.101E-07</td><td>5.14E-05</td><td>DOWN</td><td>yes</td><td>gi|593764484|ref|XP_007120821.1|</td><td>399</td><td>2e-44</td><td>PREDICTED: UPF0729 protein C18orf32 homolog [Physeter catodon]</td><td>gi|742164099|ref|XM_010849824.1|</td><td>1045</td><td>0</td><td>PREDICTED: Bison bison bison chromosome unknown open reading frame, human C18orf32 (LOC104995807), transcript variant X2, mRNA</td><td>Q3ZC78</td><td>155</td><td>4e-45</td><td>UPF0729 protein C18orf32 homolog</td><td>.</td><td>.</td><td>.</td><td>.</td><td>pfam14975</td><td>DUF4512</td><td>Domain of unknown function (DUF4512). This family of proteins is found in eukaryotes. Proteins in this family are typically between</td><td>GO:0043123|positive regulation of I-kappaB kinase/NF-kappaB signaling;</td><td>.</td><td>GO:0004871|signal transducer activity;</td><td>.</td><td>.</td></tr>
<tr><td>UQCR10</td><td>505.3908</td><td>-0.73813</td><td>0.142777809</td><td>-5.16977</td><td>2.344E-07</td><td>5.61E-05</td><td>DOWN</td><td>yes</td><td>gi|6841524|gb|AAF29115.1|AF161500_1</td><td>324</td><td>3e-35</td><td>HSPC151 [Homo sapiens]</td><td>gi|982950822|ref|XM_005218100.3|</td><td>541</td><td>0</td><td>PREDICTED: Bos taurus ubiquinol-cytochrome c reductase, complex III subunit X (UQCR10), transcript variant X1, mRNA</td><td>P00130</td><td>120</td><td>2e-33</td><td>Cytochrome b-c1 complex subunit 9</td><td>.</td><td>.</td><td>.</td><td>.</td><td>pfam05365</td><td>UCR_UQCRX_QCR9</td><td>Ubiquinol-cytochrome C reductase, UQCRX/QCR9 like. The UQCRX/QCR9 protein is the 9/10 subunit of complex III, encoding a</td><td>GO:0009060|aerobic respiration;GO:1902600|hydrogen ion transmembrane transport;GO:0006122|mitochondrial electron transport, ubiquinol to cytochrome c;GO:0034551|mitochondrial respiratory chain complex III assembly;</td><td>GO:0070062|extracellular exosome;GO:0005750|mitochondrial respiratory chain complex III;</td><td>.</td><td>K00419</td><td>QCR9, UCRC; ubiquinol-cytochrome c reductase subunit 9</td></tr>
<tr><td>NID2</td><td>27506.24</td><td>0.519373</td><td>0.100644171</td><td>5.160489</td><td>2.463E-07</td><td>5.78E-05</td><td>Not_Sig</td><td>no</td><td>gi|742134398|ref|XP_010840584.1|</td><td>6525</td><td>0</td><td>PREDICTED: nidogen-2 [Bison bison bison]</td><td>gi|942051305|ref|XM_005894543.2|</td><td>4604</td><td>0</td><td>PREDICTED: Bos mutus nidogen 2 (osteonidogen) (NID2), mRNA</td><td>Q14112</td><td>1889</td><td>0</td><td>Nidogen-2</td><td>.</td><td>.</td><td>.</td><td>.</td><td>pfam07474</td><td>G2F</td><td>G2F domain. Nidogen, an invariant component of basement membranes, is a multifunctional protein that interacts with most</td><td>GO:0071711|basement membrane organization;GO:0007155|cell adhesion;GO:0007160|cell-matrix adhesion;GO:0030198|extracellular matrix organization;</td><td>GO:0005604|basement membrane;GO:0070062|extracellular exosome;GO:0031012|extracellular matrix;GO:0005576|extracellular region;</td><td>GO:0005509|calcium ion binding;GO:0005518|collagen binding;</td><td>K06826</td><td>NID; nidogen (entactin)</td></tr>
<tr><td>CLUH</td><td>1284.652</td><td>0.590193</td><td>0.114573826</td><td>5.151202</td><td>2.588E-07</td><td>5.95E-05</td><td>UP</td><td>yes</td><td>gi|742208209|ref|XP_010859611.1|</td><td>7611</td><td>0</td><td>PREDICTED: clustered mitochondria protein homolog [Bison bison bison]</td><td>gi|742208208|ref|XM_010861309.1|</td><td>5730</td><td>0</td><td>PREDICTED: Bison bison bison clustered mitochondria (cluA/CLU1) homolog (CLUH), mRNA</td><td>O75153</td><td>2479</td><td>0</td><td>Clustered mitochondria protein homolog</td><td>.</td><td>.</td><td>.</td><td>.</td><td>pfam13236</td><td>CLU</td><td>Clustered mitochondria. The CLU domain (CLUstered mitochondria) is a eukaryotic domain found in proteins from fungi, protozoa,</td><td>GO:0048312|intracellular distribution of mitochondria;GO:0007005|mitochondrion organization;</td><td>GO:0005737|cytoplasm;</td><td>GO:0003729|mRNA binding;</td><td>K03255</td><td>TIF31, CLU1; protein TIF31</td></tr>
<tr><td>MASP1</td><td>84.7666</td><td>1.113889</td><td>0.216550498</td><td>5.143782</td><td>2.693E-07</td><td>6.07E-05</td><td>UP</td><td>yes</td><td>gi|555975455|ref|XP_005899709.1|</td><td>3714</td><td>0</td><td>PREDICTED: mannan-binding lectin serine protease 1 isoform X1 [Bos mutus]</td><td>gi|742178707|ref|XM_010853913.1|</td><td>3939</td><td>0</td><td>PREDICTED: Bison bison bison mannan-binding lectin serine peptidase 1 (C4/C2 activating component of Ra-reactive factor) (MASP1), transcript variant X1, mRNA</td><td>P48740</td><td>947</td><td>0</td><td>Mannan-binding lectin serine protease 1</td><td>mlr3307</td><td>100</td><td>7.87e-22</td><td>COG5640</td><td>pfam00089</td><td>Trypsin</td><td>Trypsin.</td><td>GO:0006956|complement activation;GO:0001867|complement activation, lectin pathway;GO:0045916|negative regulation of complement activation;GO:0006898|receptor-mediated endocytosis;</td><td>GO:0005576|extracellular region;GO:0005615|extracellular space;</td><td>GO:0005509|calcium ion binding;GO:0048306|calcium-dependent</td><td>K03992</td><td>MASP1; mannan-binding lectin serine protease 1 [EC:3.4.21.-]</td></tr>
<tr><td>KLF2</td><td>408.3083</td><td>0.832857</td><td>0.162280563</td><td>5.132203</td><td>2.864E-07</td><td>6.33E-05</td><td>UP</td><td>yes</td><td>gi|602698038|ref|XP_007461050.1|</td><td>991</td><td>7e-122</td><td>PREDICTED: Krueppel-like factor 2 [Lipotes vexillifer]</td><td>gi|965826665|ref|XM_012128901.2|</td><td>2588</td><td>0</td><td>PREDICTED: Ovis aries musimon Kruppel-like factor 2 (KLF2), transcript variant X2, mRNA</td><td>Q9Y5W3</td><td>230</td><td>8e-66</td><td>Krueppel-like factor 2</td><td>.</td><td>.</td><td>.</td><td>.</td><td>pfam13465</td><td>zf-H2C2_2</td><td>Zinc-finger double domain.</td><td>GO:0000902|cell morphogenesis;GO:0071409|cellular response to cycloheximide;GO:0070301|cellular response to hydrogen peroxide;GO:0071347|cellular response to interleukin-1;GO:0071499|cellular response to laminar fluid shear stress;GO:1901653|cellular response to peptide;GO:0071356|cellular response to tumor necrosis factor;GO:0043249|erythrocyte maturation;GO:0001701|in utero embryonic development;GO:0035264|multicellular organism growth;GO:0032715|negative regulation of interleukin-6</td><td>GO:0005634|nucleus;</td><td>GO:0046872|metal ion binding;GO:0043565|sequence-specific</td><td>K17845</td><td>KLF2; krueppel-like factor 2</td></tr>
<tr><td>CYP7B1</td><td>621.1957</td><td>-0.69882</td><td>0.137161593</td><td>-5.09488</td><td>3.49E-07</td><td>7.57E-05</td><td>DOWN</td><td>yes</td><td>gi|555961840|ref|XP_005893014.1|</td><td>2493</td><td>0</td><td>PREDICTED: 25-hydroxycholesterol 7-alpha-hydroxylase [Bos mutus]</td><td>gi|741889594|ref|XM_608003.7|</td><td>6846</td><td>0</td><td>PREDICTED: Bos taurus cytochrome P450, family 7, subfamily B, polypeptide 1 (CYP7B1), mRNA</td><td>O75881</td><td>669</td><td>0</td><td>25-hydroxycholesterol 7-alpha-hydroxylase</td><td>PA3679</td><td>67.8</td><td>1.94e-10</td><td>COG2124</td><td>pfam00067</td><td>p450</td><td>Cytochrome P450. Cytochrome P450s are haem-thiolate proteins involved in the oxidative degradation of various</td><td>GO:0035754|B cell chemotaxis;GO:0006699|bile acid biosynthetic process;GO:0008203|cholesterol metabolic process;GO:0033147|negative regulation of intracellular estrogen receptor signaling pathway;GO:0050679|positive regulation of epithelial cell proliferation;GO:0060740|prostate gland epithelium morphogenesis;GO:0016125|sterol metabolic process;</td><td>GO:0005789|endoplasmic reticulum membrane;GO:0031090|organelle membrane;</td><td>GO:0033783|25-hydroxycholesterol 7alpha-hydroxylase activity;GO:0020037</td><td>K07430</td><td>CYP7B; oxysterol 7-alpha-hydroxylase [EC:1.14.13.100]</td></tr>
<tr><td>LOC102269246</td><td>645.4687</td><td>0.814503</td><td>0.160375569</td><td>5.078725</td><td>3.8E-07</td><td>8.09E-05</td><td>UP</td><td>yes</td><td>gi|555958383|ref|XP_005891312.1|</td><td>357</td><td>7e-38</td><td>PREDICTED: growth-regulated protein homolog gamma [Bos mutus]</td><td>gi|942042149|ref|XM_005891250.2|</td><td>1101</td><td>0</td><td>PREDICTED: Bos mutus growth-regulated protein homolog gamma (LOC102269246), mRNA</td><td>O46676</td><td>136</td><td>3e-37</td><td>Growth-regulated protein homolog alpha</td><td>.</td><td>.</td><td>.</td><td>.</td><td>pfam00048</td><td>IL8</td><td>Small cytokines (intecrine/chemokine), interleukin-8 like. Includes a number of secreted growth factors and</td><td>GO:0060326|cell chemotaxis;GO:0006955|immune response;GO:0006954|inflammatory response;</td><td>GO:0005615|extracellular space;</td><td>.</td><td>K05505</td><td>CXCL1_2_3, GRO; C-X-C motif chemokine 1/2/3</td></tr>
<tr><td>MGP</td><td>168.2308</td><td>-0.94328</td><td>0.185976697</td><td>-5.07202</td><td>3.936E-07</td><td>8.22E-05</td><td>DOWN</td><td>yes</td><td>gi|440899332|gb|ELR50647.1|</td><td>626</td><td>7e-80</td><td>hypothetical protein M91_04351, partial [Bos mutus]</td><td>gi|942078179|ref|XM_005903620.2|</td><td>626</td><td>0</td><td>PREDICTED: Bos mutus matrix Gla protein (MGP), transcript variant X2, mRNA</td><td>P07507</td><td>179</td><td>6e-56</td><td>Matrix Gla protein</td><td>.</td><td>.</td><td>.</td><td>.</td><td>pfam00594</td><td>Gla</td><td>Vitamin K-dependent carboxylation/gamma-carboxyglutamic (GLA) domain. This domain is responsible for the</td><td>GO:0051216|cartilage development;GO:0030154|cell differentiation;GO:0001503|ossification;GO:0030500|regulation of bone mineralization;</td><td>GO:0031012|extracellular matrix;</td><td>GO:0005509|calcium ion binding;GO:0005201|extracellular matrix</td><td>K19481</td><td>MGP; matrix Gla protein</td></tr>
<tr><td>TGM2</td><td>2771.291</td><td>0.568653</td><td>0.112639608</td><td>5.048425</td><td>4.455E-07</td><td>9.14E-05</td><td>Not_Sig</td><td>no</td><td>gi|29135313|ref|NP_803473.1|</td><td>3603</td><td>0</td><td>protein-glutamine gamma-glutamyltransferase 2 [Bos taurus]</td><td>gi|555968024|ref|XM_005895992.1|</td><td>3883</td><td>0</td><td>PREDICTED: Bos mutus transglutaminase 2 (TGM2), transcript variant X1, mRNA</td><td>P51176</td><td>1392</td><td>0</td><td>Protein-glutamine gamma-glutamyltransferase 2</td><td>.</td><td>.</td><td>.</td><td>.</td><td>pfam00868</td><td>Transglut_N</td><td>Transglutaminase family.</td><td>GO:0018149|peptide cross-linking;</td><td>.</td><td>GO:0046872|metal ion binding;GO:0003810|protein-glutamine</td><td>K05625</td><td>TGM2; transglutaminase 2 [EC:2.3.2.13]</td></tr>
<tr><td>TFPI2</td><td>5075.891</td><td>-0.53427</td><td>0.105952818</td><td>-5.04253</td><td>4.594E-07</td><td>9.26E-05</td><td>Not_Sig</td><td>no</td><td>gi|742117538|ref|XP_010838075.1|</td><td>1392</td><td>0</td><td>PREDICTED: tissue factor pathway inhibitor 2 [Bison bison bison]</td><td>gi|594111095|ref|XM_006078317.1|</td><td>1679</td><td>0</td><td>PREDICTED: Bubalus bubalis tissue factor pathway inhibitor 2 (TFPI2), mRNA</td><td>Q7YRQ8</td><td>428</td><td>6e-142</td><td>Tissue factor pathway inhibitor 2</td><td>.</td><td>.</td><td>.</td><td>.</td><td>pfam00014</td><td>Kunitz_BPTI</td><td>Kunitz/Bovine pancreatic trypsin inhibitor domain. Indicative of a protease inhibitor, usually a serine protease inhibitor. Structure is a</td><td>GO:0007596|blood coagulation;</td><td>GO:0005576|extracellular region;GO:0005634|nucleus;</td><td>GO:0004867|serine-type endopeptidase inhibitor activity;</td><td>.</td><td>.</td></tr>
<tr><td>WIZ</td><td>729.3097</td><td>0.769732</td><td>0.152827536</td><td>5.036607</td><td>4.739E-07</td><td>9.3E-05</td><td>UP</td><td>yes</td><td>gi|741931577|ref|XP_010804931.1|</td><td>7823</td><td>0</td><td>PREDICTED: protein Wiz isoform X1 [Bos taurus]</td><td>gi|965907094|ref|XM_012166757.2|</td><td>6359</td><td>0</td><td>PREDICTED: Ovis aries musimon widely interspaced zinc finger motifs (WIZ), transcript variant X2, mRNA</td><td>O95785</td><td>1187</td><td>0</td><td>Protein Wiz</td><td>.</td><td>.</td><td>.</td><td>.</td><td>pfam15449</td><td>Retinal</td><td>Retinal protein. This family of proteins is found in the photoreceptor cells of the retina. Mutations of the gene encoding</td><td>GO:0010571|positive regulation of nuclear cell cycle DNA replication;GO:0070208|protein heterotrimerization;GO:0050821|protein stabilization;</td><td>GO:0070062|extracellular exosome;GO:0005634|nucleus;</td><td>GO:0046872|metal ion binding;</td><td>.</td><td>.</td></tr>
<tr><td>DHCR7</td><td>2076.211</td><td>0.585776</td><td>0.116411143</td><td>5.031959</td><td>4.855E-07</td><td>9.3E-05</td><td>UP</td><td>yes</td><td>gi|555986533|ref|XP_005905126.1|</td><td>2441</td><td>0</td><td>PREDICTED: 7-dehydrocholesterol reductase [Bos mutus]</td><td>gi|742108869|ref|XM_010837644.1|</td><td>2579</td><td>0</td><td>PREDICTED: Bison bison bison 7-dehydrocholesterol reductase (DHCR7), transcript variant X1, mRNA</td><td>Q5E9J5</td><td>938</td><td>0</td><td>7-dehydrocholesterol reductase</td><td>.</td><td>.</td><td>.</td><td>.</td><td>pfam01222</td><td>ERG4_ERG24</td><td>Ergosterol biosynthesis ERG4/ERG24 family.</td><td>GO:0006695|cholesterol biosynthetic process;</td><td>GO:0030176|integral component of endoplasmic reticulum membrane;</td><td>GO:0047598|7-dehydrocholesterol reductase activity;GO:0050661</td><td>K00213</td><td>DHCR7; 7-dehydrocholesterol reductase [EC:1.3.1.21]</td></tr>
<tr><td>RARRES2</td><td>601.7686</td><td>-0.65375</td><td>0.129953196</td><td>-5.03063</td><td>4.889E-07</td><td>9.3E-05</td><td>DOWN</td><td>yes</td><td>gi|114051129|ref|NP_001039485.1|</td><td>772</td><td>4e-100</td><td>retinoic acid receptor responder protein 2 precursor [Bos taurus]</td><td>gi|402692146|ref|NM_001046020.2|</td><td>877</td><td>0</td><td>Bos taurus retinoic acid receptor responder (tazarotene induced) 2 (RARRES2), mRNA</td><td>Q29RS5</td><td>301</td><td>1e-101</td><td>Retinoic acid receptor responder protein 2</td><td>.</td><td>.</td><td>.</td><td>.</td><td>pfam02029</td><td>Caldesmon</td><td>Caldesmon.</td><td>GO:0050873|brown fat cell differentiation;GO:0006935|chemotaxis;GO:0048566|embryonic digestive tract development;GO:0001701|in utero embryonic development;GO:0006954|inflammatory response;GO:0050921|positive regulation of chemotaxis;GO:0045600|positive regulation of fat cell differentiation;GO:2001275|positive regulation of glucose import in response to insulin stimulus;GO:0010759|positive regulation of macrophage chemotaxis;GO:0001934|positive regulation of protein phosphorylation;GO:0050994|regulation of lipid catabolic</td><td>GO:0070062|extracellular exosome;GO:0031012|extracellular matrix;GO:0005576|extracellular region;</td><td>.</td><td>K10044</td><td>RARRES2; retinoic acid receptor responder protein 2</td></tr>
<tr><td>PHF19</td><td>506.6938</td><td>0.674276</td><td>0.134118536</td><td>5.02746</td><td>4.97E-07</td><td>9.3E-05</td><td>UP</td><td>yes</td><td>gi|296484331|tpg|DAA26446.1|</td><td>2923</td><td>0</td><td>TPA: PHD finger protein 19 [Bos taurus]</td><td>gi|594060175|ref|XM_006054138.1|</td><td>2902</td><td>0</td><td>PREDICTED: Bubalus bubalis PHD finger protein 19 (PHF19), transcript variant X2, mRNA</td><td>Q5T6S3</td><td>1082</td><td>0</td><td>PHD finger protein 19</td><td>.</td><td>.</td><td>.</td><td>.</td><td>pfam14061</td><td>Mtf2_C</td><td>Polycomb-like MTF2 factor 2. Mammalian Polycomb-like gene MTF2/PCL2 forms a complex with Polycomb repressive complex-2</td><td>GO:0016569|covalent chromatin modification;GO:0045814|negative regulation of gene expression, epigenetic;GO:0061087|positive regulation of histone H3-K27 methylation;GO:0006355|regulation of transcription, DNA-templated;GO:0006351|transcription, DNA-templated;</td><td>GO:0005654|nucleoplasm;</td><td>GO:0035064|methylated histone binding;GO:0008270|zinc ion binding;</td><td>K11486</td><td>PHF19, PCL3; polycomb-like protein 3</td></tr>
<tr><td>NCAPD3</td><td>462.0622</td><td>0.645368</td><td>0.128413302</td><td>5.025707</td><td>5.016E-07</td><td>9.3E-05</td><td>UP</td><td>yes</td><td>gi|528978954|ref|XP_005216655.1|</td><td>7093</td><td>0</td><td>PREDICTED: condensin-2 complex subunit D3 isoform X1 [Bos taurus]</td><td>gi|982947262|ref|XM_005216598.3|</td><td>5227</td><td>0</td><td>PREDICTED: Bos taurus non-SMC condensin II complex subunit D3 (NCAPD3), transcript variant X1, mRNA</td><td>P42695</td><td>1770</td><td>0</td><td>Condensin-2 complex subunit D3</td><td>.</td><td>.</td><td>.</td><td>.</td><td>pfam12717</td><td>Cnd1</td><td>non-SMC mitotic condensation complex subunit 1. The three non-SMC (structural maintenance of chromosomes) subunits of the</td><td>GO:0051301|cell division;GO:0051304|chromosome separation;GO:0010032|meiotic chromosome condensation;GO:0007076|mitotic chromosome condensation;</td><td>GO:0000779|condensed chromosome, centromeric region;GO:0016020|membrane;GO:0000799|nuclear condensin</td><td>GO:0003682|chromatin binding;GO:0035064|methylated histone</td><td>K11491</td><td>NCAPD3; condensin-2 complex subunit D3</td></tr>
<tr><td>TNS2</td><td>580.0022</td><td>0.867248</td><td>0.172801671</td><td>5.018747</td><td>5.201E-07</td><td>9.49E-05</td><td>UP</td><td>yes</td><td>gi|528904291|ref|XP_005198294.1|</td><td>6619</td><td>0</td><td>PREDICTED: tensin-2 isoform X4 [Bos taurus]</td><td>gi|742194110|ref|XM_010857894.1|</td><td>4918</td><td>0</td><td>PREDICTED: Bison bison bison tensin like C1 domain containing phosphatase (tensin 2) (TENC1), mRNA</td><td>Q63HR2</td><td>2389</td><td>0</td><td>Tensin-2</td><td>SPBC609.02</td><td>69.3</td><td>2.37e-11</td><td>COG2453</td><td>pfam08416</td><td>PTB</td><td>Phosphotyrosine-binding domain. The phosphotyrosine-binding domain (PTB, also phosphotyrosine-interaction or PI</td><td>GO:0019725|cellular homeostasis;GO:0032963|collagen metabolic process;GO:0035556|intracellular signal transduction;GO:0001822|kidney development;GO:0035264|multicellular organism growth;GO:0048871|multicellular organismal homeostasis;GO:0008285|negative regulation of cell proliferation;GO:0014850|response to muscle activity;</td><td>GO:0005925|focal adhesion;GO:0005622|intracellular;GO:0005886|plasma membrane;</td><td>GO:0019900|kinase binding;GO:0046872|metal ion binding;GO:0004721</td><td>K18080</td><td>TNS; tensin</td></tr>
<tr><td>ZMAT3</td><td>1471.629</td><td>-0.69415</td><td>0.139214339</td><td>-4.98618</td><td>6.158E-07</td><td>0.000111</td><td>DOWN</td><td>yes</td><td>gi|594060532|ref|XP_006054368.1|</td><td>1375</td><td>1e-169</td><td>PREDICTED: zinc finger matrin-type protein 3 isoform X1 [Bubalus bubalis]</td><td>gi|982911143|ref|XM_005201661.3|</td><td>8253</td><td>0</td><td>PREDICTED: Bos taurus zinc finger, matrin-type 3 (ZMAT3), transcript variant X2, mRNA</td><td>Q0IIC4</td><td>533</td><td>9e-171</td><td>Zinc finger matrin-type protein 3</td><td>.</td><td>.</td><td>.</td><td>.</td><td>pfam12874</td><td>zf-met</td><td>Zinc-finger of C2H2 type. This is a zinc-finger domain with the CxxCx(12)Hx(6)H motif, found in multiple copies in a wide range of</td><td>GO:0006974|cellular response to DNA damage stimulus;GO:0072332|intrinsic apoptotic signaling pathway by p53 class mediator;GO:0015031|protein transport;GO:0040008|regulation of growth;</td><td>GO:0005730|nucleolus;</td><td>GO:0002039|p53 binding;GO:0044822|poly(A) RNA binding;GO:0003723</td><td>K10137</td><td>ZMAT3; zinc finger, matrin type 3</td></tr>
<tr><td>NDUFC1</td><td>272.5803</td><td>-1.01298</td><td>0.203303939</td><td>-4.98259</td><td>6.274E-07</td><td>0.000111</td><td>DOWN</td><td>yes</td><td>gi|594113145|ref|XP_006079329.1|</td><td>524</td><td>3e-65</td><td>PREDICTED: NADH dehydrogenase [ubiquinone] 1 subunit C1, mitochondrial [Bubalus bubalis]</td><td>gi|741955853|ref|XM_005217536.2|</td><td>542</td><td>0</td><td>PREDICTED: Bos taurus NADH:ubiquinone oxidoreductase subunit C1 (NDUFC1), transcript variant X1, mRNA</td><td>Q02376</td><td>157</td><td>3e-48</td><td>NADH dehydrogenase [ubiquinone] 1 subunit C1, mitochondrial</td><td>.</td><td>.</td><td>.</td><td>.</td><td>pfam15088</td><td>NADH_dh_m_C1</td><td>NADH dehydrogenase [ubiquinone] 1 subunit C1, mitochondrial.</td><td>GO:0055114|oxidation-reduction process;</td><td>GO:0016021|integral component of membrane;GO:0005747|mitochondrial respiratory chain complex I;</td><td>.</td><td>K03967</td><td>NDUFC1; NADH dehydrogenase (ubiquinone) 1 subunit C1</td></tr>
<tr><td>PLK1</td><td>524.3783</td><td>0.708331</td><td>0.142406429</td><td>4.974007</td><td>6.558E-07</td><td>0.000114</td><td>UP</td><td>yes</td><td>gi|363548454|sp|Q2TA25.2|PLK1_BOVIN</td><td>2770</td><td>0</td><td>RecName: Full=Serine/threonine-protein kinase PLK1; AltName: Full=Polo-like kinase 1; Short=PLK-1</td><td>gi|594095901|ref|XM_006071163.1|</td><td>2667</td><td>0</td><td>PREDICTED: Bubalus bubalis polo-like kinase 1 (PLK1), mRNA</td><td>Q2TA25</td><td>1071</td><td>0</td><td>Serine/threonine-protein kinase PLK1</td><td>.</td><td>.</td><td>.</td><td>.</td><td>pfam00069</td><td>Pkinase</td><td>Protein kinase domain.</td><td>GO:0051297|centrosome organization;GO:0045184|establishment of protein localization;GO:0016321|female meiosis chromosome segregation;GO:0031572|G2 DNA damage checkpoint;GO:0000086|G2/M transition of mitotic cell cycle;GO:0045143|homologous chromosome segregation;GO:0001578|microtubule bundle formation;GO:0000281|mitotic cytokinesis;GO:0007067|mitotic nuclear division;GO:0000070|mitotic sister chromatid segregation;GO:0007094|mitotic spindle assembly checkpoint;GO:0043066|negative regulation</td><td>GO:0005813|centrosome;GO:0000785|chromatin;GO:0000942|condensed nuclear chromosome outer kinetochore;GO:0005737|cytoplasm;GO:00</td><td>GO:0005524|ATP binding;GO:0008017|microtubule binding;GO:0004674</td><td>K06631</td><td>PLK1; polo-like kinase 1 [EC:2.7.11.21]</td></tr>
<tr><td>SCARB1</td><td>879.6903</td><td>0.608223</td><td>0.122536628</td><td>4.963605</td><td>6.92E-07</td><td>0.000119</td><td>UP</td><td>yes</td><td>gi|594051452|ref|XP_006050059.1|</td><td>2711</td><td>0</td><td>PREDICTED: scavenger receptor class B member 1 isoform X1 [Bubalus bubalis]</td><td>gi|982950173|ref|XM_005217738.3|</td><td>2525</td><td>0</td><td>PREDICTED: Bos taurus scavenger receptor class B member 1 (SCARB1), transcript variant X1, mRNA</td><td>O18824</td><td>1048</td><td>0</td><td>Scavenger receptor class B member 1</td><td>.</td><td>.</td><td>.</td><td>.</td><td>pfam01130</td><td>CD36</td><td>CD36 family. The CD36 family is thought to be a novel class of scavenger receptors. There is also evidence suggesting a possible role</td><td>GO:0044406|adhesion of symbiont to host;GO:0043534|blood vessel endothelial cell migration;GO:0006707|cholesterol catabolic process;GO:0033344|cholesterol efflux;GO:0042632|cholesterol homeostasis;GO:0070508|cholesterol import;GO:0032497|detection of lipopolysaccharide;GO:0001935|endothelial cell proliferation;GO:0034384|high-density lipoprotein particle clearance;GO:0034375|high-density lipoprotein particle remodeling;GO:0050892|intestinal absorption;GO:0015920|lipopolysaccharide transport;GO:0034383|low-density</td><td>GO:0005901|caveola;GO:0009986|cell surface;GO:0070062|extracellular exosome;GO:0005887|integral component of plasma</td><td>GO:0008035|high-density lipoprotein particle binding;GO:0070506</td><td>K13885</td><td>SCARB1; scavenger receptor class B, member 1</td></tr>
<tr><td>DNMT1</td><td>1234.87</td><td>0.568377</td><td>0.114584291</td><td>4.960339</td><td>7.037E-07</td><td>0.000119</td><td>Not_Sig</td><td>no</td><td>gi|317108162|ref|NP_872592.2|</td><td>7673</td><td>0</td><td>DNA (cytosine-5)-methyltransferase 1 [Bos taurus]</td><td>gi|742218771|ref|XM_010828384.1|</td><td>5185</td><td>0</td><td>PREDICTED: Bison bison bison DNA (cytosine-5-)-methyltransferase 1 (DNMT1), mRNA</td><td>Q24K09</td><td>2930</td><td>0</td><td>DNA (cytosine-5)-methyltransferase 1</td><td>alr4815</td><td>103</td><td>2.22e-22</td><td>COG0270</td><td>pfam00145</td><td>DNA_methylase</td><td>C-5 cytosine-specific DNA methylase.</td><td>GO:0071230|cellular response to amino acid stimulus;GO:0016569|covalent chromatin modification;GO:0016458|gene silencing;GO:0010216|maintenance of DNA methylation;GO:0051573|negative regulation of histone H3-K9 methylation;GO:0000122|negative regulation of transcription from RNA polymerase II promoter;GO:0010628|positive regulation of gene expression;GO:0051571|positive regulation of histone H3-K4 methylation;GO:0090309|positive regulation of methylation-dependent chromatin silencing;GO:0007265|Ras</td><td>GO:0005634|nucleus;GO:0005721|pericentric heterochromatin;GO:0005657|replication fork;</td><td>GO:0003886|DNA (cytosine-5-)-methyltransferase activity;GO:0008327</td><td>K00558</td><td>DNMT1, dcm; DNA (cytosine-5)-methyltransferase 1 [EC:2.1.1.37]</td></tr>
<tr><td>PIGT</td><td>1150.857</td><td>0.696018</td><td>0.140419603</td><td>4.956702</td><td>7.17E-07</td><td>0.000119</td><td>UP</td><td>yes</td><td>gi|440907784|gb|ELR57882.1|</td><td>2831</td><td>0</td><td>GPI transamidase component PIG-T, partial [Bos mutus]</td><td>gi|982942902|ref|XM_005214532.3|</td><td>2164</td><td>0</td><td>PREDICTED: Bos taurus phosphatidylinositol glycan anchor biosynthesis class T (PIGT), transcript variant X1, mRNA</td><td>Q969N2</td><td>1021</td><td>0</td><td>GPI transamidase component PIG-T</td><td>.</td><td>.</td><td>.</td><td>.</td><td>pfam04113</td><td>Gpi16</td><td>Gpi16 subunit, GPI transamidase component. GPI (glycosyl phosphatidyl inositol) transamidase is a multi-protein</td><td>GO:0016255|attachment of GPI anchor to protein;GO:0051402|neuron apoptotic process;GO:0030182|neuron differentiation;</td><td>GO:0016023|cytoplasmic membrane-bounded vesicle;GO:0005789|endoplasmic reticulum membrane;GO:0042765|GPI-anchor transamidase</td><td>GO:0003923|GPI-anchor transamidase activity;</td><td>K05292</td><td>PIGT; phosphatidylinositol glycan, class T</td></tr>
<tr><td>MYBBP1A</td><td>1713.553</td><td>0.497538</td><td>0.100470422</td><td>4.952088</td><td>7.342E-07</td><td>0.00012</td><td>Not_Sig</td><td>no</td><td>gi|440896467|gb|ELR48384.1|</td><td>6296</td><td>0</td><td>Myb-binding protein 1A [Bos mutus]</td><td>gi|942087645|ref|XM_014481789.1|</td><td>4675</td><td>0</td><td>PREDICTED: Bos mutus MYB binding protein (P160) 1a (MYBBP1A), transcript variant X2, mRNA</td><td>Q9BQG0</td><td>1769</td><td>0</td><td>Myb-binding protein 1A</td><td>.</td><td>.</td><td>.</td><td>.</td><td>pfam04931</td><td>DNA_pol_phi</td><td>DNA polymerase phi. This family includes the fifth essential DNA polymerase in yeast EC:2.7.7.7. Pol5p is localized exclusively to the</td><td>GO:0042149|cellular response to glucose starvation;GO:0032922|circadian regulation of gene expression;GO:0072332|intrinsic apoptotic signaling pathway by p53 class mediator;GO:0045892|negative regulation of transcription, DNA-templated;GO:0006913|nucleocytoplasmic transport;GO:0001649|osteoblast differentiation;GO:2000210|positive regulation of anoikis;GO:0071158|positive regulation of cell cycle arrest;GO:0045815|positive regulation of gene expression, epigenetic;GO:0006355|regulation of transcription, DNA-</td><td>GO:0005737|cytoplasm;GO:0043231|intracellular membrane-bounded organelle;GO:0016020|membrane;GO:0042564|NLS-dependent protein nuclear import</td><td>GO:0001047|core promoter binding;GO:0003887|DNA-directed DNA</td><td>K02331</td><td>POL5, MYBBP1A; DNA polymerase phi [EC:2.7.7.7]</td></tr>
<tr><td>BICRA</td><td>380.0821</td><td>1.005144</td><td>0.203097766</td><td>4.949063</td><td>7.457E-07</td><td>0.00012</td><td>UP</td><td>yes</td><td>gi|594088085|ref|XP_006067500.1|</td><td>2852</td><td>0</td><td>PREDICTED: LOW QUALITY PROTEIN: glioma tumor suppressor candidate region gene 1 protein [Bubalus bubalis]</td><td>gi|594088084|ref|XM_006067438.1|</td><td>5130</td><td>0</td><td>PREDICTED: Bubalus bubalis glioma tumor suppressor candidate region gene 1 (GLTSCR1), mRNA</td><td>Q9NZM4</td><td>1052</td><td>0</td><td>Glioma tumor suppressor candidate region gene 1 protein</td><td>.</td><td>.</td><td>.</td><td>.</td><td>pfam15249</td><td>GLTSCR1</td><td>Glioma tumor suppressor candidate region. This domain family is found in eukaryotes, and is typically between 105 and 124</td><td>.</td><td>.</td><td>.</td><td>.</td><td>.</td></tr>
<tr><td>FASN</td><td>3780.25</td><td>0.56325</td><td>0.113859088</td><td>4.9469</td><td>7.54E-07</td><td>0.00012</td><td>Not_Sig</td><td>no</td><td>gi|528996770|ref|XP_005221054.1|</td><td>12370</td><td>0</td><td>PREDICTED: fatty acid synthase isoform X1 [Bos taurus]</td><td>gi|942083500|ref|XM_005905364.2|</td><td>8169</td><td>0</td><td>PREDICTED: Bos mutus fatty acid synthase (FASN), mRNA</td><td>Q71SP7</td><td>4662</td><td>0</td><td>Fatty acid synthase</td><td>CAC3355</td><td>418</td><td>8.01e-118</td><td>COG3321</td><td>pfam00698</td><td>Acyl_transf_1</td><td>Acyl transferase domain.</td><td>GO:0006633|fatty acid biosynthetic process;</td><td>GO:0005737|cytoplasm;GO:0042470|melanosome;</td><td>GO:0047451|3-hydroxyoctanoyl-[acyl-carrier-protein]</td><td>K00665</td><td>FASN; fatty acid synthase, animal type [EC:2.3.1.85]</td></tr>
<tr><td>SIRT1</td><td>196.9332</td><td>-0.81038</td><td>0.163958646</td><td>-4.9426</td><td>7.709E-07</td><td>0.000121</td><td>DOWN</td><td>yes</td><td>gi|300794673|ref|NP_001179909.1|</td><td>3339</td><td>0</td><td>NAD-dependent protein deacetylase sirtuin-1 [Bos taurus]</td><td>gi|594096651|ref|XM_006071524.1|</td><td>2391</td><td>0</td><td>PREDICTED: Bubalus bubalis sirtuin 1 (SIRT1), mRNA</td><td>Q96EB6</td><td>1166</td><td>0</td><td>NAD-dependent protein deacetylase sirtuin-1</td><td>SPBC16D10.07c</td><td>231</td><td>6.73e-67</td><td>COG0846</td><td>pfam02146</td><td>SIR2</td><td>Sir2 family. This region is characteristic of Silent information regulator 2 (Sir2) proteins, or sirtuins. These are protein</td><td>GO:0001525|angiogenesis;GO:0042595|behavioral response to starvation;GO:0007569|cell aging;GO:0001678|cellular glucose homeostasis;GO:0006974|cellular response to DNA damage stimulus;GO:0070301|cellular response to hydrogen peroxide;GO:0071456|cellular response to hypoxia;GO:0071479|cellular response to ionizing radiation;GO:0009267|cellular response to starvation;GO:0071356|cellular response to tumor necrosis factor;GO:0035356|cellular triglyceride homeostasis;GO:0042632|cholesterol</td><td>GO:0005677|chromatin silencing complex;GO:0005737|cytoplasm;GO:0005739|mitochondrion;GO:0000790|nuclear chromatin;GO:0005635|nuclear</td><td>GO:0043425|bHLH transcription factor binding;GO:0001046|core promoter</td><td>K11411</td><td>SIRT1, SIR2L1; NAD-dependent deacetylase sirtuin 1 [EC:3.5.1.-]</td></tr>
<tr><td>RGCC</td><td>450.657</td><td>-0.61355</td><td>0.125267152</td><td>-4.89793</td><td>9.685E-07</td><td>0.000148</td><td>DOWN</td><td>yes</td><td>gi|156121197|ref|NP_001095746.1|</td><td>406</td><td>2e-45</td><td>regulator of cell cycle RGCC [Bos taurus]</td><td>gi|402693448|ref|NM_001102276.2|</td><td>541</td><td>0</td><td>Bos taurus regulator of cell cycle (RGCC), mRNA</td><td>Q9Z2P4</td><td>143</td><td>3e-40</td><td>Regulator of cell cycle RGCC</td><td>.</td><td>.</td><td>.</td><td>.</td><td>pfam15151</td><td>RGCC</td><td>Response gene to complement 32 protein family. This family of proteins is found in eukaryotes. Proteins in this family are typically</td><td>GO:0071456|cellular response to hypoxia;GO:0006956|complement activation;GO:0072537|fibroblast activation;GO:0071850|mitotic cell cycle arrest;GO:0016525|negative regulation of angiogenesis;GO:0043537|negative regulation of blood vessel endothelial cell migration;GO:2000048|negative regulation of cell-cell adhesion mediated by cadherin;GO:0050710|negative regulation of cytokine secretion;GO:0001937|negative regulation of endothelial cell proliferation;GO:0001100|negative regulation of exit from</td><td>GO:0005813|centrosome;GO:0005737|cytoplasm;GO:0005634|nucleus;</td><td>GO:0030295|protein kinase activator activity;GO:0019901|protein kinase</td><td>.</td><td>.</td></tr>
<tr><td>FSCN1</td><td>3865.485</td><td>0.617642</td><td>0.126124358</td><td>4.897091</td><td>9.727E-07</td><td>0.000148</td><td>UP</td><td>yes</td><td>gi|78045491|ref|NP_001030217.1|</td><td>2551</td><td>0</td><td>fascin [Bos taurus]</td><td>gi|78045490|ref|NM_001035045.1|</td><td>2673</td><td>0</td><td>Bos taurus fascin actin-bundling protein 1 (FSCN1), mRNA</td><td>Q16658</td><td>932</td><td>0</td><td>Fascin</td><td>.</td><td>.</td><td>.</td><td>.</td><td>pfam06268</td><td>Fascin</td><td>Fascin domain. This family consists of several eukaryotic fascin or singed proteins. The fascins are a structurally unique and</td><td>GO:0030036|actin cytoskeleton organization;GO:0051017|actin filament bundle assembly;GO:0009653|anatomical structure morphogenesis;GO:0016477|cell migration;GO:0048870|cell motility;GO:0008283|cell proliferation;GO:0007043|cell-cell junction assembly;GO:0035089|establishment of apical/basal cell polarity;GO:0030035|microspike assembly;GO:0090091|positive regulation of extracellular matrix disassembly;GO:0051491|positive regulation of filopodium assembly;GO:0010592|positive regulation of lamellipodium</td><td>GO:0015629|actin cytoskeleton;GO:0031253|cell projection membrane;GO:0005913|cell-cell adherens junction;GO:0005911|cell-cell</td><td>GO:0003779|actin binding;GO:0051015|actin filament binding;GO:0098641</td><td>K17455</td><td>FSCN1_2; fascin 1/2</td></tr>
<tr><td>SETD1A</td><td>541.2413</td><td>0.732064</td><td>0.149601798</td><td>4.893416</td><td>9.91E-07</td><td>0.000148</td><td>UP</td><td>yes</td><td>gi|329664196|ref|NP_001192362.1|</td><td>3901</td><td>0</td><td>histone-lysine N-methyltransferase SETD1A [Bos taurus]</td><td>gi|982967042|ref|XM_010819452.2|</td><td>5307</td><td>0</td><td>PREDICTED: Bos taurus SET domain containing 1A (SETD1A), transcript variant X5, mRNA</td><td>O15047</td><td>1416</td><td>0</td><td>Histone-lysine N-methyltransferase SETD1A</td><td>SPCC306.04c</td><td>203</td><td>1.65e-52</td><td>COG2940</td><td>pfam11764</td><td>N-SET</td><td>COMPASS (Complex proteins associated with Set1p) component N. The n-SET or N-SET domain is a component of the COMPASS</td><td>GO:2000179|positive regulation of neural precursor cell proliferation;GO:2000648|positive regulation of stem cell proliferation;GO:0006355|regulation of transcription, DNA-templated;GO:0019827|stem cell population maintenance;GO:0006351|transcription, DNA-templated;</td><td>GO:0035097|histone methyltransferase complex;GO:0005719|nuclear euchromatin;GO:0016607|nuclear speck;GO:0005654|nucleoplasm;GO:000563</td><td>GO:0008013|beta-catenin binding;GO:1990188|euchromatin</td><td>K11422</td><td>SETD1, SET1; histone-lysine N-methyltransferase SETD1 [EC:2.1.1.43]</td></tr>
<tr><td>CDC25B</td><td>187.2992</td><td>0.821401</td><td>0.167969795</td><td>4.890172</td><td>1.007E-06</td><td>0.000148</td><td>UP</td><td>yes</td><td>gi|742103475|ref|XP_010834777.1|</td><td>2849</td><td>0</td><td>PREDICTED: M-phase inducer phosphatase 2 isoform X1 [Bison bison bison]</td><td>gi|982942921|ref|XM_005214565.3|</td><td>1928</td><td>0</td><td>PREDICTED: Bos taurus cell division cycle 25B (CDC25B), transcript variant X1, mRNA</td><td>P30305</td><td>906</td><td>0</td><td>M-phase inducer phosphatase 2</td><td>SPAC24H6.05</td><td>137</td><td>1.66e-33</td><td>COG5105</td><td>pfam06617</td><td>M-inducer_phosp</td><td>M-phase inducer phosphatase. This family represents a region within eukaryotic M-phase inducer phosphatases (EC:3.1.3.48), which</td><td>GO:0051301|cell division;GO:0007144|female meiosis I;GO:0000086|G2/M transition of mitotic cell cycle;GO:0000278|mitotic cell cycle;GO:0007067|mitotic nuclear division;GO:0001556|oocyte maturation;GO:0008284|positive regulation of cell proliferation;GO:0032467|positive regulation of cytokinesis;GO:0045931|positive regulation of mitotic cell cycle;GO:0045860|positive regulation of protein kinase activity;GO:0006468|protein phosphorylation;</td><td>GO:0005813|centrosome;GO:0005737|cytoplasm;GO:0005829|cytosol;GO:0005654|nucleoplasm;GO:0000922|spindle pole;</td><td>GO:0004721|phosphoprotein phosphatase activity;GO:0019901</td><td>K05866</td><td>CDC25B; M-phase inducer phosphatase 2 [EC:3.1.3.48]</td></tr>
</table>

<table>
<tr><td>HINT1</td><td>851.7708</td><td>-0.82518</td><td>0.168800625</td><td>-4.8885</td><td>1.016E-06</td><td>0.000148</td><td>DOWN</td><td>yes</td><td>gi|926696466|ref|XP_005682700.2|</td><td>698</td><td>2e-90</td><td>PREDICTED: histidine triad nucleotide-binding protein 1 [Capra hircus]</td><td>gi|942077166|ref|XM_005903289.2|</td><td>621</td><td>0</td><td>PREDICTED: Bos mutus histidine triad nucleotide binding protein 1 (HINT1), mRNA</td><td>P62958</td><td>261</td><td>1e-87</td><td>Histidine triad nucleotide-binding protein 1</td><td>all4087</td><td>157</td><td>9.28e-49</td><td>COG0537</td><td>pfam01230</td><td>HIT</td><td>HIT domain.</td><td>GO:0072332|intrinsic apoptotic signaling pathway by p53 class mediator;GO:0050850|positive regulation of calcium-mediated signaling;GO:0009154|purine ribonucleotide catabolic process;GO:0006355|regulation of transcription, DNA-templated;GO:0006351|transcription, DNA-templated;</td><td>GO:0005737|cytoplasm;GO:0070062|extracellular exosome;GO:0000118|histone deacetylase complex;GO:0005886|plasma membrane;</td><td>GO:0016787|hydrolase activity;GO:0000166|nucleotide binding;</td><td>K02503</td><td>HINT1, hinT, hit; histidine triad (HIT) family protein</td></tr>
<tr><td>ZDHHC8</td><td>426.6378</td><td>1.002772</td><td>0.205141305</td><td>4.8882</td><td>1.018E-06</td><td>0.000148</td><td>UP</td><td>yes</td><td>gi|803322736|ref|XP_012019578.1|</td><td>2859</td><td>0</td><td>PREDICTED: probable palmitoyltransferase ZDHHC8 isoform X3 [Ovis aries musimon]</td><td>gi|982998806|ref|XM_015467253.1|</td><td>3201</td><td>0</td><td>PREDICTED: Bos taurus probable palmitoyltransferase ZDHHC8 (LOC526847), transcript variant X2, mRNA</td><td>Q2THW8</td><td>1037</td><td>0</td><td>Probable palmitoyltransferase ZDHHC8</td><td>SPBC3H7.09</td><td>119</td><td>4.80e-28</td><td>COG5273</td><td>pfam01529</td><td>zf-DHHC</td><td>DHHC palmitoyltransferase. This family includes the well known DHHC zinc binding domain as well as three of the four conserved</td><td>GO:0018345|protein palmitoylation;</td><td>GO:0030659|cytoplasmic vesicle membrane;GO:0005794|Golgi apparatus;GO:0016021|integral component of membrane;</td><td>GO:0016409|palmitoyltransferase activity;GO:0019706|protein-cysteine S-</td><td>.</td><td>.</td></tr>
<tr><td>PCNX3</td><td>905.5476</td><td>0.828128</td><td>0.169707962</td><td>4.879725</td><td>1.062E-06</td><td>0.000153</td><td>UP</td><td>yes</td><td>gi|300798131|ref|NP_001179822.1|</td><td>9192</td><td>0</td><td>pecanex-like protein 3 [Bos taurus]</td><td>gi|982971992|ref|XM_005227131.3|</td><td>6929</td><td>0</td><td>PREDICTED: Bos taurus pecanex homolog 3 (Drosophila) (PCNX3), transcript variant X1, mRNA</td><td>Q9H6A9</td><td>3321</td><td>0</td><td>Pecanex-like protein 3</td><td>.</td><td>.</td><td>.</td><td>.</td><td>pfam05041</td><td>Pecanex_C</td><td>Pecanex protein (C-terminus). This family consists of C terminal region of the pecanex protein homologues. The pecanex protein</td><td>.</td><td>GO:0016021|integral component of membrane;</td><td>.</td><td>.</td><td>.</td></tr>
<tr><td>MAP1A</td><td>5000.187</td><td>0.51271</td><td>0.105285915</td><td>4.869696</td><td>1.118E-06</td><td>0.000159</td><td>Not_Sig</td><td>no</td><td>gi|741898604|ref|XP_010823231.1|</td><td>12572</td><td>0</td><td>PREDICTED: microtubule-associated protein 1A isoform X1 [Bos taurus]</td><td>gi|983003960|ref|XM_010824929.2|</td><td>10616</td><td>0</td><td>PREDICTED: Bos taurus microtubule associated protein 1A (MAP1A), transcript variant X1, mRNA</td><td>P78559</td><td>3358</td><td>0</td><td>Microtubule-associated protein 1A</td><td>.</td><td>.</td><td>.</td><td>.</td><td>pfam09528</td><td>Ehrlichia_rpt</td><td>Ehrlichia tandem repeat (Ehrlichia_rpt). This entry represents 77 residues of an 80 amino acid (240 nucleotide)</td><td>GO:0000226|microtubule cytoskeleton organization;</td><td>GO:0005737|cytoplasm;GO:0005874|microtubule;GO:0005875|microtubule associated complex;</td><td>GO:0005198|structural molecule activity;</td><td>K10429</td><td>MAP1; microtubule-associated protein 1</td></tr>
<tr><td>LOC138989917</td><td>1581.825</td><td>-0.76731</td><td>0.157692914</td><td>-4.86585</td><td>1.14E-06</td><td>0.00016</td><td>DOWN</td><td>yes</td><td>gi|594076095|ref|XP_006061809.1|</td><td>318</td><td>3e-34</td><td>PREDICTED: thymosin beta-4-like [Bubalus bubalis]</td><td>gi|124829100|gb|BC133478.1|</td><td>608</td><td>0</td><td>Bos taurus thymosin beta 4, X-linked, mRNA (cDNA clone MGC:157367 IMAGE:8260878), complete cds</td><td>P20065</td><td>89.4</td><td>1e-21</td><td>Thymosin beta-4</td><td>.</td><td>.</td><td>.</td><td>.</td><td>pfam01290</td><td>Thymosin</td><td>Thymosin beta-4 family.</td><td>GO:0030036|actin cytoskeleton organization;GO:0007015|actin filament organization;GO:0001649|osteoblast differentiation;GO:0051152|positive regulation of smooth muscle cell differentiation;GO:0014911|positive regulation of smooth muscle cell migration;GO:0045893|positive regulation of transcription, DNA-templated;GO:0030334|regulation of cell migration;GO:0042989|sequestering of actin monomers;</td><td>GO:0005829|cytosol;GO:0031941|filamentous actin;GO:0005634|nucleus;</td><td>GO:0003785|actin monomer binding;</td><td>K05764</td><td>TMSB4; thymosin, beta 4</td></tr>
<tr><td>STK38L</td><td>1419.816</td><td>-0.50566</td><td>0.104066444</td><td>-4.85905</td><td>1.18E-06</td><td>0.000163</td><td>Not_Sig</td><td>no</td><td>gi|859927559|ref|XP_012903255.1|</td><td>2524</td><td>0</td><td>PREDICTED: serine/threonine-protein kinase 38-like isoform X1 [Mustela putorius furo]</td><td>gi|942074797|ref|XM_005902453.2|</td><td>3107</td><td>0</td><td>PREDICTED: Bos mutus serine/threonine kinase 38 like (STK38L), mRNA</td><td>Q9Y2H1</td><td>963</td><td>0</td><td>Serine/threonine-protein kinase 38-like</td><td>.</td><td>.</td><td>.</td><td>.</td><td>pfam00069</td><td>Pkinase</td><td>Protein kinase domain.</td><td>GO:0035556|intracellular signal transduction;GO:0018105|peptidyl-serine phosphorylation;GO:0006468|protein phosphorylation;GO:0051128|regulation of cellular component organization;</td><td>GO:0015629|actin cytoskeleton;GO:0005737|cytoplasm;GO:0016020|membrane;</td><td>GO:0005524|ATP binding;GO:0000287|magnesium ion binding;GO:0004674</td><td>K08790</td><td>STK38, NDR; serine/threonine kinase 38 [EC:2.7.11.1]</td></tr>
<tr><td>PLEKHG2</td><td>612.1159</td><td>0.671334</td><td>0.138569093</td><td>4.844759</td><td>1.268E-06</td><td>0.000172</td><td>UP</td><td>yes</td><td>gi|741959685|ref|XP_010813238.1|</td><td>5841</td><td>0</td><td>PREDICTED: pleckstrin homology domain-containing family G member 2 isoform X1 [Bos taurus]</td><td>gi|594090632|ref|XM_006068655.1|</td><td>5161</td><td>0</td><td>PREDICTED: Bubalus bubalis pleckstrin homology domain containing, family G (with RhoGef domain) member 2 (PLEKHG2), transcript variant X2, mRNA</td><td>Q9H7P9</td><td>1649</td><td>0</td><td>Pleckstrin homology domain-containing family G member 2</td><td>.</td><td>.</td><td>.</td><td>.</td><td>pfam00621</td><td>RhoGEF</td><td>RhoGEF domain. Guanine nucleotide exchange factor for Rho/Rac/Cdc42-like GTPases Also called Dbl-homologous (DH)</td><td>GO:0043065|positive regulation of apoptotic process;GO:0035023|regulation of Rho protein signal transduction;GO:0051056|regulation of small GTPase mediated signal transduction;</td><td>GO:0005829|cytosol;</td><td>GO:0005085|guanyl-nucleotide exchange factor activity;GO:0005089</td><td>.</td><td>.</td></tr>
<tr><td>NDUFA2</td><td>398.9735</td><td>-0.81386</td><td>0.167998676</td><td>-4.84445</td><td>1.27E-06</td><td>0.000172</td><td>DOWN</td><td>yes</td><td>gi|28461257|ref|NP_787009.1|</td><td>489</td><td>1e-60</td><td>NADH dehydrogenase [ubiquinone] 1 alpha subcomplex subunit 2 [Bos taurus]</td><td>gi|742166133|ref|XM_010850362.1|</td><td>454</td><td>0</td><td>PREDICTED: Bison bison bison NADH dehydrogenase (ubiquinone) 1 alpha subcomplex, 2, 8kDa (NDUFA2), mRNA</td><td>Q02370</td><td>192</td><td>4e-62</td><td>NADH dehydrogenase [ubiquinone] 1 alpha subcomplex subunit 2</td><td>.</td><td>.</td><td>.</td><td>.</td><td>pfam05047</td><td>L51_S25_CI-B8</td><td>Mitochondrial ribosomal protein L51 / S25 / CI-B8 domain. The proteins in this family are located in the mitochondrion. The family</td><td>.</td><td>GO:0031966|mitochondrial membrane;GO:0005747|mitochondrial respiratory chain complex I;</td><td>GO:0008137|NADH dehydrogenase (ubiquinone) activity;</td><td>K03946</td><td>NDUFA2; NADH dehydrogenase (ubiquinone) 1 alpha subcomplex subunit</td></tr>
<tr><td>FAM234A</td><td>586.6096</td><td>0.743649</td><td>0.15365287</td><td>4.839796</td><td>1.3E-06</td><td>0.000174</td><td>UP</td><td>yes</td><td>gi|115496776|ref|NP_001068786.1|</td><td>2620</td><td>0</td><td>protein ITFG3 [Bos taurus]</td><td>gi|982965974|ref|XM_005200983.3|</td><td>2444</td><td>0</td><td>PREDICTED: Bos taurus family with sequence similarity 234 member A (FAM234A), transcript variant X1, mRNA</td><td>Q2HJE5</td><td>1013</td><td>0</td><td>Protein FAM234A</td><td>.</td><td>.</td><td>.</td><td>.</td><td>pfam13360</td><td>PQQ_2</td><td>PQQ-like domain. This domain contains several repeats of the PQQ repeat.</td><td>.</td><td>GO:0016021|integral component of membrane;</td><td>.</td><td>K17258</td><td>ITFG3; integrin alpha FG-GAP repeat containing protein 3</td></tr>
<tr><td>SLC30A4</td><td>1362.724</td><td>-0.55817</td><td>0.115492446</td><td>-4.83294</td><td>1.345E-06</td><td>0.000178</td><td>Not_Sig</td><td>no</td><td>gi|119902645|ref|XP_613527.3|</td><td>2171</td><td>0</td><td>PREDICTED: zinc transporter 4 [Bos taurus]</td><td>gi|982989720|ref|XM_613527.9|</td><td>5702</td><td>0</td><td>PREDICTED: Bos taurus solute carrier family 30 (zinc transporter), member 4 (SLC30A4), transcript variant X1, mRNA</td><td>O14863</td><td>752</td><td>0</td><td>Zinc transporter 4</td><td>ECU11g1510</td><td>166</td><td>3.75e-44</td><td>COG1230</td><td>pfam01545</td><td>Cation_efflux</td><td>Cation efflux family. Members of this family are integral membrane proteins, that are found to increase tolerance to divalent</td><td>GO:0061088|regulation of sequestering of zinc ion;GO:0009636|response to toxic substance;GO:0010043|response to zinc ion;GO:0055069|zinc ion homeostasis;</td><td>GO:0005737|cytoplasm;GO:0016023|cytoplasmic membrane-bounded vesicle;GO:0016021|integral component of membrane;GO:0005770|late</td><td>GO:0005385|zinc ion transmembrane transporter activity;</td><td>K14691</td><td>SLC30A4, ZNT4; solute carrier family 30 (zinc transporter), member 4</td></tr>
<tr><td>CHERP</td><td>588.5544</td><td>0.708685</td><td>0.147091053</td><td>4.818001</td><td>1.45E-06</td><td>0.000189</td><td>UP</td><td>yes</td><td>gi|528954493|ref|XP_005208565.1|</td><td>2694</td><td>0</td><td>PREDICTED: calcium homeostasis endoplasmic reticulum protein isoform X1 [Bos taurus]</td><td>gi|982929609|ref|XM_005208508.3|</td><td>3909</td><td>0</td><td>PREDICTED: Bos taurus calcium homeostasis endoplasmic reticulum protein (CHERP), transcript variant X1, mRNA</td><td>Q8IWX8</td><td>980</td><td>0</td><td>Calcium homeostasis endoplasmic reticulum protein</td><td>.</td><td>.</td><td>.</td><td>.</td><td>pfam01805</td><td>Surp</td><td>Surp module. This domain is also known as the SWAP domain. SWAP stands for Suppressor-of-White-APricot. It has been</td><td>GO:0006874|cellular calcium ion homeostasis;GO:0008285|negative regulation of cell proliferation;GO:0007399|nervous system development;GO:0051533|positive regulation of NFAT protein import into nucleus;GO:0051209|release of sequestered calcium ion into cytosol;GO:0006396|RNA processing;</td><td>GO:0005737|cytoplasm;GO:0016020|membrane;GO:0048471|perinuclear region of cytoplasm;GO:0033017|sarcoplasmic reticulum membrane;</td><td>GO:0044325|ion channel binding;GO:0044822|poly(A) RNA</td><td>K12841</td><td>CHERP; calcium homeostasis endoplasmic reticulum protein</td></tr>
<tr><td>KIF18B</td><td>223.2427</td><td>0.891009</td><td>0.185182829</td><td>4.811509</td><td>1.498E-06</td><td>0.000193</td><td>UP</td><td>yes</td><td>gi|555983005|ref|XP_005903406.1|</td><td>4025</td><td>0</td><td>PREDICTED: kinesin-like protein KIF18B [Bos mutus]</td><td>gi|742086583|ref|XM_010832420.1|</td><td>3033</td><td>0</td><td>PREDICTED: Bison bison bison kinesin family member 18B (KIF18B), transcript variant X1, mRNA</td><td>Q86Y91</td><td>1227</td><td>0</td><td>Kinesin-like protein KIF18B</td><td>.</td><td>.</td><td>.</td><td>.</td><td>pfam00225</td><td>Kinesin</td><td>Kinesin motor domain.</td><td>GO:0051301|cell division;GO:0007019|microtubule depolymerization;GO:0007018|microtubule-based movement;GO:0000278|mitotic cell cycle;GO:0000070|mitotic sister chromatid segregation;GO:0051302|regulation of cell division;</td><td>GO:0000235|astral microtubule;GO:0005737|cytoplasm;GO:0005871|kinesin complex;GO:1990752|microtubule</td><td>GO:0005524|ATP binding;GO:0008574|ATP-dependent microtubule motor</td><td>.</td><td>.</td></tr>
<tr><td>RAVER1</td><td>858.792</td><td>0.801801</td><td>0.166886326</td><td>4.804474</td><td>1.552E-06</td><td>0.000198</td><td>UP</td><td>yes</td><td>gi|741932000|ref|XP_010805073.1|</td><td>2574</td><td>0</td><td>PREDICTED: ribonucleoprotein PTB-binding 1 isoform X1 [Bos taurus]</td><td>gi|742218708|ref|XM_010828368.1|</td><td>3586</td><td>0</td><td>PREDICTED: Bison bison bison ribonucleoprotein, PTB-binding 1 (RAVER1), transcript variant X1, mRNA</td><td>Q5XI28</td><td>840</td><td>0</td><td>Ribonucleoprotein PTB-binding 1</td><td>.</td><td>.</td><td>.</td><td>.</td><td>pfam00076</td><td>RRM_1</td><td>RNA recognition motif. (a.k.a. RRM, RBD, or RNP domain). The RRM motif is probably diagnostic of an RNA binding protein. RRMs</td><td>GO:0000398|mRNA splicing, via spliceosome;</td><td>GO:0005737|cytoplasm;GO:0005634|nucleus;</td><td>GO:0003676|nucleic acid binding;GO:0000166|nucleotide</td><td>.</td><td>.</td></tr>
<tr><td>ABCA2</td><td>408.5736</td><td>0.768593</td><td>0.160272412</td><td>4.795544</td><td>1.622E-06</td><td>0.000205</td><td>UP</td><td>yes</td><td>gi|594077891|ref|XP_006062658.1|</td><td>11127</td><td>0</td><td>PREDICTED: ATP-binding cassette sub-family A member 2, partial [Bubalus bubalis]</td><td>gi|965898903|ref|XM_012161031.2|</td><td>7101</td><td>0</td><td>PREDICTED: Ovis aries musimon ATP binding cassette subfamily A member 2 (ABCA2), transcript variant X3, mRNA</td><td>P41234</td><td>4012</td><td>0</td><td>ATP-binding cassette sub-family A member 2</td><td>MA1536</td><td>172</td><td>9.91e-46</td><td>COG1131</td><td>pfam00005</td><td>ABC_tran</td><td>ABC transporter. ABC transporters for a large family of proteins responsible for translocation of a variety of compounds across</td><td>GO:0033344|cholesterol efflux;GO:0042632|cholesterol homeostasis;GO:0033700|phospholipid efflux;GO:0032383|regulation of intracellular cholesterol transport;GO:0006357|regulation of transcription from RNA polymerase II promoter;GO:0048545|response to steroid hormone;</td><td>GO:0016023|cytoplasmic membrane-bounded vesicle;GO:0005768|endosome;GO:0010008|endosome</td><td>GO:0005524|ATP binding;GO:0016887|ATPase activity;GO:0042626</td><td>.</td><td>.</td></tr>
<tr><td>LRP5</td><td>639.9048</td><td>0.691493</td><td>0.144431037</td><td>4.787705</td><td>1.687E-06</td><td>0.000211</td><td>UP</td><td>yes</td><td>gi|296471561|tpg|DAA13676.1|</td><td>8234</td><td>0</td><td>TPA: low density lipoprotein receptor-related protein 5 [Bos taurus]</td><td>gi|594080809|ref|XM_006063975.1|</td><td>4634</td><td>0</td><td>PREDICTED: Bubalus bubalis low density lipoprotein receptor-related protein 5 (LRP5), mRNA</td><td>O75197</td><td>3117</td><td>0</td><td>Low-density lipoprotein receptor-related protein 5</td><td>MT0958_2</td><td>55.1</td><td>5.41e-07</td><td>COG3391</td><td>pfam00058</td><td>Ldl_recept_b</td><td>Low-density lipoprotein receptor repeat class B. This domain is also known as the YWTD motif after the most conserved region of the</td><td>GO:0060612|adipose tissue development;GO:0060033|anatomical structure regression;GO:0009952|anterior/posterior pattern specification;GO:1902262|apoptotic process involved in patterning of blood vessels;GO:0048539|bone marrow development;GO:0060349|bone morphogenesis;GO:0046849|bone remodeling;GO:0060444|branching involved in mammary gland duct morphogenesis;GO:0060070|canonical Wnt signaling pathway;GO:0042074|cell migration involved in gastrulation;GO:0060764|cell-cell</td><td>GO:0005783|endoplasmic reticulum;GO:0016021|integral component of membrane;GO:0005739|mitochondrion;GO:</td><td>GO:0071936|coreceptor activity involved in Wnt signaling</td><td>K03068</td><td>LRP5_6; low density lipoprotein receptor-related protein 5/6</td></tr>
<tr><td>POLD1</td><td>507.7062</td><td>0.628993</td><td>0.132266166</td><td>4.755506</td><td>1.98E-06</td><td>0.000245</td><td>UP</td><td>yes</td><td>gi|27806073|ref|NP_776852.1|</td><td>5237</td><td>0</td><td>DNA polymerase delta catalytic subunit [Bos taurus]</td><td>gi|31342028|ref|NM_174427.2|</td><td>3388</td><td>0</td><td>Bos taurus polymerase (DNA directed), delta 1, catalytic subunit (POLD1), mRNA</td><td>P28339</td><td>2022</td><td>0</td><td>DNA polymerase delta catalytic subunit</td><td>SPBC336.04</td><td>1125</td><td>0</td><td>COG0417</td><td>pfam00136</td><td>DNA_pol_B</td><td>DNA polymerase family B. This region of DNA polymerase B appears to consist of more than one structural domain, possibly</td><td>GO:0006287|base-excision repair, gap-filling;GO:0034644|cellular response to UV;GO:0006260|DNA replication;GO:0045004|DNA replication proofreading;GO:0055089|fatty acid homeostasis;GO:0006297|nucleotide-excision repair, DNA gap filling;</td><td>GO:0043625|delta DNA polymerase complex;GO:0005634|nucleus;</td><td>GO:0008296|3'-5'-exodeoxyribonuclease activity;GO:0051539</td><td>K02327</td><td>POLD1; DNA polymerase delta subunit 1 [EC:2.7.7.7]</td></tr>
<tr><td>CCNF</td><td>183.6777</td><td>0.827658</td><td>0.175408619</td><td>4.718454</td><td>2.376E-06</td><td>0.00029</td><td>UP</td><td>yes</td><td>gi|149642847|ref|NP_001092340.1|</td><td>3886</td><td>0</td><td>cyclin-F [Bos taurus]</td><td>gi|742119914|ref|XM_010840323.1|</td><td>2595</td><td>0</td><td>PREDICTED: Bison bison bison cyclin F (CCNF), mRNA</td><td>A5PK16</td><td>1499</td><td>0</td><td>Cyclin-F</td><td>.</td><td>.</td><td>.</td><td>.</td><td>pfam00134</td><td>Cyclin_N</td><td>Cyclin, N-terminal domain. Cyclins regulate cyclin dependent kinases (CDKs). Human cyclin-O is a Uracil-DNA glycosylase that is related to</td><td>GO:0051301|cell division;GO:0007067|mitotic nuclear division;GO:0010826|negative regulation of centrosome duplication;GO:0001890|placenta development;GO:0016567|protein ubiquitination;GO:0000320|re-entry into mitotic cell cycle;GO:0031146|SCF-dependent proteasomal ubiquitin-dependent protein catabolic process;</td><td>GO:0005814|centriole;GO:0005634|nucleus;GO:0019005|SCF ubiquitin ligase complex;</td><td>.</td><td>K10289</td><td>CCNF, FBXO1; F-box protein 1 (cyclin F)</td></tr>
<tr><td>RPS19</td><td>4435.947</td><td>-0.78357</td><td>0.166438642</td><td>-4.70784</td><td>2.504E-06</td><td>0.000303</td><td>DOWN</td><td>yes</td><td>gi|887492898|pdb|5A2Q|T</td><td>727</td><td>9e-96</td><td>Chain T, Structure Of The Hcv Ires Bound To The Human Ribosome</td><td>gi|942095005|ref|XM_005911732.2|</td><td>496</td><td>0</td><td>PREDICTED: Bos mutus ribosomal protein S19 (RPS19), mRNA</td><td>Q5R8M9</td><td>284</td><td>3e-97</td><td>40S ribosomal protein S19</td><td>SPBC649.02</td><td>171</td><td>9.32e-55</td><td>COG2238</td><td>pfam01090</td><td>Ribosomal_S19e</td><td>Ribosomal protein S19e.</td><td>GO:0030218|erythrocyte differentiation;GO:0000462|maturation of SSU-rRNA from tricistronic rRNA transcript (SSU-rRNA, 5.8S rRNA, LSU-rRNA);GO:0002548|monocyte chemotaxis;GO:0060266|negative regulation of respiratory burst involved in inflammatory response;GO:0007219|Notch signaling pathway;GO:0007000|nucleolus organization;GO:0060265|positive regulation of respiratory burst involved in inflammatory response;GO:0051262|protein tetramerization;GO:0000028|ribosomal small subunit</td><td>GO:0022627|cytosolic small ribosomal subunit;GO:0070062|extracellular exosome;GO:0005925|focal adhesion;GO:0016020|membrane;GO:0005</td><td>GO:0044822|poly(A) RNA binding;GO:0003735|structural</td><td>K02966</td><td>RP-S19e, RPS19; small subunit ribosomal protein S19e</td></tr>
<tr><td>RNF11</td><td>802.4163</td><td>-0.58981</td><td>0.12538079</td><td>-4.70416</td><td>2.549E-06</td><td>0.000305</td><td>DOWN</td><td>yes</td><td>gi|12004970|gb|AAG44245.1|</td><td>713</td><td>1e-84</td><td>Nedd4 WW domain-binding protein 2 [Mus musculus]</td><td>gi|594070881|ref|XM_006059255.1|</td><td>2525</td><td>0</td><td>PREDICTED: Bubalus bubalis ring finger protein 11 (RNF11), mRNA</td><td>Q9Y3C5</td><td>266</td><td>3e-81</td><td>RING finger protein 11</td><td>.</td><td>.</td><td>.</td><td>.</td><td>pfam13639</td><td>zf-RING_2</td><td>Ring finger domain.</td><td>GO:0051865|protein autoubiquitination;GO:0042787|protein ubiquitination involved in ubiquitin-dependent protein catabolic process;</td><td>GO:0005769|early endosome;GO:0070062|extracellular exosome;GO:0005634|nucleus;GO:0055037|recycling endosome;GO:0000151|ubiquitin</td><td>GO:0003677|DNA binding;GO:0004842|ubiquitin-protein transferase</td><td>K11980</td><td>RNF11; E3 ubiquitin-protein ligase RNF11</td></tr>
<tr><td>GNA11</td><td>911.0395</td><td>0.517123</td><td>0.110198091</td><td>4.692671</td><td>2.697E-06</td><td>0.000319</td><td>Not_Sig</td><td>no</td><td>gi|466088400|ref|XP_004286353.1|</td><td>1903</td><td>0</td><td>PREDICTED: guanine nucleotide-binding protein subunit alpha-11 [Orcinus orca]</td><td>gi|134085330|ref|NM_174322.3|</td><td>1500</td><td>0</td><td>Bos taurus guanine nucleotide binding protein (G protein), alpha 11 (Gq class) (GNA11), mRNA</td><td>P38409</td><td>737</td><td>0</td><td>Guanine nucleotide-binding protein subunit alpha-11</td><td>.</td><td>.</td><td>.</td><td>.</td><td>pfam00503</td><td>G-alpha</td><td>G-protein alpha subunit. G proteins couple receptors of extracellular signals to intracellular signaling pathways. The G protein</td><td>GO:0001508|action potential;GO:0071467|cellular response to pH;GO:0048066|developmental pigmentation;GO:0009649|entrainment of circadian clock;GO:0007213|G-protein coupled acetylcholine receptor signaling pathway;GO:0007507|heart development;GO:0060158|phospholipase C-activating dopamine receptor signaling pathway;GO:0007603|phototransduction, visible light;GO:0045634|regulation of melanocyte differentiation;GO:0001501|skeletal system development;</td><td>GO:0070062|extracellular exosome;GO:0005765|lysosomal membrane;GO:0016020|membrane;GO:0001750|photoreceptor outer</td><td>GO:0005525|GTP binding;GO:0003924|GTPase activity;GO:0046872</td><td>K04635</td><td>GNA11; guanine nucleotide-binding protein subunit alpha-11</td></tr>
<tr><td>HCFC1</td><td>1803.051</td><td>0.754901</td><td>0.161053085</td><td>4.687281</td><td>2.769E-06</td><td>0.000325</td><td>UP</td><td>yes</td><td>gi|803323989|ref|XP_012020199.1|</td><td>7755</td><td>0</td><td>PREDICTED: host cell factor 1 isoform X2 [Ovis aries musimon]</td><td>gi|594038180|ref|XM_006043649.1|</td><td>8707</td><td>0</td><td>PREDICTED: Bubalus bubalis host cell factor C1 (VP16-accessory protein) (HCFC1), transcript variant X1, mRNA</td><td>P51611</td><td>2647</td><td>0</td><td>Host cell factor 1</td><td>.</td><td>.</td><td>.</td><td>.</td><td>pfam13415</td><td>Kelch_3</td><td>Galactose oxidase, central domain.</td><td>GO:0007049|cell cycle;GO:0043984|histone H4-K16 acetylation;GO:0043981|histone H4-K5 acetylation;GO:0043982|histone H4-K8 acetylation;GO:0000122|negative regulation of transcription from RNA polymerase II promoter;GO:0045931|positive regulation of mitotic cell cycle;GO:0050821|protein stabilization;</td><td>GO:0005737|cytoplasm;GO:0000123|histone acetyltransferase complex;GO:0071339|MLL1 complex;GO:0043025|neuronal cell</td><td>GO:0003682|chromatin binding;GO:0003713|transcription</td><td>.</td><td>.</td></tr>
<tr><td>NCKAP5L</td><td>1027.989</td><td>0.720344</td><td>0.153762137</td><td>4.684791</td><td>2.802E-06</td><td>0.000325</td><td>UP</td><td>yes</td><td>gi|741925904|ref|XP_010803243.1|</td><td>5090</td><td>0</td><td>PREDICTED: nck-associated protein 5-like isoform X1 [Bos taurus]</td><td>gi|982924915|ref|XM_010804941.2|</td><td>4871</td><td>0</td><td>PREDICTED: Bos taurus NCK associated protein 5 like (NCKAP5L), transcript variant X1, mRNA</td><td>Q9HCH0</td><td>1649</td><td>0</td><td>Nck-associated protein 5-like</td><td>.</td><td>.</td><td>.</td><td>.</td><td>pfam15246</td><td>NCKAP5</td><td>Nck-associated protein 5, Peripheral clock protein. NCKAP5 is short for Nck-associated protein 5, which is also known as the</td><td>GO:0001578|microtubule bundle formation;GO:0007019|microtubule depolymerization;</td><td>GO:0005813|centrosome;GO:0005737|cytoplasm;GO:0035371|microtubule plus-end;</td><td>.</td><td>.</td><td>.</td></tr>
<tr><td>MCM4</td><td>1109.273</td><td>0.491019</td><td>0.105002145</td><td>4.676276</td><td>2.921E-06</td><td>0.000336</td><td>Not_Sig</td><td>no</td><td>gi|115495629|ref|NP_001068626.1|</td><td>4350</td><td>0</td><td>DNA replication licensing factor MCM4 [Bos taurus]</td><td>gi|115495628|ref|NM_001075158.1|</td><td>3044</td><td>0</td><td>Bos taurus minichromosome maintenance complex component 4 (MCM4), mRNA</td><td>P33991</td><td>1574</td><td>0</td><td>DNA replication licensing factor MCM4</td><td>YPR019w</td><td>676</td><td>0</td><td>COG1241</td><td>pfam00493</td><td>MCM</td><td>MCM2/3/5 family.</td><td>GO:0006260|DNA replication;GO:0006270|DNA replication initiation;GO:0006268|DNA unwinding involved in DNA replication;GO:0000082|G1/S transition of mitotic cell cycle;</td><td>GO:0042555|MCM complex;GO:0016020|membrane;GO:0000784|nuclear chromosome, telomeric region;GO:0005654|nucleoplasm;GO:00056</td><td>GO:0005524|ATP binding;GO:0003678|DNA helicase activity;GO:0003697</td><td>K02212</td><td>MCM4, CDC54; DNA replication licensing factor MCM4 [EC:3.6.4.12]</td></tr>
<tr><td>SIPA1L3</td><td>390.2416</td><td>0.766933</td><td>0.16410506</td><td>4.673429</td><td>2.962E-06</td><td>0.000336</td><td>UP</td><td>yes</td><td>gi|803253452|ref|XP_011986687.1|</td><td>8552</td><td>0</td><td>PREDICTED: signal-induced proliferation-associated 1-like protein 3 isoform X2 [Ovis aries musimon]</td><td>gi|982999765|ref|XM_015467479.1|</td><td>8007</td><td>0</td><td>PREDICTED: Bos taurus signal-induced proliferation-associated 1 like 3 (SIPA1L3), transcript variant X2, mRNA</td><td>O60292</td><td>3066</td><td>0</td><td>Signal-induced proliferation-associated 1-like protein 3</td><td>.</td><td>.</td><td>.</td><td>.</td><td>pfam02145</td><td>Rap_GAP</td><td>Rap/ran-GAP.</td><td>GO:0007010|cytoskeleton organization;GO:0003382|epithelial cell morphogenesis;GO:0090162|establishment of epithelial cell polarity;GO:0001654|eye development;GO:0002244|hematopoietic progenitor cell differentiation;GO:0051056|regulation of small GTPase mediated signal transduction;</td><td>GO:0045177|apical part of cell;GO:0016324|apical plasma membrane;GO:0005615|extracellular space;GO:0001725|stress</td><td>GO:0005096|GTPase activator activity;</td><td>K17703</td><td>SIPA1L3, SPAL3; signal-induced proliferation-associated 1 like</td></tr>
<tr><td>KAT2A</td><td>395.3721</td><td>0.664142</td><td>0.142156334</td><td>4.67191</td><td>2.984E-06</td><td>0.000336</td><td>UP</td><td>yes</td><td>gi|594063584|ref|XP_006055821.1|</td><td>3647</td><td>0</td><td>PREDICTED: histone acetyltransferase KAT2A isoform X1 [Bubalus bubalis]</td><td>gi|942064692|ref|XM_014479265.1|</td><td>3081</td><td>0</td><td>PREDICTED: Bos mutus K(lysine) acetyltransferase 2A (KAT2A), transcript variant X1, mRNA</td><td>Q92830</td><td>1393</td><td>0</td><td>Histone acetyltransferase KAT2A</td><td>SPAC1952.05</td><td>317</td><td>1.98e-97</td><td>COG5076</td><td>pfam06466</td><td>PCAF_N</td><td>PCAF (P300/CBP-associated factor) N-terminal domain. This region is spliced out of human histone acetyltranferase KAT2A isoform 2.</td><td>GO:0071929|alpha-tubulin acetylation;GO:0055007|cardiac muscle cell differentiation;GO:0008283|cell proliferation;GO:1990090|cellular response to nerve growth factor stimulus;GO:0071356|cellular response to tumor necrosis factor;GO:0006338|chromatin remodeling;GO:0016578|histone deubiquitination;GO:0043966|histone H3 acetylation;GO:0044154|histone H3-K14 acetylation;GO:0001701|in utero embryonic development;GO:0048312|intracellular distribution of</td><td>GO:0005671|Ada2/Gcn5/Ada3 transcription activator complex;GO:0005615|extracellular space;GO:0072686|mitotic spindle;GO:0000790|nuclear</td><td>GO:0003682|chromatin binding;GO:0010484|H3 histone</td><td>K06062</td><td>PCAF, KAT2, GCN5; histone acetyltransferase [EC:2.3.1.48]</td></tr>
<tr><td>AKT1</td><td>3293.572</td><td>0.433196</td><td>0.092969277</td><td>4.659558</td><td>3.169E-06</td><td>0.000352</td><td>Not_Sig</td><td>no</td><td>gi|595763479|ref|NP_001277770.1|</td><td>2589</td><td>0</td><td>v-akt murine thymoma viral oncogene homolog 1 [Bubalus bubalis]</td><td>gi|594036166|ref|XM_006042689.1|</td><td>2232</td><td>0</td><td>PREDICTED: Bubalus bubalis v-akt murine thymoma viral oncogene homolog 1 (AKT1), transcript variant X2, mRNA</td><td>Q01314</td><td>995</td><td>0</td><td>RAC-alpha serine/threonine-protein kinase</td><td>SPAC17G8.14c_2</td><td>327</td><td>8.03e-104</td><td>COG0515</td><td>pfam00069</td><td>Pkinase</td><td>Protein kinase domain.</td><td>GO:0006915|apoptotic process;GO:0008643|carbohydrate transport;GO:0006006|glucose metabolic process;GO:0005978|glycogen biosynthetic process;GO:0035556|intracellular signal transduction;GO:0043154|negative regulation of cysteine-type endopeptidase activity involved in apoptotic process;GO:0090201|negative regulation of release of cytochrome c from mitochondria;GO:0018105|peptidyl-serine phosphorylation;GO:0016310|phosphorylation;GO:0001938|positive regulation of endothelial cell proliferation;GO:0006468|protein</td><td>GO:0005737|cytoplasm;GO:0005622|intracellular;GO:0005634|nucleus;GO:0005886|plasma membrane;</td><td>GO:0005524|ATP binding;GO:0004674|protein serine/threonine</td><td>K04456</td><td>AKT; RAC serine/threonine-protein kinase [EC:2.7.11.1]</td></tr>
<tr><td>PLOD2</td><td>12316.62</td><td>-0.54034</td><td>0.115983725</td><td>-4.65872</td><td>3.182E-06</td><td>0.000352</td><td>Not_Sig</td><td>no</td><td>gi|528937565|ref|XP_005201855.1|</td><td>3761</td><td>0</td><td>PREDICTED: procollagen-lysine,2-oxoglutarate 5-dioxygenase 2 isoform X1 [Bos taurus]</td><td>gi|965907749|ref|XM_015090608.1|</td><td>3505</td><td>0</td><td>PREDICTED: Ovis aries musimon procollagen-lysine, 2-oxoglutarate 5-dioxygenase 2 (PLOD2), transcript variant X2, mRNA</td><td>O00469</td><td>1394</td><td>0</td><td>Procollagen-lysine,2-oxoglutarate 5-dioxygenase 2</td><td>.</td><td>.</td><td>.</td><td>.</td><td>pfam03171</td><td>2OG-FeII_Oxy</td><td>2OG-Fe(II) oxygenase superfamily. This family contains members of the 2-oxoglutarate (2OG) and Fe(II)-dependent oxygenase</td><td>GO:0006464|cellular protein modification process;GO:0001666|response to hypoxia;</td><td>GO:0005783|endoplasmic reticulum;GO:0005789|endoplasmic reticulum membrane;GO:0070062|extracellular</td><td>GO:0005506|iron ion binding;GO:0031418|L-ascorbic acid</td><td>K13645</td><td>PLOD2; procollagen-lysine,2-oxoglutarate 5-dioxygenase 2 [EC:1.14.11.4]</td></tr>
<tr><td>C21H15orf39</td><td>323.822</td><td>0.809504</td><td>0.173983671</td><td>4.652761</td><td>3.275E-06</td><td>0.000358</td><td>UP</td><td>yes</td><td>gi|119913806|ref|XP_001252070.1|</td><td>4242</td><td>0</td><td>PREDICTED: uncharacterized protein C15orf39 homolog isoform X1 [Bos taurus]</td><td>gi|983003774|ref|XM_001252069.6|</td><td>4330</td><td>0</td><td>PREDICTED: Bos taurus chromosome 21 open reading frame, human C15orf39 (C21H15orf39), transcript variant X1, mRNA</td><td>Q3TEI4</td><td>1190</td><td>0</td><td>Uncharacterized protein C15orf39 homolog</td><td>.</td><td>.</td><td>.</td><td>.</td><td>pfam03154</td><td>Atrophin-1</td><td>Atrophin-1 family. Atrophin-1 is the protein product of the dentatorubral-pallidoluysian atrophy (DRPLA) gene. DRPLA</td><td>.</td><td>GO:0005737|cytoplasm;</td><td>.</td><td>.</td><td>.</td></tr>
<tr><td>LGALS3BP</td><td>2818.899</td><td>0.507138</td><td>0.109138221</td><td>4.646754</td><td>3.372E-06</td><td>0.000359</td><td>Not_Sig</td><td>no</td><td>gi|942098736|ref|XP_014338632.1|</td><td>2922</td><td>0</td><td>PREDICTED: galectin-3-binding protein isoform X1 [Bos mutus]</td><td>gi|942098738|ref|XM_005910317.2|</td><td>2418</td><td>0</td><td>PREDICTED: Bos mutus lectin, galactoside-binding, soluble, 3 binding protein (LGALS3BP), transcript variant X2, mRNA</td><td>A7E3W2</td><td>1105</td><td>0</td><td>Galectin-3-binding protein</td><td>.</td><td>.</td><td>.</td><td>.</td><td>pfam00530</td><td>SRCR</td><td>Scavenger receptor cysteine-rich domain. These domains are disulphide rich extracellular domains. These domains are found</td><td>GO:0007155|cell adhesion;</td><td>GO:0072562|blood microparticle;GO:0070062|extracellular exosome;GO:0016020|membrane;GO:0005578|proteinaceous extracellular matrix;</td><td>GO:0005044|scavenger receptor activity;</td><td>K17300</td><td>LGALS3BP; galectin-3-binding protein</td></tr>
<tr><td>LOC138987806</td><td>510.4337</td><td>-0.53292</td><td>0.1147026</td><td>-4.64614</td><td>3.382E-06</td><td>0.000359</td><td>Not_Sig</td><td>no</td><td>gi|4239700|emb|CAA10770.1|</td><td>380</td><td>4e-36</td><td>reverse transcriptase-like [Bos taurus]</td><td>gi|850480667|gb|CP011888.1|</td><td>1476</td><td>0</td><td>Ovis canadensis canadensis isolate 43U chromosome 3 sequence</td><td>O00370</td><td>85.5</td><td>3e-15</td><td>LINE-1 retrotransposable element ORF2 protein</td><td>.</td><td>.</td><td>.</td><td>.</td><td>.</td><td>.</td><td>.</td><td>GO:0006310|DNA recombination;GO:0090305|nucleic acid phosphodiester bond hydrolysis;GO:0032199|reverse transcription involved in RNA-mediated transposition;GO:0032197|transposition, RNA-mediated;</td><td>.</td><td>GO:0046872|metal ion binding;GO:0003964|RNA-directed DNA</td><td>.</td><td>.</td></tr>
<tr><td>OGDH</td><td>2053.768</td><td>0.537647</td><td>0.115784356</td><td>4.643519</td><td>3.425E-06</td><td>0.000359</td><td>Not_Sig</td><td>no</td><td>gi|555974944|ref|XP_005899459.1|</td><td>5511</td><td>0</td><td>PREDICTED: 2-oxoglutarate dehydrogenase, mitochondrial isoform X1 [Bos mutus]</td><td>gi|982921876|ref|XM_005205596.3|</td><td>4137</td><td>0</td><td>PREDICTED: Bos taurus oxoglutarate (alpha-ketoglutarate) dehydrogenase (lipoamide) (OGDH), transcript variant X1, mRNA</td><td>Q148N0</td><td>2087</td><td>0</td><td>2-oxoglutarate dehydrogenase, mitochondrial</td><td>SPBC3H7.03c</td><td>870</td><td>0</td><td>COG0567</td><td>pfam00676</td><td>E1_dh</td><td>Dehydrogenase E1 component. This family uses thiamine pyrophosphate as a cofactor. This family includes pyruvate</td><td>GO:0021695|cerebellar cortex development;GO:0006096|glycolytic process;GO:0021766|hippocampus development;GO:0061034|olfactory bulb mitral cell layer development;GO:0021860|pyramidal neuron development;GO:0021756|striatum development;GO:0022028|tangential migration from the subventricular zone to the olfactory bulb;GO:0021794|thalamus development;GO:0006099|tricarboxylic acid cycle;</td><td>GO:0005759|mitochondrial matrix;GO:0005739|mitochondrion;GO:0045252|oxoglutarate dehydrogenase complex;</td><td>GO:0046872|metal ion binding;GO:0034602|oxoglutarate</td><td>K00164</td><td>OGDH, sucA; 2-oxoglutarate dehydrogenase E1 component</td></tr>
<tr><td>PLXNA1</td><td>1659.826</td><td>0.603125</td><td>0.129899066</td><td>4.643026</td><td>3.433E-06</td><td>0.000359</td><td>UP</td><td>yes</td><td>gi|983004867|ref|XP_015324309.1|</td><td>9663</td><td>0</td><td>PREDICTED: LOW QUALITY PROTEIN: plexin-A1 isoform X2 [Bos taurus]</td><td>gi|983004866|ref|XM_015468823.1|</td><td>8549</td><td>0</td><td>PREDICTED: Bos taurus plexin A1 (PLXNA1), transcript variant X2, mRNA</td><td>Q9UIW2</td><td>3594</td><td>0</td><td>Plexin-A1</td><td>.</td><td>.</td><td>.</td><td>.</td><td>pfam08337</td><td>Plexin_cytopl</td><td>Plexin cytoplasmic RasGAP domain. This family features the C-terminal regions of various plexins. Plexins are receptors for</td><td>GO:0021785|branchiomotor neuron axon guidance;GO:0060666|dichotomous subdivision of terminal units involved in salivary gland branching;GO:0007275|multicellular organismal development;GO:1990138|neuron projection extension;GO:0048841|regulation of axon extension involved in axon guidance;GO:0030334|regulation of cell migration;GO:0014910|regulation of smooth muscle cell migration;GO:1902287|semaphorin-plexin signaling pathway involved in axon guidance;</td><td>GO:0005737|cytoplasm;GO:0070062|extracellular exosome;GO:0005887|integral component of plasma membrane;GO:0005634|nucleus;GO:00058</td><td>GO:0004872|receptor activity;GO:0017154|semaphorin</td><td>K06820</td><td>PLXNA; plexin A</td></tr>
<tr><td>RCN3</td><td>3583.966</td><td>0.533632</td><td>0.115001982</td><td>4.640201</td><td>3.481E-06</td><td>0.000359</td><td>Not_Sig</td><td>no</td><td>gi|555986386|ref|XP_005905057.1|</td><td>1685</td><td>0</td><td>PREDICTED: reticulocalbin-3 [Bos mutus]</td><td>gi|742163364|ref|XM_010849622.1|</td><td>1499</td><td>0</td><td>PREDICTED: Bison bison bison reticulocalbin 3, EF-hand calcium binding domain (RCN3), mRNA</td><td>Q2KJ39</td><td>650</td><td>0</td><td>Reticulocalbin-3</td><td>.</td><td>.</td><td>.</td><td>.</td><td>pfam13499</td><td>EF-hand_7</td><td>EF-hand domain pair.</td><td>.</td><td>GO:0005788|endoplasmic reticulum lumen;</td><td>GO:0005509|calcium ion binding;</td><td>.</td><td>.</td></tr>
<tr><td>SKIL</td><td>723.6147</td><td>-0.68634</td><td>0.147936477</td><td>-4.63943</td><td>3.494E-06</td><td>0.000359</td><td>DOWN</td><td>yes</td><td>gi|555959357|ref|XP_005891789.1|</td><td>3466</td><td>0</td><td>PREDICTED: ski-like protein isoform X2 [Bos mutus]</td><td>gi|742098234|ref|XM_010835234.1|</td><td>7084</td><td>0</td><td>PREDICTED: Bison bison bison SKI-like proto-oncogene (SKIL), transcript variant X3, mRNA</td><td>Q5R431</td><td>1213</td><td>0</td><td>Ski-like protein</td><td>.</td><td>.</td><td>.</td><td>.</td><td>pfam08782</td><td>c-SKI_SMAD_bind</td><td>c-SKI Smad4 binding domain. c-SKI is an oncoprotein that inhibits TGF-beta signaling through interaction with Smad proteins. This domain</td><td>GO:0000122|negative regulation of transcription from RNA polymerase II promoter;GO:0030512|negative regulation of transforming growth factor beta receptor signaling pathway;</td><td>.</td><td>.</td><td>K18499</td><td>SKIL; Ski-like protein</td></tr>
<tr><td>VASN</td><td>1947.299</td><td>0.714287</td><td>0.153978475</td><td>4.638874</td><td>3.503E-06</td><td>0.000359</td><td>UP</td><td>yes</td><td>gi|139949116|ref|NP_001077265.1|</td><td>2467</td><td>0</td><td>vasorin precursor [Bos taurus]</td><td>gi|139949115|ref|NM_001083796.1|</td><td>2596</td><td>0</td><td>Bos taurus vasorin (VASN), mRNA</td><td>Q6EMK4</td><td>787</td><td>0</td><td>Vasorin</td><td>SPy1798</td><td>52</td><td>8.50e-06</td><td>COG4886</td><td>pfam13855</td><td>LRR_8</td><td>Leucine rich repeat.</td><td>GO:0071456|cellular response to hypoxia;GO:0071461|cellular response to redox state;GO:0010719|negative regulation of epithelial to mesenchymal transition;GO:0030512|negative regulation of transforming growth factor beta receptor signaling pathway;</td><td>GO:0009986|cell surface;GO:0005913|cell-cell adherens junction;GO:0070062|extracellular exosome;GO:0005615|extracellular</td><td>GO:0098641|cadherin binding involved in cell-cell adhesion;GO:00504</td><td>.</td><td>.</td></tr>
<tr><td>FOXRED2</td><td>70.06583</td><td>1.094947</td><td>0.236747357</td><td>4.624962</td><td>3.747E-06</td><td>0.000381</td><td>UP</td><td>yes</td><td>gi|742205008|ref|XP_010858843.1|</td><td>3365</td><td>0</td><td>PREDICTED: FAD-dependent oxidoreductase domain-containing protein 2 isoform X1 [Bison bison bison]</td><td>gi|742205007|ref|XM_010860541.1|</td><td>2578</td><td>0</td><td>PREDICTED: Bison bison bison FAD-dependent oxidoreductase domain containing 2 (FOXRED2), transcript variant X1, mRNA</td><td>Q8IWF2</td><td>1093</td><td>0</td><td>FAD-dependent oxidoreductase domain-containing protein 2</td><td>mll7934</td><td>59.3</td><td>2.33e-08</td><td>COG2072</td><td>pfam13738</td><td>Pyr_redox_3</td><td>Pyridine nucleotide-disulphide oxidoreductase.</td><td>GO:0030433|ER-associated ubiquitin-dependent protein catabolic process;GO:0055114|oxidation-reduction process;</td><td>GO:0005788|endoplasmic reticulum lumen;</td><td>GO:0050660|flavin adenine dinucleotide binding;GO:0001948</td><td>.</td><td>.</td></tr>
<tr><td>AP1M1</td><td>1145.716</td><td>0.597616</td><td>0.129296742</td><td>4.622049</td><td>3.8E-06</td><td>0.000383</td><td>UP</td><td>yes</td><td>gi|431921951|gb|ELK19124.1|</td><td>2258</td><td>0</td><td>AP-1 complex subunit mu-1 [Pteropus alecto]</td><td>gi|164420747|ref|NM_001045884.2|</td><td>2199</td><td>0</td><td>Bos taurus adaptor related protein complex 1 mu 1 subunit (AP1M1), mRNA</td><td>Q9BXS5</td><td>873</td><td>0</td><td>AP-1 complex subunit mu-1</td><td>.</td><td>.</td><td>.</td><td>.</td><td>pfam00928</td><td>Adap_comp_sub</td><td>Adaptor complexes medium subunit family. This family also contains members which are coatomer subunits.</td><td>GO:0019886|antigen processing and presentation of exogenous peptide antigen via MHC class II;GO:0035646|endosome to melanosome transport;GO:0006886|intracellular protein transport;GO:0032438|melanosome organization;GO:0050690|regulation of defense response to virus by virus;GO:0016192|vesicle-mediated transport;GO:0016032|viral process;</td><td>GO:0030131|clathrin adaptor complex;GO:0030669|clathrin-coated endocytic vesicle membrane;GO:0030659|cytoplasmic vesicle</td><td>.</td><td>K12393</td><td>AP1M; AP-1 complex subunit mu</td></tr>
<tr><td>DYNC1H1</td><td>8365.185</td><td>0.431807</td><td>0.093514229</td><td>4.61755</td><td>3.883E-06</td><td>0.000388</td><td>Not_Sig</td><td>no</td><td>gi|329663701|ref|NP_001193067.1|</td><td>24402</td><td>0</td><td>cytoplasmic dynein 1 heavy chain 1 [Bos taurus]</td><td>gi|329663700|ref|NM_001206138.1|</td><td>14117</td><td>0</td><td>Bos taurus dynein, cytoplasmic 1, heavy chain 1 (DYNC1H1), mRNA</td><td>Q14204</td><td>9339</td><td>0</td><td>Cytoplasmic dynein 1 heavy chain 1</td><td>YKR054c</td><td>1848</td><td>0</td><td>COG5245</td><td>pfam03028</td><td>Dynein_heavy</td><td>Dynein heavy chain and region D6 of dynein motor. This family represents the C-terminal region of dynein heavy chain. The chain</td><td>GO:0019886|antigen processing and presentation of exogenous peptide antigen via MHC class II;GO:0033962|cytoplasmic mRNA processing body assembly;GO:0006888|ER to Golgi vesicle-mediated transport;GO:0051293|establishment of spindle localization;GO:0000086|G2/M transition of mitotic cell cycle;GO:0007018|microtubule-based movement;GO:0007052|mitotic spindle organization;GO:0034063|stress granule assembly;</td><td>GO:0005813|centrosome;GO:0005868|cytoplasmic dynein complex;GO:0005829|cytosol;GO:0070062|extracellular</td><td>GO:0005524|ATP binding;GO:0016887|ATPase activity;GO:0051959</td><td>K10413</td><td>DYNC1H; dynein heavy chain 1, cytosolic</td></tr>
<tr><td>SP2</td><td>255.901</td><td>0.889354</td><td>0.192856186</td><td>4.611486</td><td>3.998E-06</td><td>0.000395</td><td>UP</td><td>yes</td><td>gi|62751781|ref|NP_001015654.1|</td><td>2471</td><td>0</td><td>transcription factor Sp2 [Bos taurus]</td><td>gi|62751780|ref|NM_001015654.1|</td><td>2242</td><td>0</td><td>Bos taurus Sp2 transcription factor (SP2), mRNA</td><td>Q5E9U0</td><td>956</td><td>0</td><td>Transcription factor Sp2</td><td>.</td><td>.</td><td>.</td><td>.</td><td>pfam13465</td><td>zf-H2C2_2</td><td>Zinc-finger double domain.</td><td>GO:0035264|multicellular organism growth;GO:0006357|regulation of transcription from RNA polymerase II promoter;GO:0006351|transcription, DNA-templated;</td><td>GO:0005634|nucleus;</td><td>GO:0003677|DNA binding;GO:0046872|metal ion binding;GO:0000981</td><td>K09192</td><td>SP2; transcription factor Sp2</td></tr>
</table>

ASF1B	358.1353	0.648239	0.140605327	4.610344	4.02E-06	0.000395	UP	yes	gi|115495803|ref|NP_001068921.1|	1069	1e-139	histone chaperone ASF1B [Bos taurus]	gi|742184768|ref|XM_010855438.1|	1878	0	PREDICTED: Bison bison bison anti-silencing function 1B histone chaperone (ASF1B), mRNA	Q17QJ0	416	4e-141	Histone chaperone ASF1B	YJL115w	195	5.18e-57	COG5137	pfam04729	ASF1_hist_chap	ASF1 like histone chaperone. This family includes the yeast and human ASF1 protein. These proteins have histone chaperone	GO:0016569|covalent chromatin modification;GO:0006335|DNA replication-dependent nucleosome assembly;GO:0006336|DNA replication-independent nucleosome assembly;GO:0006355|regulation of transcription, DNA-templated;GO:0006351|transcription, DNA-templated;	GO:0000790|nuclear chromatin;GO:0005654|nucleoplasm;GO:0043234|protein complex;	.	K10753	ASF1; histone chaperone ASF1
TAF13	497.3204	-0.76384	0.166070621	-4.5995	4.235E-06	0.000412	DOWN	yes	gi|677462861|gb|KFQ52275.1|	515	5e-60	Transcription initiation factor TFIID subunit 13, partial [Pelecanus crispus]	gi|742232483|ref|XM_010830926.1|	1513	0	PREDICTED: Bison bison bison TAF13 RNA polymerase II, TATA box binding protein (TBP)-associated factor, 18kDa (TAF13), mRNA	Q5R9W6	202	4e-61	Transcription initiation factor TFIID subunit 13	SPCC1494.02c	80.1	2.61e-17	COG5248	pfam02269	TFIID-18kDa	Transcription initiation factor IID, 18kD subunit. This family includes the Spt3 yeast transcription factors and the 18kD subunit from human	GO:0006355|regulation of transcription, DNA-templated;GO:0006366|transcription from RNA polymerase II promoter;	GO:0005634|nucleus;	.	K03127	TAF13; transcription initiation factor TFIID subunit 13
ATN1	1641.599	0.759607	0.165304671	4.595195	4.323E-06	0.000416	UP	yes	gi|970730587|ref|XP_015100397.1|	1533	0	PREDICTED: atrophin-1 isoform X4 [Vicugna pacos]	gi|329664667|ref|NM_001205748.1|	4396	0	Bos taurus atrophin 1 (ATN1), mRNA	Q5IS70	591	0	Atrophin-1	.	.	.	.	pfam03154	Atrophin-1	Atrophin-1 family. Atrophin-1 is the protein product of the dentatorubral-pallidoluysian atrophy (DRPLA) gene. DRPLA	GO:0006355|regulation of transcription, DNA-templated;GO:0006351|transcription, DNA-templated;	GO:0030054|cell junction;GO:0005634|nucleus;GO:0048471|perinuclear region of cytoplasm;	.	K05626	DRPLA; atrophin-1 (dentatorubral-pallidoluysian atrophy protein)
CUEDC1	640.841	0.646906	0.140816597	4.593958	4.349E-06	0.000416	UP	yes	gi|528994043|ref|XP_005219965.1|	1606	0	PREDICTED: CUE domain-containing protein 1 isoform X1 [Bos taurus]	gi|982955905|ref|XM_005219908.3|	1320	0	PREDICTED: Bos taurus CUE domain containing 1 (CUEDC1), transcript variant X1, mRNA	Q9NWM3	550	0	CUE domain-containing protein 1	.	.	.	.	pfam02845	CUE	CUE domain. CUE domains have been shown to bind ubiquitin. It has been suggested that CUE domains are related to pfam00627	.	.	.	.	.
SF3A1	1378.507	0.499572	0.10884592	4.589713	4.439E-06	0.000421	Not_Sig	no	gi|560982692|ref|XP_006213573.1|	2920	0	PREDICTED: splicing factor 3A subunit 1 [Vicugna pacos]	gi|942031982|ref|XM_005887514.2|	2908	0	PREDICTED: Bos mutus splicing factor 3a, subunit 1, 120kDa (SF3A1), transcript variant X1, mRNA	A2VDN6	1129	0	Splicing factor 3A subunit 1	.	.	.	.	pfam12230	PRP21_like_P	Pre-mRNA splicing factor PRP21 like protein. This domain family is found in eukaryotes, and is typically between 212 and 238	GO:0000389|mRNA 3'-splice site recognition;GO:0000398|mRNA splicing, via spliceosome;GO:0000381|regulation of alternative mRNA splicing, via spliceosome;	GO:0071013|catalytic step 2 spliceosome;GO:0005654|nucleoplasm;GO:0005686|U2 snRNP;GO:0071004|U2-type prespliceosome;GO:0005684|U2-type	GO:0044822|poly(A) RNA binding;GO:0003723|RNA binding;	K12825	SF3A1, SAP114; splicing factor 3A subunit 1
DR1	1101.025	-0.53034	0.115806284	-4.57959	4.659E-06	0.000435	Not_Sig	no	gi|224057416|ref|XP_002192825.1|	606	3e-68	PREDICTED: protein Dr1 [Taeniopygia guttata]	gi|965862359|ref|XM_012145698.2|	4227	0	PREDICTED: Ovis aries musimon down-regulator of transcription 1 (DR1), mRNA	Q91WV0	238	1e-69	Protein Dr1	SPBC30D10.02	111	2.83e-27	COG5150	pfam00808	CBFD_NFYB_HMF	Histone-like transcription factor (CBF/NF-Y) and archaeal histone. This family includes archaebacterial histones and	GO:0043966|histone H3 acetylation;GO:0006355|regulation of transcription, DNA-templated;GO:0006351|transcription, DNA-templated;	GO:0005671|Ada2/Gcn5/Ada3 transcription activator complex;GO:0005634|nucleus;	GO:0003677|DNA binding;GO:0017025|TBP-class protein binding;	.	.
GLIS2	1106.994	0.751467	0.164133537	4.578391	4.686E-06	0.000435	UP	yes	gi|329664222|ref|NP_001192877.1|	1548	0	zinc finger protein GLIS2 [Bos taurus]	gi|982966166|ref|XM_005224485.3|	3543	0	PREDICTED: Bos taurus GLIS family zinc finger 2 (GLIS2), transcript variant X1, mRNA	Q9BZE0	570	0	Zinc finger protein GLIS2	.	.	.	.	pfam13465	zf-H2C2_2	Zinc-finger double domain.	GO:0061005|cell differentiation involved in kidney development;GO:0043433|negative regulation of sequence-specific DNA binding transcription factor activity;GO:0045879|negative regulation of smoothened signaling pathway;GO:0000122|negative regulation of transcription from RNA polymerase II promoter;GO:0045892|negative regulation of transcription, DNA-templated;GO:0007399|nervous system development;GO:1900182|positive regulation of protein localization to nucleus;GO:0045944|positive regulation of transcription from	GO:0005737|cytoplasm;GO:0031513|nonmotile primary cilium;GO:0016607|nuclear speck;GO:0005634|nucleus;	GO:0046872|metal ion binding;GO:0000977|RNA polymerase II	K09233	GLIS2; zinc finger protein GLIS2
RHOQ	3548.001	-0.45322	0.099001751	-4.57793	4.696E-06	0.000435	Not_Sig	no	gi|395731858|ref|XP_002812107.2|	1141	1e-142	PREDICTED: rho-related GTP-binding protein RhoQ [Pongo abelii]	gi|942054090|ref|XM_005895614.2|	3672	0	PREDICTED: Bos mutus ras homolog family member Q (RHOQ), mRNA	P17081	425	3e-137	Rho-related GTP-binding protein RhoQ	SPAC110.03	259	1.21e-78	COG1100	pfam00071	Ras	Ras family. Includes sub-families Ras, Rab, Rac, Ral, Ran, Rap Ypt1 and more. Shares P-loop motif with GTP_EFTU, arf and	GO:0032869|cellular response to insulin stimulus;GO:0030866|cortical actin cytoskeleton organization;GO:0046039|GTP metabolic process;GO:0008286|insulin receptor signaling pathway;GO:0090005|negative regulation of establishment of protein localization to plasma membrane;GO:0051491|positive regulation of filopodium assembly;GO:0046326|positive regulation of glucose import;GO:0045944|positive regulation of transcription from RNA polymerase II promoter;GO:0032956|regulation of actin cytoskeleton	GO:0005884|actin filament;GO:0005829|cytosol;GO:0070062|extracellular exosome;GO:0030660|Golgi-associated vesicle	GO:0032427|GBD domain binding;GO:0005525|GTP	K07194	RHOQ, TC10; Ras homolog gene family, member Q
ACTN4	19225.89	0.52725	0.115225697	4.575803	4.744E-06	0.000436	Not_Sig	no	gi|594090713|ref|XP_006068757.1|	4938	0	PREDICTED: alpha-actinin-4 isoform X1 [Bubalus bubalis]	gi|942098334|ref|XM_005910148.2|	3864	0	PREDICTED: Bos mutus actinin, alpha 4 (ACTN4), mRNA	A5D7D1	1821	0	Alpha-actinin-4	SPAC15A10.08	259	2.05e-73	COG5069	pfam08726	EFhand_Ca_insen	Ca2+ insensitive EF hand. EF hands are helix-loop-helix binding motifs involved in the regulation of many cellular processes. EF hands	GO:0051017|actin filament bundle assembly;GO:0070830|bicellular tight junction assembly;GO:0051271|negative regulation of cellular component movement;GO:1900025|negative regulation of substrate adhesion-dependent cell spreading;GO:0035357|peroxisome proliferator activated receptor signaling pathway;GO:0030335|positive regulation of cell migration;GO:1901224|positive regulation of NIK/NF-kappaB signaling;GO:0048549|positive regulation of pinocytosis;GO:1902396|protein localization to bicellular tight junction;GO:0015031|protein	GO:0005903|brush border;GO:0005911|cell-cell junction;GO:0030863|cortical cytoskeleton;GO:0005737|cytoplasm;GO:00	GO:0005509|calcium ion binding;GO:0031490|chromatin DNA	K05699	ACTN; actinin alpha
CAV2	814.7787	-0.711	0.155554308	-4.57073	4.86E-06	0.000441	DOWN	yes	gi|56119130|ref|NP_001007809.1|	843	1e-103	caveolin-2 [Bos taurus]	gi|942064456|ref|XM_005899129.2|	2622	0	PREDICTED: Bos mutus caveolin 2 (CAV2), transcript variant X1, mRNA	Q66WT7	329	3e-105	Caveolin-2	.	.	.	.	pfam01146	Caveolin	Caveolin. All three known Caveolin forms have the FEDVIAEP caveolin 'signature motif' within their hydrophilic N-terminal domain.	GO:0070836|caveola assembly;GO:0007029|endoplasmic reticulum organization;GO:0008286|insulin receptor signaling pathway;GO:0007005|mitochondrion organization;GO:0001937|negative regulation of endothelial cell proliferation;GO:0030512|negative regulation of transforming growth factor beta receptor signaling pathway;GO:0044791|positive regulation by host of viral release from host cell;GO:0060161|positive regulation of dopamine receptor signaling pathway;GO:0001938|positive regulation of endothelial cell	GO:0002080|acrosomal membrane;GO:0005901|caveola;GO:0031234|extrinsic component of cytoplasmic side of plasma membrane;GO:0005925|focal	GO:0031748|D1 dopamine receptor binding;	K12958	CAV2; caveolin 2
ZC3H4	598.4912	0.53034	0.116048501	4.569986	4.878E-06	0.000441	Not_Sig	no	gi|329663751|ref|NP_001192818.1|	2296	0	zinc finger CCCH domain-containing protein 4 [Bos taurus]	gi|982953541|ref|XM_005219286.3|	6205	0	PREDICTED: Bos taurus zinc finger CCCH-type containing 4 (ZC3H4), transcript variant X3, mRNA	Q9UPT8	799	0	Zinc finger CCCH domain-containing protein 4	ECU10g1505	50.8	1.84e-06	COG5084	pfam10446	DUF2457	Protein of unknown function (DUF2457). This is a family of uncharacterized proteins.	.	.	GO:0046872|metal ion binding;GO:0044822|poly(A) RNA	.	.
BCAR1	1138.373	0.645985	0.141577939	4.562752	5.049E-06	0.000453	UP	yes	gi|594079467|ref|XP_006063401.1|	3467	0	PREDICTED: breast cancer anti-estrogen resistance protein 1 isoform X5 [Bubalus bubalis]	gi|331999982|ref|NM_001206698.1|	2971	0	Bos taurus breast cancer anti-estrogen resistance 1 (BCAR1), mRNA	P56945	1178	0	Breast cancer anti-estrogen resistance protein 1	.	.	.	.	pfam12026	DUF3513	Domain of unknown function (DUF3513). This presumed domain is functionally uncharacterized. This domain is found in	GO:0007015|actin filament organization;GO:0050851|antigen receptor-mediated signaling pathway;GO:0050853|B cell receptor signaling pathway;GO:0007155|cell adhesion;GO:0060326|cell chemotaxis;GO:0051301|cell division;GO:0016477|cell migration;GO:0008283|cell proliferation;GO:0035729|cellular response to hepatocyte growth factor stimulus;GO:0007173|epidermal growth factor receptor signaling pathway;GO:0007186|G-protein coupled receptor signaling pathway;GO:0048012|hepatocyte growth factor receptor signaling	GO:0015629|actin cytoskeleton;GO:0005737|cytoplasm;GO:0005829|cytosol;GO:0005925|focal adhesion;GO:0030027|lamellipodium;GO:00	GO:0019901|protein kinase binding;GO:0004871|signal transducer	K05726	BCAR1, CAS; breast cancer anti-estrogen resistance 1
FBLN1	1368.181	0.549362	0.120549101	4.557164	5.185E-06	0.000462	Not_Sig	no	gi|296486900|tpg|DAA29013.1|	3405	0	TPA: fibulin 1 [Bos taurus]	gi|742108739|ref|XM_010837612.1|	2672	0	PREDICTED: Bison bison bison fibulin 1 (FBLN1), transcript variant X1, mRNA	P23142	1191	0	Fibulin-1	.	.	.	.	pfam14670	FXa_inhibition	Coagulation Factor Xa inhibitory site. This short domain on coagulation enzyme factor Xa is found to be the target for a potent	GO:0072378|blood coagulation, fibrin clot formation;GO:0007566|embryo implantation;GO:0030198|extracellular matrix organization;GO:0007162|negative regulation of cell adhesion;GO:2000146|negative regulation of cell motility;GO:0070373|negative regulation of ERK1 and ERK2 cascade;GO:0001933|negative regulation of protein phosphorylation;GO:2000647|negative regulation of stem cell proliferation;GO:1900025|negative regulation of substrate adhesion-dependent cell spreading;GO:1904188|negative regulation of	GO:0005604|basement membrane;GO:0071953|elastic fiber;GO:0070062|extracellular exosome;GO:0005576|extracellular	GO:0005509|calcium ion binding;GO:0005201|extracellular matrix	K17307	FBLN1_2; fibulin 1/2
SLC2A13	387.5895	-0.65643	0.1441543	-4.55367	5.272E-06	0.000466	DOWN	yes	gi|300794344|ref|NP_001179892.1|	3044	0	proton myo-inositol cotransporter [Bos taurus]	gi|850480667|gb|CP011888.1|	7391	0	Ovis canadensis canadensis isolate 43U chromosome 3 sequence	Q96QE2	1150	0	Proton myo-inositol cotransporter	.	.	.	.	pfam00083	Sugar_tr	Sugar (and other) transporter.	GO:0046323|glucose import;GO:0035428|hexose transmembrane transport;	GO:0005887|integral component of plasma membrane;GO:0005886|plasma membrane;	GO:0005355|glucose transmembrane transporter activity;GO:0005366	K08150	SLC2A13, ITR; MFS transporter, SP family, solute carrier family 2 (myo-
NFIC	3282.511	0.668436	0.146881888	4.550838	5.343E-06	0.000469	UP	yes	gi|803270238|ref|XP_011994850.1|	2261	0	PREDICTED: nuclear factor 1 C-type isoform X5 [Ovis aries musimon]	gi|594047082|ref|XM_006047906.1|	3936	0	PREDICTED: Bubalus bubalis nuclear factor I/C (CCAAT-binding transcription factor) (NFIC), mRNA	P21999	867	0	Nuclear factor 1 C-type	.	.	.	.	pfam00859	CTF_NFI	CTF/NF-I family transcription modulation region.	GO:0006260|DNA replication;GO:0000122|negative regulation of transcription from RNA polymerase II promoter;GO:0045944|positive regulation of transcription from RNA polymerase II promoter;GO:0006351|transcription, DNA-templated;	GO:0005634|nucleus;	GO:0003677|DNA binding;GO:0000981|RNA polymerase II transcription factor	.	.
NRAS	1415.85	-0.53532	0.117733865	-4.54688	5.445E-06	0.000474	Not_Sig	no	gi|332237751|ref|XP_003268071.1|	985	6e-120	PREDICTED: GTPase NRas isoform X1 [Nomascus leucogenys]	gi|555949939|ref|XM_005887113.1|	4252	0	PREDICTED: Bos mutus neuroblastoma RAS viral (v-ras) oncogene homolog (NRAS), mRNA	Q2MJK3	370	8e-117	GTPase NRas	SPAC17H9.09c	206	1.44e-59	COG1100	pfam00071	Ras	Ras family. Includes sub-families Ras, Rab, Rac, Ral, Ran, Rap Ypt1 and more. Shares P-loop motif with GTP_EFTU, arf and	GO:0072600|establishment of protein localization to Golgi;GO:0007264|small GTPase mediated signal transduction;	GO:0070062|extracellular exosome;GO:0000139|Golgi membrane;GO:0005886|plasma membrane;	GO:0005525|GTP binding;	K07828	NRAS; GTPase NRas
NME1	367.9648	-0.72145	0.158972939	-4.53821	5.673E-06	0.000487	DOWN	yes	gi|35068|emb|CAA35621.1|	820	9e-108	Nm23 protein [Homo sapiens]	gi|942040067|ref|XM_005890266.2|	807	0	PREDICTED: Bos mutus NME/NM23 nucleoside diphosphate kinase 1 (NME1), transcript variant X1, mRNA	P52175	317	3e-108	Nucleoside diphosphate kinase A 2	YKL067w	197	1.68e-63	COG0105	pfam00334	NDK	Nucleoside diphosphate kinase.	GO:0030154|cell differentiation;GO:0006241|CTP biosynthetic process;GO:0006897|endocytosis;GO:0006183|GTP biosynthetic process;GO:0007399|nervous system development;GO:0009142|nucleoside triphosphate biosynthetic process;GO:0006163|purine nucleotide metabolic process;GO:0006220|pyrimidine nucleotide metabolic process;GO:0042981|regulation of apoptotic process;GO:0006228|UTP biosynthetic process;	GO:0005737|cytoplasm;GO:0005622|intracellular;GO:0005634|nucleus;GO:0005886|plasma membrane;	GO:0005524|ATP binding;GO:0046872|metal ion binding;GO:0004550	.	.
SH3BGRL	515.4771	-0.70162	0.154613787	-4.53787	5.682E-06	0.000487	DOWN	yes	gi|507572137|ref|XP_004669663.1|	481	8e-54	PREDICTED: SH3 domain-binding glutamic acid-rich-like protein [Jaculus jaculus]	gi|594103937|ref|XM_006074905.1|	1739	0	PREDICTED: Bubalus bubalis SH3 domain binding glutamic acid-rich protein like (SH3BGRL), transcript variant X1, mRNA	Q5RFN7	187	1e-54	SH3 domain-binding glutamic acid-rich-like protein	.	.	.	.	pfam04908	SH3BGR	SH3-binding, glutamic acid-rich protein.	.	GO:0070062|extracellular exosome;GO:0005615|extracellular space;	.	.	.
SLC7A11	481.4081	-0.6719	0.148328697	-4.52978	5.905E-06	0.000499	DOWN	yes	gi|555967703|ref|XP_005895896.1|	2456	0	PREDICTED: cystine/glutamate transporter isoform X1 [Bos mutus]	gi|850491151|gb|CP011902.1|	3724	0	Ovis canadensis canadensis isolate 43U chromosome 17 sequence	Q5RAG7	892	0	Cystine/glutamate transporter	BS_ykbA	150	1.89e-37	COG0531	pfam13520	AA_permease_2	Amino acid permease.	.	GO:0016021|integral component of membrane;	GO:0015171|amino acid transmembrane transporter activity;	K13869	SLC7A11; solute carrier family 7 (L-type amino acid transporter),
PRAG1	70.63112	1.118938	0.247089976	4.528466	5.941E-06	0.000499	UP	yes	gi|440895747|gb|ELR47864.1|	6598	0	Tyrosine-protein kinase SgK223 [Bos mutus]	gi|983008566|ref|XM_015469735.1|	4684	0	PREDICTED: Bos taurus tyrosine-protein kinase SgK223 (LOC533821), transcript variant X2, mRNA	Q86YV5	1902	0	Tyrosine-protein kinase SgK223	.	.	.	.	pfam00069	Pkinase	Protein kinase domain.	.	.	GO:0005524|ATP binding;GO:0004715|non-membrane spanning protein	K17537	SGK223; tyrosine-protein kinase SgK223 [EC:2.7.10.2]
PCSK6	196.6455	0.718083	0.1585781	4.528259	5.947E-06	0.000499	UP	yes	gi|926719214|ref|XP_013828480.1|	4679	0	PREDICTED: proprotein convertase subtilisin/kexin type 6 isoform X1 [Capra hircus]	gi|942040528|ref|XM_014476602.1|	3835	0	PREDICTED: Bos mutus proprotein convertase subtilisin/kexin type 6 (PCSK6), mRNA	P29122	1698	0	Proprotein convertase subtilisin/kexin type 6	YNL238w_1	280	3.40e-82	COG1404	pfam00082	Peptidase_S8	Subtilase family. Subtilases are a family of serine proteases. They appear to have independently and convergently evolved an	GO:0007368|determination of left/right symmetry;GO:0009100|glycoprotein metabolic process;GO:0032455|nerve growth factor processing;GO:0032902|nerve growth factor production;GO:0016486|peptide hormone processing;GO:0016485|protein processing;GO:0030510|regulation of BMP signaling pathway;GO:0032940|secretion by cell;GO:0007354|zygotic determination of anterior/posterior axis, embryo;	GO:0009986|cell surface;GO:0005783|endoplasmic reticulum;GO:0031012|extracellular matrix;GO:0005615|extracellular	GO:0004175|endopeptidase activity;GO:0008201|heparin	K08672	PCSK6; proprotein convertase subtilisin/kexin type 6 [EC:3.4.21.-]
ABCA3	273.7679	0.752096	0.166218539	4.524744	6.047E-06	0.000503	UP	yes	gi|741973463|ref|XP_010817370.1|	8612	0	PREDICTED: ATP-binding cassette sub-family A member 3 isoform X1 [Bos taurus]	gi|982966040|ref|XM_010819068.2|	6457	0	PREDICTED: Bos taurus ATP binding cassette subfamily A member 3 (ABCA3), transcript variant X1, mRNA	Q99758	2962	0	ATP-binding cassette sub-family A member 3	MA0868	176	1.56e-48	COG1131	pfam00005	ABC_tran	ABC transporter. ABC transporters for a large family of proteins responsible for translocation of a variety of compounds across	GO:0044267|cellular protein metabolic process;GO:0006869|lipid transport;GO:0042493|response to drug;GO:0051384|response to glucocorticoid;GO:0055085|transmembrane transport;GO:0006810|transport;	GO:0097208|alveolar lamellar body;GO:0097233|alveolar lamellar body membrane;GO:0005615|extracellular space;GO:0016021|integral component of	GO:0005524|ATP binding;GO:0042626|ATPase activity, coupled to	K05643	ABCA3; ATP-binding cassette, subfamily A (ABC1), member 3
NCLN	1208.356	0.576836	0.127646874	4.519002	6.213E-06	0.00051	Not_Sig	no	gi|555952405|ref|XP_005888388.1|	2739	0	PREDICTED: nicalin isoform X1 [Bos mutus]	gi|982931023|ref|XM_005209007.3|	3220	0	PREDICTED: Bos taurus nicalin (NCLN), transcript variant X1, mRNA	Q969V3	999	0	Nicalin	.	.	.	.	pfam04389	Peptidase_M28	Peptidase family M28.	GO:0031648|protein destabilization;GO:0043254|regulation of protein complex assembly;GO:0009966|regulation of signal transduction;	GO:0005789|endoplasmic reticulum membrane;GO:0016021|integral component of membrane;GO:0016020|membrane;	.	.	.
MBNL2	1580.218	-0.48237	0.106824555	-4.51556	6.315E-06	0.00051	Not_Sig	no	gi|153791183|ref|NP_001093180.1|	1647	0	muscleblind-like protein 2 [Bos taurus]	gi|742235126|ref|XM_010831514.1|	4807	0	PREDICTED: Bison bison bison muscleblind-like splicing regulator 2 (MBNL2), transcript variant X3, mRNA	Q5R4F5	572	0	Muscleblind-like protein 2	.	.	.	.	pfam00642	zf-CCCH	Zinc finger C-x8-C-x5-C-x3-H type (and similar).	GO:0006397|mRNA processing;GO:0043484|regulation of RNA splicing;GO:0008380|RNA splicing;	GO:0005737|cytoplasm;GO:0005634|nucleus;	GO:0046872|metal ion binding;GO:0003723|RNA binding;	K14943	MBNL; muscleblind
LOC102271603	1693.948	-0.4977	0.110228089	-4.51521	6.325E-06	0.00051	Not_Sig	no	gi|27806317|ref|NP_776666.1|	1667	0	annexin A8 [Bos taurus]	gi|942097924|ref|XM_005909992.2|	1923	0	PREDICTED: Bos mutus annexin A8 (LOC102271603), transcript variant X2, mRNA	Q95L54	646	0	Annexin A8	.	.	.	.	pfam00191	Annexin	Annexin. This family of annexins also includes giardin that has been shown to function as an annexin.	GO:0007596|blood coagulation;	.	GO:0005509|calcium ion binding;GO:0005544|calcium-dependent	K17096	ANXA8; annexin A8
JADE2	354.541	0.588197	0.130291844	4.51446	6.348E-06	0.00051	UP	yes	gi|982931991|ref|XP_015327709.1|	3715	0	PREDICTED: protein Jade-2 isoform X2 [Bos taurus]	gi|982985329|ref|XM_015464272.1|	4136	0	PREDICTED: Bos taurus jade family PHD finger 2 (JADE2), transcript variant X2, mRNA	Q9NQC1	1241	0	Protein Jade-2	YPR031w	170	1.31e-42	COG5141	pfam13832	zf-HC5HC2H_2	PHD-zinc-finger like domain.	GO:0043966|histone H3 acetylation;GO:0043983|histone H4-K12 acetylation;GO:0043981|histone H4-K5 acetylation;GO:0043982|histone H4-K8 acetylation;	GO:0070062|extracellular exosome;GO:0000123|histone acetyltransferase complex;GO:0005654|nucleoplasm;	GO:0008270|zinc ion binding;	.	.
PI4KA	2112.38	0.524987	0.116294619	4.514287	6.353E-06	0.00051	Not_Sig	no	gi|998455148|sp|O02811.2|PI4KA_BOVIN	10551	0	RecName: Full=Phosphatidylinositol 4-kinase alpha; Short=PI4-kinase alpha; Short=PI4K-alpha; Short=PtdIns-4-kinase alpha	gi|982951047|ref|XM_010814204.2|	6473	0	PREDICTED: Bos taurus phosphatidylinositol 4-kinase, catalytic, alpha (PI4KA), transcript variant X1, mRNA	O02811	3988	0	Phosphatidylinositol 4-kinase alpha	SPBC577.06c	520	2.68e-151	COG5032	pfam00454	PI3_PI4_kinase	Phosphatidylinositol 3- and 4-kinase. Some members of this family probably do not have lipid kinase activity and are protein	GO:0046854|phosphatidylinositol phosphorylation;GO:0048015|phosphatidylinositol-mediated signaling;	GO:0005737|cytoplasm;GO:0030660|Golgi-associated vesicle membrane;GO:0005886|plasma membrane;	GO:0004430|1-phosphatidylinositol 4-kinase activity;GO:0005524	K00888	PI4K; phosphatidylinositol 4-kinase [EC:2.7.1.67]
SSRP1	2881.047	0.454893	0.100792601	4.513161	6.387E-06	0.00051	Not_Sig	no	gi|594116224|ref|XP_006080782.1|	3000	0	PREDICTED: FACT complex subunit SSRP1 isoform X2 [Bubalus bubalis]	gi|942093387|ref|XM_005908365.2|	2624	0	PREDICTED: Bos mutus structure specific recognition protein 1 (SSRP1), mRNA	Q08945	1142	0	FACT complex subunit SSRP1	SPBC609.05	320	1.28e-98	COG5165	pfam03531	SSrecog	Structure-specific recognition protein (SSRP1). SSRP1 has been implicated in transcriptional initiation and elongation and in	GO:0006281|DNA repair;GO:0006260|DNA replication;GO:1901796|regulation of signal transduction by p53 class mediator;GO:0006355|regulation of transcription, DNA-templated;GO:0006368|transcription elongation from RNA polymerase II promoter;GO:0006366|transcription from RNA polymerase II promoter;	GO:0005694|chromosome;GO:0005737|cytoplasm;GO:0005730|nucleolus;GO:0005654|nucleoplasm;GO:0005634|nucleus;	GO:0003682|chromatin binding;GO:0003677|DNA	K09272	SSRP1; structure-specific recognition protein 1
ZMIZ2	551.1591	0.880697	0.195219862	4.511311	6.443E-06	0.000511	UP	yes	gi|555959652|ref|XP_005891935.1|	3459	0	PREDICTED: zinc finger MIZ domain-containing protein 2 isoform X1 [Bos mutus]	gi|982921882|ref|XM_005205602.3|	3808	0	PREDICTED: Bos taurus zinc finger, MIZ-type containing 2 (ZMIZ2), transcript variant X3, mRNA	Q8NF64	1181	0	Zinc finger MIZ domain-containing protein 2	.	.	.	.	pfam02891	zf-MIZ	MIZ/SP-RING zinc finger. This domain has SUMO (small ubiquitin-like modifier) ligase activity and is involved in DNA	GO:0045944|positive regulation of transcription from RNA polymerase II promoter;GO:0006351|transcription, DNA-templated;	GO:0005739|mitochondrion;GO:0043596|nuclear replication fork;GO:0005654|nucleoplasm;GO:0005634|nucleus;	GO:0030374|ligand-dependent nuclear receptor transcription	.	.
TCOF1	429.7091	0.610596	0.135489997	4.506574	6.588E-06	0.000514	UP	yes	gi|742166889|ref|XP_010848869.1|	4111	0	PREDICTED: treacle protein isoform X1 [Bison bison bison]	gi|742166888|ref|XM_010850567.1|	4756	0	PREDICTED: Bison bison bison Treacher Collins-Franceschetti syndrome 1 (TCOF1), transcript variant X1, mRNA	Q13428	378	1e-106	Treacle protein	.	.	.	.	pfam03546	Treacle	Treacher Collins syndrome protein Treacle.	GO:0001501|skeletal system development;	GO:0005737|cytoplasm;GO:0005730|nucleolus;GO:0005634|nucleus;	GO:0044822|poly(A) RNA binding;GO:0005215|transporter	K14562	TCOF1; Treacher Collins syndrome protein
DSTN	9601.325	-0.56841	0.126157165	-4.50554	6.62E-06	0.000514	Not_Sig	no	gi|157829918|pdb|1AK6|A	863	7e-110	Chain A, Destrin, Nmr, Minimized Average Structure	gi|982942503|ref|XM_015474028.1|	1554	0	PREDICTED: Bos taurus destrin (actin depolymerizing factor) (DSTN), transcript variant X1, mRNA	P60982	336	3e-111	Destrin	.	.	.	.	pfam00241	Cofilin_ADF	Cofilin/tropomyosin-type actin-binding protein. Severs actin filaments and binds to actin monomers.	GO:0030042|actin filament depolymerization;GO:0051014|actin filament severing;GO:0030836|positive regulation of actin filament depolymerization;	GO:0030864|cortical actin cytoskeleton;GO:0005737|cytoplasm;	.	K10363	DSTN; destrin (actin-depolymerizing factor)
UQCRH	519.9151	-0.78568	0.174450883	-4.50374	6.677E-06	0.000514	DOWN	yes	gi|555964427|ref|XP_005894282.1|	471	1e-56	PREDICTED: uncharacterized protein LOC102282170 isoform X2 [Bos mutus]	gi|942050504|ref|XM_014477736.1|	661	0	PREDICTED: Bos mutus cytochrome b-c1 complex subunit 6, mitochondrial (LOC102282170), transcript variant X4, mRNA	P00126	129	3e-36	Cytochrome b-c1 complex subunit 6, mitochondrial	.	.	.	.	pfam02320	UCR_hinge	Ubiquinol-cytochrome C reductase hinge protein. The ubiquinol-cytochrome C reductase complex (cytochrome bc1 complex) is a	GO:0006122|mitochondrial electron transport, ubiquinol to cytochrome c;	GO:0005743|mitochondrial inner membrane;GO:0070469|respiratory chain;	GO:0008121|ubiquinol-cytochrome-c reductase activity;	K00416	QCR6, UQCRH; ubiquinol-cytochrome c reductase subunit 6
RAB23	1154.592	-0.47426	0.10532333	-4.50294	6.702E-06	0.000514	Not_Sig	no	gi|332634768|ref|NP_001193835.1|	1200	7e-151	ras-related protein Rab-23 [Bos taurus]	gi|742159430|ref|XM_010848523.1|	3798	0	PREDICTED: Bison bison bison RAB23, member RAS oncogene family (RAB23), mRNA	Q9ULC3	445	2e-144	Ras-related protein Rab-23	.	.	.	.	pfam00071	Ras	Ras family. Includes sub-families Ras, Rab, Rac, Ral, Ran, Rap Ypt1 and more. Shares P-loop motif with GTP_EFTU, arf and	GO:0000045|autophagosome assembly;GO:0006968|cellular defense response;GO:0042384|cilium assembly;GO:0097094|craniofacial suture morphogenesis;GO:0042992|negative regulation of transcription factor import into nucleus;GO:0015031|protein transport;GO:0007264|small GTPase mediated signal transduction;	GO:0005776|autophagosome;GO:0005737|cytoplasm;GO:0010008|endosome membrane;GO:0070062|extracellular exosome;GO:0045335|phagocytic	GO:0005525|GTP binding;GO:0003924|GTPase activity;	K06234	RAB23; Ras-related protein Rab-23
TGFBR1	1827.789	-0.57991	0.128795072	-4.50257	6.714E-06	0.000514	Not_Sig	no	gi|528959016|ref|XP_005210258.1|	2572	0	PREDICTED: TGF-beta receptor type-1 isoform X1 [Bos taurus]	gi|982933770|ref|XM_005210201.3|	5520	0	PREDICTED: Bos taurus transforming growth factor beta receptor I (TGFBR1), transcript variant X1, mRNA	Q5CD18	988	0	TGF-beta receptor type-1	.	.	.	.	pfam00069	Pkinase	Protein kinase domain.	GO:0032924|activin receptor signaling pathway;GO:0006915|apoptotic process;GO:0060317|cardiac epithelial to mesenchymal transition;GO:0042118|endothelial cell activation;GO:0007507|heart development;GO:0035556|intracellular signal transduction;GO:0048762|mesenchymal cell differentiation;GO:0001938|positive regulation of endothelial cell proliferation;GO:0010628|positive regulation of gene expression;GO:0045893|positive regulation of transcription, DNA-	GO:0005923|bicellular tight junction;GO:0005623|cell;GO:0009986|cell surface;GO:0016021|integral component of membrane;GO:0005622|intracellular;GO:00	GO:0005524|ATP binding;GO:0046872|metal ion binding;GO:0005057	K04674	TGFBR1, ALK5; TGF-beta receptor type-1 [EC:2.7.11.30]
RPLP2	3120.456	-0.67779	0.150809112	-4.49437	6.978E-06	0.000529	DOWN	yes	gi|594042682|ref|XP_006045870.1|	399	1e-45	PREDICTED: 60S acidic ribosomal protein P2 [Bubalus bubalis]	gi|529013114|ref|XM_005227298.1|	527	0	PREDICTED: Bos taurus ribosomal protein, large, P2 (RPLP2), transcript variant X1, mRNA	P42899	131	1e-37	60S acidic ribosomal protein P2	SPBC23G7.15c	75.9	1.52e-17	COG2058	pfam00428	Ribosomal_60s	60s Acidic ribosomal protein. This family includes archaebacterial L12, eukaryotic P0, P1 and P2.	GO:0002181|cytoplasmic translation;GO:0006414|translational elongation;	GO:0022625|cytosolic large ribosomal subunit;GO:0030687|preribosome, large subunit precursor;	GO:0070180|large ribosomal subunit rRNA binding;GO:0003735	K02943	RP-LP2, RPLP2; large subunit ribosomal protein LP2
LOC102281282	132.2266	-0.98484	0.219157334	-4.49374	6.998E-06	0.000529	DOWN	yes	gi|548529146|ref|XP_005700201.1|	600	5e-77	PREDICTED: 60S ribosomal protein L36a [Capra hircus]	gi|402747154|ref|NG_029137.2|	414	0	Bos taurus ribosomal protein L36a (RPL36A) pseudogene on chromosome 15	P83883	213	4e-70	60S ribosomal protein L36a	YNL162w	136	5.05e-42	COG1631	pfam00935	Ribosomal_L44	Ribosomal protein L44.	GO:0032526|response to retinoic acid;GO:0006412|translation;	GO:0022625|cytosolic large ribosomal subunit;	GO:0003735|structural constituent of ribosome;	K02929	RP-L44e, RPL44; large subunit ribosomal protein L44e
GNAI1	273.1001	-0.62016	0.138126999	-4.48976	7.13E-06	0.000533	DOWN	yes	gi|528946374|ref|XP_005205385.1|	1989	0	PREDICTED: guanine nucleotide-binding protein G(i) subunit alpha-1 isoform X1 [Bos taurus]	gi|742157974|ref|XM_010848103.1|	3153	0	PREDICTED: Bison bison bison guanine nucleotide binding protein (G protein), alpha inhibiting activity polypeptide 1 (GNAI1), transcript variant X2, mRNA	B2RSH2	735	0	Guanine nucleotide-binding protein G(i) subunit alpha-1	.	.	.	.	pfam00503	G-alpha	G-protein alpha subunit. G proteins couple receptors of extracellular signals to intracellular signaling pathways. The G protein	GO:0007188|adenylate cyclase-modulating G-protein coupled receptor signaling pathway;GO:0007049|cell cycle;GO:0051301|cell division;GO:1904322|cellular response to forskolin;GO:0007186|G-protein coupled receptor signaling pathway;GO:0050805|negative regulation of synaptic transmission;GO:0043949|regulation of cAMP-mediated signaling;	GO:0005813|centrosome;GO:0005737|cytoplasm;GO:0070062|extracellular exosome;GO:0005834|heterotrimeric G-protein	GO:0031683|G-protein beta/gamma-subunit complex	K04630	GNAI; guanine nucleotide-binding protein G(i) subunit alpha
GAS2L1	670.0878	0.714361	0.159126917	4.489256	7.147E-06	0.000533	UP	yes	gi|686764101|ref|XP_009232499.1|	1529	0	PREDICTED: LOW QUALITY PROTEIN: GAS2-like protein 1 [Pongo abelii]	gi|139948563|ref|NM_001083698.1|	2472	0	Bos taurus growth arrest specific 2 like 1 (GAS2L1), mRNA	Q99501	472	2e-153	GAS2-like protein 1	.	.	.	.	pfam02187	GAS2	Growth-Arrest-Specific Protein 2 Domain.	GO:0007050|cell cycle arrest;GO:0009267|cellular response to starvation;GO:0097067|cellular response to thyroid hormone stimulus;GO:0001578|microtubule bundle formation;GO:0030308|negative regulation of cell growth;GO:0045647|negative regulation of erythrocyte differentiation;GO:0010629|negative regulation of gene expression;GO:0007026|negative regulation of microtubule depolymerization;GO:0051726|regulation of cell cycle;	GO:0005737|cytoplasm;GO:0005856|cytoskeleton;	GO:0008093|cytoskeletal adaptor activity;GO:0008017|microtubule	.	.
MINK1	1083.73	0.576794	0.128756863	4.479711	7.474E-06	0.000554	Not_Sig	no	gi|803235997|ref|XP_011978136.1|	5806	0	PREDICTED: misshapen-like kinase 1 isoform X2 [Ovis aries musimon]	gi|555988554|ref|XM_005906054.1|	4989	0	PREDICTED: Bos mutus misshapen-like kinase 1 (MINK1), transcript variant X1, mRNA	Q8N4C8	2154	0	Misshapen-like kinase 1	.	.	.	.	pfam00069	Pkinase	Protein kinase domain.	GO:0031532|actin cytoskeleton reorganization;GO:0007268|synaptic transmission;GO:0048813|dendrite morphogenesis;GO:0035556|intracellular signal transduction;GO:0007254|JNK cascade;GO:0007275|multicellular organismal development;GO:0046330|positive regulation of JNK cascade;GO:0046777|protein autophosphorylation;GO:0006468|protein phosphorylation;GO:2000311|regulation of alpha-amino-3-hydroxy-5-methyl-4-isoxazole propionate selective glutamate receptor	GO:0030424|axon;GO:0030054|cell junction;GO:0005737|cytoplasm;GO:0005829|cytosol;GO:0030425|dendrite;GO:0070062|extracellular exosome;GO:0005794|Golgi	GO:0005524|ATP binding;GO:0004672|protein kinase activity;GO:0004674	K04413	MINK; misshapen/NIK-related kinase [EC:2.7.11.1]

SLC9A1	986.5463	0.643542	0.143696958	4.478466	7.518E-06	0.000554	UP	yes	gi|555967825|ref|XP_005895957.1|	3779	0	PREDICTED: sodium/hydrogen exchanger 1 [Bos mutus]	gi|942054757|ref|XM_005895895.2|	4474	0	PREDICTED: Bos mutus solute carrier family 9, subfamily A (NHE1, cation proton antiporter 1), member 1 (SLC9A1), mRNA	Q28036	1439	0	Sodium/hydrogen exchanger 1	SPAC15A10.06	126	3.86e-29	COG0025	pfam00999	Na_H_Exchanger	Sodium/hydrogen exchanger family. Na/H antiporters are key transporters in maintaining the pH of actively metabolizing cells. The	GO:0071468|cellular response to acidic pH;GO:0071805|potassium ion transmembrane transport;GO:0051259|protein oligomerization;GO:0051453|regulation of intracellular pH;GO:0006885|regulation of pH;GO:0010447|response to acidic pH;GO:0071436|sodium ion export;GO:0098719|sodium ion import across plasma membrane;	GO:0005737|cytoplasm;GO:0005783|endoplasmic reticulum;GO:0005789|endoplasmic reticulum membrane;GO:0016021|integral component of	GO:0048306|calcium-dependent protein binding;GO:0015386	K05742	SLC9A1, NHE1; solute carrier family 9 (sodium/hydrogen exchanger), member
LOC138992117	138.8127	0.943141	0.2106756	4.476744	7.579E-06	0.000555	UP	yes	.	.	.	.	gi|983002147|ref|XR_228737.3|	339	5e-176	PREDICTED: Bos taurus uncharacterized LOC101906393 (LOC101906393), ncRNA	.	.	.	.	.	.	.	.	pfam00091	Tubulin	Tubulin/FtsZ family, GTPase domain. This family includes the tubulin alpha, beta and gamma chains, as well as the bacterial FtsZ	.	.	.	.	.
DNAJB9	931.0517	-0.51255	0.114526093	-4.47538	7.627E-06	0.000555	Not_Sig	no	gi|300795871|ref|NP_001179968.1|	1106	2e-141	dnaJ homolog subfamily B member 9 precursor [Bos taurus]	gi|982921601|ref|XM_005205458.3|	2209	0	PREDICTED: Bos taurus DnaJ heat shock protein family (Hsp40) member B9 (DNAJB9), transcript variant X1, mRNA	Q9QYI6	404	1e-132	DnaJ homolog subfamily B member 9	BU152	98.2	4.47e-21	COG0484	pfam00226	DnaJ	DnaJ domain. DnaJ domains (J-domains) are associated with hsp70 heat-shock system and it is thought that this domain mediates	GO:0030433|ER-associated ubiquitin-dependent protein catabolic process;	GO:0005783|endoplasmic reticulum;GO:0005788|endoplasmic reticulum lumen;GO:0070062|extracellular exosome;GO:0005730|nucleolus;	GO:0051787|misfolded protein binding;	K09515	DNAJB9; DnaJ homolog subfamily B member 9
TICRR	220.3668	0.680337	0.152311187	4.466757	7.941E-06	0.000574	UP	yes	gi|555966359|ref|XP_005895232.1|	9679	0	PREDICTED: treslin [Bos mutus]	gi|983003559|ref|XM_005196237.3|	6273	0	PREDICTED: Bos taurus TOPBP1-interacting checkpoint and replication regulator (TICRR), mRNA	Q7Z2Z1	2544	0	Treslin	.	.	.	.	pfam15292	Treslin_N	Treslin N-terminus. This family represents the N-terminus of treslin, a checkpoint regulator which plays a role in DNA	GO:0000075|cell cycle checkpoint;GO:0006281|DNA repair;GO:0006260|DNA replication;GO:0001731|formation of translation preinitiation complex;GO:0033314|mitotic DNA replication checkpoint;GO:0030174|regulation of DNA-dependent DNA replication initiation;GO:0010212|response to ionizing radiation;	GO:0005654|nucleoplasm;GO:0005634|nucleus;	GO:0003682|chromatin binding;	.	.
SMIM14	1639.982	-0.66224	0.148582402	-4.45706	8.309E-06	0.000594	DOWN	yes	gi|946789173|ref|XP_005877509.2|	573	8e-65	PREDICTED: small integral membrane protein 14 [Myotis brandtii]	gi|982928282|ref|XM_005207893.3|	3759	0	PREDICTED: Bos taurus small integral membrane protein 14 (SMIM14), transcript variant X1, mRNA	Q2KIK3	206	8e-60	Small integral membrane protein 14	.	.	.	.	pfam11027	DUF2615	Protein of unknown function (DUF2615). This small. approximately 100 residue, family is conserved from worms to	.	GO:0005783|endoplasmic reticulum;GO:0005789|endoplasmic reticulum membrane;GO:0016021|integral component of membrane;	.	.	.
E2F8	215.4864	0.711379	0.159617891	4.456762	8.321E-06	0.000594	UP	yes	gi|742196304|ref|XP_010856725.1|	4337	0	PREDICTED: transcription factor E2F8 isoform X1 [Bison bison bison]	gi|942069219|ref|XM_005900672.2|	3512	0	PREDICTED: Bos mutus E2F transcription factor 8 (E2F8), mRNA	E1BKK0	1659	0	Transcription factor E2F8	.	.	.	.	pfam02319	E2F_TDP	E2F/DP family winged-helix DNA-binding domain. This family contains the transcription factor E2F and its dimerization partners	GO:0033301|cell cycle comprising mitosis without cytokinesis;GO:0008283|cell proliferation;GO:0060718|chorionic trophoblast cell differentiation;GO:0070365|hepatocyte differentiation;GO:0032466|negative regulation of cytokinesis;GO:0000122|negative regulation of transcription from RNA polymerase II promoter;GO:0001890|placenta development;GO:0032877|positive regulation of DNA endoreduplication;GO:0045944|positive regulation of transcription from RNA polymerase II promoter;GO:0002040|sprouting	GO:0005634|nucleus;GO:0005667|transcription factor complex;	GO:0001047|core promoter binding;GO:0000978|RNA polymerase II	K09391	E2F7_8; transcription factor E2F7/8
HSPG2	18918.64	0.602481	0.135262895	4.454148	8.423E-06	0.000597	UP	yes	gi|982915759|ref|XP_015316345.1|	21806	0	PREDICTED: basement membrane-specific heparan sulfate proteoglycan core protein isoform X8 [Bos taurus]	gi|982977977|ref|XM_010826688.2|	14080	0	PREDICTED: Bos taurus heparan sulfate proteoglycan 2 (HSPG2), transcript variant X11, mRNA	P98160	7613	0	Basement membrane-specific heparan sulfate proteoglycan core protein	.	.	.	.	pfam00054	Laminin_G_1	Laminin G domain.	GO:0001525|angiogenesis;GO:0044267|cellular protein metabolic process;GO:0022617|extracellular matrix disassembly;GO:0030198|extracellular matrix organization;GO:0006024|glycosaminoglycan biosynthetic process;GO:0006027|glycosaminoglycan catabolic process;GO:0030203|glycosaminoglycan metabolic process;GO:0042157|lipoprotein metabolic process;GO:0001523|retinoid metabolic process;	GO:0005604|basement membrane;GO:0070062|extracellular exosome;GO:0031012|extracellular matrix;GO:0005576|extracellular	GO:0005509|calcium ion binding;GO:0008022|protein C-terminus	K06255	HSPG2; heparan sulfate proteoglycan 2 (perlecan)
CPED1	410.2467	-0.54098	0.121577836	-4.44964	8.602E-06	0.000606	Not_Sig	no	gi|555964833|ref|XP_005894478.1|	5228	0	PREDICTED: cadherin-like and PC-esterase domain-containing protein 1 [Bos mutus]	gi|742110575|ref|XM_010838064.1|	5291	0	PREDICTED: Bison bison bison cadherin-like and PC-esterase domain containing 1 (CPED1), transcript variant X2, mRNA	A4D0V7	1658	0	Cadherin-like and PC-esterase domain-containing protein 1	.	.	.	.	pfam12733	Cadherin-like	Cadherin-like beta sandwich domain. This domain is found in several bacterial, metazoan and chlorophyte algal proteins. A	.	GO:0005783|endoplasmic reticulum;	.	.	.
AP1B1	1489.956	0.583176	0.131752881	4.426285	9.587E-06	0.000672	Not_Sig	no	gi|555950702|ref|XP_005887552.1|	4556	0	PREDICTED: AP-1 complex subunit beta-1 isoform X4 [Bos mutus]	gi|555950701|ref|XM_005887490.1|	3792	0	PREDICTED: Bos mutus adaptor-related protein complex 1, beta 1 subunit (AP1B1), transcript variant X4, mRNA	Q10567	1713	0	AP-1 complex subunit beta-1	SPBC947.02	558	0	COG5096	pfam01602	Adaptin_N	Adaptin N terminal region. This family consists of the N terminal region of various alpha, beta and gamma subunits of the AP-1, AP-2	GO:0019886|antigen processing and presentation of exogenous peptide antigen via MHC class II;GO:0007368|determination of left/right symmetry;GO:0007507|heart development;GO:0006886|intracellular protein transport;GO:0050690|regulation of defense response to virus by virus;GO:0016192|vesicle-mediated transport;	GO:0030131|clathrin adaptor complex;GO:0030669|clathrin-coated endocytic vesicle membrane;GO:0030659|cytoplasmic vesicle	GO:0019901|protein kinase binding;GO:0008565|protein transporter	K12392	AP1B1; AP-1 complex subunit beta-1
GAK	1316.825	0.576028	0.130192703	4.424429	9.67E-06	0.000673	Not_Sig	no	gi|114052420|ref|NP_001039549.1|	5647	0	cyclin-G-associated kinase [Bos taurus]	gi|402746665|ref|NM_001046084.2|	4186	0	Bos taurus cyclin G associated kinase (GAK), mRNA	O14976	1425	0	Cyclin-G-associated kinase	.	.	.	.	pfam00069	Pkinase	Protein kinase domain.	GO:0007049|cell cycle;GO:0072318|clathrin coat disassembly;GO:0072583|clathrin-mediated endocytosis;GO:0007029|endoplasmic reticulum organization;GO:0072600|establishment of protein localization to Golgi;GO:0090002|establishment of protein localization to plasma membrane;GO:0007030|Golgi organization;GO:0090160|Golgi to lysosome transport;GO:0010977|negative regulation of neuron projection development;GO:0006898|receptor-mediated endocytosis;	GO:0005829|cytosol;GO:0005925|focal adhesion;GO:0005794|Golgi apparatus;GO:0043231|intracellular membrane-bounded	GO:0005524|ATP binding;GO:0030332|cyclin binding;GO:0004674	K08855	GAK; cyclin G-associated kinase [EC:2.7.11.1]
TUBG1	616.1725	0.592873	0.134046489	4.422892	9.739E-06	0.000674	UP	yes	gi|115495769|ref|NP_001069723.1|	2342	0	tubulin gamma-1 chain [Bos taurus]	gi|742135108|ref|XM_010842503.1|	1665	0	PREDICTED: Bison bison bison tubulin gamma-1 chain (LOC104990432), mRNA	Q0VCD2	906	0	Tubulin gamma-1 chain	SPBC32F12.04	644	0	COG5023	pfam00091	Tubulin	Tubulin/FtsZ family, GTPase domain. This family includes the tubulin alpha, beta and gamma chains, as well as the bacterial FtsZ	GO:0031122|cytoplasmic microtubule organization;GO:0007020|microtubule nucleation;	GO:0005813|centrosome;GO:0005737|cytoplasm;GO:0000930|gamma-tubulin complex;GO:0005827|polar microtubule;	GO:0005525|GTP binding;GO:0003924|GTPase activity;	K10389	TUBG; tubulin gamma
CBFB	988.4321	-0.55083	0.124670955	-4.4183	9.948E-06	0.000684	Not_Sig	no	gi|300794490|ref|NP_001178364.1|	793	3e-95	core-binding factor subunit beta [Bos taurus]	gi|982951787|ref|XM_005218718.3|	2903	0	PREDICTED: Bos taurus core-binding factor, beta subunit (CBFB), transcript variant X1, mRNA	Q13951	308	4e-96	Core-binding factor subunit beta	.	.	.	.	pfam02312	CBF_beta	Core binding factor beta subunit. Core binding factor (CBF) is a heterodimeric transcription factor essential for genetic regulation of	GO:0048469|cell maturation;GO:0060216|definitive hemopoiesis;GO:0030098|lymphocyte differentiation;GO:0030099|myeloid cell differentiation;GO:0001649|osteoblast differentiation;GO:0045944|positive regulation of transcription from RNA polymerase II promoter;GO:0000209|protein polyubiquitination;GO:0006357|regulation of transcription from RNA polymerase II promoter;GO:0006366|transcription from RNA polymerase II promoter;	GO:0016513|core-binding factor complex;GO:0016020|membrane;	GO:0003677|DNA binding;GO:0003713|transcription coactivator	.	.
FAP	522.6024	-0.56012	0.126821395	-4.41661	1.003E-05	0.000686	Not_Sig	no	gi|148233352|ref|NP_001091470.1|	4109	0	prolyl endopeptidase FAP [Bos taurus]	gi|942058519|ref|XM_005897286.2|	2769	0	PREDICTED: Bos mutus fibroblast activation protein, alpha (FAP), transcript variant X2, mRNA	A5D7B7	1587	0	Prolyl endopeptidase FAP	SPACUNK4.08	296	1.08e-86	COG1506	pfam00930	DPPIV_N	Dipeptidyl peptidase IV (DPP IV) N-terminal region. This family is an alignment of the region to the N-terminal side of the active site. The	GO:0001525|angiogenesis;GO:0007155|cell adhesion;GO:0043542|endothelial cell migration;GO:1902362|melanocyte apoptotic process;GO:0097325|melanocyte proliferation;GO:0071850|mitotic cell cycle arrest;GO:0060244|negative regulation of cell proliferation involved in contact inhibition;GO:0010716|negative regulation of extracellular matrix disassembly;GO:0071158|positive regulation of cell cycle arrest;GO:1900119|positive regulation of execution phase of apoptosis;GO:0051603|proteolysis involved in cellular protein catabolic	GO:0045177|apical part of cell;GO:0045178|basal part of cell;GO:0009986|cell surface;GO:0005615|extracellular	GO:0008239|dipeptidyl-peptidase activity;GO:0004252|serine-type	K08674	FAP; fibroblast activation protein, alpha [EC:3.4.21.-]
LOC138991014	271.0268	0.698603	0.158289713	4.413447	1.017E-05	0.000692	UP	yes	.	.	.	.	gi|850489648|gb|CP011900.1|	219	2e-109	Ovis canadensis canadensis isolate 43U chromosome 15 sequence	.	.	.	.	.	.	.	.	pfam13433	Peripla_BP_5	Periplasmic binding protein domain. This domain is found in a variety of bacterial periplasmic binding proteins.	.	.	.	.	.
ATP5MK	612.5891	-0.84782	0.192318083	-4.4084	1.041E-05	0.000704	DOWN	yes	gi|431895474|gb|ELK04990.1|	311	6e-34	Up-regulated during skeletal muscle growth protein 5 [Pteropus alecto]	gi|926725078|ref|XM_005698422.2|	397	0	PREDICTED: Capra hircus up-regulated during skeletal muscle growth 5 homolog (mouse) (USMG5), mRNA	Q3ZBI7	121	9e-35	Up-regulated during skeletal muscle growth protein 5	.	.	.	.	pfam14960	ATP_synth_reg	ATP synthase regulation. Members of this family are subunits of mitochondrial ATP synthase (F-ATPase) and vacuolar ATPase (V-	.	GO:0016021|integral component of membrane;GO:0005753|mitochondrial proton-transporting ATP synthase complex;	.	K18194	USMG5, DAPIT; up-regulated during skeletal muscle growth protein 5
CDCA8	417.8554	0.552811	0.125460453	4.406255	1.052E-05	0.000707	Not_Sig	no	gi|139947520|ref|NP_001077121.1|	1412	0	borealin [Bos taurus]	gi|742214176|ref|XM_010862774.1|	2161	0	PREDICTED: Bison bison bison cell division cycle associated 8 (CDCA8), mRNA	Q5RBS5	471	5e-160	Borealin	.	.	.	.	pfam10512	Borealin	Cell division cycle-associated protein 8. The chromosomal passenger complex of Aurora B kinase, INCENP, and Survivin has	GO:0051301|cell division;GO:0007067|mitotic nuclear division;	GO:0032133|chromosome passenger complex;GO:0000775|chromosome, centromeric region;GO:0005737|cytoplasm;GO:0005730	.	K11514	CDCA8; Borealin
FOS	1375.368	0.48451	0.110022062	4.403754	1.064E-05	0.000707	Not_Sig	no	gi|33354267|ref|NP_877587.1|	1593	0	proto-oncogene c-Fos [Bos taurus]	gi|742185777|ref|XM_010855721.1|	2111	0	PREDICTED: Bison bison bison FBJ murine osteosarcoma viral oncogene homolog (FOS), mRNA	O77628	618	0	Proto-oncogene c-Fos	.	.	.	.	pfam07716	bZIP_2	Basic region leucine zipper.	GO:0071277|cellular response to calcium ion;GO:0031668|cellular response to extracellular stimulus;GO:0034614|cellular response to reactive oxygen species;GO:0007399|nervous system development;GO:0045672|positive regulation of osteoclast differentiation;GO:0006355|regulation of transcription, DNA-templated;GO:0042493|response to drug;GO:0035994|response to muscle stretch;GO:0035914|skeletal muscle cell differentiation;GO:0060395|SMAD protein signal transduction;GO:0007179|transforming growth	GO:0005829|cytosol;GO:0005783|endoplasmic reticulum;GO:0005654|nucleoplasm;GO:0005634|nucleus;GO:0005667|transcription	GO:0003682|chromatin binding;GO:0003677|DNA	K04379	FOS; proto-oncogene protein c-fos
PHF12	615.131	0.621345	0.141098741	4.403616	1.065E-05	0.000707	UP	yes	gi|743725195|ref|XP_010956551.1|	4959	0	PREDICTED: PHD finger protein 12 isoform X1 [Camelus bactrianus]	gi|300798250|ref|NM_001192131.1|	3940	0	Bos taurus PHD finger protein 12 (PHF12), mRNA	Q96QT6	1852	0	PHD finger protein 12	.	.	.	.	pfam00628	PHD	PHD-finger. PHD folds into an interleaved type of Zn-finger chelating 2 Zn ions in a similar manner to that of the RING and	GO:0000122|negative regulation of transcription from RNA polymerase II promoter;GO:0045892|negative regulation of transcription, DNA-templated;GO:0006351|transcription, DNA-templated;	GO:0005654|nucleoplasm;GO:0005634|nucleus;GO:0016580|Sin3 complex;GO:0017053|transcriptional repressor complex;	GO:0003682|chromatin binding;GO:0042393|histone	.	.
KLHL7	579.4883	-0.56881	0.129242131	-4.40109	1.077E-05	0.000711	Not_Sig	no	gi|115496810|ref|NP_001069505.1|	3051	0	kelch-like protein 7 [Bos taurus]	gi|742114540|ref|XM_010839042.1|	3102	0	PREDICTED: Bison bison bison kelch-like family member 7 (KLHL7), transcript variant X1, mRNA	Q8IXQ5	1172	0	Kelch-like protein 7	.	.	.	.	pfam07707	BACK	BTB And C-terminal Kelch. This domain is found associated with pfam00651 and pfam01344. The BACK domain is found juxtaposed	GO:0016567|protein ubiquitination;GO:0042787|protein ubiquitination involved in ubiquitin-dependent protein catabolic process;	GO:0031463|Cul3-RING ubiquitin ligase complex;GO:0005737|cytoplasm;GO:0005730|nucleolus;GO:0005654|nucleoplasm;GO:0005634|nucleus;GO:0005886|plasma	GO:0042802|identical protein binding;GO:0042803|protein	K10445	KLHL7; kelch-like protein 7
RPS15	3935.153	-0.49275	0.11206301	-4.39707	1.097E-05	0.00072	Not_Sig	no	gi|836710397|ref|XP_012789585.1|	579	6e-73	PREDICTED: 40S ribosomal protein S15 [Sorex araneus]	gi|74353852|gb|BC102068.1|	528	0	Bos taurus ribosomal protein S15, mRNA (cDNA clone MGC:127050 IMAGE:7942821), complete cds	Q56K10	225	1e-73	40S ribosomal protein S15	SPCC1393.03	172	3.90e-55	COG0185	pfam00203	Ribosomal_S19	Ribosomal protein S19.	GO:0000028|ribosomal small subunit assembly;GO:0006412|translation;	GO:0022627|cytosolic small ribosomal subunit;	GO:0003723|RNA binding;GO:0003735|structural constituent of	K02958	RP-S15e, RPS15; small subunit ribosomal protein S15e
UFC1	798.6189	-0.54985	0.125123681	-4.39441	1.111E-05	0.000725	Not_Sig	no	gi|674077614|ref|XP_008846233.1|	989	2e-131	PREDICTED: ubiquitin-fold modifier-conjugating enzyme 1 isoform X1 [Nannospalax galili]	gi|942095694|ref|XM_005909201.2|	1090	0	PREDICTED: Bos mutus ubiquitin-fold modifier conjugating enzyme 1 (UFC1), transcript variant X2, mRNA	Q5E953	349	2e-119	Ubiquitin-fold modifier-conjugating enzyme 1	.	.	.	.	pfam08694	UFC1	Ubiquitin-fold modifier-conjugating enzyme 1. Ubiquitin-like (UBL) post-translational modifiers are covalently linked to	GO:1990592|protein K69-linked ufmylation;GO:0071569|protein ufmylation;GO:0034976|response to endoplasmic reticulum stress;	GO:0005737|cytoplasm;GO:0070062|extracellular exosome;	GO:0071568|UFM1 transferase activity;	K12165	UFC1; ufm1-conjugating enzyme 1
PLEKHA3	345.9695	-0.58484	0.133308544	-4.38711	1.149E-05	0.000746	Not_Sig	no	gi|555991601|ref|XP_005907584.1|	1455	0	PREDICTED: pleckstrin homology domain-containing family A member 3 [Bos mutus]	gi|965908499|ref|XM_012168382.2|	2888	0	PREDICTED: Ovis aries musimon pleckstrin homology domain containing A3 (PLEKHA3), transcript variant X3, mRNA	Q9HB20	515	1e-172	Pleckstrin homology domain-containing family A member 3	.	.	.	.	pfam00169	PH	PH domain. PH stands for pleckstrin homology.	.	GO:0005794|Golgi apparatus;GO:0016020|membrane;	GO:0005545|1-phosphatidylinositol binding;GO:0005543|phospholipid	.	.
PRRC2A	3675.412	0.619652	0.141528476	4.378287	1.196E-05	0.00076	UP	yes	gi|555984720|ref|XP_005904245.1|	6516	0	PREDICTED: protein PRRC2A [Bos mutus]	gi|329663256|ref|NM_001205557.1|	6777	0	Bos taurus proline-rich coiled-coil 2A (PRRC2A), mRNA	Q5TM26	1359	0	Protein PRRC2A	.	.	.	.	pfam07001	BAT2_N	BAT2 N-terminus. This family represents the N-terminus (approximately 200 residues) of the proline-rich protein BAT2.	GO:0030154|cell differentiation;	GO:0005737|cytoplasm;GO:0005634|nucleus;	.	.	.
NDUFS4	475.7446	-0.7403	0.169106173	-4.3777	1.199E-05	0.00076	DOWN	yes	gi|28461229|ref|NP_786994.1|	762	2e-98	NADH dehydrogenase [ubiquinone] iron-sulfur protein 4, mitochondrial precursor [Bos taurus]	gi|555950450|ref|XM_005887366.1|	664	0	PREDICTED: Bos mutus NADH dehydrogenase (ubiquinone) Fe-S protein 4, 18kDa (NADH-coenzyme Q reductase) (NDUFS4), mRNA	Q02375	298	5e-100	NADH dehydrogenase [ubiquinone] iron-sulfur protein 4, mitochondrial	.	.	.	.	pfam04800	ETC_C1_NDUFA4	ETC complex I subunit conserved region. Family of pankaryotic NADH-ubiquinone oxidoreductase subunits (EC:1.6.5.3) (EC:1.6.99.3)	GO:0007420|brain development;GO:0019933|cAMP-mediated signaling;GO:0045333|cellular respiration;GO:0022900|electron transport chain;GO:0032981|mitochondrial respiratory chain complex I assembly;GO:0048146|positive regulation of fibroblast proliferation;GO:0072593|reactive oxygen species metabolic process;GO:0001932|regulation of protein phosphorylation;GO:0051591|response to cAMP;	GO:0005747|mitochondrial respiratory chain complex I;	GO:0008137|NADH dehydrogenase (ubiquinone) activity;	K03937	NDUFS4; NADH dehydrogenase (ubiquinone) Fe-S protein 4
BRPF1	506.9232	0.567792	0.129700868	4.377702	1.199E-05	0.00076	Not_Sig	no	gi|594095009|ref|XP_006070793.1|	5638	0	PREDICTED: peregrin isoform X1 [Bubalus bubalis]	gi|742133563|ref|XM_010842080.1|	4759	0	PREDICTED: Bison bison bison bromodomain and PHD finger containing, 1 (BRPF1), transcript variant X2, mRNA	P55201	2062	0	Peregrin	YPR031w	220	7.20e-59	COG5141	pfam13832	zf-HC5HC2H_2	PHD-zinc-finger like domain.	GO:0043966|histone H3 acetylation;GO:0045893|positive regulation of transcription, DNA-templated;GO:1901796|regulation of signal transduction by p53 class mediator;GO:0006351|transcription, DNA-templated;	GO:0005737|cytoplasm;GO:0070776|MOZ/MORF histone acetyltransferase complex;GO:0005654|nucleoplasm;GO:0005634|nucleus;	GO:0003677|DNA binding;GO:0008270|zinc ion binding;	K11348	BRPF1; bromodomain and PHD finger-containing protein 1
RNPEPL1	1690.568	0.716923	0.163789439	4.3771	1.203E-05	0.00076	UP	yes	gi|803308473|ref|XP_012012616.1|	3240	0	PREDICTED: arginyl aminopeptidase-like 1 [Ovis aries musimon]	gi|965889900|ref|XM_012157226.2|	2545	0	PREDICTED: Ovis aries musimon arginyl aminopeptidase-like 1 (LOC105612003), mRNA	Q9HAU8	909	0	Arginyl aminopeptidase-like 1	SPCC1322.05c	267	1.80e-77	COG0308	pfam01433	Peptidase_M1	Peptidase family M1. Members of this family are aminopeptidases. The members differ widely in specificity, hydrolysing acidic, basic	GO:0043171|peptide catabolic process;GO:0006508|proteolysis;	GO:0005737|cytoplasm;GO:0005886|plasma membrane;	GO:0070006|metalloaminopeptidase activity;GO:0042277|peptide	K09605	RNPEPL1; arginyl aminopeptidase-like 1 [EC:3.4.11.-]
SLC30A1	910.45	-0.62317	0.14237868	-4.37684	1.204E-05	0.00076	DOWN	yes	gi|329663783|ref|NP_001192822.1|	2054	0	zinc transporter 1 [Bos taurus]	gi|742175472|ref|XM_010853017.1|	5277	0	PREDICTED: Bison bison bison solute carrier family 30 (zinc transporter), member 1 (SLC30A1), mRNA	Q4R6K2	717	0	Zinc transporter 1	YMR243c	92	2.22e-18	COG1230	pfam01545	Cation_efflux	Cation efflux family. Members of this family are integral membrane proteins, that are found to increase tolerance to divalent	GO:0006829|zinc II ion transport;	GO:0016021|integral component of membrane;GO:0005886|plasma membrane;	GO:0008324|cation transmembrane transporter activity;	K14688	SLC30A1, ZNT1; solute carrier family 30 (zinc transporter), member 1
NSD2	811.7418	0.451294	0.103141568	4.375485	1.212E-05	0.000761	Not_Sig	no	gi|742080066|ref|XP_010848870.1|	3114	0	PREDICTED: histone-lysine N-methyltransferase NSD2-like [Bison bison bison]	gi|982983812|ref|XM_005198721.3|	6849	0	PREDICTED: Bos taurus Wolf-Hirschhorn syndrome candidate 1 (WHSC1), transcript variant X2, mRNA	O96028	949	0	Histone-lysine N-methyltransferase NSD2	.	.	.	.	pfam00855	PWWP	PWWP domain. The PWWP domain is named after a conserved Pro-Trp-Trp-Pro motif. The domain binds to Histone-4 methylated at	GO:0009653|anatomical structure morphogenesis;GO:0003289|atrial septum primum morphogenesis;GO:0003290|atrial septum secundum morphogenesis;GO:0060348|bone development;GO:0006303|double-strand break repair via nonhomologous end joining;GO:0010452|histone H3-K36 methylation;GO:0003149|membranous septum morphogenesis;GO:0000122|negative regulation of transcription from RNA polymerase II promoter;GO:0048298|positive regulation of isotype switching to IgA isotypes;GO:2001032|regulation of	GO:0005694|chromosome;GO:0005737|cytoplasm;GO:0005654|nucleoplasm;GO:0005634|nucleus;	GO:0003682|chromatin binding;GO:0042799|histone	K11424	WHSC1, MMSET, NSD2; histone-lysine N-methyltransferase NSD2 [EC:2.1.1.43]
LOC138989945	131.6467	1.079359	0.24697806	4.370264	1.241E-05	0.000767	UP	yes	gi|742078684|ref|XP_010840047.1|	1345	9e-176	PREDICTED: uncharacterized protein LOC104989911 [Bison bison bison]	gi|742078683|ref|XM_010841745.1|	1762	0	PREDICTED: Bison bison bison uncharacterized LOC104989911 (LOC104989911), mRNA	.	.	.	.	.	.	.	.	pfam15402	Spc7_N	N-terminus of kinetochore NMS complex subunit Spc7.	.	.	.	.	.
SRCAP	1469.454	0.615532	0.140907115	4.368351	1.252E-05	0.000767	UP	yes	gi|670984890|ref|XP_008683534.1|	8256	0	PREDICTED: helicase SRCAP [Ursus maritimus]	gi|742107376|ref|XM_010837333.1|	10488	0	PREDICTED: Bison bison bison Snf2-related CREBBP activator protein (SRCAP), mRNA	Q6ZRS2	2448	0	Helicase SRCAP	YDR334w	468	3.32e-135	COG0553	pfam00176	SNF2_N	SNF2 family N-terminal domain. This domain is found in proteins involved in a variety of processes including transcription regulation	GO:0006357|regulation of transcription from RNA polymerase II promoter;GO:0006351|transcription, DNA-templated;GO:0016032|viral process;	GO:0005794|Golgi apparatus;GO:0005634|nucleus;GO:0048471|perinuclear region of cytoplasm;GO:0043234|protein complex;	GO:0005524|ATP binding;GO:0003677|DNA binding;GO:0004386	K11661	SRCAP; helicase SRCAP [EC:3.6.4.-]
KHSRP	1699.267	0.531764	0.12173443	4.368233	1.253E-05	0.000767	Not_Sig	no	gi|528955563|ref|XP_003586314.2|	2416	0	PREDICTED: far upstream element-binding protein 2 isoform X1, partial [Bos taurus]	gi|528955562|ref|XM_003586266.2|	3838	0	PREDICTED: Bos taurus KH-type splicing regulatory protein (KHSRP), transcript variant X1, partial mRNA	Q3U0V1	926	0	Far upstream element-binding protein 2	.	.	.	.	pfam00013	KH_1	KH domain. KH motifs bind RNA in vitro. Autoantibodies to Nova, a KH domain protein, cause paraneoplastic opsoclonus ataxia.	GO:0061158|3'-UTR-mediated mRNA destabilization;GO:0071345|cellular response to cytokine stimulus;GO:0006402|mRNA catabolic process;GO:0006397|mRNA processing;GO:0051028|mRNA transport;GO:0045019|negative regulation of nitric oxide biosynthetic process;GO:0061014|positive regulation of mRNA catabolic process;GO:2000628|regulation of miRNA metabolic process;GO:0043488|regulation of mRNA stability;GO:0006355|regulation of transcription, DNA-templated;GO:0008380|RNA	GO:0010494|cytoplasmic stress granule;GO:0005829|cytosol;GO:0016020|membrane;GO:0005654|nucleoplasm;	GO:0003677|DNA binding;GO:0035925|mRNA 3'-UTR AU-rich region	K13210	FUBP; far upstream element-binding protein
TGFB2	1438.791	-0.45062	0.103163947	-4.36801	1.254E-05	0.000767	Not_Sig	no	gi|560962572|ref|XP_006203718.1|	2094	0	PREDICTED: transforming growth factor beta-2 isoform X1 [Vicugna pacos]	gi|965865117|ref|XM_012147081.2|	5787	0	PREDICTED: Ovis aries musimon transforming growth factor beta 2 (TGFB2), transcript variant X1, mRNA	Q07257	768	0	Transforming growth factor beta-2	.	.	.	.	pfam00019	TGF_beta	Transforming growth factor beta like domain.	GO:0032147|activation of protein kinase activity;GO:0007568|aging;GO:0060317|cardiac epithelial to mesenchymal transition;GO:0060038|cardiac muscle cell proliferation;GO:0010002|cardioblast differentiation;GO:0042637|catagen;GO:0007050|cell cycle arrest;GO:0008219|cell death;GO:0048468|cell development;GO:0016049|cell growth;GO:0016477|cell migration;GO:0000902|cell morphogenesis;GO:0045216|cell-cell junction organization;GO:0030199|collagen fibril organization;GO:0048565|digestive tract	GO:0030424|axon;GO:0005604|basement membrane;GO:0009986|cell surface;GO:0005737|cytoplasm;GO:0031012|extracellular	GO:0001540|beta-amyloid binding;GO:0005125|cytokine	K13376	TGFB2; transforming growth factor beta-2
JUP	573.1377	0.71215	0.163108835	4.366102	1.265E-05	0.000767	UP	yes	gi|440903256|gb|ELR53943.1|	3811	0	Junction plakoglobin, partial [Bos mutus]	gi|942064593|ref|XM_005899231.2|	3198	0	PREDICTED: Bos mutus junction plakoglobin (JUP), transcript variant X1, mRNA	Q8SPJ1	1469	0	Junction plakoglobin	.	.	.	.	pfam00514	Arm	Armadillo/beta-catenin-like repeat. Approx. 40 amino acid repeat. Tandem repeats form super-helix of helices that is	GO:0034333|adherens junction assembly;GO:0086073|bundle of His cell-Purkinje myocyte adhesion involved in cell communication;GO:0016477|cell migration;GO:0071681|cellular response to indole-3-methanol;GO:0002159|desmosome assembly;GO:0050982|detection of mechanical stimulus;GO:0071603|endothelial cell-cell adhesion;GO:0090002|establishment of protein localization to plasma membrane;GO:0042307|positive regulation of protein import into nucleus;GO:0051091|positive regulation of sequence-	GO:0016342|catenin complex;GO:0005913|cell-cell adherens junction;GO:0009898|cytoplasmic side of plasma	GO:0045294|alpha-catenin binding;GO:0045296|cadherin	K10056	JUP; junction plakoglobin
FOXN2	352.6582	-0.65274	0.149517558	-4.36567	1.267E-05	0.000767	DOWN	yes	gi|76629303|ref|XP_600009.2|	2246	0	PREDICTED: forkhead box protein N2 [Bos taurus]	gi|742186400|ref|XM_010855890.1|	3255	0	PREDICTED: Bison bison bison forkhead box N2 (FOXN2), transcript variant X3, mRNA	P32314	782	0	Forkhead box protein N2	SPBC16G5.15c	98.2	7.54e-20	COG5025	pfam00250	Fork_head	Fork head domain.	GO:0030154|cell differentiation;GO:0045893|positive regulation of transcription, DNA-templated;GO:0035914|skeletal muscle cell differentiation;GO:0006351|transcription, DNA-templated;	GO:0043231|intracellular membrane-bounded organelle;GO:0005634|nucleus;	GO:0043565|sequence-specific DNA binding;GO:0003700|transcription factor	K09407	FOXN; forkhead box protein N
PTPRS	2512.519	0.472163	0.1081622	4.365319	1.269E-05	0.000767	Not_Sig	no	gi|741932609|ref|XP_010805292.1|	10004	0	PREDICTED: receptor-type tyrosine-protein phosphatase S isoform X7 [Bos taurus]	gi|982984845|ref|XM_015464129.1|	7034	0	PREDICTED: Bos taurus protein tyrosine phosphatase, receptor type S (PTPRS), transcript variant X7, mRNA	Q13332	3741	0	Receptor-type tyrosine-protein phosphatase S	SPAC11E3.09	146	1.67e-37	COG5599	pfam00102	Y_phosphatase	Protein-tyrosine phosphatase.	GO:0007155|cell adhesion;GO:0007268|synaptic transmission;	GO:0070062|extracellular exosome;GO:0005887|integral component of plasma membrane;GO:0005886|plasma membrane;	GO:0005001|transmembrane receptor protein tyrosine phosphatase	K06778	PTPRS; receptor-type tyrosine-protein phosphatase S [EC:3.1.3.48]
BCL9	1419.611	0.529349	0.121291782	4.364263	1.276E-05	0.000767	Not_Sig	no	gi|742089947|ref|XP_010831541.1|	5857	0	PREDICTED: B-cell CLL/lymphoma 9 protein isoform X1 [Bison bison bison]	gi|742089946|ref|XM_010833239.1|	5591	0	PREDICTED: Bison bison bison B-cell CLL/lymphoma 9 (BCL9), transcript variant X1, mRNA	O00512	2136	0	B-cell CLL/lymphoma 9 protein	.	.	.	.	pfam11502	BCL9	B-cell lymphoma 9 protein. The Wnt pathway plays a role in embryonic development, stem cell growth and tumorigenesis. BCL9	GO:0060070|canonical Wnt signaling pathway;GO:0014908|myotube differentiation involved in skeletal muscle regeneration;GO:0045944|positive regulation of transcription from RNA polymerase II promoter;GO:0035914|skeletal muscle cell differentiation;GO:0035019|somatic stem cell population maintenance;	GO:0005801|cis-Golgi network;GO:0005794|Golgi apparatus;GO:0005654|nucleoplasm;	.	.	.
POM121C	514.4446	0.781428	0.179365798	4.356615	1.321E-05	0.00079	UP	yes	gi|297490496|ref|XP_002698237.1|	3624	0	PREDICTED: nuclear envelope pore membrane protein POM 121C isoform X1 [Bos taurus]	gi|742180099|ref|XM_010854306.1|	5654	0	PREDICTED: Bison bison bison POM121 transmembrane nucleoporin C (POM121C), transcript variant X1, mRNA	A8CG34	647	0	Nuclear envelope pore membrane protein POM 121C	.	.	.	.	pfam15229	POM121	POM121 family.	GO:0031047|gene silencing by RNA;GO:0075733|intracellular transport of virus;GO:0007077|mitotic nuclear envelope disassembly;GO:0006406|mRNA export from nucleus;GO:0006606|protein import into nucleus;GO:0016925|protein sumoylation;GO:1900034|regulation of cellular response to heat;GO:0010827|regulation of glucose transport;GO:0006405|RNA export from nucleus;GO:0006409|tRNA export from nucleus;GO:0016032|viral process;GO:0019083|viral transcription;	GO:0005789|endoplasmic reticulum membrane;GO:0016021|integral component of membrane;GO:0005635|nuclear	GO:0008139|nuclear localization sequence binding;GO:0005487	.	.
SEMA3F	485.2546	0.625609	0.143753137	4.351968	1.349E-05	0.0008	UP	yes	gi|119914707|ref|XP_609627.3|	3942	0	PREDICTED: semaphorin-3F isoform X1 [Bos taurus]	gi|965813013|ref|XM_012122051.2|	3535	0	PREDICTED: Ovis aries musimon sema domain, immunoglobulin domain (Ig), short basic domain, secreted, (semaphorin) 3F (SEMA3F), transcript variant X2, mRNA	Q13275	1448	0	Semaphorin-3F	.	.	.	.	pfam01403	Sema	Sema domain. The Sema domain occurs in semaphorins, which are a large family of secreted and transmembrane proteins, some of	GO:0048846|axon extension involved in axon guidance;GO:0007411|axon guidance;GO:0021785|branchiomotor neuron axon guidance;GO:0021612|facial nerve structural organization;GO:0048843|negative regulation of axon extension involved in axon guidance;GO:0021675|nerve development;GO:0001755|neural crest cell migration;GO:1901166|neural crest cell migration involved in autonomic nervous system development;GO:0030335|positive regulation of cell migration;GO:0071526|semaphorin-plexin signaling	GO:0005615|extracellular space;	GO:0045499|chemorepellent activity;GO:0038191|neuropilin	K06840	SEMA3; semaphorin 3
GNG12	1793.536	-0.50113	0.115159678	-4.35159	1.352E-05	0.0008	Not_Sig	no	gi|27807509|ref|NP_777210.1|	378	7e-39	guanine nucleotide-binding protein G(I)/G(S)/G(O) subunit gamma-12 precursor [Bos taurus]	gi|742165896|ref|XM_010850307.1|	4007	0	PREDICTED: Bison bison bison guanine nucleotide binding protein (G protein), gamma 12 (GNG12), transcript variant X1, mRNA	Q28024	150	2e-40	Guanine nucleotide-binding protein G(I)/G(S)/G(O) subunit gamma-12	.	.	.	.	pfam00631	G-gamma	GGL domain. G-protein gamma like domains (GGL) are found in the gamma subunit of the heterotrimeric G protein complex	GO:0007186|G-protein coupled receptor signaling pathway;	GO:0070062|extracellular exosome;GO:0005834|heterotrimeric G-protein complex;	GO:0004871|signal transducer activity;	K04347	GNG12; guanine nucleotide-binding protein G(I)/G(S)/G(O)

GATA6	974.1069	0.607638	0.139843453	4.345132	1.392E-05	0.00082	UP	yes	gi|803294256|ref|XP_012006784.1|	1976	0	PREDICTED: transcription factor GATA-6 [Ovis aries musimon]	gi|965877580|ref|XM_012151394.2|	3034	0	PREDICTED: Ovis aries musimon GATA binding protein 6 (GATA6), transcript variant X2, mRNA	Q92908	711	0	Transcription factor GATA-6	SPCC1393.08	114	1.36e-25	COG5641	pfam05349	GATA-N	GATA-type transcription activator, N-terminal. GATA transcription factors mediate cell differentiation in a diverse range of tissues.	GO:0048645|organ formation;GO:0007596|blood coagulation;GO:0055007|cardiac muscle cell differentiation;GO:0014898|cardiac muscle hypertrophy in response to stress;GO:0060947|cardiac vascular smooth muscle cell differentiation;GO:0048468|cell development;GO:0045165|cell fate commitment;GO:0044267|cellular protein metabolic process;GO:0071773|cellular response to BMP stimulus;GO:0071371|cellular response to gonadotropin stimulus;GO:0071456|cellular response to hypoxia;GO:0060486|Clara cell	GO:0005654|nucleoplasm;GO:0005634|nucleus;GO:0005667|transcription factor complex;	GO:0003682|chromatin binding;GO:0019901|protein kinase	K17897	GATA6; GATA-binding protein 6
TTLL12	1624.868	0.624473	0.143998592	4.336662	1.447E-05	0.000848	UP	yes	gi|115495709|ref|NP_001068978.1|	3235	0	tubulin--tyrosine ligase-like protein 12 [Bos taurus]	gi|115495708|ref|NM_001075510.1|	3134	0	Bos taurus tubulin tyrosine ligase like 12 (TTLL12), mRNA	Q14166	1099	0	Tubulin--tyrosine ligase-like protein 12	.	.	.	.	pfam03133	TTL	Tubulin-tyrosine ligase family. Tubulins and microtubules are subjected to several post-translational modifications of	GO:0006464|cellular protein modification process;	.	GO:0005524|ATP binding;	K16609	TTLL12; tubulin--tyrosine ligase-like protein 12
C18H19orf33	496.7023	-0.77634	0.179660179	-4.32114	1.552E-05	0.000905	DOWN	yes	gi|333440457|ref|NP_001193975.1|	202	1e-17	immortalization up-regulated protein [Bos taurus]	gi|555993087|ref|XM_005908248.1|	442	0	PREDICTED: Bos mutus chromosome unknown open reading frame, human C19orf33 (LOC102265374), mRNA	.	.	.	.	.	.	.	.	pfam09770	PAT1	Topoisomerase II-associated protein PAT1. Members of this family are necessary for accurate chromosome transmission during	.	.	.	.	.
MCM5	727.4392	0.497046	0.115479322	4.304202	1.676E-05	0.000973	Not_Sig	no	gi|555972663|ref|XP_005898333.1|	3920	0	PREDICTED: DNA replication licensing factor MCM5 [Bos mutus]	gi|942061062|ref|XM_005898271.2|	2469	0	PREDICTED: Bos mutus minichromosome maintenance complex component 5 (MCM5), transcript variant X1, mRNA	Q0V8B7	1513	0	DNA replication licensing factor MCM5	YLR274w	660	0	COG1241	pfam00493	MCM	MCM2/3/5 family.	GO:0007049|cell cycle;GO:0006270|DNA replication initiation;	GO:0042555|MCM complex;GO:0005634|nucleus;	GO:0005524|ATP binding;GO:0003682|chromatin binding;GO:0003678	K02209	MCM5, CDC46; DNA replication licensing factor MCM5 [EC:3.6.4.12]
TMEM47	2508.091	-0.52819	0.122871916	-4.29873	1.718E-05	0.000992	Not_Sig	no	gi|13899273|ref|NP_113630.1|	778	3e-92	transmembrane protein 47 [Homo sapiens]	gi|942101321|ref|XM_005911031.2|	3613	0	PREDICTED: Bos mutus transmembrane protein 47 (TMEM47), mRNA	Q9BQJ4	304	1e-93	Transmembrane protein 47	.	.	.	.	pfam13903	Claudin_2	PMP-22/EMP/MP20/Claudin tight junction. Members of this family are claudins, that form tight junctions between cells.	GO:0098609|cell-cell adhesion;	GO:0005912|adherens junction;GO:0030054|cell junction;GO:0005911|cell-cell junction;GO:0016021|integral component	.	.	.
HJURP	474.4669	0.563392	0.131318402	4.290273	1.785E-05	0.001025	Not_Sig	no	gi|741875462|ref|XP_010798969.1|	3158	0	PREDICTED: Holliday junction recognition protein [Bos taurus]	gi|741875463|ref|XM_010800668.1|	2201	0	PREDICTED: Bos taurus Holliday junction recognition protein (HJURP), transcript variant X2, mRNA	Q8NCD3	593	0	Holliday junction recognition protein	.	.	.	.	pfam12346	HJURP_mid	Holliday junction recognition protein-associated repeat. Vertebral Holliday junction recognition proteins carry an	GO:0007049|cell cycle;GO:0034080|CENP-A containing nucleosome assembly;GO:0007059|chromosome segregation;GO:0051101|regulation of DNA binding;GO:0043254|regulation of protein complex assembly;	GO:0000775|chromosome, centromeric region;GO:0000777|condensed chromosome kinetochore;GO:0005737|cytoplasm;GO:00	GO:0003677|DNA binding;GO:0042393|histone binding;GO:0042802	.	.
TOM1	795.0315	0.536212	0.125013008	4.289253	1.793E-05	0.001025	Not_Sig	no	gi|528950061|ref|XP_005206830.1|	2604	0	PREDICTED: target of Myb protein 1 isoform X1 [Bos taurus]	gi|982926252|ref|XM_005206773.3|	2037	0	PREDICTED: Bos taurus target of myb1 membrane trafficking protein (TOM1), transcript variant X1, mRNA	O60784	891	0	Target of Myb protein 1	.	.	.	.	pfam00790	VHS	VHS domain. Domain present in VPS-27, Hrs and STAM.	GO:0006897|endocytosis;GO:0016197|endosomal transport;GO:0006886|intracellular protein transport;GO:0015031|protein transport;	GO:0005737|cytoplasm;GO:0005829|cytosol;GO:0005769|early endosome;GO:0005768|endosome;GO:0070062|extracellular	GO:0030276|clathrin binding;	.	.
TOB2	237.5588	0.697255	0.162787519	4.283223	1.842E-05	0.001048	UP	yes	gi|926693171|ref|XP_013819826.1|	1161	7e-151	PREDICTED: protein Tob2 [Capra hircus]	gi|982927365|ref|XM_010805745.2|	1925	0	PREDICTED: Bos taurus transducer of ERBB2, 2 (TOB2), transcript variant X1, mRNA	Q14106	435	6e-146	Protein Tob2	.	.	.	.	pfam07742	BTG	BTG family.	GO:0007292|female gamete generation;GO:0008285|negative regulation of cell proliferation;GO:0045671|negative regulation of osteoclast differentiation;GO:0045778|positive regulation of ossification;GO:0010468|regulation of gene expression;GO:0023052|signaling;	GO:0005737|cytoplasm;GO:0005634|nucleus;	GO:0003714|transcription corepressor activity;	K14443	TOB; protein Tob/BTG
EHMT2	1228.577	0.542213	0.126635016	4.281696	1.855E-05	0.00105	Not_Sig	no	gi|329664696|ref|NP_001193192.1|	5627	0	histone-lysine N-methyltransferase EHMT2 [Bos taurus]	gi|329664695|ref|NM_001206263.1|	3919	0	Bos taurus euchromatic histone-lysine N-methyltransferase 2 (EHMT2), mRNA	Q96KQ7	2122	0	Histone-lysine N-methyltransferase EHMT2	SPBC428.08c	147	1.13e-36	COG2940	pfam05033	Pre-SET	Pre-SET motif. This protein motif is a zinc binding motif. It contains 9 conserved cysteines that coordinate three zinc ions. It is	GO:0009267|cellular response to starvation;GO:0006306|DNA methylation;GO:0034968|histone lysine methylation;GO:0016571|histone methylation;GO:0000122|negative regulation of transcription from RNA polymerase II promoter;GO:0018027|peptidyl-lysine dimethylation;GO:0006275|regulation of DNA replication;GO:1901796|regulation of signal transduction by p53 class mediator;	GO:0000790|nuclear chromatin;GO:0005654|nucleoplasm;GO:0005634|nucleus;	GO:0070742|C2H2 zinc finger domain binding;GO:0046976|histone	K11420	EHMT; euchromatic histone-lysine N-methyltransferase [EC:2.1.1.43]
RPS21	1161.398	-0.56622	0.132301569	-4.27976	1.871E-05	0.001051	Not_Sig	no	gi|803207736|ref|XP_011964399.1|	536	2e-67	PREDICTED: 40S ribosomal protein S21 isoform X4 [Ovis aries musimon]	gi|528974049|ref|XM_005214850.1|	404	0	PREDICTED: Bos taurus ribosomal protein S21 (RPS21), transcript variant X1, mRNA	P63221	171	3e-54	40S ribosomal protein S21	.	.	.	.	pfam01249	Ribosomal_S21e	Ribosomal protein S21e.	GO:0000447|endonucleolytic cleavage in ITS1 to separate SSU-rRNA from 5.8S rRNA and LSU-rRNA from tricistronic rRNA transcript (SSU-rRNA, 5.8S rRNA, LSU-rRNA);GO:0000461|endonucleolytic cleavage to generate mature 3'-end of SSU-rRNA from (SSU-rRNA, 5.8S rRNA, LSU-rRNA);GO:0006412|translation;	GO:0022627|cytosolic small ribosomal subunit;	GO:0043022|ribosome binding;GO:0003735|structural	K02971	RP-S21e, RPS21; small subunit ribosomal protein S21e
PPP6R1	1486.701	0.595247	0.139100336	4.279264	1.875E-05	0.001051	UP	yes	gi|594080971|ref|XP_006064114.1|	3747	0	PREDICTED: serine/threonine-protein phosphatase 6 regulatory subunit 1 [Bubalus bubalis]	gi|965904302|ref|XM_012163626.2|	3459	0	PREDICTED: Ovis aries musimon protein phosphatase 6 regulatory subunit 1 (PPP6R1), transcript variant X4, mRNA	Q9UPN7	1197	0	Serine/threonine-protein phosphatase 6 regulatory subunit 1	.	.	.	.	pfam04499	SAPS	SIT4 phosphatase-associated protein. This family includes a conserved region from a group of yeast proteins that associate with	GO:0048208|COPII vesicle coating;GO:0043666|regulation of phosphoprotein phosphatase activity;	GO:0005737|cytoplasm;GO:0005829|cytosol;GO:0000139|Golgi membrane;	GO:0019903|protein phosphatase binding;	K15499	PPP6R1, SAPS1; serine/threonine-protein phosphatase 6 regulatory subunit
TM4SF1	2786.454	-0.52907	0.123720079	-4.27637	1.9E-05	0.00106	Not_Sig	no	gi|115496071|ref|NP_001069448.1|	976	3e-127	transmembrane 4 L6 family member 1 [Bos taurus]	gi|942102890|ref|XM_005911463.2|	1555	0	PREDICTED: Bos mutus transmembrane 4 L six family member 1 (TM4SF1), mRNA	Q5RE43	334	7e-111	Transmembrane 4 L6 family member 1	.	.	.	.	pfam05805	L6_membrane	L6 membrane protein. This family consists of several eukaryotic L6 membrane proteins. L6, IL-TMP, and TM4SF5 are cell surface	.	GO:0016021|integral component of membrane;	.	.	.
MIGA1	543.1064	-0.68585	0.160496587	-4.2733	1.926E-05	0.001069	DOWN	yes	gi|742192999|ref|XP_010855914.1|	3086	0	PREDICTED: protein FAM73A [Bison bison bison]	gi|742192998|ref|XM_010857612.1|	5049	0	PREDICTED: Bison bison bison family with sequence similarity 73, member A (FAM73A), mRNA	Q8NAN2	1097	0	Mitoguardin 1	.	.	.	.	pfam10265	DUF2217	Uncharacterized conserved protein (DUF2217). This is a family of conserved proteins of from 500 - 600 residues found from worms to	GO:0008053|mitochondrial fusion;	GO:0045203|integral component of cell outer membrane;GO:0005741|mitochondrial outer membrane;	GO:0046982|protein heterodimerization activity;GO:0042803	.	.
MYO1C	9740.662	0.486263	0.114146363	4.26	2.044E-05	0.001125	Not_Sig	no	gi|346421399|ref|NP_776821.3|	5378	0	unconventional myosin-Ic [Bos taurus]	gi|147906575|ref|NM_174396.3|	4432	0	Bos taurus myosin IC (MYO1C), mRNA	Q27966	2076	0	Unconventional myosin-Ic	YMR109w	570	8.58e-179	COG5022	pfam00063	Myosin_head	Myosin head (motor domain).	GO:0051028|mRNA transport;GO:0038089|positive regulation of cell migration by vascular endothelial growth factor signaling pathway;GO:0090314|positive regulation of protein targeting to membrane;GO:1900748|positive regulation of vascular endothelial growth factor signaling pathway;GO:0006612|protein targeting to membrane;GO:2000810|regulation of bicellular tight junction assembly;	GO:0009925|basal plasma membrane;GO:0005903|brush border;GO:0016023|cytoplasmic membrane-bounded	GO:0005524|ATP binding;GO:0000146|microfilament motor activity;	K10356	MYO1; myosin I
NCAPD2	857.525	0.48741	0.114422366	4.259741	2.047E-05	0.001125	Not_Sig	no	gi|440895652|gb|ELR47790.1|	7159	0	Condensin complex subunit 1, partial [Bos mutus]	gi|742143992|ref|XM_010844971.1|	5120	0	PREDICTED: Bison bison bison non-SMC condensin I complex, subunit D2 (NCAPD2), transcript variant X1, mRNA	Q15021	2415	0	Condensin complex subunit 1	YLR272c	308	1.29e-85	COG5098	pfam12717	Cnd1	non-SMC mitotic condensation complex subunit 1. The three non-SMC (structural maintenance of chromosomes) subunits of the	GO:0051301|cell division;GO:0051304|chromosome separation;GO:0010032|meiotic chromosome condensation;GO:0007076|mitotic chromosome condensation;	GO:0000793|condensed chromosome;GO:0000779|condensed chromosome, centromeric region;GO:0000796|condensin	GO:0003682|chromatin binding;GO:0042393|histone binding;	K06677	YCS4, CNAP1, CAPD2; condensin complex subunit 1
CIC	1084.769	0.711353	0.167300107	4.25196	2.119E-05	0.001159	UP	yes	gi|942095037|ref|XP_014338154.1|	8726	0	PREDICTED: LOW QUALITY PROTEIN: protein capicua homolog [Bos mutus]	gi|983000040|ref|XM_015467547.1|	8423	0	PREDICTED: Bos taurus capicua transcriptional repressor (CIC), transcript variant X3, mRNA	Q924A2	2690	0	Protein capicua homolog	.	.	.	.	pfam00505	HMG_box	HMG (high mobility group) box.	GO:0048286|lung alveolus development;GO:0000122|negative regulation of transcription from RNA polymerase II promoter;GO:0006351|transcription, DNA-templated;	GO:0005634|nucleus;GO:0043234|protein complex;	GO:0003682|chromatin binding;GO:0003677|DNA binding;	.	.
POLE4	655.8667	-0.62925	0.148341645	-4.24187	2.217E-05	0.0012	DOWN	yes	gi|403260303|ref|XP_003922615.1|	412	1e-47	PREDICTED: DNA polymerase epsilon subunit 4 [Saimiri boliviensis boliviensis]	gi|402745671|ref|NM_001101225.2|	658	0	Bos taurus polymerase (DNA-directed), epsilon 4, accessory subunit (POLE4), mRNA	Q9CQ36	163	4e-49	DNA polymerase epsilon subunit 4	YOR358w	63.5	2.34e-11	COG5208	pfam00808	CBFD_NFYB_HMF	Histone-like transcription factor (CBF/NF-Y) and archaeal histone. This family includes archaebacterial histones and	GO:0043966|histone H3 acetylation;	GO:0005671|Ada2/Gcn5/Ada3 transcription activator complex;GO:0008622|epsilon DNA polymerase complex;	GO:0003677|DNA binding;GO:0003887|DNA-directed DNA polymerase activity;	K03506	POLE4; DNA polymerase epsilon subunit 4 [EC:2.7.7.7]
RPL37A	3883.99	-0.61044	0.143931981	-4.24116	2.224E-05	0.0012	DOWN	yes	gi|928182240|ref|XP_536063.2|	494	1e-58	PREDICTED: 60S ribosomal protein L37a, partial [Canis lupus familiaris]	gi|402744473|ref|NM_001035008.2|	638	0	Bos taurus ribosomal protein L37a (RPL37A), mRNA	P61515	184	1e-57	Putative 60S ribosomal protein L37a	YPR043w	126	1.49e-36	COG1997	pfam01780	Ribosomal_L37ae	Ribosomal L37ae protein family. This ribosomal protein is found in archaebacteria and eukaryotes. It contains four conserved cysteine	GO:0006412|translation;	GO:0005840|ribosome;	GO:0046872|metal ion binding;GO:0003735|structural	.	.
SOX12	238.3054	0.696957	0.16434891	4.240716	2.228E-05	0.0012	UP	yes	gi|965965895|ref|XP_014954973.1|	866	2e-101	PREDICTED: LOW QUALITY PROTEIN: transcription factor SOX-12 isoform X1 [Ovis aries]	gi|982993535|ref|XM_015466134.1|	3080	0	PREDICTED: Bos taurus SRY-box 12 (SOX12), mRNA	Q04890	171	3e-45	Transcription factor SOX-12	.	.	.	.	pfam00505	HMG_box	HMG (high mobility group) box.	GO:0045165|cell fate commitment;GO:0045944|positive regulation of transcription from RNA polymerase II promoter;GO:0065004|protein-DNA complex assembly;GO:0021510|spinal cord development;	GO:0005654|nucleoplasm;GO:0032993|protein-DNA complex;	GO:0001105|RNA polymerase II transcription coactivator	K09268	SOX4_11_12; transcription factor SOX4/11/12 (SOX group C)
RPS29	2417.072	-0.65621	0.154763638	-4.2401	2.234E-05	0.0012	DOWN	yes	gi|759100021|ref|XP_011375277.1|	389	6e-45	PREDICTED: LOW QUALITY PROTEIN: 40S ribosomal protein S29 [Pteropus vampyrus]	gi|942050652|ref|XM_005894294.2|	363	0	PREDICTED: Bos mutus ribosomal protein S29 (RPS29), transcript variant X2, mRNA	P62275	118	4e-34	40S ribosomal protein S29	YLR388w	82.8	1.30e-21	COG0199	pfam00253	Ribosomal_S14	Ribosomal protein S14p/S29e. This family includes both ribosomal S14 from prokaryotes and S29 from eukaryotes.	GO:0043065|positive regulation of apoptotic process;GO:0006412|translation;	GO:0022627|cytosolic small ribosomal subunit;	GO:0003735|structural constituent of ribosome;GO:0008270|zinc ion binding;	K02980	RP-S29e, RPS29; small subunit ribosomal protein S29e
MCM2	1183.033	0.449057	0.106160119	4.230001	2.337E-05	0.001248	Not_Sig	no	gi|983004864|ref|XP_015324308.1|	4358	0	PREDICTED: LOW QUALITY PROTEIN: DNA replication licensing factor MCM2 isoform X2 [Bos taurus]	gi|983004863|ref|XM_015468822.1|	3300	0	PREDICTED: Bos taurus minichromosome maintenance complex component 2 (MCM2), transcript variant X2, mRNA	P49736	1633	0	DNA replication licensing factor MCM2	SPBC4.04c	793	0	COG1241	pfam00493	MCM	MCM2/3/5 family.	GO:0007049|cell cycle;GO:0071353|cellular response to interleukin-4;GO:0006260|DNA replication;GO:0006270|DNA replication initiation;GO:0006268|DNA unwinding involved in DNA replication;GO:0000082|G1/S transition of mitotic cell cycle;GO:0006334|nucleosome assembly;	GO:0000785|chromatin;GO:0005737|cytoplasm;GO:0042555|MCM complex;GO:0015630|microtubule cytoskeleton;GO:0000784|nuclear	GO:0005524|ATP binding;GO:0003677|DNA binding;GO:0003678	K02540	MCM2; DNA replication licensing factor MCM2 [EC:3.6.4.12]
OSBPL8	957.5715	-0.57025	0.134841069	-4.22905	2.347E-05	0.001248	Not_Sig	no	gi|742092523|ref|XP_010832162.1|	4724	0	PREDICTED: oxysterol-binding protein-related protein 8 isoform X1 [Bison bison bison]	gi|982981449|ref|XM_010827549.2|	6727	0	PREDICTED: Bos taurus oxysterol binding protein like 8 (OSBPL8), transcript variant X1, mRNA	Q9BZF1	1731	0	Oxysterol-binding protein-related protein 8	.	.	.	.	pfam01237	Oxysterol_BP	Oxysterol-binding protein.	GO:0032148|activation of protein kinase B activity;GO:0045444|fat cell differentiation;GO:0030336|negative regulation of cell migration;GO:0010891|negative regulation of sequestering of triglyceride;GO:0015914|phospholipid transport;GO:2001275|positive regulation of glucose import in response to insulin stimulus;GO:0046628|positive regulation of insulin receptor signaling pathway;GO:0051897|positive regulation of protein kinase B signaling;GO:0090204|protein localization to nuclear pore;	GO:0032541|cortical endoplasmic reticulum;GO:0005783|endoplasmic reticulum;GO:0005789|endoplasmic reticulum membrane;GO:0016021|integral	GO:0015485|cholesterol binding;GO:0070273|phosphatidylinosit	.	.
AP2A1	1967.52	0.543369	0.128564796	4.226424	2.374E-05	0.001252	Not_Sig	no	gi|329755305|ref|NP_001178369.1|	4778	0	AP-2 complex subunit alpha-1 [Bos taurus]	gi|329755304|ref|NM_001191440.1|	3307	0	Bos taurus adaptor-related protein complex 2, alpha 1 subunit (AP2A1), mRNA	O95782	1815	0	AP-2 complex subunit alpha-1	.	.	.	.	pfam01602	Adaptin_N	Adaptin N terminal region. This family consists of the N terminal region of various alpha, beta and gamma subunits of the AP-1, AP-2	GO:0019886|antigen processing and presentation of exogenous peptide antigen via MHC class II;GO:0072583|clathrin-mediated endocytosis;GO:0006897|endocytosis;GO:0048013|ephrin receptor signaling pathway;GO:0006895|Golgi to endosome transport;GO:0006886|intracellular protein transport;GO:0007018|microtubule-based movement;GO:0042059|negative regulation of epidermal growth factor receptor signaling pathway;GO:1900126|negative regulation of hyaluronan biosynthetic	GO:0030122|AP-2 adaptor complex;GO:0016324|apical plasma membrane;GO:0016323|basolateral plasma membrane;GO:0030130|clathrin coat of	GO:0019901|protein kinase binding;GO:0008565|protein transporter	K11824	AP2A; AP-2 complex subunit alpha
NECTIN3	915.1134	-0.48279	0.114277942	-4.22471	2.393E-05	0.001252	Not_Sig	no	gi|742174400|ref|XP_010851025.1|	2430	0	PREDICTED: nectin-3 isoform X2 [Bison bison bison]	gi|850474981|gb|CP011886.1|	5388	0	Ovis canadensis canadensis isolate 43U chromosome 1 sequence	Q9NQS3	642	0	Nectin-3	.	.	.	.	pfam08205	C2-set_2	CD80-like C2-set immunoglobulin domain. These domains belong to the immunoglobulin superfamily.	GO:0034332|adherens junction organization;GO:0008037|cell recognition;GO:0009566|fertilization;GO:0007157|heterophilic cell-cell adhesion via plasma membrane cell adhesion molecules;GO:0007156|homophilic cell adhesion via plasma membrane adhesion molecules;GO:0002089|lens morphogenesis in camera-type eye;GO:0060042|retina morphogenesis in camera-type eye;GO:0016337|single organismal cell-cell adhesion;GO:0007286|spermatid development;	GO:0043296|apical junction complex;GO:0005913|cell-cell adherens junction;GO:0005887|integral component of plasma membrane;GO:0005886|plasma	GO:0050839|cell adhesion molecule binding;GO:0042803|protein	K06592	PVRL3; poliovirus receptor-related protein 3
RNPEP	414.8134	0.563299	0.133338815	4.224569	2.394E-05	0.001252	Not_Sig	no	gi|148227162|ref|NP_001091032.1|	3002	0	aminopeptidase B [Bos taurus]	gi|148227161|ref|NM_001097563.1|	2237	0	Bos taurus arginyl aminopeptidase (aminopeptidase B) (RNPEP), mRNA	Q9H4A4	1021	0	Aminopeptidase B	SPCC1322.05c	291	1.32e-87	COG0308	pfam01433	Peptidase_M1	Peptidase family M1. Members of this family are aminopeptidases. The members differ widely in specificity, hydrolysing acidic, basic	GO:0045776|negative regulation of blood pressure;GO:0043171|peptide catabolic process;GO:0006508|proteolysis;	GO:0070062|extracellular exosome;GO:0005576|extracellular region;GO:0005615|extracellular space;GO:0005886|plasma	GO:0004177|aminopeptidase activity;GO:0004301|epoxide hydrolase	K01260	RNPEP; aminopeptidase B [EC:3.4.11.6]
ARHGAP23	1546.994	0.525555	0.124415106	4.224208	2.398E-05	0.001252	Not_Sig	no	gi|466072604|ref|XP_004282749.1|	5897	0	PREDICTED: rho GTPase-activating protein 23 [Orcinus orca]	gi|965854850|ref|XM_012141940.2|	5384	0	PREDICTED: Ovis aries musimon Rho GTPase activating protein 23 (ARHGAP23), transcript variant X5, mRNA	Q9P227	2219	0	Rho GTPase-activating protein 23	.	.	.	.	pfam00620	RhoGAP	RhoGAP domain. GTPase activator proteins towards Rho/Rac/Cdc42-like small GTPases.	GO:0043547|positive regulation of GTPase activity;GO:0051056|regulation of small GTPase mediated signal transduction;GO:0007165|signal transduction;	GO:0005829|cytosol;GO:0070062|extracellular exosome;	GO:0005096|GTPase activator activity;	.	.
PKD1	2503.201	0.561675	0.133031747	4.222116	2.42E-05	0.001258	Not_Sig	no	gi|358418816|ref|XP_003584052.1|	19489	0	PREDICTED: LOW QUALITY PROTEIN: polycystin-1 [Bos taurus]	gi|983006772|ref|XM_003584004.4|	13679	0	PREDICTED: Bos taurus polycystic kidney disease 1 (autosomal dominant) (PKD1), mRNA	P98161	6022	0	Polycystin-1	MA0850_4	84.3	7.96e-15	COG3291	pfam02010	REJ	REJ domain. The REJ (Receptor for Egg Jelly) domain is found in PKD1, and the sperm receptor for egg jelly. The function of this domain is	GO:0009653|anatomical structure morphogenesis;GO:0048754|branching morphogenesis of an epithelial tube;GO:0070588|calcium ion transmembrane transport;GO:0007161|calcium-independent cell-matrix adhesion;GO:0001502|cartilage condensation;GO:0051216|cartilage development;GO:0007050|cell cycle arrest;GO:0007160|cell-matrix adhesion;GO:0042994|cytoplasmic sequestering of transcription factor;GO:0050982|detection of mechanical stimulus;GO:0048565|digestive tract development;GO:0001892|embryonic placenta	GO:0016323|basolateral plasma membrane;GO:0009986|cell surface;GO:0060170|ciliary membrane;GO:0005929|cilium;GO:0005737	GO:0005262|calcium channel activity;GO:0030246|carbohydrate	K04985	PKD1; polycystin 1
GRTP1	1377.834	-0.50763	0.120307699	-4.21944	2.449E-05	0.001268	Not_Sig	no	gi|157074184|ref|NP_001096806.1|	1792	0	growth hormone-regulated TBC protein 1 [Bos taurus]	gi|157074183|ref|NM_001103336.1|	2037	0	Bos taurus growth hormone regulated TBC protein 1 (GRTP1), mRNA	Q5TC63	563	0	Growth hormone-regulated TBC protein 1	YOL112w	168	8.44e-44	COG5210	pfam00566	RabGAP-TBC	Rab-GTPase-TBC domain. Identification of a TBC domain in GYP6_YEAST and GYP7_YEAST, which are GTPase activator	GO:0090630|activation of GTPase activity;GO:0006886|intracellular protein transport;GO:0031338|regulation of vesicle fusion;	GO:0012505|endomembrane system;GO:0005622|intracellular;	GO:0005096|GTPase activator activity;GO:0017137|Rab GTPase	.	.
SYMPK	1021.051	0.477895	0.113356609	4.215851	2.488E-05	0.001272	Not_Sig	no	gi|742174056|ref|XP_010850926.1|	6070	0	PREDICTED: symplekin [Bison bison bison]	gi|742174055|ref|XM_010852624.1|	4260	0	PREDICTED: Bison bison bison symplekin (SYMPK), transcript variant X1, mRNA	Q92797	2279	0	Symplekin	.	.	.	.	pfam11935	DUF3453	Domain of unknown function (DUF3453). This presumed domain is functionally uncharacterized. This domain is found in	GO:0007155|cell adhesion;GO:0006378|mRNA polyadenylation;GO:0035307|positive regulation of protein dephosphorylation;	GO:0005923|bicellular tight junction;GO:0005737|cytoplasm;GO:0005856|cytoskeleton;GO:0005654|nucleoplasm;GO:0005886|plasma membrane;	.	K06100	SYMPK; symplekin
NDUFA1	284.8609	-0.90441	0.214530993	-4.21575	2.49E-05	0.001272	DOWN	yes	gi|555950165|ref|XP_005887287.1|	375	6e-44	PREDICTED: NADH dehydrogenase [ubiquinone] 1 alpha subcomplex subunit 1 [Bos mutus]	gi|942030738|ref|XM_005887225.2|	431	0	PREDICTED: Bos mutus NADH dehydrogenase (ubiquinone) 1 alpha subcomplex, 1, 7.5kDa (NDUFA1), mRNA	Q02377	147	6e-45	NADH dehydrogenase [ubiquinone] 1 alpha subcomplex subunit 1	.	.	.	.	pfam02952	Fucose_iso_C	L-fucose isomerase, C-terminal domain.	GO:0055114|oxidation-reduction process;	GO:0016021|integral component of membrane;GO:0005747|mitochondrial respiratory chain complex I;	.	K03945	NDUFA1; NADH dehydrogenase (ubiquinone) 1 alpha subcomplex subunit
MAP4	4975.767	0.511138	0.121254524	4.215412	2.493E-05	0.001272	Not_Sig	no	gi|742212440|ref|XP_010860643.1|	10414	0	PREDICTED: microtubule-associated protein 4 isoform X1 [Bison bison bison]	gi|982961740|ref|XM_010817654.2|	6851	0	PREDICTED: Bos taurus microtubule associated protein 4 (MAP4), transcript variant X1, mRNA	P36225	1182	0	Microtubule-associated protein 4	.	.	.	.	pfam00418	Tubulin-binding	Tau and MAP protein, tubulin-binding repeat. This family includes the vertebrate proteins MAP2, MAP4 and Tau, as well as	GO:0000226|microtubule cytoskeleton organization;GO:0031175|neuron projection development;	GO:0005737|cytoplasm;GO:0005874|microtubule;GO:0005875|microtubule associated complex;GO:0043005|neuron projection;	GO:0008017|microtubule binding;	K10431	MAP4; microtubule-associated protein 4
CDC20	536.0383	0.567989	0.13476358	4.214706	2.501E-05	0.001272	Not_Sig	no	gi|586995291|ref|XP_006934717.1|	2625	0	PREDICTED: cell division cycle protein 20 homolog isoform X1 [Felis catus]	gi|942061365|ref|XM_014478996.1|	1709	0	PREDICTED: Bos mutus cell division cycle 20 (CDC20), mRNA	Q5H7C0	960	0	Cell division cycle protein 20 homolog	.	.	.	.	pfam00400	WD40	WD domain, G-beta repeat.	GO:0031145|anaphase-promoting complex-dependent proteasomal ubiquitin-dependent protein catabolic process;GO:0051301|cell division;GO:0007067|mitotic nuclear division;GO:0008054|negative regulation of cyclin-dependent protein serine/threonine kinase by cyclin degradation;GO:1904146|positive regulation of meiotic cell cycle process involved in oocyte maturation;GO:0090129|positive regulation of synapse maturation;GO:0031915|positive regulation of synaptic plasticity;GO:1904668|positive regulation of ubiquitin protein ligase	GO:0005680|anaphase-promoting complex;GO:0005737|cytoplasm;GO:0005815|microtubule organizing center;GO:0005654|nucleoplasm;GO:00009	GO:0097027|ubiquitin-protein transferase activator activity;	K03363	CDC20; cell division cycle 20, cofactor of APC complex
PLXNB2	6422.409	0.484793	0.115065206	4.213204	2.518E-05	0.001274	Not_Sig	no	gi|982927589|ref|XP_015326940.1|	9684	0	PREDICTED: plexin-B2 isoform X1 [Bos taurus]	gi|982927593|ref|XM_005207506.3|	6139	0	PREDICTED: Bos taurus plexin B2 (PLXNB2), transcript variant X4, mRNA	O15031	3288	0	Plexin-B2	.	.	.	.	pfam08337	Plexin_cytopl	Plexin cytoplasmic RasGAP domain. This family features the C-terminal regions of various plexins. Plexins are receptors for	GO:0007420|brain development;GO:0007156|homophilic cell adhesion via plasma membrane adhesion molecules;GO:0007162|negative regulation of cell adhesion;GO:0001843|neural tube closure;GO:0007405|neuroblast proliferation;GO:0050772|positive regulation of axonogenesis;GO:0010976|positive regulation of neuron projection development;GO:0030334|regulation of cell migration;GO:0008360|regulation of cell shape;GO:0043087|regulation of GTPase activity;GO:2001222|regulation of neuron	GO:0009986|cell surface;GO:0070062|extracellular exosome;GO:0005887|integral component of plasma	GO:0017154|semaphorin receptor activity;	K06821	PLXNB; plexin B
TMEM258	503.8228	-0.62618	0.148720006	-4.21045	2.549E-05	0.001284	DOWN	yes	gi|940781872|ref|XP_006108343.2|	405	8e-48	PREDICTED: transmembrane protein 258 isoform X1 [Myotis lucifugus]	gi|942070990|ref|XM_005901283.2|	447	0	PREDICTED: Bos mutus transmembrane protein 258 (TMEM258), mRNA	Q32P84	124	2e-35	Transmembrane protein 258	.	.	.	.	pfam05251	UPF0197	Uncharacterized protein family (UPF0197). This family of proteins is functionally uncharacterized.	.	GO:0016021|integral component of membrane;	.	.	.
IPO9	1396.471	0.55401	0.131652064	4.208136	2.575E-05	0.001292	Not_Sig	no	gi|982996686|ref|XP_003584743.2|	5091	0	PREDICTED: LOW QUALITY PROTEIN: importin-9 isoform X2 [Bos taurus]	gi|982996685|ref|XM_003584695.4|	3768	0	PREDICTED: Bos taurus importin 9 (IPO9), transcript variant X2, mRNA	Q96P70	1933	0	Importin-9	SPAC22H10.03c	186	4.50e-47	COG5657	pfam03810	IBN_N	Importin-beta N-terminal domain.	GO:0006606|protein import into nucleus;GO:0042991|transcription factor import into nucleus;	GO:0005737|cytoplasm;GO:0005829|cytosol;GO:0016020|membrane;GO:0005635|nuclear envelope;	GO:0042393|histone binding;GO:0008565|protein transporter	.	.
CTHRC1	8539.182	-0.42598	0.101310478	-4.20468	2.614E-05	0.001306	Not_Sig	no	gi|470651516|ref|XP_004328981.1|	1141	3e-151	PREDICTED: collagen triple helix repeat-containing protein 1-like [Tursiops truncatus]	gi|402692018|ref|NM_001081536.2|	1397	0	Bos taurus collagen triple helix repeat containing 1 (CTHRC1), mRNA	Q96CG8	431	3e-148	Collagen triple helix repeat-containing protein 1	.	.	.	.	pfam01391	Collagen	Collagen triple helix repeat (20 copies). Members of this family belong to the collagen superfamily. Collagens are generally	GO:0016477|cell migration;GO:0090103|cochlea morphogenesis;GO:0090177|establishment of planar polarity involved in neural tube closure;GO:0060122|inner ear receptor stereocilium organization;GO:0090090|negative regulation of canonical Wnt signaling pathway;GO:0043932|ossification involved in bone remodeling;GO:0045669|positive regulation of osteoblast differentiation;GO:0033690|positive regulation of osteoblast proliferation;GO:0032092|positive regulation of protein	GO:0005581|collagen trimer;GO:0005737|cytoplasm;GO:0005615|extracellular space;GO:0005578|proteinaceous	.	.	.
GNG10	1283.038	-0.51337	0.122243117	-4.19958	2.674E-05	0.001327	Not_Sig	no	gi|63055067|ref|NP_001017998.1|	298	8e-30	guanine nucleotide-binding protein G(I)/G(S)/G(O) subunit gamma-10 precursor [Homo sapiens]	gi|982934384|ref|XR_001500918.1|	1389	0	PREDICTED: Bos taurus guanine nucleotide binding protein (G protein), gamma 10 (GNG10), transcript variant X1, misc_RNA	P50151	119	3e-31	Guanine nucleotide-binding protein G(I)/G(S)/G(O) subunit gamma-10	.	.	.	.	pfam00631	G-gamma	GGL domain. G-protein gamma like domains (GGL) are found in the gamma subunit of the heterotrimeric G protein complex	GO:0071377|cellular response to glucagon stimulus;GO:0007186|G-protein coupled receptor signaling pathway;GO:0007165|signal transduction;	GO:0070062|extracellular exosome;GO:0005834|heterotrimeric G-protein complex;GO:0005886|plasma membrane;	GO:0003924|GTPase activity;GO:0004871|signal transducer	K04545	GNG10; guanine nucleotide-binding protein G(I)/G(S)/G(O)
ATXN2L	2548.8	0.723648	0.172334376	4.199093	2.68E-05	0.001327	UP	yes	gi|329664983|ref|NP_001192459.1|	3928	0	ataxin-2-like protein [Bos taurus]	gi|594094567|ref|XM_006070522.1|	4064	0	PREDICTED: Bubalus bubalis ataxin 2-like (ATXN2L), transcript variant X2, mRNA	Q8WWM7	1402	0	Ataxin-2-like protein	.	.	.	.	pfam14438	SM-ATX	Ataxin 2 SM domain. This SM domain is found in Ataxin-2.	GO:0010603|regulation of cytoplasmic mRNA processing body assembly;GO:0034063|stress granule assembly;	GO:0005913|cell-cell adherens junction;GO:0005737|cytoplasm;GO:0010494|cytoplasmic stress granule;GO:0016020|membrane;GO:00166	GO:0098641|cadherin binding involved in cell-cell adhesion;GO:00448	.	.
C10H15orf48	277.5002	-0.73489	0.175108612	-4.19679	2.707E-05	0.001335	DOWN	yes	gi|555954549|ref|XP_005889441.1|	426	6e-50	PREDICTED: normal mucosa of esophagus-specific gene 1 protein [Bos mutus]	gi|742156865|ref|XM_010847755.1|	698	0	PREDICTED: Bison bison bison chromosome unknown open reading frame, human C15orf48 (LOC104994238), mRNA	Q9C002	142	1e-41	Normal mucosa of esophagus-specific gene 1 protein	.	.	.	.	pfam06522	B12D	NADH-ubiquinone reductase complex 1 MLRQ subunit. The MLRQ subunit of mitochondrial NADH-ubiquinone reductase	GO:1902600|hydrogen ion transmembrane transport;	GO:0005751|mitochondrial respiratory chain complex IV;GO:0005634|nucleus;	GO:0008137|NADH dehydrogenase (ubiquinone) activity;	.	.

CRIPT	645.2697	-0.66144	0.157685285	-4.19468	2.733E-05	0.001342	DOWN	yes	gi|488510613|ref|XP_004447392.1|	565	2e-64	PREDICTED: cysteine-rich PDZ-binding protein [Dasypus novemcinctus]	gi|742112803|ref|XM_010838635.1|	1210	0	PREDICTED: Bison bison bison cysteine-rich PDZ-binding protein (CRIPT), mRNA	Q9P021	209	4e-63	Cysteine-rich PDZ-binding protein	.	.	.	.	pfam10235	Cript	Microtubule-associated protein CRIPT. The CRIPT protein is a cytoskeletal protein involved in microtubule production. The C-	GO:0031122|cytoplasmic microtubule organization;GO:0045184|establishment of protein localization;GO:0035372|protein localization to microtubule;GO:1902897|regulation of postsynaptic density protein 95 clustering;	GO:0030054|cell junction;GO:0005737|cytoplasm;GO:0030425|dendrite;GO:0043198|dendritic shaft;GO:0043197|dendritic	GO:0008017|microtubule binding;GO:0030165|PDZ domain	.	.
TMEM160	353.6693	-0.59755	0.142588175	-4.19073	2.781E-05	0.00136	DOWN	yes	gi|73948101|ref|XP_533633.2|	617	1e-77	PREDICTED: transmembrane protein 160 [Canis lupus familiaris]	gi|402744959|ref|NM_001046019.2|	639	0	Bos taurus transmembrane protein 160 (TMEM160), mRNA	Q24JY6	241	1e-78	Transmembrane protein 160	.	.	.	.	pfam12004	DUF3498	Domain of unknown function (DUF3498). This presumed domain is functionally uncharacterized.	.	GO:0016021|integral component of membrane;GO:0005739|mitochondrion;	.	.	.
GRINA	718.2255	0.717061	0.171310559	4.185739	2.842E-05	0.001372	UP	yes	gi|594032747|ref|XP_006041131.1|	1005	2e-127	PREDICTED: protein lifeguard 1 [Bubalus bubalis]	gi|83035044|ref|NM_001037593.1|	1737	0	Bos taurus glutamate receptor, ionotropic, N-methyl D-aspartate-associated protein 1 (glutamate binding) (GRINA), mRNA	Q32L53	390	1e-128	Protein lifeguard 1	.	.	.	.	pfam01027	Bax1-I	This domain is found in Inhibitor of apoptosis-promoting Bax1. Programmed cell-death involves a set of Bcl-2 family proteins, some of which inhibit	GO:0032469|endoplasmic reticulum calcium ion homeostasis;GO:1902236|negative regulation of endoplasmic reticulum stress-induced intrinsic apoptotic signaling pathway;	GO:0005783|endoplasmic reticulum;GO:0005794|Golgi apparatus;GO:0016021|integral component of membrane;	.	K06890	K06890; uncharacterized protein
NBEAL1	573.2608	-0.71911	0.171823706	-4.18519	2.849E-05	0.001372	DOWN	yes	gi|742207076|ref|XP_010859309.1|	13961	0	PREDICTED: neurobeachin-like protein 1 isoform X2 [Bison bison bison]	gi|742207083|ref|XM_010861009.1|	10370	0	PREDICTED: Bison bison bison neurobeachin-like 1 (NBEAL1), transcript variant X8, mRNA	Q6ZS30	5052	0	Neurobeachin-like protein 1	.	.	.	.	pfam02138	Beach	Beige/BEACH domain.	.	GO:0012505|endomembrane system;GO:0019898|extrinsic component of membrane;	GO:0005543|phospholipid binding;	.	.
PML	381.5326	0.661735	0.158118931	4.185048	2.851E-05	0.001372	UP	yes	gi|528999217|ref|XP_005221963.1|	4313	0	PREDICTED: protein PML isoform X2 [Bos taurus]	gi|594045966|ref|XM_006047384.1|	4506	0	PREDICTED: Bubalus bubalis promyelocytic leukemia (PML), transcript variant X1, mRNA	P29590	1238	0	Protein PML	.	.	.	.	pfam12126	DUF3583	Protein of unknown function (DUF3583). This domain is found in eukaryotes, and is typically between 302 and 338 amino acids	GO:0006919|activation of cysteine-type endopeptidase activity involved in apoptotic process;GO:0006915|apoptotic process;GO:0060444|branching involved in mammary gland duct morphogenesis;GO:0007050|cell cycle arrest;GO:0045165|cell fate commitment;GO:0071353|cellular response to interleukin-4;GO:0090398|cellular senescence;GO:0032922|circadian regulation of gene expression;GO:0007182|common-partner SMAD protein phosphorylation;GO:0051607|defense response to virus;GO:0006977|DNA damage	GO:0005737|cytoplasm;GO:0005829|cytosol;GO:0031901|early endosome membrane;GO:0042406|extrinsic component of endoplasmic reticulum	GO:0050897|cobalt ion binding;GO:0003677|DNA	K10054	PML, TRIM19; probable transcription factor PML
MLLT1	471.7368	0.591948	0.141451258	4.184822	2.854E-05	0.001372	UP	yes	gi|982984803|ref|XP_015319601.1|	2223	0	PREDICTED: protein ENL isoform X3 [Bos taurus]	gi|982984802|ref|XM_015464115.1|	2714	0	PREDICTED: Bos taurus myeloid/lymphoid or mixed-lineage leukemia; translocated to, 1 (MLLT1), transcript variant X3, mRNA	Q03111	755	0	Protein ENL	YNL107w	84.7	1.49e-17	COG5033	pfam03366	YEATS	YEATS family. We have named this family the YEATS family, after `YNK7', `ENL', `AF-9', and `TFIIF small subunit'. This family also	GO:0006469|negative regulation of protein kinase activity;GO:0006355|regulation of transcription, DNA-templated;GO:0006366|transcription from RNA polymerase II promoter;	GO:0005737|cytoplasm;GO:0005730|nucleolus;GO:0005654|nucleoplasm;GO:0005634|nucleus;GO:0008023|transcription elongation factor complex;	GO:0003677|DNA binding;	K15187	MLLT1_3, ENL, AF9; YEATS domain-containing protein 1/3
RPL11	3314.215	-0.57435	0.137414113	-4.17968	2.919E-05	0.001398	Not_Sig	no	gi|725552974|ref|XP_010347552.1|	941	3e-127	PREDICTED: 60S ribosomal protein L11 isoform X1 [Saimiri boliviensis boliviensis]	gi|942082977|ref|XM_005905165.2|	595	0	PREDICTED: Bos mutus ribosomal protein L11 (RPL11), transcript variant X2, mRNA	P62914	360	5e-126	60S ribosomal protein L11	SPBC17G9.10	267	6.96e-92	COG0094	pfam00673	Ribosomal_L5_C	ribosomal L5P family C-terminus. This region is found associated with pfam00281.	GO:0043433|negative regulation of sequence-specific DNA binding transcription factor activity;GO:0034504|protein localization to nucleus;GO:0000027|ribosomal large subunit assembly;GO:0006412|translation;	GO:0022625|cytosolic large ribosomal subunit;GO:0022626|cytosolic ribosome;GO:0005730|nucleolus;	GO:0042975|peroxisome proliferator activated receptor binding;GO:0019843	.	.
TMEM50A	840.875	-0.51049	0.122311785	-4.17366	2.997E-05	0.001429	Not_Sig	no	gi|593746620|ref|XP_007128621.1|	818	6e-106	PREDICTED: transmembrane protein 50A isoform X2 [Physeter catodon]	gi|942054719|ref|XM_005895853.2|	1113	0	PREDICTED: Bos mutus transmembrane protein 50A (TMEM50A), transcript variant X1, mRNA	O95807	309	2e-103	Transmembrane protein 50A	.	.	.	.	pfam05255	UPF0220	Uncharacterized protein family (UPF0220). This family of proteins is functionally uncharacterized.	.	GO:0005783|endoplasmic reticulum;GO:0016021|integral component of membrane;	.	.	.
ITGAV	33041.33	-0.5843	0.140082089	-4.17111	3.031E-05	0.001439	Not_Sig	no	gi|528938720|ref|XP_005202309.1|	5311	0	PREDICTED: integrin alpha-V isoform X1 [Bos taurus]	gi|982913004|ref|XM_005202252.3|	7099	0	PREDICTED: Bos taurus integrin subunit alpha V (ITGAV), transcript variant X1, mRNA	P80746	2046	0	Integrin alpha-V	.	.	.	.	pfam08441	Integrin_alpha2	Integrin alpha. This domain is found in integrin alpha and integrin alpha precursors to the C terminus of a number of	GO:0001568|blood vessel development;GO:0033627|cell adhesion mediated by integrin;GO:0007229|integrin-mediated signaling pathway;	GO:0005925|focal adhesion;GO:0008305|integrin complex;	GO:0046872|metal ion binding;GO:0001618|virus receptor	K06487	ITGAV; integrin alpha V
EHD2	4359.308	0.557349	0.133814519	4.165084	3.112E-05	0.001472	Not_Sig	no	gi|444730643|gb|ELW71018.1|	2798	0	EH domain-containing protein 2 [Tupaia chinensis]	gi|942070077|ref|XM_005900922.2|	3047	0	PREDICTED: Bos mutus EH-domain containing 2 (EHD2), transcript variant X1, mRNA	Q9NZN4	1068	0	EH domain-containing protein 2	.	.	.	.	pfam00350	Dynamin_N	Dynamin family.	GO:0007596|blood coagulation;GO:0030866|cortical actin cytoskeleton organization;GO:0032456|endocytic recycling;GO:0006897|endocytosis;GO:0097320|membrane tubulation;GO:2001137|positive regulation of endocytic recycling;GO:1901741|positive regulation of myoblast fusion;GO:0072659|protein localization to plasma membrane;	GO:0005901|caveola;GO:0005829|cytosol;GO:0010008|endosome membrane;GO:0070062|extracellular exosome;GO:0019898|extrinsic component	GO:0005524|ATP binding;GO:0005509|calcium ion binding;GO:0005525	K12469	EHD2; EH domain-containing protein 2
MCL1	7098.719	-0.37514	0.090116754	-4.1628	3.144E-05	0.001479	Not_Sig	no	gi|149642835|ref|NP_001092676.1|	1478	0	induced myeloid leukemia cell differentiation protein Mcl-1 [Bos taurus]	gi|149642834|ref|NM_001099206.1|	3665	0	Bos taurus myeloid cell leukemia 1 (MCL1), mRNA	Q7YRZ9	513	4e-169	Induced myeloid leukemia cell differentiation protein Mcl-1 homolog	.	.	.	.	pfam00452	Bcl-2	Apoptosis regulator proteins, Bcl-2 family.	GO:0030154|cell differentiation;GO:0097192|extrinsic apoptotic signaling pathway in absence of ligand;GO:0008630|intrinsic apoptotic signaling pathway in response to DNA damage;GO:0007275|multicellular organismal development;GO:2001243|negative regulation of intrinsic apoptotic signaling pathway;	GO:0005829|cytosol;GO:0016021|integral component of membrane;GO:0016020|membrane;GO:0005741|mitochondrial outer	GO:0046982|protein heterodimerization activity;GO:0042803	K02539	MCL1; myeloid leukemia cell differntiation protein MCL-1
LAMA1	922.8949	0.429037	0.103080604	4.162152	3.153E-05	0.001479	Not_Sig	no	gi|742199022|ref|XP_010857399.1|	15728	0	PREDICTED: laminin subunit alpha-1 isoform X2 [Bison bison bison]	gi|742199021|ref|XM_010859097.1|	9252	0	PREDICTED: Bison bison bison laminin, alpha 1 (LAMA1), transcript variant X2, mRNA	P25391	4959	0	Laminin subunit alpha-1	.	.	.	.	pfam00055	Laminin_N	Laminin N-terminal (Domain VI).	GO:0007155|cell adhesion;GO:0007166|cell surface receptor signaling pathway;GO:0030198|extracellular matrix organization;GO:0030155|regulation of cell adhesion;GO:0030334|regulation of cell migration;GO:0045995|regulation of embryonic development;	GO:0005604|basement membrane;GO:0031012|extracellular matrix;GO:0005576|extracellular region;GO:0005615|extracellular	GO:0005201|extracellular matrix structural constituent;	K05637	LAMA1_2; laminin, alpha 1/2
ZDHHC5	1189.995	0.571859	0.137542463	4.15769	3.215E-05	0.0015	Not_Sig	no	gi|300795464|ref|NP_001179692.1|	3277	0	palmitoyltransferase ZDHHC5 [Bos taurus]	gi|741953171|ref|XM_005200959.2|	4470	0	PREDICTED: Bos taurus zinc finger, DHHC-type containing 5 (ZDHHC5), transcript variant X1, mRNA	E1BLT8	1266	0	Palmitoyltransferase ZDHHC5	SPBC3H7.09	129	1.54e-31	COG5273	pfam01529	zf-DHHC	DHHC palmitoyltransferase. This family includes the well known DHHC zinc binding domain as well as three of the four conserved	GO:0018345|protein palmitoylation;	GO:0005737|cytoplasm;GO:0030425|dendrite;GO:0016021|integral component of membrane;GO:0005886|plasma membrane;	GO:0016409|palmitoyltransferase activity;GO:0019706|protein-cysteine S-	.	.
PRPF8	4880.26	0.424175	0.10203738	4.157054	3.224E-05	0.0015	Not_Sig	no	gi|521020961|gb|EPQ02749.1|	12211	0	Pre-mRNA-processing-splicing factor 8 [Myotis brandtii]	gi|555952226|ref|XM_005888241.1|	7330	0	PREDICTED: Bos mutus pre-mRNA processing factor 8 (PRPF8), transcript variant X2, mRNA	Q6P2Q9	4703	0	Pre-mRNA-processing-splicing factor 8	SPAC4F8.12c	3502	0	COG5178	pfam08083	PROCN	PROCN (NUC071) domain. The PROCN domain is the central domain in pre-mRNA splicing factors of PRO8 family.	GO:0071222|cellular response to lipopolysaccharide;GO:0071356|cellular response to tumor necrosis factor;GO:0006397|mRNA processing;GO:0000398|mRNA splicing, via spliceosome;GO:0008380|RNA splicing;GO:0000375|RNA splicing, via transesterification reactions;GO:0000244|spliceosomal tri-snRNP complex assembly;	GO:0071013|catalytic step 2 spliceosome;GO:0016020|membrane;GO:0016607|nuclear speck;GO:0005654|nucleoplasm;GO:000563	GO:0070530|K63-linked polyubiquitin binding;GO:0044822|poly(A) RNA	K12856	PRPF8, PRP8; pre-mRNA-processing factor 8
TNIP1	5069.402	0.472212	0.113618828	4.156104	3.237E-05	0.0015	Not_Sig	no	gi|742166966|ref|XP_010848887.1|	2597	0	PREDICTED: TNFAIP3-interacting protein 1 isoform X1 [Bison bison bison]	gi|742166965|ref|XM_010850585.1|	2572	0	PREDICTED: Bison bison bison TNFAIP3 interacting protein 1 (TNIP1), transcript variant X1, mRNA	Q15025	848	0	TNFAIP3-interacting protein 1	.	.	.	.	pfam12128	DUF3584	Protein of unknown function (DUF3584). This protein is found in bacteria and eukaryotes. Proteins in this family are typically between	GO:0006952|defense response;GO:0009101|glycoprotein biosynthetic process;GO:0006954|inflammatory response;GO:0007159|leukocyte cell-cell adhesion;GO:0085032|modulation by symbiont of host I-kappaB kinase/NF-kappaB cascade;GO:0002755|MyD88-dependent toll-like receptor signaling pathway;GO:0070373|negative regulation of ERK1 and ERK2 cascade;GO:0043124|negative regulation of I-kappaB kinase/NF-kappaB signaling;GO:0045071|negative regulation of viral genome replication;GO:0050729|positive regulation of inflammatory	GO:0005737|cytoplasm;GO:0005622|intracellular;GO:0005654|nucleoplasm;	GO:0051019|mitogen-activated protein kinase binding;	.	.
LOC102269855	85.71822	-0.95822	0.230811848	-4.15153	3.303E-05	0.001524	DOWN	yes	gi|512958236|ref|XP_004841425.1|	368	7e-42	PREDICTED: guanine nucleotide-binding protein G(I)/G(S)/G(O) subunit gamma-5 [Heterocephalus glaber]	gi|742216542|ref|XM_010863350.1|	491	0	PREDICTED: Bison bison bison guanine nucleotide-binding protein G(I)/G(S)/G(O) subunit gamma-5 (LOC105005375), mRNA	P63219	137	2e-40	Guanine nucleotide-binding protein G(I)/G(S)/G(O) subunit gamma-5	.	.	.	.	pfam00631	G-gamma	GGL domain. G-protein gamma like domains (GGL) are found in the gamma subunit of the heterotrimeric G protein complex	GO:0007186|G-protein coupled receptor signaling pathway;	GO:0070062|extracellular exosome;GO:0031680|G-protein beta/gamma-subunit complex;GO:0005834|heterotrimeric G-	GO:0004871|signal transducer activity;	K04542	GNG5; guanine nucleotide-binding protein G(I)/G(S)/G(O)
PPP2R5D	874.0433	0.495366	0.119357085	4.150285	3.321E-05	0.001526	Not_Sig	no	gi|478499720|ref|XP_004424185.1|	2965	0	PREDICTED: serine/threonine-protein phosphatase 2A 56 kDa regulatory subunit delta isoform [Ceratotherium simum simum]	gi|329744635|ref|NM_001206358.1|	2649	0	Bos taurus protein phosphatase 2 regulatory subunit B', delta (PPP2R5D), mRNA	Q14738	1123	0	Serine/threonine-protein phosphatase 2A 56 kDa regulatory subunit delta isoform	.	.	.	.	pfam01603	B56	Protein phosphatase 2A regulatory B subunit (B56 family). Protein phosphatase 2A (PP2A) is a major intracellular protein phosphatase	GO:0007399|nervous system development;GO:0007165|signal transduction;	GO:0005829|cytosol;GO:0005654|nucleoplasm;GO:0005634|nucleus;GO:0000159|protein phosphatase type 2A complex;	GO:0008601|protein phosphatase type 2A regulator activity;GO:0004722	K11584	PPP2R5; serine/threonine-protein phosphatase 2A regulatory
ERF	430.6734	0.721471	0.174027911	4.14572	3.387E-05	0.001539	UP	yes	gi|532105255|ref|XP_005338217.1|	1879	0	PREDICTED: ETS domain-containing transcription factor ERF [Ictidomys tridecemlineatus]	gi|942095039|ref|XM_005908976.2|	1865	0	PREDICTED: Bos mutus Ets2 repressor factor (ERF), transcript variant X1, mRNA	P70459	718	0	ETS domain-containing transcription factor ERF	.	.	.	.	pfam00178	Ets	Ets-domain.	GO:0060710|chorio-allantoic fusion;GO:0010668|ectodermal cell differentiation;GO:0001701|in utero embryonic development;GO:0000122|negative regulation of transcription from RNA polymerase II promoter;GO:0006357|regulation of transcription from RNA polymerase II promoter;GO:0006351|transcription, DNA-templated;GO:0060707|trophoblast giant cell differentiation;	GO:0005794|Golgi apparatus;GO:0005634|nucleus;	GO:0000981|RNA polymerase II transcription factor activity, sequence-	K09434	ERF; ETS domain-containing transcription factor ERF
TMEM263	2088.815	-0.534	0.128823384	-4.14524	3.395E-05	0.001539	Not_Sig	no	gi|602702544|ref|XP_007462931.1|	465	3e-50	PREDICTED: UPF0444 transmembrane protein C12orf23 homolog [Lipotes vexillifer]	gi|402692218|ref|NM_001034673.2|	3449	0	Bos taurus transmembrane protein 263 (TMEM263), mRNA	Q3SYV1	183	9e-52	Transmembrane protein 263	.	.	.	.	pfam15475	UPF0444	Transmembrane protein C12orf23, UPF0444. This family of proteins is found in eukaryotes. Proteins in this family are typically between	.	GO:0016021|integral component of membrane;	.	.	.
NACC1	1351.388	0.549763	0.132639301	4.1448	3.401E-05	0.001539	Not_Sig	no	gi|300797615|ref|NP_001179541.1|	2341	0	nucleus accumbens-associated protein 1 [Bos taurus]	gi|982929967|ref|XM_005208611.3|	4275	0	PREDICTED: Bos taurus nucleus accumbens associated 1, BEN and BTB (POZ) domain containing (NACC1), transcript variant X1, mRNA	Q96RE7	856	0	Nucleus accumbens-associated protein 1	.	.	.	.	pfam00651	BTB	BTB/POZ domain. The BTB (for BR-C, ttk and bab) or POZ (for Pox virus and Zinc finger) domain is present near the N-terminus of a	GO:0045892|negative regulation of transcription, DNA-templated;GO:0008284|positive regulation of cell proliferation;GO:0051260|protein homooligomerization;GO:0006351|transcription, DNA-templated;	GO:0005737|cytoplasm;GO:0016604|nuclear body;GO:0005634|nucleus;	.	K10486	BTBD14; BTB/POZ domain-containing protein 14
LUM	6112.327	-0.43668	0.105355917	-4.14476	3.402E-05	0.001539	Not_Sig	no	gi|555996837|ref|XP_005910152.1|	1580	0	PREDICTED: lumican [Bos mutus]	gi|942098207|ref|XM_005910090.2|	1997	0	PREDICTED: Bos mutus lumican (LUM), transcript variant X1, mRNA	Q05443	613	0	Lumican	alr0124_1	75.1	2.00e-13	COG4886	pfam13855	LRR_8	Leucine rich repeat.	GO:0030199|collagen fibril organization;GO:0045944|positive regulation of transcription from RNA polymerase II promoter;GO:0032914|positive regulation of transforming growth factor beta1 production;GO:0007601|visual perception;	GO:0070062|extracellular exosome;GO:0005615|extracellular space;GO:0005583|fibrillar collagen trimer;GO:0005578|proteinaceous	GO:0005518|collagen binding;	K08122	LUM; lumican
LRRC58	744.8822	-0.60217	0.14542963	-4.14063	3.464E-05	0.001556	DOWN	yes	gi|332164692|ref|NP_001193689.1|	1296	4e-157	leucine-rich repeat-containing protein 58 [Bos taurus]	gi|850474981|gb|CP011886.1|	5005	0	Ovis canadensis canadensis isolate 43U chromosome 1 sequence	Q96CX6	489	2e-153	Leucine-rich repeat-containing protein 58	.	.	.	.	pfam13855	LRR_8	Leucine rich repeat.	.	.	.	.	.
NOTCH1	1900.557	0.555875	0.134255598	4.140423	3.467E-05	0.001556	Not_Sig	no	gi|594077088|ref|XP_006062272.1|	12208	0	PREDICTED: neurogenic locus notch homolog protein 1 [Bubalus bubalis]	gi|982991464|ref|XM_015465548.1|	9247	0	PREDICTED: Bos taurus notch 1 (NOTCH1), transcript variant X2, mRNA	P46531	4296	0	Neurogenic locus notch homolog protein 1	all2748	88.2	7.16e-17	COG0666	pfam06816	NOD	NOTCH protein. NOTCH signalling plays a fundamental role during a great number of developmental processes in multicellular animals.	GO:0031100|organ regeneration;GO:0003180|aortic valve morphogenesis;GO:1902263|apoptotic process involved in embryonic digit morphogenesis;GO:0060842|arterial endothelial cell differentiation;GO:0048708|astrocyte differentiation;GO:0003162|atrioventricular node development;GO:0003181|atrioventricular valve morphogenesis;GO:0009912|auditory receptor cell fate commitment;GO:0007409|axonogenesis;GO:0048754|branching morphogenesis of an epithelial tube;GO:0003209|cardiac atrium	GO:0001669|acrosomal vesicle;GO:0005912|adherens junction;GO:0016324|apical plasma membrane;GO:0009986|cell	GO:0005509|calcium ion binding;GO:0031490|chromatin DNA	K02599	NOTCH; Notch
KLHL24	474.0832	-0.57706	0.139668474	-4.13163	3.602E-05	0.00161	Not_Sig	no	gi|26381170|dbj|BAB29759.2|	3207	0	unnamed protein product [Mus musculus]	gi|982910974|ref|XM_010801303.2|	6596	0	PREDICTED: Bos taurus kelch like family member 24 (KLHL24), transcript variant X3, mRNA	Q6TFL4	1214	0	Kelch-like protein 24	.	.	.	.	pfam07707	BACK	BTB And C-terminal Kelch. This domain is found associated with pfam00651 and pfam01344. The BACK domain is found juxtaposed	GO:0016567|protein ubiquitination;GO:2000312|regulation of kainate selective glutamate receptor activity;	GO:0030424|axon;GO:0031463|Cul3-RING ubiquitin ligase complex;GO:0005737|cytoplasm;GO:0043204|perikaryon;	.	K10461	KLHL24_35; kelch-like protein 24/35
E2F1	340.8654	0.597913	0.144893788	4.126559	3.682E-05	0.001628	UP	yes	gi|329663321|ref|NP_001193008.1|	1641	0	transcription factor E2F1 [Bos taurus]	gi|965798175|ref|XM_012114953.2|	2195	0	PREDICTED: Ovis aries musimon E2F transcription factor 1 (E2F1), transcript variant X2, mRNA	Q01094	592	0	Transcription factor E2F1	.	.	.	.	pfam02319	E2F_TDP	E2F/DP family winged-helix DNA-binding domain. This family contains the transcription factor E2F and its dimerization partners	GO:0043276|anoikis;GO:0071398|cellular response to fatty acid;GO:0071456|cellular response to hypoxia;GO:1990090|cellular response to nerve growth factor stimulus;GO:0071466|cellular response to xenobiotic stimulus;GO:0000077|DNA damage checkpoint;GO:0006977|DNA damage response, signal transduction by p53 class mediator resulting in cell cycle arrest;GO:0030900|forebrain development;GO:0072332|intrinsic apoptotic signaling pathway by p53 class mediator;GO:0008630|intrinsic apoptotic signaling pathway in	GO:0005739|mitochondrion;GO:0000790|nuclear chromatin;GO:0005654|nucleoplasm;GO:0005634|nucleus;GO:0035189|Rb-E2F	GO:0001047|core promoter binding;GO:0003677|DNA	K17454	E2F1; transcription factor E2F1
NAV1	1118.651	0.570715	0.138304353	4.126515	3.683E-05	0.001628	Not_Sig	no	gi|982996682|ref|XP_015322336.1|	9037	0	PREDICTED: LOW QUALITY PROTEIN: neuron navigator 1 isoform X4 [Bos taurus]	gi|982996681|ref|XM_015466850.1|	7015	0	PREDICTED: Bos taurus neuron navigator 1 (LOC531974), transcript variant X4, mRNA	Q8NEY1	2770	0	Neuron navigator 1	.	.	.	.	pfam09770	PAT1	Topoisomerase II-associated protein PAT1. Members of this family are necessary for accurate chromosome transmission during	GO:0001578|microtubule bundle formation;GO:0001764|neuron migration;	GO:0005737|cytoplasm;GO:0005874|microtubule;	.	K16776	NAV1; neuron navigator 1
RPL23	4499.045	-0.62447	0.151334748	-4.12643	3.684E-05	0.001628	DOWN	yes	gi|965930210|ref|XP_014950645.1|	735	1e-96	PREDICTED: 60S ribosomal protein L23-like, partial [Ovis aries]	gi|742135726|ref|XM_010842689.1|	527	0	PREDICTED: Bison bison bison ribosomal protein L23 (RPL23), mRNA	P62832	263	6e-89	60S ribosomal protein L23	YER117w	221	1.29e-74	COG0093	pfam00238	Ribosomal_L14	Ribosomal protein L14p/L23e.	GO:0006412|translation;	GO:0022625|cytosolic large ribosomal subunit;GO:0070062|extracellular exosome;GO:0005925|focal adhesion;GO:0016020|membrane;GO:0005	GO:0070180|large ribosomal subunit rRNA binding;GO:0044822	K02894	RP-L23e, RPL23; large subunit ribosomal protein L23e
FOXK1	187.0069	0.877814	0.212977334	4.121633	3.762E-05	0.001656	UP	yes	gi|741975469|ref|XP_010818016.1|	2846	0	PREDICTED: forkhead box protein K1 [Bos taurus]	gi|965833906|ref|XM_012132720.2|	1888	0	PREDICTED: Ovis aries musimon forkhead box K1 (FOXK1), transcript variant X2, mRNA	P85037	1037	0	Forkhead box protein K1	SPBC16G5.15c	112	1.06e-24	COG5025	pfam00250	Fork_head	Fork head domain.	GO:0030154|cell differentiation;GO:0007517|muscle organ development;GO:0030308|negative regulation of cell growth;GO:0045892|negative regulation of transcription, DNA-templated;GO:0045893|positive regulation of transcription, DNA-templated;GO:0006351|transcription, DNA-templated;	GO:0005634|nucleus;	GO:0000977|RNA polymerase II regulatory region sequence-specific	K09404	FOXK; forkhead box protein K
COPS9	650.1799	-0.69275	0.168119212	-4.12058	3.779E-05	0.001656	DOWN	yes	gi|803199039|ref|XP_004021528.2|	230	7e-22	PREDICTED: myeloma-overexpressed gene 2 protein isoform X1 [Ovis aries]	gi|742077763|ref|XM_010835936.1|	457	0	PREDICTED: Bison bison bison myeloma overexpressed 2 (MYEOV2), mRNA	Q6DDA3	84.7	2e-20	Myeloma-overexpressed gene 2 protein homolog	.	.	.	.	pfam15004	MYEOV2	Myeloma-overexpressed-like. This family of proteins is found in eukaryotes. It includes human myeloma-overexpressed gene 2	.	.	.	.	.
TMEM167B	587.0255	-0.55488	0.134682157	-4.11993	3.79E-05	0.001656	Not_Sig	no	gi|190360731|ref|NP_001121975.1|	374	9e-39	protein kish-B precursor [Bos taurus]	gi|942042254|ref|XM_005891325.2|	2735	0	PREDICTED: Bos mutus transmembrane protein 167B (TMEM167B), transcript variant X1, mRNA	Q80X45	148	3e-40	Protein kish-B	.	.	.	.	pfam06842	DUF1242	Protein of unknown function (DUF1242). This family consists of a number of eukaryotic proteins of around 72 residues in length. The	.	GO:0000139|Golgi membrane;GO:0016021|integral component of membrane;	.	.	.
MRTFA	1534.585	0.683876	0.166090754	4.117481	3.83E-05	0.001665	UP	yes	gi|803264280|ref|XP_011991856.1|	3989	0	PREDICTED: MKL/myocardin-like protein 1 isoform X2 [Ovis aries musimon]	gi|982982678|ref|XM_010827987.2|	4227	0	PREDICTED: Bos taurus megakaryoblastic leukemia (translocation) 1 (MKL1), transcript variant X3, mRNA	Q8K4J6	766	0	MKL/myocardin-like protein 1	.	.	.	.	pfam02037	SAP	SAP domain. The SAP (after SAF-A/B, Acinus and PIAS) motif is a putative DNA/RNA binding domain found in diverse nuclear and	GO:0030036|actin cytoskeleton organization;GO:0030900|forebrain development;GO:2001234|negative regulation of apoptotic signaling pathway;GO:0043154|negative regulation of cysteine-type endopeptidase activity involved in apoptotic process;GO:0001764|neuron migration;GO:0031175|neuron projection development;GO:0045944|positive regulation of transcription from RNA polymerase II promoter;GO:0010735|positive regulation of transcription via serum response element binding;GO:0045893|positive regulation of	GO:0005737|cytoplasm;GO:0005654|nucleoplasm;GO:0005634|nucleus;	GO:0003779|actin binding;GO:0003785|actin monomer binding;GO:0043522	.	.
BMPR2	1295.05	-0.47107	0.11443879	-4.11638	3.849E-05	0.001665	Not_Sig	no	gi|747165293|ref|NP_001291214.1|	4892	0	bone morphogenetic protein receptor type-2 precursor [Bos taurus]	gi|942080206|ref|XM_005904256.2|	11087	0	PREDICTED: Bos mutus bone morphogenetic protein receptor, type II (serine/threonine kinase) (BMPR2), mRNA	O35607	1831	0	Bone morphogenetic protein receptor type-2	.	.	.	.	pfam00069	Pkinase	Protein kinase domain.	GO:0009952|anterior/posterior pattern specification;GO:0060840|artery development;GO:0060413|atrial septum morphogenesis;GO:0001974|blood vessel remodeling;GO:0030509|BMP signaling pathway;GO:0007420|brain development;GO:0071773|cellular response to BMP stimulus;GO:0009267|cellular response to starvation;GO:0002063|chondrocyte development;GO:0003197|endocardial cushion development;GO:0060350|endochondral bone morphogenesis;GO:0072577|endothelial cell	GO:0016324|apical plasma membrane;GO:0009925|basal plasma membrane;GO:0005901|caveola;GO:0009986|cell surface;GO:0005911|cell-cell	GO:0005524|ATP binding;GO:0036122|BMP binding;GO:0098821	K04671	BMPR2; bone morphogenetic protein receptor type-2 [EC:2.7.11.30]
FAM199X	327.0029	-0.74912	0.182002502	-4.11597	3.856E-05	0.001665	DOWN	yes	gi|329664488|ref|NP_001193166.1|	1525	0	protein FAM199X [Bos taurus]	gi|965863129|ref|XM_012146086.2|	4466	0	PREDICTED: Ovis aries musimon family with sequence similarity 199, X-linked (FAM199X), transcript variant X1, mRNA	Q6PEV8	568	0	Protein FAM199X	.	.	.	.	pfam11208	DUF2992	Protein of unknown function (DUF2992). This bacterial family of proteins has no known function. However, the cis-regulatory yjdF	.	.	.	.	.
TMEM59	1409.691	-0.54324	0.132140341	-4.11105	3.939E-05	0.001685	Not_Sig	no	gi|942058402|ref|XP_014334152.1|	1483	0	PREDICTED: transmembrane protein 59 isoform X1 [Bos mutus]	gi|942058401|ref|XM_014478666.1|	1562	0	PREDICTED: Bos mutus transmembrane protein 59 (TMEM59), transcript variant X1, mRNA	Q3T0Q2	539	0	Transmembrane protein 59	.	.	.	.	pfam12280	BSMAP	Brain specific membrane anchored protein. This family of proteins is found in eukaryotes. Proteins in this family are typically between	GO:0006914|autophagy;GO:0090005|negative regulation of establishment of protein localization to plasma membrane;GO:0090285|negative regulation of protein glycosylation in Golgi;GO:0010955|negative regulation of protein processing;GO:0010508|positive regulation of autophagy;	GO:0070062|extracellular exosome;GO:0000137|Golgi cis cisterna;GO:0005797|Golgi medial cisterna;GO:0000139|Golgi	GO:0004175|endopeptidase activity;	.	.
MARK2	689.2616	0.563632	0.137143894	4.109783	3.96E-05	0.001685	Not_Sig	no	gi|555964263|ref|XP_005894201.1|	4083	0	PREDICTED: serine/threonine-protein kinase MARK2 isoform X1 [Bos mutus]	gi|965802384|ref|XM_012117030.2|	4617	0	PREDICTED: Ovis aries musimon serine/threonine-protein kinase MARK2 (LOC101121302), transcript variant X10, mRNA	Q7KZI7	1551	0	Serine/threonine-protein kinase MARK2	.	.	.	.	pfam00069	Pkinase	Protein kinase domain.	GO:0032147|activation of protein kinase activity;GO:0030010|establishment of cell polarity;GO:0045197|establishment or maintenance of epithelial cell apical/basal polarity;GO:0035556|intracellular signal transduction;GO:0000226|microtubule cytoskeleton organization;GO:0051646|mitochondrion localization;GO:0000422|mitophagy;GO:0001764|neuron migration;GO:0018107|peptidyl-threonine phosphorylation;GO:0010976|positive regulation of neuron projection development;GO:0046777|protein	GO:0005884|actin filament;GO:0005913|cell-cell adherens junction;GO:0016328|lateral plasma membrane;GO:0016020|membrane;GO:009	GO:0005524|ATP binding;GO:0098641|cadherin binding involved in cell-cell	K08798	MARK; MAP/microtubule affinity-regulating kinase [EC:2.7.11.1]
MEGF8	1066.117	0.558903	0.136029129	4.108702	3.979E-05	0.001685	Not_Sig	no	gi|965902223|ref|XP_014945418.1|	12476	0	PREDICTED: multiple epidermal growth factor-like domains protein 8 isoform X2 [Ovis aries musimon]	gi|965902222|ref|XM_015089932.1|	9499	0	PREDICTED: Ovis aries musimon multiple EGF-like-domains 8 (MEGF8), transcript variant X2, mRNA	Q7Z7M0	4647	0	Multiple epidermal growth factor-like domains protein 8	.	.	.	.	pfam00431	CUB	CUB domain.	GO:0030509|BMP signaling pathway;GO:0042074|cell migration involved in gastrulation;GO:0060976|coronary vasculature development;GO:0097094|craniofacial suture morphogenesis;GO:0071907|determination of digestive tract left/right asymmetry;GO:0061371|determination of heart left/right asymmetry;GO:0060971|embryonic heart tube left/right pattern formation;GO:0003143|embryonic heart tube morphogenesis;GO:0030326|embryonic limb morphogenesis;GO:0048704|embryonic skeletal	GO:0070062|extracellular exosome;GO:0016021|integral component of membrane;GO:0005634|nucleus;	GO:0005509|calcium ion binding;	.	.
LRCH4	400.3483	0.61952	0.150795635	4.108342	3.985E-05	0.001685	UP	yes	gi|594044077|ref|XP_006046542.1|	2913	0	PREDICTED: leucine-rich repeat and calponin homology domain-containing protein 4 isoform X2 [Bubalus bubalis]	gi|594044076|ref|XM_006046480.1|	2564	0	PREDICTED: Bubalus bubalis leucine-rich repeats and calponin homology (CH) domain containing 4 (LRCH4), transcript variant X2, mRNA	O75427	945	0	Leucine-rich repeat and calponin homology domain-containing protein 4	MA2301_1	94	2.67e-20	COG4886	pfam00307	CH	Calponin homology (CH) domain. The CH domain is found in both cytoskeletal proteins and signal transduction proteins. The CH	GO:0007399|nervous system development;	GO:0016605|PML body;	.	.	.
MBD6	364.7645	0.796796	0.193963303	4.107971	3.991E-05	0.001685	UP	yes	gi|742115871|ref|XP_010837676.1|	1032	5e-117	PREDICTED: methyl-CpG-binding domain protein 6 [Bison bison bison]	gi|982981849|ref|XM_015463352.1|	4209	0	PREDICTED: Bos taurus methyl-CpG binding domain protein 6 (MBD6), transcript variant X7, mRNA	Q96DN6	253	8e-68	Methyl-CpG-binding domain protein 6	.	.	.	.	pfam09770	PAT1	Topoisomerase II-associated protein PAT1. Members of this family are necessary for accurate chromosome transmission during	.	GO:0010369|chromocenter;GO:0005730|nucleolus;GO:0005654|nucleoplasm;GO:0005634|nucleus;	GO:0003682|chromatin binding;	.	.
RECQL4	187.4121	0.772385	0.188051749	4.107301	4.003E-05	0.001685	UP	yes	gi|148229208|ref|NP_001091506.1|	5663	0	ATP-dependent DNA helicase Q4 [Bos taurus]	gi|402691803|ref|NM_001098037.2|	3782	0	Bos taurus RecQ helicase-like 4 (RECQL4), mRNA	O94761	1305	0	ATP-dependent DNA helicase Q4	recQ	229	4.21e-63	COG0514	pfam00270	DEAD	DEAD/DEAH box helicase. Members of this family include the DEAD and DEAH box helicases. Helicases are involved in	GO:0006284|base-excision repair;GO:0032508|DNA duplex unwinding;GO:0006310|DNA recombination;GO:0006281|DNA repair;GO:0006260|DNA replication;GO:0000733|DNA strand renaturation;GO:0006302|double-strand break repair;GO:0007275|multicellular organismal development;	GO:0005694|chromosome;GO:0005737|cytoplasm;GO:0016020|membrane;GO:0005634|nucleus;	GO:0036310|annealing helicase activity;GO:0005524|ATP	K10730	RECQL4; ATP-dependent DNA helicase Q4 [EC:3.6.4.12]

GIPC3	1876.861	0.535683	0.130423556	4.107256	4.004E-05	0.001685	Not_Sig	no	gi|594047032|ref|XP_006047944.1|	4163	0	PREDICTED: serine/arginine repetitive matrix protein 2-like isoform X1 [Bubalus bubalis]	gi|594047031|ref|XM_006047882.1|	3367	0	PREDICTED: Bubalus bubalis GIPC PDZ domain containing family, member 3 (GIPC3), transcript variant X1, mRNA	Q8TF64	408	3e-134	PDZ domain-containing protein GIPC3	.	.	.	.	pfam04652	DUF605	Vta1 like. Vta1 (VPS20-associated protein 1) is a positive regulator of Vps4. Vps4 is an ATPase that is required in the multivesicular body	.	.	.	.	.
NDUFB3	231.9734	-0.7857	0.191441391	-4.10414	4.058E-05	0.001702	DOWN	yes	gi|942097608|ref|XP_014338447.1|	608	1e-77	PREDICTED: NADH dehydrogenase [ubiquinone] 1 beta subcomplex subunit 3 isoform X1 [Bos mutus]	gi|942097607|ref|XM_014482961.1|	590	0	PREDICTED: Bos mutus NADH dehydrogenase (ubiquinone) 1 beta subcomplex, 3, 12kDa (NDUFB3), transcript variant X1, mRNA	Q02365	172	3e-53	NADH dehydrogenase [ubiquinone] 1 beta subcomplex subunit 3	.	.	.	.	pfam08122	NDUF_B12	NADH-ubiquinone oxidoreductase B12 subunit family. This family consists of the NADH-ubiquinone oxidoreductase B12 subunit	GO:0022900|electron transport chain;	GO:0070062|extracellular exosome;GO:0016021|integral component of membrane;GO:0005747|mitochondrial respiratory chain complex I;	.	K03959	NDUFB3; NADH dehydrogenase (ubiquinone) 1 beta subcomplex subunit
HIGD1A	446.2145	-0.6306	0.154274685	-4.08752	4.36E-05	0.001821	DOWN	yes	gi|440909903|gb|ELR59762.1|	437	2e-52	HIG1 domain family member 1A, partial [Bos mutus]	gi|742099063|ref|XM_010835432.1|	491	0	PREDICTED: Bison bison bison HIG1 hypoxia inducible domain family, member 1A (HIGD1A), mRNA	Q5NVQ1	155	1e-47	HIG1 domain family member 1A, mitochondrial	.	.	.	.	pfam04588	HIG_1_N	Hypoxia induced protein conserved region. This family is found in proteins thought to be involved in the response to	GO:0042149|cellular response to glucose starvation;GO:0071456|cellular response to hypoxia;GO:0090201|negative regulation of release of cytochrome c from mitochondria;GO:0055114|oxidation-reduction process;GO:0071902|positive regulation of protein serine/threonine kinase activity;	GO:0016021|integral component of membrane;GO:0005743|mitochondrial inner membrane;GO:0005634|nucleus;GO:00704	.	.	.
CASKIN2	321.7757	0.730228	0.178681791	4.086749	4.375E-05	0.001821	UP	yes	gi|300795631|ref|NP_001178177.1|	4566	0	caskin-2 [Bos taurus]	gi|803209363|ref|XM_012109770.1|	4352	0	PREDICTED: Ovis aries musimon CASK interacting protein 2 (CASKIN2), transcript variant X2, mRNA	Q8WXE0	1517	0	Caskin-2	all2748	90.5	7.08e-18	COG0666	pfam12796	Ank_2	Ankyrin repeats (3 copies).	GO:0007165|signal transduction;	GO:0005737|cytoplasm;	.	.	.
MEN1	504.1425	0.539129	0.132304179	4.074921	4.603E-05	0.001907	Not_Sig	no	gi|942050030|ref|XP_014333156.1|	2344	0	PREDICTED: menin [Bos mutus]	gi|982972009|ref|XM_005227153.3|	2688	0	PREDICTED: Bos taurus menin 1 (MEN1), transcript variant X1, mRNA	Q0P5I0	894	0	Menin	.	.	.	.	pfam05053	Menin	Menin. MEN1, the gene responsible for multiple endocrine neoplasia type 1, is a tumor suppressor gene that encodes a	GO:0006974|cellular response to DNA damage stimulus;GO:0034968|histone lysine methylation;GO:0000165|MAPK cascade;GO:0045786|negative regulation of cell cycle;GO:0008285|negative regulation of cell proliferation;GO:0045736|negative regulation of cyclin-dependent protein serine/threonine kinase activity;GO:0046329|negative regulation of JNK cascade;GO:0045668|negative regulation of osteoblast differentiation;GO:0043433|negative regulation of sequence-specific DNA binding transcription factor	GO:0000785|chromatin;GO:0032154|cleavage furrow;GO:0005829|cytosol;GO:0035097|histone methyltransferase	GO:0003682|chromatin binding;GO:0003690|double-stranded	K14970	MEN1, MNN1; menin
TMEM104	392.8082	0.722511	0.177332431	4.074333	4.615E-05	0.001907	UP	yes	gi|555992424|ref|XP_005907988.1|	2446	0	PREDICTED: transmembrane protein 104 isoform X1 [Bos mutus]	gi|742187256|ref|XM_010856131.1|	3177	0	PREDICTED: Bison bison bison transmembrane protein 104 (TMEM104), transcript variant X1, mRNA	Q8NE00	877	0	Transmembrane protein 104	.	.	.	.	pfam01490	Aa_trans	Transmembrane amino acid transporter protein. This transmembrane region is found in many amino acid transporters	.	GO:0016021|integral component of membrane;	.	.	.
SF3A2	561.7894	0.657101	0.161438714	4.070282	4.696E-05	0.001928	UP	yes	gi|958712945|ref|XP_014701195.1|	883	8e-111	PREDICTED: splicing factor 3A subunit 2 [Equus asinus]	gi|942034359|ref|XM_005888346.2|	1693	0	PREDICTED: Bos mutus splicing factor 3a, subunit 2, 66kDa (SF3A2), mRNA	Q6AXT8	348	1e-111	Splicing factor 3A subunit 2	SPBC21C3.05	204	1.18e-61	COG5246	pfam13388	DUF4106	Protein of unknown function (DUF4106). This family of proteins are found in large numbers in the Trichomonas vaginalis proteome.	GO:0010976|positive regulation of neuron projection development;GO:0000245|spliceosomal complex assembly;	GO:0071013|catalytic step 2 spliceosome;GO:0016607|nuclear speck;GO:0005686|U2 snRNP;GO:0071004|U2-type	GO:0044822|poly (A) RNA binding;GO:0008270|zinc ion binding;	K12826	SF3A2, SAP62; splicing factor 3A subunit 2
PRNP	4436.031	-0.44531	0.109408969	-4.07017	4.698E-05	0.001928	Not_Sig	no	gi|410110916|ref|NP_851358.2|	752	4e-87	major prion protein precursor [Bos taurus]	gi|1000297225|ref|NM_001271626.2|	4086	0	Bos taurus prion protein (PRNP), transcript variant 1, mRNA	P10279	294	1e-88	Major prion protein	.	.	.	.	pfam00377	Prion	Prion/Doppel alpha-helical domain. The prion protein is thought to be the infectious agent that causes transmissible	GO:0071280|cellular response to copper ion;GO:0035690|cellular response to drug;GO:0046007|negative regulation of activated T cell proliferation;GO:0043066|negative regulation of apoptotic process;GO:0070885|negative regulation of calcineurin-NFAT signaling cascade;GO:0032689|negative regulation of interferon-gamma production;GO:0032700|negative regulation of interleukin-17 production;GO:0032703|negative regulation of interleukin-2 production;GO:0001933|negative regulation of protein	GO:0031225|anchored component of membrane;GO:0009986|cell surface;GO:0005783|endoplasmic reticulum;GO:0070062|extracellular	GO:0005507|copper ion binding;	.	.
MCM3	809.3814	0.461006	0.113319603	4.068193	4.738E-05	0.001937	Not_Sig	no	gi|803188109|ref|XP_004018938.2|	4206	0	PREDICTED: DNA replication licensing factor MCM3 isoform X1 [Ovis aries]	gi|942057318|ref|XM_005896849.2|	3054	0	PREDICTED: Bos mutus minichromosome maintenance complex component 3 (MCM3), mRNA	A4FUD9	1573	0	DNA replication licensing factor MCM3	SPCC1682.02c	675	0	COG1241	pfam00493	MCM	MCM2/3/5 family.	GO:0007049|cell cycle;GO:0006270|DNA replication initiation;	GO:0042555|MCM complex;GO:0005634|nucleus;	GO:0005524|ATP binding;GO:0003677|DNA binding;GO:0003678	K02541	MCM3; DNA replication licensing factor MCM3 [EC:3.6.4.12]
PIMREG	356.4266	0.561943	0.138314981	4.06278	4.849E-05	0.001973	Not_Sig	no	gi|742155746|ref|XP_010845737.1|	1144	4e-152	PREDICTED: protein FAM64A [Bison bison bison]	gi|942087647|ref|XM_005906659.2|	1452	0	PREDICTED: Bos mutus family with sequence similarity 64, member A (FAM64A), mRNA	Q9BSJ6	350	3e-116	Protein FAM64A	.	.	.	.	pfam07326	DUF1466	Protein of unknown function (DUF1466). This family consists of several hypothetical mammalian proteins of around 240 residues in	GO:0051301|cell division;GO:0007067|mitotic nuclear division;	GO:0005737|cytoplasm;GO:0005730|nucleolus;GO:0005634|nucleus;	.	.	.
SAMTOR	363.3144	-0.65225	0.160562687	-4.0623	4.859E-05	0.001973	DOWN	yes	gi|556749517|ref|XP_005970182.1|	1849	0	PREDICTED: probable methyltransferase BTM2 homolog [Pantholops hodgsonii]	gi|942031660|ref|XM_005887433.2|	3864	0	PREDICTED: Bos mutus chromosome unknown open reading frame, human C7orf60 (LOC102272702), mRNA	Q1RMZ1	705	0	Probable methyltransferase BMT2 homolog	.	.	.	.	pfam15351	JCAD	Junctional protein associated with coronary artery disease. JCAD is a component of VE-cadherin-based cell-cell junctions in endothelial	.	.	GO:0016433|rRNA (adenine) methyltransferase activity;	.	.
LOC138991260	210.7478	-0.70834	0.174462686	-4.06013	4.905E-05	0.001975	DOWN	yes	gi|965998341|ref|XP_014958889.1|	169	6e-14	PREDICTED: uncharacterized protein LOC106991855 [Ovis aries]	gi|850487286|gb|CP011897.1|	1217	0	Ovis canadensis canadensis isolate 43U chromosome 12 sequence	.	.	.	.	.	.	.	.	pfam12877	DUF3827	Domain of unknown function (DUF3827). This family contains the human KIAA1549 protein which has been found to be fused	.	.	.	.	.
DYNLT3	633.8039	-0.61705	0.151979742	-4.06008	4.906E-05	0.001975	DOWN	yes	gi|655875411|ref|XP_002719877.2|	677	4e-81	PREDICTED: dynein light chain Tctex-type 3 [Oryctolagus cuniculus]	gi|555954624|ref|XM_005889417.1|	2114	0	PREDICTED: Bos mutus dynein, light chain, Tctex-type 3 (DYNLT3), mRNA	Q6XXL8	246	7e-76	Dynein light chain Tctex-type 3	.	.	.	.	pfam03645	Tctex-1	Tctex-1 family. Tctex-1 is a dynein light chain. It has been shown that Tctex-1 can bind to the cytoplasmic tail of rhodopsin. C-	GO:0051301|cell division;GO:0007067|mitotic nuclear division;GO:0007346|regulation of mitotic cell cycle;GO:0006810|transport;	GO:0000777|condensed chromosome kinetochore;GO:0005737|cytoplasm;GO:0005868|cytoplasmic dynein complex;GO:0005874|microtubule;GO:0005	GO:0003774|motor activity;	K10420	DYNLT; dynein light chain Tctex-type 1
CAPN15	407.272	0.684734	0.168672303	4.059551	4.917E-05	0.001975	UP	yes	gi|528925328|ref|XP_005196918.1|	4959	0	PREDICTED: calpain-15 isoform X1 [Bos taurus]	gi|983006678|ref|XM_600341.8|	4819	0	PREDICTED: Bos taurus calpain 15 (CAPN15), transcript variant X5, mRNA	Q9JLG8	1628	0	Calpain-15	.	.	.	.	pfam00648	Peptidase_C2	Calpain family cysteine protease.	GO:0006508|proteolysis;	GO:0005737|cytoplasm;	GO:0004198|calcium-dependent cysteine-type endopeptidase	K08582	CAPN15; calpain-15 [EC:3.4.22.-]
ABR	919.6417	0.528962	0.130423029	4.055744	4.998E-05	0.002001	Not_Sig	no	gi|803276648|ref|XP_011998004.1|	5799	0	PREDICTED: active breakpoint cluster region-related protein isoform X4 [Ovis aries musimon]	gi|965856177|ref|XM_012142614.2|	3929	0	PREDICTED: Ovis aries musimon active BCR-related (ABR), transcript variant X4, mRNA	A6QNS3	1729	0	Active breakpoint cluster region-related protein	.	.	.	.	pfam00620	RhoGAP	RhoGAP domain. GTPase activator proteins towards Rho/Rac/Cdc42-like small GTPases.	GO:0030036|actin cytoskeleton organization;GO:0007420|brain development;GO:0042472|inner ear morphogenesis;GO:0035556|intracellular signal transduction;GO:0060313|negative regulation of blood vessel remodeling;GO:0002692|negative regulation of cellular extravasation;GO:0050728|negative regulation of inflammatory response;GO:0043314|negative regulation of neutrophil degranulation;GO:0050885|neuromuscular process controlling	GO:0005829|cytosol;GO:0016020|membrane;GO:0005886|plasma membrane;	GO:0005096|GTPase activator activity;GO:0005089|Rho guanyl-	.	.
C1R	928.5464	0.482486	0.119021034	4.053787	5.04E-05	0.002008	Not_Sig	no	gi|942090244|ref|XP_014337555.1|	3656	0	PREDICTED: LOW QUALITY PROTEIN: complement C1r subcomponent [Bos mutus]	gi|942090243|ref|XM_014482069.1|	2339	0	PREDICTED: Bos mutus complement component 1, r subcomponent (C1R), mRNA	Q5R544	1203	0	Complement C1r subcomponent	VC1649	94.7	2.41e-19	COG5640	pfam00089	Trypsin	Trypsin.	GO:0006958|complement activation, classical pathway;GO:0045087|innate immune response;	GO:0005576|extracellular region;	GO:0005509|calcium ion binding;GO:0004252|serine-type	K01330	C1R; complement component 1, r subcomponent [EC:3.4.21.41]
AKT3	726.3707	-0.46572	0.114937986	-4.05191	5.08E-05	0.002008	Not_Sig	no	gi|533118702|ref|XP_005374815.1|	2604	0	PREDICTED: RAC-gamma serine/threonine-protein kinase isoform X1 [Chinchilla lanigera]	gi|741953820|ref|XM_010813110.1|	6849	0	PREDICTED: Bos taurus v-akt murine thymoma viral oncogene homolog 3 (AKT3), transcript variant X3, mRNA	Q9Y243	967	0	RAC-gamma serine/threonine-protein kinase	.	.	.	.	pfam00069	Pkinase	Protein kinase domain.	GO:0035556|intracellular signal transduction;GO:0000002|mitochondrial genome maintenance;GO:0018105|peptidyl-serine phosphorylation;GO:0006468|protein phosphorylation;GO:0007165|signal transduction;	GO:0005737|cytoplasm;GO:0005794|Golgi apparatus;GO:0005622|intracellular;GO:0005654|nucleoplasm;GO:0005886|plasma membrane;	GO:0005524|ATP binding;GO:0004672|protein kinase activity;GO:0004674	K04456	AKT; RAC serine/threonine-protein kinase [EC:2.7.11.1]
CYP26B1	485.9001	0.564423	0.139324048	4.051154	5.097E-05	0.002008	Not_Sig	no	gi|300796077|ref|NP_001179722.1|	2655	0	cytochrome P450 26B1 [Bos taurus]	gi|742227875|ref|XM_010829775.1|	4929	0	PREDICTED: Bison bison bison cytochrome P450 26B1 (LOC104980921), mRNA	E1BHJ4	1023	0	Cytochrome P450 26B1	slr0574	266	2.25e-77	COG2124	pfam00067	p450	Cytochrome P450. Cytochrome P450s are haem-thiolate proteins involved in the oxidative degradation of various	GO:0060349|bone morphogenesis;GO:0001709|cell fate determination;GO:0071300|cellular response to retinoic acid;GO:0070268|cornification;GO:0030326|embryonic limb morphogenesis;GO:0061436|establishment of skin barrier;GO:0001768|establishment of T cell polarity;GO:0006954|inflammatory response;GO:0007140|male meiosis;GO:0048387|negative regulation of retinoic acid receptor signaling pathway;GO:0010628|positive regulation of gene expression;GO:2001037|positive regulation of	GO:0005789|endoplasmic reticulum membrane;GO:0031090|organelle membrane;	GO:0020037|heme binding;GO:0005506|iron ion binding;GO:0016709	K12664	CYP26B; cytochrome P450, family 26, subfamily B
RNFT1	932.6016	-0.45838	0.113159062	-4.0508	5.104E-05	0.002008	Not_Sig	no	gi|440908862|gb|ELR58840.1|	2133	0	RING finger and transmembrane domain-containing protein 1, partial [Bos mutus]	gi|982955929|ref|XM_005219927.3|	2135	0	PREDICTED: Bos taurus ring finger protein, transmembrane 1 (RNFT1), transcript variant X1, mRNA	Q5M7Z0	713	0	RING finger and transmembrane domain-containing protein 1	.	.	.	.	pfam13639	zf-RING_2	Ring finger domain.	.	GO:0016021|integral component of membrane;	GO:0008270|zinc ion binding;	.	.
ARID1A	1083.617	0.499343	0.123332957	4.048743	5.149E-05	0.002008	Not_Sig	no	gi|329664977|ref|NP_001192714.1|	7150	0	AT-rich interactive domain-containing protein 1A [Bos taurus]	gi|329664976|ref|NM_001205785.1|	8115	0	Bos taurus AT rich interactive domain 1A (SWI-like) (ARID1A), mRNA	O14497	2703	0	AT-rich interactive domain-containing protein 1A	.	.	.	.	pfam12031	DUF3518	Domain of unknown function (DUF3518). This presumed domain is functionally uncharacterized.	GO:0030521|androgen receptor signaling pathway;GO:0043044|ATP-dependent chromatin remodeling;GO:0003205|cardiac chamber development;GO:0055007|cardiac muscle cell differentiation;GO:0016569|covalent chromatin modification;GO:0006338|chromatin remodeling;GO:0048096|chromatin-mediated maintenance of transcription;GO:0030900|forebrain development;GO:0042921|glucocorticoid receptor signaling pathway;GO:0030520|intracellular estrogen receptor signaling pathway;GO:0006344|maintenance of chromatin	GO:0071565|nBAF complex;GO:0071564|npBAF complex;GO:0000790|nuclear chromatin;GO:0005654|nucleoplasm;GO:00	GO:0003677|DNA binding;GO:0016922|ligand-dependent nuclear receptor	K11653	ARID1; AT-rich interactive domain-containing protein 1
PI15	535.585	-0.61381	0.15162355	-4.04823	5.161E-05	0.002008	DOWN	yes	gi|440906302|gb|ELR56579.1|	1318	4e-163	Peptidase inhibitor 15, partial [Bos mutus]	gi|742150191|ref|XM_010845873.1|	6820	0	PREDICTED: Bison bison bison peptidase inhibitor 15 (PI15), transcript variant X2, mRNA	O43692	504	4e-162	Peptidase inhibitor 15	YJL079c	56.6	2.87e-07	COG2340	pfam00188	CAP	This domain is found in Cysteine-rich secretory protein family. This is a large family of cysteine-rich secretory proteins, antigen 5, and pathogenesis-	.	GO:0070062|extracellular exosome;	GO:0030414|peptidase inhibitor activity;	.	.
RPS14	3559.563	-0.56813	0.140346134	-4.04805	5.165E-05	0.002008	Not_Sig	no	gi|440901667|gb|ELR52566.1|	769	4e-101	40S ribosomal protein S14, partial [Bos mutus]	gi|528957165|ref|XM_005209547.1|	646	0	PREDICTED: Bos taurus ribosomal protein S14 (RPS14), transcript variant X1, mRNA	P62264	285	8e-97	40S ribosomal protein S14	YCR031c	210	1.84e-69	COG0100	pfam00411	Ribosomal_S11	Ribosomal protein S11.	GO:0030218|erythrocyte differentiation;GO:0030490|maturation of SSU-rRNA;GO:0000462|maturation of SSU-rRNA from tricistronic rRNA transcript (SSU-rRNA, 5.8S rRNA, LSU-rRNA);GO:0000122|negative regulation of transcription from RNA polymerase II promoter;GO:0000028|ribosomal small subunit assembly;GO:0006412|translation;	GO:0005737|cytoplasm;GO:0022627|cytosolic small ribosomal subunit;GO:0070062|extracellular exosome;GO:0031012|extracellular	GO:0048027|mRNA 5'-UTR binding;GO:0044822|poly(A) RNA	K02955	RP-S14e, RPS14; small subunit ribosomal protein S14e
SCAF1	1020.409	0.497356	0.122870893	4.047794	5.17E-05	0.002008	Not_Sig	no	gi|358416854|ref|XP_001253211.3|	1514	4e-177	PREDICTED: splicing factor, arginine/serine-rich 19 isoform X2 [Bos taurus]	gi|983000299|ref|XM_005195415.3|	6273	0	PREDICTED: Bos taurus SR-related CTD-associated factor 1 (SCAF1), transcript variant X2, mRNA	Q9H7N4	496	3e-146	Splicing factor, arginine/serine-rich 19	.	.	.	.	pfam12604	gp37_C	Tail fiber protein gp37 C terminal. This domain family is found in bacteria and viruses, and is typically between 49 and 166	GO:0006397|mRNA processing;GO:0008380|RNA splicing;GO:0006366|transcription from RNA polymerase II promoter;	GO:0005634|nucleus;	GO:0003723|RNA binding;	.	.
TRAF7	1267.512	0.427759	0.10568323	4.047558	5.175E-05	0.002008	Not_Sig	no	gi|66792830|ref|NP_001019692.1|	3368	0	E3 ubiquitin-protein ligase TRAF7 [Bos taurus]	gi|982966067|ref|XM_005224426.3|	2638	0	PREDICTED: Bos taurus TNF receptor associated factor 7 (TRAF7), transcript variant X2, mRNA	Q6Q0C0	1274	0	E3 ubiquitin-protein ligase TRAF7	.	.	.	.	pfam00400	WD40	WD domain, G-beta repeat.	GO:0000185|activation of MAPKKK activity;GO:0006915|apoptotic process;GO:2001235|positive regulation of apoptotic signaling pathway;GO:0043410|positive regulation of MAPK cascade;GO:0016567|protein ubiquitination;GO:0006355|regulation of transcription, DNA-templated;GO:0006351|transcription, DNA-templated;	GO:0016023|cytoplasmic membrane-bounded vesicle;GO:0043231|intracellular membrane-bounded organelle;GO:0005886|plasma	GO:0016874|ligase activity;GO:0004842|ubiquitin-protein transferase	K10646	TRAF7, RFWD1; E3 ubiquitin-protein ligase TRAF7 [EC:2.3.2.27]
DHX40	1213.749	-0.48089	0.118844236	-4.0464	5.201E-05	0.002008	Not_Sig	no	gi|116004011|ref|NP_001070365.1|	4094	0	probable ATP-dependent RNA helicase DHX40 [Bos taurus]	gi|742209514|ref|XM_010861613.1|	3520	0	PREDICTED: Bison bison bison DEAH (Asp-Glu-Ala-His) box polypeptide 40 (DHX40), transcript variant X1, mRNA	Q5R864	1561	0	Probable ATP-dependent RNA helicase DHX40	SPAC10F6.02c_2	523	1.43e-170	COG1643	pfam07717	OB_NTP_bind	Oligonucleotide/oligosaccharide-binding (OB)-fold. This family is found towards the C-terminus of the DEAD-box helicases	.	.	GO:0005524|ATP binding;GO:0008026|ATP-dependent helicase	K18711	DHX40, DDX40; ATP-dependent RNA helicase DHX40 [EC:3.6.4.13]
MICOS10	1181.614	-0.58153	0.143724883	-4.04611	5.207E-05	0.002008	Not_Sig	no	gi|803333106|ref|XP_012024663.1|	444	4e-53	PREDICTED: MICOS complex subunit MIC10 isoform X5 [Ovis aries musimon]	gi|982978049|ref|XM_003581904.3|	525	0	PREDICTED: Bos taurus mitochondrial inner membrane organizing system 1 (MINOS1), mRNA	Q5TGZ0	159	8e-49	MICOS complex subunit MIC10	.	.	.	.	pfam04418	DUF543	Domain of unknown function (DUF543). This family of short eukaryotic proteins has no known function. Most of the members of	.	GO:0016021|integral component of membrane;GO:0005743|mitochondrial inner membrane;GO:0005739|mitochondrion;	.	K17784	MINOS1, MOS1; mitochondrial inner membrane organizing system
SCAMP1	534.1485	-0.61892	0.153054441	-4.04381	5.259E-05	0.002018	DOWN	yes	gi|466014187|ref|XP_004270884.1|	1504	0	PREDICTED: secretory carrier-associated membrane protein 1 isoform X2 [Orcinus orca]	gi|942066222|ref|XM_005899715.2|	3731	0	PREDICTED: Bos mutus secretory carrier membrane protein 1 (SCAMP1), transcript variant X3, mRNA	O77735	577	0	Secretory carrier-associated membrane protein 1	.	.	.	.	pfam04144	SCAMP	SCAMP family. In vertebrates, secretory carrier membrane proteins (SCAMPs) 1-3 constitute a family of putative membrane-	GO:0015031|protein transport;	GO:0016021|integral component of membrane;GO:0055038|recycling endosome membrane;GO:0005802|trans-Golgi network;	.	.	.
FBXO30	593.9594	-0.57066	0.141135537	-4.04332	5.27E-05	0.002018	Not_Sig	no	gi|742170239|ref|XP_010849797.1|	3948	0	PREDICTED: F-box only protein 30 [Bison bison bison]	gi|850485237|gb|CP011893.1|	3536	0	Ovis canadensis canadensis isolate 43U chromosome 8 sequence	Q8TB52	1404	0	F-box only protein 30	.	.	.	.	pfam00646	F-box	F-box domain. This domain is approximately 50 amino acids long, and is usually found in the N-terminal half of a variety of	.	.	GO:0061630|ubiquitin protein ligase activity;GO:0008270|zinc ion binding;	K10307	FBXO30; F-box protein 30
TXNL4A	296.532	-0.57929	0.143430476	-4.03885	5.371E-05	0.00205	Not_Sig	no	gi|675718199|ref|XP_002757397.2|	774	2e-100	PREDICTED: thioredoxin-like protein 4A [Callithrix jacchus]	gi|942088101|ref|XM_014481842.1|	783	0	PREDICTED: Bos mutus thioredoxin-like 4A (TXNL4A), transcript variant X1, mRNA	P83877	298	3e-101	Thioredoxin-like protein 4A	.	.	.	.	pfam02966	DIM1	Mitosis protein DIM1.	GO:0051301|cell division;GO:0007067|mitotic nuclear division;GO:0000245|spliceosomal complex assembly;	GO:0005737|cytoplasm;GO:0005634|nucleus;GO:0005681|spliceosomal complex;GO:0046540|U4/U6 x U5 tri-snRNP complex;GO:0005682|U5 snRNP;	.	K12859	TXNL4A, DIB1; U5 snRNP protein, DIM1 family
AHCY	778.2095	0.495884	0.122803479	4.03803	5.39E-05	0.002051	Not_Sig	no	gi|555977084|ref|XP_005900500.1|	2279	0	PREDICTED: adenosylhomocysteinase [Bos mutus]	gi|942068479|ref|XM_005900438.2|	2134	0	PREDICTED: Bos mutus adenosylhomocysteinase (AHCY), mRNA	Q3MHL4	872	0	Adenosylhomocysteinase	SPBC8D2.18c	677	0	COG0499	pfam05221	AdoHcyase	S-adenosyl-L-homocysteine hydrolase.	GO:0006730|one-carbon metabolic process;GO:0033353|S-adenosylmethionine cycle;	GO:0005829|cytosol;GO:0070062|extracellular exosome;GO:0042470|melanosome;	GO:0004013|adenosylhomocysteinase activity;	K01251	E3.3.1.1, ahcY; adenosylhomocysteinase [EC:3.3.1.1]
RPS17	5521.068	-0.63727	0.157890383	-4.03616	5.433E-05	0.00206	DOWN	yes	gi|594100882|ref|XP_006073535.1|	1076	7e-147	PREDICTED: 40S ribosomal protein S17 [Bubalus bubalis]	gi|594100881|ref|XM_006073473.1|	646	0	PREDICTED: Bubalus bubalis ribosomal protein S17-like (RPS17L), mRNA	Q6QAP7	277	1e-93	40S ribosomal protein S17	SPCC24B10.09	173	5.68e-55	COG1383	pfam00833	Ribosomal_S17e	Ribosomal S17.	GO:0000028|ribosomal small subunit assembly;GO:0006412|translation;	GO:0022627|cytosolic small ribosomal subunit;	GO:0003735|structural constituent of ribosome;	K02962	RP-S17e, RPS17; small subunit ribosomal protein S17e
NDUFA5	474.1464	-1.68403	0.417362909	-4.03494	5.462E-05	0.002064	DOWN	yes	gi|594073644|ref|XP_006060638.1|	521	1e-61	PREDICTED: NADH dehydrogenase [ubiquinone] 1 alpha subcomplex subunit 5 isoform X1 [Bubalus bubalis]	gi|850481938|gb|CP011889.1|	470	0	Ovis canadensis canadensis isolate 43U chromosome 4 sequence	P23935	205	5e-63	NADH dehydrogenase [ubiquinone] 1 alpha subcomplex subunit 5	.	.	.	.	pfam04716	ETC_C1_NDUFA5	ETC complex I subunit conserved region. Family of eukaryotic NADH-ubiquinone oxidoreductase subunits (EC:1.6.5.3) (EC:1.6.99.3)	GO:0022904|respiratory electron transport chain;	GO:0005747|mitochondrial respiratory chain complex I;	GO:0008137|NADH dehydrogenase (ubiquinone) activity;	K03949	NDUFA5; NADH dehydrogenase (ubiquinone) 1 alpha subcomplex subunit
ETNK1	1402.729	-0.6398	0.158755522	-4.03009	5.576E-05	0.0021	DOWN	yes	gi|556775834|ref|XP_005983029.1|	2164	0	PREDICTED: LOW QUALITY PROTEIN: ethanolamine kinase 1 [Pantholops hodgsonii]	gi|742186933|ref|XM_010856043.1|	6877	0	PREDICTED: Bison bison bison ethanolamine kinase 1 (ETNK1), mRNA	Q9HBU6	774	0	Ethanolamine kinase 1	YDR147w	84.3	1.65e-15	COG0510	pfam01633	Choline_kinase	Choline/ethanolamine kinase. Choline kinase catalyses the committed step in the synthesis of phosphatidylcholine by the CDP-	GO:0006646|phosphatidylethanolamine biosynthetic process;	GO:0005737|cytoplasm;GO:0005829|cytosol;GO:0016020|membrane;GO:0005654|nucleoplasm;GO:0005886|plasma membrane;	GO:0005524|ATP binding;GO:0004305|ethanolamine kinase activity;	K00894	ETNK, EKI; ethanolamine kinase [EC:2.7.1.82]
TNRC18	1338.33	0.50407	0.125184553	4.026619	5.658E-05	0.002121	Not_Sig	no	gi|528926112|ref|XP_005197069.1|	8925	0	PREDICTED: trinucleotide repeat-containing gene 18 protein isoform X1 [Bos taurus]	gi|983007480|ref|XM_005197012.3|	10732	0	PREDICTED: Bos taurus trinucleotide repeat containing 18 (TNRC18), transcript variant X1, mRNA	O15417	2844	0	Trinucleotide repeat-containing gene 18 protein	.	.	.	.	pfam01426	BAH	BAH domain. This domain has been called BAH (Bromo adjacent homology) domain and has also been called ELM1 and BAM	GO:0006342|chromatin silencing;GO:0031507|heterochromatin assembly;	GO:0000785|chromatin;GO:0005677|chromatin silencing complex;GO:0005737|cytoplasm;GO:0005739|mitochondrion;GO:0031965|nuclear	GO:0003682|chromatin binding;GO:0000976|transcription	.	.
TWSG1	914.0045	-0.46492	0.115476654	-4.02609	5.671E-05	0.002121	Not_Sig	no	gi|440890383|gb|ELR44817.1|	1112	2e-139	Twisted gastrulation protein-like protein 1, partial [Bos mutus]	gi|742198964|ref|XM_010859084.1|	3565	0	PREDICTED: Bison bison bison twisted gastrulation BMP signaling modulator 1 (TWSG1), mRNA	Q9GZX9	419	6e-136	Twisted gastrulation protein homolog 1	.	.	.	.	pfam04668	Tsg	Twisted gastrulation (Tsg) protein conserved region. Tsg was identified in Drosophila as being required to specify the dorsal-most	GO:0030509|BMP signaling pathway;GO:0043010|camera-type eye development;GO:0030154|cell differentiation;GO:0030900|forebrain development;GO:0030097|hemopoiesis;GO:0001707|mesoderm formation;GO:0030514|negative regulation of BMP signaling pathway;GO:0045668|negative regulation of osteoblast differentiation;GO:0001503|ossification;GO:0030513|positive regulation of BMP signaling pathway;GO:0007435|salivary gland morphogenesis;	GO:0070062|extracellular exosome;GO:0005615|extracellular space;	.	.	.
CDR2L	2987.514	0.591838	0.147025363	4.025412	5.688E-05	0.002121	UP	yes	gi|300794441|ref|NP_001179897.1|	1991	0	cerebellar degeneration-related protein 2-like [Bos taurus]	gi|965788227|ref|XM_012109797.2|	2954	0	PREDICTED: Ovis aries musimon cerebellar degeneration-related protein 2-like (CDR2L), transcript variant X2, mRNA	Q86X02	726	0	Cerebellar degeneration-related protein 2-like	.	.	.	.	pfam05483	SCP-1	Synaptonemal complex protein 1 (SCP-1). Synaptonemal complex protein 1 (SCP-1) is the major component of the transverse	.	.	.	.	.
GPC1	21807.56	0.472099	0.117299935	4.02472	5.704E-05	0.002121	Not_Sig	no	gi|741922716|ref|XP_010802238.1|	2482	0	PREDICTED: glypican-1 [Bos taurus]	gi|157279067|gb|BC153219.1|	3183	0	Bos taurus glypican 1, mRNA (cDNA clone MGC:159481 IMAGE:8402544), complete cds	G3X745	959	0	Glypican-1	.	.	.	.	pfam01153	Glypican	Glypican.	GO:0030200|heparan sulfate proteoglycan catabolic process;GO:0040037|negative regulation of fibroblast growth factor receptor signaling pathway;GO:2001016|positive regulation of skeletal muscle cell differentiation;	GO:0031225|anchored component of membrane;GO:0005768|endosome;GO:0070062|extracellular exosome;GO:0005615|extracellular	GO:0005507|copper ion binding;GO:0017134|fibroblast growth	K08107	GPC1; glypican 1
RPTOR	522.1009	0.564401	0.14034007	4.02167	5.779E-05	0.002142	Not_Sig	no	gi|742187831|ref|XP_010854602.1|	6935	0	PREDICTED: regulatory-associated protein of mTOR isoform X1 [Bison bison bison]	gi|742187830|ref|XM_010856300.1|	6312	0	PREDICTED: Bison bison bison regulatory associated protein of MTOR, complex 1 (RPTOR), transcript variant X1, mRNA	Q8K4Q0	2575	0	Regulatory-associated protein of mTOR	.	.	.	.	pfam14538	Raptor_N	Raptor N-terminal CASPase like domain. This domain is found at the N-terminus of the Raptor protein. It has been identified to	GO:0016049|cell growth;GO:0071230|cellular response to amino acid stimulus;GO:0031669|cellular response to nutrient levels;GO:0046676|negative regulation of insulin secretion;GO:0071902|positive regulation of protein serine/threonine kinase activity;GO:0032008|positive regulation of TOR signaling;GO:0045945|positive regulation of transcription from RNA polymerase III promoter;GO:0008361|regulation of cell size;GO:0001932|regulation of protein phosphorylation;GO:0031929|TOR signaling;	GO:0005737|cytoplasm;GO:0043231|intracellular membrane-bounded organelle;GO:0005765|lysosomal membrane;GO:0005764|lysosome;GO:0005	GO:0071889|14-3-3 protein binding;GO:0030674|protein binding,	.	.
C22H11orf24	658.8934	0.612364	0.152527499	4.014779	5.95E-05	0.002198	UP	yes	gi|115495101|ref|NP_001068856.1|	725	1e-85	uncharacterized protein C11orf24 homolog precursor [Bos taurus]	gi|741980055|ref|XM_005227033.2|	1836	0	PREDICTED: Bos taurus chromosome 29 open reading frame, human C11orf24 (C29H11orf24), transcript variant X1, mRNA	Q4V7A5	120	1e-27	Uncharacterized protein C11orf24 homolog	.	.	.	.	pfam05109	Herpes_BLLF1	Herpes virus major outer envelope glycoprotein (BLLF1). This family consists of the BLLF1 viral late glycoprotein, also termed	.	GO:0005794|Golgi apparatus;GO:0016021|integral component of membrane;GO:0005886|plasma membrane;	.	.	.

<table>
<tr><td>YWHAZ</td><td>5051.213</td><td>-0.43563</td><td>0.10855544</td><td>-4.01296</td><td>5.996E-05</td><td>0.002208</td><td>Not_Sig</td><td>no</td><td>gi|83754467|pdb|2C1J|A</td><td>1234</td><td>9e-159</td><td>Chain A, Molecular Basis For The Recognition Of Phosphorylated And Phosphoacetylated Histone H3 By 14-3-3</td><td>gi|742217165|ref|XM_010863514.1|</td><td>2960</td><td>0</td><td>PREDICTED: Bison bison bison tyrosine 3-monooxygenase/tryptophan 5-monooxygenase activation protein, zeta (YWHAZ), mRNA</td><td>Q5R651</td><td>478</td><td>1e-159</td><td>14-3-3 protein zeta/delta</td><td>SPAC8E11.02c</td><td>325</td><td>4.00e-103</td><td>COG5040</td><td>pfam00244</td><td>2014/3/3</td><td>14-3-3 protein.</td><td>GO:0006605|protein targeting;GO:0010941|regulation of cell death;</td><td>GO:0042470|melanosome;GO:0005739|mitochondrion;GO:0005634|nucleus;</td><td>.</td><td>K16197</td><td>YWHAB_Q_Z; 14-3-3 protein beta/theta/zeta</td></tr>
<tr><td>ERGIC1</td><td>2148.849</td><td>0.448524</td><td>0.111960901</td><td>4.006075</td><td>6.174E-05</td><td>0.002266</td><td>Not_Sig</td><td>no</td><td>gi|395817675|ref|XP_003782285.1|</td><td>1562</td><td>0</td><td>PREDICTED: endoplasmic reticulum-Golgi intermediate compartment protein 1 [Otolemur garnettii]</td><td>gi|115497381|ref|NM_001076417.1|</td><td>2672</td><td>0</td><td>Bos taurus endoplasmic reticulum-golgi intermediate compartment 1 (ERGIC1), mRNA</td><td>Q969X5</td><td>569</td><td>0</td><td>Endoplasmic reticulum-Golgi intermediate compartment protein 1</td><td>.</td><td>.</td><td>.</td><td>.</td><td>pfam07970</td><td>COPIIcoated_ERV</td><td>Endoplasmic reticulum vesicle transporter. This family is conserved from plants and fungi to humans. Erv46 works in close</td><td>GO:0006888|ER to Golgi vesicle-mediated transport;</td><td>GO:0005789|endoplasmic reticulum membrane;GO:0005793|endoplasmic reticulum-Golgi intermediate compartment;GO:0033116|endoplasmic</td><td>.</td><td>.</td><td>.</td></tr>
<tr><td>TUBB</td><td>20759.13</td><td>0.467581</td><td>0.116763131</td><td>4.00453</td><td>6.214E-05</td><td>0.002267</td><td>Not_Sig</td><td>no</td><td>gi|847045060|ref|XP_012807536.1|</td><td>2207</td><td>0</td><td>PREDICTED: tubulin beta chain isoform X1 [Jaculus jaculus]</td><td>gi|942088480|ref|XM_014481893.1|</td><td>2188</td><td>0</td><td>PREDICTED: Bos mutus tubulin, beta class I (TUBB), transcript variant X1, mRNA</td><td>P09244</td><td>852</td><td>0</td><td>Tubulin beta-7 chain</td><td>ECU03g0820i</td><td>719</td><td>0</td><td>COG5023</td><td>pfam03953</td><td>Tubulin_C</td><td>Tubulin C-terminal domain. This family includes the tubulin alpha, beta and gamma chains. Members of this family are involved in</td><td>GO:0007017|microtubule-based process;</td><td>GO:0005737|cytoplasm;GO:0005874|microtubule;</td><td>GO:0005525|GTP binding;GO:0003924|GTPase activity;GO:0005200</td><td>K07375</td><td>TUBB; tubulin beta</td></tr>
<tr><td>MGRN1</td><td>1320.796</td><td>0.53896</td><td>0.13458911</td><td>4.004484</td><td>6.215E-05</td><td>0.002267</td><td>Not_Sig</td><td>no</td><td>gi|555951365|ref|XP_005887880.1|</td><td>2665</td><td>0</td><td>PREDICTED: E3 ubiquitin-protein ligase MGRN1 isoform X1 [Bos mutus]</td><td>gi|555951364|ref|XM_005887818.1|</td><td>3119</td><td>0</td><td>PREDICTED: Bos mutus mahogunin ring finger 1, E3 ubiquitin protein ligase (MGRN1), transcript variant X1, mRNA</td><td>O60291</td><td>925</td><td>0</td><td>E3 ubiquitin-protein ligase MGRN1</td><td>.</td><td>.</td><td>.</td><td>.</td><td>pfam13920</td><td>zf-C3HC4_3</td><td>Zinc finger, C3HC4 type (RING finger).</td><td>GO:0008333|endosome to lysosome transport;GO:0043951|negative regulation of cAMP-mediated signaling;GO:0045744|negative regulation of G-protein coupled receptor protein signaling pathway;GO:0006513|protein monoubiquitination;GO:0000209|protein polyubiquitination;</td><td>GO:0005737|cytoplasm;GO:0005829|cytosol;GO:0005769|early endosome;GO:0070062|extracellular exosome;GO:0016020|membrane;GO:0005</td><td>GO:0016874|ligase activity;GO:0061630|ubiquitin protein ligase</td><td>K10604</td><td>MGRN1; E3 ubiquitin-protein ligase MGRN1 [EC:2.3.2.27]</td></tr>
<tr><td>MAP3K3</td><td>852.7922</td><td>0.48399</td><td>0.1209067</td><td>4.003</td><td>6.254E-05</td><td>0.002274</td><td>Not_Sig</td><td>no</td><td>gi|528996713|ref|XP_005221038.1|</td><td>3522</td><td>0</td><td>PREDICTED: mitogen-activated protein kinase kinase kinase 3 isoform X1 [Bos taurus]</td><td>gi|983002341|ref|XM_015468199.1|</td><td>4751</td><td>0</td><td>PREDICTED: Bos taurus mitogen-activated protein kinase kinase kinase 3 (MAP3K3), transcript variant X1, mRNA</td><td>Q99759</td><td>1250</td><td>0</td><td>Mitogen-activated protein kinase kinase kinase 3</td><td>.</td><td>.</td><td>.</td><td>.</td><td>pfam00069</td><td>Pkinase</td><td>Protein kinase domain.</td><td>GO:0001568|blood vessel development;GO:0035556|intracellular signal transduction;GO:0000165|MAPK cascade;GO:0043123|positive regulation of I-kappaB kinase/NF-kappaB signaling;GO:1900745|positive regulation of p38MAPK cascade;GO:0046777|protein autophosphorylation;</td><td>GO:0005737|cytoplasm;GO:0005829|cytosol;</td><td>GO:0005524|ATP binding;GO:0004709|MAP kinase kinase kinase</td><td>K04421</td><td>MAP3K3, MEKK3; mitogen-activated protein kinase kinase kinase 3</td></tr>
<tr><td>FGD1</td><td>563.4049</td><td>0.559699</td><td>0.139913957</td><td>4.000312</td><td>6.326E-05</td><td>0.002275</td><td>Not_Sig</td><td>no</td><td>gi|156523200|ref|NP_001096014.1|</td><td>4275</td><td>0</td><td>FYVE, RhoGEF and PH domain-containing protein 1 [Bos taurus]</td><td>gi|156523199|ref|NM_001102544.1|</td><td>3969</td><td>0</td><td>Bos taurus FYVE, RhoGEF and PH domain containing 1 (FGD1), mRNA</td><td>P98174</td><td>1565</td><td>0</td><td>FYVE, RhoGEF and PH domain-containing protein 1</td><td>.</td><td>.</td><td>.</td><td>.</td><td>pfam00621</td><td>RhoGEF</td><td>RhoGEF domain. Guanine nucleotide exchange factor for Rho/Rac/Cdc42-like GTPases Also called Dbl-homologous (DH)</td><td>GO:0030036|actin cytoskeleton organization;GO:0009887|organ morphogenesis;GO:0007010|cytoskeleton organization;GO:0046847|filopodium assembly;GO:0007275|multicellular organismal development;GO:0043065|positive regulation of apoptotic process;GO:0008360|regulation of cell shape;GO:0043087|regulation of GTPase activity;GO:0035023|regulation of Rho protein signal transduction;GO:0051056|regulation of small GTPase mediated signal transduction;GO:0007165|signal transduction;</td><td>GO:0005737|cytoplasm;GO:0005856|cytoskeleton;GO:0005829|cytosol;GO:0005794|Golgi apparatus;GO:0030027|lamellipodium;GO:0</td><td>GO:0005085|guanyl-nucleotide exchange factor activity;GO:0046872</td><td>K05720</td><td>FGD1; FYVE, RhoGEF and PH domain containing 1</td></tr>
<tr><td>USP20</td><td>400.9521</td><td>0.549964</td><td>0.137542057</td><td>3.998514</td><td>6.374E-05</td><td>0.002275</td><td>Not_Sig</td><td>no</td><td>gi|157427794|ref|NP_001098803.1|</td><td>4574</td><td>0</td><td>ubiquitin carboxyl-terminal hydrolase 20 [Bos taurus]</td><td>gi|157427793|ref|NM_001105333.1|</td><td>4145</td><td>0</td><td>Bos taurus ubiquitin specific peptidase 20 (USP20), mRNA</td><td>A7Z056</td><td>1767</td><td>0</td><td>Ubiquitin carboxyl-terminal hydrolase 20</td><td>.</td><td>.</td><td>.</td><td>.</td><td>pfam00443</td><td>UCH</td><td>Ubiquitin carboxyl-terminal hydrolase.</td><td>GO:0006897|endocytosis;GO:0016579|protein deubiquitination;GO:0071108|protein K48-linked deubiquitination;GO:0070536|protein K63-linked deubiquitination;GO:0008277|regulation of G-protein coupled receptor protein signaling pathway;GO:0006511|ubiquitin-dependent protein catabolic process;</td><td>GO:0005813|centrosome;GO:0048471|perinuclear region of cytoplasm;</td><td>GO:0004197|cysteine-type endopeptidase activity;GO:0004843</td><td>K11848</td><td>USP20_33; ubiquitin carboxyl-terminal hydrolase 20/33 [EC:3.4.19.12]</td></tr>
<tr><td>SLC35A3</td><td>447.6456</td><td>-0.60748</td><td>0.151932331</td><td>-3.99833</td><td>6.379E-05</td><td>0.002275</td><td>DOWN</td><td>yes</td><td>gi|528943282|ref|XP_005204174.1|</td><td>1723</td><td>0</td><td>PREDICTED: UDP-N-acetylglucosamine transporter isoform X1 [Bos taurus]</td><td>gi|982916979|ref|XM_005204117.3|</td><td>4989</td><td>0</td><td>PREDICTED: Bos taurus solute carrier family 35 (UDP-N-acetylglucosamine (UDP-GlcNAc) transporter), member A3 (SLC35A3), transcript variant X1, mRNA</td><td>Q6YC49</td><td>631</td><td>0</td><td>UDP-N-acetylglucosamine transporter</td><td>SPCC1795.03</td><td>240</td><td>1.67e-69</td><td>COG0697</td><td>pfam04142</td><td>Nuc_sug_transp</td><td>Nucleotide-sugar transporter. This family of membrane proteins transport nucleotide sugars from the cytoplasm into golgi vesicles.</td><td>GO:0015788|UDP-N-acetylglucosamine transport;</td><td>GO:0000139|Golgi membrane;GO:0016021|integral component of membrane;</td><td>GO:0005351|sugar:proton symporter activity;</td><td>K15272</td><td>SLC35A1_2_3; solute carrier family 35 (UDP-sugar transporter),</td></tr>
<tr><td>NEGR1</td><td>102.5701</td><td>-0.85284</td><td>0.213301678</td><td>-3.99828</td><td>6.381E-05</td><td>0.002275</td><td>DOWN</td><td>yes</td><td>gi|555975803|ref|XP_005899882.1|</td><td>1673</td><td>0</td><td>PREDICTED: neuronal growth regulator 1 isoform X1 [Bos mutus]</td><td>gi|942066531|ref|XM_005899820.2|</td><td>4926</td><td>0</td><td>PREDICTED: Bos mutus neuronal growth regulator 1 (NEGR1), transcript variant X1, mRNA</td><td>Q7Z3B1</td><td>642</td><td>0</td><td>Neuronal growth regulator 1</td><td>.</td><td>.</td><td>.</td><td>.</td><td>pfam07679</td><td>I-set</td><td>Immunoglobulin I-set domain.</td><td>GO:0007631|feeding behavior;GO:0007626|locomotory behavior;GO:0010976|positive regulation of neuron projection development;GO:0016337|single organismal cell-cell adhesion;</td><td>GO:0031225|anchored component of membrane;GO:0070062|extracellular exosome;GO:0005886|plasma membrane;</td><td>.</td><td>K06775</td><td>NEGR1; neuronal growth regulator 1</td></tr>
<tr><td>THBS2</td><td>29277.85</td><td>0.357342</td><td>0.089411457</td><td>3.9966</td><td>6.426E-05</td><td>0.002275</td><td>Not_Sig</td><td>no</td><td>gi|296483818|tpg|DAA25933.1|</td><td>5728</td><td>0</td><td>TPA: thrombospondin-2 precursor [Bos taurus]</td><td>gi|594075302|ref|XM_006061369.1|</td><td>4742</td><td>0</td><td>PREDICTED: Bubalus bubalis thrombospondin 2 (THBS2), mRNA</td><td>Q95116</td><td>2206</td><td>0</td><td>Thrombospondin-2</td><td>.</td><td>.</td><td>.</td><td>.</td><td>pfam05735</td><td>TSP_C</td><td>Thrombospondin C-terminal region. This region is found at the C-terminus of thrombospondin and related proteins.</td><td>GO:0007155|cell adhesion;GO:0016525|negative regulation of angiogenesis;</td><td>GO:0031012|extracellular matrix;</td><td>GO:0005509|calcium ion binding;GO:0008201|heparin binding;</td><td>K04659</td><td>THBS2S; thrombospondin 2/3/4/5</td></tr>
<tr><td>LOXL1</td><td>2319.621</td><td>0.498015</td><td>0.124611697</td><td>3.996534</td><td>6.428E-05</td><td>0.002275</td><td>Not_Sig</td><td>no</td><td>gi|117306278|gb|AAI26601.1|</td><td>2627</td><td>0</td><td>Lysyl oxidase-like 1 [Bos taurus]</td><td>gi|117306277|gb|BC126600.1|</td><td>2401</td><td>0</td><td>Bos taurus lysyl oxidase-like 1, mRNA (cDNA clone MGC:142693 IMAGE:8092499), complete cds</td><td>Q95L39</td><td>988</td><td>0</td><td>Lysyl oxidase homolog 1</td><td>.</td><td>.</td><td>.</td><td>.</td><td>pfam01186</td><td>Lysyl_oxidase</td><td>Lysyl oxidase.</td><td>.</td><td>GO:0005615|extracellular space;</td><td>GO:0005507|copper ion binding;GO:0016641|oxidoreductase</td><td>K14678</td><td>LOXL1; lysyl oxidase-like protein 1 [EC:1.4.3.-]</td></tr>
<tr><td>ZNF574</td><td>322.7816</td><td>0.674865</td><td>0.168863814</td><td>3.996502</td><td>6.429E-05</td><td>0.002275</td><td>UP</td><td>yes</td><td>gi|440894141|gb|ELR46672.1|</td><td>3387</td><td>0</td><td>Zinc finger protein 574, partial [Bos mutus]</td><td>gi|942095021|ref|XM_005908983.2|</td><td>3123</td><td>0</td><td>PREDICTED: Bos mutus zinc finger protein 574 (ZNF574), transcript variant X1, mRNA</td><td>Q29RK0</td><td>1295</td><td>0</td><td>Zinc finger protein 574</td><td>.</td><td>.</td><td>.</td><td>.</td><td>pfam13465</td><td>zf-H2C2_2</td><td>Zinc-finger double domain.</td><td>GO:0006355|regulation of transcription, DNA-templated;GO:0006351|transcription, DNA-templated;</td><td>GO:0005634|nucleus;</td><td>GO:0003677|DNA binding;GO:0046872|metal ion binding;</td><td>.</td><td>.</td></tr>
<tr><td>MOSPD1</td><td>333.0277</td><td>-0.56262</td><td>0.140783995</td><td>-3.99634</td><td>6.433E-05</td><td>0.002275</td><td>Not_Sig</td><td>no</td><td>gi|84000261|ref|NP_001033226.1|</td><td>864</td><td>3e-106</td><td>motile sperm domain-containing protein 1 [Bos taurus]</td><td>gi|942101922|ref|XR_001352000.1|</td><td>2575</td><td>0</td><td>PREDICTED: Bos mutus motile sperm domain containing 1 (MOSPD1), transcript variant X3, misc_RNA</td><td>Q2T9W7</td><td>337</td><td>1e-107</td><td>Motile sperm domain-containing protein 1</td><td>.</td><td>.</td><td>.</td><td>.</td><td>pfam00635</td><td>Motile_Sperm</td><td>MSP (Major sperm protein) domain. Major sperm proteins are involved in sperm motility. These proteins oligomerise to form</td><td>GO:0000122|negative regulation of transcription from RNA polymerase II promoter;GO:0045944|positive regulation of transcription from RNA polymerase II promoter;</td><td>GO:0016021|integral component of membrane;GO:0005634|nucleus;GO:0048471|perinuclear region of cytoplasm;</td><td>.</td><td>.</td><td>.</td></tr>
<tr><td>SEC61A1</td><td>6703.604</td><td>0.476435</td><td>0.119220261</td><td>3.996261</td><td>6.435E-05</td><td>0.002275</td><td>Not_Sig</td><td>no</td><td>gi|94966817|ref|NP_001035594.1|</td><td>2504</td><td>0</td><td>protein transport protein Sec61 subunit alpha isoform 1 [Bos taurus]</td><td>gi|154426139|gb|BC151365.1|</td><td>3034</td><td>0</td><td>Bos taurus Sec61 alpha 1 subunit (S. cerevisiae), mRNA (cDNA clone MGC:166396 IMAGE:8481163), complete cds</td><td>Q5EA68</td><td>969</td><td>0</td><td>Protein transport protein Sec61 subunit alpha isoform 1</td><td>YLR378c</td><td>545</td><td>0</td><td>COG0201</td><td>pfam00344</td><td>SecY</td><td>SecY translocase.</td><td>GO:0016049|cell growth;GO:0007029|endoplasmic reticulum organization;GO:0006620|posttranslational protein targeting to membrane;GO:0045047|protein targeting to ER;GO:0006614|SRP-dependent cotranslational protein targeting to membrane;</td><td>GO:0030176|integral component of endoplasmic reticulum membrane;</td><td>GO:0043022|ribosome binding;GO:0022857|transmembrane</td><td>K10956</td><td>SEC61A; protein transport protein SEC61 subunit alpha</td></tr>
<tr><td>ARID1B</td><td>969.1005</td><td>0.464531</td><td>0.116278471</td><td>3.99499</td><td>6.47E-05</td><td>0.00228</td><td>Not_Sig</td><td>no</td><td>gi|742123649|ref|XP_010839160.1|</td><td>7459</td><td>0</td><td>PREDICTED: AT-rich interactive domain-containing protein 1B isoform X1 [Bison bison bison]</td><td>gi|742123648|ref|XM_010840858.1|</td><td>6786</td><td>0</td><td>PREDICTED: Bison bison bison AT rich interactive domain 1B (SWI1-like) (ARID1B), transcript variant X1, mRNA</td><td>Q8NFD5</td><td>2117</td><td>0</td><td>AT-rich interactive domain-containing protein 1B</td><td>.</td><td>.</td><td>.</td><td>.</td><td>pfam12031</td><td>DUF3518</td><td>Domain of unknown function (DUF3518). This presumed domain is functionally uncharacterized. This domain is found in</td><td>GO:0016569|covalent chromatin modification;GO:0048096|chromatin-mediated maintenance of transcription;GO:0007399|nervous system development;GO:0006351|transcription, DNA-templated;</td><td>GO:0005737|cytoplasm;GO:0071565|nBAF complex;GO:0005654|nucleoplasm;GO:0016514|SWI/SNF complex;</td><td>GO:0003677|DNA binding;GO:0003713|transcription coactivator activity;</td><td>K11653</td><td>ARID1; AT-rich interactive domain-containing protein 1</td></tr>
<tr><td>WDR62</td><td>344.5448</td><td>0.611454</td><td>0.153102172</td><td>3.993766</td><td>6.503E-05</td><td>0.002285</td><td>UP</td><td>yes</td><td>gi|297485521|ref|XP_002694993.1|</td><td>7322</td><td>0</td><td>PREDICTED: WD repeat-containing protein 62 isoform X1 [Bos taurus]</td><td>gi|594090854|ref|XM_006068762.1|</td><td>5608</td><td>0</td><td>PREDICTED: Bubalus bubalis WD repeat domain 62 (WDR62), mRNA</td><td>Q8HXL3</td><td>2375</td><td>0</td><td>WD repeat-containing protein 62</td><td>.</td><td>.</td><td>.</td><td>.</td><td>pfam00400</td><td>WD40</td><td>WD domain, G-beta repeat.</td><td>GO:0021987|cerebral cortex development;GO:0007052|mitotic spindle organization;GO:0022008|neurogenesis;</td><td>GO:0005814|centriole;GO:0005634|nucleus;GO:0000922|spindle pole;</td><td>.</td><td>.</td><td>.</td></tr>
<tr><td>GCH1</td><td>428.309</td><td>-0.59483</td><td>0.149374741</td><td>-3.9821</td><td>6.831E-05</td><td>0.002388</td><td>DOWN</td><td>yes</td><td>gi|803284660|ref|XP_012001995.1|</td><td>1000</td><td>7e-120</td><td>PREDICTED: GTP cyclohydrolase 1 isoform X2 [Ovis aries musimon]</td><td>gi|850484816|gb|CP011892.1|</td><td>1688</td><td>0</td><td>Ovis canadensis canadensis isolate 43U chromosome 7 sequence</td><td>P30793</td><td>276</td><td>2e-81</td><td>GTP cyclohydrolase 1</td><td>SPAC17A5.13</td><td>161</td><td>1.33e-43</td><td>COG0302</td><td>pfam01227</td><td>GTP_cyclohydroI</td><td>GTP cyclohydrolase I. This family includes GTP cyclohydrolase enzymes and a family of related bacterial proteins.</td><td>GO:0035998|7,8-dihydroneopterin 3'-triphosphate biosynthetic process;GO:0042416|dopamine biosynthetic process;GO:0045776|negative regulation of blood pressure;GO:0050884|neuromuscular process controlling posture;GO:0006809|nitric oxide biosynthetic process;GO:0051000|positive regulation of nitric-oxide synthase activity;GO:0051291|protein heterooligomerization;GO:0051260|protein homooligomerization;GO:0042559|pteridine-containing compound biosynthetic process;GO:0008217|regulation of blood</td><td>GO:0005737|cytoplasm;GO:0031410|cytoplasmic vesicle;GO:0005829|cytosol;GO:0031965|nuclear</td><td>GO:0005509|calcium ion binding;GO:0050662|coenzyme</td><td>K01495</td><td>GCH1, folE; GTP cyclohydrolase I [EC:3.5.4.16]</td></tr>
<tr><td>EZH2</td><td>521.9228</td><td>0.560958</td><td>0.140899775</td><td>3.981258</td><td>6.855E-05</td><td>0.002388</td><td>Not_Sig</td><td>no</td><td>gi|742139739|ref|XP_010842174.1|</td><td>3662</td><td>0</td><td>PREDICTED: histone-lysine N-methyltransferase EZH2 isoform X2 [Bison bison bison]</td><td>gi|742139738|ref|XM_010843872.1|</td><td>2511</td><td>0</td><td>PREDICTED: Bison bison bison enhancer of zeste 2 polycomb repressive complex 2 subunit (EZH2), transcript variant X2, mRNA</td><td>Q15910</td><td>1391</td><td>0</td><td>Histone-lysine N-methyltransferase EZH2</td><td>ECU09g1750</td><td>166</td><td>1.14e-42</td><td>COG2940</td><td>pfam00856</td><td>SET</td><td>SET domain. SET domains are protein lysine methyltransferase enzymes. SET domains appear to be protein-protein interaction</td><td>GO:0014898|cardiac muscle hypertrophy in response to stress;GO:0070301|cellular response to hydrogen peroxide;GO:0035984|cellular response to trichostatin A;GO:0021695|cerebellar cortex development;GO:0006325|chromatin organization;GO:0006306|DNA methylation;GO:0070314|G1 to G0 transition;GO:0036333|hepatocyte homeostasis;GO:0021766|hippocampus development;GO:0070734|histone H3-K27 methylation;GO:0098532|histone H3-K27 trimethylation;GO:0097421|liver</td><td>GO:0005737|cytoplasm;GO:0035098|ESC/E(Z) complex;GO:0000790|nuclear chromatin;GO:0005654|nucleoplasm;GO:0005634|nucleus;GO:0045120|pronucleus;</td><td>GO:0003682|chromatin binding;GO:0031490|chromatin DNA</td><td>K11430</td><td>EZH2; histone-lysine N-methyltransferase EZH2 [EC:2.1.1.43]</td></tr>
<tr><td>CLEC3B</td><td>1537.792</td><td>-0.41056</td><td>0.10314075</td><td>-3.98054</td><td>6.876E-05</td><td>0.002388</td><td>Not_Sig</td><td>no</td><td>gi|555968261|ref|XP_005896167.1|</td><td>998</td><td>6e-134</td><td>PREDICTED: tetranectin [Bos mutus]</td><td>gi|942055442|ref|XM_005896105.2|</td><td>890</td><td>0</td><td>PREDICTED: Bos mutus C-type lectin domain family 3, member B (CLEC3B), mRNA</td><td>Q2KIS7</td><td>388</td><td>5e-135</td><td>Tetranectin</td><td>.</td><td>.</td><td>.</td><td>.</td><td>pfam00059</td><td>Lectin_C</td><td>Lectin C-type domain. This family includes both long and short form C-type</td><td>GO:0030282|bone mineralization;GO:0071560|cellular response to transforming growth factor beta stimulus;GO:0010756|positive regulation of plasminogen activation;</td><td>GO:0005737|cytoplasm;GO:0070062|extracellular exosome;GO:0005615|extracellular space;GO:0001652|granular component;</td><td>GO:0005509|calcium ion binding;GO:0030246|carbohydrate</td><td>K17520</td><td>CLEC3B; C-type lectin domain family 3 member B</td></tr>
<tr><td>CNIH4</td><td>779.5863</td><td>-0.57606</td><td>0.144724983</td><td>-3.98038</td><td>6.88E-05</td><td>0.002388</td><td>Not_Sig</td><td>no</td><td>gi|77735629|ref|NP_001029510.1|</td><td>493</td><td>2e-59</td><td>protein cornichon homolog 4 [Bos taurus]</td><td>gi|741953854|ref|XM_005216808.2|</td><td>697</td><td>0</td><td>PREDICTED: Bos taurus cornichon family AMPA receptor auxiliary protein 4 (CNIH4), transcript variant X1, mRNA</td><td>Q3T126</td><td>194</td><td>5e-61</td><td>Protein cornichon homolog 4</td><td>.</td><td>.</td><td>.</td><td>.</td><td>pfam03311</td><td>Cornichon</td><td>Cornichon protein.</td><td>GO:0006888|ER to Golgi vesicle-mediated transport;GO:0015031|protein transport;</td><td>GO:0005783|endoplasmic reticulum;GO:0005793|endoplasmic reticulum-Golgi intermediate compartment;GO:0016021|integral</td><td>.</td><td>.</td><td>.</td></tr>
<tr><td>RBM14</td><td>971.5757</td><td>0.498265</td><td>0.125218837</td><td>3.979156</td><td>6.916E-05</td><td>0.002394</td><td>Not_Sig</td><td>no</td><td>gi|62460492|ref|NP_001014901.1|</td><td>1676</td><td>0</td><td>RNA-binding protein 14 [Bos taurus]</td><td>gi|555983401|ref|XM_005903539.1|</td><td>2769</td><td>0</td><td>PREDICTED: Bos mutus RNA-binding protein 14 (LOC102283042), transcript variant X1, mRNA</td><td>Q5EA36</td><td>650</td><td>0</td><td>RNA-binding protein 14</td><td>.</td><td>.</td><td>.</td><td>.</td><td>pfam00076</td><td>RRM_1</td><td>RNA recognition motif. (a.k.a. RRM, RBD, or RNP domain). The RRM motif is probably diagnostic of an RNA binding protein. RRMs</td><td>GO:0098534|centriole assembly;GO:0006355|regulation of transcription, DNA-templated;GO:0006351|transcription, DNA-templated;</td><td>GO:0005737|cytoplasm;GO:0005730|nucleolus;GO:0005634|nucleus;</td><td>GO:0000166|nucleotide binding;GO:0003723|RNA binding;</td><td>K13189</td><td>RBM14; RNA-binding protein 14</td></tr>
<tr><td>PTBP3</td><td>1598.359</td><td>-0.51652</td><td>0.12989372</td><td>-3.9765</td><td>6.994E-05</td><td>0.002413</td><td>Not_Sig</td><td>no</td><td>gi|741937016|ref|XP_010806578.1|</td><td>2695</td><td>0</td><td>PREDICTED: polypyrimidine tract-binding protein 3 isoform X3 [Bos taurus]</td><td>gi|742138713|ref|XM_010843581.1|</td><td>6607</td><td>0</td><td>PREDICTED: Bison bison bison polypyrimidine tract binding protein 3 (PTBP3), transcript variant X1, mRNA</td><td>O95758</td><td>999</td><td>0</td><td>Polypyrimidine tract-binding protein 3</td><td>.</td><td>.</td><td>.</td><td>.</td><td>pfam13893</td><td>RRM_5</td><td>RNA recognition motif. (a.k.a. RRM, RBD, or RNP domain). The RRM motif is probably diagnostic of an RNA binding protein. RRMs</td><td>GO:0009653|anatomical structure morphogenesis;GO:0043249|erythrocyte maturation;GO:0006397|mRNA processing;GO:0033119|negative regulation of RNA splicing;GO:0045595|regulation of cell differentiation;GO:0008380|RNA splicing;</td><td>GO:0005634|nucleus;</td><td>GO:0000166|nucleotide binding;GO:0044822|poly(A) RNA</td><td>K17844</td><td>PTBP3, ROD1; polypyrimidine tract-binding protein 3</td></tr>
<tr><td>TMEM94</td><td>433.7536</td><td>0.630097</td><td>0.158484675</td><td>3.975763</td><td>7.015E-05</td><td>0.002413</td><td>UP</td><td>yes</td><td>gi|528921839|ref|XP_005195926.1|</td><td>6454</td><td>0</td><td>PREDICTED: uncharacterized protein KIAA0195 homolog isoform X3 [Bos taurus]</td><td>gi|983002723|ref|XM_005195869.3|</td><td>5038</td><td>0</td><td>PREDICTED: Bos taurus transmembrane protein 94 (TMEM94), transcript variant X3, mRNA</td><td>Q12767</td><td>2295</td><td>0</td><td>Transmembrane protein 94</td><td>.</td><td>.</td><td>.</td><td>.</td><td>pfam03999</td><td>MAP65_ASE1</td><td>Microtubule associated protein (MAP65/ASE1 family).</td><td>.</td><td>GO:0016021|integral component of membrane;GO:0005654|nucleoplasm;</td><td>.</td><td>.</td><td>.</td></tr>
<tr><td>NUDT6</td><td>266.1998</td><td>-0.62525</td><td>0.157356032</td><td>-3.97347</td><td>7.083E-05</td><td>0.00243</td><td>DOWN</td><td>yes</td><td>gi|742203932|ref|XP_010858572.1|</td><td>1386</td><td>0</td><td>PREDICTED: nucleoside diphosphate-linked moiety X motif 6 isoform X2 [Bison bison bison]</td><td>gi|555975618|ref|XM_005899729.1|</td><td>1237</td><td>0</td><td>PREDICTED: Bos mutus nudix (nucleoside diphosphate linked moiety X)-type motif 6 (NUDT6), transcript variant X2, mRNA</td><td>P53370</td><td>457</td><td>6e-159</td><td>Nucleoside diphosphate-linked moiety X motif 6</td><td>BS_yjhB</td><td>59.7</td><td>7.73e-10</td><td>COG1051</td><td>pfam00293</td><td>NUDIX</td><td>NUDIX domain.</td><td>.</td><td>GO:0005739|mitochondrion;GO:0005634|nucleus;</td><td>GO:0008083|growth factor activity;GO:0016787|hydrolase activity;</td><td>.</td><td>.</td></tr>
<tr><td>PLXND1</td><td>361.8964</td><td>0.602603</td><td>0.151697741</td><td>3.972396</td><td>7.115E-05</td><td>0.002433</td><td>UP</td><td>yes</td><td>gi|983004752|ref|XP_015324283.1|</td><td>9470</td><td>0</td><td>PREDICTED: LOW QUALITY PROTEIN: plexin-D1 isoform X2 [Bos taurus]</td><td>gi|983004751|ref|XM_015468797.1|</td><td>5823</td><td>0</td><td>PREDICTED: Bos taurus plexin D1 (PLXND1), mRNA</td><td>Q9Y4D7</td><td>3385</td><td>0</td><td>Plexin-D1</td><td>.</td><td>.</td><td>.</td><td>.</td><td>pfam08337</td><td>Plexin_cytopl</td><td>Plexin cytoplasmic RasGAP domain. This family features the C-terminal regions of various plexins. Plexins are receptors for</td><td>GO:0001525|angiogenesis;GO:0035904|aorta development;GO:0021785|branchiomotor neuron axon guidance;GO:0003279|cardiac septum development;GO:0060976|coronary vasculature development;GO:0060666|dichotomous subdivision of terminal units involved in salivary gland branching;GO:0043542|endothelial cell migration;GO:0003151|outflow tract morphogenesis;GO:0001569|patterning of blood vessels;GO:0032092|positive regulation of protein binding;GO:0045765|regulation of angiogenesis;GO:0048841|regulation of axon extension</td><td>GO:0005887|integral component of plasma membrane;GO:0030027|lamellipodium;GO:0005886|plasma membrane;GO:0002116|semaphorin</td><td>GO:0019904|protein domain specific binding;GO:0017154|semaphorin</td><td>K06822</td><td>PLXND; plexin D</td></tr>
<tr><td>ZNF568</td><td>106.8683</td><td>-0.79875</td><td>0.201370607</td><td>-3.96655</td><td>7.292E-05</td><td>0.002476</td><td>DOWN</td><td>yes</td><td>gi|965809685|ref|XP_011976020.2|</td><td>3052</td><td>0</td><td>PREDICTED: LOW QUALITY PROTEIN: zinc finger protein 568 isoform X3 [Ovis aries musimon]</td><td>gi|742161657|ref|XM_010849122.1|</td><td>4083</td><td>0</td><td>PREDICTED: Bison bison bison zinc finger protein 568 (ZNF568), transcript variant X4, mRNA</td><td>Q3ZCX4</td><td>1052</td><td>0</td><td>Zinc finger protein 568</td><td>.</td><td>.</td><td>.</td><td>.</td><td>pfam01352</td><td>KRAB</td><td>KRAB box. The KRAB domain (or Kruppel-associated box) is present in about a third of zinc finger proteins containing C2H2 fingers.</td><td>GO:0006355|regulation of transcription, DNA-templated;GO:0006351|transcription, DNA-templated;</td><td>GO:0005634|nucleus;</td><td>GO:0003677|DNA binding;GO:0046872|metal ion binding;GO:0003700</td><td>K09228</td><td>KRAB; KRAB domain-containing zinc finger protein</td></tr>
<tr><td>NACAD</td><td>273.6311</td><td>0.799683</td><td>0.20163645</td><td>3.965964</td><td>7.31E-05</td><td>0.002476</td><td>UP</td><td>yes</td><td>gi|982921906|ref|XP_015325536.1|</td><td>9117</td><td>0</td><td>PREDICTED: LOW QUALITY PROTEIN: NAC-alpha domain-containing protein 1 isoform X1, partial [Bos taurus]</td><td>gi|982980796|ref|XM_609695.9|</td><td>5194</td><td>0</td><td>PREDICTED: Bos taurus NAC alpha domain containing (NACAD), transcript variant X2, mRNA</td><td>O15069</td><td>487</td><td>1e-140</td><td>NAC-alpha domain-containing protein 1</td><td>SPBC25H2.05</td><td>108</td><td>1.01e-25</td><td>COG1308</td><td>pfam01849</td><td>NAC</td><td>NAC domain.</td><td>GO:0015031|protein transport;</td><td>GO:0005737|cytoplasm;GO:0005634|nucleus;</td><td>.</td><td>.</td><td>.</td></tr>
<tr><td>OSBP</td><td>1145.79</td><td>0.444563</td><td>0.112107602</td><td>3.965504</td><td>7.324E-05</td><td>0.002476</td><td>Not_Sig</td><td>no</td><td>gi|329664400|ref|NP_001192899.1|</td><td>3561</td><td>0</td><td>oxysterol-binding protein 1 [Bos taurus]</td><td>gi|329664399|ref|NM_001205970.1|</td><td>3308</td><td>0</td><td>Bos taurus oxysterol binding protein (OSBP), mRNA</td><td>P16258</td><td>1337</td><td>0</td><td>Oxysterol-binding protein 1</td><td>.</td><td>.</td><td>.</td><td>.</td><td>pfam01237</td><td>Oxysterol_BP</td><td>Oxysterol-binding protein.</td><td>GO:0015918|sterol transport;</td><td>GO:0005829|cytosol;GO:0005789|endoplasmic reticulum membrane;GO:0000139|Golgi membrane;GO:0048471|perinuclear region of cytoplasm;</td><td>GO:0015485|cholesterol binding;GO:0070273|phosphatidylinosit</td><td>.</td><td>.</td></tr>
<tr><td>SLC10A3</td><td>506.6333</td><td>0.575177</td><td>0.145046733</td><td>3.965458</td><td>7.326E-05</td><td>0.002476</td><td>Not_Sig</td><td>no</td><td>gi|555996883|ref|XP_005910175.1|</td><td>1955</td><td>0</td><td>PREDICTED: P3 protein isoform X1 [Bos mutus]</td><td>gi|594038110|ref|XM_006043615.1|</td><td>2462</td><td>0</td><td>PREDICTED: Bubalus bubalis solute carrier family 10, member 3 (SLC10A3), transcript variant X1, mRNA</td><td>Q0V8N6</td><td>753</td><td>0</td><td>P3 protein</td><td>Cgl2276</td><td>57</td><td>1.07e-07</td><td>COG0385</td><td>pfam01758</td><td>SBF</td><td>Sodium Bile acid symporter family. This family consists of Na+/bile acid co-transporters. These transmembrane proteins function</td><td>GO:0032526|response to retinoic acid;</td><td>GO:0005887|integral component of plasma membrane;</td><td>GO:0008508|bile acid:sodium symporter activity;</td><td>K14343</td><td>SLC10A3_5; solute carrier family 10 (sodium/bile acid cotransporter),</td></tr>
<tr><td>ASPM</td><td>749.169</td><td>0.499266</td><td>0.125988983</td><td>3.962774</td><td>7.408E-05</td><td>0.002489</td><td>Not_Sig</td><td>no</td><td>gi|555970514|ref|XP_005897278.1|</td><td>16408</td><td>0</td><td>PREDICTED: abnormal spindle-like microcephaly-associated protein isoform X2 [Bos mutus]</td><td>gi|741955292|ref|XM_010813572.1|</td><td>11244</td><td>0</td><td>PREDICTED: Bos taurus abnormal spindle microtubule assembly (ASPM), transcript variant X1, mRNA</td><td>P62285</td><td>6118</td><td>0</td><td>Abnormal spindle-like microcephaly-associated protein homolog (Fragment)</td><td>.</td><td>.</td><td>.</td><td>.</td><td>pfam11971</td><td>CAMSAP_CH</td><td>CAMSAP CH domain. This domain is the N-terminal CH domain from the CAMSAP proteins.i</td><td>GO:0051301|cell division;GO:0007067|mitotic nuclear division;GO:0007051|spindle organization;</td><td>GO:0005737|cytoplasm;GO:0005634|nucleus;GO:0005819|spindle;</td><td>.</td><td>.</td><td>.</td></tr>
<tr><td>TMEM30A</td><td>1468.578</td><td>-0.46314</td><td>0.116871861</td><td>-3.96277</td><td>7.409E-05</td><td>0.002489</td><td>Not_Sig</td><td>no</td><td>gi|555981927|ref|XP_005902875.1|</td><td>1903</td><td>0</td><td>PREDICTED: LOW QUALITY PROTEIN: cell cycle control protein 50A [Bos mutus]</td><td>gi|942076010|ref|XM_005902813.2|</td><td>3736</td><td>0</td><td>PREDICTED: Bos mutus transmembrane protein 30A (TMEM30A), mRNA</td><td>Q17QL5</td><td>686</td><td>0</td><td>Cell cycle control protein 50A</td><td>YCR094w</td><td>185</td><td>3.11e-50</td><td>COG5035</td><td>pfam03381</td><td>CDC50</td><td>LEM3 (ligand-effect modulator 3) family / CDC50 family. Members of this family have been predicted to contain transmembrane helices.</td><td>GO:0006855|drug transmembrane transport;GO:0045332|phospholipid translocation;GO:0010976|positive regulation of neuron projection development;GO:0070863|positive regulation of protein exit from endoplasmic reticulum;GO:0036010|protein localization to endosome;</td><td>GO:0016324|apical plasma membrane;GO:0005783|endoplasmic reticulum;GO:0005794|Golgi apparatus;GO:0016021|integral component</td><td>.</td><td>.</td><td>.</td></tr>
<tr><td>MTURN</td><td>789.8323</td><td>-0.44756</td><td>0.112973882</td><td>-3.96166</td><td>7.443E-05</td><td>0.002493</td><td>Not_Sig</td><td>no</td><td>gi|116008440|ref|NP_690006.2|</td><td>665</td><td>2e-76</td><td>maturin [Homo sapiens]</td><td>gi|555979055|ref|XM_005901400.1|</td><td>5738</td><td>0</td><td>PREDICTED: Bos mutus maturin, neural progenitor differentiation regulator homolog (Xenopus) (MTURN), mRNA</td><td>Q8N3F0</td><td>260</td><td>5e-78</td><td>Maturin</td><td>.</td><td>.</td><td>.</td><td>.</td><td>pfam15167</td><td>DUF4581</td><td>Domain of unknown function (DUF4581). This family of proteins is found in eukaryotes. Proteins in this family are typically 131 amino</td><td>GO:0007275|multicellular organismal development;</td><td>.</td><td>.</td><td>.</td><td>.</td></tr>
<tr><td>BRD4</td><td>1511.574</td><td>0.506077</td><td>0.127853447</td><td>3.95826</td><td>7.55E-05</td><td>0.002515</td><td>Not_Sig</td><td>no</td><td>gi|759189738|ref|XP_011382407.1|</td><td>2261</td><td>0</td><td>PREDICTED: bromodomain-containing protein 4 isoform X1 [Pteropus vampyrus]</td><td>gi|982984231|ref|XM_015463983.1|</td><td>5557</td><td>0</td><td>PREDICTED: Bos taurus bromodomain containing 4 (BRD4), mRNA</td><td>O60885</td><td>874</td><td>0</td><td>Bromodomain-containing protein 4</td><td>SPAC631.02</td><td>113</td><td>2.47e-24</td><td>COG5076</td><td>pfam00439</td><td>Bromodomain</td><td>Bromodomain. Bromodomains are 110 amino acid long domains, that are found in many chromatin associated proteins.</td><td>GO:0006974|cellular response to DNA damage stimulus;GO:0016569|covalent chromatin modification;GO:0006338|chromatin remodeling;GO:2000002|negative regulation of DNA damage checkpoint;GO:0010971|positive regulation of G2/M transition of mitotic cell cycle;GO:0043123|positive regulation of I-kappaB kinase/NF-kappaB signaling;GO:0032968|positive regulation of transcription elongation from RNA polymerase II promoter;GO:0045944|positive regulation of transcription from RNA polymerase II promoter;GO:0050727|regulation</td><td>GO:0005694|chromosome;GO:0000794|condensed nuclear chromosome;GO:0005737|cytoplasm;GO:0005654|nucleoplasm;GO:0005634|nucleus;</td><td>GO:0003682|chromatin binding;GO:0070577|lysine-acetylated</td><td>K11722</td><td>BRD4; bromodomain-containing protein 4</td></tr>
<tr><td>HNRNPUL1</td><td>3064.484</td><td>0.451215</td><td>0.114001284</td><td>3.95798</td><td>7.559E-05</td><td>0.002515</td><td>Not_Sig</td><td>no</td><td>gi|134085878|ref|NP_001076935.1|</td><td>2905</td><td>0</td><td>heterogeneous nuclear ribonucleoprotein U-like protein 1 [Bos taurus]</td><td>gi|594055822|ref|XM_006052067.1|</td><td>3654</td><td>0</td><td>PREDICTED: Bubalus bubalis heterogeneous nuclear ribonucleoprotein U-like 1 (HNRNPUL1), transcript variant X1, mRNA</td><td>Q9BUJ2</td><td>1091</td><td>0</td><td>Heterogeneous nuclear ribonucleoprotein U-like protein 1</td><td>.</td><td>.</td><td>.</td><td>.</td><td>pfam13671</td><td>AAA_33</td><td>AAA domain. This family of domains contain only a P-loop motif, that is characteristic of the AAA superfamily. Many of the</td><td>GO:0000398|mRNA splicing, via spliceosome;GO:0006355|regulation of transcription, DNA-templated;GO:0009615|response to virus;GO:0006396|RNA processing;GO:0006351|transcription, DNA-templated;</td><td>GO:0030529|ribonucleoprotein complex;GO:0005654|nucleoplasm;GO:0005634|nucleus;</td><td>GO:0019899|enzyme binding;GO:0044822|poly(A) RNA</td><td>K15047</td><td>HNRNPUL1, E1BAP5; heterogeneous nuclear ribonucleoprotein U-</td></tr>
<tr><td>KATNB1</td><td>452.904</td><td>0.531216</td><td>0.134229986</td><td>3.957503</td><td>7.574E-05</td><td>0.002515</td><td>Not_Sig</td><td>no</td><td>gi|528990350|ref|XP_005218557.1|</td><td>3314</td><td>0</td><td>PREDICTED: katanin p80 WD40 repeat-containing subunit B1 isoform X1 [Bos taurus]</td><td>gi|982951552|ref|XM_005218500.3|</td><td>2505</td><td>0</td><td>PREDICTED: Bos taurus katanin p80 (WD repeat containing) subunit B 1 (KATNB1), transcript variant X1, mRNA</td><td>Q9BVA0</td><td>1050</td><td>0</td><td>Katanin p80 WD40 repeat-containing subunit B1</td><td>.</td><td>.</td><td>.</td><td>.</td><td>pfam13925</td><td>Katanin_con80</td><td>con80 domain of Katanin. The con80 domain of katanin is the C-terminal region of the protein that binds to the N-terminal domain of</td><td>GO:0051301|cell division;GO:0051013|microtubule severing;GO:0007079|mitotic chromosome movement towards spindle pole;GO:0007026|negative regulation of microtubule depolymerization;GO:0010942|positive regulation of cell death;GO:0031117|positive regulation of microtubule depolymerization;GO:0010976|positive regulation of neuron projection development;GO:0006605|protein targeting;</td><td>GO:0030424|axon;GO:0005813|centrosome;GO:0005737|cytoplasm;GO:0030426|growth cone;GO:0008352|katanin complex;GO:0016020|membrane;GO:00058</td><td>GO:0008017|microtubule binding;GO:0046982|protein</td><td>K18643</td><td>KATNB1; katanin p80 WD40 repeat-containing subunit B1</td></tr>
<tr><td>LRP1</td><td>45503.74</td><td>0.445647</td><td>0.112631232</td><td>3.956695</td><td>7.599E-05</td><td>0.002516</td><td>Not_Sig</td><td>no</td><td>gi|982981863|ref|XP_003582266.2|</td><td>23111</td><td>0</td><td>PREDICTED: LOW QUALITY PROTEIN: prolow-density lipoprotein receptor-related protein 1 isoform X2 [Bos taurus]</td><td>gi|942072131|ref|XR_001351650.1|</td><td>14848</td><td>0</td><td>PREDICTED: Bos mutus low density lipoprotein receptor-related protein 1 (LRP1), transcript variant X2, misc_RNA</td><td>Q07954</td><td>8779</td><td>0</td><td>Prolow-density lipoprotein receptor-related protein 1</td><td>.</td><td>.</td><td>.</td><td>.</td><td>pfam00058</td><td>Ldl_recept_b</td><td>Low-density lipoprotein receptor repeat class B. This domain is also known as the YWTD motif after the most conserved region of the</td><td>GO:0007568|aging;GO:0035909|aorta morphogenesis;GO:0043277|apoptotic cell clearance;GO:0097242|beta-amyloid clearance;GO:0008283|cell proliferation;GO:0021987|cerebral cortex development;GO:0042157|lipoprotein metabolic process;GO:0042953|lipoprotein transport;GO:0043524|negative regulation of neuron apoptotic process;GO:0010977|negative regulation of neuron projection development;GO:2000587|negative regulation of platelet-derived growth factor receptor-beta signaling</td><td>GO:0005905|coated pit;GO:0030136|clathrin-coated vesicle;GO:0005737|cytoplasm;GO:0030425|dendrite;GO:0030666|endocytic vesicle</td><td>GO:0034185|apolipoprotein binding;GO:0005509|calcium ion</td><td>K04550</td><td>LRP1, CD91; low-density lipoprotein receptor-related protein 1 (alpha-2-</td></tr>
<tr><td>QPCTL</td><td>326.2804</td><td>0.586138</td><td>0.14822852</td><td>3.954284</td><td>7.676E-05</td><td>0.002527</td><td>UP</td><td>yes</td><td>gi|115495265|ref|NP_001069408.1|</td><td>1822</td><td>0</td><td>glutaminyl-peptide cyclotransferase-like protein [Bos taurus]</td><td>gi|942068159|ref|XM_014479639.1|</td><td>1806</td><td>0</td><td>PREDICTED: Bos mutus glutaminyl-peptide cyclotransferase-like (QPCTL), transcript variant X2, mRNA</td><td>Q0V8G3</td><td>703</td><td>0</td><td>Glutaminyl-peptide cyclotransferase-like protein</td><td>.</td><td>.</td><td>.</td><td>.</td><td>pfam04389</td><td>Peptidase_M28</td><td>Peptidase family M28.</td><td>GO:0017186|peptidyl-pyroglutamic acid biosynthetic process, using glutaminyl-peptide cyclotransferase;</td><td>GO:0000139|Golgi membrane;GO:0016021|integral component of membrane;</td><td>GO:0016603|glutaminyl-peptide cyclotransferase activity;GO:0008270</td><td>K00683</td><td>QPCT; glutaminyl-peptide cyclotransferase [EC:2.3.2.5]</td></tr>
<tr><td>PTBP1</td><td>5259.24</td><td>0.511755</td><td>0.129431209</td><td>3.95388</td><td>7.689E-05</td><td>0.002527</td><td>Not_Sig</td><td>no</td><td>gi|726965387|ref|NP_001289713.1|</td><td>2573</td><td>0</td><td>polypyrimidine tract-binding protein 1 isoform 1 [Bos taurus]</td><td>gi|942097366|ref|XM_005909713.2|</td><td>3071</td><td>0</td><td>PREDICTED: Bos mutus polypyrimidine tract binding protein 1 (PTBP1), transcript variant X1, mRNA</td><td>Q29099</td><td>971</td><td>0</td><td>Polypyrimidine tract-binding protein 1</td><td>.</td><td>.</td><td>.</td><td>.</td><td>pfam13893</td><td>RRM_5</td><td>RNA recognition motif. (a.k.a. RRM, RBD, or RNP domain). The RRM motif is probably diagnostic of an RNA binding protein. RRMs</td><td>GO:0006397|mRNA processing;GO:0048025|negative regulation of mRNA splicing, via spliceosome;GO:0051148|negative regulation of muscle cell differentiation;GO:0000381|regulation of alternative mRNA splicing, via spliceosome;GO:0008380|RNA splicing;</td><td>GO:0005634|nucleus;</td><td>GO:0000166|nucleotide binding;GO:0003723|RNA binding;</td><td>K13218</td><td>PTBP1, PTB; polypyrimidine tract-binding protein 1</td></tr>
<tr><td>KDM6B</td><td>805.1009</td><td>0.641877</td><td>0.162351791</td><td>3.953618</td><td>7.698E-05</td><td>0.002527</td><td>UP</td><td>yes</td><td>gi|528995322|ref|XP_003587460.2|</td><td>5645</td><td>0</td><td>PREDICTED: lysine-specific demethylase 6B isoform X1 [Bos taurus]</td><td>gi|983001689|ref|XM_015468034.1|</td><td>5954</td><td>0</td><td>PREDICTED: Bos taurus lysine (K)-specific demethylase 6B (KDM6B), transcript variant X1, mRNA</td><td>O15054</td><td>2060</td><td>0</td><td>Lysine-specific demethylase 6B</td><td>.</td><td>.</td><td>.</td><td>.</td><td>pfam02373</td><td>JmjC</td><td>JmjC domain, hydroxylase. The JmjC domain belongs to the Cupin superfamily. JmjC-domain proteins may be protein hydroxylases that</td><td>GO:0055007|cardiac muscle cell differentiation;GO:0045165|cell fate commitment;GO:0070301|cellular response to hydrogen peroxide;GO:0045446|endothelial cell differentiation;GO:0021766|hippocampus development;GO:0002437|inflammatory response to antigenic stimulus;GO:0048333|mesodermal cell differentiation;GO:0045944|positive regulation of transcription from RNA polymerase II promoter;GO:0014823|response to activity;GO:0060992|response to fungicide;</td><td>GO:0005654|nucleoplasm;GO:0005634|nucleus;</td><td>GO:0003682|chromatin binding;GO:0051213|dioxygenase</td><td>K11448</td><td>KDM6B, JMJD3; lysine-specific demethylase 6B [EC:1.14.11.-]</td></tr>
</table>

ETV4	185.6544	0.727852	0.184159278	3.952294	7.741E-05	0.002534	UP	yes	gi|741964175|ref|XP_010814689.1|	2595	0	PREDICTED: ETS translocation variant 4 isoform X1 [Bos taurus]	gi|983002193|ref|XM_015468168.1|	2351	0	PREDICTED: Bos taurus ets variant 4 (ETV4), transcript variant X1, mRNA	P43268	908	0	ETS translocation variant 4	.	.	.	.	pfam04621	ETS_PEA3_N	PEA3 subfamily ETS-domain transcription factor N terminal domain. The N terminus of the PEA3 transcription factors is	GO:0060444|branching involved in mammary gland duct morphogenesis;GO:0030154|cell differentiation;GO:0008045|motor neuron axon guidance;GO:0033600|negative regulation of mammary gland epithelial cell proliferation;GO:0045944|positive regulation of transcription from RNA polymerase II promoter;GO:0060762|regulation of branching involved in mammary gland duct morphogenesis;GO:0048863|stem cell differentiation;	GO:0005730|nucleolus;GO:0005654|nucleoplasm;GO:0005634|nucleus;	GO:0000978|RNA polymerase II core promoter proximal region sequence-	K15592	ETV4; ets translocation variant 4
TOM1L2	752.7897	0.566385	0.14341214	3.949353	7.836E-05	0.002558	Not_Sig	no	gi|803245652|ref|XP_011982822.1|	2403	0	PREDICTED: TOM1-like protein 2 isoform X2 [Ovis aries musimon]	gi|594032117|ref|XM_006040782.1|	4109	0	PREDICTED: Bubalus bubalis TOM1-like protein 2-like (LOC102388904), partial mRNA	Q6ZVM7	814	0	TOM1-like protein 2	.	.	.	.	pfam00790	VHS	VHS domain. Domain present in VPS-27, Hrs and STAM.	GO:0006886|intracellular protein transport;GO:0045839|negative regulation of mitotic nuclear division;GO:0007165|signal transduction;	GO:0070062|extracellular exosome;GO:0005622|intracellular;	GO:0030276|clathrin binding;GO:0019901|protein kinase	.	.
ARHGDIA	7736.319	0.368723	0.093468897	3.944876	7.984E-05	0.002592	Not_Sig	no	gi|584037397|ref|XP_006753081.1|	1133	4e-148	PREDICTED: rho GDP-dissociation inhibitor 1 isoform X1 [Myotis davidii]	gi|982958319|ref|XM_005221011.3|	2016	0	PREDICTED: Bos taurus Rho GDP dissociation inhibitor (GDI) alpha (ARHGDIA), transcript variant X1, mRNA	P19803	422	9e-143	Rho GDP-dissociation inhibitor 1	.	.	.	.	pfam02115	Rho_GDI	RHO protein GDP dissociation inhibitor.	GO:0032880|regulation of protein localization;GO:0007266|Rho protein signal transduction;GO:0071526|semaphorin-plexin signaling pathway;	GO:0005737|cytoplasm;GO:0001772|immunological synapse;GO:0005634|nucleus;	GO:0005096|GTPase activator activity;GO:0005094|Rho GDP-	K12462	ARHGDI, RHOGDI; Rho GDP-dissociation inhibitor
RNF157	642.3469	0.499878	0.126727972	3.944498	7.997E-05	0.002592	Not_Sig	no	gi|555992575|ref|XP_005908062.1|	3082	0	PREDICTED: RING finger protein 157 [Bos mutus]	gi|942092164|ref|XM_005908000.2|	4903	0	PREDICTED: Bos mutus ring finger protein 157 (RNF157), mRNA	Q96PX1	1079	0	RING finger protein 157	.	.	.	.	pfam13920	zf-C3HC4_3	Zinc finger, C3HC4 type (RING finger).	.	.	GO:0008270|zinc ion binding;	.	.
INF2	1740.767	0.534978	0.135637033	3.944185	8.007E-05	0.002592	Not_Sig	no	gi|440903059|gb|ELR53767.1|	3041	0	Inverted formin-2 [Bos mutus]	gi|742077186|ref|XM_010832615.1|	2647	0	PREDICTED: Bison bison bison inverted formin-2-like (LOC104983142), mRNA	Q27J81	907	0	Inverted formin-2	.	.	.	.	pfam02181	FH2	Formin Homology 2 Domain.	GO:0030036|actin cytoskeleton organization;GO:0090140|regulation of mitochondrial fission;	GO:0048471|perinuclear region of cytoplasm;	.	.	.
COMT	736.9947	0.623589	0.158224574	3.941166	8.109E-05	0.002617	UP	yes	gi|741957646|ref|XP_010812582.1|	1208	1e-155	PREDICTED: catechol O-methyltransferase [Bos taurus]	gi|982998797|ref|XM_015467250.1|	2468	0	PREDICTED: Bos taurus catechol-O-methyltransferase (COMT), transcript variant X2, mRNA	A7MBI7	466	8e-156	Catechol O-methyltransferase	SPBC119.03	129	1.94e-32	COG4122	pfam01596	Methyltransf_3	O-methyltransferase. Members of this family are O-methyltransferases. The family includes catechol o-	GO:0006584|catecholamine metabolic process;GO:0042135|neurotransmitter catabolic process;	GO:0005737|cytoplasm;GO:0016021|integral component of membrane;GO:0005886|plasma membrane;	GO:0016206|catechol O-methyltransferase activity;GO:0000287	K00545	COMT; catechol O-methyltransferase [EC:2.1.1.6]
LOC106701192	289.6153	-0.64087	0.162651173	-3.94015	8.143E-05	0.002621	DOWN	yes	.	.	.	.	gi|594071884|ref|XR_327664.1|	429	0	PREDICTED: Bubalus bubalis uncharacterized LOC102414455 (LOC102414455), transcript variant X4, misc_RNA	.	.	.	.	.	.	.	.	pfam13668	Ferritin_2	Ferritin-like domain. This family contains ferritins and other ferritin-like proteins such as members of the DPS family and	.	.	.	.	.
LOC102279088	432.8132	-0.51446	0.130645709	-3.93782	8.222E-05	0.002632	Not_Sig	no	gi|440903360|gb|ELR54031.1|	4171	0	RE1-silencing transcription factor [Bos mutus]	gi|742201333|ref|XM_010859642.1|	6363	0	PREDICTED: Bison bison bison RE1-silencing transcription factor-like (LOC105002820), transcript variant X1, mRNA	Q13127	897	0	RE1-silencing transcription factor	.	.	.	.	pfam13465	zf-H2C2_2	Zinc-finger double domain.	GO:0060379|cardiac muscle cell myoblast differentiation;GO:0035690|cellular response to drug;GO:0071257|cellular response to electrical stimulus;GO:0071385|cellular response to glucocorticoid stimulus;GO:0002244|hematopoietic progenitor cell differentiation;GO:0070933|histone H4 deacetylation;GO:0043922|negative regulation by host of viral transcription;GO:0032348|negative regulation of aldosterone biosynthetic process;GO:2000798|negative regulation of amniotic stem cell differentiation;GO:0045955|negative	GO:0005829|cytosol;GO:0005654|nucleoplasm;GO:0005634|nucleus;GO:0017053|transcriptional repressor complex;	GO:0003682|chromatin binding;GO:0001047|core promoter	K09222	REST; RE1-silencing transcription factor
IFRD1	1869.832	-0.40931	0.103957675	-3.93732	8.24E-05	0.002632	Not_Sig	no	gi|555950577|ref|XP_005887490.1|	2258	0	PREDICTED: interferon-related developmental regulator 1 isoform X1 [Bos mutus]	gi|942031645|ref|XM_005887428.2|	2008	0	PREDICTED: Bos mutus interferon-related developmental regulator 1 (IFRD1), transcript variant X1, mRNA	O00458	852	0	Interferon-related developmental regulator 1	.	.	.	.	pfam05004	IFRD	Interferon-related developmental regulator (IFRD). Interferon-related developmental regulator (IFRD1) is the human homologue	GO:0007518|myoblast fate determination;GO:0030517|negative regulation of axon extension;GO:0048671|negative regulation of collateral sprouting;GO:0043403|skeletal muscle tissue regeneration;GO:0014706|striated muscle tissue development;	GO:0005737|cytoplasm;GO:0005634|nucleus;	.	.	.
TBCA	408.8767	-0.69291	0.175994926	-3.93713	8.246E-05	0.002632	DOWN	yes	gi|830006099|ref|XP_004678244.2|	352	1e-38	PREDICTED: tubulin-specific chaperone A [Condylura cristata]	gi|84992990|ref|NM_175803.3|	580	0	Bos taurus tubulin folding cofactor A (TBCA), mRNA	P48427	122	6e-34	Tubulin-specific chaperone A	.	.	.	.	pfam02970	TBCA	Tubulin binding cofactor A.	GO:0007023|post-chaperonin tubulin folding pathway;GO:0007021|tubulin complex assembly;	GO:0005829|cytosol;GO:0070062|extracellular exosome;GO:0005874|microtubule;GO:0005730|nucleolus;	GO:0044822|poly(A) RNA binding;	K17292	TBCA; tubulin-specific chaperone A
ZC2HC1A	91.09033	-0.82264	0.209035469	-3.93542	8.305E-05	0.002643	DOWN	yes	gi|134085724|ref|NP_001076906.1|	1430	0	zinc finger C2HC domain-containing protein 1A [Bos taurus]	gi|742160335|ref|XM_010848770.1|	3418	0	PREDICTED: Bison bison bison zinc finger, C2HC-type containing 1A (ZC2HC1A), mRNA	A4FUE7	555	0	Zinc finger C2HC domain-containing protein 1A	.	.	.	.	pfam13913	zf-C2HC_2	zinc-finger of a C2HC-type. This family contains a number of divergent C2H2 type zinc fingers.	.	.	GO:0046872|metal ion binding;	.	.
DCLK1	2201.608	-0.49385	0.125541286	-3.93378	8.362E-05	0.002651	Not_Sig	no	gi|548488284|ref|XP_005687567.1|	3685	0	PREDICTED: serine/threonine-protein kinase DCLK1 [Capra hircus]	gi|982940658|ref|XM_010810736.2|	8307	0	PREDICTED: Bos taurus doublecortin like kinase 1 (DCLK1), transcript variant X1, mRNA	O15075	1395	0	Serine/threonine-protein kinase DCLK1	.	.	.	.	pfam00069	Pkinase	Protein kinase domain.	GO:0048675|axon extension;GO:0007417|central nervous system development;GO:0021952|central nervous system projection neuron axonogenesis;GO:0048813|dendrite morphogenesis;GO:0016197|endosomal transport;GO:0030900|forebrain development;GO:0035556|intracellular signal transduction;GO:1900181|negative regulation of protein localization to nucleus;GO:0007399|nervous system development;GO:0001764|neuron migration;GO:0018105|peptidyl-serine	GO:0005887|integral component of plasma membrane;GO:0005622|intracellular;	GO:0005524|ATP binding;GO:0004672|protein kinase activity;GO:0004674	K08805	DCLK1_2; doublecortin-like kinase 1/2 [EC:2.7.11.1]
LOC102274386	122.6316	0.794245	0.201925024	3.933365	8.376E-05	0.002651	UP	yes	gi|148698041|gb|EDL29988.1|	687	6e-73	serine/arginine repetitive matrix 1, isoform CRA_d, partial [Mus musculus]	gi|982977919|ref|XM_005197597.3|	2992	0	PREDICTED: Bos taurus serine/arginine repetitive matrix 1 (SRRM1), transcript variant X2, mRNA	Q5ZMJ9	255	6e-70	Serine/arginine repetitive matrix protein 1	.	.	.	.	pfam01480	PWI	PWI domain.	GO:0006397|mRNA processing;GO:0008380|RNA splicing;	GO:0005634|nucleus;	.	K13171	SRRM1, SRM160; serine/arginine repetitive matrix protein 1
RPS26	4053.894	-0.53191	0.13526583	-3.93233	8.413E-05	0.002656	Not_Sig	no	gi|532100091|ref|XP_005335675.1|	434	4e-51	PREDICTED: 40S ribosomal protein S26 [Ictidomys tridecemlineatus]	gi|942085019|ref|XM_005905855.2|	587	0	PREDICTED: Bos mutus ribosomal protein S26 (RPS26), mRNA	P62856	162	3e-49	40S ribosomal protein S26	YER131w	121	4.99e-35	COG4830	pfam01283	Ribosomal_S26e	Ribosomal protein S26e.	GO:0006412|translation;	GO:0022627|cytosolic small ribosomal subunit;	GO:0003729|mRNA binding;GO:0003735|structural constituent of	K02976	RP-S26e, RPS26; small subunit ribosomal protein S26e
VBP1	640.2482	-0.51678	0.131525505	-3.92912	8.526E-05	0.002684	Not_Sig	no	gi|426257352|ref|XP_004022293.1|	1041	1e-134	PREDICTED: prefoldin subunit 3 [Ovis aries]	gi|942065790|ref|XM_005899568.2|	2112	0	PREDICTED: Bos mutus von Hippel-Lindau binding protein 1 (VBP1), transcript variant X1, mRNA	Q2TBX2	402	5e-135	Prefoldin subunit 3	.	.	.	.	pfam02996	Prefoldin	Prefoldin subunit. This family comprises of several prefoldin subunits. The biogenesis of the cytoskeletal proteins actin and	GO:0051086|chaperone mediated protein folding independent of cofactor;GO:0007017|microtubule-based process;GO:0006457|protein folding;GO:0007021|tubulin complex assembly;	GO:0005737|cytoplasm;GO:0005829|cytosol;GO:0005634|nucleus;GO:0005844|polysome;GO:0016272|prefoldin complex;	GO:0015631|tubulin binding;	.	.
ARL5B	301.0031	-0.64422	0.163994801	-3.9283	8.555E-05	0.002686	DOWN	yes	gi|27229225|ref|NP_083742.2|	964	6e-116	ADP-ribosylation factor-like protein 5B [Mus musculus]	gi|742202313|ref|XM_010859893.1|	6975	0	PREDICTED: Bison bison bison ADP-ribosylation factor-like 5B (ARL5B), mRNA	Q9D4P0	373	1e-116	ADP-ribosylation factor-like protein 5B	YDL192w	198	2.48e-57	COG1100	pfam00025	Arf	ADP-ribosylation factor family. Pfam combines a number of different Prosite families together	GO:0007264|small GTPase mediated signal transduction;	GO:0005622|intracellular;	GO:0005525|GTP binding;	K07950	ARL5B; ADP-ribosylation factor-like protein 5B
HIVEP1	638.8203	0.532531	0.135721516	3.923707	8.72E-05	0.00273	Not_Sig	no	gi|742101051|ref|XP_010834179.1|	12216	0	PREDICTED: zinc finger protein 40 isoform X1 [Bison bison bison]	gi|742101050|ref|XM_010835877.1|	8706	0	PREDICTED: Bison bison bison human immunodeficiency virus type I enhancer binding protein 1 (HIVEP1), transcript variant X1, mRNA	P15822	3281	0	Zinc finger protein 40	.	.	.	.	pfam13465	zf-H2C2_2	Zinc-finger double domain.	GO:0007275|multicellular organismal development;GO:0007165|signal transduction;GO:0006366|transcription from RNA polymerase II promoter;	GO:0005739|mitochondrion;GO:0005634|nucleus;	GO:0003677|DNA binding;GO:0046872|metal ion binding;GO:0000980	K09239	HIVEP; human immunodeficiency virus type I enhancer-binding
DNM2	1700.836	0.459062	0.117161778	3.918188	8.922E-05	0.002786	Not_Sig	no	gi|803072851|ref|XP_012033287.1|	3953	0	PREDICTED: dynamin-2 isoform X9 [Ovis aries]	gi|594090049|ref|XM_006068376.1|	3369	0	PREDICTED: Bubalus bubalis dynamin 2 (DNM2), transcript variant X3, mRNA	A6H7I5	1495	0	Dynamin-2	SPBC12C2.08	478	1.37e-152	COG0699	pfam01031	Dynamin_M	Dynamin central region. This region lies between the GTPase domain, see pfam00350, and the pleckstrin homology (PH) domain,	GO:0003374|dynamin polymerization involved in mitochondrial fission;GO:0061025|membrane fusion;GO:0000266|mitochondrial fission;GO:0048812|neuron projection morphogenesis;GO:0006909|phagocytosis;GO:0031623|receptor internalization;GO:0006898|receptor-mediated endocytosis;GO:0030516|regulation of axon extension;	GO:0030054|cell junction;GO:0042995|cell projection;GO:0005905|coated pit;GO:0005874|microtubule;GO:0030496|midbody;GO:0031966|mitochondrial	GO:0005525|GTP binding;GO:0003924|GTPase activity;GO:0008017	K01528	DNM; dynamin GTPase [EC:3.6.5.5]
ZNF358	484.1059	0.655885	0.167448595	3.916934	8.968E-05	0.002786	UP	yes	gi|594044677|ref|XP_006046831.1|	2421	0	PREDICTED: zinc finger protein 358 isoform X1 [Bubalus bubalis]	gi|982984693|ref|XM_015464093.1|	1864	0	PREDICTED: Bos taurus zinc finger protein 358 (ZNF358), transcript variant X1, mRNA	Q9NW07	686	0	Zinc finger protein 358	.	.	.	.	pfam13465	zf-H2C2_2	Zinc-finger double domain.	GO:0035115|embryonic forelimb morphogenesis;GO:0021915|neural tube development;GO:0006355|regulation of transcription, DNA-templated;GO:0019827|stem cell population maintenance;GO:0006351|transcription, DNA-templated;	GO:0005634|nucleus;	GO:0003677|DNA binding;GO:0046872|metal ion binding;	.	.
ECHDC1	1229.056	-0.44025	0.112400075	-3.91683	8.972E-05	0.002786	Not_Sig	no	gi|742210058|ref|XP_010860052.1|	1591	0	PREDICTED: ethylmalonyl-CoA decarboxylase isoform X1 [Bison bison bison]	gi|742210057|ref|XM_010861750.1|	2372	0	PREDICTED: Bison bison bison ethylmalonyl-CoA decarboxylase 1 (ECHDC1), transcript variant X1, mRNA	Q2HJD5	610	0	Ethylmalonyl-CoA decarboxylase	DR1151	115	2.58e-27	COG1024	pfam00378	ECH	Enoyl-CoA hydratase/isomerase family. This family contains a diverse set of enzymes including: Enoyl-CoA hydratase. Napthoate	GO:0008152|metabolic process;	GO:0005829|cytosol;	GO:0016831|carboxy-lyase activity;GO:0004492|methylmalonyl-	K18426	ECHDC1; ethylmalonyl-CoA/methylmalonyl-CoA decarboxylase
RAP1A	474.5709	-0.53832	0.137589208	-3.91251	9.134E-05	0.002829	Not_Sig	no	gi|565309092|gb|ETE62745.1|	899	2e-115	Ras-related protein Rap-1A [Ophiophagus hannah]	gi|742232069|ref|XM_010830821.1|	1427	0	PREDICTED: Bison bison bison RAP1A, member of RAS oncogene family (RAP1A), transcript variant X2, mRNA	P62836	332	2e-110	Ras-related protein Rap-1A	.	.	.	.	pfam00071	Ras	Ras family. Includes sub-families Ras, Rab, Rac, Ral, Ran, Rap Ypt1 and more. Shares P-loop motif with GTP_EFTU, arf and	GO:0000186|activation of MAPKK activity;GO:0071320|cellular response to cAMP;GO:0035690|cellular response to drug;GO:0071333|cellular response to glucose stimulus;GO:1990090|cellular response to nerve growth factor stimulus;GO:0071407|cellular response to organic cyclic compound;GO:0061028|establishment of endothelial barrier;GO:0097421|liver regeneration;GO:0032966|negative regulation of collagen biosynthetic process;GO:2000301|negative regulation of synaptic vesicle exocytosis;GO:0038180|nerve growth factor signaling	GO:0030054|cell junction;GO:0005737|cytoplasm;GO:0005829|cytosol;GO:0005769|early endosome;GO:0070062|extracellular	GO:0005525|GTP binding;GO:0003924|GTPase activity;GO:0032403	K04353	RAP1A; Ras-related protein Rap-1A
HTT	1485.272	0.431181	0.110282408	3.90979	9.238E-05	0.002846	Not_Sig	no	gi|119894330|ref|XP_871851.2|	8504	0	PREDICTED: huntingtin [Bos taurus]	gi|594103283|ref|XM_006074610.1|	6196	0	PREDICTED: Bubalus bubalis huntingtin (HTT), mRNA	P42858	3005	0	Huntingtin	.	.	.	.	pfam12372	DUF3652	Huntingtin protein region. This domain family is found in eukaryotes, and is approximately 40 amino acids in length. The	GO:0048513|organ development;GO:0006915|apoptotic process;GO:0000132|establishment of mitotic spindle orientation;GO:0007030|Golgi organization;GO:2001237|negative regulation of extrinsic apoptotic signaling pathway;GO:0045724|positive regulation of cilium assembly;GO:0031587|positive regulation of inositol 1,4,5-trisphosphate-sensitive calcium-release channel activity;GO:1904504|positive regulation of lipophagy;GO:1903599|positive regulation of mitophagy;GO:0034047|regulation of protein phosphatase type 2A	GO:0005776|autophagosome;GO:0030424|axon;GO:0005814|centriole;GO:0005737|cytoplasm;GO:0030659|cytoplasmic vesicle membrane;GO:0005829|cytosol;GO:003042	GO:0048487|beta-tubulin binding;GO:0034452|dynactin	K04533	HD; huntingtin
HMOX1	827.7647	0.479105	0.122541215	3.909742	9.239E-05	0.002846	Not_Sig	no	gi|440904023|gb|ELR54594.1|	1559	0	Heme oxygenase 1, partial [Bos mutus]	gi|942061067|ref|XM_005898270.2|	1593	0	PREDICTED: Bos mutus heme oxygenase 1 (HMOX1), mRNA	Q5E9F2	587	0	Heme oxygenase 1	sll1184	205	4.00e-62	COG5398	pfam01126	Heme_oxygenase	Heme oxygenase.	GO:0006915|apoptotic process;GO:0006879|cellular iron ion homeostasis;GO:0071243|cellular response to arsenic-containing substance;GO:0071276|cellular response to cadmium ion;GO:0072719|cellular response to cisplatin;GO:0034605|cellular response to heat;GO:0071456|cellular response to hypoxia;GO:0034101|erythrocyte homeostasis;GO:0042167|heme catabolic process;GO:0006788|heme oxidation;GO:0043922|negative regulation by host of viral transcription;GO:1902042|negative regulation of	GO:0005789|endoplasmic reticulum membrane;GO:0005634|nucleus;GO:0048471|perinuclear region of cytoplasm;	GO:0020037|heme binding;GO:0004392|heme oxygenase (decyclizing)	K00510	HMOX, hmuO, ho; heme oxygenase [EC:1.14.99.3]
MED16	818.5558	0.526534	0.134757271	3.907277	9.334E-05	0.002868	Not_Sig	no	gi|555996084|ref|XP_005909781.1|	4315	0	PREDICTED: mediator of RNA polymerase II transcription subunit 16 [Bos mutus]	gi|742142374|ref|XM_010844589.1|	4137	0	PREDICTED: Bison bison bison mediator complex subunit 16 (MED16), transcript variant X2, mRNA	Q9Y2X0	1509	0	Mediator of RNA polymerase II transcription subunit 16	.	.	.	.	pfam11635	Med16	Mediator complex subunit 16. Mediator is a large complex of up to 33 proteins that is conserved from plants through fungi to	GO:0030521|androgen receptor signaling pathway;GO:0030518|intracellular steroid hormone receptor signaling pathway;GO:0045893|positive regulation of transcription, DNA-templated;GO:0006357|regulation of transcription from RNA polymerase II promoter;GO:0006366|transcription from RNA polymerase II promoter;GO:0006367|transcription initiation from RNA polymerase II promoter;	GO:0016592|mediator complex;GO:0016020|membrane;GO:0005654|nucleoplasm;GO:0005634|nucleus;	GO:0003824|catalytic activity;GO:0004872|receptor	K15159	MED16; mediator of RNA polymerase II transcription subunit 16
GSE1	249.3877	0.574646	0.147197085	3.903924	9.465E-05	0.0029	Not_Sig	no	gi|965836027|ref|XP_011988950.2|	4884	0	PREDICTED: genetic suppressor element 1 isoform X4 [Ovis aries musimon]	gi|982999024|ref|XM_015467292.1|	7061	0	PREDICTED: Bos taurus KIAA0182 ortholog (KIAA0182), transcript variant X1, mRNA	Q14687	1084	0	Genetic suppressor element 1	.	.	.	.	pfam12540	DUF3736	Protein of unknown function (DUF3736). This domain family is found in eukaryotes, and is typically between 135 and 160	.	.	.	.	.
PPRC1	917.2772	0.606224	0.155399656	3.901062	9.577E-05	0.00292	UP	yes	gi|742200563|ref|XP_010857751.1|	5703	0	PREDICTED: peroxisome proliferator-activated receptor gamma coactivator-related protein 1 isoform X1 [Bison bison bison]	gi|742200591|ref|XM_010859454.1|	5926	0	PREDICTED: Bison bison bison peroxisome proliferator-activated receptor gamma, coactivator-related 1 (PPRC1), transcript variant X5, mRNA	Q5VV67	1559	0	Peroxisome proliferator-activated receptor gamma coactivator-related protein 1	.	.	.	.	pfam00076	RRM_1	RNA recognition motif. (a.k.a. RRM, RBD, or RNP domain). The RRM motif is probably diagnostic of an RNA binding protein. RRMs	GO:0007005|mitochondrion organization;GO:0051091|positive regulation of sequence-specific DNA binding transcription factor activity;GO:0045944|positive regulation of transcription from RNA polymerase II promoter;GO:0006351|transcription, DNA-templated;	GO:0005654|nucleoplasm;GO:0005634|nucleus;	GO:0030374|ligand-dependent nuclear receptor transcription	K17963	PPRC1, PRC; peroxisome proliferator-activated receptor
LOC102282210	365.724	-0.5193	0.133139181	-3.90044	9.602E-05	0.00292	Not_Sig	no	gi|942080470|ref|XP_005904414.2|	1031	1e-135	PREDICTED: LOW QUALITY PROTEIN: retinol-binding protein 1 [Bos mutus]	gi|942080469|ref|XM_005904352.2|	758	0	PREDICTED: Bos mutus retinol binding protein 1, cellular (RBP1), mRNA	P02694	286	4e-93	Retinol-binding protein 1	YGR010w_2	111	9.96e-27	COG1057	pfam01467	CTP_transf_2	Cytidylyltransferase. This family includes: Cholinephosphate cytidylyltransferase and Glycerol-3-phosphate cytidylyltransferase.	GO:0030852|regulation of granulocyte differentiation;GO:0033189|response to vitamin A;GO:0002138|retinoic acid biosynthetic process;GO:0042572|retinol metabolic process;GO:0006776|vitamin A metabolic process;	GO:0005829|cytosol;	GO:0016918|retinal binding;GO:0019841|retinol binding;GO:0005215	.	.
RPL36A	1281.385	-0.67717	0.173627294	-3.90013	9.614E-05	0.00292	DOWN	yes	gi|555978279|ref|XP_005901084.1|	750	3e-99	PREDICTED: 60S ribosomal protein L36a isoform X2 [Bos mutus]	gi|555978278|ref|XM_005901022.1|	498	0	PREDICTED: Bos mutus ribosomal protein L36a (RPL36A), transcript variant X2, mRNA	P83883	214	3e-70	60S ribosomal protein L36a	YNL162w	138	2.53e-42	COG1631	pfam00935	Ribosomal_L44	Ribosomal protein L44.	GO:0032526|response to retinoic acid;GO:0006412|translation;	GO:0022625|cytosolic large ribosomal subunit;	GO:0003735|structural constituent of ribosome;	K02929	RP-L44e, RPL44; large subunit ribosomal protein L44e
SLC2A4RG	132.4101	0.812263	0.208289316	3.899687	9.632E-05	0.00292	UP	yes	gi|982993090|ref|XP_015321529.1|	1207	6e-158	PREDICTED: SLC2A4 regulator isoform X2 [Bos taurus]	gi|982993089|ref|XM_015466043.1|	1503	0	PREDICTED: Bos taurus SLC2A4 regulator (SLC2A4RG), transcript variant X2, mRNA	Q9NR83	295	2e-92	SLC2A4 regulator	.	.	.	.	pfam09770	PAT1	Topoisomerase II-associated protein PAT1. Members of this family are necessary for accurate chromosome transmission during	GO:0006355|regulation of transcription, DNA-templated;GO:0006351|transcription, DNA-templated;	GO:0005737|cytoplasm;GO:0005654|nucleoplasm;GO:0005634|nucleus;	GO:0003677|DNA binding;GO:0046872|metal ion binding;GO:0003700	.	.
MAPK1	3046.189	0.454675	0.116682734	3.896676	9.752E-05	0.002944	Not_Sig	no	gi|947197833|ref|XP_014442327.1|	1782	0	PREDICTED: mitogen-activated protein kinase 1 [Tupaia chinensis]	gi|594084200|ref|XM_006065600.1|	3506	0	PREDICTED: Bubalus bubalis mitogen-activated protein kinase 1 (MAPK1), mRNA	P46196	690	0	Mitogen-activated protein kinase 1	.	.	.	.	pfam00069	Pkinase	Protein kinase domain.	GO:0006915|apoptotic process;GO:0007049|cell cycle;GO:0018105|peptidyl-serine phosphorylation;GO:0018107|peptidyl-threonine phosphorylation;GO:0031647|regulation of protein stability;GO:0070849|response to epidermal growth factor;	GO:0005737|cytoplasm;GO:0005815|microtubule organizing center;GO:0072686|mitotic spindle;GO:0005634|nucleus;	GO:0005524|ATP binding;GO:0004707|MAP kinase activity;GO:0004674	K04371	MAPK1_3; mitogen-activated protein kinase 1/3 [EC:2.7.11.24]
HDLBP	11293.62	0.365849	0.093899609	3.896176	9.772E-05	0.002944	Not_Sig	no	gi|594108616|ref|XP_006077186.1|	6351	0	PREDICTED: vigilin isoform X1 [Bubalus bubalis]	gi|742088576|ref|XM_010832882.1|	4213	0	PREDICTED: Bison bison bison high density lipoprotein binding protein (HDLBP), transcript variant X1, mRNA	Q8VDJ3	2370	0	Vigilin	.	.	.	.	pfam00013	KH_1	KH domain. KH motifs bind RNA in vitro. Autoantibodies to Nova, a KH domain protein, cause paraneoplastic opsoclonus ataxia.	GO:0008203|cholesterol metabolic process;GO:0006869|lipid transport;	GO:0005913|cell-cell adherens junction;GO:0005737|cytoplasm;GO:0034364|high-density lipoprotein particle;GO:0005634|nucleus;	GO:0098641|cadherin binding involved in cell-cell adhesion;GO:00448	.	.
TENT5B	108.1738	0.787962	0.202271448	3.895567	9.797E-05	0.002944	UP	yes	gi|555967823|ref|XP_005895956.1|	1925	0	PREDICTED: protein FAM46B [Bos mutus]	gi|114051951|ref|NM_001045950.1|	2300	0	Bos taurus family with sequence similarity 46 member B (FAM46B), mRNA	Q29RH2	741	0	Protein FAM46B	.	.	.	.	pfam07984	DUF1693	Domain of unknown function (DUF1693). This family contains many hypothetical proteins. It also includes four nematode prion-like	.	.	.	.	.
SLC38A4	203.1044	-0.61397	0.157668437	-3.89404	9.859E-05	0.002944	DOWN	yes	gi|329664184|ref|NP_001192872.1|	2617	0	sodium-coupled neutral amino acid transporter 4 [Bos taurus]	gi|982925228|ref|XM_010805041.2|	3945	0	PREDICTED: Bos taurus solute carrier family 38 member 4 (SLC38A4), transcript variant X2, mRNA	Q5RE87	957	0	Sodium-coupled neutral amino acid transporter 4	YEL064c	59.3	5.32e-08	COG0814	pfam01490	Aa_trans	Transmembrane amino acid transporter protein. This transmembrane region is found in many amino acid transporters	GO:0006865|amino acid transport;GO:0006814|sodium ion transport;	GO:0016021|integral component of membrane;GO:0005886|plasma membrane;	GO:0015293|symporter activity;	K14991	SLC38A4, SNAT4; solute carrier family 38 (sodium-coupled neutral amino acid
TESK1	442.8219	0.671241	0.172378883	3.893988	9.861E-05	0.002944	UP	yes	gi|440901925|gb|ELR52785.1|	2407	0	Dual specificity testis-specific protein kinase 1, partial [Bos mutus]	gi|288557354|ref|NM_001172227.1|	2388	0	Bos taurus testis-specific kinase 1 (TESK1), mRNA	Q15569	877	0	Dual specificity testis-specific protein kinase 1	.	.	.	.	pfam00069	Pkinase	Protein kinase domain.	GO:0007283|spermatogenesis;	GO:0005829|cytosol;	GO:0005524|ATP binding;GO:0046872|metal ion binding;GO:0004674	K08841	TESK1; testis-specific kinase 1 [EC:2.7.12.1]
TACC3	462.8683	0.476748	0.122435246	3.893881	9.865E-05	0.002944	Not_Sig	no	gi|154152109|ref|NP_001093775.1|	3816	0	transforming acidic coiled-coil-containing protein 3 [Bos taurus]	gi|982928987|ref|XM_005208291.3|	2723	0	PREDICTED: Bos taurus transforming, acidic coiled-coil containing protein 3 (TACC3), transcript variant X1, mRNA	Q9Y6A5	618	0	Transforming acidic coiled-coil-containing protein 3	.	.	.	.	pfam05010	TACC	Transforming acidic coiled-coil-containing protein (TACC). This family contains the proteins TACC 1, 2 and 3 the genes for which are	GO:0008283|cell proliferation;GO:0021987|cerebral cortex development;GO:0007091|metaphase/anaphase transition of mitotic cell cycle;GO:0000226|microtubule cytoskeleton organization;GO:1902850|microtubule cytoskeleton organization involved in mitosis;GO:0060236|regulation of mitotic spindle organization;	GO:0005737|cytoplasm;GO:0015630|microtubule cytoskeleton;	.	K14283	TACC3, maskin; transforming acidic coiled-coil-containing protein 3
MBOAT7	944.1323	0.599517	0.154081385	3.890913	9.987E-05	0.002973	UP	yes	gi|115496722|ref|NP_001068620.1|	2049	0	lysophospholipid acyltransferase 7 [Bos taurus]	gi|742077925|ref|XM_010836970.1|	2266	0	PREDICTED: Bison bison bison membrane bound O-acyltransferase domain containing 7 (MBOAT7), transcript variant X1, mRNA	Q0VCY6	793	0	Lysophospholipid acyltransferase 7	YOR175c	103	6.21e-22	COG5202	pfam03062	MBOAT	MBOAT, membrane-bound O-acyltransferase family. The MBOAT (membrane bound O-acyl transferase) family of membrane	GO:0021819|layer formation in cerebral cortex;GO:0046488|phosphatidylinositol metabolic process;GO:0008654|phospholipid biosynthetic process;GO:0021591|ventricular system development;	GO:0016021|integral component of membrane;	GO:0071617|lysophospholipid acyltransferase activity;	K13516	MBOAT7; lysophospholipid acyltransferase 7 [EC:2.3.1.-]
SLK	496.4264	-0.48749	0.125346287	-3.88918	0.0001006	0.00298	Not_Sig	no	gi|555983748|ref|XP_005903769.1|	5654	0	PREDICTED: STE20-like serine/threonine-protein kinase isoform X1 [Bos mutus]	gi|982968147|ref|XM_005225584.3|	7547	0	PREDICTED: Bos taurus STE20 like kinase (SLK), transcript variant X1, mRNA	Q9H2G2	1867	0	STE20-like serine/threonine-protein kinase	.	.	.	.	pfam00069	Pkinase	Protein kinase domain.	GO:0006915|apoptotic process;GO:0031122|cytoplasmic microtubule organization;GO:0046777|protein autophosphorylation;GO:0042981|regulation of apoptotic process;GO:0030334|regulation of cell migration;GO:0051893|regulation of focal adhesion assembly;	GO:0031252|cell leading edge;GO:0005913|cell-cell adherens junction;GO:0005737|cytoplasm;GO:0070062|extracellular	GO:0005524|ATP binding;GO:0098641|cadherin binding involved in cell-cell	K08836	SLK; STE20-like kinase [EC:2.7.11.1]
ELOB	460.1762	-0.56731	0.14588082	-3.88889	0.0001007	0.00298	Not_Sig	no	gi|77736578|ref|NP_001029972.1|	614	6e-79	transcription elongation factor B polypeptide 2 [Bos taurus]	gi|803280508|ref|XM_012144546.1|	481	0	PREDICTED: Ovis aries musimon transcription elongation factor B (SIII), polypeptide 2 (18kDa, elongin B) (TCEB2), transcript variant X2, mRNA	Q15370	234	7e-78	Transcription elongation factor B polypeptide 2	.	.	.	.	pfam00240	ubiquitin	Ubiquitin family. This family contains a number of ubiquitin-like proteins: SUMO (smt3 homologue) Nedd8, Elongin B, Rub1, and	GO:0006461|protein complex assembly;GO:0016567|protein ubiquitination;GO:0061418|regulation of transcription from RNA polymerase II promoter in response to hypoxia;GO:0006368|transcription elongation from RNA polymerase II promoter;GO:0006366|transcription from RNA polymerase II promoter;	GO:0031462|Cul2-RING ubiquitin ligase complex;GO:0031466|Cul5-RING ubiquitin ligase complex;GO:0005829|cytosol;GO:0070449|	GO:0031625|ubiquitin protein ligase binding;	K03873	TCEB2; transcription elongation factor B, polypeptide 2
ARLN	1204.783	-0.53376	0.137269164	-3.88845	0.0001009	0.00298	Not_Sig	no	gi|555972239|ref|XP_005898126.1|	448	8e-50	PREDICTED: uncharacterized protein C4orf3 homolog [Bos mutus]	gi|942060456|ref|XM_005898064.2|	1684	0	PREDICTED: Bos mutus chromosome unknown open reading frame, human C4orf3 (LOC102285921), mRNA	Q8WVX3	71.6	1e-13	Uncharacterized protein C4orf3	.	.	.	.	.	.	.	.	GO:0016021|integral component of membrane;	.	.	.

ATP13A2	510.819	0.523878	0.13476993	3.8872	0.0001014	0.002988	Not_Sig	no	gi|300795867|ref|NP_001179200.1|	5584	0	probable cation-transporting ATPase 13A2 [Bos taurus]	gi|300795866|ref|NM_001192271.1|	3963	0	Bos taurus ATPase type 13A2 (ATP13A2), mRNA	Q9NQ11	1814	0	Probable cation-transporting ATPase 13A2	.	.	.	.	pfam00122	E1-E2_ATPase	E1-E2 ATPase.	GO:0006874|cellular calcium ion homeostasis;GO:0030003|cellular cation homeostasis;GO:0006879|cellular iron ion homeostasis;GO:0071287|cellular response to manganese ion;GO:0034599|cellular response to oxidative stress;GO:0071294|cellular response to zinc ion;GO:0006882|cellular zinc ion homeostasis;GO:0034220|ion transmembrane transport;GO:1901215|negative regulation of neuron death;GO:1903543|positive regulation of exosomal secretion;GO:0050714|positive regulation of protein	GO:0005776|autophagosome;GO:0016021|integral component of membrane;GO:0005887|integral component of plasma	GO:0005524|ATP binding;GO:0016887|ATPase activity;GO:0019829	K13526	ATP13A2; cation-transporting ATPase 13A2 [EC:3.6.3.-]
RPL27A	5702.241	-0.60851	0.156626439	-3.88513	0.0001023	0.003003	DOWN	yes	gi|594111234|ref|XP_006078444.1|	699	1e-90	PREDICTED: 60S ribosomal protein L27a-like, partial [Bubalus bubalis]	gi|942052719|ref|XM_005895039.2|	507	0	PREDICTED: Bos mutus ribosomal protein L27a (RPL27A), transcript variant X1, mRNA	Q56K03	261	3e-88	60S ribosomal protein L27a	SPBC776.11	159	6.57e-50	COG0200	pfam00828	Ribosomal_L18e	Ribosomal protein L18e/L15. This family includes eukaryotic L18 as well as prokaryotic L15.	GO:0006412|translation;	GO:0022625|cytosolic large ribosomal subunit;	GO:0003735|structural constituent of ribosome;	K02900	RP-L27Ae, RPL27A; large subunit ribosomal protein L27Ae
IKBIP	1849.459	-0.42087	0.108338579	-3.88473	0.0001024	0.003003	Not_Sig	no	gi|528949687|ref|XP_005206722.1|	1981	0	PREDICTED: inhibitor of nuclear factor kappa-B kinase-interacting protein isoform X1 [Bos taurus]	gi|982925901|ref|XM_005206665.3|	3519	0	PREDICTED: Bos taurus IKBKB interacting protein (IKBIP), transcript variant X1, mRNA	Q9DBZ1	584	0	Inhibitor of nuclear factor kappa-B kinase-interacting protein	.	.	.	.	pfam12128	DUF3584	Protein of unknown function (DUF3584). This protein is found in bacteria and eukaryotes. Proteins in this family are typically between	.	GO:0005783|endoplasmic reticulum;GO:0005789|endoplasmic reticulum membrane;GO:0016021|integral component of	.	.	.
SNRNP200	2736.181	0.49806	0.128243353	3.883706	0.0001029	0.003004	Not_Sig	no	gi|555959119|ref|XP_005891670.1|	10914	0	PREDICTED: U5 small nuclear ribonucleoprotein 200 kDa helicase [Bos mutus]	gi|942042919|ref|XM_005891608.2|	6751	0	PREDICTED: Bos mutus small nuclear ribonucleoprotein 200kDa (U5) (SNRNP200), mRNA	O75643	4194	0	U5 small nuclear ribonucleoprotein 200 kDa helicase	SPAC9.03c	1996	0	COG1204	pfam02889	Sec63	Sec63 Brl domain. This domain (also known as the Brl domain) is required for assembly of functional endoplasmic reticulum	GO:0000354|cis assembly of pre-catalytic spliceosome;GO:0000398|mRNA splicing, via spliceosome;GO:0001649|osteoblast differentiation;	GO:0071013|catalytic step 2 spliceosome;GO:0016020|membrane;GO:0005654|nucleoplasm;GO:0005634|nucleus;GO:0005681|spliceosomal	GO:0005524|ATP binding;GO:0008026|ATP-dependent helicase	K12854	SNRNP200, BRR2; pre-mRNA-splicing helicase BRR2 [EC:3.6.4.13]
PGAP6	673.1621	0.643839	0.165791801	3.883419	0.000103	0.003004	UP	yes	gi|529005746|ref|XP_005224464.1|	3703	0	PREDICTED: transmembrane protein 8A isoform X1 [Bos taurus]	gi|529005745|ref|XM_005224407.1|	2470	0	PREDICTED: Bos taurus transmembrane protein 8A (TMEM8A), transcript variant X1, mRNA	Q9HCN3	1043	0	Transmembrane protein 8A	.	.	.	.	pfam12036	DUF3522	Protein of unknown function (DUF3522). This family of proteins is functionally uncharacterized. This protein is found in eukaryotes.	GO:0007155|cell adhesion;	GO:0070062|extracellular exosome;GO:0005887|integral component of plasma membrane;GO:0005765|lysosomal	.	.	.
NEDD8	750.5427	-0.65264	0.168224027	-3.87958	0.0001046	0.003044	DOWN	yes	gi|507556251|ref|XP_004661885.1|	259	2e-25	PREDICTED: NEDD8 [Jaculus jaculus]	gi|942082248|ref|XM_005904921.2|	658	0	PREDICTED: Bos mutus neural precursor cell expressed, developmentally down-regulated 8 (NEDD8), mRNA	Q71UE8	103	7e-27	NEDD8	SPBC12D12.08c	80.9	1.85e-19	COG5272	pfam00240	ubiquitin	Ubiquitin family. This family contains a number of ubiquitin-like proteins: SUMO (smt3 homologue) Nedd8, Elongin B, Rub1, and	GO:0008104|protein localization;GO:0045116|protein neddylation;GO:0006357|regulation of transcription from RNA polymerase II promoter;GO:0014070|response to organic cyclic compound;	GO:0070062|extracellular exosome;GO:0005634|nucleus;	.	K12158	NEDD8; ubiquitin-like protein Nedd8
RABAC1	4667.725	-0.41286	0.106446592	-3.87861	0.0001051	0.003047	Not_Sig	no	gi|594033159|ref|XP_006041319.1|	1154	4e-157	PREDICTED: prenylated Rab acceptor protein 1 [Bubalus bubalis]	gi|942095050|ref|XM_005908987.2|	832	0	PREDICTED: Bos mutus Rab acceptor 1 (prenylated) (RABAC1), transcript variant X1, mRNA	Q1RMH4	377	3e-131	Prenylated Rab acceptor protein 1	SPCC306.02c	61.6	7.51e-11	COG5130	pfam03208	PRA1	PRA1 family protein. This family includes the PRA1 (Prenylated rab acceptor) protein which is a Rab guanine dissociation inhibitor (GDI)	.	GO:0030054|cell junction;GO:0005794|Golgi apparatus;GO:0016021|integral component of membrane;GO:0005886|plasma	.	.	.
RPS12	7475.647	-0.56793	0.146445299	-3.87809	0.0001053	0.003047	Not_Sig	no	gi|823458574|ref|XP_002191710.2|	694	9e-90	PREDICTED: 40S ribosomal protein S12 [Taeniopygia guttata]	gi|982935244|ref|XM_005210930.3|	531	0	PREDICTED: Bos taurus ribosomal protein S12 (RPS12), transcript variant X1, mRNA	P46405	265	8e-90	40S ribosomal protein S12	SPCC962.04	157	6.21e-49	COG1358	pfam01248	Ribosomal_L7Ae	Ribosomal protein L7Ae/L30e/S12e/Gadd45 family. This family includes: Ribosomal L7A from metazoa, Ribosomal L8-A	GO:0006412|translation;	GO:0022627|cytosolic small ribosomal subunit;GO:0016020|membrane;	GO:0044822|poly (A) RNA binding;GO:0003735|structural	K02951	RP-S12e, RPS12; small subunit ribosomal protein S12e
LOC102283474	1433.87	-0.60038	0.15483663	-3.87748	0.0001055	0.003047	DOWN	yes	gi|674078135|ref|XP_008846521.1|	1486	0	PREDICTED: MOB-like protein phocein isoform X3 [Nannospalax galili]	gi|803308007|ref|XM_012157011.1|	2628	0	PREDICTED: Ovis aries musimon 10 kDa heat shock protein, mitochondrial (LOC101122667), transcript variant X4, mRNA	Q9QYW3	446	3e-148	MOB-like protein phocein	.	.	.	.	pfam03637	Mob1_phocein	Mob1/phocein family. Mob1 is an essential Saccharomyces cerevisiae protein, identified from a two-hybrid screen, that binds Mps1p, a	GO:0006900|membrane budding;	GO:0005737|cytoplasm;GO:0005829|cytosol;GO:0043197|dendritic spine;GO:0005794|Golgi apparatus;GO:0032580|Golgi cisterna	GO:0019900|kinase binding;GO:0046872|metal ion binding;	.	.
FSIP1	916.6254	-2.53295	0.653506096	-3.87594	0.0001062	0.003059	DOWN	yes	gi|555953639|ref|XP_005888992.1|	2776	0	PREDICTED: fibrous sheath-interacting protein 1 [Bos mutus]	gi|982936771|ref|XM_010809205.2|	4559	0	PREDICTED: Bos taurus fibrous sheath interacting protein 1 (FSIP1), transcript variant X1, mRNA	Q8NA03	773	0	Fibrous sheath-interacting protein 1	.	.	.	.	pfam15554	FSIP1	FSIP1 family.	.	.	.	.	.
PTAR1	475.3823	-0.58389	0.150864285	-3.87027	0.0001087	0.003123	Not_Sig	no	gi|528958524|ref|XP_005210075.1|	2294	0	PREDICTED: protein prenyltransferase alpha subunit repeat-containing protein 1 isoform X1 [Bos taurus]	gi|982933415|ref|XM_005210018.3|	9177	0	PREDICTED: Bos taurus protein prenyltransferase alpha subunit repeat containing 1 (PTAR1), transcript variant X1, mRNA	Q7Z6K3	761	0	Protein prenyltransferase alpha subunit repeat-containing protein 1	.	.	.	.	pfam01239	PPTA	Protein prenyltransferase alpha subunit repeat. Both farnesyltransferase (FT) and geranylgeranyltransferase 1	GO:0018342|protein prenylation;	GO:0005737|cytoplasm;GO:1990234|transferase complex;	GO:0008318|protein prenyltransferase activity;	K14137	PTAR1; protein prenyltransferase alpha subunit repeat containing protein 1
VARS1	1527.038	0.437462	0.113066936	3.869051	0.0001093	0.003131	Not_Sig	no	gi|296474311|tpg|DAA16426.1|	5153	0	TPA: valyl-tRNA synthetase-like [Bos taurus]	gi|942079958|ref|XM_014480955.1|	3126	0	PREDICTED: Bos mutus valyl-tRNA synthetase (VARS), mRNA	P26640	1888	0	Valine--tRNA ligase	YGR094w	984	0	COG0525	pfam00133	tRNA-synt_1	tRNA synthetases class I (I, L, M and V). Other tRNA synthetase sub-families are too dissimilar to be included.	GO:0006418|tRNA aminoacylation for protein translation;GO:0006438|valyl-tRNA aminoacylation;	GO:0005829|cytosol;GO:0005739|mitochondrion;	GO:0002161|aminoacyl-tRNA editing activity;GO:0005524|ATP	K01873	VARS, valS; valyl-tRNA synthetase [EC:6.1.1.9]
CTDSP1	1166.861	0.546124	0.141194499	3.867885	0.0001098	0.003138	Not_Sig	no	gi|528940219|ref|XP_005202895.1|	1374	0	PREDICTED: carboxy-terminal domain RNA polymerase II polypeptide A small phosphatase 1 isoform X3 [Bos taurus]	gi|742207744|ref|XM_010861187.1|	2703	0	PREDICTED: Bison bison bison CTD (carboxy-terminal domain, RNA polymerase II, polypeptide A) small phosphatase 1 (CTDSP1), transcript variant X2, mRNA	Q9GZU7	529	1e-180	Carboxy-terminal domain RNA polymerase II polypeptide A small phosphatase 1	SPAC2F7.02c	229	2.49e-67	COG5190	pfam03031	NIF	NLI interacting factor-like phosphatase. This family contains a number of NLI interacting factor isoforms and also an N-terminal	GO:2000134|negative regulation of G1/S transition of mitotic cell cycle;GO:0045665|negative regulation of neuron differentiation;GO:0001933|negative regulation of protein phosphorylation;GO:0006470|protein dephosphorylation;GO:0006357|regulation of transcription from RNA polymerase II promoter;	GO:0070062|extracellular exosome;GO:0005654|nucleoplasm;GO:0005634|nucleus;	GO:0008420|CTD phosphatase activity;GO:0046872|metal ion binding;	K15731	CTDSP; carboxy-terminal domain RNA polymerase II polypeptide A small
PITPNM2	605.4467	0.532303	0.137654769	3.866943	0.0001102	0.003142	Not_Sig	no	gi|594045595|ref|XP_006047271.1|	6395	0	PREDICTED: membrane-associated phosphatidylinositol transfer protein 2 isoform X1 [Bubalus bubalis]	gi|982950342|ref|XM_005217856.3|	6782	0	PREDICTED: Bos taurus phosphatidylinositol transfer protein, membrane associated 2 (PITPNM2), transcript variant X11, mRNA	Q9BZ72	2223	0	Membrane-associated phosphatidylinositol transfer protein 2	CAC2938	103	1.68e-22	COG5083	pfam02121	IP_trans	Phosphatidylinositol transfer protein. Along with the structurally unrelated Sec14p family (found in pfam00650), this family can	GO:0008152|metabolic process;GO:0006810|transport;	GO:0012505|endomembrane system;GO:0016021|integral component of membrane;GO:0043231|intracellular membrane-bounded organelle;	GO:0005509|calcium ion binding;GO:0008289|lipid binding;	.	.
PROSER3	114.5439	0.783718	0.202747089	3.865494	0.0001109	0.003153	UP	yes	gi|942092804|ref|XP_014337833.1|	1222	4e-151	PREDICTED: LOW QUALITY PROTEIN: proline and serine-rich protein 3 [Bos mutus]	gi|742162250|ref|XM_010849297.1|	2091	0	PREDICTED: Bison bison bison proline and serine rich 3 (PROSER3), mRNA	Q7TSA6	249	1e-69	Proline and serine-rich protein 3	.	.	.	.	pfam06003	SMN	Survival motor neuron protein (SMN). This family consists of several eukaryotic survival motor neuron (SMN) proteins. The	.	.	.	.	.
LOX	5920.983	-0.38148	0.098724388	-3.86414	0.0001115	0.003163	Not_Sig	no	gi|148229898|ref|NP_776357.2|	1748	0	protein-lysine 6-oxidase precursor [Bos taurus]	gi|742151637|ref|XM_010846285.1|	5056	0	PREDICTED: Bison bison bison lysyl oxidase (LOX), transcript variant X1, mRNA	P33072	672	0	Protein-lysine 6-oxidase	.	.	.	.	pfam01186	Lysyl_oxidase	Lysyl oxidase.	.	GO:0005615|extracellular space;	GO:0005507|copper ion binding;GO:0004720|protein-lysine 6-	.	.
GSKIP	254.6962	-0.62579	0.162091484	-3.86072	0.0001131	0.003199	DOWN	yes	gi|118151190|ref|NP_001071524.1|	730	2e-88	GSK3-beta interaction protein [Bos taurus]	gi|942078291|ref|XM_005903674.2|	2148	0	PREDICTED: Bos mutus GSK3B interacting protein (GSKIP), transcript variant X1, mRNA	Q0P5A3	285	8e-90	GSK3-beta interaction protein	.	.	.	.	pfam05303	DUF727	Protein of unknown function (DUF727). This family consists of several uncharacterized eukaryotic proteins of unknown function.	.	GO:0005737|cytoplasm;	.	.	.
VMA21	855.0585	-0.51266	0.132862538	-3.85858	0.000114	0.00321	Not_Sig	no	gi|440910875|gb|ELR60623.1|	640	3e-73	Vacuolar ATPase assembly integral membrane protein VMA21, partial [Bos mutus]	gi|402744479|ref|NM_001081616.2|	4254	0	Bos taurus VMA21 vacuolar H+-ATPase homolog (S. cerevisiae) (VMA21), mRNA	A2VDK9	123	1e-30	Vacuolar ATPase assembly integral membrane protein VMA21	.	.	.	.	pfam09446	VMA21	VMA21-like domain. This presumed short domain appears to contain two potential transmembrane helices. VMA21 is	GO:0070072|vacuolar proton-transporting V-type ATPase complex assembly;	GO:0030127|COPII vesicle coat;GO:0005789|endoplasmic reticulum membrane;GO:0033116|endoplasmic reticulum-Golgi intermediate compartment	.	.	.
NUP188	1325.067	0.432821	0.112186165	3.858063	0.0001143	0.00321	Not_Sig	no	gi|528969859|ref|XP_005213249.1|	8733	0	PREDICTED: nucleoporin NUP188 homolog isoform X1 [Bos taurus]	gi|942094458|ref|XM_005908793.2|	5653	0	PREDICTED: Bos mutus nucleoporin 188kDa (NUP188), mRNA	Q5SRE5	3251	0	Nucleoporin NUP188 homolog	.	.	.	.	pfam10487	Nup188	Nucleoporin subcomplex protein binding to Pom34. This is one of the many peptides that make up the nucleoporin complex (NPC),	GO:0031047|gene silencing by RNA;GO:0090521|glomerular visceral epithelial cell migration;GO:0075733|intracellular transport of virus;GO:0007077|mitotic nuclear envelope disassembly;GO:0006406|mRNA export from nucleus;GO:0006606|protein import into nucleus;GO:0016925|protein sumoylation;GO:1900034|regulation of cellular response to heat;GO:0010827|regulation of glucose transport;GO:0006405|RNA export from nucleus;GO:0006409|tRNA export from nucleus;GO:0016032|viral process;GO:0019083|viral	GO:0016020|membrane;GO:0005635|nuclear envelope;GO:0044611|nuclear pore inner ring;	GO:0017056|structural constituent of nuclear pore;	K14311	NUP188; nuclear pore complex protein Nup188
GBGT1	425.295	0.630067	0.163317096	3.857935	0.0001143	0.00321	UP	yes	gi|528970297|ref|XP_005213465.1|	1887	0	PREDICTED: globoside alpha-1,3-N-acetylgalactosaminyltransferase 1 isoform X1 [Bos taurus]	gi|982939977|ref|XM_005213408.3|	1892	0	PREDICTED: Bos taurus globoside alpha-1,3-N-acetylgalactosaminyltransferase 1 (GBGT1), transcript variant X1, mRNA	Q95158	551	0	Globoside alpha-1,3-N-acetylgalactosaminyltransferase 1	.	.	.	.	pfam03414	Glyco_transf_6	Glycosyltransferase family 6.	GO:0006486|protein glycosylation;	GO:0000139|Golgi membrane;GO:0016021|integral component of membrane;	GO:0047277|globoside alpha-N-acetylgalactosaminyltransferase	K00722	GBGT1; globoside alpha-N-acetylgalactosaminyltransferase
NDUFA13	391.7573	-0.55206	0.143109998	-3.85756	0.0001145	0.00321	Not_Sig	no	gi|440892285|gb|ELR45541.1|	830	6e-111	NADH dehydrogenase [ubiquinone] 1 alpha subcomplex subunit 13, partial [Bos mutus]	gi|942099137|ref|XM_005910429.2|	546	0	PREDICTED: Bos mutus NADH dehydrogenase (ubiquinone) 1 alpha subcomplex, 13 (NDUFA13), transcript variant X2, mRNA	Q95KV7	296	1e-101	NADH dehydrogenase [ubiquinone] 1 alpha subcomplex subunit 13	.	.	.	.	pfam06212	GRIM-19	GRIM-19 protein. This family consists of several eukaryotic gene associated with retinoic-interferon-induced mortality 19	GO:0097190|apoptotic signaling pathway;GO:0097191|extrinsic apoptotic signaling pathway;GO:0030308|negative regulation of cell growth;GO:0045892|negative regulation of transcription, DNA-templated;	GO:0005737|cytoplasm;GO:0016021|integral component of membrane;GO:0005746|mitochondrial respiratory	GO:0008137|NADH dehydrogenase (ubiquinone) activity;	K11353	NDUFA13; NADH dehydrogenase (ubiquinone) 1 alpha subcomplex subunit
TMSB4X	14292.63	-0.4805	0.12465535	-3.85459	0.0001159	0.003239	Not_Sig	no	gi|965999417|ref|XP_014959023.1|	235	1e-22	PREDICTED: thymosin beta-4 isoform X1 [Ovis aries]	gi|942041684|ref|XM_005891028.2|	554	0	PREDICTED: Bos mutus thymosin beta 4, X-linked (TMSB4X), transcript variant X2, mRNA	P20065	89.4	7e-22	Thymosin beta-4	.	.	.	.	pfam01290	Thymosin	Thymosin beta-4 family.	GO:0030036|actin cytoskeleton organization;GO:0007015|actin filament organization;GO:0001649|osteoblast differentiation;GO:0051152|positive regulation of smooth muscle cell differentiation;GO:0014911|positive regulation of smooth muscle cell migration;GO:0045893|positive regulation of transcription, DNA-templated;GO:0030334|regulation of cell migration;GO:0042989|sequestering of actin monomers;	GO:0005829|cytosol;GO:0031941|filamentous actin;GO:0005634|nucleus;	GO:0003785|actin monomer binding;	K05764	TMSB4; thymosin, beta 4
LDAF1	718.3813	-0.47717	0.123808074	-3.8541	0.0001162	0.003239	Not_Sig	no	gi|742106565|ref|XP_010835475.1|	521	5e-60	PREDICTED: promethin [Bison bison bison]	gi|942059301|ref|XM_005897584.2|	1470	0	PREDICTED: Bos mutus transmembrane protein 159 (TMEM159), mRNA	Q96B96	136	4e-36	Promethin	.	.	.	.	pfam00623	RNA_pol_Rpb1_2	RNA polymerase Rpb1, domain 2. RNA polymerases catalyse the DNA dependent polymerization of RNA. Prokaryotes contain a single RNA	.	GO:0016021|integral component of membrane;	.	.	.
CIZ1	445.4211	0.526971	0.136758881	3.853284	0.0001165	0.003242	Not_Sig	no	gi|296482152|tpg|DAA24267.1|	3027	0	TPA: CDKN1A interacting zinc finger protein 1-like [Bos taurus]	gi|982991306|ref|XM_610020.9|	2724	0	PREDICTED: Bos taurus CDKN1A interacting zinc finger protein 1 (CIZ1), transcript variant X3, mRNA	Q9ULV3	600	0	Cip1-interacting zinc finger protein	.	.	.	.	pfam09770	PAT1	Topoisomerase II-associated protein PAT1. Members of this family are necessary for accurate chromosome transmission during	.	GO:0005634|nucleus;	GO:0003676|nucleic acid binding;GO:0008270|zinc ion binding;	.	.
PRRC2B	1539.361	0.479123	0.124368018	3.852465	0.0001169	0.003243	Not_Sig	no	gi|528970399|ref|XP_005213502.1|	9187	0	PREDICTED: protein PRRC2B isoform X5 [Bos taurus]	gi|942075866|ref|XM_005902767.2|	7141	0	PREDICTED: Bos mutus proline-rich coiled-coil 2B (PRRC2B), mRNA	Q5JSZ5	1482	0	Protein PRRC2B	.	.	.	.	pfam07001	BAT2_N	BAT2 N-terminus. This family represents the N-terminus (approximately 200 residues) of the proline-rich protein BAT2.	GO:0030154|cell differentiation;	.	GO:0044822|poly (A) RNA binding;	.	.
BCL9L	1089.507	0.656999	0.170564865	3.851902	0.0001172	0.003243	UP	yes	gi|594111806|ref|XP_006078712.1|	5638	0	PREDICTED: B-cell CLL/lymphoma 9-like protein isoform X1 [Bubalus bubalis]	gi|982945928|ref|XM_010812505.2|	5786	0	PREDICTED: Bos taurus B-cell CLL/lymphoma 9-like (BCL9L), transcript variant X2, mRNA	Q86UU0	2061	0	B-cell CLL/lymphoma 9-like protein	.	.	.	.	pfam11502	BCL9	B-cell lymphoma 9 protein. The Wnt pathway plays a role in embryonic development, stem cell growth and tumorigenesis. BCL9	GO:0060070|canonical Wnt signaling pathway;GO:0030512|negative regulation of transforming growth factor beta receptor signaling pathway;GO:0010718|positive regulation of epithelial to mesenchymal transition;GO:0045944|positive regulation of transcription from RNA polymerase II promoter;GO:0022604|regulation of cell morphogenesis;GO:0035914|skeletal muscle cell differentiation;GO:0035019|somatic stem cell population maintenance;GO:0006351|transcription, DNA-templated;	GO:0005730|nucleolus;GO:0005654|nucleoplasm;GO:0005634|nucleus;	GO:0008013|beta-catenin binding;GO:0003713|transcription	.	.
RPL6	6627.895	-0.39025	0.101325299	-3.85149	0.0001174	0.003243	Not_Sig	no	gi|942094960|ref|XP_014338143.1|	1096	5e-147	PREDICTED: 60S ribosomal protein L6 isoform X1 [Bos mutus]	gi|741956441|ref|XM_005217785.2|	957	0	PREDICTED: Bos taurus ribosomal protein L6 (RPL6), transcript variant X1, mRNA	Q58DQ3	426	2e-148	60S ribosomal protein L6	SPCC622.18	128	2.34e-35	COG2163	pfam01159	Ribosomal_L6e	Ribosomal protein L6e.	GO:0002181|cytoplasmic translation;GO:0000027|ribosomal large subunit assembly;	GO:0022625|cytosolic large ribosomal subunit;	GO:0003735|structural constituent of ribosome;	K02934	RP-L6e, RPL6; large subunit ribosomal protein L6e
SLC25A36	2590.166	-0.45328	0.117788189	-3.8483	0.0001189	0.003272	Not_Sig	no	gi|431916930|gb|ELK16686.1|	1693	0	Solute carrier family 25 member 36 [Pteropus alecto]	gi|742195352|ref|XM_010858201.1|	4797	0	PREDICTED: Bison bison bison solute carrier family 25 (pyrimidine nucleotide carrier), member 36 (SLC25A36), mRNA	Q96CQ1	627	0	Solute carrier family 25 member 36	.	.	.	.	pfam00153	Mito_carr	Mitochondrial carrier protein.	GO:0000002|mitochondrial genome maintenance;GO:0007005|mitochondrion organization;GO:0006864|pyrimidine nucleotide transport;GO:0051881|regulation of mitochondrial membrane potential;GO:0006412|translation;	GO:0016021|integral component of membrane;GO:0005743|mitochondrial inner membrane;GO:0005739|mitochondrion;	GO:0015218|pyrimidine nucleotide transmembrane transporter	K15116	SLC25A33_36, RIM2; solute carrier family 25, member 33/36
PID1	4478.896	-0.33292	0.086531893	-3.84742	0.0001194	0.003272	Not_Sig	no	gi|51555802|dbj|BAD38656.1|	1187	1e-153	putative protein product of HMFN2073 [Homo sapiens]	gi|982914874|ref|XM_005202921.3|	2488	0	PREDICTED: Bos taurus phosphotyrosine interaction domain containing 1 (PID1), transcript variant X1, mRNA	Q3UBG2	418	5e-139	PTB-containing, cubilin and LRP1-interacting protein	.	.	.	.	pfam14719	PID_2	Phosphotyrosine interaction domain (PTB/PID).	GO:0071345|cellular response to cytokine stimulus;GO:0071398|cellular response to fatty acid;GO:0071354|cellular response to interleukin-6;GO:0044320|cellular response to leptin stimulus;GO:0071356|cellular response to tumor necrosis factor;GO:0070584|mitochondrion morphogenesis;GO:2001170|negative regulation of ATP biosynthetic process;GO:0090005|negative regulation of establishment of protein localization to plasma membrane;GO:0046325|negative regulation of glucose import;GO:2001274|negative regulation of glucose import in	GO:0005737|cytoplasm;GO:0005654|nucleoplasm;	.	.	.
CLPTM1	2494.427	0.534484	0.138928413	3.847188	0.0001195	0.003272	Not_Sig	no	gi|803247171|ref|XP_011983582.1|	2851	0	PREDICTED: cleft lip and palate transmembrane protein 1 isoform X2 [Ovis aries musimon]	gi|742173933|ref|XM_010852588.1|	2451	0	PREDICTED: Bison bison bison cleft lip and palate associated transmembrane protein 1 (CLPTM1), mRNA	Q2NL17	1089	0	Cleft lip and palate transmembrane protein 1 homolog	.	.	.	.	pfam05602	CLPTM1	Cleft lip and palate transmembrane protein 1 (CLPTM1). This family consists of several eukaryotic cleft lip and	GO:0030154|cell differentiation;GO:0007275|multicellular organismal development;	GO:0016021|integral component of membrane;	.	.	.
TCEAL9	234.3665	-0.60923	0.158366034	-3.84696	0.0001196	0.003272	DOWN	yes	gi|4239700|emb|CAA10770.1|	896	7e-134	reverse transcriptase-like [Bos taurus]	gi|982973350|ref|XM_010821704.2|	2167	0	PREDICTED: Bos taurus WW domain binding protein 5 (WBP5), transcript variant X1, mRNA	P11369	60.5	1e-13	LINE-1 retrotransposable element ORF2 protein	.	.	.	.	pfam00078	RVT_1	Reverse transcriptase (RNA-dependent DNA polymerase). A reverse transcriptase gene is usually indicative of a mobile	GO:0006310|DNA recombination;	.	GO:0004519|endonuclease activity;GO:0046872|metal ion	.	.
AEBP1	4201.353	0.49013	0.1274401	3.845964	0.0001201	0.003277	Not_Sig	no	gi|982921913|ref|XP_015325570.1|	3774	0	PREDICTED: adipocyte enhancer-binding protein 1 isoform X1 [Bos taurus]	gi|982921912|ref|XM_015470084.1|	3783	0	PREDICTED: Bos taurus AE binding protein 1 (AEBP1), transcript variant X1, mRNA	Q8IUX7	1338	0	Adipocyte enhancer-binding protein 1	.	.	.	.	pfam00246	Peptidase_M14	Zinc carboxypeptidase.	GO:0007517|muscle organ development;GO:0000122|negative regulation of transcription from RNA polymerase II promoter;GO:0006518|peptide metabolic process;GO:0016485|protein processing;GO:0001501|skeletal system development;GO:0006351|transcription, DNA-templated;	GO:0005737|cytoplasm;GO:0070062|extracellular exosome;GO:0005615|extracellular space;GO:0005634|nucleus;	GO:0004180|carboxypeptidase activity;GO:0004181|metallocarboxypep	.	.
ZFP36	758.0068	0.712072	0.185239593	3.844057	0.000121	0.003292	UP	yes	gi|111307030|gb|AAI20044.1|	965	1e-122	ZFP36 protein [Bos taurus]	gi|111307029|gb|BC120043.1|	1698	0	Bos taurus zinc finger protein 36, C3H type, homolog (mouse), mRNA (cDNA clone MGC:140545 IMAGE:8271704), complete cds	Q6S9E0	372	2e-122	mRNA decay activator protein ZFP36	ECU01g0830	100	2.71e-22	COG5063	pfam00642	zf-CCCH	Zinc finger C-x8-C-x5-C-x3-H type (and similar).	GO:0061158|3'-UTR-mediated mRNA destabilization;GO:0070935|3'-UTR-mediated mRNA stabilization;GO:0071364|cellular response to epidermal growth factor stimulus;GO:0044344|cellular response to fibroblast growth factor stimulus;GO:0071385|cellular response to glucocorticoid stimulus;GO:0097011|cellular response to granulocyte macrophage colony-stimulating factor stimulus;GO:0071222|cellular response to lipopolysaccharide;GO:0071356|cellular response to tumor necrosis factor;GO:0000165|MAPK cascade;GO:0035278|negative	GO:0005737|cytoplasm;GO:0000932|cytoplasmic mRNA processing body;GO:0010494|cytoplasmic stress granule;GO:0030529|ribonucleoprotein	GO:0071889|14-3-3 protein binding;GO:0017091|AU-rich element	K15308	TTP; tristetraprolin
BCKDK	463.3366	0.52965	0.137811233	3.8433	0.0001214	0.003292	Not_Sig	no	gi|114052434|ref|NP_001039371.1|	1941	0	[3-methyl-2-oxobutanoate dehydrogenase [lipoamide]] kinase, mitochondrial precursor [Bos taurus]	gi|942029615|ref|XM_005886976.2|	1818	0	PREDICTED: Bos mutus branched chain ketoacid dehydrogenase kinase (BCKDK), transcript variant X1, mRNA	Q2KJG8	752	0	[3-methyl-2-oxobutanoate dehydrogenase [lipoamide]] kinase, mitochondrial	SPAC644.11c	141	5.72e-36	COG0642	pfam10436	BCDHK_Adom3	Mitochondrial branched-chain alpha-ketoacid dehydrogenase kinase. Catabolism and synthesis of leucine, isoleucine and valine	GO:0009083|branched-chain amino acid catabolic process;GO:0016310|phosphorylation;	GO:0005947|mitochondrial alpha-ketoglutarate dehydrogenase complex;	GO:0047323|[3-methyl-2-oxobutanoate dehydrogenase	K00905	BCKDK; [3-methyl-2-oxobutanoate dehydrogenase (acetyl-transferring)]
FRMD6	1083.067	-0.40648	0.105775581	-3.84289	0.0001216	0.003292	Not_Sig	no	gi|555965096|ref|XP_005894608.1|	2928	0	PREDICTED: FERM domain-containing protein 6 isoform X1 [Bos mutus]	gi|982936928|ref|XM_005211692.3|	4789	0	PREDICTED: Bos taurus FERM domain containing 6 (FRMD6), transcript variant X1, mRNA	Q96NE9	1112	0	FERM domain-containing protein 6	.	.	.	.	pfam00373	FERM_M	FERM central domain. This domain is the central structural domain of the FERM domain.	GO:0031032|actomyosin structure organization;GO:0003383|apical constriction;GO:0034613|cellular protein localization;GO:0032970|regulation of actin filament-based process;	GO:0043296|apical junction complex;GO:0005737|cytoplasm;GO:0005856|cytoskeleton;GO:0005886|plasma membrane;	GO:0005200|structural constituent of cytoskeleton;	K16822	FRMD6; FERM domain-containing protein 6
RREB1	751.9358	0.535406	0.139336841	3.842527	0.0001218	0.003292	Not_Sig	no	gi|529004367|ref|XP_003587789.2|	6590	0	PREDICTED: ras-responsive element-binding protein 1 isoform X1 [Bos taurus]	gi|983005885|ref|XM_015469053.1|	8381	0	PREDICTED: Bos taurus ras responsive element binding protein 1 (RREB1), transcript variant X2, mRNA	Q92766	1677	0	Ras-responsive element-binding protein 1	.	.	.	.	pfam13465	zf-H2C2_2	Zinc-finger double domain.	GO:0007275|multicellular organismal development;GO:0000122|negative regulation of transcription from RNA polymerase II promoter;GO:0010634|positive regulation of epithelial cell migration;GO:2000394|positive regulation of lamellipodium morphogenesis;GO:0033601|positive regulation of mammary gland epithelial cell proliferation;GO:1900026|positive regulation of substrate adhesion-dependent cell spreading;GO:0045893|positive regulation of transcription, DNA-templated;GO:1903691|positive regulation of	GO:0005737|cytoplasm;GO:0070062|extracellular exosome;GO:0016604|nuclear body;GO:0016607|nuclear speck;GO:0005730|nucleolus;GO:0005654|	GO:0046872|metal ion binding;GO:0000979|RNA polymerase II	.	.
ARAF	1081.74	0.50159	0.130690731	3.837993	0.000124	0.003342	Not_Sig	no	gi|803200901|ref|XP_011961502.1|	3184	0	PREDICTED: serine/threonine-protein kinase A-Raf isoform X2 [Ovis aries]	gi|594069445|ref|XM_006058564.1|	2422	0	PREDICTED: Bubalus bubalis v-raf murine sarcoma 3611 viral oncogene homolog (ARAF), transcript variant X3, mRNA	O19004	1162	0	Serine/threonine-protein kinase A-Raf	.	.	.	.	pfam00069	Pkinase	Protein kinase domain.	GO:0035556|intracellular signal transduction;GO:0032434|regulation of proteasomal ubiquitin-dependent protein catabolic process;GO:0032006|regulation of TOR signaling;	GO:0005739|mitochondrion;	GO:0005524|ATP binding;GO:0046872|metal ion binding;GO:0004674	K08845	ARAF, ARAF1; A-Raf proto-oncogene serine/threonine-protein kinase
SIRPA	472.9948	0.54329	0.14157018	3.837602	0.0001242	0.003342	Not_Sig	no	gi|440898626|gb|ELR50080.1|	2421	0	Tyrosine-protein phosphatase non-receptor type substrate 1, partial [Bos mutus]	gi|942080481|ref|XM_005904362.2|	3736	0	PREDICTED: Bos mutus signal-regulatory protein alpha (SIRPA), mRNA	O46631	907	0	Tyrosine-protein phosphatase non-receptor type substrate 1	.	.	.	.	pfam07686	V-set	Immunoglobulin V-set domain. This domain is found in antibodies as well as neural protein P0 and CTL4 amongst others.	.	GO:0016021|integral component of membrane;	.	K06551	SIRPA_B1_G; signal-regulatory protein alpha/beta1/gamma
LDB1	880.3821	0.563307	0.146821503	3.836676	0.0001247	0.003342	Not_Sig	no	gi|537197247|gb|ERE78634.1|	1964	0	LIM domain-binding protein 1 [Cricetulus griseus]	gi|983007881|ref|XM_015469585.1|	1758	0	PREDICTED: Bos taurus LIM domain binding 1 (LDB1), transcript variant X3, mRNA	P70662	758	0	LIM domain-binding protein 1	.	.	.	.	pfam01803	LIM_bind	LIM-domain binding protein. The LIM-domain binding protein, binds to the LIM domain pfam00412 of LIM homeodomain proteins which	GO:0009948|anterior/posterior axis specification;GO:0022607|cellular component assembly;GO:0021702|cerebellar Purkinje cell differentiation;GO:0021549|cerebellum development;GO:0010669|epithelial structure maintenance;GO:0001702|gastrulation with mouth forming second;GO:0001942|hair follicle development;GO:0060322|head development;GO:0043973|histone H3-K4 acetylation;GO:0045647|negative regulation of erythrocyte differentiation;GO:0045892|negative regulation of transcription, DNA-	GO:0031252|cell leading edge;GO:0000790|nuclear chromatin;GO:0005634|nucleus;GO:0043234|protein	GO:0003682|chromatin binding;GO:0001158|enhancer	K15617	LDB1; LIM domain-binding protein 1

LOC102279357	585.5872	-0.4872	0.12699284	-3.83647	0.0001248	0.003342	Not_Sig	no	gi|742102497|ref|XP_010834531.1|	754	4e-96	PREDICTED: interferon alpha-inducible protein 27-like protein 2B isoform X3 [Bison bison bison]	gi|742102496|ref|XM_010836229.1|	846	0	PREDICTED: Bison bison bison interferon alpha-inducible protein 27, mitochondrial-like (LOC104985839), transcript variant X3, mRNA	P40305	81.6	9e-18	Interferon alpha-inducible protein 27, mitochondrial	.	.	.	.	pfam06140	Ifi-6-16	Interferon-induced 6-16 family.	GO:0006919|activation of cysteine-type endopeptidase activity involved in apoptotic process;GO:0097190|apoptotic signaling pathway;GO:0000122|negative regulation of transcription from RNA polymerase II promoter;GO:0046825|regulation of protein export from nucleus;GO:0060337|type I interferon signaling pathway;	GO:0016021|integral component of membrane;GO:0005741|mitochondrial outer membrane;GO:0005739|mitochondrion;GO:	GO:0005521|lamin binding;GO:0001102|RNA polymerase II activating	.	.
PITPNM1	574.8218	0.494	0.128781883	3.835946	0.0001251	0.003342	Not_Sig	no	gi|983009974|ref|XP_015325571.1|	6015	0	PREDICTED: LOW QUALITY PROTEIN: membrane-associated phosphatidylinositol transfer protein 1 isoform X2 [Bos taurus]	gi|983009973|ref|XM_015470085.1|	4146	0	PREDICTED: Bos taurus phosphatidylinositol transfer protein, membrane associated 1 (PITPNM1), transcript variant X2, mRNA	O00562	2164	0	Membrane-associated phosphatidylinositol transfer protein 1	CAC2938	101	3.24e-22	COG5083	pfam02121	IP_trans	Phosphatidylinositol transfer protein. Along with the structurally unrelated Sec14p family (found in pfam00650), this family can	GO:0007420|brain development;GO:0006629|lipid metabolic process;GO:0015914|phospholipid transport;GO:0007602|phototransduction;GO:0015031|protein transport;	GO:0032154|cleavage furrow;GO:0005737|cytoplasm;GO:0005789|endoplasmic reticulum membrane;GO:0032580|Golgi cisterna	GO:0046872|metal ion binding;GO:0070300|phosphatidic acid	.	.
NDUFAF8	92.77444	-0.83472	0.217791089	-3.83268	0.0001268	0.003372	DOWN	yes	gi|358417578|ref|XP_003583682.1|	305	2e-32	PREDICTED: uncharacterized protein C17orf89 homolog [Bos taurus]	gi|528921663|ref|XM_003583634.2|	638	0	PREDICTED: Bos taurus chromosome 19 open reading frame, human C17orf89 (C19H17orf89), mRNA	A1L188	104	3e-27	NADH dehydrogenase [ubiquinone] 1 alpha subcomplex assembly factor 8	.	.	.	.	.	.	.	.	GO:0005739|mitochondrion;	.	.	.
MCM7	1369.262	0.398947	0.104092678	3.832616	0.0001268	0.003372	Not_Sig	no	gi|115529274|ref|NP_001020516.2|	3794	0	DNA replication licensing factor MCM7 [Bos taurus]	gi|555962222|ref|XM_005893141.1|	2461	0	PREDICTED: Bos mutus minichromosome maintenance complex component 7 (MCM7), mRNA	Q3ZBH9	1466	0	DNA replication licensing factor MCM7	SPBC25D12.03c	663	0	COG1241	pfam00493	MCM	MCM2/3/5 family.	GO:0007049|cell cycle;GO:0008283|cell proliferation;GO:0006974|cellular response to DNA damage stimulus;GO:0071466|cellular response to xenobiotic stimulus;GO:0006270|DNA replication initiation;GO:0006268|DNA unwinding involved in DNA replication;GO:0042325|regulation of phosphorylation;	GO:0005737|cytoplasm;GO:0042555|MCM complex;GO:0016020|membrane;GO:000784|nuclear chromosome, telomeric region;GO:0005654|nucleoplasm;GO:00056	GO:0005524|ATP binding;GO:0004003|ATP-dependent DNA helicase	K02210	MCM7, CDC47; DNA replication licensing factor MCM7 [EC:3.6.4.12]
FURIN	2360.536	0.492132	0.128668094	3.824817	0.0001309	0.003461	Not_Sig	no	gi|154426092|gb|AAI51597.1|	3873	0	Furin (paired basic amino acid cleaving enzyme) [Bos taurus]	gi|594068825|ref|XM_006058270.1|	4018	0	PREDICTED: Bubalus bubalis furin (paired basic amino acid cleaving enzyme) (FURIN), transcript variant X2, mRNA	Q28193	1490	0	Furin	YNL238w_1	266	2.38e-77	COG1404	pfam00082	Peptidase_S8	Subtilase family. Subtilases are a family of serine proteases. They appear to have independently and convergently evolved an	GO:0016485|protein processing;	GO:0005615|extracellular space;GO:0005794|Golgi apparatus;GO:0016021|integral component of membrane;GO:0005886|plasma	GO:0046872|metal ion binding;GO:0004252|serine-type	K01349	FURIN, PCSK3; furin [EC:3.4.21.75]
RAP1GDS1	565.799	-0.43447	0.113603572	-3.82447	0.0001311	0.003461	Not_Sig	no	gi|440892682|gb|ELR45769.1|	3067	0	Rap1 GTPase-GDP dissociation stimulator 1, partial [Bos mutus]	gi|742158706|ref|XM_010848322.1|	3581	0	PREDICTED: Bison bison bison RAP1, GTP-GDP dissociation stimulator 1 (RAP1GDS1), transcript variant X1, mRNA	P52306	1137	0	Rap1 GTPase-GDP dissociation stimulator 1	.	.	.	.	pfam00514	Arm	Armadillo/beta-catenin-like repeat. Approx. 40 amino acid repeat. Tandem repeats form super-helix of helices that is	GO:0031034|myosin filament assembly;GO:0032471|negative regulation of endoplasmic reticulum calcium ion concentration;GO:0043547|positive regulation of GTPase activity;GO:0051561|positive regulation of mitochondrial calcium ion concentration;GO:0007264|small GTPase mediated signal transduction;GO:0014829|vascular smooth muscle contraction;	GO:0005829|cytosol;GO:0005783|endoplasmic reticulum;GO:0070062|extracellular exosome;GO:0005739|mitochondrion;	GO:0005096|GTPase activator activity;	.	.
ARHGEF39	168.0259	0.626683	0.163877194	3.824103	0.0001312	0.003461	UP	yes	gi|556749281|ref|XP_005970072.1|	1577	0	PREDICTED: rho guanine nucleotide exchange factor 39 [Pantholops hodgsonii]	gi|594081602|ref|XM_006064353.1|	1438	0	PREDICTED: Bubalus bubalis Rho guanine nucleotide exchange factor (GEF) 39 (ARHGEF39), transcript variant X2, mRNA	Q0P5E3	603	0	Rho guanine nucleotide exchange factor 39	.	.	.	.	pfam00621	RhoGEF	RhoGEF domain. Guanine nucleotide exchange factor for Rho/Rac/Cdc42-like GTPases Also called Dbl-homologous (DH)	GO:0030335|positive regulation of cell migration;GO:0035023|regulation of Rho protein signal transduction;	GO:0005886|plasma membrane;	GO:0005089|Rho guanyl-nucleotide exchange factor activity;	.	.
ATOX1	450.5587	-0.55783	0.145878518	-3.82395	0.0001313	0.003461	Not_Sig	no	gi|926698472|ref|XP_005683251.2|	452	2e-50	PREDICTED: copper transport protein ATOX1 [Capra hircus]	gi|982932467|ref|XM_005209648.3|	1533	0	PREDICTED: Bos taurus antioxidant 1 copper chaperone (ATOX1), transcript variant X2, mRNA	Q9XT28	143	1e-39	Copper transport protein ATOX1	SPBC1709.10c	48.1	9.76e-07	COG2608	pfam00403	HMA	Heavy-metal-associated domain.	GO:0006825|copper ion transport;	.	GO:0046872|metal ion binding;	K07213	ATOX1, ATX1, copZ, golB; copper chaperone
PTCH2	98.16097	0.811444	0.21233495	3.821527	0.0001326	0.003483	UP	yes	gi|528902519|ref|XP_005197961.1|	5466	0	PREDICTED: protein patched homolog 2 isoform X1 [Bos taurus]	gi|594056904|ref|XM_006052579.1|	4586	0	PREDICTED: Bubalus bubalis patched 2 (PTCH2), transcript variant X4, mRNA	Q9Y6C5	1975	0	Protein patched homolog 2	.	.	.	.	pfam12349	Sterol-sensing	Sterol-sensing domain of SREBP cleavage-activation. Sterol regulatory element-binding proteins (SREBPs) are membrane-	GO:0001709|cell fate determination;GO:0009957|epidermal cell fate specification;GO:0042633|hair cycle;GO:0045879|negative regulation of smoothened signaling pathway;GO:0045606|positive regulation of epidermal cell differentiation;GO:0043588|skin development;	GO:0016021|integral component of membrane;	GO:0097108|hedgehog family protein binding;GO:0008158|hedgehog receptor	K11101	PTCH2; patched 2
RAP1B	2349.349	-0.48192	0.126118922	-3.82119	0.0001328	0.003483	Not_Sig	no	gi|440894067|gb|ELR46625.1|	910	4e-115	Ras-related protein Rap-1b, partial [Bos mutus]	gi|982925391|ref|XM_005206449.3|	2105	0	PREDICTED: Bos taurus RAP1B, member of RAS oncogene family (RAP1B), transcript variant X1, mRNA	Q5RDM6	350	8e-115	Ras-related protein Rap-1b	.	.	.	.	pfam00071	Ras	Ras family. Includes sub-families Ras, Rab, Rac, Ral, Ran, Rap Ypt1 and more. Shares P-loop motif with GTP_EFTU, arf and	GO:0008283|cell proliferation;GO:0071320|cellular response to cAMP;GO:0061028|establishment of endothelial barrier;GO:2000301|negative regulation of synaptic vesicle exocytosis;GO:0070374|positive regulation of ERK1 and ERK2 cascade;GO:0032486|Rap protein signal transduction;GO:1901888|regulation of cell junction assembly;GO:2000114|regulation of establishment of cell polarity;	GO:0005911|cell-cell junction;GO:0005829|cytosol;GO:0070062|extracellular exosome;GO:0005811|lipid particle;GO:0005886|plasma membrane;	GO:0019003|GDP binding;GO:0005525|GTP binding;GO:0003924	K07836	RAP1B; Ras-related protein Rap-1B
BAG3	1239.624	0.571461	0.149569037	3.82072	0.0001331	0.003483	Not_Sig	no	gi|594048193|ref|XP_006048496.1|	2061	0	PREDICTED: BAG family molecular chaperone regulator 3 isoform X1 [Bubalus bubalis]	gi|942075976|ref|XM_014480466.1|	2555	0	PREDICTED: Bos mutus BCL2-associated athanogene 3 (BAG3), mRNA	Q9JLV1	467	2e-152	BAG family molecular chaperone regulator 3	.	.	.	.	pfam02179	BAG	BAG domain. Domain present in Hsp70 regulators.	GO:0007420|brain development;GO:0071260|cellular response to mechanical stimulus;GO:0097192|extrinsic apoptotic signaling pathway in absence of ligand;GO:0008625|extrinsic apoptotic signaling pathway via death domain receptors;GO:0010664|negative regulation of striated muscle cell apoptotic process;GO:0050821|protein stabilization;GO:0021510|spinal cord development;	GO:0005913|cell-cell adherens junction;GO:0005737|cytoplasm;GO:0005829|cytosol;GO:0043005|neuron projection;GO:0005886|plasma	GO:0000774|adenyl-nucleotide exchange factor activity;GO:0098641	K09557	BAG3; BCL2-associated athanogene 3
SCAMP4	574.0823	0.56884	0.148911257	3.819995	0.0001335	0.003485	Not_Sig	no	gi|965938129|ref|XP_004008834.3|	992	4e-124	PREDICTED: secretory carrier-associated membrane protein 4 isoform X1 [Ovis aries]	gi|742142147|ref|XM_010844538.1|	2077	0	PREDICTED: Bison bison bison secretory carrier membrane protein 4 (SCAMP4), transcript variant X2, mRNA	Q58DF6	348	4e-113	Secretory carrier-associated membrane protein 4	.	.	.	.	pfam04144	SCAMP	SCAMP family. In vertebrates, secretory carrier membrane proteins (SCAMPs) 1-3 constitute a family of putative membrane-	GO:0015031|protein transport;	GO:0016021|integral component of membrane;	.	.	.
PLXNA3	1041.269	0.542677	0.142083805	3.819412	0.0001338	0.003485	Not_Sig	no	gi|555996885|ref|XP_005910176.1|	9707	0	PREDICTED: plexin-A3 [Bos mutus]	gi|942098260|ref|XM_005910114.2|	6541	0	PREDICTED: Bos mutus plexin A3 (PLXNA3), mRNA	P51805	3539	0	Plexin-A3	.	.	.	.	pfam08337	Plexin_cytopl	Plexin cytoplasmic RasGAP domain. This family features the C-terminal regions of various plexins. Plexins are receptors for	GO:0021785|branchiomotor neuron axon guidance;GO:0021612|facial nerve structural organization;GO:0021766|hippocampus development;GO:0007275|multicellular organismal development;GO:0050919|negative chemotaxis;GO:0048843|negative regulation of axon extension involved in axon guidance;GO:1990138|neuron projection extension;GO:0051495|positive regulation of cytoskeleton organization;GO:0021860|pyramidal neuron development;GO:0048841|regulation of axon extension involved in axon	GO:0030054|cell junction;GO:0005887|integral component of plasma membrane;GO:0043231|intracellular	GO:0017154|semaphorin receptor activity;GO:0004888|transmembrane	K06820	PLXNA; plexin A
SPRYD7	411.0089	-0.49194	0.128832896	-3.81845	0.0001343	0.003486	Not_Sig	no	gi|84000383|ref|NP_001033295.1|	1056	5e-133	SPRY domain-containing protein 7 [Bos taurus]	gi|742100120|ref|XM_010835680.1|	2902	0	PREDICTED: Bison bison bison SPRY domain containing 7 (SPRYD7), mRNA	Q2T9X3	411	2e-134	SPRY domain-containing protein 7	.	.	.	.	pfam00622	SPRY	SPRY domain. SPRY Domain is named from SPla and the RYanodine Receptor. Domain of unknown function. Distant	.	.	.	.	.
TTYH3	479.434	0.584596	0.153120523	3.817881	0.0001346	0.003486	Not_Sig	no	gi|529007979|ref|XP_005225372.1|	2275	0	PREDICTED: protein tweety homolog 3 isoform X2 [Bos taurus]	gi|983007541|ref|XM_015469461.1|	4531	0	PREDICTED: Bos taurus tweety family member 3 (TTYH3), transcript variant X2, mRNA	Q9C0H2	804	0	Protein tweety homolog 3	.	.	.	.	pfam04906	Tweety	Tweety. The tweety (tty) gene has not been characterized at the protein level. However, it is thought to form a membrane	GO:0006821|chloride transport;GO:0034220|ion transmembrane transport;	GO:0034707|chloride channel complex;GO:0070062|extracellular exosome;GO:0005886|plasma membrane;	GO:0005254|chloride channel activity;GO:0005229|intracellular	.	.
AHDC1	417.8384	0.64918	0.170043912	3.81772	0.0001347	0.003486	UP	yes	gi|803220443|ref|XP_011970529.1|	5021	0	PREDICTED: AT-hook DNA-binding motif-containing protein 1 isoform X1 [Ovis aries musimon]	gi|330340350|ref|NM_001206409.1|	5780	0	Bos taurus AT hook, DNA binding motif, containing 1 (AHDC1), mRNA	Q5TGY3	1845	0	AT-hook DNA-binding motif-containing protein 1	.	.	.	.	pfam09770	PAT1	Topoisomerase II-associated protein PAT1. Members of this family are necessary for accurate chromosome transmission during	.	.	GO:0003677|DNA binding;	.	.
EIF4G1	8442.697	0.342602	0.089800748	3.815139	0.0001361	0.003513	Not_Sig	no	gi|555995668|ref|XP_005909575.1|	6275	0	PREDICTED: eukaryotic translation initiation factor 4 gamma 1 [Bos mutus]	gi|982976369|ref|XM_015462063.1|	5435	0	PREDICTED: Bos taurus eukaryotic translation initiation factor 4 gamma, 1 (EIF4G1), transcript variant X1, mRNA	Q04637	2275	0	Eukaryotic translation initiation factor 4 gamma 1	.	.	.	.	pfam02854	MIF4G	MIF4G domain. MIF4G is named after Middle domain of eukaryotic initiation factor 4G (eIF4G). Also occurs in NMD2p and CBP80. The	GO:0001662|behavioral fear response;GO:0000184|nuclear-transcribed mRNA catabolic process, nonsense-mediated decay;GO:0000289|nuclear-transcribed mRNA poly(A) tail shortening;GO:0045666|positive regulation of neuron differentiation;GO:0043488|regulation of mRNA stability;GO:0006446|regulation of translational initiation;GO:0006413|translational initiation;GO:0016032|viral process;	GO:0005913|cell-cell adherens junction;GO:0005737|cytoplasm;GO:0005829|cytosol;GO:0016281|eukaryotic translation initiation factor 4F	GO:0098641|cadherin binding involved in cell-cell adhesion;GO:00081	K03260	EIF4G; translation initiation factor 4G
CHAF1B	360.5408	0.494587	0.129654144	3.814665	0.0001364	0.003513	Not_Sig	no	gi|742096674|ref|XP_010833143.1|	2452	0	PREDICTED: chromatin assembly factor 1 subunit B isoform X3 [Bison bison bison]	gi|742096673|ref|XM_010834841.1|	2882	0	PREDICTED: Bison bison bison chromatin assembly factor 1, subunit B (p60) (CHAF1B), transcript variant X3, mRNA	Q13112	797	0	Chromatin assembly factor 1 subunit B	.	.	.	.	pfam15512	CAF-1_p60_C	Chromatin assembly factor complex 1 subunit p60, C-terminal. CAF-1_p60_C is a family of vertebral proteins that is involved	GO:0007049|cell cycle;GO:0031497|chromatin assembly;GO:0006281|DNA repair;GO:0006260|DNA replication;GO:0006335|DNA replication-dependent nucleosome assembly;GO:0006461|protein complex assembly;GO:0006355|regulation of transcription, DNA-templated;GO:0006351|transcription, DNA-templated;	GO:0033186|CAF-1 complex;GO:0005737|cytoplasm;GO:000790|nuclear chromatin;GO:0005654|nucleoplasm;GO:00	GO:0003682|chromatin binding;GO:0042393|histone	K10751	CHAF1B; chromatin assembly factor 1 subunit B
HIRA	629.064	0.445185	0.116766344	3.812613	0.0001375	0.003528	Not_Sig	no	gi|982951202|ref|XP_015331126.1|	4928	0	PREDICTED: protein HIRA isoform X1 [Bos taurus]	gi|942077091|ref|XM_005903278.2|	3692	0	PREDICTED: Bos mutus protein HIRA (LOC102277427), mRNA	P54198	1748	0	Protein HIRA	.	.	.	.	pfam07569	Hira	TUP1-like enhancer of split. The Hira proteins are found in a range of eukaryotes and are implicated in the assembly of repressive	GO:0009653|anatomical structure morphogenesis;GO:0016569|covalent chromatin modification;GO:0006336|DNA replication-independent nucleosome assembly;GO:0007369|gastrulation;GO:0042692|muscle cell differentiation;GO:0001649|osteoblast differentiation;GO:0006357|regulation of transcription from RNA polymerase II promoter;GO:0006351|transcription, DNA-templated;	GO:0070062|extracellular exosome;GO:0000790|nuclear chromatin;GO:0005654|nucleoplasm;GO:0005634|nucleus;GO:0016605|PML	GO:0003714|transcription corepressor activity;GO:0003700|transcription factor	K11293	HIRA, HIR1; protein HIRA/HIR1
TKT	3590.985	0.454055	0.11909584	3.812516	0.0001376	0.003528	Not_Sig	no	gi|440905488|gb|ELR55864.1|	3274	0	Transketolase, partial [Bos mutus]	gi|942056364|ref|XM_005896475.2|	2117	0	PREDICTED: Bos mutus transketolase (TKT), transcript variant X1, mRNA	Q6B855	1247	0	Transketolase	all4052	577	0	COG0021	pfam00456	Transketolase_N	Transketolase, thiamine diphosphate binding domain. This family includes transketolase enzymes EC:2.2.1.1. and also	GO:0008152|metabolic process;GO:0040008|regulation of growth;	.	GO:0046872|metal ion binding;GO:0004802|transketolase	K00615	E2.2.1.1, tktA, tktB; transketolase [EC:2.2.1.1]
KATNIP	340.5789	0.521364	0.136774183	3.811857	0.0001379	0.00353	Not_Sig	no	gi|555965041|ref|XP_005894581.1|	7613	0	PREDICTED: uncharacterized protein KIAA0556 homolog [Bos mutus]	gi|594094649|ref|XM_006070561.1|	6148	0	PREDICTED: Bubalus bubalis KIAA0556 ortholog (KIAA0556), mRNA	O60303	2331	0	Protein KIAA0556	.	.	.	.	pfam14652	DUF4457	Domain of unknown function (DUF4457). This family of proteins is found in eukaryotes. It is found repeated several times in the	.	GO:0042995|cell projection;GO:0005737|cytoplasm;GO:0005856|cytoskeleton;GO:0005615|extracellular space;GO:0005654|nucleoplasm;GO:000563	.	.	.
MAP3K2	555.5115	-0.51828	0.13598731	-3.8112	0.0001383	0.003531	Not_Sig	no	gi|358410815|ref|XP_580739.4|	3136	0	PREDICTED: mitogen-activated protein kinase kinase kinase 2 [Bos taurus]	gi|982977111|ref|XM_580739.9|	10589	0	PREDICTED: Bos taurus mitogen-activated protein kinase kinase kinase 2 (MAP3K2), mRNA	Q9Y2U5	1182	0	Mitogen-activated protein kinase kinase kinase 2	.	.	.	.	pfam00069	Pkinase	Protein kinase domain.	GO:0007257|activation of JUN kinase activity;GO:0000187|activation of MAPK activity;GO:0071260|cellular response to mechanical stimulus;GO:0045893|positive regulation of transcription, DNA-templated;GO:0006468|protein phosphorylation;	GO:0005737|cytoplasm;GO:0005829|cytosol;GO:0005654|nucleoplasm;	GO:0005524|ATP binding;GO:0004709|MAP kinase kinase kinase	K04420	MAP3K2, MEKK2; mitogen-activated protein kinase kinase kinase 2
FAM20C	351.5451	0.698472	0.183300736	3.810527	0.0001387	0.003533	UP	yes	gi|594111885|ref|XP_006078751.1|	2624	0	PREDICTED: extracellular serine/threonine protein kinase FAM20C [Bubalus bubalis]	gi|983007588|ref|XM_614520.9|	2230	0	PREDICTED: Bos taurus family with sequence similarity 20 member C (FAM20C), mRNA	Q5MJS3	914	0	Extracellular serine/threonine protein kinase FAM20C	.	.	.	.	pfam06702	DUF1193	Protein of unknown function (DUF1193). This family represents the C-terminus of several hypothetical eukaryotic proteins of	GO:0031214|biomineral tissue development;GO:0097187|dentinogenesis;GO:0070166|enamel mineralization;GO:0071895|odontoblast differentiation;GO:0036179|osteoclast maturation;GO:0030501|positive regulation of bone mineralization;GO:0045669|positive regulation of osteoblast differentiation;GO:0006468|protein phosphorylation;GO:0040036|regulation of fibroblast growth factor receptor signaling pathway;GO:0051174|regulation of phosphorus metabolic process;GO:0001501|skeletal system development;	GO:0070062|extracellular exosome;GO:0005615|extracellular space;GO:0005794|Golgi apparatus;	GO:0005524|ATP binding;GO:0005509|calcium ion binding;GO:0030145	.	.
LOC106701030	1434.215	-0.65868	0.172895885	-3.8097	0.0001391	0.003537	DOWN	yes	.	.	.	.	gi|942066034|ref|XR_001351589.1|	598	0	PREDICTED: Bos mutus uncharacterized LOC106701030 (LOC106701030), ncRNA	.	.	.	.	.	.	.	.	.	.	.	.	.	.	.	.
RNF44	283.955	0.699322	0.183590231	3.809147	0.0001394	0.003537	UP	yes	gi|528956112|ref|XP_005209198.1|	1953	0	PREDICTED: RING finger protein 44 isoform X1 [Bos taurus]	gi|594105301|ref|XM_006075546.1|	4880	0	PREDICTED: Bubalus bubalis ring finger protein 44 (RNF44), transcript variant X4, mRNA	Q7L0R7	675	0	RING finger protein 44	.	.	.	.	pfam13639	zf-RING_2	Ring finger domain.	GO:0043161|proteasome-mediated ubiquitin-dependent protein catabolic process;GO:0006355|regulation of transcription, DNA-templated;	GO:0005737|cytoplasm;GO:0005654|nucleoplasm;	GO:0061630|ubiquitin protein ligase activity;GO:0008270|zinc ion binding;	K19041	RNF38_44; E3 ubiquitin-protein ligase RNF38/44 [EC:2.3.2.27]
FKBP14	684.1715	-0.5247	0.137774052	-3.80843	0.0001399	0.003539	Not_Sig	no	gi|511885625|ref|XP_004762619.1|	1046	9e-132	PREDICTED: peptidyl-prolyl cis-trans isomerase FKBP14 [Mustela putorius furo]	gi|942071330|ref|XM_005901402.2|	2920	0	PREDICTED: Bos mutus FK506 binding protein 14, 22 kDa (FKBP14), mRNA	Q5R941	385	4e-125	Peptidyl-prolyl cis-trans isomerase FKBP14	YDR519w	103	2.81e-25	COG0545	pfam00254	FKBP_C	FKBP-type peptidyl-prolyl cis-trans isomerase.	GO:0006457|protein folding;	GO:0005788|endoplasmic reticulum lumen;	GO:0005509|calcium ion binding;GO:0003755|peptidyl-prolyl cis-	K09577	FKBP14; FK506-binding protein 14 [EC:5.2.1.8]
LOC138984345	499.7242	0.50674	0.133096562	3.807309	0.0001405	0.003548	Not_Sig	no	gi|742077187|ref|XP_010830917.1|	174	2e-21	PREDICTED: inverted formin-2-like [Bison bison bison]	gi|983004176|ref|XR_820611.2|	813	0	PREDICTED: Bos taurus uncharacterized LOC104975460 (LOC104975460), transcript variant X3, ncRNA	Q27J81	68.6	5e-19	Inverted formin-2	.	.	.	.	pfam02181	FH2	Formin Homology 2 Domain.	GO:0030036|actin cytoskeleton organization;GO:0090140|regulation of mitochondrial fission;	GO:0048471|perinuclear region of cytoplasm;	.	.	.
LOC138987978	1258.346	0.646543	0.170033364	3.802447	0.0001433	0.00361	UP	yes	.	.	.	.	gi|594038935|ref|XR_326303.1|	311	2e-160	PREDICTED: Bubalus bubalis uncharacterized LOC102408576 (LOC102408576), transcript variant X3, misc_RNA	.	.	.	.	.	.	.	.	pfam05788	Orbi_VP1	Orbivirus RNA-dependent RNA polymerase (VP1). This family consists of the RNA-dependent RNA polymerase protein VP1 from	.	.	.	.	.
RPS27L	1114.961	-0.57156	0.150422454	-3.79972	0.0001449	0.003642	Not_Sig	no	gi|556735404|ref|XP_005963314.1|	708	2e-91	PREDICTED: 40S ribosomal protein S27 isoform X1 [Pantholops hodgsonii]	gi|942051273|ref|XM_014477864.1|	693	0	PREDICTED: Bos mutus ribosomal protein S27-like (RPS27L), transcript variant X2, mRNA	Q3T0B7	156	8e-47	40S ribosomal protein S27-like	SPBC1685.10	120	9.29e-35	COG2051	pfam01667	Ribosomal_S27e	Ribosomal protein S27.	GO:0000028|ribosomal small subunit assembly;GO:0006412|translation;	GO:0022627|cytosolic small ribosomal subunit;	GO:0046872|metal ion binding;GO:0003735|structural	K02978	RP-S27e, RPS27; small subunit ribosomal protein S27e
FAM204A	517.8313	-0.4435	0.1168224	-3.79635	0.0001468	0.003674	Not_Sig	no	gi|555981869|ref|XP_005902847.1|	1032	3e-124	PREDICTED: protein FAM204A [Bos mutus]	gi|742199761|ref|XM_010859273.1|	5839	0	PREDICTED: Bison bison bison family with sequence similarity 204, member A (FAM204A), transcript variant X2, mRNA	Q3ZCI6	397	3e-124	Protein FAM204A	.	.	.	.	pfam07565	Band_3_cyto	Band 3 cytoplasmic domain. This family contains the cytoplasmic domain of the Band 3 anion exchange proteins that exchange	.	.	.	.	.
POLR2B	3278.353	-0.50424	0.132823646	-3.79635	0.0001468	0.003674	Not_Sig	no	gi|154707850|ref|NP_001092552.1|	6111	0	DNA-directed RNA polymerase II subunit RPB2 [Bos taurus]	gi|555974200|ref|XM_005899033.1|	3793	0	PREDICTED: Bos mutus polymerase (RNA) II (DNA directed) polypeptide B, 140kDa (POLR2B), transcript variant X1, mRNA	P30876	2356	0	DNA-directed RNA polymerase II subunit RPB2	YOR151c	1392	0	COG0085	pfam00562	RNA_pol_Rpb2_6	RNA polymerase Rpb2, domain 6. RNA polymerases catalyse the DNA dependent polymerization of RNA. Prokaryotes contain a single RNA	GO:0006370|7-methylguanosine mRNA capping;GO:0008543|fibroblast growth factor receptor signaling pathway;GO:0010467|gene expression;GO:0031047|gene silencing by RNA;GO:0000398|mRNA splicing, via spliceosome;GO:0050434|positive regulation of viral transcription;GO:0042795|snRNA transcription from RNA polymerase II promoter;GO:0035019|somatic stem cell population maintenance;GO:0006368|transcription elongation from RNA polymerase II promoter;GO:0006366|transcription from RNA polymerase II	GO:0005665|DNA-directed RNA polymerase II, core complex;GO:0016020|membrane;GO:0005654|nucleoplasm;GO:0005634|nucleus;	GO:0003682|chromatin binding;GO:0003677|DNA	K03010	RPB2, POLR2B; DNA-directed RNA polymerase II subunit RPB2
NR2F1	625.4577	0.512214	0.134959163	3.795325	0.0001475	0.003674	Not_Sig	no	gi|28461227|ref|NP_786998.1|	1872	0	COUP transcription factor 1 [Bos taurus]	gi|31343567|ref|NM_175804.2|	2130	0	Bos taurus nuclear receptor subfamily 2 group F member 1 (NR2F1), mRNA	Q9TTR8	725	0	COUP transcription factor 1	.	.	.	.	pfam00104	Hormone_recep	Ligand-binding domain of nuclear hormone receptor. This all helical domain is involved in binding the hormone in these receptors.	GO:0006351|transcription, DNA-templated;	GO:0005634|nucleus;	GO:0004879|RNA polymerase II transcription factor activity, ligand-	K08547	NR2F1, TFCOUP1; COUP transcription factor 1
ITGA3	1774.935	0.448992	0.118306686	3.795155	0.0001476	0.003674	Not_Sig	no	gi|942039958|ref|XP_005890393.2|	5188	0	PREDICTED: integrin alpha-3 [Bos mutus]	gi|942039957|ref|XM_005890331.2|	4878	0	PREDICTED: Bos mutus integrin, alpha 3 (antigen CD49C, alpha 3 subunit of VLA-3 receptor) (ITGA3), mRNA	F1MMS9	2005	0	Integrin alpha-3	.	.	.	.	pfam08441	Integrin_alpha2	Integrin alpha. This domain is found in integrin alpha and integrin alpha precursors to the C terminus of a number of	GO:0007155|cell adhesion;GO:0097062|dendritic spine maintenance;GO:0035640|exploration behavior;GO:0007229|integrin-mediated signaling pathway;GO:0030324|lung development;GO:0007613|memory;GO:0048333|mesodermal cell differentiation;GO:0035024|negative regulation of Rho protein signal transduction;GO:0072006|nephron development;GO:0001764|neuron migration;GO:0090004|positive regulation of establishment of protein localization to plasma membrane;GO:0030510|regulation of BMP signaling	GO:0016323|basolateral plasma membrane;GO:0060076|excitatory synapse;GO:0009897|external side of plasma	GO:0001948|glycoprotein binding;GO:0046872|metal ion	K06482	ITGA3; integrin alpha 3
LOC102287762	4348.953	-0.43677	0.115102306	-3.79459	0.0001479	0.003674	Not_Sig	no	gi|742096912|ref|XP_010833203.1|	778	2e-102	PREDICTED: cystatin-B isoform X1 [Bison bison bison]	gi|942090613|ref|XM_005907579.2|	638	0	PREDICTED: Bos mutus cystatin-B (LOC102287762), mRNA	P25417	207	2e-66	Cystatin-B	.	.	.	.	pfam00031	Cystatin	Cystatin domain. Very diverse family. Attempts to define separate sub-families failed. Typically, either the N-terminal or	.	GO:0005737|cytoplasm;	GO:0004869|cysteine-type endopeptidase inhibitor activity;	K13907	CSTA_B; cystatin-A/B
DIPK1A	822.8267	-0.5191	0.136829508	-3.7938	0.0001484	0.003674	Not_Sig	no	gi|134085777|ref|NP_001076969.1|	2231	0	protein FAM69A [Bos taurus]	gi|555965368|ref|XM_005894680.1|	2549	0	PREDICTED: Bos mutus family with sequence similarity 69, member A (FAM69A), transcript variant X1, mRNA	Q5R634	858	0	Protein FAM69A	.	.	.	.	pfam12260	PIP49_C	Protein-kinase domain of FAM69. This is the C-terminal region of a family of FAM69 proteins from Metazoa and Viridiplantae that are	.	GO:0005789|endoplasmic reticulum membrane;GO:0016021|integral component of membrane;	.	.	.
TSC2	868.6459	0.471567	0.124300098	3.793779	0.0001484	0.003674	Not_Sig	no	gi|555949259|ref|XP_005886844.1|	9095	0	PREDICTED: LOW QUALITY PROTEIN: tuberin [Bos mutus]	gi|983006773|ref|XM_003584007.4|	5686	0	PREDICTED: Bos taurus tuberous sclerosis 2 (TSC2), transcript variant X1, mRNA	P49816	3173	0	Tuberin	.	.	.	.	pfam03542	Tuberin	Tuberin. Tuberous sclerosis complex (TSC) is an autosomal dominant disorder and is characterized by the presence of	GO:0030030|cell projection organization;GO:0045184|establishment of protein localization;GO:0045792|negative regulation of cell size;GO:0050680|negative regulation of epithelial cell proliferation;GO:0046627|negative regulation of insulin receptor signaling pathway;GO:0043407|negative regulation of MAP kinase activity;GO:0051898|negative regulation of protein kinase B signaling;GO:0032007|negative regulation of TOR signaling;GO:0030178|negative regulation of Wnt signaling pathway;GO:0051291|protein	GO:0005901|caveola;GO:0005856|cytoskeleton;GO:0005829|cytosol;GO:0030425|dendrite;GO:0030426|growth cone;GO:0043231|intracellular membrane-	GO:0005096|GTPase activator activity;GO:0019902|phosphatase	K07207	TSC2; tuberous sclerosis 2
GALNT4	137.5089	-0.70328	0.185475733	-3.79174	0.0001496	0.003682	DOWN	yes	gi|555989113|ref|XP_005906388.1|	2917	0	PREDICTED: polypeptide N-acetylgalactosaminyltransferase 4 [Bos mutus]	gi|742237915|ref|XM_010832055.1|	5128	0	PREDICTED: Bison bison bison polypeptide N-acetylgalactosaminyltransferase 4 (LOC104982623), mRNA	Q8N4A0	1028	0	Polypeptide N-acetylgalactosaminyltransferase 4	CC2077	58.2	8.52e-08	COG1216	pfam00535	Glycos_transf_2	Glycosyl transferase family 2. Diverse family, transferring sugar from UDP-glucose, UDP-N-acetyl-galactosamine, GDP-mannose or	GO:0005975|carbohydrate metabolic process;GO:0016266|O-glycan processing;	GO:0070062|extracellular exosome;GO:0005794|Golgi apparatus;GO:0000139|Golgi membrane;GO:0016021|integral	GO:0030246|carbohydrate binding;GO:0046872|metal ion	K00710	GALNT; polypeptide N-acetylgalactosaminyltransferase
SLC16A7	111.9868	-0.80726	0.212939069	-3.79106	0.00015	0.003682	DOWN	yes	gi|115496420|ref|NP_001069804.1|	2399	0	monocarboxylate transporter 2 [Bos taurus]	gi|965927126|ref|XM_015094773.1|	6376	0	PREDICTED: Ovis aries solute carrier family 16 (monocarboxylate transporter), member 7 (SLC16A7), transcript variant X3, mRNA	O60669	744	0	Monocarboxylate transporter 2	AF0367	89	4.60e-17	COG0477	pfam07690	MFS_1	Major Facilitator Superfamily.	GO:0035873|lactate transmembrane transport;GO:0035879|plasma membrane lactate transport;GO:1901475|pyruvate transmembrane transport;	GO:0005887|integral component of plasma membrane;GO:0005886|plasma membrane;	GO:0015129|lactate transmembrane transporter activity;GO:0008028	K08184	SLC16A7; MFS transporter, MCP family, solute carrier family 16
TUBGCP3	1113.05	0.396669	0.104637881	3.790877	0.0001501	0.003682	Not_Sig	no	gi|555985654|ref|XP_005904699.1|	4565	0	PREDICTED: gamma-tubulin complex component 3 isoform X1 [Bos mutus]	gi|942081365|ref|XM_005904637.2|	3675	0	PREDICTED: Bos mutus tubulin, gamma complex associated protein 3 (TUBGCP3), transcript variant X1, mRNA	Q96CW5	1639	0	Gamma-tubulin complex component 3	.	.	.	.	pfam04130	Spc97_Spc98	Spc97 / Spc98 family. The spindle pole body (SPB) functions as the microtubule-organizing centre in yeast. Members of this family are	GO:0051298|centrosome duplication;GO:0031122|cytoplasmic microtubule organization;GO:0051415|interphase microtubule nucleation by interphase microtubule organizing center;GO:0007126|meiotic nuclear division;GO:0007020|microtubule nucleation;GO:0090307|mitotic spindle assembly;GO:0007338|single fertilization;	GO:0005814|centriole;GO:0005813|centrosome;GO:0005737|cytoplasm;GO:0005829|cytosol;GO:0000923|equatorial microtubule organizing center;GO:0008275|gamma-	GO:0043015|gamma-tubulin binding;GO:0005200|structural	K16570	TUBGCP3, GCP3; gamma-tubulin complex component 3

NXT2	781.6231	-0.52686	0.139007148	-3.79017	0.0001505	0.003682	Not_Sig	no	gi|154152161|ref|NP_001093823.1|	658	3e-77	NTF2-related export protein 2 [Bos taurus]	gi|982973401|ref|XM_005227868.3|	2633	0	PREDICTED: Bos taurus nuclear transport factor 2-like export factor 2 (NXT2), transcript variant X1, mRNA	A6QNX3	258	9e-79	NTF2-related export protein 2	.	.	.	.	pfam02136	NTF2	Nuclear transport factor 2 (NTF2) domain. This family includes the NTF2-like Delta-5-3-ketosteroid isomerase proteins.	GO:0051028|mRNA transport;GO:0015031|protein transport;	GO:0005737|cytoplasm;GO:0005654|nucleoplasm;GO:0005634|nucleus;GO:0048471|perinuclear region of cytoplasm;	.	K14285	NXT1_2, P15; NTF2-related export protein 1/2
LTBR	1137.829	0.518862	0.136897519	3.790147	0.0001506	0.003682	Not_Sig	no	gi|157073978|ref|NP_001096698.1|	1727	0	tumor necrosis factor receptor superfamily member 3 precursor [Bos taurus]	gi|402693479|ref|NM_001103228.2|	2060	0	Bos taurus lymphotoxin beta receptor (LTBR), mRNA	P36941	439	2e-145	Tumor necrosis factor receptor superfamily member 3	.	.	.	.	pfam00020	TNFR_c6	TNFR/NGFR cysteine-rich region.	GO:0006915|apoptotic process;GO:0071260|cellular response to mechanical stimulus;GO:0006955|immune response;GO:0006954|inflammatory response;GO:0007275|multicellular organismal development;GO:0043011|myeloid dendritic cell differentiation;GO:2001238|positive regulation of extrinsic apoptotic signaling pathway;GO:0043123|positive regulation of I-kappaB kinase/NF-kappaB signaling;GO:0046330|positive regulation of JNK cascade;GO:0042127|regulation of cell proliferation;GO:0032496|response	GO:0005887|integral component of plasma membrane;GO:0005886|plasma membrane;	GO:0005031|tumor necrosis factor-activated receptor activity;GO:0031625	K03159	TNFRSF3, LTBR; lymphotoxin beta receptor TNFR superfamily member
CARNMT1	1038.268	-0.60323	0.159163158	-3.79003	0.0001506	0.003682	DOWN	yes	gi|803337961|ref|XP_012027064.1|	2036	0	PREDICTED: carnosine N-methyltransferase isoform X2 [Ovis aries musimon]	gi|982986319|ref|XM_580351.8|	3913	0	PREDICTED: Bos taurus carnosine N-methyltransferase 1 (CARNMT1), mRNA	Q8N4J0	724	0	Carnosine N-methyltransferase	.	.	.	.	pfam07942	N2227	N2227-like protein. This family features sequences that are similar to a region of hypothetical yeast gene product N2227. This is	GO:0035498|carnosine metabolic process;	GO:0005829|cytosol;GO:0005634|nucleus;	GO:0030735|carnosine N-methyltransferase activity;	.	.
ZNF865	107.5192	0.998657	0.263580617	3.788809	0.0001514	0.003693	UP	yes	gi|965904340|ref|XP_014945571.1|	3108	0	PREDICTED: zinc finger protein 865 isoform X1 [Ovis aries musimon]	gi|983000762|ref|XM_010827753.2|	3392	0	PREDICTED: Bos taurus zinc finger protein 865 (ZNF865), mRNA	P0CJ78	707	0	Zinc finger protein 865	.	.	.	.	pfam13465	zf-H2C2_2	Zinc-finger double domain.	GO:0006357|regulation of transcription from RNA polymerase II promoter;GO:0006351|transcription, DNA-templated;	GO:0005634|nucleus;	GO:0003677|DNA binding;GO:0046872|metal ion binding;GO:0003712	.	.
LARP1	3375.496	0.448573	0.118547717	3.783905	0.0001544	0.003758	Not_Sig	no	gi|803285606|ref|XP_012002452.1|	4534	0	PREDICTED: la-related protein 1 isoform X3 [Ovis aries musimon]	gi|742226358|ref|XM_010829400.1|	4785	0	PREDICTED: Bison bison bison La ribonucleoprotein domain family, member 1 (LARP1), mRNA	Q6PKG0	1655	0	La-related protein 1	SPAC1527.03	75.1	8.42e-13	COG5193	pfam05383	La	La domain. This presumed domain is found at the N-terminus of La RNA-binding proteins as well as other proteins. The function of this	GO:0008283|cell proliferation;GO:0016239|positive regulation of macroautophagy;GO:0045070|positive regulation of viral genome replication;GO:0031929|TOR signaling;GO:0006413|translational initiation;	GO:0005913|cell-cell adherens junction;GO:0005737|cytoplasm;GO:0016020|membrane;GO:0005654|nucleoplasm;	GO:0098641|cadherin binding involved in cell-cell adhesion;GO:00081	K18757	LARP1; la-related protein 1
GGA1	478.6347	0.515057	0.136151924	3.782961	0.000155	0.003764	Not_Sig	no	gi|555966447|ref|XP_005895275.1|	2945	0	PREDICTED: ADP-ribosylation factor-binding protein GGA1 isoform X1 [Bos mutus]	gi|942053169|ref|XM_005895216.2|	1629	0	PREDICTED: Bos mutus golgi-associated, gamma adaptin ear containing, ARF binding protein 1 (GGA1), transcript variant X4, mRNA	Q9UJY5	1047	0	ADP-ribosylation factor-binding protein GGA1	.	.	.	.	pfam00790	VHS	VHS domain. Domain present in VPS-27, Hrs and STAM.	GO:0044267|cellular protein metabolic process;GO:0006886|intracellular protein transport;GO:0045732|positive regulation of protein catabolic process;GO:1901998|toxin transport;GO:0016192|vesicle-mediated transport;	GO:0030131|clathrin adaptor complex;GO:0010008|endosome membrane;GO:0005794|Golgi apparatus;GO:0043231|intracellular	.	K12404	GGA; ADP-ribosylation factor-binding protein GGA
MAP3K4	1542.997	0.364412	0.096343023	3.782445	0.0001553	0.003764	Not_Sig	no	gi|982935618|ref|XP_015328417.1|	7947	0	PREDICTED: mitogen-activated protein kinase kinase kinase 4 isoform X1 [Bos taurus]	gi|982988238|ref|XM_015464891.1|	5198	0	PREDICTED: Bos taurus mitogen-activated protein kinase kinase kinase 4 (MAP3K4), transcript variant X1, mRNA	Q9Y6R4	2827	0	Mitogen-activated protein kinase kinase kinase 4	.	.	.	.	pfam00069	Pkinase	Protein kinase domain.	GO:0000186|activation of MAPKK activity;GO:0060718|chorionic trophoblast cell differentiation;GO:0035556|intracellular signal transduction;GO:0019100|male germ-line sex determination;GO:0001890|placenta development;GO:0043507|positive regulation of JUN kinase activity;GO:1900745|positive regulation of p38MAPK cascade;GO:0051973|positive regulation of telomerase activity;GO:1904355|positive regulation of telomere capping;GO:0032212|positive regulation of telomere maintenance via	GO:0005737|cytoplasm;GO:0048471|perinuclear region of cytoplasm;	GO:0005524|ATP binding;GO:0004709|MAP kinase kinase kinase	K04428	MAP3K4, MEKK4; mitogen-activated protein kinase kinase kinase 4
TSC22D4	1383.225	0.600425	0.158799526	3.781023	0.0001562	0.003778	UP	yes	gi|594044095|ref|XP_006046551.1|	1432	0	PREDICTED: TSC22 domain family protein 4 [Bubalus bubalis]	gi|594044094|ref|XM_006046489.1|	2330	0	PREDICTED: Bubalus bubalis TSC22 domain family, member 4 (TSC22D4), mRNA	Q9Y3Q8	483	5e-161	TSC22 domain family protein 4	.	.	.	.	pfam01166	TSC22	TSC-22/dip/bun family.	GO:0045892|negative regulation of transcription, DNA-templated;GO:0006351|transcription, DNA-templated;	GO:0005737|cytoplasm;GO:0005634|nucleus;	GO:0003700|transcription factor activity, sequence-specific DNA binding;	.	.
NFRKB	453.1868	0.491726	0.130074095	3.780353	0.0001566	0.00378	Not_Sig	no	gi|297491963|ref|XP_002699276.1|	4728	0	PREDICTED: nuclear factor related to kappa-B-binding protein isoform X3 [Bos taurus]	gi|983009744|ref|XM_002703497.5|	5270	0	PREDICTED: Bos taurus nuclear factor related to kappaB binding protein (NFRKB), transcript variant X5, mRNA	Q6P4R8	1682	0	Nuclear factor related to kappa-B-binding protein	.	.	.	.	pfam14465	NFRKB_winged	NFRKB Winged Helix-like. This domain covers regions 370-495 of human nuclear factor related to kappaB binding (NFRKB) protein.	GO:0006310|DNA recombination;GO:0006281|DNA repair;GO:0006954|inflammatory response;GO:0006355|regulation of transcription, DNA-templated;GO:0006366|transcription from RNA polymerase II promoter;	GO:0031011|Ino80 complex;GO:0005654|nucleoplasm;GO:0005634|nucleus;	GO:0003677|DNA binding;GO:0002020|protease binding;	K11671	NFRKB, INO80G; nuclear factor related to kappa-B-binding protein
NDUFS5	417.3591	-0.52398	0.13864829	-3.77922	0.0001573	0.00379	Not_Sig	no	gi|28603768|ref|NP_788825.1|	558	3e-70	NADH dehydrogenase [ubiquinone] iron-sulfur protein 5 [Bos taurus]	gi|942091411|ref|XM_005907778.2|	588	0	PREDICTED: Bos mutus NADH dehydrogenase (ubiquinone) Fe-S protein 5, 15kDa (NADH-coenzyme Q reductase) (NDUFS5), mRNA	Q02379	219	9e-72	NADH dehydrogenase [ubiquinone] iron-sulfur protein 5	.	.	.	.	pfam10200	Ndufs5	NADH:ubiquinone oxidoreductase, NDUFS5-15kDa. This is a family of short, approximately 105 amino acid residue, proteins which form	GO:0032981|mitochondrial respiratory chain complex I assembly;GO:0055114|oxidation-reduction process;	GO:0005758|mitochondrial intermembrane space;GO:0005747|mitochondrial respiratory chain complex I;	.	K03938	NDUFS5; NADH dehydrogenase (ubiquinone) Fe-S protein 5
TGFB1I1	2083.464	0.572963	0.151639692	3.77845	0.0001578	0.003793	Not_Sig	no	gi|440913325|gb|ELR62789.1|	1989	0	Transforming growth factor beta-1-induced transcript 1 protein, partial [Bos mutus]	gi|982966978|ref|XM_005224909.3|	2472	0	PREDICTED: Bos taurus transforming growth factor beta 1 induced transcript 1 (TGFB1I1), transcript variant X2, mRNA	Q3MHZ4	769	0	Transforming growth factor beta-1-induced transcript 1 protein	.	.	.	.	pfam00412	LIM	LIM domain. This family represents two copies of the LIM structural domain.	GO:0030154|cell differentiation;GO:0030512|negative regulation of transforming growth factor beta receptor signaling pathway;GO:0010718|positive regulation of epithelial to mesenchymal transition;GO:0045893|positive regulation of transcription, DNA-templated;GO:0030511|positive regulation of transforming growth factor beta receptor signaling pathway;GO:0030579|ubiquitin-dependent SMAD protein catabolic process;GO:0016055|Wnt signaling pathway;	GO:0005737|cytoplasm;GO:0005856|cytoskeleton;GO:0031012|extracellular matrix;GO:0005925|focal adhesion;GO:0016363|nuclear matrix;	GO:0003713|transcription coactivator activity;GO:0008270|zinc ion binding;	.	.
SIDT2	1688.568	0.505458	0.133793702	3.777891	0.0001582	0.003794	Not_Sig	no	gi|555970455|ref|XP_005897249.1|	4250	0	PREDICTED: SID1 transmembrane family member 2 isoform X2 [Bos mutus]	gi|742230343|ref|XM_010830390.1|	4370	0	PREDICTED: Bison bison bison SID1 transmembrane family, member 2 (SIDT2), mRNA	Q8NBJ9	1576	0	SID1 transmembrane family member 2	.	.	.	.	pfam13965	SID-1_RNA_chan	dsRNA-gated channel SID-1. This is a family of proteins that are transmembrane dsRNA-gated channels. They passively transport	GO:0000902|cell morphogenesis;GO:0033227|dsRNA transport;GO:0042593|glucose homeostasis;GO:0061178|regulation of insulin secretion involved in cellular response to glucose stimulus;GO:0009749|response to glucose;GO:0003323|type B pancreatic cell development;GO:0044342|type B pancreatic cell proliferation;	GO:0016021|integral component of membrane;GO:0005765|lysosomal membrane;	GO:0003725|double-stranded RNA binding;GO:0051033|RNA	.	.
CEP170B	1088.145	0.498631	0.132093569	3.774829	0.0001601	0.003833	Not_Sig	no	gi|741967860|ref|XP_010815671.1|	6432	0	PREDICTED: centrosomal protein of 170 kDa protein B isoform X1 [Bos taurus]	gi|983004177|ref|XM_015468626.1|	5361	0	PREDICTED: Bos taurus centrosomal protein 170B (CEP170B), transcript variant X1, mRNA	Q9Y4F5	1920	0	Centrosomal protein of 170 kDa protein B	.	.	.	.	pfam15308	CEP170_C	CEP170 C-terminus. This family includes the C-terminus of centrosomal protein of 170 kDa (CEP170).	.	GO:0005737|cytoplasm;GO:0005874|microtubule;	.	K16463	CEP170; centrosomal protein CEP170
LOC138990207	79.92978	-0.86116	0.228855243	-3.76291	0.0001679	0.004012	DOWN	yes	gi|511832257|ref|XP_004740787.1|	588	2e-74	PREDICTED: C-Myc-binding protein [Mustela putorius furo]	gi|942091415|ref|XM_005907781.2|	604	0	PREDICTED: Bos mutus MYC binding protein (MYCBP), mRNA	Q2TBP7	204	5e-66	C-Myc-binding protein	.	.	.	.	pfam10359	Fmp27_WPPW	RNA pol II promoter Fmp27 protein domain. Fmp27_WPPW is a conserved domain of a family of proteins involved in RNA	GO:0006355|regulation of transcription, DNA-templated;GO:0006351|transcription, DNA-templated;	GO:0005737|cytoplasm;GO:0005634|nucleus;	GO:0003713|transcription coactivator activity;	.	.
POLE	262.7677	0.537848	0.143001577	3.761131	0.0001691	0.004032	Not_Sig	no	gi|329744594|ref|NP_001178358.1|	12152	0	DNA polymerase epsilon catalytic subunit A [Bos taurus]	gi|982950056|ref|XM_005217684.3|	8186	0	PREDICTED: Bos taurus polymerase (DNA directed), epsilon, catalytic subunit (POLE), transcript variant X1, mRNA	Q07864	4375	0	DNA polymerase epsilon catalytic subunit A	SPBC25H2.13c	1691	0	COG0417	pfam08490	DUF1744	Domain of unknown function (DUF1744). This domain is found on the epsilon catalytic subunit of DNA polymerase. It is found C	GO:0006287|base-excision repair, gap-filling;GO:0006260|DNA replication;GO:0006270|DNA replication initiation;GO:0000731|DNA synthesis involved in DNA repair;GO:0048568|embryonic organ development;GO:0000082|G1/S transition of mitotic cell cycle;GO:0006297|nucleotide-excision repair, DNA gap filling;GO:0000722|telomere maintenance via recombination;	GO:0008622|epsilon DNA polymerase complex;GO:0005654|nucleoplasm;GO:0005634|nucleus;GO:0005886|plasma membrane;	GO:0008408|3'-5' exonuclease activity;GO:0051539|4 iron, 4 sulfur	K02324	POLE1; DNA polymerase epsilon subunit 1 [EC:2.7.7.7]
G6PD	1616.369	0.509423	0.135495198	3.759713	0.0001701	0.004047	Not_Sig	no	gi|529014085|ref|XP_005227717.1|	2828	0	PREDICTED: glucose-6-phosphate 1-dehydrogenase isoform X1 [Bos taurus]	gi|982973046|ref|XM_005227660.3|	1953	0	PREDICTED: Bos taurus glucose-6-phosphate dehydrogenase (G6PD), transcript variant X1, mRNA	O55044	1008	0	Glucose-6-phosphate 1-dehydrogenase	YNL241c	456	1.15e-153	COG0364	pfam02781	G6PD_C	Glucose-6-phosphate dehydrogenase, C-terminal domain.	GO:0051156|glucose 6-phosphate metabolic process;GO:0006006|glucose metabolic process;GO:0006739|NADP metabolic process;GO:0006098|pentose-phosphate shunt;	.	GO:0004345|glucose-6-phosphate dehydrogenase activity;GO:0050661	K00036	G6PD, zwf; glucose-6-phosphate 1-dehydrogenase [EC:1.1.1.49]
FEM1C	516.2928	-0.45625	0.121393555	-3.75846	0.000171	0.004058	Not_Sig	no	gi|155372159|ref|NP_001094689.1|	3233	0	protein fem-1 homolog C [Bos taurus]	gi|982935836|ref|XM_010808835.2|	4085	0	PREDICTED: Bos taurus fem-1 homolog c (C. elegans) (FEM1C), transcript variant X2, mRNA	A7MB89	1249	0	Protein fem-1 homolog C	all2748	92.8	8.49e-19	COG0666	pfam12796	Ank_2	Ankyrin repeats (3 copies).	GO:0016567|protein ubiquitination;	GO:0005737|cytoplasm;GO:0005654|nucleoplasm;	.	.	.
AP2A2	1670.375	0.45453	0.120950215	3.757994	0.0001713	0.004058	Not_Sig	no	gi|115496658|ref|NP_001069170.1|	4676	0	AP-2 complex subunit alpha-2 [Bos taurus]	gi|115496657|ref|NM_001075702.1|	3201	0	Bos taurus adaptor related protein complex 2 alpha 2 subunit (AP2A2), mRNA	Q0VCK5	1807	0	AP-2 complex subunit alpha-2	.	.	.	.	pfam01602	Adaptin_N	Adaptin N terminal region. This family consists of the N terminal region of various alpha, beta and gamma subunits of the AP-1, AP-2	GO:0048268|clathrin coat assembly;GO:0006897|endocytosis;GO:0006886|intracellular protein transport;	GO:0030122|AP-2 adaptor complex;	GO:0008289|lipid binding;GO:0008565|protein transporter activity;	K11824	AP2A; AP-2 complex subunit alpha
NUP214	646.0186	0.543787	0.144725786	3.757361	0.0001717	0.00406	Not_Sig	no	gi|742189636|ref|XP_010855096.1|	6171	0	PREDICTED: nuclear pore complex protein Nup214 isoform X2 [Bison bison bison]	gi|942075872|ref|XM_005902770.2|	6452	0	PREDICTED: Bos mutus nucleoporin 214kDa (NUP214), mRNA	Q80U93	1806	0	Nuclear pore complex protein Nup214	.	.	.	.	pfam03154	Atrophin-1	Atrophin-1 family. Atrophin-1 is the protein product of the dentatorubral-pallidoluysian atrophy (DRPLA) gene. DRPLA	GO:0006406|mRNA export from nucleus;GO:0006611|protein export from nucleus;GO:0006606|protein import into nucleus;GO:0051726|regulation of cell cycle;	GO:1990876|cytoplasmic side of nuclear pore;GO:0005925|focal adhesion;GO:0043231|intracellular membrane-bounded	GO:0005049|nuclear export signal receptor activity;GO:0008139	K14317	NUP214, CAN; nuclear pore complex protein Nup214
RC3H2	1006.081	-0.48222	0.128450649	-3.75412	0.000174	0.004096	Not_Sig	no	gi|742140246|ref|XP_010842318.1|	5864	0	PREDICTED: roquin-2 isoform X1 [Bison bison bison]	gi|982991220|ref|XM_010817918.2|	8859	0	PREDICTED: Bos taurus ring finger and CCCH-type domains 2 (RC3H2), transcript variant X1, mRNA	Q9HBD1	2208	0	Roquin-2	.	.	.	.	pfam00642	zf-CCCH	Zinc finger C-x8-C-x5-C-x3-H type (and similar).	GO:0001782|B cell homeostasis;GO:0060173|limb development;GO:0048286|lung alveolus development;GO:0048535|lymph node development;GO:0035264|multicellular organism growth;GO:1901224|positive regulation of NIK/NF-kappaB signaling;GO:0009791|post-embryonic development;GO:0010608|posttranscriptional regulation of gene expression;GO:0000209|protein polyubiquitination;GO:0048536|spleen development;GO:0043029|T cell homeostasis;GO:0042098|T cell proliferation;GO:0061470|T follicular	GO:0009986|cell surface;GO:0005737|cytoplasm;GO:0000932|cytoplasmic mRNA processing body;GO:0016020|membrane;GO:0005654|	GO:0003677|DNA binding;GO:0016874|ligase activity;GO:0003729	K15690	RC3H; RING finger and CCCH-type zinc finger domain-containing protein
RPS23	4641.576	-0.59162	0.157591813	-3.75411	0.000174	0.004096	DOWN	yes	gi|556727449|ref|XP_005959475.1|	951	7e-126	PREDICTED: uncharacterized protein LOC102325901 [Pantholops hodgsonii]	gi|965795279|ref|XM_015089503.1|	669	0	PREDICTED: Ovis aries musimon ribosomal protein S23 (RPS23), mRNA	P62268	293	7e-100	40S ribosomal protein S23	YPR132w	233	2.53e-78	COG0048	pfam00164	Ribosom_S12_S23	Ribosomal protein S12/S23. This protein is known as S12 in bacteria and archaea and S23 in eukaryotes.	GO:0006412|translation;	GO:0022627|cytosolic small ribosomal subunit;GO:0005840|ribosome;GO:0015935|small ribosomal subunit;	GO:0003735|structural constituent of ribosome;GO:0045182|translation	K02973	RP-S23e, RPS23; small subunit ribosomal protein S23e
POSTN	24531.12	-0.36218	0.096510867	-3.75276	0.0001749	0.00411	Not_Sig	no	gi|528970766|ref|XP_005213601.1|	4144	0	PREDICTED: periostin isoform X1 [Bos taurus]	gi|942089104|ref|XM_005907142.2|	3372	0	PREDICTED: Bos mutus periostin, osteoblast specific factor (POSTN), transcript variant X1, mRNA	Q15063	1517	0	Periostin	all4647	81.6	2.66e-17	COG2335	pfam02469	Fasciclin	Fasciclin domain. This extracellular domain is found repeated four times in grasshopper fasciclin I as well as in proteins from mammals,	GO:1990523|bone regeneration;GO:0007155|cell adhesion;GO:0044344|cellular response to fibroblast growth factor stimulus;GO:0071560|cellular response to transforming growth factor beta stimulus;GO:0071356|cellular response to tumor necrosis factor;GO:0071307|cellular response to vitamin K;GO:0030198|extracellular matrix organization;GO:0001953|negative regulation of cell-matrix adhesion;GO:1900025|negative regulation of substrate adhesion-dependent cell spreading;GO:1990138|neuron projection	GO:0031012|extracellular matrix;GO:0005615|extracellular space;GO:0031594|neuromuscular junction;GO:0005578|proteinaceous	GO:0050839|cell adhesion molecule binding;GO:0008201|heparin	.	.
BCOR	515.1281	0.526348	0.140276036	3.752228	0.0001753	0.00411	Not_Sig	no	gi|529015634|ref|XP_005228352.1|	8836	0	PREDICTED: BCL-6 corepressor isoform X1 [Bos taurus]	gi|594069710|ref|XM_006058691.1|	6229	0	PREDICTED: Bubalus bubalis BCL6 corepressor (BCOR), transcript variant X1, mRNA	Q6W2J9	3111	0	BCL-6 corepressor	.	.	.	.	pfam12796	Ank_2	Ankyrin repeats (3 copies).	GO:0007507|heart development;GO:0035518|histone H2A monoubiquitination;GO:0030502|negative regulation of bone mineralization;GO:0000415|negative regulation of histone H3-K36 methylation;GO:0051572|negative regulation of histone H3-K4 methylation;GO:0070171|negative regulation of tooth mineralization;GO:0000122|negative regulation of transcription from RNA polymerase II promoter;GO:0045892|negative regulation of transcription, DNA-templated;GO:0042476|odontogenesis;GO:0060021|palate	GO:0005739|mitochondrion;GO:0005634|nucleus;	GO:0031072|heat shock protein binding;GO:0042826|histone	.	.
PER1	411.8571	0.703026	0.187513597	3.749199	0.0001774	0.004142	UP	yes	gi|741962997|ref|XP_010814325.1|	4136	0	PREDICTED: period circadian protein homolog 1 isoform X1 [Bos taurus]	gi|982957218|ref|XM_010816024.2|	8267	0	PREDICTED: Bos taurus period circadian clock 1 (PER1), transcript variant X1, mRNA	O15534	1170	0	Period circadian protein homolog 1	.	.	.	.	pfam12114	Period_C	Period protein 2/3C-terminal region. This domain is found in eukaryotes. This domain is typically between 164 to 200	GO:0032922|circadian regulation of gene expression;GO:0097167|circadian regulation of translation;GO:0007623|circadian rhythm;GO:0009649|entrainment of circadian clock;GO:0043153|entrainment of circadian clock by photoperiod;GO:0043966|histone H3 acetylation;GO:0070932|histone H3 deacetylation;GO:0043967|histone H4 acetylation;GO:2000323|negative regulation of glucocorticoid receptor signaling pathway;GO:0043124|negative regulation of I-kappaB kinase/NF-kappaB signaling;GO:0046329|negative regulation of JNK	GO:0005737|cytoplasm;GO:0005634|nucleus;	GO:0031490|chromatin DNA binding;GO:0070888|E-box	K02633	PER; period circadian protein
RARA	1094.667	0.502861	0.134128439	3.749102	0.0001775	0.004142	Not_Sig	no	gi|586448937|ref|XP_006832649.1|	2055	0	PREDICTED: retinoic acid receptor alpha isoform X1 [Chrysochloris asiatica]	gi|742135445|ref|XM_010842606.1|	3336	0	PREDICTED: Bison bison bison retinoic acid receptor, alpha (RARA), transcript variant X1, mRNA	P10276	780	0	Retinoic acid receptor alpha	.	.	.	.	pfam00105	zf-C4	Zinc finger, C4 type (two domains). In nearly all cases, this is the DNA binding domain of a nuclear hormone receptor. The alignment	GO:0043277|apoptotic cell clearance;GO:0071391|cellular response to estrogen stimulus;GO:0071222|cellular response to lipopolysaccharide;GO:0071300|cellular response to retinoic acid;GO:0060591|chondroblast differentiation;GO:0031076|embryonic camera-type eye development;GO:0060324|face development;GO:0007565|female pregnancy;GO:0007281|germ cell development;GO:0002068|glandular epithelial cell development;GO:0003417|growth plate cartilage development;GO:0021766|hippocampus	GO:0015629|actin cytoskeleton;GO:0009986|cell surface;GO:0005737|cytoplasm;GO:0030425|dendrite;GO:0043025|neuronal cell	GO:0051393|alpha-actinin binding;GO:0031490|chromatin DNA	K08527	NR1B1, RARA; retinoic acid receptor alpha
CAB39	926.2454	-0.43107	0.114990648	-3.74875	0.0001777	0.004142	Not_Sig	no	gi|742105638|ref|XP_010835284.1|	1877	0	PREDICTED: calcium-binding protein 39 [Bison bison bison]	gi|402692823|ref|NM_001046087.2|	3748	0	Bos taurus calcium binding protein 39 (CAB39), mRNA	Q9Y376	702	0	Calcium-binding protein 39	.	.	.	.	pfam08569	Mo25	Mo25-like. Mo25-like proteins are involved in both polarised growth and cytokinesis. In fission yeast Mo25 is localized alternately to the	GO:0007050|cell cycle arrest;GO:0071476|cellular hypotonic response;GO:0035556|intracellular signal transduction;GO:1901380|negative regulation of potassium ion transmembrane transport;GO:1901017|negative regulation of potassium ion transmembrane transporter activity;GO:2000687|negative regulation of rubidium ion transmembrane transporter activity;GO:2000681|negative regulation of rubidium ion transport;GO:0018105|peptidyl-serine phosphorylation;GO:0010800|positive regulation of peptidyl-threonine	GO:0005829|cytosol;GO:0070062|extracellular exosome;GO:1902554|serine/threonine protein kinase complex;	GO:0019900|kinase binding;GO:0030295|protein kinase activator	K08272	CAB39, MO25; calcium binding protein 39
LOC106701184	1660.832	-0.37322	0.09958993	-3.74757	0.0001786	0.004153	Not_Sig	no	gi|53986565|gb|AAV27183.1|	429	2e-49	unknown [Bos taurus]	gi|942075714|ref|XR_001351690.1|	737	0	PREDICTED: Bos mutus uncharacterized LOC106701184 (LOC106701184), ncRNA	.	.	.	.	.	.	.	.	pfam11236	DUF3037	Protein of unknown function (DUF3037). This bacterial family of proteins has no known function.	.	.	.	.	.
DPYSL4	209.2252	0.611228	0.163173555	3.745877	0.0001798	0.004173	UP	yes	gi|255003721|ref|NP_001157255.1|	2994	0	dihydropyrimidinase-related protein 4 [Bos taurus]	gi|255003720|ref|NM_001163783.1|	2357	0	Bos taurus dihydropyrimidinase like 4 (DPYSL4), mRNA	O35098	1088	0	Dihydropyrimidinase-related protein 4	BMEI1644	406	1.07e-132	COG0044	pfam01979	Amidohydro_1	Amidohydrolase family. This family of enzymes are a a large metal dependent hydrolase superfamily. The family includes Adenine	GO:0070997|neuron death;GO:0097485|neuron projection guidance;	GO:0005737|cytoplasm;	GO:0031005|filamin binding;GO:0016810|hydrolase activity, acting on carbon-	.	.
CXCL2	561.8612	0.598658	0.159858585	3.744921	0.0001805	0.004175	UP	yes	gi|296486444|tpg|DAA28557.1|	364	2e-39	TPA: chemokine (C-X-C motif) ligand 2 [Bos taurus]	gi|402745660|ref|NM_001046513.2|	1021	0	Bos taurus chemokine (C-X-C motif) ligand 3 (CXCL3), mRNA	O46678	126	7e-34	Growth-regulated alpha protein	.	.	.	.	pfam00048	IL8	Small cytokines (intecrine/chemokine), interleukin-8 like. Includes a number of secreted growth factors and	GO:0060326|cell chemotaxis;GO:0006955|immune response;GO:0006954|inflammatory response;	GO:0005615|extracellular space;	.	K05505	CXCL1_2_3, GRO; C-X-C motif chemokine 1/2/3
CAMTA2	1201.249	0.639724	0.170832874	3.744738	0.0001806	0.004175	UP	yes	gi|555988591|ref|XP_005906134.1|	5456	0	PREDICTED: calmodulin-binding transcription activator 2 isoform X1 [Bos mutus]	gi|982956484|ref|XM_005220176.3|	4393	0	PREDICTED: Bos taurus calmodulin binding transcription activator 2 (CAMTA2), transcript variant X4, mRNA	O94983	1742	0	Calmodulin-binding transcription activator 2	.	.	.	.	pfam03859	CG-1	CG-1 domain. CG-1 domains are highly conserved domains of about 130 amino-acid residues containing a predicted bipartite	GO:0014898|cardiac muscle hypertrophy in response to stress;GO:0045944|positive regulation of transcription from RNA polymerase II promoter;GO:0006357|regulation of transcription from RNA polymerase II promoter;	GO:0005634|nucleus;	GO:0003682|chromatin binding;GO:0042826|histone	.	.
MICU3	269.2125	-0.66848	0.178567319	-3.74359	0.0001814	0.004185	DOWN	yes	gi|329663430|ref|NP_001193026.1|	2289	0	calcium uptake protein 3, mitochondrial [Bos taurus]	gi|982969223|ref|XM_005225990.3|	4019	0	PREDICTED: Bos taurus mitochondrial calcium uptake family member 3 (MICU3), transcript variant X2, mRNA	Q86XE3	843	0	Calcium uptake protein 3, mitochondrial	.	.	.	.	pfam13833	EF-hand_8	EF-hand domain pair.	.	GO:0016021|integral component of membrane;GO:0005739|mitochondrion;	GO:0005509|calcium ion binding;	.	.
COX5B	1338.909	-0.50402	0.134745468	-3.74056	0.0001836	0.004215	Not_Sig	no	gi|555959147|ref|XP_005891684.1|	689	4e-90	PREDICTED: cytochrome c oxidase subunit 5B, mitochondrial isoform X2 [Bos mutus]	gi|942043023|ref|XM_005891622.2|	531	0	PREDICTED: Bos mutus cytochrome c oxidase subunit 5B, mitochondrial (LOC102286844), transcript variant X2, mRNA	P00428	267	2e-90	Cytochrome c oxidase subunit 5B, mitochondrial	.	.	.	.	pfam01215	COX5B	Cytochrome c oxidase subunit Vb.	GO:0042776|mitochondrial ATP synthesis coupled proton transport;GO:0006123|mitochondrial electron transport, cytochrome c to oxygen;	GO:0005751|mitochondrial respiratory chain complex IV;GO:0045277|respiratory chain complex IV;	GO:0004129|cytochrome-c oxidase activity;GO:0046872|metal ion binding;	K02265	COX5B; cytochrome c oxidase subunit 5b
FAM83H	301.7434	0.639348	0.170939965	3.740187	0.0001839	0.004215	UP	yes	gi|528975329|ref|XP_005215330.1|	5298	0	PREDICTED: protein FAM83H isoform X2 [Bos taurus]	gi|982994117|ref|XM_015466259.1|	5018	0	PREDICTED: Bos taurus family with sequence similarity 83 member H (FAM83H), transcript variant X1, mRNA	Q6ZRV2	1745	0	Protein FAM83H	.	.	.	.	pfam07894	DUF1669	Protein of unknown function (DUF1669). This family is composed of sequences derived from hypothetical eukaryotic	GO:0031214|biomineral tissue development;GO:0045104|intermediate filament cytoskeleton organization;GO:0030335|positive regulation of cell migration;GO:0044380|protein localization to cytoskeleton;	GO:0005737|cytoplasm;GO:0005856|cytoskeleton;	GO:1990254|keratin filament binding;GO:0019901|protein kinase	.	.
MYO19	646.9074	0.507288	0.135635946	3.740071	0.000184	0.004215	Not_Sig	no	gi|555960545|ref|XP_005892376.1|	4783	0	PREDICTED: unconventional myosin-XIX [Bos mutus]	gi|254939550|ref|NM_001024501.2|	3131	0	Bos taurus myosin XIX (MYO19), mRNA	Q96H55	1502	0	Unconventional myosin-XIX	SPCC1919.10c	480	1.38e-145	COG5022	pfam00063	Myosin_head	Myosin head (motor domain).	.	GO:0005737|cytoplasm;GO:0005741|mitochondrial outer membrane;GO:0016459|myosin complex;	GO:0005524|ATP binding;GO:0016887|ATPase activity;GO:0060002	.	.
PSPH	889.5246	-0.47697	0.127540859	-3.73976	0.0001842	0.004215	Not_Sig	no	gi|440913316|gb|ELR62780.1|	1215	2e-161	Phosphoserine phosphatase, partial [Bos mutus]	gi|742107657|ref|XM_010837387.1|	1406	0	PREDICTED: Bison bison bison phosphoserine phosphatase (PSPH), transcript variant X1, mRNA	Q2KHU0	466	2e-160	Phosphoserine phosphatase	L0085	89	2.32e-19	COG0560	pfam00702	Hydrolase	haloacid dehalogenase-like hydrolase. This family is structurally different from the alpha/beta hydrolase family	GO:0006564|L-serine biosynthetic process;GO:0006563|L-serine metabolic process;	GO:0005737|cytoplasm;	GO:0005509|calcium ion binding;GO:0000287|magnesium ion	K01079	serB, PSPH; phosphoserine phosphatase [EC:3.1.3.3]
PRKD2	258.0246	0.544557	0.145631356	3.739286	0.0001845	0.004215	Not_Sig	no	gi|983000223|ref|XP_001251452.5|	4078	0	PREDICTED: serine/threonine-protein kinase D2 isoform X2 [Bos taurus]	gi|742174113|ref|XM_010852642.1|	3293	0	PREDICTED: Bison bison bison protein kinase D2 (PRKD2), transcript variant X3, mRNA	Q9BZL6	1517	0	Serine/threonine-protein kinase D2	.	.	.	.	pfam00069	Pkinase	Protein kinase domain.	GO:0002250|adaptive immune response;GO:0001525|angiogenesis;GO:0007155|cell adhesion;GO:0008219|cell death;GO:0035924|cellular response to vascular endothelial growth factor stimulus;GO:0061154|endothelial tube morphogenesis;GO:0035556|intracellular signal transduction;GO:0018105|peptidyl-serine phosphorylation;GO:0045766|positive regulation of angiogenesis;GO:0043536|positive regulation of blood vessel endothelial cell migration;GO:0045785|positive regulation of cell adhesion;GO:0032793|positive regulation of CREB	GO:0005737|cytoplasm;GO:0005794|Golgi apparatus;GO:0005654|nucleoplasm;GO:0005634|nucleus;GO:0005886|plasma membrane;	GO:0005524|ATP binding;GO:0046872|metal ion binding;GO:0004672	K06070	PKD; protein kinase D [EC:2.7.11.13]
unassigned_gene_4	49.28494	-1.06281	0.284262354	-3.73883	0.0001849	0.004215	DOWN	yes	gi|345543|pir||JC1348	187	2e-21	hypothetical 18K protein - goldfish mitochondrion	gi|817597817|gb|KR011113.1|	1571	0	Bos grunniens breed Qinghai Plateau yak mitochondrion, complete genome	.	.	.	.	.	.	.	.	pfam15040	Humanin	Humanin family. This family of proteins is found exclusively in humans. Humanin is a short anti-apoptotic peptide that interacts	.	.	.	.	.
MRPL33	468.9545	-0.73285	0.196085842	-3.73741	0.0001859	0.00423	DOWN	yes	gi|164518974|ref|NP_001106779.1|	323	1e-35	39S ribosomal protein L33, mitochondrial [Bos taurus]	gi|926704577|ref|XM_005686871.2|	505	0	PREDICTED: Capra hircus mitochondrial ribosomal protein L33 (MRPL33), mRNA	Q3SZ47	129	3e-37	39S ribosomal protein L33, mitochondrial	DR2049	47	8.33e-07	COG0267	pfam00471	Ribosomal_L33	Ribosomal protein L33.	GO:0070125|mitochondrial translational elongation;GO:0070124|mitochondrial translational initiation;	GO:0005743|mitochondrial inner membrane;GO:0005762|mitochondrial large ribosomal subunit;	GO:0003735|structural constituent of ribosome;	K02913	RP-L33, MRPL33, rpmG; large subunit ribosomal protein L33
PPFIBP1	1960.37	-0.39291	0.105196456	-3.73505	0.0001877	0.004255	Not_Sig	no	gi|741927425|ref|XP_010803694.1|	4952	0	PREDICTED: liprin-beta-1 isoform X1 [Bos taurus]	gi|982926590|ref|XM_010805392.2|	5135	0	PREDICTED: Bos taurus PTPRF interacting protein, binding protein 1 (liprin beta 1) (PPFIBP1), transcript variant X1, mRNA	Q86W92	1683	0	Liprin-beta-1	.	.	.	.	pfam00536	SAM_1	SAM domain (Sterile alpha motif). It has been suggested that SAM is an evolutionarily conserved protein binding domain that is	GO:0007155|cell adhesion;	GO:0005913|cell-cell adherens junction;GO:0005925|focal adhesion;GO:0005886|plasma membrane;	GO:0098641|cadherin binding involved in cell-cell adhesion;	.	.

TMEM167A	1036.143	-0.45115	0.120803167	-3.73456	0.000188	0.004255	Not_Sig	no	gi|548475862|ref|XP_005683450.1|	303	1e-28	PREDICTED: protein kish-A [Capra hircus]	gi|742216647|ref|XM_010863377.1|	4703	0	PREDICTED: Bison bison bison transmembrane protein 167A (TMEM167A), mRNA	Q5RAL1	120	4e-30	Protein kish-A	.	.	.	.	pfam06842	DUF1242	Protein of unknown function (DUF1242). This family consists of a number of eukaryotic proteins of around 72 residues in length. The	.	GO:0000139|Golgi membrane;GO:0016021|integral component of membrane;	.	.	.
ZNF384	667.3797	0.568118	0.152141581	3.734143	0.0001884	0.004255	Not_Sig	no	gi|767971224|ref|XP_011519219.1|	2570	0	PREDICTED: zinc finger protein 384 isoform X1 [Homo sapiens]	gi|742143879|ref|XM_010844949.1|	3331	0	PREDICTED: Bison bison bison zinc finger protein 384 (ZNF384), transcript variant X2, mRNA	Q8TF68	912	0	Zinc finger protein 384	.	.	.	.	pfam13465	zf-H2C2_2	Zinc-finger double domain.	GO:0006355|regulation of transcription, DNA-templated;GO:0006351|transcription, DNA-templated;	GO:0005634|nucleus;	GO:0003677|DNA binding;GO:0046872|metal ion binding;	.	.
NYAP1	495.9115	0.465607	0.12469468	3.73398	0.0001885	0.004255	Not_Sig	no	gi|529007811|ref|XP_003587890.2|	3278	0	PREDICTED: neuronal tyrosine-phosphorylated phosphoinositide-3-kinase adapter 1 isoform X1 [Bos taurus]	gi|983007277|ref|XM_015469389.1|	3430	0	PREDICTED: Bos taurus neuronal tyrosine-phosphorylated phosphoinositide-3-kinase adaptor 1 (NYAP1), transcript variant X1, mRNA	Q6ZVC0	1159	0	Neuronal tyrosine-phosphorylated phosphoinositide-3-kinase adapter 1	.	.	.	.	pfam15452	NYAP_C	Neuronal tyrosine-phosphorylated phosphoinositide-3-kinase adapter. NYAP_C is a C-terminal family of eukaryotic proteins that	GO:0048812|neuron projection morphogenesis;GO:0014065|phosphatidylinositol 3-kinase signaling;	GO:0005622|intracellular;	.	.	.
ND5	2934.088	0.533549	0.142944792	3.732552	0.0001895	0.004267	Not_Sig	no	gi|147744501|ref|YP_001218801.1|	2379	0	NADH dehydrogenase subunit 5 [Bos grunniens]	gi|817597817|gb|KR011113.1|	1821	0	Bos grunniens breed Qinghai Plateau yak mitochondrion, complete genome	Q576B4	894	0	NADH-ubiquinone oxidoreductase chain 5	AGc2360	279	1.34e-84	COG1009	pfam00361	Oxidored_q1	NADH-Ubiquinone/plastoquinone (complex I), various chains. This family is part of complex I which catalyses the transfer of two	GO:0042773|ATP synthesis coupled electron transport;	GO:0016021|integral component of membrane;GO:0005743|mitochondrial inner membrane;GO:0070469|respiratory chain;	GO:0008137|NADH dehydrogenase (ubiquinone) activity;	.	.
XIRP1	339.6451	0.611865	0.163956266	3.731876	0.0001901	0.004267	UP	yes	gi|555978496|ref|XP_005901187.1|	8289	0	PREDICTED: xin actin-binding repeat-containing protein 1 isoform X1 [Bos mutus]	gi|555978495|ref|XM_005901125.1|	6223	0	PREDICTED: Bos mutus xin actin binding repeat containing 1 (XIRP1), transcript variant X1, mRNA	Q702N8	2176	0	Xin actin-binding repeat-containing protein 1	.	.	.	.	pfam08043	Xin	Xin repeat. The repeat has the consensus sequence GDV(K/Q/R)(T/S/G)X(R/K/T) WLFETXPLD. This repeat motif is	GO:0030036|actin cytoskeleton organization;GO:0007507|heart development;	GO:0030054|cell junction;	GO:0044822|poly (A) RNA binding;	.	.
MRPS2	756.2351	0.581767	0.155912954	3.731357	0.0001905	0.004267	Not_Sig	no	gi|149642651|ref|NP_001092347.1|	1242	8e-167	28S ribosomal protein S2, mitochondrial [Bos taurus]	gi|402743770|ref|NM_001098877.2|	1294	0	Bos taurus mitochondrial ribosomal protein S2 (MRPS2), mRNA	P82923	483	3e-168	28S ribosomal protein S2, mitochondrial	RC0112	126	2.18e-32	COG0052	pfam00318	Ribosomal_S2	Ribosomal protein S2.	GO:0032543|mitochondrial translation;GO:0070125|mitochondrial translational elongation;GO:0070124|mitochondrial translational initiation;GO:0098779|mitophagy in response to mitochondrial depolarization;GO:0002230|positive regulation of defense response to virus by host;GO:0098792|xenophagy;	GO:0005743|mitochondrial inner membrane;GO:0005763|mitochondrial small ribosomal subunit;	GO:0003735|structural constituent of ribosome;	K02967	RP-S2, MRPS2, rpsB; small subunit ribosomal protein S2
TRRAP	1230.646	0.45029	0.120679994	3.731277	0.0001905	0.004267	Not_Sig	no	gi|914927235|ref|XP_013220383.1|	19322	0	PREDICTED: transformation/transcription domain-associated protein [Ictidomys tridecemlineatus]	gi|982967192|ref|XM_005225048.3|	9019	0	PREDICTED: Bos taurus transformation/transcription domain-associated protein (TRRAP), transcript variant X1, mRNA	Q9Y4A5	7405	0	Transformation/transcription domain-associated protein	.	.	.	.	pfam02259	FAT	FAT domain. The FAT domain is named after FRAP, ATM and TRRAP.	GO:0006281|DNA repair;GO:0016573|histone acetylation;GO:0016578|histone deubiquitination;GO:0043968|histone H2A acetylation;GO:0043967|histone H4 acetylation;GO:0006355|regulation of transcription, DNA-templated;GO:0006351|transcription, DNA-templated;	GO:0005794|Golgi apparatus;GO:0035267|NuA4 histone acetyltransferase complex;GO:0005654|nucleoplasm;GO:000	GO:0016301|kinase activity;GO:0003712|transcription cofactor activity;	K08874	TRRAP; transformation/transcription domain-associated protein
MIER3	300.6416	-0.53555	0.143635506	-3.72854	0.0001926	0.004305	Not_Sig	no	gi|754169297|ref|NP_001291684.1|	2631	0	mesoderm induction early response protein 3 [Bos taurus]	gi|942058987|ref|XM_005897457.2|	5173	0	PREDICTED: Bos mutus mesoderm induction early response 1, family member 3 (MIER3), transcript variant X2, mRNA	Q4R3R9	993	0	Mesoderm induction early response protein 3	.	.	.	.	pfam01448	ELM2	ELM2 domain. The ELM2 (Egl-27 and MTA1 homology 2) domain is a small domain of unknown function. It is found in the MTA1	GO:0006355|regulation of transcription, DNA-templated;GO:0006351|transcription, DNA-templated;	GO:0005634|nucleus;	GO:0003677|DNA binding;	.	.
LOC138988566	348.9097	-0.50509	0.135609407	-3.72459	0.0001956	0.004365	Not_Sig	no	gi|529008331|ref|XP_005225506.1|	157	3e-10	PREDICTED: peroxisomal N(1)-acetyl-spermine/spermidine oxidase isoform X1 [Bos taurus]	gi|742166728|ref|XR_826171.1|	3016	0	PREDICTED: Bison bison bison uncharacterized LOC104996298 (LOC104996298), ncRNA	.	.	.	.	.	.	.	.	.	.	.	.	.	.	.	.
LOC138988001	101.816	-0.82755	0.222230085	-3.72383	0.0001962	0.004369	DOWN	yes	.	.	.	.	gi|850480667|gb|CP011888.1|	177	9e-86	Ovis canadensis canadensis isolate 43U chromosome 3 sequence	.	.	.	.	.	.	.	.	pfam09909	DUF2138	Uncharacterized protein conserved in bacteria (DUF2138). This domain, found in various hypothetical prokaryotic proteins,	.	.	.	.	.
BCAP29	377.2701	-0.51774	0.139077525	-3.7227	0.0001971	0.00438	Not_Sig	no	gi|84000125|ref|NP_001033164.1|	1030	1e-134	B-cell receptor-associated protein 29 [Bos taurus]	gi|742131032|ref|XM_010841469.1|	1549	0	PREDICTED: Bison bison bison B-cell receptor-associated protein 29 (BCAP29), transcript variant X1, mRNA	Q32KL9	401	4e-136	B-cell receptor-associated protein 29	.	.	.	.	pfam05529	Bap31	B-cell receptor-associated protein 31-like. Bap31 is a polytopic integral protein of the endoplasmic reticulum membrane	GO:0006915|apoptotic process;GO:0006888|ER to Golgi vesicle-mediated transport;GO:0006886|intracellular protein transport;GO:0001649|osteoblast differentiation;GO:0070973|protein localization to endoplasmic reticulum exit site;	GO:0005789|endoplasmic reticulum membrane;GO:0005887|integral component of plasma membrane;	.	.	.
METTL5	410.6411	-0.59875	0.160890477	-3.72147	0.0001981	0.004393	DOWN	yes	gi|432098398|gb|ELK28198.1|	833	3e-102	Methyltransferase-like protein 5 [Myotis davidii]	gi|850477813|gb|CP011887.1|	1385	0	Ovis canadensis canadensis isolate 43U chromosome 2 sequence	Q9NRN9	317	4e-100	Methyltransferase-like protein 5	ECU08g1860	116	5.87e-29	COG2263	pfam05175	MTS	Methyltransferase small domain. This domain is found in ribosomal RNA small subunit methyltransferase C as well as	.	.	GO:0008168|methyltransferase activity;GO:0003676|nucleic acid	.	.
ZFP64	476.6024	0.521731	0.140221027	3.720776	0.0001986	0.004395	Not_Sig	no	gi|157168343|ref|NP_001096757.1|	3406	0	zinc finger protein 64 [Bos taurus]	gi|157168342|ref|NM_001103287.1|	2585	0	Bos taurus ZFP64 zinc finger protein (ZFP64), mRNA	Q9NPA5	1163	0	Zinc finger protein 64 homolog, isoforms 1 and 2	.	.	.	.	pfam13912	zf-C2H2_6	C2H2-type zinc finger.	GO:0006355|regulation of transcription, DNA-templated;GO:0006351|transcription, DNA-templated;	GO:0005634|nucleus;	GO:0003677|DNA binding;GO:0046872|metal ion binding;	.	.
TNFAIP6	69.52791	-0.8951	0.240590802	-3.72041	0.0001989	0.004395	DOWN	yes	gi|56118243|ref|NP_001007814.1|	1388	0	tumor necrosis factor-inducible gene 6 protein precursor [Bos taurus]	gi|942051917|ref|XM_005894773.2|	1439	0	PREDICTED: Bos mutus tumor necrosis factor, alpha-induced protein 6 (TNFAIP6), mRNA	P98065	511	2e-179	Tumor necrosis factor-inducible gene 6 protein	.	.	.	.	pfam00431	CUB	CUB domain.	GO:0007155|cell adhesion;GO:0050728|negative regulation of inflammatory response;	.	GO:0005540|hyaluronic acid binding;	K19018	TNFAIP6, TSG6; tumor necrosis factor-inducible gene 6 protein
SLC4A2	3331.898	0.385153	0.103599664	3.717706	0.000201	0.004434	Not_Sig	no	gi|440895344|gb|ELR47558.1|	5112	0	Anion exchange protein 2, partial [Bos mutus]	gi|741924627|ref|XM_005205793.2|	4023	0	PREDICTED: Bos taurus solute carrier family 4 (anion exchanger), member 2 (SLC4A2), transcript variant X1, mRNA	Q6SJP2	1851	0	Anion exchange protein 2	.	.	.	.	pfam00955	HCO3_cotransp	HCO3- transporter family. This family contains Band 3 anion exchange proteins that exchange CL-/HCO3-. This family also	GO:1902476|chloride transmembrane transport;GO:0051453|regulation of intracellular pH;	GO:0005887|integral component of plasma membrane;	GO:0008509|anion transmembrane transporter activity;GO:0015301	K13855	SLC4A2, AE2; solute carrier family 4 (anion exchanger), member 2
ENG	2729.18	0.533654	0.143597609	3.716316	0.0002021	0.00445	Not_Sig	no	gi|555993494|ref|XP_005908508.1|	3148	0	PREDICTED: endoglin isoform X2 [Bos mutus]	gi|942093593|ref|XM_005908446.2|	2948	0	PREDICTED: Bos mutus endoglin (ENG), transcript variant X2, mRNA	P37176	885	0	Endoglin	.	.	.	.	pfam00100	Zona_pellucida	Zona pellucida-like domain.	GO:0001525|angiogenesis;GO:0007155|cell adhesion;GO:0007179|transforming growth factor beta receptor signaling pathway;	GO:0016021|integral component of membrane;	GO:0005539|glycosaminoglycan binding;GO:0034713|type I transforming	K06526	ENG, CD105; endoglin
STMP1	1404.865	-0.42485	0.114464718	-3.71162	0.0002059	0.004524	Not_Sig	no	gi|742236613|ref|XP_010830050.1|	519	3e-59	PREDICTED: uncharacterized protein C7orf73 homolog [Bison bison bison]	gi|942078191|ref|XM_005903640.2|	1636	0	PREDICTED: Bos mutus chromosome unknown open reading frame, human C7orf73 (LOC102267809), transcript variant X1, mRNA	E0CX11	95.5	3e-22	Uncharacterized protein C7orf73	.	.	.	.	pfam15054	DUF4535	Domain of unknown function (DUF4535). This family includes the uncharacterized protein C7orf73 that is found in	.	GO:0016021|integral component of membrane;GO:0031966|mitochondrial membrane;	.	.	.
FANCA	278.1085	0.572244	0.15420258	3.710987	0.0002065	0.004527	Not_Sig	no	gi|742139971|ref|XP_010842242.1|	7060	0	PREDICTED: LOW QUALITY PROTEIN: Fanconi anemia group A protein [Bison bison bison]	gi|742139970|ref|XM_010843940.1|	4315	0	PREDICTED: Bison bison bison Fanconi anemia, complementation group A (FANCA), mRNA	O15360	1791	0	Fanconi anemia group A protein	.	.	.	.	pfam03511	Fanconi_A	Fanconi anaemia group A protein.	GO:0006281|DNA repair;GO:0008585|female gonad development;GO:0036297|interstrand cross-link repair;GO:0008584|male gonad development;GO:0007140|male meiosis;GO:0006461|protein complex assembly;GO:2000348|regulation of CD40 signaling pathway;GO:0042127|regulation of cell proliferation;GO:0050727|regulation of inflammatory response;GO:0045589|regulation of regulatory T cell differentiation;GO:0051090|regulation of sequence-specific DNA binding transcription factor activity;	GO:0005737|cytoplasm;GO:0043240|Fanconi anaemia nuclear complex;GO:0005654|nucleoplasm;GO:0005634|nucleus;	.	K10888	FANCA; fanconi anemia group A protein
SMCR8	443.3895	0.458125	0.123516573	3.709018	0.0002081	0.004547	Not_Sig	no	gi|555950285|ref|XP_005887346.1|	4535	0	PREDICTED: Smith-Magenis syndrome chromosomal region candidate gene 8 protein [Bos mutus]	gi|965823774|ref|XM_012127416.2|	5486	0	PREDICTED: Ovis aries musimon Smith-Magenis syndrome chromosome region, candidate 8 (SMCR8), mRNA	Q8TEV9	1508	0	Smith-Magenis syndrome chromosomal region candidate gene 8 protein	.	.	.	.	pfam04094	DUF390	Protein of unknown function (DUF390). This is a family of long proteins currently only found in the rice genome. They have no	.	GO:0005654|nucleoplasm;	.	.	.
ZC3HAV1L	445.8738	-0.47028	0.126797395	-3.70889	0.0002082	0.004547	Not_Sig	no	gi|742182482|ref|XP_010853235.1|	1399	1e-175	PREDICTED: zinc finger CCCH-type antiviral protein 1-like [Bison bison bison]	gi|742182481|ref|XM_010854933.1|	5324	0	PREDICTED: Bison bison bison zinc finger CCCH-type, antiviral 1-like (ZC3HAV1L), mRNA	Q96H79	443	2e-140	Zinc finger CCCH-type antiviral protein 1-like	.	.	.	.	pfam11102	Cap_synth_GfcB	Group 4 capsule polysaccharide formation lipoprotein gfcB. This family includes lipoprotein GfcB (YmcC), involved in group 4	.	.	.	.	.
DHX30	959.5864	0.448545	0.120986678	3.707394	0.0002094	0.004563	Not_Sig	no	gi|529001746|ref|XP_005222983.1|	5765	0	PREDICTED: putative ATP-dependent RNA helicase DHX30 isoform X1 [Bos taurus]	gi|982962212|ref|XM_005222926.3|	3993	0	PREDICTED: Bos taurus DEAH-box helicase 30 (DHX30), transcript variant X1, mRNA	Q2NKY8	2082	0	Putative ATP-dependent RNA helicase DHX30	SPBC15C4.05	308	2.37e-85	COG1643	pfam04408	HA2	Helicase associated domain (HA2). This presumed domain is about 90 amino acid residues in length. It is found is a diverse set of RNA	GO:1902775|mitochondrial large ribosomal subunit assembly;GO:0006396|RNA processing;	GO:0005737|cytoplasm;GO:0042645|mitochondrial nucleoid;GO:0005634|nucleus;GO:0035770|ribonucleoprotein granule;	GO:0005524|ATP binding;GO:0004004|ATP-dependent RNA helicase	K13185	DHX30; ATP-dependent RNA helicase DHX30 [EC:3.6.4.13]
PPIL2	578.3576	0.519019	0.140007731	3.707071	0.0002097	0.004563	Not_Sig	no	gi|982951095|ref|XP_015331100.1|	2611	0	PREDICTED: peptidyl-prolyl cis-trans isomerase-like 2 isoform X2 [Bos taurus]	gi|942089843|ref|XM_005907341.2|	2593	0	PREDICTED: Bos mutus peptidylprolyl isomerase (cyclophilin)-like 2 (PPIL2), mRNA	Q13356	932	0	Peptidyl-prolyl cis-trans isomerase-like 2	SPAC21E11.05c	322	1.32e-99	COG0652	pfam00160	Pro_isomerase	Cyclophilin type peptidyl-prolyl cis-trans isomerase/CLD. The peptidyl-prolyl cis-trans isomerases, also known as cyclophilins, share this	GO:0050900|leukocyte migration;GO:0006457|protein folding;GO:0072659|protein localization to plasma membrane;GO:0000209|protein polyubiquitination;	GO:0005737|cytoplasm;GO:0005796|Golgi lumen;GO:0005654|nucleoplasm;GO:0005634|nucleus;GO:0005886|plasma membrane;	GO:0016874|ligase activity;GO:0003755|peptidyl-prolyl cis-trans isomerase	K10598	PPIL2, CYC4, CHP60; peptidyl-prolyl cis-trans isomerase-like 2 [EC:5.2.1.8]
HS6ST1	1088.982	0.423757	0.114352274	3.705717	0.0002108	0.004578	Not_Sig	no	gi|300795146|ref|NP_001179420.1|	1872	0	heparan-sulfate 6-O-sulfotransferase 1 [Bos taurus]	gi|594048158|ref|XM_006048419.1|	3519	0	PREDICTED: Bubalus bubalis heparan sulfate 6-O-sulfotransferase 1 (HS6ST1), mRNA	O60243	698	0	Heparan-sulfate 6-O-sulfotransferase 1	.	.	.	.	pfam03567	Sulfotransfer_2	Sulfotransferase family. This family includes a variety of sulfotransferase enzymes. Chondroitin 6-sulfotransferase	GO:0001525|angiogenesis;GO:0006024|glycosaminoglycan biosynthetic process;GO:0015015|heparan sulfate proteoglycan biosynthetic process, enzymatic modification;GO:0060716|labyrinthine layer blood vessel development;GO:0048286|lung alveolus development;GO:0048666|neuron development;	GO:0000139|Golgi membrane;GO:0005887|integral component of plasma membrane;	GO:0017095|heparan sulfate 6-O-sulfotransferase activity;GO:0008146	K02514	HS6ST1; heparan sulfate 6-O-sulfotransferase HS6ST1 [EC:2.8.2.-]
TMEM63B	3120.021	0.503009	0.135811991	3.703719	0.0002125	0.004596	Not_Sig	no	gi|594091972|ref|XP_006069351.1|	3769	0	PREDICTED: transmembrane protein 63B isoform X1 [Bubalus bubalis]	gi|982963498|ref|XM_010818208.2|	3507	0	PREDICTED: Bos taurus transmembrane protein 63B (TMEM63B), transcript variant X1, mRNA	Q5T3F8	1429	0	CSC1-like protein 2	.	.	.	.	pfam02714	DUF221	Domain of unknown function DUF221. This family consists of hypothetical transmembrane proteins none of which have any	GO:0006811|ion transport;	GO:0015629|actin cytoskeleton;GO:0016021|integral component of membrane;GO:0005886|plasma membrane;	GO:0000166|nucleotide binding;	.	.
BCR	289.5193	0.537561	0.145190058	3.702466	0.0002135	0.004596	Not_Sig	no	gi|803309451|ref|XP_012013111.1|	5973	0	PREDICTED: breakpoint cluster region protein isoform X2 [Ovis aries musimon]	gi|594084222|ref|XM_006065611.1|	3767	0	PREDICTED: Bubalus bubalis breakpoint cluster region (BCR), transcript variant X1, mRNA	Q6PAJ1	2047	0	Breakpoint cluster region protein	.	.	.	.	pfam00620	RhoGAP	RhoGAP domain. GTPase activator proteins towards Rho/Rac/Cdc42-like small GTPases.	GO:0030036|actin cytoskeleton organization;GO:0007420|brain development;GO:0042472|inner ear morphogenesis;GO:0035556|intracellular signal transduction;GO:0060313|negative regulation of blood vessel remodeling;GO:0030336|negative regulation of cell migration;GO:0002692|negative regulation of cellular extravasation;GO:0050728|negative regulation of inflammatory response;GO:0043314|negative regulation of neutrophil degranulation;GO:0050885|neuromuscular process	GO:0030054|cell junction;GO:0005829|cytosol;GO:0070062|extracellular exosome;GO:0016020|membrane;GO:0005	GO:0005524|ATP binding;GO:0005096|GTPase activator activity;GO:0004674	K08878	BCR1, BCR; breakpoint cluster region protein [EC:2.7.11.1]
EXTL3	829.0293	0.400207	0.108097513	3.702277	0.0002137	0.004596	Not_Sig	no	gi|126722849|ref|NP_001075942.1|	4749	0	exostosin-like 3 [Bos taurus]	gi|982932977|ref|XM_010807727.2|	6318	0	PREDICTED: Bos taurus exostosin-like glycosyltransferase 3 (EXTL3), transcript variant X3, mRNA	O43909	1789	0	Exostosin-like 3	.	.	.	.	pfam09258	Glyco_transf_64	Glycosyl transferase family 64 domain. Members of this family catalyse the transfer reaction of N-acetylglucosamine and N-	GO:0006024|glycosaminoglycan biosynthetic process;GO:0015012|heparan sulfate proteoglycan biosynthetic process;GO:0036498|IRE1-mediated unfolded protein response;GO:0030307|positive regulation of cell growth;	GO:0005783|endoplasmic reticulum;GO:0005789|endoplasmic reticulum membrane;GO:0016021|integral component of membrane;	GO:0001888|glucuronyl-galactosyl-proteoglycan 4-alpha-N-	K02370	EXTL3; alpha-1,4-N-acetylglucosaminyltransferase EXTL3 [EC:2.4.1.223
PTP4A1	2817.205	-0.46459	0.125487373	-3.70227	0.0002137	0.004596	Not_Sig	no	gi|329663606|ref|NP_001193053.1|	935	2e-120	protein tyrosine phosphatase type IVA 1 [Bos taurus]	gi|329663605|ref|NM_001206124.1|	1807	0	Bos taurus protein tyrosine phosphatase type IVA, member 1 (PTP4A1), mRNA	Q78EG7	360	4e-120	Protein tyrosine phosphatase type IVA 1	DR2161	57	1.08e-08	COG2453	pfam00102	Y_phosphatase	Protein-tyrosine phosphatase.	GO:0007049|cell cycle;GO:0007275|multicellular organismal development;GO:0030335|positive regulation of cell migration;	GO:0005737|cytoplasm;GO:0005769|early endosome;GO:0005783|endoplasmic reticulum;GO:0005634|nucleus;GO:0005886|plasma membrane;GO:0005819|spindle;	GO:0004725|protein tyrosine phosphatase activity;GO:0008138	K18041	PTP4A; protein tyrosine phosphatase type IVA [EC:3.1.3.48]
PHC2	1819.701	0.438824	0.118540494	3.701892	0.000214	0.004596	Not_Sig	no	gi|741917940|ref|XP_010800734.1|	3385	0	PREDICTED: polyhomeotic-like protein 2 isoform X1 [Bos taurus]	gi|982914992|ref|XM_010802432.2|	2543	0	PREDICTED: Bos taurus polyhomeotic homolog 2 (PHC2), transcript variant X1, mRNA	Q8IXK0	1128	0	Polyhomeotic-like protein 2	.	.	.	.	pfam03154	Atrophin-1	Atrophin-1 family. Atrophin-1 is the protein product of the dentatorubral-pallidoluysian atrophy (DRPLA) gene. DRPLA	GO:0007275|multicellular organismal development;GO:0016925|protein sumoylation;GO:0007283|spermatogenesis;	GO:0000792|heterochromatin;GO:0005654|nucleoplasm;GO:0005634|nucleus;GO:0031519|PcG protein complex;GO:0035102|PRC1 complex;	GO:0003677|DNA binding;GO:0008270|zinc ion binding;	K11457	PHC2, EDR2; polyhomeotic-like protein 2
C7H5orf24	539.3018	-0.53918	0.145649211	-3.70189	0.000214	0.004596	Not_Sig	no	gi|115497880|ref|NP_001069578.1|	996	2e-121	UPF0461 protein C5orf24 homolog [Bos taurus]	gi|742141996|ref|XM_010844496.1|	4832	0	PREDICTED: Bison bison bison chromosome unknown open reading frame, human C5orf24 (LOC104991849), mRNA	Q0II29	388	7e-123	UPF0461 protein C5orf24 homolog	.	.	.	.	.	.	.	.	.	.	.	.
PRDX5	1041.818	-0.46543	0.125781529	-3.7003	0.0002153	0.004616	Not_Sig	no	gi|555964155|ref|XP_005894149.1|	1058	2e-142	PREDICTED: peroxiredoxin-5, mitochondrial [Bos mutus]	gi|942050187|ref|XM_005894087.2|	963	0	PREDICTED: Bos mutus peroxiredoxin 5 (PRDX5), mRNA	Q9BGI1	410	4e-143	Peroxiredoxin-5, mitochondrial	AGc1423	119	4.36e-32	COG0678	pfam08534	Redoxin	Redoxin. This family of redoxins includes peroxiredoxin, thioredoxin and glutaredoxin proteins.	GO:0045454|cell redox homeostasis;GO:0034614|cellular response to reactive oxygen species;GO:0016480|negative regulation of transcription from RNA polymerase III promoter;	GO:0031410|cytoplasmic vesicle;GO:0005829|cytosol;GO:0070062|extracellular exosome;GO:0005615|extracellular	GO:0043027|cysteine-type endopeptidase inhibitor activity	K11187	PRDX5; peroxiredoxin 5, atypical 2-Cys peroxiredoxin
GMFB	968.3846	-0.6541	0.176905369	-3.69746	0.0002178	0.004659	DOWN	yes	gi|675690160|ref|XP_009004309.1|	842	8e-101	PREDICTED: glia maturation factor beta [Callithrix jacchus]	gi|742156725|ref|XM_010847724.1|	4174	0	PREDICTED: Bison bison bison glia maturation factor, beta (GMFB), transcript variant X2, mRNA	Q5R6P6	289	8e-89	Glia maturation factor beta	.	.	.	.	pfam00241	Cofilin_ADF	Cofilin/tropomyosin-type actin-binding protein. Severs actin filaments and binds to actin monomers.	GO:0034316|negative regulation of Arp2/3 complex-mediated actin nucleation;	GO:0005622|intracellular;	.	.	.
S100A13	264.4586	-0.57543	0.155724865	-3.6952	0.0002197	0.004683	Not_Sig	no	gi|594660717|ref|XP_007178621.1|	545	8e-67	PREDICTED: protein S100-A13 [Balaenoptera acutorostrata scammoni]	gi|742111944|ref|XM_010838409.1|	756	0	PREDICTED: Bison bison bison S100 calcium binding protein A13 (S100A13), transcript variant X1, mRNA	P79342	196	5e-62	Protein S100-A13	.	.	.	.	pfam01023	S_100	S-100/ICaBP type calcium binding domain. The S-100 domain is a subfamily of the EF-hand calcium binding proteins.	GO:0050663|cytokine secretion;GO:0050703|interleukin-1 alpha secretion;GO:0043303|mast cell degranulation;GO:0043123|positive regulation of I-kappaB kinase/NF-kappaB signaling;	GO:0005829|cytosol;GO:0070062|extracellular exosome;GO:0005615|extracellular space;GO:0042629|mast cell granule;GO:0005634|nucleus;GO:0048471|	GO:0005509|calcium ion binding;GO:0005507|copper ion	.	.
GNB4	1699.645	-0.54632	0.147862621	-3.69479	0.0002201	0.004683	Not_Sig	no	gi|149642747|ref|NP_001092503.1|	1721	0	guanine nucleotide-binding protein subunit beta-4 [Bos taurus]	gi|942045681|ref|XM_005892500.2|	4197	0	PREDICTED: Bos mutus guanine nucleotide binding protein (G protein), beta polypeptide 4 (GNB4), mRNA	Q9HAV0	665	0	Guanine nucleotide-binding protein subunit beta-4	.	.	.	.	pfam00400	WD40	WD domain, G-beta repeat.	GO:0071377|cellular response to glucagon stimulus;GO:0006457|protein folding;GO:0021762|substantia nigra development;	GO:0005829|cytosol;GO:0070062|extracellular exosome;GO:0005765|lysosomal membrane;GO:0043209|myelin sheath;GO:0005886|plasma membrane;	GO:0032403|protein complex binding;GO:0004871|signal transducer	K04538	GNB4; guanine nucleotide-binding protein subunit beta-4
HMGN3	584.7162	-0.55855	0.151174309	-3.69475	0.0002201	0.004683	Not_Sig	no	gi|594628893|ref|XP_007168032.1|	507	2e-59	PREDICTED: high mobility group nucleosome-binding domain-containing protein 3 isoform X3 [Balaenoptera acutorostrata scammoni]	gi|742183974|ref|XM_010855242.1|	994	0	PREDICTED: Bison bison bison high mobility group nucleosomal binding domain 3 (HMGN3), transcript variant X2, mRNA	Q3ZBV4	87	1e-19	High mobility group nucleosome-binding domain-containing protein 3	.	.	.	.	pfam01101	HMG14_17	HMG14 and HMG17.	GO:0016569|covalent chromatin modification;GO:0051091|positive regulation of sequence-specific DNA binding transcription factor activity;GO:0045944|positive regulation of transcription from RNA polymerase II promoter;GO:0061178|regulation of insulin secretion involved in cellular response to glucose stimulus;	GO:0000785|chromatin;GO:0005634|nucleus;	GO:0003682|chromatin binding;GO:0003677|DNA binding;	K11301	HMGN3, TRIP7; high-mobility group nucleosome-binding domain-containing
RAB18	1692.592	-0.43114	0.116730648	-3.69344	0.0002212	0.004699	Not_Sig	no	gi|466052693|ref|XP_004278696.1|	1033	1e-132	PREDICTED: ras-related protein Rab-18 [Orcinus orca]	gi|594034005|ref|XM_006041658.1|	2171	0	PREDICTED: Bubalus bubalis RAB18, member RAS oncogene family (RAB18), transcript variant X1, mRNA	Q0IIG8	402	3e-134	Ras-related protein Rab-18	.	.	.	.	pfam00071	Ras	Ras family. Includes sub-families Ras, Rab, Rac, Ral, Ran, Rap Ypt1 and more. Shares P-loop motif with GTP_EFTU, arf and	GO:0007420|brain development;GO:0071786|endoplasmic reticulum tubular network organization;GO:0001654|eye development;GO:0034389|lipid particle organization;GO:0015031|protein transport;GO:0007264|small GTPase mediated signal transduction;	GO:0071782|endoplasmic reticulum tubular network;GO:0070062|extracellular exosome;GO:0005886|plasma membrane;	GO:0019003|GDP binding;GO:0005525|GTP binding;	K07910	RAB18; Ras-related protein Rab-18
CLIC4	15688.79	-0.41049	0.111172448	-3.69241	0.0002221	0.004709	Not_Sig	no	gi|635110599|ref|XP_007978188.1|	1389	6e-175	PREDICTED: chloride intracellular channel protein 4 [Chlorocebus sabaeus]	gi|742229055|ref|XM_010830070.1|	4038	0	PREDICTED: Bison bison bison chloride intracellular channel 4 (CLIC4), mRNA	Q9XSA7	522	2e-172	Chloride intracellular channel protein 4	NMB1953	50.1	9.68e-06	COG0625	pfam13409	GST_N_2	Glutathione S-transferase, N-terminal domain. This family is closely related to pfam02798.	GO:0001525|angiogenesis;GO:0006915|apoptotic process;GO:0048754|branching morphogenesis of an epithelial tube;GO:0071277|cellular response to calcium ion;GO:1902476|chloride transmembrane transport;GO:0001886|endothelial cell morphogenesis;GO:0009566|fertilization;GO:0030216|keratinocyte differentiation;GO:0035264|multicellular organism growth;GO:0030336|negative regulation of cell migration;GO:0061299|retina vasculature morphogenesis in camera-type	GO:0045177|apical part of cell;GO:0009986|cell surface;GO:0005911|cell-cell junction;GO:0005813|centrosome;GO:0034	GO:0005254|chloride channel activity;GO:0005244|voltage-gated ion	K05024	CLIC4; chloride intracellular channel protein 4
CDCA3	254.1926	0.536906	0.145446284	3.691441	0.000223	0.004714	Not_Sig	no	gi|803055852|ref|XP_012030895.1|	1230	3e-166	PREDICTED: cell division cycle-associated protein 3 isoform X1 [Ovis aries]	gi|742143835|ref|XM_010844937.1|	1123	0	PREDICTED: Bison bison bison cell division cycle associated 3 (CDCA3), mRNA	Q99618	335	3e-112	Cell division cycle-associated protein 3	.	.	.	.	pfam00297	Ribosomal_L3	Ribosomal protein L3.	GO:0051301|cell division;GO:0007067|mitotic nuclear division;GO:0016567|protein ubiquitination;	GO:0005911|cell-cell junction;GO:0005829|cytosol;	.	.	.
ZRANB2	869.1981	-0.5241	0.141999602	-3.69083	0.0002235	0.004714	Not_Sig	no	gi|944460268|gb|KQL97483.1|	641	2e-95	zinc finger, RAN-binding domain-containing 2 [Alligator mississippiensis]	gi|742160227|ref|XM_010848747.1|	2899	0	PREDICTED: Bison bison bison zinc finger, RAN-binding domain containing 2 (ZRANB2), transcript variant X3, mRNA	Q5R580	253	2e-74	Zinc finger Ran-binding domain-containing protein 2	.	.	.	.	pfam00641	zf-RanBP	Zn-finger in Ran binding protein and others.	GO:0006397|mRNA processing;GO:0008380|RNA splicing;	GO:0005634|nucleus;	GO:0003723|RNA binding;GO:0008270|zinc ion binding;	.	.
FHOD1	419.2372	0.561028	0.152008396	3.69077	0.0002236	0.004714	Not_Sig	no	gi|803210937|ref|XP_011965892.1|	5061	0	PREDICTED: FH1/FH2 domain-containing protein 1 isoform X2 [Ovis aries musimon]	gi|330417867|ref|NM_001206339.1|	3722	0	Bos taurus formin homology 2 domain containing 1 (FHOD1), mRNA	Q9Y613	1732	0	FH1/FH2 domain-containing protein 1	.	.	.	.	pfam02181	FH2	Formin Homology 2 Domain.	GO:0051496|positive regulation of stress fiber assembly;GO:0045944|positive regulation of transcription from RNA polymerase II promoter;	GO:0032059|bleb;GO:0005737|cytoplasm;GO:0005856|cytoskeleton;GO:0016020|membrane;GO:0005634|nucleus;	GO:0042802|identical protein binding;	.	.

PXN	636.4169	0.571698	0.154990272	3.688608	0.0002255	0.004745	Not_Sig	no	gi|982950663|ref|XP_010812366.2|	4573	0	PREDICTED: paxillin [Bos taurus]	gi|982998384|ref|XM_015467168.1|	5049	0	PREDICTED: Bos taurus paxillin (PXN), mRNA	P49023	611	0	Paxillin	.	.	.	.	pfam03535	Paxillin	Paxillin family.	GO:0007155|cell adhesion;GO:0007160|cell-matrix adhesion;GO:0034614|cellular response to reactive oxygen species;GO:0007173|epidermal growth factor receptor signaling pathway;GO:0060396|growth hormone receptor signaling pathway;GO:0006936|muscle contraction;GO:0007172|signal complex assembly;GO:0007165|signal transduction;GO:0007179|transforming growth factor beta receptor signaling pathway;GO:0048010|vascular endothelial growth factor receptor signaling pathway;	GO:0005938|cell cortex;GO:0005737|cytoplasm;GO:0005829|cytosol;GO:0005925|focal adhesion;GO:0030027|lamellipodium;GO:00	GO:0008013|beta-catenin binding;GO:0017166|vinculin	K05760	PXN; paxillin
UBR4	3538.272	0.401824	0.108962635	3.68772	0.0002263	0.004753	Not_Sig	no	gi|528940927|ref|XP_005203194.1|	25308	0	PREDICTED: E3 ubiquitin-protein ligase UBR4 isoform X1 [Bos taurus]	gi|965870637|ref|XM_012148381.2|	14130	0	PREDICTED: Ovis aries musimon ubiquitin protein ligase E3 component n-recognin 4 (UBR4), transcript variant X2, mRNA	Q5T4S7	9486	0	E3 ubiquitin-protein ligase UBR4	.	.	.	.	pfam13764	E3_UbLigase_R4	E3 ubiquitin-protein ligase UBR4. This is a family of E## ubiquitin ligase enzymes.	GO:0042787|protein ubiquitination involved in ubiquitin-dependent protein catabolic process;GO:0016032|viral process;	GO:0005813|centrosome;GO:0005737|cytoplasm;GO:0016021|integral component of membrane;GO:0016020|membrane;GO:0005654|nucleoplasm;	GO:0016874|ligase activity;GO:0004842|ubiquitin-protein transferase	K10691	UBR4, ZUBR1; E3 ubiquitin-protein ligase UBR4 [EC:2.3.2.27]
MYLK	11504.12	-0.35146	0.095492653	-3.68049	0.0002328	0.004881	Not_Sig	no	gi|594111589|ref|XP_006078611.1|	10338	0	PREDICTED: myosin light chain kinase, smooth muscle isoform X1 [Bubalus bubalis]	gi|942048343|ref|XM_005893405.2|	5611	0	PREDICTED: Bos mutus myosin light chain kinase (MYLK), transcript variant X8, mRNA	Q28824	2184	0	Myosin light chain kinase, smooth muscle	.	.	.	.	pfam00069	Pkinase	Protein kinase domain.	GO:0030335|positive regulation of cell migration;GO:0006939|smooth muscle contraction;	GO:0032154|cleavage furrow;GO:0005737|cytoplasm;GO:0005856|cytoskeleton;GO:0030027|lamellipodium;	GO:0005524|ATP binding;GO:0046872|metal ion binding;GO:0004687	K00907	MYLK; myosin-light-chain kinase [EC:2.7.11.18]
TUBB4B	3264.266	0.355339	0.096590251	3.678824	0.0002343	0.004904	Not_Sig	no	gi|634865305|ref|XP_007945164.1|	2220	0	PREDICTED: tubulin beta-4B chain isoform X1 [Orycteropus afer afer]	gi|402693019|ref|NM_001034663.2|	1539	0	Bos taurus tubulin beta 4B class IVb (TUBB4B), mRNA	P68372	858	0	Tubulin beta-4B chain	ECU03g0820i	732	0	COG5023	pfam03953	Tubulin_C	Tubulin C-terminal domain. This family includes the tubulin alpha, beta and gamma chains. Members of this family are involved in	GO:0007017|microtubule-based process;	GO:0005737|cytoplasm;GO:0070062|extracellular exosome;GO:0031012|extracellular matrix;GO:1903561|extracellular vesicle;GO:0005874|microtubule;GO:00432	GO:0003725|double-stranded RNA binding;GO:0005525|GTP	K07375	TUBB; tubulin beta
MIB1	1969.334	-0.4531	0.123194192	-3.67797	0.0002351	0.004911	Not_Sig	no	gi|926722623|ref|XP_005697112.2|	5298	0	PREDICTED: E3 ubiquitin-protein ligase MIB1 [Capra hircus]	gi|742224406|ref|XM_010828915.1|	8172	0	PREDICTED: Bison bison bison mindbomb E3 ubiquitin protein ligase 1 (MIB1), mRNA	Q86YT6	2037	0	E3 ubiquitin-protein ligase MIB1	all2748	123	1.48e-28	COG0666	pfam06701	MIB_HERC2	Mib_herc2. Named mib/herc2 domain in. Usually the protein also contains an E3 ligase domain (either Ring or Hect).	GO:0001568|blood vessel development;GO:0006897|endocytosis;GO:0001947|heart looping;GO:0001701|in utero embryonic development;GO:0045665|negative regulation of neuron differentiation;GO:0001841|neural tube formation;GO:0007219|Notch signaling pathway;GO:0045807|positive regulation of endocytosis;GO:0001756|somitogenesis;	GO:0005813|centrosome;GO:0005737|cytoplasm;GO:0031410|cytoplasmic vesicle;GO:0005829|cytosol;GO:0005886|plasma membrane;GO:0014069|postsynaptic	GO:0016874|ligase activity;GO:0004842|ubiquitin-protein transferase	K10645	MIB; E3 ubiquitin-protein ligase mind-bomb [EC:2.3.2.27]
SELENOF	1544.013	-0.50549	0.137466507	-3.67716	0.0002358	0.004918	Not_Sig	no	gi|955543439|ref|XP_004442970.2|	796	1e-100	PREDICTED: 15 kDa selenoprotein [Ceratotherium simum simum]	gi|942034448|ref|XM_005888423.2|	1417	0	PREDICTED: Bos mutus 15 kDa selenoprotein (LOC102269598), transcript variant X1, mRNA	A8YXY3	289	2e-94	15 kDa selenoprotein	.	.	.	.	pfam08806	Sep15_SelM	Sep15/SelM redox domain. Sep15 and SelM are eukaryotic selenoproteins that have a thioredoxin-like domain and a	.	GO:0005788|endoplasmic reticulum lumen;	.	.	.
PIK3C2B	462.7464	0.489777	0.13326204	3.675296	0.0002376	0.004945	Not_Sig	no	gi|555972895|ref|XP_005898448.1|	7661	0	PREDICTED: phosphatidylinositol 4-phosphate 3-kinase C2 domain-containing subunit beta [Bos mutus]	gi|982995993|ref|XM_015466687.1|	3443	0	PREDICTED: Bos taurus phosphatidylinositol-4-phosphate 3-kinase catalytic subunit type 2 beta (PIK3C2B), mRNA	O00750	2792	0	Phosphatidylinositol 4-phosphate 3-kinase C2 domain-containing subunit beta	SPAC458.05	190	1.34e-48	COG5032	pfam00454	PI3_PI4_kinase	Phosphatidylinositol 3- and 4-kinase. Some members of this family probably do not have lipid kinase activity and are protein	GO:0009267|cellular response to starvation;GO:0006661|phosphatidylinositol biosynthetic process;GO:0048015|phosphatidylinositol-mediated signaling;GO:0043491|protein kinase B signaling;	GO:0005829|cytosol;GO:0030139|endocytic vesicle;GO:0005783|endoplasmic reticulum;GO:0005634|nucleus;GO:0005942|phosphatidylinositol 3-kinase	GO:0016303|1-phosphatidylinositol-3-kinase activity;GO:0035005	K00923	PIK3C2; phosphatidylinositol-4-phosphate 3-kinase [EC:2.7.1.154]
FNIP1	949.2885	-0.47716	0.129884291	-3.67376	0.000239	0.004966	Not_Sig	no	gi|742217368|ref|XP_010861872.1|	6086	0	PREDICTED: folliculin-interacting protein 1 isoform X1 [Bison bison bison]	gi|942034320|ref|XM_005888378.2|	6226	0	PREDICTED: Bos mutus folliculin interacting protein 1 (FNIP1), transcript variant X1, mRNA	Q8TF40	2201	0	Folliculin-interacting protein 1	.	.	.	.	pfam14637	FNIP_M	Folliculin-interacting protein middle domain. This is the middle domain of folliculin-interacting proteins.	GO:0009267|cellular response to starvation;GO:0002327|immature B cell differentiation;GO:0043154|negative regulation of cysteine-type endopeptidase activity involved in apoptotic process;GO:0032007|negative regulation of TOR signaling;GO:0000122|negative regulation of transcription from RNA polymerase II promoter;GO:0002904|positive regulation of B cell apoptotic process;GO:0033138|positive regulation of peptidyl-serine phosphorylation;GO:0031334|positive regulation of protein complex assembly;GO:0001934|positive regulation of protein	GO:0005737|cytoplasm;	GO:0005085|guanyl-nucleotide exchange factor activity;	.	.
APOE	5095.247	-0.41994	0.114341357	-3.67269	0.00024	0.004975	Not_Sig	no	gi|942068213|ref|XP_014335131.1|	1605	0	PREDICTED: apolipoprotein E isoform X1 [Bos mutus]	gi|942068212|ref|XM_014479645.1|	1322	0	PREDICTED: Bos mutus apolipoprotein E (APOE), transcript variant X1, mRNA	P0DN41	554	0	Apolipoprotein E	.	.	.	.	pfam01442	Apolipoprotein	Apolipoprotein A1/A4/E domain. These proteins contain several 22 residue repeats which form a pair of alpha helices. This family	GO:0006869|lipid transport;GO:0042157|lipoprotein metabolic process;	GO:0042627|chylomicron;GO:0034364|high-density lipoprotein particle;GO:0034361|very-low-density lipoprotein particle;	GO:0008201|heparin binding;GO:0008289|lipid binding;	.	.
SELENON	3027.06	0.516101	0.140536365	3.672365	0.0002403	0.004975	Not_Sig	no	gi|169234651|ref|NP_001108448.1|	2501	0	selenoprotein N [Bos taurus]	gi|933121554|ref|NM_001114976.2|	3868	0	Bos taurus selenoprotein N, 1 (SEPN1), mRNA	Q9NZV5	878	0	Selenoprotein N	.	.	.	.	pfam01328	Peroxidase_2	Peroxidase, family 2. The peroxidases in this family do not have similarity to other peroxidases.	GO:0055074|calcium ion homeostasis;GO:0071313|cellular response to caffeine;GO:0034599|cellular response to oxidative stress;GO:0048286|lung alveolus development;GO:0007005|mitochondrion organization;GO:0033555|multicellular organismal response to stress;GO:1902884|positive regulation of response to oxidative stress;GO:0014858|positive regulation of skeletal muscle cell proliferation;GO:0060314|regulation of ryanodine-sensitive calcium-release channel activity;GO:0003016|respiratory system	GO:0005789|endoplasmic reticulum membrane;	GO:0005509|calcium ion binding;GO:0016491|oxidoreductase	.	.
FAT1	10034.3	0.37239	0.101455507	3.670477	0.0002421	0.005003	Not_Sig	no	gi|555974151|ref|XP_005899070.1|	23778	0	PREDICTED: protocadherin Fat 1 [Bos mutus]	gi|982969151|ref|XM_010820249.2|	14725	0	PREDICTED: Bos taurus FAT atypical cadherin 1 (FAT1), transcript variant X1, mRNA	Q14517	8346	0	Protocadherin Fat 1	.	.	.	.	pfam02210	Laminin_G_2	Laminin G domain. This family includes the Thrombospondin N-terminal-like domain, a Laminin G subfamily.	GO:0007015|actin filament organization;GO:0009653|anatomical structure morphogenesis;GO:0007155|cell adhesion;GO:0016477|cell migration;GO:0007267|cell-cell signaling;GO:0007163|establishment or maintenance of cell polarity;GO:0007156|homophilic cell adhesion via plasma membrane adhesion molecules;GO:0016337|single organismal cell-cell adhesion;	GO:0005911|cell-cell junction;GO:0070062|extracellular exosome;GO:0030175|filopodium;GO:0005925|focal adhesion;GO:0005887|integral	GO:0005509|calcium ion binding;	K16506	FAT1_2_3; protocadherin Fat 1/2/3
SCARF2	1720.375	0.602297	0.164141169	3.669384	0.0002431	0.005015	UP	yes	gi|528989856|ref|XP_005218360.1|	2973	0	PREDICTED: scavenger receptor class F member 2 isoform X1 [Bos taurus]	gi|982998777|ref|XM_005195168.3|	3270	0	PREDICTED: Bos taurus scavenger receptor class F member 2 (SCARF2), transcript variant X1, mRNA	P59222	1011	0	Scavenger receptor class F member 2	.	.	.	.	pfam03999	MAP65_ASE1	Microtubule associated protein (MAP65/ASE1 family).	GO:0007157|heterophilic cell-cell adhesion via plasma membrane cell adhesion molecules;	GO:0005925|focal adhesion;GO:0016021|integral component of membrane;	GO:0005044|scavenger receptor activity;	.	.
COPG1	3140.524	0.368106	0.100358278	3.66792	0.0002445	0.005035	Not_Sig	no	gi|83715992|ref|NP_001032904.1|	4413	0	coatomer subunit gamma-1 [Bos taurus]	gi|1066164|emb|X92987.1|	2937	0	B.primigenius mRNA for coat protein gamma-cop	P53620	1700	0	Coatomer subunit gamma-1	SPAC57A7.10c	566	0	COG5240	pfam01602	Adaptin_N	Adaptin N terminal region. This family consists of the N terminal region of various alpha, beta and gamma subunits of the AP-1, AP-2	GO:0006891|intra-Golgi vesicle-mediated transport;GO:0006886|intracellular protein transport;GO:0006890|retrograde vesicle-mediated transport, Golgi to ER;	GO:0030126|COPI vesicle coat;GO:0005829|cytosol;GO:0000139|Golgi membrane;	GO:0005198|structural molecule activity;	K17267	COPG; coatomer protein complex, subunit gamma
TP53INP1	2275.899	-0.41784	0.113953893	-3.66672	0.0002457	0.00505	Not_Sig	no	gi|555967103|ref|XP_005895600.1|	1186	4e-146	PREDICTED: tumor protein p53-inducible nuclear protein 1 isoform X1 [Bos mutus]	gi|982945167|ref|XM_005215685.3|	5553	0	PREDICTED: Bos taurus tumor protein p53 inducible nuclear protein 1 (TP53INP1), transcript variant X2, mRNA	Q96A56	423	5e-134	Tumor protein p53-inducible nuclear protein 1	.	.	.	.	pfam14839	DOR	DOR family. This family of proteins regulate autophagy and gene transcription.	GO:0006915|apoptotic process;GO:0048102|autophagic cell death;GO:0000045|autophagosome assembly;GO:0007050|cell cycle arrest;GO:0071361|cellular response to ethanol;GO:0071447|cellular response to hydroperoxide;GO:0072703|cellular response to methyl methanesulfonate;GO:0034644|cellular response to UV;GO:0030336|negative regulation of cell migration;GO:0008285|negative regulation of cell proliferation;GO:2001235|positive regulation of apoptotic signaling pathway;GO:0010508|positive regulation of	GO:0005776|autophagosome;GO:0005737|cytoplasm;GO:0031410|cytoplasmic vesicle;GO:0005829|cytosol;GO:0005654|nucleoplasm;GO:0005634|nucleus;GO:00166	GO:0016209|antioxidant activity;	K15310	TP53INP1; tumor protein p53-inducible nuclear protein 1
GAA	877.4767	0.498713	0.136068761	3.665154	0.0002472	0.005068	Not_Sig	no	gi|555954830|ref|XP_005889581.1|	4842	0	PREDICTED: LOW QUALITY PROTEIN: lysosomal alpha-glucosidase [Bos mutus]	gi|742187779|ref|XM_010856284.1|	3960	0	PREDICTED: Bison bison bison glucosidase, alpha; acid (GAA), transcript variant X2, mRNA	Q9MYM4	1855	0	Lysosomal alpha-glucosidase	alr4773	339	1.31e-99	COG1501	pfam01055	Glyco_hydro_31	Glycosyl hydrolases family 31. Glycosyl hydrolases are key enzymes of carbohydrate metabolizm. Family 31 comprises	GO:0060048|cardiac muscle contraction;GO:0002086|diaphragm contraction;GO:0005980|glycogen catabolic process;GO:0003007|heart morphogenesis;GO:0007626|locomotory behavior;GO:0007040|lysosome organization;GO:0046716|muscle cell cellular homeostasis;GO:0050885|neuromuscular process controlling balance;GO:0050884|neuromuscular process controlling posture;GO:0002026|regulation of the force of heart contraction;GO:0009888|tissue development;GO:0043181|vacuolar sequestering;	GO:0070062|extracellular exosome;GO:0005765|lysosomal membrane;	GO:0004558|alpha-1,4-glucosidase activity;GO:0030246|carbohydrate	K12316	GAA; lysosomal alpha-glucosidase [EC:3.2.1.20]
INTS1	960.8552	0.489109	0.133474342	3.664445	0.0002479	0.005068	Not_Sig	no	gi|300795201|ref|NP_001178399.1|	10341	0	integrator complex subunit 1 [Bos taurus]	gi|982967479|ref|XM_005225250.3|	6904	0	PREDICTED: Bos taurus integrator complex subunit 1 (INTS1), transcript variant X1, mRNA	Q6P4S8	3591	0	Integrator complex subunit 1	.	.	.	.	pfam12432	DUF3677	Protein of unknown function (DUF3677). This domain family is found in eukaryotes, and is approximately 80 amino acids in	GO:0001832|blastocyst growth;GO:0001833|inner cell mass cell proliferation;GO:0043066|negative regulation of apoptotic process;GO:0043154|negative regulation of cysteine-type endopeptidase activity involved in apoptotic process;GO:0016180|snRNA processing;GO:0034474|U2 snRNA 3'-end processing;	GO:0016021|integral component of membrane;GO:0032039|integrator complex;GO:0016020|membrane;GO:0031965|nuclear	.	K13138	INTS1; integrator complex subunit 1
SFT2D1	382.8431	-0.55768	0.152204857	-3.66398	0.0002483	0.005068	Not_Sig	no	gi|77736049|ref|NP_001029723.1|	782	7e-101	vesicle transport protein SFT2A [Bos taurus]	gi|402691812|ref|NM_001034551.2|	1035	0	Bos taurus SFT2 domain containing 1 (SFT2D1), mRNA	Q8WV19	293	2e-97	Vesicle transport protein SFT2A	.	.	.	.	pfam04178	Got1	Got1/Sft2-like family. Traffic through the yeast Golgi complex depends on a member of the syntaxin family of SNARE proteins,	GO:0015031|protein transport;GO:0016192|vesicle-mediated transport;	GO:0016021|integral component of membrane;GO:0016020|membrane;	.	.	.
TONSL	256.6709	0.562227	0.15344676	3.663985	0.0002483	0.005068	Not_Sig	no	gi|942059913|ref|XP_014334328.1|	6132	0	PREDICTED: LOW QUALITY PROTEIN: tonsoku-like protein [Bos mutus]	gi|942059912|ref|XM_014478842.1|	4182	0	PREDICTED: Bos mutus tonsoku-like, DNA repair protein (TONSL), mRNA	Q0P5G1	2348	0	Tonsoku-like protein	.	.	.	.	pfam12796	Ank_2	Ankyrin repeats (3 copies).	GO:0000724|double-strand break repair via homologous recombination;GO:0031297|replication fork processing;	GO:0005737|cytoplasm;GO:0005662|DNA replication factor A complex;GO:0035101|FACT complex;GO:0042555|MCM	GO:0042393|histone binding;	K09257	NFKBIL2; NF-kappa-B inhibitor-like protein 2
CFL1	11484.39	0.443057	0.12095476	3.662998	0.0002493	0.005078	Not_Sig	no	gi|585176964|ref|XP_006740222.1|	890	1e-115	PREDICTED: cofilin-1 isoform X2 [Leptonychotes weddellii]	gi|555964054|ref|XM_005894037.1|	1199	0	PREDICTED: Bos mutus cofilin 1 (non-muscle) (CFL1), transcript variant X1, mRNA	Q6B7M7	331	1e-111	Cofilin-1	.	.	.	.	pfam00241	Cofilin_ADF	Cofilin/tropomyosin-type actin-binding protein. Severs actin filaments and binds to actin monomers.	GO:0030042|actin filament depolymerization;GO:0007010|cytoskeleton organization;GO:0022604|regulation of cell morphogenesis;	GO:0015629|actin cytoskeleton;GO:0005737|cytoplasm;GO:0031258|lamellipodium membrane;GO:0016363|nuclear	GO:0051015|actin filament binding;	K05765	CFL; cofilin
CIT	431.9492	0.482617	0.131818005	3.661241	0.000251	0.005104	Not_Sig	no	gi|803172860|ref|XP_011953200.1|	10281	0	PREDICTED: citron Rho-interacting kinase isoform X1 [Ovis aries]	gi|742130725|ref|XM_010841399.1|	6595	0	PREDICTED: Bison bison bison citron rho-interacting serine/threonine kinase (CIT), transcript variant X2, mRNA	O14578	3734	0	Citron Rho-interacting kinase	.	.	.	.	pfam00780	CNH	CNH domain. Domain found in NIK1-like kinase, mouse citron and yeast ROM1, ROM2. Unpublished observations.	GO:0000910|cytokinesis;GO:0000086|G2/M transition of mitotic cell cycle;GO:0048699|generation of neurons;GO:0007030|Golgi organization;GO:0035556|intracellular signal transduction;GO:0007067|mitotic nuclear division;GO:0032467|positive regulation of cytokinesis;GO:0008064|regulation of actin polymerization or depolymerization;	GO:0005829|cytosol;GO:0031985|Golgi cisterna;GO:0016020|membrane;GO:0043025|neuronal cell body;GO:0005886|plasma membrane;	GO:0005524|ATP binding;GO:0046872|metal ion binding;GO:0030165	K16308	CIT; citron Rho-interacting kinase [EC:2.7.11.1]
GATA4	730.3906	0.557478	0.152297864	3.660443	0.0002518	0.005111	Not_Sig	no	gi|803278437|ref|XP_011998893.1|	1477	0	PREDICTED: transcription factor GATA-4 isoform X1 [Ovis aries musimon]	gi|982932903|ref|XM_005209824.3|	3356	0	PREDICTED: Bos taurus GATA binding protein 4 (GATA4), transcript variant X1, mRNA	Q0Q0E4	565	0	Transcription factor GATA-4	SPCC1393.08	116	6.19e-26	COG5641	pfam05349	GATA-N	GATA-type transcription activator, N-terminal. GATA transcription factors mediate cell differentiation in a diverse range of tissues.	GO:0048646|anatomical structure formation involved in morphogenesis;GO:0009887|organ morphogenesis;GO:0003290|atrial septum secundum morphogenesis;GO:0048468|cell development;GO:0045165|cell fate commitment;GO:0048565|digestive tract development;GO:0003197|endocardial cushion development;GO:0007507|heart development;GO:0060575|intestinal epithelial cell differentiation;GO:0008584|male gonad development;GO:0045893|positive regulation of transcription, DNA-	GO:0005634|nucleus;GO:0090575|RNA polymerase II transcription factor complex;GO:0005667|transcription factor complex;	GO:0003682|chromatin binding;GO:0003677|DNA	K09183	GATA4; GATA-binding protein 4
CAMK2G	282.1414	0.499495	0.136525937	3.658608	0.0002536	0.005139	Not_Sig	no	gi|803228147|ref|XP_011974356.1|	2929	0	PREDICTED: calcium/calmodulin-dependent protein kinase type II subunit gamma isoform X6 [Ovis aries musimon]	gi|965806543|ref|XM_012118966.2|	3776	0	PREDICTED: Ovis aries musimon calcium/calmodulin-dependent protein kinase II gamma (CAMK2G), transcript variant X6, mRNA	Q13555	1092	0	Calcium/calmodulin-dependent protein kinase type II subunit gamma	.	.	.	.	pfam00069	Pkinase	Protein kinase domain.	GO:0006816|calcium ion transport;GO:0030154|cell differentiation;GO:0000082|G1/S transition of mitotic cell cycle;GO:0030073|insulin secretion;GO:0060333|interferon-gamma-mediated signaling pathway;GO:0000165|MAPK cascade;GO:0007399|nervous system development;GO:0046777|protein autophosphorylation;GO:0051259|protein oligomerization;GO:0051924|regulation of calcium ion transport;GO:1900034|regulation of cellular response to heat;GO:1901897|regulation of relaxation of cardiac muscle;GO:0014733|regulation	GO:0005829|cytosol;GO:0030666|endocytic vesicle membrane;GO:0016020|membrane;GO:0005654|nucleoplasm;GO:0005886|plasma	GO:0005524|ATP binding;GO:0004723|calcium-dependent protein	K04515	CAMK2; calcium/calmodulin-dependent protein kinase (CaM kinase)
MARK4	440.6672	0.61916	0.169286344	3.657474	0.0002547	0.005147	UP	yes	gi|926714841|ref|XP_013826905.1|	3350	0	PREDICTED: MAP/microtubule affinity-regulating kinase 4 [Capra hircus]	gi|466015920|ref|XM_004271171.1|	2742	0	PREDICTED: Orcinus orca MAP/microtubule affinity-regulating kinase 4 (MARK4), transcript variant X1, mRNA	Q96L34	1291	0	MAP/microtubule affinity-regulating kinase 4	.	.	.	.	pfam00069	Pkinase	Protein kinase domain.	GO:0051301|cell division;GO:0030030|cell projection organization;GO:0030010|establishment of cell polarity;GO:0001578|microtubule bundle formation;GO:0000226|microtubule cytoskeleton organization;GO:0007067|mitotic nuclear division;GO:0007399|nervous system development;GO:0043068|positive regulation of programmed cell death;GO:0006468|protein phosphorylation;	GO:0005813|centrosome;GO:0036064|ciliary basal body;GO:0005829|cytosol;GO:0015630|microtubule	GO:0005524|ATP binding;GO:0043015|gamma-tubulin binding;GO:0008017	K08798	MARK; MAP/microtubule affinity-regulating kinase [EC:2.7.11.1]
CABIN1	744.1871	0.521804	0.142675358	3.657284	0.0002549	0.005147	Not_Sig	no	gi|296478352|tpg|DAA20467.1|	9354	0	TPA: calcineurin binding protein 1 [Bos taurus]	gi|982998750|ref|XM_015467241.1|	6736	0	PREDICTED: Bos taurus calcineurin binding protein 1 (CABIN1), transcript variant X1, mRNA	Q9Y6J0	3077	0	Calcineurin-binding protein cabin-1	.	.	.	.	pfam09047	MEF2_binding	MEF2 binding. The myocyte enhancer factor-2 (MEF2) binding domain, predominantly found in the calcineurin-binding protein	GO:0007166|cell surface receptor signaling pathway;GO:0016569|covalent chromatin modification;GO:0006336|DNA replication-independent nucleosome assembly;	GO:0016235|aggresome;GO:0005737|cytoplasm;GO:0005654|nucleoplasm;GO:0005634|nucleus;	GO:0004864|protein phosphatase inhibitor activity;	K17613	CABIN1; calcineurin-binding protein cabin-1
E2F4	548.7214	0.541996	0.148237369	3.656271	0.0002559	0.005159	Not_Sig	no	gi|741958280|ref|XP_005218671.2|	1654	0	PREDICTED: transcription factor E2F4 isoform X1 [Bos taurus]	gi|982951686|ref|XM_005218614.3|	2071	0	PREDICTED: Bos taurus E2F transcription factor 4 (E2F4), transcript variant X1, mRNA	Q16254	544	0	Transcription factor E2F4	.	.	.	.	pfam02319	E2F_TDP	E2F/DP family winged-helix DNA-binding domain. This family contains the transcription factor E2F and its dimerization partners	GO:0009887|organ morphogenesis;GO:0008015|blood circulation;GO:0006884|cell volume homeostasis;GO:0098534|centriole assembly;GO:0006977|DNA damage response, signal transduction by p53 class mediator resulting in cell cycle arrest;GO:0002064|epithelial cell development;GO:0044458|motile cilium assembly;GO:1903251|multi-ciliated epithelial cell differentiation;GO:0045944|positive regulation of transcription from RNA polymerase II promoter;GO:0042127|regulation of cell proliferation;GO:0000083|regulation of	GO:0000790|nuclear chromatin;GO:0005654|nucleoplasm;GO:0005667|transcription factor complex;	GO:0003677|DNA binding;GO:1990841|promoter-specific chromatin	K04682	E2F4_5; transcription factor E2F4/5
LOC102267002	1925.609	0.488839	0.133763563	3.654498	0.0002577	0.005183	Not_Sig	no	gi|965822881|ref|XP_014961591.1|	4441	0	PREDICTED: protocadherin gamma-B7 isoform X25 [Ovis aries musimon]	gi|965822880|ref|XM_015106105.1|	4337	0	PREDICTED: Ovis aries musimon protocadherin gamma-C4 (LOC101105495), transcript variant X27, mRNA	Q5DRA5	1500	0	Protocadherin gamma-B7	.	.	.	.	pfam08266	Cadherin_2	Cadherin-like. This cadherin domain is usually the most N-terminal copy of the domain.	GO:0007156|homophilic cell adhesion via plasma membrane adhesion molecules;	GO:0016021|integral component of membrane;GO:0005886|plasma membrane;	GO:0005509|calcium ion binding;	K16496	PCDHGB; protocadherin gamma subfamily B
CAPZA2	1471.555	-0.44583	0.122007254	-3.65414	0.000258	0.005183	Not_Sig	no	gi|426227627|ref|XP_004007919.1|	1497	0	PREDICTED: F-actin-capping protein subunit alpha-2 isoform X1 [Ovis aries]	gi|942064445|ref|XM_005899133.2|	2241	0	PREDICTED: Bos mutus capping protein (actin filament) muscle Z-line, alpha 2 (CAPZA2), mRNA	Q09YJ9	563	0	F-actin-capping protein subunit alpha-2	.	.	.	.	pfam01267	F-actin_cap_A	F-actin capping protein alpha subunit.	GO:0051016|barbed-end actin filament capping;	GO:0008290|F-actin capping protein complex;	.	.	.
SERP1	1745.355	-0.49975	0.136793943	-3.65328	0.0002589	0.005192	Not_Sig	no	gi|440898216|gb|ELR49760.1|	289	9e-51	Stress-associated endoplasmic reticulum protein 1, partial [Bos mutus]	gi|942081784|ref|XM_005904798.2|	3103	0	PREDICTED: Bos mutus stress-associated endoplasmic reticulum protein 1 (SERP1), mRNA	Q9R2C1	129	2e-33	Stress-associated endoplasmic reticulum protein 1	.	.	.	.	pfam06624	RAMP4	Ribosome associated membrane protein RAMP4. This family consists of several ribosome associated membrane protein	GO:0030968|endoplasmic reticulum unfolded protein response;GO:0006006|glucose metabolic process;GO:0010259|multicellular organismal aging;GO:0048644|muscle organ morphogenesis;GO:0060124|positive regulation of growth hormone secretion;GO:0032024|positive regulation of insulin secretion;GO:0046622|positive regulation of organ growth;GO:0045727|positive regulation of translation;GO:0009791|post-embryonic development;GO:0006486|protein glycosylation;GO:0015031|protein	GO:0005881|cytoplasmic microtubule;GO:0005783|endoplasmic reticulum;GO:0005789|endoplasmic reticulum membrane;GO:0016021|integral	.	.	.
RAB8A	1122.201	0.451944	0.123772962	3.651393	0.0002608	0.005221	Not_Sig	no	gi|664772107|ref|XP_008505261.1|	1145	8e-151	PREDICTED: ras-related protein Rab-8A [Equus przewalskii]	gi|942071496|ref|XM_005901468.2|	1847	0	PREDICTED: Bos mutus RAB8A, member RAS oncogene family (RAB8A), mRNA	A4FV54	426	2e-145	Ras-related protein Rab-8A	.	.	.	.	pfam00071	Ras	Ras family. Includes sub-families Ras, Rab, Rac, Ral, Ran, Rap Ypt1 and more. Shares P-loop motif with GTP_EFTU, arf and	GO:0007409|axonogenesis;GO:0032869|cellular response to insulin stimulus;GO:0042384|cilium assembly;GO:0048210|Golgi vesicle fusion to target membrane;GO:0072659|protein localization to plasma membrane;GO:0009306|protein secretion;GO:0007264|small GTPase mediated signal transduction;GO:0006904|vesicle docking involved in exocytosis;	GO:0005814|centriole;GO:0036064|ciliary basal body;GO:0097546|ciliary base;GO:0005768|endosome;GO:0070062|extracellular	GO:0019003|GDP binding;GO:0005525|GTP binding;GO:0003924	K07901	RAB8A, MEL; Ras-related protein Rab-8A
RHOBTB3	2749.234	-0.37696	0.103277195	-3.64995	0.0002623	0.005241	Not_Sig	no	gi|926698723|ref|XP_013821250.1|	3242	0	PREDICTED: rho-related BTB domain-containing protein 3 [Capra hircus]	gi|982985779|ref|XM_015464384.1|	4942	0	PREDICTED: Bos taurus Rho-related BTB domain containing 3 (RHOBTB3), transcript variant X1, mRNA	O94955	1214	0	Rho-related BTB domain-containing protein 3	.	.	.	.	pfam00651	BTB	BTB/POZ domain. The BTB (for BR-C, ttk and bab) or POZ (for Pox virus and Zinc finger) domain is present near the N-terminus of a	GO:0042147|retrograde transport, endosome to Golgi;GO:0007264|small GTPase mediated signal transduction;	GO:0005829|cytosol;GO:0070062|extracellular exosome;GO:0032588|trans-Golgi network membrane;	GO:0005524|ATP binding;GO:0016887|ATPase activity;GO:0017137	K07869	RHOBTB3; Rho-related BTB domain-containing protein 3
LIN7C	1092.824	-0.58492	0.16032479	-3.64834	0.0002639	0.005265	Not_Sig	no	gi|431915664|gb|ELK15997.1|	965	4e-116	Lin-7 like protein C [Pteropus alecto]	gi|942040942|ref|XM_005890666.2|	3816	0	PREDICTED: Bos mutus lin-7 homolog C (C. elegans) (LIN7C), mRNA	Q792I0	362	5e-114	Protein lin-7 homolog C	.	.	.	.	pfam00595	PDZ	PDZ domain (Also known as DHR or GLGF). PDZ domains are found in diverse signaling proteins.	GO:0006887|exocytosis;GO:0045199|maintenance of epithelial cell apical/basal polarity;GO:0002011|morphogenesis of an epithelial sheet;GO:0007269|neurotransmitter secretion;GO:1903361|protein localization to basolateral plasma membrane;GO:0015031|protein transport;	GO:0016323|basolateral plasma membrane;GO:0005923|bicellular tight junction;GO:0005911|cell-cell junction;GO:0005737|cytoplasm;GO:007006	GO:0030165|PDZ domain binding;	.	.
MLXIP	1305.04	0.436779	0.119738844	3.647763	0.0002645	0.005266	Not_Sig	no	gi|803210464|ref|XP_011965654.1|	4145	0	PREDICTED: MLX-interacting protein isoform X2 [Ovis aries musimon]	gi|965789209|ref|XM_012110264.2|	5782	0	PREDICTED: Ovis aries musimon MLX interacting protein (MLXIP), transcript variant X2, mRNA	Q2VPU4	1293	0	MLX-interacting protein	.	.	.	.	pfam00010	HLH	Helix-loop-helix DNA-binding domain.	GO:0006913|nucleocytoplasmic transport;GO:1900402|regulation of carbohydrate metabolic process by regulation of transcription from RNA polymerase II promoter;GO:0006357|regulation of transcription from RNA polymerase II promoter;GO:0006351|transcription, DNA-templated;	GO:0005737|cytoplasm;GO:0005741|mitochondrial outer membrane;GO:0005634|nucleus;	GO:0003677|DNA binding;GO:0003700|transcription factor activity, sequence-	K09113	MLX; MAX-like protein X
ACAA2	1398.834	-0.38742	0.106228267	-3.64706	0.0002653	0.005266	Not_Sig	no	gi|78369436|ref|NP_001030419.1|	2088	0	3-ketoacyl-CoA thiolase, mitochondrial [Bos taurus]	gi|742164079|ref|XM_010849817.1|	1589	0	PREDICTED: Bison bison bison acetyl-CoA acyltransferase 2 (ACAA2), mRNA	Q3T0R7	808	0	3-ketoacyl-CoA thiolase, mitochondrial	BH1997	403	2.56e-135	COG0183	pfam00108	Thiolase_N	Thiolase, N-terminal domain. Thiolase is reported to be structurally related to beta-ketoacyl synthase (pfam00109),	GO:0071456|cellular response to hypoxia;GO:0006635|fatty acid beta-oxidation;GO:1902109|negative regulation of mitochondrial membrane permeability involved in apoptotic process;GO:1901029|negative regulation of mitochondrial outer membrane permeabilization involved in apoptotic signaling pathway;	GO:0070062|extracellular exosome;GO:0005743|mitochondrial inner membrane;GO:0005739|mitochondrion;	GO:0003988|acetyl-CoA C-acyltransferase activity;GO:0044822	K07508	ACAA2; acetyl-CoA acyltransferase 2 [EC:2.3.1.16]
LOC138991278	121.1744	-0.73097	0.200434344	-3.64691	0.0002654	0.005266	DOWN	yes	.	.	.	.	gi|742080315|ref|XR_826328.1|	2866	0	PREDICTED: Bison bison bison uncharacterized LOC104997381 (LOC104997381), ncRNA	.	.	.	.	.	.	.	.	.	.	.	.	.	.	.	.
STYX	392.6766	-0.55872	0.15321982	-3.64652	0.0002658	0.005266	Not_Sig	no	gi|602679420|ref|XP_007446021.1|	1273	4e-160	PREDICTED: serine/threonine/tyrosine-interacting protein [Lipotes vexillifer]	gi|594059852|ref|XM_006053985.1|	4218	0	PREDICTED: Bubalus bubalis serine/threonine/tyrosine interacting protein (STYX), transcript variant X1, mRNA	Q5RDP3	457	4e-148	Serine/threonine/tyrosine-interacting protein	SPBC17A3.06	82	1.21e-15	COG2453	pfam00782	DSPc	Dual specificity phosphatase, catalytic domain. Ser/Thr and Tyr protein phosphatases. The enzyme's tertiary fold is highly	GO:0045204|MAPK export from nucleus;GO:0070372|regulation of ERK1 and ERK2 cascade;	GO:0005737|cytoplasm;GO:0005829|cytosol;GO:0005634|nucleus;	GO:0008138|protein tyrosine/serine/threonine phosphatase	K18042	STYX; serine/threonine/tyrosine-interacting protein
LAMB2	2400.911	0.431489	0.118367815	3.645325	0.0002671	0.005273	Not_Sig	no	gi|555995374|ref|XP_005909429.1|	8844	0	PREDICTED: laminin subunit beta-2 [Bos mutus]	gi|555995373|ref|XM_005909367.1|	5570	0	PREDICTED: Bos mutus laminin, beta 2 (laminin S) (LAMB2), mRNA	P55268	3037	0	Laminin subunit beta-2	.	.	.	.	pfam00055	Laminin_N	Laminin N-terminal (Domain VI).	GO:0014002|astrocyte development;GO:0048677|axon extension involved in regeneration;GO:0007411|axon guidance;GO:0007155|cell adhesion;GO:0030198|extracellular matrix organization;GO:0072274|metanephric glomerular basement membrane development;GO:0072249|metanephric glomerular visceral epithelial cell development;GO:0007528|neuromuscular junction development;GO:0060041|retina development in camera-type eye;GO:0014044|Schwann cell development;GO:0007601|visual perception;	GO:0005605|basal lamina;GO:0005604|basement membrane;GO:0070062|extracellular exosome;GO:0005576|extracellular	GO:0005198|structural molecule activity;	K06243	LAMB2; laminin, beta 2
AURKA	337.0857	0.54778	0.150270556	3.645291	0.0002671	0.005273	Not_Sig	no	gi|84000031|ref|NP_001033117.1|	2081	0	aurora kinase A [Bos taurus]	gi|982942896|ref|XM_005214525.3|	1777	0	PREDICTED: Bos taurus aurora kinase A (AURKA), transcript variant X1, mRNA	Q2TA06	806	0	Aurora kinase A	.	.	.	.	pfam00069	Pkinase	Protein kinase domain.	GO:0009948|anterior/posterior axis specification;GO:0051301|cell division;GO:0051642|centrosome localization;GO:0000212|meiotic spindle organization;GO:0007100|mitotic centrosome separation;GO:0007052|mitotic spindle organization;GO:0043066|negative regulation of apoptotic process;GO:0032091|negative regulation of protein binding;GO:1990138|neuron projection extension;GO:1900195|positive regulation of oocyte maturation;GO:0032436|positive regulation of proteasomal ubiquitin-dependent protein catabolic	GO:0043203|axon hillock;GO:0005814|centriole;GO:0005813|centrosome;GO:0032133|chromosome passenger	GO:0005524|ATP binding;GO:0035174|histone serine kinase	K11481	AURKA; aurora kinase A [EC:2.7.11.1]
ZYX	8283.591	0.594526	0.163151124	3.644021	0.0002684	0.00529	UP	yes	gi|528947750|ref|XP_005205947.1|	1827	0	PREDICTED: zyxin isoform X1 [Bos taurus]	gi|741924770|ref|XM_005205890.2|	2330	0	PREDICTED: Bos taurus zyxin (ZYX), transcript variant X1, mRNA	Q62523	556	0	Zyxin	.	.	.	.	pfam00412	LIM	LIM domain. This family represents two copies of the LIM structural domain.	GO:0007160|cell-matrix adhesion;GO:0007229|integrin-mediated signaling pathway;GO:0050727|regulation of inflammatory response;GO:0043149|stress fiber assembly;GO:0007179|transforming growth factor beta receptor signaling pathway;	GO:0005913|cell-cell adherens junction;GO:0005737|cytoplasm;GO:0005925|focal adhesion;GO:0005634|nucleus;GO:0001725	GO:0044822|poly(A) RNA binding;GO:0008270|zinc ion binding;	K06273	ZYX; zyxin
DGCR2	1001.053	0.479097	0.131522205	3.642709	0.0002698	0.005305	Not_Sig	no	gi|116004287|ref|NP_001070504.1|	2181	0	integral membrane protein DGCR2/IDD precursor [Bos taurus]	gi|594084258|ref|XM_006065629.1|	2848	0	PREDICTED: Bubalus bubalis DiGeorge syndrome critical region gene 2 (DGCR2), partial mRNA	P98153	766	0	Integral membrane protein DGCR2/IDD	.	.	.	.	pfam00059	Lectin_C	Lectin C-type domain. This family includes both long and short form C-type	GO:0009887|organ morphogenesis;GO:0007155|cell adhesion;GO:0050890|cognition;	GO:0016021|integral component of membrane;	GO:0030246|carbohydrate binding;	.	.

CCDC91	603.3895	-0.46105	0.126594893	-3.6419	0.0002706	0.005305	Not_Sig	no	gi|555965135|ref|XP_005894627.1|	1994	0	PREDICTED: coiled-coil domain-containing protein 91 isoform X1 [Bos mutus]	gi|555965134|ref|XM_005894565.1|	1232	0	PREDICTED: Bos mutus coiled-coil domain containing 91 (CCDC91), transcript variant X1, mRNA	Q7Z6B0	709	0	Coiled-coil domain-containing protein 91	.	.	.	.	pfam02463	SMC_N	RecF/RecN/SMC N terminal domain. This domain is found at the N terminus of SMC proteins. The SMC (structural maintenance	GO:0015031|protein transport;	GO:0005794|Golgi apparatus;GO:0016020|membrane;GO:0005654|nucleoplasm;	.	.	.
MAN2A2	1027.82	0.376119	0.103287333	3.641484	0.0002711	0.005305	Not_Sig	no	gi|982960057|ref|XP_015314671.1|	5963	0	PREDICTED: alpha-mannosidase 2x isoform X1 [Bos taurus]	gi|742124381|ref|XM_010841061.1|	5195	0	PREDICTED: Bison bison bison mannosidase, alpha, class 2A, member 2 (MAN2A2), transcript variant X3, mRNA	P49641	2076	0	Alpha-mannosidase 2x	MT0676	73.2	1.15e-11	COG0383	pfam01074	Glyco_hydro_38	Glycosyl hydrolases family 38 N-terminal domain. Glycosyl hydrolases are key enzymes of carbohydrate metabolizm.	GO:0006013|mannose metabolic process;GO:0006491|N-glycan processing;GO:0006517|protein deglycosylation;	GO:0000139|Golgi membrane;GO:0016021|integral component of membrane;	GO:0004559|alpha-mannosidase activity;GO:0030246|carbohydrate	K01231	MAN2; alpha-mannosidase II [EC:3.2.1.114]
MTOR	1218.722	0.372886	0.102404535	3.6413	0.0002713	0.005305	Not_Sig	no	gi|742164375|ref|XP_010848198.1|	12691	0	PREDICTED: serine/threonine-protein kinase mTOR [Bison bison bison]	gi|942071021|ref|XM_005901305.2|	8618	0	PREDICTED: Bos mutus mechanistic target of rapamycin (serine/threonine kinase) (MTOR), mRNA	P42345	4866	0	Serine/threonine-protein kinase mTOR	SPBC30D10.10c	942	0	COG5032	pfam02259	FAT	FAT domain. The FAT domain is named after FRAP, ATM and TRRAP.	GO:0006207|'de novo' pyrimidine nucleobase biosynthetic process;GO:0007420|brain development;GO:0055013|cardiac muscle cell development;GO:0060048|cardiac muscle contraction;GO:0007569|cell aging;GO:0007050|cell cycle arrest;GO:0016049|cell growth;GO:0071456|cellular response to hypoxia;GO:0031669|cellular response to nutrient levels;GO:0006281|DNA repair;GO:0006112|energy reserve metabolic process;GO:0007281|germ cell development;GO:0040007|growth;GO:0003007|heart	GO:0005737|cytoplasm;GO:0005829|cytosol;GO:0030425|dendrite;GO:0012505|endomembrane system;GO:0005789|endoplasmic reticulum	GO:0005524|ATP binding;GO:0016301|kinase activity;GO:0051219	K07203	FRAP; FKBP12-rapamycin complex-associated protein
ARHGEF11	561.5542	0.516204	0.141771396	3.641102	0.0002715	0.005305	Not_Sig	no	gi|594055558|ref|XP_006052000.1|	6714	0	PREDICTED: rho guanine nucleotide exchange factor 11 isoform X6 [Bubalus bubalis]	gi|594055557|ref|XM_006051938.1|	6607	0	PREDICTED: Bubalus bubalis Rho guanine nucleotide exchange factor (GEF) 11 (ARHGEF11), transcript variant X6, mRNA	O15085	2193	0	Rho guanine nucleotide exchange factor 11	SPAC1006.06	86.7	8.11e-16	COG5422	pfam09128	RGS-like	Regulator of G protein signalling-like domain. Members of this family adopt a structure consisting of twelve helices that fold into a	GO:0030036|actin cytoskeleton organization;GO:0000910|cytokinesis;GO:0030010|establishment of cell polarity;GO:0007186|G-protein coupled receptor signaling pathway;GO:0006928|movement of cell or subcellular component;GO:0043065|positive regulation of apoptotic process;GO:0045893|positive regulation of transcription, DNA-templated;GO:0001558|regulation of cell growth;GO:0035023|regulation of Rho protein signal transduction;GO:0051056|regulation of small GTPase mediated signal transduction;GO:0007266|Rho protein signal	GO:0005737|cytoplasm;GO:0005829|cytosol;GO:0005622|intracellular;GO:0016020|membrane;	GO:0001664|G-protein coupled receptor binding;GO:0005096	K12331	ARHGEF11; Rho guanine nucleotide exchange factor 11
GPR146	37.15821	1.181031	0.324442475	3.640187	0.0002724	0.005315	UP	yes	gi|594111940|ref|XP_006078777.1|	1099	3e-134	PREDICTED: probable G-protein coupled receptor 146 isoform X1 [Bubalus bubalis]	gi|594111941|ref|XM_006078716.1|	2375	0	PREDICTED: Bubalus bubalis G protein-coupled receptor 146 (GPR146), transcript variant X2, mRNA	Q99LE2	279	1e-82	Probable G-protein coupled receptor 146	.	.	.	.	pfam15449	Retinal	Retinal protein. This family of proteins is found in the photoreceptor cells of the retina. Mutations of the gene encoding	.	GO:0016021|integral component of membrane;GO:0005886|plasma membrane;	GO:0004930|G-protein coupled receptor activity;	K08431	GPR146; G protein-coupled receptor 146
ZMYM5	315.2727	-0.49443	0.135938689	-3.63712	0.0002757	0.005369	Not_Sig	no	gi|303324573|ref|NP_001181947.1|	3387	0	zinc finger MYM-type protein 5 [Bos taurus]	gi|303324572|ref|NM_001195018.1|	2911	0	Bos taurus zinc finger, MYM-type 5 (ZMYM5), mRNA	A6QPH9	1310	0	Zinc finger MYM-type protein 5	.	.	.	.	pfam06467	zf-FCS	MYM-type Zinc finger with FCS sequence motif. MYM-type zinc fingers were identified in MYM family proteins. Human protein	GO:0007275|multicellular organismal development;GO:0006351|transcription, DNA-templated;	GO:0005737|cytoplasm;GO:0005654|nucleoplasm;	GO:0003677|DNA binding;GO:0000981|RNA polymerase II transcription factor	.	.
AMIGO3	459.2313	0.604632	0.166296157	3.635875	0.000277	0.005386	UP	yes	gi|297459150|ref|XP_002684533.1|	2305	0	PREDICTED: amphoterin-induced protein 3 [Bos taurus]	gi|742171478|ref|XM_010851851.1|	2716	0	PREDICTED: Bison bison bison adhesion molecule with Ig-like domain 3 (AMIGO3), mRNA	Q86WK7	688	0	Amphoterin-induced protein 3	.	.	.	.	pfam13855	LRR_8	Leucine rich repeat.	GO:0007157|heterophilic cell-cell adhesion via plasma membrane cell adhesion molecules;GO:0051965|positive regulation of synapse assembly;	GO:0016021|integral component of membrane;	.	.	.
COL5A2	45591.43	-0.32008	0.088063464	-3.63466	0.0002783	0.005402	Not_Sig	no	gi|585200918|ref|XP_006751388.1|	1481	1e-178	PREDICTED: collagen alpha-2(V) chain-like, partial [Leptonychotes weddellii]	gi|942037675|ref|XM_005889539.2|	5721	0	PREDICTED: Bos mutus collagen, type V, alpha 2 (COL5A2), mRNA	P05997	566	3e-170	Collagen alpha-2(V) chain	.	.	.	.	pfam01410	COLFI	Fibrillar collagen C-terminal domain. Found at C-termini of fibrillar collagens: Ephydatia muelleri procollagen EMF1 alpha,	GO:0071230|cellular response to amino acid stimulus;GO:0030574|collagen catabolic process;GO:0030199|collagen fibril organization;GO:0030198|extracellular matrix organization;GO:0048592|eye morphogenesis;GO:1903225|negative regulation of endodermal cell differentiation;GO:0001503|ossification;GO:0001501|skeletal system development;GO:0043588|skin development;	GO:0005588|collagen type V trimer;GO:0005788|endoplasmic reticulum lumen;GO:0031012|extracellular matrix;GO:0005576|extracellular region;	GO:0005201|extracellular matrix structural constituent;GO:004	.	.
CDC42EP1	1189.374	0.436855	0.120220527	3.633784	0.0002793	0.005412	Not_Sig	no	gi|555966441|ref|XP_005895272.1|	1192	8e-145	PREDICTED: cdc42 effector protein 1 [Bos mutus]	gi|942053186|ref|XM_005895210.2|	4061	0	PREDICTED: Bos mutus CDC42 effector protein (Rho GTPase binding) 1 (CDC42EP1), mRNA	Q17QW1	459	7e-145	Cdc42 effector protein 1	.	.	.	.	pfam14957	BORG_CEP	Cdc42 effector. The Cdc42 effector (CEP) or binder of Rho GTPases (BORG) proteins are involved in the organisation of the actin	GO:0030838|positive regulation of actin filament polymerization;GO:0031274|positive regulation of pseudopodium assembly;GO:0008360|regulation of cell shape;GO:0007266|Rho protein signal transduction;	GO:0015629|actin cytoskeleton;GO:0005737|cytoplasm;GO:0005925|focal adhesion;GO:0005794|Golgi apparatus;GO:0005886|plasma membrane;	GO:0017049|GTP-Rho binding;GO:0005096|GTPase activator	.	.
TMEM41B	615.2667	-0.55906	0.153879766	-3.63311	0.00028	0.005417	Not_Sig	no	gi|440906656|gb|ELR56889.1|	1586	0	Transmembrane protein 41B, partial [Bos mutus]	gi|942052711|ref|XM_005895051.2|	2553	0	PREDICTED: Bos mutus transmembrane protein 41B (TMEM41B), mRNA	Q5FVN2	555	0	Transmembrane protein 41B	VC1723	82.4	9.52e-17	COG0398	pfam09335	SNARE_assoc	SNARE associated Golgi protein. This is a family of SNARE associated Golgi proteins. The yeast member of this family	GO:0007399|nervous system development;	GO:0016021|integral component of membrane;	.	.	.
EPN1	1855.102	0.540104	0.148802018	3.629685	0.0002838	0.005471	Not_Sig	no	gi|528993395|ref|XP_005219737.1|	2025	0	PREDICTED: epsin-1 isoform X1 [Bos taurus]	gi|741961249|ref|XM_005219680.2|	2387	0	PREDICTED: Bos taurus epsin 1 (EPN1), transcript variant X1, mRNA	Q9Y6I3	721	0	Epsin-1	.	.	.	.	pfam01417	ENTH	ENTH domain. The ENTH (Epsin N-terminal homology) domain is found in proteins involved in endocytosis and cytoskeletal	GO:0048568|embryonic organ development;GO:0006897|endocytosis;GO:0007565|female pregnancy;GO:0001701|in utero embryonic development;GO:0042059|negative regulation of epidermal growth factor receptor signaling pathway;GO:0007219|Notch signaling pathway;	GO:0005905|coated pit;GO:0005737|cytoplasm;GO:0005634|nucleus;GO:0005886|plasma membrane;	GO:0008289|lipid binding;	K12471	EPN; epsin
ULK1	1174.029	0.622626	0.17153751	3.629676	0.0002838	0.005471	UP	yes	gi|741956184|ref|XP_010812125.1|	4721	0	PREDICTED: serine/threonine-protein kinase ULK1 isoform X1 [Bos taurus]	gi|982950026|ref|XM_010813823.2|	5039	0	PREDICTED: Bos taurus unc-51 like autophagy activating kinase 1 (ULK1), transcript variant X1, mRNA	O75385	1080	0	Serine/threonine-protein kinase ULK1	.	.	.	.	pfam00069	Pkinase	Protein kinase domain.	GO:0048675|axon extension;GO:0031669|cellular response to nutrient levels;GO:0016236|macroautophagy;GO:0031333|negative regulation of protein complex assembly;GO:0031175|neuron projection development;GO:0031102|neuron projection regeneration;GO:0018105|peptidyl-serine phosphorylation;GO:0010508|positive regulation of autophagy;GO:0016239|positive regulation of macroautophagy;GO:0046777|protein autophosphorylation;GO:0008104|protein localization;GO:0006468|protein	GO:1990316|ATG1/ULK1 kinase complex;GO:0005776|autophagosome;GO:0005737|cytoplasm;GO:0005829|cytosol;GO:0097635|extrinsic component of	GO:0005524|ATP binding;GO:0032403|protein complex binding;GO:0004674	K08269	ULK1_2_3, ATG1; serine/threonine-protein kinase ULK/ATG1
UBC	5938.438	0.556539	0.153364642	3.628861	0.0002847	0.005471	Not_Sig	no	gi|62089150|dbj|BAD93019.1|	4062	0	ubiquitin C variant [Homo sapiens]	gi|942063406|ref|XM_014479128.1|	2458	0	PREDICTED: Bos mutus ubiquitin C (UBC), mRNA	P0CG71	1531	0	Polyubiquitin-A	YLL039c	703	0	COG5272	pfam00240	ubiquitin	Ubiquitin family. This family contains a number of ubiquitin-like proteins: SUMO (smt3 homologue) Nedd8, Elongin B, Rub1, and	.	GO:0005737|cytoplasm;GO:0005634|nucleus;	.	K08770	UBC; ubiquitin C
RPL17	3975.306	-0.40368	0.111243209	-3.62882	0.0002847	0.005471	Not_Sig	no	gi|556777669|ref|XP_005983921.1|	942	1e-126	PREDICTED: 60S ribosomal protein L17-like [Pantholops hodgsonii]	gi|742164093|ref|XM_010849822.1|	618	0	PREDICTED: Bison bison bison ribosomal protein L17 (RPL17), transcript variant X2, mRNA	P24049	352	6e-123	60S ribosomal protein L17	YKL180w	205	5.58e-67	COG0091	pfam00237	Ribosomal_L22	Ribosomal protein L22p/L17e. This family includes L22 from prokaryotes and chloroplasts and L17 from eukaryotes.	GO:0034198|cellular response to amino acid starvation;GO:1900087|positive regulation of G1/S transition of mitotic cell cycle;GO:0006412|translation;	GO:0031672|A band;GO:0022625|cytosolic large ribosomal subunit;GO:0005634|nucleus;GO:0005844|polysome;	GO:0070180|large ribosomal subunit rRNA binding;GO:0003735	K02880	RP-L17e, RPL17; large subunit ribosomal protein L17e
PTPN23	1504.189	0.551248	0.15194732	3.627889	0.0002857	0.005481	Not_Sig	no	gi|330688392|ref|NP_001193455.1|	6483	0	tyrosine-protein phosphatase non-receptor type 23 [Bos taurus]	gi|330688391|ref|NM_001206526.1|	4893	0	Bos taurus protein tyrosine phosphatase, non-receptor type 23 (PTPN23), mRNA	Q9H3S7	1480	0	Tyrosine-protein phosphatase non-receptor type 23	.	.	.	.	pfam03097	BRO1	BRO1-like domain. This domain is found in a number proteins including Rhophilin and BRO1. It is known to have a role in endosomal	GO:0060271|cilium morphogenesis;GO:0010633|negative regulation of epithelial cell migration;GO:1903393|positive regulation of adherens junction organization;GO:2000643|positive regulation of early endosome to late endosome transport;GO:1903387|positive regulation of homophilic cell adhesion;GO:0015031|protein transport;GO:0043162|ubiquitin-dependent protein catabolic process via the multivesicular body sorting pathway;	GO:0036064|ciliary basal body;GO:0005737|cytoplasm;GO:0016023|cytoplasmic membrane-bounded vesicle;GO:0005769|early	GO:0019901|protein kinase binding;GO:0004725|protein tyrosine	K18040	PTPN23; tyrosine-protein phosphatase non-receptor type 23 [EC:3.1.3.48]
PSKH1	375.1897	0.517926	0.142814989	3.626551	0.0002872	0.0055	Not_Sig	no	gi|965973459|ref|XP_004015130.2|	2237	0	PREDICTED: serine/threonine-protein kinase H1 isoform X1 [Ovis aries]	gi|555969131|ref|XM_005896531.1|	3362	0	PREDICTED: Bos mutus protein serine kinase H1 (PSKH1), mRNA	Q0V7M1	801	0	Serine/threonine-protein kinase H1	.	.	.	.	pfam00069	Pkinase	Protein kinase domain.	GO:0007368|determination of left/right symmetry;GO:0007507|heart development;	GO:0005789|endoplasmic reticulum membrane;GO:0005794|Golgi apparatus;GO:0005815|microtubule organizing center;GO:0016607|nuclear	GO:0005524|ATP binding;GO:0004674|protein serine/threonine	K08808	PSKH; protein serine kinase H [EC:2.7.11.1]
TMEM109	1525.228	0.46104	0.127219235	3.623984	0.0002901	0.005546	Not_Sig	no	gi|114050781|ref|NP_001039935.1|	673	2e-79	transmembrane protein 109 precursor [Bos taurus]	gi|742116911|ref|XM_010839633.1|	2076	0	PREDICTED: Bison bison bison transmembrane protein 109 (TMEM109), mRNA	O77751	218	6e-64	Transmembrane protein 109	.	.	.	.	pfam14965	BRI3BP	Negative regulator of p53/TP53. This family of transmembrane proteins is found in eukaryotes. Proteins in this family are typically	.	GO:0005783|endoplasmic reticulum;GO:0016021|integral component of membrane;GO:0031965|nuclear membrane;GO:0005640|nuclear outer	GO:0005244|voltage-gated ion channel activity;	.	.
ELK1	884.7606	0.469399	0.129565319	3.622875	0.0002913	0.005561	Not_Sig	no	gi|440903123|gb|ELR53825.1|	1750	0	ETS domain-containing protein Elk-1, partial [Bos mutus]	gi|982973977|ref|XM_010821919.2|	2772	0	PREDICTED: Bos taurus ELK1, member of ETS oncogene family (ELK1), transcript variant X1, mRNA	A4GTP4	532	6e-179	ETS domain-containing protein Elk-1	.	.	.	.	pfam00178	Ets	Ets-domain.	GO:0030154|cell differentiation;GO:0071480|cellular response to gamma radiation;GO:0071396|cellular response to lipid;GO:0071394|cellular response to testosterone stimulus;GO:1901216|positive regulation of neuron death;GO:0045944|positive regulation of transcription from RNA polymerase II promoter;GO:0071774|response to fibroblast growth factor;GO:0009416|response to light stimulus;	GO:0043679|axon terminus;GO:0005737|cytoplasm;GO:0030425|dendrite;GO:0005739|mitochondrion;GO:0043025|neuronal cell	GO:0003682|chromatin binding;GO:0001047|core promoter	K04375	ELK1; ETS domain-containing protein Elk-1
POC1A	242.9789	0.539927	0.149083711	3.621634	0.0002927	0.005571	Not_Sig	no	gi|133778015|gb|AAI19694.1|	2058	0	WDR51A protein, partial [Homo sapiens]	gi|982962082|ref|XM_015459589.1|	1693	0	PREDICTED: Bos taurus POC1 centriolar protein A (POC1A), transcript variant X1, mRNA	Q2TBP4	789	0	POC1 centriolar protein homolog A	.	.	.	.	pfam00400	WD40	WD domain, G-beta repeat.	GO:0030030|cell projection organization;	GO:0005814|centriole;GO:0036064|ciliary basal body;GO:0000922|spindle pole;	.	K16482	POC1; centriolar protein POC1
EP300	1366.81	0.469349	0.129602545	3.621446	0.000293	0.005571	Not_Sig	no	gi|594091123|ref|XP_006068949.1|	5579	0	PREDICTED: histone acetyltransferase p300 isoform X1 [Bubalus bubalis]	gi|942096578|ref|XM_005909427.2|	8291	0	PREDICTED: Bos mutus E1A binding protein p300 (EP300), mRNA	Q09472	2028	0	Histone acetyltransferase p300	SPAC631.02	79.3	1.07e-13	COG5076	pfam08214	KAT11	Histone acetylation protein. Histone acetylation is required in many cellular processes including transcription, DNA repair, and	GO:0009887|organ morphogenesis;GO:0006915|apoptotic process;GO:0030183|B cell differentiation;GO:0034644|cellular response to UV;GO:0007623|circadian rhythm;GO:0006977|DNA damage response, signal transduction by p53 class mediator resulting in cell cycle arrest;GO:0045444|fat cell differentiation;GO:0007507|heart development;GO:0043969|histone H2B acetylation;GO:0043967|histone H4 acetylation;GO:0018393|internal peptidyl-lysine acetylation;GO:0006475|internal protein amino acid acetylation;GO:0042771|intrinsic	GO:0005737|cytoplasm;GO:0000123|histone acetyltransferase complex;GO:0005654|nucleoplasm;GO:0005634|nucleus;GO:0005667|transcription	GO:0016407|acetyltransferase activity;GO:0033613|activating	K04498	EP300, CREBBP, KAT3; E1A/CREB-binding protein [EC:2.3.1.48]
ZBTB41	377.7752	-0.51539	0.142329382	-3.62111	0.0002933	0.005571	Not_Sig	no	gi|555970520|ref|XP_005897281.1|	4490	0	PREDICTED: zinc finger and BTB domain-containing protein 41 [Bos mutus]	gi|982997371|ref|XM_003583275.4|	8324	0	PREDICTED: Bos taurus zinc finger and BTB domain containing 41 (ZBTB41), transcript variant X2, mRNA	Q5SVQ8	1658	0	Zinc finger and BTB domain-containing protein 41	SPAC144.09c	91.7	3.24e-18	COG5048	pfam00651	BTB	BTB/POZ domain. The BTB (for BR-C, ttk and bab) or POZ (for Pox virus and Zinc finger) domain is present near the N-terminus of a	GO:0006355|regulation of transcription, DNA-templated;GO:0006351|transcription, DNA-templated;	GO:0005634|nucleus;	GO:0003677|DNA binding;GO:0046872|metal ion binding;	K10513	ZBTB41; zinc finger and BTB domain-containing protein 41
LOC138987848	329.7415	-0.72639	0.200646614	-3.62027	0.0002943	0.00558	DOWN	yes	gi|332240596|ref|XP_003269473.1|	1154	5e-157	PREDICTED: 60S ribosomal protein L7 [Nomascus leucogenys]	gi|942090445|ref|XM_005907515.2|	815	0	PREDICTED: Bos mutus ribosomal protein L7 (RPL7), transcript variant X2, mRNA	Q58DT1	440	2e-155	60S ribosomal protein L7	SPBC18H10.12c	248	9.60e-82	COG1841	pfam08079	Ribosomal_L30_N	Ribosomal L30 N-terminal domain. This presumed domain is found at the N-terminus of Ribosomal L30 proteins and has been termed tumor-associated protein.	GO:0002181|cytoplasmic translation;GO:0000463|maturation of LSU-rRNA from tricistronic rRNA transcript (SSU-rRNA, 5.8S rRNA, LSU-rRNA);	GO:0022625|cytosolic large ribosomal subunit;	GO:0044822|poly(A) RNA binding;GO:0003735|structural	K02937	RP-L7e, RPL7; large subunit ribosomal protein L7e
TMEM259	2636.572	0.528913	0.14612583	3.619574	0.0002951	0.005586	Not_Sig	no	gi|742142594|ref|XP_010842946.1|	2360	0	PREDICTED: membralin [Bison bison bison]	gi|594052465|ref|XM_006050484.1|	2484	0	PREDICTED: Bubalus bubalis transmembrane protein 259 (TMEM259), mRNA	Q4ZIN3	796	0	Membralin	.	.	.	.	pfam09746	Membralin	Membralin is evolutionarily highly conserved; though it seems to represent a unique protein family.	GO:1901215|negative regulation of neuron death;GO:1904294|positive regulation of ERAD pathway;GO:0034976|response to endoplasmic reticulum stress;	GO:0005789|endoplasmic reticulum membrane;GO:0016021|integral component of membrane;	.	.	.
TMEM131	2610.022	0.402963	0.11142197	3.616553	0.0002986	0.005634	Not_Sig	no	gi|297480081|ref|XP_002691187.1|	7991	0	PREDICTED: transmembrane protein 131 isoform X1 [Bos taurus]	gi|982990330|ref|XM_613474.9|	6876	0	PREDICTED: Bos taurus transmembrane protein 131 (TMEM131), transcript variant X1, mRNA	Q92545	2338	0	Transmembrane protein 131	.	.	.	.	pfam12371	DUF3651	Protein of unknown function (DUF3651). This domain family is found in eukaryotes, and is approximately 70 amino acids in	.	GO:0016021|integral component of membrane;GO:0016020|membrane;	.	.	.
NFATC4	928.692	0.470778	0.130174805	3.616503	0.0002986	0.005634	Not_Sig	no	gi|555997231|ref|XP_005910343.1|	3167	0	PREDICTED: nuclear factor of activated T-cells, cytoplasmic 4 isoform X1 [Bos mutus]	gi|942098662|ref|XM_005910281.2|	3044	0	PREDICTED: Bos mutus nuclear factor of activated T-cells, cytoplasmic, calcineurin-dependent 4 (NFATC4), transcript variant X1, mRNA	Q14934	1160	0	Nuclear factor of activated T-cells, cytoplasmic 4	.	.	.	.	pfam00554	RHD	Rel homology domain (RHD). Proteins containing the Rel homology domain (RHD) are eukaryotic transcription factors.	GO:0033173|calcineurin-NFAT signaling cascade;GO:0045333|cellular respiration;GO:0071285|cellular response to lithium ion;GO:0034644|cellular response to UV;GO:0007507|heart development;GO:0006954|inflammatory response;GO:0008630|intrinsic apoptotic signaling pathway in response to DNA damage;GO:0055001|muscle cell development;GO:0035562|negative regulation of chromatin binding;GO:0050774|negative regulation of dendrite morphogenesis;GO:1902894|negative regulation of pri-miRNA transcription from RNA	GO:0005737|cytoplasm;GO:0005829|cytosol;GO:0045111|intermediate filament cytoskeleton;GO:0005634|nucleus;GO:0005667|transcription factor complex;	GO:0000978|RNA polymerase II core promoter proximal region sequence-	K17334	NFATC4, NFAT3; nuclear factor of activated T-cells, cytoplasmic 4
PC	486.3043	0.512418	0.141719719	3.615717	0.0002995	0.005635	Not_Sig	no	gi|529012414|ref|XP_005227045.1|	5614	0	PREDICTED: pyruvate carboxylase, mitochondrial isoform X2 [Bos taurus]	gi|942077955|ref|XM_005903546.2|	3961	0	PREDICTED: Bos mutus pyruvate carboxylase (PC), mRNA	Q29RK2	2167	0	Pyruvate carboxylase, mitochondrial	SPBC17G9.11c	1120	0	COG1038	pfam02436	PYC_OADA	Conserved carboxylase domain. This domain represents a conserved region in pyruvate carboxylase (PYC), oxaloacetate	GO:0006094|gluconeogenesis;GO:0006629|lipid metabolic process;GO:0010629|negative regulation of gene expression;GO:0044791|positive regulation by host of viral release from host cell;GO:0006090|pyruvate metabolic process;GO:0019074|viral RNA genome packaging;	GO:0005743|mitochondrial inner membrane;GO:0005759|mitochondrial matrix;GO:0005739|mitochondrion;	GO:0005524|ATP binding;GO:0009374|biotin binding;GO:0004075	K01958	PC, pyc; pyruvate carboxylase [EC:6.4.1.1]
ZNF469	895.9853	0.505588	0.139845225	3.615343	0.0003	0.005635	Not_Sig	no	gi|594037177|ref|XP_006043228.1|	15945	0	PREDICTED: LOW QUALITY PROTEIN: zinc finger protein 469 [Bubalus bubalis]	gi|594037176|ref|XM_006043166.1|	11935	0	PREDICTED: Bubalus bubalis zinc finger protein 469 (ZNF469), mRNA	Q96JG9	2668	0	Zinc finger protein 469	.	.	.	.	pfam15449	Retinal	Retinal protein. This family of proteins is found in the photoreceptor cells of the retina. Mutations of the gene encoding	GO:0006355|regulation of transcription, DNA-templated;GO:0006351|transcription, DNA-templated;	GO:0005634|nucleus;	GO:0003677|DNA binding;GO:0046872|metal ion binding;	.	.
DGKZ	517.821	0.450696	0.124668217	3.61516	0.0003002	0.005635	Not_Sig	no	gi|803223920|ref|XP_011972253.1|	4885	0	PREDICTED: diacylglycerol kinase zeta isoform X2 [Ovis aries musimon]	gi|965802014|ref|XM_012116863.2|	3626	0	PREDICTED: Ovis aries musimon diacylglycerol kinase, zeta (DGKZ), transcript variant X2, mRNA	Q13574	1816	0	Diacylglycerol kinase zeta	.	.	.	.	pfam00609	DAGK_acc	Diacylglycerol kinase accessory domain. Diacylglycerol (DAG) is a second messenger that acts as a protein kinase C activator. This	GO:0016477|cell migration;GO:0046339|diacylglycerol metabolic process;GO:0046486|glycerolipid metabolic process;GO:0035556|intracellular signal transduction;GO:0046834|lipid phosphorylation;GO:0031571|mitotic G1 DNA damage checkpoint;GO:0045930|negative regulation of mitotic cell cycle;GO:0030168|platelet activation;GO:0007205|protein kinase C-activating G-protein coupled receptor signaling pathway;	GO:0005737|cytoplasm;GO:0030027|lamellipodium;GO:0005654|nucleoplasm;GO:0005634|nucleus;GO:0005886|plasma membrane;	GO:0005524|ATP binding;GO:0004143|diacylglycerol kinase	K00901	dgkA, DGK; diacylglycerol kinase (ATP) [EC:2.7.1.107]
ANKRD11	2893.959	0.404491	0.112036921	3.610341	0.0003058	0.005732	Not_Sig	no	gi|982951950|ref|XP_015331275.1|	9717	0	PREDICTED: ankyrin repeat domain-containing protein 11 isoform X3 [Bos taurus]	gi|982999138|ref|XM_015467318.1|	8884	0	PREDICTED: Bos taurus ankyrin repeat domain 11 (ANKRD11), transcript variant X3, mRNA	Q6UB99	1472	0	Ankyrin repeat domain-containing protein 11	.	.	.	.	pfam12796	Ank_2	Ankyrin repeats (3 copies).	GO:0060348|bone development;GO:0060325|face morphogenesis;GO:0001701|in utero embryonic development;GO:0035264|multicellular organism growth;GO:0042475|odontogenesis of dentin-containing tooth;GO:0048705|skeletal system morphogenesis;GO:0001894|tissue homeostasis;	GO:0005737|cytoplasm;GO:0005654|nucleoplasm;GO:0005634|nucleus;GO:0005886|plasma membrane;	.	.	.
CDC42BPB	2923.025	0.390941	0.108402626	3.606378	0.0003105	0.005805	Not_Sig	no	gi|594112642|ref|XP_006079105.1|	8346	0	PREDICTED: serine/threonine-protein kinase MRCK beta isoform X1 [Bubalus bubalis]	gi|983004148|ref|XM_015468615.1|	6099	0	PREDICTED: Bos taurus CDC42 binding protein kinase beta (CDC42BPB), transcript variant X3, mRNA	Q9Y5S2	2992	0	Serine/threonine-protein kinase MRCK beta	.	.	.	.	pfam00780	CNH	CNH domain. Domain found in NIK1-like kinase, mouse citron and yeast ROM1, ROM2. Unpublished observations.	GO:0031532|actin cytoskeleton reorganization;GO:0031032|actomyosin structure organization;GO:0016477|cell migration;GO:0007010|cytoskeleton organization;GO:0007163|establishment or maintenance of cell polarity;GO:0035556|intracellular signal transduction;GO:0006468|protein phosphorylation;GO:0007165|signal transduction;	GO:0042641|actomyosin;GO:0031252|cell leading edge;GO:0005911|cell-cell junction;GO:0005737|cytoplasm;GO:0005856|cytoskeleton;GO:0070062|extracellular	GO:0005524|ATP binding;GO:0000287|magnesium ion binding;GO:0004672	K16307	CDC42BP; serine/threonine-protein kinase MRCK [EC:2.7.11.1]
KGD4	354.4961	-0.67103	0.186076957	-3.60619	0.0003107	0.005805	DOWN	yes	gi|78369264|ref|NP_001030515.1|	455	1e-51	28S ribosomal protein S36, mitochondrial [Bos taurus]	gi|594070229|ref|XM_006058940.1|	1269	0	PREDICTED: Bubalus bubalis mitochondrial ribosomal protein S36 (MRPS36), mRNA	P82908	179	4e-53	28S ribosomal protein S36, mitochondrial	.	.	.	.	pfam10937	DUF2638	Protein of unknown function (DUF2638). This family of proteins has no known function.	GO:0006103|2-oxoglutarate metabolic process;GO:0070125|mitochondrial translational elongation;GO:0070124|mitochondrial translational initiation;GO:0055114|oxidation-reduction process;	GO:0005743|mitochondrial inner membrane;GO:0009353|mitochondrial oxoglutarate dehydrogenase complex;GO:0005763|mitochondrial small	.	K17414	MRPS36; small subunit ribosomal protein S36
YOD1	377.8345	-0.58448	0.162104136	-3.6056	0.0003114	0.005809	Not_Sig	no	gi|555959197|ref|XP_005891709.1|	1863	0	PREDICTED: ubiquitin thioesterase OTU1 [Bos mutus]	gi|965840734|ref|XM_012135585.2|	5484	0	PREDICTED: Ovis aries musimon YOD1 deubiquitinase (YOD1), transcript variant X1, mRNA	Q05B57	720	0	Ubiquitin thioesterase OTU1	SPAC24C9.14	155	2.10e-40	COG5539	pfam02338	OTU	OTU-like cysteine protease. This family is comprised of a group of predicted cysteine proteases, homologous to the Ovarian tumor	GO:0030968|endoplasmic reticulum unfolded protein response;GO:0030433|ER-associated ubiquitin-dependent protein catabolic process;GO:0035871|protein K11-linked deubiquitination;GO:1990167|protein K27-linked deubiquitination;GO:0035523|protein K29-linked deubiquitination;GO:1990168|protein K33-linked deubiquitination;GO:0071108|protein K48-linked deubiquitination;GO:0070536|protein K63-linked deubiquitination;	.	GO:1990380|Lys48-specific deubiquitinase activity;GO:0046872	K13719	OTU1, YOD1; ubiquitin thioesterase OTU1 [EC:3.1.2.-]
RPS13	2641.554	-0.54235	0.15048399	-3.60407	0.0003133	0.005828	Not_Sig	no	gi|958720315|ref|XP_014703348.1|	812	2e-107	PREDICTED: 40S ribosomal protein S13 [Equus asinus]	gi|742230900|ref|XM_010830531.1|	586	0	PREDICTED: Bison bison bison ribosomal protein S13 (RPS13), mRNA	P62278	305	4e-105	40S ribosomal protein S13	YDR064w	250	1.95e-85	COG0184	pfam08069	Ribosomal_S13_N	Ribosomal S13/S15 N-terminal domain. This domain is found at the N-terminus of ribosomal S13 and S15 proteins. This domain is	GO:0033119|negative regulation of RNA splicing;GO:0006412|translation;	GO:0022627|cytosolic small ribosomal subunit;GO:0070062|extracellular exosome;GO:0005925|focal adhesion;GO:0016020|membrane;GO:0005	GO:0003729|mRNA binding;GO:0070181|small ribosomal subunit rRNA	K02953	RP-S13e, RPS13; small subunit ribosomal protein S13e
ZDHHC20	2084.296	-0.47928	0.132990788	-3.60388	0.0003135	0.005828	Not_Sig	no	gi|115497024|ref|NP_001069856.1|	1765	0	probable palmitoyltransferase ZDHHC20 [Bos taurus]	gi|528971451|ref|XM_005213801.1|	4655	0	PREDICTED: Bos taurus zinc finger, DHHC-type containing 20 (ZDHHC20), transcript variant X1, mRNA	Q0VC89	683	0	Probable palmitoyltransferase ZDHHC20	SPBC2F12.15c	107	3.05e-24	COG5273	pfam01529	zf-DHHC	DHHC palmitoyltransferase. This family includes the well known DHHC zinc binding domain as well as three of the four conserved	GO:0018345|protein palmitoylation;	GO:0016021|integral component of membrane;GO:0005886|plasma membrane;	GO:0019706|protein-cysteine S-palmitoyltransferase activity;GO:0008270	.	.
ATP5MG	1356.699	-0.54289	0.150666837	-3.60325	0.0003143	0.005828	Not_Sig	no	gi|311307317|ref|NP_001019721.4|	529	3e-66	ATP synthase subunit g, mitochondrial [Bos taurus]	gi|942058046|ref|XM_005897149.2|	524	0	PREDICTED: Bos mutus ATP synthase, H+ transporting, mitochondrial Fo complex, subunit G (ATP5L), transcript variant X1, mRNA	Q28852	206	4e-67	ATP synthase subunit g, mitochondrial	.	.	.	.	pfam04718	ATP-synt_G	Mitochondrial ATP synthase g subunit. The Fo sector of the ATP synthase is a membrane bound complex which mediates proton	GO:0015986|ATP synthesis coupled proton transport;	GO:0005753|mitochondrial proton-transporting ATP synthase complex;GO:0000276|mitochondrial proton-transporting ATP synthase complex,	GO:0015078|hydrogen ion transmembrane transporter activity;	K02140	ATPeFG, ATP5L, ATP20; F-type H+-transporting ATPase subunit g
TMEM33	1454.655	-0.53444	0.148328972	-3.60309	0.0003145	0.005828	Not_Sig	no	gi|555998016|ref|XP_005910724.1|	1200	1e-147	PREDICTED: transmembrane protein 33 isoform X1 [Bos mutus]	gi|942099942|ref|XM_005910662.2|	6324	0	PREDICTED: Bos mutus transmembrane protein 33 (TMEM33), transcript variant X1, mRNA	P57088	457	1e-145	Transmembrane protein 33	.	.	.	.	pfam03661	UPF0121	Uncharacterized protein family (UPF0121). Uncharacterized integral membrane protein family.	GO:0034613|cellular protein localization;GO:0051292|nuclear pore complex assembly;GO:1903896|positive regulation of IRE1-mediated unfolded protein response;GO:1903899|positive regulation of PERK-mediated unfolded protein response;GO:1903371|regulation of endoplasmic reticulum tubular network organization;GO:0034976|response to endoplasmic reticulum stress;	GO:0005783|endoplasmic reticulum;GO:0005789|endoplasmic reticulum membrane;GO:0070062|extracellular	GO:0017056|structural constituent of nuclear pore;	.	.
SIPA1	337.241	0.60637	0.168343618	3.601976	0.0003158	0.005843	UP	yes	gi|156120439|ref|NP_001095365.1|	4391	0	signal-induced proliferation-associated protein 1 [Bos taurus]	gi|982971873|ref|XM_005227030.3|	3495	0	PREDICTED: Bos taurus signal-induced proliferation-associated 1 (SIPA1), transcript variant X1, mRNA	Q96FS4	1545	0	Signal-induced proliferation-associated protein 1	.	.	.	.	pfam02145	Rap_GAP	Rap/ran-GAP.	GO:0008283|cell proliferation;GO:0042631|cellular response to water deprivation;GO:0007010|cytoskeleton organization;GO:0035556|intracellular signal transduction;GO:0007162|negative regulation of cell adhesion;GO:0045786|negative regulation of cell cycle;GO:0030308|negative regulation of cell growth;GO:0051056|regulation of small GTPase mediated signal transduction;GO:0007165|signal transduction;	GO:0005829|cytosol;GO:0016020|membrane;GO:0005634|nucleus;GO:0048471|perinuclear region of cytoplasm;GO:0043234|protein	GO:0005096|GTPase activator activity;	K08013	SIPA1; signal-induced proliferation-associated gene 1
TMEM184B	1192.162	0.546469	0.151757303	3.60094	0.0003171	0.005857	Not_Sig	no	gi|528951403|ref|XP_005207380.1|	2030	0	PREDICTED: transmembrane protein 184B isoform X1 [Bos taurus]	gi|982927271|ref|XM_005207323.3|	3500	0	PREDICTED: Bos taurus transmembrane protein 184B (TMEM184B), transcript variant X1, mRNA	A2VDL9	763	0	Transmembrane protein 184B	.	.	.	.	pfam03619	Solute_trans_a	Organic solute transporter Ostalpha. This family is a transmembrane organic solute transport protein. In vertebrates	GO:0006810|transport;	GO:0016021|integral component of membrane;	GO:0005215|transporter activity;	.	.
GOPC	297.2547	-0.61444	0.170722375	-3.59906	0.0003194	0.005883	DOWN	yes	gi|329663844|ref|NP_001193086.1|	2181	0	Golgi-associated PDZ and coiled-coil motif-containing protein [Bos taurus]	gi|942067279|ref|XM_005900056.2|	4554	0	PREDICTED: Bos mutus golgi-associated PDZ and coiled-coil motif containing (GOPC), transcript variant X1, mRNA	Q9HD26	820	0	Golgi-associated PDZ and coiled-coil motif-containing protein	.	.	.	.	pfam00595	PDZ	PDZ domain (Also known as DHR or GLGF). PDZ domains are found in diverse signaling proteins.	GO:0045176|apical protein localization;GO:0043004|cytoplasmic sequestering of CFTR protein;GO:0006888|ER to Golgi vesicle-mediated transport;GO:0006893|Golgi to plasma membrane transport;GO:0051260|protein homooligomerization;GO:0015031|protein transport;GO:0007289|spermatid nucleus differentiation;	GO:0030054|cell junction;GO:0005737|cytoplasm;GO:0030425|dendrite;GO:0005794|Golgi apparatus;GO:0000139|Golgi	GO:0030695|GTPase regulator activity;GO:0044325|ion channel	.	.

BAG6	3656.697	0.433164	0.120357685	3.598974	0.0003195	0.005883	Not_Sig	no	gi|440898771|gb|ELR50196.1|	4090	0	Large proline-rich protein BAT3 [Bos mutus]	gi|982963813|ref|XM_005223611.2|	3825	0	PREDICTED: Bos taurus BCL2 associated athanogene 6 (BAG6), transcript variant X2, mRNA	A5D9M6	1485	0	Large proline-rich protein BAG6	SPAC26A3.16	53.5	2.39e-06	COG5272	pfam12057	DUF3538	Domain of unknown function (DUF3538). This presumed domain is functionally uncharacterized. This domain is found in	GO:0007420|brain development;GO:0030154|cell differentiation;GO:0016569|covalent chromatin modification;GO:0009790|embryo development;GO:0002376|immune system process;GO:0018393|internal peptidyl-lysine acetylation;GO:0042771|intrinsic apoptotic signaling pathway in response to DNA damage by p53 class mediator;GO:0070059|intrinsic apoptotic signaling pathway in response to endoplasmic reticulum stress;GO:0001822|kidney development;GO:0030324|lung development;GO:1904378|maintenance of unfolded protein involved in	GO:0071818|BAT3 complex;GO:0005829|cytosol;GO:0016020|membrane;GO:0005654|nucleoplasm;GO:0005634|nucleus;	GO:0031593|polyubiquitin binding;GO:0070628|proteasome	.	.
NDUFB9	919.239	-0.47961	0.133277953	-3.59854	0.00032	0.005883	Not_Sig	no	gi|741949800|ref|XP_005215409.2|	1021	5e-138	PREDICTED: NADH dehydrogenase [ubiquinone] 1 beta subcomplex subunit 9 isoform X1 [Bos taurus]	gi|942097777|ref|XM_014483000.1|	797	0	PREDICTED: Bos mutus NADH dehydrogenase (ubiquinone) 1 beta subcomplex, 9, 22kDa (NDUFB9), transcript variant X1, mRNA	Q02369	290	2e-97	NADH dehydrogenase [ubiquinone] 1 beta subcomplex subunit 9	.	.	.	.	pfam05347	Complex1_LYR	Complex 1 protein (LYR family). Proteins in this family include an accessory subunit of the higher eukaryotic NADH dehydrogenase	GO:0006120|mitochondrial electron transport, NADH to ubiquinone;GO:0098779|mitophagy in response to mitochondrial depolarization;	GO:0070062|extracellular exosome;GO:0005747|mitochondrial respiratory chain complex I;	GO:0008137|NADH dehydrogenase (ubiquinone) activity;	K03965	NDUFB9; NADH dehydrogenase (ubiquinone) 1 beta subcomplex subunit
E2F2	70.04628	0.846301	0.23539819	3.595189	0.0003242	0.00595	UP	yes	gi|742228914|ref|XP_010828336.1|	1802	0	PREDICTED: transcription factor E2F2 [Bison bison bison]	gi|965799067|ref|XM_012115402.2|	4016	0	PREDICTED: Ovis aries musimon E2F transcription factor 2 (E2F2), transcript variant X2, mRNA	Q14209	613	0	Transcription factor E2F2	.	.	.	.	pfam02319	E2F_TDP	E2F/DP family winged-helix DNA-binding domain. This family contains the transcription factor E2F and its dimerization partners	GO:0007049|cell cycle;GO:0072332|intrinsic apoptotic signaling pathway by p53 class mediator;GO:1990086|lens fiber cell apoptotic process;GO:0051726|regulation of cell cycle;GO:0006367|transcription initiation from RNA polymerase II promoter;	GO:0005654|nucleoplasm;GO:0005667|transcription factor complex;	GO:0001047|core promoter binding;GO:0003677|DNA	K09389	E2F2; transcription factor E2F2
HMGA1	656.9133	0.663068	0.184465952	3.59453	0.000325	0.005955	UP	yes	gi|795605332|ref|XP_011727672.1|	237	1e-18	PREDICTED: uncharacterized protein LOC105474575 [Macaca nemestrina]	gi|115496174|ref|NM_001076523.1|	1806	0	Bos taurus high mobility group AT-hook 1 (HMGA1), mRNA	.	.	.	.	.	.	.	.	pfam14655	RAB3GAP2_N	Rab3 GTPase-activating protein regulatory subunit N-terminus. This family includes the N-terminus of the Rab3 GTPase-	.	.	.	.	.
UHMK1	1243.461	-0.51926	0.144544733	-3.59235	0.0003277	0.005996	Not_Sig	no	gi|8393668|ref|NP_058989.1|	1972	0	serine/threonine-protein kinase Kist [Rattus norvegicus]	gi|742181372|ref|XM_010854673.1|	8110	0	PREDICTED: Bison bison bison U2AF homology motif (UHM) kinase 1 (UHMK1), mRNA	Q63285	764	0	Serine/threonine-protein kinase Kist	SPAC1687.15	79	5.25e-14	COG0515	pfam00069	Pkinase	Protein kinase domain.	GO:0000184|nuclear-transcribed mRNA catabolic process, nonsense-mediated decay;GO:0018105|peptidyl-serine phosphorylation;GO:0000381|regulation of alternative mRNA splicing, via spliceosome;GO:0046825|regulation of protein export from nucleus;	GO:0005737|cytoplasm;GO:0035145|exon-exon junction complex;GO:0005634|nucleus;GO:0035770|ribonucleoprotein granule;	GO:0005524|ATP binding;GO:0019899|enzyme binding;GO:0003729	K08877	UHMK1, KIS; U2AF homology motif (UHM) kinase 1 [EC:2.7.11.1]
MTSS2	493.784	0.612376	0.170868246	3.583905	0.0003385	0.006183	UP	yes	gi|982998858|ref|XP_015322749.1|	2750	0	PREDICTED: LOW QUALITY PROTEIN: MTSS1-like protein isoform X2 [Bos taurus]	gi|982998857|ref|XM_015467263.1|	3324	0	PREDICTED: Bos taurus metastasis suppressor 1-like (MTSS1L), transcript variant X2, mRNA	Q765P7	1011	0	MTSS1-like protein	.	.	.	.	pfam08397	IMD	IRSp53/MIM homology domain. The N-terminal predicted helical stretch of the insulin receptor tyrosine kinase substrate p53	GO:0097581|lamellipodium organization;GO:0007009|plasma membrane organization;	GO:0030864|cortical actin cytoskeleton;GO:0030027|lamellipodium;GO:0005886|plasma membrane;GO:0001726|ruffle;	GO:0005546|phosphatidylinositol-4,5-bisphosphate binding;	.	.
MEPCE	689.6549	0.436884	0.121928931	3.583101	0.0003395	0.006193	Not_Sig	no	gi|594044104|ref|XP_006046555.1|	2422	0	PREDICTED: 7SK snRNA methylphosphate capping enzyme [Bubalus bubalis]	gi|594044103|ref|XM_006046493.1|	3617	0	PREDICTED: Bubalus bubalis methylphosphate capping enzyme (MEPCE), mRNA	Q7L2J0	543	7e-176	7SK snRNA methylphosphate capping enzyme	.	.	.	.	pfam06859	Bin3	Bicoid-interacting protein 3 (Bin3). This family represents a conserved region of approximately 120 residues within eukaryotic Bicoid-	GO:0035562|negative regulation of chromatin binding;GO:0000122|negative regulation of transcription from RNA polymerase II promoter;GO:1900087|positive regulation of G1/S transition of mitotic cell cycle;GO:0001510|RNA methylation;GO:0016073|snRNA metabolic process;GO:0040031|snRNA modification;	.	GO:0044822|poly(A) RNA binding;GO:0008173|RNA	K15190	MEPCE, BCDIN3; 7SK snRNA methylphosphate capping enzyme
ENY2	933.7019	-0.50524	0.141172678	-3.57888	0.0003451	0.006274	Not_Sig	no	gi|148676796|gb|EDL08743.1|	628	3e-76	mCG145920, isoform CRA_b, partial [Mus musculus]	gi|742216922|ref|XM_010863453.1|	1506	0	PREDICTED: Bison bison bison enhancer of yellow 2 homolog (Drosophila) (ENY2), transcript variant X1, mRNA	Q9JIX0	182	1e-53	Transcription and mRNA export factor ENY2	.	.	.	.	pfam10163	EnY2	Transcription factor e(y)2. EnY2 is a small transcription factor which is combined in a complex with the TAFII40 protein. The protein is	GO:0016578|histone deubiquitination;GO:0061179|negative regulation of insulin secretion involved in cellular response to glucose stimulus;GO:0016973|poly(A)+ mRNA export from nucleus;GO:0045893|positive regulation of transcription, DNA-templated;GO:0015031|protein transport;GO:0006357|regulation of transcription from RNA polymerase II promoter;GO:0006368|transcription elongation from RNA polymerase II promoter;	GO:0071819|DUBm complex;GO:0005643|nuclear pore;GO:0000124|SAGA complex;GO:0070390|transcription export	GO:0003682|chromatin binding;GO:0030374|ligand-dependent	K11368	ENY2, DC6, SUS1; enhancer of yellow 2 transcription factor
PRKACB	468.8445	-0.46712	0.130542293	-3.57832	0.0003458	0.006274	Not_Sig	no	gi|27807059|ref|NP_777010.1|	1883	0	cAMP-dependent protein kinase catalytic subunit beta [Bos taurus]	gi|742204026|ref|XM_010860295.1|	4342	0	PREDICTED: Bison bison bison protein kinase, cAMP-dependent, catalytic, beta (PRKACB), transcript variant X2, mRNA	P05131	729	0	cAMP-dependent protein kinase catalytic subunit beta	.	.	.	.	pfam00069	Pkinase	Protein kinase domain.	GO:1901621|negative regulation of smoothened signaling pathway involved in dorsal/ventral neural tube patterning;GO:0001843|neural tube closure;GO:0070613|regulation of protein processing;	GO:0005813|centrosome;GO:0097546|ciliary base;GO:0005737|cytoplasm;GO:0070062|extracellular	GO:0005524|ATP binding;GO:0004691|cAMP-dependent protein kinase	K04345	PKA; protein kinase A [EC:2.7.11.11]
PIEZO1	2460.979	0.503293	0.140655871	3.578187	0.000346	0.006274	Not_Sig	no	gi|803313346|ref|XP_012015002.1|	10845	0	PREDICTED: piezo-type mechanosensitive ion channel component 1 isoform X4 [Ovis aries musimon]	gi|965895460|ref|XM_012159612.2|	7091	0	PREDICTED: Ovis aries musimon piezo-type mechanosensitive ion channel component 1 (PIEZO1), transcript variant X4, mRNA	Q92508	3506	0	Piezo-type mechanosensitive ion channel component 1	.	.	.	.	pfam12166	DUF3595	Protein of unknown function (DUF3595). This family of proteins is functionally uncharacterized.This family of proteins is found in	GO:0006812|cation transport;GO:0033634|positive regulation of cell-cell adhesion mediated by integrin;GO:0033625|positive regulation of integrin activation;	GO:0005783|endoplasmic reticulum;GO:0005789|endoplasmic reticulum membrane;GO:0033116|endoplasmic	GO:0005261|cation channel activity;GO:0008381|mechanically-gated	.	.
ANOS1	320.6643	-0.53377	0.149197076	-3.57762	0.0003467	0.006274	Not_Sig	no	gi|529016240|ref|XP_005228592.1|	3196	0	PREDICTED: anosmin-1 [Bos taurus]	gi|965822690|ref|XM_012126975.2|	4471	0	PREDICTED: Ovis aries musimon anosmin 1 (ANOS1), transcript variant X2, mRNA	P23352	1022	0	Anosmin-1	.	.	.	.	pfam00095	WAP	WAP-type (Whey Acidic Protein) 'four-disulfide core'.	GO:0007411|axon guidance;GO:0007155|cell adhesion;GO:0006935|chemotaxis;GO:0008543|fibroblast growth factor receptor signaling pathway;GO:0006928|movement of cell or subcellular component;	GO:0005576|extracellular region;GO:0005615|extracellular space;GO:0005886|plasma membrane;GO:0005578|proteinaceous	GO:0005201|extracellular matrix structural constituent;GO:000	.	.
GPAA1	1063.098	0.561769	0.157023475	3.577612	0.0003467	0.006274	Not_Sig	no	gi|528975107|ref|XP_005215236.1|	2900	0	PREDICTED: glycosylphosphatidylinositol anchor attachment 1 protein isoform X1 [Bos taurus]	gi|528975106|ref|XM_005215179.1|	1995	0	PREDICTED: Bos taurus glycosylphosphatidylinositol anchor attachment 1 (GPAA1), transcript variant X1, mRNA	O43292	1006	0	Glycosylphosphatidylinositol anchor attachment 1 protein	.	.	.	.	pfam04114	Gaa1	Gaa1-like, GPI transamidase component. GPI (glycosyl phosphatidyl inositol) transamidase is a multi-protein	GO:0016255|attachment of GPI anchor to protein;GO:0006461|protein complex assembly;GO:0006621|protein retention in ER lumen;	GO:0005789|endoplasmic reticulum membrane;GO:0042765|GPI-anchor transamidase complex;GO:0016020|membrane;	GO:0015631|tubulin binding;	K05289	GAA1; glycosylphosphatidyl inositol transamidase
TLN1	13540.37	0.342427	0.095753886	3.57612	0.0003487	0.0063	Not_Sig	no	gi|528958805|ref|XP_005210183.1|	12223	0	PREDICTED: talin-1 isoform X1 [Bos taurus]	gi|741935880|ref|XM_005210126.2|	8338	0	PREDICTED: Bos taurus talin 1 (TLN1), transcript variant X1, mRNA	Q9Y490	4629	0	Talin-1	.	.	.	.	pfam09141	Talin_middle	Talin, middle domain. Members of this family adopt a structure consisting of five alpha helices that fold into a bundle. They contain a	GO:0007043|cell-cell junction assembly;GO:0007044|cell-substrate junction assembly;GO:0030866|cortical actin cytoskeleton organization;GO:0007016|cytoskeletal anchoring at plasma membrane;GO:0036498|IRE1-mediated unfolded protein response;GO:0006928|movement of cell or subcellular component;GO:0006936|muscle contraction;GO:0070527|platelet aggregation;GO:0002576|platelet degranulation;GO:0016032|viral process;	GO:0009986|cell surface;GO:0005913|cell-cell adherens junction;GO:0005911|cell-cell junction;GO:0005737|cytoplasm;GO:0005829|cytosol;GO:0070062|extracellular	GO:0098641|cadherin binding involved in cell-cell adhesion;GO:00051	K06271	TLN; talin
NCDN	500.2254	0.497461	0.13915402	3.574896	0.0003504	0.00632	Not_Sig	no	gi|926689277|ref|XP_013818452.1|	3637	0	PREDICTED: neurochondrin [Capra hircus]	gi|982919892|ref|XM_005204670.3|	3873	0	PREDICTED: Bos taurus neurochondrin (NCDN), transcript variant X3, mRNA	Q2KJ97	1345	0	Neurochondrin	.	.	.	.	pfam05536	Neurochondrin	Neurochondrin. This family contains several eukaryotic neurochondrin proteins. Neurochondrin induces	GO:0045453|bone resorption;GO:0031175|neuron projection development;	GO:0005829|cytosol;GO:0030425|dendrite;GO:0016020|membrane;GO:0043025|neuronal cell body;	.	.	.
NDUFB8	1018.485	-0.51458	0.143975258	-3.57408	0.0003515	0.00633	Not_Sig	no	gi|27807203|ref|NP_777092.1|	989	1e-133	NADH dehydrogenase [ubiquinone] 1 beta subcomplex subunit 8, mitochondrial precursor [Bos taurus]	gi|942033780|ref|XM_005888109.2|	723	0	PREDICTED: Bos mutus NADH dehydrogenase (ubiquinone) 1 beta subcomplex, 8, 19kDa (NDUFB8), transcript variant X1, mRNA	Q02372	385	4e-135	NADH dehydrogenase [ubiquinone] 1 beta subcomplex subunit 8, mitochondrial	.	.	.	.	pfam05821	NDUF_B8	NADH-ubiquinone oxidoreductase ASHI subunit (CI-ASHI or NDUFB8). This family consists of several eukaryotic NADH-ubiquinone	GO:0006120|mitochondrial electron transport, NADH to ubiquinone;	GO:0016021|integral component of membrane;GO:0005747|mitochondrial respiratory chain complex I;	GO:0008137|NADH dehydrogenase (ubiquinone) activity;	K03964	NDUFB8; NADH dehydrogenase (ubiquinone) 1 beta subcomplex subunit
LOC138985550	1683.966	0.582659	0.163063506	3.5732	0.0003526	0.006341	Not_Sig	no	gi|741901914|ref|XP_010823966.1|	348	4e-35	PREDICTED: serine/arginine repetitive matrix protein 2 isoform X12 [Bos taurus]	gi|983006833|ref|XM_010825660.2|	1290	0	PREDICTED: Bos taurus serine/arginine repetitive matrix 2 (SRRM2), transcript variant X13, mRNA	Q8BTI8	55.5	2e-08	Serine/arginine repetitive matrix protein 2	.	.	.	.	pfam01093	Clusterin	Clusterin.	GO:0006397|mRNA processing;GO:0008380|RNA splicing;	GO:0015030|Cajal body;GO:0071013|catalytic step 2 spliceosome;GO:0016607|nuclear speck;	GO:0044822|poly(A) RNA binding;	K13172	SRRM2, SRM300; serine/arginine repetitive matrix protein 2
ZNF335	542.9597	0.590789	0.165373209	3.572461	0.0003536	0.006349	UP	yes	gi|440904616|gb|ELR55102.1|	5708	0	Zinc finger protein 335, partial [Bos mutus]	gi|982943159|ref|XM_005214795.3|	4179	0	PREDICTED: Bos taurus zinc finger protein 335 (ZNF335), transcript variant X4, mRNA	Q9H4Z2	1955	0	Zinc finger protein 335	ECU03g0790	64.7	9.87e-11	COG5048	pfam13465	zf-H2C2_2	Zinc-finger double domain.	GO:0007420|brain development;GO:0048854|brain morphogenesis;GO:0021895|cerebral cortex neuron differentiation;GO:0080182|histone H3-K4 trimethylation;GO:0001701|in utero embryonic development;GO:0048812|neuron projection morphogenesis;GO:0050671|positive regulation of lymphocyte proliferation;GO:0002052|positive regulation of neuroblast proliferation;GO:0050769|positive regulation of neurogenesis;GO:0040029|regulation of gene expression, epigenetic;GO:0051569|regulation of histone H3-K4	GO:0035097|histone methyltransferase complex;GO:0005634|nucleus;	GO:0046872|metal ion binding;GO:0000979|RNA polymerase II	.	.
POLRMT	433.9551	0.523003	0.146528347	3.569297	0.0003579	0.006413	Not_Sig	no	gi|741933370|ref|XP_010805524.1|	6004	0	PREDICTED: DNA-directed RNA polymerase, mitochondrial isoform X1 [Bos taurus]	gi|982931711|ref|XM_010807222.2|	3717	0	PREDICTED: Bos taurus polymerase (RNA) mitochondrial (DNA directed) (POLRMT), transcript variant X1, mRNA	O00411	1552	0	DNA-directed RNA polymerase, mitochondrial	YFL036w	455	2.06e-137	COG5108	pfam00940	RNA_pol	DNA-dependent RNA polymerase. This is a family of single chain RNA polymerases.	GO:0007005|mitochondrion organization;GO:0006390|transcription from mitochondrial promoter;GO:0006391|transcription initiation from mitochondrial promoter;	GO:0005759|mitochondrial matrix;GO:0042645|mitochondrial nucleoid;GO:0005739|mitochondrion;	GO:0003677|DNA binding;GO:0003899|DNA-directed RNA polymerase	K10908	POLRMT, RPO41; DNA-directed RNA polymerase, mitochondrial
SPECC1L	920.4836	0.399256	0.111867896	3.569	0.0003583	0.006413	Not_Sig	no	gi|329663575|ref|NP_001192793.1|	4852	0	cytospin-A [Bos taurus]	gi|741957497|ref|XM_005218227.2|	4991	0	PREDICTED: Bos taurus sperm antigen with calponin homology and coiled-coil domains 1-like (SPECC1L), transcript variant X2, mRNA	Q2KNA0	1781	0	Cytospin-A	ECU09g0290	102	3.60e-21	COG5069	pfam00307	CH	Calponin homology (CH) domain. The CH domain is found in both cytoskeletal proteins and signal transduction proteins. The CH	GO:0007155|cell adhesion;GO:0007049|cell cycle;GO:0051301|cell division;	GO:0005737|cytoplasm;GO:0005921|gap junction;GO:0005819|spindle;	.	.	.
PPP1R13L	440.0951	0.646757	0.181250668	3.568301	0.0003593	0.006421	UP	yes	gi|743703710|ref|XP_010945109.1|	2861	0	PREDICTED: LOW QUALITY PROTEIN: relA-associated inhibitor [Camelus bactrianus]	gi|982953397|ref|XM_005219199.3|	3241	0	PREDICTED: Bos taurus protein phosphatase 1 regulatory subunit 13 like (PPP1R13L), transcript variant X1, mRNA	Q5I1X5	1090	0	RelA-associated inhibitor	.	.	.	.	pfam12796	Ank_2	Ankyrin repeats (3 copies).	GO:0006915|apoptotic process;GO:0060048|cardiac muscle contraction;GO:0003215|cardiac right ventricle morphogenesis;GO:0031076|embryonic camera-type eye development;GO:0042633|hair cycle;GO:0035264|multicellular organism growth;GO:0048871|multicellular organismal homeostasis;GO:0000122|negative regulation of transcription from RNA polymerase II promoter;GO:0045597|positive regulation of cell differentiation;GO:0009791|post-embryonic development;GO:0006351|transcription, DNA-	GO:0030054|cell junction;GO:0005913|cell-cell adherens junction;GO:0005737|cytoplasm;GO:0045171|intercellular bridge;GO:0005634|nucleus;	GO:0098641|cadherin binding involved in cell-cell adhesion;GO:00428	.	.
PURA	428.9078	-0.47264	0.132528591	-3.5663	0.0003621	0.006454	Not_Sig	no	gi|635124099|ref|XP_008012848.1|	1310	1e-158	PREDICTED: transcriptional activator protein Pur-alpha isoform X1 [Chlorocebus sabaeus]	gi|982932142|ref|XM_010807371.2|	5378	0	PREDICTED: Bos taurus purine-rich element binding protein A (PURA), transcript variant X2, mRNA	Q00577	489	2e-154	Transcriptional activator protein Pur-alpha	.	.	.	.	pfam04845	PurA	PurA ssDNA and RNA-binding protein. This family represents most of the length of the protein.	GO:0030154|cell differentiation;GO:0006270|DNA replication initiation;GO:0006268|DNA unwinding involved in DNA replication;GO:0045892|negative regulation of transcription, DNA-templated;GO:0007399|nervous system development;GO:0008284|positive regulation of cell proliferation;GO:0006351|transcription, DNA-templated;	GO:0005737|cytoplasm;GO:0030425|dendrite;GO:0005662|DNA replication factor A complex;GO:0043025|neuronal cell body;GO:0000784|nuclear chromosome,	GO:0003691|double-stranded telomeric DNA binding;GO:0044822	.	.
DRAM2	558.9737	-0.49274	0.13817928	-3.56592	0.0003626	0.006454	Not_Sig	no	gi|555993737|ref|XP_005908625.1|	1332	1e-171	PREDICTED: DNA damage-regulated autophagy modulator protein 2 [Bos mutus]	gi|742232201|ref|XM_010830854.1|	3437	0	PREDICTED: Bison bison bison DNA-damage regulated autophagy modulator 2 (DRAM2), transcript variant X1, mRNA	Q3ZC48	517	4e-173	DNA damage-regulated autophagy modulator protein 2	.	.	.	.	pfam10277	Frag1	Frag1/DRAM/Sfk1 family. This family includes Frag1, DRAM and Sfk1 proteins. Frag1 (FGF receptor activating protein 1) is a protein	GO:0006915|apoptotic process;GO:0006914|autophagy;GO:0045494|photoreceptor cell maintenance;GO:0010506|regulation of autophagy;GO:0007601|visual perception;	GO:0016324|apical plasma membrane;GO:0005794|Golgi apparatus;GO:0016021|integral component of membrane;GO:0005765|lysosomal	.	.	.
GNAI2	5412.313	0.405703	0.113779044	3.565713	0.0003629	0.006454	Not_Sig	no	gi|472359423|ref|XP_004399328.1|	1955	0	PREDICTED: guanine nucleotide-binding protein G(i) subunit alpha-2 isoform X1 [Odobenus rosmarus divergens]	gi|198282134|ref|NM_001101040.1|	2139	0	Bos taurus guanine nucleotide binding protein (G protein), alpha inhibiting activity polypeptide 2 (GNAI2), mRNA	P04899	734	0	Guanine nucleotide-binding protein G(i) subunit alpha-2	.	.	.	.	pfam00503	G-alpha	G-protein alpha subunit. G proteins couple receptors of extracellular signals to intracellular signaling pathways. The G protein	GO:0000186|activation of MAPKK activity;GO:0001973|adenosine receptor signaling pathway;GO:0007193|adenylate cyclase-inhibiting G-protein coupled receptor signaling pathway;GO:0007049|cell cycle;GO:0051301|cell division;GO:0008283|cell proliferation;GO:0007213|G-protein coupled acetylcholine receptor signaling pathway;GO:0007186|G-protein coupled receptor signaling pathway;GO:0007214|gamma-aminobutyric acid signaling pathway;GO:0035556|intracellular signal transduction;GO:0007194|negative regulation of adenylate cyclase	GO:0044297|cell body;GO:0005813|centrosome;GO:0005737|cytoplasm;GO:0005829|cytosol;GO:0030425|dendrite;GO:0070062|extracellular	GO:0031683|G-protein beta/gamma-subunit complex	K04630	GNAI; guanine nucleotide-binding protein G(i) subunit alpha
TAPBP	3734.432	0.481059	0.134952287	3.564663	0.0003643	0.00647	Not_Sig	no	gi|555958688|ref|XP_005891460.1|	1832	0	PREDICTED: tapasin [Bos mutus]	gi|942042432|ref|XM_005891398.2|	2505	0	PREDICTED: Bos mutus TAP binding protein (tapasin) (TAPBP), mRNA	O15533	583	0	Tapasin	.	.	.	.	pfam07654	C1-set	Immunoglobulin C1-set domain.	GO:0019885|antigen processing and presentation of endogenous peptide antigen via MHC class I;GO:0002474|antigen processing and presentation of peptide antigen via MHC class I;GO:0006955|immune response;GO:0050823|peptide antigen stabilization;GO:0006461|protein complex assembly;GO:0010468|regulation of gene expression;GO:0061635|regulation of protein complex stability;GO:0006890|retrograde vesicle-mediated transport, Golgi to ER;	GO:0005783|endoplasmic reticulum;GO:0005789|endoplasmic reticulum membrane;GO:0000139|Golgi membrane;GO:0071556|integral	GO:0042288|MHC class I protein binding;GO:0042605|peptide antigen	K08058	TAPBP; TAP binding protein (tapasin)
SLCO4A1	202.4857	0.657044	0.184411789	3.562916	0.0003668	0.006503	UP	yes	gi|982942984|ref|XP_015329623.1|	3772	0	PREDICTED: solute carrier organic anion transporter family member 4A1 isoform X2 [Bos taurus]	gi|742103965|ref|XM_010836594.1|	2972	0	PREDICTED: Bison bison bison solute carrier organic anion transporter family, member 4A1 (SLCO4A1), transcript variant X2, mRNA	Q8K078	988	0	Solute carrier organic anion transporter family member 4A1	.	.	.	.	pfam03137	OATP	Organic Anion Transporter Polypeptide (OATP) family. This family consists of several eukaryotic Organic-Anion-	GO:0015711|organic anion transport;GO:0043252|sodium-independent organic anion transport;GO:0070327|thyroid hormone transport;	GO:0005887|integral component of plasma membrane;	GO:0008514|organic anion transmembrane transporter	K14354	SLCO4A; solute carrier organic anion transporter family, member 4A
GOLT1B	1037.767	-0.51537	0.144740305	-3.56069	0.0003699	0.006549	Not_Sig	no	gi|560916836|ref|XP_006184663.1|	568	4e-63	PREDICTED: vesicle transport protein GOT1B [Camelus ferus]	gi|742186568|ref|XM_010855943.1|	2950	0	PREDICTED: Bison bison bison golgi transport 1B (GOLT1B), mRNA	Q9Y3E0	197	5e-57	Vesicle transport protein GOT1B	YMR292w	83.2	5.67e-18	COG5120	pfam04178	Got1	Got1/Sft2-like family. Traffic through the yeast Golgi complex depends on a member of the syntaxin family of SNARE proteins,	GO:0043123|positive regulation of I-kappaB kinase/NF-kappaB signaling;GO:0015031|protein transport;GO:0016192|vesicle-mediated transport;	GO:0005783|endoplasmic reticulum;GO:0000139|Golgi membrane;GO:0016021|integral component of	GO:0004871|signal transducer activity;	.	.
ATG9A	1126.329	0.50441	0.141704363	3.559597	0.0003714	0.006566	Not_Sig	no	gi|533163665|ref|XP_005395608.1|	3976	0	PREDICTED: autophagy-related protein 9A isoform X3 [Chinchilla lanigera]	gi|982914760|ref|XM_010802364.2|	3925	0	PREDICTED: Bos taurus autophagy related 9A (ATG9A), transcript variant X1, mRNA	Q3T904	1527	0	Autophagy-related protein 9A	.	.	.	.	pfam04109	APG9	Autophagy protein Apg9. In yeast, 15 Apg proteins coordinate the formation of autophagosomes. Autophagy is a bulk degradation	GO:0000045|autophagosome assembly;GO:0044805|late nucleophagy;GO:0000422|mitophagy;GO:0034727|piecemeal microautophagy of nucleus;GO:0034497|protein localization to pre-autophagosomal structure;GO:0015031|protein transport;	GO:0005776|autophagosome;GO:0000421|autophagosome membrane;GO:0031410|cytoplasmic vesicle;GO:0016021|integral component of	.	K17907	ATG9; autophagy-related protein 9
SOD1	920.5687	-0.58638	0.164770505	-3.55875	0.0003726	0.006566	DOWN	yes	gi|296481248|tpg|DAA23363.1|	921	2e-122	TPA: superoxide dismutase-like [Bos taurus]	gi|742212285|ref|XM_010862308.1|	859	0	PREDICTED: Bison bison bison superoxide dismutase 1, soluble (SOD1), mRNA	Q52RN5	306	1e-103	Superoxide dismutase [Cu-Zn]	YJR104c	161	5.04e-49	COG2032	pfam00080	Sod_Cu	Copper/zinc superoxide dismutase (SODC). superoxide dismutases (SODs) catalyse the conversion of superoxide radicals to hydrogen	GO:0000187|activation of MAPK activity;GO:0006309|apoptotic DNA fragmentation;GO:0060088|auditory receptor cell stereocilium organization;GO:0007569|cell aging;GO:0006879|cellular iron ion homeostasis;GO:0006302|double-strand break repair;GO:0007566|embryo implantation;GO:0006749|glutathione metabolic process;GO:0060047|heart contraction;GO:0050665|hydrogen peroxide biosynthetic process;GO:0007626|locomotory behavior;GO:0046716|muscle cell cellular homeostasis;GO:0002262|myeloid cell	GO:0005737|cytoplasm;GO:0031410|cytoplasmic vesicle;GO:0005829|cytosol;GO:0032839|dendrite	GO:0051087|chaperone binding;GO:0005507|copper ion	.	.
H1-10	1147.471	0.375152	0.105419606	3.558651	0.0003728	0.006566	Not_Sig	no	gi|744624355|ref|XP_010998493.1|	398	2e-42	PREDICTED: histone H1x [Camelus dromedarius]	gi|983004856|ref|XM_589328.9|	1366	0	PREDICTED: Bos taurus H1 histone family, member X (H1FX), mRNA	Q92522	150	9e-41	Histone H1x	.	.	.	.	pfam00538	Linker_histone	linker histone H1 and H5 family. Linker histone H1 is an essential component of chromatin structure. H1 links nucleosomes	GO:0006334|nucleosome assembly;	GO:0005913|cell-cell adherens junction;GO:0005730|nucleolus;GO:0000786|nucleosome;GO:0005634|nucleus;	GO:0098641|cadherin binding involved in cell-cell adhesion;GO:00036	K11275	H1_5; histone H1/5
NIPSNAP3A	274.3267	-0.51348	0.1443014	-3.55838	0.0003732	0.006566	Not_Sig	no	gi|555990181|ref|XP_005906888.1|	1266	5e-169	PREDICTED: protein NipSnap homolog 3A [Bos mutus]	gi|742138538|ref|XM_010843530.1|	1770	0	PREDICTED: Bison bison bison nipsnap homolog 3A (C. elegans) (NIPSNAP3A), mRNA	Q5RAA9	458	2e-157	Protein NipSnap homolog 3A	.	.	.	.	pfam07978	NIPSNAP	NIPSNAP. Members of this family include many hypothetical proteins. It also includes members of the NIPSNAP family which have	.	GO:0005829|cytosol;GO:0005634|nucleus;	.	.	.
EMC1	3053.511	0.353288	0.099305237	3.557594	0.0003743	0.006575	Not_Sig	no	gi|741873830|ref|XP_005197700.2|	5177	0	PREDICTED: ER membrane protein complex subunit 1 isoform X2 [Bos taurus]	gi|742228547|ref|XM_010829936.1|	4404	0	PREDICTED: Bison bison bison ER membrane protein complex subunit 1 (EMC1), transcript variant X2, mRNA	Q8N766	1898	0	ER membrane protein complex subunit 1	.	.	.	.	pfam07774	DUF1620	Protein of unknown function (DUF1620). These sequences are mainly derived from predicted eukaryotic proteins. The region in	GO:0034975|protein folding in endoplasmic reticulum;	GO:0072546|ER membrane protein complex;GO:0016021|integral component of membrane;	.	.	.
MTA1	1067.533	0.522694	0.146953915	3.556857	0.0003753	0.006582	Not_Sig	no	gi|594037505|ref|XP_006043385.1|	3054	0	PREDICTED: metastasis-associated protein MTA1 isoform X1 [Bubalus bubalis]	gi|594037504|ref|XM_006043323.1|	2467	0	PREDICTED: Bubalus bubalis metastasis associated 1 (MTA1), transcript variant X1, mRNA	Q8K4B0	1143	0	Metastasis-associated protein MTA1	.	.	.	.	pfam01448	ELM2	ELM2 domain. The ELM2 (Egl-27 and MTA1 homology 2) domain is a small domain of unknown function. It is found in the MTA1	GO:0032922|circadian regulation of gene expression;GO:0006302|double-strand break repair;GO:0043153|entrainment of circadian clock by photoperiod;GO:0045475|locomotor rhythm;GO:1902499|positive regulation of protein autoubiquitination;GO:0043161|proteasome-mediated ubiquitin-dependent protein catabolic process;GO:0040029|regulation of gene expression, epigenetic;GO:0050727|regulation of inflammatory response;GO:0010212|response to ionizing radiation;GO:0032496|response to lipopolysaccharide;GO:0006351|transcription,	GO:0005737|cytoplasm;GO:0043231|intracellular membrane-bounded organelle;GO:0005874|microtubule;GO:0005635|nuclear	GO:0003682|chromatin binding;GO:0001047|core promoter	.	.
LRP10	2233.733	0.533509	0.150029449	3.556029	0.0003765	0.006582	Not_Sig	no	gi|440906317|gb|ELR56593.1|	3008	0	Low-density lipoprotein receptor-related protein 10, partial [Bos mutus]	gi|198282008|ref|NM_001100371.1|	3086	0	Bos taurus LDL receptor related protein 10 (LRP10), mRNA	Q7Z4F1	1011	0	Low-density lipoprotein receptor-related protein 10	.	.	.	.	pfam00057	Ldl_recept_a	Low-density lipoprotein receptor domain class A.	GO:0048839|inner ear development;GO:0006629|lipid metabolic process;GO:0006869|lipid transport;	GO:0005905|coated pit;GO:0016021|integral component of membrane;GO:0016020|membrane;	GO:0005041|low-density lipoprotein receptor activity;	.	.
IGF2R	4332.417	0.365878	0.102892541	3.555925	0.0003767	0.006582	Not_Sig	no	gi|27805933|ref|NP_776777.1|	12603	0	cation-independent mannose-6-phosphate receptor precursor [Bos taurus]	gi|31342245|ref|NM_174352.2|	8820	0	Bos taurus insulin like growth factor 2 receptor (IGF2R), mRNA	P08169	4858	0	Cation-independent mannose-6-phosphate receptor	.	.	.	.	pfam00878	CIMR	Cation-independent mannose-6-phosphate receptor repeat. The cation-independent mannose-6-phosphate receptor contains 15	.	GO:0009986|cell surface;GO:0030139|endocytic vesicle;GO:0005794|Golgi apparatus;GO:0016021|integral component	GO:0001948|glycoprotein binding;GO:0005520|insulin-like growth	K06564	IGF2R; insulin-like growth factor 2 receptor
LOC106700748	732.9537	-0.39277	0.110460741	-3.55573	0.0003769	0.006582	Not_Sig	no	.	.	.	.	gi|982995086|ref|XR_803850.2|	3290	0	PREDICTED: Bos taurus uncharacterized LOC100848689 (LOC100848689), transcript variant X1, ncRNA	.	.	.	.	.	.	.	.	pfam08131	Defensin_3	Defensin-like peptide family. This family consists of the defensin-like peptides (DLPs) isolated from platypus venom. These DLPs show	.	.	.	.	.
TXNDC17	378.5557	-0.59998	0.168755177	-3.55533	0.0003775	0.006582	DOWN	yes	gi|594072479|ref|XP_006060073.1|	589	1e-74	PREDICTED: thioredoxin domain-containing protein 17 [Bubalus bubalis]	gi|942087640|ref|XM_005906661.2|	600	0	PREDICTED: Bos mutus thioredoxin domain containing 17 (TXNDC17), mRNA	Q9BRA2	213	6e-69	Thioredoxin domain-containing protein 17	.	.	.	.	pfam06110	DUF953	Eukaryotic protein of unknown function (DUF953). This family consists of several hypothetical eukaryotic proteins of unknown	GO:0033209|tumor necrosis factor-mediated signaling pathway;	GO:0005829|cytosol;GO:0070062|extracellular exosome;	GO:0004601|peroxidase activity;GO:0047134|protein-disulfide	.	.
TMEM65	810.001	-0.50739	0.142761941	-3.5541	0.0003793	0.006603	Not_Sig	no	gi|545520750|ref|XP_005627967.1|	771	1e-88	PREDICTED: transmembrane protein 65 isoform X1 [Canis lupus familiaris]	gi|965835243|ref|XM_012133232.2|	3369	0	PREDICTED: Ovis aries musimon transmembrane protein 65 (TMEM65), transcript variant X2, mRNA	Q0VCH8	128	8e-31	Transmembrane protein 65	.	.	.	.	pfam10507	DUF2453	Protein of unknown function (DUF2453). Some members of this family are purported to contain GAF domains but this could not be	GO:0003231|cardiac ventricle development;GO:1903779|regulation of cardiac conduction;	GO:0016021|integral component of membrane;GO:0014704|intercalated disc;GO:0005739|mitochondrion;GO:0005886|plasma membrane;	.	.	.
GFPT1	716.6199	-0.51553	0.145070058	-3.55368	0.0003799	0.006603	Not_Sig	no	gi|594049518|ref|XP_006049134.1|	3696	0	PREDICTED: glutamine--fructose-6-phosphate aminotransferase [isomerizing] 1 isoform X1 [Bubalus bubalis]	gi|742148552|ref|XM_010845415.1|	6085	0	PREDICTED: Bison bison bison glutamine--fructose-6-phosphate transaminase 1 (GFPT1), mRNA	Q06210	1424	0	Glutamine--fructose-6-phosphate aminotransferase [isomerizing] 1	YKL104c	771	0	COG0449	pfam00310	GATase_2	Glutamine amidotransferases class-II.	GO:0005975|carbohydrate metabolic process;GO:0032922|circadian regulation of gene expression;GO:0006112|energy reserve metabolic process;GO:0006002|fructose 6-phosphate metabolic process;GO:0006541|glutamine metabolic process;GO:0036498|IRE1-mediated unfolded protein response;GO:0006048|UDP-N-acetylglucosamine biosynthetic process;GO:0006047|UDP-N-acetylglucosamine metabolic process;	GO:0005829|cytosol;GO:0070062|extracellular exosome;	GO:0030246|carbohydrate binding;GO:0004360|glutamine-	K00820	glmS, GFPT; glucosamine--fructose-6-phosphate
SNRPB2	546.707	-0.51653	0.145433762	-3.55166	0.0003828	0.00663	Not_Sig	no	gi|440893400|gb|ELR46188.1|	1133	2e-153	U2 small nuclear ribonucleoprotein B'', partial [Bos mutus]	gi|982942072|ref|XM_005200943.3|	979	0	PREDICTED: Bos taurus small nuclear ribonucleoprotein polypeptide B (SNRPB2), transcript variant X1, mRNA	P08579	418	2e-146	U2 small nuclear ribonucleoprotein B''	.	.	.	.	pfam00076	RRM_1	RNA recognition motif. (a.k.a. RRM, RBD, or RNP domain). The RRM motif is probably diagnostic of an RNA binding protein. RRMs	GO:0000398|mRNA splicing, via spliceosome;	GO:0071013|catalytic step 2 spliceosome;GO:0005730|nucleolus;GO:0005654|nucleoplasm;GO:0005681|spliceosomal complex;GO:0005686|U2 snRNP;	GO:0000166|nucleotide binding;GO:0035614|snRNA stem-loop	K11094	SNRPB2; U2 small nuclear ribonucleoprotein B''

HILPDA	1471.41	-0.37944	0.10685178	-3.55107	0.0003837	0.00663	Not_Sig	no	gi|4239700|emb|CAA10770.1|	437	4e-43	reverse transcriptase-like [Bos taurus]	gi|850481938|gb|CP011889.1|	1832	0	Ovis canadensis canadensis isolate 43U chromosome 4 sequence	Q9Y5L2	92.4	5e-20	Hypoxia-inducible lipid droplet-associated protein	.	.	.	.	pfam15220	HILPDA	Hypoxia-inducible lipid droplet-associated. This family of proteins stimulate intracellular lipid accumulation, function as	GO:0035425|autocrine signaling;GO:0008284|positive regulation of cell proliferation;GO:0001819|positive regulation of cytokine production;GO:0010884|positive regulation of lipid storage;GO:0006950|response to stress;	GO:0009986|cell surface;GO:0005615|extracellular space;GO:0016021|integral component of membrane;GO:0005811|lipid	GO:0005102|receptor binding;	.	.
PSMB6	905.1746	-0.43355	0.122097463	-3.55084	0.000384	0.00663	Not_Sig	no	gi|594072652|ref|XP_006060156.1|	983	1e-131	PREDICTED: proteasome subunit beta type-6 isoform X2 [Bubalus bubalis]	gi|742155601|ref|XM_010847390.1|	831	0	PREDICTED: Bison bison bison proteasome (prosome, macropain) subunit, beta type, 6 (PSMB6), mRNA	Q3MHN0	349	2e-119	Proteasome subunit beta type-6	.	.	.	.	pfam00227	Proteasome	Proteasome subunit. The proteasome is a multisubunit structure that degrades proteins. Protein degradation is an essential	GO:0051603|proteolysis involved in cellular protein catabolic process;	GO:0070062|extracellular exosome;GO:0005794|Golgi apparatus;GO:0005654|nucleoplasm;GO:0005839|proteasome core complex;	GO:0004298|threonine-type endopeptidase activity;	K02738	PSMB6; 20S proteasome subunit beta 1 [EC:3.4.25.1]
SGTA	1218.409	0.481653	0.135656609	3.550529	0.0003845	0.00663	Not_Sig	no	gi|594046940|ref|XP_006047901.1|	1842	0	PREDICTED: small glutamine-rich tetratricopeptide repeat-containing protein alpha isoform X1 [Bubalus bubalis]	gi|982931015|ref|XM_010807013.2|	2195	0	PREDICTED: Bos taurus small glutamine-rich tetratricopeptide repeat (TPR)-containing, alpha (SGTA), transcript variant X2, mRNA	Q32LM2	612	0	Small glutamine-rich tetratricopeptide repeat-containing protein alpha	SPAC17G6.19c	146	3.11e-38	COG0457	pfam13414	TPR_11	TPR repeat.	GO:1903071|positive regulation of ER-associated ubiquitin-dependent protein catabolic process;	GO:0005829|cytosol;GO:0016020|membrane;	.	K16365	SGTA; small glutamine-rich tetratricopeptide repeat-containing
STRN4	874.2969	0.561649	0.15819481	3.550361	0.0003847	0.00663	Not_Sig	no	gi|803246883|ref|XP_011983444.1|	2825	0	PREDICTED: striatin-4 isoform X2 [Ovis aries musimon]	gi|983000227|ref|XM_015467601.1|	2950	0	PREDICTED: Bos taurus striatin 4 (STRN4), transcript variant X1, mRNA	P58404	1016	0	Striatin-4	.	.	.	.	pfam08232	Striatin	Striatin family. Striatin is an intracellular protein which has a caveolin-binding motif, a coiled-coil structure, a calmodulin-	.	GO:0005737|cytoplasm;GO:0043197|dendritic spine;GO:0016020|membrane;GO:0000159|protein phosphatase type 2A complex;	GO:0070016|armadillo repeat domain binding;GO:0032403|protein complex	K17608	STRN1_3_4; striatin 1/3/4
GATAD2A	1379.961	0.485301	0.136705315	3.549976	0.0003853	0.00663	Not_Sig	no	gi|528954320|ref|XP_005208490.1|	2753	0	PREDICTED: transcriptional repressor p66-alpha isoform X1 [Bos taurus]	gi|982929461|ref|XM_005208434.3|	3680	0	PREDICTED: Bos taurus GATA zinc finger domain containing 2A (GATAD2A), transcript variant X4, mRNA	Q86YP4	961	0	Transcriptional repressor p66-alpha	.	.	.	.	pfam09770	PAT1	Topoisomerase II-associated protein PAT1. Members of this family are necessary for accurate chromosome transmission during	GO:0006306|DNA methylation;GO:0000122|negative regulation of transcription from RNA polymerase II promoter;GO:0045892|negative regulation of transcription, DNA-templated;GO:0006351|transcription, DNA-templated;	GO:0016607|nuclear speck;GO:0005654|nucleoplasm;GO:0005634|nucleus;GO:0016581|NuRD complex;	GO:0030674|protein binding, bridging;GO:0043565|sequence-specific	.	.
SF1	2534.685	0.423354	0.119262196	3.549775	0.0003856	0.00663	Not_Sig	no	gi|817296079|ref|XP_012318475.1|	2046	0	PREDICTED: splicing factor 1 isoform X7 [Aotus nancymaae]	gi|982972034|ref|XM_005227165.3|	3112	0	PREDICTED: Bos taurus splicing factor 1 (SF1), transcript variant X1, mRNA	Q64213	775	0	Splicing factor 1	YLR116w_1	219	6.39e-64	COG5176	pfam00013	KH_1	KH domain. KH motifs bind RNA in vitro. Autoantibodies to Nova, a KH domain protein, cause paraneoplastic opsoclonus ataxia.	GO:0033327|Leydig cell differentiation;GO:0030238|male sex determination;GO:0000398|mRNA splicing, via spliceosome;GO:0048662|negative regulation of smooth muscle cell proliferation;GO:0050810|regulation of steroid biosynthetic process;GO:0006355|regulation of transcription, DNA-templated;GO:0006351|transcription, DNA-templated;	GO:0005737|cytoplasm;GO:0005654|nucleoplasm;GO:0005634|nucleus;GO:0005681|spliceosomal complex;	GO:0044822|poly(A) RNA binding;GO:0045131|pre-mRNA branch	.	.
WDR44	408.818	-0.47811	0.134699478	-3.54946	0.000386	0.00663	Not_Sig	no	gi|555950439|ref|XP_005887422.1|	4296	0	PREDICTED: WD repeat-containing protein 44 isoform X1 [Bos mutus]	gi|966011793|ref|XM_004022399.3|	4828	0	PREDICTED: Ovis aries WD repeat domain 44 (WDR44), mRNA	Q9XSC3	1657	0	WD repeat-containing protein 44	.	.	.	.	pfam00400	WD40	WD domain, G-beta repeat.	.	GO:0005829|cytosol;GO:0010008|endosome membrane;GO:0005794|Golgi apparatus;GO:0048471|perinuclear region of cytoplasm;	.	.	.
PPP2R3A	827.3937	-0.43239	0.121866485	-3.54809	0.000388	0.006655	Not_Sig	no	gi|555955611|ref|XP_005889961.1|	5953	0	PREDICTED: serine/threonine-protein phosphatase 2A regulatory subunit B'' subunit alpha isoform X1 [Bos mutus]	gi|982976750|ref|XM_015462140.1|	6552	0	PREDICTED: Bos taurus protein phosphatase 2 regulatory subunit B'', alpha (PPP2R3A), transcript variant X4, mRNA	Q06190	2068	0	Serine/threonine-protein phosphatase 2A regulatory subunit B'' subunit alpha	.	.	.	.	pfam13499	EF-hand_7	EF-hand domain pair.	GO:0001754|eye photoreceptor cell differentiation;GO:0090090|negative regulation of canonical Wnt signaling pathway;GO:0090263|positive regulation of canonical Wnt signaling pathway;GO:0045732|positive regulation of protein catabolic process;GO:0006470|protein dephosphorylation;GO:0090249|regulation of cell motility involved in somitogenic axis elongation;GO:0007525|somatic muscle development;GO:0061053|somite development;GO:0090244|Wnt signaling pathway involved in somitogenesis;	GO:0000159|protein phosphatase type 2A complex;	GO:0005509|calcium ion binding;GO:0030674|protein binding,	K11583	PPP2R3; serine/threonine-protein phosphatase 2A regulatory
RSL24D1	540.5541	-0.51793	0.146006863	-3.54731	0.0003892	0.006664	Not_Sig	no	gi|122692367|ref|NP_001073824.1|	772	2e-98	probable ribosome biogenesis protein RLP24 [Bos taurus]	gi|742104646|ref|XM_010836765.1|	1224	0	PREDICTED: Bison bison bison ribosomal L24 domain containing 1 (RSL24D1), mRNA	Q3SZ12	301	5e-100	Probable ribosome biogenesis protein RLP24	YLR009w	152	2.55e-43	COG2075	pfam01246	Ribosomal_L24e	Ribosomal protein L24e.	GO:1902626|assembly of large subunit precursor of preribosome;GO:0000027|ribosomal large subunit assembly;GO:0006412|translation;	GO:0022625|cytosolic large ribosomal subunit;GO:0005730|nucleolus;	GO:0003735|structural constituent of ribosome;	K02896	RP-L24e, RPL24; large subunit ribosomal protein L24e
IER3IP1	2007.93	-0.5298	0.14937989	-3.54667	0.0003901	0.006671	Not_Sig	no	gi|7705819|ref|NP_057181.1|	414	2e-45	immediate early response 3-interacting protein 1 precursor [Homo sapiens]	gi|942092399|ref|XM_005908072.2|	854	0	PREDICTED: Bos mutus immediate early response 3 interacting protein 1 (IER3IP1), mRNA	Q9Y5U9	164	5e-47	Immediate early response 3-interacting protein 1	.	.	.	.	pfam08571	Yos1	Yos1-like. In yeast, Yos1 is a subunit of the Yip1p-Yif1p complex and is required for transport between the endoplasmic	GO:0006888|ER to Golgi vesicle-mediated transport;GO:2000269|regulation of fibroblast apoptotic process;	GO:0005783|endoplasmic reticulum;GO:0005789|endoplasmic reticulum membrane;GO:0030134|ER to Golgi transport vesicle;GO:0005794|Golgi	.	.	.
RPL21	4684.903	-0.50814	0.143304456	-3.54589	0.0003913	0.00668	Not_Sig	no	gi|440900636|gb|ELR51719.1|	845	6e-113	hypothetical protein M91_00440, partial [Bos mutus]	gi|942073883|ref|XM_005902186.2|	594	0	PREDICTED: Bos mutus ribosomal protein L21 (RPL21), mRNA	A5JSS2	330	2e-114	60S ribosomal protein L21	YBR191w	199	6.18e-65	COG2139	pfam01157	Ribosomal_L21e	Ribosomal protein L21e.	GO:0006412|translation;	GO:0005840|ribosome;	GO:0003735|structural constituent of ribosome;	K02889	RP-L21e, RPL21; large subunit ribosomal protein L21e
BEST1	12813.28	-0.55998	0.157985748	-3.54452	0.0003933	0.006705	Not_Sig	no	gi|742179141|ref|XP_010852332.1|	3153	0	PREDICTED: bestrophin-1 isoform X2 [Bison bison bison]	gi|982971704|ref|XM_015461234.1|	2927	0	PREDICTED: Bos taurus bestrophin 1 (BEST1), transcript variant X1, mRNA	O76090	979	0	Bestrophin-1	.	.	.	.	pfam01062	Bestrophin	Bestrophin, RFP-TM, chloride channel. Bestrophin is a 68-kDa basolateral plasma membrane protein expressed in retinal	GO:0006821|chloride transport;GO:0050908|detection of light stimulus involved in visual perception;GO:0034220|ion transmembrane transport;GO:0051924|regulation of calcium ion transport;GO:0030321|transepithelial chloride transport;GO:0007601|visual perception;	GO:0016323|basolateral plasma membrane;GO:0034707|chloride channel complex;GO:0005829|cytosol;GO:0016021|integral component of	GO:0015106|bicarbonate transmembrane transporter	K13878	BEST1, VMD2; bestrophin-1
HYCC1	958.2583	-0.44833	0.126508686	-3.54385	0.0003943	0.006711	Not_Sig	no	gi|297473673|ref|XP_002686759.1|	2609	0	PREDICTED: hyccin isoform X1 [Bos taurus]	gi|982980312|ref|XM_015462964.1|	5858	0	PREDICTED: Bos taurus family with sequence similarity 126 member A (FAM126A), transcript variant X1, mRNA	Q9BYI3	968	0	Hyccin	.	.	.	.	pfam09790	Hyccin	Hyccin. Members of this family of proteins may have a role in the beta-catenin-Tcf/Lef signaling pathway, as well as in the process	GO:0090002|establishment of protein localization to plasma membrane;GO:0042552|myelination;GO:0046854|phosphatidylinositol phosphorylation;	GO:0005737|cytoplasm;GO:0005829|cytosol;GO:0043005|neuron projection;GO:0005886|plasma membrane;	GO:0004871|signal transducer activity;	.	.
ACO2	2031.833	0.445145	0.125621842	3.543531	0.0003948	0.006711	Not_Sig	no	gi|958777144|ref|XP_014719872.1|	4118	0	PREDICTED: aconitate hydratase, mitochondrial [Equus asinus]	gi|942096618|ref|XM_005909434.2|	2756	0	PREDICTED: Bos mutus aconitase 2, mitochondrial (ACO2), mRNA	P20004	1584	0	Aconitate hydratase, mitochondrial	SPAC24C9.06c	1063	0	COG1048	pfam00330	Aconitase	Aconitase family (aconitate hydratase).	GO:0006101|citrate metabolic process;GO:0006099|tricarboxylic acid cycle;	GO:0005739|mitochondrion;GO:0043209|myelin sheath;GO:0005634|nucleus;	GO:0051539|4 iron, 4 sulfur cluster binding;GO:0003994|aconitate	K01681	ACO, acnA; aconitate hydratase [EC:4.2.1.3]
CDT1	202.6267	0.543948	0.153538777	3.542738	0.000396	0.006716	Not_Sig	no	gi|742140056|ref|XP_010842264.1|	2215	0	PREDICTED: DNA replication factor Cdt1 isoform X2 [Bison bison bison]	gi|594037143|ref|XM_006043150.1|	2105	0	PREDICTED: Bubalus bubalis chromatin licensing and DNA replication factor 1 (CDT1), transcript variant X1, mRNA	Q9H211	605	0	DNA replication factor Cdt1	.	.	.	.	pfam08839	CDT1	DNA replication factor CDT1 like. CDT1 is a component of the replication licensing system and promotes the loading of the mini-	GO:0006260|DNA replication;GO:0000076|DNA replication checkpoint;GO:0000082|G1/S transition of mitotic cell cycle;GO:0030174|regulation of DNA-dependent DNA replication initiation;GO:0033262|regulation of nuclear cell cycle DNA replication;GO:0000083|regulation of transcription involved in G1/S transition of mitotic cell cycle;	GO:0005829|cytosol;GO:0005654|nucleoplasm;GO:0005634|nucleus;	GO:0003677|DNA binding;	K10727	CDT1; chromatin licensing and DNA replication factor 1
SEC16A	1794.201	0.363899	0.102724594	3.542476	0.0003964	0.006716	Not_Sig	no	gi|194671785|ref|XP_597914.4|	4712	0	PREDICTED: protein transport protein Sec16A isoform X2 [Bos taurus]	gi|982991461|ref|XM_597914.8|	8259	0	PREDICTED: Bos taurus SEC16 homolog A, endoplasmic reticulum export factor (SEC16A), transcript variant X6, mRNA	O15027	1286	0	Protein transport protein Sec16A	.	.	.	.	pfam12931	Sec16_C	Sec23-binding domain of Sec16. Sec16 is a multi-domain vesicle coat protein. The C-terminal region is the part that binds to Sec23, a	GO:0048208|COPII vesicle coating;GO:0007029|endoplasmic reticulum organization;GO:0015031|protein transport;GO:0021762|substantia nigra development;	GO:0005737|cytoplasm;GO:0005829|cytosol;GO:0005789|endoplasmic reticulum membrane;GO:0005794|Golgi apparatus;GO:0000139|Golgi membrane;	.	.	.
PFDN5	563.3947	-0.53141	0.150024235	-3.54214	0.0003969	0.006716	Not_Sig	no	gi|641700813|ref|XP_008138969.1|	811	6e-107	PREDICTED: prefoldin subunit 5 isoform X1 [Eptesicus fuscus]	gi|555980545|ref|XM_005902135.1|	674	0	PREDICTED: Bos mutus prefoldin subunit 5 (PFDN5), transcript variant X1, mRNA	Q8HYI9	285	2e-96	Prefoldin subunit 5	SPBC215.02	121	4.15e-34	COG1730	pfam02996	Prefoldin	Prefoldin subunit. This family comprises of several prefoldin subunits. The biogenesis of the cytoskeletal proteins actin and	GO:0051086|chaperone mediated protein folding independent of cofactor;GO:0090090|negative regulation of canonical Wnt signaling pathway;GO:0045892|negative regulation of transcription, DNA-templated;GO:0006457|protein folding;GO:0006355|regulation of transcription, DNA-templated;GO:0060041|retina development in camera-type eye;	GO:0005737|cytoplasm;GO:0005829|cytosol;GO:0005634|nucleus;GO:0016272|prefoldin complex;	.	K04797	pfdA, PFDN5; prefoldin alpha subunit
TMEM208	279.4679	-0.56481	0.159504583	-3.54104	0.0003985	0.006734	Not_Sig	no	gi|795368607|ref|XP_011787456.1|	864	4e-114	PREDICTED: transmembrane protein 208 [Colobus angolensis palliatus]	gi|942056507|ref|XM_005896554.2|	774	0	PREDICTED: Bos mutus transmembrane protein 208 (TMEM208), mRNA	Q3SZZ5	326	9e-112	Transmembrane protein 208	.	.	.	.	pfam05620	DUF788	Protein of unknown function (DUF788). This family consists of several eukaryotic proteins of unknown function.	GO:0006914|autophagy;GO:0006624|vacuolar protein processing;	GO:0005789|endoplasmic reticulum membrane;GO:0016021|integral component of membrane;GO:0005773|vacuole;	.	.	.
AP3S1	703.8328	-0.49435	0.139641096	-3.54015	0.0003999	0.006747	Not_Sig	no	gi|826336802|ref|XP_012514992.1|	1034	1e-136	PREDICTED: AP-3 complex subunit sigma-1 [Propithecus coquereli]	gi|114053130|ref|NM_001046416.1|	1163	0	Bos taurus adaptor-related protein complex 3, sigma 1 subunit (AP3S1), mRNA	Q9DCR2	398	2e-137	AP-3 complex subunit sigma-1	SPAC30D11.05	191	6.63e-59	COG5030	pfam01217	Clat_adaptor_s	Clathrin adaptor complex small chain.	GO:0008089|anterograde axon cargo transport;GO:0048490|anterograde synaptic vesicle transport;GO:0006886|intracellular protein transport;GO:0016192|vesicle-mediated transport;	GO:0030123|AP-3 adaptor complex;GO:1904115|axon cytoplasm;GO:0030659|cytoplasmic vesicle membrane;GO:0043231|intracellular	GO:0008565|protein transporter activity;	K12399	AP3S; AP-3 complex subunit sigma
MICAL1	1259.58	0.385612	0.108957549	3.539107	0.0004015	0.006764	Not_Sig	no	gi|555988131|ref|XP_005905910.1|	5066	0	PREDICTED: protein-methionine sulfoxide oxidase MICAL1 isoform X2 [Bos mutus]	gi|942084943|ref|XM_005905847.2|	3527	0	PREDICTED: Bos mutus microtubule associated monooxygenase, calponin and LIM domain containing 1 (MICAL1), transcript variant X1, mRNA	F1MH07	1934	0	Protein-methionine sulfoxide oxidase MICAL1	ECU09g0290	86.3	2.16e-16	COG5069	pfam12130	DUF3585	Protein of unknown function (DUF3585). This domain is found in eukaryotes. This domain is typically between 135 and 149	GO:0030042|actin filament depolymerization;GO:0043154|negative regulation of cysteine-type endopeptidase activity involved in apoptotic process;GO:0001933|negative regulation of protein phosphorylation;GO:0055114|oxidation-reduction process;GO:0019417|sulfur oxidation;	GO:0005737|cytoplasm;GO:0005856|cytoskeleton;	GO:0003779|actin binding;GO:0071949|FAD binding;GO:0016709	.	.
SREBF2	2794.671	0.451022	0.127472529	3.538187	0.0004029	0.006778	Not_Sig	no	gi|329663519|ref|NP_001192529.1|	4874	0	sterol regulatory element-binding protein 2 [Bos taurus]	gi|329663518|ref|NM_001205600.1|	4149	0	Bos taurus sterol regulatory element binding transcription factor 2 (SREBF2), mRNA	Q12772	1763	0	Sterol regulatory element-binding protein 2	.	.	.	.	pfam00010	HLH	Helix-loop-helix DNA-binding domain.	GO:0071499|cellular response to laminar fluid shear stress;GO:0009267|cellular response to starvation;GO:0008203|cholesterol metabolic process;GO:0006629|lipid metabolic process;GO:0090370|negative regulation of cholesterol efflux;GO:0000122|negative regulation of transcription from RNA polymerase II promoter;GO:0010886|positive regulation of cholesterol storage;GO:1903955|positive regulation of protein targeting to mitochondrion;GO:0045944|positive regulation of transcription from RNA polymerase II promoter;GO:2000188|regulation	GO:0005737|cytoplasm;GO:0005829|cytosol;GO:0005783|endoplasmic reticulum;GO:0005789|endoplasmic reticulum membrane;GO:0012507|ER to	GO:0070888|E-box binding;GO:0008022|protein C-terminus binding;GO:0000978	K09107	SREBP2, SREBF2; sterol regulatory element-binding transcription factor 2
CDKN2AIPNL	297.434	-0.48315	0.13660463	-3.53684	0.000405	0.006802	Not_Sig	no	gi|528956641|ref|XP_005209396.1|	596	1e-73	PREDICTED: CDKN2AIP N-terminal-like protein isoform X1 [Bos taurus]	gi|741933581|ref|XM_005209339.2|	917	0	PREDICTED: Bos taurus CDKN2A interacting protein N-terminal like (CDKN2AIPNL), transcript variant X1, mRNA	Q24JY8	212	3e-67	CDKN2AIP N-terminal-like protein	.	.	.	.	pfam11952	DUF3469	Protein of unknown function (DUF3469). This family of proteins are functionally uncharacterized. This protein is found in eukaryotes.	GO:0006355|regulation of transcription, DNA-templated;GO:0007165|signal transduction;	GO:0005730|nucleolus;GO:0005654|nucleoplasm;	.	.	.
OSTC	1230.307	-0.42387	0.119870516	-3.53605	0.0004062	0.006813	Not_Sig	no	gi|440905686|gb|ELR56037.1|	845	2e-110	Oligosaccharyltransferase complex subunit OSTC, partial [Bos mutus]	gi|555968529|ref|XM_005896238.1|	1066	0	PREDICTED: Bos mutus oligosaccharyltransferase complex subunit (non-catalytic) (OSTC), transcript variant X1, mRNA	Q2KID7	301	9e-101	Oligosaccharyltransferase complex subunit OSTC	.	.	.	.	pfam04756	OST3_OST6	OST3 / OST6 family. The proteins in this family are part of a complex of eight ER proteins that transfers core oligosaccharide from dolichol	.	GO:0016021|integral component of membrane;GO:0008250|oligosaccharyltransferase complex;	.	.	.
ST8SIA4	125.691	-0.73652	0.208366889	-3.53474	0.0004082	0.006837	DOWN	yes	gi|752401102|ref|XP_002918806.2|	2064	0	PREDICTED: LOW QUALITY PROTEIN: CMP-N-acetylneuraminate-poly-alpha-2,8-sialyltransferase [Ailuropoda melanoleuca]	gi|742237546|ref|XM_010831966.1|	5326	0	PREDICTED: Bison bison bison ST8 alpha-N-acetyl-neuraminide alpha-2,8-sialyltransferase 4 (ST8SIA4), mRNA	Q6ZXC9	736	0	CMP-N-acetylneuraminate-poly-alpha-2,8-sialyltransferase	.	.	.	.	pfam00777	Glyco_transf_29	Glycosyltransferase family 29 (sialyltransferase). Members of this family belong to glycosyltransferase family 29.	GO:0006486|protein glycosylation;	GO:0030173|integral component of Golgi membrane;	GO:0003828|alpha-N-acetylneuraminate alpha-2,8-	K06614	SIAT8D; alpha-N-acetyl-neuraminate alpha-2,8-sialyltransferase
KRAS	764.1642	-0.62318	0.176326589	-3.53426	0.0004089	0.006839	DOWN	yes	gi|537135138|gb|ERE66116.1|	972	1e-117	GTPase KRas-like isoform 2 [Cricetulus griseus]	gi|742186758|ref|XM_010855996.1|	2674	0	PREDICTED: Bison bison bison Kirsten rat sarcoma viral oncogene homolog (KRAS), transcript variant X4, mRNA	P01116	370	4e-116	GTPase KRas	SPAC17H9.09c	204	1.03e-58	COG1100	pfam00071	Ras	Ras family. Includes sub-families Ras, Rab, Rac, Ral, Ran, Rap Ypt1 and more. Shares P-loop motif with GTP_EFTU, arf and	GO:0030036|actin cytoskeleton organization;GO:0007411|axon guidance;GO:0019221|cytokine-mediated signaling pathway;GO:0038002|endocrine signaling;GO:0007173|epidermal growth factor receptor signaling pathway;GO:0060441|epithelial tube branching involved in lung morphogenesis;GO:0038128|ERBB2 signaling pathway;GO:0038095|Fc-epsilon receptor signaling pathway;GO:0021897|forebrain astrocyte development;GO:0048873|homeostasis of number of cells within a tissue;GO:0050900|leukocyte	GO:0005737|cytoplasm;GO:0005829|cytosol;GO:0031234|extrinsic component of cytoplasmic side of plasma membrane;GO:0005925|focal	GO:0019003|GDP binding;GO:0019002|GMP binding;GO:0005525	K07827	KRAS, KRAS2; GTPase KRas
RFX1	374.0347	0.582554	0.164851706	3.533808	0.0004096	0.006841	Not_Sig	no	gi|759190860|ref|XP_011382776.1|	3957	0	PREDICTED: MHC class II regulatory factor RFX1 isoform X7 [Pteropus vampyrus]	gi|371912803|dbj|AK392460.1|	3234	0	Sus scrofa mRNA, clone: HTMT10076B10, expressed in hypothalamus	P22670	1498	0	MHC class II regulatory factor RFX1	.	.	.	.	pfam02257	RFX_DNA_binding	RFX DNA-binding domain. RFX is a regulatory factor which binds to the X box of MHC class II genes and is essential for their expression.	GO:0006955|immune response;GO:0006351|transcription, DNA-templated;	GO:0043231|intracellular membrane-bounded organelle;GO:0005654|nucleoplasm;GO:0005634|nucleus;	GO:0000978|RNA polymerase II core promoter proximal region sequence-	K09173	RFX1_2_3; regulatory factor X 1/2/3
CCNB3	99.47381	0.700337	0.198446145	3.529104	0.000417	0.006954	UP	yes	gi|741982978|ref|XP_010820246.1|	6297	0	PREDICTED: G2/mitotic-specific cyclin-B3 isoform X1 [Bos taurus]	gi|983011235|ref|XM_015470464.1|	4150	0	PREDICTED: Bos taurus cyclin B3 (CCNB3), transcript variant X1, mRNA	Q659K0	893	0	G2/mitotic-specific cyclin-B3	SPBC582.03	140	2.95e-34	COG5024	pfam00134	Cyclin_N	Cyclin, N-terminal domain. Cyclins regulate cyclin dependent kinases (CDKs). Human cyclin-O is a Uracil-DNA glycosylase that is related to	GO:0051301|cell division;GO:0051321|meiotic cell cycle;GO:0000079|regulation of cyclin-dependent protein serine/threonine kinase activity;GO:0010389|regulation of G2/M transition of mitotic cell cycle;	GO:0005634|nucleus;	.	K05868	CCNB; cyclin B
CPNE8	378.4767	-0.46959	0.133096004	-3.52822	0.0004184	0.006958	Not_Sig	no	gi|555951180|ref|XP_005887789.1|	2969	0	PREDICTED: copine-8 [Bos mutus]	gi|982925380|ref|XM_005206434.3|	3422	0	PREDICTED: Bos taurus copine VIII (CPNE8), transcript variant X1, mRNA	Q9DC53	1110	0	Copine-8	.	.	.	.	pfam07002	Copine	Copine. This family represents a conserved region approximately 180 residues long within eukaryotic copines. Copines are	.	GO:0070062|extracellular exosome;	.	.	.
POLA2	296.1179	0.575306	0.163060326	3.528179	0.0004184	0.006958	Not_Sig	no	gi|555964097|ref|XP_005894120.1|	2877	0	PREDICTED: DNA polymerase alpha subunit B isoform X2 [Bos mutus]	gi|555964096|ref|XM_005894058.1|	2454	0	PREDICTED: Bos mutus polymerase (DNA directed), alpha 2, accessory subunit (POLA2), transcript variant X2, mRNA	Q58D13	1024	0	DNA polymerase alpha subunit B	SPCC553.09c	184	7.02e-49	COG5214	pfam04042	DNA_pol_E_B	DNA polymerase alpha/epsilon subunit B. This family contains a number of DNA polymerase subunits. The B subunit of the	GO:0006270|DNA replication initiation;	GO:0005658|alpha DNA polymerase:primase complex;	GO:0003677|DNA binding;GO:0003887|DNA-directed DNA polymerase	K02321	POLA2; DNA polymerase alpha subunit B
MINDY2	514.4956	-0.59091	0.167575074	-3.52627	0.0004215	0.006991	DOWN	yes	gi|555955617|ref|XP_005889964.1|	2371	0	PREDICTED: protein FAM63B isoform X2 [Bos mutus]	gi|742104963|ref|XM_010836843.1|	7671	0	PREDICTED: Bison bison bison family with sequence similarity 63, member B (FAM63B), mRNA	Q2KI23	902	0	Ubiquitin carboxyl-terminal hydrolase MINDY-2	.	.	.	.	pfam04424	DUF544	Protein of unknown function (DUF544). Eukaryotic protein of unknown function.	.	GO:0005654|nucleoplasm;	GO:1990380|Lys48-specific deubiquitinase activity;GO:0004843	.	.
FAM241A	50.62143	-1.0642	0.301801846	-3.52615	0.0004216	0.006991	DOWN	yes	gi|440911331|gb|ELR61013.1|	625	9e-96	hypothetical protein M91_09415, partial [Bos mutus]	gi|850484145|gb|CP011891.1|	4524	0	Ovis canadensis canadensis isolate 43U chromosome 6 sequence	Q17QE0	172	4e-47	Uncharacterized protein C4orf32 homolog	.	.	.	.	pfam15378	DUF4605	Domain of unknown function (DUF4605). This protein family is a domain of unknown function, which is found in eukaryotes.	.	GO:0016021|integral component of membrane;	.	.	.
TMEM63A	659.9334	0.505355	0.143391501	3.524304	0.0004246	0.00703	Not_Sig	no	gi|440899224|gb|ELR50556.1|	4020	0	Transmembrane protein 63A, partial [Bos mutus]	gi|742174925|ref|XM_010852865.1|	3453	0	PREDICTED: Bison bison bison transmembrane protein 63A (TMEM63A), mRNA	Q5R826	1377	0	CSC1-like protein 1	SPAC2G11.09	59.7	5.16e-08	COG5594	pfam02714	DUF221	Domain of unknown function DUF221. This family consists of hypothetical transmembrane proteins none of which have any	GO:0006811|ion transport;	GO:0016021|integral component of membrane;GO:0005765|lysosomal membrane;	GO:0000166|nucleotide binding;	.	.
SZT2	323.0109	0.550128	0.156154739	3.522969	0.0004267	0.007055	Not_Sig	no	gi|528902560|ref|XP_613325.6|	15475	0	PREDICTED: protein SZT2 isoform X1 [Bos taurus]	gi|982979563|ref|XM_613325.9|	10866	0	PREDICTED: Bos taurus seizure threshold 2 homolog (mouse) (SZT2), transcript variant X1, mRNA	Q5T011	5531	0	Protein SZT2	.	.	.	.	pfam05642	Sporozoite_P67	Sporozoite P67 surface antigen. This family consists of several Theileria P67 surface antigens. A stage specific surface antigen of	GO:0007417|central nervous system development;GO:0021540|corpus callosum morphogenesis;GO:0043473|pigmentation;GO:0009791|post-embryonic development;GO:1901668|regulation of superoxide dismutase activity;	GO:0070062|extracellular exosome;GO:0005777|peroxisome;	.	.	.
LOC102273339	168.639	-0.63098	0.179184082	-3.5214	0.0004293	0.007078	DOWN	yes	gi|829820049|ref|XP_012630929.1|	2582	0	PREDICTED: LOW QUALITY PROTEIN: frizzled-3 [Microcebus murinus]	gi|742119781|ref|XM_010840288.1|	4742	0	PREDICTED: Bison bison bison frizzled class receptor 3 (FZD3), mRNA	Q61086	994	0	Frizzled-3	.	.	.	.	pfam01534	Frizzled	Frizzled/Smoothened family membrane region. This family contains the membrane spanning region of frizzled and smoothened	GO:0060070|canonical Wnt signaling pathway;GO:0033278|cell proliferation in midbrain;GO:0071679|commissural neuron axon guidance;GO:0001736|establishment of planar polarity;GO:0001942|hair follicle development;GO:0042472|inner ear morphogenesis;GO:0030901|midbrain development;GO:1900118|negative regulation of execution phase of apoptosis;GO:0045976|negative regulation of mitotic cell cycle, embryonic;GO:0001843|neural tube closure;GO:0001764|neuron migration;GO:0002052|positive regulation of	GO:0045177|apical part of cell;GO:0016324|apical plasma membrane;GO:0030424|axon;GO:0009986|cell	GO:0004930|G-protein coupled receptor activity;GO:0030165	K02329	FZD3; frizzled 3
SLC7A5	1340.125	0.517729	0.147025865	3.521345	0.0004294	0.007078	Not_Sig	no	gi|27807107|ref|NP_777038.1|	2295	0	large neutral amino acids transporter small subunit 1 [Bos taurus]	gi|31341534|ref|NM_174613.2|	3903	0	Bos taurus solute carrier family 7 (amino acid transporter light chain, L system), member 5 (SLC7A5), mRNA	Q63016	779	0	Large neutral amino acids transporter small subunit 1	SA1270	119	1.34e-27	COG0531	pfam13520	AA_permease_2	Amino acid permease.	GO:0030154|cell differentiation;GO:0007399|nervous system development;GO:0015804|neutral amino acid transport;	GO:0016324|apical plasma membrane;GO:0005829|cytosol;GO:0070062|extracellular exosome;GO:0005887|integral component	GO:0015171|amino acid transmembrane transporter activity;GO:0015297	K13780	SLC7A5, LAT1; solute carrier family 7 (L-type amino acid transporter),
DYRK1B	419.0653	0.613913	0.174360966	3.520929	0.00043	0.007079	UP	yes	gi|528991406|ref|XP_005218978.1|	2563	0	PREDICTED: dual specificity tyrosine-phosphorylation-regulated kinase 1B isoform X1 [Bos taurus]	gi|926714369|ref|XM_013971287.1|	3849	0	PREDICTED: Capra hircus dual-specificity tyrosine-(Y)-phosphorylation regulated kinase 1B (DYRK1B), mRNA	Q9Y463	937	0	Dual specificity tyrosine-phosphorylation-regulated kinase 1B	.	.	.	.	pfam00069	Pkinase	Protein kinase domain.	GO:0060612|adipose tissue development;GO:0007520|myoblast fusion;GO:0045893|positive regulation of transcription, DNA-templated;GO:0006468|protein phosphorylation;	GO:0005634|nucleus;	GO:0005524|ATP binding;GO:0004672|protein kinase activity;GO:0004674	K08825	DYRK1; dual specificity tyrosine-phosphorylation-regulated kinase 1
NLGN2	1427.396	0.572362	0.162583857	3.520411	0.0004309	0.007083	Not_Sig	no	gi|826326064|ref|XP_012511180.1|	3345	0	PREDICTED: neuroligin-2 [Propithecus coquereli]	gi|965814016|ref|XM_012122632.2|	4589	0	PREDICTED: Ovis aries musimon neuroligin 2 (NLGN2), transcript variant X4, mRNA	Q8NFZ4	1290	0	Neuroligin-2	BS_pnbA	209	2.74e-57	COG2272	pfam00135	COesterase	Carboxylesterase family.	GO:0045217|cell-cell junction maintenance;GO:0097116|gephyrin clustering involved in postsynaptic density assembly;GO:0035641|locomotory exploration behavior;GO:0050804|modulation of synaptic transmission;GO:0050885|neuromuscular process controlling balance;GO:0007158|neuron cell-cell adhesion;GO:2000463|positive regulation of excitatory postsynaptic potential;GO:0097151|positive regulation of inhibitory postsynaptic potential;GO:0032024|positive regulation of insulin	GO:0030054|cell junction;GO:0009986|cell surface;GO:0060077|inhibitory synapse;GO:0005887|integral component of plasma	GO:0052689|carboxylic ester hydrolase activity;GO:0050839|cell adhesion	K07378	NLGN; neuroligin
FBXL18	456.148	0.480218	0.136478477	3.518634	0.0004338	0.007112	Not_Sig	no	gi|528926105|ref|XP_003584105.2|	3567	0	PREDICTED: F-box/LRR-repeat protein 18 [Bos taurus]	gi|594039490|ref|XM_006044272.1|	4531	0	PREDICTED: Bubalus bubalis F-box and leucine-rich repeat protein 18 (FBXL18), transcript variant X1, mRNA	Q96ME1	1113	0	F-box/LRR-repeat protein 18	.	.	.	.	pfam12937	F-box-like	F-box-like. This is an F-box-like family.	.	.	.	.	.
ATG2A	455.7175	0.575648	0.163611727	3.518381	0.0004342	0.007112	Not_Sig	no	gi|742110356|ref|XP_010836317.1|	8304	0	PREDICTED: autophagy-related protein 2 homolog A [Bison bison bison]	gi|742110355|ref|XM_010838015.1|	6241	0	PREDICTED: Bison bison bison autophagy related 2A (ATG2A), mRNA	Q2TAZ0	2764	0	Autophagy-related protein 2 homolog A	.	.	.	.	pfam09333	ATG_C	ATG C terminal domain. ATG2 (also known as Apg2) is a peripheral membrane protein. It functions in both cytoplasm to vacuole	GO:0000045|autophagosome assembly;GO:0000422|mitophagy;GO:0044804|nucleophagy;	GO:0019898|extrinsic component of membrane;GO:0005811|lipid particle;GO:0000407|pre-autophagosomal structure;GO:0034045|pre-autophagosomal	.	K17906	ATG2; autophagy-related protein 2

<table>
<tr><td>SH3BP2</td><td>255.87</td><td>0.597277</td><td>0.169768112</td><td>3.518191</td><td>0.0004345</td><td>0.007112</td><td>UP</td><td>yes</td><td>gi|555964857|ref|XP_005894490.1|</td><td>2354</td><td>0</td><td>PREDICTED: SH3 domain-binding protein 2 [Bos mutus]</td><td>gi|942050992|ref|XM_005894428.2|</td><td>2143</td><td>0</td><td>PREDICTED: Bos mutus SH3-domain binding protein 2 (SH3BP2), mRNA</td><td>P78314</td><td>710</td><td>0</td><td>SH3 domain-binding protein 2</td><td>.</td><td>.</td><td>.</td><td>.</td><td>pfam00169</td><td>PH</td><td>PH domain. PH stands for pleckstrin homology.</td><td>GO:0007165|signal transduction;</td><td>.</td><td>GO:0005070|SH3/SH2 adaptor activity;</td><td>K07984</td><td>SH3BP2; SH3-domain binding protein 2</td></tr>
<tr><td>CLSTN1</td><td>1876.818</td><td>0.381393</td><td>0.108453589</td><td>3.516644</td><td>0.000437</td><td>0.007143</td><td>Not_Sig</td><td>no</td><td>gi|741891649|ref|XP_592305.5|</td><td>4637</td><td>0</td><td>PREDICTED: calsyntenin-1 isoform X2 [Bos taurus]</td><td>gi|965791194|ref|XM_012111241.2|</td><td>2893</td><td>0</td><td>PREDICTED: Ovis aries musimon calsyntenin 1 (CLSTN1), transcript variant X5, mRNA</td><td>O94985</td><td>1691</td><td>0</td><td>Calsyntenin-1</td><td>.</td><td>.</td><td>.</td><td>.</td><td>pfam13385</td><td>Laminin_G_3</td><td>Concanavalin A-like lectin/glucanases superfamily. This domain belongs to the Concanavalin A-like</td><td>GO:0007155|cell adhesion;GO:0007156|homophilic cell adhesion via plasma membrane adhesion molecules;GO:0051965|positive regulation of synapse assembly;GO:0050806|positive regulation of synaptic transmission;GO:0001558|regulation of cell growth;</td><td>GO:0030054|cell junction;GO:0042995|cell projection;GO:0009986|cell surface;GO:0005789|endoplasmic reticulum membrane;GO:0070062|extracellular</td><td>GO:0001540|beta-amyloid binding;GO:0005509|calcium ion</td><td>.</td><td>.</td></tr>
<tr><td>DUSP7</td><td>300.8705</td><td>0.531294</td><td>0.15133027</td><td>3.510826</td><td>0.0004467</td><td>0.007291</td><td>Not_Sig</td><td>no</td><td>gi|238231449|ref|NP_001094764.2|</td><td>1873</td><td>0</td><td>dual specificity protein phosphatase 7 [Bos taurus]</td><td>gi|151553634|gb|BC148065.1|</td><td>3065</td><td>0</td><td>Bos taurus dual specificity phosphatase 7, mRNA (cDNA clone MGC:142957 IMAGE:8315396), complete cds</td><td>Q16829</td><td>717</td><td>0</td><td>Dual specificity protein phosphatase 7</td><td>SPBC17A3.06</td><td>86.3</td><td>3.90e-17</td><td>COG2453</td><td>pfam00782</td><td>DSPc</td><td>Dual specificity phosphatase, catalytic domain. Ser/Thr and Tyr protein phosphatases. The enzyme's tertiary fold is highly</td><td>GO:0000187|activation of MAPK activity;GO:0000165|MAPK cascade;GO:0043407|negative regulation of MAP kinase activity;GO:0035335|peptidyl-tyrosine dephosphorylation;</td><td>GO:0005829|cytosol;GO:0005654|nucleoplasm;</td><td>GO:0017017|MAP kinase tyrosine/serine/threonine phosphatase</td><td>K04459</td><td>DUSP, MKP; dual specificity MAP kinase phosphatase [EC:3.1.3.16</td></tr>
<tr><td>GADD45A</td><td>1176.19</td><td>-0.37089</td><td>0.105672932</td><td>-3.50982</td><td>0.0004484</td><td>0.007308</td><td>Not_Sig</td><td>no</td><td>gi|77735449|ref|NP_001029419.1|</td><td>866</td><td>2e-112</td><td>growth arrest and DNA damage-inducible protein GADD45 alpha [Bos taurus]</td><td>gi|742165894|ref|XM_010850306.1|</td><td>1298</td><td>0</td><td>PREDICTED: Bison bison bison growth arrest and DNA-damage-inducible, alpha (GADD45A), mRNA</td><td>Q3ZBN6</td><td>338</td><td>7e-114</td><td>Growth arrest and DNA damage-inducible protein GADD45 alpha</td><td>.</td><td>.</td><td>.</td><td>.</td><td>pfam01248</td><td>Ribosomal_L7Ae</td><td>Ribosomal protein L7Ae/L30e/S12e/Gadd45 family. This family includes: Ribosomal L7A from metazoa, Ribosomal L8-A</td><td>GO:0000185|activation of MAPKKK activity;GO:0071479|cellular response to ionizing radiation;GO:0071260|cellular response to mechanical stimulus;GO:0007098|centrosome cycle;GO:0071850|mitotic cell cycle arrest;GO:0006469|negative regulation of protein kinase activity;GO:0043065|positive regulation of apoptotic process;GO:0046330|positive regulation of JNK cascade;GO:1900745|positive regulation of p38MAPK cascade;GO:2000379|positive regulation of reactive oxygen species metabolic process;GO:0042770|signal transduction in</td><td>GO:0005737|cytoplasm;GO:0005654|nucleoplasm;</td><td>GO:0001047|core promoter binding;</td><td>K04402</td><td>GADD45; growth arrest and DNA-damage-inducible protein</td></tr>
<tr><td>SPCS2</td><td>988.65</td><td>-0.50373</td><td>0.143625693</td><td>-3.50721</td><td>0.0004528</td><td>0.00737</td><td>Not_Sig</td><td>no</td><td>gi|528915715|ref|XP_001254330.3|</td><td>1138</td><td>1e-151</td><td>PREDICTED: signal peptidase complex subunit 2 [Bos taurus]</td><td>gi|741890732|ref|XM_001254329.5|</td><td>1368</td><td>0</td><td>PREDICTED: Bos taurus signal peptidase complex subunit 2 (SPCS2), mRNA</td><td>Q9CYN2</td><td>396</td><td>2e-135</td><td>Signal peptidase complex subunit 2</td><td>.</td><td>.</td><td>.</td><td>.</td><td>pfam06703</td><td>SPC25</td><td>Microsomal signal peptidase 25 kDa subunit (SPC25). This family consists of several microsomal signal peptidase 25 kDa subunit</td><td>GO:0045047|protein targeting to ER;GO:0006465|signal peptide processing;</td><td>GO:0016021|integral component of membrane;GO:0031090|organelle membrane;GO:0005787|signal peptidase complex;</td><td>GO:0008233|peptidase activity;</td><td>K12947</td><td>SPCS2, SPC2; signal peptidase complex subunit 2 [EC:3.4.-.-]</td></tr>
<tr><td>RETREG1</td><td>320.9666</td><td>-0.48791</td><td>0.139165083</td><td>-3.50596</td><td>0.000455</td><td>0.007394</td><td>Not_Sig</td><td>no</td><td>gi|734797741|gb|AJA33384.1|</td><td>1660</td><td>0</td><td>FAM134B protein [Bos grunniens]</td><td>gi|742197800|ref|XM_010858802.1|</td><td>3243</td><td>0</td><td>PREDICTED: Bison bison bison family with sequence similarity 134, member B (FAM134B), transcript variant X2, mRNA</td><td>Q5E9K8</td><td>619</td><td>0</td><td>Reticulophagy receptor FAM134B</td><td>.</td><td>.</td><td>.</td><td>.</td><td>pfam06213</td><td>CobT</td><td>Cobalamin biosynthesis protein CobT. This family consists of several bacterial cobalamin biosynthesis (CobT) proteins. CobT</td><td>GO:0043524|negative regulation of neuron apoptotic process;GO:0061709|reticulophagy;GO:0019233|sensory perception of pain;</td><td>GO:0005801|cis-Golgi network;GO:0005794|Golgi apparatus;GO:0030176|integral component of endoplasmic reticulum membrane;</td><td>.</td><td>.</td><td>.</td></tr>
<tr><td>ENTREP3</td><td>578.001</td><td>0.548128</td><td>0.156402854</td><td>3.504589</td><td>0.0004573</td><td>0.007414</td><td>Not_Sig</td><td>no</td><td>gi|803255578|ref|XP_011987743.1|</td><td>2606</td><td>0</td><td>PREDICTED: protein FAM189B isoform X5 [Ovis aries musimon]</td><td>gi|965833356|ref|XM_012132353.2|</td><td>3006</td><td>0</td><td>PREDICTED: Ovis aries musimon family with sequence similarity 189 member B (FAM189B), transcript variant X5, mRNA</td><td>P81408</td><td>908</td><td>0</td><td>Protein FAM189B</td><td>.</td><td>.</td><td>.</td><td>.</td><td>pfam04103</td><td>CD20</td><td>CD20-like family. This family includes the CD20 protein and the beta subunit of the high affinity receptor for IgE Fc. The high</td><td>.</td><td>GO:0016021|integral component of membrane;</td><td>GO:0050699|WW domain binding;</td><td>.</td><td>.</td></tr>
<tr><td>TSPAN6</td><td>927.5347</td><td>-0.45048</td><td>0.128543458</td><td>-3.50449</td><td>0.0004575</td><td>0.007414</td><td>Not_Sig</td><td>no</td><td>gi|62089338|dbj|BAD93113.1|</td><td>1336</td><td>0</td><td>transmembrane 4 superfamily member 6 variant [Homo sapiens]</td><td>gi|942034856|ref|XM_005888595.2|</td><td>1125</td><td>0</td><td>PREDICTED: Bos mutus tetraspanin 6 (TSPAN6), transcript variant X1, mRNA</td><td>Q32KU6</td><td>507</td><td>5e-180</td><td>Tetraspanin-6</td><td>.</td><td>.</td><td>.</td><td>.</td><td>pfam00335</td><td>Tetraspannin</td><td>Tetraspanin family.</td><td>GO:0007166|cell surface receptor signaling pathway;GO:1901223|negative regulation of NIK/NF-kappaB signaling;GO:0039532|negative regulation of viral-induced cytoplasmic pattern recognition receptor signaling pathway;GO:0043123|positive regulation of I-kappaB kinase/NF-kappaB signaling;</td><td>GO:0070062|extracellular exosome;GO:0005887|integral component of plasma membrane;</td><td>GO:0004871|signal transducer activity;</td><td>K17295</td><td>TSPAN6; tetraspanin-6</td></tr>
<tr><td>SMIM15</td><td>257.5352</td><td>-0.68275</td><td>0.194925239</td><td>-3.5026</td><td>0.0004607</td><td>0.007456</td><td>DOWN</td><td>yes</td><td>gi|946754916|ref|XP_014392325.1|</td><td>362</td><td>4e-37</td><td>PREDICTED: small integral membrane protein 15-like [Myotis brandtii]</td><td>gi|742148848|ref|XM_010845498.1|</td><td>1776</td><td>0</td><td>PREDICTED: Bison bison bison small integral membrane protein 15 (SMIM15), transcript variant X1, mRNA</td><td>Q5R4D8</td><td>113</td><td>4e-28</td><td>Small integral membrane protein 15</td><td>.</td><td>.</td><td>.</td><td>.</td><td>pfam15086</td><td>UPF0542</td><td>Uncharacterized protein family UPF0542. This family of proteins is functionally uncharacterized. This</td><td>.</td><td>GO:0016021|integral component of membrane;</td><td>.</td><td>.</td><td>.</td></tr>
<tr><td>CD109</td><td>2147.667</td><td>-0.34536</td><td>0.098625525</td><td>-3.50172</td><td>0.0004623</td><td>0.00747</td><td>Not_Sig</td><td>no</td><td>gi|742169384|ref|XP_010849565.1|</td><td>7302</td><td>0</td><td>PREDICTED: CD109 antigen [Bison bison bison]</td><td>gi|742169383|ref|XM_010851263.1|</td><td>4795</td><td>0</td><td>PREDICTED: Bison bison bison CD109 molecule (CD109), mRNA</td><td>Q6YHK3</td><td>2468</td><td>0</td><td>CD109 antigen</td><td>TM0984</td><td>72.4</td><td>2.54e-11</td><td>COG2373</td><td>pfam07678</td><td>A2M_comp</td><td>family of proteins is found in A-macroglobulin complement component. This family includes the complement components region of the alpha-2-</td><td>GO:0001942|hair follicle development;GO:0010839|negative regulation of keratinocyte proliferation;GO:0001933|negative regulation of protein phosphorylation;GO:0030512|negative regulation of transforming growth factor beta receptor signaling pathway;GO:0061045|negative regulation of wound healing;GO:0072675|osteoclast fusion;GO:0045616|regulation of keratinocyte differentiation;</td><td>GO:0031225|anchored component of membrane;GO:0009986|cell surface;GO:0005615|extracellular space;GO:0005886|plasma membrane;</td><td>GO:0004867|serine-type endopeptidase inhibitor activity;GO:0050431</td><td>K06530</td><td>CD109; CD109 antigen</td></tr>
<tr><td>FKBP2</td><td>406.2206</td><td>-0.46022</td><td>0.131462609</td><td>-3.5008</td><td>0.0004639</td><td>0.007476</td><td>Not_Sig</td><td>no</td><td>gi|164448580|ref|NP_001106727.1|</td><td>837</td><td>2e-111</td><td>peptidyl-prolyl cis-trans isomerase FKBP2 isoform 1 [Bos taurus]</td><td>gi|942050252|ref|XM_005894124.2|</td><td>663</td><td>0</td><td>PREDICTED: Bos mutus FK506 binding protein 2, 13kDa (FKBP2), transcript variant X1, mRNA</td><td>Q32PA9</td><td>285</td><td>2e-96</td><td>Peptidyl-prolyl cis-trans isomerase FKBP2</td><td>YDR519w</td><td>124</td><td>1.27e-35</td><td>COG0545</td><td>pfam00254</td><td>FKBP_C</td><td>FKBP-type peptidyl-prolyl cis-trans isomerase.</td><td>GO:0061077|chaperone-mediated protein folding;</td><td>GO:0005789|endoplasmic reticulum membrane;</td><td>GO:0005528|FK506 binding;GO:0003755|peptidyl-prolyl cis-trans isomerase</td><td>.</td><td>.</td></tr>
<tr><td>SYNPO2L</td><td>72.58335</td><td>0.916824</td><td>0.261917772</td><td>3.500429</td><td>0.0004645</td><td>0.007476</td><td>UP</td><td>yes</td><td>gi|594057085|ref|XP_006052727.1|</td><td>2469</td><td>0</td><td>PREDICTED: synaptopodin 2-like protein isoform X2 [Bubalus bubalis]</td><td>gi|594057084|ref|XM_006052665.1|</td><td>2458</td><td>0</td><td>PREDICTED: Bubalus bubalis synaptopodin 2-like (SYNPO2L), transcript variant X2, mRNA</td><td>Q9H987</td><td>809</td><td>0</td><td>Synaptopodin 2-like protein</td><td>.</td><td>.</td><td>.</td><td>.</td><td>pfam00595</td><td>PDZ</td><td>PDZ domain (Also known as DHR or GLGF). PDZ domains are found in diverse signaling proteins.</td><td>GO:0032233|positive regulation of actin filament bundle assembly;</td><td>GO:0015629|actin cytoskeleton;GO:0005634|nucleus;GO:0030018|Z disc;</td><td>GO:0003779|actin binding;</td><td>.</td><td>.</td></tr>
<tr><td>FLNB</td><td>17928.17</td><td>0.34899</td><td>0.099700416</td><td>3.500383</td><td>0.0004646</td><td>0.007476</td><td>Not_Sig</td><td>no</td><td>gi|555974123|ref|XP_005899056.1|</td><td>13664</td><td>0</td><td>PREDICTED: filamin-B isoform X1 [Bos mutus]</td><td>gi|942064050|ref|XM_005898994.2|</td><td>9474</td><td>0</td><td>PREDICTED: Bos mutus filamin B, beta (FLNB), transcript variant X1, mRNA</td><td>O75369</td><td>5050</td><td>0</td><td>Filamin-B</td><td>ECU09g0290</td><td>134</td><td>1.86e-31</td><td>COG5069</td><td>pfam00630</td><td>Filamin</td><td>Filamin/ABP280 repeat.</td><td>GO:0030036|actin cytoskeleton organization;GO:0007016|cytoskeletal anchoring at plasma membrane;GO:0003382|epithelial cell morphogenesis;GO:0003334|keratinocyte development;GO:0007165|signal transduction;GO:0007519|skeletal muscle tissue development;</td><td>GO:0015629|actin cytoskeleton;GO:0005903|brush border;GO:0005938|cell cortex;GO:0005913|cell-cell adherens</td><td>GO:0003779|actin binding;GO:0098641|cadherin binding involved in cell-cell</td><td>K04437</td><td>FLNA; filamin</td></tr>
<tr><td>C14H8orf82</td><td>136.4087</td><td>0.657626</td><td>0.187983387</td><td>3.49832</td><td>0.0004682</td><td>0.007515</td><td>UP</td><td>yes</td><td>gi|742108529|ref|XP_010835868.1|</td><td>802</td><td>4e-102</td><td>PREDICTED: UPF0598 protein C8orf82 homolog isoform X1 [Bison bison bison]</td><td>gi|965951879|ref|XM_004011612.3|</td><td>817</td><td>0</td><td>PREDICTED: Ovis aries chromosome 9 open reading frame, human C8orf82 (C9H8orf82), transcript variant X1, mRNA</td><td>A8E4L3</td><td>312</td><td>2e-103</td><td>UPF0598 protein C8orf82 homolog</td><td>.</td><td>.</td><td>.</td><td>.</td><td>pfam14956</td><td>DUF4505</td><td>Domain of unknown function (DUF4505). This family of proteins is found in bacteria and eukaryotes. Proteins in this family</td><td>.</td><td>.</td><td>.</td><td>.</td><td>.</td></tr>
<tr><td>ERO1A</td><td>1688.34</td><td>-0.41177</td><td>0.117708079</td><td>-3.49825</td><td>0.0004683</td><td>0.007515</td><td>Not_Sig</td><td>no</td><td>gi|741939063|ref|XP_010807159.1|</td><td>2352</td><td>0</td><td>PREDICTED: ERO1-like protein alpha isoform X1 [Bos taurus]</td><td>gi|982935909|ref|XM_010808857.2|</td><td>4750</td><td>0</td><td>PREDICTED: Bos taurus endoplasmic reticulum oxidoreductase alpha (ERO1A), transcript variant X1, mRNA</td><td>A5PJN2</td><td>904</td><td>0</td><td>ERO1-like protein alpha</td><td>SPCC1450.14c</td><td>213</td><td>4.21e-58</td><td>COG5061</td><td>pfam04137</td><td>ERO1</td><td>Endoplasmic Reticulum Oxidoreductin 1 (ERO1). Members of this family are required for the formation of disulphide bonds in</td><td>GO:0019471|4-hydroxyproline metabolic process;GO:0050873|brown fat cell differentiation;GO:0045454|cell redox homeostasis;GO:0006464|cellular protein modification process;GO:0051085|chaperone mediated protein folding requiring cofactor;GO:0030968|endoplasmic reticulum unfolded protein response;GO:0030198|extracellular matrix organization;GO:0070059|intrinsic apoptotic signaling pathway in response to endoplasmic reticulum stress;GO:0034975|protein folding in endoplasmic</td><td>GO:0005789|endoplasmic reticulum membrane;</td><td>GO:0016671|oxidoreductase activity, acting on a sulfur group of donors,</td><td>K10950</td><td>ERO1L; ERO1-like protein alpha [EC:1.8.4.-]</td></tr>
<tr><td>MYC</td><td>2576.817</td><td>0.49725</td><td>0.142157666</td><td>3.497876</td><td>0.000469</td><td>0.007515</td><td>Not_Sig</td><td>no</td><td>gi|114050751|ref|NP_001039539.1|</td><td>1595</td><td>0</td><td>myc proto-oncogene protein [Bos taurus]</td><td>gi|153792752|ref|NM_001046074.2|</td><td>2146</td><td>0</td><td>Bos taurus v-myc avian myelocytomatosis viral oncogene homolog (MYC), mRNA</td><td>Q2HJ27</td><td>619</td><td>0</td><td>Myc proto-oncogene protein</td><td>.</td><td>.</td><td>.</td><td>.</td><td>pfam01056</td><td>Myc_N</td><td>Myc amino-terminal region. The myc family belongs to the basic helix-loop-helix leucine zipper class of transcription factors, see</td><td>GO:0006919|activation of cysteine-type endopeptidase activity involved in apoptotic process;GO:0060070|canonical Wnt signaling pathway;GO:0006879|cellular iron ion homeostasis;GO:0006974|cellular response to DNA damage stimulus;GO:0006338|chromatin remodeling;GO:0051276|chromosome organization;GO:0097193|intrinsic apoptotic signaling pathway;GO:0000165|MAPK cascade;GO:0043066|negative regulation of apoptotic process;GO:0051782|negative regulation of cell division;GO:0045656|negative</td><td>GO:0016604|nuclear body;GO:0005730|nucleolus;GO:0005654|nucleoplasm;GO:0005634|nucleus;GO:0005819|spindle;</td><td>GO:0003677|DNA binding;GO:0070888|E-box binding;GO:0032403</td><td>K04377</td><td>MYC; Myc proto-oncogene protein</td></tr>
<tr><td>RBM19</td><td>620.3145</td><td>0.442125</td><td>0.126438277</td><td>3.496768</td><td>0.0004709</td><td>0.007536</td><td>Not_Sig</td><td>no</td><td>gi|803209987|ref|XP_011965443.1|</td><td>4075</td><td>0</td><td>PREDICTED: probable RNA-binding protein 19 isoform X3 [Ovis aries musimon]</td><td>gi|114051909|ref|NM_001045955.1|</td><td>3290</td><td>0</td><td>Bos taurus RNA binding motif protein 19 (RBM19), mRNA</td><td>Q9Y4C8</td><td>1368</td><td>0</td><td>Probable RNA-binding protein 19</td><td>.</td><td>.</td><td>.</td><td>.</td><td>pfam00076</td><td>RRM_1</td><td>RNA recognition motif. (a.k.a. RRM, RBD, or RNP domain). The RRM motif is probably diagnostic of an RNA binding protein. RRMs</td><td>GO:0007275|multicellular organismal development;GO:0040019|positive regulation of embryonic development;</td><td>GO:0005694|chromosome;GO:0005737|cytoplasm;GO:0016020|membrane;GO:0005730|nucleolus;GO:0005654|nucleoplasm;GO:0005634|nucleus;</td><td>GO:0000166|nucleotide binding;GO:0044822|poly(A) RNA</td><td>K14787</td><td>MRD1, RBM19; multiple RNA-binding domain-containing protein 1</td></tr>
<tr><td>PLEC</td><td>6959.973</td><td>0.441171</td><td>0.126203877</td><td>3.495698</td><td>0.0004728</td><td>0.007556</td><td>Not_Sig</td><td>no</td><td>gi|594060594|ref|XP_006054396.1|</td><td>9090</td><td>0</td><td>PREDICTED: plectin isoform X1 [Bubalus bubalis]</td><td>gi|594060593|ref|XM_006054334.1|</td><td>14375</td><td>0</td><td>PREDICTED: Bubalus bubalis plectin (PLEC), transcript variant X1, mRNA</td><td>P30427</td><td>3169</td><td>0</td><td>Plectin</td><td>ECU09g0290</td><td>169</td><td>1.78e-42</td><td>COG5069</td><td>pfam03501</td><td>S10_plectin</td><td>Plectin/S10 domain. This presumed domain is found at the N-terminus of some isoforms of the cytoskeletal muscle protein</td><td>GO:0030855|epithelial cell differentiation;GO:0007565|female pregnancy;GO:0031581|hemidesmosome assembly;GO:0007584|response to nutrient;</td><td>GO:0016324|apical plasma membrane;GO:0009925|basal plasma membrane;GO:0043034|costamere;GO:0005856|cytoskeleton;GO:0030056|hemidesm</td><td>GO:0030506|ankyrin binding;GO:0008092|cytoskeletal</td><td>.</td><td>.</td></tr>
<tr><td>RNF26</td><td>687.129</td><td>0.478929</td><td>0.13707266</td><td>3.493979</td><td>0.0004759</td><td>0.007594</td><td>Not_Sig</td><td>no</td><td>gi|555970311|ref|XP_005897178.1|</td><td>1570</td><td>0</td><td>PREDICTED: RING finger protein 26 [Bos mutus]</td><td>gi|742230618|ref|XM_010830463.1|</td><td>2780</td><td>0</td><td>PREDICTED: Bison bison bison ring finger protein 26 (RNF26), mRNA</td><td>Q9BY78</td><td>537</td><td>0</td><td>RING finger protein 26</td><td>.</td><td>.</td><td>.</td><td>.</td><td>pfam13920</td><td>zf-C3HC4_3</td><td>Zinc finger, C3HC4 type (RING finger).</td><td>.</td><td>GO:0016021|integral component of membrane;</td><td>GO:0008270|zinc ion binding;</td><td>K15693</td><td>RNF26; RING finger protein 26</td></tr>
<tr><td>RND3</td><td>2032.191</td><td>-0.3599</td><td>0.10303471</td><td>-3.49303</td><td>0.0004776</td><td>0.007602</td><td>Not_Sig</td><td>no</td><td>gi|724851156|ref|XP_010366888.1|</td><td>1242</td><td>2e-161</td><td>PREDICTED: rho-related GTP-binding protein RhoE [Rhinopithecus roxellana]</td><td>gi|742118401|ref|XM_010839964.1|</td><td>2711</td><td>0</td><td>PREDICTED: Bison bison bison Rho family GTPase 3 (RND3), mRNA</td><td>Q6SA80</td><td>482</td><td>8e-163</td><td>Rho-related GTP-binding protein RhoE</td><td>.</td><td>.</td><td>.</td><td>.</td><td>pfam00071</td><td>Ras</td><td>Ras family. Includes sub-families Ras, Rab, Rac, Ral, Ran, Rap Ypt1 and more. Shares P-loop motif with GTP_EFTU, arf and</td><td>GO:0007264|small GTPase mediated signal transduction;</td><td>GO:0070062|extracellular exosome;GO:0005925|focal adhesion;GO:0005622|intracellular;GO:0005886|plasma membrane;</td><td>GO:0005525|GTP binding;</td><td>K07859</td><td>RND3; Rho family GTPase 3</td></tr>
<tr><td>LOC106701696</td><td>1933.813</td><td>-0.41492</td><td>0.118802824</td><td>-3.4925</td><td>0.0004785</td><td>0.007602</td><td>Not_Sig</td><td>no</td><td>gi|555999450|ref|XP_005911426.1|</td><td>538</td><td>1e-64</td><td>PREDICTED: beta-2-microglobulin isoform X3 [Bos mutus]</td><td>gi|942102573|ref|XM_005911364.2|</td><td>1083</td><td>0</td><td>PREDICTED: Bos mutus beta-2-microglobulin (B2M), transcript variant X3, mRNA</td><td>P01888</td><td>210</td><td>1e-65</td><td>Beta-2-microglobulin</td><td>.</td><td>.</td><td>.</td><td>.</td><td>pfam07654</td><td>C1-set</td><td>Immunoglobulin C1-set domain.</td><td>GO:0019731|antibacterial humoral response;GO:0019885|antigen processing and presentation of endogenous peptide antigen via MHC class I;GO:0002481|antigen processing and presentation of exogenous protein antigen via MHC class Ib, TAP-dependent;GO:0071281|cellular response to iron ion;GO:0071222|cellular response to lipopolysaccharide;GO:0050829|defense response to Gram-negative bacterium;GO:0050830|defense response to Gram-positive bacterium;GO:0045087|innate immune response;GO:0055072|iron ion</td><td>GO:0009897|external side of plasma membrane;GO:0070062|extracellular exosome;GO:0005615|extracellular space;GO:0005925|focal</td><td>GO:0001948|glycoprotein binding;</td><td>K08055</td><td>B2M; beta-2-microglobulin</td></tr>
<tr><td>PANK3</td><td>612.8581</td><td>-0.544</td><td>0.155804378</td><td>-3.49153</td><td>0.0004803</td><td>0.007602</td><td>Not_Sig</td><td>no</td><td>gi|685548746|ref|XP_009207802.1|</td><td>1873</td><td>0</td><td>PREDICTED: pantothenate kinase 3 [Papio anubis]</td><td>gi|926718250|ref|XM_005694509.2|</td><td>4790</td><td>0</td><td>PREDICTED: Capra hircus pantothenate kinase 3 (PANK3), mRNA</td><td>Q08DA5</td><td>696</td><td>0</td><td>Pantothenate kinase 3</td><td>SPBC4B4.01c</td><td>166</td><td>3.45e-43</td><td>COG5146</td><td>pfam03630</td><td>Fumble</td><td>Fumble. Fumble is required for cell division in Drosophila. Mutants lacking fumble exhibit abnormalities in bipolar spindle</td><td>GO:0015937|coenzyme A biosynthetic process;</td><td>GO:0005737|cytoplasm;</td><td>GO:0005524|ATP binding;GO:0004594|pantothenate kinase activity;</td><td>K09680</td><td>coaW; type II pantothenate kinase [EC:2.7.1.33]</td></tr>
<tr><td>PLEKHO2</td><td>908.7639</td><td>0.42166</td><td>0.120770487</td><td>3.491416</td><td>0.0004805</td><td>0.007602</td><td>Not_Sig</td><td>no</td><td>gi|555965083|ref|XP_005894602.1|</td><td>1817</td><td>0</td><td>PREDICTED: pleckstrin homology domain-containing family O member 2 [Bos mutus]</td><td>gi|555965082|ref|XM_005894540.1|</td><td>3549</td><td>0</td><td>PREDICTED: Bos mutus pleckstrin homology domain containing, family O member 2 (PLEKHO2), mRNA</td><td>Q32LQ1</td><td>691</td><td>0</td><td>Pleckstrin homology domain-containing family O member 2</td><td>.</td><td>.</td><td>.</td><td>.</td><td>pfam00169</td><td>PH</td><td>PH domain. PH stands for pleckstrin homology.</td><td>.</td><td>.</td><td>.</td><td>.</td><td>.</td></tr>
<tr><td>MLLT6</td><td>565.5008</td><td>0.557646</td><td>0.159738179</td><td>3.490998</td><td>0.0004812</td><td>0.007602</td><td>Not_Sig</td><td>no</td><td>gi|297487000|ref|XP_002695970.1|</td><td>2756</td><td>0</td><td>PREDICTED: protein AF-17 isoform X2 [Bos taurus]</td><td>gi|983002014|ref|XM_581390.6|</td><td>4841</td><td>0</td><td>PREDICTED: Bos taurus myeloid/lymphoid or mixed-lineage leukemia; translocated to, 6 (MLLT6), transcript variant X2, mRNA</td><td>P55198</td><td>1014</td><td>0</td><td>Protein AF-17</td><td>YPR031w</td><td>135</td><td>1.61e-31</td><td>COG5141</td><td>pfam13832</td><td>zf-HC5HC2H_2</td><td>PHD-zinc-finger like domain.</td><td>GO:0006355|regulation of transcription, DNA-templated;</td><td>GO:0005634|nucleus;</td><td>GO:0008270|zinc ion binding;</td><td>.</td><td>.</td></tr>
<tr><td>RAI1</td><td>581.5352</td><td>0.552753</td><td>0.158338169</td><td>3.490964</td><td>0.0004813</td><td>0.007602</td><td>Not_Sig</td><td>no</td><td>gi|741963288|ref|XP_010814407.1|</td><td>8421</td><td>0</td><td>PREDICTED: retinoic acid-induced protein 1 [Bos taurus]</td><td>gi|983001891|ref|XM_010824106.2|</td><td>7053</td><td>0</td><td>PREDICTED: Bos taurus retinoic acid induced 1 (RAI1), transcript variant X6, mRNA</td><td>Q7Z5J4</td><td>2807</td><td>0</td><td>Retinoic acid-induced protein 1</td><td>.</td><td>.</td><td>.</td><td>.</td><td>pfam13771</td><td>zf-HC5HC2H</td><td>PHD-like zinc-binding domain. The members of this family are annotated as containing PHD domain, but the zinc-binding</td><td>GO:0032922|circadian regulation of gene expression;GO:0040015|negative regulation of multicellular organism growth;GO:0045944|positive regulation of transcription from RNA polymerase II promoter;GO:0045893|positive regulation of transcription, DNA-templated;GO:0001501|skeletal system development;</td><td>GO:0005739|mitochondrion;GO:0005654|nucleoplasm;GO:0005634|nucleus;</td><td>GO:0035326|enhancer binding;GO:0003700|transcription factor</td><td>.</td><td>.</td></tr>
<tr><td>S100A4</td><td>2471.971</td><td>-0.56946</td><td>0.163130513</td><td>-3.4908</td><td>0.0004816</td><td>0.007602</td><td>Not_Sig</td><td>no</td><td>gi|795346443|ref|XP_011783512.1|</td><td>562</td><td>1e-69</td><td>PREDICTED: protein S100-A4 isoform X1 [Colobus angolensis palliatus]</td><td>gi|965916249|ref|XM_004002526.3|</td><td>561</td><td>0</td><td>PREDICTED: Ovis aries S100 calcium binding protein A4 (S100A4), mRNA</td><td>P35466</td><td>208</td><td>1e-67</td><td>Protein S100-A4</td><td>.</td><td>.</td><td>.</td><td>.</td><td>pfam01023</td><td>S_100</td><td>S-100/ICaBP type calcium binding domain. The S-100 domain is a subfamily of the EF-hand calcium binding proteins.</td><td>GO:0043123|positive regulation of I-kappaB kinase/NF-kappaB signaling;</td><td>GO:0070062|extracellular exosome;GO:0005615|extracellular space;GO:0005634|nucleus;GO:0048471|perinuclear region of cytoplasm;</td><td>GO:0005509|calcium ion binding;GO:0044822|poly(A) RNA</td><td>.</td><td>.</td></tr>
<tr><td>EIF1</td><td>8037.709</td><td>-0.33104</td><td>0.094834566</td><td>-3.49075</td><td>0.0004817</td><td>0.007602</td><td>Not_Sig</td><td>no</td><td>gi|611991798|ref|XP_001364419.2|</td><td>620</td><td>4e-74</td><td>PREDICTED: uncharacterized protein LOC100012315 [Monodelphis domestica]</td><td>gi|942064731|ref|XM_005899233.2|</td><td>1291</td><td>0</td><td>PREDICTED: Bos mutus eukaryotic translation initiation factor 1 (EIF1), transcript variant X2, mRNA</td><td>Q5RFF4</td><td>204</td><td>8e-63</td><td>Eukaryotic translation initiation factor 1</td><td>YNL244c</td><td>122</td><td>2.70e-33</td><td>COG0023</td><td>pfam01253</td><td>SUI1</td><td>Translation initiation factor SUI1.</td><td>.</td><td>.</td><td>GO:0003743|translation initiation factor activity;</td><td>K03113</td><td>EIF1, SUI1; translation initiation factor 1</td></tr>
<tr><td>EDNRA</td><td>311.0024</td><td>-0.47592</td><td>0.136369978</td><td>-3.48994</td><td>0.0004831</td><td>0.007615</td><td>Not_Sig</td><td>no</td><td>gi|742115006|ref|XP_010837461.1|</td><td>2172</td><td>0</td><td>PREDICTED: endothelin-1 receptor [Bison bison bison]</td><td>gi|555996341|ref|XM_005909847.1|</td><td>3194</td><td>0</td><td>PREDICTED: Bos mutus endothelin receptor type A (EDNRA), mRNA</td><td>P21450</td><td>838</td><td>0</td><td>Endothelin-1 receptor</td><td>.</td><td>.</td><td>.</td><td>.</td><td>pfam00001</td><td>7tm_1</td><td>7 transmembrane receptor (rhodopsin family). This family contains, amongst other G-protein-coupled receptors</td><td>GO:0014824|artery smooth muscle contraction;GO:0048484|enteric nervous system development;GO:0015758|glucose transport;GO:0060322|head development;GO:0007507|heart development;GO:0001701|in utero embryonic development;GO:0030818|negative regulation of cAMP biosynthetic process;GO:0014032|neural crest cell development;GO:0001569|patterning of blood vessels;GO:0008217|regulation of blood pressure;GO:0007585|respiratory gaseous</td><td>GO:0016021|integral component of membrane;GO:0005886|plasma membrane;</td><td>GO:0004962|endothelin receptor activity;</td><td>K04197</td><td>EDNRA; endothelin receptor type A</td></tr>
<tr><td>ARPC3</td><td>1997.423</td><td>-0.47077</td><td>0.134937506</td><td>-3.48882</td><td>0.0004852</td><td>0.007631</td><td>Not_Sig</td><td>no</td><td>gi|77735497|ref|NP_001029443.1|</td><td>957</td><td>2e-128</td><td>actin-related protein 2/3 complex subunit 3 [Bos taurus]</td><td>gi|555978703|ref|XM_005901228.1|</td><td>789</td><td>0</td><td>PREDICTED: Bos mutus actin related protein 2/3 complex, subunit 3, 21kDa (ARPC3), mRNA</td><td>O15145</td><td>373</td><td>8e-130</td><td>Actin-related protein 2/3 complex subunit 3</td><td>.</td><td>.</td><td>.</td><td>.</td><td>pfam04062</td><td>P21-Arc</td><td>ARP2/3 complex ARPC3 (21 kDa) subunit. The seven component ARP2/3 actin-organizing complex is involved in actin assembly and</td><td>GO:0034314|Arp2/3 complex-mediated actin nucleation;GO:1990090|cellular response to nerve growth factor stimulus;GO:0048013|ephrin receptor signaling pathway;GO:0038096|Fc-gamma receptor signaling pathway involved in phagocytosis;GO:0006928|movement of cell or subcellular component;</td><td>GO:0015629|actin cytoskeleton;GO:0005885|Arp2/3 protein complex;GO:0005829|cytosol;GO:0070062|extracellular exosome;GO:0005925|focal</td><td>GO:0005200|structural constituent of cytoskeleton;</td><td>K05756</td><td>ARPC3; actin related protein 2/3 complex, subunit 3</td></tr>
<tr><td>ELOC</td><td>866.7603</td><td>-0.54824</td><td>0.157149776</td><td>-3.48864</td><td>0.0004855</td><td>0.007631</td><td>Not_Sig</td><td>no</td><td>gi|593755294|ref|XP_007116508.1|</td><td>637</td><td>1e-80</td><td>PREDICTED: transcription elongation factor B polypeptide 1 isoform X3 [Physeter catodon]</td><td>gi|982944822|ref|XM_005215527.3|</td><td>816</td><td>0</td><td>PREDICTED: Bos taurus transcription elongation factor B (SIII), polypeptide 1 (15kDa, elongin C) (TCEB1), transcript variant X1, mRNA</td><td>P83941</td><td>234</td><td>2e-76</td><td>Transcription elongation factor B polypeptide 1</td><td>.</td><td>.</td><td>.</td><td>.</td><td>pfam03931</td><td>Skp1_POZ</td><td>Skp1 family, tetramerization domain.</td><td>GO:0032968|positive regulation of transcription elongation from RNA polymerase II promoter;GO:0042787|protein ubiquitination involved in ubiquitin-dependent protein catabolic process;GO:0006351|transcription, DNA-templated;</td><td>GO:0070449|elongin complex;GO:0030891|VCB complex;</td><td>GO:0032403|protein complex binding;</td><td>K03872</td><td>TCEB1; transcription elongation factor B, polypeptide 1</td></tr>
<tr><td>MCOLN1</td><td>946.4381</td><td>0.436056</td><td>0.12506852</td><td>3.486539</td><td>0.0004893</td><td>0.007678</td><td>Not_Sig</td><td>no</td><td>gi|803072987|ref|XP_004008602.2|</td><td>3002</td><td>0</td><td>PREDICTED: mucolipin-1 isoform X1 [Ovis aries]</td><td>gi|555957364|ref|XM_005890753.1|</td><td>2036</td><td>0</td><td>PREDICTED: Bos mutus mucolipin 1 (MCOLN1), mRNA</td><td>Q9GZU1</td><td>1020</td><td>0</td><td>Mucolipin-1</td><td>.</td><td>.</td><td>.</td><td>.</td><td>pfam08016</td><td>PKD_channel</td><td>Polycystin cation channel. This family contains the cation channel region of PKD1 and PKD2 proteins.</td><td>GO:0097352|autophagosome maturation;GO:0070588|calcium ion transmembrane transport;GO:0006812|cation transport;GO:0033572|transferrin transport;</td><td>GO:0005737|cytoplasm;GO:0010008|endosome membrane;GO:0016021|integral component of membrane;GO:0005887|integral</td><td>GO:0005262|calcium channel activity;GO:0005261|cation channel</td><td>K04992</td><td>MCOLN1; mucolipin 1</td></tr>
<tr><td>UBE2S</td><td>1603.218</td><td>0.384899</td><td>0.110414378</td><td>3.485948</td><td>0.0004904</td><td>0.007678</td><td>Not_Sig</td><td>no</td><td>gi|345829|pir||B42856</td><td>875</td><td>1e-113</td><td>ubiquitin carrier protein E2 - human</td><td>gi|402691749|ref|NM_001076472.2|</td><td>1017</td><td>0</td><td>Bos taurus ubiquitin conjugating enzyme E2S (UBE2S), mRNA</td><td>Q16763</td><td>316</td><td>1e-105</td><td>Ubiquitin-conjugating enzyme E2 S</td><td>SPAC11E3.04c</td><td>123</td><td>1.92e-33</td><td>COG5078</td><td>pfam00179</td><td>UQ_con</td><td>Ubiquitin-conjugating enzyme. Proteins destined for proteasome-mediated degradation may be ubiquitinated. Ubiquitination</td><td>GO:0031145|anaphase-promoting complex-dependent proteasomal ubiquitin-dependent protein catabolic process;GO:0051301|cell division;GO:0006464|cellular protein modification process;GO:0010458|exit from mitosis;GO:0010994|free ubiquitin chain polymerization;GO:1904668|positive regulation of ubiquitin protein ligase activity;GO:0070979|protein K11-linked ubiquitination;GO:0044314|protein K27-linked ubiquitination;GO:0035519|protein K29-linked ubiquitination;GO:0085020|protein K6-linked</td><td>GO:0005680|anaphase-promoting complex;GO:0005737|cytoplasm;</td><td>GO:0005524|ATP binding;GO:0061631|ubiquitin conjugating enzyme</td><td>K10583</td><td>UBE2S, E2EPF; ubiquitin-conjugating enzyme E2 S [EC:2.3.2.23]</td></tr>
<tr><td>PEAR1</td><td>372.3533</td><td>0.579097</td><td>0.166125594</td><td>3.485896</td><td>0.0004905</td><td>0.007678</td><td>Not_Sig</td><td>no</td><td>gi|155372315|ref|NP_001094770.1|</td><td>4798</td><td>0</td><td>platelet endothelial aggregation receptor 1 precursor [Bos taurus]</td><td>gi|982916442|ref|XM_010802949.2|</td><td>4155</td><td>0</td><td>PREDICTED: Bos taurus platelet endothelial aggregation receptor 1 (PEAR1), transcript variant X3, mRNA</td><td>Q5VY43</td><td>1587</td><td>0</td><td>Platelet endothelial aggregation receptor 1</td><td>.</td><td>.</td><td>.</td><td>.</td><td>pfam07546</td><td>EMI</td><td>EMI domain. The Pfam alignment is truncated at the C-terminus and does not include the final cysteine defined in Callebaut et al. This is to</td><td>GO:0045746|negative regulation of Notch signaling pathway;GO:0043654|recognition of apoptotic cell;</td><td>GO:0016021|integral component of membrane;GO:0001891|phagocytic cup;</td><td>.</td><td>.</td><td>.</td></tr>
<tr><td>TICAM1</td><td>247.4202</td><td>0.543474</td><td>0.155922467</td><td>3.485541</td><td>0.0004911</td><td>0.007678</td><td>Not_Sig</td><td>no</td><td>gi|528955604|ref|XP_005208987.1|</td><td>2954</td><td>0</td><td>PREDICTED: TIR domain-containing adapter molecule 1 isoform X1 [Bos taurus]</td><td>gi|742217886|ref|XM_010828165.1|</td><td>2728</td><td>0</td><td>PREDICTED: Bison bison bison toll-like receptor adaptor molecule 1 (TICAM1), mRNA</td><td>Q4JF29</td><td>1135</td><td>0</td><td>TIR domain-containing adapter molecule 1</td><td>.</td><td>.</td><td>.</td><td>.</td><td>pfam12721</td><td>RHIM</td><td>RIP homotypic interaction motif. RIP proteins are receptor-interacting serine/threonine-protein kinases or cell death</td><td>GO:0006915|apoptotic process;GO:0051607|defense response to virus;GO:0006954|inflammatory response;GO:0045087|innate immune response;</td><td>GO:0005776|autophagosome;GO:0031410|cytoplasmic vesicle;</td><td>GO:0004871|signal transducer activity;</td><td>K05842</td><td>TRIF; toll-like receptor adapter molecule 1</td></tr>
<tr><td>HYPK</td><td>509.3375</td><td>-0.58791</td><td>0.168765362</td><td>-3.48361</td><td>0.0004947</td><td>0.007723</td><td>DOWN</td><td>yes</td><td>gi|803181374|ref|XP_011954559.1|</td><td>626</td><td>7e-72</td><td>PREDICTED: huntingtin-interacting protein K isoform X1 [Ovis aries]</td><td>gi|226437714|gb|AC150650.7|</td><td>2820</td><td>0</td><td>Bos taurus BAC CH240-447L3 (Children's Hospital Oakland Research Institute Bovine BAC Library complete sequence</td><td>Q2PFU1</td><td>203</td><td>5e-59</td><td>Huntingtin-interacting protein K</td><td>.</td><td>.</td><td>.</td><td>.</td><td>pfam03348</td><td>Serinc</td><td>Serine incorporator (Serinc). This is a family of eukaryotic membrane proteins which incorporate serine into membranes and facilitate the</td><td>.</td><td>GO:0005737|cytoplasm;GO:0005634|nucleus;</td><td>.</td><td>.</td><td>.</td></tr>
<tr><td>BMPR1A</td><td>568.5469</td><td>-0.44993</td><td>0.129191119</td><td>-3.48269</td><td>0.0004964</td><td>0.007736</td><td>Not_Sig</td><td>no</td><td>gi|529011164|ref|XP_005226588.1|</td><td>2824</td><td>0</td><td>PREDICTED: bone morphogenetic protein receptor type-1A isoform X1 [Bos taurus]</td><td>gi|982970660|ref|XM_005226531.3|</td><td>5671</td><td>0</td><td>PREDICTED: Bos taurus bone morphogenetic protein receptor type IA (BMPR1A), transcript variant X1, mRNA</td><td>P36894</td><td>1073</td><td>0</td><td>Bone morphogenetic protein receptor type-1A</td><td>ECU02g0550</td><td>80.9</td><td>1.56e-14</td><td>COG0515</td><td>pfam00069</td><td>Pkinase</td><td>Protein kinase domain.</td><td>GO:0030509|BMP signaling pathway;GO:0061312|BMP signaling pathway involved in heart development;GO:0003161|cardiac conduction system development;GO:0003215|cardiac right ventricle morphogenesis;GO:0071773|cellular response to BMP stimulus;GO:0002062|chondrocyte differentiation;GO:0048589|developmental growth;GO:0035912|dorsal aorta morphogenesis;GO:0009950|dorsal/ventral axis specification;GO:0007398|ectoderm development;GO:0042733|embryonic digit</td><td>GO:0030425|dendrite;GO:0009897|external side of plasma membrane;GO:1990712|HFE-transferrin receptor complex;GO:0016021|integral</td><td>GO:0005524|ATP binding;GO:0098821|BMP receptor activity;GO:0001948</td><td>K04673</td><td>BMPR1A, ALK3; bone morphogenetic protein receptor type-1A</td></tr>
<tr><td>PHRF1</td><td>902.0119</td><td>0.447078</td><td>0.128380841</td><td>3.482435</td><td>0.0004969</td><td>0.007736</td><td>Not_Sig</td><td>no</td><td>gi|440906583|gb|ELR56831.1|</td><td>3728</td><td>0</td><td>PHD and RING finger domain-containing protein 1 [Bos mutus]</td><td>gi|982972191|ref|XM_005227256.3|</td><td>4909</td><td>0</td><td>PREDICTED: Bos taurus PHD and ring finger domains 1 (PHRF1), transcript variant X1, mRNA</td><td>Q9P1Y6</td><td>909</td><td>0</td><td>PHD and RING finger domain-containing protein 1</td><td>.</td><td>.</td><td>.</td><td>.</td><td>pfam00628</td><td>PHD</td><td>PHD-finger. PHD folds into an interleaved type of Zn-finger chelating 2 Zn ions in a similar manner to that of the RING and</td><td>GO:0006397|mRNA processing;GO:0006366|transcription from RNA polymerase II promoter;</td><td>GO:0016020|membrane;</td><td>GO:0070063|RNA polymerase binding;GO:0008270|zinc ion binding;</td><td>K17586</td><td>PHRF1; PHD and RING finger domain-containing protein 1</td></tr>
<tr><td>PIP5K1C</td><td>647.5986</td><td>0.519683</td><td>0.149340589</td><td>3.47985</td><td>0.0005017</td><td>0.0078</td><td>Not_Sig</td><td>no</td><td>gi|803270275|ref|XP_011994870.1|</td><td>2815</td><td>0</td><td>PREDICTED: phosphatidylinositol 4-phosphate 5-kinase type-1 gamma isoform X1 [Ovis aries musimon]</td><td>gi|982930838|ref|XM_005208919.3|</td><td>4698</td><td>0</td><td>PREDICTED: Bos taurus phosphatidylinositol-4-phosphate 5-kinase, type I, gamma (PIP5K1C), transcript variant X6, mRNA</td><td>Q5I6B8</td><td>977</td><td>0</td><td>Phosphatidylinositol 4-phosphate 5-kinase type-1 gamma</td><td>SPAC19G12.14</td><td>172</td><td>3.67e-43</td><td>COG5253</td><td>pfam01504</td><td>PIP5K</td><td>Phosphatidylinositol-4-phosphate 5-Kinase. This family contains a region from the common kinase core found in the type I</td><td>GO:0007155|cell adhesion;GO:0006935|chemotaxis;GO:0006887|exocytosis;GO:0006909|phagocytosis;</td><td>GO:0005737|cytoplasm;GO:0012505|endomembrane system;GO:0005925|focal adhesion;GO:0001891|phagocytic cup;GO:0032587|ruffle</td><td>GO:0016308|1-phosphatidylinositol-4-phosphate 5-kinase</td><td>K00889</td><td>PIP5K; 1-phosphatidylinositol-4-phosphate 5-kinase [EC:2.7.1.68]</td></tr>
<tr><td>UBE2V2</td><td>959.9626</td><td>-0.44876</td><td>0.12899479</td><td>-3.47892</td><td>0.0005034</td><td>0.007815</td><td>Not_Sig</td><td>no</td><td>gi|594079646|ref|XP_006063487.1|</td><td>947</td><td>6e-113</td><td>PREDICTED: ubiquitin-conjugating enzyme E2 variant 2 isoform X2 [Bubalus bubalis]</td><td>gi|742114140|ref|XM_010838940.1|</td><td>4857</td><td>0</td><td>PREDICTED: Bison bison bison ubiquitin-conjugating enzyme E2 variant 2 (UBE2V2), mRNA</td><td>Q5R6C9</td><td>290</td><td>2e-87</td><td>Ubiquitin-conjugating enzyme E2 variant 2</td><td>.</td><td>.</td><td>.</td><td>.</td><td>pfam00179</td><td>UQ_con</td><td>Ubiquitin-conjugating enzyme. Proteins destined for proteasome-mediated degradation may be ubiquitinated. Ubiquitination</td><td>GO:0000729|DNA double-strand break processing;GO:0042275|error-free postreplication DNA repair;</td><td>GO:0070062|extracellular exosome;GO:0005634|nucleus;GO:0031372|UBC13-MMS2 complex;</td><td>GO:0061631|ubiquitin conjugating enzyme activity;</td><td>K10704</td><td>UBE2V; ubiquitin-conjugating enzyme E2 variant</td></tr>
</table>

TVP23C	1136.695	-0.42884	0.123287568	-3.47838	0.0005044	0.007815	Not_Sig	no	gi|114051588|ref|NP_001039617.1|	1100	3e-144	Golgi apparatus membrane protein TVP23 homolog B [Bos taurus]	gi|942030938|ref|XM_005887252.2|	1884	0	PREDICTED: Bos mutus Golgi apparatus membrane protein TVP23 homolog B (LOC102266227), transcript variant X1, mRNA	Q29S14	428	9e-146	Golgi apparatus membrane protein TVP23 homolog B	.	.	.	.	pfam05832	DUF846	Eukaryotic protein of unknown function (DUF846). This family consists of several of unknown function from a variety of	GO:0009306|protein secretion;GO:0016192|vesicle-mediated transport;	GO:0030173|integral component of Golgi membrane;	.	.	.
POMP	1592.886	-0.5105	0.146767457	-3.47827	0.0005047	0.007815	Not_Sig	no	gi|953877504|ref|XP_003363225.3|	713	3e-89	PREDICTED: proteasome maturation protein [Equus caballus]	gi|555980615|ref|XM_005902170.1|	1321	0	PREDICTED: Bos mutus proteasome maturation protein (POMP), transcript variant X2, mRNA	Q3SZV5	255	5e-82	Proteasome maturation protein	.	.	.	.	pfam05348	UMP1	Proteasome maturation factor UMP1. UMP1 is a short-lived chaperone present in the precursor form of the 20S	GO:0043248|proteasome assembly;	GO:0005737|cytoplasm;GO:0005829|cytosol;GO:0005783|endoplasmic reticulum;GO:0005654|nucleoplasm;GO:0005634|nucleus;GO:0031090|organelle	.	K11599	POMP, UMP1; proteasome maturation protein
FRS2	418.8953	-0.45775	0.131653583	-3.4769	0.0005072	0.007844	Not_Sig	no	gi|829886401|ref|XP_012644170.1|	2418	0	PREDICTED: fibroblast growth factor receptor substrate 2 isoform X1 [Microcebus murinus]	gi|742131326|ref|XM_010841544.1|	6381	0	PREDICTED: Bison bison bison fibroblast growth factor receptor substrate 2 (FRS2), transcript variant X5, mRNA	Q8WU20	918	0	Fibroblast growth factor receptor substrate 2	.	.	.	.	pfam02174	IRS	PTB domain (IRS-1 type).	GO:0000187|activation of MAPK activity;GO:0000186|activation of MAPKK activity;GO:0008595|anterior/posterior axis specification, embryo;GO:0008543|fibroblast growth factor receptor signaling pathway;GO:0030900|forebrain development;GO:0007186|G-protein coupled receptor signaling pathway;GO:0001702|gastrulation with mouth forming second;GO:0070307|lens fiber cell development;GO:0000165|MAPK cascade;GO:0007405|neuroblast proliferation;GO:0046619|optic placode formation involved in camera-type	GO:0005911|cell-cell junction;GO:0005768|endosome;GO:0005887|integral component of plasma membrane;GO:0016020|membrane;GO:000	GO:0016303|1-phosphatidylinositol-3-kinase activity;GO:0005104	K12461	FRS2; fibroblast growth factor receptor substrate 2
SUCNR1	533.8846	-0.45802	0.13176511	-3.47604	0.0005089	0.007851	Not_Sig	no	gi|555996873|ref|XP_005910170.1|	1731	0	PREDICTED: LOW QUALITY PROTEIN: succinate receptor 1 [Bos mutus]	gi|803285838|ref|XM_012147170.1|	1708	0	PREDICTED: Ovis aries musimon succinate receptor 1 (SUCNR1), mRNA	Q9BXA5	509	6e-168	Succinate receptor 1	.	.	.	.	pfam00001	7tm_1	7 transmembrane receptor (rhodopsin family). This family contains, amongst other G-protein-coupled receptors	.	GO:0070062|extracellular exosome;GO:0016021|integral component of membrane;GO:0005886|plasma membrane;	GO:0004930|G-protein coupled receptor activity;	K10042	SUCNR1, GPR91; succinate receptor 1
LOC138986311	93.44407	-0.79672	0.229209452	-3.47594	0.0005091	0.007851	DOWN	yes	.	.	.	.	gi|594050678|ref|XR_326758.1|	1382	0	PREDICTED: Bubalus bubalis uncharacterized LOC102400283 (LOC102400283), transcript variant X2, misc_RNA	.	.	.	.	.	.	.	.	pfam11506	DUF3217	Protein of unknown function (DUF3217). This family of proteins with unknown function appears to be restricted to Mycoplasma.	.	.	.	.	.
TMSB10	12099.22	-0.48411	0.139356326	-3.47391	0.0005129	0.0079	Not_Sig	no	gi|119619953|gb|EAW99547.1|	320	7e-34	thymosin, beta 10, isoform CRA_a, partial [Homo sapiens]	gi|594063729|ref|XM_006055831.1|	478	0	PREDICTED: Bubalus bubalis thymosin beta 10 (TMSB10), mRNA	P21752	84.3	4e-20	Thymosin beta-10	.	.	.	.	pfam01290	Thymosin	Thymosin beta-4 family.	GO:0030036|actin cytoskeleton organization;GO:0007015|actin filament organization;GO:0042989|sequestering of actin monomers;	GO:0005737|cytoplasm;GO:0031941|filamentous actin;	GO:0003785|actin monomer binding;	K13785	TMSB10; thymosin beta 10
EEF2	34277.33	0.324308	0.093400515	3.472226	0.0005162	0.007939	Not_Sig	no	gi|115497900|ref|NP_001068589.1|	4462	0	elongation factor 2 [Bos taurus]	gi|942055052|ref|XM_005895956.2|	3176	0	PREDICTED: Bos mutus eukaryotic translation elongation factor 2 (EEF2), mRNA	Q3SYU2	1724	0	Elongation factor 2	YOR133w	1146	0	COG0480	pfam00009	GTP_EFTU	Elongation factor Tu GTP binding domain. This domain contains a P-loop motif, also found in several other families such as pfam00071,	GO:0002244|hematopoietic progenitor cell differentiation;GO:0045727|positive regulation of translation;GO:0006412|translation;	GO:0016235|aggresome;GO:0005737|cytoplasm;GO:0070062|extracellular exosome;GO:0005622|intracellular;GO:0005634|nucleus;GO:0005886|plasma	GO:0005525|GTP binding;GO:0003924|GTPase activity;GO:0044822	K03234	EEF2; elongation factor 2
PHC1	378.3276	0.532536	0.153421408	3.471068	0.0005184	0.007959	Not_Sig	no	gi|329664732|ref|NP_001192685.1|	2200	0	polyhomeotic-like protein 1 [Bos taurus]	gi|982926855|ref|XM_005207072.3|	3912	0	PREDICTED: Bos taurus polyhomeotic homolog 1 (PHC1), transcript variant X3, mRNA	P78364	797	0	Polyhomeotic-like protein 1	.	.	.	.	pfam00536	SAM_1	SAM domain (Sterile alpha motif). It has been suggested that SAM is an evolutionarily conserved protein binding domain that is	GO:0016574|histone ubiquitination;GO:0007275|multicellular organismal development;GO:0016925|protein sumoylation;	GO:0005654|nucleoplasm;GO:0005634|nucleus;GO:0031519|PcG protein complex;GO:0035102|PRC1 complex;	GO:0003677|DNA binding;GO:0008270|zinc ion binding;	K11456	PHC1, EDR1; polyhomeotic-like protein 1
LOC138985915	127.8498	-0.6458	0.186082148	-3.4705	0.0005195	0.007959	DOWN	yes	.	.	.	.	gi|742113633|ref|XR_825165.1|	886	0	PREDICTED: Bison bison bison uncharacterized LOC104987767 (LOC104987767), transcript variant X1, ncRNA	.	.	.	.	.	.	.	.	.	.	.	.	.	.	.	.
CHMP2B	546.8452	-0.55153	0.158932083	-3.4702	0.0005201	0.007959	Not_Sig	no	gi|742182033|ref|XP_010853139.1|	1295	2e-169	PREDICTED: charged multivesicular body protein 2b isoform X1 [Bison bison bison]	gi|942054034|ref|XM_005895592.2|	2465	0	PREDICTED: Bos mutus charged multivesicular body protein 2B (CHMP2B), transcript variant X1, mRNA	Q3SX42	332	4e-106	Charged multivesicular body protein 2b	.	.	.	.	pfam03357	Snf7	Snf7. This family of proteins are involved in protein sorting and transport from the endosome to the vacuole/lysosome in	GO:0000920|cell separation after cytokinesis;GO:0050890|cognition;GO:0007032|endosome organization;GO:0007080|mitotic metaphase plate congression;GO:0070050|neuron cellular homeostasis;GO:0006997|nucleus organization;GO:1902188|positive regulation of viral release from host cell;GO:0015031|protein transport;GO:0010824|regulation of centrosome duplication;GO:1901673|regulation of mitotic spindle assembly;GO:0007034|vacuolar transport;GO:0039702|viral budding via host ESCRT complex;	GO:0005829|cytosol;GO:0000815|ESCRT III complex;GO:0070062|extracellular exosome;GO:0031902|late endosome membrane;GO:0005764|lysosome;GO:0005	.	K12192	CHMP2B; charged multivesicular body protein 2B
CSNK1G2	1580.907	0.427534	0.123204557	3.470113	0.0005202	0.007959	Not_Sig	no	gi|155371843|ref|NP_001094526.1|	2003	0	casein kinase I isoform gamma-2 [Bos taurus]	gi|742142139|ref|XM_010844535.1|	2566	0	PREDICTED: Bison bison bison casein kinase 1, gamma 2 (CSNK1G2), mRNA	P78368	747	0	Casein kinase I isoform gamma-2	.	.	.	.	pfam00069	Pkinase	Protein kinase domain.	GO:0006897|endocytosis;GO:0018105|peptidyl-serine phosphorylation;GO:0046777|protein autophosphorylation;GO:0006468|protein phosphorylation;GO:0008360|regulation of cell shape;GO:0007165|signal transduction;GO:0030148|sphingolipid biosynthetic process;GO:0016055|Wnt signaling pathway;	GO:0005737|cytoplasm;GO:0005829|cytosol;GO:0016020|membrane;GO:0005634|nucleus;	GO:0005524|ATP binding;GO:0001948|glycoprotein binding;GO:0000287	K08958	CSNK1G; casein kinase 1, gamma [EC:2.7.11.1]
WDR90	377.6288	0.619069	0.178440737	3.469327	0.0005218	0.007972	UP	yes	gi|741973744|ref|XP_010817453.1|	9022	0	PREDICTED: WD repeat-containing protein 90 isoform X3 [Bos taurus]	gi|983006699|ref|XM_015469239.1|	6110	0	PREDICTED: Bos taurus WD repeat domain 90 (WDR90), transcript variant X3, mRNA	Q6ZPG2	2263	0	WD repeat-containing protein 90	.	.	.	.	pfam05018	DUF667	Protein of unknown function (DUF667). This family of proteins are highly conserved in eukaryotes. Some proteins in the	.	.	.	.	.
POLR2F	471.9146	-0.4711	0.135810903	-3.46881	0.0005228	0.007977	Not_Sig	no	gi|521022702|gb|EPQ04490.1|	488	2e-58	DNA-directed RNA polymerases I, II, and III subunit RPABC2 [Myotis brandtii]	gi|742144428|ref|XM_010845086.1|	590	0	PREDICTED: Bison bison bison polymerase (RNA) II (DNA directed) polypeptide F (POLR2F), mRNA	Q5R592	185	9e-58	DNA-directed RNA polymerases I, II, and III subunit RPABC2	SPCC1020.04c	130	2.71e-38	COG1758	pfam01192	RNA_pol_Rpb6	RNA polymerase Rpb6. Rpb6 is an essential subunit in the eukaryotic polymerases Pol I, II and III. This family also contains the bacterial	GO:0006366|transcription from RNA polymerase II promoter;	GO:0005665|DNA-directed RNA polymerase II, core complex;GO:0005634|nucleus;	GO:0003677|DNA binding;GO:0003899|DNA-directed RNA polymerase activity;	K03014	RPB6, POLR2F; DNA-directed RNA polymerases I, II, and III subunit RPABC2
SLC30A7	792.1183	-0.45276	0.130560449	-3.4678	0.0005247	0.007993	Not_Sig	no	gi|139948426|ref|NP_001077229.1|	1658	0	zinc transporter 7 [Bos taurus]	gi|742232680|ref|XM_010830969.1|	7654	0	PREDICTED: Bison bison bison solute carrier family 30 (zinc transporter), member 7 (SLC30A7), mRNA	A4IFD7	643	0	Zinc transporter 7	SPAC17D4.03c	120	2.27e-26	COG1230	pfam01545	Cation_efflux	Cation efflux family. Members of this family are integral membrane proteins, that are found to increase tolerance to divalent	GO:0061088|regulation of sequestering of zinc ion;GO:0010043|response to zinc ion;GO:0032119|sequestering of zinc ion;	GO:0016023|cytoplasmic membrane-bounded vesicle;GO:0070062|extracellular exosome;GO:0005794|Golgi apparatus;GO:0016021|integral component	GO:0005385|zinc ion transmembrane transporter activity;	K14692	SLC30A5_7, ZNT5_7, MTP, MSC2; solute carrier family 30 (zinc transporter),
RAB1B	1712.358	0.339652	0.097951201	3.467565	0.0005252	0.007993	Not_Sig	no	gi|664703871|ref|XP_008531408.1|	1150	6e-151	PREDICTED: ras-related protein Rab-1B [Equus przewalskii]	gi|942050094|ref|XM_005894018.2|	1974	0	PREDICTED: Bos mutus RAB1B, member RAS oncogene family (RAB1B), transcript variant X1, mRNA	Q2HJH2	417	2e-141	Ras-related protein Rab-1B	.	.	.	.	pfam00071	Ras	Ras family. Includes sub-families Ras, Rab, Rac, Ral, Ran, Rap Ypt1 and more. Shares P-loop motif with GTP_EFTU, arf and	GO:0006914|autophagy;GO:0006888|ER to Golgi vesicle-mediated transport;GO:0015031|protein transport;GO:0007264|small GTPase mediated signal transduction;	GO:0005789|endoplasmic reticulum membrane;GO:0000139|Golgi membrane;GO:0034045|pre-autophagosomal structure membrane;	GO:0005525|GTP binding;	K07875	RAB1B; Ras-related protein Rab-1B
SGTB	168.0244	-0.61734	0.178058684	-3.46703	0.0005262	0.007998	DOWN	yes	gi|300796037|ref|NP_001179720.1|	1471	0	small glutamine-rich tetratricopeptide repeat-containing protein beta [Bos taurus]	gi|742148962|ref|XM_010845527.1|	4133	0	PREDICTED: Bison bison bison small glutamine-rich tetratricopeptide repeat (TPR)-containing, beta (SGTB), mRNA	Q96EQ0	558	0	Small glutamine-rich tetratricopeptide repeat-containing protein beta	SPAC17G6.19c	142	1.94e-36	COG0457	pfam13414	TPR_11	TPR repeat.	.	.	.	.	.
SAMD4B	1855.773	0.515248	0.148638518	3.466448	0.0005274	0.008005	Not_Sig	no	gi|594090649|ref|XP_006068725.1|	3113	0	PREDICTED: protein Smaug homolog 2 isoform X1 [Bubalus bubalis]	gi|965830909|ref|XM_012131165.2|	4327	0	PREDICTED: Ovis aries musimon sterile alpha motif domain containing 4B (SAMD4B), transcript variant X4, mRNA	Q5PRF9	1176	0	Protein Smaug homolog 2	.	.	.	.	pfam00536	SAM_1	SAM domain (Sterile alpha motif). It has been suggested that SAM is an evolutionarily conserved protein binding domain that is	GO:0017148|negative regulation of translation;GO:0000289|nuclear-transcribed mRNA poly(A) tail shortening;GO:0043488|regulation of mRNA stability;GO:0006355|regulation of transcription, DNA-templated;GO:0006351|transcription, DNA-templated;	GO:0005737|cytoplasm;GO:0000932|cytoplasmic mRNA processing body;GO:0005634|nucleus;	GO:0003729|mRNA binding;GO:0044822|poly(A) RNA binding;GO:0030371	.	.
POLR2K	277.4959	-0.68066	0.196377124	-3.46606	0.0005281	0.008006	DOWN	yes	gi|817282185|ref|XP_012311254.1|	323	1e-33	PREDICTED: DNA-directed RNA polymerases I, II, and III subunit RPABC4 isoform X1 [Aotus nancymaae]	gi|746816125|ref|NM_001303536.1|	1027	0	Bos taurus polymerase (RNA) II (DNA directed) polypeptide K, 7.0kDa (POLR2K), transcript variant 1, mRNA	P53803	121	1e-32	DNA-directed RNA polymerases I, II, and III subunit RPABC4	SPBC19C2.03	67.4	7.43e-14	COG1996	pfam03604	DNA_RNApol_7kD	DNA directed RNA polymerase, 7 kDa subunit.	GO:0006370|7-methylguanosine mRNA capping;GO:0008543|fibroblast growth factor receptor signaling pathway;GO:0010467|gene expression;GO:0031047|gene silencing by RNA;GO:0000398|mRNA splicing, via spliceosome;GO:0045815|positive regulation of gene expression, epigenetic;GO:0032481|positive regulation of type I interferon production;GO:0050434|positive regulation of viral transcription;GO:0006356|regulation of transcription from RNA polymerase I promoter;GO:0042795|snRNA transcription from RNA	GO:0005829|cytosol;GO:0005736|DNA-directed RNA polymerase I complex;GO:0005665|DNA-directed RNA polymerase II, core	GO:0003677|DNA binding;GO:0003899|DNA-directed RNA polymerase	K03009	RPB12, POLR2K; DNA-directed RNA polymerases I, II, and III subunit RPABC4
RNF19A	1446.754	-0.40534	0.116961066	-3.46556	0.0005291	0.00801	Not_Sig	no	gi|440913292|gb|ELR62759.1|	3796	0	E3 ubiquitin-protein ligase RNF19A [Bos mutus]	gi|300797582|ref|NM_001191331.1|	4319	0	Bos taurus ring finger protein 19A, RBR E3 ubiquitin protein ligase (RNF19A), mRNA	Q2VJ60	1413	0	E3 ubiquitin-protein ligase RNF19A	.	.	.	.	pfam01485	IBR	IBR domain. The IBR (In Between Ring fingers) domain is often found to occur between pairs of ring fingers (pfam00097). This domain	GO:0032436|positive regulation of proteasomal ubiquitin-dependent protein catabolic process;GO:0000209|protein polyubiquitination;GO:0042787|protein ubiquitination involved in ubiquitin-dependent protein catabolic process;	GO:0005813|centrosome;GO:0005737|cytoplasm;GO:0016021|integral component of membrane;GO:0000151|ubiquitin ligase complex;	GO:0016874|ligase activity;GO:0031624|ubiquitin conjugating enzyme	K11972	RNF19A; E3 ubiquitin-protein ligase RNF19A [EC:2.3.2.27]
RALGDS	554.9597	0.56194	0.162276981	3.462847	0.0005345	0.008081	Not_Sig	no	gi|803226444|ref|XP_011973532.1|	3894	0	PREDICTED: ral guanine nucleotide dissociation stimulator isoform X4 [Ovis aries musimon]	gi|942075806|ref|XM_005902755.2|	3175	0	PREDICTED: Bos mutus ral guanine nucleotide dissociation stimulator (RALGDS), mRNA	Q03386	1325	0	Ral guanine nucleotide dissociation stimulator	.	.	.	.	pfam00617	RasGEF	RasGEF domain. Guanine nucleotide exchange factor for Ras-like small GTPases.	GO:0007265|Ras protein signal transduction;GO:0007264|small GTPase mediated signal transduction;	GO:0005829|cytosol;GO:0005634|nucleus;	GO:0008321|Ral guanyl-nucleotide exchange factor activity;	K08732	RALGDS; ral guanine nucleotide dissociation stimulator
RNF125	298.3081	-0.5	0.144434967	-3.46173	0.0005367	0.008081	Not_Sig	no	gi|329663542|ref|NP_001192788.1|	1236	1e-153	E3 ubiquitin-protein ligase RNF125 [Bos taurus]	gi|982965133|ref|XM_005223982.3|	5127	0	PREDICTED: Bos taurus ring finger protein 125, E3 ubiquitin protein ligase (RNF125), transcript variant X1, mRNA	Q95KF1	440	2e-140	E3 ubiquitin-protein ligase RNF125	SPBC17A3.10	51.6	9.99e-06	COG5574	pfam05605	zf-Di19	Drought induced 19 protein (Di19), zinc-binding. This family consists of several drought induced 19 (Di19) like proteins. Di19 has been found	GO:0002250|adaptive immune response;GO:0016567|protein ubiquitination;	GO:0000139|Golgi membrane;GO:0043231|intracellular membrane-bounded organelle;	GO:0036435|K48-linked polyubiquitin binding;GO:0016874|ligase	K12170	RNF125; E3 ubiquitin-protein ligase RNF125 [EC:2.3.2.27]
XYLT2	345.8242	0.480041	0.138673141	3.461669	0.0005368	0.008081	Not_Sig	no	gi|926717805|ref|XP_013827988.1|	4240	0	PREDICTED: xylosyltransferase 2 [Capra hircus]	gi|965824202|ref|XM_012127613.2|	2728	0	PREDICTED: Ovis aries musimon xylosyltransferase II (XYLT2), transcript variant X1, mRNA	Q5QQ50	1573	0	Xylosyltransferase 2	.	.	.	.	pfam12529	Xylo_C	Xylosyltransferase C terminal. This domain family is found in eukaryotes, and is typically between 169 and 183 amino acids	GO:0030206|chondroitin sulfate biosynthetic process;GO:0050650|chondroitin sulfate proteoglycan biosynthetic process;GO:0015012|heparan sulfate proteoglycan biosynthetic process;GO:0030210|heparin biosynthetic process;	GO:0005789|endoplasmic reticulum membrane;GO:0000139|Golgi membrane;GO:0016021|integral component of membrane;	GO:0008375|acetylglucosaminyltransferase activity;GO:0030158	K00771	XYLT; protein xylosyltransferase [EC:2.4.2.26]
CHTF18	216.7019	0.584622	0.168885649	3.461644	0.0005369	0.008081	Not_Sig	no	gi|555949359|ref|XP_005886890.1|	4357	0	PREDICTED: chromosome transmission fidelity protein 18 homolog [Bos mutus]	gi|942029206|ref|XM_005886828.2|	3183	0	PREDICTED: Bos mutus chromosome transmission fidelity factor 18 (CHTF18), mRNA	Q8WVB6	1223	0	Chromosome transmission fidelity protein 18 homolog	.	.	.	.	pfam00004	AAA	ATPase family associated with various cellular activities (AAA). AAA family proteins often perform chaperone-like functions that	GO:0007049|cell cycle;GO:0006260|DNA replication;GO:1900264|positive regulation of DNA-directed DNA polymerase activity;	GO:0031390|Ctf18 RFC-like complex;GO:0005737|cytoplasm;GO:0016020|membrane;GO:0005654|nucleoplasm;	GO:0005524|ATP binding;GO:0003677|DNA binding;	K11269	CTF18, CHL12; chromosome transmission fidelity protein 18
SSPN	474.3621	-0.42565	0.122987627	-3.46089	0.0005384	0.008081	Not_Sig	no	gi|555981176|ref|XP_005902506.1|	1051	3e-127	PREDICTED: sarcospan [Bos mutus]	gi|742186794|ref|XM_010856003.1|	4600	0	PREDICTED: Bison bison bison sarcospan (SSPN), transcript variant X1, mRNA	Q14714	369	4e-114	Sarcospan	.	.	.	.	pfam04103	CD20	CD20-like family. This family includes the CD20 protein and the beta subunit of the high affinity receptor for IgE Fc. The high	GO:0007155|cell adhesion;GO:0006936|muscle contraction;	GO:0030054|cell junction;GO:0016010|dystrophin-associated glycoprotein complex;GO:0005887|integral component of plasma	.	.	.
LOC102271974	884.6017	0.474704	0.137176162	3.460544	0.0005391	0.008081	Not_Sig	no	gi|635043000|ref|XP_007997068.1|	377	6e-40	PREDICTED: growth-regulated protein homolog gamma-like [Chlorocebus sabaeus]	gi|965840567|ref|XM_012135519.2|	1079	0	PREDICTED: Ovis aries musimon growth-regulated alpha protein (LOC101104661), transcript variant X4, mRNA	O46676	144	4e-40	Growth-regulated protein homolog alpha	.	.	.	.	pfam00048	IL8	Small cytokines (intecrine/chemokine), interleukin-8 like. Includes a number of secreted growth factors and	GO:0060326|cell chemotaxis;GO:0006955|immune response;GO:0006954|inflammatory response;	GO:0005615|extracellular space;	.	K05505	CXCL1_2_3, GRO; C-X-C motif chemokine 1/2/3
MAZ	1385.504	0.425515	0.122986986	3.45984	0.0005405	0.008081	Not_Sig	no	gi|803240201|ref|XP_011980196.1|	1506	0	PREDICTED: myc-associated zinc finger protein isoform X3 [Ovis aries musimon]	gi|965818379|ref|XM_012124806.2|	2332	0	PREDICTED: Ovis aries musimon MYC-associated zinc finger protein (purine-binding transcription factor) (MAZ), transcript variant X3, mRNA	P56270	564	0	Myc-associated zinc finger protein	.	.	.	.	pfam13465	zf-H2C2_2	Zinc-finger double domain.	GO:0006355|regulation of transcription, DNA-templated;GO:0006369|termination of RNA polymerase II transcription;GO:0006367|transcription initiation from RNA polymerase II promoter;	GO:0005634|nucleus;	GO:0046872|metal ion binding;GO:0044822|poly(A) RNA	.	.
LDLR	8589.088	0.398116	0.115068253	3.459821	0.0005405	0.008081	Not_Sig	no	gi|262073126|ref|NP_001160002.1|	4204	0	low-density lipoprotein receptor precursor [Bos taurus]	gi|742218555|ref|XM_010828334.1|	4291	0	PREDICTED: Bison bison bison low density lipoprotein receptor (LDLR), mRNA	Q28832	1424	0	Low-density lipoprotein receptor (Fragment)	.	.	.	.	pfam00058	Ldl_recept_b	Low-density lipoprotein receptor repeat class B. This domain is also known as the YWTD motif after the most conserved region of the	GO:0008203|cholesterol metabolic process;GO:0006897|endocytosis;GO:0006869|lipid transport;GO:0045056|transcytosis;	GO:0009986|cell surface;GO:0005905|coated pit;GO:0005769|early endosome;GO:0005794|Golgi	GO:0005509|calcium ion binding;	K12473	LDLR; low-density lipoprotein receptor
CHPF	4491.979	0.463392	0.133943418	3.459609	0.000541	0.008081	Not_Sig	no	gi|329664692|ref|NP_001192680.1|	3636	0	chondroitin sulfate synthase 2 [Bos taurus]	gi|942038070|ref|XM_005889696.2|	2998	0	PREDICTED: Bos mutus chondroitin polymerizing factor (CHPF), mRNA	Q8IZ52	1309	0	Chondroitin sulfate synthase 2	.	.	.	.	pfam05679	CHGN	Chondroitin N-acetylgalactosaminyltransferase.	GO:0030206|chondroitin sulfate biosynthetic process;	GO:0005829|cytosol;GO:0032580|Golgi cisterna membrane;GO:0000139|Golgi membrane;GO:0016021|integral component of	GO:0047238|glucuronosyl-N-acetylgalactosaminyl-proteoglycan 4-	K00747	CHPF; chondroitin polymerizing factor [EC:2.4.1.175 2.4.1.226]
DNAJB14	345.518	-0.52775	0.152569247	-3.4591	0.000542	0.008081	Not_Sig	no	gi|594056711|ref|XP_006052549.1|	1742	0	PREDICTED: dnaJ homolog subfamily B member 14 isoform X1 [Bubalus bubalis]	gi|982927933|ref|XM_010805949.2|	4468	0	PREDICTED: Bos taurus DnaJ heat shock protein family (Hsp40) member B14 (DNAJB14), transcript variant X1, mRNA	Q0IIE8	673	0	DnaJ homolog subfamily B member 14	.	.	.	.	pfam09320	DUF1977	Domain of unknown function (DUF1977). Members of this family of functionally uncharacterized domains are predominantly found	.	GO:0005789|endoplasmic reticulum membrane;GO:0016021|integral component of membrane;	.	K09520	DNAJB14; DnaJ homolog subfamily B member 14
CYB5R3	6221.779	0.38388	0.110980641	3.458982	0.0005422	0.008081	Not_Sig	no	gi|157151718|ref|NP_001096720.1|	1485	0	NADH-cytochrome b5 reductase 3 [Bos taurus]	gi|157151717|ref|NM_001103250.1|	1860	0	Bos taurus cytochrome b5 reductase 3 (CYB5R3), mRNA	P07514	576	0	NADH-cytochrome b5 reductase 3	SPCC970.03	164	6.01e-45	COG0543	pfam00970	FAD_binding_6	Oxidoreductase FAD-binding domain.	GO:0006695|cholesterol biosynthetic process;	GO:0005789|endoplasmic reticulum membrane;GO:0005741|mitochondrial outer membrane;GO:0005739|mitochondrion;	GO:0004128|cytochrome-b5 reductase activity, acting on NAD(P)H;GO:007194	K00326	E1.6.2.2; cytochrome-b5 reductase [EC:1.6.2.2]
TP53	661.1327	0.431321	0.124696171	3.458976	0.0005422	0.008081	Not_Sig	no	gi|555965613|ref|XP_005894864.1|	1819	0	PREDICTED: cellular tumor antigen p53 [Bos mutus]	gi|942051950|ref|XM_005894802.2|	2204	0	PREDICTED: Bos mutus tumor protein p53 (TP53), mRNA	P67939	702	0	Cellular tumor antigen p53	.	.	.	.	pfam00870	P53	P53 DNA-binding domain. This family contains one anomalous member, viz: Zea mays (Q6JAD8). This sequence is identical to	GO:0007569|cell aging;GO:0007050|cell cycle arrest;GO:0035690|cellular response to drug;GO:0042149|cellular response to glucose starvation;GO:0071456|cellular response to hypoxia;GO:0071479|cellular response to ionizing radiation;GO:0034644|cellular response to UV;GO:0031497|chromatin assembly;GO:0048512|circadian behavior;GO:0006978|DNA damage response, signal transduction by p53 class mediator resulting in transcription of p21 class mediator;GO:0000733|DNA strand renaturation;GO:0043153|entrainment of circadian clock by	GO:0000785|chromatin;GO:0005737|cytoplasm;GO:0005829|cytosol;GO:0005783|endoplasmic reticulum;GO:0005759|mitochondrial	GO:0005524|ATP binding;GO:0003682|chromatin binding;GO:0005507	K04451	TP53, P53; tumor protein p53
MPZL3	82.20767	-0.77535	0.224212176	-3.4581	0.000544	0.008097	DOWN	yes	gi|148226845|ref|NP_001091520.1|	1178	5e-149	myelin protein zero-like protein 3 precursor [Bos taurus]	gi|742230430|ref|XM_010830412.1|	3633	0	PREDICTED: Bison bison bison myelin protein zero-like 3 (MPZL3), mRNA	A5D7C3	458	2e-150	Myelin protein zero-like protein 3	.	.	.	.	pfam07686	V-set	Immunoglobulin V-set domain. This domain is found in antibodies as well as neural protein P0 and CTL4 amongst others.	GO:0007155|cell adhesion;	GO:0016021|integral component of membrane;	.	.	.
TNK2	1513.716	0.41049	0.118784116	3.455766	0.0005487	0.008156	Not_Sig	no	gi|982910358|ref|XP_015326899.1|	4837	0	PREDICTED: activated CDC42 kinase 1 isoform X2 [Bos taurus]	gi|982910355|ref|XM_010801156.2|	4325	0	PREDICTED: Bos taurus tyrosine kinase, non-receptor, 2 (TNK2), transcript variant X1, mRNA	Q17R13	1808	0	Activated CDC42 kinase 1	.	.	.	.	pfam07714	Pkinase_Tyr	Protein tyrosine kinase.	GO:0030154|cell differentiation;GO:0016477|cell migration;GO:0006897|endocytosis;GO:0045087|innate immune response;GO:0038083|peptidyl-tyrosine autophosphorylation;GO:0016310|phosphorylation;GO:0042127|regulation of cell proliferation;GO:0007169|transmembrane receptor protein tyrosine kinase signaling pathway;	GO:0005912|adherens junction;GO:0005905|coated pit;GO:0030136|clathrin-coated vesicle;GO:0005737|cytoplasm;GO:0030659	GO:0005524|ATP binding;GO:0046872|metal ion binding;GO:0004715	K08886	TNK2, ACK1; activated CDC42 kinase 1 [EC:2.7.10.2 2.7.11.1]
TECR	776.6066	0.527402	0.152648219	3.455018	0.0005503	0.008157	Not_Sig	no	gi|528770105|gb|EPY89764.1|	1726	0	trans-2,3-enoyl-CoA reductase [Camelus ferus]	gi|402744923|ref|NM_001034748.2|	1029	0	Bos taurus trans-2,3-enoyl-CoA reductase (TECR), mRNA	Q3ZCD7	632	0	Very-long-chain enoyl-CoA reductase	.	.	.	.	pfam02544	Steroid_dh	3-oxo-5-alpha-steroid 4-dehydrogenase. This family consists of 3-oxo-5-alpha-steroid 4-dehydrogenases, EC:1.3.99.5	GO:0030497|fatty acid elongation;GO:0006694|steroid biosynthetic process;GO:0042761|very long-chain fatty acid biosynthetic process;	GO:0030176|integral component of endoplasmic reticulum membrane;GO:0005634|nucleus;	GO:0016627|oxidoreductase activity, acting on the CH-CH group of donors;	K10258	TER, TSC13, CER10; very-long-chain enoyl-CoA reductase [EC:1.3.1.93]
POMGNT2	324.9208	0.510122	0.147647272	3.455007	0.0005503	0.008157	Not_Sig	no	gi|926720332|ref|XP_013828909.1|	3081	0	PREDICTED: protein O-linked-mannose beta-1,4-N-acetylglucosaminyltransferase 2 [Capra hircus]	gi|942041768|ref|XM_005891082.2|	2518	0	PREDICTED: Bos mutus protein O-linked mannose N-acetylglucosaminyltransferase 2 (beta 1,4-) (POMGNT2), mRNA	Q5NDF2	1132	0	Protein O-linked-mannose beta-1,4-N-acetylglucosaminyltransferase 2	.	.	.	.	pfam04577	DUF563	Protein of unknown function (DUF563). Family of uncharacterized proteins.	GO:0001764|neuron migration;GO:0006493|protein O-linked glycosylation;GO:0035269|protein O-linked mannosylation;	GO:0005783|endoplasmic reticulum;GO:0005789|endoplasmic reticulum membrane;GO:0016021|integral component of membrane;	GO:0008375|acetylglucosaminyltransferase activity;GO:0097363	K18207	GTDC2; protein O-mannose beta-1,4-N-acetylglucosaminyltransferase
TCF20	842.7723	0.40758	0.117991022	3.454333	0.0005517	0.008157	Not_Sig	no	gi|528951591|ref|XP_005207453.1|	8423	0	PREDICTED: transcription factor 20 isoform X4 [Bos taurus]	gi|741928519|ref|XM_005207396.2|	7261	0	PREDICTED: Bos taurus transcription factor 20 (AR1) (TCF20), transcript variant X4, mRNA	Q9UGU0	3035	0	Transcription factor 20	.	.	.	.	pfam13771	zf-HC5HC2H	PHD-like zinc-binding domain. The members of this family are annotated as containing PHD domain, but the zinc-binding	GO:0045944|positive regulation of transcription from RNA polymerase II promoter;GO:0006355|regulation of transcription, DNA-templated;GO:0006351|transcription, DNA-templated;	GO:0005654|nucleoplasm;GO:0005634|nucleus;	GO:0003677|DNA binding;GO:0044822|poly(A) RNA binding;GO:0003713	.	.
ID3	825.0614	0.39389	0.114031551	3.454217	0.0005519	0.008157	Not_Sig	no	gi|295148214|ref|NP_001171149.1|	632	7e-79	DNA-binding protein inhibitor ID-3 [Ovis aries]	gi|742228917|ref|XM_010830035.1|	958	0	PREDICTED: Bison bison bison inhibitor of DNA binding 3, dominant negative helix-loop-helix protein (ID3), mRNA	Q5E981	204	7e-64	DNA-binding protein inhibitor ID-3	.	.	.	.	pfam00010	HLH	Helix-loop-helix DNA-binding domain.	GO:0072750|cellular response to leptomycin B;GO:0007417|central nervous system development;GO:0007623|circadian rhythm;GO:0030855|epithelial cell differentiation;GO:0007507|heart development;GO:0001656|metanephros development;GO:0007517|muscle organ development;GO:0045662|negative regulation of myoblast differentiation;GO:0045668|negative regulation of osteoblast differentiation;GO:0043433|negative regulation of sequence-specific DNA binding transcription factor	GO:0005737|cytoplasm;GO:0015630|microtubule cytoskeleton;GO:0005654|nucleoplasm;	GO:1901707|leptomycin B binding;GO:0003700|transcription factor	K17694	ID3; DNA-binding protein inhibitor ID3
UBALD1	260.7972	0.603982	0.174863958	3.454011	0.0005523	0.008157	UP	yes	gi|594061754|ref|XP_006054950.1|	430	1e-49	PREDICTED: UBA-like domain-containing protein 1 [Bubalus bubalis]	gi|594061753|ref|XM_006054888.1|	675	0	PREDICTED: Bubalus bubalis UBA-like domain containing 1 (UBALD1), mRNA	Q8TB05	152	2e-44	UBA-like domain-containing protein 1	.	.	.	.	pfam14555	UBA_4	UBA-like domain.	.	.	.	.	.
EFHD2	2330.652	0.556727	0.161222858	3.453151	0.0005541	0.008168	Not_Sig	no	gi|594099446|ref|XP_006072856.1|	903	3e-112	PREDICTED: EF-hand domain-containing protein D2 [Bubalus bubalis]	gi|157074011|ref|NM_001103245.1|	2334	0	Bos taurus EF-hand domain family member D2 (EFHD2), mRNA	A5D7A0	352	2e-113	EF-hand domain-containing protein D2	.	.	.	.	pfam13499	EF-hand_7	EF-hand domain pair.	.	GO:0045121|membrane raft;	GO:0005509|calcium ion binding;	.	.

LOC138991289	39.02792	-1.13313	0.328182652	-3.45275	0.0005549	0.008168	DOWN	yes	.	.	.	.	gi|942041369|ref|XR_001351298.1|	77	2e-31	PREDICTED: Bos mutus uncharacterized LOC106700620 (LOC106700620), ncRNA	.	.	.	.	.	.	.	.	pfam02449	Glyco_hydro_42	Beta-galactosidase. This group of beta-galactosidase enzymes belong to the glycosyl hydrolase 42 family. The enzyme catalyses the	.	.	.	.	.
ATP6V0A1	616.5517	0.467228	0.135326707	3.45259	0.0005552	0.008168	Not_Sig	no	gi|548509103|ref|XP_005693909.1|	4281	0	PREDICTED: V-type proton ATPase 116 kDa subunit a isoform 1 isoform X2 [Capra hircus]	gi|157278896|gb|BC153213.1|	4017	0	Bos taurus ATPase, H+ transporting, lysosomal V0 subunit a1, mRNA (cDNA clone MGC:139181 IMAGE:8089880), complete cds	Q29466	1619	0	V-type proton ATPase 116 kDa subunit a isoform 1	YOR270c	612	0	COG1269	pfam01496	V_ATPase_I	V-type ATPase 116kDa subunit family. This family consists of the 116kDa V-type ATPase (vacuolar (H+)-ATPases) subunits, as well as	GO:0015991|ATP hydrolysis coupled proton transport;GO:0015986|ATP synthesis coupled proton transport;GO:0007035|vacuolar acidification;GO:0070072|vacuolar proton-transporting V-type ATPase complex assembly;	GO:0030659|cytoplasmic vesicle membrane;GO:0016021|integral component of membrane;GO:0042470|melanosome;GO:0	GO:0051117|ATPase binding;GO:0046961|proton-	K02154	ATPeV0A, ATP6N; V-type H+-transporting ATPase subunit a
CSRP2	3127.019	-0.30724	0.088995555	-3.45226	0.0005559	0.008168	Not_Sig	no	gi|431892075|gb|ELK02522.1|	909	7e-120	Cysteine and glycine-rich protein 2 [Pteropus alecto]	gi|742092565|ref|XM_010833871.1|	933	0	PREDICTED: Bison bison bison cysteine and glycine-rich protein 2 (CSRP2), mRNA	Q16527	352	7e-121	Cysteine and glycine-rich protein 2	.	.	.	.	pfam00412	LIM	LIM domain. This family represents two copies of the LIM structural domain.	GO:0030154|cell differentiation;GO:0007275|multicellular organismal development;	GO:0005925|focal adhesion;GO:0005634|nucleus;	GO:0008270|zinc ion binding;	K09377	CSRP; cysteine and glycine-rich protein
PDE4A	622.1603	0.494651	0.143369029	3.450197	0.0005602	0.00822	Not_Sig	no	gi|594089985|ref|XP_006068406.1|	3634	0	PREDICTED: cAMP-specific 3',5'-cyclic phosphodiesterase 4A isoform X1 [Bubalus bubalis]	gi|965841832|ref|XM_012136021.2|	4326	0	PREDICTED: Ovis aries musimon cAMP-specific 3',5'-cyclic phosphodiesterase 4A (LOC101117505), transcript variant X8, mRNA	P27815	1237	0	cAMP-specific 3',5'-cyclic phosphodiesterase 4A	.	.	.	.	pfam00233	PDEase_I	3'5'-cyclic nucleotide phosphodiesterase.	GO:0006198|cAMP catabolic process;GO:0035690|cellular response to drug;GO:0043949|regulation of cAMP-mediated signaling;GO:0010738|regulation of protein kinase A signaling;GO:0007608|sensory perception of smell;GO:0007165|signal transduction;	GO:0005829|cytosol;GO:0016020|membrane;GO:0048471|perinuclear region of cytoplasm;GO:0032587|ruffle membrane;	GO:0004115|3',5'-cyclic-AMP phosphodiesterase activity;GO:0030552	K13293	PDE4; cAMP-specific phosphodiesterase 4 [EC:3.1.4.53]
TNPO2	1664.109	0.3569	0.103462335	3.449563	0.0005615	0.008229	Not_Sig	no	gi|440902066|gb|ELR52909.1|	4720	0	Transportin-2, partial [Bos mutus]	gi|982930085|ref|XM_005208659.3|	5188	0	PREDICTED: Bos taurus transportin 2 (TNPO2), transcript variant X1, mRNA	Q99LG2	1717	0	Transportin-2	.	.	.	.	pfam13513	HEAT_EZ	HEAT-like repeat. The HEAT repeat family is related to armadillo/beta-catenin-like repeats (see pfam00514). These EZ repeats are	GO:0051148|negative regulation of muscle cell differentiation;GO:0006607|NLS-bearing protein import into nucleus;GO:0006606|protein import into nucleus;GO:0000059|protein import into nucleus, docking;GO:0000060|protein import into nucleus, translocation;GO:0006610|ribosomal protein import into nucleus;	GO:0005737|cytoplasm;GO:0031965|nuclear membrane;GO:0034399|nuclear periphery;	GO:0008139|nuclear localization sequence binding;GO:0008565	K18727	TNPO2; transportin-2
LOC102269751	371.4618	-0.51097	0.148140714	-3.44923	0.0005622	0.008229	Not_Sig	no	gi|555978778|ref|XP_005901327.1|	741	8e-96	PREDICTED: histone H3.3 [Bos mutus]	gi|136256445|ref|NM_001014389.2|	826	0	Bos taurus H3 histone, family 3A (H3F3A), mRNA	Q6P823	273	3e-91	Histone H3.3	.	.	.	.	pfam00125	Histone	Core histone H2A/H2B/H3/H4.	GO:0006334|nucleosome assembly;	GO:0000786|nucleosome;GO:0005634|nucleus;	GO:0031492|nucleosomal DNA binding;	K11253	H3; histone H3
GNG5	1015.593	-0.4277	0.124075473	-3.4471	0.0005666	0.008283	Not_Sig	no	gi|829964768|ref|XP_012603023.1|	378	5e-43	PREDICTED: guanine nucleotide-binding protein G(I)/G(S)/G(O) subunit gamma-5 [Microcebus murinus]	gi|942031597|ref|XM_005887414.2|	598	0	PREDICTED: Bos mutus guanine nucleotide binding protein (G protein), gamma 5 (GNG5), mRNA	P63219	137	5e-40	Guanine nucleotide-binding protein G(I)/G(S)/G(O) subunit gamma-5	.	.	.	.	pfam00631	G-gamma	GGL domain. G-protein gamma like domains (GGL) are found in the gamma subunit of the heterotrimeric G protein complex	GO:0007186|G-protein coupled receptor signaling pathway;	GO:0070062|extracellular exosome;GO:0031680|G-protein beta/gamma-subunit complex;GO:0005834|heterotrimeric G-	GO:0004871|signal transducer activity;	K04542	GNG5; guanine nucleotide-binding protein G(I)/G(S)/G(O)
RCHY1	415.7319	-0.52702	0.152906845	-3.44665	0.0005676	0.008287	Not_Sig	no	gi|139948305|ref|NP_001077223.1|	1115	8e-144	RING finger and CHY zinc finger domain-containing protein 1 [Bos taurus]	gi|555958407|ref|XM_005891262.1|	1013	0	PREDICTED: Bos mutus ring finger and CHY zinc finger domain containing 1, E3 ubiquitin protein ligase (RCHY1), transcript variant X1, mRNA	Q96PM5	409	8e-136	RING finger and CHY zinc finger domain-containing protein 1	.	.	.	.	pfam05495	zf-CHY	CHY zinc finger. This family of domains are likely to bind to zinc ions. They contain many conserved cysteine and histidine residues. We	GO:0070987|error-free translesion synthesis;GO:0032436|positive regulation of proteasomal ubiquitin-dependent protein catabolic process;GO:0031398|positive regulation of protein ubiquitination;GO:0051865|protein autoubiquitination;GO:0016567|protein ubiquitination;GO:0042787|protein ubiquitination involved in ubiquitin-dependent protein catabolic process;	GO:0005737|cytoplasm;GO:0016607|nuclear speck;GO:0005654|nucleoplasm;GO:0005634|nucleus;GO:0000151|ubiquitin ligase	GO:0016874|ligase activity;GO:0002039|p53 binding;GO:0042803	K10144	RCHY1, PIRH2; RING finger and CHY zinc finger domain-containing protein 1
FLNA	47521.69	0.360925	0.1047478	3.445659	0.0005697	0.008305	Not_Sig	no	gi|330688463|ref|NP_001193443.1|	13527	0	filamin-A [Bos taurus]	gi|330688462|ref|NM_001206514.1|	8290	0	Bos taurus filamin A, alpha (FLNA), mRNA	P21333	5055	0	Filamin-A	ECU09g0290	147	1.46e-35	COG5069	pfam00630	Filamin	Filamin/ABP280 repeat.	GO:0051764|actin crosslink formation;GO:0031532|actin cytoskeleton reorganization;GO:0007195|adenylate cyclase-inhibiting dopamine receptor signaling pathway;GO:0034329|cell junction assembly;GO:0042384|cilium assembly;GO:0051220|cytoplasmic sequestering of protein;GO:0045184|establishment of protein localization;GO:0090307|mitotic spindle assembly;GO:0042789|mRNA transcription from RNA polymerase II promoter;GO:0043066|negative regulation of apoptotic process;GO:0042177|negative regulation of protein catabolic	GO:0015629|actin cytoskeleton;GO:0005884|actin filament;GO:0097440|apical dendrite;GO:0005913|cell-cell adherens	GO:0051015|actin filament binding;GO:0098641|cadherin binding	K04437	FLNA; filamin
UBAP2	1303.504	0.426631	0.123843844	3.444909	0.0005713	0.008305	Not_Sig	no	gi|982934001|ref|XP_015328095.1|	4050	0	PREDICTED: ubiquitin-associated protein 2 isoform X1 [Bos taurus]	gi|982934000|ref|XM_015472609.1|	4263	0	PREDICTED: Bos taurus ubiquitin associated protein 2 (UBAP2), transcript variant X1, mRNA	Q5T6F2	1295	0	Ubiquitin-associated protein 2	.	.	.	.	pfam12478	DUF3697	Ubiquitin-associated protein 2. This domain family is found in eukaryotes, and is approximately 30 amino acids in length. The	.	GO:0005913|cell-cell adherens junction;	GO:0098641|cadherin binding involved in cell-cell adhesion;GO:00448	.	.
FGFRL1	99.72481	0.85685	0.248734893	3.444832	0.0005714	0.008305	UP	yes	gi|329664606|ref|NP_001192925.1|	2129	0	fibroblast growth factor receptor-like 1 precursor [Bos taurus]	gi|329664605|ref|NM_001205996.1|	1753	0	Bos taurus fibroblast growth factor receptor-like 1 (FGFRL1), mRNA	Q8N441	757	0	Fibroblast growth factor receptor-like 1	.	.	.	.	pfam07679	I-set	Immunoglobulin I-set domain.	GO:0098742|cell-cell adhesion via plasma-membrane adhesion molecules;GO:0060539|diaphragm development;GO:0008543|fibroblast growth factor receptor signaling pathway;GO:0003179|heart valve morphogenesis;GO:0008285|negative regulation of cell proliferation;GO:0051260|protein homooligomerization;GO:0001501|skeletal system development;GO:0060412|ventricular septum morphogenesis;	GO:0044291|cell-cell contact zone;GO:0005794|Golgi apparatus;GO:0016021|integral component of membrane;GO:0005886|plasma	GO:0005007|fibroblast growth factor-activated receptor activity;GO:0008201	.	.
PKMYT1	209.8175	0.579143	0.16812693	3.444676	0.0005717	0.008305	Not_Sig	no	gi|555949229|ref|XP_005886829.1|	2063	0	PREDICTED: membrane-associated tyrosine- and threonine-specific cdc2-inhibitory kinase isoform X1 [Bos mutus]	gi|983006847|ref|XM_005196844.3|	1943	0	PREDICTED: Bos taurus protein kinase, membrane associated tyrosine/threonine 1 (PKMYT1), transcript variant X1, mRNA	Q99640	692	0	Membrane-associated tyrosine- and threonine-specific cdc2-inhibitory kinase	.	.	.	.	pfam00069	Pkinase	Protein kinase domain.	GO:0000082|G1/S transition of mitotic cell cycle;GO:0000086|G2/M transition of mitotic cell cycle;GO:0007067|mitotic nuclear division;GO:0010923|negative regulation of phosphatase activity;GO:0051726|regulation of cell cycle;GO:0000079|regulation of cyclin-dependent protein serine/threonine kinase activity;GO:0007088|regulation of mitotic nuclear division;	GO:0005829|cytosol;GO:0005783|endoplasmic reticulum;GO:0005789|endoplasmic reticulum membrane;GO:0005794|Golgi apparatus;GO:0000139|Golgi	GO:0005524|ATP binding;GO:0016301|kinase activity;GO:0046872	K06633	PKMYT; membrane-associated tyrosine- and threonine-specific cdc2-
ROR2	1208.959	0.396733	0.115204651	3.443725	0.0005738	0.008315	Not_Sig	no	gi|965918824|ref|XP_014948251.1|	4624	0	PREDICTED: LOW QUALITY PROTEIN: tyrosine-protein kinase transmembrane receptor ROR2 isoform X1 [Ovis aries]	gi|219521803|ref|NM_001105464.2|	3700	0	Bos taurus receptor tyrosine kinase-like orphan receptor 2 (ROR2), mRNA	Q01974	1645	0	Tyrosine-protein kinase transmembrane receptor ROR2	.	.	.	.	pfam07714	Pkinase_Tyr	Protein tyrosine kinase.	GO:0030509|BMP signaling pathway;GO:0001502|cartilage condensation;GO:0045165|cell fate commitment;GO:0042733|embryonic digit morphogenesis;GO:0030538|embryonic genitalia morphogenesis;GO:0042472|inner ear morphogenesis;GO:0007254|JNK cascade;GO:0007275|multicellular organismal development;GO:0090090|negative regulation of canonical Wnt signaling pathway;GO:0008285|negative regulation of cell proliferation;GO:0090263|positive regulation of canonical Wnt signaling	GO:0030669|clathrin-coated endocytic vesicle membrane;GO:0005887|integral component of plasma membrane;GO:0005886|plasma membrane;	GO:0005524|ATP binding;GO:0046872|metal ion binding;GO:0004714	K05123	ROR2, NTRKR2; receptor tyrosine kinase-like orphan receptor 2
LRFN4	167.3859	0.753621	0.218882771	3.443036	0.0005752	0.008315	UP	yes	gi|329664624|ref|NP_001193183.1|	2774	0	leucine-rich repeat and fibronectin type-III domain-containing protein 4 precursor [Bos taurus]	gi|982971966|ref|XM_005227110.3|	2214	0	PREDICTED: Bos taurus leucine rich repeat and fibronectin type III domain containing 4 (LRFN4), transcript variant X1, mRNA	Q6PJG9	1035	0	Leucine-rich repeat and fibronectin type-III domain-containing protein 4	SPy1798	63.5	2.08e-09	COG4886	pfam07679	I-set	Immunoglobulin I-set domain.	GO:0007409|axonogenesis;GO:0007268|synaptic transmission;	GO:0009986|cell surface;GO:0016021|integral component of membrane;GO:0005886|plasma membrane;	.	K16357	LRFN4, SALM3; leucine-rich repeat and fibronectin type-III domain-containing
SNRPD2	728.4696	-0.49207	0.142917392	-3.44303	0.0005752	0.008315	Not_Sig	no	gi|817277638|ref|XP_012308924.1|	516	1e-63	PREDICTED: small nuclear ribonucleoprotein Sm D2 [Aotus nancymaae]	gi|942068120|ref|XM_005900332.2|	559	0	PREDICTED: Bos mutus small nuclear ribonucleoprotein D2 polypeptide 16.5kDa (SNRPD2), transcript variant X1, mRNA	P62317	185	4e-58	Small nuclear ribonucleoprotein Sm D2	SPAC2C4.03c	146	1.00e-44	COG1958	pfam01423	LSM	LSM domain. The LSM domain contains Sm proteins as well as other related LSM (Like Sm) proteins. The U1, U2, U4/U6, and	GO:0000387|spliceosomal snRNP assembly;	GO:0071013|catalytic step 2 spliceosome;GO:0005829|cytosol;GO:0070062|extracellular exosome;GO:0034709|methylosome;GO:00	GO:0044822|poly(A) RNA binding;	K11096	SNRPD2, SMD2; small nuclear ribonucleoprotein D2
SCP2	715.85	-0.52936	0.153750896	-3.443	0.0005753	0.008315	Not_Sig	no	gi|296489064|tpg|DAA31177.1|	2873	0	TPA: non-specific lipid-transfer protein [Bos taurus]	gi|402745034|ref|NM_001033990.3|	2564	0	Bos taurus sterol carrier protein 2 (SCP2), mRNA	P07857	1087	0	Non-specific lipid-transfer protein	.	.	.	.	pfam02036	SCP2	SCP-2 sterol transfer family. This domain is involved in binding sterols. It is found in the SCP2 protein, as well as the C terminus	GO:1901373|lipid hydroperoxide transport;GO:0015914|phospholipid transport;GO:0032385|positive regulation of intracellular cholesterol transport;GO:0006694|steroid biosynthetic process;	GO:0005739|mitochondrion;GO:0005777|peroxisome;	GO:0033814|propanoyl-CoA C-acyltransferase activity;GO:0032934	K08764	SCP2, SCPX; sterol carrier protein 2 [EC:2.3.1.176]
ANKRD13B	437.6998	0.610749	0.177414344	3.442499	0.0005764	0.00832	UP	yes	gi|465993629|ref|XP_004267151.1|	2888	0	PREDICTED: ankyrin repeat domain-containing protein 13B [Orcinus orca]	gi|594035042|ref|XM_006042146.1|	2972	0	PREDICTED: Bubalus bubalis ankyrin repeat domain 13B (ANKRD13B), transcript variant X1, mRNA	Q86YJ7	1107	0	Ankyrin repeat domain-containing protein 13B	.	.	.	.	pfam11904	GPCR_chapero_1	GPCR-chaperone. This domain, and the associated ANK family repeat pfam00023 domain, together act as a chaperone for biogenesis and	.	GO:0005737|cytoplasm;GO:0005769|early endosome;GO:0043231|intracellular membrane-bounded organelle;GO:0005770|late	.	.	.
REXO1	763.4075	0.426235	0.123844242	3.441705	0.0005781	0.008334	Not_Sig	no	gi|528956468|ref|XP_005209339.1|	5324	0	PREDICTED: RNA exonuclease 1 homolog isoform X2 [Bos taurus]	gi|982931768|ref|XM_005209282.3|	4388	0	PREDICTED: Bos taurus REX1, RNA exonuclease 1 homolog (REXO1), transcript variant X2, mRNA	Q8N1G1	1619	0	RNA exonuclease 1 homolog	SPAC1782.08c	154	2.07e-38	COG0847	pfam00929	RNase_T	Exonuclease. This family includes a variety of exonuclease proteins, such as ribonuclease T and the epsilon subunit of DNA polymerase	.	GO:0005634|nucleus;	GO:0004527|exonuclease activity;GO:0003676|nucleic acid	K14570	REX1, REXO1, RNH70; RNA exonuclease 1 [EC:3.1.-.-]
PIGY	434.9042	-0.44722	0.129981554	-3.44064	0.0005803	0.008356	Not_Sig	no	gi|400529122|ref|NP_001257875.1|	362	2e-40	phosphatidylinositol N-acetylglucosaminyltransferase subunit Y [Bos taurus]	gi|942059686|ref|XM_014478807.1|	789	0	PREDICTED: Bos mutus phosphatidylinositol glycan anchor biosynthesis, class Y (PIGY), mRNA	P0C1N9	144	8e-42	Phosphatidylinositol N-acetylglucosaminyltransferase subunit Y	.	.	.	.	pfam15159	PIG-Y	Phosphatidylinositol N-acetylglucosaminyltransferase subunit Y. This family of proteins represents subunit Y of the GPI-N-	GO:0006506|GPI anchor biosynthetic process;	GO:0005789|endoplasmic reticulum membrane;GO:0000506|glycosylphosphatidylinositol-N-acetylglucosaminyltransferase (GPI-GnT) complex;GO:0016021|integral	.	K11001	PIGY; phosphatidylinositol glycan anchor biosynthesis, class Y
FAM193A	386.8997	0.553564	0.16099947	3.438297	0.0005854	0.008418	Not_Sig	no	gi|594103374|ref|XP_006074701.1|	5582	0	PREDICTED: protein FAM193A isoform X6 [Bubalus bubalis]	gi|594103373|ref|XM_006074639.1|	4465	0	PREDICTED: Bubalus bubalis family with sequence similarity 193, member A (FAM193A), transcript variant X6, mRNA	P78312	1473	0	Protein FAM193A	.	.	.	.	pfam04094	DUF390	Protein of unknown function (DUF390). This is a family of long proteins currently only found in the rice genome. They have no	.	.	.	.	.
LOC102275456	1213.006	-0.44344	0.129005712	-3.43737	0.0005874	0.008436	Not_Sig	no	gi|555987419|ref|XP_005905561.1|	492	2e-58	PREDICTED: transmembrane protein 14C [Bos mutus]	gi|942083949|ref|XM_005905499.2|	947	0	PREDICTED: Bos mutus transmembrane protein 14C (TMEM14C), mRNA	Q3ZCI1	191	6e-59	Transmembrane protein 14C	.	.	.	.	pfam03647	Tmemb_14	Transmembrane proteins 14C. This family of short membrane proteins are as yet uncharacterized.	GO:0030218|erythrocyte differentiation;GO:0006783|heme biosynthetic process;GO:0006839|mitochondrial transport;GO:0070453|regulation of heme biosynthetic process;	GO:0016021|integral component of membrane;GO:0005743|mitochondrial inner membrane;	.	.	.
NPRL3	172.8456	0.746834	0.21728921	3.437051	0.0005881	0.008436	UP	yes	gi|329663182|ref|NP_001193244.1|	2745	0	nitrogen permease regulator 3-like protein [Bos taurus]	gi|555949400|ref|XM_005886849.1|	2117	0	PREDICTED: Bos mutus NPR3-like, GATOR1 complex subunit (NPRL3), transcript variant X1, mRNA	Q8VIJ8	1005	0	Nitrogen permease regulator 3-like protein	.	.	.	.	pfam03666	NPR3	Nitrogen Permease regulator of amino acid transport activity 3. This family, also known in yeasts as Rmd11, complexes with NPR2,	GO:0035909|aorta morphogenesis;GO:0048738|cardiac muscle tissue development;GO:0034198|cellular response to amino acid starvation;GO:0032007|negative regulation of TOR signaling;GO:0060021|palate development;GO:2000785|regulation of autophagosome assembly;GO:0038202|TORC1 signaling;GO:0003281|ventricular septum development;	GO:1990130|Iml1 complex;GO:0005773|vacuole;	GO:0005096|GTPase activator activity;	.	.
ABHD14A	458.7802	0.424845	0.12363201	3.436369	0.0005896	0.008447	Not_Sig	no	gi|296474932|tpg|DAA17047.1|	1250	1e-167	TPA: abhydrolase domain containing 14A [Bos taurus]	gi|742171203|ref|XM_010851772.1|	1409	0	PREDICTED: Bison bison bison abhydrolase domain containing 14A (ABHD14A), transcript variant X4, mRNA	Q9BUJ0	388	3e-131	Protein ABHD14A	SSO2262	87.8	2.38e-19	COG0596	pfam12695	Abhydrolase_5	Alpha/beta hydrolase family. This family contains a diverse range of alpha/beta hydrolase enzymes.	.	GO:0005737|cytoplasm;GO:0070062|extracellular exosome;GO:0016021|integral component of membrane;	GO:0016787|hydrolase activity;	K13706	ABHD14; abhydrolase domain-containing protein 14
CREB1	334.5438	-0.50957	0.148468326	-3.43218	0.0005988	0.008559	Not_Sig	no	gi|470643309|ref|XP_004326198.1|	1451	7e-180	PREDICTED: cyclic AMP-responsive element-binding protein 1 isoform 1 [Tursiops truncatus]	gi|594054240|ref|XM_006051328.1|	6826	0	PREDICTED: Bubalus bubalis cAMP responsive element binding protein 1 (CREB1), transcript variant X1, mRNA	P16220	561	0	Cyclic AMP-responsive element-binding protein 1	.	.	.	.	pfam02173	pKID	pKID domain. CBP and P300 bind to the pKID (phosphorylated kinase-inducible-domain) domain of CREB.	GO:0007568|aging;GO:0007411|axon guidance;GO:0071398|cellular response to fatty acid;GO:0035729|cellular response to hepatocyte growth factor stimulus;GO:1990314|cellular response to insulin-like growth factor stimulus;GO:1990090|cellular response to nerve growth factor stimulus;GO:0036120|cellular response to platelet-derived growth factor stimulus;GO:0071294|cellular response to zinc ion;GO:0034670|chemotaxis to arachidonic acid;GO:0007623|circadian rhythm;GO:0007595|lactation;GO:0060430|lung saccule	GO:1990589|ATF4-CREB1 transcription factor complex;GO:0030424|axon;GO:0005759|mitochondrial matrix;GO:0005719|nuclear	GO:0035497|cAMP response element binding;GO:0019899|enzyme	K05870	CREB1; cyclic AMP-responsive element-binding protein 1
SOX13	95.61383	0.721761	0.210297217	3.432099	0.0005989	0.008559	UP	yes	gi|528979194|ref|XP_005216752.1|	2661	0	PREDICTED: transcription factor SOX-13 isoform X2 [Bos taurus]	gi|594077340|ref|XM_006062328.1|	3818	0	PREDICTED: Bubalus bubalis SRY (sex determining region Y)-box 13 (SOX13), transcript variant X2, mRNA	Q9UN79	881	0	Transcription factor SOX-13	.	.	.	.	pfam00505	HMG_box	HMG (high mobility group) box.	GO:0009653|anatomical structure morphogenesis;GO:0045586|regulation of gamma-delta T cell differentiation;GO:0006351|transcription, DNA-templated;	GO:0005654|nucleoplasm;GO:0005634|nucleus;	GO:0000977|RNA polymerase II regulatory region sequence-specific	K09269	SOX5_6_13; transcription factor SOX5/6/13 (SOX group D)
CCNB1IP1	264.5271	-0.5614	0.163649994	-3.43049	0.0006025	0.008586	Not_Sig	no	gi|602701514|ref|XP_007462599.1|	1501	0	PREDICTED: E3 ubiquitin-protein ligase CCNB1IP1 [Lipotes vexillifer]	gi|594048039|ref|XM_006048363.1|	2364	0	PREDICTED: Bubalus bubalis cyclin B1 interacting protein 1, E3 ubiquitin protein ligase (CCNB1IP1), transcript variant X1, mRNA	Q9NPC3	555	1e-178	E3 ubiquitin-protein ligase CCNB1IP1	.	.	.	.	pfam14634	zf-RING_5	zinc-RING finger domain.	GO:0001825|blastocyst formation;GO:0051026|chiasma assembly;GO:0016567|protein ubiquitination;GO:0007131|reciprocal meiotic recombination;GO:0007286|spermatid development;	GO:0000795|synaptonemal complex;	GO:0016874|ligase activity;GO:0061630|ubiquitin protein ligase	K10639	CCNB1IP1, HEI10; E3 ubiquitin-protein ligase CCNP1IP1 [EC:2.3.2.27]
RPS25	3314.133	-0.47586	0.138726092	-3.43023	0.0006031	0.008586	Not_Sig	no	gi|444706073|gb|ELW47435.1|	351	4e-45	40S ribosomal protein S25 [Tupaia chinensis]	gi|942058228|ref|XM_005897133.2|	571	0	PREDICTED: Bos mutus ribosomal protein S25 (RPS25), mRNA	Q6Q311	150	1e-44	40S ribosomal protein S25	YGR027c	75.5	2.13e-17	COG4901	pfam03297	Ribosomal_S25	S25 ribosomal protein.	.	GO:0005840|ribosome;	.	K02975	RP-S25e, RPS25; small subunit ribosomal protein S25e
DDX27	667.8821	0.40084	0.116865486	3.429924	0.0006037	0.008586	Not_Sig	no	gi|122692565|ref|NP_001073740.1|	3039	0	probable ATP-dependent RNA helicase DDX27 [Bos taurus]	gi|742097596|ref|XM_010835080.1|	2479	0	PREDICTED: Bison bison bison DEAD (Asp-Glu-Ala-Asp) box polypeptide 27 (DDX27), mRNA	A1A4H6	1175	0	Probable ATP-dependent RNA helicase DDX27	SPAC30D11.03	389	8.12e-123	COG0513	pfam00270	DEAD	DEAD/DEAH box helicase. Members of this family include the DEAD and DEAH box helicases. Helicases are involved in	GO:0010501|RNA secondary structure unwinding;GO:0006364|rRNA processing;	GO:0005694|chromosome;GO:0005730|nucleolus;	GO:0005524|ATP binding;GO:0004004|ATP-dependent RNA helicase	K13181	DDX27, DRS1; ATP-dependent RNA helicase DDX27 [EC:3.6.4.13]
ARPP19	1374.07	-0.42808	0.124807624	-3.4299	0.0006038	0.008586	Not_Sig	no	gi|731276812|ref|XP_010607367.1|	509	5e-56	PREDICTED: cAMP-regulated phosphoprotein 19 isoform X1 [Fukomys damarensis]	gi|942097275|ref|XM_005909672.2|	4486	0	PREDICTED: Bos mutus cAMP-regulated phosphoprotein, 19kDa (ARPP19), transcript variant X1, mRNA	Q28055	200	2e-57	cAMP-regulated phosphoprotein 19	.	.	.	.	pfam04667	Endosulfine	cAMP-regulated phosphoprotein/endosulfine conserved region. Conserved region found in both cAMP-	GO:0051301|cell division;GO:0000086|G2/M transition of mitotic cell cycle;GO:0007067|mitotic nuclear division;	GO:0005737|cytoplasm;	GO:0019212|phosphatase inhibitor activity;GO:0051721|protein	.	.
KBTBD3	62.62205	-0.85323	0.248828585	-3.42899	0.0006058	0.008604	DOWN	yes	gi|528915028|ref|XP_005194613.1|	2902	0	PREDICTED: kelch repeat and BTB domain-containing protein 3 isoform X2 [Bos taurus]	gi|982994850|ref|XM_602528.9|	7802	0	PREDICTED: Bos taurus kelch repeat and BTB (POZ) domain containing 3 (KBTBD3), transcript variant X1, mRNA	Q8NAB2	1036	0	Kelch repeat and BTB domain-containing protein 3	.	.	.	.	pfam07707	BACK	BTB And C-terminal Kelch. This domain is found associated with pfam00651 and pfam01344. The BACK domain is found juxtaposed	GO:0016567|protein ubiquitination;	GO:0031463|Cul3-RING ubiquitin ligase complex;	.	K10471	KBTBD3; kelch repeat and BTB domain-containing protein 3
CREBZF	1070.407	-0.41217	0.120234575	-3.42806	0.0006079	0.008623	Not_Sig	no	gi|529011554|ref|XP_005226735.1|	925	6e-108	PREDICTED: CREB/ATF bZIP transcription factor isoform X1 [Bos taurus]	gi|594103138|ref|XM_006074541.1|	4479	0	PREDICTED: Bubalus bubalis CREB/ATF bZIP transcription factor (CREBZF), transcript variant X4, mRNA	Q9NS37	310	5e-92	CREB/ATF bZIP transcription factor	.	.	.	.	pfam00170	bZIP_1	bZIP transcription factor. The Pfam entry includes the basic region and the leucine zipper region.	GO:0045814|negative regulation of gene expression, epigenetic;GO:0045892|negative regulation of transcription, DNA-templated;GO:0051090|regulation of sequence-specific DNA binding transcription factor activity;GO:0009615|response to virus;GO:0006351|transcription, DNA-templated;	GO:0005634|nucleus;	GO:0003677|DNA binding;GO:0042802|identical protein binding;GO:0043565	.	.
LOC106701327	1036.353	-0.43189	0.12601633	-3.42728	0.0006097	0.008634	Not_Sig	no	.	.	.	.	gi|942034687|ref|XR_001351797.1|	865	0	PREDICTED: Bos mutus uncharacterized LOC106701327 (LOC106701327), ncRNA	.	.	.	.	.	.	.	.	pfam07942	N2227	N2227-like protein. This family features sequences that are similar to a region of hypothetical yeast gene product N2227. This is	.	.	.	.	.
SSR4	2158.297	-0.41579	0.121333447	-3.42688	0.0006106	0.008634	Not_Sig	no	gi|426257396|ref|XP_004022313.1|	808	1e-106	PREDICTED: translocon-associated protein subunit delta isoform X1 [Ovis aries]	gi|529014106|ref|XM_005227671.1|	651	0	PREDICTED: Bos taurus signal sequence receptor, delta (SSR4), transcript variant X1, mRNA	Q2TBX5	315	5e-108	Translocon-associated protein subunit delta	.	.	.	.	pfam05404	TRAP-delta	Translocon-associated protein, delta subunit precursor (TRAP-delta). This family consists of several eukaryotic translocon-	.	GO:0005789|endoplasmic reticulum membrane;GO:0070062|extracellular exosome;GO:0016021|integral component of membrane;	.	K04571	SSR4; translocon-associated protein subunit delta
UBE4B	2860.209	0.397977	0.116139433	3.426721	0.0006109	0.008634	Not_Sig	no	gi|742164450|ref|XP_010848217.1|	5691	0	PREDICTED: ubiquitin conjugation factor E4 B isoform X1 [Bison bison bison]	gi|965791227|ref|XM_012111262.2|	5720	0	PREDICTED: Ovis aries musimon ubiquitination factor E4B (UBE4B), transcript variant X2, mRNA	O95155	1738	0	Ubiquitin conjugation factor E4 B	SPAC20H4.10	382	6.07e-111	COG5113	pfam10408	Ufd2P_core	Ubiquitin elongating factor core. This is the most conserved part of the core region of Ufd2P ubiquitin elongating factor or E4, running	GO:0008626|granzyme-mediated apoptotic signaling pathway;GO:0031175|neuron projection development;GO:0043161|proteasome-mediated ubiquitin-dependent protein catabolic process;GO:0051865|protein autoubiquitination;GO:0006513|protein monoubiquitination;GO:0000209|protein polyubiquitination;GO:0042787|protein ubiquitination involved in ubiquitin-dependent protein catabolic process;GO:0034976|response to endoplasmic reticulum stress;GO:0009411|response to UV;GO:0003222|ventricular trabecula	GO:0005737|cytoplasm;GO:0005634|nucleus;GO:0000151|ubiquitin ligase complex;	GO:0005524|ATP binding;GO:0019899|enzyme binding;GO:0016874	K10597	UBE4B, UFD2; ubiquitin conjugation factor E4 B [EC:2.3.2.27]
DCAF15	444.4068	0.533978	0.155905737	3.425003	0.0006148	0.008663	Not_Sig	no	gi|77736315|ref|NP_001029857.1|	2651	0	DDB1- and CUL4-associated factor 15 [Bos taurus]	gi|742184779|ref|XM_010855440.1|	2269	0	PREDICTED: Bison bison bison DDB1 and CUL4 associated factor 15 (DCAF15), mRNA	Q3SZD5	1025	0	DDB1- and CUL4-associated factor 15	.	.	.	.	pfam14939	DCAF15_WD40	DDB1-and CUL4-substrate receptor 15, WD repeat. DCAFs, Ddb1- and Cul4-associated factors, are substrate receptors for the Cul4-	GO:0016567|protein ubiquitination;	.	.	K11791	DCAF15; DDB1- and CUL4-associated factor 15
TERF1	436.8197	-0.43127	0.125917597	-3.42499	0.0006148	0.008663	Not_Sig	no	gi|296480547|tpg|DAA22662.1|	2235	0	TPA: telomeric repeat binding factor (NIMA-interacting) 1 [Bos taurus]	gi|942089443|ref|XM_005907228.2|	2313	0	PREDICTED: Bos mutus telomeric repeat binding factor (NIMA-interacting) 1 (TERF1), transcript variant X1, mRNA	P54274	708	0	Telomeric repeat-binding factor 1	.	.	.	.	pfam08558	TRF	Telomere repeat binding factor (TRF). Telomere repeat binding factor (TRF) family proteins are important for the regulation of	GO:0051301|cell division;GO:0000086|G2/M transition of mitotic cell cycle;GO:0045141|meiotic telomere clustering;GO:0007067|mitotic nuclear division;GO:0007094|mitotic spindle assembly checkpoint;GO:0008156|negative regulation of DNA replication;GO:0051974|negative regulation of telomerase activity;GO:0032214|negative regulation of telomere maintenance via semi-conservative replication;GO:0032211|negative regulation of telomere maintenance via telomerase;GO:0043065|positive regulation of apoptotic	GO:0000781|chromosome, telomeric region;GO:0005737|cytoplasm;GO:0000784|nuclear chromosome, telomeric region;GO:0000783|nuclear telomere cap	GO:0003677|DNA binding;GO:0008301|DNA binding, bending;GO:000369	K11110	TERF1, TRF1; telomeric repeat-binding factor 1
HTR2A	149.8959	-0.59024	0.172361064	-3.42446	0.000616	0.008663	DOWN	yes	gi|555969557|ref|XP_005896803.1|	2418	0	PREDICTED: 5-hydroxytryptamine receptor 2A [Bos mutus]	gi|742181280|ref|XM_010854645.1|	5100	0	PREDICTED: Bison bison bison 5-hydroxytryptamine (serotonin) receptor 2A, G protein-coupled (HTR2A), mRNA	Q75Z89	886	0	5-hydroxytryptamine receptor 2A	.	.	.	.	pfam00001	7tm_1	7 transmembrane receptor (rhodopsin family). This family contains, amongst other G-protein-coupled receptors	GO:0007202|activation of phospholipase C activity;GO:0007193|adenylate cyclase-inhibiting G-protein coupled receptor signaling pathway;GO:0007610|behavior;GO:0007268|synaptic transmission;GO:0014065|phosphatidylinositol 3-kinase signaling;GO:0007200|phospholipase C-activating G-protein coupled receptor signaling pathway;GO:0070374|positive regulation of ERK1 and ERK2 cascade;GO:0045600|positive regulation of fat cell differentiation;GO:0045821|positive regulation of glycolytic	GO:0030424|axon;GO:0005901|caveola;GO:0016023|cytoplasmic membrane-bounded vesicle;GO:0030425|dendrite;GO:0043198|dendritic shaft;GO:0005887|integral	GO:0071886|1-(4-iodo-2,5-dimethoxyphenyl)propan-2-amine	K04157	HTR2; 5-hydroxytryptamine receptor 2
RALGPS2	312.2175	-0.50165	0.146496821	-3.42433	0.0006163	0.008663	Not_Sig	no	gi|548499355|ref|XP_005690976.1|	2893	0	PREDICTED: ras-specific guanine nucleotide-releasing factor RalGPS2 isoform X1 [Capra hircus]	gi|982948257|ref|XM_005217159.3|	6951	0	PREDICTED: Bos taurus Ral GEF with PH domain and SH3 binding motif 2 (RALGPS2), transcript variant X1, mRNA	Q86X27	1109	0	Ras-specific guanine nucleotide-releasing factor RalGPS2	.	.	.	.	pfam00617	RasGEF	RasGEF domain. Guanine nucleotide exchange factor for Ras-like small GTPases.	GO:0032485|regulation of Ral protein signal transduction;GO:0007264|small GTPase mediated signal transduction;	GO:0005737|cytoplasm;GO:0005886|plasma membrane;	GO:0005085|guanyl-nucleotide exchange factor activity;	.	.
REPIN1	446.4694	0.500403	0.146139098	3.424156	0.0006167	0.008663	Not_Sig	no	gi|965857468|ref|XP_014963738.1|	2739	0	PREDICTED: replication initiator 1 isoform X2 [Ovis aries musimon]	gi|965857499|ref|XM_015108262.1|	2766	0	PREDICTED: Ovis aries musimon replication initiator 1 (LOC105615174), transcript variant X12, mRNA	Q0VCC5	979	0	Replication initiator 1	.	.	.	.	pfam13465	zf-H2C2_2	Zinc-finger double domain.	GO:0006260|DNA replication;GO:2000191|regulation of fatty acid transport;GO:2001273|regulation of glucose import in response to insulin stimulus;	GO:0022626|cytosolic ribosome;GO:0005811|lipid particle;GO:0031965|nuclear membrane;	GO:0003677|DNA binding;GO:0046872|metal ion binding;GO:0044822	.	.

SLC30A9	1090.339	-0.41053	0.119981181	-3.42165	0.0006224	0.008705	Not_Sig	no	gi|297475812|ref|XP_002688262.1|	2785	0	PREDICTED: zinc transporter 9 [Bos taurus]	gi|982983174|ref|XM_003585272.4|	4906	0	PREDICTED: Bos taurus solute carrier family 30 (zinc transporter), member 9 (SLC30A9), mRNA	Q6PML9	1003	0	Zinc transporter 9	mll4860	131	1.46e-32	COG0053	pfam01545	Cation_efflux	Cation efflux family. Members of this family are integral membrane proteins, that are found to increase tolerance to divalent	GO:0006289|nucleotide-excision repair;GO:0045944|positive regulation of transcription from RNA polymerase II promoter;GO:0006351|transcription, DNA-templated;GO:0006829|zinc II ion transport;	GO:0005856|cytoskeleton;GO:0016021|integral component of membrane;GO:0005634|nucleus;	GO:0008324|cation transmembrane transporter activity;GO:0003682	K14696	SLC30A9, ZNT9; solute carrier family 30 (zinc transporter), member 9
CDK17	548.3812	-0.41787	0.122131696	-3.42145	0.0006229	0.008705	Not_Sig	no	gi|296487516|tpg|DAA29629.1|	3013	0	TPA: PCTAIRE protein kinase 2-like [Bos taurus]	gi|741877735|ref|XM_005198330.2|	4327	0	PREDICTED: Bos taurus cyclin-dependent kinase 17 (CDK17), transcript variant X3, mRNA	O35831	1065	0	Cyclin-dependent kinase 17	.	.	.	.	pfam00069	Pkinase	Protein kinase domain.	.	.	GO:0005524|ATP binding;GO:0004693|cyclin-dependent protein	.	.
MFSD14A	907.8698	-0.36006	0.105246459	-3.42113	0.0006236	0.008705	Not_Sig	no	gi|537271325|gb|ERE92038.1|	2320	0	hippocampus abundant transcript 1 protein [Cricetulus griseus]	gi|156120722|ref|NM_001102038.1|	2540	0	Bos taurus major facilitator superfamily domain containing 14A (MFSD14A), mRNA	P70187	888	0	Hippocampus abundant transcript 1 protein	.	.	.	.	pfam07690	MFS_1	Major Facilitator Superfamily.	GO:0055085|transmembrane transport;	GO:0016021|integral component of membrane;	GO:0005215|transporter activity;	.	.
EFTUD2	1457.654	0.360125	0.105266753	3.421075	0.0006237	0.008705	Not_Sig	no	gi|852794871|ref|XP_012887487.1|	4984	0	PREDICTED: 116 kDa U5 small nuclear ribonucleoprotein component [Dipodomys ordii]	gi|742086603|ref|XM_010832425.1|	3430	0	PREDICTED: Bison bison bison elongation factor Tu GTP binding domain containing 2 (EFTUD2), mRNA	Q15029	1925	0	116 kDa U5 small nuclear ribonucleoprotein component	SPBC215.12	1081	0	COG0480	pfam00009	GTP_EFTU	Elongation factor Tu GTP binding domain. This domain contains a P-loop motif, also found in several other families such as pfam00071,	GO:0035690|cellular response to drug;GO:0006397|mRNA processing;GO:0000398|mRNA splicing, via spliceosome;GO:0042220|response to cocaine;GO:0008380|RNA splicing;	GO:0015030|Cajal body;GO:0071013|catalytic step 2 spliceosome;GO:0005737|cytoplasm;GO:0031012|extracellular	GO:0005525|GTP binding;GO:0003924|GTPase activity;GO:0044822	K12852	EFTUD2; 116 kDa U5 small nuclear ribonucleoprotein component
PXDN	6833.383	0.424521	0.124091175	3.42104	0.0006238	0.008705	Not_Sig	no	gi|741984139|ref|XP_010799143.1|	7739	0	PREDICTED: peroxidasin homolog isoform X1 [Bos taurus]	gi|983012326|ref|XM_015470659.1|	5957	0	PREDICTED: Bos taurus peroxidasin (PXDN), transcript variant X1, mRNA	Q92626	2645	0	Peroxidasin homolog	.	.	.	.	pfam03098	An_peroxidase	Animal haem peroxidase.	GO:0030198|extracellular matrix organization;GO:0042744|hydrogen peroxide catabolic process;GO:0006955|immune response;GO:0055114|oxidation-reduction process;GO:0006979|response to oxidative stress;	GO:0005783|endoplasmic reticulum;GO:0070062|extracellular exosome;GO:0031012|extracellular matrix;GO:0005615|extracellular	GO:0005201|extracellular matrix structural constituent;GO:002	K19511	PXDN, VPO1; peroxidase [EC:1.11.1.7]
GPR153	220.8379	0.614282	0.17957039	3.420843	0.0006243	0.008705	UP	yes	gi|76637520|ref|XP_597109.2|	2610	0	PREDICTED: probable G-protein coupled receptor 153 [Bos taurus]	gi|982996633|ref|XM_597109.9|	4023	0	PREDICTED: Bos taurus G protein-coupled receptor 153 (GPR153), transcript variant X1, mRNA	Q6NV75	868	0	Probable G-protein coupled receptor 153	.	.	.	.	pfam00001	7tm_1	7 transmembrane receptor (rhodopsin family). This family contains, amongst other G-protein-coupled receptors	.	GO:0016021|integral component of membrane;GO:0005886|plasma membrane;	GO:0004930|G-protein coupled receptor activity;	K08437	GPR153; G protein-coupled receptor 153
TBX2	385.7954	0.521068	0.152374133	3.41966	0.000627	0.008732	Not_Sig	no	gi|330864741|ref|NP_001178372.1|	2717	0	T-box transcription factor TBX2 [Bos taurus]	gi|330864740|ref|NM_001191443.1|	2584	0	Bos taurus T-box 2 (TBX2), mRNA	Q863A2	1010	0	T-box transcription factor TBX2	.	.	.	.	pfam00907	T-box	T-box. The T-box encodes a 180 amino acid domain that binds to DNA. Genes encoding T-box proteins are found in a wide range	GO:0090398|cellular senescence;GO:0035050|embryonic heart tube development;GO:0001947|heart looping;GO:0008016|regulation of heart contraction;GO:0006351|transcription, DNA-templated;	GO:0005634|nucleus;	GO:0003677|DNA binding;GO:0003700|transcription factor activity, sequence-	K10176	TBX2; T-box protein 2
TMEM106B	456.7299	-0.56506	0.165334171	-3.41768	0.0006316	0.008775	Not_Sig	no	gi|743700658|ref|XP_010970352.1|	1360	2e-168	PREDICTED: transmembrane protein 106B [Camelus bactrianus]	gi|742211524|ref|XM_010862136.1|	6791	0	PREDICTED: Bison bison bison transmembrane protein 106B (TMEM106B), transcript variant X1, mRNA	Q3ZC25	525	3e-169	Transmembrane protein 106B	.	.	.	.	pfam07092	DUF1356	Protein of unknown function (DUF1356). This family consists of several hypothetical mammalian proteins of around 250 residues in	GO:0048813|dendrite morphogenesis;GO:0007041|lysosomal transport;GO:0032418|lysosome localization;GO:0007040|lysosome organization;GO:1900006|positive regulation of dendrite development;	GO:0070062|extracellular exosome;GO:0016021|integral component of membrane;GO:0031902|late endosome membrane;GO:0005765|lysosomal	.	.	.
THAP4	522.7415	0.456981	0.133723461	3.417355	0.0006323	0.008775	Not_Sig	no	gi|288558829|sp|Q2TBI2.2|THAP4_BOVIN	2313	0	RecName: Full=THAP domain-containing protein 4	gi|84579828|ref|NM_001038669.1|	1897	0	Bos taurus THAP domain containing 4 (THAP4), mRNA	Q2TBI2	895	0	THAP domain-containing protein 4	.	.	.	.	pfam08768	DUF1794	Domain of unknown function (DUF1794). This domain forms a beta barrel structure but the function is unknown. The GO	.	.	GO:0003677|DNA binding;GO:0046872|metal ion binding;	.	.
NDFIP1	2537.352	-0.41714	0.122065238	-3.41735	0.0006323	0.008775	Not_Sig	no	gi|594077978|ref|XP_006062700.1|	901	5e-114	PREDICTED: NEDD4 family-interacting protein 1 [Bubalus bubalis]	gi|594077977|ref|XM_006062638.1|	1853	0	PREDICTED: Bubalus bubalis Nedd4 family interacting protein 1 (NDFIP1), mRNA	Q9BT67	338	2e-110	NEDD4 family-interacting protein 1	.	.	.	.	pfam10176	DUF2370	Protein of unknown function (DUF2370). This family is conserved from fungi to humans. The human member is annotated	GO:0006879|cellular iron ion homeostasis;GO:0030001|metal ion transport;GO:0010629|negative regulation of gene expression;GO:0050728|negative regulation of inflammatory response;GO:0032713|negative regulation of interleukin-4 production;GO:0048294|negative regulation of isotype switching to IgE isotypes;GO:0051224|negative regulation of protein transport;GO:0042130|negative regulation of T cell proliferation;GO:0032410|negative regulation of transporter	GO:0005938|cell cortex;GO:0010008|endosome membrane;GO:0005576|extracellular region;GO:0000139|Golgi	GO:0004871|signal transducer activity;	.	.
TGFB1	1074.948	0.556643	0.162918643	3.416692	0.0006339	0.008785	Not_Sig	no	gi|742162150|ref|XP_010847566.1|	1879	0	PREDICTED: transforming growth factor beta-1 isoform X1 [Bison bison bison]	gi|555957077|ref|XM_005890614.1|	1925	0	PREDICTED: Bos mutus transforming growth factor, beta 1 (TGFB1), mRNA	P18341	722	0	Transforming growth factor beta-1	.	.	.	.	pfam00688	TGFb_propeptide	TGF-beta propeptide. This propeptide is known as latency associated peptide (LAP) in TGF-beta. LAP is a homodimer which is	GO:0006754|ATP biosynthetic process;GO:0007050|cell cycle arrest;GO:0008219|cell death;GO:0048468|cell development;GO:0016049|cell growth;GO:0016477|cell migration;GO:0045216|cell-cell junction organization;GO:0071407|cellular response to organic cyclic compound;GO:0071560|cellular response to transforming growth factor beta stimulus;GO:0002062|chondrocyte differentiation;GO:0007182|common-partner SMAD protein phosphorylation;GO:0007173|epidermal growth factor receptor signaling	GO:0072562|blood microparticle;GO:0009986|cell surface;GO:0005737|cytoplasm;GO:0005615|extracellular	GO:0003823|antigen binding;GO:0005125|cytokine	K13375	TGFB1; transforming growth factor beta-1
IMPDH1	474.959	0.452164	0.132362577	3.416103	0.0006352	0.008794	Not_Sig	no	gi|118150800|ref|NP_001071309.1|	3028	0	inosine-5'-monophosphate dehydrogenase 1 [Bos taurus]	gi|402693081|ref|NM_001077841.2|	2494	0	Bos taurus IMP (inosine 5'-monophosphate) dehydrogenase 1 (IMPDH1), mRNA	A0JNA3	1052	0	Inosine-5'-monophosphate dehydrogenase 1	.	.	.	.	pfam00478	IMPDH	IMP dehydrogenase / GMP reductase domain. This family is involved in biosynthesis of guanosine nucleotide. Members of	GO:0006177|GMP biosynthetic process;GO:0006183|GTP biosynthetic process;	GO:0005737|cytoplasm;GO:0005634|nucleus;	GO:0003938|IMP dehydrogenase activity;GO:0046872|metal ion	K00088	guaB; IMP dehydrogenase [EC:1.1.1.205]
TOMM5	523.0676	-0.61755	0.180798103	-3.41567	0.0006363	0.008797	DOWN	yes	gi|724907717|ref|XP_010378456.1|	315	4e-34	PREDICTED: mitochondrial import receptor subunit TOM5 homolog [Rhinopithecus roxellana]	gi|742154485|ref|XM_010847086.1|	573	0	PREDICTED: Bison bison bison translocase of outer mitochondrial membrane 5 homolog (yeast) (TOMM5), mRNA	Q5R676	102	8e-27	Mitochondrial import receptor subunit TOM5 homolog	.	.	.	.	pfam15178	TOM_sub5	Mitochondrial import receptor subunit TOM5 homolog. This is a family of transmembrane proteins thought to form part of the pre-	GO:0006626|protein targeting to mitochondrion;	GO:0016021|integral component of membrane;GO:0005742|mitochondrial outer membrane translocase complex;	.	K17773	TOM5; mitochondrial import receptor subunit TOM5
ZNF598	588.7176	0.495631	0.145137444	3.41491	0.000638	0.008806	Not_Sig	no	gi|359079551|ref|XP_003587855.1|	3329	0	PREDICTED: zinc finger protein 598 [Bos taurus]	gi|983006770|ref|XM_003583999.4|	3235	0	PREDICTED: Bos taurus zinc finger protein 598 (ZNF598), mRNA	Q80YR4	785	0	Zinc finger protein 598	YDR266c	120	3.12e-27	COG5236	pfam05109	Herpes_BLLF1	Herpes virus major outer envelope glycoprotein (BLLF1). This family consists of the BLLF1 viral late glycoprotein, also termed	.	.	GO:0044822|poly (A) RNA binding;GO:0008270|zinc ion binding;	.	.
EEIG2	743.2225	-0.48817	0.142959375	-3.41474	0.0006384	0.008806	Not_Sig	no	gi|194665550|ref|XP_592474.4|	1751	0	PREDICTED: protein FAM102B [Bos taurus]	gi|742232534|ref|XM_010830939.1|	4939	0	PREDICTED: Bison bison bison family with sequence similarity 102, member B (FAM102B), mRNA	Q5T8I3	645	0	Protein FAM102B	.	.	.	.	pfam10358	NT-C2	N-terminal C2 in EEIG1 and EHBP1 proteins. This version of the C2 domain was initally identified in the vertebrate estrogen early-	.	.	.	.	.
MAP3K11	335.2871	0.45627	0.13367029	3.413402	0.0006416	0.008837	Not_Sig	no	gi|555964073|ref|XP_005894108.1|	2956	0	PREDICTED: mitogen-activated protein kinase kinase kinase 11 isoform X1 [Bos mutus]	gi|594065052|ref|XM_006056464.1|	3809	0	PREDICTED: Bubalus bubalis mitogen-activated protein kinase kinase kinase 11 (MAP3K11), transcript variant X1, mRNA	Q80XI6	1083	0	Mitogen-activated protein kinase kinase kinase 11	.	.	.	.	pfam07714	Pkinase_Tyr	Protein tyrosine kinase.	GO:0007257|activation of JUN kinase activity;GO:0000187|activation of MAPK activity;GO:0008219|cell death;GO:0008283|cell proliferation;GO:0007017|microtubule-based process;GO:0046330|positive regulation of JNK cascade;GO:0043507|positive regulation of JUN kinase activity;GO:0043525|positive regulation of neuron apoptotic process;GO:0046777|protein autophosphorylation;GO:0006468|protein phosphorylation;	GO:0005813|centrosome;GO:0005737|cytoplasm;GO:0016020|membrane;GO:0005874|microtubule;	GO:0005524|ATP binding;GO:0042802|identical protein binding;GO:0004706	K04419	MAP3K11, MLK3; mitogen-activated protein kinase kinase kinase 11
FGF1	76.80485	-0.76859	0.225190235	-3.41307	0.0006424	0.008837	DOWN	yes	gi|27806633|ref|NP_776480.1|	827	4e-99	fibroblast growth factor 1 [Bos taurus]	gi|742166339|ref|XM_010850421.1|	4176	0	PREDICTED: Bison bison bison fibroblast growth factor 1 (acidic) (FGF1), transcript variant X2, mRNA	P03968	323	1e-100	Fibroblast growth factor 1	.	.	.	.	pfam00167	FGF	Fibroblast growth factor. Fibroblast growth factors are a family of proteins involved in growth and differentiation in a	GO:0000187|activation of MAPK activity;GO:0032148|activation of protein kinase B activity;GO:0001525|angiogenesis;GO:0060681|branch elongation involved in ureteric bud branching;GO:0030154|cell differentiation;GO:0034605|cellular response to heat;GO:0008543|fibroblast growth factor receptor signaling pathway;GO:0030324|lung development;GO:0072163|mesonephric epithelium development;GO:0001759|organ induction;GO:0045766|positive regulation of angiogenesis;GO:0051781|positive regulation of cell	GO:0005938|cell cortex;GO:0005829|cytosol;GO:0005576|extracellular region;GO:0005615|extracellular space;GO:0005654|nucleoplasm;GO:000557	GO:0005104|fibroblast growth factor receptor binding;GO:0008083	K18496	FGF1; fibroblast growth factor 1
TTC39B	187.7196	-0.58305	0.170843027	-3.41278	0.000643	0.008837	Not_Sig	no	gi|594044521|ref|XP_006080914.1|	3142	0	PREDICTED: LOW QUALITY PROTEIN: tetratricopeptide repeat domain 39B [Bubalus bubalis]	gi|982986108|ref|XM_585202.8|	5233	0	PREDICTED: Bos taurus tetratricopeptide repeat domain 39B (TTC39B), transcript variant X1, mRNA	Q5VTQ0	1077	0	Tetratricopeptide repeat protein 39B	.	.	.	.	pfam10300	DUF3808	Protein of unknown function (DUF3808). This is a family of proteins conserved from fungi to humans. Members of this family	.	.	.	.	.
RPS27A	5317.967	-0.49836	0.146041739	-3.41243	0.0006439	0.008837	Not_Sig	no	gi|803252037|ref|XP_011985960.1|	729	7e-95	PREDICTED: ubiquitin-40S ribosomal protein S27a [Ovis aries musimon]	gi|942037488|ref|XM_005889475.2|	568	0	PREDICTED: Bos mutus ribosomal protein S27a (RPS27A), transcript variant X1, mRNA	P62979	250	2e-83	Ubiquitin-40S ribosomal protein S27a	ECU02g0740i	153	2.35e-48	COG5272	pfam00240	ubiquitin	Ubiquitin family. This family contains a number of ubiquitin-like proteins: SUMO (smt3 homologue) Nedd8, Elongin B, Rub1, and	GO:0000187|activation of MAPK activity;GO:0031145|anaphase-promoting complex-dependent proteasomal ubiquitin-dependent protein catabolic process;GO:0044267|cellular protein metabolic process;GO:0042769|DNA damage response, detection of DNA damage;GO:0006977|DNA damage response, signal transduction by p53 class mediator resulting in cell cycle arrest;GO:0016197|endosomal transport;GO:0038128|ERBB2 signaling pathway;GO:0070987|error-free translesion synthesis;GO:0042276|error-prone translesion	GO:0005737|cytoplasm;GO:0005829|cytosol;GO:0022627|cytosolic small ribosomal subunit;GO:0030666|endocytic vesicle membrane;GO:0010008|endosome	GO:0046872|metal ion binding;GO:0044822|poly(A) RNA	K02977	RP-S27Ae, RPS27A; small subunit ribosomal protein S27Ae
FST	9870.233	0.289024	0.084708479	3.411981	0.0006449	0.008837	Not_Sig	no	gi|926718430|ref|XP_005694775.2|	1837	0	PREDICTED: follistatin isoform X1 [Capra hircus]	gi|982959376|ref|XM_005221516.3|	1753	0	PREDICTED: Bos taurus follistatin (FST), transcript variant X1, mRNA	P50291	628	0	Follistatin	.	.	.	.	pfam07648	Kazal_2	Kazal-type serine protease inhibitor domain. Usually indicative of serine protease inhibitors. However, kazal-like	GO:0002244|hematopoietic progenitor cell differentiation;GO:0032926|negative regulation of activin receptor signaling pathway;GO:0000122|negative regulation of transcription from RNA polymerase II promoter;GO:0051798|positive regulation of hair follicle development;	GO:0005576|extracellular region;	.	K04661	FST; follistatin
SGPL1	698.0973	-0.36114	0.105849481	-3.41184	0.0006453	0.008837	Not_Sig	no	gi|555955458|ref|XP_005889887.1|	3055	0	PREDICTED: sphingosine-1-phosphate lyase 1 [Bos mutus]	gi|555955457|ref|XM_005889825.1|	4019	0	PREDICTED: Bos mutus sphingosine-1-phosphate lyase 1 (SGPL1), mRNA	O95470	1093	0	Sphingosine-1-phosphate lyase 1	YDR294c	371	2.46e-112	COG0076	pfam00282	Pyridoxal_deC	Pyridoxal-dependent decarboxylase conserved domain.	GO:0008209|androgen metabolic process;GO:0097190|apoptotic signaling pathway;GO:0006672|ceramide metabolic process;GO:0008210|estrogen metabolic process;GO:0060325|face morphogenesis;GO:0006631|fatty acid metabolic process;GO:0010761|fibroblast migration;GO:0030097|hemopoiesis;GO:0001822|kidney development;GO:0033327|Leydig cell differentiation;GO:0001553|luteinization;GO:0060021|palate development;GO:0048008|platelet-derived growth factor receptor signaling	GO:0005783|endoplasmic reticulum;GO:0005789|endoplasmic reticulum membrane;GO:0030176|integral component of endoplasmic reticulum	GO:0016831|carboxy-lyase activity;GO:0030170|pyridoxal	K01634	SGPL1, DPL1; sphinganine-1-phosphate aldolase [EC:4.1.2.27]
COX7B	821.5674	-0.5536	0.162302617	-3.41094	0.0006474	0.008856	Not_Sig	no	gi|443609469|ref|NP_786989.3|	459	3e-56	cytochrome c oxidase subunit 7B, mitochondrial precursor [Bos taurus]	gi|336126326|ref|NM_175795.3|	465	0	Bos taurus cytochrome c oxidase subunit VIIb (COX7B), mRNA	P13183	170	1e-53	Cytochrome c oxidase subunit 7B, mitochondrial	.	.	.	.	pfam05392	COX7B	Cytochrome C oxidase chain VIIB.	GO:0007417|central nervous system development;GO:1902600|hydrogen ion transmembrane transport;	GO:0016021|integral component of membrane;GO:0005746|mitochondrial respiratory chain;GO:0005739|mitochondrion;GO:0045	GO:0004129|cytochrome-c oxidase activity;	K02271	COX7B; cytochrome c oxidase subunit 7b
TOMM7	319.961	-0.62648	0.183774078	-3.409	0.000652	0.008908	DOWN	yes	gi|655837022|ref|XP_002713756.2|	295	4e-28	PREDICTED: mitochondrial import receptor subunit TOM7 homolog [Oryctolagus cuniculus]	gi|982980309|ref|XR_001496239.1|	1457	0	PREDICTED: Bos taurus uncharacterized LOC101907978 (LOC101907978), ncRNA	A1XQS2	103	4e-25	Mitochondrial import receptor subunit TOM7 homolog	.	.	.	.	pfam08038	Tom7	TOM7 family. This family consists of TOM7 family of mitochondrial import receptors. TOM7 forms part of the translocase of the outer	GO:0098779|mitophagy in response to mitochondrial depolarization;GO:1903955|positive regulation of protein targeting to mitochondrion;GO:0030150|protein import into mitochondrial matrix;GO:0045040|protein import into mitochondrial outer membrane;GO:0031647|regulation of protein stability;	GO:0031307|integral component of mitochondrial outer membrane;GO:0005742|mitochondrial outer membrane translocase complex;	.	K17771	TOM7; mitochondrial import receptor subunit TOM7
NHERF1	222.3486	0.539494	0.158285777	3.408356	0.0006536	0.008914	Not_Sig	no	gi|742187249|ref|XP_010854431.1|	1536	0	PREDICTED: Na(+)/H(+) exchange regulatory cofactor NHE-RF1 [Bison bison bison]	gi|402743299|ref|NM_001077852.2|	1942	0	Bos taurus solute carrier family 9, subfamily A (NHE3, cation proton antiporter 3), member 3 regulator 1 (SLC9A3R1), mRNA	Q3SZK8	595	0	Na(+)/H(+) exchange regulatory cofactor NHE-RF1	.	.	.	.	pfam09007	EBP50_C-term	EBP50, C-terminal. This C terminal domain allows interaction of EBP50 with FERM (four-point one ERM) domains, resulting in the	GO:0030036|actin cytoskeleton organization;GO:0007191|adenylate cyclase-activating dopamine receptor signaling pathway;GO:0060088|auditory receptor cell stereocilium organization;GO:0032782|bile acid secretion;GO:0019933|cAMP-mediated signaling;GO:0030643|cellular phosphate ion homeostasis;GO:0045198|establishment of epithelial cell apical/basal polarity;GO:0051683|establishment of Golgi localization;GO:0040023|establishment of nucleus localization;GO:0090002|establishment of	GO:0016324|apical plasma membrane;GO:0031526|brush border membrane;GO:0071944|cell periphery;GO:0005813|centrosome;GO:000	GO:0017081|chloride channel regulator activity;	K13365	SLC9A3R1, NHERF1; Na(+)/H(+) exchange regulatory cofactor NHE-RF1
UFM1	2522.186	-0.38694	0.113531756	-3.40818	0.000654	0.008914	Not_Sig	no	gi|926705900|ref|XP_005687546.2|	526	2e-59	PREDICTED: ubiquitin-fold modifier 1 [Capra hircus]	gi|742099929|ref|XM_010835642.1|	2392	0	PREDICTED: Bison bison bison ubiquitin-fold modifier 1 (UFM1), mRNA	Q2KJG2	174	7e-50	Ubiquitin-fold modifier 1	.	.	.	.	pfam03671	Ufm1	Ubiquitin fold modifier 1 protein. This is a family of short ubiquitin-like proteins, that is like neither type-1 or type-2. It is a ubiquitin-	GO:1990592|protein K69-linked ufmylation;GO:0071569|protein ufmylation;GO:0033146|regulation of intracellular estrogen receptor signaling pathway;GO:0034976|response to endoplasmic reticulum stress;	GO:0005737|cytoplasm;GO:0005634|nucleus;	.	K12162	UFM1; ubiquitin-fold modifier 1
LZTR1	761.151	0.433394	0.127201997	3.407135	0.0006565	0.008937	Not_Sig	no	gi|300797832|ref|NP_001179040.1|	4269	0	leucine-zipper-like transcriptional regulator 1 [Bos taurus]	gi|594084133|ref|XM_006065567.1|	2903	0	PREDICTED: Bubalus bubalis leucine-zipper-like transcription regulator 1 (LZTR1), mRNA	Q8N653	1573	0	Leucine-zipper-like transcriptional regulator 1	.	.	.	.	pfam00651	BTB	BTB/POZ domain. The BTB (for BR-C, ttk and bab) or POZ (for Pox virus and Zinc finger) domain is present near the N-terminus of a	GO:0009653|anatomical structure morphogenesis;	GO:0005794|Golgi apparatus;	GO:0003700|transcription factor activity, sequence-specific DNA binding;	.	.
GPS1	1016.182	0.462269	0.135722692	3.405985	0.0006593	0.008964	Not_Sig	no	gi|803208493|ref|XP_011964755.1|	2603	0	PREDICTED: COP9 signalosome complex subunit 1 isoform X3 [Ovis aries musimon]	gi|965787210|ref|XM_012109363.2|	2004	0	PREDICTED: Ovis aries musimon G protein pathway suppressor 1 (GPS1), transcript variant X1, mRNA	Q13098	925	0	COP9 signalosome complex subunit 1	.	.	.	.	pfam10602	RPN7	26S proteasome subunit RPN7. RPN7 (known as the non ATPase regulatory subunit 6 in higher eukaryotes) is one of the lid	GO:0007049|cell cycle;GO:0010387|COP9 signalosome assembly;GO:0010388|cullin deneddylation;GO:0000188|inactivation of MAPK activity;GO:0007254|JNK cascade;GO:0000715|nucleotide-excision repair, DNA damage recognition;GO:0006283|transcription-coupled nucleotide-excision repair;	GO:0008180|COP9 signalosome;GO:0005737|cytoplasm;GO:0005654|nucleoplasm;	GO:0005095|GTPase inhibitor activity;	K12175	GPS1, COPS1, CSN1; COP9 signalosome complex subunit 1
CCDC126	64.70006	-0.87228	0.256181104	-3.40494	0.0006618	0.008985	DOWN	yes	gi|126723215|ref|NP_001075941.1|	554	4e-63	coiled-coil domain-containing protein 126 precursor [Bos taurus]	gi|982921172|ref|XM_005205280.3|	2284	0	PREDICTED: Bos taurus coiled-coil domain containing 126 (CCDC126), transcript variant X2, mRNA	A2VE00	218	1e-64	Coiled-coil domain-containing protein 126	.	.	.	.	pfam15027	DUF4525	Domain of unknown function (DUF4525). This domain is found in eukaryotes. It is often found at the N-terminus of glycosyltransferase	GO:0006487|protein N-linked glycosylation;	GO:0005576|extracellular region;GO:0016020|membrane;	GO:0030144|alpha-1,6-mannosylglycoprotein 6-beta-N-	.	.
SNAPC4	211.6385	0.633017	0.185923744	3.40471	0.0006623	0.008985	UP	yes	gi|965804396|ref|XP_014953445.1|	4362	0	PREDICTED: snRNA-activating protein complex subunit 4 isoform X2 [Ovis aries musimon]	gi|594077073|ref|XM_006062203.1|	3564	0	PREDICTED: Bubalus bubalis small nuclear RNA activating complex, polypeptide 4, 190kDa (SNAPC4), mRNA	Q5SXM2	769	0	snRNA-activating protein complex subunit 4	YKR099w	76.3	5.96e-13	COG5147	pfam00249	Myb_DNA-binding	Myb-like DNA-binding domain. This family contains the DNA binding domains from Myb proteins, as well as the SANT	GO:0030154|cell differentiation;GO:0006357|regulation of transcription from RNA polymerase II promoter;GO:0009301|snRNA transcription;GO:0042795|snRNA transcription from RNA polymerase II promoter;GO:0042796|snRNA transcription from RNA polymerase III promoter;GO:0006366|transcription from RNA polymerase II promoter;GO:0006383|transcription from RNA polymerase III promoter;	GO:0005654|nucleoplasm;GO:0005634|nucleus;GO:0019185|snRNA-activating protein complex;	GO:0003677|DNA binding;GO:0000981|RNA polymerase II transcription factor	K09453	SNAPC4; snRNA-activating protein complex subunit 4
CD164	6583.13	-0.36322	0.106720422	-3.40343	0.0006655	0.009017	Not_Sig	no	gi|440897271|gb|ELR49002.1|	503	1e-54	Sialomucin core protein 24 [Bos mutus]	gi|742188990|ref|XM_010856628.1|	3014	0	PREDICTED: Bison bison bison CD164 molecule, sialomucin (CD164), transcript variant X1, mRNA	Q2YDH0	194	6e-55	Sialomucin core protein 24	.	.	.	.	pfam05283	MGC-24	Multi-glycosylated core protein 24 (MGC-24). This family consists of several MGC-24 (or Cd164 antigen) proteins from eukaryotic	GO:0007157|heterophilic cell-cell adhesion via plasma membrane cell adhesion molecules;GO:0007517|muscle organ development;	GO:0010008|endosome membrane;GO:0016021|integral component of membrane;GO:0005765|lysosomal	.	K06546	CD164, MGC24; CD164 antigen
COMMD3	548.1133	-0.4221	0.124042257	-3.40291	0.0006667	0.009023	Not_Sig	no	gi|562823776|ref|XP_006141684.1|	1051	2e-141	PREDICTED: polycomb complex protein BMI-1 isoform X4 [Tupaia chinensis]	gi|942072102|ref|XM_005901647.2|	919	0	PREDICTED: Bos mutus COMM domain containing 3 (COMMD3), transcript variant X2, mRNA	Q3SZ76	402	2e-140	COMM domain-containing protein 3	.	.	.	.	pfam07258	HCaRG	HCaRG protein. This family consists of several mammalian HCaRG(hypertension-related, calcium-regulated gene) proteins.	GO:0006355|regulation of transcription, DNA-templated;GO:0006814|sodium ion transport;GO:0006351|transcription, DNA-templated;	GO:0005737|cytoplasm;GO:0005634|nucleus;	.	.	.
IRF2BP1	439.5574	0.476292	0.140145285	3.398556	0.0006774	0.009157	Not_Sig	no	gi|560972512|ref|XP_006208601.1|	2538	0	PREDICTED: interferon regulatory factor 2-binding protein 1 [Vicugna pacos]	gi|965825112|ref|XM_015106260.1|	2389	0	PREDICTED: Ovis aries musimon interferon regulatory factor 2-binding protein 1 (LOC105608449), mRNA	Q8IU81	974	0	Interferon regulatory factor 2-binding protein 1	.	.	.	.	pfam11261	IRF-2BP1_2	Interferon regulatory factor 2-binding protein zinc finger. IRF-2BP1 and IRF-2BP2 are nuclear transcriptional repressor proteins	GO:0006355|regulation of transcription, DNA-templated;GO:0006351|transcription, DNA-templated;	GO:0005654|nucleoplasm;	GO:0016874|ligase activity;GO:0046872|metal ion binding;	.	.
PLD3	2865.413	0.510015	0.150174281	3.396153	0.0006834	0.009224	Not_Sig	no	gi|118151178|ref|NP_001071509.1|	2414	0	phospholipase D3 [Bos taurus]	gi|742162042|ref|XM_010849233.1|	2208	0	PREDICTED: Bison bison bison phospholipase D family, member 3 (PLD3), transcript variant X1, mRNA	Q2KJJ8	913	0	Phospholipase D3	.	.	.	.	pfam13918	PLDc_3	PLD-like domain.	GO:0016042|lipid catabolic process;	GO:0005789|endoplasmic reticulum membrane;GO:0070062|extracellular exosome;GO:0016021|integral component of membrane;	GO:0070290|N-acylphosphatidylethanolamine-specific phospholipase D	K16860	PLD3_4; phospholipase D3/4 [EC:3.1.4.4]
LOC102284748	114.9236	-0.76409	0.225002624	-3.39592	0.000684	0.009224	DOWN	yes	gi|351709486|gb|EHB12405.1|	340	6e-38	Small EDRK-rich factor 1 [Heterocephalus glaber]	gi|742177451|ref|XM_010853573.1|	543	0	PREDICTED: Bison bison bison small EDRK-rich factor 1 (LOC104998425), mRNA	Q32P76	118	4e-33	Small EDRK-rich factor 1	.	.	.	.	pfam04419	4F5	4F5 protein family. Members of this family are short proteins that are rich in aspartate, glutamate, lysine and arginine. Although the	.	.	.	.	.
TENT5A	2269.569	-0.31251	0.09207939	-3.39387	0.0006891	0.009283	Not_Sig	no	gi|528909137|ref|XP_005194642.1|	2331	0	PREDICTED: protein FAM46A isoform X2 [Bos taurus]	gi|982987361|ref|XM_005194585.3|	5508	0	PREDICTED: Bos taurus family with sequence similarity 46 member A (FAM46A), transcript variant X2, mRNA	Q96IP4	832	0	Protein FAM46A	.	.	.	.	pfam07984	DUF1693	Domain of unknown function (DUF1693). This family contains many hypothetical proteins. It also includes four nematode prion-like	GO:0030193|regulation of blood coagulation;GO:0010468|regulation of gene expression;	.	GO:0044822|poly (A) RNA binding;	.	.
LMBRD2	449.2507	-0.51891	0.152967392	-3.39231	0.0006931	0.009325	Not_Sig	no	gi|742178187|ref|XP_010852089.1|	3501	0	PREDICTED: LMBR1 domain-containing protein 2 isoform X1 [Bison bison bison]	gi|803170297|ref|XM_012097057.1|	7381	0	PREDICTED: Ovis aries LMBR1 domain containing 2 (LMBRD2), transcript variant X1, mRNA	Q68DH5	1280	0	LMBR1 domain-containing protein 2	.	.	.	.	pfam04791	LMBR1	LMBR1-like membrane protein. Members of this family are integral membrane proteins that are around 500 residues in length.	.	GO:0016021|integral component of membrane;GO:0016020|membrane;	.	.	.
PTEN	802.488	-0.4019	0.118584922	-3.38916	0.0007011	0.009421	Not_Sig	no	gi|985701093|ref|NP_001306827.1|	2352	0	phosphatidylinositol-3,4,5-trisphosphate 3-phosphatase and dual-specificity protein phosphatase PTEN [Bos taurus]	gi|983007627|ref|XM_015469496.1|	5039	0	PREDICTED: Bos taurus phosphatase and tensin homolog (PTEN), transcript variant X1, mRNA	P60484	733	0	Phosphatidylinositol 3,4,5-trisphosphate 3-phosphatase and dual-specificity protein	SPBC609.02	110	7.49e-25	COG2453	pfam10409	PTEN_C2	C2 domain of PTEN tumor-suppressor protein. This is the C2 domain-like domain, in greek key form, of the PTEN protein,	GO:0030534|adult behavior;GO:0007568|aging;GO:0001525|angiogenesis;GO:0006915|apoptotic process;GO:0048854|brain morphogenesis;GO:0060070|canonical Wnt signaling pathway;GO:0048738|cardiac muscle tissue development;GO:0016477|cell migration;GO:0008283|cell proliferation;GO:0071456|cellular response to hypoxia;GO:0007417|central nervous system development;GO:0032286|central nervous system myelin maintenance;GO:0021955|central nervous system neuron	GO:0016324|apical plasma membrane;GO:0042995|cell projection;GO:0005737|cytoplasm;GO:0009898|cytoplasmic side of plasma	GO:0010997|anaphase-promoting complex binding;GO:0019899	K01110	PTEN; phosphatidylinositol-3,4,5-trisphosphate 3-phosphatase and
INPPL1	6208.732	0.39371	0.116180707	3.388776	0.0007021	0.009424	Not_Sig	no	gi|300794196|ref|NP_001178105.1|	5463	0	phosphatidylinositol 3,4,5-trisphosphate 5-phosphatase 2 [Bos taurus]	gi|965885366|ref|XM_012155204.2|	4497	0	PREDICTED: Ovis aries musimon inositol polyphosphate phosphatase like 1 (INPPL1), transcript variant X2, mRNA	O15357	2051	0	Phosphatidylinositol 3,4,5-trisphosphate 5-phosphatase 2	YIL002c_2	133	5.45e-32	COG5411	pfam00017	SH2	SH2 domain.	GO:0007015|actin filament organization;GO:0007155|cell adhesion;GO:0001958|endochondral ossification;GO:0006897|endocytosis;GO:0006006|glucose metabolic process;GO:0002376|immune system process;GO:0043647|inositol phosphate metabolic process;GO:0008285|negative regulation of cell proliferation;GO:0010629|negative regulation of gene expression;GO:0006661|phosphatidylinositol biosynthetic process;GO:0046856|phosphatidylinositol dephosphorylation;GO:0009791|post-	GO:0005737|cytoplasm;GO:0005856|cytoskeleton;GO:0005829|cytosol;GO:0030175|filopodium;GO:0005794|Golgi apparatus;GO:0030027|lamellipodium;GO:0	GO:0052659|inositol-1,3,4,5-tetrakisphosphate 5-phosphatase	K15909	SHIP2, INPPL1; phosphatidylinositol-3,4,5-trisphosphate 5-phosphatase 2
ELF4	1258	0.409407	0.120867138	3.387247	0.000706	0.00946	Not_Sig	no	gi|555993174|ref|XP_005908353.1|	3111	0	PREDICTED: ETS-related transcription factor Elf-4 [Bos mutus]	gi|982972647|ref|XM_005227486.3|	5093	0	PREDICTED: Bos taurus E74-like factor 4 (ets domain transcription factor) (ELF4), transcript variant X1, mRNA	Q99607	1067	0	ETS-related transcription factor Elf-4	.	.	.	.	pfam00178	Ets	Ets-domain.	GO:0030154|cell differentiation;GO:0045087|innate immune response;GO:0001787|natural killer cell proliferation;GO:0001866|NK T cell proliferation;GO:0045944|positive regulation of transcription from RNA polymerase II promoter;GO:0045893|positive regulation of transcription, DNA-templated;	GO:0005794|Golgi apparatus;GO:0031965|nuclear membrane;GO:0005654|nucleoplasm;GO:0016605|PML body;	GO:0000978|RNA polymerase II core promoter proximal region sequence-	K09428	ELF1_2_4; E74-like factor 1/2/4

GPR137	371.2492	0.540824	0.159672508	3.387082	0.0007064	0.00946	Not_Sig	no	gi|440907343|gb|ELR57498.1|	1666	0	Integral membrane protein GPR137, partial [Bos mutus]	gi|982971853|ref|XM_010821057.2|	1717	0	PREDICTED: Bos taurus G protein-coupled receptor 137 (GPR137), transcript variant X1, mRNA	Q17QQ5	593	0	Integral membrane protein GPR137	.	.	.	.	pfam04684	BAF1_ABF1	BAF1 / ABF1 chromatin reorganizing factor. ABF1 is a sequence-specific DNA binding protein involved in transcription	.	GO:0016021|integral component of membrane;	.	.	.
LRRN2	145.5983	0.694798	0.205237875	3.385329	0.0007109	0.009509	UP	yes	gi|555972905|ref|XP_005898453.1|	3284	0	PREDICTED: leucine-rich repeat neuronal protein 2 [Bos mutus]	gi|942061501|ref|XM_005898391.2|	3034	0	PREDICTED: Bos mutus leucine rich repeat neuronal 2 (LRRN2), mRNA	O75325	1180	0	Leucine-rich repeat neuronal protein 2	alr0124_1	72.4	5.27e-12	COG4886	pfam07679	I-set	Immunoglobulin I-set domain.	GO:0007155|cell adhesion;GO:0007165|signal transduction;	GO:0016021|integral component of membrane;	GO:0004872|receptor activity;	.	.
DNAJB4	4540.261	-0.40471	0.119577346	-3.38452	0.000713	0.009526	Not_Sig	no	gi|440898066|gb|ELR49639.1|	1711	0	DnaJ-like protein subfamily B member 4, partial [Bos mutus]	gi|594090178|ref|XM_006068440.1|	3644	0	PREDICTED: Bubalus bubalis DnaJ (Hsp40) homolog, subfamily B, member 4 (DNAJB4), mRNA	Q2KIT4	647	0	DnaJ homolog subfamily B member 4	.	.	.	.	pfam00226	DnaJ	DnaJ domain. DnaJ domains (J-domains) are associated with hsp70 heat-shock system and it is thought that this domain mediates	GO:0006457|protein folding;	GO:0005829|cytosol;GO:0070062|extracellular exosome;GO:0005654|nucleoplasm;GO:0005886|plasma membrane;	.	K09510	DNAJB4; DnaJ homolog subfamily B member 4
SEC61B	985.9027	-0.36381	0.107518266	-3.38373	0.0007151	0.009543	Not_Sig	no	gi|965918853|ref|XP_011975701.2|	433	2e-50	PREDICTED: protein transport protein Sec61 subunit beta isoform X1 [Ovis aries]	gi|942057753|ref|XM_005897026.2|	623	0	PREDICTED: Bos mutus Sec61 translocon beta subunit (SEC61B), transcript variant X2, mRNA	Q5RB31	130	6e-37	Protein transport protein Sec61 subunit beta	.	.	.	.	pfam03911	Sec61_beta	Sec61beta family. This family consists of homologues of Sec61beta - a component of the Sec61/SecYEG protein secretory	GO:0030433|ER-associated ubiquitin-dependent protein catabolic process;GO:0000060|protein import into nucleus, translocation;GO:0030970|retrograde protein transport, ER to cytosol;	GO:0031205|endoplasmic reticulum Sec complex;GO:0016021|integral component of membrane;GO:0005784|Sec61 translocon complex;	GO:0044822|poly(A) RNA binding;	K09481	SEC61B, SBH2; protein transport protein SEC61 subunit beta
COMMD4	311.4194	-0.5476	0.161909797	-3.38213	0.0007193	0.009587	Not_Sig	no	gi|94966996|ref|NP_001035687.1|	952	8e-127	COMM domain-containing protein 4 [Bos taurus]	gi|555959424|ref|XM_005891760.1|	909	0	PREDICTED: Bos mutus COMM domain containing 4 (COMMD4), mRNA	Q5E9V6	371	2e-128	COMM domain-containing protein 4	.	.	.	.	pfam07258	HCaRG	HCaRG protein. This family consists of several mammalian HCaRG(hypertension-related, calcium-regulated gene) proteins.	GO:0006355|regulation of transcription, DNA-templated;GO:0006351|transcription, DNA-templated;	GO:0005737|cytoplasm;GO:0005634|nucleus;	.	.	.
RPL30	3327.389	-0.49959	0.147742314	-3.38152	0.0007209	0.009598	Not_Sig	no	gi|617596964|ref|XP_007521900.1|	624	7e-80	PREDICTED: 60S ribosomal protein L30-like [Erinaceus europaeus]	gi|942080578|ref|XM_005904379.2|	539	0	PREDICTED: Bos mutus ribosomal protein L30 (RPL30), transcript variant X1, mRNA	P62890	236	2e-78	60S ribosomal protein L30	SPAC9G1.03c	153	4.59e-48	COG1911	pfam01248	Ribosomal_L7Ae	Ribosomal protein L7Ae/L30e/S12e/Gadd45 family. This family includes: Ribosomal L7A from metazoa, Ribosomal L8-A	GO:0097421|liver regeneration;GO:1904571|positive regulation of selenocysteine incorporation;GO:0016259|selenocysteine metabolic process;GO:0006412|translation;	GO:0005829|cytosol;GO:0022625|cytosolic large ribosomal subunit;GO:0005840|ribosome;	GO:0035368|selenocysteine insertion sequence binding;GO:0003735	K02908	RP-L30e, RPL30; large subunit ribosomal protein L30e
TGFBRAP1	463.9676	0.440645	0.130352035	3.380421	0.0007237	0.009625	Not_Sig	no	gi|742227533|ref|XP_010827991.1|	4315	0	PREDICTED: transforming growth factor-beta receptor-associated protein 1 [Bison bison bison]	gi|156120662|ref|NM_001102008.1|	4877	0	Bos taurus transforming growth factor beta receptor associated protein 1 (TGFBRAP1), mRNA	A7MB11	1654	0	Transforming growth factor-beta receptor-associated protein 1	.	.	.	.	pfam10367	Vps39_2	Vacuolar sorting protein 39 domain 2. This domain is found on the vacuolar sorting protein Vps39 which is a component of the C-Vps	GO:0006886|intracellular protein transport;GO:0007165|signal transduction;GO:0016192|vesicle-mediated transport;	GO:0005769|early endosome;	GO:0046332|SMAD binding;	.	.
ARL5A	702.4299	-0.44754	0.132442337	-3.37913	0.0007272	0.009659	Not_Sig	no	gi|730370842|gb|KHJ42451.1|	1951	0	reverse transcriptase [Trichuris suis]	gi|850474981|gb|CP011886.1|	2218	0	Ovis canadensis canadensis isolate 43U chromosome 1 sequence	O02751	323	3e-93	Craniofacial development protein 2	.	.	.	.	pfam00078	RVT_1	Reverse transcriptase (RNA-dependent DNA polymerase). A reverse transcriptase gene is usually indicative of a mobile	.	GO:0005737|cytoplasm;GO:0005634|nucleus;	.	.	.
LOC102285651	72.51096	-0.97068	0.287389976	-3.37757	0.0007313	0.009703	DOWN	yes	gi|530617155|ref|XP_005298314.1|	664	8e-86	PREDICTED: 60S ribosomal protein L23a isoform X2 [Chrysemys picta bellii]	gi|402747155|ref|NG_028344.2|	534	0	Bos taurus ribosomal protein L23a pseudogene (LOC616830) on chromosome 9	P62752	259	4e-87	60S ribosomal protein L23a	SPBC4F6.04	166	9.02e-53	COG0089	pfam00276	Ribosomal_L23	Ribosomal protein L23.	GO:0000027|ribosomal large subunit assembly;GO:0006412|translation;	GO:0022625|cytosolic large ribosomal subunit;GO:0005634|nucleus;	GO:0070180|large ribosomal subunit rRNA binding;GO:0000166	K02893	RP-L23Ae, RPL23A; large subunit ribosomal protein L23Ae
FADS3	3820.532	0.37281	0.110457474	3.375145	0.0007378	0.009774	Not_Sig	no	gi|139948648|ref|NP_001077160.1|	2279	0	fatty acid desaturase 3 [Bos taurus]	gi|965863592|ref|XM_012146315.2|	2161	0	PREDICTED: Ovis aries musimon fatty acid desaturase 3 (FADS3), transcript variant X2, mRNA	A4IFP3	882	0	Fatty acid desaturase 3	YNL111c	57.8	2.91e-09	COG5274	pfam00487	FA_desaturase	Fatty acid desaturase.	GO:0006633|fatty acid biosynthetic process;	GO:0005789|endoplasmic reticulum membrane;GO:0016021|integral component of membrane;	GO:0016491|oxidoreductase activity;	K10225	FADS3; fatty acid desaturase 3 (delta-6 desaturase) [EC:1.14.19.-]
FEM1A	1053.786	0.38686	0.114627931	3.37492	0.0007384	0.009774	Not_Sig	no	gi|555994655|ref|XP_005909077.1|	3060	0	PREDICTED: protein fem-1 homolog A isoform X1 [Bos mutus]	gi|942095102|ref|XM_005909015.2|	3544	0	PREDICTED: Bos mutus fem-1 homolog a (C. elegans) (FEM1A), transcript variant X1, mRNA	Q29RM5	1165	0	Protein fem-1 homolog A	all2748	90.5	4.15e-18	COG0666	pfam12796	Ank_2	Ankyrin repeats (3 copies).	GO:0050728|negative regulation of inflammatory response;GO:0051438|regulation of ubiquitin-protein transferase activity;	GO:0005737|cytoplasm;	GO:0004842|ubiquitin-protein transferase activity;	.	.
CAD	912.837	0.357203	0.105858163	3.374358	0.0007399	0.009783	Not_Sig	no	gi|926704617|ref|XP_013823259.1|	11442	0	PREDICTED: CAD protein [Capra hircus]	gi|926704616|ref|XM_013967805.1|	6511	0	PREDICTED: Capra hircus carbamoyl-phosphate synthetase 2, aspartate transcarbamylase, and dihydroorotase (CAD), mRNA	P27708	4274	0	CAD protein	SPAC22G7.06c_2	1240	0	COG0458	pfam02786	CPSase_L_D2	Carbamoyl-phosphate synthase L chain, ATP binding domain. Carbamoyl-phosphate synthase catalyses the ATP-dependent	GO:0006207|'de novo' pyrimidine nucleobase biosynthetic process;GO:0044205|'de novo' UMP biosynthetic process;GO:0031100|organ regeneration;GO:0006526|arginine biosynthetic process;GO:0035690|cellular response to drug;GO:0071364|cellular response to epidermal growth factor stimulus;GO:0019240|citrulline biosynthetic process;GO:0017144|drug metabolic process;GO:0007565|female pregnancy;GO:0006541|glutamine metabolic process;GO:0007507|heart development;GO:0007595|lactation;GO:0001889|liver	GO:0042995|cell projection;GO:0005829|cytosol;GO:0070062|extracellular exosome;GO:0016020|membrane;GO:0043	GO:0070335|aspartate binding;GO:0004070|aspartate	K11540	CAD; carbamoyl-phosphate synthase / aspartate carbamoyltransferas
TRAPPC6B	236.1947	-0.57924	0.171680272	-3.37395	0.000741	0.009786	Not_Sig	no	gi|955529843|ref|XP_004436435.2|	913	1e-115	PREDICTED: trafficking protein particle complex subunit 6B [Ceratotherium simum simum]	gi|942074213|ref|XM_005902286.2|	1874	0	PREDICTED: Bos mutus trafficking protein particle complex 6B (TRAPPC6B), transcript variant X1, mRNA	Q86SZ2	333	1e-109	Trafficking protein particle complex subunit 6B	.	.	.	.	pfam04051	TRAPP	Transport protein particle (TRAPP) component. TRAPP plays a key role in the targeting and/or fusion of ER-to-Golgi transport vesicles with	GO:0048208|COPII vesicle coating;	GO:0005829|cytosol;GO:0005783|endoplasmic reticulum;GO:0000139|Golgi membrane;	.	.	.
SHKBP1	680.0219	0.477261	0.141488694	3.373138	0.0007432	0.009804	Not_Sig	no	gi|160333397|ref|NP_001076076.1|	3145	0	SH3KBP1-binding protein 1 [Bos taurus]	gi|942040782|ref|XM_005890597.2|	2337	0	PREDICTED: Bos mutus SH3KBP1 binding protein 1 (SHKBP1), mRNA	A3KMV1	1216	0	SH3KBP1-binding protein 1	.	.	.	.	pfam02214	BTB_2	BTB/POZ domain. In voltage-gated K+ channels this domain is responsible for subfamily-specific assembly of alpha-subunits into	GO:0045742|positive regulation of epidermal growth factor receptor signaling pathway;GO:0051260|protein homooligomerization;	.	.	.	.
SPOPL	557.4852	-0.52278	0.155108592	-3.37041	0.0007506	0.00988	Not_Sig	no	gi|965921800|ref|XP_014948918.1|	2173	0	PREDICTED: speckle-type POZ protein-like isoform X3 [Ovis aries]	gi|742177815|ref|XM_010853676.1|	5613	0	PREDICTED: Bison bison bison speckle-type POZ protein-like (SPOPL), transcript variant X1, mRNA	Q6IQ16	761	0	Speckle-type POZ protein-like	.	.	.	.	pfam00651	BTB	BTB/POZ domain. The BTB (for BR-C, ttk and bab) or POZ (for Pox virus and Zinc finger) domain is present near the N-terminus of a	GO:0031397|negative regulation of protein ubiquitination;GO:0043161|proteasome-mediated ubiquitin-dependent protein catabolic process;GO:0042787|protein ubiquitination involved in ubiquitin-dependent protein catabolic process;GO:0030162|regulation of proteolysis;	GO:0031463|Cul3-RING ubiquitin ligase complex;GO:0005737|cytoplasm;GO:0005634|nucleus;GO:0019005|SCF ubiquitin ligase complex;	GO:0031625|ubiquitin protein ligase binding;	K10523	SPOP; speckle-type POZ protein
TRPS1	595.7517	-0.43604	0.12937289	-3.37037	0.0007507	0.00988	Not_Sig	no	gi|555970571|ref|XP_005897306.1|	6395	0	PREDICTED: zinc finger transcription factor Trps1 isoform X1 [Bos mutus]	gi|594101929|ref|XM_006073981.1|	8200	0	PREDICTED: Bubalus bubalis trichorhinophalangeal syndrome I (TRPS1), transcript variant X1, mRNA	Q9UHF7	2331	0	Zinc finger transcription factor Trps1	ECU08g1400	64.7	5.14e-10	COG5641	pfam00320	GATA	GATA zinc finger. This domain uses four cysteine residues to coordinate a zinc ion. This domain binds to DNA. Two GATA zinc	GO:0048646|anatomical structure formation involved in morphogenesis;GO:0009887|organ morphogenesis;GO:0048468|cell development;GO:0045165|cell fate commitment;GO:0048565|digestive tract development;GO:0007507|heart development;GO:0000122|negative regulation of transcription from RNA polymerase II promoter;GO:0006607|NLS-bearing protein import into nucleus;GO:0032330|regulation of chondrocyte differentiation;GO:0001501|skeletal system development;GO:0009888|tissue	GO:0000790|nuclear chromatin;GO:0005654|nucleoplasm;GO:0005634|nucleus;GO:0005667|transcription factor complex;	GO:0003682|chromatin binding;GO:0000977|RNA polymerase II	.	.
LTBP3	1297.298	0.461783	0.137033427	3.369859	0.0007521	0.009881	Not_Sig	no	gi|529012676|ref|XP_005227172.1|	5899	0	PREDICTED: latent-transforming growth factor beta-binding protein 3 isoform X2 [Bos taurus]	gi|982971968|ref|XM_005227114.3|	4512	0	PREDICTED: Bos taurus latent transforming growth factor beta binding protein 3 (LTBP3), transcript variant X1, mRNA	Q9NS15	2180	0	Latent-transforming growth factor beta-binding protein 3	.	.	.	.	pfam00683	TB	TB domain. This domain is also known as the 8 cysteine domain. This family includes the hybrid domains. This cysteine rich repeat	GO:2000741|positive regulation of mesenchymal stem cell differentiation;GO:1902462|positive regulation of mesenchymal stem cell proliferation;GO:0036363|transforming growth factor beta activation;	GO:0070062|extracellular exosome;GO:0031012|extracellular matrix;GO:0005576|extracellular region;	GO:0005509|calcium ion binding;GO:0050431|transforming	K08023	LTBP2_3_4; latent transforming growth factor beta binding protein 2/3/4
HAUS2	479.1537	-0.44639	0.132482458	-3.36946	0.0007531	0.009881	Not_Sig	no	gi|594085450|ref|XP_006066255.1|	1293	2e-169	PREDICTED: LOW QUALITY PROTEIN: HAUS augmin-like complex subunit 2 [Bubalus bubalis]	gi|942079271|ref|XM_005903996.2|	2524	0	PREDICTED: Bos mutus HAUS augmin-like complex, subunit 2 (HAUS2), transcript variant X1, mRNA	Q9NVX0	417	3e-138	HAUS augmin-like complex subunit 2	.	.	.	.	pfam15003	HAUS2	HAUS augmin-like complex subunit 2. This family of proteins is found in eukaryotes. Proteins in this family are typically between 203	GO:0051301|cell division;GO:0051297|centrosome organization;GO:0000086|G2/M transition of mitotic cell cycle;GO:0007067|mitotic nuclear division;GO:0051225|spindle assembly;	GO:0005813|centrosome;GO:0005737|cytoplasm;GO:0005829|cytosol;GO:0005794|Golgi apparatus;GO:0070652|HAUS complex;GO:0005654|nucleoplasm;GO:000	.	K16585	HAUS2; HAUS augmin-like complex subunit 2
KMT2D	1283.847	0.397973	0.118119104	3.369248	0.0007537	0.009881	Not_Sig	no	gi|741877412|ref|XP_010825989.1|	10592	0	PREDICTED: histone-lysine N-methyltransferase 2D [Bos taurus]	gi|982981707|ref|XM_010827687.2|	12682	0	PREDICTED: Bos taurus lysine (K)-specific methyltransferase 2D (KMT2D), mRNA	O14686	3665	0	Histone-lysine N-methyltransferase 2D	SPCC306.04c	135	2.15e-30	COG2940	pfam05965	FYRC	F/Y rich C-terminus. This region is normally found in the trithorax/ALL1 family proteins. It is similar to SMART:SM00542.	GO:0006342|chromatin silencing;GO:0051568|histone H3-K4 methylation;GO:0001555|oocyte growth;GO:0048477|oogenesis;GO:0008284|positive regulation of cell proliferation;GO:0033148|positive regulation of intracellular estrogen receptor signaling pathway;GO:0045944|positive regulation of transcription from RNA polymerase II promoter;GO:0006355|regulation of transcription, DNA-templated;GO:0043627|response to estrogen;GO:0006351|transcription, DNA-templated;	GO:0035097|histone methyltransferase complex;GO:0044666|MLL3/4 complex;GO:0005654|nucleoplasm;GO:0005634|nucleus;	GO:0003677|DNA binding;GO:0042800|histone methyltransferase	K09187	MLL2, ALR; histone-lysine N-methyltransferase MLL2 [EC:2.1.1.43]
PSAT1	1134.703	-0.35263	0.104667861	-3.36907	0.0007542	0.009881	Not_Sig	no	gi|742168952|ref|XP_010849442.1|	2145	0	PREDICTED: phosphoserine aminotransferase [Bison bison bison]	gi|156120946|ref|NM_001102150.1|	2164	0	Bos taurus phosphoserine aminotransferase 1 (PSAT1), mRNA	Q9Y617	701	0	Phosphoserine aminotransferase	PA3167	365	2.05e-119	COG1932	pfam00266	Aminotran_5	Aminotransferase class-V. This domain is found in amino transferases, and other enzymes including cysteine desulphurase	GO:0006564|L-serine biosynthetic process;GO:0008615|pyridoxine biosynthetic process;	GO:0005737|cytoplasm;GO:0005829|cytosol;GO:0070062|extracellular exosome;	GO:0004648|O-phospho-L-serine:2-oxoglutarate aminotransferase	K00831	serC, PSAT1; phosphoserine aminotransferase [EC:2.6.1.52]
SGSH	415.3683	0.457281	0.135747694	3.368613	0.0007555	0.009887	Not_Sig	no	gi|555954836|ref|XP_005889584.1|	2626	0	PREDICTED: N-sulphoglucosamine sulphohydrolase [Bos mutus]	gi|555954835|ref|XM_005889522.1|	2311	0	PREDICTED: Bos mutus N-sulfoglucosamine sulfohydrolase (SGSH), mRNA	P51688	910	0	N-sulphoglucosamine sulphohydrolase	Rv0296c	134	1.66e-32	COG3119	pfam00884	Sulfatase	Sulfatase.	GO:0006027|glycosaminoglycan catabolic process;GO:0030200|heparan sulfate proteoglycan catabolic process;	GO:0070062|extracellular exosome;GO:0043202|lysosomal lumen;GO:0005764|lysosome;	GO:0046872|metal ion binding;GO:0016250|N-	K01565	SGSH; N-sulfoglucosamine sulfohydrolase [EC:3.10.1.1]
EVI5L	374.6535	0.494015	0.146669065	3.368227	0.0007565	0.009889	Not_Sig	no	gi|149944719|ref|NP_001092545.1|	3663	0	EVI5-like protein [Bos taurus]	gi|741932140|ref|XM_005208765.2|	3670	0	PREDICTED: Bos taurus ecotropic viral integration site 5-like (EVI5L), transcript variant X1, mRNA	Q96CN4	1365	0	EVI5-like protein	.	.	.	.	pfam00566	RabGAP-TBC	Rab-GTPase-TBC domain. Identification of a TBC domain in GYP6_YEAST and GYP7_YEAST, which are GTPase activator	GO:0090630|activation of GTPase activity;GO:0006886|intracellular protein transport;GO:1902018|negative regulation of cilium assembly;GO:0031338|regulation of vesicle fusion;	GO:0012505|endomembrane system;GO:0005622|intracellular;	GO:0005096|GTPase activator activity;GO:0017137|Rab GTPase	.	.
PARP1	2358.792	0.309595	0.091992076	3.365452	0.0007642	0.00995	Not_Sig	no	gi|296479253|tpg|DAA21368.1|	4926	0	TPA: poly [ADP-ribose] polymerase 1 [Bos taurus]	gi|742174962|ref|XM_010852872.1|	3647	0	PREDICTED: Bison bison bison poly (ADP-ribose) polymerase 1 (PARP1), transcript variant X1, mRNA	P18493	1883	0	Poly [ADP-ribose] polymerase 1	.	.	.	.	pfam00644	PARP	Poly(ADP-ribose) polymerase catalytic domain. Poly(ADP-ribose) polymerase catalyses the covalent attachment of ADP-ribose units	GO:0006974|cellular response to DNA damage stimulus;GO:0051103|DNA ligation involved in DNA repair;GO:0006302|double-strand break repair;GO:0006273|lagging strand elongation;GO:0010613|positive regulation of cardiac muscle hypertrophy;GO:0006471|protein ADP-ribosylation;GO:0006355|regulation of transcription, DNA-templated;GO:0006351|transcription, DNA-templated;	GO:0005737|cytoplasm;GO:0005635|nuclear envelope;GO:0005730|nucleolus;	GO:0003677|DNA binding;GO:0003910|DNA ligase (ATP) activity;GO:0051287	K10798	PARP; poly [ADP-ribose] polymerase [EC:2.4.2.30]
CNOT6L	782.7551	-0.41453	0.123182841	-3.36519	0.0007649	0.00995	Not_Sig	no	gi|594071340|ref|XP_006059530.1|	2717	0	PREDICTED: CCR4-NOT transcription complex subunit 6-like [Bubalus bubalis]	gi|942039650|ref|XM_005890198.2|	8777	0	PREDICTED: Bos mutus CCR4-NOT transcription complex, subunit 6-like (CNOT6L), mRNA	Q96LI5	1050	0	CCR4-NOT transcription complex subunit 6-like	SPCC31H12.08c_2	278	1.14e-82	COG5239	pfam03372	Exo_endo_phos	Endonuclease/Exonuclease/phosphatase family. This large family of proteins includes magnesium dependent endonucleases and a	GO:0006977|DNA damage response, signal transduction by p53 class mediator resulting in cell cycle arrest;GO:0031047|gene silencing by RNA;GO:0061157|mRNA destabilization;GO:0006397|mRNA processing;GO:0000289|nuclear-transcribed mRNA poly(A) tail shortening;GO:0008284|positive regulation of cell proliferation;GO:0010606|positive regulation of cytoplasmic mRNA processing body assembly;GO:0006355|regulation of transcription, DNA-templated;GO:0006351|transcription, DNA-templated;	GO:0030014|CCR4-NOT complex;GO:0005737|cytoplasm;GO:0005829|cytosol;GO:0005634|nucleus;	GO:0046872|metal ion binding;GO:0004535|poly(A)-specific	K12603	CNOT6, CCR4; CCR4-NOT transcription complex subunit 6 [EC:3.1.13.4]
FBXO32	2989.508	-0.32812	0.097507829	-3.36502	0.0007654	0.00995	Not_Sig	no	gi|635050006|ref|XP_007999672.1|	1908	0	PREDICTED: F-box only protein 32 [Chlorocebus sabaeus]	gi|742114032|ref|XM_010838912.1|	6930	0	PREDICTED: Bison bison bison F-box protein 32 (FBXO32), mRNA	Q2KHT6	737	0	F-box only protein 32	.	.	.	.	pfam13088	BNR_2	BNR repeat-like domain. This family of proteins contains BNR-like repeats suggesting these proteins may act as sialidases.	GO:0071549|cellular response to dexamethasone stimulus;GO:0016567|protein ubiquitination;GO:0014894|response to denervation involved in regulation of muscle adaptation;GO:0006511|ubiquitin-dependent protein catabolic process;	GO:0005737|cytoplasm;GO:0005654|nucleoplasm;GO:0005634|nucleus;GO:0019005|SCF ubiquitin ligase complex;GO:0030018|Z disc;	GO:0004842|ubiquitin-protein transferase activity;	K10305	FBXO25_32; F-box protein 25/32
SELENOS	968.6709	-0.37583	0.111694767	-3.36479	0.000766	0.00995	Not_Sig	no	gi|742149964|ref|XP_010844113.1|	857	1e-110	PREDICTED: selenoprotein S isoform X2 [Bison bison bison]	gi|742149963|ref|XM_010845811.1|	1277	0	PREDICTED: Bison bison bison VCP-interacting membrane protein (VIMP), transcript variant X2, mRNA	Q2KI76	290	1e-94	Selenoprotein S	.	.	.	.	pfam06936	Selenoprotein_S	Selenoprotein S (SelS). This family consists of several mammalian selenoprotein S (SelS) sequences. SelS is a plasma membrane protein	GO:0006886|intracellular protein transport;	GO:0005881|cytoplasmic microtubule;GO:0030176|integral component of endoplasmic reticulum membrane;	.	K14025	SELS, VIMP; selenoprotein S
LRRC8A	2360.411	0.449644	0.133632453	3.364779	0.000766	0.00995	Not_Sig	no	gi|116003833|ref|NP_001070275.1|	4088	0	volume-regulated anion channel subunit LRRC8A [Bos taurus]	gi|942094492|ref|XM_005908775.2|	4054	0	PREDICTED: Bos mutus leucine rich repeat containing 8 family, member A (LRRC8A), transcript variant X2, mRNA	Q8IWT6	1564	0	Volume-regulated anion channel subunit LRRC8A	alr0124_1	113	2.31e-25	COG4886	pfam12534	DUF3733	Leucine-rich repeat containing protein 8. This domain family is found in eukaryotes, and is approximately 60 amino acids in	GO:0006820|anion transport;GO:0006884|cell volume homeostasis;GO:0002329|pre-B cell differentiation;GO:0006970|response to osmotic stress;	GO:0009986|cell surface;GO:0034702|ion channel complex;GO:0016020|membrane;GO:0005886|plasma membrane;	GO:0005225|volume-sensitive anion channel activity;	.	.
TSTD1	49.12366	-0.9856	0.292928009	-3.36466	0.0007664	0.00995	DOWN	yes	gi|942095731|ref|XP_014338227.1|	714	1e-92	PREDICTED: thiosulfate sulfurtransferase/rhodanese-like domain-containing protein 1 isoform X1 [Bos mutus]	gi|594048325|ref|XM_006048499.1|	683	0	PREDICTED: Bubalus bubalis thiosulfate sulfurtransferase (rhodanese)-like domain containing 1 (TSTD1), transcript variant X1, mRNA	Q8NFU3	172	2e-52	Thiosulfate sulfurtransferase/rhodanese-like domain-containing protein 1	SPAC4H3.07c	65.5	1.61e-12	COG0607	pfam00581	Rhodanese	Rhodanese-like domain. Rhodanese has an internal duplication. This Pfam represents a single copy of this duplicated	.	GO:0005737|cytoplasm;GO:0005739|mitochondrion;GO:0048471|perinuclear region of cytoplasm;	.	.	.
LOC138990572	49.46241	0.964938	0.28703522	3.361741	0.0007745	0.010044	UP	yes	.	.	.	.	gi|850488330|gb|CP011898.1|	86	3e-36	Ovis canadensis canadensis isolate 43U chromosome 13 sequence	.	.	.	.	.	.	.	.	pfam01593	Amino_oxidase	Flavin containing amine oxidoreductase. This family consists of various amine oxidases, including maze polyamine oxidase	.	.	.	.	.
STAT6	2548.63	0.431579	0.128472918	3.359296	0.0007814	0.010122	Not_Sig	no	gi|742115628|ref|XP_010837614.1|	4261	0	PREDICTED: signal transducer and activator of transcription 6 isoform X1 [Bison bison bison]	gi|594110151|ref|XM_006077855.1|	3808	0	PREDICTED: Bubalus bubalis signal transducer and activator of transcription 6, interleukin-4 induced (STAT6), transcript variant X1, mRNA	P42226	1528	0	Signal transducer and activator of transcription 6	.	.	.	.	pfam02864	STAT_bind	STAT protein, DNA binding domain. STAT proteins (Signal Transducers and Activators of Transcription) are a family of	GO:0070301|cellular response to hydrogen peroxide;GO:1902170|cellular response to reactive nitrogen species;GO:0035771|interleukin-4-mediated signaling pathway;GO:0033598|mammary gland epithelial cell proliferation;GO:0060443|mammary gland morphogenesis;GO:0000122|negative regulation of transcription from RNA polymerase II promoter;GO:0002829|negative regulation of type 2 immune response;GO:0048295|positive regulation of isotype switching to IgE isotypes;GO:0045944|positive regulation of transcription from	GO:0005737|cytoplasm;GO:0005829|cytosol;GO:0045121|membrane raft;GO:0000790|nuclear chromatin;GO:0005654|nucleoplasm;	GO:0019903|protein phosphatase binding;GO:0000979|RNA polymerase II	K11225	STAT6; signal transducer and activator of transcription 6
TM6SF1	659.3952	-0.43128	0.128407629	-3.35865	0.0007833	0.010135	Not_Sig	no	gi|593749309|ref|XP_007129891.1|	1798	0	PREDICTED: transmembrane 6 superfamily member 1 [Physeter catodon]	gi|402745535|ref|NM_001102295.2|	2031	0	Bos taurus transmembrane 6 superfamily member 1 (TM6SF1), mRNA	Q9BZW5	696	0	Transmembrane 6 superfamily member 1	.	.	.	.	pfam10914	DUF2781	Protein of unknown function (DUF2781). This is a eukaryotic family of uncharacterized proteins. Some of the proteins in this family	.	GO:0016021|integral component of membrane;GO:0005765|lysosomal membrane;	.	.	.
WDR1	9558.79	0.344524	0.102592055	3.358195	0.0007845	0.01014	Not_Sig	no	gi|926694455|ref|XP_013820243.1|	3360	0	PREDICTED: WD repeat-containing protein 1 [Capra hircus]	gi|942047779|ref|XM_005893199.2|	2772	0	PREDICTED: Bos mutus WD repeat domain 1 (WDR1), mRNA	Q2KJH4	1240	0	WD repeat-containing protein 1	.	.	.	.	pfam00400	WD40	WD domain, G-beta repeat.	.	GO:0030054|cell junction;GO:0042995|cell projection;GO:0005737|cytoplasm;GO:0002102|podosome;	GO:0051015|actin filament binding;	.	.
UQCRB	1037.17	-0.52196	0.155470332	-3.35731	0.0007871	0.010161	Not_Sig	no	gi|555977983|ref|XP_005900941.1|	488	2e-59	PREDICTED: cytochrome b-c1 complex subunit 7 isoform X1 [Bos mutus]	gi|942069854|ref|XM_014479823.1|	642	0	PREDICTED: Bos mutus cytochrome b-c1 complex subunit 7 (LOC102283409), transcript variant X2, mRNA	P00129	192	1e-60	Cytochrome b-c1 complex subunit 7	.	.	.	.	pfam02271	UCR_14kD	Ubiquinol-cytochrome C reductase complex 14kD subunit. The ubiquinol-cytochrome C reductase complex (cytochrome bc1	GO:0009060|aerobic respiration;GO:1902600|hydrogen ion transmembrane transport;GO:0006122|mitochondrial electron transport, ubiquinol to cytochrome c;GO:0034551|mitochondrial respiratory chain complex III assembly;	GO:0005750|mitochondrial respiratory chain complex III;	.	K00417	QCR7, UQCRB; ubiquinol-cytochrome c reductase subunit 7
USP36	440.0164	0.439361	0.130915467	3.356066	0.0007906	0.010195	Not_Sig	no	gi|440892473|gb|ELR45652.1|	4863	0	Ubiquitin carboxyl-terminal hydrolase 36 [Bos mutus]	gi|742187736|ref|XM_010856270.1|	5649	0	PREDICTED: Bison bison bison ubiquitin specific peptidase 36 (USP36), mRNA	Q9P275	1264	0	Ubiquitin carboxyl-terminal hydrolase 36	.	.	.	.	pfam00443	UCH	Ubiquitin carboxyl-terminal hydrolase.	GO:1903955|positive regulation of protein targeting to mitochondrion;GO:0016579|protein deubiquitination;GO:1903146|regulation of mitophagy;GO:0006511|ubiquitin-dependent protein catabolic process;	GO:0005730|nucleolus;GO:0005634|nucleus;	GO:0044822|poly(A) RNA binding;GO:0004843|ubiquitin-specific	K11855	USP36_42; ubiquitin carboxyl-terminal hydrolase 36/42 [EC:3.4.19.12]
SERINC1	6023.874	-0.41121	0.122538707	-3.35576	0.0007915	0.010195	Not_Sig	no	gi|836709889|ref|XP_012789391.1|	2276	0	PREDICTED: serine incorporator 1 [Sorex araneus]	gi|942039459|ref|XM_005911727.2|	2795	0	PREDICTED: Bos mutus serine incorporator 1 (SERINC1), mRNA	Q3MHV9	878	0	Serine incorporator 1	.	.	.	.	pfam03348	Serinc	Serine incorporator (Serinc). This is a family of eukaryotic membrane proteins which incorporate serine into membranes and facilitate the	GO:0006658|phosphatidylserine metabolic process;GO:0008654|phospholipid biosynthetic process;GO:0006665|sphingolipid metabolic process;	GO:0005789|endoplasmic reticulum membrane;GO:0016021|integral component of membrane;	GO:0015194|L-serine transmembrane transporter activity;	.	.
CLDND1	1200.875	-0.39609	0.11806149	-3.35499	0.0007937	0.010212	Not_Sig	no	gi|594046871|ref|XP_006047868.1|	1395	0	PREDICTED: claudin domain-containing protein 1 isoform X2 [Bubalus bubalis]	gi|942057283|ref|XM_014478559.1|	2048	0	PREDICTED: Bos mutus claudin domain containing 1 (CLDND1), transcript variant X3, mRNA	Q5RDV7	507	4e-175	Claudin domain-containing protein 1	.	.	.	.	pfam13903	Claudin_2	PMP-22/EMP/MP20/Claudin tight junction. Members of this family are claudins, that form tight junctions between cells.	.	GO:0016324|apical plasma membrane;GO:0016021|integral component of membrane;	.	.	.
TMBIM4	2144.276	-0.44381	0.132337288	-3.35363	0.0007976	0.010251	Not_Sig	no	gi|742131473|ref|XP_010839876.1|	903	2e-112	PREDICTED: protein lifeguard 4 [Bison bison bison]	gi|742131472|ref|XM_010841574.1|	2323	0	PREDICTED: Bison bison bison transmembrane BAX inhibitor motif containing 4 (TMBIM4), mRNA	Q9DA39	306	3e-96	Protein lifeguard 4	SPCC576.04	115	6.36e-28	COG0670	pfam01027	Bax1-I	Inhibitor of apoptosis-promoting Bax1. Programmed cell-death involves a set of Bcl-2 family proteins, some of which inhibit	GO:0006915|apoptotic process;GO:0043066|negative regulation of apoptotic process;GO:0050848|regulation of calcium-mediated signaling;	GO:0000139|Golgi membrane;GO:0005795|Golgi stack;GO:0016021|integral component of membrane;	.	K06890	K06890; uncharacterized protein
MFGE8	94546.13	0.435782	0.130042789	3.35107	0.000805	0.010323	Not_Sig	no	gi|555997060|ref|XP_005910259.1|	2341	0	PREDICTED: lactadherin isoform X1 [Bos mutus]	gi|742124222|ref|XM_010841020.1|	1945	0	PREDICTED: Bison bison bison milk fat globule-EGF factor 8 protein (MFGE8), mRNA	Q95114	883	0	Lactadherin	.	.	.	.	pfam00754	F5_F8_type_C	F5/8 type C domain. This domain is also known as the discoidin (DS) domain family.	GO:0001525|angiogenesis;GO:0007155|cell adhesion;GO:0007338|single fertilization;	GO:0005576|extracellular region;GO:0016020|membrane;	.	K17253	MFGE8; lactadherin
MTPN	7410.793	-0.36578	0.109154524	-3.35107	0.000805	0.010323	Not_Sig	no	gi|302148690|pdb|3AAA|C	624	7e-72	Chain C, Crystal Structure Of Actin Capping Protein In Complex With V-1	gi|942078208|ref|XM_005903642.2|	3661	0	PREDICTED: Bos mutus myotrophin (MTPN), mRNA	P58546	243	5e-73	Myotrophin	.	.	.	.	pfam12796	Ank_2	Ankyrin repeats (3 copies).	GO:0006584|catecholamine metabolic process;GO:0016049|cell growth;GO:0071260|cellular response to mechanical stimulus;GO:0021707|cerebellar granule cell differentiation;GO:0030182|neuron differentiation;GO:0010613|positive regulation of cardiac muscle hypertrophy;GO:0030307|positive regulation of cell growth;GO:0010557|positive regulation of macromolecule biosynthetic process;GO:0051092|positive regulation of NF-kappaB transcription factor activity;GO:0051247|positive regulation of protein metabolic	GO:0030424|axon;GO:0005829|cytosol;GO:0070062|extracellular exosome;GO:0008290|F-actin capping protein	GO:0043565|sequence-specific DNA binding;	.	.

DNAH11	143.488	-0.6477	0.193304483	-3.35067	0.0008062	0.010327	DOWN	yes	gi|982921117|ref|XP_015324036.1|	23115	0	PREDICTED: dynein heavy chain 11, axonemal isoform X1 [Bos taurus]	gi|742114471|ref|XM_010839026.1|	10786	0	PREDICTED: Bison bison bison dynein, axonemal, heavy chain 11 (DNAH11), mRNA	Q96DT5	7888	0	Dynein heavy chain 11, axonemal	YKR054c	674	0	COG5245	pfam03028	Dynein_heavy	Dynein heavy chain and region D6 of dynein motor. This family represents the C-terminal region of dynein heavy chain. The chain	GO:0003341|cilium movement;GO:0007368|determination of left/right symmetry;GO:0030317|sperm motility;GO:0007507|heart development;GO:0003356|regulation of cilium beat frequency;	GO:0005737|cytoplasm;GO:0030286|dynein complex;GO:0005874|microtubule;GO:0031512|motile primary cilium;	GO:0005524|ATP binding;GO:0016887|ATPase activity;GO:0003777	K10408	DNAH; dynein heavy chain, axonemal
SBNO2	1719.183	0.470007	0.140305577	3.349879	0.0008085	0.010344	Not_Sig	no	gi|528956476|ref|XP_005209343.1|	6013	0	PREDICTED: protein strawberry notch homolog 2 isoform X4 [Bos taurus]	gi|594052497|ref|XM_006050500.1|	7591	0	PREDICTED: Bubalus bubalis strawberry notch homolog 2 (Drosophila) (SBNO2), transcript variant X4, mRNA	A0JND4	2283	0	Protein strawberry notch homolog 2	.	.	.	.	pfam13872	AAA_34	P-loop containing NTP hydrolase pore-1.	GO:0030282|bone mineralization;GO:0061430|bone trabecula morphogenesis;GO:0071348|cellular response to interleukin-11;GO:0071354|cellular response to interleukin-6;GO:1990830|cellular response to leukemia inhibitory factor;GO:0071222|cellular response to lipopolysaccharide;GO:0002281|macrophage activation involved in immune response;GO:0045892|negative regulation of transcription, DNA-templated;GO:0072675|osteoclast fusion;GO:0045944|positive regulation of transcription from RNA polymerase II	.	.	.	.
COX16	189.6993	-0.65814	0.196498779	-3.34932	0.0008101	0.010346	DOWN	yes	gi|114053029|ref|NP_001040033.1|	554	5e-70	cytochrome c oxidase assembly protein COX16 homolog, mitochondrial [Bos taurus]	gi|942084714|ref|XM_005905759.2|	524	0	PREDICTED: Bos mutus cytochrome c oxidase assembly protein COX16 homolog, mitochondrial (LOC102269677), transcript variant X1, mRNA	Q2NKS2	218	2e-71	Cytochrome c oxidase assembly protein COX16 homolog, mitochondrial	.	.	.	.	pfam14138	COX16	Cytochrome c oxidase assembly protein COX16. This family represents homologues of COX16 which has been shown to be	.	GO:0016021|integral component of membrane;GO:0031966|mitochondrial membrane;	.	K18182	COX16; cytochrome c oxidase assembly protein subunit 16
TXN	6139.223	-0.44694	0.133446688	-3.34921	0.0008104	0.010346	Not_Sig	no	gi|27806783|ref|NP_776393.1|	554	2e-69	thioredoxin [Bos taurus]	gi|942048107|ref|XM_005893314.2|	654	0	PREDICTED: Bos mutus thioredoxin (LOC102265694), transcript variant X1, mRNA	O97680	218	7e-71	Thioredoxin	.	.	.	.	pfam00085	Thioredoxin	Thioredoxin. Thioredoxins are small enzymes that participate in redox reactions, via the reversible oxidation of an active centre	GO:0045454|cell redox homeostasis;GO:0034599|cellular response to oxidative stress;GO:0006662|glycerol ether metabolic process;GO:0055114|oxidation-reduction process;GO:0043388|positive regulation of DNA binding;GO:0006457|protein folding;GO:0033158|regulation of protein import into nucleus, translocation;GO:0006355|regulation of transcription, DNA-templated;GO:0009314|response to radiation;GO:0000103|sulfate assimilation;GO:0006351|transcription, DNA-templated;	GO:0005576|extracellular region;GO:0005739|mitochondrion;GO:0005634|nucleus;	GO:0016671|oxidoreductase activity, acting on a sulfur group of donors,	K03671	trxA; thioredoxin 1
RFK	891.0691	-0.506	0.151132442	-3.34806	0.0008138	0.010378	Not_Sig	no	gi|74354693|gb|AAI02763.1|	1091	1e-139	RFK protein [Bos taurus]	gi|942047131|ref|XM_005892980.2|	2571	0	PREDICTED: Bos mutus riboflavin kinase (RFK), mRNA	Q969G6	311	5e-99	Riboflavin kinase	SPCC18.16c	135	6.47e-36	COG0196	pfam01687	Flavokinase	Riboflavin kinase. This family represents the C-terminal region of the bifunctional riboflavin biosynthesis protein known as	GO:0006915|apoptotic process;GO:0009398|FMN biosynthetic process;GO:0033864|positive regulation of NAD(P)H oxidase activity;GO:0072593|reactive oxygen species metabolic process;GO:0009231|riboflavin biosynthetic process;GO:0006771|riboflavin metabolic process;	GO:0005737|cytoplasm;GO:0005829|cytosol;GO:0005739|mitochondrion;	GO:0005524|ATP binding;GO:0046872|metal ion binding;GO:0008531	K00861	RFK, FMN1; riboflavin kinase [EC:2.7.1.26]
NID1	9560.071	0.32325	0.096629908	3.345233	0.0008221	0.010473	Not_Sig	no	gi|155372035|ref|NP_001094625.1|	6301	0	nidogen-1 precursor [Bos taurus]	gi|742203209|ref|XM_010860106.1|	5497	0	PREDICTED: Bison bison bison nidogen 1 (NID1), transcript variant X2, mRNA	P14543	2120	0	Nidogen-1	.	.	.	.	pfam07474	G2F	G2F domain. Nidogen, an invariant component of basement membranes, is a multifunctional protein that interacts with most	GO:0071711|basement membrane organization;GO:0007160|cell-matrix adhesion;GO:0022617|extracellular matrix disassembly;GO:0030198|extracellular matrix organization;GO:0032836|glomerular basement membrane development;GO:0010811|positive regulation of cell-substrate adhesion;	GO:0005605|basal lamina;GO:0005604|basement membrane;GO:0071944|cell periphery;GO:0070062|extracellular	GO:0005509|calcium ion binding;GO:0005518|collagen	K06826	NID; nidogen (entactin)
SPTBN1	5154.489	0.312992	0.093580099	3.344643	0.0008239	0.010473	Not_Sig	no	gi|300795972|ref|NP_001179205.1|	11840	0	spectrin beta chain, non-erythrocytic 1 [Bos taurus]	gi|555991162|ref|XM_005907306.1|	8266	0	PREDICTED: Bos mutus spectrin, beta, non-erythrocytic 1 (SPTBN1), transcript variant X1, mRNA	Q01082	4528	0	Spectrin beta chain, non-erythrocytic 1	SPAC15A10.08	260	3.03e-73	COG5069	pfam00307	CH	Calponin homology (CH) domain. The CH domain is found in both cytoskeletal proteins and signal transduction proteins. The CH	GO:0051693|actin filament capping;GO:0007411|axon guidance;GO:0007182|common-partner SMAD protein phosphorylation;GO:0006888|ER to Golgi vesicle-mediated transport;GO:0043001|Golgi to plasma membrane protein transport;GO:0000165|MAPK cascade;GO:0071709|membrane assembly;GO:0000281|mitotic cytokinesis;GO:0007009|plasma membrane organization;GO:1900042|positive regulation of interleukin-2 secretion;GO:1903078|positive regulation of protein localization to plasma	GO:0030673|axolemma;GO:0005913|cell-cell adherens junction;GO:0032437|cuticular plate;GO:0005737|cytoplasm;GO:0005829|	GO:0003779|actin binding;GO:0030506|ankyrin binding;GO:0098641	K06115	SPTB; spectrin beta
UBAP2L	2996.058	0.46662	0.139524208	3.344367	0.0008247	0.010473	Not_Sig	no	gi|594039288|ref|XP_006044238.1|	3936	0	PREDICTED: ubiquitin-associated protein 2-like isoform X1 [Bubalus bubalis]	gi|982916241|ref|XM_010802849.2|	4054	0	PREDICTED: Bos taurus ubiquitin associated protein 2 like (UBAP2L), transcript variant X2, mRNA	Q80X50	1391	0	Ubiquitin-associated protein 2-like	.	.	.	.	pfam12478	DUF3697	Ubiquitin-associated protein 2. This domain family is found in eukaryotes, and is approximately 30 amino acids in length. The	GO:0007339|binding of sperm to zona pellucida;GO:0061484|hematopoietic stem cell homeostasis;	GO:0005671|Ada2/Gcn5/Ada3 transcription activator complex;GO:0031519|PcG protein complex;	GO:0044822|poly(A) RNA binding;	.	.
AMPD2	897.0591	0.404457	0.120939048	3.344301	0.0008249	0.010473	Not_Sig	no	gi|555958496|ref|XP_005891366.1|	4220	0	PREDICTED: AMP deaminase 2 isoform X2 [Bos mutus]	gi|555958495|ref|XM_005891304.1|	3698	0	PREDICTED: Bos mutus adenosine monophosphate deaminase 2 (AMPD2), transcript variant X2, mRNA	Q01433	1600	0	AMP deaminase 2	YML035c	642	0	COG1816	pfam00962	A_deaminase	Adenosine/AMP deaminase.	GO:0046033|AMP metabolic process;GO:0046034|ATP metabolic process;GO:0042632|cholesterol homeostasis;GO:0052652|cyclic purine nucleotide metabolic process;GO:0097009|energy homeostasis;GO:0072015|glomerular visceral epithelial cell development;GO:0046039|GTP metabolic process;GO:0006188|IMP biosynthetic process;GO:0032264|IMP salvage;GO:0043101|purine-containing compound salvage;	GO:0005829|cytosol;	GO:0003876|AMP deaminase activity;GO:0046872|metal ion binding;	K01490	AMPD; AMP deaminase [EC:3.5.4.6]
VPS29	782.9478	-0.42002	0.12560671	-3.34391	0.0008261	0.010476	Not_Sig	no	gi|625274529|ref|XP_007628853.1|	1099	1e-139	PREDICTED: vacuolar protein sorting-associated protein 29 isoform X2 [Cricetulus griseus]	gi|594047918|ref|XM_006048305.1|	2711	0	PREDICTED: Bubalus bubalis vacuolar protein sorting 29 homolog (S. cerevisiae) (VPS29), transcript variant X1, mRNA	Q3T0M0	382	1e-124	Vacuolar protein sorting-associated protein 29	SPAC15E1.06	182	6.98e-52	COG0622	pfam12850	Metallophos_2	Calcineurin-like phosphoesterase superfamily domain. Members of this family are part of the Calcineurin-like phosphoesterase	GO:0006886|intracellular protein transport;GO:0042147|retrograde transport, endosome to Golgi;GO:1990126|retrograde transport, endosome to plasma membrane;	GO:0005829|cytosol;GO:0005769|early endosome;GO:0005768|endosome;GO:0010008|endosome membrane;GO:0005770|late	GO:0046872|metal ion binding;	K18467	VPS29; vacuolar protein sorting-associated protein 29
DLGAP4	1676.829	0.526456	0.15746889	3.34324	0.0008281	0.01049	Not_Sig	no	gi|528973769|ref|XP_005214768.1|	4093	0	PREDICTED: disks large-associated protein 4 isoform X2 [Bos taurus]	gi|982943069|ref|XM_005214711.3|	4559	0	PREDICTED: Bos taurus discs large homolog associated protein 4 (DLGAP4), transcript variant X2, mRNA	Q9Y2H0	1488	0	Disks large-associated protein 4	.	.	.	.	pfam03359	GKAP	Guanylate-kinase-associated protein (GKAP) protein.	GO:0023052|signaling;	GO:0016020|membrane;GO:0045202|synapse;	.	.	.
SMPD1	778.445	0.428994	0.128353858	3.342272	0.000831	0.010507	Not_Sig	no	gi|440896013|gb|ELR48055.1|	3044	0	Sphingomyelin phosphodiesterase, partial [Bos mutus]	gi|942089018|ref|XM_005907123.2|	2168	0	PREDICTED: Bos mutus sphingomyelin phosphodiesterase 1, acid lysosomal (SMPD1), mRNA	Q0VD19	1112	0	Sphingomyelin phosphodiesterase	.	.	.	.	pfam00149	Metallophos	Calcineurin-like phosphoesterase. This family includes a diverse range of phosphoesterases, including protein phosphoserine	GO:0046513|ceramide biosynthetic process;GO:0043407|negative regulation of MAP kinase activity;GO:0035307|positive regulation of protein dephosphorylation;GO:0006685|sphingomyelin catabolic process;GO:0023021|termination of signal transduction;	GO:0005768|endosome;GO:0070062|extracellular exosome;GO:0005615|extracellular space;GO:0005765|lysosomal membrane;GO:0005764|lysosome;GO:0045	GO:0016798|hydrolase activity, acting on glycosyl bonds;GO:0008270|	K12350	SMPD1, ASM; sphingomyelin phosphodiesterase [EC:3.1.4.12]
YIPF6	945.7941	-0.42068	0.125870554	-3.34218	0.0008312	0.010507	Not_Sig	no	gi|155372133|ref|NP_001094676.1|	1235	4e-153	protein YIPF6 [Bos taurus]	gi|982973754|ref|XM_010821854.2|	5349	0	PREDICTED: Bos taurus Yip1 domain family member 6 (YIPF6), transcript variant X1, mRNA	A6QLC6	480	1e-154	Protein YIPF6	SPAC644.13c	152	8.89e-41	COG5080	pfam04893	Yip1	Yip1 domain. The Yip1 integral membrane domain contains four transmembrane alpha helices. The domain is characterized by the	GO:0060576|intestinal epithelial cell development;	GO:0005801|cis-Golgi network;GO:0005783|endoplasmic reticulum;GO:0030134|ER to Golgi transport vesicle;GO:0016021|integral	.	.	.
NAGLU	747.7684	0.447104	0.13383436	3.340729	0.0008356	0.01055	Not_Sig	no	gi|528996185|ref|XP_005220827.1|	3668	0	PREDICTED: alpha-N-acetylglucosaminidase isoform X1 [Bos taurus]	gi|982957885|ref|XM_005220770.3|	2468	0	PREDICTED: Bos taurus N-acetylglucosaminidase, alpha (NAGLU), transcript variant X1, mRNA	P54802	1251	0	Alpha-N-acetylglucosaminidase	.	.	.	.	pfam05089	NAGLU	Alpha-N-acetylglucosaminidase (NAGLU) tim-barrel domain. Alpha-N-acetylglucosaminidase, a lysosomal enzyme required for the	GO:0021680|cerebellar Purkinje cell layer development;GO:0006027|glycosaminoglycan catabolic process;GO:0060119|inner ear receptor cell development;GO:0045475|locomotor rhythm;GO:0007040|lysosome organization;GO:0042474|middle ear morphogenesis;GO:0007399|nervous system development;GO:0046548|retinal rod cell development;	GO:0070062|extracellular exosome;GO:0043202|lysosomal lumen;GO:0005764|lysosome;	GO:0004561|alpha-N-acetylglucosaminidase activity;	K01205	NAGLU; alpha-N-acetylglucosaminidase [EC:3.2.1.50]
MOB1B	1075.577	-0.46408	0.138956604	-3.33974	0.0008386	0.010576	Not_Sig	no	gi|971393650|ref|XP_015131897.1|	1181	9e-144	PREDICTED: MOB kinase activator 1B [Gallus gallus]	gi|999844016|ref|NM_001192412.2|	6372	0	Bos taurus MOB kinase activator 1B (MOB1B), mRNA	Q8BPB0	454	3e-145	MOB kinase activator 1B	.	.	.	.	pfam03637	Mob1_phocein	Mob1/phocein family. Mob1 is an essential Saccharomyces cerevisiae protein, identified from a two-hybrid screen, that binds Mps1p, a	GO:0035329|hippo signaling;GO:0031952|regulation of protein autophosphorylation;	GO:0005737|cytoplasm;GO:0070062|extracellular exosome;GO:0005634|nucleus;	GO:0019209|kinase activator activity;GO:0019900|kinase	K06685	MOB1, Mats; MOB kinase activator 1
UQCRQ	495.9558	-0.65098	0.194951438	-3.33917	0.0008403	0.010579	DOWN	yes	gi|594052613|ref|XP_006050618.1|	683	4e-89	PREDICTED: cytochrome b-c1 complex subunit 8-like [Bubalus bubalis]	gi|594052612|ref|XM_006050556.1|	480	0	PREDICTED: Bubalus bubalis cytochrome b-c1 complex subunit 8-like (LOC102393778), mRNA	P13271	171	9e-54	Cytochrome b-c1 complex subunit 8	.	.	.	.	pfam02939	UcrQ	UcrQ family. The ubiquinol-cytochrome C reductase complex (cytochrome bc1 complex) is a respiratory multienzyme complex.	GO:0021680|cerebellar Purkinje cell layer development;GO:0021766|hippocampus development;GO:0021854|hypothalamus development;GO:0030901|midbrain development;GO:0021548|pons development;GO:0021860|pyramidal neuron development;GO:0021539|subthalamus development;GO:0021794|thalamus development;	GO:0005743|mitochondrial inner membrane;GO:0070469|respiratory chain;	GO:0008121|ubiquinol-cytochrome-c reductase activity;	K00418	QCR8, UQCRQ; ubiquinol-cytochrome c reductase subunit 8
SEC14L1	1893.783	0.335057	0.100344833	3.339061	0.0008406	0.010579	Not_Sig	no	gi|742187608|ref|XP_010854533.1|	3757	0	PREDICTED: SEC14-like protein 1 isoform X1 [Bison bison bison]	gi|742187607|ref|XM_010856231.1|	4307	0	PREDICTED: Bison bison bison SEC14-like 1 (S. cerevisiae) (SEC14L1), transcript variant X1, mRNA	Q92503	1355	0	SEC14-like protein 1	.	.	.	.	pfam04707	PRELI	PRELI-like family. This family includes a conserved region found in the PRELI protein and yeast YLR168C gene MSF1 product. The	GO:0015871|choline transport;GO:0045087|innate immune response;GO:0039536|negative regulation of RIG-I signaling pathway;	GO:0005737|cytoplasm;GO:0005794|Golgi apparatus;GO:0016021|integral component of membrane;	GO:0098772|molecular function regulator;GO:0039552|RIG-I binding;	.	.
IRF7	306.7702	0.491457	0.147280546	3.336878	0.0008473	0.010642	Not_Sig	no	gi|982972188|ref|XP_015316807.1|	2243	0	PREDICTED: interferon regulatory factor 7 isoform X1 [Bos taurus]	gi|555966245|ref|XM_005895114.1|	1873	0	PREDICTED: Bos mutus interferon regulatory factor 7 (IRF7), transcript variant X1, mRNA	Q92985	514	2e-174	Interferon regulatory factor 7	.	.	.	.	pfam10401	IRF-3	Interferon-regulatory factor 3. This is the interferon-regulatory factor 3 chain of the hetero-dimeric structure which also contains the	GO:0006974|cellular response to DNA damage stimulus;GO:0019043|establishment of viral latency;GO:0016064|immunoglobulin mediated immune response;GO:0045087|innate immune response;GO:0032607|interferon-alpha production;GO:0032608|interferon-beta production;GO:0060333|interferon-gamma-mediated signaling pathway;GO:0039530|MDA-5 signaling pathway;GO:2000110|negative regulation of macrophage apoptotic process;GO:0000122|negative regulation of transcription from RNA polymerase II	GO:0005737|cytoplasm;GO:0005829|cytosol;GO:0010008|endosome membrane;GO:0005654|nucleoplasm;GO:0005634|nucleus;	GO:0003677|DNA binding;GO:0000979|RNA polymerase II core promoter	K09447	IRF7; interferon regulatory factor 7
DAZAP1	958.6802	0.369882	0.110849162	3.336801	0.0008475	0.010642	Not_Sig	no	gi|803222506|ref|XP_011971526.1|	1260	3e-164	PREDICTED: DAZ-associated protein 1 isoform X4 [Ovis aries musimon]	gi|965800619|ref|XM_012116133.2|	2025	0	PREDICTED: Ovis aries musimon DAZ associated protein 1 (DAZAP1), transcript variant X2, mRNA	Q96EP5	489	6e-165	DAZ-associated protein 1	.	.	.	.	pfam00076	RRM_1	RNA recognition motif. (a.k.a. RRM, RBD, or RNP domain). The RRM motif is probably diagnostic of an RNA binding protein. RRMs	GO:0030154|cell differentiation;GO:0008283|cell proliferation;GO:0001893|maternal placenta development;GO:0048026|positive regulation of mRNA splicing, via spliceosome;GO:0007283|spermatogenesis;	GO:0005737|cytoplasm;GO:0005654|nucleoplasm;	GO:0000166|nucleotide binding;GO:0044822|poly(A) RNA	K14411	MSI; RNA-binding protein Musashi
YWHAH	3175.412	0.293492	0.087980811	3.335859	0.0008504	0.010667	Not_Sig	no	gi|6756037|ref|NP_035868.1|	1301	6e-175	14-3-3 protein eta [Mus musculus]	gi|162591|gb|J03868.1|BOV1433P	1673	0	Bovine brain-specific 14-3-3 protein eta chain mRNA, complete cds	P68511	505	2e-176	14-3-3 protein eta	SPAC8E11.02c	309	2.00e-101	COG5040	pfam00244	2014/3/3	14-3-3 protein.	GO:0086010|membrane depolarization during action potential;GO:2000649|regulation of sodium ion transmembrane transporter activity;GO:0002028|regulation of sodium ion transport;	GO:0005737|cytoplasm;	GO:0017080|sodium channel regulator activity;	K16198	YWHAG_H; 14-3-3 protein gamma/eta
SLC12A2	532.9802	-0.48432	0.145240815	-3.33458	0.0008543	0.010703	Not_Sig	no	gi|803073562|ref|XP_012033566.1|	5624	0	PREDICTED: solute carrier family 12 member 2 isoform X1 [Ovis aries]	gi|741932906|ref|XM_005209086.2|	6422	0	PREDICTED: Bos taurus solute carrier family 12 (sodium/potassium/chloride transporter), member 2 (SLC12A2), transcript variant X1, mRNA	P55011	2063	0	Solute carrier family 12 member 2	SPBC18H10.16	261	1.75e-70	COG0531	pfam00324	AA_permease	Amino acid permease.	GO:0007568|aging;GO:0015696|ammonium transport;GO:0007214|gamma-aminobutyric acid signaling pathway;GO:0006972|hyperosmotic response;GO:0006811|ion transport;GO:0045795|positive regulation of cell volume;GO:0006813|potassium ion transport;GO:0006814|sodium ion transport;GO:0070634|transepithelial ammonium transport;GO:0030321|transepithelial chloride transport;GO:0006810|transport;	GO:0016324|apical plasma membrane;GO:0070062|extracellular exosome;GO:1903561|extracellular vesicle;GO:0005887|integral component of	GO:0008519|ammonium transmembrane transporter	K10951	SLC12A2, NKCC1; solute carrier family 12 (sodium/potassium/
WBP2	1368.654	0.507465	0.1521947	3.334315	0.0008551	0.010703	Not_Sig	no	gi|594041572|ref|XP_006045332.1|	1018	1e-129	PREDICTED: WW domain-binding protein 2 isoform X1 [Bubalus bubalis]	gi|942092107|ref|XM_005907959.2|	1866	0	PREDICTED: Bos mutus WW domain binding protein 2 (WBP2), transcript variant X1, mRNA	Q969T9	337	2e-109	WW domain-binding protein 2	.	.	.	.	pfam10349	WWbp	WW-domain ligand protein. The WWbp domain is characterized by several short PY and PT-like motifs of the PPPPY form. These appear	GO:0071391|cellular response to estrogen stimulus;GO:0045184|establishment of protein localization;GO:0045815|positive regulation of gene expression, epigenetic;GO:0071442|positive regulation of histone H3-K14 acetylation;GO:0045944|positive regulation of transcription from RNA polymerase II promoter;	GO:0000790|nuclear chromatin;	GO:0031490|chromatin DNA binding;GO:0000979|RNA polymerase II	.	.
ATP13A1	963.2806	0.445498	0.133742228	3.331023	0.0008653	0.010818	Not_Sig	no	gi|742155079|ref|XP_010845543.1|	5993	0	PREDICTED: manganese-transporting ATPase 13A1 [Bison bison bison]	gi|300796400|ref|NM_001192809.1|	3677	0	Bos taurus ATPase type 13A1 (ATP13A1), mRNA	Q9HD20	2144	0	Manganese-transporting ATPase 13A1	SPACUNK4.07c	796	0	COG0474	pfam00122	E1-E2_ATPase	E1-E2 ATPase.	GO:0006874|cellular calcium ion homeostasis;GO:0034220|ion transmembrane transport;	GO:0005789|endoplasmic reticulum membrane;GO:0005887|integral component of plasma membrane;GO:0043231|intracellular	GO:0005524|ATP binding;GO:0019829|cation-transporting ATPase	K14950	ATP13A1; cation-transporting ATPase 13A1 [EC:3.6.3.-]
TRIQK	147.2132	-0.59772	0.179502986	-3.32987	0.0008689	0.010852	DOWN	yes	gi|741889896|ref|XP_010821023.1|	405	1e-41	PREDICTED: triple QxxK/R motif-containing protein isoform X1 [Bos taurus]	gi|982994755|ref|XM_015466397.1|	3563	0	PREDICTED: Bos taurus triple QxxK/R motif containing (TRIQK), transcript variant X5, mRNA	Q5RDR6	79.7	9e-16	Triple QxxK/R motif-containing protein	.	.	.	.	pfam15168	TRIQK	Triple QxxK/R motif-containing protein family. TRIQK member-proteins share a characteristic triple repeat of the sequence	.	GO:0005789|endoplasmic reticulum membrane;GO:0016021|integral component of membrane;	.	.	.
PSMA2	1090.821	-0.44984	0.135118059	-3.32926	0.0008708	0.010858	Not_Sig	no	gi|823426975|ref|XP_004411006.2|	1305	7e-179	PREDICTED: LOW QUALITY PROTEIN: proteasome subunit alpha type-2 [Odobenus rosmarus divergens]	gi|742225068|ref|XM_010829074.1|	920	0	PREDICTED: Bison bison bison proteasome (prosome, macropain) subunit, alpha type, 2 (PSMA2), mRNA	P25787	485	1e-172	Proteasome subunit alpha type-2	.	.	.	.	pfam00227	Proteasome	Proteasome subunit. The proteasome is a multisubunit structure that degrades proteins. Protein degradation is an essential	GO:0031145|anaphase-promoting complex-dependent proteasomal ubiquitin-dependent protein catabolic process;GO:0002479|antigen processing and presentation of exogenous peptide antigen via MHC class I, TAP-dependent;GO:0038095|Fc-epsilon receptor signaling pathway;GO:0000165|MAPK cascade;GO:0090090|negative regulation of canonical Wnt signaling pathway;GO:0051436|negative regulation of ubiquitin-protein ligase activity involved in mitotic cell cycle;GO:0038061|NIK/NF-kappaB signaling;GO:0090263|positive regulation of	GO:0000932|cytoplasmic mRNA processing body;GO:0005829|cytosol;GO:0070062|extracellular exosome;GO:0005654|nucleoplasm;GO:000	GO:0004298|threonine-type endopeptidase activity;	K02726	PSMA2; 20S proteasome subunit alpha 2 [EC:3.4.25.1]
ITPK1	530.1159	0.477843	0.143535104	3.329105	0.0008713	0.010858	Not_Sig	no	gi|300795104|ref|NP_001179418.1|	2174	0	inositol-tetrakisphosphate 1-kinase [Bos taurus]	gi|982960730|ref|XM_015459328.1|	3204	0	PREDICTED: Bos taurus inositol-tetrakisphosphate 1-kinase (ITPK1), transcript variant X2, mRNA	P0C0T1	842	0	Inositol-tetrakisphosphate 1-kinase	.	.	.	.	pfam05770	Ins134_P3_kin	Inositol 1, 3, 4-trisphosphate 5/6-kinase. This family consists of several inositol 1, 3, 4-trisphosphate 5/6-kinase proteins.	GO:0032957|inositol trisphosphate metabolic process;GO:0021915|neural tube development;	GO:0016324|apical plasma membrane;GO:0005622|intracellular;	GO:0005524|ATP binding;GO:0016787|hydrolase activity;GO:0047325	K00913	ITPK1; inositol-1,3,4-trisphosphate 5/6-kinase / inositol-tetrakisphosphate 1-
ADAMTS4	223.9688	0.530142	0.159307462	3.327794	0.0008754	0.010893	Not_Sig	no	gi|555995042|ref|XP_005909268.1|	4325	0	PREDICTED: A disintegrin and metalloproteinase with thrombospondin motifs 4 [Bos mutus]	gi|742091792|ref|XM_010833683.1|	3725	0	PREDICTED: Bison bison bison ADAM metallopeptidase with thrombospondin type 1 motif, 4 (ADAMTS4), mRNA	Q9TT93	1571	0	A disintegrin and metalloproteinase with thrombospondin motifs 4	.	.	.	.	pfam05986	ADAM_spacer1	ADAM-TS Spacer 1. This family represents the Spacer-1 region from the ADAM-TS family of metalloproteinases.	.	GO:0005578|proteinaceous extracellular matrix;	GO:0004222|metalloendopeptidase activity;GO:0008237|metallopeptidase	K07764	ADAMTS4; a disintegrin and metalloproteinase with
COMMD1	128.011	-0.67044	0.201479534	-3.32758	0.000876	0.010893	DOWN	yes	gi|594050189|ref|XP_006049453.1|	907	4e-121	PREDICTED: COMM domain-containing protein 1 [Bubalus bubalis]	gi|742216250|ref|XM_010863281.1|	723	0	PREDICTED: Bison bison bison copper metabolism (Murr1) domain containing 1 (COMMD1), mRNA	Q2M2T5	348	1e-120	COMM domain-containing protein 1	.	.	.	.	pfam07258	HCaRG	HCaRG protein. This family consists of several mammalian HCaRG(hypertension-related, calcium-regulated gene) proteins.	GO:0055070|copper ion homeostasis;GO:0032088|negative regulation of NF-kappaB transcription factor activity;GO:2000009|negative regulation of protein localization to cell surface;GO:1902306|negative regulation of sodium ion transmembrane transport;GO:0031398|positive regulation of protein ubiquitination;GO:0015031|protein transport;GO:0032434|regulation of proteasomal ubiquitin-dependent protein catabolic process;GO:0006351|transcription, DNA-templated;	GO:0031462|Cul2-RING ubiquitin ligase complex;GO:0005737|cytoplasm;GO:0016023|cytoplasmic membrane-bounded vesicle;GO:0005769|early	GO:0005507|copper ion binding;	.	.
ACTB	84769.92	0.369538	0.111098678	3.326211	0.0008804	0.010935	Not_Sig	no	gi|45269029|gb|AAS55927.1|	2071	0	cytoskeletal beta actin, partial [Sus scrofa]	gi|75832053|ref|NM_173979.3|	1808	0	Bos taurus actin, beta (ACTB), mRNA	P60707	779	0	Actin, cytoplasmic 1	SPBC32H8.12c	719	0	COG5277	pfam00022	Actin	Actin.	.	GO:0005856|cytoskeleton;GO:0097433|dense body;GO:0005925|focal adhesion;GO:0005886|plasma membrane;	GO:0005524|ATP binding;	.	.
LOC102278808	321.3461	-0.52961	0.159286791	-3.32489	0.0008845	0.010976	Not_Sig	no	gi|297475892|ref|XP_002688313.1|	4167	0	PREDICTED: RE1-silencing transcription factor [Bos taurus]	gi|982983281|ref|XM_867691.7|	6837	0	PREDICTED: Bos taurus RE1-silencing transcription factor (LOC615792), transcript variant X2, mRNA	Q13127	893	0	RE1-silencing transcription factor	.	.	.	.	pfam13465	zf-H2C2_2	Zinc-finger double domain.	GO:0060379|cardiac muscle cell myoblast differentiation;GO:0035690|cellular response to drug;GO:0071257|cellular response to electrical stimulus;GO:0071385|cellular response to glucocorticoid stimulus;GO:0002244|hematopoietic progenitor cell differentiation;GO:0070933|histone H4 deacetylation;GO:0043922|negative regulation by host of viral transcription;GO:0032348|negative regulation of aldosterone biosynthetic process;GO:2000798|negative regulation of amniotic stem cell differentiation;GO:0045955|negative	GO:0005829|cytosol;GO:0005654|nucleoplasm;GO:0005634|nucleus;GO:0017053|transcriptional repressor complex;	GO:0003682|chromatin binding;GO:0001047|core promoter	K09222	REST; RE1-silencing transcription factor
PPP1R26	593.1914	0.415746	0.125130071	3.322509	0.0008921	0.011058	Not_Sig	no	gi|296481977|tpg|DAA24092.1|	4079	0	TPA: hypothetical protein BOS_12285 [Bos taurus]	gi|982991637|ref|XM_005194069.3|	5346	0	PREDICTED: Bos taurus protein phosphatase 1 regulatory subunit 26 (PPP1R26), transcript variant X3, mRNA	Q5T8A7	315	1e-86	Protein phosphatase 1 regulatory subunit 26	.	.	.	.	pfam11725	AvrE	Pathogenicity factor. This family is secreted by gram-negative Gammaproteobacteria such as Pseudomonas syringae of tomato	GO:0010923|negative regulation of phosphatase activity;	GO:0005730|nucleolus;GO:0005654|nucleoplasm;	GO:0019902|phosphatase binding;GO:0004864|protein	K17565	PPP1R26; protein phosphatase 1 regulatory subunit 26
NDUFA12	281.2832	-0.48315	0.145475978	-3.32117	0.0008964	0.011096	Not_Sig	no	gi|555997205|ref|XP_005910330.1|	874	2e-116	PREDICTED: NADH dehydrogenase [ubiquinone] 1 alpha subcomplex subunit 12 [Bos mutus]	gi|926691241|ref|XM_005679827.2|	565	0	PREDICTED: Capra hircus NADH dehydrogenase (ubiquinone) 1 alpha subcomplex, 12 (NDUFA12), transcript variant X2, mRNA	O97725	301	3e-103	NADH dehydrogenase [ubiquinone] 1 alpha subcomplex subunit 12	AGc2444	57.4	3.56e-10	COG3761	pfam05071	NDUFA12	NADH ubiquinone oxidoreductase subunit NDUFA12. This family contains the 17.2 kD subunit of complex I (NDUFA12) and its	GO:0006979|response to oxidative stress;	GO:0005747|mitochondrial respiratory chain complex I;	GO:0009055|electron carrier activity;GO:0008137|NADH	K11352	NDUFA12; NADH dehydrogenase (ubiquinone) 1 alpha subcomplex subunit
TMEM126A	380.0146	-0.46833	0.141024211	-3.32095	0.0008971	0.011096	Not_Sig	no	gi|529011546|ref|XP_005226731.1|	925	5e-123	PREDICTED: transmembrane protein 126A isoform X1 [Bos taurus]	gi|982971061|ref|XM_005226674.3|	829	0	PREDICTED: Bos taurus transmembrane protein 126A (TMEM126A), transcript variant X1, mRNA	Q32L86	337	1e-115	Transmembrane protein 126A	.	.	.	.	pfam07114	DUF1370	Protein of unknown function (DUF1370). This family consists of several hypothetical eukaryotic proteins of around 200 residues in	.	GO:0016021|integral component of membrane;GO:0005743|mitochondrial inner membrane;GO:0005739|mitochondrion;	.	K18157	TMEM126A; transmembrane protein 126A
MOB3A	739.9799	0.505224	0.152230797	3.318804	0.000904	0.011157	Not_Sig	no	gi|440912182|gb|ELR61774.1|	1257	2e-165	Mps one binder kinase activator-like 2A, partial [Bos mutus]	gi|742217552|ref|XM_010863619.1|	2334	0	PREDICTED: Bison bison bison MOB kinase activator 3A (MOB3A), transcript variant X3, mRNA	Q58D63	458	3e-155	MOB kinase activator 3A	.	.	.	.	pfam03637	Mob1_phocein	Mob1/phocein family. Mob1 is an essential Saccharomyces cerevisiae protein, identified from a two-hybrid screen, that binds Mps1p, a	.	GO:0005622|intracellular;	GO:0046872|metal ion binding;	.	.
GBF1	1456.323	0.361946	0.109059414	3.318799	0.0009041	0.011157	Not_Sig	no	gi|555951860|ref|XP_005888125.1|	9175	0	PREDICTED: Golgi-specific brefeldin A-resistance guanine nucleotide exchange factor 1 isoform X1 [Bos mutus]	gi|555951859|ref|XM_005888063.1|	6407	0	PREDICTED: Bos mutus golgi brefeldin A resistant guanine nucleotide exchange factor 1 (GBF1), transcript variant X1, mRNA	Q92538	3375	0	Golgi-specific brefeldin A-resistance guanine nucleotide exchange factor 1	SPBC211.03c	234	3.36e-61	COG5307	pfam01369	Sec7	Sec7 domain. The Sec7 domain is a guanine-nucleotide-exchange-factor (GEF) for the pfam00025 family.	GO:0002263|cell activation involved in immune response;GO:0048205|COPI coating of Golgi vesicle;GO:0097111|endoplasmic reticulum-Golgi intermediate compartment organization;GO:0006888|ER to Golgi vesicle-mediated transport;GO:0061162|establishment of monopolar cell polarity;GO:0090166|Golgi disassembly;GO:0007030|Golgi organization;GO:0006895|Golgi to endosome transport;GO:0030593|neutrophil chemotaxis;GO:0006892|post-Golgi vesicle-mediated transport;GO:0070973|protein localization to endoplasmic reticulum exit	GO:0031252|cell leading edge;GO:0005801|cis-Golgi network;GO:0005829|cytosol;GO:0005788|endoplasmic reticulum	GO:0005086|ARF guanyl-nucleotide exchange factor activity;GO:0005085	K18443	GBF1; golgi-specific brefeldin A-resistance guanine nucleotide exchange
FBXW5	673.9983	0.63518	0.191489897	3.317039	0.0009098	0.011208	UP	yes	gi|300794688|ref|NP_001179398.1|	2836	0	F-box/WD repeat-containing protein 5 [Bos taurus]	gi|982940253|ref|XM_010810611.2|	1687	0	PREDICTED: Bos taurus F-box and WD repeat domain containing 5 (FBXW5), transcript variant X2, mRNA	Q969U6	1012	0	F-box/WD repeat-containing protein 5	.	.	.	.	pfam00400	WD40	WD domain, G-beta repeat.	GO:0007067|mitotic nuclear division;GO:0043161|proteasome-mediated ubiquitin-dependent protein catabolic process;GO:0016567|protein ubiquitination;GO:0010824|regulation of centrosome duplication;GO:0031146|SCF-dependent proteasomal ubiquitin-dependent protein catabolic process;	GO:0080008|Cul4-RING E3 ubiquitin ligase complex;GO:0005737|cytoplasm;GO:0019005|SCF ubiquitin ligase complex;	GO:0019901|protein kinase binding;	K10263	FBXW5; F-box and WD-40 domain protein 5
ZNF777	324.163	0.488395	0.147258136	3.316593	0.0009112	0.011208	Not_Sig	no	gi|926690844|ref|XP_013819023.1|	3951	0	PREDICTED: zinc finger protein 777 [Capra hircus]	gi|300794766|ref|NM_001192473.1|	2804	0	Bos taurus zinc finger protein 777 (ZNF777), mRNA	Q9ULD5	1218	0	Zinc finger protein 777	.	.	.	.	pfam01352	KRAB	KRAB box. The KRAB domain (or Kruppel-associated box) is present in about a third of zinc finger proteins containing C2H2 fingers.	GO:0006355|regulation of transcription, DNA-templated;GO:0006351|transcription, DNA-templated;	GO:0005634|nucleus;	GO:0003677|DNA binding;GO:0046872|metal ion binding;	K09228	KRAB; KRAB domain-containing zinc finger protein
MED15	824.1997	0.496893	0.149820524	3.316587	0.0009112	0.011208	Not_Sig	no	gi|741957543|ref|XP_010812553.1|	1434	0	PREDICTED: mediator of RNA polymerase II transcription subunit 15 isoform X1 [Bos taurus]	gi|402745556|ref|NM_001099175.2|	2768	0	Bos taurus mediator complex subunit 15 (MED15), mRNA	Q96RN5	511	9e-166	Mediator of RNA polymerase II transcription subunit 15	.	.	.	.	pfam09606	Med15	ARC105 or Med15 subunit of Mediator complex non-fungal. The approx. 70 residue Med15 domain of the ARC-Mediator co-activator	GO:0006367|transcription initiation from RNA polymerase II promoter;	GO:0005737|cytoplasm;GO:0016592|mediator complex;GO:0016020|membrane;GO:0005654|nucleoplasm;GO:0005634|nucleus;	GO:0001104|RNA polymerase II transcription cofactor activity;	K15157	MED15; mediator of RNA polymerase II transcription subunit 15
UBE2B	1200.656	-0.46112	0.13904363	-3.31633	0.0009121	0.011208	Not_Sig	no	gi|472366596|ref|XP_004402835.1|	749	9e-93	PREDICTED: ubiquitin-conjugating enzyme E2 B-like isoform X1 [Odobenus rosmarus divergens]	gi|942040203|ref|XM_005890411.2|	1493	0	PREDICTED: Bos mutus ubiquitin-conjugating enzyme E2B (UBE2B), transcript variant X1, mRNA	P63149	301	2e-97	Ubiquitin-conjugating enzyme E2 B	SPAC18B11.07c	224	1.20e-69	COG5078	pfam00179	UQ_con	Ubiquitin-conjugating enzyme. Proteins destined for proteasome-mediated degradation may be ubiquitinated. Ubiquitination	GO:0032869|cellular response to insulin stimulus;GO:0006281|DNA repair;GO:0016574|histone ubiquitination;GO:0043161|proteasome-mediated ubiquitin-dependent protein catabolic process;GO:0070979|protein K11-linked ubiquitination;GO:0070936|protein K48-linked ubiquitination;GO:0070534|protein K63-linked ubiquitination;GO:0000209|protein polyubiquitination;GO:0001666|response to hypoxia;GO:0006511|ubiquitin-dependent protein catabolic process;	GO:0005737|cytoplasm;GO:0033503|HULC complex;GO:0000790|nuclear chromatin;GO:0005886|plasma membrane;	GO:0005524|ATP binding;GO:0061630|ubiquitin protein ligase	K10574	UBE2B, RAD6B; ubiquitin-conjugating enzyme E2 B [EC:2.3.2.23]

TMTC3	725.3435	-0.53739	0.162085772	-3.31546	0.0009149	0.011231	Not_Sig	no	gi|358412129|ref|XP_600406.6|	4548	0	PREDICTED: transmembrane and TPR repeat-containing protein 3 [Bos taurus]	gi|982981516|ref|XM_600406.9|	6267	0	PREDICTED: Bos taurus transmembrane and tetratricopeptide repeat containing 3 (TMTC3), mRNA	Q6ZXV5	1642	0	Transmembrane and TPR repeat-containing protein 3	MTH72	77	1.40e-13	COG0457	pfam08409	DUF1736	Domain of unknown function (DUF1736). This domain of unknown function is found in various hypothetical metazoan	.	GO:0016021|integral component of membrane;	.	.	.
LAMTOR4	357.5309	-0.48579	0.146544839	-3.31494	0.0009166	0.01124	Not_Sig	no	gi|440908206|gb|ELR58253.1|	598	6e-76	hypothetical protein M91_05947, partial [Bos mutus]	gi|942047566|ref|XM_005893145.2|	607	0	PREDICTED: Bos mutus late endosomal/lysosomal adaptor, MAPK and MTOR activator 4 (LAMTOR4), mRNA	Q2M2U3	200	3e-64	Ragulator complex protein LAMTOR4	.	.	.	.	pfam05404	TRAP-delta	Translocon-associated protein, delta subunit precursor (TRAP-delta). This family consists of several eukaryotic translocon-	GO:0071230|cellular response to amino acid stimulus;GO:0032008|positive regulation of TOR signaling;GO:0061462|protein localization to lysosome;GO:0008361|regulation of cell size;	GO:0005764|lysosome;GO:0071986|Ragulator complex;	GO:0005085|guanyl-nucleotide exchange factor activity;	.	.
GLIPR1	275.1084	-0.46272	0.139600104	-3.31462	0.0009177	0.011241	Not_Sig	no	gi|555995926|ref|XP_005909702.1|	1293	1e-176	PREDICTED: glioma pathogenesis-related protein 1 [Bos mutus]	gi|594048667|ref|XM_006048661.1|	951	0	PREDICTED: Bubalus bubalis GLI pathogenesis-related 1 (GLIPR1), transcript variant X2, mRNA	P48060	406	2e-140	Glioma pathogenesis-related protein 1	YJL078c_1	65.9	3.02e-12	COG2340	pfam00188	CAP	Cysteine-rich secretory protein family. This is a large family of cysteine-rich secretory proteins, antigen 5, and pathogenesis-	.	GO:0005576|extracellular region;GO:0016021|integral component of membrane;GO:0016020|membrane;GO:0005886|plasma membrane;	.	.	.
CD276	2824.278	0.505429	0.152508531	3.314101	0.0009194	0.01125	Not_Sig	no	gi|594075432|ref|XP_006061492.1|	2585	0	PREDICTED: CD276 antigen isoform X1 [Bubalus bubalis]	gi|982988686|ref|XM_005193592.3|	3037	0	PREDICTED: Bos taurus CD276 molecule (CD276), transcript variant X2, mRNA	Q5ZPR3	931	0	CD276 antigen	.	.	.	.	pfam08205	C2-set_2	CD80-like C2-set immunoglobulin domain. These domains belong to the immunoglobulin superfamily.	GO:0008283|cell proliferation;GO:0006955|immune response;GO:0050728|negative regulation of inflammatory response;GO:0045077|negative regulation of interferon-gamma biosynthetic process;GO:0045085|negative regulation of interleukin-2 biosynthetic process;GO:0042130|negative regulation of T cell proliferation;GO:0030501|positive regulation of bone mineralization;GO:0045078|positive regulation of interferon-gamma biosynthetic process;GO:1900042|positive regulation of interleukin-2	GO:0009897|external side of plasma membrane;GO:0070062|extracellular exosome;GO:0016021|integral component of membrane;	GO:0005102|receptor binding;	K06746	B7H3, CD276; immune costimulatory protein B7-H3
DENND4B	308.8094	0.590068	0.178198469	3.311299	0.0009286	0.011351	UP	yes	gi|594039220|ref|XP_006044204.1|	6300	0	PREDICTED: DENN domain-containing protein 4B isoform X1 [Bubalus bubalis]	gi|594039219|ref|XM_006044142.1|	5092	0	PREDICTED: Bubalus bubalis DENN/MADD domain containing 4B (DENND4B), transcript variant X1, mRNA	O75064	2335	0	DENN domain-containing protein 4B	.	.	.	.	pfam02141	DENN	DENN (AEX-3) domain. DENN (after differentially expressed in neoplastic vs normal cells) is a domain which occurs in several	GO:0032483|regulation of Rab protein signal transduction;	GO:0005794|Golgi apparatus;GO:0005634|nucleus;	GO:0017112|Rab guanyl-nucleotide exchange factor activity;	.	.
SLC4A7	821.6415	-0.38833	0.117294574	-3.31075	0.0009305	0.011361	Not_Sig	no	gi|529000504|ref|XP_005222460.1|	5889	0	PREDICTED: sodium bicarbonate cotransporter 3 isoform X1 [Bos taurus]	gi|982961158|ref|XM_010817430.2|	7785	0	PREDICTED: Bos taurus solute carrier family 4, sodium bicarbonate cotransporter, member 7 (SLC4A7), transcript variant X2, mRNA	Q9R1N3	2072	0	Sodium bicarbonate cotransporter 3	.	.	.	.	pfam00955	HCO3_cotransp	HCO3- transporter family. This family contains Band 3 anion exchange proteins that exchange CL-/HCO3-. This family also	GO:0098656|anion transmembrane transport;GO:0060117|auditory receptor cell development;GO:0015701|bicarbonate transport;GO:0051453|regulation of intracellular pH;GO:0006814|sodium ion transport;	GO:0016324|apical plasma membrane;GO:0016323|basolateral plasma membrane;GO:0031410|cytoplasmic vesicle;GO:0005887|integral component of	GO:0008509|anion transmembrane transporter activity;GO:0005452	K13858	SLC4A7; solute carrier family 4 (sodium bicarbonate cotransporter),
PLS3	3331.321	-0.41355	0.125027695	-3.30764	0.0009409	0.011476	Not_Sig	no	gi|529014725|ref|XP_005227971.1|	3290	0	PREDICTED: plastin-3 isoform X1 [Bos taurus]	gi|982973493|ref|XM_005227914.3|	3206	0	PREDICTED: Bos taurus plastin 3 (PLS3), transcript variant X1, mRNA	A7E3Q8	1271	0	Plastin-3	SPBC1778.06c	424	2.56e-135	COG5069	pfam00307	CH	Calponin homology (CH) domain. The CH domain is found in both cytoskeletal proteins and signal transduction proteins. The CH	GO:0051764|actin crosslink formation;GO:0051017|actin filament bundle assembly;GO:0051639|actin filament network formation;	GO:0005884|actin filament;GO:0032432|actin filament bundle;GO:0005737|cytoplasm;	GO:0051015|actin filament binding;GO:0005509|calcium ion	.	.
SREBF1	1170.866	0.44735	0.135285239	3.306716	0.000944	0.011502	Not_Sig	no	gi|296476600|tpg|DAA18715.1|	4448	0	TPA: sterol regulatory element binding transcription factor 1 [Bos taurus]	gi|742136487|ref|XM_010842897.1|	4016	0	PREDICTED: Bison bison bison sterol regulatory element binding transcription factor 1 (SREBF1), mRNA	O97676	1468	0	Sterol regulatory element-binding protein 1	.	.	.	.	pfam00010	HLH	Helix-loop-helix DNA-binding domain.	GO:0008203|cholesterol metabolic process;GO:0008610|lipid biosynthetic process;GO:0010867|positive regulation of triglyceride biosynthetic process;GO:0006355|regulation of transcription, DNA-templated;GO:0006351|transcription, DNA-templated;	GO:0005737|cytoplasm;GO:0005789|endoplasmic reticulum membrane;GO:0012507|ER to Golgi transport vesicle	GO:0003677|DNA binding;	K07197	SREBP1, SREBF1; sterol regulatory element-binding transcription factor 1
UHRF2	1051.586	-0.35569	0.107578223	-3.30634	0.0009452	0.011505	Not_Sig	no	gi|555990351|ref|XP_005906970.1|	4163	0	PREDICTED: E3 ubiquitin-protein ligase UHRF2 [Bos mutus]	gi|965904931|ref|XM_012164326.2|	3470	0	PREDICTED: Ovis aries musimon ubiquitin-like with PHD and ring finger domains 2, E3 ubiquitin protein ligase (UHRF2), mRNA	Q96PU4	1472	0	E3 ubiquitin-protein ligase UHRF2	DR1533	103	3.83e-23	COG3440	pfam02182	SAD_SRA	SAD/SRA domain. The domain goes by several names including SAD, SRA and YDG. It adopts a beta barrel, modified PUA-like, fold that	GO:0007049|cell cycle;GO:0030154|cell differentiation;GO:0008283|cell proliferation;GO:0010216|maintenance of DNA methylation;GO:0071158|positive regulation of cell cycle arrest;GO:0051865|protein autoubiquitination;GO:0016567|protein ubiquitination;GO:0051726|regulation of cell cycle;GO:0090308|regulation of methylation-dependent chromatin silencing;	GO:0005720|nuclear heterochromatin;GO:0005654|nucleoplasm;GO:0005634|nucleus;	GO:0003677|DNA binding;GO:0042393|histone binding;GO:0016874	K15713	UHRF2, NIRF; E3 ubiquitin-protein ligase UHRF2 [EC:2.3.2.27]
CDC42EP4	333.3213	0.422512	0.127820534	3.305506	0.000948	0.011527	Not_Sig	no	gi|114050763|ref|NP_001039936.1|	986	8e-121	cdc42 effector protein 4 [Bos taurus]	gi|982958748|ref|XM_005221257.3|	2558	0	PREDICTED: Bos taurus CDC42 effector protein 4 (CDC42EP4), transcript variant X6, mRNA	Q9H3Q1	299	2e-90	Cdc42 effector protein 4	.	.	.	.	pfam14957	BORG_CEP	Cdc42 effector. The Cdc42 effector (CEP) or binder of Rho GTPases (BORG) proteins are involved in the organisation of the actin	GO:0030838|positive regulation of actin filament polymerization;GO:0031274|positive regulation of pseudopodium assembly;GO:0008360|regulation of cell shape;GO:0007266|Rho protein signal transduction;	GO:0015629|actin cytoskeleton;GO:0005911|cell-cell junction;GO:0005737|cytoplasm;GO:0012505|endomembrane	GO:0017049|GTP-Rho binding;GO:0005096|GTPase activator	.	.
SARNP	442.2688	-0.62739	0.189898881	-3.3038	0.0009538	0.011581	DOWN	yes	gi|528949768|ref|XP_005206762.1|	761	9e-97	PREDICTED: SAP domain-containing ribonucleoprotein isoform X1 [Bos taurus]	gi|982925970|ref|XM_005206705.3|	1091	0	PREDICTED: Bos taurus SAP domain containing ribonucleoprotein (SARNP), transcript variant X1, mRNA	Q9D1J3	256	4e-82	SAP domain-containing ribonucleoprotein	.	.	.	.	pfam02037	SAP	SAP domain. The SAP (after SAF-A/B, Acinus and PIAS) motif is a putative DNA/RNA binding domain found in diverse nuclear and	GO:0006406|mRNA export from nucleus;GO:0000122|negative regulation of transcription from RNA polymerase II promoter;GO:0006417|regulation of translation;GO:0006351|transcription, DNA-templated;	GO:0043231|intracellular membrane-bounded organelle;GO:0016607|nuclear speck;GO:0005654|nucleoplasm;GO:0005634|nucleus;GO:0000346|transcription export	GO:0044822|poly (A) RNA binding;GO:0008022|protein C-terminus	K18732	SARNP, CIP29, THO1; SAP domain-containing ribonucleoprotein
MMS19	680.255	0.458392	0.138755336	3.303598	0.0009545	0.011581	Not_Sig	no	gi|555978574|ref|XP_005901225.1|	4956	0	PREDICTED: MMS19 nucleotide excision repair protein homolog isoform X1 [Bos mutus]	gi|555978573|ref|XM_005901163.1|	3482	0	PREDICTED: Bos mutus MMS19 homolog, cytosolic iron-sulfur assembly component (MMS19), transcript variant X1, mRNA	E1BP36	1911	0	MMS19 nucleotide excision repair protein homolog	.	.	.	.	pfam14500	MMS19_N	Dos2-interacting transcription regulator of RNA-Pol-II. This domain, along with the C-terminal part, pfam12460, is an essential	GO:0006974|cellular response to DNA damage stimulus;GO:0007059|chromosome segregation;GO:0006259|DNA metabolic process;GO:0006281|DNA repair;GO:0016226|iron-sulfur cluster assembly;GO:0006355|regulation of transcription, DNA-templated;GO:0006351|transcription, DNA-templated;	GO:0097361|CIA complex;GO:0005737|cytoplasm;GO:0005634|nucleus;GO:0005819|spindle;	.	K15075	MET18, MMS19; DNA repair/transcription protein
UPF1	1565.018	0.324379	0.098198161	3.303306	0.0009555	0.011581	Not_Sig	no	gi|528954426|ref|XP_005208537.1|	5617	0	PREDICTED: regulator of nonsense transcripts 1 isoform X1 [Bos taurus]	gi|982984035|ref|XM_015463936.1|	5190	0	PREDICTED: Bos taurus UPF1 regulator of nonsense transcripts homolog (yeast) (UPF1), transcript variant X1, mRNA	Q92900	2158	0	Regulator of nonsense transcripts 1	SPAC16C9.06c	1048	0	COG1112	pfam09416	UPF1_Zn_bind	RNA helicase (UPF2 interacting domain). UPF1 is an essential RNA helicase that detects mRNAs containing premature stop codons	GO:0061158|3'-UTR-mediated mRNA destabilization;GO:0071347|cellular response to interleukin-1;GO:0071222|cellular response to lipopolysaccharide;GO:0006281|DNA repair;GO:0006260|DNA replication;GO:0009048|dosage compensation by inactivation of X chromosome;GO:0071044|histone mRNA catabolic process;GO:0006406|mRNA export from nucleus;GO:0000956|nuclear-transcribed mRNA catabolic process;GO:0000294|nuclear-transcribed mRNA catabolic process, endonucleolytic cleavage-dependent decay;GO:0000184|nuclear-	GO:0000785|chromatin;GO:0005737|cytoplasm;GO:0000932|cytoplasmic mRNA processing body;GO:0005829|cytosol;GO:0035145|exo	GO:0005524|ATP binding;GO:0004004|ATP-dependent RNA helicase	K14326	UPF1, RENT1; regulator of nonsense transcripts 1 [EC:3.6.4.-]
RCAN1	1430.119	-0.35239	0.106701508	-3.3026	0.0009579	0.011597	Not_Sig	no	gi|528935790|ref|XP_005201167.1|	1186	7e-154	PREDICTED: calcipressin-1 isoform X1 [Bos taurus]	gi|982909390|ref|XM_005201110.3|	2435	0	PREDICTED: Bos taurus regulator of calcineurin 1 (RCAN1), transcript variant X1, mRNA	P53805	417	2e-138	Calcipressin-1	.	.	.	.	pfam04847	Calcipressin	Calcipressin. Calcipressin is also known as calcineurin-binding protein, since it inhibits calcineurin-mediated	GO:0008015|blood circulation;GO:0033173|calcineurin-NFAT signaling cascade;GO:0007417|central nervous system development;GO:0070884|regulation of calcineurin-NFAT signaling cascade;GO:0007165|signal transduction;	GO:0005737|cytoplasm;GO:0005634|nucleus;	GO:0008597|calcium-dependent protein serine/threonine	K17901	RCAN1, MCIP1; calcipressin-1
MAPK3	996.8088	0.489825	0.148327591	3.302322	0.0009589	0.011597	Not_Sig	no	gi|158517957|ref|NP_001103488.1|	1866	0	mitogen-activated protein kinase 3 [Bos taurus]	gi|982966988|ref|XM_005224919.3|	1789	0	PREDICTED: Bos taurus mitogen-activated protein kinase 3 (MAPK3), transcript variant X1, mRNA	P21708	714	0	Mitogen-activated protein kinase 3	.	.	.	.	pfam00069	Pkinase	Protein kinase domain.	GO:0006915|apoptotic process;GO:0019369|arachidonic acid metabolic process;GO:0060020|Bergmann glial cell differentiation;GO:0030509|BMP signaling pathway;GO:0061308|cardiac neural crest cell development involved in heart development;GO:0051216|cartilage development;GO:0072584|caveolin-mediated endocytosis;GO:0007049|cell cycle;GO:0071260|cellular response to mechanical stimulus;GO:0006975|DNA damage induced protein phosphorylation;GO:0070371|ERK1 and ERK2	GO:0005901|caveola;GO:0005856|cytoskeleton;GO:0005829|cytosol;GO:0005769|early endosome;GO:0070062|extracellular exosome;GO:0005925|focal	GO:0005524|ATP binding;GO:0004707|MAP kinase activity;GO:0004674	K04371	MAPK1_3; mitogen-activated protein kinase 1/3 [EC:2.7.11.24]
ZNF592	499.1551	0.521375	0.157994569	3.299952	0.000967	0.011683	Not_Sig	no	gi|965835548|ref|XP_014962425.1|	5655	0	PREDICTED: zinc finger protein 592 isoform X1 [Ovis aries musimon]	gi|742124463|ref|XM_010841080.1|	4999	0	PREDICTED: Bison bison bison zinc finger protein 592 (ZNF592), transcript variant X2, mRNA	Q92610	1824	0	Zinc finger protein 592	.	.	.	.	pfam03154	Atrophin-1	Atrophin-1 family. Atrophin-1 is the protein product of the dentatorubral-pallidoluysian atrophy (DRPLA) gene. DRPLA	GO:0006355|regulation of transcription, DNA-templated;GO:0006351|transcription, DNA-templated;	GO:0005634|nucleus;	GO:0003677|DNA binding;GO:0046872|metal ion binding;	K19325	ZNF592; zinc finger protein 592
PPTC7	992.1751	-0.43051	0.130504225	-3.29882	0.0009709	0.011718	Not_Sig	no	gi|507663213|ref|XP_004635970.1|	1505	0	PREDICTED: protein phosphatase PTC7 homolog [Octodon degus]	gi|742130613|ref|XM_010841371.1|	4889	0	PREDICTED: Bison bison bison PTC7 protein phosphatase homolog (S. cerevisiae) (PPTC7), mRNA	Q8NI37	580	0	Protein phosphatase PTC7 homolog	SPAC1556.03	120	8.09e-29	COG0631	pfam07228	SpoIIE	Stage II sporulation protein E (SpoIIE). This family contains a number of bacterial stage II sporulation E proteins	GO:0006470|protein dephosphorylation;	GO:0005739|mitochondrion;	GO:0046872|metal ion binding;GO:0004722|protein	.	.
PLTP	62.89898	-0.79478	0.240951754	-3.2985	0.000972	0.011719	DOWN	yes	gi|528973460|ref|XP_005214615.1|	2417	0	PREDICTED: phospholipid transfer protein isoform X1 [Bos taurus]	gi|982942916|ref|XM_005214558.2|	1773	0	PREDICTED: Bos taurus phospholipid transfer protein (PLTP), transcript variant X1, mRNA	P55058	853	0	Phospholipid transfer protein	.	.	.	.	pfam02886	LBP_BPI_CETP_C	LBP / BPI / CETP family, C-terminal domain. The N and C terminal domains of the LBP/BPI/CETP family are structurally similar.	GO:0030317|sperm motility;GO:0034375|high-density lipoprotein particle remodeling;GO:0006629|lipid metabolic process;GO:0006869|lipid transport;GO:0010875|positive regulation of cholesterol efflux;GO:0010189|vitamin E biosynthetic process;	GO:0005576|extracellular region;GO:0005615|extracellular space;	GO:0008289|lipid binding;	K08761	PLTP; phospholipid transfer protein
ZDHHC9	1650.41	0.36313	0.110098989	3.298213	0.000973	0.011719	Not_Sig	no	gi|11360232|pir||T47144	1710	0	hypothetical protein DKFZp761E1347.1 - human (fragment)	gi|982972651|ref|XM_005227490.2|	2901	0	PREDICTED: Bos taurus zinc finger, DHHC-type containing 9 (ZDHHC9), transcript variant X1, mRNA	Q58DA8	658	0	Palmitoyltransferase ZDHHC9	SPBC3H7.09	143	1.32e-36	COG5273	pfam01529	zf-DHHC	DHHC palmitoyltransferase. This family includes the well known DHHC zinc binding domain as well as three of the four conserved	GO:0018230|peptidyl-L-cysteine S-palmitoylation;	GO:0005783|endoplasmic reticulum;GO:0005789|endoplasmic reticulum membrane;GO:0016021|integral component of	GO:0019706|protein-cysteine S-palmitoyltransferase activity;GO:0043849	K16675	ZDHHC9_14_18; palmitoyltransferase ZDHHC9/14/18 [EC:2.3.1.225]
CRKL	2630.48	0.399408	0.121130903	3.297324	0.0009761	0.011722	Not_Sig	no	gi|555974669|ref|XP_005899327.1|	1308	5e-167	PREDICTED: crk-like protein [Bos mutus]	gi|982998711|ref|XM_015467227.1|	3282	0	PREDICTED: Bos taurus v-crk avian sarcoma virus CT10 oncogene homolog-like (CRKL), transcript variant X2, mRNA	P46109	483	5e-159	Crk-like protein	.	.	.	.	pfam00017	SH2	SH2 domain.	GO:0000186|activation of MAPKK activity;GO:0009887|organ morphogenesis;GO:0009952|anterior/posterior pattern specification;GO:0001568|blood vessel development;GO:0007507|heart development;GO:0035556|intracellular signal transduction;GO:0007254|JNK cascade;GO:0060017|parathyroid gland development;GO:0008284|positive regulation of cell proliferation;GO:1900026|positive regulation of substrate adhesion-dependent cell spreading;GO:0007265|Ras protein signal	GO:0005913|cell-cell adherens junction;GO:0005829|cytosol;GO:0005768|endosome;GO:0070062|extracellular exosome;	GO:0098641|cadherin binding involved in cell-cell adhesion;GO:00448	K04438	CRK, CRKII; proto-oncogene C-crk
JAZF1	259.8708	-0.50288	0.152516889	-3.29719	0.0009766	0.011722	Not_Sig	no	gi|91822911|ref|NP_778231.2|	1011	5e-126	juxtaposed with another zinc finger protein 1 [Homo sapiens]	gi|942042442|ref|XM_005891431.2|	2904	0	PREDICTED: Bos mutus JAZF zinc finger 1 (JAZF1), mRNA	Q5RDF5	373	1e-119	Juxtaposed with another zinc finger protein 1	SPAC16.05c	63.2	1.86e-09	COG5189	pfam15002	ERK-JNK_inhib	ERK and JNK pathways, inhibitor. This coiled-coiled domain, CCDC134, is a secretory protein that inhibits Mitogen activated	GO:0006629|lipid metabolic process;GO:0006355|regulation of transcription, DNA-templated;GO:0006351|transcription, DNA-templated;	GO:0005634|nucleus;	GO:0046872|metal ion binding;GO:0003676|nucleic acid	K19495	JAZF1; juxtaposed with another zinc finger protein 1
HASPIN	639.2977	0.398986	0.121012886	3.297055	0.000977	0.011722	Not_Sig	no	gi|115495205|ref|NP_001070012.1|	3779	0	serine/threonine-protein kinase haspin [Bos taurus]	gi|594114046|ref|XM_006079694.1|	2562	0	PREDICTED: Bubalus bubalis germ cell associated 2 (haspin) (GSG2), mRNA	Q2KIP2	1437	0	Serine/threonine-protein kinase haspin	SPAC23C4.03	179	1.04e-47	COG5072	pfam12330	DUF3635	Domain of unknown function (DUF3635). This family may be a potential Haspin-related leucine-zipper. A leucine zipper was	GO:2000751|histone H3-T3 phosphorylation involved in chromosome passenger complex localization to kinetochore;GO:0035556|intracellular signal transduction;GO:0007064|mitotic sister chromatid cohesion;GO:0071459|protein localization to chromosome, centromeric region;GO:0090231|regulation of spindle checkpoint;	GO:0005813|centrosome;GO:0005694|chromosome;GO:0005737|cytoplasm;GO:0005634|nucleus;GO:0005819|spindle;	GO:0005524|ATP binding;GO:0072354|histone kinase activity (H3-T3	K16315	GSG2; serine/threonine-protein kinase haspin [EC:2.7.11.1]
PAQR4	206.1329	0.579874	0.175881358	3.296958	0.0009774	0.011722	Not_Sig	no	gi|149642563|ref|NP_001092367.1|	1211	2e-159	progestin and adipoQ receptor family member 4 [Bos taurus]	gi|149642562|ref|NM_001098897.1|	1914	0	Bos taurus progestin and adipoQ receptor family member IV (PAQR4), mRNA	Q8N4S7	447	9e-152	Progestin and adipoQ receptor family member 4	.	.	.	.	pfam03006	HlyIII	Haemolysin-III related. Members of this family are integral membrane proteins. This family includes a protein with hemolytic	.	GO:0016021|integral component of membrane;	GO:0004872|receptor activity;	.	.
IRAK1	1217.645	0.398829	0.120996088	3.296212	0.00098	0.011741	Not_Sig	no	gi|296471074|tpg|DAA13189.1|	2607	0	TPA: interleukin-1 receptor-associated kinase 1 [Bos taurus]	gi|926730384|ref|XR_001297594.1|	2956	0	PREDICTED: Capra hircus interleukin-1 receptor-associated kinase 1 (IRAK1), transcript variant X1, misc_RNA	Q2LGB3	1007	0	Interleukin-1 receptor-associated kinase 1	Cgl2127_1	89.4	9.07e-18	COG0515	pfam00069	Pkinase	Protein kinase domain.	GO:0045087|innate immune response;GO:0070498|interleukin-1-mediated signaling pathway;GO:0035556|intracellular signal transduction;GO:0043123|positive regulation of I-kappaB kinase/NF-kappaB signaling;GO:0043406|positive regulation of MAP kinase activity;GO:0051092|positive regulation of NF-kappaB transcription factor activity;GO:0034142|toll-like receptor 4 signaling pathway;	GO:0005737|cytoplasm;GO:0005811|lipid particle;GO:0005634|nucleus;	GO:0005524|ATP binding;GO:0004674|protein serine/threonine	K04730	IRAK1; interleukin-1 receptor-associated kinase 1 [EC:2.7.11.1]
TRIM2	1855.647	-0.35371	0.107338757	-3.2953	0.0009832	0.011767	Not_Sig	no	gi|440899161|gb|ELR50510.1|	4094	0	Tripartite motif-containing protein 2, partial [Bos mutus]	gi|982949563|ref|XM_010813659.2|	6361	0	PREDICTED: Bos taurus tripartite motif containing 2 (TRIM2), transcript variant X3, mRNA	A4IF63	1543	0	Tripartite motif-containing protein 2	.	.	.	.	pfam00630	Filamin	Filamin/ABP280 repeat.	GO:0043523|regulation of neuron apoptotic process;	GO:0005737|cytoplasm;	GO:0016874|ligase activity;GO:0004842|ubiquitin-protein transferase	K11997	TRIM2_3; tripartite motif-containing protein 2/3
COX6B1	927.0752	-0.43276	0.131366519	-3.29428	0.0009867	0.011798	Not_Sig	no	gi|743709903|ref|XP_010948401.1|	574	1e-72	PREDICTED: cytochrome c oxidase subunit 6B1 [Camelus bactrianus]	gi|742161291|ref|XM_010849019.1|	547	0	PREDICTED: Bison bison bison cytochrome c oxidase subunit 6B1 (LOC104995249), mRNA	P00429	184	1e-58	Cytochrome c oxidase subunit 6B1	.	.	.	.	pfam02297	COX6B	Cytochrome oxidase c subunit VIb. Cytochrome c oxidase, a 13 sub-unit complex, EC:1.9.3.1 is the terminal oxidase in the	.	GO:0005743|mitochondrial inner membrane;GO:0005758|mitochondrial intermembrane space;GO:0005739|mitochondrion;GO:0045	GO:0004129|cytochrome-c oxidase activity;	K02267	COX6B; cytochrome c oxidase subunit 6b
P2RY2	429.7184	0.502916	0.152735265	3.292729	0.0009922	0.011851	Not_Sig	no	gi|982946310|ref|XP_015330243.1|	1721	0	PREDICTED: P2Y purinoceptor 2 isoform X1 [Bos taurus]	gi|742162981|ref|XM_010849507.1|	2232	0	PREDICTED: Bison bison bison purinergic receptor P2Y, G-protein coupled, 2 (P2RY2), transcript variant X3, mRNA	P41231	568	0	P2Y purinoceptor 2	.	.	.	.	pfam00001	7tm_1	7 transmembrane receptor (rhodopsin family). This family contains, amongst other G-protein-coupled receptors	GO:0006873|cellular ion homeostasis;GO:0007200|phospholipase C-activating G-protein coupled receptor signaling pathway;GO:0070257|positive regulation of mucus secretion;GO:0042312|regulation of vasodilation;	GO:0005887|integral component of plasma membrane;GO:0005886|plasma membrane;	GO:0045028|G-protein coupled purinergic nucleotide receptor	K04269	P2RY2; purinergic receptor P2Y, G protein-coupled, 2
CDC42BPG	357.8043	0.461145	0.140131285	3.290807	0.000999	0.011914	Not_Sig	no	gi|741980134|ref|XP_010819388.1|	6697	0	PREDICTED: serine/threonine-protein kinase MRCK gamma isoform X5 [Bos taurus]	gi|982971947|ref|XM_010821086.2|	5789	0	PREDICTED: Bos taurus CDC42 binding protein kinase gamma (CDC42BPG), transcript variant X5, mRNA	Q6DT37	2276	0	Serine/threonine-protein kinase MRCK gamma	.	.	.	.	pfam00069	Pkinase	Protein kinase domain.	GO:0031532|actin cytoskeleton reorganization;GO:0035556|intracellular signal transduction;GO:0006468|protein phosphorylation;	GO:0031252|cell leading edge;GO:0005737|cytoplasm;	GO:0005524|ATP binding;GO:0000287|magnesium ion binding;GO:0004674	K16307	CDC42BP; serine/threonine-protein kinase MRCK [EC:2.7.11.1]
GTF3C1	1123.098	0.340146	0.103381623	3.290195	0.0010012	0.011914	Not_Sig	no	gi|300794773|ref|NP_001180170.1|	10462	0	general transcription factor 3C polypeptide 1 [Bos taurus]	gi|742106895|ref|XM_010837240.1|	7037	0	PREDICTED: Bison bison bison general transcription factor IIIC, polypeptide 1, alpha 220kDa (GTF3C1), mRNA	Q12789	3191	0	General transcription factor 3C polypeptide 1	.	.	.	.	pfam04182	B-block_TFIIIC	B-block binding subunit of TFIIIC. Yeast transcription factor IIIC (TFIIIC) is a multi-subunit protein complex that interacts with two	GO:0042791|5S class rRNA transcription from RNA polymerase III type 1 promoter;GO:0009303|rRNA transcription;GO:0006383|transcription from RNA polymerase III promoter;GO:0006351|transcription, DNA-templated;GO:0009304|tRNA transcription;GO:0042797|tRNA transcription from RNA polymerase III promoter;	GO:0030529|ribonucleoprotein complex;GO:0016020|membrane;GO:0005730|nucleolus;GO:0005654|nucleoplasm;GO:0005634|nucleus;GO:0000127|transcription	GO:0003677|DNA binding;	K15199	GTF3C1; general transcription factor 3C polypeptide 1
CMAS	1068.632	-0.42535	0.129282333	-3.29008	0.0010016	0.011914	Not_Sig	no	gi|78369508|ref|NP_001030475.1|	2242	0	N-acylneuraminate cytidylyltransferase [Bos taurus]	gi|942047877|ref|XM_005893235.2|	2035	0	PREDICTED: Bos mutus cytidine monophosphate N-acetylneuraminic acid synthetase (CMAS), mRNA	Q3SZM5	844	0	N-acylneuraminate cytidylyltransferase	MA3766_1	152	2.41e-41	COG1083	pfam02348	CTP_transf_3	Cytidylyltransferase. This family consists of two main Cytidylyltransferase activities: 1) 3-deoxy-manno-octulosonate	GO:0006054|N-acetylneuraminate metabolic process;	GO:0005829|cytosol;GO:0005634|nucleus;	GO:0090633|keto-deoxynonulosonic acid (KDN) cytidylyltransferase	K00983	E2.7.7.43, neuA, CMAS; N-acylneuraminate cytidylyltransferase
SLC35F5	1191.536	-0.37087	0.112722916	-3.29006	0.0010016	0.011914	Not_Sig	no	gi|155372203|ref|NP_001094712.1|	2438	0	solute carrier family 35 member F5 [Bos taurus]	gi|742177605|ref|XM_010853615.1|	2684	0	PREDICTED: Bison bison bison solute carrier family 35, member F5 (SLC35F5), mRNA	A6QL92	943	0	Solute carrier family 35 member F5	YML018c	113	4.78e-26	COG0697	pfam06027	DUF914	Eukaryotic protein of unknown function (DUF914). This family consists of several hypothetical proteins of unknown function.	GO:0006810|transport;	GO:0016021|integral component of membrane;	.	K15289	SLC35F5; solute carrier family 35, member F5
STARD4	532.9369	-0.46624	0.141775727	-3.28858	0.001007	0.011965	Not_Sig	no	gi|555949594|ref|XP_005887006.1|	1120	7e-138	PREDICTED: stAR-related lipid transfer protein 4 [Bos mutus]	gi|742151131|ref|XM_010846137.1|	1852	0	PREDICTED: Bison bison bison StAR-related lipid transfer (START) domain containing 4 (STARD4), transcript variant X1, mRNA	Q96DR4	393	3e-124	StAR-related lipid transfer protein 4	.	.	.	.	pfam01852	START	START domain.	GO:0070508|cholesterol import;GO:0010879|cholesterol transport involved in cholesterol storage;GO:0032367|intracellular cholesterol transport;GO:0070859|positive regulation of bile acid biosynthetic process;GO:0010873|positive regulation of cholesterol esterification;	GO:0016023|cytoplasmic membrane-bounded vesicle;GO:0005739|mitochondrion;	GO:0015485|cholesterol binding;GO:0017127|cholesterol	.	.
DAG1	3936.062	0.37872	0.115181338	3.288031	0.0010089	0.011976	Not_Sig	no	gi|594058317|ref|XP_006053323.1|	3976	0	PREDICTED: dystroglycan isoform X2 [Bubalus bubalis]	gi|982961760|ref|XM_010817663.2|	5370	0	PREDICTED: Bos taurus dystroglycan 1 (DAG1), transcript variant X1, mRNA	O18738	1493	0	Dystroglycan	.	.	.	.	pfam05454	DAG1	Dystroglycan (Dystrophin-associated glycoprotein 1). Dystroglycan is one of the dystrophin-associated	GO:0007016|cytoskeletal anchoring at plasma membrane;	GO:0030054|cell junction;GO:0005737|cytoplasm;GO:0005856|cytoskeleton;GO:0016010|dystrophin-associated glycoprotein	GO:0005509|calcium ion binding;GO:0043236|laminin binding;	K06265	DAG1; dystroglycan 1
ANKRD52	1614.593	0.373153	0.113512288	3.287332	0.0010114	0.011987	Not_Sig	no	gi|594104204|ref|XP_006075093.1|	5689	0	PREDICTED: serine/threonine-protein phosphatase 6 regulatory ankyrin repeat subunit C isoform X1 [Bubalus bubalis]	gi|555983241|ref|XM_005903460.1|	8525	0	PREDICTED: Bos mutus ankyrin repeat domain 52 (ANKRD52), mRNA	Q8NB46	2185	0	Serine/threonine-protein phosphatase 6 regulatory ankyrin repeat subunit C	all2748	177	8.18e-47	COG0666	pfam12796	Ank_2	Ankyrin repeats (3 copies).	.	.	.	K15504	ANKRD52; serine/threonine-protein phosphatase 6 regulatory ankyrin
SKI	1020.628	0.42431	0.129080015	3.287186	0.0010119	0.011987	Not_Sig	no	gi|965847093|ref|XP_014963078.1|	2765	0	PREDICTED: ski oncogene isoform X2 [Ovis aries musimon]	gi|982996539|ref|XM_015466808.1|	4469	0	PREDICTED: Bos taurus SKI proto-oncogene (SKI), mRNA	Q9TUG2	1028	0	Ski oncogene	.	.	.	.	pfam08782	c-SKI_SMAD_bind	c-SKI Smad4 binding domain. c-SKI is an oncoprotein that inhibits TGF-beta signaling through interaction with Smad proteins. This domain	GO:0032926|negative regulation of activin receptor signaling pathway;GO:0030514|negative regulation of BMP signaling pathway;GO:0045596|negative regulation of cell differentiation;GO:0008285|negative regulation of cell proliferation;GO:0045668|negative regulation of osteoblast differentiation;GO:0000122|negative regulation of transcription from RNA polymerase II promoter;GO:0030512|negative regulation of transforming growth factor beta receptor signaling pathway;GO:0048666|neuron	GO:0005737|cytoplasm;GO:0005634|nucleus;GO:0017053|transcriptional repressor complex;	GO:0003682|chromatin binding;GO:0019901|protein kinase	.	.
MRPS18C	141.292	-0.80189	0.244009206	-3.2863	0.0010151	0.012012	DOWN	yes	gi|114050735|ref|NP_001039973.1|	760	1e-100	28S ribosomal protein S18c, mitochondrial [Bos taurus]	gi|942087081|ref|XM_005906495.2|	519	0	PREDICTED: Bos mutus mitochondrial ribosomal protein S18C (MRPS18C), transcript variant X1, mRNA	P82917	297	3e-102	28S ribosomal protein S18c, mitochondrial	SP1539	56.6	1.40e-10	COG0238	pfam01084	Ribosomal_S18	Ribosomal protein S18.	GO:0032543|mitochondrial translation;GO:0070125|mitochondrial translational elongation;GO:0070124|mitochondrial translational initiation;	GO:0022627|cytosolic small ribosomal subunit;GO:0005743|mitochondrial inner membrane;GO:0005763|mitochondrial small ribosomal subunit;	GO:0003735|structural constituent of ribosome;	K02963	RP-S18, MRPS18, rpsR; small subunit ribosomal protein S18
SLC35A4	539.7982	0.579338	0.176304907	3.286003	0.0010162	0.012012	Not_Sig	no	gi|118601818|ref|NP_001073080.1|	956	5e-121	probable UDP-sugar transporter protein SLC35A4 [Bos taurus]	gi|942067789|ref|XM_014479589.1|	1865	0	PREDICTED: Bos mutus solute carrier family 35, member A4 (SLC35A4), transcript variant X2, mRNA	Q05B73	372	2e-122	Probable UDP-sugar transporter protein SLC35A4	.	.	.	.	pfam04142	Nuc_sug_transp	Nucleotide-sugar transporter. This family of membrane proteins transport nucleotide sugars from the cytoplasm into golgi vesicles.	.	GO:0000139|Golgi membrane;GO:0016021|integral component of membrane;	GO:0005351|sugar:proton symporter activity;	K15273	SLC35A4; solute carrier family 35 (probable UDP-sugar transporter),
SLX4	346.6392	0.507841	0.154614876	3.284557	0.0010214	0.012062	Not_Sig	no	gi|555978905|ref|XP_005901388.1|	7730	0	PREDICTED: structure-specific endonuclease subunit SLX4 [Bos mutus]	gi|983006913|ref|XM_005193005.3|	6534	0	PREDICTED: Bos taurus SLX4 structure-specific endonuclease subunit (SLX4), transcript variant X2, mRNA	Q8IY92	1467	0	Structure-specific endonuclease subunit SLX4	.	.	.	.	pfam09494	Slx4	Slx4 endonuclease. The Slx4 protein is a heteromeric structure-specific endonuclease found from fungi to mammals. Slx4 with Slx1	GO:0010792|DNA double-strand break processing involved in repair via single-strand annealing;GO:0006281|DNA repair;GO:0006260|DNA replication;GO:0000724|double-strand break repair via homologous recombination;GO:0036297|interstrand cross-link repair;GO:0000706|meiotic DNA double-strand break processing;GO:0006289|nucleotide-excision repair;GO:1904431|positive regulation of t-circle formation;GO:0000712|resolution of meiotic recombination intermediates;GO:0072429|response to intra-S DNA damage checkpoint	GO:0030054|cell junction;GO:0005737|cytoplasm;GO:0005654|nucleoplasm;GO:0033557|Slx1-Slx4 complex;	GO:0017108|5'-flap endonuclease activity;GO:0004520|endodeoxyribonucl	K10484	BTBD12, SLX4; structure-specific endonuclease subunit SLX4
CCND3	1375.3	0.484823	0.147631916	3.284001	0.0010234	0.012073	Not_Sig	no	gi|187937002|ref|NP_001120761.1|	1308	4e-160	G1/S-specific cyclin-D3 [Ovis aries]	gi|850492819|gb|CP011905.1|	4861	0	Ovis canadensis canadensis isolate 43U chromosome 20 sequence	Q3MHH5	506	8e-161	G1/S-specific cyclin-D3	.	.	.	.	pfam00134	Cyclin_N	Cyclin, N-terminal domain. Cyclins regulate cyclin dependent kinases (CDKs). Human cyclin-O is a Uracil-DNA glycosylase that is related to	GO:0007049|cell cycle;GO:0051301|cell division;	GO:0005737|cytoplasm;GO:0016020|membrane;GO:0005634|nucleus;	.	K10152	CCND3; cyclin D3

DOCK6	586.1121	0.474469	0.144496448	3.283602	0.0010249	0.012074	Not_Sig	no	gi|803263610|ref|XP_011991509.1|	10178	0	PREDICTED: dedicator of cytokinesis protein 6 isoform X3 [Ovis aries musimon]	gi|965842003|ref|XM_012136119.2|	6092	0	PREDICTED: Ovis aries musimon dedicator of cytokinesis 6 (DOCK6), transcript variant X3, mRNA	Q8VDR9	3600	0	Dedicator of cytokinesis protein 6	.	.	.	.	pfam06920	Ded_cyto	Dedicator of cytokinesis. This family represents a conserved region approximately 200 residues long within a number of eukaryotic	GO:0007264|small GTPase mediated signal transduction;	GO:0005737|cytoplasm;GO:0048471|perinuclear region of cytoplasm;	GO:0005085|guanyl-nucleotide exchange factor activity;	.	.
TMEM190	961.0643	-0.41042	0.12499811	-3.2834	0.0010256	0.012074	Not_Sig	no	gi|329663270|ref|NP_001192488.1|	843	6e-109	transmembrane protein 190 precursor [Bos taurus]	gi|965904323|ref|XM_012163642.2|	1030	0	PREDICTED: Ovis aries musimon transmembrane protein 190 (TMEM190), transcript variant X2, mRNA	E1BJD3	329	2e-110	Transmembrane protein 190	.	.	.	.	pfam15431	TMEM190	Transmembrane protein 190.	GO:0002244|hematopoietic progenitor cell differentiation;	GO:0002079|inner acrosomal membrane;GO:0016021|integral component of membrane;GO:0005634|nucleus;	.	.	.
SLC38A9	333.0826	-0.42673	0.130030975	-3.28176	0.0010316	0.012108	Not_Sig	no	gi|742177227|ref|XP_010851814.1|	2846	0	PREDICTED: putative sodium-coupled neutral amino acid transporter 9 isoform X1 [Bison bison bison]	gi|965839533|ref|XM_012135048.2|	4371	0	PREDICTED: Ovis aries musimon solute carrier family 38 member 9 (SLC38A9), transcript variant X1, mRNA	Q8NBW4	991	0	Sodium-coupled neutral amino acid transporter 9	.	.	.	.	pfam01490	Aa_trans	Transmembrane amino acid transporter protein. This transmembrane region is found in many amino acid transporters	GO:0003333|amino acid transmembrane transport;GO:0071230|cellular response to amino acid stimulus;GO:0032008|positive regulation of TOR signaling;	GO:0005783|endoplasmic reticulum;GO:0016021|integral component of membrane;GO:0005770|late endosome;GO:0031902|late endosome	GO:0015171|amino acid transmembrane transporter activity;	K14995	SLC38A9; solute carrier family 38 (sodium-coupled neutral amino acid
SPTSSA	502.1417	-0.4934	0.150348221	-3.28173	0.0010317	0.012108	Not_Sig	no	gi|940726681|ref|XP_014308860.1|	418	2e-46	PREDICTED: serine palmitoyltransferase small subunit A [Myotis lucifugus]	gi|942099792|ref|XM_005910627.2|	1257	0	PREDICTED: Bos mutus serine palmitoyltransferase, small subunit A (SPTSSA), mRNA	Q969W0	143	5e-40	Serine palmitoyltransferase small subunit A	.	.	.	.	pfam11779	DUF3317	Protein of unknown function (DUF3317). This is a short family of proteins conserved from fungi and plants to human. One each of the	GO:0046513|ceramide biosynthetic process;GO:0030148|sphingolipid biosynthetic process;	GO:0016021|integral component of membrane;GO:0017059|serine C-palmitoyltransferase complex;	GO:0004758|serine C-palmitoyltransferase activity;	.	.
RTRAF	1520.653	-0.46284	0.141035813	-3.28171	0.0010318	0.012108	Not_Sig	no	gi|78369460|ref|NP_001030357.1|	1138	6e-154	UPF0568 protein C14orf166 homolog [Bos taurus]	gi|942051312|ref|XM_005894544.2|	1003	0	PREDICTED: Bos mutus chromosome unknown open reading frame, human C14orf166 (LOC102265885), transcript variant X1, mRNA	Q5R8I2	434	4e-152	UPF0568 protein C14orf166 homolog	.	.	.	.	pfam10036	RLL	Putative carnitine deficiency-associated protein. This family of proteins conserved from nematodes to humans is of	GO:0045944|positive regulation of transcription from RNA polymerase II promoter;GO:0006351|transcription, DNA-templated;GO:0006388|tRNA splicing, via endonucleolytic cleavage and ligation;	GO:0005737|cytoplasm;GO:0005829|cytosol;GO:0005815|microtubule organizing center;GO:0005654|nucleoplasm;GO:0005634|nucleus;GO:0048471|perinuclear region	GO:0044822|poly(A) RNA binding;GO:0003723|RNA	K15433	CGI99, CLE7, RLLM1; RLL motif containing protein 1
ACTN1	23977.15	0.345193	0.105200771	3.281274	0.0010334	0.012108	Not_Sig	no	gi|528966774|ref|XP_005212035.1|	4785	0	PREDICTED: alpha-actinin-1 isoform X1 [Bos taurus]	gi|942084680|ref|XM_005905735.2|	3530	0	PREDICTED: Bos mutus actinin, alpha 1 (ACTN1), transcript variant X3, mRNA	Q3B7N2	1816	0	Alpha-actinin-1	SPAC15A10.08	271	5.92e-78	COG5069	pfam00307	CH	Calponin homology (CH) domain. The CH domain is found in both cytoskeletal proteins and signal transduction proteins. The CH	GO:0051764|actin crosslink formation;GO:0051017|actin filament bundle assembly;GO:0051639|actin filament network formation;GO:0051234|establishment of localization;GO:0048041|focal adhesion assembly;GO:0051271|negative regulation of cellular component movement;GO:0030220|platelet formation;GO:0043462|regulation of ATPase activity;GO:0046928|regulation of neurotransmitter secretion;GO:0045214|sarcomere organization;GO:0048741|skeletal muscle fiber development;	GO:0005884|actin filament;GO:0005923|bicellular tight junction;GO:0005903|brush border;GO:0031252|cell leading	GO:0051015|actin filament binding;GO:0051393|alpha-actinin	K05699	ACTN; actinin alpha
MAPK8	334.2779	-0.43159	0.13153494	-3.28118	0.0010337	0.012108	Not_Sig	no	gi|555977457|ref|XP_005900682.1|	2210	0	PREDICTED: mitogen-activated protein kinase 8 isoform X1 [Bos mutus]	gi|742180460|ref|XM_010854408.1|	2290	0	PREDICTED: Bison bison bison mitogen-activated protein kinase 8 (MAPK8), transcript variant X1, mRNA	P45983	832	0	Mitogen-activated protein kinase 8	.	.	.	.	pfam00069	Pkinase	Protein kinase domain.	GO:0071222|cellular response to lipopolysaccharide;GO:0071260|cellular response to mechanical stimulus;GO:0038095|Fc-epsilon receptor signaling pathway;GO:0007254|JNK cascade;GO:0007258|JUN phosphorylation;GO:0043066|negative regulation of apoptotic process;GO:0032091|negative regulation of protein binding;GO:0018105|peptidyl-serine phosphorylation;GO:0018107|peptidyl-threonine phosphorylation;GO:0043065|positive regulation of apoptotic process;GO:0031281|positive regulation of cyclase	GO:0005829|cytosol;GO:0005739|mitochondrion;GO:0005654|nucleoplasm;GO:0005634|nucleus;	GO:0005524|ATP binding;GO:0019899|enzyme binding;GO:0042826	K04440	JNK; c-Jun N-terminal kinase [EC:2.7.11.24]
LMF2	1048.25	0.485635	0.148064852	3.279877	0.0010385	0.012151	Not_Sig	no	gi|172044109|sp|A1L504.2|LMF2_BOVIN	3131	0	RecName: Full=Lipase maturation factor 2	gi|119936027|gb|BT029791.1|	2558	0	Bos taurus hypothetical protein BC002942 (BC002942), mRNA, complete cds	A1L504	1210	0	Lipase maturation factor 2	.	.	.	.	pfam06762	LMF1	Lipase maturation factor. This family of transmembrane proteins includes the lipase maturation factor, LMF1. Lipoprotein lipase	GO:0051604|protein maturation;	GO:0005789|endoplasmic reticulum membrane;GO:0016021|integral component of membrane;	.	.	.
SERF2	1869.531	-0.36484	0.111258137	-3.27926	0.0010408	0.012166	Not_Sig	no	gi|958717786|ref|XP_014702585.1|	689	6e-87	PREDICTED: small EDRK-rich factor 2 isoform X2 [Equus asinus]	gi|982960739|ref|XM_005222207.3|	586	0	PREDICTED: Bos taurus small EDRK-rich factor 2 (SERF2), transcript variant X1, mRNA	Q5R7C4	78.2	2e-17	Small EDRK-rich factor 2	.	.	.	.	pfam04419	4F5	4F5 protein family. Members of this family are short proteins that are rich in aspartate, glutamate, lysine and arginine. Although the	.	GO:0005829|cytosol;GO:0005634|nucleus;	.	.	.
HGS	1240.617	0.390993	0.119244942	3.278904	0.0010421	0.012169	Not_Sig	no	gi|528996842|ref|XP_005221090.1|	3328	0	PREDICTED: hepatocyte growth factor-regulated tyrosine kinase substrate isoform X1 [Bos taurus]	gi|110331762|gb|BT026148.1|	2937	0	Bos taurus hepatocyte growth factor-regulated tyrosine kinase substrate (HGS), mRNA, complete cds	Q0V8S0	1286	0	Hepatocyte growth factor-regulated tyrosine kinase substrate	.	.	.	.	pfam00790	VHS	VHS domain. Domain present in VPS-27, Hrs and STAM.	GO:0010324|membrane invagination;GO:0046426|negative regulation of JAK-STAT cascade;GO:1903543|positive regulation of exosomal secretion;GO:0010628|positive regulation of gene expression;GO:0072657|protein localization to membrane;GO:0006622|protein targeting to lysosome;GO:0043405|regulation of MAP kinase activity;	GO:0031901|early endosome membrane;GO:0070062|extracellular exosome;GO:0032585|multivesicular body membrane;	GO:0046872|metal ion binding;	K12182	HGS, HRS, VPS27; hepatocyte growth factor-regulated tyrosine kinase
USP53	718.0796	-0.38528	0.117538126	-3.27795	0.0010456	0.012197	Not_Sig	no	gi|555972243|ref|XP_005898128.1|	5220	0	PREDICTED: inactive ubiquitin carboxyl-terminal hydrolase 53 [Bos mutus]	gi|942060453|ref|XM_005898066.2|	3031	0	PREDICTED: Bos mutus ubiquitin specific peptidase 53 (USP53), mRNA	Q70EK8	1636	0	Inactive ubiquitin carboxyl-terminal hydrolase 53	.	.	.	.	pfam00443	UCH	Ubiquitin carboxyl-terminal hydrolase.	GO:0016579|protein deubiquitination;GO:0007605|sensory perception of sound;	.	GO:0036459|ubiquitinyl hydrolase activity;	.	.
MAST3	263.2774	0.535553	0.163431788	3.276919	0.0010495	0.01223	Not_Sig	no	gi|528906579|ref|XP_005198857.1|	5339	0	PREDICTED: microtubule-associated serine/threonine-protein kinase 3 isoform X5 [Bos taurus]	gi|982984063|ref|XM_005198800.3|	5635	0	PREDICTED: Bos taurus microtubule associated serine/threonine kinase 3 (MAST3), transcript variant X5, mRNA	O60307	1816	0	Microtubule-associated serine/threonine-protein kinase 3	.	.	.	.	pfam08926	DUF1908	Domain of unknown function (DUF1908). This domain is found in a set of hypothetical/structural eukaryotic proteins.	GO:0035556|intracellular signal transduction;GO:0018105|peptidyl-serine phosphorylation;	GO:0005622|intracellular;	GO:0005524|ATP binding;GO:0000287|magnesium ion binding;GO:0004674	K08789	MAST; microtubule-associated serine/threonine kinase [EC:2.7.11.1]
ORC1	139.8264	0.595067	0.181678219	3.275387	0.0010552	0.012269	UP	yes	gi|982919714|ref|XP_015320897.1|	4348	0	PREDICTED: origin recognition complex subunit 1 isoform X1 [Bos taurus]	gi|742154832|ref|XM_010847179.1|	2985	0	PREDICTED: Bison bison bison origin recognition complex, subunit 1 (ORC1), transcript variant X1, mRNA	Q58DC8	1627	0	Origin recognition complex subunit 1	SPBC29A10.15	263	1.40e-74	COG1474	pfam01426	BAH	BAH domain. This domain has been called BAH (Bromo adjacent homology) domain and has also been called ELM1 and BAM	GO:0006260|DNA replication;	GO:0005664|nuclear origin of replication recognition complex;	GO:0005524|ATP binding;GO:0003682|chromatin binding;GO:0003677	K02603	ORC1; origin recognition complex subunit 1
LOC106700705	695.3322	-0.43789	0.13369846	-3.27518	0.001056	0.012269	Not_Sig	no	.	.	.	.	gi|982982928|ref|XR_138729.3|	597	0	PREDICTED: Bos taurus uncharacterized LOC100847143 (LOC100847143), ncRNA	.	.	.	.	.	.	.	.	pfam09797	NatB_MDM20	N-acetyltransferase B complex (NatB) non catalytic subunit. This is the non-catalytic subunit of the N-terminal acetyltransferase B	.	.	.	.	.
ARHGEF10L	1221.249	0.376712	0.115021228	3.275152	0.0010561	0.012269	Not_Sig	no	gi|555986520|ref|XP_005905120.1|	5803	0	PREDICTED: rho guanine nucleotide exchange factor 10-like protein [Bos mutus]	gi|594086233|ref|XM_006066567.1|	3920	0	PREDICTED: Bubalus bubalis Rho guanine nucleotide exchange factor (GEF) 10-like (ARHGEF10L), transcript variant X4, mRNA	Q29RM4	2231	0	Rho guanine nucleotide exchange factor 10-like protein	.	.	.	.	pfam00621	RhoGEF	RhoGEF domain. Guanine nucleotide exchange factor for Rho/Rac/Cdc42-like GTPases Also called Dbl-homologous (DH)	GO:0035023|regulation of Rho protein signal transduction;	GO:0005737|cytoplasm;	GO:0005089|Rho guanyl-nucleotide exchange factor activity;	K16727	ARHGEF10; Rho guanine nucleotide exchange factor 10
BUB1	618.3213	0.397871	0.121559123	3.273067	0.0010639	0.012327	Not_Sig	no	gi|942042983|ref|XP_014332442.1|	5860	0	PREDICTED: LOW QUALITY PROTEIN: mitotic checkpoint serine/threonine-protein kinase BUB1 [Bos mutus]	gi|982938143|ref|XM_005212316.2|	3678	0	PREDICTED: Bos taurus BUB1 mitotic checkpoint serine/threonine kinase (BUB1), transcript variant X1, mRNA	O43683	1778	0	Mitotic checkpoint serine/threonine-protein kinase BUB1	.	.	.	.	pfam08311	Mad3_BUB1_I	Mad3/BUB1 homology region 1. Proteins containing this domain are checkpoint proteins involved in cell division. This region has been	GO:0006915|apoptotic process;GO:0051301|cell division;GO:0008283|cell proliferation;GO:0007093|mitotic cell cycle checkpoint;GO:0007067|mitotic nuclear division;GO:0007094|mitotic spindle assembly checkpoint;GO:0051983|regulation of chromosome segregation;GO:0007063|regulation of sister chromatid cohesion;GO:0007062|sister chromatid cohesion;GO:0071173|spindle assembly checkpoint;GO:0016032|viral process;	GO:0000777|condensed chromosome kinetochore;GO:0000942|condensed nuclear chromosome outer kinetochore;GO:0005737|cytoplasm;GO:00	GO:0005524|ATP binding;GO:0004672|protein kinase activity;GO:0004674	K02178	BUB1; checkpoint serine/threonine-protein kinase [EC:2.7.11.1]
SRI	314.1869	-0.49994	0.152746364	-3.27301	0.0010641	0.012327	Not_Sig	no	gi|555978952|ref|XP_005901411.1|	907	4e-120	PREDICTED: sorcin isoform X1 [Bos mutus]	gi|942071156|ref|XM_005901349.2|	878	0	PREDICTED: Bos mutus sorcin (SRI), transcript variant X1, mRNA	Q5R4U9	351	1e-120	Sorcin	.	.	.	.	pfam13833	EF-hand_8	EF-hand domain pair.	GO:0006816|calcium ion transport;GO:0042994|cytoplasmic sequestering of transcription factor;GO:0010459|negative regulation of heart rate;GO:0060315|negative regulation of ryanodine-sensitive calcium-release channel activity;GO:2000678|negative regulation of transcription regulatory region DNA binding;GO:0035774|positive regulation of insulin secretion involved in cellular response to glucose stimulus;GO:0051281|positive regulation of release of sequestered calcium ion into cytosol;GO:0086004|regulation of cardiac muscle cell	GO:0042584|chromaffin granule membrane;GO:0005829|cytosol;GO:0070062|extracellular exosome;GO:0005654|nucleoplasm;GO:000	GO:0005509|calcium ion binding;	.	.
BTG1	4394.502	-0.29483	0.090079805	-3.27296	0.0010643	0.012327	Not_Sig	no	gi|296212508|ref|XP_002752860.1|	951	1e-121	PREDICTED: protein BTG1 [Callithrix jacchus]	gi|742117264|ref|XM_010839710.1|	1771	0	PREDICTED: Bison bison bison B-cell translocation gene 1, anti-proliferative (BTG1), mRNA	P53348	358	2e-119	Protein BTG1	.	.	.	.	pfam07742	BTG	BTG family.	GO:0008285|negative regulation of cell proliferation;GO:0045766|positive regulation of angiogenesis;GO:0045603|positive regulation of endothelial cell differentiation;GO:2000271|positive regulation of fibroblast apoptotic process;GO:0045663|positive regulation of myoblast differentiation;	GO:0005737|cytoplasm;GO:0005634|nucleus;	GO:0019899|enzyme binding;	K14443	TOB; protein Tob/BTG
IQCE	398.4674	0.49836	0.15235348	3.271075	0.0010714	0.012397	Not_Sig	no	gi|529007939|ref|XP_005225353.1|	2896	0	PREDICTED: IQ domain-containing protein E isoform X7 [Bos taurus]	gi|983007508|ref|XM_005197024.3|	4781	0	PREDICTED: Bos taurus IQ motif containing E (IQCE), transcript variant X7, mRNA	Q6PCQ0	708	0	IQ domain-containing protein E	.	.	.	.	pfam07888	CALCOCO1	Calcium binding and coiled-coil domain (CALCOCO1) like. Proteins found in this family are similar to the coiled-coil transcriptional	.	GO:0005739|mitochondrion;	.	.	.
LOC102281772	221.2887	-0.6092	0.186259847	-3.27069	0.0010729	0.0124	DOWN	yes	gi|927111499|ref|XP_003125182.2|	873	1e-108	PREDICTED: prolyl-tRNA synthetase associated domain-containing protein 1 [Sus scrofa]	gi|594091103|ref|XM_006068879.1|	1613	0	PREDICTED: Bubalus bubalis prolyl-tRNA synthetase associated domain-containing protein 1-like (LOC102391491), mRNA	A1A4Q2	308	4e-99	Prolyl-tRNA synthetase associated domain-containing protein 1	CC0111	81.3	4.08e-17	COG3760	pfam04073	tRNA_edit	Aminoacyl-tRNA editing domain. This domain is found either on its own or in association with the tRNA synthetase class II core	.	.	GO:0002161|aminoacyl-tRNA editing activity;	.	.
HMGN4	359.6445	-0.46431	0.141970804	-3.27045	0.0010738	0.0124	Not_Sig	no	gi|829939105|ref|XP_012597552.1|	213	1e-16	PREDICTED: high mobility group nucleosome-binding domain-containing protein 4 isoform X1 [Microcebus murinus]	gi|114053062|ref|NM_001046547.1|	2293	0	Bos taurus high mobility group nucleosomal binding domain 4 (HMGN4), mRNA	Q2YDK4	77.4	3e-15	High mobility group nucleosome-binding domain-containing protein 4	.	.	.	.	pfam01101	HMG14_17	HMG14 and HMG17.	.	GO:0000785|chromatin;GO:0005634|nucleus;	GO:0003677|DNA binding;	K11302	HMGN4; high-mobility group nucleosome-binding domain-containing
RELA	1659.879	0.475135	0.145303462	3.269953	0.0010757	0.012409	Not_Sig	no	gi|122692509|ref|NP_001073711.1|	2654	0	transcription factor p65 [Bos taurus]	gi|594065044|ref|XM_006056460.1|	2639	0	PREDICTED: Bubalus bubalis v-rel avian reticuloendotheliosis viral oncogene homolog A (RELA), mRNA	Q04207	919	0	Transcription factor p65	.	.	.	.	pfam00554	RHD	Rel homology domain (RHD). Proteins containing the Rel homology domain (RHD) are eukaryotic transcription factors.	GO:0006117|acetaldehyde metabolic process;GO:0007568|aging;GO:0009887|organ morphogenesis;GO:0035729|cellular response to hepatocyte growth factor stimulus;GO:0070301|cellular response to hydrogen peroxide;GO:0071347|cellular response to interleukin-1;GO:0071354|cellular response to interleukin-6;GO:0071222|cellular response to lipopolysaccharide;GO:0071316|cellular response to nicotine;GO:0071375|cellular response to peptide hormone stimulus;GO:0071356|cellular response to tumor necrosis	GO:0005737|cytoplasm;GO:0005829|cytosol;GO:0033256|I-kappaB/NF-kappaB complex;GO:0071159|NF-kappaB complex;GO:0035525|NF-kappaB p50/p65	GO:0042805|actinin binding;GO:0033613|activating transcription factor	K04735	RELA; transcription factor p65
AURKB	312.6889	0.480101	0.146840615	3.269535	0.0010772	0.012415	Not_Sig	no	gi|115749607|ref|NP_898907.2|	1835	0	aurora kinase B [Bos taurus]	gi|942052036|ref|XM_005894821.2|	2102	0	PREDICTED: Bos mutus aurora kinase B (AURKB), transcript variant X1, mRNA	Q7YRC6	709	0	Aurora kinase B	.	.	.	.	pfam00069	Pkinase	Protein kinase domain.	GO:0009838|abscission;GO:0034644|cellular response to UV;GO:0036089|cleavage furrow formation;GO:0043988|histone H3-S28 phosphorylation;GO:0051256|mitotic spindle midzone assembly;GO:0007052|mitotic spindle organization;GO:0002903|negative regulation of B cell apoptotic process;GO:0032466|negative regulation of cytokinesis;GO:0000122|negative regulation of transcription from RNA polymerase II promoter;GO:0032467|positive regulation of cytokinesis;GO:0034501|protein localization to kinetochore;GO:0006468|protein	GO:0005694|chromosome;GO:0032133|chromosome passenger complex;GO:0000775|chromosome, centromeric region;GO:0000780|condensed	GO:0005524|ATP binding;GO:0035174|histone serine kinase	.	.
TPPP3	54.35985	-0.85799	0.262498231	-3.26855	0.001081	0.012446	DOWN	yes	gi|594627117|ref|XP_007198171.1|	916	1e-120	PREDICTED: tubulin polymerization-promoting protein family member 3 isoform X1 [Balaenoptera acutorostrata scammoni]	gi|942056438|ref|XM_005896550.2|	1066	0	PREDICTED: Bos mutus tubulin polymerization-promoting protein family member 3 (TPPP3), transcript variant X2, mRNA	Q3ZCC8	354	3e-121	Tubulin polymerization-promoting protein family member 3	.	.	.	.	pfam05517	p25-alpha	p25-alpha. This family encodes a 25 kDa protein that is phosphorylated by a Ser/Thr-Pro kinase. It has been described as a	GO:0001578|microtubule bundle formation;GO:0046785|microtubule polymerization;	GO:0005737|cytoplasm;GO:0070062|extracellular exosome;GO:0005874|microtubule;	GO:0015631|tubulin binding;	.	.
HSF1	809.9027	0.445975	0.136465821	3.268032	0.001083	0.012456	Not_Sig	no	gi|741949335|ref|XP_010810127.1|	2731	0	PREDICTED: heat shock factor protein 1 isoform X1 [Bos taurus]	gi|982944043|ref|XM_010811825.2|	2136	0	PREDICTED: Bos taurus heat shock transcription factor 1 (HSF1), transcript variant X1, mRNA	Q08DJ8	899	0	Heat shock factor protein 1	YGL073w	140	7.22e-34	COG5169	pfam06546	Vert_HS_TF	Vertebrate heat shock transcription factor. This family represents the C-terminal region of vertebrate heat shock	GO:0034605|cellular response to heat;GO:0001892|embryonic placenta development;GO:0060136|embryonic process involved in female pregnancy;GO:0007143|female meiotic division;GO:0009299|mRNA transcription;GO:0008285|negative regulation of cell proliferation;GO:0032720|negative regulation of tumor necrosis factor production;GO:0040018|positive regulation of multicellular organism growth;GO:0045944|positive regulation of transcription from RNA polymerase II promoter;GO:0006468|protein	GO:0005737|cytoplasm;GO:0005654|nucleoplasm;GO:0045120|pronucleus;GO:0043234|protein complex;	GO:0003682|chromatin binding;GO:0000978|RNA polymerase II	K09414	HSF1; heat shock transcription factor 1
CORO1B	665.118	0.382874	0.117242114	3.265667	0.0010921	0.012542	Not_Sig	no	gi|555983444|ref|XP_005903621.1|	2613	0	PREDICTED: coronin-1B [Bos mutus]	gi|942077957|ref|XM_005903559.2|	1837	0	PREDICTED: Bos mutus coronin, actin binding protein, 1B (CORO1B), mRNA	Q5NVK4	931	0	Coronin-1B	.	.	.	.	pfam08954	DUF1900	Domain of unknown function (DUF1900). This domain is predominantly found in the structural protein coronin, and is	.	GO:0005737|cytoplasm;GO:0005856|cytoskeleton;	.	K13886	CORO1B_1C_6; coronin-1B/1C/6
PNPLA2	298.8331	0.687744	0.210621538	3.265306	0.0010935	0.012542	UP	yes	gi|114050731|ref|NP_001039470.1|	2136	0	patatin-like phospholipase domain-containing protein 2 [Bos taurus]	gi|982972288|ref|XM_005227323.3|	1956	0	PREDICTED: Bos taurus patatin like phospholipase domain containing 2 (PNPLA2), transcript variant X1, mRNA	Q2KI18	827	0	Patatin-like phospholipase domain-containing protein 2	.	.	.	.	pfam01734	Patatin	Patatin-like phospholipase. This family consists of various patatin glycoproteins from plants. The patatin protein accounts for up to	GO:0055088|lipid homeostasis;GO:0010891|negative regulation of sequestering of triglyceride;GO:0010898|positive regulation of triglyceride catabolic process;GO:0019433|triglyceride catabolic process;	GO:0005737|cytoplasm;GO:0016021|integral component of membrane;GO:0005811|lipid particle;GO:0016020|membrane;GO:00058	GO:0004806|triglyceride lipase activity;	K16816	PNPLA2, ATGL; patatin-like phospholipase domain-containing
SNX19	998.5773	0.372071	0.113948797	3.265243	0.0010937	0.012542	Not_Sig	no	gi|942032071|ref|XP_014333790.1|	4297	0	PREDICTED: sorting nexin-19 [Bos mutus]	gi|729042228|ref|NM_001302940.1|	4327	0	Bos taurus sorting nexin 19 (SNX19), mRNA	Q92543	1269	0	Sorting nexin-19	.	.	.	.	pfam02194	PXA	PXA domain. This domain is associated with PX domains pfam00787.	GO:0002062|chondrocyte differentiation;GO:1990502|dense core granule maturation;GO:0006887|exocytosis;GO:0030073|insulin secretion;	GO:0005737|cytoplasm;GO:0030659|cytoplasmic vesicle membrane;GO:0031901|early endosome membrane;	GO:0032266|phosphatidylinositol-3-phosphate binding;	K17930	SNX19; sorting nexin-19
GIT1	607.6229	0.603329	0.184812584	3.264543	0.0010964	0.01256	UP	yes	gi|803276743|ref|XP_011998052.1|	3810	0	PREDICTED: ARF GTPase-activating protein GIT1 isoform X4 [Ovis aries musimon]	gi|983001385|ref|XM_015467970.1|	3384	0	PREDICTED: Bos taurus G protein-coupled receptor kinase interacting ArfGAP 1 (GIT1), transcript variant X3, mRNA	Q68FF6	1448	0	ARF GTPase-activating protein GIT1	SPBC17G9.08c_2	63.5	2.17e-10	COG5347	pfam12205	GIT1_C	G protein-coupled receptor kinase-interacting protein 1 C term. This domain family is found in eukaryotes, and is approximately	GO:0060996|dendritic spine development;GO:0048013|ephrin receptor signaling pathway;GO:0001771|immunological synapse formation;GO:0032465|regulation of cytokinesis;	GO:0016235|aggresome;GO:0005737|cytoplasm;GO:0005829|cytosol;GO:0005925|focal adhesion;GO:0016020|membrane;	GO:0005096|GTPase activator activity;GO:0046872|metal ion	K05737	GIT1; G protein-coupled receptor kinase interactor 1
SF3B4	934.8574	0.436125	0.133620695	3.2639	0.0010989	0.012576	Not_Sig	no	gi|528759952|gb|EPY79611.1|	1087	6e-163	myotubularin-related protein 11-like protein [Camelus ferus]	gi|742113195|ref|XM_010838730.1|	1554	0	PREDICTED: Bison bison bison splicing factor 3b, subunit 4, 49kDa (SF3B4), mRNA	Q15427	456	6e-155	Splicing factor 3B subunit 4	.	.	.	.	pfam00076	RRM_1	RNA recognition motif. (a.k.a. RRM, RBD, or RNP domain). The RRM motif is probably diagnostic of an RNA binding protein. RRMs	GO:0006397|mRNA processing;GO:0000398|mRNA splicing, via spliceosome;GO:0048026|positive regulation of mRNA splicing, via spliceosome;GO:0008380|RNA splicing;GO:0000375|RNA splicing, via transesterification reactions;	GO:0005654|nucleoplasm;GO:0005681|spliceosomal complex;GO:0005689|U12-type spliceosomal complex;	GO:0000166|nucleotide binding;GO:0044822|poly(A) RNA	K12831	SF3B4, SAP49; splicing factor 3B subunit 4
SYPL1	1596.112	-0.49017	0.150267139	-3.26198	0.0011064	0.012649	Not_Sig	no	gi|528946465|ref|XP_005205430.1|	1261	7e-156	PREDICTED: synaptophysin-like protein 1 isoform X1 [Bos taurus]	gi|942071407|ref|XM_014480015.1|	2738	0	PREDICTED: Bos mutus synaptophysin-like 1 (SYPL1), mRNA	Q16563	405	4e-127	Synaptophysin-like protein 1	.	.	.	.	pfam01284	MARVEL	Membrane-associating domain. MARVEL domain-containing proteins are often found in lipid-associating proteins - such as	GO:0007268|synaptic transmission;	GO:0070062|extracellular exosome;GO:0016021|integral component of membrane;GO:0005887|integral component of plasma	GO:0005215|transporter activity;	.	.
CRABP2	430.508	-0.39109	0.119926805	-3.26107	0.0011099	0.01266	Not_Sig	no	gi|440903652|gb|ELR54289.1|	755	1e-97	Cellular retinoic acid-binding protein 2, partial [Bos mutus]	gi|942063125|ref|XM_005898744.2|	970	0	PREDICTED: Bos mutus cellular retinoic acid binding protein 2 (CRABP2), mRNA	Q5PXY7	283	2e-94	Cellular retinoic acid-binding protein 2	.	.	.	.	pfam00061	Lipocalin	Lipocalin / cytosolic fatty-acid binding protein family. Lipocalins are transporters for small hydrophobic molecules, such as	GO:0048385|regulation of retinoic acid receptor signaling pathway;	GO:0005737|cytoplasm;GO:0005783|endoplasmic reticulum;GO:0005634|nucleus;	GO:0016918|retinal binding;GO:0001972|retinoic acid binding;GO:0019841	K17289	CRABP2; cellular retinoic acid-binding protein 2
SPPL2A	1208.471	-0.44229	0.135633183	-3.26094	0.0011104	0.01266	Not_Sig	no	gi|743738421|ref|XP_010963607.1|	2428	0	PREDICTED: signal peptide peptidase-like 2A isoform X1 [Camelus bactrianus]	gi|965805700|ref|XM_012118614.2|	4683	0	PREDICTED: Ovis aries musimon signal peptide peptidase like 2A (SPPL2A), transcript variant X4, mRNA	Q8TCT8	832	0	Signal peptide peptidase-like 2A	.	.	.	.	pfam04258	Peptidase_A22B	Signal peptide peptidase. The members of this family are membrane proteins. In some proteins this region is found	GO:0006509|membrane protein ectodomain proteolysis;GO:0031293|membrane protein intracellular domain proteolysis;GO:0033619|membrane protein proteolysis;GO:0050776|regulation of immune response;GO:0006465|signal peptide processing;	GO:0070062|extracellular exosome;GO:0030660|Golgi-associated vesicle membrane;GO:0071458|integral component of cytoplasmic side of	GO:0042500|aspartic endopeptidase activity, intramembrane	K09596	SPPL2A; signal peptide peptidase-like 2A [EC:3.4.23.-]
DNAJC19	135.4379	-0.62705	0.19229422	-3.26088	0.0011107	0.01266	DOWN	yes	gi|470648917|ref|XP_004327990.1|	707	3e-89	PREDICTED: mitochondrial import inner membrane translocase subunit TIM14-like [Tursiops truncatus]	gi|742185481|ref|XM_010855634.1|	581	0	PREDICTED: Bison bison bison DnaJ (Hsp40) homolog, subfamily C, member 19 (DNAJC19), mRNA	Q3ZBN8	237	1e-75	Mitochondrial import inner membrane translocase subunit TIM14	SPAC824.06	85.9	1.82e-19	COG2214	pfam00226	DnaJ	DnaJ domain. DnaJ domains (J-domains) are associated with hsp70 heat-shock system and it is thought that this domain mediates	GO:0015031|protein transport;	GO:0016021|integral component of membrane;GO:0005743|mitochondrial inner membrane;	.	K09539	DNAJC19; DnaJ homolog subfamily C member 19
SSH3	350.1293	0.50854	0.156030562	3.259231	0.0011171	0.012722	Not_Sig	no	gi|742109175|ref|XP_010836016.1|	3092	0	PREDICTED: protein phosphatase Slingshot homolog 3 isoform X2 [Bison bison bison]	gi|982971926|ref|XM_005227072.3|	2755	0	PREDICTED: Bos taurus slingshot protein phosphatase 3 (SSH3), transcript variant X1, mRNA	Q5XIS1	956	0	Protein phosphatase Slingshot homolog 3	SPBC17A3.06	77.8	1.65e-14	COG2453	pfam00782	DSPc	Dual specificity phosphatase, catalytic domain. Ser/Thr and Tyr protein phosphatases. The enzyme's tertiary fold is highly	GO:0008064|regulation of actin polymerization or depolymerization;GO:0050770|regulation of axonogenesis;GO:0010591|regulation of lamellipodium assembly;	GO:0005737|cytoplasm;GO:0005856|cytoskeleton;GO:0005634|nucleus;	GO:0003779|actin binding;GO:0003677|DNA binding;GO:0004725	K05766	SSH; protein phosphatase slingshot [EC:3.1.3.16
PTRH2	302.9266	-0.50436	0.154784368	-3.25845	0.0011202	0.012744	Not_Sig	no	gi|440908858|gb|ELR58836.1|	940	4e-125	Peptidyl-tRNA hydrolase 2, mitochondrial, partial [Bos mutus]	gi|982955900|ref|XM_005219894.3|	926	0	PREDICTED: Bos taurus peptidyl-tRNA hydrolase 2 (PTRH2), transcript variant X1, mRNA	Q3ZBL5	366	2e-126	Peptidyl-tRNA hydrolase 2, mitochondrial	YBL057c	126	2.15e-34	COG1990	pfam01981	PTH2	Peptidyl-tRNA hydrolase PTH2. Peptidyl-tRNA hydrolases are enzymes that release tRNAs from peptidyl-tRNA during translation.	.	GO:0005739|mitochondrion;	GO:0004045|aminoacyl-tRNA hydrolase activity;	K04794	PTH2; peptidyl-tRNA hydrolase, PTH2 family [EC:3.1.1.29]
UBA1	4896.214	0.337103	0.103482113	3.257595	0.0011236	0.01277	Not_Sig	no	gi|156523068|ref|NP_001095947.1|	5631	0	ubiquitin-like modifier-activating enzyme 1 [Bos taurus]	gi|942065092|ref|XM_005899353.2|	3528	0	PREDICTED: Bos mutus ubiquitin-like modifier activating enzyme 1 (UBA1), transcript variant X1, mRNA	A3KMV5	2173	0	Ubiquitin-like modifier-activating enzyme 1	YKL210w	1069	0	COG0476	pfam09358	UBA_e1_C	Ubiquitin-activating enzyme e1 C-terminal domain. This presumed domain found at the C-terminus of Ubiquitin-activating enzyme e1	GO:0006974|cellular response to DNA damage stimulus;GO:0019941|modification-dependent protein catabolic process;	GO:0005737|cytoplasm;GO:0005829|cytosol;GO:0030057|desmosome;GO:0010008|endosome membrane;GO:0070062|extracellular	GO:0005524|ATP binding;GO:0044822|poly(A) RNA binding;GO:0004839	K03178	UBE1, UBA1; ubiquitin-activating enzyme E1 [EC:6.2.1.45]
VPS11	575.3644	0.417909	0.128307095	3.257099	0.0011256	0.012779	Not_Sig	no	gi|555970335|ref|XP_005897190.1|	4615	0	PREDICTED: vacuolar protein sorting-associated protein 11 homolog isoform X2 [Bos mutus]	gi|942058222|ref|XM_005897128.2|	2950	0	PREDICTED: Bos mutus vacuolar protein sorting 11 homolog (S. cerevisiae) (VPS11), transcript variant X2, mRNA	Q9H270	1763	0	Vacuolar protein sorting-associated protein 11 homolog	.	.	.	.	pfam12451	VPS11_C	Vacuolar protein sorting protein 11 C terminal. This domain family is found in eukaryotes, and is approximately 50 amino acids in	GO:0006914|autophagy;GO:0034058|endosomal vesicle fusion;GO:0007032|endosome organization;GO:0008333|endosome to lysosome transport;GO:0006886|intracellular protein transport;GO:0007040|lysosome organization;GO:1903364|positive regulation of cellular protein catabolic process;GO:2000643|positive regulation of early endosome to late endosome transport;GO:1903955|positive regulation of protein targeting to mitochondrion;GO:1902115|regulation of organelle assembly;GO:0031647|regulation of protein stability;GO:0035542|regulation	GO:0005776|autophagosome;GO:0030136|clathrin-coated vesicle;GO:0005769|early endosome;GO:0030139|endocytic vesicle;GO:0005768|endosome;GO:003089	GO:0000166|nucleotide binding;GO:0030674|protein binding,	.	.
RO60	880.4375	-0.49671	0.152531386	-3.25647	0.0011281	0.012795	Not_Sig	no	gi|440904744|gb|ELR55214.1|	2834	0	60 kDa SS-A/Ro ribonucleoprotein, partial [Bos mutus]	gi|742238117|ref|XM_010832108.1|	7897	0	PREDICTED: Bison bison bison TROVE domain family, member 2 (TROVE2), mRNA	P10155	1048	0	60 kDa SS-A/Ro ribonucleoprotein	.	.	.	.	pfam05731	TROVE	TROVE domain. This presumed domain is found in TEP1 and Ro60 proteins, that are RNA-binding components of Telomerase, Ro	GO:0060271|cilium morphogenesis;GO:0002520|immune system development;GO:0009411|response to UV;GO:0007224|smoothened signaling pathway;GO:0006383|transcription from RNA polymerase III promoter;	GO:0005737|cytoplasm;GO:0030529|ribonucleoprotein complex;GO:0005634|nucleus;	GO:0046872|metal ion binding;GO:0003723|RNA	K11089	TROVE2, SSA2; 60 kDa SS-A/Ro ribonucleoprotein

COX10	303.467	0.50976	0.156557283	3.256058	0.0011297	0.012801	Not_Sig	no	gi|555950199|ref|XP_005887303.1|	2270	0	PREDICTED: protoheme IX farnesyltransferase, mitochondrial [Bos mutus]	gi|742136803|ref|XM_010842988.1|	2775	0	PREDICTED: Bison bison bison protoheme IX farnesyltransferase, mitochondrial (LOC104990706), mRNA	Q12887	721	0	Protoheme IX farnesyltransferase, mitochondrial	SPBC365.02c	199	8.98e-56	COG0109	pfam01040	UbiA	UbiA prenyltransferase family.	GO:0009060|aerobic respiration;GO:0045333|cellular respiration;GO:0006784|heme a biosynthetic process;GO:0006783|heme biosynthetic process;GO:0048034|heme O biosynthetic process;GO:1902600|hydrogen ion transmembrane transport;GO:0006123|mitochondrial electron transport, cytochrome c to oxygen;GO:0000266|mitochondrial fission;GO:0008535|respiratory chain complex IV assembly;	GO:0070069|cytochrome complex;GO:0016021|integral component of membrane;GO:0005743|mitochondrial inner	GO:0004311|farnesyltranstransferase activity;GO:0008495|protoheme IX	K02257	COX10; protoheme IX farnesyltransferase [EC:2.5.1.-]
MADD	501.0261	0.449625	0.138107955	3.255607	0.0011315	0.012809	Not_Sig	no	gi|742134795|ref|XP_010840701.1|	7755	0	PREDICTED: MAP kinase-activating death domain protein isoform X14 [Bison bison bison]	gi|742134794|ref|XM_010842399.1|	5821	0	PREDICTED: Bison bison bison MAP-kinase activating death domain (MADD), transcript variant X19, mRNA	Q8WXG6	2801	0	MAP kinase-activating death domain protein	.	.	.	.	pfam02141	DENN	DENN (AEX-3) domain. DENN (after differentially expressed in neoplastic vs normal cells) is a domain which occurs in several	GO:0000187|activation of MAPK activity;GO:0007166|cell surface receptor signaling pathway;GO:0097194|execution phase of apoptosis;GO:0042981|regulation of apoptotic process;GO:0051726|regulation of cell cycle;GO:2001236|regulation of extrinsic apoptotic signaling pathway;GO:1902041|regulation of extrinsic apoptotic signaling pathway via death domain receptors;GO:0032483|regulation of Rab protein signal transduction;GO:0010803|regulation of tumor necrosis factor-mediated signaling pathway;	GO:0005737|cytoplasm;GO:0005829|cytosol;GO:0016021|integral component of membrane;GO:0005886|plasma membrane;	GO:0005123|death receptor binding;GO:0030295|protein kinase	.	.
FKBP7	615.0826	-0.4701	0.144413599	-3.25522	0.001133	0.012814	Not_Sig	no	gi|555991597|ref|XP_005907582.1|	1162	3e-158	PREDICTED: peptidyl-prolyl cis-trans isomerase FKBP7 isoform X1 [Bos mutus]	gi|942090473|ref|XM_005907520.2|	908	0	PREDICTED: Bos mutus FK506 binding protein 7 (FKBP7), transcript variant X1, mRNA	Q5RET2	412	3e-144	Peptidyl-prolyl cis-trans isomerase FKBP7	YDR519w	87.4	1.64e-20	COG0545	pfam00254	FKBP_C	FKBP-type peptidyl-prolyl cis-trans isomerase.	GO:0006457|protein folding;	GO:0005788|endoplasmic reticulum lumen;	GO:0005509|calcium ion binding;GO:0003755|peptidyl-prolyl cis-	K09573	FKBP7; FK506-binding protein 7 [EC:5.2.1.8]
CISD2	449.6605	-0.47435	0.145777707	-3.25391	0.0011383	0.012845	Not_Sig	no	gi|440902686|gb|ELR53445.1|	656	2e-80	CDGSH iron-sulfur domain-containing protein 2, partial [Bos mutus]	gi|942066519|ref|XM_005899809.2|	1456	0	PREDICTED: Bos mutus CDGSH iron sulfur domain 2 (CISD2), mRNA	Q8N5K1	232	1e-72	CDGSH iron-sulfur domain-containing protein 2	.	.	.	.	pfam10660	MitoNEET_N	Iron-containing outer mitochondrial membrane protein N-terminus. MitoNEET_N is the N-terminal region of the MitoNEET	GO:0000422|mitophagy;GO:0010259|multicellular organismal aging;GO:0010506|regulation of autophagy;	GO:0005783|endoplasmic reticulum;GO:0005789|endoplasmic reticulum membrane;GO:0016021|integral component of	GO:0051537|2 iron, 2 sulfur cluster binding;GO:0046872|metal ion	.	.
RETREG2	1396.034	0.438701	0.134827031	3.253803	0.0011387	0.012845	Not_Sig	no	gi|329663573|ref|NP_001192537.1|	1697	0	protein FAM134A [Bos taurus]	gi|329663572|ref|NM_001205608.1|	2554	0	Bos taurus family with sequence similarity 134, member A (FAM134A), mRNA	Q8NC44	614	0	Protein FAM134A	.	.	.	.	pfam01690	PLRV_ORF5	Potato leaf roll virus readthrough protein. This family consists mainly of the potato leaf roll virus readthrough protein. This is	.	GO:0016021|integral component of membrane;	.	.	.
ATP5MF	444.2525	-0.49834	0.153162295	-3.25369	0.0011391	0.012845	Not_Sig	no	gi|594677986|ref|XP_007186763.1|	298	1e-31	PREDICTED: ATP synthase subunit f, mitochondrial [Balaenoptera acutorostrata scammoni]	gi|942047679|ref|XM_005893123.2|	416	0	PREDICTED: Bos mutus ATP synthase, H+ transporting, mitochondrial Fo complex, subunit F2 (ATP5J2), transcript variant X2, mRNA	Q28851	103	1e-27	ATP synthase subunit f, mitochondrial	.	.	.	.	pfam10206	WRW	Mitochondrial F1F0-ATP synthase, subunit f. This is a family of small proteins of approximately 110 amino acids, which are highly	GO:0006754|ATP biosynthetic process;GO:0015992|proton transport;	GO:0070062|extracellular exosome;GO:0016021|integral component of membrane;GO:0005753|mitochondrial proton-transporting ATP synthase	GO:0016887|ATPase activity;	K02130	ATPeF0F, ATP5J2; F-type H+-transporting ATPase subunit f
AGTRAP	344.3909	0.482092	0.148232789	3.252265	0.0011449	0.012897	Not_Sig	no	gi|741954246|ref|XP_005217011.2|	820	5e-99	PREDICTED: type-1 angiotensin II receptor-associated protein isoform X1 [Bos taurus]	gi|926711102|ref|XM_013970137.1|	1872	0	PREDICTED: Capra hircus angiotensin II receptor-associated protein (AGTRAP), mRNA	Q6RW13	249	6e-75	Type-1 angiotensin II receptor-associated protein	.	.	.	.	pfam06396	AGTRAP	Angiotensin II, type I receptor-associated protein (AGTRAP). This family consists of several angiotensin II, type I receptor-	GO:0008217|regulation of blood pressure;GO:0001666|response to hypoxia;	GO:0005938|cell cortex;GO:0005737|cytoplasm;GO:0030659|cytoplasmic vesicle membrane;GO:0005789|endoplasmic	GO:0004945|angiotensin type II receptor activity;	.	.
FBLN2	14289.41	0.457127	0.140627564	3.250624	0.0011515	0.012953	Not_Sig	no	gi|297488818|ref|XP_002697177.1|	5656	0	PREDICTED: fibulin-2 isoform X1 [Bos taurus]	gi|983004839|ref|XM_589271.8|	4153	0	PREDICTED: Bos taurus fibulin 2 (FBLN2), transcript variant X1, mRNA	P37889	1768	0	Fibulin-2	.	.	.	.	pfam07645	EGF_CA	Calcium-binding EGF domain.	GO:0010811|positive regulation of cell-substrate adhesion;	GO:0070062|extracellular exosome;GO:0031012|extracellular matrix;GO:1903561|extracellular vesicle;GO:0005578|proteinaceous	GO:0005509|calcium ion binding;GO:0050840|extracellular matrix	K17307	FBLN1_2; fibulin 1/2
RAPGEF1	824.2626	0.420503	0.129381438	3.250101	0.0011536	0.012953	Not_Sig	no	gi|528970000|ref|XP_005213318.1|	6003	0	PREDICTED: rap guanine nucleotide exchange factor 1 isoform X3 [Bos taurus]	gi|982939828|ref|XM_010810421.2|	5879	0	PREDICTED: Bos taurus Rap guanine nucleotide exchange factor 1 (RAPGEF1), transcript variant X7, mRNA	Q13905	1053	0	Rap guanine nucleotide exchange factor 1	.	.	.	.	pfam00617	RasGEF	RasGEF domain. Guanine nucleotide exchange factor for Ras-like small GTPases.	GO:0000186|activation of MAPKK activity;GO:0001568|blood vessel development;GO:0071320|cellular response to cAMP;GO:1990090|cellular response to nerve growth factor stimulus;GO:0061028|establishment of endothelial barrier;GO:0090090|negative regulation of canonical Wnt signaling pathway;GO:0070373|negative regulation of ERK1 and ERK2 cascade;GO:2000178|negative regulation of neural precursor cell proliferation;GO:0051898|negative regulation of protein kinase B signaling;GO:0046580|negative regulation of Ras	GO:0005829|cytosol;GO:0005769|early endosome;GO:0005768|endosome;	GO:0017034|Rap guanyl-nucleotide exchange factor activity;	K06277	RAPGEF1, GRF2; Rap guanine nucleotide exchange factor 1
DYNLT2B	71.63833	-0.85402	0.262805905	-3.24962	0.0011556	0.012953	DOWN	yes	gi|982910384|ref|XP_015326946.1|	1361	0	PREDICTED: tctex1 domain-containing protein 2 isoform X1 [Bos taurus]	gi|742094479|ref|XM_010834337.1|	877	0	PREDICTED: Bison bison bison Tctex1 domain containing 2 (TCTEX1D2), mRNA	Q32P71	325	5e-111	Tctex1 domain-containing protein 2	.	.	.	.	pfam03645	Tctex-1	Tctex-1 family. Tctex-1 is a dynein light chain. It has been shown that Tctex-1 can bind to the cytoplasmic tail of rhodopsin. C-	.	.	.	.	.
MDH2	2779.321	0.311414	0.09583866	3.249359	0.0011567	0.012953	Not_Sig	no	gi|635139449|ref|XP_008016570.1|	1704	0	PREDICTED: malate dehydrogenase, mitochondrial isoform X1 [Chlorocebus sabaeus]	gi|942046874|ref|XM_005892826.2|	1309	0	PREDICTED: Bos mutus malate dehydrogenase 2, NAD (mitochondrial) (MDH2), transcript variant X1, mRNA	Q32LG3	658	0	Malate dehydrogenase, mitochondrial	mdh	338	4.90e-114	COG0039	pfam02866	Ldh_1_C	lactate/malate dehydrogenase, alpha/beta C-terminal domain. L-lactate dehydrogenases are metabolic enzymes which catalyse	GO:0005975|carbohydrate metabolic process;GO:0006475|internal protein amino acid acetylation;GO:0006108|malate metabolic process;GO:0006099|tricarboxylic acid cycle;	GO:0005759|mitochondrial matrix;	GO:0030060|L-malate dehydrogenase activity;	.	.
URB1	194.0309	0.529051	0.162823721	3.249228	0.0011572	0.012953	Not_Sig	no	gi|742212259|ref|XP_010860606.1|	10357	0	PREDICTED: nucleolar pre-ribosomal-associated protein 1 isoform X2 [Bison bison bison]	gi|329664477|ref|NM_001205980.1|	7199	0	Bos taurus URB1 ribosome biogenesis 1 homolog (S. cerevisiae) (URB1), mRNA	O60287	3150	0	Nucleolar pre-ribosomal-associated protein 1	.	.	.	.	pfam11707	Npa1	Ribosome 60S biogenesis N-terminal. Npa1p is required for ribosome biogenesis and operates in the same functional	.	GO:0005730|nucleolus;	GO:0044822|poly (A) RNA binding;	K14861	URB1; nucleolar pre-ribosomal-associated protein 1
ASB6	474.3489	0.458677	0.141168963	3.249135	0.0011576	0.012953	Not_Sig	no	gi|329663972|ref|NP_001192590.1|	2216	0	ankyrin repeat and SOCS box protein 6 [Bos taurus]	gi|329663971|ref|NM_001205661.1|	2035	0	Bos taurus ankyrin repeat and SOCS box containing 6 (ASB6), mRNA	Q9NWX5	817	0	Ankyrin repeat and SOCS box protein 6	all2748	71.6	3.35e-12	COG0666	pfam12796	Ank_2	Ankyrin repeats (3 copies).	GO:0035556|intracellular signal transduction;GO:0016567|protein ubiquitination;	GO:0005737|cytoplasm;	.	K10328	ASB6; ankyrin repeat and SOCS box protein 6
PDXK	2715.843	0.329834	0.101515611	3.249094	0.0011577	0.012953	Not_Sig	no	gi|115497140|ref|NP_001069119.1|	1635	0	pyridoxal kinase [Bos taurus]	gi|926684292|ref|XM_013966831.1|	1370	0	PREDICTED: Capra hircus pyridoxal (pyridoxine, vitamin B6) kinase (PDXK), mRNA	Q0II59	634	0	Pyridoxal kinase	SPAC6F6.11c	190	1.14e-52	COG2240	pfam00294	PfkB	pfkB family carbohydrate kinase. This family includes a variety of carbohydrate and pyrimidine kinases.	GO:0008283|cell proliferation;GO:0009443|pyridoxal 5'-phosphate salvage;GO:0042816|vitamin B6 metabolic process;	GO:0005829|cytosol;GO:0070062|extracellular exosome;GO:0005654|nucleoplasm;	GO:0005524|ATP binding;GO:0031403|lithium ion binding;GO:0000287	K00868	pdxK, pdxY; pyridoxine kinase [EC:2.7.1.35]
CNOT3	470.1185	0.511076	0.157340512	3.248219	0.0011613	0.012956	Not_Sig	no	gi|831245891|ref|XP_012668074.1|	1492	0	PREDICTED: CCR4-NOT transcription complex subunit 3 isoform X6 [Otolemur garnettii]	gi|983000950|ref|XM_005192848.2|	2676	0	PREDICTED: Bos taurus CCR4-NOT transcription complex subunit 3 (CNOT3), transcript variant X4, mRNA	O75175	568	0	CCR4-NOT transcription complex subunit 3	SPAC1B3.05	201	3.01e-54	COG5665	pfam04065	Not3	Not1 N-terminal domain, CCR4-Not complex component.	GO:0006977|DNA damage response, signal transduction by p53 class mediator resulting in cell cycle arrest;GO:0031047|gene silencing by RNA;GO:0017148|negative regulation of translation;GO:0000288|nuclear-transcribed mRNA catabolic process, deadenylation-dependent decay;GO:0000289|nuclear-transcribed mRNA poly(A) tail shortening;GO:2000036|regulation of stem cell population maintenance;GO:0006355|regulation of transcription, DNA-templated;GO:0006351|transcription, DNA-	GO:0030014|CCR4-NOT complex;GO:0030015|CCR4-NOT core complex;GO:0000932|cytoplasmic mRNA processing	.	K12580	CNOT3, NOT3; CCR4-NOT transcription complex subunit 3
SAR1B	1098.315	-0.47254	0.145482245	-3.24806	0.0011619	0.012956	Not_Sig	no	gi|795530233|ref|XP_011714856.1|	1073	2e-130	PREDICTED: GTP-binding protein SAR1b isoform X1 [Macaca nemestrina]	gi|742142038|ref|XM_010844505.1|	5100	0	PREDICTED: Bison bison bison secretion associated, Ras related GTPase 1B (SAR1B), mRNA	Q3T0T7	410	1e-130	GTP-binding protein SAR1b	.	.	.	.	pfam00025	Arf	ADP-ribosylation factor family. Pfam combines a number of different Prosite families together	GO:0006886|intracellular protein transport;GO:0016192|vesicle-mediated transport;	GO:0005789|endoplasmic reticulum membrane;GO:0032580|Golgi cisterna membrane;	GO:0005525|GTP binding;GO:0046872|metal ion binding;	K07953	SAR1; GTP-binding protein SAR1 [EC:3.6.5.-]
EID3	173.9478	-0.54634	0.168209478	-3.248	0.0011622	0.012956	Not_Sig	no	gi|154152023|ref|NP_001093782.1|	1854	0	EP300-interacting inhibitor of differentiation 3 [Bos taurus]	gi|742176860|ref|XM_010853409.1|	1779	0	PREDICTED: Bison bison bison EP300 interacting inhibitor of differentiation 3 (EID3), mRNA	A6QPC8	718	0	EP300-interacting inhibitor of differentiation 3	ECU08g1820	60.5	2.80e-09	COG5125	pfam08743	Nse4_C	Nse4 C-terminal. Nse4 is a component of the Smc5/6 DNA repair complex. It forms interactions with Smc5 and Nse1.	GO:0006310|DNA recombination;GO:0006281|DNA repair;GO:0006355|regulation of transcription, DNA-templated;GO:0006351|transcription, DNA-templated;	GO:0000781|chromosome, telomeric region;GO:0005737|cytoplasm;GO:0005634|nucleus;GO:0030915|Smc5-Smc6 complex;	.	.	.
NRM	248.1408	0.514869	0.15852401	3.247894	0.0011626	0.012956	Not_Sig	no	gi|83035053|ref|NP_001032685.1|	1025	5e-129	nurim [Bos taurus]	gi|742158142|ref|XM_010848153.1|	2697	0	PREDICTED: Bison bison bison nurim (nuclear envelope membrane protein) (NRM), mRNA	Q32LM8	399	2e-130	Nurim	.	.	.	.	pfam13657	Couple_hipA	HipA N-terminal domain. This domain is found to the N-terminus of HipA-like proteins. It is also found in isolation in some	.	GO:0016021|integral component of membrane;GO:0005635|nuclear envelope;GO:0005637|nuclear inner membrane;	.	.	.
OCIAD1	876.4617	-0.37642	0.115923849	-3.24717	0.0011656	0.012956	Not_Sig	no	gi|62751843|ref|NP_001015648.1|	1329	1e-180	OCIA domain-containing protein 1 [Bos taurus]	gi|942059395|ref|XM_014478778.1|	1409	0	PREDICTED: Bos mutus OCIA domain containing 1 (OCIAD1), transcript variant X1, mRNA	Q5E948	516	0	OCIA domain-containing protein 1	.	.	.	.	pfam07051	OCIA	Ovarian carcinoma immunoreactive antigen (OCIA). This family consists of several ovarian carcinoma	.	GO:0005768|endosome;	.	.	.
RAP2A	432.8338	-0.45817	0.141098352	-3.24714	0.0011657	0.012956	Not_Sig	no	gi|686744183|ref|XP_009247013.1|	901	1e-108	PREDICTED: ras-related protein Rap-2a [Pongo abelii]	gi|594059931|ref|XM_006054022.1|	3632	0	PREDICTED: Bubalus bubalis RAP2A, member of RAS oncogene family (RAP2A), transcript variant X1, mRNA	Q5R988	348	2e-109	Ras-related protein Rap-2a	.	.	.	.	pfam00071	Ras	Ras family. Includes sub-families Ras, Rab, Rac, Ral, Ran, Rap Ypt1 and more. Shares P-loop motif with GTP_EFTU, arf and	GO:0031532|actin cytoskeleton reorganization;GO:0034613|cellular protein localization;GO:0031954|positive regulation of protein autophosphorylation;GO:0032486|Rap protein signal transduction;GO:0048814|regulation of dendrite morphogenesis;GO:0046328|regulation of JNK cascade;	GO:0030496|midbody;GO:0055038|recycling endosome membrane;	GO:0005525|GTP binding;GO:0003924|GTPase activity;	.	.
LAMTOR2	381.6334	-0.45561	0.140313688	-3.2471	0.0011659	0.012956	Not_Sig	no	gi|7661728|ref|NP_054736.1|	652	6e-84	ragulator complex protein LAMTOR2 isoform 1 [Homo sapiens]	gi|942063171|ref|XM_005898714.2|	632	0	PREDICTED: Bos mutus late endosomal/lysosomal adaptor, MAPK and MTOR activator 2 (LAMTOR2), transcript variant X1, mRNA	Q9Y2Q5	255	2e-85	Ragulator complex protein LAMTOR2	.	.	.	.	pfam03259	Robl_LC7	Roadblock/LC7 domain. This family includes proteins that are about 100 amino acids long and have been shown to be related.	GO:0000186|activation of MAPKK activity;GO:0007050|cell cycle arrest;GO:0016049|cell growth;GO:0034613|cellular protein localization;GO:0071230|cellular response to amino acid stimulus;GO:0016236|macroautophagy;GO:0000165|MAPK cascade;GO:0043547|positive regulation of GTPase activity;GO:0032008|positive regulation of TOR signaling;	GO:0010008|endosome membrane;GO:0070062|extracellular exosome;GO:0005770|late endosome;GO:0005765|lysosomal	.	.	.
NPLOC4	2086.941	0.310958	0.095781975	3.24652	0.0011683	0.012969	Not_Sig	no	gi|300794221|ref|NP_001179118.1|	3302	0	nuclear protein localization protein 4 homolog [Bos taurus]	gi|742187882|ref|XM_010856315.1|	4003	0	PREDICTED: Bison bison bison nuclear protein localization 4 homolog (S. cerevisiae) (NPLOC4), mRNA	P60670	1245	0	Nuclear protein localization protein 4 homolog	YBR170c	337	2.05e-101	COG5100	pfam05021	NPL4	NPL4 family. The HRD4 gene was identical to NPL4, a gene previously implicated in nuclear transport. Using a diverse set of	GO:0030433|ER-associated ubiquitin-dependent protein catabolic process;GO:0007030|Golgi organization;GO:0030970|retrograde protein transport, ER to cytosol;GO:0006511|ubiquitin-dependent protein catabolic process;	GO:0005829|cytosol;GO:0005783|endoplasmic reticulum;GO:0042175|nuclear outer membrane-endoplasmic reticulum membrane	GO:0043130|ubiquitin binding;GO:0031625|ubiquitin protein	K14015	NPLOC4, NPL4; nuclear protein localization protein 4 homolog
RAF1	1486.496	0.424478	0.130778957	3.24577	0.0011713	0.012991	Not_Sig	no	gi|156523122|ref|NP_001095975.1|	3441	0	RAF proto-oncogene serine/threonine-protein kinase [Bos taurus]	gi|156523121|ref|NM_001102505.1|	3155	0	Bos taurus Raf-1 proto-oncogene, serine/threonine kinase (RAF1), mRNA	A7E3S4	1271	0	RAF proto-oncogene serine/threonine-protein kinase	.	.	.	.	pfam00069	Pkinase	Protein kinase domain.	GO:0035556|intracellular signal transduction;	GO:0005737|cytoplasm;GO:0005739|mitochondrion;GO:0005634|nucleus;GO:0005886|plasma membrane;	GO:0005524|ATP binding;GO:0046872|metal ion binding;GO:0004674	K04366	RAF1; RAF proto-oncogene serine/threonine-protein kinase
LOC138991827	764.2682	0.509796	0.157103453	3.244969	0.0011746	0.013015	Not_Sig	no	gi|440912800|gb|ELR62335.1|	197	1e-14	hypothetical protein M91_01857, partial [Bos mutus]	gi|594070489|ref|XR_327605.1|	1638	0	PREDICTED: Bubalus bubalis uncharacterized LOC102394554 (LOC102394554), misc_RNA	Q96FN4	77.4	7e-13	Copine-2	.	.	.	.	pfam07002	Copine	Copine. This family represents a conserved region approximately 180 residues long within eukaryotic copines. Copines are	GO:0071277|cellular response to calcium ion;	GO:0005737|cytoplasm;GO:0070062|extracellular exosome;GO:0005634|nucleus;GO:0005886|plasma membrane;	.	.	.
LOC102275123	75.58274	-0.76832	0.236816305	-3.24438	0.0011771	0.013029	DOWN	yes	gi|488558799|ref|XP_004469682.1|	418	2e-49	PREDICTED: 40S ribosomal protein S27-like [Dasypus novemcinctus]	gi|982979528|ref|XM_010827051.2|	504	0	PREDICTED: Bos taurus 40S ribosomal protein S27-like (LOC101903478), mRNA	Q3T0B7	157	3e-48	40S ribosomal protein S27-like	SPBC1685.10	119	9.24e-35	COG2051	pfam01667	Ribosomal_S27e	Ribosomal protein S27.	GO:0000028|ribosomal small subunit assembly;GO:0006412|translation;	GO:0022627|cytosolic small ribosomal subunit;	GO:0046872|metal ion binding;GO:0003735|structural	K02978	RP-S27e, RPS27; small subunit ribosomal protein S27e
RPL9	5781.735	-0.38005	0.11716999	-3.24357	0.0011804	0.013054	Not_Sig	no	gi|164420694|ref|NP_001019640.2|	939	1e-122	60S ribosomal protein L9 [Bos taurus]	gi|982986956|ref|XR_801922.2|	1301	0	PREDICTED: Bos taurus 60S ribosomal protein L9 pseudogene (LOC101905894), misc_RNA	Q3SYR7	366	5e-124	60S ribosomal protein L9	SPAC4G9.16c	199	2.10e-61	COG0097	pfam00347	Ribosomal_L6	Ribosomal protein L6.	GO:0002181|cytoplasmic translation;	GO:0022625|cytosolic large ribosomal subunit;	GO:0003723|RNA binding;GO:0019843|rRNA binding;GO:0003735	K02940	RP-L9e, RPL9; large subunit ribosomal protein L9e
NFAT5	989.6742	-0.42648	0.131551808	-3.24188	0.0011874	0.013119	Not_Sig	no	gi|942056637|ref|XP_014333975.1|	6690	0	PREDICTED: nuclear factor of activated T-cells 5 isoform X1 [Bos mutus]	gi|982999462|ref|XM_015467392.1|	12347	0	PREDICTED: Bos taurus nuclear factor of activated T-cells 5, tonicity-responsive (NFAT5), transcript variant X1, mRNA	O94916	2372	0	Nuclear factor of activated T-cells 5	.	.	.	.	pfam00554	RHD	Rel homology domain (RHD). Proteins containing the Rel homology domain (RHD) are eukaryotic transcription factors.	GO:0001816|cytokine production;GO:0007588|excretion;GO:0045944|positive regulation of transcription from RNA polymerase II promoter;GO:0070884|regulation of calcineurin-NFAT signaling cascade;GO:0006970|response to osmotic stress;GO:0007165|signal transduction;GO:0006366|transcription from RNA polymerase II promoter;	GO:0005737|cytoplasm;GO:0005654|nucleoplasm;GO:0005634|nucleus;	GO:0000978|RNA polymerase II core promoter proximal region sequence-	K17335	NFAT5; nuclear factor of activated T-cells 5
NATD1	536.6589	0.441755	0.136302225	3.240998	0.0011911	0.013147	Not_Sig	no	gi|296238075|ref|XP_002764010.1|	327	6e-32	PREDICTED: protein GTLF3B [Callithrix jacchus]	gi|982957337|ref|XM_005220417.3|	2754	0	PREDICTED: Bos taurus N-acetyltransferase domain containing 1 (NATD1), transcript variant X1, mRNA	Q8N6N6	130	3e-33	Protein NATD1	.	.	.	.	pfam14542	Acetyltransf_CG	GCN5-related N-acetyl-transferase. This family of GCN5-related N-acetyl-transferases bind both CoA and acetyl-CoA. They are	.	.	.	K06975	K06975; uncharacterized protein
LOC102287927	3880.492	-0.37951	0.11715408	-3.23938	0.0011979	0.013209	Not_Sig	no	gi|724803913|ref|XP_010382506.1|	701	3e-88	PREDICTED: tetratricopeptide repeat protein 28-like [Rhinopithecus roxellana]	gi|742212888|ref|XM_010862454.1|	541	0	PREDICTED: Bison bison bison 60S ribosomal protein L32 (LOC105004738), mRNA	Q3SZQ6	252	3e-84	60S ribosomal protein L32	SPAC3H5.10	143	1.08e-43	COG1717	pfam01655	Ribosomal_L32e	Ribosomal protein L32. This family includes ribosomal protein L32 from eukaryotes and archaebacteria.	GO:0006412|translation;	GO:0022625|cytosolic large ribosomal subunit;GO:0016020|membrane;	GO:0044822|poly (A) RNA binding;GO:0003735|structural	K02912	RP-L32e, RPL32; large subunit ribosomal protein L32e
MED13	1439.982	-0.36686	0.113269434	-3.23881	0.0012003	0.01322	Not_Sig	no	gi|983001250|ref|XP_015323413.1|	10626	0	PREDICTED: mediator of RNA polymerase II transcription subunit 13 isoform X2 [Bos taurus]	gi|983001249|ref|XM_015467927.1|	10406	0	PREDICTED: Bos taurus mediator complex subunit 13 (MED13), transcript variant X2, mRNA	Q9UHV7	3954	0	Mediator of RNA polymerase II transcription subunit 13	.	.	.	.	pfam06333	Med13_C	Mediator complex subunit 13 C-terminal. Mediator is a large complex of up to 33 proteins that is conserved from plants through	GO:0030521|androgen receptor signaling pathway;GO:0042632|cholesterol homeostasis;GO:0030518|intracellular steroid hormone receptor signaling pathway;GO:1904168|negative regulation of thyroid hormone receptor activity;GO:0045944|positive regulation of transcription from RNA polymerase II promoter;GO:0045893|positive regulation of transcription, DNA-templated;GO:0006367|transcription initiation from RNA polymerase II promoter;GO:0070328|triglyceride homeostasis;	GO:0016592|mediator complex;GO:0016020|membrane;GO:0005654|nucleoplasm;GO:0005634|nucleus;	GO:0030374|ligand-dependent nuclear receptor transcription	K15164	MED13; mediator of RNA polymerase II transcription subunit 13
RFFL	492.2109	0.407911	0.125952639	3.238605	0.0012012	0.01322	Not_Sig	no	gi|942066089|ref|XP_014334884.1|	2028	0	PREDICTED: E3 ubiquitin-protein ligase rififylin [Bos mutus]	gi|742209011|ref|XM_010861494.1|	3691	0	PREDICTED: Bison bison bison ring finger and FYVE-like domain containing E3 ubiquitin protein ligase (RFFL), transcript variant X1, mRNA	Q8WZ73	630	0	E3 ubiquitin-protein ligase rififylin	.	.	.	.	pfam13920	zf-C3HC4_3	Zinc finger, C3HC4 type (RING finger).	GO:0006915|apoptotic process;GO:2001271|negative regulation of cysteine-type endopeptidase activity involved in execution phase of apoptosis;GO:1902042|negative regulation of extrinsic apoptotic signaling pathway via death domain receptors;GO:1901797|negative regulation of signal transduction by p53 class mediator;GO:0010804|negative regulation of tumor necrosis factor-mediated signaling pathway;GO:0043161|proteasome-mediated ubiquitin-dependent protein catabolic process;GO:0070936|protein K48-linked	GO:0005737|cytoplasm;GO:0005829|cytosol;GO:0010008|endosome membrane;GO:0016020|membrane;GO:0005654|nucleoplasm;GO:0005886|plasma	GO:0016874|ligase activity;GO:0002039|p53 binding;GO:0002020	.	.
SMAD5	1277.7	-0.45091	0.139302997	-3.23694	0.0012082	0.013284	Not_Sig	no	gi|440909355|gb|ELR59268.1|	2022	0	Mothers against decapentaplegic-like protein 5, partial [Bos mutus]	gi|942043246|ref|XM_005891686.2|	6852	0	PREDICTED: Bos mutus SMAD family member 5 (SMAD5), transcript variant X2, mRNA	Q5R6H7	777	0	Mothers against decapentaplegic homolog 5	.	.	.	.	pfam03166	MH2	MH2 domain. This is the MH2 (MAD homology 2) domain found at the carboxy terminus of MAD related proteins such as Smads.	GO:0030509|BMP signaling pathway;GO:0060348|bone development;GO:0060048|cardiac muscle contraction;GO:0051216|cartilage development;GO:0071407|cellular response to organic cyclic compound;GO:0009880|embryonic pattern specification;GO:0030218|erythrocyte differentiation;GO:0007281|germ cell development;GO:0002051|osteoblast fate commitment;GO:0045669|positive regulation of osteoblast differentiation;GO:1901522|positive regulation of transcription from RNA	GO:0005737|cytoplasm;GO:0005654|nucleoplasm;GO:0005667|transcription factor complex;	GO:0046872|metal ion binding;GO:0000979|RNA polymerase II	K16790	SMAD5; mothers against decapentaplegic homolog 5
RASSF8	1786.317	-0.39761	0.122897248	-3.23534	0.001215	0.013346	Not_Sig	no	gi|440900368|gb|ELR51519.1|	1901	0	Ras association domain-containing protein 8, partial [Bos mutus]	gi|742186783|ref|XM_010856002.1|	5438	0	PREDICTED: Bison bison bison Ras association (RalGDS/AF-6) domain family (N-terminal) member 8 (RASSF8), transcript variant X4, mRNA	Q8NHQ8	719	0	Ras association domain-containing protein 8	.	.	.	.	pfam13166	AAA_13	AAA domain. This family of domains contain a P-loop motif that is characteristic of the AAA superfamily. Many of the proteins	GO:0034334|adherens junction maintenance;GO:0007165|signal transduction;	.	.	K09855	RASSF7_8; Ras association domain-containing protein 7/8
MIDN	665.4356	0.483723	0.149560481	3.234294	0.0012194	0.013382	Not_Sig	no	gi|528907537|ref|XP_005199082.1|	1861	0	PREDICTED: midnolin isoform X1 [Bos taurus]	gi|982985264|ref|XM_015464254.1|	3427	0	PREDICTED: Bos taurus midnolin (MIDN), transcript variant X2, mRNA	Q504T8	553	2e-179	Midnolin	.	.	.	.	pfam00240	ubiquitin	Ubiquitin family. This family contains a number of ubiquitin-like proteins: SUMO (smt3 homologue) Nedd8, Elongin B, Rub1, and	GO:0033132|negative regulation of glucokinase activity;GO:0046676|negative regulation of insulin secretion;	GO:0005829|cytosol;GO:0005730|nucleolus;	.	.	.
TJP1	2303.409	0.343252	0.106163261	3.233243	0.0012239	0.013408	Not_Sig	no	gi|742137625|ref|XP_010841557.1|	8445	0	PREDICTED: tight junction protein ZO-1 isoform X1 [Bison bison bison]	gi|742137628|ref|XM_010843257.1|	6591	0	PREDICTED: Bison bison bison tight junction protein 1 (TJP1), transcript variant X2, mRNA	O97758	2866	0	Tight junction protein ZO-1	.	.	.	.	pfam00791	ZU5	ZU5 domain. Domain present in ZO-1 and Unc5-like netrin receptors Domain of unknown function.	GO:0070830|bicellular tight junction assembly;GO:0071277|cellular response to calcium ion;	GO:0005923|bicellular tight junction;GO:0009986|cell surface;GO:0005911|cell-cell junction;GO:0031410|cytoplasmic	.	K05701	TJP1, ZO1; tight junction protein 1
SLC19A1	256.4985	0.501485	0.155115711	3.232972	0.0012251	0.013408	Not_Sig	no	gi|742096793|ref|XP_010833173.1|	2475	0	PREDICTED: folate transporter 1 isoform X1 [Bison bison bison]	gi|594056142|ref|XM_006052222.1|	2546	0	PREDICTED: Bubalus bubalis solute carrier family 19 (folate transporter), member 1 (SLC19A1), transcript variant X2, mRNA	P41440	654	0	Folate transporter 1	.	.	.	.	pfam01770	Folate_carrier	Reduced folate carrier. The reduced folate carrier (a transmembrane glycoprotein) transports reduced folate into	GO:0046655|folic acid metabolic process;GO:0015884|folic acid transport;GO:0098838|reduced folate transmembrane transport;	GO:0016324|apical plasma membrane;GO:0016323|basolateral plasma membrane;GO:0005887|integral component of plasma	GO:0005542|folic acid binding;GO:0008517|folic acid	K14609	SLC19A1, RFC1; solute carrier family 19 (folate transporter),
LOC102276080	1926.551	-0.53389	0.165140602	-3.23294	0.0012252	0.013408	Not_Sig	no	gi|15718687|ref|NP_000996.2|	1265	4e-174	40S ribosomal protein S3 isoform 1 [Homo sapiens]	gi|742230800|ref|XM_010830508.1|	828	0	PREDICTED: Bison bison bison 40S ribosomal protein S3 (LOC104981419), mRNA	Q0Z8U2	491	1e-175	40S ribosomal protein S3	SPBC16G5.14c	325	5.69e-112	COG0092	pfam00189	Ribosomal_S3_C	Ribosomal protein S3, C-terminal domain. This family contains a central domain pfam00013, hence the amino and carboxyl terminal	GO:0006915|apoptotic process;GO:0051301|cell division;GO:0006281|DNA repair;GO:0007067|mitotic nuclear division;GO:0006355|regulation of transcription, DNA-templated;GO:0006417|regulation of translation;GO:0006351|transcription, DNA-templated;GO:0006412|translation;	GO:0022627|cytosolic small ribosomal subunit;GO:0030529|ribonucleoprotein complex;GO:0005743|mitochondrial inner membrane;GO:0005730|nucleolus;GO:0005	GO:0003684|damaged DNA binding;GO:0008534|oxidized purine	K02985	RP-S3e, RPS3; small subunit ribosomal protein S3e
PIGS	925.9421	0.422295	0.130649305	3.232277	0.0012281	0.013414	Not_Sig	no	gi|296476837|tpg|DAA18952.1|	2641	0	TPA: GPI transamidase component PIG-S [Bos taurus]	gi|742208678|ref|XM_010861419.1|	2513	0	PREDICTED: Bison bison bison phosphatidylinositol glycan anchor biosynthesis, class S (PIGS), mRNA	Q3SZL5	1019	0	GPI transamidase component PIG-S	.	.	.	.	pfam10510	PIG-S	Phosphatidylinositol-glycan biosynthesis class S protein. PIG-S is one of several key, core, components of the	GO:0016255|attachment of GPI anchor to protein;	GO:0042765|GPI-anchor transamidase complex;	.	K05291	PIGS; phosphatidylinositol glycan, class S
PSRC1	106.0416	0.657649	0.203464018	3.23226	0.0012282	0.013414	UP	yes	gi|114051025|ref|NP_001039966.1|	1392	0	proline/serine-rich coiled-coil protein 1 [Bos taurus]	gi|594056021|ref|XM_006052163.1|	1745	0	PREDICTED: Bubalus bubalis proline/serine-rich coiled-coil 1 (PSRC1), transcript variant X1, mRNA	Q29RJ9	540	0	Proline/serine-rich coiled-coil protein 1	.	.	.	.	pfam15259	GTSE1_N	G-2 and S-phase expressed 1. This family is the N-terminus of GTSE1 proteins. GTSE-1 (G2 and S phase-expressed-1) protein is specifically	GO:0051301|cell division;GO:0001578|microtubule bundle formation;GO:0007080|mitotic metaphase plate congression;GO:0030308|negative regulation of cell growth;GO:0045737|positive regulation of cyclin-dependent protein serine/threonine kinase activity;GO:0031116|positive regulation of microtubule polymerization;GO:0045893|positive regulation of transcription, DNA-templated;GO:0060236|regulation of mitotic spindle organization;	GO:0005829|cytosol;GO:0030496|midbody;GO:0005876|spindle microtubule;GO:0000922|spindle pole;	.	.	.

NISCH	1150.649	0.387913	0.120025349	3.231925	0.0012296	0.013417	Not_Sig	no	gi|742171770|ref|XP_010850241.1|	7133	0	PREDICTED: nischarin, partial [Bison bison bison]	gi|742171769|ref|XM_010851939.1|	5071	0	PREDICTED: Bison bison bison nischarin (NISCH), partial mRNA	Q9Y2I1	2517	0	Nischarin	alr0124_1	60.8	1.99e-08	COG4886	pfam00787	PX	PX domain. PX domains bind to phosphoinositides.	GO:0030036|actin cytoskeleton organization;GO:0006915|apoptotic process;GO:0006006|glucose metabolic process;GO:0030336|negative regulation of cell migration;GO:0048243|norepinephrine secretion;GO:0016601|Rac protein signal transduction;GO:0008217|regulation of blood pressure;GO:0032228|regulation of synaptic transmission, GABAergic;	GO:0005829|cytosol;GO:0005769|early endosome;GO:0016020|membrane;GO:0005886|plasma membrane;GO:0055037|recycling	GO:0035091|phosphatidylinositol binding;	.	.
CCDC85B	1025.005	0.49295	0.152592405	3.230503	0.0012357	0.013471	Not_Sig	no	gi|1488314|gb|AAB05928.1|	457	7e-50	hepatitis delta antigen interacting protein A [Homo sapiens]	gi|742109434|ref|XM_010837777.1|	1527	0	PREDICTED: Bison bison bison coiled-coil domain containing 85B (CCDC85B), mRNA	Q15834	179	8e-51	Coiled-coil domain-containing protein 85B	.	.	.	.	pfam10226	DUF2216	Uncharacterized conserved proteins (DUF2216). This is the conserved N-terminal half of a proteins which are found from	GO:0030154|cell differentiation;GO:0030308|negative regulation of cell growth;GO:0045599|negative regulation of fat cell differentiation;GO:0045892|negative regulation of transcription, DNA-templated;GO:0006351|transcription, DNA-templated;	GO:0005813|centrosome;GO:0005737|cytoplasm;GO:0005634|nucleus;	.	K16758	CCDC85B; coiled-coil domain-containing protein 85B
TYK2	1237.815	0.390082	0.120878622	3.227052	0.0012507	0.013622	Not_Sig	no	gi|440899931|gb|ELR51172.1|	6120	0	Non-receptor tyrosine-protein kinase TYK2, partial [Bos mutus]	gi|982930396|ref|XM_005208737.3|	5764	0	PREDICTED: Bos taurus tyrosine kinase 2 (TYK2), transcript variant X1, mRNA	P29597	1956	0	Non-receptor tyrosine-protein kinase TYK2	.	.	.	.	pfam07714	Pkinase_Tyr	Protein tyrosine kinase.	GO:0030154|cell differentiation;GO:0016477|cell migration;GO:0045087|innate immune response;GO:0035556|intracellular signal transduction;GO:0038083|peptidyl-tyrosine autophosphorylation;GO:0006468|protein phosphorylation;GO:0042127|regulation of cell proliferation;GO:0060338|regulation of type I interferon-mediated signaling pathway;GO:0007169|transmembrane receptor protein tyrosine kinase signaling pathway;GO:0060337|type I interferon signaling pathway;	GO:0005737|cytoplasm;GO:0005856|cytoskeleton;GO:0005829|cytosol;GO:0070062|extracellular exosome;GO:0031234|extrinsic component of cytoplasmic side of plasma	GO:0005524|ATP binding;GO:0005131|growth hormone receptor	K11219	TYK2; non-receptor tyrosine-protein kinase TYK2 [EC:2.7.10.2]
CCDC120	167.8571	0.582837	0.180872563	3.222362	0.0012714	0.013817	Not_Sig	no	gi|555951022|ref|XP_005887711.1|	2182	0	PREDICTED: coiled-coil domain-containing protein 120 isoform X1 [Bos mutus]	gi|982973884|ref|XM_010821886.2|	3962	0	PREDICTED: Bos taurus coiled-coil domain containing 120 (CCDC120), transcript variant X2, mRNA	Q96HB5	743	0	Coiled-coil domain-containing protein 120	.	.	.	.	pfam11819	DUF3338	Domain of unknown function (DUF3338). This family of proteins are functionally uncharacterized. This family is found in eukaryotes.	GO:0007275|multicellular organismal development;	GO:0005768|endosome;GO:0030426|growth cone;	.	.	.
RITA1	524.2479	0.47192	0.146458101	3.222218	0.001272	0.013817	Not_Sig	no	gi|942081618|ref|XP_014336613.1|	1409	0	PREDICTED: RBPJ-interacting and tubulin-associated protein 1 isoform X1 [Bos mutus]	gi|982950382|ref|XM_005217878.3|	1797	0	PREDICTED: Bos taurus RBPJ interacting and tubulin associated 1 (RITA1), transcript variant X2, mRNA	Q2HJ75	496	1e-171	RBPJ-interacting and tubulin-associated protein 1	.	.	.	.	pfam09770	PAT1	Topoisomerase II-associated protein PAT1. Members of this family are necessary for accurate chromosome transmission during	GO:0045746|negative regulation of Notch signaling pathway;GO:0022008|neurogenesis;GO:0007219|Notch signaling pathway;GO:0051168|nuclear export;	GO:0005813|centrosome;GO:0005737|cytoplasm;GO:0005634|nucleus;	GO:0015631|tubulin binding;	.	.
LOC102272412	794.9314	-0.48698	0.151132769	-3.22217	0.0012722	0.013817	Not_Sig	no	gi|594044792|ref|XP_006046886.1|	227	2e-20	PREDICTED: 26S proteasome complex subunit DSS1-like [Bubalus bubalis]	gi|402692083|ref|NM_001113317.2|	258	6e-131	Bos taurus split hand/foot malformation (ectrodactyly) type 1 (SHFM1), mRNA	P60897	67.4	1e-13	26S proteasome complex subunit DSS1	.	.	.	.	pfam05160	DSS1_SEM1	DSS1/SEM1 family. This family contains the breast cancer tumor suppressor BRCA2-interacting protein DSS1 and its homologue	GO:0000724|double-strand break repair via homologous recombination;GO:0006406|mRNA export from nucleus;GO:0043248|proteasome assembly;GO:0006508|proteolysis;	GO:0000502|proteasome complex;GO:0008541|proteasome regulatory particle, lid subcomplex;	.	K10881	SHFM1, DSS1; 26 proteasome complex subunit DSS1
SCOC	575.5284	-0.60254	0.187090856	-3.2206	0.0012792	0.013837	DOWN	yes	gi|114596139|ref|XP_001139435.1|	512	7e-58	PREDICTED: short coiled-coil protein isoform X1 [Pan troglodytes]	gi|942054599|ref|XM_005895817.2|	1892	0	PREDICTED: Bos mutus short coiled-coil protein (SCOC), transcript variant X1, mRNA	Q9UIL1	199	2e-58	Short coiled-coil protein	.	.	.	.	pfam10224	DUF2205	Predicted coiled-coil protein (DUF2205). This entry represent a highly conserved 100 residue region which is likely to be a	GO:0016239|positive regulation of macroautophagy;GO:0061635|regulation of protein complex stability;	GO:0005829|cytosol;GO:0000139|Golgi membrane;	.	.	.
STIP1	2327.836	0.297954	0.092525021	3.220254	0.0012808	0.013837	Not_Sig	no	gi|942050251|ref|XP_005894194.2|	2773	0	PREDICTED: stress-induced-phosphoprotein 1 isoform X2 [Bos mutus]	gi|942050250|ref|XM_005894132.2|	2147	0	PREDICTED: Bos mutus stress-induced phosphoprotein 1 (STIP1), transcript variant X2, mRNA	Q3ZBZ8	1055	0	Stress-induced-phosphoprotein 1	.	.	.	.	pfam13414	TPR_11	TPR repeat.	.	GO:0005737|cytoplasm;GO:0043209|myelin sheath;GO:0005634|nucleus;	GO:0044822|poly(A) RNA binding;	K09553	STIP1; stress-induced-phosphoprotein 1
HECTD4	922.9298	0.382805	0.118883516	3.220004	0.0012819	0.013837	Not_Sig	no	gi|803322583|ref|XP_012019494.1|	21354	0	PREDICTED: probable E3 ubiquitin-protein ligase HECTD4 isoform X2 [Ovis aries musimon]	gi|982998369|ref|XM_010823423.2|	15467	0	PREDICTED: Bos taurus HECT domain containing E3 ubiquitin protein ligase 4 (HECTD4), transcript variant X7, mRNA	Q9Y4D8	7309	0	Probable E3 ubiquitin-protein ligase HECTD4	SPAC11G7.02	76.3	2.35e-12	COG5021	pfam00632	HECT	HECT-domain (ubiquitin-transferase). The name HECT comes from Homologous to the E6-AP Carboxyl Terminus.	GO:0042593|glucose homeostasis;GO:0006006|glucose metabolic process;GO:0042787|protein ubiquitination involved in ubiquitin-dependent protein catabolic process;	GO:0005737|cytoplasm;GO:0016021|integral component of membrane;GO:0005634|nucleus;	GO:0004842|ubiquitin-protein transferase activity;	.	.
KIAA2013	906.8183	0.441752	0.137196909	3.219837	0.0012826	0.013837	Not_Sig	no	gi|114052028|ref|NP_001039407.1|	2846	0	uncharacterized protein KIAA2013 homolog precursor [Bos taurus]	gi|926711248|ref|XM_005690704.2|	2438	0	PREDICTED: Capra hircus KIAA2013 ortholog (KIAA2013), mRNA	Q2KHV9	1100	0	Uncharacterized protein KIAA2013 homolog	.	.	.	.	pfam10222	DUF2152	Uncharacterized conserved protein (DUF2152). This is a family of proteins conserved from worms to humans. Its function is unknown.	.	GO:0016021|integral component of membrane;	.	.	.
FGFR1	3938.004	0.442515	0.13743409	3.219835	0.0012826	0.013837	Not_Sig	no	gi|594094733|ref|XP_006070663.1|	4282	0	PREDICTED: fibroblast growth factor receptor 1 isoform X1 [Bubalus bubalis]	gi|982969447|ref|XM_005226096.3|	3429	0	PREDICTED: Bos taurus fibroblast growth factor receptor 1 (FGFR1), transcript variant X3, mRNA	P11362	1576	0	Fibroblast growth factor receptor 1	.	.	.	.	pfam07714	Pkinase_Tyr	Protein tyrosine kinase.	GO:0001525|angiogenesis;GO:0060117|auditory receptor cell development;GO:0060445|branching involved in salivary gland morphogenesis;GO:0048469|cell maturation;GO:0016477|cell migration;GO:0002062|chondrocyte differentiation;GO:0043009|chordate embryonic development;GO:0030326|embryonic limb morphogenesis;GO:0008543|fibroblast growth factor receptor signaling pathway;GO:0035607|fibroblast growth factor receptor signaling pathway involved in orbitofrontal cortex development;GO:0001701|in utero	GO:0016023|cytoplasmic membrane-bounded vesicle;GO:0005829|cytosol;GO:0005576|extracellular region;GO:0016021|integral	GO:0016303|1-phosphatidylinositol-3-kinase activity;GO:0005524	K04362	FGFR1; fibroblast growth factor receptor 1 [EC:2.7.10.1]
USP33	857.6302	-0.37869	0.117630187	-3.21932	0.001285	0.013837	Not_Sig	no	gi|440905214|gb|ELR55625.1|	4870	0	Ubiquitin carboxyl-terminal hydrolase 33, partial [Bos mutus]	gi|742192924|ref|XM_010857593.1|	3984	0	PREDICTED: Bison bison bison ubiquitin specific peptidase 33 (USP33), transcript variant X1, mRNA	A6QNM7	1849	0	Ubiquitin carboxyl-terminal hydrolase 33	.	.	.	.	pfam00443	UCH	Ubiquitin carboxyl-terminal hydrolase.	GO:0007411|axon guidance;GO:0016477|cell migration;GO:0009267|cellular response to starvation;GO:0051298|centrosome duplication;GO:0006897|endocytosis;GO:0032091|negative regulation of protein binding;GO:0032092|positive regulation of protein binding;GO:0016579|protein deubiquitination;GO:0071108|protein K48-linked deubiquitination;GO:0070536|protein K63-linked deubiquitination;GO:0050821|protein stabilization;GO:0010506|regulation of autophagy;GO:0008277|regulation of G-protein coupled	GO:0005776|autophagosome;GO:0044297|cell body;GO:0005813|centrosome;GO:0005925|focal adhesion;GO:0005794|Golgi	GO:0004197|cysteine-type endopeptidase activity;GO:0004843	K11848	USP20_33; ubiquitin carboxyl-terminal hydrolase 20/33 [EC:3.4.19.12]
IDUA	495.3271	0.592769	0.184129844	3.219296	0.0012851	0.013837	UP	yes	gi|741879515|ref|XP_877410.4|	3068	0	PREDICTED: alpha-L-iduronidase isoform X1 [Bos taurus]	gi|528906328|ref|XM_872317.5|	2114	0	PREDICTED: Bos taurus iduronidase, alpha-L- (IDUA), transcript variant X1, mRNA	P35475	997	0	Alpha-L-iduronidase	BH1068	67	8.29e-11	COG3664	pfam01229	Glyco_hydro_39	Glycosyl hydrolases family 39.	GO:0030207|chondroitin sulfate catabolic process;GO:0030209|dermatan sulfate catabolic process;GO:0005984|disaccharide metabolic process;GO:0006027|glycosaminoglycan catabolic process;	GO:0030135|coated vesicle;GO:0070062|extracellular exosome;GO:0043202|lysosomal lumen;	GO:0003940|L-iduronidase activity;	K01217	IDUA; L-iduronidase [EC:3.2.1.76]
LOC102280361	130.405	-0.83694	0.260003936	-3.21897	0.0012865	0.013837	DOWN	yes	gi|743742979|ref|XP_010966045.1|	846	7e-109	PREDICTED: calmodulin [Camelus bactrianus]	gi|742177375|ref|XM_010853552.1|	1101	0	PREDICTED: Bison bison bison calmodulin-alpha-like (LOC104998408), mRNA	P62155	296	2e-98	Calmodulin	SPAC3A12.14	232	1.84e-75	COG5126	pfam13499	EF-hand_7	EF-hand domain pair.	.	.	GO:0005509|calcium ion binding;GO:0005102|receptor binding;	K02183	CALM; calmodulin
ZNF618	269.3431	0.450751	0.140037416	3.218792	0.0012873	0.013837	Not_Sig	no	gi|528908927|ref|XP_005199354.1|	4973	0	PREDICTED: zinc finger protein 618 isoform X1 [Bos taurus]	gi|742098066|ref|XM_010835192.1|	8240	0	PREDICTED: Bison bison bison zinc finger protein 618 (ZNF618), mRNA	Q5T7W0	1781	0	Zinc finger protein 618	.	.	.	.	pfam09770	PAT1	Topoisomerase II-associated protein PAT1. Members of this family are necessary for accurate chromosome transmission during	GO:0006355|regulation of transcription, DNA-templated;GO:0006351|transcription, DNA-templated;	GO:0005634|nucleus;	GO:0003677|DNA binding;GO:0046872|metal ion binding;	.	.
LOC102265481	376.3181	0.46218	0.14360029	3.21852	0.0012885	0.013837	Not_Sig	no	gi|156120547|ref|NP_001095419.1|	1754	0	aflatoxin B1 aldehyde reductase member 2 [Bos taurus]	gi|156120546|ref|NM_001101949.1|	1276	0	Bos taurus aldo-keto reductase family 7, member A2 (AKR7A2), mRNA	Q8CG76	585	0	Aflatoxin B1 aldehyde reductase member 2	.	.	.	.	pfam00248	Aldo_ket_red	Aldo/keto reductase family. This family includes a number of K+ ion channel beta chain regulatory domains - these are reported to	GO:0044597|daunorubicin metabolic process;GO:0044598|doxorubicin metabolic process;	GO:0070062|extracellular exosome;GO:0005794|Golgi apparatus;GO:0005739|mitochondrion;	GO:0016616|oxidoreductase activity, acting on the CH-OH group of donors,	K15303	AKR7; aflatoxin B1 aldehyde reductase
ELK4	823.4936	-0.45674	0.141925691	-3.21817	0.0012901	0.013837	Not_Sig	no	gi|440893344|gb|ELR46148.1|	1824	0	ETS domain-containing protein Elk-4, partial [Bos mutus]	gi|742179613|ref|XM_010854171.1|	8974	0	PREDICTED: Bison bison bison ELK4, ETS-domain protein (SRF accessory protein 1) (ELK4), mRNA	P28324	618	0	ETS domain-containing protein Elk-4	.	.	.	.	pfam00178	Ets	Ets-domain.	GO:0030154|cell differentiation;GO:0070932|histone H3 deacetylation;GO:0000122|negative regulation of transcription from RNA polymerase II promoter;GO:0006351|transcription, DNA-templated;	GO:0005737|cytoplasm;GO:0005654|nucleoplasm;GO:0005634|nucleus;	GO:0003682|chromatin binding;GO:0001047|core promoter	K04376	ELK4, SAP1; ETS domain-containing protein Elk-4
MTCL2	1308.365	0.343301	0.106680746	3.218021	0.0012908	0.013837	Not_Sig	no	gi|803219698|ref|XP_011970171.1|	6812	0	PREDICTED: protein SOGA1 isoform X2 [Ovis aries musimon]	gi|982993716|ref|XM_005192490.3|	12155	0	PREDICTED: Bos taurus suppressor of glucose, autophagy associated 1 (SOGA1), mRNA	O94964	2309	0	Protein SOGA1	.	.	.	.	pfam14818	DUF4482	Domain of unknown function (DUF4482). This family is found in eukaryotes, and is approximately 140 amino acids in length. The	GO:0008286|insulin receptor signaling pathway;GO:0045721|negative regulation of gluconeogenesis;GO:0010506|regulation of autophagy;	GO:0070062|extracellular exosome;GO:0005615|extracellular space;	.	.	.
C2CD2L	557.1841	0.551339	0.171335039	3.2179	0.0012913	0.013837	Not_Sig	no	gi|157427842|ref|NP_001098828.1|	2952	0	C2 domain-containing protein 2-like [Bos taurus]	gi|982945744|ref|XM_005215936.3|	3384	0	PREDICTED: Bos taurus C2CD2 like (C2CD2L), transcript variant X1, mRNA	O14523	1072	0	C2 domain-containing protein 2-like	.	.	.	.	pfam15070	GOLGA2L5	Putative golgin subfamily A member 2-like protein 5. The function of the GOLGA2L5 protein family remains unknown. This	GO:0035774|positive regulation of insulin secretion involved in cellular response to glucose stimulus;	GO:0016021|integral component of membrane;	.	.	.
POLR2G	407.981	-0.46839	0.145564516	-3.21775	0.001292	0.013837	Not_Sig	no	gi|625226524|ref|XP_003514346.2|	842	2e-110	PREDICTED: DNA-directed RNA polymerase II subunit RPB7 isoform X1 [Cricetulus griseus]	gi|742178964|ref|XM_010853983.1|	818	0	PREDICTED: Bison bison bison polymerase (RNA) II (DNA directed) polypeptide G (POLR2G), transcript variant X1, mRNA	P62489	325	4e-111	DNA-directed RNA polymerase II subunit RPB7	SPACUNK4.06c	170	2.12e-52	COG1095	pfam03876	SHS2_Rpb7-N	SHS2 domain found in N terminus of Rpb7p/Rpc25p/MJ0397. Rpb7 bind to Rpb4 to form a heterodimer. This complex is	GO:0006915|apoptotic process;GO:0000291|nuclear-transcribed mRNA catabolic process, exonucleolytic;GO:0060213|positive regulation of nuclear-transcribed mRNA poly(A) tail shortening;GO:0045948|positive regulation of translational initiation;GO:0006366|transcription from RNA polymerase II promoter;GO:0006367|transcription initiation from RNA polymerase II promoter;	GO:0000932|cytoplasmic mRNA processing body;GO:0005665|DNA-directed RNA polymerase II, core complex;GO:0005634|nucleus;	GO:0003899|DNA-directed RNA polymerase activity;GO:0003697	K03015	RPB7, POLR2G; DNA-directed RNA polymerase II subunit RPB7
SREK1IP1	116.179	-0.65378	0.203181675	-3.2177	0.0012922	0.013837	DOWN	yes	gi|670998015|ref|XP_008689893.1|	400	6e-40	PREDICTED: protein SREK1IP1 [Ursus maritimus]	gi|942046525|ref|XM_005892769.2|	2797	0	PREDICTED: Bos mutus SREK1-interacting protein 1 (SREK1IP1), mRNA	Q5RJP9	107	1e-24	Protein SREK1IP1	.	.	.	.	pfam13917	zf-CCHC_3	Zinc knuckle. The zinc knuckle is a zinc binding motif composed of the the following CX2CX4HX4C where X can be any amino acid. The	GO:0006397|mRNA processing;GO:0008380|RNA splicing;	.	GO:0003676|nucleic acid binding;GO:0008270|zinc ion binding;	.	.
SNX16	171.5597	-0.55188	0.17155174	-3.21697	0.0012955	0.01386	Not_Sig	no	gi|555993943|ref|XP_005908726.1|	1587	0	PREDICTED: sorting nexin-16 [Bos mutus]	gi|942094222|ref|XM_005908664.2|	2286	0	PREDICTED: Bos mutus sorting nexin 16 (SNX16), mRNA	P57768	544	0	Sorting nexin-16	.	.	.	.	pfam00787	PX	PX domain. PX domains bind to phosphoinositides.	GO:0045022|early endosome to late endosome transport;GO:0008333|endosome to lysosome transport;GO:0006622|protein targeting to lysosome;	GO:0005829|cytosol;GO:0005769|early endosome;GO:0031901|early endosome membrane;GO:0031313|extrinsic component of endosome	GO:0042802|identical protein binding;GO:0035091|phosphatidylinositol	K17928	SNX16; sorting nexin-16
MRPL27	366.9994	-0.56267	0.174985957	-3.21549	0.0013022	0.013919	Not_Sig	no	gi|555956472|ref|XP_005890384.1|	703	5e-91	PREDICTED: 39S ribosomal protein L27, mitochondrial isoform X1 [Bos mutus]	gi|742136105|ref|XM_010842791.1|	678	0	PREDICTED: Bison bison bison mitochondrial ribosomal protein L27 (MRPL27), mRNA	Q32PC3	273	1e-91	39S ribosomal protein L27, mitochondrial	RC1163	70.5	2.71e-15	COG0211	pfam01016	Ribosomal_L27	Ribosomal L27 protein.	GO:0070125|mitochondrial translational elongation;GO:0070124|mitochondrial translational initiation;	GO:0005743|mitochondrial inner membrane;GO:0005762|mitochondrial large ribosomal subunit;	GO:0044822|poly(A) RNA binding;GO:0003735|structural	K02899	RP-L27, MRPL27, rpmA; large subunit ribosomal protein L27
FIS1	829.8926	-0.36354	0.113106612	-3.21416	0.0013083	0.01397	Not_Sig	no	gi|671011831|ref|XP_008696744.1|	719	2e-92	PREDICTED: mitochondrial fission 1 protein [Ursus maritimus]	gi|942046704|ref|XM_005892806.2|	773	0	PREDICTED: Bos mutus fission, mitochondrial 1 (FIS1), transcript variant X3, mRNA	Q3T0I5	247	4e-81	Mitochondrial fission 1 protein	.	.	.	.	pfam14853	Fis1_TPR_C	Fis1 C-terminal tetratricopeptide repeat. The mitochondrial fission protein Fis1 consists of two tetratricopeptide repeats. This	GO:0035584|calcium-mediated signaling using intracellular calcium source;GO:0000266|mitochondrial fission;GO:0043653|mitochondrial fragmentation involved in apoptotic process;GO:0008053|mitochondrial fusion;GO:0070584|mitochondrion morphogenesis;GO:0000422|mitophagy;GO:0032471|negative regulation of endoplasmic reticulum calcium ion concentration;GO:0016559|peroxisome fission;GO:0043280|positive regulation of cysteine-type endopeptidase activity involved in apoptotic	GO:0005783|endoplasmic reticulum;GO:0031307|integral component of mitochondrial outer membrane;GO:0005779|integral	.	K17969	FIS1, TTC11, MDV2; mitochondrial fission 1 protein
TM4SF19	212.236	-0.54959	0.171032401	-3.21339	0.0013118	0.013995	Not_Sig	no	gi|927220929|ref|XP_013846583.1|	897	4e-105	PREDICTED: transmembrane 4 L6 family member 19 [Sus scrofa]	gi|850474981|gb|CP011886.1|	3933	0	Ovis canadensis canadensis isolate 43U chromosome 1 sequence	Q96DZ7	274	3e-81	Transmembrane 4 L6 family member 19	.	.	.	.	pfam05805	L6_membrane	L6 membrane protein. This family consists of several eukaryotic L6 membrane proteins. L6, IL-TMP, and TM4SF5 are cell surface	.	GO:0016021|integral component of membrane;	.	.	.
PDIA4	4336.949	0.285555	0.088902086	3.212017	0.0013181	0.014031	Not_Sig	no	gi|296488156|tpg|DAA30269.1|	2976	0	TPA: protein disulfide isomerase A4 precursor [Bos taurus]	gi|594098209|ref|XM_006072215.1|	2376	0	PREDICTED: Bubalus bubalis protein disulfide isomerase family A, member 4 (PDIA4), mRNA	Q29RV1	1147	0	Protein disulfide-isomerase A4	SPAC1F5.02	239	4.43e-69	COG0526	pfam00085	Thioredoxin	Thioredoxin. Thioredoxins are small enzymes that participate in redox reactions, via the reversible oxidation of an active centre	GO:0045454|cell redox homeostasis;GO:0006457|protein folding;GO:0034976|response to endoplasmic reticulum stress;	GO:0005783|endoplasmic reticulum;GO:0005788|endoplasmic reticulum lumen;GO:0042470|melanosome;	GO:0003756|protein disulfide isomerase activity;	K09582	PDIA4, ERP72; protein disulfide-isomerase A4 [EC:5.3.4.1]
DPF2	1319	0.384415	0.11968034	3.212016	0.0013181	0.014031	Not_Sig	no	gi|635012306|ref|XP_007985887.1|	1913	0	PREDICTED: LOW QUALITY PROTEIN: zinc finger protein ubi-d4 [Chlorocebus sabaeus]	gi|742110527|ref|XM_010838051.1|	2474	0	PREDICTED: Bison bison bison D4, zinc and double PHD fingers family 2 (DPF2), transcript variant X1, mRNA	P58268	702	0	Zinc finger protein ubi-d4	.	.	.	.	pfam14051	Requiem_N	N-terminal domain of DPF2/REQ. This putative domain has been detected on the human DPF2 protein and was subsequently	GO:0006915|apoptotic process;GO:0006355|regulation of transcription, DNA-templated;GO:0006351|transcription, DNA-templated;	GO:0005737|cytoplasm;GO:0005634|nucleus;	GO:0008270|zinc ion binding;	K13196	DPF2, REQ; zinc finger protein ubi-d4
ISYNA1	44.53931	0.962944	0.299809436	3.211854	0.0013188	0.014031	UP	yes	gi|594068182|ref|XP_006058022.1|	2879	0	PREDICTED: inositol-3-phosphate synthase 1 [Bubalus bubalis]	gi|594068181|ref|XM_006057960.1|	1854	0	PREDICTED: Bubalus bubalis inositol-3-phosphate synthase 1 (ISYNA1), mRNA	Q2NL29	1112	0	Inositol-3-phosphate synthase 1	YJL153c	520	8.98e-179	COG1260	pfam07994	NAD_binding_5	Myo-inositol-1-phosphate synthase. This is a family of myo-inositol-1-phosphate synthases. Inositol-1-phosphate catalyses the	GO:0006021|inositol biosynthetic process;GO:0008654|phospholipid biosynthetic process;	GO:0005737|cytoplasm;	GO:0004512|inositol-3-phosphate synthase activity;	K01858	INO1, ISYNA1; myo-inositol-1-phosphate synthase [EC:5.5.1.4]
ARHGEF1	976.8604	0.347154	0.10819082	3.208719	0.0013333	0.014171	Not_Sig	no	gi|741894449|ref|XP_010822004.1|	4480	0	PREDICTED: rho guanine nucleotide exchange factor 1 isoform X2 [Bos taurus]	gi|983000054|ref|XM_005195333.3|	3398	0	PREDICTED: Bos taurus Rho guanine nucleotide exchange factor 1 (ARHGEF1), transcript variant X5, mRNA	Q61210	1277	0	Rho guanine nucleotide exchange factor 1	SPCC645.07	78.6	9.33e-14	COG5422	pfam09128	RGS-like	Regulator of G protein signalling-like domain. Members of this family adopt a structure consisting of twelve helices that fold into a	GO:0035023|regulation of Rho protein signal transduction;	GO:0005737|cytoplasm;GO:0005886|plasma membrane;	GO:0005096|GTPase activator activity;GO:0044822|poly(A) RNA	K12330	ARHGEF1; Rho guanine nucleotide exchange factor 1
VARS2	207.0846	0.526073	0.163976283	3.208226	0.0013356	0.014175	Not_Sig	no	gi|555990463|ref|XP_005907025.1|	5045	0	PREDICTED: valine--tRNA ligase, mitochondrial [Bos mutus]	gi|594056336|ref|XM_006052312.1|	4026	0	PREDICTED: Bubalus bubalis valyl-tRNA synthetase 2, mitochondrial (VARS2), transcript variant X1, mRNA	Q767M3	1777	0	Valine--tRNA ligase, mitochondrial	SPBC1709.02c	756	0	COG0525	pfam00133	tRNA-synt_1	tRNA synthetases class I (I, L, M and V). Other tRNA synthetase sub-families are too dissimilar to be included.	GO:0006438|valyl-tRNA aminoacylation;	GO:0005739|mitochondrion;	GO:0002161|aminoacyl-tRNA editing activity;GO:0005524|ATP	K01873	VARS, valS; valyl-tRNA synthetase [EC:6.1.1.9]
RAB7A	4734.376	0.35937	0.112019314	3.208111	0.0013361	0.014175	Not_Sig	no	gi|529002406|ref|XP_005223227.1|	1110	6e-145	PREDICTED: ras-related protein Rab-7a isoform X1 [Bos taurus]	gi|741969890|ref|XM_005223170.2|	1979	0	PREDICTED: Bos taurus RAB7A, member RAS oncogene family (RAB7A), transcript variant X1, mRNA	Q3T0F5	430	1e-145	Ras-related protein Rab-7a	.	.	.	.	pfam00071	Ras	Ras family. Includes sub-families Ras, Rab, Rac, Ral, Ran, Rap Ypt1 and more. Shares P-loop motif with GTP_EFTU, arf and	GO:0045022|early endosome to late endosome transport;GO:0008333|endosome to lysosome transport;GO:0061724|lipophagy;GO:0090383|phagosome acidification;GO:0090385|phagosome-lysosome fusion;GO:0015031|protein transport;GO:0007264|small GTPase mediated signal transduction;	GO:0000421|autophagosome membrane;GO:0005829|cytosol;GO:0032419|extrinsic component of lysosome membrane;GO:0005770|late	GO:0005525|GTP binding;	K07897	RAB7A; Ras-related protein Rab-7A
PAXIP1	236.0509	0.461799	0.143995662	3.207036	0.0013411	0.014215	Not_Sig	no	gi|118151388|ref|NP_001071385.1|	2497	0	PAX-interacting protein 1 [Bos taurus]	gi|118151387|ref|NM_001077917.1|	3014	0	Bos taurus PAX interacting (with transcription-activation domain) protein 1 (PAXIP1), mRNA	A0JNA8	964	0	PAX-interacting protein 1	.	.	.	.	pfam12738	PTCB-BRCT	twin BRCT domain. This is a BRCT domain that appears in duplicate in most member sequences. BRCT domains are peptide- and	GO:0006310|DNA recombination;GO:0006281|DNA repair;GO:0006355|regulation of transcription, DNA-templated;GO:0006351|transcription, DNA-templated;	GO:0016363|nuclear matrix;	.	K14972	PAXIP1, PTIP; PAX-interacting protein 1
B4GALT6	77.14739	-0.70684	0.220436914	-3.20655	0.0013434	0.014226	DOWN	yes	gi|586530306|ref|XP_006920064.1|	1988	0	PREDICTED: beta-1,4-galactosyltransferase 6 isoform X1 [Pteropus alecto]	gi|982965138|ref|XM_010818685.2|	4511	0	PREDICTED: Bos taurus UDP-Gal:betaGlcNAc beta 1,4- galactosyltransferase, polypeptide 6 (B4GALT6), transcript variant X1, mRNA	Q9UBX8	744	0	Beta-1,4-galactosyltransferase 6	.	.	.	.	pfam13733	Glyco_transf_7N	N-terminal region of glycosyl transferase group 7. This is the N-terminal half of a family of galactosyltransferases from a wide	GO:0018146|keratan sulfate biosynthetic process;GO:0006486|protein glycosylation;	GO:0032580|Golgi cisterna membrane;GO:0000139|Golgi membrane;GO:0016021|integral component of membrane;	GO:0003831|beta-N-acetylglucosaminylglycopeptide beta-	K07553	B4GALT6; beta-1,4-galactosyltransferase 6 [EC:2.4.1.274]
TMEM128	364.5845	-0.53024	0.16546653	-3.20453	0.0013528	0.014313	Not_Sig	no	gi|528953832|ref|XP_005208305.1|	1104	1e-147	PREDICTED: transmembrane protein 128 isoform X1 [Bos taurus]	gi|982928878|ref|XM_005208248.3|	1290	0	PREDICTED: Bos taurus transmembrane protein 128 (TMEM128), transcript variant X1, mRNA	Q3T0S0	316	3e-105	Transmembrane protein 128	.	.	.	.	pfam02544	Steroid_dh	3-oxo-5-alpha-steroid 4-dehydrogenase. This family consists of 3-oxo-5-alpha-steroid 4-dehydrogenases, EC:1.3.99.5	.	GO:0016021|integral component of membrane;	.	.	.
PPP2R1A	2598.754	0.367625	0.114735601	3.204103	0.0013548	0.014321	Not_Sig	no	gi|478529814|ref|XP_004439075.1|	3117	0	PREDICTED: serine/threonine-protein phosphatase 2A 65 kDa regulatory subunit A alpha isoform [Ceratotherium simum simum]	gi|742235216|ref|XM_010831528.1|	2248	0	PREDICTED: Bison bison bison protein phosphatase 2, regulatory subunit A, alpha (PPP2R1A), transcript variant X1, mRNA	Q32PI5	1201	0	Serine/threonine-protein phosphatase 2A 65 kDa regulatory subunit A alpha isoform	.	.	.	.	pfam13513	HEAT_EZ	HEAT-like repeat. The HEAT repeat family is related to armadillo/beta-catenin-like repeats (see pfam00514). These EZ repeats are	GO:0007059|chromosome segregation;GO:0006461|protein complex assembly;	GO:0000775|chromosome, centromeric region;GO:0005737|cytoplasm;	GO:0008601|protein phosphatase type 2A regulator activity;	K03456	PPP2R1; serine/threonine-protein phosphatase 2A regulatory
TFAP4	61.03792	0.864305	0.269945747	3.201771	0.0013659	0.014403	UP	yes	gi|155372151|ref|NP_001094685.1|	1194	8e-155	transcription factor AP-4 [Bos taurus]	gi|982966235|ref|XM_005224545.3|	2089	0	PREDICTED: Bos taurus transcription factor AP-4 (activating enhancer binding protein 4) (TFAP4), transcript variant X1, mRNA	Q01664	441	3e-147	Transcription factor AP-4	.	.	.	.	pfam00010	HLH	Helix-loop-helix DNA-binding domain.	GO:0071549|cellular response to dexamethasone stimulus;GO:0006978|DNA damage response, signal transduction by p53 class mediator resulting in transcription of p21 class mediator;GO:0043922|negative regulation by host of viral transcription;GO:0071157|negative regulation of cell cycle arrest;GO:0008285|negative regulation of cell proliferation;GO:0045736|negative regulation of cyclin-dependent protein serine/threonine kinase activity;GO:0043392|negative regulation of DNA binding;GO:0045892|negative regulation of transcription, DNA-	GO:0005634|nucleus;GO:0017053|transcriptional repressor complex;	GO:0003677|DNA binding;GO:0070888|E-box binding;GO:0042826	K09108	TFAP4; transcription factor AP-4
NAPA	875.6125	0.435461	0.136011856	3.201637	0.0013665	0.014403	Not_Sig	no	gi|593712552|ref|XP_007102809.1|	1426	0	PREDICTED: alpha-soluble NSF attachment protein [Physeter catodon]	gi|555978079|ref|XM_005900925.1|	1710	0	PREDICTED: Bos mutus N-ethylmaleimide-sensitive factor attachment protein, alpha (NAPA), transcript variant X2, mRNA	Q9DB05	541	0	Alpha-soluble NSF attachment protein	.	.	.	.	pfam14938	SNAP	Soluble NSF attachment protein, SNAP. The soluble NSF attachment protein (SNAP) proteins are involved in vesicular transport	GO:0045176|apical protein localization;GO:0007420|brain development;GO:0006886|intracellular protein transport;GO:0061025|membrane fusion;GO:0030182|neuron differentiation;GO:0010807|regulation of synaptic vesicle priming;GO:0035494|SNARE complex disassembly;GO:0035249|synaptic transmission, glutamatergic;	GO:0070062|extracellular exosome;GO:0016020|membrane;GO:0043209|myelin sheath;GO:0070044|synaptobrevin 2-SNAP-	GO:0000149|SNARE binding;GO:0005483|soluble NSF attachment protein	K15296	NAPA, SNAPA, SEC17; alpha-soluble NSF attachment protein
UBE2W	628.7639	-0.47258	0.147616366	-3.20141	0.0013676	0.014403	Not_Sig	no	gi|545225777|ref|XP_001491727.3|	881	4e-104	PREDICTED: ubiquitin-conjugating enzyme E2 W [Equus caballus]	gi|942090424|ref|XM_005907512.2|	4078	0	PREDICTED: Bos mutus ubiquitin-conjugating enzyme E2W (putative) (UBE2W), mRNA	B5DEI4	318	2e-97	Ubiquitin-conjugating enzyme E2 W	YBR082c	80.5	1.94e-16	COG5078	pfam00179	UQ_con	Ubiquitin-conjugating enzyme. Proteins destined for proteasome-mediated degradation may be ubiquitinated. Ubiquitination	GO:0071218|cellular response to misfolded protein;GO:0006281|DNA repair;GO:0006515|misfolded or incompletely synthesized protein catabolic process;GO:0043161|proteasome-mediated ubiquitin-dependent protein catabolic process;GO:0006513|protein monoubiquitination;GO:0000209|protein polyubiquitination;	GO:0005737|cytoplasm;GO:0005634|nucleus;	GO:0005524|ATP binding;GO:0061630|ubiquitin protein ligase	K10688	UBE2W, UBC16; ubiquitin-conjugating enzyme E2 W [EC:2.3.2.25]
NOL6	462.9757	0.437265	0.13658538	3.201401	0.0013676	0.014403	Not_Sig	no	gi|555982357|ref|XP_005903085.1|	5514	0	PREDICTED: nucleolar protein 6 isoform X1 [Bos mutus]	gi|555982356|ref|XM_005903023.1|	4534	0	PREDICTED: Bos mutus nucleolar protein 6 (RNA-associated) (NOL6), transcript variant X1, mRNA	Q9H6R4	1936	0	Nucleolar protein 6	.	.	.	.	pfam03813	Nrap	Nrap protein. Members of this family are nucleolar RNA-associated proteins (Nrap) which are highly conserved from yeast	GO:0006364|rRNA processing;GO:0006409|tRNA export from nucleus;	GO:0000794|condensed nuclear chromosome;GO:0032545|CURI complex;GO:0005739|mitochondrion;GO:0005730|nucleolus;GO:0005654|nucleoplasm;	GO:0044822|poly(A) RNA binding;	K14544	UTP22, NOL6; U3 small nucleolar RNA-associated protein 22

HIP1R	385.0392	0.415365	0.129764206	3.20092	0.0013699	0.014414	Not_Sig	no	gi|358416320|ref|XP_585048.5|	5233	0	PREDICTED: huntingtin-interacting protein 1-related protein isoform X1 [Bos taurus]	gi|982998140|ref|XM_585048.8|	3492	0	PREDICTED: Bos taurus huntingtin interacting protein 1 related (HIP1R), transcript variant X1, mRNA	O75146	1865	0	Huntingtin-interacting protein 1-related protein	.	.	.	.	pfam07651	ANTH	ANTH domain. AP180 is an endocytotic accessory proteins that has been implicated in the formation of clathrin-coated pits.	GO:0006915|apoptotic process;GO:0072583|clathrin-mediated endocytosis;GO:0006898|receptor-mediated endocytosis;	GO:0005905|coated pit;GO:0030136|clathrin-coated vesicle;GO:0030665|clathrin-coated vesicle membrane;GO:0005737|cytoplasm;GO:000	GO:0035091|phosphatidylinositol binding;	.	.
NDUFA8	509.2181	-0.37328	0.116651153	-3.2	0.0013743	0.014441	Not_Sig	no	gi|28461275|ref|NP_787020.1|	925	1e-123	NADH dehydrogenase [ubiquinone] 1 alpha subcomplex subunit 8 [Bos taurus]	gi|555966154|ref|XM_005895071.1|	797	0	PREDICTED: Bos mutus NADH dehydrogenase (ubiquinone) 1 alpha subcomplex, 8, 19kDa (NDUFA8), mRNA	P42029	360	4e-125	NADH dehydrogenase [ubiquinone] 1 alpha subcomplex subunit 8	.	.	.	.	pfam06747	CHCH	CHCH domain. we have identified a conserved motif in the LOC118487 protein that we have called the CHCH motif. Alignment of this	.	GO:0005758|mitochondrial intermembrane space;GO:0005747|mitochondrial respiratory chain complex I;	GO:0008137|NADH dehydrogenase (ubiquinone) activity;	K03952	NDUFA8; NADH dehydrogenase (ubiquinone) 1 alpha subcomplex subunit
PPARD	463.4951	0.541045	0.169084534	3.199846	0.001375	0.014441	Not_Sig	no	gi|803213942|ref|XP_011967360.1|	2265	0	PREDICTED: peroxisome proliferator-activated receptor delta isoform X2 [Ovis aries musimon]	gi|139948624|ref|NM_001083636.1|	3159	0	Bos taurus peroxisome proliferator-activated receptor delta (PPARD), mRNA	Q03181	808	0	Peroxisome proliferator-activated receptor delta	.	.	.	.	pfam00105	zf-C4	Zinc finger, C4 type (two domains). In nearly all cases, this is the DNA binding domain of a nuclear hormone receptor. The alignment	GO:0060612|adipose tissue development;GO:0006915|apoptotic process;GO:0097190|apoptotic signaling pathway;GO:0008366|axon ensheathment;GO:0030154|cell differentiation;GO:0008283|cell proliferation;GO:0031589|cell-substrate adhesion;GO:0071456|cellular response to hypoxia;GO:0071222|cellular response to lipopolysaccharide;GO:0008203|cholesterol metabolic process;GO:0046697|decidualization;GO:0007566|embryo implantation;GO:0006635|fatty acid beta-oxidation;GO:0009062|fatty acid	GO:0000790|nuclear chromatin;GO:0005654|nucleoplasm;GO:0005634|nucleus;	GO:0003677|DNA binding;GO:0008144|drug binding;GO:0070539	K04504	NR1C2, PPARD; peroxisome proliferator-activated receptor
CMIP	727.0972	0.454949	0.142198156	3.199402	0.0013771	0.01445	Not_Sig	no	gi|594039922|ref|XP_006044538.1|	4070	0	PREDICTED: c-Maf-inducing protein [Bubalus bubalis]	gi|965908515|ref|XM_012168385.2|	3976	0	PREDICTED: Ovis aries musimon c-Maf inducing protein (CMIP), transcript variant X2, mRNA	Q8IY22	1550	0	C-Maf-inducing protein	.	.	.	.	pfam00169	PH	PH domain. PH stands for pleckstrin homology.	.	GO:0005737|cytoplasm;GO:0005634|nucleus;	.	.	.
MANF	1819.018	-0.422	0.131920286	-3.19893	0.0013794	0.014461	Not_Sig	no	gi|155372143|ref|NP_001094681.1|	801	1e-104	mesencephalic astrocyte-derived neurotrophic factor precursor [Bos taurus]	gi|742171270|ref|XM_010851791.1|	851	0	PREDICTED: Bison bison bison mesencephalic astrocyte-derived neurotrophic factor (MANF), mRNA	P80513	313	4e-106	Mesencephalic astrocyte-derived neurotrophic factor	.	.	.	.	pfam10208	Armet	Degradation arginine-rich protein for mis-folding. This is a family of small proteins of approximately 170 residues which contain four di-	GO:0071542|dopaminergic neuron differentiation;GO:0031175|neuron projection development;GO:0006986|response to unfolded protein;	GO:0005783|endoplasmic reticulum;GO:0005615|extracellular space;	.	.	.
SUGT1	889.0813	-0.40367	0.126208858	-3.19842	0.0013818	0.01447	Not_Sig	no	gi|555984003|ref|XP_005903895.1|	1680	0	PREDICTED: protein SGT1 homolog isoform X1 [Bos mutus]	gi|850486327|gb|CP011895.1|	4018	0	Ovis canadensis canadensis isolate 43U chromosome 10 sequence	Q2KIK0	645	0	Protein SGT1 homolog	SPBC36.12c	116	3.52e-26	COG5091	pfam05002	SGS	SGS domain. This domain was thought to be unique to the SGT1-like proteins, but is also found in calcyclin binding proteins.	.	GO:0005737|cytoplasm;GO:0005634|nucleus;	.	K12795	SUGT1, SGT1; suppressor of G2 allele of SKP1
TAF6	462.1562	0.49091	0.153494659	3.198225	0.0013828	0.01447	Not_Sig	no	gi|982967164|ref|XP_015315899.1|	2781	0	PREDICTED: transcription initiation factor TFIID subunit 6 isoform X1 [Bos taurus]	gi|742143045|ref|XM_010844757.1|	2519	0	PREDICTED: Bison bison bison TAF6 RNA polymerase II, TATA box binding protein (TBP)-associated factor, 80kDa (TAF6), transcript variant X2, mRNA	P49848	1027	0	Transcription initiation factor TFIID subunit 6	SPCC16C4.18c	257	2.24e-76	COG5095	pfam07571	DUF1546	Protein of unknown function (DUF1546). Associated with pfam02969 in Transcription initiation factor TFIID subunit 6	GO:0006352|DNA-templated transcription, initiation;GO:0045786|negative regulation of cell cycle;GO:0008285|negative regulation of cell proliferation;GO:0051090|regulation of sequence-specific DNA binding transcription factor activity;GO:1901796|regulation of signal transduction by p53 class mediator;GO:0042795|snRNA transcription from RNA polymerase II promoter;GO:0006368|transcription elongation from RNA polymerase II promoter;GO:0006366|transcription from RNA polymerase II promoter;GO:0006367|transcription initiation from RNA	GO:0005737|cytoplasm;GO:0071339|MLL1 complex;GO:0005654|nucleoplasm;GO:0005634|nucleus;GO:0005669|transcription factor TFIID	GO:0003677|DNA binding;GO:0003700|transcription factor activity, sequence-	K03131	TAF6; transcription initiation factor TFIID subunit 6
FAM222B	374.2371	0.481547	0.150618139	3.197138	0.001388	0.014511	Not_Sig	no	gi|555952139|ref|XP_005888259.1|	2641	0	PREDICTED: protein FAM222B [Bos mutus]	gi|983001418|ref|XM_005195598.2|	4159	0	PREDICTED: Bos taurus family with sequence similarity 222 member B (FAM222B), transcript variant X4, mRNA	Q8WU58	967	0	Protein FAM222B	.	.	.	.	pfam15258	FAM222A	Protein family of FAM222A. This protein family, FAM222A are a domain of unknown function. This family of proteins is found in	.	GO:0005654|nucleoplasm;	.	.	.
PRR14	188.3969	0.564068	0.176454687	3.196671	0.0013902	0.014522	Not_Sig	no	gi|529006998|ref|XP_005224977.1|	2189	0	PREDICTED: proline-rich protein 14 isoform X1 [Bos taurus]	gi|965906537|ref|XM_012166136.2|	3032	0	PREDICTED: Ovis aries musimon proline rich 14 (PRR14), transcript variant X2, mRNA	Q0VBZ8	704	0	Proline-rich protein 14	.	.	.	.	pfam15386	Tantalus	Drosophila Tantalus-like. An alpha+beta fold domain found in metazoan proteins such as Drosophila Tantalus. Drosophila	.	.	.	.	.
HNRNPA0	1102.587	-0.3691	0.115476895	-3.19628	0.0013921	0.014528	Not_Sig	no	gi|743750334|ref|XP_010969964.1|	887	4e-108	PREDICTED: heterogeneous nuclear ribonucleoprotein A0, partial [Camelus bactrianus]	gi|982985377|ref|XM_593096.6|	2768	0	PREDICTED: Bos taurus heterogeneous nuclear ribonucleoprotein A0 (HNRNPA0), mRNA	Q13151	344	2e-108	Heterogeneous nuclear ribonucleoprotein A0	.	.	.	.	pfam00076	RRM_1	RNA recognition motif. (a.k.a. RRM, RBD, or RNP domain). The RRM motif is probably diagnostic of an RNA binding protein. RRMs	GO:0070935|3'-UTR-mediated mRNA stabilization;GO:0010467|gene expression;GO:0006954|inflammatory response;GO:0006397|mRNA processing;GO:0000398|mRNA splicing, via spliceosome;GO:0032496|response to lipopolysaccharide;	GO:0030529|ribonucleoprotein complex;GO:0005654|nucleoplasm;GO:0005634|nucleus;	GO:0017091|AU-rich element binding;GO:0000166|nucleotide	K12894	HNRNPA0; heterogeneous nuclear ribonucleoprotein
FAM91A1	2012.491	-0.36656	0.11472062	-3.19526	0.001397	0.014566	Not_Sig	no	gi|440909147|gb|ELR59090.1|	4519	0	Protein FAM91A1, partial [Bos mutus]	gi|982994352|ref|XM_583552.9|	5411	0	PREDICTED: Bos taurus family with sequence similarity 91 member A1 (FAM91A1), transcript variant X1, mRNA	Q658Y4	1665	0	Protein FAM91A1	.	.	.	.	pfam14648	FAM91_C	FAM91 C-terminus.	.	.	.	.	.
CFB	554.4511	0.435956	0.13646416	3.194654	0.0014	0.014584	Not_Sig	no	gi|95147674|ref|NP_001035616.1|	3992	0	complement factor B precursor [Bos taurus]	gi|742090404|ref|XM_010833346.1|	2461	0	PREDICTED: Bison bison bison complement factor B (CFB), mRNA	P81187	1542	0	Complement factor B	.	.	.	.	pfam00089	Trypsin	Trypsin.	GO:0006957|complement activation, alternative pathway;	GO:0005576|extracellular region;	GO:0004252|serine-type endopeptidase activity;	K01335	CFB; component factor B [EC:3.4.21.47]
ABRACL	340.3098	-0.47396	0.148388147	-3.19406	0.0014029	0.0146	Not_Sig	no	gi|829975883|ref|XP_012605976.1|	464	1e-53	PREDICTED: uncharacterized protein LOC105863453 isoform X1 [Microcebus murinus]	gi|942043974|ref|XM_005891957.2|	889	0	PREDICTED: Bos mutus ABRA C-terminal like (ABRACL), mRNA	Q3ZBN0	162	2e-48	Costars family protein ABRACL	.	.	.	.	pfam14705	Costars	Costars. This domain is found both alone and at the C-terminus of actin-binding Rho-activating protein (ABRA). It binds to actin,	.	.	.	.	.
SLU7	830.3699	-0.37631	0.117850971	-3.19309	0.0014076	0.014627	Not_Sig	no	gi|670999493|ref|XP_008690641.1|	2657	0	PREDICTED: pre-mRNA-splicing factor SLU7 [Ursus maritimus]	gi|942059120|ref|XM_005897507.2|	3254	0	PREDICTED: Bos mutus SLU7 homolog, splicing factor (SLU7), mRNA	Q3ZBE5	1018	0	Pre-mRNA-splicing factor SLU7	.	.	.	.	pfam11708	Slu7	Pre-mRNA splicing Prp18-interacting factor. The spliceosome, an assembly of snRNAs (U1, U2, U4/U6, and U5)	GO:0000380|alternative mRNA splicing, via spliceosome;GO:0034605|cellular response to heat;GO:0006886|intracellular protein transport;GO:0000389|mRNA 3'-splice site recognition;	GO:0071013|catalytic step 2 spliceosome;GO:0005737|cytoplasm;GO:0016020|membrane;GO:0016607|nuclear speck;GO:0005634|nucleus;GO:0030532|s	GO:0030628|pre-mRNA 3'-splice site binding;GO:0000386|second	K12819	SLU7; pre-mRNA-processing factor SLU7
UBL3	803.2875	-0.36369	0.113902353	-3.19302	0.0014079	0.014627	Not_Sig	no	gi|664700892|ref|XP_008515626.1|	659	6e-76	PREDICTED: ubiquitin-like protein 3 [Equus przewalskii]	gi|742101113|ref|XM_010835894.1|	3835	0	PREDICTED: Bison bison bison ubiquitin-like 3 (UBL3), transcript variant X2, mRNA	O95164	246	1e-73	Ubiquitin-like protein 3	.	.	.	.	pfam13881	Rad60-SLD_2	Ubiquitin-2 like Rad60 SUMO-like.	.	GO:0070062|extracellular exosome;GO:0005622|intracellular;GO:0005886|plasma membrane;	.	.	.
PPM1H	396.1414	-0.41985	0.131507803	-3.19261	0.0014099	0.014634	Not_Sig	no	gi|300796079|ref|NP_001179978.1|	2605	0	protein phosphatase 1H [Bos taurus]	gi|982925936|ref|XR_234571.3|	1870	0	PREDICTED: Bos taurus protein phosphatase, Mg2+/Mn2+ dependent 1H (PPM1H), transcript variant X1, misc_RNA	Q9ULR3	986	0	Protein phosphatase 1H	SPAC2G11.07c	53.1	1.74e-06	COG0631	pfam00481	PP2C	Protein phosphatase 2C. Protein phosphatase 2C is a Mn++ or Mg++ dependent protein serine/threonine phosphatase.	.	GO:0005737|cytoplasm;GO:0005634|nucleus;	GO:0004721|phosphoprotein phosphatase activity;GO:0004722	K17503	PPM1H, ARHCL1; protein phosphatase 1H [EC:3.1.3.16]
MYCBP	352.786	-0.46123	0.144523539	-3.1914	0.0014159	0.014683	Not_Sig	no	gi|148698421|gb|EDL30368.1|	378	6e-39	c-myc binding protein, isoform CRA_a, partial [Mus musculus]	gi|850474981|gb|CP011886.1|	1062	0	Ovis canadensis canadensis isolate 43U chromosome 1 sequence	Q2TBP7	145	9e-40	C-Myc-binding protein	.	.	.	.	pfam07218	RAP1	Rhoptry-associated protein 1 (RAP-1). This family consists of several rhoptry-associated protein 1 (RAP-1) sequences which appear to be	GO:0006355|regulation of transcription, DNA-templated;GO:0006351|transcription, DNA-templated;	GO:0005737|cytoplasm;GO:0005634|nucleus;	GO:0003713|transcription coactivator activity;	.	.
ATP6V1C1	2399.114	-0.33061	0.103643463	-3.18988	0.0014233	0.014746	Not_Sig	no	gi|28603816|ref|NP_788849.1|	1965	0	V-type proton ATPase subunit C 1 [Bos taurus]	gi|742217084|ref|XM_010863496.1|	2141	0	PREDICTED: Bison bison bison ATPase, H+ transporting, lysosomal 42kDa, V1 subunit C1 (ATP6V1C1), mRNA	P21282	761	0	V-type proton ATPase subunit C 1	YKL080w	225	4.74e-66	COG5127	pfam03223	V-ATPase_C	V-ATPase subunit C.	GO:0015991|ATP hydrolysis coupled proton transport;	GO:0070062|extracellular exosome;GO:0005765|lysosomal membrane;GO:0005886|plasma membrane;GO:0033180|proton-	GO:0008553|hydrogen-exporting ATPase activity, phosphorylative	K02148	ATPeV1C, ATP6C; V-type H+-transporting ATPase subunit C
MEX3D	1030.383	0.435908	0.136665304	3.189599	0.0014247	0.014748	Not_Sig	no	gi|594052658|ref|XP_006050640.1|	1856	0	PREDICTED: RNA-binding protein MEX3D, partial [Bubalus bubalis]	gi|982985279|ref|XM_015464259.1|	2164	0	PREDICTED: Bos taurus mex-3 RNA binding family member D (MEX3D), partial mRNA	Q86XN8	649	0	RNA-binding protein MEX3D	.	.	.	.	pfam00013	KH_1	KH domain. KH motifs bind RNA in vitro. Autoantibodies to Nova, a KH domain protein, cause paraneoplastic opsoclonus ataxia.	GO:0061157|mRNA destabilization;GO:0010609|mRNA localization resulting in posttranscriptional regulation of gene expression;	GO:0005634|nucleus;GO:0048471|perinuclear region of cytoplasm;	GO:0017091|AU-rich element binding;GO:0044822|poly(A) RNA	K15686	MEX3, RKHD; RNA-binding protein MEX3 [EC:2.3.2.27]
TMEM26	58.29441	-0.81117	0.254428556	-3.18822	0.0014315	0.014805	DOWN	yes	gi|555971713|ref|XP_005897865.1|	1705	0	PREDICTED: transmembrane protein 26 [Bos mutus]	gi|742192225|ref|XM_010857419.1|	4993	0	PREDICTED: Bison bison bison transmembrane protein 26 (TMEM26), transcript variant X2, mRNA	Q6ZUK4	550	5e-179	Transmembrane protein 26	.	.	.	.	pfam09772	Tmem26	Transmembrane protein 26. The function of this family of transmembrane proteins has not, as yet, been determined.	.	GO:0016021|integral component of membrane;	.	.	.
TRIM47	291.4053	0.45482	0.142722343	3.18675	0.0014388	0.014867	Not_Sig	no	gi|803209273|ref|XP_011965116.1|	2406	0	PREDICTED: tripartite motif-containing protein 47 isoform X5 [Ovis aries musimon]	gi|741964832|ref|XM_005221278.2|	2195	0	PREDICTED: Bos taurus tripartite motif containing 47 (TRIM47), transcript variant X3, mRNA	Q96LD4	889	0	Tripartite motif-containing protein 47	.	.	.	.	pfam00643	zf-B_box	B-box zinc finger.	.	GO:0005737|cytoplasm;GO:0005634|nucleus;	GO:0008270|zinc ion binding;	K12023	TRIM47; tripartite motif-containing protein 47
APOA1	344.4349	-0.4794	0.150465408	-3.18612	0.0014419	0.014886	Not_Sig	no	gi|75832056|ref|NP_776667.2|	1290	1e-176	apolipoprotein A-I preproprotein [Bos taurus]	gi|803225469|ref|XM_012117653.1|	966	0	PREDICTED: Ovis aries musimon apolipoprotein A-I (LOC101117785), transcript variant X2, mRNA	P15497	501	4e-178	Apolipoprotein A-I	.	.	.	.	pfam01442	Apolipoprotein	Apolipoprotein A1/A4/E domain. These proteins contain several 22 residue repeats which form a pair of alpha helices. This family	GO:0030325|adrenal gland development;GO:0043534|blood vessel endothelial cell migration;GO:0006695|cholesterol biosynthetic process;GO:0033344|cholesterol efflux;GO:0042632|cholesterol homeostasis;GO:0070508|cholesterol import;GO:0001935|endothelial cell proliferation;GO:0007186|G-protein coupled receptor signaling pathway;GO:0008211|glucocorticoid metabolic process;GO:0034380|high-density lipoprotein particle assembly;GO:0034375|high-density lipoprotein particle remodeling;GO:0007229|integrin-mediated signaling	GO:0072562|blood microparticle;GO:0042627|chylomicron;GO:0030139|endocytic vesicle;GO:0070062|extracellular	GO:0001540|beta-amyloid binding;GO:0045499|chemorepellent	K08757	APOA1; apolipoprotein A-I
PCMT1	1632.707	-0.44832	0.140742999	-3.18539	0.0014456	0.014905	Not_Sig	no	gi|926701113|ref|XP_013822052.1|	1639	0	PREDICTED: protein-L-isoaspartate(D-aspartate) O-methyltransferase [Capra hircus]	gi|528961265|ref|XM_005211039.1|	1988	0	PREDICTED: Bos taurus protein-L-isoaspartate (D-aspartate) O-methyltransferase (PCMT1), transcript variant X1, mRNA	P80895	462	4e-158	Protein-L-isoaspartate(D-aspartate) O-methyltransferase	SPAC869.08	190	2.32e-55	COG2518	pfam01135	PCMT	Protein-L-isoaspartate(D-aspartate) O-methyltransferase (PCMT).	.	GO:0005737|cytoplasm;	GO:0004719|protein-L-isoaspartate (D-aspartate) O-methyltransferase	K00573	E2.1.1.77, pcm; protein-L-isoaspartate(D-aspartate) O-
SLC12A7	370.0549	0.446927	0.140312175	3.185233	0.0014464	0.014905	Not_Sig	no	gi|803240957|ref|XP_011980575.1|	5369	0	PREDICTED: solute carrier family 12 member 7 isoform X1 [Ovis aries musimon]	gi|983003327|ref|XM_015468402.1|	4175	0	PREDICTED: Bos taurus solute carrier family 12 (potassium/chloride transporter), member 7 (SLC12A7), transcript variant X2, mRNA	Q5RK27	1860	0	Solute carrier family 12 member 7	SPBC18H10.16	166	1.46e-40	COG0531	pfam00324	AA_permease	Amino acid permease.	GO:0006884|cell volume homeostasis;GO:0007268|synaptic transmission;	GO:0005887|integral component of plasma membrane;	GO:0022820|potassium ion symporter activity;GO:0015379|potassium:chloride	K13627	SLC12A7, KCC4; solute carrier family 12 (potassium/chloride
NCOR1	2874.556	0.356349	0.111935292	3.183531	0.0014549	0.014975	Not_Sig	no	gi|555950231|ref|XP_005887319.1|	10663	0	PREDICTED: nuclear receptor corepressor 1 isoform X1 [Bos mutus]	gi|942030925|ref|XM_005887257.2|	8555	0	PREDICTED: Bos mutus nuclear receptor corepressor 1 (NCOR1), transcript variant X1, mRNA	O75376	3732	0	Nuclear receptor corepressor 1	.	.	.	.	pfam00249	Myb_DNA-binding	Myb-like DNA-binding domain. This family contains the DNA binding domains from Myb proteins, as well as the SANT	GO:0044255|cellular lipid metabolic process;GO:0016569|covalent chromatin modification;GO:0007623|circadian rhythm;GO:0046329|negative regulation of JNK cascade;GO:1903799|negative regulation of production of miRNAs involved in gene silencing by miRNA;GO:0000122|negative regulation of transcription from RNA polymerase II promoter;GO:2000191|regulation of fatty acid transport;GO:0072362|regulation of glycolytic process by negative regulation of transcription from RNA polymerase II	GO:0016020|membrane;GO:0000790|nuclear chromatin;GO:0005654|nucleoplasm;GO:0005634|nucleus;GO:0005876|spindle	GO:0042826|histone deacetylase binding;GO:0016922|ligand-dependent	K04650	NCOR1, N-CoR; nuclear receptor co-repressor 1
CASD1	272.256	-0.47867	0.150365988	-3.18336	0.0014558	0.014975	Not_Sig	no	gi|528903073|ref|XP_005198057.1|	4138	0	PREDICTED: CAS1 domain-containing protein 1 isoform X1 [Bos taurus]	gi|982980144|ref|XM_005198000.3|	4121	0	PREDICTED: Bos taurus CAS1 domain containing 1 (CASD1), transcript variant X1, mRNA	Q96PB1	1467	0	CAS1 domain-containing protein 1	.	.	.	.	pfam07779	Cas1_AcylT	10 TM Acyl Transferase domain found in Cas1p. Cas1p protein of Cryptococcus neoformans is required for the synthesis of O-	.	GO:0016021|integral component of membrane;	.	.	.
CMC1	163.0233	-0.63291	0.198850011	-3.18288	0.0014582	0.014986	DOWN	yes	gi|115497522|ref|NP_001070016.1|	555	1e-69	COX assembly mitochondrial protein homolog [Bos taurus]	gi|926720172|ref|XM_005695504.2|	557	0	PREDICTED: Capra hircus C-x(9)-C motif containing 1 (CMC1), mRNA	Q3SZM6	218	4e-71	COX assembly mitochondrial protein homolog	.	.	.	.	pfam08583	Cmc1	Cytochrome c oxidase biogenesis protein Cmc1 like. Cmc1 is a metallo-chaperone like protein which is known to localize to the	.	GO:0005739|mitochondrion;	GO:0046872|metal ion binding;	K18171	CMC1; COX assembly mitochondrial protein 1
PCGF5	629.1857	-0.45281	0.142276927	-3.18257	0.0014597	0.014989	Not_Sig	no	gi|555987003|ref|XP_005905356.1|	1296	2e-160	PREDICTED: polycomb group RING finger protein 5 isoform X1 [Bos mutus]	gi|742134139|ref|XM_010842208.1|	6618	0	PREDICTED: Bison bison bison polycomb group ring finger 5 (PCGF5), transcript variant X1, mRNA	A0JN86	498	9e-160	Polycomb group RING finger protein 5	.	.	.	.	pfam00097	zf-C3HC4	Zinc finger, C3HC4 type (RING finger). The C3HC4 type zinc-finger (RING finger) is a cysteine-rich domain of 40 to 60 residues that	GO:0045944|positive regulation of transcription from RNA polymerase II promoter;GO:0006351|transcription, DNA-templated;	GO:0005813|centrosome;GO:0005730|nucleolus;GO:0031519|PcG protein complex;	GO:0008270|zinc ion binding;	K11489	PCGF5; polycomb group RING finger protein 5
STEAP2	1687.409	-0.40296	0.12663486	-3.18209	0.0014622	0.015	Not_Sig	no	gi|594104639|ref|XP_006081037.1|	2518	0	PREDICTED: LOW QUALITY PROTEIN: STEAP family member 2, metalloreductase [Bubalus bubalis]	gi|942043694|ref|XM_005891858.2|	2376	0	PREDICTED: Bos mutus STEAP family member 2, metalloreductase (STEAP2), mRNA	Q8NFT2	944	0	Metalloreductase STEAP2	RSp1147	70.5	2.80e-12	COG2085	pfam03807	F420_oxidored	NADP oxidoreductase coenzyme F420-dependent.	GO:0015677|copper ion import;GO:0006897|endocytosis;GO:0097461|ferric iron import into cell;GO:0006893|Golgi to plasma membrane transport;GO:0055072|iron ion homeostasis;GO:0045055|regulated secretory pathway;GO:0009725|response to hormone;	GO:0005829|cytosol;GO:0005769|early endosome;GO:0010008|endosome membrane;GO:0030173|integral component of Golgi	GO:0008823|cupric reductase activity;GO:0052851|ferric-chelate	K14738	STEAP2; metalloreductase STEAP2 [EC:1.16.1.-]
SMARCC2	974.3635	0.440009	0.138326949	3.180936	0.001468	0.015047	Not_Sig	no	gi|803315564|ref|XP_012016064.1|	4870	0	PREDICTED: SWI/SNF complex subunit SMARCC2 isoform X2 [Ovis aries musimon]	gi|965898066|ref|XM_012160674.2|	4011	0	PREDICTED: Ovis aries musimon SWI/SNF related, matrix associated, actin dependent regulator of chromatin, subfamily c, member 2 (SMARCC2), transcript variant X2, mRNA	Q8TAQ2	1779	0	SWI/SNF complex subunit SMARCC2	SPAC23H3.10	185	3.16e-49	COG5259	pfam04433	SWIRM	SWIRM domain. This SWIRM domain is a small alpha-helical domain of about 85 amino acid residues found in chromosomal	GO:0043044|ATP-dependent chromatin remodeling;GO:0016569|covalent chromatin modification;GO:0006338|chromatin remodeling;GO:0000122|negative regulation of transcription from RNA polymerase II promoter;GO:0045892|negative regulation of transcription, DNA-templated;GO:0006337|nucleosome disassembly;GO:0045893|positive regulation of transcription, DNA-templated;GO:0006357|regulation of transcription from RNA polymerase II promoter;GO:0021882|regulation of transcription from RNA	GO:0071565|nBAF complex;GO:0071564|npBAF complex;GO:0000790|nuclear chromatin;GO:0005654|nucleoplasm;GO:00	GO:0003682|chromatin binding;GO:0003677|DNA	K11649	SMARCC; SWI/SNF related-matrix-associated actin-dependent regulator
RHBDF1	569.6111	0.447638	0.140746312	3.180456	0.0014704	0.015058	Not_Sig	no	gi|594033700|ref|XP_006041576.1|	4263	0	PREDICTED: inactive rhomboid protein 1 isoform X1 [Bubalus bubalis]	gi|741973373|ref|XM_010800790.1|	2996	0	PREDICTED: Bos taurus rhomboid 5 homolog 1 (Drosophila) (RHBDF1), transcript variant X4, mRNA	A7YWH9	1593	0	Inactive rhomboid protein 1	L173329	62.8	6.40e-10	COG0705	pfam12595	Rhomboid_SP	Rhomboid serine protease. This domain family is found in eukaryotes, and is approximately 210 amino acids in length. The	GO:0016477|cell migration;GO:0008283|cell proliferation;GO:0050709|negative regulation of protein secretion;GO:0015031|protein transport;GO:0006508|proteolysis;GO:0042058|regulation of epidermal growth factor receptor signaling pathway;GO:0061136|regulation of proteasomal protein catabolic process;GO:0050708|regulation of protein secretion;	GO:0005789|endoplasmic reticulum membrane;GO:0000139|Golgi membrane;GO:0016021|integral component of membrane;	.	.	.
RBMS1	1648.762	-0.33229	0.104547407	-3.17841	0.0014808	0.015151	Not_Sig	no	gi|759094952|ref|XP_011356953.1|	2312	0	PREDICTED: RNA-binding motif, single-stranded-interacting protein 1 isoform X1 [Pteropus vampyrus]	gi|742198538|ref|XM_010858975.1|	3963	0	PREDICTED: Bison bison bison RNA binding motif, single stranded interacting protein 1 (RBMS1), transcript variant X1, mRNA	P29558	705	0	RNA-binding motif, single-stranded-interacting protein 1	.	.	.	.	pfam00076	RRM_1	RNA recognition motif. (a.k.a. RRM, RBD, or RNP domain). The RRM motif is probably diagnostic of an RNA binding protein. RRMs	GO:0006260|DNA replication;GO:0006396|RNA processing;	GO:0005634|nucleus;	GO:0003690|double-stranded DNA binding;GO:0000166|nucleotide	.	.
WDTC1	899.2519	0.398236	0.125322016	3.177699	0.0014845	0.015175	Not_Sig	no	gi|803220508|ref|XP_011970562.1|	3494	0	PREDICTED: WD and tetratricopeptide repeats protein 1 isoform X5 [Ovis aries musimon]	gi|329663847|ref|NM_001205398.1|	3085	0	Bos taurus WD and tetratricopeptide repeats 1 (WDTC1), mRNA	Q8N5D0	1335	0	WD and tetratricopeptide repeats protein 1	.	.	.	.	pfam13414	TPR_11	TPR repeat.	GO:0055082|cellular chemical homeostasis;GO:0032869|cellular response to insulin stimulus;GO:0006006|glucose metabolic process;GO:0001701|in utero embryonic development;GO:0035264|multicellular organism growth;GO:0045717|negative regulation of fatty acid biosynthetic process;GO:0000122|negative regulation of transcription from RNA polymerase II promoter;GO:0016567|protein ubiquitination;GO:0008361|regulation of cell size;	GO:0005829|cytosol;GO:0005634|nucleus;	GO:0004857|enzyme inhibitor activity;	K11807	WDTC1; WD and tetratricopeptide repeats protein 1
GNG2	2224.296	-0.33868	0.106598864	-3.17717	0.0014872	0.015189	Not_Sig	no	gi|640823357|ref|XP_008068701.1|	396	3e-41	PREDICTED: guanine nucleotide-binding protein G(I)/G(S)/G(O) subunit gamma-2 isoform X1 [Tarsius syrichta]	gi|942051239|ref|XM_005894545.2|	3398	0	PREDICTED: Bos mutus guanine nucleotide binding protein (G protein), gamma 2 (GNG2), transcript variant X1, mRNA	Q5R7U4	144	1e-38	Guanine nucleotide-binding protein G(I)/G(S)/G(O) subunit gamma-2	.	.	.	.	pfam00631	G-gamma	GGL domain. G-protein gamma like domains (GGL) are found in the gamma subunit of the heterotrimeric G protein complex	GO:0008283|cell proliferation;GO:0007186|G-protein coupled receptor signaling pathway;	GO:0070062|extracellular exosome;GO:0005834|heterotrimeric G-protein complex;	GO:0031681|G-protein beta-subunit binding;GO:0004871|signal transducer	K07826	GNG2; guanine nucleotide-binding protein G(I)/G(S)/G(O)
ATP6V1G1	1634.295	-0.44873	0.141322948	-3.17524	0.0014971	0.015257	Not_Sig	no	gi|27806309|ref|NP_776670.1|	432	5e-49	V-type proton ATPase subunit G 1 [Bos taurus]	gi|942070463|ref|XM_005901084.2|	1090	0	PREDICTED: Bos mutus ATPase, H+ transporting, lysosomal 13kDa, V1 subunit G1 (ATP6V1G1), transcript variant X1, mRNA	P79251	171	2e-50	V-type proton ATPase subunit G 1	.	.	.	.	pfam03179	V-ATPase_G	Vacuolar (H+)-ATPase G subunit. This family represents the eukaryotic vacuolar (H+)-ATPase (V-ATPase) G subunit. V-ATPases	.	GO:0005829|cytosol;GO:0070062|extracellular exosome;GO:0005765|lysosomal membrane;GO:0005886|plasma membrane;GO:0016471|vacuolar proton-	GO:0008553|hydrogen-exporting ATPase activity, phosphorylative	K02152	ATPeV1G, ATP6G; V-type H+-transporting ATPase subunit G
STX1A	49.10414	0.969194	0.305240582	3.175182	0.0014974	0.015257	UP	yes	gi|139947868|ref|NP_001077267.1|	1284	4e-168	syntaxin-1A [Bos taurus]	gi|741975077|ref|XR_240047.2|	2201	0	PREDICTED: Bos taurus syntaxin 1A (STX1A), transcript variant X2, misc_RNA	P32850	352	5e-118	Syntaxin-1A	YMR183c	60.5	1.55e-12	COG5074	pfam00804	Syntaxin	Syntaxin. Syntaxins are the prototype family of SNARE proteins. They usually consist of three main regions - a C-terminal	GO:0006886|intracellular protein transport;GO:0045956|positive regulation of calcium ion-dependent exocytosis;GO:0033605|positive regulation of catecholamine secretion;GO:0010701|positive regulation of norepinephrine secretion;GO:0031629|synaptic vesicle fusion to presynaptic membrane;GO:0048278|vesicle docking;	GO:0030054|cell junction;GO:0016021|integral component of membrane;GO:0043005|neuron projection;GO:0005886|plasma	GO:0017022|myosin binding;GO:0005484|SNAP receptor	.	.
XRCC1	233.4287	0.501136	0.157882274	3.174113	0.001503	0.015257	Not_Sig	no	gi|742173779|ref|XP_010850848.1|	2848	0	PREDICTED: DNA repair protein XRCC1 isoform X1 [Bison bison bison]	gi|983000091|ref|XM_015467564.1|	2162	0	PREDICTED: Bos taurus X-ray repair complementing defective repair in Chinese hamster cells 1 (XRCC1), transcript variant X2, mRNA	P18887	923	0	DNA repair protein XRCC1	.	.	.	.	pfam01834	XRCC1_N	XRCC1 N terminal domain.	GO:0006284|base-excision repair;GO:0006288|base-excision repair, DNA ligation;GO:0000724|double-strand break repair via homologous recombination;GO:0021766|hippocampus development;GO:0006297|nucleotide-excision repair, DNA gap filling;GO:0042493|response to drug;GO:0001666|response to hypoxia;GO:0010033|response to organic substance;GO:0000012|single strand break repair;GO:0006283|transcription-coupled nucleotide-excision repair;	GO:0005654|nucleoplasm;GO:0005634|nucleus;	GO:0003684|damaged DNA binding;GO:0003909|DNA ligase	K10803	XRCC1; DNA-repair protein XRCC1
DPM3	121.4325	-0.59197	0.186509794	-3.17394	0.0015038	0.015257	DOWN	yes	gi|440903610|gb|ELR54247.1|	417	1e-49	Dolichol-phosphate mannosyltransferase subunit 3, partial [Bos mutus]	gi|965833331|ref|XM_012132342.2|	423	0	PREDICTED: Ovis aries musimon dolichyl-phosphate mannosyltransferase polypeptide 3 (DPM3), transcript variant X2, mRNA	Q3ZC71	162	3e-50	Dolichol-phosphate mannosyltransferase subunit 3	.	.	.	.	pfam08285	DPM3	Dolichol-phosphate mannosyltransferase subunit 3 (DPM3). This family corresponds to subunit 3 of dolichol-phosphate	GO:0006506|GPI anchor biosynthetic process;GO:0035269|protein O-linked mannosylation;GO:0031647|regulation of protein stability;	GO:0033185|dolichol-phosphate-mannose synthase complex;GO:0005789|endoplasmic reticulum membrane;GO:0030176|integral	GO:0004582|dolichyl-phosphate beta-D-mannosyltransferas	K09659	DPM3; dolichyl-phosphate mannosyltransferase polypeptide 3
PYGB	824.3273	0.382589	0.120544026	3.173856	0.0015043	0.015257	Not_Sig	no	gi|78369232|ref|NP_001030347.1|	4084	0	glycogen phosphorylase, brain form [Bos taurus]	gi|78369231|ref|NM_001035270.1|	2791	0	Bos taurus phosphorylase, glycogen; brain (PYGB), mRNA	Q3B7M9	1580	0	Glycogen phosphorylase, brain form	CPn0307	802	0	COG0058	pfam00343	Phosphorylase	Carbohydrate phosphorylase. The members of this family catalyse the formation of glucose 1-phosphate from one of the	GO:0005980|glycogen catabolic process;	GO:0005737|cytoplasm;	GO:0008184|glycogen phosphorylase activity;GO:0030170|pyridoxal	K00688	E2.4.1.1, glgP, PYG; starch phosphorylase [EC:2.4.1.1]

ELMOD2	233.9204	-0.53864	0.169711849	-3.17383	0.0015044	0.015257	Not_Sig	no	gi|115496638|ref|NP_001069173.1|	1576	0	ELMO domain-containing protein 2 [Bos taurus]	gi|982949762|ref|XM_005217554.3|	4851	0	PREDICTED: Bos taurus ELMO/CED-12 domain containing 2 (ELMOD2), transcript variant X1, mRNA	Q08DZ3	611	0	ELMO domain-containing protein 2	.	.	.	.	pfam04727	ELMO_CED12	ELMO/CED-12 family. This family represents a conserved domain which is found in a number of eukaryotic proteins including CED-	GO:0050688|regulation of defense response to virus;	GO:0016020|membrane;GO:0043234|protein complex;	GO:0005096|GTPase activator activity;	.	.
FYTTD1	1273.228	-0.43535	0.137170435	-3.17379	0.0015046	0.015257	Not_Sig	no	gi|594111482|ref|XP_006078558.1|	1582	0	PREDICTED: UAP56-interacting factor [Bubalus bubalis]	gi|594111481|ref|XM_006078496.1|	3235	0	PREDICTED: Bubalus bubalis forty-two-three domain containing 1 (FYTTD1), mRNA	Q17QU6	615	0	UAP56-interacting factor	.	.	.	.	pfam07078	FYTT	Forty-two-three protein. This family consists of several mammalian proteins of around 320 residues in length called 40-2-	GO:0006406|mRNA export from nucleus;	GO:0016607|nuclear speck;GO:0005654|nucleoplasm;	GO:0003729|mRNA binding;	.	.
ARHGAP33	42.7619	1.016257	0.320224334	3.173579	0.0015057	0.015257	UP	yes	gi|741959482|ref|XP_010813164.1|	4826	0	PREDICTED: rho GTPase-activating protein 33 isoform X3 [Bos taurus]	gi|982999604|ref|XM_010826959.2|	4113	0	PREDICTED: Bos taurus Rho GTPase activating protein 33 (ARHGAP33), transcript variant X3, mRNA	O14559	1653	0	Rho GTPase-activating protein 33	.	.	.	.	pfam00620	RhoGAP	RhoGAP domain. GTPase activator proteins towards Rho/Rac/Cdc42-like small GTPases.	GO:0015031|protein transport;GO:0051056|regulation of small GTPase mediated signal transduction;GO:0007264|small GTPase mediated signal transduction;	GO:0005737|cytoplasm;GO:0005829|cytosol;GO:0005886|plasma membrane;	GO:0005096|GTPase activator activity;GO:0035091|phosphatidylinosit	K17933	ARHGAP33, SNX26; Rho GTPase-activating protein 33
SRRT	1210.864	0.332938	0.104909629	3.173567	0.0015058	0.015257	Not_Sig	no	gi|742109952|ref|XP_010836216.1|	1953	0	PREDICTED: serrate RNA effector molecule homolog isoform X2 [Bison bison bison]	gi|942100455|ref|XM_005910793.2|	3033	0	PREDICTED: Bos mutus serrate, RNA effector molecule (SRRT), mRNA	A4IFB1	748	0	Serrate RNA effector molecule homolog	.	.	.	.	pfam04959	ARS2	Arsenite-resistance protein 2. Arsenite is a carcinogenic compound which can act as a co-mutagen by inhibiting DNA repair.	GO:0008283|cell proliferation;GO:0097150|neuronal stem cell population maintenance;GO:0031053|primary miRNA processing;GO:0006355|regulation of transcription, DNA-templated;GO:0006351|transcription, DNA-templated;	GO:0005737|cytoplasm;GO:0005654|nucleoplasm;	GO:0003677|DNA binding;GO:0000166|nucleotide binding;	.	.
IVNS1ABP	4255.553	-0.28874	0.090999975	-3.17301	0.0015087	0.015273	Not_Sig	no	gi|555962145|ref|XP_005893166.1|	3430	0	PREDICTED: influenza virus NS1A-binding protein [Bos mutus]	gi|742175678|ref|XM_010853076.1|	3600	0	PREDICTED: Bison bison bison influenza virus NS1A binding protein (IVNS1ABP), transcript variant X1, mRNA	Q920Q8	1291	0	Influenza virus NS1A-binding protein homolog	.	.	.	.	pfam00651	BTB	BTB/POZ domain. The BTB (for BR-C, ttk and bab) or POZ (for Pox virus and Zinc finger) domain is present near the N-terminus of a	GO:2001243|negative regulation of intrinsic apoptotic signaling pathway;	GO:0015629|actin cytoskeleton;GO:0005737|cytoplasm;GO:0005634|nucleus;	.	K15046	NS1BP; influenza virus NS1A-binding protein
PEX13	308.174	-0.54927	0.17316139	-3.172	0.0015139	0.015313	Not_Sig	no	gi|555953857|ref|XP_005889098.1|	1815	0	PREDICTED: peroxisome biogenesis factor 13 [Bos mutus]	gi|742214978|ref|XM_010862964.1|	3459	0	PREDICTED: Bison bison bison peroxisomal biogenesis factor 13 (PEX13), mRNA	Q0P5B1	700	0	Peroxisomal membrane protein PEX13	.	.	.	.	pfam04088	Peroxin-13_N	Peroxin 13, N-terminal region. Both termini of the Peroxin-13 are oriented to the cytosol. Peroxin-13 is required for peroxisomal	GO:0021795|cerebral cortex cell migration;GO:0001561|fatty acid alpha-oxidation;GO:0007626|locomotory behavior;GO:0060152|microtubule-based peroxisome localization;GO:0098779|mitophagy in response to mitochondrial depolarization;GO:0001764|neuron migration;GO:0002230|positive regulation of defense response to virus by host;GO:0016560|protein import into peroxisome matrix, docking;GO:0001967|suckling behavior;GO:0098792|xenophagy;	GO:0005779|integral component of peroxisomal membrane;GO:0005778|peroxisomal membrane;	.	K13344	PEX13; peroxin-13
RNF126	1124.256	0.519153	0.163719846	3.170982	0.0015192	0.015353	Not_Sig	no	gi|115496796|ref|NP_001068782.1|	1290	1e-172	E3 ubiquitin-protein ligase RNF126 [Bos taurus]	gi|965800872|ref|XM_012116269.2|	1437	0	PREDICTED: Ovis aries musimon ring finger protein 126 (RNF126), transcript variant X2, mRNA	Q0II22	501	5e-174	E3 ubiquitin-protein ligase RNF126	.	.	.	.	pfam13639	zf-RING_2	Ring finger domain.	GO:0071629|cytoplasm-associated proteasomal ubiquitin-dependent protein catabolic process;GO:0042059|negative regulation of epidermal growth factor receptor signaling pathway;GO:0043161|proteasome-mediated ubiquitin-dependent protein catabolic process;GO:0070936|protein K48-linked ubiquitination;GO:0070534|protein K63-linked ubiquitination;GO:0006513|protein monoubiquitination;GO:0000209|protein polyubiquitination;GO:0042787|protein ubiquitination involved in ubiquitin-dependent protein	GO:0005737|cytoplasm;GO:0005829|cytosol;GO:0005634|nucleus;	GO:0016874|ligase activity;GO:0061630|ubiquitin protein ligase	K11982	RNF115_126; E3 ubiquitin-protein ligase RNF115/126 [EC:2.3.2.27]
MDC1	757.5092	0.442526	0.139568764	3.170668	0.0015209	0.015356	Not_Sig	no	gi|555990441|ref|XP_005907014.1|	8440	0	PREDICTED: LOW QUALITY PROTEIN: mediator of DNA damage checkpoint protein 1 [Bos mutus]	gi|594056364|ref|XM_006052325.1|	6108	0	PREDICTED: Bubalus bubalis mediator of DNA-damage checkpoint 1 (MDC1), transcript variant X1, mRNA	Q767L8	1429	0	Mediator of DNA damage checkpoint protein 1	.	.	.	.	pfam00498	FHA	FHA domain. The FHA (Forkhead-associated) domain is a phosphopeptide binding motif.	GO:0007049|cell cycle;GO:0006281|DNA repair;	GO:0005694|chromosome;GO:0005634|nucleus;	.	.	.
RNF13	1335.835	-0.33175	0.104681859	-3.16908	0.0015292	0.015426	Not_Sig	no	gi|115497538|ref|NP_001069610.1|	1898	0	E3 ubiquitin-protein ligase RNF13 precursor [Bos taurus]	gi|555989034|ref|XM_005906287.1|	2247	0	PREDICTED: Bos mutus ring finger protein 13 (RNF13), transcript variant X1, mRNA	Q0VD51	709	0	E3 ubiquitin-protein ligase RNF13	SPAC57A7.09	69.3	1.27e-11	COG5540	pfam13639	zf-RING_2	Ring finger domain.	GO:0051865|protein autoubiquitination;	GO:0005789|endoplasmic reticulum membrane;GO:0000139|Golgi membrane;GO:0016021|integral component of membrane;GO:0031902|late	GO:0016874|ligase activity;GO:0061630|ubiquitin protein ligase	K15692	RNF13, RZF; E3 ubiquitin-protein ligase RNF13 [EC:2.3.2.27]
ADAP1	267.9953	0.550476	0.173756291	3.168091	0.0015344	0.015465	Not_Sig	no	gi|529007246|ref|XP_005225071.1|	2019	0	PREDICTED: arf-GAP with dual PH domain-containing protein 1 isoform X2 [Bos taurus]	gi|982967157|ref|XM_005225014.3|	2811	0	PREDICTED: Bos taurus ArfGAP with dual PH domains 1 (ADAP1), transcript variant X2, mRNA	O75689	721	0	Arf-GAP with dual PH domain-containing protein 1	YIL044c	91.7	3.20e-19	COG5347	pfam01412	ArfGap	Putative GTPase activating protein for Arf. Putative zinc fingers with GTPase activating proteins (GAPs) towards the small GTPase, Arf. The	GO:0007166|cell surface receptor signaling pathway;GO:0043087|regulation of GTPase activity;	GO:0005737|cytoplasm;GO:0005634|nucleus;GO:0005886|plasma membrane;	GO:0005096|GTPase activator activity;GO:0043533|inositol 1,3,4,5	.	.
ZNF609	731.0666	0.392252	0.123829925	3.167669	0.0015367	0.015474	Not_Sig	no	gi|555965079|ref|XP_005894600.1|	5879	0	PREDICTED: zinc finger protein 609 [Bos mutus]	gi|982936881|ref|XM_005211662.3|	8661	0	PREDICTED: Bos taurus zinc finger protein 609 (ZNF609), transcript variant X1, mRNA	O15014	2221	0	Zinc finger protein 609	.	.	.	.	pfam13908	Shisa	Wnt and FGF inhibitory regulator. Shisa is a transcription factor-type molecule that physically interacts with immature forms of the Wnt	.	GO:0005634|nucleus;	GO:0046872|metal ion binding;	.	.
NTN4	169.7057	-0.52461	0.165668578	-3.16663	0.0015422	0.015516	Not_Sig	no	gi|741926719|ref|XP_010803496.1|	3140	0	PREDICTED: netrin-4 isoform X1 [Bos taurus]	gi|982925831|ref|XM_010805194.2|	3254	0	PREDICTED: Bos taurus netrin 4 (NTN4), transcript variant X1, mRNA	Q9JI33	1122	0	Netrin-4	.	.	.	.	pfam00055	Laminin_N	Laminin N-terminal (Domain VI).	GO:0030198|extracellular matrix organization;GO:0016322|neuron remodeling;GO:0060668|regulation of branching involved in salivary gland morphogenesis by extracellular matrix-epithelial cell signaling;	GO:0005604|basement membrane;GO:0005886|plasma membrane;	GO:0043237|laminin-1 binding;	K06845	NTN4; netrin 4
GPCPD1	603.4515	-0.40334	0.12747163	-3.16417	0.0015553	0.015634	Not_Sig	no	gi|297481642|ref|XP_002692410.1|	3417	0	PREDICTED: glycerophosphocholine phosphodiesterase GPCPD1 isoform X2 [Bos taurus]	gi|942041726|ref|XM_005891059.2|	3233	0	PREDICTED: Bos mutus glycerophosphocholine phosphodiesterase 1 (GPCPD1), mRNA	Q9NPB8	1296	0	Glycerophosphocholine phosphodiesterase GPCPD1	YPL110c_3	150	1.05e-38	COG0584	pfam03009	GDPD	Glycerophosphoryl diester phosphodiesterase family. Escherichia coli has two sequence related isozymes of	GO:0030643|cellular phosphate ion homeostasis;GO:0046475|glycerophospholipid catabolic process;GO:0007519|skeletal muscle tissue development;	GO:0005829|cytosol;	GO:0047389|glycerophosphocholine phosphodiesterase activity;GO:0008889	K18695	GPCPD1; glycerophosphocholine phosphodiesterase
CHIC2	605.8056	-0.35052	0.110846085	-3.16218	0.0015659	0.015728	Not_Sig	no	gi|4239700|emb|CAA10770.1|	742	2e-139	reverse transcriptase-like [Bos taurus]	gi|850484145|gb|CP011891.1|	4805	0	Ovis canadensis canadensis isolate 43U chromosome 6 sequence	Q9UKJ5	272	6e-81	Cysteine-rich hydrophobic domain-containing protein 2	.	.	.	.	pfam10256	Erf4	Golgin subfamily A member 7/ERF4 family. This family of proteins includes Golgin subfamily A member 7 proteins as well as	GO:0006893|Golgi to plasma membrane transport;	GO:0005798|Golgi-associated vesicle;GO:0005886|plasma membrane;	.	.	.
TCF7L1	315.9686	0.622193	0.196800963	3.161534	0.0015694	0.015749	UP	yes	gi|741943340|ref|XP_002691454.3|	2092	0	PREDICTED: transcription factor 7-like 1 isoform X1 [Bos taurus]	gi|982990799|ref|XM_015465393.1|	2951	0	PREDICTED: Bos taurus transcription factor 7-like 1 (T-cell specific, HMG-box) (TCF7L1), transcript variant X2, mRNA	Q9HCS4	743	0	Transcription factor 7-like 1	.	.	.	.	pfam08347	CTNNB1_binding	N-terminal CTNNB1 binding. This region tends to appear at the N-terminus of proteins also containing DNA-binding HMG (high	GO:0060070|canonical Wnt signaling pathway;GO:0006325|chromatin organization;GO:0006357|regulation of transcription from RNA polymerase II promoter;GO:0006355|regulation of transcription, DNA-templated;GO:0030111|regulation of Wnt signaling pathway;GO:0006351|transcription, DNA-templated;	GO:0005654|nucleoplasm;GO:0005634|nucleus;GO:0005667|transcription factor complex;	GO:0008013|beta-catenin binding;GO:0003677|DNA	K04490	TCF7L1; transcription factor 7-like 1
ATXN3	301.5226	-0.50544	0.159930185	-3.16037	0.0015757	0.015766	Not_Sig	no	gi|555997181|ref|XP_005910318.1|	1837	0	PREDICTED: ataxin-3 isoform X2 [Bos mutus]	gi|982960789|ref|XM_010817282.2|	5191	0	PREDICTED: Bos taurus ataxin 3 (ATXN3), transcript variant X1, mRNA	O35815	590	0	Ataxin-3	.	.	.	.	pfam02099	Josephin	Josephin.	GO:0030036|actin cytoskeleton organization;GO:0034605|cellular response to heat;GO:0071218|cellular response to misfolded protein;GO:0035640|exploration behavior;GO:0070932|histone H3 deacetylation;GO:0045104|intermediate filament cytoskeleton organization;GO:0000226|microtubule cytoskeleton organization;GO:0006515|misfolded or incompletely synthesized protein catabolic process;GO:0035520|monoubiquitinated protein deubiquitination;GO:0043161|proteasome-mediated ubiquitin-dependent protein catabolic	GO:0005829|cytosol;GO:0005759|mitochondrial matrix;GO:0031966|mitochondrial membrane;GO:0042405|nuclear inclusion body;GO:0005634|nucleus;	GO:0051117|ATPase binding;GO:0042802|identical protein	K11863	ATXN3, MJD; Ataxin-3 [EC:3.4.22.-]
PCNP	1505.266	-0.44254	0.140045342	-3.15998	0.0015778	0.015766	Not_Sig	no	gi|742133156|ref|XP_010840274.1|	681	1e-80	PREDICTED: PEST proteolytic signal-containing nuclear protein [Bison bison bison]	gi|942039278|ref|XM_005890062.2|	2321	0	PREDICTED: Bos mutus PEST proteolytic signal containing nuclear protein (PCNP), transcript variant X1, mRNA	Q32PF3	253	2e-77	PEST proteolytic signal-containing nuclear protein	.	.	.	.	pfam15473	PCNP	PEST, proteolytic signal-containing nuclear protein family. PCNP is a PEST-containing nuclear protein that is ubiquitinated by NIRF, a	GO:0007049|cell cycle;GO:0043161|proteasome-mediated ubiquitin-dependent protein catabolic process;GO:0016567|protein ubiquitination;	GO:0005634|nucleus;	.	.	.
C23H6orf136	137.4604	0.686541	0.217263496	3.159947	0.001578	0.015766	UP	yes	gi|942088468|ref|XP_014337378.1|	1717	0	PREDICTED: LOW QUALITY PROTEIN: uncharacterized protein C6orf136 homolog [Bos mutus]	gi|942088467|ref|XM_014481892.1|	1638	0	PREDICTED: Bos mutus chromosome unknown open reading frame, human C6orf136 (LOC102276225), mRNA	Q32KT5	427	5e-144	Uncharacterized protein C6orf136 homolog	.	.	.	.	pfam10184	DUF2358	Uncharacterized conserved protein (DUF2358). DUF2358 is a family of conserved proteins found from plants to humans. The function is	.	.	.	.	.
MZT1	267.3914	-0.66634	0.210879471	-3.15981	0.0015787	0.015766	DOWN	yes	gi|965956050|ref|XP_014953701.1|	353	1e-35	PREDICTED: mitotic-spindle organizing protein 1 [Ovis aries]	gi|742085337|ref|XM_010832001.1|	2037	0	PREDICTED: Bison bison bison mitotic spindle organizing protein 1 (MZT1), mRNA	Q08AG7	131	1e-34	Mitotic-spindle organizing protein 1	.	.	.	.	pfam12554	MOZART1	Mitotic-spindle organizing gamma-tubulin ring associated. The name MOZART is derived from letters of 'mitotic-spindle organizing	GO:0033566|gamma-tubulin complex localization;GO:0051415|interphase microtubule nucleation by interphase microtubule organizing center;GO:0090307|mitotic spindle assembly;	GO:0005813|centrosome;GO:0000923|equatorial microtubule organizing center;GO:0008274|gamma-tubulin ring complex;GO:0031021|interphase	GO:0043015|gamma-tubulin binding;	K18633	MZT1, GIP1, GIP2; mitotic-spindle organizing protein 1
C2H2orf69	254.0789	-0.52488	0.16611364	-3.15979	0.0015789	0.015766	Not_Sig	no	gi|555966083|ref|XP_005895098.1|	1859	0	PREDICTED: UPF0565 protein C2orf69 homolog [Bos mutus]	gi|742206719|ref|XM_010860930.1|	4768	0	PREDICTED: Bison bison bison chromosome unknown open reading frame, human C2orf69 (LOC105003631), mRNA	Q8N8R5	632	0	UPF0565 protein C2orf69	.	.	.	.	pfam10561	UPF0565	Uncharacterized protein family UPF0565. This family of proteins has no known function.	.	GO:0005576|extracellular region;	.	.	.
ORMDL1	321.5236	-0.48923	0.154846211	-3.15948	0.0015805	0.015766	Not_Sig	no	gi|548453020|ref|XP_005675918.1|	818	3e-102	PREDICTED: ORM1-like protein 1 [Capra hircus]	gi|555954855|ref|XM_005889531.1|	1983	0	PREDICTED: Bos mutus ORMDL sphingolipid biosynthesis regulator 1 (ORMDL1), transcript variant X1, mRNA	Q29RQ9	313	9e-102	ORM1-like protein 1	SPBC119.09c	117	1.04e-29	COG5081	pfam04061	ORMDL	ORMDL family. Evidence form suggests that ORMDLs are involved in protein folding in the ER. Orm proteins have been identified as	GO:0090156|cellular sphingolipid homeostasis;GO:0006672|ceramide metabolic process;GO:1900060|negative regulation of ceramide biosynthetic process;	GO:0005789|endoplasmic reticulum membrane;GO:0016021|integral component of membrane;GO:0035339|SPOTS complex;	.	.	.
CLIC5	60.6345	0.850607	0.269243214	3.15925	0.0015818	0.015766	UP	yes	gi|742215466|ref|XP_010861383.1|	2153	0	PREDICTED: chloride intracellular channel protein 5 [Bison bison bison]	gi|742215465|ref|XM_010863081.1|	5951	0	PREDICTED: Bison bison bison chloride intracellular channel 5 (CLIC5), mRNA	P35526	824	0	Chloride intracellular channel protein 5	.	.	.	.	pfam13409	GST_N_2	Glutathione S-transferase, N-terminal domain. This family is closely related to pfam02798.	GO:0060088|auditory receptor cell stereocilium organization;GO:0006821|chloride transport;GO:0007605|sensory perception of sound;	GO:0034707|chloride channel complex;GO:0016023|cytoplasmic membrane-bounded vesicle;GO:0005794|Golgi	GO:0005254|chloride channel activity;GO:0005244|voltage-gated ion	K05025	CLIC5; chloride intracellular channel protein 5
MOV10	1077.753	0.392925	0.124384297	3.158963	0.0015833	0.015766	Not_Sig	no	gi|440913207|gb|ELR62687.1|	4975	0	Putative helicase MOV-10, partial [Bos mutus]	gi|742231975|ref|XM_010830798.1|	3241	0	PREDICTED: Bison bison bison Mov10 RISC complex RNA helicase (MOV10), transcript variant X1, mRNA	Q0V8H6	1911	0	Putative helicase MOV-10	SPAC16C9.06c	162	5.63e-40	COG1112	pfam13087	AAA_12	AAA domain. This family of domains contain a P-loop motif that is characteristic of the AAA superfamily. Many of the proteins	GO:0035195|gene silencing by miRNA;GO:0035279|mRNA cleavage involved in gene silencing by miRNA;	GO:0000932|cytoplasmic mRNA processing body;GO:0005615|extracellular space;	GO:0005524|ATP binding;GO:0004386|helicase activity;GO:0044822	K18422	MOV10; helicase MOV-10 [EC:3.6.4.13]
ATP5PD	767.8557	-0.41581	0.131629383	-3.15893	0.0015835	0.015766	Not_Sig	no	gi|741964614|ref|XP_010814810.1|	920	2e-123	PREDICTED: ATP synthase subunit d, mitochondrial isoform X1 [Bos taurus]	gi|942091908|ref|XM_014482241.1|	670	0	PREDICTED: Bos mutus ATP synthase, H+ transporting, mitochondrial Fo complex, subunit d (ATP5H), transcript variant X2, mRNA	P13620	329	8e-114	ATP synthase subunit d, mitochondrial	.	.	.	.	pfam05873	Mt_ATP-synt_D	ATP synthase D chain, mitochondrial (ATP5H). This family consists of several ATP synthase D chain, mitochondrial (ATP5H)	GO:0015986|ATP synthesis coupled proton transport;	GO:0005753|mitochondrial proton-transporting ATP synthase complex;GO:0000274|mitochondrial proton-transporting ATP synthase, stator	GO:0015078|hydrogen ion transmembrane transporter activity;	K02138	ATPeF0D, ATP5H, ATP7; F-type H+-transporting ATPase subunit d
FAM120C	377.0112	-0.40762	0.129051904	-3.15854	0.0015856	0.015774	Not_Sig	no	gi|983011363|ref|XP_015325981.1|	4896	0	PREDICTED: LOW QUALITY PROTEIN: constitutive coactivator of PPAR-gamma-like protein 2 isoform X2 [Bos taurus]	gi|594091343|ref|XM_006068992.1|	7848	0	PREDICTED: Bubalus bubalis family with sequence similarity 120C (FAM120C), mRNA	Q9NX05	1770	0	Constitutive coactivator of PPAR-gamma-like protein 2	.	.	.	.	pfam13388	DUF4106	Protein of unknown function (DUF4106). This family of proteins are found in large numbers in the Trichomonas vaginalis proteome.	.	GO:0005634|nucleus;	GO:0044822|poly(A) RNA binding;	.	.
FBRS	776.4268	0.503527	0.159558643	3.155747	0.0016009	0.015912	Not_Sig	no	gi|741974598|ref|XP_005225017.2|	1687	0	PREDICTED: probable fibrosin-1 [Bos taurus]	gi|965906536|ref|XM_012166135.2|	4398	0	PREDICTED: Ovis aries musimon fibrosin (FBRS), transcript variant X2, mRNA	Q9HAH7	556	0	Probable fibrosin-1	.	.	.	.	pfam15336	Auts2	Autism susceptibility gene 2 protein. Auts2, or FBRSL2, Fibrosin-1-like protein 2, is a family of eukaryotic proteins associated	GO:0048146|positive regulation of fibroblast proliferation;	GO:0005615|extracellular space;	.	.	.
EIF4E	1211.368	-0.43685	0.138450521	-3.15527	0.0016035	0.015924	Not_Sig	no	gi|556737644|ref|XP_005964407.1|	1100	3e-136	PREDICTED: eukaryotic translation initiation factor 4E [Pantholops hodgsonii]	gi|965902888|ref|XM_012162834.2|	3559	0	PREDICTED: Ovis aries musimon eukaryotic translation initiation factor 4E (EIF4E), transcript variant X4, mRNA	P06730	410	6e-132	Eukaryotic translation initiation factor 4E	SPAC16E8.15	140	5.53e-37	COG5053	pfam01652	IF4E	Eukaryotic initiation factor 4E.	GO:0001662|behavioral fear response;GO:0000082|G1/S transition of mitotic cell cycle;GO:0030324|lung development;GO:0006406|mRNA export from nucleus;GO:0045665|negative regulation of neuron differentiation;GO:0017148|negative regulation of translation;GO:0000289|nuclear-transcribed mRNA poly(A) tail shortening;GO:0045931|positive regulation of mitotic cell cycle;GO:0006417|regulation of translation;GO:0006405|RNA export from nucleus;GO:0019827|stem cell population	GO:0033391|chromatoid body;GO:0005737|cytoplasm;GO:0000932|cytoplasmic mRNA processing body;GO:0010494|cytoplasmic stress	GO:0019899|enzyme binding;GO:0031370|eukaryotic	K03259	EIF4E; translation initiation factor 4E
AMD1	2262.484	-0.37809	0.119852231	-3.15464	0.001607	0.01594	Not_Sig	no	gi|27806745|ref|NP_776415.1|	1688	0	S-adenosylmethionine decarboxylase proenzyme [Bos taurus]	gi|742188917|ref|XM_010856612.1|	5813	0	PREDICTED: Bison bison bison adenosylmethionine decarboxylase 1 (AMD1), transcript variant X1, mRNA	P50243	653	0	S-adenosylmethionine decarboxylase proenzyme	.	.	.	.	pfam01536	SAM_decarbox	Adenosylmethionine decarboxylase. This is a family of S-adenosylmethionine decarboxylase (SAMDC)	GO:0006557|S-adenosylmethioninamine biosynthetic process;GO:0008295|spermidine biosynthetic process;GO:0006597|spermine biosynthetic process;	GO:0005829|cytosol;	GO:0004014|adenosylmethionine decarboxylase activity;GO:0019810	K01611	speD, AMD1; S-adenosylmethionine decarboxylase [EC:4.1.1.50]
ZCCHC10	136.1863	-0.60238	0.190959941	-3.15447	0.0016079	0.01594	DOWN	yes	gi|655639804|ref|XP_008253223.1|	359	4e-36	PREDICTED: zinc finger CCHC domain-containing protein 10 [Oryctolagus cuniculus]	gi|742142113|ref|XM_010844526.1|	2122	0	PREDICTED: Bison bison bison zinc finger, CCHC domain containing 10 (ZCCHC10), mRNA	Q5EB97	116	3e-28	Zinc finger CCHC domain-containing protein 10	.	.	.	.	pfam13917	zf-CCHC_3	Zinc knuckle. The zinc knuckle is a zinc binding motif composed of the the following CX2CX4HX4C where X can be any amino acid. The	.	.	GO:0003676|nucleic acid binding;GO:0008270|zinc ion binding;	.	.
GYS1	3709.406	0.456802	0.144840124	3.153835	0.0016114	0.015961	Not_Sig	no	gi|555966759|ref|XP_005895430.1|	3583	0	PREDICTED: glycogen [starch] synthase, muscle isoform X1 [Bos mutus]	gi|555966758|ref|XM_005895368.1|	3568	0	PREDICTED: Bos mutus glycogen synthase 1 (muscle) (GYS1), transcript variant X1, mRNA	A7MB78	1375	0	Glycogen [starch] synthase, muscle	YFR015c	713	0	COG0438	pfam05693	Glycogen_syn	Glycogen synthase. This family consists of the eukaryotic glycogen synthase proteins GYS1, GYS2 and GYS3. Glycogen synthase (GS) is	GO:0005978|glycogen biosynthetic process;GO:0007507|heart development;	GO:0005737|cytoplasm;GO:0016234|inclusion body;GO:0016020|membrane;	GO:0004373|glycogen (starch) synthase activity;GO:0061547|glycogen synthase	K00693	GYS; glycogen(starch) synthase [EC:2.4.1.11]
WNK1	1983.399	0.370085	0.117373555	3.153051	0.0016157	0.01599	Not_Sig	no	gi|594093336|ref|XP_006069989.1|	8391	0	PREDICTED: serine/threonine-protein kinase WNK1 isoform X2 [Bubalus bubalis]	gi|982982510|ref|XM_015463544.1|	9425	0	PREDICTED: Bos taurus WNK lysine deficient protein kinase 1 (WNK1), transcript variant X19, mRNA	Q9H4A3	1763	0	Serine/threonine-protein kinase WNK1	.	.	.	.	pfam00069	Pkinase	Protein kinase domain.	GO:0035556|intracellular signal transduction;GO:0006811|ion transport;GO:0090188|negative regulation of pancreatic juice secretion;GO:0010923|negative regulation of phosphatase activity;GO:0010766|negative regulation of sodium ion transport;GO:0048666|neuron development;GO:0018107|peptidyl-threonine phosphorylation;GO:0032414|positive regulation of ion transmembrane transporter activity;GO:0003084|positive regulation of systemic arterial blood pressure;GO:0046777|protein	GO:0005737|cytoplasm;GO:0005829|cytosol;GO:0016020|membrane;	GO:0005524|ATP binding;GO:0019869|chloride channel inhibitor	K08867	WNK, PRKWNK; WNK lysine deficient protein kinase [EC:2.7.11.1]
NYNRIN	342.7004	0.438509	0.139154586	3.151233	0.0016258	0.016076	Not_Sig	no	gi|300794498|ref|NP_001179388.1|	8578	0	protein NYNRIN [Bos taurus]	gi|300794497|ref|NM_001192459.1|	7384	0	Bos taurus NYN domain and retroviral integrase containing (NYNRIN), mRNA	Q9P2P1	2800	0	Protein NYNRIN	SPAC167.08	70.5	8.24e-11	COG2801	pfam11977	RNase_Zc3h12a	Zc3h12a-like Ribonuclease NYN domain. This domain is found in the Zc3h12a protein which has shown to be a ribonuclease that	GO:0015074|DNA integration;	GO:0016021|integral component of membrane;	GO:0003676|nucleic acid binding;	.	.
MSX1	126.2335	0.60702	0.192650609	3.150886	0.0016278	0.016082	UP	yes	gi|555962345|ref|XP_005893264.1|	962	3e-122	PREDICTED: homeobox protein MSX-1 [Bos mutus]	gi|742156980|ref|XM_010847785.1|	1870	0	PREDICTED: Bison bison bison msh homeobox 1 (MSX1), mRNA	O02786	366	3e-120	Homeobox protein MSX-1	.	.	.	.	pfam00046	Homeobox	Homeobox domain.	GO:0007275|multicellular organismal development;GO:0006355|regulation of transcription, DNA-templated;GO:0006351|transcription, DNA-templated;	GO:0005634|nucleus;	GO:0043565|sequence-specific DNA binding;	K09341	MSX; homeobox protein MSX
SYVN1	917.6819	0.50353	0.159845361	3.150105	0.0016321	0.016111	Not_Sig	no	gi|585170316|ref|XP_006737179.1|	2145	0	PREDICTED: E3 ubiquitin-protein ligase synoviolin [Leptonychotes weddellii]	gi|742110503|ref|XM_010838044.1|	3256	0	PREDICTED: Bison bison bison synovial apoptosis inhibitor 1, synoviolin (SYVN1), mRNA	Q86TM6	702	0	E3 ubiquitin-protein ligase synoviolin	SPBC17D11.02c	149	2.47e-36	COG5243	pfam13639	zf-RING_2	Ring finger domain.	GO:1904380|endoplasmic reticulum mannose trimming;GO:0030968|endoplasmic reticulum unfolded protein response;GO:0030433|ER-associated ubiquitin-dependent protein catabolic process;GO:0036503|ERAD pathway;GO:0036498|IRE1-mediated unfolded protein response;GO:1902236|negative regulation of endoplasmic reticulum stress-induced intrinsic apoptotic signaling pathway;GO:0070936|protein K48-linked ubiquitination;GO:0018279|protein N-linked glycosylation via asparagine;GO:0050821|protein stabilization;GO:0016567|protein	GO:0036513|Derlin-1 retrotranslocation complex;GO:0005783|endoplasmic reticulum;GO:0005789|endoplasmic reticulum	GO:0051117|ATPase binding;GO:0051087|chaperone	K10601	SYVN1, HRD1; E3 ubiquitin-protein ligase synoviolin [EC:2.3.2.27]
MME	381.5973	-0.40747	0.129373292	-3.14954	0.0016353	0.016117	Not_Sig	no	gi|300797944|ref|NP_001179813.1|	3742	0	neprilysin [Bos taurus]	gi|555969506|ref|XM_005896716.1|	3612	0	PREDICTED: Bos mutus membrane metallo-endopeptidase (MME), transcript variant X1, mRNA	P08473	1329	0	Neprilysin	MA2001	293	2.33e-85	COG3590	pfam05649	Peptidase_M13_N	Peptidase family M13. M13 peptidases are well-studied proteases found in a wide range of organisms including mammals and	GO:0002003|angiotensin maturation;GO:0050435|beta-amyloid metabolic process;GO:0071345|cellular response to cytokine stimulus;GO:0071492|cellular response to UV-A;GO:0071493|cellular response to UV-B;GO:0046449|creatinine metabolic process;GO:0001822|kidney development;GO:0006518|peptide metabolic process;GO:0006508|proteolysis;GO:0090399|replicative senescence;GO:0019233|sensory perception of pain;	GO:0030424|axon;GO:0005903|brush border;GO:0005737|cytoplasm;GO:0030425|dendrite;GO:0070062|extracellular exosome;GO:0005925|focal	GO:0004175|endopeptidase activity;GO:0008238|exopeptidase	K01389	MME; neprilysin [EC:3.4.24.11]
ALDH3B1	1128.088	0.443739	0.140900372	3.149313	0.0016365	0.016117	Not_Sig	no	gi|151554628|gb|AAI47959.1|	2094	0	Aldehyde dehydrogenase 3 family, member B1 [Bos taurus]	gi|982971910|ref|XM_005227061.3|	1609	0	PREDICTED: Bos taurus aldehyde dehydrogenase 3 family member B1 (ALDH3B1), transcript variant X1, mRNA	Q1JPA0	810	0	Aldehyde dehydrogenase family 3 member B1	alr3672	384	6.04e-126	COG1012	pfam00171	Aldedh	Aldehyde dehydrogenase family. This family of dehydrogenases act on aldehyde substrates. Members use NADP as a cofactor. The family	GO:0046185|aldehyde catabolic process;GO:0034599|cellular response to oxidative stress;GO:0006068|ethanol catabolic process;	GO:0005829|cytosol;GO:0070062|extracellular exosome;GO:0005886|plasma membrane;	GO:0004028|3-chloroallyl aldehyde dehydrogenase activity;GO:0004029	K00129	E1.2.1.5; aldehyde dehydrogenase (NAD(P)+) [EC:1.2.1.5]
CUL5	639.7416	-0.48144	0.152873872	-3.14923	0.001637	0.016117	Not_Sig	no	gi|742214393|ref|XP_010861132.1|	4122	0	PREDICTED: cullin-5 isoform X1 [Bison bison bison]	gi|742214400|ref|XM_010862833.1|	7122	0	PREDICTED: Bison bison bison cullin 5 (CUL5), transcript variant X3, mRNA	Q93034	1561	0	Cullin-5	SPAC17G6.12	241	2.29e-65	COG5647	pfam00888	Cullin	Cullin family.	GO:0007050|cell cycle arrest;GO:0008283|cell proliferation;GO:0038128|ERBB2 signaling pathway;GO:0000082|G1/S transition of mitotic cell cycle;GO:0097193|intrinsic apoptotic signaling pathway;GO:0008285|negative regulation of cell proliferation;GO:0042787|protein ubiquitination involved in ubiquitin-dependent protein catabolic process;GO:0016032|viral process;	GO:0031466|Cul5-RING ubiquitin ligase complex;GO:0005829|cytosol;	GO:0005262|calcium channel activity;GO:0004872|receptor	K10612	CUL5; cullin 5
SLC25A39	1470.28	0.396887	0.126106256	3.147245	0.0016482	0.016214	Not_Sig	no	gi|528996393|ref|XP_005220900.1|	1878	0	PREDICTED: solute carrier family 25 member 39 isoform X1 [Bos taurus]	gi|982958041|ref|XM_005220843.3|	1593	0	PREDICTED: Bos taurus solute carrier family 25 member 39 (SLC25A39), transcript variant X1, mRNA	Q17QI7	728	0	Solute carrier family 25 member 39	.	.	.	.	pfam00153	Mito_carr	Mitochondrial carrier protein.	GO:0006783|heme biosynthetic process;GO:0006412|translation;GO:0006810|transport;	GO:0016021|integral component of membrane;GO:0005743|mitochondrial inner membrane;	GO:0003735|structural constituent of ribosome;	K15119	SLC25A39_40; solute carrier family 25, member 39/40
ZDHHC21	227.1182	-0.50883	0.161730584	-3.14619	0.0016541	0.016258	Not_Sig	no	gi|970724959|ref|XP_015098177.1|	1409	1e-173	PREDICTED: palmitoyltransferase ZDHHC21 isoform X1 [Vicugna pacos]	gi|594075094|ref|XM_006061269.1|	6174	0	PREDICTED: Bubalus bubalis probable palmitoyltransferase ZDHHC21-like (LOC102404206), transcript variant X5, mRNA	A2VDT6	516	1e-164	Probable palmitoyltransferase ZDHHC21	SPBC2F12.15c	87.8	3.16e-17	COG5273	pfam01529	zf-DHHC	DHHC palmitoyltransferase. This family includes the well known DHHC zinc binding domain as well as three of the four conserved	GO:0001942|hair follicle development;GO:0018230|peptidyl-L-cysteine S-palmitoylation;GO:0048733|sebaceous gland development;	GO:0005794|Golgi apparatus;GO:0016021|integral component of membrane;GO:0005886|plasma membrane;	GO:0019706|protein-cysteine S-palmitoyltransferase activity;GO:0008270	K18932	ZDHHC; palmitoyltransferase [EC:2.3.1.225]

PIK3R2	332.4587	0.525691	0.167111403	3.145753	0.0016566	0.016264	Not_Sig	no	gi|27807041|ref|NP_777001.1|	2958	0	phosphatidylinositol 3-kinase regulatory subunit beta [Bos taurus]	gi|110347581|ref|NM_174576.2|	3578	0	Bos taurus phosphoinositide-3-kinase regulatory subunit 2 (PIK3R2), mRNA	P23726	1144	0	Phosphatidylinositol 3-kinase regulatory subunit beta	.	.	.	.	pfam00017	SH2	SH2 domain.	GO:0001678|cellular glucose homeostasis;GO:0032869|cellular response to insulin stimulus;GO:0008286|insulin receptor signaling pathway;GO:0014065|phosphatidylinositol 3-kinase signaling;GO:0046854|phosphatidylinositol phosphorylation;GO:0045785|positive regulation of cell adhesion;GO:2001275|positive regulation of glucose import in response to insulin stimulus;GO:0042993|positive regulation of transcription factor import into nucleus;GO:0045944|positive regulation of transcription from RNA polymerase II	GO:0005925|focal adhesion;GO:0005634|nucleus;GO:0005942|phosphatidylinositol 3-kinase complex;	GO:0016303|1-phosphatidylinositol-3-kinase activity;GO:0046935	K02649	PIK3R; phosphoinositide-3-kinase, regulatory subunit
OSTF1	1171.629	-0.33708	0.107159134	-3.14558	0.0016576	0.016264	Not_Sig	no	gi|594066650|ref|XP_006057292.1|	1074	1e-142	PREDICTED: osteoclast-stimulating factor 1 isoform X1 [Bubalus bubalis]	gi|742143677|ref|XM_010844895.1|	1327	0	PREDICTED: Bison bison bison osteoclast stimulating factor 1 (OSTF1), transcript variant X2, mRNA	Q8MJ50	375	2e-127	Osteoclast-stimulating factor 1	all1426	59.3	1.37e-09	COG0666	pfam12796	Ank_2	Ankyrin repeats (3 copies).	.	GO:0005737|cytoplasm;GO:0070062|extracellular exosome;	.	.	.
NAA60	706.4192	0.451877	0.143683753	3.144942	0.0016612	0.016286	Not_Sig	no	gi|115497156|ref|NP_001069117.1|	1293	3e-170	N-alpha-acetyltransferase 60 [Bos taurus]	gi|982966080|ref|XR_239937.3|	2385	0	PREDICTED: Bos taurus N(alpha)-acetyltransferase 60, NatF catalytic subunit (NAA60), transcript variant X1, misc_RNA	Q17QK9	502	9e-172	N-alpha-acetyltransferase 60	MJ1530	49.7	3.15e-06	COG0456	pfam00583	Acetyltransf_1	Acetyltransferase (GNAT) family. This family contains proteins with N-acetyltransferase functions such as Elp3-related proteins.	GO:0008283|cell proliferation;GO:0007059|chromosome segregation;GO:0043967|histone H4 acetylation;GO:0017196|N-terminal peptidyl-methionine acetylation;GO:0006334|nucleosome assembly;	GO:0000139|Golgi membrane;	GO:0010485|H4 histone acetyltransferase activity;GO:0004596	.	.
PTGDR	308.5153	0.473827	0.150688742	3.144406	0.0016642	0.016302	Not_Sig	no	gi|147900255|ref|NP_001091503.1|	1319	4e-162	prostaglandin D2 receptor [Bos taurus]	gi|555965084|ref|XM_005894541.1|	3032	0	PREDICTED: Bos mutus prostaglandin D2 receptor (DP) (PTGDR), mRNA	A5D7K8	512	1e-163	Prostaglandin D2 receptor	.	.	.	.	pfam00001	7tm_1	7 transmembrane receptor (rhodopsin family). This family contains, amongst other G-protein-coupled receptors	GO:0046085|adenosine metabolic process;GO:0071799|cellular response to prostaglandin D stimulus;GO:0006954|inflammatory response;GO:0030238|male sex determination;GO:0007204|positive regulation of cytosolic calcium ion concentration;GO:0045909|positive regulation of vasodilation;GO:0030431|sleep;	GO:0005887|integral component of plasma membrane;	GO:0016501|prostacyclin receptor activity;GO:0004956|prostaglandin D	K04332	PTGDR; prostaglandin D2 receptor
P4HA1	7612.122	-0.33877	0.107760472	-3.14377	0.0016679	0.016319	Not_Sig	no	gi|529010797|ref|XP_005226443.1|	2724	0	PREDICTED: prolyl 4-hydroxylase subunit alpha-1 isoform X1 [Bos taurus]	gi|926726414|ref|XM_005699184.2|	2907	0	PREDICTED: Capra hircus prolyl 4-hydroxylase, alpha polypeptide I (P4HA1), transcript variant X1, mRNA	Q1RMU3	1034	0	Prolyl 4-hydroxylase subunit alpha-1	.	.	.	.	pfam08336	P4Ha_N	Prolyl 4-Hydroxylase alpha-subunit, N-terminal region. The members of this family are eukaryotic proteins, and include all	.	GO:0005788|endoplasmic reticulum lumen;	GO:0005506|iron ion binding;GO:0031418|L-ascorbic acid	K00472	E1.14.11.2; prolyl 4-hydroxylase [EC:1.14.11.2]
QSOX2	760.4878	0.38363	0.122047944	3.143276	0.0016707	0.016319	Not_Sig	no	gi|297480848|ref|XP_002691662.1|	2861	0	PREDICTED: sulfhydryl oxidase 2 [Bos taurus]	gi|982991525|ref|XM_015465569.1|	2801	0	PREDICTED: Bos taurus quiescin sulfhydryl oxidase 2 (QSOX2), mRNA	Q6ZRP7	917	0	Sulfhydryl oxidase 2	.	.	.	.	pfam04777	Evr1_Alr	Erv1 / Alr family. Biogenesis of Fe/S clusters involves a number of essential mitochondrial proteins. Erv1p of Saccharomyces cerevisiae	GO:0045454|cell redox homeostasis;GO:0006457|protein folding;	GO:0005615|extracellular space;GO:0030173|integral component of Golgi membrane;GO:0031965|nuclear membrane;GO:0005886|plasma membrane;	GO:0016971|flavin-linked sulfhydryl oxidase activity;GO:0003756	K10758	QSOX; thiol oxidase [EC:1.8.3.2]
LOC138987987	3655.931	-0.36602	0.116446401	-3.14324	0.0016709	0.016319	Not_Sig	no	gi|555964763|ref|XP_005894445.1|	167	6e-10	PREDICTED: taste receptor type 2 member 42, partial [Bos mutus]	gi|982982633|ref|XR_001496509.1|	907	0	PREDICTED: Bos taurus uncharacterized LOC100847870 (LOC100847870), transcript variant X2, ncRNA	.	.	.	.	.	.	.	.	pfam07887	Calmodulin_bind	Calmodulin binding protein-like. The members of this family are putative or actual calmodulin binding proteins expressed by	.	.	.	.	.
ELOVL7	420.9029	-0.45009	0.143203051	-3.14305	0.0016719	0.016319	Not_Sig	no	gi|118151176|ref|NP_001071510.1|	1500	0	elongation of very long chain fatty acids protein 7 [Bos taurus]	gi|942036229|ref|XM_005889058.2|	3494	0	PREDICTED: Bos mutus ELOVL fatty acid elongase 7 (ELOVL7), transcript variant X1, mRNA	A0JNC4	582	0	Elongation of very long chain fatty acids protein 7	.	.	.	.	pfam01151	ELO	GNS1/SUR4 family. Members of this family are involved in long chain fatty acid elongation systems that produce the 26-carbon	GO:0034625|fatty acid elongation, monounsaturated fatty acid;GO:0034626|fatty acid elongation, polyunsaturated fatty acid;GO:0019367|fatty acid elongation, saturated fatty acid;GO:0035338|long-chain fatty-acyl-CoA biosynthetic process;GO:0030148|sphingolipid biosynthetic process;GO:0006636|unsaturated fatty acid biosynthetic process;GO:0042761|very long-chain fatty acid biosynthetic process;	GO:0005783|endoplasmic reticulum;GO:0030176|integral component of endoplasmic reticulum membrane;	GO:0009922|fatty acid elongase activity;	K10250	ELOVL7; elongation of very long chain fatty acids protein 7 [EC:2.3.1.199]
LOC138988276	191.6895	-0.61748	0.196472571	-3.14285	0.0016731	0.016319	DOWN	yes	gi|674087316|ref|XP_008851515.1|	308	6e-31	PREDICTED: protein kish-A isoform X1 [Nannospalax galili]	gi|742216647|ref|XM_010863377.1|	1162	0	PREDICTED: Bison bison bison transmembrane protein 167A (TMEM167A), mRNA	Q5RAL1	121	2e-32	Protein kish-A	.	.	.	.	pfam06842	DUF1242	Protein of unknown function (DUF1242). This family consists of a number of eukaryotic proteins of around 72 residues in length. The	.	GO:0000139|Golgi membrane;GO:0016021|integral component of membrane;	.	.	.
ZNF593	177.7286	-0.52075	0.16571558	-3.14242	0.0016756	0.016329	Not_Sig	no	gi|300794162|ref|NP_001179115.1|	699	8e-91	zinc finger protein 593 [Bos taurus]	gi|942054917|ref|XM_005895873.2|	652	0	PREDICTED: Bos mutus zinc finger protein 593 (ZNF593), mRNA	O00488	256	1e-85	Zinc finger protein 593	SPAC19B12.11c	80.9	5.71e-19	COG5112	pfam12171	zf-C2H2_jaz	Zinc-finger double-stranded RNA-binding. This domain family is found in archaea and eukaryotes, and is approximately 30 amino	GO:0000122|negative regulation of transcription from RNA polymerase II promoter;GO:0006351|transcription, DNA-templated;	GO:0005730|nucleolus;GO:0005634|nucleus;	GO:0003677|DNA binding;GO:0003714|transcription corepressor	K14821	BUD20; bud site selection protein 20
CCNK	558.9846	0.442104	0.140731434	3.141475	0.001681	0.016368	Not_Sig	no	gi|926719825|ref|XP_013828717.1|	1499	0	PREDICTED: cyclin-K [Capra hircus]	gi|982960751|ref|XM_010817262.2|	2581	0	PREDICTED: Bos taurus cyclin K (CCNK), transcript variant X1, mRNA	O75909	579	0	Cyclin-K	SPBC32F12.06	127	5.28e-31	COG5333	pfam00134	Cyclin_N	Cyclin, N-terminal domain. Cyclins regulate cyclin dependent kinases (CDKs). Human cyclin-O is a Uracil-DNA glycosylase that is related to	GO:0051301|cell division;GO:0006974|cellular response to DNA damage stimulus;GO:0007067|mitotic nuclear division;GO:0044828|negative regulation by host of viral genome replication;GO:0071157|negative regulation of cell cycle arrest;GO:0045737|positive regulation of cyclin-dependent protein serine/threonine kinase activity;GO:2001165|positive regulation of phosphorylation of RNA polymerase II C-terminal domain serine 2 residues;GO:0045944|positive regulation of transcription from RNA polymerase II promoter;GO:0000079|regulation of cyclin-	GO:0002944|cyclin K-CDK12 complex;GO:0002945|cyclin K-CDK13 complex;GO:0005654|nucleoplasm;GO:0005634|nucleus;	GO:0004693|cyclin-dependent protein serine/threonine kinase	.	.
PACS1	1381.385	0.491962	0.156645331	3.14061	0.001686	0.016403	Not_Sig	no	gi|219804842|ref|NP_001137342.1|	4135	0	phosphofurin acidic cluster sorting protein 1 [Bos taurus]	gi|594064973|ref|XM_006056425.1|	3959	0	PREDICTED: Bubalus bubalis phosphofurin acidic cluster sorting protein 1 (PACS1), transcript variant X1, mRNA	Q6VY07	1566	0	Phosphofurin acidic cluster sorting protein 1	.	.	.	.	pfam10254	Pacs-1	PACS-1 cytosolic sorting protein. PACS-1 is a cytosolic sorting protein that directs the localisation of membrane proteins in the trans-	GO:0000042|protein targeting to Golgi;GO:0072661|protein targeting to plasma membrane;GO:0050690|regulation of defense response to virus by virus;GO:0016032|viral process;	GO:0030137|COPI-coated vesicle;GO:0005829|cytosol;	GO:0044325|ion channel binding;	.	.
AEBP2	457.0513	-0.42868	0.136616008	-3.13786	0.0017019	0.016543	Not_Sig	no	gi|594071659|ref|XP_006059681.1|	1579	0	PREDICTED: zinc finger protein AEBP2-like [Bubalus bubalis]	gi|594071658|ref|XM_006059619.1|	3812	0	PREDICTED: Bubalus bubalis zinc finger protein AEBP2-like (LOC102410074), mRNA	A4FV57	610	0	Zinc finger protein AEBP2	.	.	.	.	pfam13465	zf-H2C2_2	Zinc-finger double domain.	GO:0016569|covalent chromatin modification;GO:0006351|transcription, DNA-templated;	GO:0035098|ESC/E(Z) complex;	GO:0046872|metal ion binding;GO:0000978|RNA polymerase II	K17452	AEBP2; zinc finger protein AEBP2
RPS9	4176.587	-0.36222	0.115490489	-3.13634	0.0017107	0.016616	Not_Sig	no	gi|465998371|gb|EMP40612.1|	935	4e-125	40S ribosomal protein S9 [Chelonia mydas]	gi|151554778|gb|BC148016.1|	698	0	Bos taurus ribosomal protein S9, mRNA (cDNA clone MGC:179248 IMAGE:7946025), complete cds	P29314	363	2e-126	40S ribosomal protein S9	SPBC29A3.12	284	1.22e-97	COG0522	pfam00163	Ribosomal_S4	Ribosomal protein S4/S9 N-terminal domain. This family includes small ribosomal subunit S9 from prokaryotes and S16 from	GO:0045903|positive regulation of translational fidelity;GO:0006412|translation;	GO:0022627|cytosolic small ribosomal subunit;GO:0030529|ribonucleoprotein complex;	GO:0003735|structural constituent of ribosome;	K02997	RP-S9e, RPS9; small subunit ribosomal protein S9e
ARL14EP	665.3319	-0.39832	0.127019339	-3.1359	0.0017132	0.016626	Not_Sig	no	gi|488509699|ref|XP_004446958.1|	1255	2e-169	PREDICTED: ARL14 effector protein [Dasypus novemcinctus]	gi|982946507|ref|XM_005216280.3|	1255	0	PREDICTED: Bos taurus ADP ribosylation factor like GTPase 14 effector protein (ARL14EP), transcript variant X1, mRNA	Q5EA92	483	1e-169	ARL14 effector protein	.	.	.	.	pfam14949	ARF7EP_C	ARF7 effector protein C-terminus. This family represents the C-terminus of the ARF7 effector protein (ARF7EP). ARF7EP interacts	.	GO:0005737|cytoplasm;	.	.	.
STK35	312.5881	0.494942	0.157856071	3.135403	0.0017162	0.01664	Not_Sig	no	gi|528973996|ref|XP_005214881.1|	2356	0	PREDICTED: serine/threonine-protein kinase 35 isoform X1 [Bos taurus]	gi|982943190|ref|XM_005214824.3|	6182	0	PREDICTED: Bos taurus serine/threonine kinase 35 (STK35), transcript variant X2, mRNA	Q8TDR2	836	0	Serine/threonine-protein kinase 35	.	.	.	.	pfam00069	Pkinase	Protein kinase domain.	.	GO:0005737|cytoplasm;GO:0005730|nucleolus;	GO:0005524|ATP binding;GO:0004674|protein serine/threonine	K08859	STK35, PDIK1, CLIK1; serine/threonine kinase 35 [EC:2.7.11.1]
CREBBP	1000.644	0.449318	0.143330402	3.134841	0.0017195	0.016658	Not_Sig	no	gi|255683523|ref|NP_001157494.1|	5814	0	CREB-binding protein [Bos taurus]	gi|255683522|ref|NM_001164022.1|	8397	0	Bos taurus CREB binding protein (CREBBP), mRNA	Q6JHU9	2105	0	CREB-binding protein	SPAC631.02	81.6	2.27e-14	COG5076	pfam08214	KAT11	Histone acetylation protein. Histone acetylation is required in many cellular processes including transcription, DNA repair, and	GO:0008283|cell proliferation;GO:0098609|cell-cell adhesion;GO:0034644|cellular response to UV;GO:0016573|histone acetylation;GO:0060355|positive regulation of cell adhesion molecule production;GO:1900087|positive regulation of G1/S transition of mitotic cell cycle;GO:1901224|positive regulation of NIK/NF-kappaB signaling;GO:0045944|positive regulation of transcription from RNA polymerase II promoter;GO:0045893|positive regulation of transcription, DNA-templated;GO:0006355|regulation of transcription, DNA-	GO:0005737|cytoplasm;GO:0000123|histone acetyltransferase complex;GO:0016604|nuclear body;GO:0005634|nucleus;GO:0043234|pro	GO:0016407|acetyltransferase activity;GO:0033613|activating	.	.
SNRPA	393.4072	0.441764	0.141010021	3.132855	0.0017311	0.016757	Not_Sig	no	gi|440910289|gb|ELR60098.1|	950	3e-123	U1 small nuclear ribonucleoprotein A, partial [Bos mutus]	gi|965969575|ref|XM_012189510.2|	1125	0	PREDICTED: Ovis aries small nuclear ribonucleoprotein polypeptide A (SNRPA), transcript variant X1, mRNA	Q2KIR1	360	5e-121	U1 small nuclear ribonucleoprotein A	.	.	.	.	pfam00076	RRM_1	RNA recognition motif. (a.k.a. RRM, RBD, or RNP domain). The RRM motif is probably diagnostic of an RNA binding protein. RRMs	GO:0000398|mRNA splicing, via spliceosome;	GO:0005737|cytoplasm;GO:0005730|nucleolus;GO:0005654|nucleoplasm;GO:0005681|spliceosomal complex;GO:0005685|U1 snRNP;	GO:0000166|nucleotide binding;GO:0044822|poly(A) RNA	K11091	SNRPA; U1 small nuclear ribonucleoprotein A
KIF20A	867.6929	0.383244	0.122381329	3.131552	0.0017388	0.016808	Not_Sig	no	gi|555959319|ref|XP_005891770.1|	4231	0	PREDICTED: kinesin-like protein KIF20A [Bos mutus]	gi|942043231|ref|XM_005891708.2|	2748	0	PREDICTED: Bos mutus kinesin family member 20A (KIF20A), mRNA	Q29RT6	1629	0	Kinesin-like protein KIF20A	YGL216w	113	9.90e-25	COG5059	pfam00225	Kinesin	Kinesin motor domain.	GO:0000920|cell separation after cytokinesis;GO:0000910|cytokinesis;GO:0001578|microtubule bundle formation;GO:0007018|microtubule-based movement;GO:0000281|mitotic cytokinesis;GO:0015031|protein transport;	GO:0005794|Golgi apparatus;GO:0005871|kinesin complex;GO:0005874|microtubule;GO:0030496|midbody;GO:0005819|spindle;	GO:0005524|ATP binding;GO:0016887|ATPase activity;GO:0003777	K10402	KIF20; kinesin family member 20
MED12	863.8256	0.449309	0.143491783	3.131253	0.0017406	0.016808	Not_Sig	no	gi|803275959|ref|XP_011997650.1|	9880	0	PREDICTED: mediator of RNA polymerase II transcription subunit 12 isoform X2 [Ovis aries musimon]	gi|982973720|ref|XM_005228019.3|	6863	0	PREDICTED: Bos taurus mediator complex subunit 12 (MED12), transcript variant X1, mRNA	Q5RCU2	3629	0	Mediator of RNA polymerase II transcription subunit 12	.	.	.	.	pfam12145	Med12-LCEWAV	Eukaryotic Mediator 12 subunit domain. This domain is found in eukaryotes, and is typically between 325 and 354 amino acids	GO:0006351|transcription, DNA-templated;	GO:0016592|mediator complex;	GO:0001104|RNA polymerase II transcription cofactor activity;	.	.
RSBN1	365.9939	-0.47392	0.1513594	-3.13109	0.0017416	0.016808	Not_Sig	no	gi|803260628|ref|XP_011990050.1|	3474	0	PREDICTED: round spermatid basic protein 1 [Ovis aries musimon]	gi|742231915|ref|XM_010830784.1|	6474	0	PREDICTED: Bison bison bison round spermatid basic protein 1 (RSBN1), mRNA	Q5VWQ0	1277	0	Round spermatid basic protein 1	.	.	.	.	pfam12410	rpo30_N	Poxvirus DNA dependent RNA polymerase 30kDa subunit. This family of proteins is found in viruses. Proteins in this family are	.	GO:0005634|nucleus;	.	.	.
CSF1	4221.505	0.350739	0.112021924	3.130985	0.0017422	0.016808	Not_Sig	no	gi|742232317|ref|XP_010829190.1|	2439	0	PREDICTED: macrophage colony-stimulating factor 1 isoform X1 [Bison bison bison]	gi|742232316|ref|XM_010830888.1|	3809	0	PREDICTED: Bison bison bison colony stimulating factor 1 (macrophage) (CSF1), transcript variant X1, mRNA	P09603	665	0	Macrophage colony-stimulating factor 1	.	.	.	.	pfam05337	CSF-1	Macrophage colony stimulating factor-1 (CSF-1). Colony stimulating factor 1 (CSF-1) is a homodimeric polypeptide growth	GO:0060444|branching involved in mammary gland duct morphogenesis;GO:0030154|cell differentiation;GO:0008283|cell proliferation;GO:0003006|developmental process involved in reproduction;GO:0030097|hemopoiesis;GO:0048873|homeostasis of number of cells within a tissue;GO:0006954|inflammatory response;GO:0045087|innate immune response;GO:0038145|macrophage colony-stimulating factor signaling pathway;GO:0030225|macrophage differentiation;GO:0060763|mammary duct terminal end bud	GO:1990682|CSF1-CSF1R complex;GO:0070062|extracellular exosome;GO:0005615|extracellular space;GO:0016021|integral component of	GO:0005125|cytokine activity;GO:0008083|growth factor	K05453	CSF1, MCSF; macrophage colony-stimulating factor 1
GGA3	538.5708	0.425574	0.135960072	3.130141	0.0017472	0.016842	Not_Sig	no	gi|942092042|ref|XP_014337745.1|	3113	0	PREDICTED: ADP-ribosylation factor-binding protein GGA3 isoform X1 [Bos mutus]	gi|942092041|ref|XM_014482259.1|	3760	0	PREDICTED: Bos mutus golgi-associated, gamma adaptin ear containing, ARF binding protein 3 (GGA3), transcript variant X1, mRNA	Q9NZ52	937	0	ADP-ribosylation factor-binding protein GGA3	.	.	.	.	pfam00790	VHS	VHS domain. Domain present in VPS-27, Hrs and STAM.	GO:0044267|cellular protein metabolic process;GO:0006886|intracellular protein transport;GO:0045732|positive regulation of protein catabolic process;GO:0016192|vesicle-mediated transport;	GO:0030131|clathrin adaptor complex;GO:0010008|endosome membrane;GO:0005802|trans-Golgi network;	GO:0030306|ADP-ribosylation factor binding;	K12404	GGA; ADP-ribosylation factor-binding protein GGA
KIF2C	416.487	0.404544	0.129266012	3.129544	0.0017508	0.016856	Not_Sig	no	gi|440907281|gb|ELR57441.1|	3495	0	Kinesin-like protein KIF2C, partial [Bos mutus]	gi|555964350|ref|XM_005894183.1|	2803	0	PREDICTED: Bos mutus kinesin family member 2C (KIF2C), transcript variant X1, mRNA	Q99661	1238	0	Kinesin-like protein KIF2C	ECU11g0470	230	9.19e-65	COG5059	pfam00225	Kinesin	Kinesin motor domain.	GO:0019886|antigen processing and presentation of exogenous peptide antigen via MHC class II;GO:0051315|attachment of mitotic spindle microtubules to kinetochore;GO:0051301|cell division;GO:0008283|cell proliferation;GO:0030951|establishment or maintenance of microtubule cytoskeleton polarity;GO:0051310|metaphase plate congression;GO:0007019|microtubule depolymerization;GO:0007018|microtubule-based movement;GO:0007080|mitotic metaphase plate congression;GO:0007067|mitotic nuclear	GO:0000775|chromosome, centromeric region;GO:0000777|condensed chromosome kinetochore;GO:0005829|cytosol;GO:00058	GO:0005524|ATP binding;GO:0016887|ATPase activity;GO:0019237	K10393	KIF2_24, MCAK; kinesin family member 2/24
ATP5IF1	891.9832	-0.40308	0.128802659	-3.12941	0.0017516	0.016856	Not_Sig	no	gi|28461251|ref|NP_787010.1|	484	2e-59	ATPase inhibitor, mitochondrial precursor [Bos taurus]	gi|742229399|ref|XM_010830162.1|	520	0	PREDICTED: Bison bison bison ATPase inhibitory factor 1 (ATPIF1), mRNA	P01096	191	8e-61	ATPase inhibitor, mitochondrial	.	.	.	.	pfam04568	IATP	Mitochondrial ATPase inhibitor, IATP. ATP synthase inhibitor prevents the enzyme from switching to ATP hydrolysis during	GO:0030218|erythrocyte differentiation;GO:0006783|heme biosynthetic process;GO:0032780|negative regulation of ATPase activity;GO:0001937|negative regulation of endothelial cell proliferation;GO:0051346|negative regulation of hydrolase activity;GO:0051260|protein homooligomerization;GO:0051289|protein homotetramerization;	GO:0009986|cell surface;GO:0005739|mitochondrion;	GO:0043532|angiostatin binding;GO:0051117|ATPase	.	.
HIPK2	488.5003	0.399839	0.127798251	3.128676	0.001756	0.016862	Not_Sig	no	gi|803269922|ref|XP_011994680.1|	5542	0	PREDICTED: homeodomain-interacting protein kinase 2 isoform X2 [Ovis aries musimon]	gi|926690744|ref|XM_013963531.1|	9322	0	PREDICTED: Capra hircus homeodomain interacting protein kinase 2 (HIPK2), transcript variant X1, mRNA	Q9H2X6	2070	0	Homeodomain-interacting protein kinase 2	.	.	.	.	pfam00069	Pkinase	Protein kinase domain.	GO:0007628|adult walking behavior;GO:0009952|anterior/posterior pattern specification;GO:0071456|cellular response to hypoxia;GO:0006978|DNA damage response, signal transduction by p53 class mediator resulting in transcription of p21 class mediator;GO:0048596|embryonic camera-type eye morphogenesis;GO:0060059|embryonic retina morphogenesis in camera-type eye;GO:0030218|erythrocyte differentiation;GO:0001654|eye development;GO:0097193|intrinsic apoptotic signaling	GO:0005737|cytoplasm;GO:0016604|nuclear body;GO:0005654|nucleoplasm;GO:0005634|nucleus;GO:0016605|PML	GO:0005524|ATP binding;GO:0004672|protein kinase activity;GO:0004674	K08826	HIPK; homeodomain interacting protein kinase [EC:2.7.11.1]
ABHD17A	759.8402	0.466978	0.149259426	3.128635	0.0017562	0.016862	Not_Sig	no	gi|594052594|ref|XP_006050610.1|	1415	0	PREDICTED: alpha/beta hydrolase domain-containing protein 17A [Bubalus bubalis]	gi|114052371|ref|NM_001046536.1|	1438	0	Bos taurus abhydrolase domain containing 17A (ABHD17A), mRNA	Q2HJ19	548	0	Protein ABHD17A	all8511	145	2.16e-39	COG1073	pfam12695	Abhydrolase_5	Alpha/beta hydrolase family. This family contains a diverse range of alpha/beta hydrolase enzymes.	.	GO:0005576|extracellular region;	GO:0016787|hydrolase activity;	.	.
CD63	8405.056	-0.33237	0.106237432	-3.12856	0.0017567	0.016862	Not_Sig	no	gi|440897260|gb|ELR48992.1|	1095	5e-148	CD63 antigen, partial [Bos mutus]	gi|74354662|gb|BC102657.1|	856	0	Bos taurus CD63 molecule, mRNA (cDNA clone MGC:127997 IMAGE:30956986), complete cds	Q9XSK2	418	2e-146	CD63 antigen	.	.	.	.	pfam00335	Tetraspannin	Tetraspanin family.	GO:0007155|cell adhesion;GO:0016477|cell migration;GO:0007166|cell surface receptor signaling pathway;GO:0007160|cell-matrix adhesion;GO:0035646|endosome to melanosome transport;GO:0048757|pigment granule maturation;GO:0045807|positive regulation of endocytosis;GO:2001046|positive regulation of integrin-mediated signaling pathway;GO:0002092|positive regulation of receptor internalization;GO:0015031|protein transport;GO:1900746|regulation of vascular endothelial growth factor signaling pathway;	GO:0012505|endomembrane system;GO:0070062|extracellular exosome;GO:0005887|integral component of plasma membrane;GO:0031226|intrinsic	.	K06497	CD63, MLA1, TSPAN30; CD63 antigen
ZHX3	671.7135	0.41477	0.132593491	3.128133	0.0017592	0.016873	Not_Sig	no	gi|942038445|ref|XP_014331874.1|	4285	0	PREDICTED: zinc fingers and homeoboxes protein 3 [Bos mutus]	gi|982993797|ref|XM_010822631.2|	8628	0	PREDICTED: Bos taurus zinc fingers and homeoboxes 3 (ZHX3), transcript variant X6, mRNA	Q9H4I2	1417	0	Zinc fingers and homeoboxes protein 3	.	.	.	.	pfam11569	Homez	Homeodomain leucine-zipper encoding, Homez. Homez contains two leucine zipper-like motifs and an acidic domain and belongs to	GO:0030154|cell differentiation;GO:0000122|negative regulation of transcription from RNA polymerase II promoter;GO:0045892|negative regulation of transcription, DNA-templated;GO:0045669|positive regulation of osteoblast differentiation;GO:0006351|transcription, DNA-templated;	GO:0005654|nucleoplasm;GO:0005634|nucleus;	GO:0003677|DNA binding;GO:0046872|metal ion binding;GO:0046982	.	.
MMADHC	1560.987	-0.33702	0.107775459	-3.12709	0.0017655	0.016918	Not_Sig	no	gi|555966139|ref|XP_005895125.1|	1533	0	PREDICTED: methylmalonic aciduria and homocystinuria type D protein, mitochondrial isoform X1 [Bos mutus]	gi|942052739|ref|XM_005895063.2|	1353	0	PREDICTED: Bos mutus methylmalonic aciduria (cobalamin deficiency) cblD type, with homocystinuria (MMADHC), transcript variant X1, mRNA	.	.	.	.	.	.	.	.	pfam10229	DUF2246	Uncharacterized conserved protein (DUF2246). This is a family of proteins conserved from worms to humans of approximately 300	.	.	.	.	.
SORBS3	1593.177	0.438052	0.140226841	3.123882	0.0017848	0.01709	Not_Sig	no	gi|528959116|ref|XP_005210295.1|	3385	0	PREDICTED: vinexin isoform X1 [Bos taurus]	gi|982933832|ref|XM_005210238.3|	3205	0	PREDICTED: Bos taurus sorbin and SH3 domain containing 3 (SORBS3), transcript variant X1, mRNA	O60504	1017	0	Vinexin	.	.	.	.	pfam00018	SH3_1	SH3 domain. SH3 (Src homology 3) domains are often indicative of a protein involved in signal transduction related to	GO:0007015|actin filament organization;GO:0007155|cell adhesion;GO:0031589|cell-substrate adhesion;GO:0006936|muscle contraction;GO:0000122|negative regulation of transcription from RNA polymerase II promoter;GO:0051495|positive regulation of cytoskeleton organization;GO:0043410|positive regulation of MAPK cascade;GO:0051496|positive regulation of stress fiber assembly;	GO:0005856|cytoskeleton;GO:0005829|cytosol;GO:0005925|focal adhesion;GO:0005634|nucleus;	GO:0005200|structural constituent of cytoskeleton;GO:0017166|vinculin	.	.
FBLIM1	2286.928	0.413868	0.132524198	3.122959	0.0017904	0.017129	Not_Sig	no	gi|528980307|ref|XP_005217192.1|	1542	0	PREDICTED: filamin-binding LIM protein 1 isoform X1 [Bos taurus]	gi|742164924|ref|XM_010850036.1|	2741	0	PREDICTED: Bison bison bison filamin binding LIM protein 1 (FBLIM1), transcript variant X2, mRNA	Q1JQB5	548	0	Filamin-binding LIM protein 1	.	.	.	.	pfam00412	LIM	LIM domain. This family represents two copies of the LIM structural domain.	GO:0008360|regulation of cell shape;GO:0033623|regulation of integrin activation;GO:0016337|single organismal cell-cell adhesion;	GO:0005938|cell cortex;GO:0005925|focal adhesion;GO:0001725|stress fiber;	GO:0031005|filamin binding;GO:0008270|zinc ion binding;	.	.
PLOD3	4209.54	0.374096	0.119820672	3.122134	0.0017954	0.017163	Not_Sig	no	gi|555961542|ref|XP_005892865.1|	3703	0	PREDICTED: procollagen-lysine,2-oxoglutarate 5-dioxygenase 3 [Bos mutus]	gi|300795071|ref|NM_001193255.1|	2849	0	Bos taurus procollagen-lysine, 2-oxoglutarate 5-dioxygenase 3 (PLOD3), mRNA	Q5R6K5	1342	0	Procollagen-lysine,2-oxoglutarate 5-dioxygenase 3	.	.	.	.	pfam03171	2OG-FeII_Oxy	2OG-Fe(II) oxygenase superfamily. This family contains members of the 2-oxoglutarate (2OG) and Fe(II)-dependent oxygenase	.	GO:0030867|rough endoplasmic reticulum membrane;	GO:0005506|iron ion binding;GO:0031418|L-ascorbic acid	K13646	PLOD3; lysyl hydroxylase/galactosyltransferase/glucosyltransferase
LOC106700672	498.2227	-0.45212	0.144942607	-3.11927	0.001813	0.017316	Not_Sig	no	gi|296488377|tpg|DAA30490.1|	289	3e-30	TPA: hypothetical protein BOS_4392 [Bos taurus]	gi|742224915|ref|XR_824254.1|	379	0	PREDICTED: Bison bison bison uncharacterized LOC104980366 (LOC104980366), ncRNA	.	.	.	.	.	.	.	.	pfam13255	DUF4046	Protein of unknown function (DUF4046). This family of proteins is functionally uncharacterized.	.	.	.	.	.
S100A14	2247.218	-0.32363	0.103773227	-3.1186	0.0018171	0.017341	Not_Sig	no	gi|118601864|ref|NP_001073102.1|	542	1e-64	protein S100-A14 [Bos taurus]	gi|742111950|ref|XM_010838411.1|	1300	0	PREDICTED: Bison bison bison S100 calcium binding protein A14 (S100A14), transcript variant X1, mRNA	Q3MHP3	213	4e-66	Protein S100-A14	.	.	.	.	pfam01023	S_100	This family of proteins is found in S-100/ICaBP type calcium binding domain. The S-100 domain is a subfamily of the EF-hand calcium binding proteins.	GO:0006915|apoptotic process;GO:0042742|defense response to bacterium;GO:0071624|positive regulation of granulocyte chemotaxis;GO:0090026|positive regulation of monocyte chemotaxis;GO:0032496|response to lipopolysaccharide;GO:0034142|toll-like receptor 4 signaling pathway;	GO:0030054|cell junction;GO:0070062|extracellular exosome;GO:0015630|microtubule cytoskeleton;GO:0005654|nucleoplasm;GO:	GO:0005509|calcium ion binding;	.	.
ST3GAL6	2169.79	-0.49981	0.160281345	-3.11833	0.0018188	0.017343	Not_Sig	no	gi|440905195|gb|ELR55611.1|	1800	0	Type 2 lactosamine alpha-2,3-sialyltransferase, partial [Bos mutus]	gi|742132979|ref|XR_825456.1|	3014	0	PREDICTED: Bison bison bison ST3 beta-galactoside alpha-2,3-sialyltransferase 6 (ST3GAL6), transcript variant X2, misc_RNA	Q6H8M7	690	0	Type 2 lactosamine alpha-2,3-sialyltransferase	.	.	.	.	pfam00777	Glyco_transf_29	Glycosyltransferase family 29 (sialyltransferase). Members of this family belong to glycosyltransferase family 29.	GO:0006464|cellular protein modification process;GO:0071354|cellular response to interleukin-6;GO:0001574|ganglioside biosynthetic process;GO:0006664|glycolipid metabolic process;GO:0009311|oligosaccharide metabolic process;GO:0018279|protein N-linked glycosylation via asparagine;	GO:0030173|integral component of Golgi membrane;	GO:0003836|beta-galactoside (CMP) alpha-2,3-sialyltransferase	K03792	SIAT10, ST3GAL6; beta-galactoside alpha-2,3-sialyltransferase
SLC41A1	1893.962	0.41099	0.131811835	3.118002	0.0018208	0.017345	Not_Sig	no	gi|329664930|ref|NP_001192965.1|	2521	0	solute carrier family 41 member 1 [Bos taurus]	gi|594057578|ref|XM_006052903.1|	4614	0	PREDICTED: Bubalus bubalis solute carrier family 41 (magnesium transporter), member 1 (SLC41A1), transcript variant X1, mRNA	Q5R839	933	0	Solute carrier family 41 member 1	MA1437	66.2	4.30e-11	COG1824	pfam01769	MgtE	Divalent cation transporter. This region is the integral membrane part of the eubacterial MgtE family of magnesium transporters.	GO:0010961|cellular magnesium ion homeostasis;GO:0071286|cellular response to magnesium ion;GO:0015693|magnesium ion transport;	GO:0016323|basolateral plasma membrane;GO:0016021|integral component of membrane;GO:0043234|protein complex;	GO:0015491|cation:cation antiporter activity;GO:0015095|magnesium ion	K15122	SLC41A; solute carrier family 41
RPS15A	3161.589	-0.42272	0.135591192	-3.11759	0.0018234	0.017345	Not_Sig	no	gi|927224215|ref|XP_005658675.2|	710	1e-90	PREDICTED: 40S ribosomal protein S15a [Sus scrofa]	gi|966002765|ref|XM_004020794.3|	861	0	PREDICTED: Ovis aries ribosomal protein S15a (RPS15A), transcript variant X2, mRNA	P62246	268	2e-88	40S ribosomal protein S15a	YJL190c	218	7.61e-71	COG0096	pfam00410	Ribosomal_S8	Ribosomal protein S8.	GO:0045787|positive regulation of cell cycle;GO:0008284|positive regulation of cell proliferation;GO:0009615|response to virus;GO:0006412|translation;	GO:0022627|cytosolic small ribosomal subunit;GO:0070062|extracellular exosome;GO:0016020|membrane;GO:0005739|mitochondrion;	GO:0044822|poly(A) RNA binding;GO:0003735|structural	K02957	RP-S15Ae, RPS15A; small subunit ribosomal protein S15Ae
NR1D1	279.5221	0.542829	0.17411969	3.11756	0.0018236	0.017345	Not_Sig	no	gi|594090513|ref|XP_006068658.1|	2738	0	PREDICTED: nuclear receptor subfamily 1 group D member 1 [Bubalus bubalis]	gi|742135489|ref|XM_010842620.1|	2742	0	PREDICTED: Bison bison bison nuclear receptor subfamily 1, group D, member 1 (NR1D1), mRNA	Q08E02	1059	0	Nuclear receptor subfamily 1 group D member 1	.	.	.	.	pfam00105	zf-C4	Zinc finger, C4 type (two domains). In nearly all cases, this is the DNA binding domain of a nuclear hormone receptor. The alignment	GO:0030154|cell differentiation;GO:0071222|cellular response to lipopolysaccharide;GO:0032922|circadian regulation of gene expression;GO:0060086|circadian temperature homeostasis;GO:0005978|glycogen biosynthetic process;GO:0010871|negative regulation of receptor biosynthetic process;GO:0034144|negative regulation of toll-like receptor 4 signaling pathway;GO:0045892|negative regulation of transcription, DNA-templated;GO:0070859|positive regulation of bile acid biosynthetic process;GO:0045893|positive regulation of	GO:0005737|cytoplasm;GO:0030425|dendrite;GO:0043197|dendritic spine;GO:0000790|nuclear chromatin;GO:0005634|nucleus;	GO:0001046|core promoter sequence-specific DNA binding;GO:0020037	K03728	NR1D1; nuclear receptor subfamily 1 group D member 1

RBM3	3355.523	-0.32411	0.103993154	-3.11664	0.0018292	0.017384	Not_Sig	no	gi|676266860|gb|KFO22699.1|	444	5e-51	Putative RNA-binding protein 3 [Fukomys damarensis]	gi|942032495|ref|XM_005887675.2|	774	0	PREDICTED: Bos mutus RNA binding motif (RNP1, RRM) protein 3 (RBM3), transcript variant X1, mRNA	W8E7I1	171	2e-51	RNA-binding protein 3	TP0356	71.6	2.85e-15	COG0724	pfam00076	RRM_1	RNA recognition motif. (a.k.a. RRM, RBD, or RNP domain). The RRM motif is probably diagnostic of an RNA binding protein. RRMs	GO:0070417|cellular response to cold;GO:0009631|cold acclimation;GO:0045727|positive regulation of translation;GO:0006417|regulation of translation;GO:0009409|response to cold;	GO:0005737|cytoplasm;GO:0030425|dendrite;GO:0005634|nucleus;	GO:0000166|nucleotide binding;GO:0043023|ribosomal large	K13186	RBM3; RNA-binding protein 3
SPAG5	447.2882	0.38795	0.124486085	3.116415	0.0018306	0.017384	Not_Sig	no	gi|555952119|ref|XP_005888249.1|	5526	0	PREDICTED: sperm-associated antigen 5 [Bos mutus]	gi|742208657|ref|XM_010861417.1|	3727	0	PREDICTED: Bison bison bison sperm associated antigen 5 (SPAG5), mRNA	Q96R06	1559	0	Sperm-associated antigen 5	.	.	.	.	pfam02463	SMC_N	RecF/RecN/SMC N terminal domain. This domain is found at the N terminus of SMC proteins. The SMC (structural maintenance	GO:0051301|cell division;GO:0007059|chromosome segregation;GO:0051294|establishment of spindle orientation;GO:0000070|mitotic sister chromatid segregation;GO:0071539|protein localization to centrosome;GO:0051988|regulation of attachment of spindle microtubules to kinetochore;GO:0007051|spindle organization;	GO:0034451|centriolar satellite;GO:0000777|condensed chromosome kinetochore;GO:0005737|cytoplasm;GO:00	.	.	.
CLN8	183.4033	0.662639	0.212694153	3.115452	0.0018366	0.017414	UP	yes	gi|347360932|ref|NP_001069409.2|	1316	1e-176	protein CLN8 [Bos taurus]	gi|942036514|ref|XM_014483732.1|	1625	0	PREDICTED: Bos mutus ceroid-lipofuscinosis, neuronal 8 (CLN8), mRNA	Q5JZQ7	396	5e-133	Protein CLN8	.	.	.	.	pfam03798	TRAM_LAG1_CLN8	TLC domain.	GO:0007628|adult walking behavior;GO:0001306|age-dependent response to oxidative stress;GO:0008306|associative learning;GO:0044257|cellular protein catabolic process;GO:0006672|ceramide metabolic process;GO:0008203|cholesterol metabolic process;GO:0051935|glutamate reuptake;GO:0007040|lysosome organization;GO:0007006|mitochondrial membrane organization;GO:0050881|musculoskeletal movement;GO:0043066|negative regulation of apoptotic process;GO:0007399|nervous system	GO:0005783|endoplasmic reticulum;GO:0005789|endoplasmic reticulum membrane;GO:0005793|endoplasmic	.	K12360	CLN8; ceroid-lipofuscinosis neuronal protein 8
ACIN1	2333.681	0.330079	0.105950921	3.1154	0.001837	0.017414	Not_Sig	no	gi|358413983|ref|XP_003582712.1|	4378	0	PREDICTED: apoptotic chromatin condensation inducer in the nucleus isoform X1 [Bos taurus]	gi|982988738|ref|XM_003582664.4|	4611	0	PREDICTED: Bos taurus apoptotic chromatin condensation inducer 1 (ACIN1), transcript variant X1, mRNA	Q9UKV3	1512	0	Apoptotic chromatin condensation inducer in the nucleus	.	.	.	.	pfam02037	SAP	SAP domain. The SAP (after SAF-A/B, Acinus and PIAS) motif is a putative DNA/RNA binding domain found in diverse nuclear and	GO:0030263|apoptotic chromosome condensation;GO:0006921|cellular component disassembly involved in execution phase of apoptosis;GO:0030218|erythrocyte differentiation;GO:0006397|mRNA processing;GO:0048025|negative regulation of mRNA splicing, via spliceosome;GO:0043065|positive regulation of apoptotic process;GO:0045657|positive regulation of monocyte differentiation;GO:0008380|RNA splicing;	GO:0061574|ASAP complex;GO:0005737|cytoplasm;GO:0005829|cytosol;GO:0016607|nuclear speck;GO:0005730|nucleolus;GO:0005654|	GO:0016887|ATPase activity;GO:0019899|enzyme	K12875	ACIN1, ACINUS; apoptotic chromatin condensation inducer in the
LOC102277803	55.30799	-0.81431	0.261403193	-3.11515	0.0018385	0.017414	DOWN	yes	gi|296474336|tpg|DAA16451.1|	1197	2e-161	TPA: BOLA class II histocompatibility antigen, DQB*0101 beta chain precursor [Bos taurus]	gi|402746714|ref|NM_001034668.3|	1055	0	Bos taurus MHC class II antigen (BLA-DQB), mRNA	Q9TVC8	456	2e-159	BoLa class II histocompatibility antigen, DQB*0101 beta chain	.	.	.	.	pfam00969	MHC_II_beta	Class II histocompatibility antigen, beta domain.	GO:0002504|antigen processing and presentation of peptide or polysaccharide antigen via MHC class II;GO:0006955|immune response;	GO:0016021|integral component of membrane;GO:0042613|MHC class II protein complex;	.	.	.
JKAMP	659.7723	-0.35878	0.115181853	-3.11493	0.0018399	0.017414	Not_Sig	no	gi|528966724|ref|XP_005212012.1|	1507	0	PREDICTED: JNK1/MAPK8-associated membrane protein isoform X3 [Bos taurus]	gi|742156543|ref|XM_010847672.1|	1809	0	PREDICTED: Bison bison bison JNK1/MAPK8-associated membrane protein (JKAMP), transcript variant X1, mRNA	Q8BI36	572	0	JNK1/MAPK8-associated membrane protein	.	.	.	.	pfam05571	DUF766	Protein of unknown function (DUF766). This family consists of several eukaryotic proteins of unknown function.	GO:0030433|ER-associated ubiquitin-dependent protein catabolic process;GO:0006986|response to unfolded protein;	GO:0005789|endoplasmic reticulum membrane;GO:0016021|integral component of membrane;	GO:0031625|ubiquitin protein ligase binding;	.	.
NDUFB4	351.1948	-0.49707	0.15959604	-3.11458	0.0018421	0.01742	Not_Sig	no	gi|742148472|ref|XP_010843692.1|	674	5e-88	PREDICTED: NADH dehydrogenase [ubiquinone] 1 beta subcomplex subunit 4 [Bison bison bison]	gi|742148471|ref|XM_010845390.1|	472	0	PREDICTED: Bison bison bison NADH dehydrogenase (ubiquinone) 1 beta subcomplex, 4, 15kDa (NDUFB4), mRNA	P48305	263	4e-89	NADH dehydrogenase [ubiquinone] 1 beta subcomplex subunit 4	.	.	.	.	pfam07225	NDUF_B4	NADH-ubiquinone oxidoreductase B15 subunit (NDUFB4). This family consists of several NADH-ubiquinone oxidoreductase B15	GO:0006979|response to oxidative stress;	GO:0016021|integral component of membrane;GO:0005747|mitochondrial respiratory chain complex I;	GO:0008137|NADH dehydrogenase (ubiquinone) activity;	K03960	NDUFB4; NADH dehydrogenase (ubiquinone) 1 beta subcomplex subunit
AIMP1	597.6493	-0.45178	0.145083285	-3.11395	0.001846	0.017443	Not_Sig	no	gi|555950325|ref|XP_005887365.1|	1494	0	PREDICTED: aminoacyl tRNA synthase complex-interacting multifunctional protein 1 [Bos mutus]	gi|942031112|ref|XM_005887303.2|	1178	0	PREDICTED: Bos mutus aminoacyl tRNA synthetase complex-interacting multifunctional protein 1 (AIMP1), mRNA	Q12904	512	1e-180	Aminoacyl tRNA synthase complex-interacting multifunctional protein 1	YGL105w	163	7.19e-46	COG0073	pfam01588	tRNA_bind	Putative tRNA binding domain. This domain is found in prokaryotic methionyl-tRNA synthetases, prokaryotic phenylalanyl tRNA	GO:0001525|angiogenesis;GO:0006915|apoptotic process;GO:0007155|cell adhesion;GO:0007267|cell-cell signaling;GO:0006935|chemotaxis;GO:0051607|defense response to virus;GO:0006006|glucose metabolic process;GO:0006954|inflammatory response;GO:0050900|leukocyte migration;GO:0001937|negative regulation of endothelial cell proliferation;GO:0009611|response to wounding;GO:0007165|signal transduction;GO:0006418|tRNA aminoacylation for protein translation;	GO:0017101|aminoacyl-tRNA synthetase multienzyme complex;GO:0009986|cell surface;GO:0005737|cytoplasm;GO:0005829|cytosol;GO:0005783|endoplasmic	GO:0005125|cytokine activity;GO:0051020|GTPase	K15437	AIMP1, ARC1; aminoacyl tRNA synthase complex-interacting
MAPK7	592.4953	0.517236	0.166295569	3.11034	0.0018687	0.017643	Not_Sig	no	gi|149944747|ref|NP_001092550.1|	2479	0	mitogen-activated protein kinase 7 [Bos taurus]	gi|965823707|ref|XM_012127386.2|	3313	0	PREDICTED: Ovis aries musimon mitogen-activated protein kinase 7 (MAPK7), transcript variant X2, mRNA	A5PKJ4	959	0	Mitogen-activated protein kinase 7	.	.	.	.	pfam00069	Pkinase	Protein kinase domain.	GO:0007049|cell cycle;GO:0030154|cell differentiation;GO:0070301|cellular response to hydrogen peroxide;GO:0071499|cellular response to laminar fluid shear stress;GO:0071560|cellular response to transforming growth factor beta stimulus;GO:2000352|negative regulation of endothelial cell apoptotic process;GO:0070377|negative regulation of ERK5 cascade;GO:2001240|negative regulation of extrinsic apoptotic signaling pathway in absence of ligand;GO:0034115|negative regulation of heterotypic cell-cell adhesion;GO:0043407|negative regulation of	GO:0005737|cytoplasm;GO:0005829|cytosol;GO:0005634|nucleus;GO:0016605|PML body;	GO:0005524|ATP binding;GO:0004707|MAP kinase activity;	K04464	MAPK7; mitogen-activated protein kinase 7 [EC:2.7.11.24]
SALL2	76.88805	0.711152	0.22870185	3.109518	0.0018739	0.017678	UP	yes	gi|742151509|ref|XP_010844552.1|	4396	0	PREDICTED: sal-like protein 2 [Bison bison bison]	gi|742151508|ref|XM_010846250.1|	4084	0	PREDICTED: Bison bison bison spalt-like transcription factor 2 (SALL2), mRNA	Q9Y467	1310	0	Sal-like protein 2	.	.	.	.	pfam13465	zf-H2C2_2	Zinc-finger double domain.	GO:0001654|eye development;GO:0000122|negative regulation of transcription from RNA polymerase II promoter;GO:0021915|neural tube development;GO:0007165|signal transduction;GO:0006366|transcription from RNA polymerase II promoter;	GO:0005634|nucleus;GO:0016581|NuRD complex;	GO:0046872|metal ion binding;GO:0043565|sequence-specific	.	.
DGKD	548.9461	0.372177	0.119706369	3.109085	0.0018767	0.017681	Not_Sig	no	gi|164259356|ref|NP_001030359.2|	5646	0	diacylglycerol kinase delta [Bos taurus]	gi|164259355|ref|NM_001035282.2|	4784	0	Bos taurus diacylglycerol kinase delta (DGKD), mRNA	Q16760	2050	0	Diacylglycerol kinase delta	.	.	.	.	pfam00609	DAGK_acc	Diacylglycerol kinase accessory domain. Diacylglycerol (DAG) is a second messenger that acts as a protein kinase C activator. This	GO:0016049|cell growth;GO:0046339|diacylglycerol metabolic process;GO:0006897|endocytosis;GO:0007173|epidermal growth factor receptor signaling pathway;GO:0046834|lipid phosphorylation;GO:0007275|multicellular organismal development;GO:0030168|platelet activation;GO:0051260|protein homooligomerization;GO:0007205|protein kinase C-activating G-protein coupled receptor signaling pathway;GO:0015031|protein transport;GO:0010033|response to organic substance;GO:0019932|second-messenger-mediated	GO:0005737|cytoplasm;GO:0016023|cytoplasmic membrane-bounded vesicle;GO:0005634|nucleus;GO:0005886|plasma membrane;	GO:0005524|ATP binding;GO:0019992|diacylglycerol binding;GO:0004143	K00901	dgkA, DGK; diacylglycerol kinase (ATP) [EC:2.7.1.107]
RUNX1	1138.536	-0.3526	0.113421651	-3.10875	0.0018788	0.017681	Not_Sig	no	gi|375151592|ref|NP_001243507.1|	2275	0	runt-related transcription factor 1 [Bos taurus]	gi|982912457|ref|XM_005202079.3|	6063	0	PREDICTED: Bos taurus runt-related transcription factor 1 (RUNX1), transcript variant X1, mRNA	Q01196	800	0	Runt-related transcription factor 1	.	.	.	.	pfam00853	Runt	Runt domain.	GO:0002062|chondrocyte differentiation;GO:0071425|hematopoietic stem cell proliferation;GO:0030097|hemopoiesis;GO:0030099|myeloid cell differentiation;GO:0030853|negative regulation of granulocyte differentiation;GO:0001503|ossification;GO:0048935|peripheral nervous system neuron development;GO:0045766|positive regulation of angiogenesis;GO:0030854|positive regulation of granulocyte differentiation;GO:0032743|positive regulation of interleukin-2 production;GO:0045944|positive regulation of transcription from RNA	GO:0005737|cytoplasm;GO:0043231|intracellular membrane-bounded organelle;GO:0005654|nucleoplasm;GO:0005634|nucleus;	GO:0005524|ATP binding;GO:0005509|calcium ion binding;GO:0001047	K08367	RUNX1, AML1; runt-related transcription factor 1
LOC102280541	657.1873	0.399072	0.128370833	3.10874	0.0018789	0.017681	Not_Sig	no	gi|744538506|ref|XP_010973953.1|	1410	0	PREDICTED: 60S ribosomal protein L5 [Camelus dromedarius]	gi|402691847|ref|NM_001035306.2|	1012	0	Bos taurus ribosomal protein L5 (RPL5), mRNA	Q58DW5	522	0	60S ribosomal protein L5	SPAC3H5.12c	283	5.81e-94	COG0256	pfam14204	Ribosomal_L18_c	Ribosomal L18 C-terminal region. This domain is the C-terminal end of ribosomal L18/L5 proteins.	GO:0000027|ribosomal large subunit assembly;GO:0006412|translation;	GO:0005737|cytoplasm;GO:0022625|cytosolic large ribosomal subunit;GO:0005730|nucleolus;	GO:0008097|5S rRNA binding;GO:0003735|structural	K02932	RP-L5e, RPL5; large subunit ribosomal protein L5e
SMG5	1237.288	0.330737	0.106413227	3.108044	0.0018833	0.017708	Not_Sig	no	gi|139948860|ref|NP_001077148.1|	4662	0	protein SMG5 [Bos taurus]	gi|139948859|ref|NM_001083679.1|	4479	0	Bos taurus SMG5 nonsense mediated mRNA decay factor (SMG5), mRNA	Q9UPR3	1736	0	Protein SMG5	.	.	.	.	pfam10373	EST1_DNA_bind	Est1 DNA/RNA binding domain. Est1 is a protein which recruits or activates telomerase at the site of polymerization. This is the	GO:0006406|mRNA export from nucleus;GO:0000184|nuclear-transcribed mRNA catabolic process, nonsense-mediated decay;GO:0035303|regulation of dephosphorylation;GO:0043487|regulation of RNA stability;GO:0032204|regulation of telomere maintenance;GO:0032210|regulation of telomere maintenance via telomerase;	GO:0005737|cytoplasm;GO:0005829|cytosol;GO:0005634|nucleus;GO:0005697|telomerase holoenzyme complex;	GO:0070182|DNA polymerase binding;GO:0042826|histone	K11125	SMG5, EST1B; protein SMG5
KMT2B	456.8828	0.517052	0.166382245	3.107613	0.001886	0.017712	Not_Sig	no	gi|359075420|ref|XP_003587289.1|	8701	0	PREDICTED: histone-lysine N-methyltransferase 2B isoform X2 [Bos taurus]	gi|982999584|ref|XM_015467426.1|	8385	0	PREDICTED: Bos taurus lysine (K)-specific methyltransferase 2B (KMT2B), transcript variant X2, mRNA	Q9UMN6	3159	0	Histone-lysine N-methyltransferase 2B	SPCC306.04c	164	1.09e-39	COG2940	pfam00856	SET	SET domain. SET domains are protein lysine methyltransferase enzymes. SET domains appear to be protein-protein interaction	GO:0048096|chromatin-mediated maintenance of transcription;GO:0016458|gene silencing;GO:0051568|histone H3-K4 methylation;GO:0080182|histone H3-K4 trimethylation;GO:0007613|memory;GO:0009994|oocyte differentiation;GO:0001541|ovarian follicle development;GO:0030728|ovulation;GO:0051569|regulation of histone H3-K4 methylation;GO:0006351|transcription, DNA-templated;	GO:0035097|histone methyltransferase complex;GO:0005654|nucleoplasm;GO:0005634|nucleus;	GO:0003677|DNA binding;GO:0042800|histone methyltransferase	K14959	MLL4; histone-lysine N-methyltransferase MLL4 [EC:2.1.1.43]
NFIX	794.1458	0.467566	0.150471701	3.107336	0.0018878	0.017712	Not_Sig	no	gi|56549649|ref|NP_002492.2|	2179	0	nuclear factor 1 X-type isoform 2 [Homo sapiens]	gi|821404323|ref|XM_004277449.2|	4646	0	PREDICTED: Orcinus orca nuclear factor I/X (CCAAT-binding transcription factor) (NFIX), transcript variant X2, mRNA	P13623	842	0	Nuclear factor 1 X-type	.	.	.	.	pfam00859	CTF_NFI	CTF/NF-I family transcription modulation region.	GO:0006260|DNA replication;GO:0006351|transcription, DNA-templated;	GO:0005634|nucleus;	GO:0003677|DNA binding;GO:0003700|transcription factor activity, sequence-	.	.
BAK1	744.3359	0.348951	0.112312678	3.10696	0.0018902	0.017712	Not_Sig	no	gi|118151394|ref|NP_001071386.1|	944	8e-120	bcl-2 homologous antagonist/killer [Bos taurus]	gi|982963001|ref|XM_005223241.3|	2099	0	PREDICTED: Bos taurus BCL2-antagonist/killer 1 (BAK1), transcript variant X1, mRNA	Q16611	308	3e-98	Bcl-2 homologous antagonist/killer	.	.	.	.	pfam00452	Bcl-2	Apoptosis regulator proteins, Bcl-2 family.	GO:0097202|activation of cysteine-type endopeptidase activity;GO:0008635|activation of cysteine-type endopeptidase activity involved in apoptotic process by cytochrome c;GO:0006987|activation of signaling protein activity involved in unfolded protein response;GO:0007568|aging;GO:0031100|organ regeneration;GO:1902262|apoptotic process involved in patterning of blood vessels;GO:0097190|apoptotic signaling pathway;GO:0001783|B cell apoptotic process;GO:0001782|B cell homeostasis;GO:0002352|B cell	GO:0005829|cytosol;GO:0005783|endoplasmic reticulum;GO:0031307|integral component of mitochondrial outer membrane;GO:0005741|mitochondrial	GO:0042802|identical protein binding;GO:0044325|ion channel	K14021	BAK, BAK1; Bcl-2 homologous antagonist/killer
EHD1	5377.996	0.342944	0.110383754	3.106831	0.001891	0.017712	Not_Sig	no	gi|803224495|ref|XP_011972549.1|	2702	0	PREDICTED: EH domain-containing protein 1 isoform X2 [Ovis aries musimon]	gi|742110351|ref|XM_010838014.1|	3089	0	PREDICTED: Bison bison bison EH-domain containing 1 (EHD1), mRNA	Q5E9R3	1043	0	EH domain-containing protein 1	.	.	.	.	pfam00350	Dynamin_N	Dynamin family.	GO:1990090|cellular response to nerve growth factor stimulus;GO:0042384|cilium assembly;GO:0032456|endocytic recycling;GO:0006897|endocytosis;GO:0006886|intracellular protein transport;GO:0031175|neuron projection development;GO:2001137|positive regulation of endocytic recycling;GO:1901741|positive regulation of myoblast fusion;GO:0061512|protein localization to cilium;	GO:0020018|ciliary pocket membrane;GO:0031901|early endosome membrane;GO:0010008|endosome membrane;GO:0005886|plasma	GO:0005524|ATP binding;GO:0005509|calcium ion binding;GO:0005525	K12483	EHD1; EH domain-containing protein 1
NEDD4L	564.5034	0.381789	0.122889568	3.106764	0.0018915	0.017712	Not_Sig	no	gi|803232115|ref|XP_011976234.1|	5700	0	PREDICTED: E3 ubiquitin-protein ligase NEDD4-like isoform X12 [Ovis aries musimon]	gi|803232114|ref|XM_012120844.1|	5755	0	PREDICTED: Ovis aries musimon neural precursor cell expressed, developmentally down-regulated 4-like, E3 ubiquitin protein ligase (NEDD4L), transcript variant X1, mRNA	Q96PU5	1593	0	E3 ubiquitin-protein ligase NEDD4-like	SPAC11G7.02	539	2.06e-167	COG5021	pfam00632	HECT	HECT-domain (ubiquitin-transferase). The name HECT comes from Homologous to the E6-AP Carboxyl Terminus.	GO:0006883|cellular sodium ion homeostasis;GO:0007588|excretion;GO:0034220|ion transmembrane transport;GO:1901380|negative regulation of potassium ion transmembrane transport;GO:1901017|negative regulation of potassium ion transmembrane transporter activity;GO:2000009|negative regulation of protein localization to cell surface;GO:1902306|negative regulation of sodium ion transmembrane transport;GO:2000650|negative regulation of sodium ion transmembrane transporter activity;GO:0000122|negative regulation of transcription	GO:0005737|cytoplasm;GO:0005829|cytosol;GO:0070062|extracellular exosome;GO:0005622|intracellular;GO:0005654|nucleoplasm;GO:0005634|nucleus;	GO:0044325|ion channel binding;GO:0019870|potassium channel	K13305	NEDD4L; E3 ubiquitin-protein ligase NEDD4-like [EC:2.3.2.26]
FZR1	664.3408	0.387454	0.124723021	3.106517	0.0018931	0.017713	Not_Sig	no	gi|528955643|ref|XP_005209006.1|	2653	0	PREDICTED: fizzy-related protein homolog isoform X1 [Bos taurus]	gi|982930879|ref|XM_005208949.3|	3076	0	PREDICTED: Bos taurus fizzy/cell division cycle 20 related 1 (FZR1), transcript variant X1, mRNA	Q9UM11	1015	0	Fizzy-related protein homolog	.	.	.	.	pfam00400	WD40	WD domain, G-beta repeat.	GO:0031145|anaphase-promoting complex-dependent proteasomal ubiquitin-dependent protein catabolic process;GO:0051301|cell division;GO:0006281|DNA repair;GO:0031572|G2 DNA damage checkpoint;GO:0070306|lens fiber cell differentiation;GO:0007067|mitotic nuclear division;GO:0090344|negative regulation of cell aging;GO:0051436|negative regulation of ubiquitin-protein ligase activity involved in mitotic cell cycle;GO:0008284|positive regulation of cell proliferation;GO:0045732|positive regulation of protein catabolic	GO:0005829|cytosol;GO:0031965|nuclear membrane;GO:0005654|nucleoplasm;	GO:0097027|ubiquitin-protein transferase activator activity;	K03364	CDH1; cell division cycle 20-like protein 1, cofactor of APC complex
CACNB3	764.8137	0.4427	0.142579288	3.104936	0.0019032	0.017793	Not_Sig	no	gi|528948753|ref|XP_005206314.1|	2424	0	PREDICTED: voltage-dependent L-type calcium channel subunit beta-3 isoform X1 [Bos taurus]	gi|982924908|ref|XM_005206257.3|	3067	0	PREDICTED: Bos taurus calcium channel, voltage-dependent, beta 3 subunit (CACNB3), transcript variant X1, mRNA	Q9MZL3	906	0	Voltage-dependent L-type calcium channel subunit beta-3	.	.	.	.	pfam00625	Guanylate_kin	Guanylate kinase.	GO:0007268|synaptic transmission;GO:0007528|neuromuscular junction development;GO:1901385|regulation of voltage-gated calcium channel activity;GO:0050852|T cell receptor signaling pathway;	GO:0005891|voltage-gated calcium channel complex;	GO:0008331|high voltage-gated calcium channel activity;	K04864	CACNB3; voltage-dependent calcium channel beta-3
ARHGAP35	4620.816	0.315576	0.101657518	3.104306	0.0019073	0.017804	Not_Sig	no	gi|300798482|ref|NP_001179327.1|	7458	0	rho GTPase-activating protein 35 [Bos taurus]	gi|594088128|ref|XM_006067460.1|	8016	0	PREDICTED: Bubalus bubalis Rho GTPase activating protein 35 (ARHGAP35), mRNA	P83509	2852	0	Rho GTPase-activating protein 35	.	.	.	.	pfam00620	RhoGAP	RhoGAP domain. GTPase activator proteins towards Rho/Rac/Cdc42-like small GTPases.	GO:0007411|axon guidance;GO:0007413|axonal fasciculation;GO:0016477|cell migration;GO:0031668|cellular response to extracellular stimulus;GO:0021955|central nervous system neuron axonogenesis;GO:0030950|establishment or maintenance of actin cytoskeleton polarity;GO:0030879|mammary gland development;GO:0045892|negative regulation of transcription, DNA-templated;GO:0097485|neuron projection guidance;GO:0045724|positive regulation of cilium assembly;GO:0043547|positive regulation of GTPase	GO:0036064|ciliary basal body;GO:0005737|cytoplasm;GO:0005634|nucleus;	GO:0003677|DNA binding;GO:0005525|GTP binding;GO:0005096	K05732	GRLF1; glucocorticoid receptor DNA-binding factor 1
DDOST	3094.453	0.335338	0.108029379	3.104135	0.0019084	0.017804	Not_Sig	no	gi|440897815|gb|ELR49427.1|	2272	0	Dolichyl-diphosphooligosaccharide--protein glycosyltransferase 48 kDa subunit, partial [Bos mutus]	gi|555986832|ref|XM_005905210.1|	1607	0	PREDICTED: Bos mutus dolichyl-diphosphooligosaccharide--protein glycosyltransferase subunit (non-catalytic) (DDOST), mRNA	A6QPY0	843	0	Dolichyl-diphosphooligosaccharide--protein glycosyltransferase 48 kDa	.	.	.	.	pfam03345	DDOST_48kD	Oligosaccharyltransferase 48 kDa subunit beta. Members of this family are involved in asparagine-linked protein glycosylation. In	GO:0006486|protein glycosylation;GO:0018279|protein N-linked glycosylation via asparagine;	GO:0016021|integral component of membrane;GO:0008250|oligosaccharyltransferase complex;	GO:0016757|transferase activity, transferring glycosyl groups;	K12670	WBP1; oligosaccharyltransferase complex subunit beta
COQ10B	602.1919	-0.37214	0.119889983	-3.10403	0.001909	0.017804	Not_Sig	no	gi|555965181|ref|XP_005894649.1|	1223	1e-162	PREDICTED: coenzyme Q-binding protein COQ10 homolog B, mitochondrial [Bos mutus]	gi|942051419|ref|XM_005894587.2|	1810	0	PREDICTED: Bos mutus coenzyme Q10B (COQ10B), mRNA	Q9H8M1	430	2e-146	Coenzyme Q-binding protein COQ10 homolog B, mitochondrial	RSc1425	75.9	1.41e-15	COG2867	pfam03364	Polyketide_cyc	Polyketide cyclase / dehydrase and lipid transport. This family contains polyketide cylcases/dehydrases which are enzymes involved in	.	GO:0005743|mitochondrial inner membrane;	.	K18588	COQ10; coenzyme Q-binding protein COQ10
FAM162A	311.6646	-0.43374	0.139756053	-3.10354	0.0019122	0.017819	Not_Sig	no	gi|114051522|ref|NP_001040057.1|	827	1e-109	protein FAM162A [Bos taurus]	gi|555962790|ref|XM_005893421.1|	667	0	PREDICTED: Bos mutus family with sequence similarity 162, member A (FAM162A), mRNA	Q2NKR7	323	4e-111	Protein FAM162A	.	.	.	.	pfam06388	DUF1075	Protein of unknown function (DUF1075). This family consists of several eukaryotic proteins of unknown function.	GO:0006919|activation of cysteine-type endopeptidase activity involved in apoptotic process;GO:0006915|apoptotic process;GO:0071456|cellular response to hypoxia;GO:0043065|positive regulation of apoptotic process;GO:0090200|positive regulation of release of cytochrome c from mitochondria;	GO:0016021|integral component of membrane;GO:0005739|mitochondrion;	.	.	.
LOC102267212	1627.619	0.344953	0.111169703	3.102944	0.0019161	0.01784	Not_Sig	no	gi|296479202|tpg|DAA21317.1|	4186	0	TPA: hexose-6-phosphate dehydrogenase (glucose 1-dehydrogenase) [Bos taurus]	gi|594107716|ref|XM_006076689.1|	4607	0	PREDICTED: Bubalus bubalis hexose-6-phosphate dehydrogenase (glucose 1-dehydrogenase) (H6PD), mRNA	O95479	1373	0	GDH/6PGL endoplasmic bifunctional protein	YNL241c	219	1.89e-60	COG0364	pfam01182	Glucosamine_iso	Glucosamine-6-phosphate isomerases/6-phosphogluconolactonase.	GO:0006006|glucose metabolic process;GO:0006098|pentose-phosphate shunt;	GO:0005788|endoplasmic reticulum lumen;	GO:0017057|6-phosphogluconolactonase activity;GO:0047936	K13937	H6PD; hexose-6-phosphate dehydrogenase [EC:1.1.1.47
MYL6	13625.64	-0.32382	0.104367366	-3.10272	0.0019175	0.01784	Not_Sig	no	gi|731261225|ref|XP_010643011.1|	821	6e-108	PREDICTED: myosin light polypeptide 6 isoform X4 [Fukomys damarensis]	gi|555983170|ref|XM_005903426.1|	768	0	PREDICTED: Bos mutus myosin, light chain 6, alkali, smooth muscle and non-muscle (MYL6), transcript variant X6, mRNA	Q5R844	315	1e-107	Myosin light polypeptide 6	SPAC3A12.14	117	3.03e-32	COG5126	pfam13499	EF-hand_7	EF-hand domain pair.	.	GO:0016459|myosin complex;	GO:0005509|calcium ion binding;	K12751	MYL6; myosin light chain 6
DCUN1D1	614.0097	-0.47384	0.152782239	-3.10141	0.001926	0.017899	Not_Sig	no	gi|594104805|ref|XP_006075374.1|	1379	7e-170	PREDICTED: DCN1-like protein 1 isoform X1 [Bubalus bubalis]	gi|742185504|ref|XM_010855641.1|	6444	0	PREDICTED: Bison bison bison DCN1, defective in cullin neddylation 1, domain containing 1 (DCUN1D1), transcript variant X3, mRNA	Q96GG9	531	4e-170	DCN1-like protein 1	.	.	.	.	pfam03556	Cullin_binding	Cullin binding. This domain binds to cullins and to Rbx-1, components of an E3 ubiquitin ligase complex for neddylation.	GO:0051443|positive regulation of ubiquitin-protein transferase activity;GO:0045116|protein neddylation;	GO:0005634|nucleus;GO:0000151|ubiquitin ligase complex;	GO:0097602|cullin family protein binding;GO:0031624|ubiquitin	K17822	DCUN1D1_2; DCN1-like protein 1/2
CDK2AP2	236.1666	0.472971	0.152514447	3.101158	0.0019277	0.017899	Not_Sig	no	gi|686725716|ref|XP_009241257.1|	575	6e-68	PREDICTED: LOW QUALITY PROTEIN: cyclin-dependent kinase 2-associated protein 2-like, partial [Pongo abelii]	gi|942077984|ref|XM_005903566.2|	1435	0	PREDICTED: Bos mutus cyclin-dependent kinase 2 associated protein 2 (CDK2AP2), transcript variant X2, mRNA	O75956	174	1e-50	Cyclin-dependent kinase 2-associated protein 2	.	.	.	.	pfam09806	CDK2AP	Cyclin-dependent kinase 2-associated protein. Members of this family of proteins are cell-growth suppressors, associating	.	.	.	.	.
BPGM	601.5778	-0.35704	0.115137591	-3.10102	0.0019286	0.017899	Not_Sig	no	gi|440898660|gb|ELR50106.1|	1405	0	hypothetical protein M91_16189 [Bos mutus]	gi|742236020|ref|XM_010831643.1|	1657	0	PREDICTED: Bison bison bison 2,3-bisphosphoglycerate mutase (BPGM), mRNA	Q3T014	531	2e-179	Bisphosphoglycerate mutase	XF1893	256	1.72e-77	COG0588	pfam00300	His_Phos_1	Histidine phosphatase superfamily (branch 1). The histidine phosphatase superfamily is so named because catalysis centres	GO:0006094|gluconeogenesis;GO:0006096|glycolytic process;GO:0043456|regulation of pentose-phosphate shunt;	GO:0005829|cytosol;	GO:0046538|2,3-bisphosphoglycerate-dependent phosphoglycerate	K01837	BPGM; bisphosphoglycerate mutase [EC:5.4.2.4 5.4.2.11 3.1.3.13]
EMC4	811.5441	-0.40368	0.130207684	-3.10026	0.0019335	0.017924	Not_Sig	no	gi|395837619|ref|XP_003791728.1|	843	5e-111	PREDICTED: ER membrane protein complex subunit 4 isoform X1 [Otolemur garnettii]	gi|742140761|ref|XM_010844165.1|	814	0	PREDICTED: Bison bison bison ER membrane protein complex subunit 4 (EMC4), transcript variant X1, mRNA	Q3T0K8	327	8e-112	ER membrane protein complex subunit 4	.	.	.	.	pfam06417	DUF1077	Protein of unknown function (DUF1077). This family consists of several hypothetical eukaryotic proteins of unknown function.	GO:0006915|apoptotic process;	GO:0072546|ER membrane protein complex;GO:0016021|integral component of membrane;	.	.	.
RPAP1	347.8666	0.470623	0.151807238	3.100133	0.0019343	0.017924	Not_Sig	no	gi|555984275|ref|XP_005904028.1|	5853	0	PREDICTED: RNA polymerase II-associated protein 1 [Bos mutus]	gi|942079231|ref|XM_005903966.2|	4621	0	PREDICTED: Bos mutus RNA polymerase II associated protein 1 (RPAP1), mRNA	A0JN53	2228	0	RNA polymerase II-associated protein 1	.	.	.	.	pfam08620	RPAP1_C	RPAP1-like, C-terminal. Inhibition of RPAP1 synthesis in Saccharomyces cerevisiae results in changes in global gene	GO:0006366|transcription from RNA polymerase II promoter;	GO:0005634|nucleus;	GO:0003677|DNA binding;GO:0003899|DNA-directed RNA polymerase activity;	.	.
DLGAP3	40.39902	1.079228	0.348230543	3.099177	0.0019406	0.017967	UP	yes	gi|300794080|ref|NP_001179367.1|	3925	0	disks large-associated protein 3 [Bos taurus]	gi|982920056|ref|XM_005204733.3|	3791	0	PREDICTED: Bos taurus discs large homolog associated protein 3 (DLGAP3), transcript variant X1, mRNA	O95886	1490	0	Disks large-associated protein 3	.	.	.	.	pfam03359	GKAP	Guanylate-kinase-associated protein (GKAP) protein.	GO:0023052|signaling;	GO:0030054|cell junction;GO:0014069|postsynaptic density;GO:0045211|postsynaptic membrane;	GO:0001540|beta-amyloid binding;	.	.
STK25	717.4322	0.394633	0.127347437	3.09887	0.0019426	0.017971	Not_Sig	no	gi|311273415|ref|XP_003133853.1|	1999	0	PREDICTED: serine/threonine-protein kinase 25 [Sus scrofa]	gi|74356312|gb|BC104597.1|	1898	0	Bos taurus serine/threonine kinase 25 (STE20 homolog, yeast), mRNA (cDNA clone MGC:128993 IMAGE:8120917), complete cds	Q3SWY6	773	0	Serine/threonine-protein kinase 25	.	.	.	.	pfam00069	Pkinase	Protein kinase domain.	.	GO:0005737|cytoplasm;GO:0005794|Golgi apparatus;	GO:0005524|ATP binding;GO:0046872|metal ion binding;GO:0004702	K08838	STK24_25_MST4; serine/threonine-protein kinase 24/25/MST4
CUL9	491.1613	0.436411	0.14085145	3.098376	0.0019458	0.017987	Not_Sig	no	gi|741970729|ref|XP_010816562.1|	11765	0	PREDICTED: cullin-9 isoform X1 [Bos taurus]	gi|983005176|ref|XM_010799061.2|	7608	0	PREDICTED: Bos taurus cullin 9 (CUL9), transcript variant X1, mRNA	Q8IWT3	4054	0	Cullin-9	.	.	.	.	pfam00888	Cullin	Cullin family.	GO:0000226|microtubule cytoskeleton organization;GO:0016567|protein ubiquitination;GO:0007088|regulation of mitotic nuclear division;GO:0006511|ubiquitin-dependent protein catabolic process;	GO:0031461|cullin-RING ubiquitin ligase complex;GO:0005737|cytoplasm;	GO:0005524|ATP binding;GO:0008270|zinc ion binding;	K11970	CUL9, PARC; p53-associated parkin-like cytoplasmic protein
CD2AP	928.4907	-0.39464	0.127387637	-3.09792	0.0019489	0.018	Not_Sig	no	gi|296474362|tpg|DAA16477.1|	2629	0	TPA: CD2-associated protein [Bos taurus]	gi|594106510|ref|XM_006076117.1|	4716	0	PREDICTED: Bubalus bubalis CD2-associated protein (CD2AP), transcript variant X1, mRNA	Q9Y5K6	868	0	CD2-associated protein	.	.	.	.	pfam14604	SH3_9	Variant SH3 domain.	GO:0051301|cell division;GO:0007067|mitotic nuclear division;GO:0032911|negative regulation of transforming growth factor beta1 production;GO:1900182|positive regulation of protein localization to nucleus;GO:0043161|proteasome-mediated ubiquitin-dependent protein catabolic process;GO:0006461|protein complex assembly;GO:2000249|regulation of actin cytoskeleton reorganization;GO:0048259|regulation of receptor-mediated endocytosis;GO:0007165|signal transduction;GO:0016337|single organismal cell-cell adhesion;GO:0006930|substrate-	GO:0015629|actin cytoskeleton;GO:0005913|cell-cell adherens junction;GO:0005737|cytoplasm;GO:0030139|endocytic	GO:0098641|cadherin binding involved in cell-cell adhesion;GO:00171	K13738	CD2AP; CD2-associated protein
TBC1D12	250.8096	-0.46659	0.150626633	-3.09765	0.0019506	0.018002	Not_Sig	no	gi|529008420|ref|XP_005225549.1|	3529	0	PREDICTED: TBC1 domain family member 12 isoform X1 [Bos taurus]	gi|982968046|ref|XM_005225492.3|	4174	0	PREDICTED: Bos taurus TBC1 domain family member 12 (TBC1D12), transcript variant X1, mRNA	O60347	1145	0	TBC1 domain family member 12	SPAC23D3.03c	140	4.02e-34	COG5210	pfam00566	RabGAP-TBC	Rab-GTPase-TBC domain. Identification of a TBC domain in GYP6_YEAST and GYP7_YEAST, which are GTPase activator	GO:0090630|activation of GTPase activity;GO:0006886|intracellular protein transport;GO:2000785|regulation of autophagosome assembly;GO:0031338|regulation of vesicle fusion;	GO:0005776|autophagosome;GO:0055037|recycling endosome;	GO:0005096|GTPase activator activity;GO:0017137|Rab GTPase	.	.
RPLP1	7158.126	-0.40205	0.129819083	-3.09703	0.0019547	0.018024	Not_Sig	no	gi|641694801|ref|XP_008158123.1|	361	9e-41	PREDICTED: 60S acidic ribosomal protein P1 isoform X1 [Eptesicus fuscus]	gi|942097290|ref|XM_005909682.2|	528	0	PREDICTED: Bos mutus 60S acidic ribosomal protein P1 (LOC102287882), transcript variant X1, mRNA	P05386	142	6e-42	60S acidic ribosomal protein P1	SPAC644.15	65.9	9.85e-14	COG2058	pfam00428	Ribosomal_60s	60s Acidic ribosomal protein. This family includes archaebacterial L12, eukaryotic P0, P1 and P2.	GO:0002181|cytoplasmic translation;GO:0000184|nuclear-transcribed mRNA catabolic process, nonsense-mediated decay;GO:0045860|positive regulation of protein kinase activity;GO:0006364|rRNA processing;GO:0006614|SRP-dependent cotranslational protein targeting to membrane;GO:0006412|translation;GO:0006414|translational elongation;GO:0006413|translational initiation;GO:0019083|viral transcription;	GO:0005737|cytoplasm;GO:0005829|cytosol;GO:0022625|cytosolic large ribosomal subunit;GO:0070062|extracellular exosome;GO:0005925|focal	GO:0070180|large ribosomal subunit rRNA binding;GO:0030295	K02942	RP-LP1, RPLP1; large subunit ribosomal protein LP1
CC2D1A	476.508	0.4846	0.156483431	3.096816	0.0019561	0.018024	Not_Sig	no	gi|555951756|ref|XP_005888073.1|	3843	0	PREDICTED: coiled-coil and C2 domain-containing protein 1A [Bos mutus]	gi|942033534|ref|XM_005888011.2|	3549	0	PREDICTED: Bos mutus coiled-coil and C2 domain containing 1A (CC2D1A), mRNA	Q6P1N0	1382	0	Coiled-coil and C2 domain-containing protein 1A	.	.	.	.	pfam00168	C2	C2 domain.	GO:0000122|negative regulation of transcription from RNA polymerase II promoter;GO:0043123|positive regulation of I-kappaB kinase/NF-kappaB signaling;GO:0006351|transcription, DNA-templated;	GO:0005913|cell-cell adherens junction;GO:0005737|cytoplasm;GO:0070062|extracellular exosome;GO:0016020|membrane;GO:0005	GO:0098641|cadherin binding involved in cell-cell adhesion;GO:00009	K18260	CC2D1; coiled-coil and C2 domain-containing protein 1

SLC66A1	489.02	0.515635	0.166521887	3.096499	0.0019582	0.018029	Not_Sig	no	gi|555992775|ref|XP_005908158.1|	1227	1e-161	PREDICTED: lysosomal amino acid transporter 1 homolog [Bos mutus]	gi|942092475|ref|XM_005908096.2|	1562	0	PREDICTED: Bos mutus PQ loop repeat containing 2 (PQLC2), mRNA	Q6ZP29	385	2e-127	Lysosomal amino acid transporter 1 homolog	.	.	.	.	pfam04193	PQ-loop	PQ loop repeat. Members of this family are all membrane bound proteins possessing a pair of repeats each spanning two	GO:0080144|amino acid homeostasis;GO:0015809|arginine transport;GO:0015819|lysine transport;	GO:0031301|integral component of organelle membrane;GO:0043231|intracellular membrane-bounded	GO:0015181|arginine transmembrane transporter activity;GO:0015174	.	.
RPS3	4840.402	-0.43173	0.139445053	-3.09607	0.0019611	0.018035	Not_Sig	no	gi|15718687|ref|NP_000996.2|	1264	1e-173	40S ribosomal protein S3 isoform 1 [Homo sapiens]	gi|942076978|ref|XM_005903228.2|	846	0	PREDICTED: Bos mutus ribosomal protein S3 (RPS3), mRNA	Q0Z8U2	491	4e-175	40S ribosomal protein S3	SPBC16G5.14c	324	2.35e-111	COG0092	pfam00189	Ribosomal_S3_C	Ribosomal protein S3, C-terminal domain. This family contains a central domain pfam00013, hence the amino and carboxyl terminal	GO:0006915|apoptotic process;GO:0051301|cell division;GO:0006281|DNA repair;GO:0007067|mitotic nuclear division;GO:0006355|regulation of transcription, DNA-templated;GO:0006417|regulation of translation;GO:0006351|transcription, DNA-templated;GO:0006412|translation;	GO:0022627|cytosolic small ribosomal subunit;GO:0030529|ribonucleoprotein complex;GO:0005743|mitochondrial inner membrane;GO:0005730|nucleolus;GO:0005	GO:0003684|damaged DNA binding;GO:0008534|oxidized purine	K02985	RP-S3e, RPS3; small subunit ribosomal protein S3e
WASHC4	1248.202	-0.39469	0.12748877	-3.09592	0.001962	0.018035	Not_Sig	no	gi|297474980|ref|XP_002687692.1|	5941	0	PREDICTED: WASH complex subunit 7 [Bos taurus]	gi|742176791|ref|XM_010853391.1|	5760	0	PREDICTED: Bison bison bison KIAA1033 ortholog (KIAA1033), mRNA	Q2M389	2272	0	WASH complex subunit SWIP	.	.	.	.	pfam14745	WASH-7_N	WASH complex subunit 7, N-terminal. This family is the conserved N-terminal region of proteins that form subunit 7 of the	GO:0016197|endosomal transport;GO:0007032|endosome organization;GO:0015031|protein transport;	GO:0005769|early endosome;GO:0005768|endosome;GO:0005654|nucleoplasm;GO:0071203|WASH complex;	.	K18465	MRT43, SWIP; WASH complex subunit 7
CHML	328.9734	-0.48647	0.157302034	-3.09257	0.0019843	0.018213	Not_Sig	no	gi|555970484|ref|XP_005897263.1|	3368	0	PREDICTED: rab proteins geranylgeranyltransferase component A 2 [Bos mutus]	gi|742104305|ref|XM_010836685.1|	5204	0	PREDICTED: Bison bison bison choroideremia-like (Rab escort protein 2) (CHML), mRNA	P26374	1050	0	Rab proteins geranylgeranyltransferase component A 2	YER136w	130	1.34e-30	COG5044	pfam00996	GDI	GDP dissociation inhibitor.	GO:0006886|intracellular protein transport;GO:0018344|protein geranylgeranylation;GO:0007264|small GTPase mediated signal transduction;	GO:0005737|cytoplasm;GO:0005829|cytosol;GO:0005654|nucleoplasm;GO:0005968|Rab-protein geranylgeranyltransferase complex;	GO:0005092|GDP-dissociation inhibitor activity;GO:0005096	.	.
ADD1	1972.884	0.359955	0.116394689	3.092535	0.0019845	0.018213	Not_Sig	no	gi|528953792|ref|XP_005208285.1|	3549	0	PREDICTED: alpha-adducin isoform X1 [Bos taurus]	gi|982928858|ref|XM_005208229.3|	3884	0	PREDICTED: Bos taurus adducin 1 (ADD1), transcript variant X2, mRNA	P35611	1162	0	Alpha-adducin	CC1201	176	2.16e-48	COG0235	pfam00596	Aldolase_II	Class II Aldolase and Adducin N-terminal domain. This family includes class II aldolases and adducins which have not been	GO:0030036|actin cytoskeleton organization;GO:0051017|actin filament bundle assembly;GO:0051016|barbed-end actin filament capping;GO:0000902|cell morphogenesis;GO:0006884|cell volume homeostasis;GO:0030218|erythrocyte differentiation;GO:0020027|hemoglobin metabolic process;GO:0048873|homeostasis of number of cells within a tissue;GO:0001701|in utero embryonic development;GO:0036498|IRE1-mediated unfolded protein response;GO:0035264|multicellular organism	GO:0005913|cell-cell adherens junction;GO:0005829|cytosol;GO:0008290|F-actin capping protein complex;GO:0005925|focal	GO:0003779|actin binding;GO:0051015|actin filament binding;GO:0098641	K18622	ADD; adducin
COG7	504.587	0.42745	0.138247872	3.091908	0.0019887	0.018237	Not_Sig	no	gi|555987057|ref|XP_005905382.1|	4005	0	PREDICTED: conserved oligomeric Golgi complex subunit 7 [Bos mutus]	gi|555987056|ref|XM_005905320.1|	2695	0	PREDICTED: Bos mutus component of oligomeric golgi complex 7 (COG7), mRNA	A2VDR8	1546	0	Conserved oligomeric Golgi complex subunit 7	.	.	.	.	pfam10191	COG7	Golgi complex component 7 (COG7). COG7 is a component of the conserved oligomeric Golgi complex which is required for	GO:0007030|Golgi organization;GO:0006886|intracellular protein transport;GO:0006486|protein glycosylation;GO:0034067|protein localization to Golgi apparatus;GO:0033365|protein localization to organelle;GO:0050821|protein stabilization;GO:0006890|retrograde vesicle-mediated transport, Golgi to ER;	GO:0005794|Golgi apparatus;GO:0000139|Golgi membrane;GO:0017119|Golgi transport complex;GO:0005730|nucleolus;	.	.	.
AGFG2	294.4722	0.525858	0.170128774	3.090943	0.0019952	0.018282	Not_Sig	no	gi|742109869|ref|XP_010836197.1|	1509	0	PREDICTED: arf-GAP domain and FG repeat-containing protein 2 isoform X1 [Bison bison bison]	gi|742109868|ref|XM_010837895.1|	2672	0	PREDICTED: Bison bison bison ArfGAP with FG repeats 2 (AGFG2), transcript variant X1, mRNA	O95081	530	2e-177	Arf-GAP domain and FG repeat-containing protein 2	YNL204c	54.7	4.35e-07	COG5347	pfam01412	ArfGap	Putative GTPase activating protein for Arf. Putative zinc fingers with GTPase activating proteins (GAPs) towards the small GTPase, Arf. The	.	GO:0016020|membrane;	GO:0005096|GTPase activator activity;GO:0046872|metal ion binding;	.	.
CDKN1B	456.2863	-0.37766	0.122199803	-3.09048	0.0019983	0.018293	Not_Sig	no	gi|154152099|ref|NP_001093816.1|	1025	7e-133	cyclin-dependent kinase inhibitor 1B [Bos taurus]	gi|942039229|ref|XM_005890030.2|	1956	0	PREDICTED: Bos mutus cyclin-dependent kinase inhibitor 1B (p27, Kip1) (CDKN1B), mRNA	O19001	379	2e-126	Cyclin-dependent kinase inhibitor 1B	.	.	.	.	pfam02234	CDI	Cyclin-dependent kinase inhibitor. Cell cycle progression is negatively controlled by cyclin-dependent kinases inhibitors (CDIs). CDIs are	GO:0048102|autophagic cell death;GO:0071236|cellular response to antibiotic;GO:0071285|cellular response to lithium ion;GO:0071407|cellular response to organic cyclic compound;GO:0000082|G1/S transition of mitotic cell cycle;GO:0048839|inner ear development;GO:0071850|mitotic cell cycle arrest;GO:0043066|negative regulation of apoptotic process;GO:0030308|negative regulation of cell growth;GO:0051271|negative regulation of cellular component movement;GO:0045736|negative regulation of cyclin-dependent protein	GO:0031464|Cul4A-RING E3 ubiquitin ligase complex;GO:0005829|cytosol;GO:0005768|endosome;GO:0005634|nucleus;	GO:0004861|cyclin-dependent protein serine/threonine kinase inhibitor	K06624	CDKN1B, P27, KIP1; cyclin-dependent kinase inhibitor 1B
PGRMC2	3472.202	-0.33864	0.109582488	-3.09028	0.0019997	0.018293	Not_Sig	no	gi|555994728|ref|XP_005909113.1|	750	9e-90	PREDICTED: LOW QUALITY PROTEIN: membrane-associated progesterone receptor component 2 [Bos mutus]	gi|742203804|ref|XM_010860239.1|	2389	0	PREDICTED: Bison bison bison progesterone receptor membrane component 2 (PGRMC2), mRNA	O15173	281	6e-87	Membrane-associated progesterone receptor component 2	.	.	.	.	pfam00173	Cyt-b5	Cytochrome b5-like Heme/Steroid binding domain. This family includes heme binding domains from a diverse range of proteins.	.	GO:0012505|endomembrane system;GO:0016021|integral component of membrane;GO:0016020|membrane;GO:0005635|nuclear envelope;	GO:0020037|heme binding;GO:0005496|steroid binding;GO:0003707	K17278	PGRMC1_2; membrane-associated progesterone
ZC3H7B	751.4548	0.382664	0.12386096	3.089464	0.0020052	0.018329	Not_Sig	no	gi|742084672|ref|XP_010828396.1|	5037	0	PREDICTED: zinc finger CCCH domain-containing protein 7B isoform X3 [Bison bison bison]	gi|982927421|ref|XM_015471414.1|	5576	0	PREDICTED: Bos taurus zinc finger CCCH-type containing 7B (ZC3H7B), transcript variant X5, mRNA	Q9UGR2	1850	0	Zinc finger CCCH domain-containing protein 7B	SPAC17A2.04c	70.1	1.85e-11	COG0457	pfam13414	TPR_11	TPR repeat.	GO:0016032|viral process;	GO:0005634|nucleus;	GO:0046872|metal ion binding;GO:0044822|poly(A) RNA	.	.
SBSPON	66.36053	-0.74978	0.24272674	-3.08898	0.0020084	0.018344	DOWN	yes	gi|115497484|ref|NP_001069337.1|	1378	0	somatomedin-B and thrombospondin type-1 domain-containing protein precursor [Bos taurus]	gi|965806998|ref|XM_012119216.2|	2118	0	PREDICTED: Ovis aries musimon somatomedin B and thrombospondin type 1 domain containing (SBSPON), transcript variant X2, mRNA	Q32L50	535	0	Somatomedin-B and thrombospondin type-1 domain-containing protein	.	.	.	.	pfam05609	LAP1C	Lamina-associated polypeptide 1C (LAP1C). This family contains rat LAP1C proteins and several uncharacterized highly related	GO:0006955|immune response;	GO:0005578|proteinaceous extracellular matrix;	GO:0030247|polysaccharide binding;GO:0005044|scavenger receptor	.	.
CFL2	3460.647	-0.40107	0.129850711	-3.08874	0.0020101	0.018345	Not_Sig	no	gi|521025183|gb|EPQ06971.1|	845	1e-102	Cofilin-2 [Myotis brandtii]	gi|555959533|ref|XM_005891814.1|	2868	0	PREDICTED: Bos mutus cofilin 2 (muscle) (CFL2), mRNA	Q5G6V9	329	6e-104	Cofilin-2	.	.	.	.	pfam00241	Cofilin_ADF	Cofilin/tropomyosin-type actin-binding protein. Severs actin filaments and binds to actin monomers.	GO:0030042|actin filament depolymerization;GO:0046716|muscle cell cellular homeostasis;GO:0030836|positive regulation of actin filament depolymerization;GO:0045214|sarcomere organization;	GO:0015629|actin cytoskeleton;GO:0070062|extracellular exosome;GO:0005615|extracellular space;GO:0031674|I	GO:0051015|actin filament binding;	K05765	CFL; cofilin
SST	4830.276	0.460474	0.149134455	3.087645	0.0020175	0.018398	Not_Sig	no	gi|57163953|ref|NP_001009196.1|	595	1e-75	somatostatin precursor [Ovis aries]	gi|942066047|ref|XM_005899651.2|	620	0	PREDICTED: Bos mutus somatostatin (SST), mRNA	O46688	233	4e-77	Somatostatin	.	.	.	.	pfam03002	Somatostatin	Somatostatin/Cortistatin family. Members of this family are hormones. Somatostatin inhibits the release of somatotropin.	GO:0030334|regulation of cell migration;	GO:0005615|extracellular space;	.	K05237	SST; somatostatin
FOXS1	410.2455	0.436381	0.141424781	3.085604	0.0020314	0.018492	Not_Sig	no	gi|555951548|ref|XP_005887971.1|	1419	0	PREDICTED: forkhead box protein S1 [Bos mutus]	gi|942033288|ref|XM_005887909.2|	1251	0	PREDICTED: Bos mutus forkhead box S1 (FOXS1), mRNA	O43638	479	3e-167	Forkhead box protein S1	ECU11g1330	100	2.69e-23	COG5025	pfam00250	Fork_head	Fork head domain.	GO:0001568|blood vessel development;GO:0043433|negative regulation of sequence-specific DNA binding transcription factor activity;GO:0045892|negative regulation of transcription, DNA-templated;GO:0050885|neuromuscular process controlling balance;GO:0040018|positive regulation of multicellular organism growth;GO:0006351|transcription, DNA-templated;	GO:0005634|nucleus;	GO:0003677|DNA binding;GO:0000981|RNA polymerase II transcription factor	K09412	FKHL18; forkhead-like 18 protein
PGAM5	609.3346	0.349905	0.113404582	3.085459	0.0020324	0.018492	Not_Sig	no	gi|300794260|ref|NP_001179120.1|	1450	0	serine/threonine-protein phosphatase PGAM5, mitochondrial [Bos taurus]	gi|300794259|ref|NM_001192191.1|	1791	0	Bos taurus PGAM family member 5, serine/threonine protein phosphatase, mitochondrial (PGAM5), mRNA	Q96HS1	533	0	Serine/threonine-protein phosphatase PGAM5, mitochondrial	.	.	.	.	pfam00300	His_Phos_1	Histidine phosphatase superfamily (branch 1). The histidine phosphatase superfamily is so named because catalysis centres	GO:0070266|necroptotic process;	GO:0016021|integral component of membrane;GO:0005741|mitochondrial outer membrane;GO:0005739|mitochondrion;	GO:0005096|GTPase activator activity;GO:0016791|phosphatase	K15637	PGAM5; serine/threonine-protein phosphatase PGAM5 [EC:3.1.3.16]
C2CD3	424.0763	0.404134	0.130983163	3.085387	0.0020329	0.018492	Not_Sig	no	gi|942076983|ref|XP_014336099.1|	11910	0	PREDICTED: C2 domain-containing protein 3 isoform X2 [Bos mutus]	gi|742160660|ref|XM_010848851.1|	7763	0	PREDICTED: Bison bison bison C2 calcium-dependent domain containing 3 (C2CD3), transcript variant X1, mRNA	Q4AC94	3717	0	C2 domain-containing protein 3	.	.	.	.	pfam07111	HCR	Alpha helical coiled-coil rod protein (HCR). This family consists of several mammalian alpha helical coiled-coil rod HCR proteins. The	GO:0007420|brain development;GO:0061511|centriole elongation;GO:0042733|embryonic digit morphogenesis;GO:0001947|heart looping;GO:0001701|in utero embryonic development;GO:0021997|neural plate axis specification;GO:0021915|neural tube development;GO:0035058|nonmotile primary cilium assembly;GO:0071539|protein localization to centrosome;GO:0016485|protein processing;GO:0030162|regulation of proteolysis;GO:0008589|regulation of smoothened signaling pathway;	GO:0034451|centriolar satellite;GO:0005814|centriole;GO:0005813|centrosome;GO:0036064|ciliary basal body;GO:0005829|cytosol;	.	K16751	C2CD3; C2 domain-containing protein 3
HELZ	695.289	0.403379	0.130747054	3.085185	0.0020343	0.018492	Not_Sig	no	gi|742186954|ref|XP_010854352.1|	8929	0	PREDICTED: probable helicase with zinc finger domain isoform X1 [Bison bison bison]	gi|742186959|ref|XM_010856052.1|	6965	0	PREDICTED: Bison bison bison helicase with zinc finger (HELZ), transcript variant X3, mRNA	P42694	3214	0	Probable helicase with zinc finger domain	YKL017c	128	3.57e-29	COG1112	pfam13086	AAA_11	AAA domain. This family of domains contain a P-loop motif that is characteristic of the AAA superfamily. Many of the proteins	.	GO:0016020|membrane;GO:0005634|nucleus;	GO:0005524|ATP binding;GO:0004386|helicase activity;GO:0046872	.	.
ADAM9	3652.773	-0.28124	0.09118053	-3.08446	0.0020392	0.018522	Not_Sig	no	gi|328751692|ref|NP_001179747.2|	3905	0	disintegrin and metalloproteinase domain-containing protein 9 precursor [Bos taurus]	gi|555971156|ref|XM_005897532.1|	4014	0	PREDICTED: Bos mutus ADAM metallopeptidase domain 9 (ADAM9), mRNA	Q13443	1327	0	Disintegrin and metalloproteinase domain-containing protein 9	.	.	.	.	pfam01421	Reprolysin	Reprolysin (M12B) family zinc metalloprotease. The members of this family are enzymes that cleave peptides. These proteases require	GO:0000186|activation of MAPKK activity;GO:0007155|cell adhesion;GO:0033627|cell adhesion mediated by integrin;GO:0016477|cell migration;GO:0033631|cell-cell adhesion mediated by integrin;GO:0007160|cell-matrix adhesion;GO:0071222|cellular response to lipopolysaccharide;GO:0007229|integrin-mediated signaling pathway;GO:0030216|keratinocyte differentiation;GO:0006509|membrane protein ectodomain proteolysis;GO:0042117|monocyte activation;GO:0051088|PMA-inducible membrane protein ectodomain	GO:0016323|basolateral plasma membrane;GO:0009986|cell surface;GO:0070062|extracellular exosome;GO:0005615|extracellular	GO:0005518|collagen binding;GO:0005178|integrin	K06834	ADAM9; disintegrin and metalloproteinase domain-containing
DOT1L	1661.485	0.482226	0.156359866	3.084079	0.0020418	0.018531	Not_Sig	no	gi|528955922|ref|XP_005209125.1|	7034	0	PREDICTED: histone-lysine N-methyltransferase, H3 lysine-79 specific isoform X1 [Bos taurus]	gi|982984976|ref|XM_015464177.1|	7530	0	PREDICTED: Bos taurus DOT1-like histone H3K79 methyltransferase (DOT1L), transcript variant X2, mRNA	Q8TEK3	2152	0	Histone-lysine N-methyltransferase, H3 lysine-79 specific	.	.	.	.	pfam08123	DOT1	Histone methylation protein DOT1. The DOT1 domain regulates gene expression by methylating histone H3. H3 methylation by DOT1 has	GO:0006348|chromatin silencing at telomere;GO:0000077|DNA damage checkpoint;GO:0006281|DNA repair;GO:0034729|histone H3-K79 methylation;GO:0008284|positive regulation of cell proliferation;GO:0045944|positive regulation of transcription from RNA polymerase II promoter;GO:0051726|regulation of cell cycle;GO:0046425|regulation of JAK-STAT cascade;GO:2000677|regulation of transcription regulatory region DNA binding;GO:0032200|telomere organization;	GO:0000781|chromosome, telomeric region;GO:0005654|nucleoplasm;GO:0005634|nucleus;GO:0043234|protein complex;	GO:0003677|DNA binding;GO:0042054|histone methyltransferase	K11427	DOT1L, DOT1; histone-lysine N-methyltransferase, H3 lysine-79 specific
MICALL1	1653.452	0.429799	0.139381683	3.083609	0.0020451	0.018546	Not_Sig	no	gi|528951407|ref|XP_005207382.1|	3307	0	PREDICTED: MICAL-like protein 1 isoform X1 [Bos taurus]	gi|982927272|ref|XM_005207325.3|	4358	0	PREDICTED: Bos taurus MICAL like 1 (MICALL1), transcript variant X1, mRNA	E1BBG2	1271	0	MICAL-like protein 1	ECU09g0290	112	1.65e-24	COG5069	pfam12130	DUF3585	Protein of unknown function (DUF3585). This domain is found in eukaryotes. This domain is typically between 135 and 149	GO:0006897|endocytosis;GO:0097320|membrane tubulation;GO:0031175|neuron projection development;GO:0036010|protein localization to endosome;GO:0006612|protein targeting to membrane;GO:0006898|receptor-mediated endocytosis;GO:1990126|retrograde transport, endosome to plasma membrane;GO:0032458|slow endocytic recycling;	GO:0019898|extrinsic component of membrane;GO:0005770|late endosome;GO:0031902|late endosome membrane;GO:0055038|recycling	GO:0070300|phosphatidic acid binding;GO:0008270|zinc ion binding;	.	.
GRN	1793.427	0.455516	0.147744133	3.083142	0.0020483	0.01856	Not_Sig	no	gi|555991947|ref|XP_005907753.1|	3014	0	PREDICTED: granulins [Bos mutus]	gi|983002255|ref|XM_010824214.2|	2193	0	PREDICTED: Bos taurus granulin (GRN), mRNA	P28799	917	0	Granulins	.	.	.	.	pfam00396	Granulin	Granulin.	GO:0007165|signal transduction;	GO:0070062|extracellular exosome;GO:0005615|extracellular space;GO:0043231|intracellular membrane-bounded organelle;	GO:0008083|growth factor activity;GO:0044822|poly(A) RNA	.	.
SHC1	2344.23	0.36549	0.118569017	3.08251	0.0020526	0.018568	Not_Sig	no	gi|965916346|ref|XP_014947759.1|	2644	0	PREDICTED: SHC-transforming protein 1 isoform X4 [Ovis aries]	gi|982916325|ref|XM_010802886.2|	3583	0	PREDICTED: Bos taurus SHC (Src homology 2 domain containing) transforming protein 1 (SHC1), transcript variant X1, mRNA	P29353	942	0	SHC-transforming protein 1	.	.	.	.	pfam00640	PID	Phosphotyrosine interaction domain (PTB/PID).	GO:0031532|actin cytoskeleton reorganization;GO:0000187|activation of MAPK activity;GO:0001525|angiogenesis;GO:0071363|cellular response to growth factor stimulus;GO:0007173|epidermal growth factor receptor signaling pathway;GO:0038128|ERBB2 signaling pathway;GO:0038095|Fc-epsilon receptor signaling pathway;GO:0007507|heart development;GO:0008286|insulin receptor signaling pathway;GO:0036498|IRE1-mediated unfolded protein response;GO:0050900|leukocyte migration;GO:0000165|MAPK	GO:0005829|cytosol;GO:0005759|mitochondrial matrix;GO:0005886|plasma membrane;GO:0070435|Shc-EGFR complex;	GO:0046875|ephrin receptor binding;GO:0005154|epidermal growth	K06279	SHC1; SHC-transforming protein 1
COMMD6	78.3533	-0.70431	0.228497477	-3.08235	0.0020538	0.018568	DOWN	yes	gi|982949880|ref|XP_015330886.1|	309	4e-29	PREDICTED: COMM domain-containing protein 6 [Bos taurus]	gi|850486327|gb|CP011895.1|	3970	0	Ovis canadensis canadensis isolate 43U chromosome 10 sequence	Q2KIY0	123	2e-30	COMM domain-containing protein 6	.	.	.	.	pfam07258	HCaRG	HCaRG protein. This family consists of several mammalian HCaRG(hypertension-related, calcium-regulated gene) proteins.	GO:0006355|regulation of transcription, DNA-templated;GO:0006351|transcription, DNA-templated;	GO:0005737|cytoplasm;GO:0005634|nucleus;	.	.	.
TBC1D24	587.5889	0.42698	0.138525547	3.082317	0.002054	0.018568	Not_Sig	no	gi|942028809|ref|XP_014336235.1|	2609	0	PREDICTED: TBC1 domain family member 24 [Bos mutus]	gi|982966187|ref|XM_010819102.2|	4012	0	PREDICTED: Bos taurus TBC1 domain family member 24 (TBC1D24), transcript variant X11, mRNA	Q9ULP9	947	0	TBC1 domain family member 24	.	.	.	.	pfam07534	TLD	TLD. This domain is predicted to be an enzyme and is often found associated with pfam01476. It's structure consists of a beta-	GO:0031175|neuron projection development;	GO:0005737|cytoplasm;GO:0031594|neuromuscular junction;GO:0043195|terminal bouton;	GO:0005096|GTPase activator activity;	.	.
UBE2Q2	1062.426	-0.40122	0.130183963	-3.08198	0.0020563	0.018575	Not_Sig	no	gi|148693894|gb|EDL25841.1|	1817	0	ubiquitin-conjugating enzyme E2Q (putative) 2, isoform CRA_a, partial [Mus musculus]	gi|528999249|ref|XM_005221922.1|	2876	0	PREDICTED: Bos taurus ubiquitin conjugating enzyme E2Q family member 2 (UBE2Q2), transcript variant X1, mRNA	Q8WVN8	681	0	Ubiquitin-conjugating enzyme E2 Q2	.	.	.	.	pfam00179	UQ_con	Ubiquitin-conjugating enzyme. Proteins destined for proteasome-mediated degradation may be ubiquitinated. Ubiquitination	GO:0070936|protein K48-linked ubiquitination;	GO:0005737|cytoplasm;	GO:0005524|ATP binding;GO:0004842|ubiquitin-protein transferase activity;	K10582	UBE2Q; ubiquitin-conjugating enzyme E2 Q [EC:2.3.2.23]
AMACR	525.7371	-0.3593	0.116613602	-3.08113	0.0020621	0.018605	Not_Sig	no	gi|119913313|ref|XP_593629.3|	2013	0	PREDICTED: alpha-methylacyl-CoA racemase [Bos taurus]	gi|742178354|ref|XM_010853827.1|	1576	0	PREDICTED: Bison bison bison alpha-methylacyl-CoA racemase (AMACR), mRNA	Q9UHK6	675	0	Alpha-methylacyl-CoA racemase	Rv1143	337	1.38e-111	COG1804	pfam02515	CoA_transf_3	CoA-transferase family III. CoA-transferases are found in organisms from all lines of descent. Most of these enzymes	GO:0006699|bile acid biosynthetic process;GO:0008206|bile acid metabolic process;GO:0033540|fatty acid beta-oxidation using acyl-CoA oxidase;	GO:0005737|cytoplasm;GO:0005739|mitochondrion;GO:0005782|peroxisomal matrix;GO:0005777|peroxisome;	GO:0008111|alpha-methylacyl-CoA racemase activity;GO:0005102	K01796	E5.1.99.4, AMACR, mcr; alpha-methylacyl-CoA racemase
ZZEF1	752.7946	0.359643	0.116728824	3.081017	0.0020629	0.018605	Not_Sig	no	gi|528994820|ref|XP_005220258.1|	14837	0	PREDICTED: zinc finger ZZ-type and EF-hand domain-containing protein 1 isoform X1 [Bos taurus]	gi|982956521|ref|XM_005220201.3|	11292	0	PREDICTED: Bos taurus zinc finger, ZZ-type with EF-hand domain 1 (ZZEF1), transcript variant X1, mRNA	O43149	5242	0	Zinc finger ZZ-type and EF-hand domain-containing protein 1	.	.	.	.	pfam03256	APC10	Anaphase-promoting complex, subunit 10 (APC10).	.	.	GO:0005509|calcium ion binding;GO:0008270|zinc ion binding;	.	.
TXNRD1	6932.431	-0.26675	0.086591946	-3.08051	0.0020665	0.018614	Not_Sig	no	gi|555974279|ref|XP_005899133.1|	2577	0	PREDICTED: thioredoxin reductase 1, cytoplasmic isoform X1 [Bos mutus]	gi|942064232|ref|XM_005899071.2|	3338	0	PREDICTED: Bos mutus thioredoxin reductase 1 (TXNRD1), transcript variant X1, mRNA	O62768	959	0	Thioredoxin reductase 1, cytoplasmic	HI0161	267	1.90e-78	COG1249	pfam07992	Pyr_redox_2	Pyridine nucleotide-disulphide oxidoreductase. This family includes both class I and class II oxidoreductases and also NADH	GO:0045454|cell redox homeostasis;GO:0001887|selenium compound metabolic process;	GO:0005829|cytosol;	GO:0050660|flavin adenine dinucleotide binding;GO:0098623	K00384	trxB; thioredoxin reductase (NADPH) [EC:1.8.1.9]
SMARCA4	1898.394	0.325307	0.105609728	3.080272	0.0020681	0.018614	Not_Sig	no	gi|742218560|ref|XP_010826637.1|	6430	0	PREDICTED: transcription activator BRG1 isoform X1 [Bison bison bison]	gi|742218559|ref|XM_010828335.1|	5379	0	PREDICTED: Bison bison bison SWI/SNF related, matrix associated, actin dependent regulator of chromatin, subfamily a, member 4 (SMARCA4), transcript variant X1, mRNA	P51532	2423	0	Transcription activator BRG1	.	.	.	.	pfam00176	SNF2_N	SNF2 family N-terminal domain. This domain is found in proteins involved in a variety of processes including transcription regulation	GO:0043044|ATP-dependent chromatin remodeling;GO:0016569|covalent chromatin modification;GO:0006338|chromatin remodeling;GO:0060766|negative regulation of androgen receptor signaling pathway;GO:0030308|negative regulation of cell growth;GO:2000134|negative regulation of G1/S transition of mitotic cell cycle;GO:0000122|negative regulation of transcription from RNA polymerase II promoter;GO:0007070|negative regulation of transcription from RNA polymerase II promoter during	GO:0005615|extracellular space;GO:0016020|membrane;GO:0071565|nBAF complex;GO:0071564|npBAF complex;GO:0000790|nuclear	GO:0050681|androgen receptor binding;GO:0005524|ATP	K11647	SMARCA2_4; SWI/SNF-related matrix-associated actin-dependent
UBE2D1	144.0791	-0.53727	0.174427232	-3.08018	0.0020688	0.018614	Not_Sig	no	gi|313754445|pdb|3OJ4|A	790	4e-96	Chain A, Crystal Structure Of The A20 Znf4, Ubiquitin And Ubch5a Complex	gi|965906749|ref|XM_012166341.2|	2367	0	PREDICTED: Ovis aries musimon ubiquitin conjugating enzyme E2D 1 (UBE2D1), transcript variant X3, mRNA	D3ZDK2	308	1e-97	Ubiquitin-conjugating enzyme E2 D1	SPBC119.02	259	7.26e-81	COG5078	pfam00179	UQ_con	Ubiquitin-conjugating enzyme. Proteins destined for proteasome-mediated degradation may be ubiquitinated. Ubiquitination	GO:0031398|positive regulation of protein ubiquitination;GO:0070936|protein K48-linked ubiquitination;GO:0006511|ubiquitin-dependent protein catabolic process;	GO:0005737|cytoplasm;GO:0000151|ubiquitin ligase complex;	GO:0005524|ATP binding;GO:0061631|ubiquitin conjugating enzyme	K06689	UBE2D_E, UBC4, UBC5; ubiquitin-conjugating enzyme E2 D/E [EC:2.3.2.23]
EP400	861.9024	0.339799	0.110371643	3.07868	0.0020792	0.018687	Not_Sig	no	gi|528988296|ref|XP_005217726.1|	10584	0	PREDICTED: E1A-binding protein p400 isoform X3 [Bos taurus]	gi|982950039|ref|XM_005217669.3|	11984	0	PREDICTED: Bos taurus E1A binding protein p400 (EP400), transcript variant X4, mRNA	Q96L91	3172	0	E1A-binding protein p400	YDR334w	290	5.18e-78	COG0553	pfam00176	SNF2_N	SNF2 family N-terminal domain. This domain is found in proteins involved in a variety of processes including transcription regulation	GO:0043968|histone H2A acetylation;GO:0043967|histone H4 acetylation;	GO:0035267|NuA4 histone acetyltransferase complex;GO:0016607|nuclear speck;GO:0005654|nucleoplasm;GO:000081	GO:0005524|ATP binding;GO:0003682|chromatin binding;GO:0003677	K11320	EP400; E1A-binding protein p400 [EC:3.6.4.-]
LOC138987895	425.4942	0.607904	0.197464357	3.078549	0.0020801	0.018687	UP	yes	gi|556719699|ref|XP_005955730.1|	5860	0	PREDICTED: TRIO and F-actin-binding protein [Pantholops hodgsonii]	gi|942053145|ref|XM_005895237.2|	3131	0	PREDICTED: Bos mutus TRIO and F-actin binding protein (TRIOBP), mRNA	Q99KW3	1174	0	TRIO and F-actin-binding protein	.	.	.	.	pfam09770	PAT1	Topoisomerase II-associated protein PAT1. Members of this family are necessary for accurate chromosome transmission during	GO:0051301|cell division;GO:0007067|mitotic nuclear division;GO:1900026|positive regulation of substrate adhesion-dependent cell spreading;GO:0032956|regulation of actin cytoskeleton organization;	GO:0015629|actin cytoskeleton;GO:0005737|cytoplasm;GO:0005925|focal adhesion;GO:0005815|microtubule	GO:0051015|actin filament binding;GO:0031625|ubiquitin protein	.	.
SMARCB1	823.1677	0.35246	0.114500541	3.078235	0.0020823	0.018692	Not_Sig	no	gi|94966901|ref|NP_001035647.1|	1980	0	SWI/SNF-related matrix-associated actin-dependent regulator of chromatin subfamily B member 1 [Bos taurus]	gi|126723391|ref|NM_001040557.2|	1591	0	Bos taurus SWI/SNF related, matrix associated, actin dependent regulator of chromatin, subfamily b, member 1 (SMARCB1), mRNA	Q5BIN2	767	0	SWI/SNF-related matrix-associated actin-dependent regulator of chromatin subfamily B	.	.	.	.	pfam04855	SNF5	SNF5 / SMARCB1 / INI1. SNF5 is a component of the yeast SWI/SNF complex, which is an ATP-dependent nucleosome-	GO:0043044|ATP-dependent chromatin remodeling;GO:0001835|blastocyst hatching;GO:0007049|cell cycle;GO:0030154|cell differentiation;GO:0016569|covalent chromatin modification;GO:0006281|DNA repair;GO:0008285|negative regulation of cell proliferation;GO:1900110|negative regulation of histone H3-K9 dimethylation;GO:1900113|negative regulation of histone H3-K9 trimethylation;GO:0007399|nervous system development;GO:0006337|nucleosome disassembly;GO:0043923|positive regulation by host of	GO:0090544|BAF-type complex;GO:0071565|nBAF complex;GO:0071564|npBAF complex;GO:0000790|nuclear	GO:0003677|DNA binding;GO:0001164|RNA polymerase I CORE element	K11648	SMARCB1, SNF5, INI1; SWI/SNF-related matrix-associated actin-
ADISSP	384.4737	0.448836	0.145977043	3.074704	0.0021071	0.018888	Not_Sig	no	gi|555963887|ref|XP_005894018.1|	922	3e-120	PREDICTED: UPF0687 protein C20orf27 homolog isoform X2 [Bos mutus]	gi|402744548|ref|NM_001046205.2|	1245	0	Bos taurus chromosome 13 open reading frame, human C20orf27 (C13H20orf27), mRNA	Q2KIM1	359	1e-121	UPF0687 protein C20orf27 homolog	.	.	.	.	pfam15006	DUF4517	Domain of unknown function (DUF4517). The function of this protein remains unknown. This	.	.	.	.	.
FBRSL1	528.556	0.478912	0.15576153	3.074651	0.0021075	0.018888	Not_Sig	no	gi|803306763|ref|XP_012011799.1|	3014	0	PREDICTED: fibrosin-1-like protein isoform X19 [Ovis aries musimon]	gi|965887850|ref|XM_012156409.2|	4297	0	PREDICTED: Ovis aries musimon fibrosin-1-like protein (LOC101116937), transcript variant X19, mRNA	Q9HCM7	675	0	Fibrosin-1-like protein	.	.	.	.	pfam15336	Auts2	family of proteins is found in Autism susceptibility gene 2 protein. Auts2, or FBRSL2, Fibrosin-1-like protein 2, is a family of eukaryotic proteins associated	.	.	GO:0044822|poly(A) RNA binding;	.	.
ARRDC4	575.4132	-0.36046	0.117263145	-3.07398	0.0021123	0.018916	Not_Sig	no	gi|300796255|ref|NP_001178963.1|	1987	0	arrestin domain-containing protein 4 [Bos taurus]	gi|893688328|gb|KP731623.1|	3957	0	Bos taurus arrestin domain-containing protein 4 (ARRDC4) mRNA, complete cds	Q8NCT1	731	0	Arrestin domain-containing protein 4	.	.	.	.	pfam00339	Arrestin_N	Arrestin (or S-antigen), N-terminal domain. Ig-like beta-sandwich fold. Scop reports duplication with C-terminal domain.	GO:0051443|positive regulation of ubiquitin-protein transferase activity;	GO:0016023|cytoplasmic membrane-bounded vesicle;GO:0005769|early endosome;GO:0005768|endosome;GO:0005886|plasma membrane;	.	.	.
SRP9	670.9083	-0.51785	0.16848233	-3.07362	0.0021148	0.018916	Not_Sig	no	gi|73961487|ref|XP_854739.1|	451	5e-51	PREDICTED: signal recognition particle 9 kDa protein [Canis lupus familiaris]	gi|942078436|ref|XM_005903715.2|	1470	0	PREDICTED: Bos mutus signal recognition particle 9kDa (SRP9), mRNA	P21262	176	1e-51	Signal recognition particle 9 kDa protein	.	.	.	.	pfam05486	SRP9-21	Signal recognition particle 9 kDa protein (SRP9). This family consists of several eukaryotic SRP9 proteins. SRP9 together with the	GO:0045900|negative regulation of translational elongation;GO:0006614|SRP-dependent cotranslational protein targeting to membrane;GO:0006616|SRP-dependent cotranslational protein targeting to membrane, translocation;	GO:0005829|cytosol;GO:0005785|signal recognition particle receptor complex;GO:0005786|signal recognition particle, endoplasmic reticulum targeting;	GO:0008312|7S RNA binding;GO:0005047|signal recognition	.	.
C1GALT1	487.6232	-0.49402	0.160760905	-3.07302	0.002119	0.018916	Not_Sig	no	gi|528945924|ref|XP_005205214.1|	2048	0	PREDICTED: glycoprotein-N-acetylgalactosamine 3-beta-galactosyltransferase 1 isoform X1 [Bos taurus]	gi|742117366|ref|XM_010839731.1|	5846	0	PREDICTED: Bison bison bison core 1 synthase, glycoprotein-N-acetylgalactosamine 3-beta-galactosyltransferase 1 (C1GALT1), transcript variant X3, mRNA	Q0VC84	778	0	Glycoprotein-N-acetylgalactosamine 3-beta-galactosyltransferase 1	.	.	.	.	pfam02434	Fringe	Fringe-like. The drosophila protein fringe (FNG) is a glucosaminyltransferase that controls the response of the Notch	GO:0001525|angiogenesis;GO:0060576|intestinal epithelial cell development;GO:0001822|kidney development;GO:0016267|O-glycan processing, core 1;	GO:0016021|integral component of membrane;	GO:0016263|glycoprotein-N-acetylgalactosamine 3-beta-	K00731	C1GALT1; glycoprotein-N-acetylgalactosamine 3-beta-
TPRKB	201.0402	-0.51338	0.167065926	-3.07294	0.0021196	0.018916	Not_Sig	no	gi|556757463|ref|XP_005974074.1|	975	3e-125	PREDICTED: TP53RK-binding protein-like [Pantholops hodgsonii]	gi|942059435|ref|XM_005897632.2|	1472	0	PREDICTED: Bos mutus TP53RK binding protein (TPRKB), transcript variant X2, mRNA	Q9Y3C4	325	9e-106	EKC/KEOPS complex subunit TPRKB	.	.	.	.	pfam08617	CGI-121	Kinase binding protein CGI-121. CGI-121 has been shown to bind to the p53-related protein kinase (PRPK). PRPK is a novel protein	GO:0008033|tRNA processing;	GO:0005737|cytoplasm;GO:0005829|cytosol;GO:0005634|nucleus;	GO:0019901|protein kinase binding;	K15901	CGI121, TPRKB; EKC/KEOPS complex subunit CGI121/TPRKB

GIT2	1051.851	0.350854	0.11417845	3.072854	0.0021202	0.018916	Not_Sig	no	gi|300797130|ref|NP_001180030.1|	3840	0	ARF GTPase-activating protein GIT2 [Bos taurus]	gi|982950297|ref|XM_005217833.3|	5316	0	PREDICTED: Bos taurus G protein-coupled receptor kinase interacting ArfGAP 2 (GIT2), transcript variant X1, mRNA	Q14161	1420	0	ARF GTPase-activating protein GIT2	SPBC17G9.08c_2	62.8	6.83e-10	COG5347	pfam12205	GIT1_C	G protein-coupled receptor kinase-interacting protein 1 C term. This domain family is found in eukaryotes, and is approximately	GO:0048266|behavioral response to pain;GO:0008277|regulation of G-protein coupled receptor protein signaling pathway;	GO:0005925|focal adhesion;GO:0005654|nucleoplasm;	GO:0005096|GTPase activator activity;GO:0046872|metal ion binding;	K12487	GIT2; G protein-coupled receptor kinase interactor 2
GPS2	314.6043	0.43724	0.142300168	3.072663	0.0021216	0.018916	Not_Sig	no	gi|594623780|ref|XP_007166515.1|	1332	2e-180	PREDICTED: G protein pathway suppressor 2 [Balaenoptera acutorostrata scammoni]	gi|594072776|ref|XM_006060156.1|	1209	0	PREDICTED: Bubalus bubalis G protein pathway suppressor 2 (GPS2), transcript variant X1, mRNA	Q13227	505	3e-177	G protein pathway suppressor 2	.	.	.	.	pfam11600	CAF-1_p150	Chromatin assembly factor 1 complex p150 subunit, N-terminal. CAF-1_p150 is a polypeptide subunit of CAF-1, which functions	GO:0007049|cell cycle;GO:0000188|inactivation of MAPK activity;GO:0007254|JNK cascade;GO:0046329|negative regulation of JNK cascade;GO:0000122|negative regulation of transcription from RNA polymerase II promoter;	GO:0005654|nucleoplasm;GO:0017053|transcriptional repressor complex;	GO:0005095|GTPase inhibitor activity;GO:0003714|transcription	K15307	GPS2; G protein pathway suppressor 2
MAGT1	1180.478	-0.38623	0.125712571	-3.07231	0.0021241	0.018916	Not_Sig	no	gi|926728581|ref|XP_005700661.2|	1699	0	PREDICTED: magnesium transporter protein 1 [Capra hircus]	gi|555953116|ref|XM_005888675.1|	2859	0	PREDICTED: Bos mutus magnesium transporter 1 (MAGT1), mRNA	Q9H0U3	631	0	Magnesium transporter protein 1	.	.	.	.	pfam04756	OST3_OST6	OST3 / OST6 family. The proteins in this family are part of a complex of eight ER proteins that transfers core oligosaccharide from dolichol	GO:0050890|cognition;GO:0015693|magnesium ion transport;GO:0006487|protein N-linked glycosylation;GO:0018279|protein N-linked glycosylation via asparagine;GO:0055085|transmembrane transport;	GO:0005887|integral component of plasma membrane;GO:0016020|membrane;GO:0008250|oligosaccharyltransferase complex;GO:0005886|plasma membrane;	GO:0015095|magnesium ion transmembrane transporter activity;	K19478	MAGT1; magnesium transporter 1
DERPC	488.0723	0.371973	0.12107786	3.072184	0.002125	0.018916	Not_Sig	no	gi|440905407|gb|ELR55784.1|	1684	0	Chromosome transmission fidelity protein 8-like protein isoform 2 [Bos mutus]	gi|555969093|ref|XM_005896513.1|	1868	0	PREDICTED: Bos mutus chromosome transmission fidelity factor 8 (CHTF8), mRNA	P0CG10	650	0	Chromosome transmission fidelity protein 8 homolog isoform 2	.	.	.	.	pfam08580	KAR9	Yeast cortical protein KAR9. The KAR9 protein in Saccharomyces cerevisiae is a cytoskeletal protein required for karyogamy, correct	.	GO:0005634|nucleus;	.	K11270	CTF8; chromosome transmission fidelity protein 8
MTHFD2	1014.939	-0.33228	0.108160917	-3.07213	0.0021254	0.018916	Not_Sig	no	gi|594041888|ref|XP_006045485.1|	1817	0	PREDICTED: bifunctional methylenetetrahydrofolate dehydrogenase/cyclohydrolase, mitochondrial [Bubalus bubalis]	gi|942059444|ref|XM_005897648.2|	2181	0	PREDICTED: Bos mutus methylenetetrahydrofolate dehydrogenase (NADP+ dependent) 2, methenyltetrahydrofolate cyclohydrolase	Q0P5C2	663	0	Bifunctional methylenetetrahydrofolate dehydrogenase/cyclohydr	BS_folD	248	3.55e-76	COG0190	pfam02882	THF_DHG_CYH_C	Tetrahydrofolate dehydrogenase/cyclohydrolase, NAD(P)-binding domain.	GO:0009396|folic acid-containing compound biosynthetic process;GO:0006730|one-carbon metabolic process;GO:0046653|tetrahydrofolate metabolic process;	GO:0005615|extracellular space;GO:0005739|mitochondrion;	GO:0000287|magnesium ion binding;GO:0004477|methenyltetrahydr	K13403	MTHFD2; methylenetetrahydrofolate dehydrogenase(NAD
CLPTM1L	1732.387	0.354399	0.115388926	3.07134	0.002131	0.018938	Not_Sig	no	gi|528998529|ref|XP_005221717.1|	2713	0	PREDICTED: cleft lip and palate transmembrane protein 1-like protein isoform X1 [Bos taurus]	gi|402692899|ref|NM_001082426.2|	2116	0	Bos taurus CLPTM1-like (CLPTM1L), mRNA	A2VE61	1037	0	Cleft lip and palate transmembrane protein 1-like protein	.	.	.	.	pfam05602	CLPTM1	Cleft lip and palate transmembrane protein 1 (CLPTM1). This family consists of several eukaryotic cleft lip and	GO:0006915|apoptotic process;	GO:0016021|integral component of membrane;	.	.	.
GANAB	4436.115	0.288578	0.093959331	3.07131	0.0021312	0.018938	Not_Sig	no	gi|440898141|gb|ELR49696.1|	5001	0	Neutral alpha-glucosidase AB, partial [Bos mutus]	gi|555986060|ref|XM_005904834.1|	3706	0	PREDICTED: Bos mutus glucosidase, alpha; neutral AB (GANAB), transcript variant X1, mRNA	P79403	1764	0	Neutral alpha-glucosidase AB	SPAC1002.03c	640	0	COG1501	pfam01055	Glyco_hydro_31	Glycosyl hydrolases family 31. Glycosyl hydrolases are key enzymes of carbohydrate metabolizm. Family 31 comprises	GO:0005975|carbohydrate metabolic process;	GO:0005783|endoplasmic reticulum;GO:0005794|Golgi apparatus;GO:0042470|melanosome;	GO:0030246|carbohydrate binding;GO:0033919|glucan 1,3-alpha-	K05546	GANAB; alpha 1,3-glucosidase [EC:3.2.1.84]
MPPE1	126.6242	0.598241	0.194806486	3.070951	0.0021338	0.018938	UP	yes	gi|803314016|ref|XP_012015321.1|	2143	0	PREDICTED: metallophosphoesterase 1 isoform X3 [Ovis aries musimon]	gi|942045175|ref|XM_014477170.1|	2088	0	PREDICTED: Bos mutus metallophosphoesterase 1 (MPPE1), mRNA	D2I2M6	580	0	Metallophosphoesterase 1	.	.	.	.	pfam00149	Metallophos	Calcineurin-like phosphoesterase. This family includes a diverse range of phosphoesterases, including protein phosphoserine	GO:0006888|ER to Golgi vesicle-mediated transport;GO:0006506|GPI anchor biosynthetic process;	GO:0005801|cis-Golgi network;GO:0070971|endoplasmic reticulum exit site;GO:0005793|endoplasmic reticulum-	GO:0034235|GPI anchor binding;GO:0030145|manganese ion	.	.
LOC102270686	840.4699	-0.34942	0.113784776	-3.07086	0.0021345	0.018938	Not_Sig	no	gi|440904993|gb|ELR55443.1|	1701	0	Purine nucleoside phosphorylase, partial [Bos mutus]	gi|742114801|ref|XM_010839111.1|	1311	0	PREDICTED: Bison bison bison purine nucleoside phosphorylase (LOC104987976), mRNA	P55859	577	0	Purine nucleoside phosphorylase	YLR209c	272	4.56e-88	COG0005	pfam01048	PNP_UDP_1	Phosphorylase superfamily. Members of this family include: purine nucleoside phosphorylase (PNP) Uridine phosphorylase	GO:0009116|nucleoside metabolic process;GO:0043101|purine-containing compound salvage;	GO:0005737|cytoplasm;GO:0005856|cytoskeleton;	GO:0004731|purine-nucleoside phosphorylase activity;	.	.
NACC2	81.39487	0.819165	0.266779666	3.070567	0.0021365	0.018942	UP	yes	gi|300798379|ref|NP_001179578.1|	2552	0	nucleus accumbens-associated protein 2 [Bos taurus]	gi|965804495|ref|XM_015098068.1|	3236	0	PREDICTED: Ovis aries musimon NACC family member 2, BEN and BTB (POZ) domain containing (NACC2), transcript variant X3, mRNA	Q96BF6	947	0	Nucleus accumbens-associated protein 2	.	.	.	.	pfam00651	BTB	BTB/POZ domain. The BTB (for BR-C, ttk and bab) or POZ (for Pox virus and Zinc finger) domain is present near the N-terminus of a	GO:0034629|cellular protein complex localization;GO:0008285|negative regulation of cell proliferation;GO:1900477|negative regulation of G1/S transition of mitotic cell cycle by negative regulation of transcription from RNA polymerase II promoter;GO:0045892|negative regulation of transcription, DNA-templated;GO:0008284|positive regulation of cell proliferation;GO:1902231|positive regulation of intrinsic apoptotic signaling pathway in response to DNA damage;GO:0010608|posttranscriptional regulation of gene expression;GO:0051260|protein	GO:0016604|nuclear body;GO:0000790|nuclear chromatin;GO:0005634|nucleus;	GO:0004407|histone deacetylase activity;GO:0042826|histone	K10486	BTBD14; BTB/POZ domain-containing protein 14
LYSMD3	455.7075	-0.48155	0.156841616	-3.07031	0.0021384	0.018944	Not_Sig	no	gi|555991627|ref|XP_005907597.1|	1553	0	PREDICTED: lysM and putative peptidoglycan-binding domain-containing protein 3 [Bos mutus]	gi|742237322|ref|XM_010831921.1|	4266	0	PREDICTED: Bison bison bison LysM, putative peptidoglycan-binding, domain containing 3 (LYSMD3), mRNA	Q7Z3D4	540	3e-178	LysM and putative peptidoglycan-binding domain-containing protein 3	.	.	.	.	pfam01476	LysM	LysM domain. The LysM (lysin motif) domain is about 40 residues long. It is found in a variety of enzymes involved in bacterial cell	.	GO:0016021|integral component of membrane;	.	.	.
KIAA1217	3227.946	0.280696	0.091452651	3.069306	0.0021456	0.018977	Not_Sig	no	gi|982992663|ref|XP_015321400.1|	8162	0	PREDICTED: sickle tail protein homolog isoform X5 [Bos taurus]	gi|742202419|ref|XM_010859913.1|	6530	0	PREDICTED: Bison bison bison KIAA1217 ortholog (KIAA1217), transcript variant X1, mRNA	Q5T5P2	2486	0	Sickle tail protein homolog	.	.	.	.	pfam03915	AIP3	Actin interacting protein 3.	GO:0048706|embryonic skeletal system development;	GO:0005737|cytoplasm;	.	.	.
LPIN3	170.2039	0.542651	0.176806279	3.069187	0.0021464	0.018977	Not_Sig	no	gi|942038459|ref|XP_014331876.1|	4112	0	PREDICTED: phosphatidate phosphatase LPIN3 [Bos mutus]	gi|982943073|ref|XM_015474152.1|	4173	0	PREDICTED: Bos taurus lipin 3 (LPIN3), transcript variant X2, mRNA	Q9BQK8	1293	0	Phosphatidate phosphatase LPIN3	YMR165c_1	228	2.77e-62	COG5083	pfam08235	LNS2	LNS2 (Lipin/Ned1/Smp2). This domain is found in Saccharomyces cerevisiae protein SMP2, proteins with an N-terminal lipin domain	GO:0009062|fatty acid catabolic process;GO:0006656|phosphatidylcholine biosynthetic process;GO:0006646|phosphatidylethanolamine biosynthetic process;GO:0045944|positive regulation of transcription from RNA polymerase II promoter;GO:0019432|triglyceride biosynthetic process;	GO:0005789|endoplasmic reticulum membrane;GO:0005634|nucleus;	GO:0008195|phosphatidate phosphatase activity;GO:0003713	K15728	LPIN; phosphatidate phosphatase LPIN [EC:3.1.3.4]
LOC102270382	1318.69	0.375275	0.12227573	3.069091	0.0021471	0.018977	Not_Sig	no	gi|359065316|ref|XP_003586100.1|	2261	0	PREDICTED: tubulin alpha-1C chain-like [Bos taurus]	gi|741925901|ref|XM_005206263.2|	1515	0	PREDICTED: Bos taurus tubulin, alpha 1c (TUBA1C), transcript variant X1, mRNA	Q3ZCJ7	875	0	Tubulin alpha-1C chain	SPBC800.05c	709	0	COG5023	pfam03953	Tubulin_C	Tubulin C-terminal domain. This family includes the tubulin alpha, beta and gamma chains. Members of this family are involved in	GO:0007017|microtubule-based process;	GO:0005737|cytoplasm;GO:0005874|microtubule;	GO:0005525|GTP binding;GO:0003924|GTPase activity;GO:0005200	K07374	TUBA; tubulin alpha
ELMO2	1047.128	0.354892	0.115649841	3.068676	0.0021501	0.018987	Not_Sig	no	gi|594054351|ref|XP_006051442.1|	3897	0	PREDICTED: engulfment and cell motility protein 2 isoform X2 [Bubalus bubalis]	gi|555971218|ref|XM_005897562.1|	3475	0	PREDICTED: Bos mutus engulfment and cell motility 2 (ELMO2), transcript variant X2, mRNA	Q96JJ3	1464	0	Engulfment and cell motility protein 2	.	.	.	.	pfam11841	DUF3361	Domain of unknown function (DUF3361). This domain is functionally uncharacterized. This domain is found in eukaryotes.	GO:0006915|apoptotic process;GO:0060326|cell chemotaxis;GO:0098609|cell-cell adhesion;GO:0007010|cytoskeleton organization;GO:0038096|Fc-gamma receptor signaling pathway involved in phagocytosis;GO:0048010|vascular endothelial growth factor receptor signaling pathway;	GO:0005737|cytoplasm;GO:0005829|cytosol;GO:0016020|membrane;	GO:0030971|receptor tyrosine kinase binding;	K18985	ELMO2, CED12A; engulfment and cell motility protein 2
SPTAN1	7696.744	0.262279	0.085480613	3.068289	0.0021529	0.018987	Not_Sig	no	gi|555993564|ref|XP_005908543.1|	12697	0	PREDICTED: spectrin alpha chain, non-erythrocytic 1 isoform X2 [Bos mutus]	gi|555993563|ref|XM_005908481.1|	7771	0	PREDICTED: Bos mutus spectrin, alpha, non-erythrocytic 1 (SPTAN1), transcript variant X2, mRNA	Q13813	4845	0	Spectrin alpha chain, non-erythrocytic 1	SPAC3A12.14	63.5	1.76e-10	COG5126	pfam08726	EFhand_Ca_insen	Ca2+ insensitive EF hand. EF hands are helix-loop-helix binding motifs involved in the regulation of many cellular processes. EF hands	GO:0051693|actin filament capping;GO:0007411|axon guidance;GO:0006888|ER to Golgi vesicle-mediated transport;GO:0000165|MAPK cascade;	GO:0005913|cell-cell adherens junction;GO:0005829|cytosol;GO:0070062|extracellular exosome;GO:1903561|extracellular	GO:0003779|actin binding;GO:0098641|cadherin binding involved in cell-cell	K06114	SPTA; spectrin alpha
WDR81	637.8647	0.374218	0.121964604	3.06825	0.0021532	0.018987	Not_Sig	no	gi|555952231|ref|XP_005888305.1|	9017	0	PREDICTED: WD repeat-containing protein 81 isoform X1 [Bos mutus]	gi|983001495|ref|XM_005195619.3|	7007	0	PREDICTED: Bos taurus WD repeat domain 81 (WDR81), mRNA	Q562E7	3125	0	WD repeat-containing protein 81	.	.	.	.	pfam02138	Beach	Beige/BEACH domain.	GO:0000132|establishment of mitotic spindle orientation;GO:0007281|germ cell development;GO:0031023|microtubule organizing center organization;GO:0010923|negative regulation of phosphatase activity;GO:0022008|neurogenesis;GO:0007097|nuclear migration;GO:2000574|regulation of microtubule motor activity;GO:0008090|retrograde axon cargo transport;GO:0047496|vesicle transport along microtubule;	GO:1904115|axon cytoplasm;GO:0005938|cell cortex;GO:0005813|centrosome;GO:0000776|kinetochore;GO:0005874|microtubule;G	GO:0045502|dynein binding;GO:0008017|microtubule binding;	K17601	WDR81; WD repeat-containing protein 81
UGDH	942.0402	-0.35783	0.116679864	-3.06674	0.0021641	0.019069	Not_Sig	no	gi|27806365|ref|NP_776636.1|	2652	0	UDP-glucose 6-dehydrogenase [Bos taurus]	gi|742091360|ref|XM_010833567.1|	3105	0	PREDICTED: Bison bison bison UDP-glucose 6-dehydrogenase (UGDH), transcript variant X1, mRNA	P12378	1026	0	UDP-glucose 6-dehydrogenase	SMc02641	287	2.06e-86	COG1004	pfam03721	UDPG_MGDP_dh_N	UDP-glucose/GDP-mannose dehydrogenase family, NAD binding domain. The UDP-glucose/GDP-mannose	GO:0005975|carbohydrate metabolic process;GO:0001702|gastrulation with mouth forming second;GO:0006024|glycosaminoglycan biosynthetic process;GO:0006065|UDP-glucuronate biosynthetic process;	GO:0005829|cytosol;GO:0070062|extracellular exosome;GO:0005654|nucleoplasm;GO:0005634|nucleus;	GO:0051287|NAD binding;GO:0003979|UDP-glucose 6-dehydrogenase	K00012	UGDH, ugd; UDPglucose 6-dehydrogenase [EC:1.1.1.22]
EIF3J	1080.597	-0.35955	0.117297069	-3.06534	0.0021743	0.019143	Not_Sig	no	gi|926703419|ref|XP_013822851.1|	578	6e-65	PREDICTED: eukaryotic translation initiation factor 3 subunit J [Capra hircus]	gi|742145444|ref|XM_010845190.1|	2470	0	PREDICTED: Bison bison bison eukaryotic translation initiation factor 3, subunit J (EIF3J), mRNA	Q0VCU8	226	1e-65	Eukaryotic translation initiation factor 3 subunit J	.	.	.	.	pfam08597	eIF3_subunit	Translation initiation factor eIF3 subunit. This is a family of proteins which are subunits of the eukaryotic translation initiation	GO:0002181|cytoplasmic translation;GO:0001731|formation of translation preinitiation complex;GO:0006446|regulation of translational initiation;	GO:0016282|eukaryotic 43S preinitiation complex;GO:0033290|eukaryotic 48S preinitiation complex;GO:0005852|eukaryotic translation	GO:0003743|translation initiation factor activity;	K03245	EIF3J; translation initiation factor 3 subunit J
ABCF2	1296.141	0.302165	0.098590961	3.064833	0.0021779	0.019161	Not_Sig	no	gi|961722582|ref|XP_014921558.1|	2788	0	PREDICTED: ATP-binding cassette sub-family F member 2 [Acinonyx jubatus]	gi|742191434|ref|XM_010857239.1|	2550	0	PREDICTED: Bison bison bison ATP-binding cassette, sub-family F (GCN20), member 2 (ABCF2), mRNA	Q2KJA2	1074	0	ATP-binding cassette sub-family F member 2	YER036c	497	7.80e-165	COG0488	pfam00005	ABC_tran	ABC transporter. ABC transporters for a large family of proteins responsible for translocation of a variety of compounds across	.	GO:0016020|membrane;GO:0005739|mitochondrion;	GO:0005524|ATP binding;GO:0016887|ATPase activity;	K06185	ABCF2; ATP-binding cassette, subfamily F, member 2
KCNH2	222.5118	0.581974	0.18995312	3.06378	0.0021856	0.019203	Not_Sig	no	gi|803262111|ref|XP_011990757.1|	5188	0	PREDICTED: potassium voltage-gated channel subfamily H member 2 isoform X2 [Ovis aries musimon]	gi|965840231|ref|XM_012135367.2|	3831	0	PREDICTED: Ovis aries musimon potassium voltage-gated channel subfamily H member 2 (LOC101106315), transcript variant X2, mRNA	Q12809	1942	0	Potassium voltage-gated channel subfamily H member 2	CC0285_1	72.8	3.42e-13	COG2202	pfam00520	Ion_trans	Ion transport protein. This family contains Sodium, Potassium, Calcium ion channels. This family is 6 transmembrane helices in which	GO:0061337|cardiac conduction;GO:0060048|cardiac muscle contraction;GO:0035690|cellular response to drug;GO:0086010|membrane depolarization during action potential;GO:0086011|membrane repolarization during action potential;GO:0086013|membrane repolarization during cardiac muscle cell action potential;GO:0098915|membrane repolarization during ventricular cardiac muscle cell action potential;GO:1902303|negative regulation of potassium ion export;GO:1901380|negative regulation of potassium ion transmembrane	GO:0009986|cell surface;GO:0048471|perinuclear region of cytoplasm;GO:0005886|plasma membrane;GO:0008076|voltage-gated	GO:0055131|C3HC4-type RING finger domain binding;GO:0005251	K04905	KCNH2; potassium voltage-gated channel Eag-related subfamily H member
ARPC5	4756.076	-0.30375	0.099143253	-3.06372	0.002186	0.019203	Not_Sig	no	gi|528753875|gb|EPY73534.1|	888	3e-112	actin-related protein 2/3 complex subunit 5 [Camelus ferus]	gi|402693243|ref|NM_001035447.2|	1837	0	Bos taurus actin related protein 2/3 complex subunit 5 (ARPC5), mRNA	Q9CPW4	304	4e-98	Actin-related protein 2/3 complex subunit 5	.	.	.	.	pfam04699	P16-Arc	ARP2/3 complex 16 kDa subunit (p16-Arc). The Arp2/3 protein complex has been implicated in the control of actin	GO:0034314|Arp2/3 complex-mediated actin nucleation;GO:0016477|cell migration;	GO:0005885|Arp2/3 protein complex;GO:0005737|cytoplasm;GO:0070062|extracellular exosome;GO:0005925|focal	GO:0005200|structural constituent of cytoskeleton;	K05754	ARPC5; actin related protein 2/3 complex, subunit 5
ZNF385A	246.3211	0.704725	0.230061022	3.063209	0.0021898	0.019221	UP	yes	gi|594080238|ref|XP_006063760.1|	1420	0	PREDICTED: zinc finger protein 385A isoform X2 [Bubalus bubalis]	gi|803290128|ref|XM_012149342.1|	1921	0	PREDICTED: Ovis aries musimon zinc finger protein 385A (ZNF385A), transcript variant X3, mRNA	Q96PM9	541	0	Zinc finger protein 385A	.	.	.	.	pfam12874	zf-met	Zinc-finger of C2H2 type. This is a zinc-finger domain with the CxxCx(12)Hx(6)H motif, found in multiple copies in a wide range of	GO:0006915|apoptotic process;GO:0006974|cellular response to DNA damage stimulus;GO:0006977|DNA damage response, signal transduction by p53 class mediator resulting in cell cycle arrest;GO:0007599|hemostasis;GO:0007611|learning or memory;GO:0007626|locomotory behavior;GO:0035855|megakaryocyte development;GO:0010609|mRNA localization resulting in posttranscriptional regulation of gene expression;GO:1902166|negative regulation of intrinsic apoptotic signaling pathway in response to DNA	GO:0005737|cytoplasm;GO:0030425|dendrite;GO:0043025|neuronal cell body;GO:0005730|nucleolus;GO:0005654|nucleoplasm;	GO:0003677|DNA binding;GO:0003730|mRNA 3'-UTR binding;GO:0002039	.	.
TMEM161A	346.3179	0.466185	0.152200355	3.062972	0.0021915	0.019222	Not_Sig	no	gi|148234763|ref|NP_001091613.1|	1910	0	transmembrane protein 161A precursor [Bos taurus]	gi|148234762|ref|NM_001098144.1|	2940	0	Bos taurus transmembrane protein 161A (TMEM161A), mRNA	Q9NX61	636	0	Transmembrane protein 161A	.	.	.	.	pfam10268	Tmemb_161AB	Predicted transmembrane protein 161AB. Transmemb_161AB is a family of conserved proteins found from worms to humans. Members	GO:0034599|cellular response to oxidative stress;GO:0034644|cellular response to UV;GO:1902230|negative regulation of intrinsic apoptotic signaling pathway in response to DNA damage;GO:0045739|positive regulation of DNA repair;GO:0032526|response to retinoic acid;	GO:0016021|integral component of membrane;	.	.	.
TMED5	502.4952	-0.44924	0.146762358	-3.06099	0.002206	0.019334	Not_Sig	no	gi|742204698|ref|XP_010858761.1|	1075	2e-134	PREDICTED: transmembrane emp24 domain-containing protein 5 isoform X1 [Bison bison bison]	gi|742204697|ref|XM_010860459.1|	3424	0	PREDICTED: Bison bison bison transmembrane emp24 protein transport domain containing 5 (TMED5), transcript variant X1, mRNA	Q2KJ84	393	2e-126	Transmembrane emp24 domain-containing protein 5	.	.	.	.	pfam01105	EMP24_GP25L	emp24/gp25L/p24 family/GOLD. Members of this family are implicated in bringing cargo forward from the ER and binding	GO:0090161|Golgi ribbon formation;GO:0015031|protein transport;	GO:0005801|cis-Golgi network;GO:0070971|endoplasmic reticulum exit site;GO:0005789|endoplasmic reticulum	.	K14825	ERP2; protein ERP2
SF3B2	1814.519	0.316224	0.103345007	3.059882	0.0022142	0.019391	Not_Sig	no	gi|594064978|ref|XP_006056489.1|	3273	0	PREDICTED: splicing factor 3B subunit 2 isoform X1 [Bubalus bubalis]	gi|742109385|ref|XM_010837765.1|	2856	0	PREDICTED: Bison bison bison splicing factor 3b, subunit 2, 145kDa (SF3B2), transcript variant X1, mRNA	Q13435	1217	0	Splicing factor 3B subunit 2	SPAC22F8.10c	307	2.10e-92	COG5182	pfam04037	DUF382	Domain of unknown function (DUF382). This domain is specific to the human splicing factor 3b subunit 2 and it's orthologues.	GO:0006397|mRNA processing;GO:0000398|mRNA splicing, via spliceosome;GO:0008380|RNA splicing;GO:0016032|viral process;	GO:0071013|catalytic step 2 spliceosome;GO:0005654|nucleoplasm;GO:0071011|precatalytic spliceosome;GO:0005681|spliceosomal	GO:0044822|poly(A) RNA binding;	K12829	SF3B2, SAP145, CUS1; splicing factor 3B subunit 2
NDFIP2	596.5515	-0.37164	0.121465933	-3.0596	0.0022163	0.019395	Not_Sig	no	gi|329664444|ref|NP_001192649.1|	1287	2e-161	NEDD4 family-interacting protein 2 [Bos taurus]	gi|742183285|ref|XM_010855107.1|	4235	0	PREDICTED: Bison bison bison Nedd4 family interacting protein 2 (NDFIP2), transcript variant X2, mRNA	Q9NV92	427	1e-135	NEDD4 family-interacting protein 2	.	.	.	.	pfam10176	DUF2370	Protein of unknown function (DUF2370). This family is conserved from fungi to humans. The human member is annotated	GO:0010629|negative regulation of gene expression;GO:0051224|negative regulation of protein transport;GO:0032410|negative regulation of transporter activity;GO:0043123|positive regulation of I-kappaB kinase/NF-kappaB signaling;GO:0031398|positive regulation of protein ubiquitination;	GO:0005737|cytoplasm;GO:0005783|endoplasmic reticulum;GO:0005794|Golgi apparatus;GO:0000139|Golgi membrane;GO:0016021|integral	GO:0004871|signal transducer activity;GO:0050699|WW domain	.	.
LMBRD1	808.0898	-0.37187	0.121550011	-3.05937	0.0022181	0.019395	Not_Sig	no	gi|742159372|ref|XP_010846805.1|	2078	0	PREDICTED: probable lysosomal cobalamin transporter [Bison bison bison]	gi|742159371|ref|XM_010848503.1|	2055	0	PREDICTED: Bison bison bison LMBR1 domain containing 1 (LMBRD1), mRNA	Q3SYY9	803	0	Probable lysosomal cobalamin transporter	.	.	.	.	pfam04791	LMBR1	LMBR1-like membrane protein. Members of this family are integral membrane proteins that are around 500 residues in length.	GO:0006810|transport;	GO:0016021|integral component of membrane;GO:0005765|lysosomal membrane;	GO:0031419|cobalamin binding;	K14617	LMBRD1; LMBR1 domain-containing protein 1
SNRPE	485.3349	-0.49117	0.160624478	-3.05789	0.002229	0.019477	Not_Sig	no	gi|560138833|emb|CDJ82682.1|	500	8e-72	RNA-directed DNA polymerase (reverse transcriptase) domain containing protein [Haemonchus contortus]	gi|850487286|gb|CP011897.1|	3885	0	Ovis canadensis canadensis isolate 43U chromosome 12 sequence	A1XQR9	163	4e-44	Small nuclear ribonucleoprotein E	SPBC11G11.06c	92	3.53e-21	COG1958	pfam01423	LSM	LSM domain. The LSM domain contains Sm proteins as well as other related LSM (Like Sm) proteins. The U1, U2, U4/U6, and	GO:0042633|hair cycle;GO:0000387|spliceosomal snRNP assembly;	GO:0005829|cytosol;GO:0034709|methylosome;GO:0005634|nucleus;GO:0034715|pICln-Sm protein complex;GO:0071011|precatalytic	GO:0003723|RNA binding;	K11097	SNRPE, SME; small nuclear ribonucleoprotein E
CTBP1	1236.688	0.354781	0.116066964	3.056693	0.0022379	0.019537	Not_Sig	no	gi|594087976|ref|XP_006067448.1|	2081	0	PREDICTED: C-terminal-binding protein 1 isoform X1 [Bubalus bubalis]	gi|982983774|ref|XM_015463879.1|	2215	0	PREDICTED: Bos taurus C-terminal binding protein 1 (CTBP1), transcript variant X2, mRNA	Q9Z2F5	795	0	C-terminal-binding protein 1	MJ1018	187	2.44e-50	COG0111	pfam00389	2-Hacid_dh	D-isomer specific 2-hydroxyacid dehydrogenase, catalytic domain. This family represents the largest portion of the catalytic domain of	GO:0061025|membrane fusion;GO:0000122|negative regulation of transcription from RNA polymerase II promoter;GO:0045892|negative regulation of transcription, DNA-templated;GO:0006351|transcription, DNA-templated;GO:0050872|white fat cell differentiation;	GO:0005737|cytoplasm;GO:0005634|nucleus;GO:0017053|transcriptional repressor complex;	GO:0051287|NAD binding;GO:0016616|oxidoreductase activity, acting on	K04496	CTBP; C-terminal binding protein
EFS	182.4981	0.583221	0.190812811	3.056506	0.0022393	0.019537	Not_Sig	no	gi|555996240|ref|XP_005909859.1|	2028	0	PREDICTED: embryonal Fyn-associated substrate isoform X1 [Bos mutus]	gi|965827399|ref|XM_012129255.2|	3498	0	PREDICTED: Ovis aries musimon embryonal Fyn-associated substrate (EFS), transcript variant X4, mRNA	O43281	657	0	Embryonal Fyn-associated substrate	.	.	.	.	pfam12026	DUF3513	Domain of unknown function (DUF3513). This presumed domain is functionally uncharacterized. This domain is found in	GO:0007155|cell adhesion;GO:0035556|intracellular signal transduction;	GO:0005737|cytoplasm;	GO:0019904|protein domain specific binding;	.	.
PLEKHM2	2417.824	0.366668	0.119976071	3.056173	0.0022418	0.019537	Not_Sig	no	gi|300793649|ref|NP_001179345.1|	5128	0	pleckstrin homology domain-containing family M member 2 [Bos taurus]	gi|942035605|ref|XM_005888920.2|	3877	0	PREDICTED: Bos mutus pleckstrin homology domain containing, family M (with RUN domain) member 2 (PLEKHM2), mRNA	Q8IWE5	1751	0	Pleckstrin homology domain-containing family M member 2	.	.	.	.	pfam02759	RUN	RUN domain. This domain is present in several proteins that are linked to the functions of GTPases in the Rap and Rab families. They	GO:0007030|Golgi organization;GO:0032418|lysosome localization;GO:1903527|positive regulation of membrane tubulation;GO:0032880|regulation of protein localization;	GO:0010008|endosome membrane;	GO:0019894|kinesin binding;	K15348	PLEKHM2, SKIP; pleckstrin homology domain-containing family M member 2
GHITM	2426.748	-0.36454	0.119285685	-3.05604	0.0022428	0.019537	Not_Sig	no	gi|742180329|ref|XP_010852670.1|	1594	0	PREDICTED: growth hormone-inducible transmembrane protein [Bison bison bison]	gi|942075334|ref|XM_014480403.1|	2465	0	PREDICTED: Bos mutus growth hormone inducible transmembrane protein (GHITM), mRNA	Q9H3K2	585	0	Growth hormone-inducible transmembrane protein	jhp0854	53.1	7.47e-07	COG0670	pfam12811	BaxI_1	Bax inhibitor 1 like. The Bax-inhibitor-1 region of the receptor molecules is conserved from bacteria to humans.	GO:0006915|apoptotic process;	GO:0070062|extracellular exosome;GO:0016021|integral component of membrane;GO:0005743|mitochondrial inner membrane;	.	.	.
ZNF326	921.6945	-0.34625	0.113319561	-3.05553	0.0022466	0.019555	Not_Sig	no	gi|926688151|ref|XP_005678177.2|	2255	0	PREDICTED: DBIRD complex subunit ZNF326 [Capra hircus]	gi|329664863|ref|NM_001206028.1|	2406	0	Bos taurus zinc finger protein 326 (ZNF326), mRNA	F1MJM0	871	0	DBIRD complex subunit ZNF326	.	.	.	.	pfam04988	AKAP95	A-kinase anchoring protein 95 (AKAP95). A-kinase (or PKA)-anchoring protein AKAP95 is implicated in mitotic chromosome	GO:0006397|mRNA processing;GO:0032784|regulation of DNA-templated transcription, elongation;GO:0043484|regulation of RNA splicing;GO:0008380|RNA splicing;GO:0006351|transcription, DNA-templated;	GO:0044609|DBIRD complex;GO:0016363|nuclear matrix;GO:0005654|nucleoplasm;GO:0005681|spliceosomal complex;	GO:0003677|DNA binding;GO:0046872|metal ion binding;GO:0044822	K13204	ZNF326; zinc finger protein 326
FAM3C	2564.396	-0.38917	0.127374592	-3.05531	0.0022483	0.019555	Not_Sig	no	gi|555964827|ref|XP_005894475.1|	1141	4e-145	PREDICTED: protein FAM3C [Bos mutus]	gi|942050950|ref|XM_005894413.2|	2946	0	PREDICTED: Bos mutus family with sequence similarity 3, member C (FAM3C), mRNA	A5PKI3	442	6e-146	Protein FAM3C	.	.	.	.	pfam01522	Polysacc_deac_1	Polysaccharide deacetylase. This domain is found in polysaccharide deacetylase. This family of polysaccharide deacetylases	GO:0007275|multicellular organismal development;	GO:0016023|cytoplasmic membrane-bounded vesicle;GO:0070062|extracellular exosome;GO:0005794|Golgi apparatus;	.	.	.
TSPAN4	1605.13	0.498824	0.163300849	3.054631	0.0022534	0.019585	Not_Sig	no	gi|440906570|gb|ELR56818.1|	1201	5e-158	Tetraspanin-4, partial [Bos mutus]	gi|982972272|ref|XM_005227311.3|	1581	0	PREDICTED: Bos taurus tetraspanin 4 (TSPAN4), transcript variant X1, mRNA	Q5RAP3	410	9e-138	Tetraspanin-4	.	.	.	.	pfam00335	Tetraspannin	Tetraspanin family.	.	GO:0016021|integral component of membrane;	.	K17294	TSPAN4; tetraspanin-4
NDST2	308.0788	0.439535	0.143912494	3.054185	0.0022567	0.019599	Not_Sig	no	gi|158186716|ref|NP_777202.2|	4343	0	bifunctional heparan sulfate N-deacetylase/N-sulfotransferase 2 [Bos taurus]	gi|158186715|ref|NM_174777.3|	3674	0	Bos taurus N-deacetylase/N-sulfotransferase (heparan glucosaminyl) 2 (NDST2), mRNA	P52849	1646	0	Bifunctional heparan sulfate N-deacetylase/N-sulfotransferase 2	.	.	.	.	pfam12062	HSNSD	heparan sulfate-N-deacetylase. This family of proteins is are heparan sulfate N-deacetylase enzymes. This protein is found in	GO:0006024|glycosaminoglycan biosynthetic process;GO:0015012|heparan sulfate proteoglycan biosynthetic process;GO:0030210|heparin biosynthetic process;	GO:0000139|Golgi membrane;GO:0016021|integral component of membrane;	GO:0015016|[heparan sulfate]-glucosamine N-sulfotransferase	K02577	NDST2; heparan sulfate N-deacetylase/N-sulfotransferase
TCF25	1173.455	0.334759	0.109625226	3.053669	0.0022606	0.019618	Not_Sig	no	gi|982951984|ref|XP_010812902.2|	3208	0	PREDICTED: transcription factor 25 isoform X1 [Bos taurus]	gi|942045475|ref|XM_005892386.2|	3205	0	PREDICTED: Bos mutus transcription factor 25 (basic helix-loop-helix) (TCF25), mRNA	Q8R3L2	1070	0	Transcription factor 25	.	.	.	.	pfam04910	Tcf25	Transcriptional repressor TCF25. Members of this family are transcriptional repressors. They may act by increasing histone	GO:0000122|negative regulation of transcription from RNA polymerase II promoter;GO:0006351|transcription, DNA-templated;	GO:0005634|nucleus;	GO:0003677|DNA binding;GO:0003700|transcription factor activity, sequence-	.	.
APRT	617.7269	-0.39582	0.129644996	-3.05313	0.0022647	0.019629	Not_Sig	no	gi|70778754|ref|NP_001020505.1|	914	8e-122	adenine phosphoribosyltransferase [Bos taurus]	gi|942045337|ref|XM_005892403.2|	804	0	PREDICTED: Bos mutus adenine phosphoribosyltransferase (APRT), mRNA	Q56JW4	356	2e-123	Adenine phosphoribosyltransferase	alr4582	154	7.21e-46	COG0503	pfam00156	Pribosyltran	Phosphoribosyl transferase domain. This family includes a range of diverse phosphoribosyl transferase enzymes. This family	GO:0006168|adenine salvage;GO:0044209|AMP salvage;GO:0007625|grooming behavior;	GO:0005737|cytoplasm;GO:0070062|extracellular exosome;GO:0005654|nucleoplasm;	GO:0002055|adenine binding;GO:0003999|adenine	K00759	APRT, apt; adenine phosphoribosyltransferase [EC:2.4.2.7]

<table>
<tr><td>SUPT6H</td><td>2059.932</td><td>0.339297</td><td>0.111134043</td><td>3.053047</td><td>0.0022653</td><td>0.019629</td><td>Not_Sig</td><td>no</td><td>gi|300797661|ref|NP_001180055.1|</td><td>7448</td><td>0</td><td>transcription elongation factor SPT6 [Bos taurus]</td><td>gi|742208627|ref|XM_010861413.1|</td><td>5918</td><td>0</td><td>PREDICTED: Bison bison bison suppressor of Ty 6 homolog (S. cerevisiae) (SUPT6H), transcript variant X1, mRNA</td><td>Q62383</td><td>2814</td><td>0</td><td>Transcription elongation factor SPT6</td><td>SPAC1F7.01c</td><td>377</td><td>2.04e-107</td><td>COG2183</td><td>pfam14633</td><td>SH2_2</td><td>SH2 domain.</td><td>GO:0006397|mRNA processing;GO:0051028|mRNA transport;GO:0061086|negative regulation of histone H3-K27 methylation;GO:0032968|positive regulation of transcription elongation from RNA polymerase II promoter;GO:0045191|regulation of isotype switching;GO:0010793|regulation of mRNA export from nucleus;GO:0050684|regulation of mRNA processing;GO:0051147|regulation of muscle cell differentiation;GO:0008380|RNA splicing;GO:0006351|transcription, DNA-templated;</td><td>GO:0005634|nucleus;</td><td>GO:0003677|DNA binding;GO:0042393|histone binding;GO:0044822</td><td>K11292</td><td>SUPT6H, SPT6; transcription elongation factor SPT6</td></tr>
<tr><td>ATP6V0D1</td><td>3430.935</td><td>0.37546</td><td>0.123003126</td><td>3.052443</td><td>0.0022699</td><td>0.019654</td><td>Not_Sig</td><td>no</td><td>gi|655790980|ref|XP_002711660.2|</td><td>1915</td><td>0</td><td>PREDICTED: V-type proton ATPase subunit d 1 [Oryctolagus cuniculus]</td><td>gi|742168568|ref|XM_010851037.1|</td><td>1577</td><td>0</td><td>PREDICTED: Bison bison bison ATPase, H+ transporting, lysosomal 38kDa, V0 subunit d1 (ATP6V0D1), mRNA</td><td>Q5R6I1</td><td>729</td><td>0</td><td>V-type proton ATPase subunit d 1</td><td>SPAC17A2.03c</td><td>390</td><td>1.78e-132</td><td>COG1527</td><td>pfam01992</td><td>vATP-synt_AC39</td><td>ATP synthase (C/AC39) subunit. This family includes the AC39 subunit from vacuolar ATP synthase, and the C subunit from</td><td>GO:0015991|ATP hydrolysis coupled proton transport;GO:0042384|cilium assembly;</td><td>GO:0033179|proton-transporting V-type ATPase, V0 domain;</td><td>GO:0015078|hydrogen ion transmembrane transporter activity;</td><td>K02146</td><td>ATPeV0D, ATP6D; V-type H+-transporting ATPase subunit d</td></tr>
<tr><td>DHCR24</td><td>6572.181</td><td>0.286664</td><td>0.093936499</td><td>3.051677</td><td>0.0022757</td><td>0.019689</td><td>Not_Sig</td><td>no</td><td>gi|555987734|ref|XP_005905714.1|</td><td>2764</td><td>0</td><td>PREDICTED: delta(24)-sterol reductase [Bos mutus]</td><td>gi|742154592|ref|XM_010847118.1|</td><td>4191</td><td>0</td><td>PREDICTED: Bison bison bison 24-dehydrocholesterol reductase (DHCR24), mRNA</td><td>Q8VCH6</td><td>1038</td><td>0</td><td>Delta(24)-sterol reductase</td><td>Cgl0556</td><td>132</td><td>1.82e-31</td><td>COG0277</td><td>pfam01565</td><td>FAD_binding_4</td><td>FAD binding domain. This family consists of various enzymes that use FAD as a co-factor, most of the enzymes are similar to oxygen</td><td>GO:0042987|amyloid precursor protein catabolic process;GO:0006695|cholesterol biosynthetic process;GO:0008203|cholesterol metabolic process;GO:0030539|male genitalia development;GO:0061024|membrane organization;GO:0043066|negative regulation of apoptotic process;GO:0008285|negative regulation of cell proliferation;GO:0043154|negative regulation of cysteine-type endopeptidase activity involved in apoptotic process;GO:0055114|oxidation-reduction process;GO:0031639|plasminogen activation;GO:0008104|protein</td><td>GO:0005856|cytoskeleton;GO:0005829|cytosol;GO:0005783|endoplasmic reticulum;GO:0005789|endoplasmic reticulum membrane;GO:0000139|Golgi</td><td>GO:0000246|delta24(24-1) sterol reductase activity;GO:0050614</td><td>K09828</td><td>DHCR24; delta24-sterol reductase [EC:1.3.1.72]</td></tr>
<tr><td>S100B</td><td>409.4979</td><td>-0.40128</td><td>0.131506845</td><td>-3.05144</td><td>0.0022775</td><td>0.01969</td><td>Not_Sig</td><td>no</td><td>gi|77736057|ref|NP_001029727.1|</td><td>376</td><td>4e-40</td><td>protein S100-B [Bos taurus]</td><td>gi|942074371|ref|XM_005902328.2|</td><td>1485</td><td>0</td><td>PREDICTED: Bos mutus S100 calcium binding protein B (S100B), mRNA</td><td>P02638</td><td>149</td><td>1e-41</td><td>Protein S100-B</td><td>.</td><td>.</td><td>.</td><td>.</td><td>pfam01023</td><td>S_100</td><td>S-100/ICaBP type calcium binding domain. The S-100 domain is a subfamily of the EF-hand calcium binding proteins.</td><td>GO:0048143|astrocyte activation;GO:0007409|axonogenesis;GO:0007611|learning or memory;GO:0007613|memory;GO:0071638|negative regulation of monocyte chemotactic protein-1 production;GO:0016310|phosphorylation;GO:0045917|positive regulation of complement activation;GO:0043123|positive regulation of I-kappaB kinase/NF-kappaB signaling;GO:0048168|regulation of neuronal synaptic plasticity;GO:0006417|regulation of translation;</td><td>GO:0005737|cytoplasm;GO:0043025|neuronal cell body;GO:0005634|nucleus;GO:0048471|perinuclear region of</td><td>GO:0005509|calcium ion binding;GO:0019210|kinase inhibitor</td><td>.</td><td>.</td></tr>
<tr><td>RHBDF2</td><td>317.8668</td><td>0.54144</td><td>0.177479647</td><td>3.050717</td><td>0.002283</td><td>0.019723</td><td>Not_Sig</td><td>no</td><td>gi|528921792|ref|XP_005195907.1|</td><td>4213</td><td>0</td><td>PREDICTED: inactive rhomboid protein 2 [Bos taurus]</td><td>gi|983002686|ref|XM_010824325.2|</td><td>3715</td><td>0</td><td>PREDICTED: Bos taurus rhomboid 5 homolog 2 (Drosophila) (RHBDF2), transcript variant X1, mRNA</td><td>Q00M95</td><td>1521</td><td>0</td><td>Inactive rhomboid protein 2</td><td>CAC0954</td><td>62.8</td><td>1.87e-09</td><td>COG0705</td><td>pfam12595</td><td>Rhomboid_SP</td><td>Rhomboid serine protease. This domain family is found in eukaryotes, and is approximately 210 amino acids in length. The</td><td>GO:0050709|negative regulation of protein secretion;GO:0015031|protein transport;GO:0006508|proteolysis;GO:0042058|regulation of epidermal growth factor receptor signaling pathway;GO:0050708|regulation of protein secretion;</td><td>GO:0005789|endoplasmic reticulum membrane;GO:0016021|integral component of membrane;GO:0005886|plasma membrane;</td><td>.</td><td>.</td><td>.</td></tr>
<tr><td>NCKIPSD</td><td>197.1648</td><td>0.526514</td><td>0.17261287</td><td>3.05026</td><td>0.0022864</td><td>0.019738</td><td>Not_Sig</td><td>no</td><td>gi|529001542|ref|XP_005222883.1|</td><td>3003</td><td>0</td><td>PREDICTED: NCK-interacting protein with SH3 domain isoform X1 [Bos taurus]</td><td>gi|982962019|ref|XM_005222826.3|</td><td>2512</td><td>0</td><td>PREDICTED: Bos taurus NCK interacting protein with SH3 domain (NCKIPSD), transcript variant X1, mRNA</td><td>Q9NZQ3</td><td>1083</td><td>0</td><td>NCK-interacting protein with SH3 domain</td><td>.</td><td>.</td><td>.</td><td>.</td><td>pfam09431</td><td>DUF2013</td><td>Protein of unknown function (DUF2013). This region is found at the C terminal of a group of cytoskeletal proteins.</td><td>GO:0007010|cytoskeleton organization;GO:0038096|Fc-gamma receptor signaling pathway involved in phagocytosis;GO:0006607|NLS-bearing protein import into nucleus;GO:0010976|positive regulation of neuron projection development;GO:0007165|signal transduction;</td><td>GO:0005829|cytosol;GO:0005882|intermediate filament;GO:0005634|nucleus;</td><td>GO:0008092|cytoskeletal protein binding;</td><td>.</td><td>.</td></tr>
<tr><td>ADIPOR1</td><td>1472.365</td><td>0.395271</td><td>0.129609485</td><td>3.049711</td><td>0.0022906</td><td>0.019759</td><td>Not_Sig</td><td>no</td><td>gi|440893791|gb|ELR46440.1|</td><td>1798</td><td>0</td><td>Adiponectin receptor protein 1, partial [Bos mutus]</td><td>gi|742233106|ref|XM_010831069.1|</td><td>2019</td><td>0</td><td>PREDICTED: Bison bison bison adiponectin receptor 1 (ADIPOR1), mRNA</td><td>Q96A54</td><td>664</td><td>0</td><td>Adiponectin receptor protein 1</td><td>SPBC12C2.09c</td><td>142</td><td>6.15e-37</td><td>COG1272</td><td>pfam03006</td><td>HlyIII</td><td>Haemolysin-III related. Members of this family are integral membrane proteins. This family includes a protein with hemolytic</td><td>GO:0033211|adiponectin-activated signaling pathway;GO:0019395|fatty acid oxidation;GO:0042593|glucose homeostasis;GO:0009755|hormone-mediated signaling pathway;GO:0033210|leptin-mediated signaling pathway;GO:0030308|negative regulation of cell growth;GO:0010633|negative regulation of epithelial cell migration;GO:0010719|negative regulation of epithelial to mesenchymal transition;GO:0046426|negative regulation of JAK-STAT cascade;GO:1901223|negative regulation of NIK/NF-kappaB</td><td>GO:0016021|integral component of membrane;GO:0031226|intrinsic component of plasma membrane;GO:0016020|membrane;GO:000</td><td>GO:0097003|adipokinetic hormone receptor activity;GO:0055100</td><td>K07297</td><td>ADIPOR; adiponectin receptor</td></tr>
<tr><td>LOC138983905</td><td>1523.343</td><td>0.305207</td><td>0.100101404</td><td>3.048976</td><td>0.0022962</td><td>0.01979</td><td>Not_Sig</td><td>no</td><td>gi|62460420|ref|NP_001014860.1|</td><td>2082</td><td>0</td><td>heterogeneous nuclear ribonucleoprotein F [Bos taurus]</td><td>gi|942063735|ref|XM_005898904.2|</td><td>1522</td><td>0</td><td>PREDICTED: Bos mutus heterogeneous nuclear ribonucleoprotein F (LOC102265903), transcript variant X9, mRNA</td><td>Q5E9J1</td><td>806</td><td>0</td><td>Heterogeneous nuclear ribonucleoprotein F</td><td>.</td><td>.</td><td>.</td><td>.</td><td>pfam08080</td><td>zf-RNPHF</td><td>RNPHF zinc finger. This domain is a putative zinc-binding domain (CHHC motif) in RNP H and F. The domain is often associated with</td><td>GO:0006397|mRNA processing;GO:0043484|regulation of RNA splicing;GO:0008380|RNA splicing;</td><td>GO:0005654|nucleoplasm;GO:0005681|spliceosomal complex;</td><td>GO:0000166|nucleotide binding;GO:0003727|single-stranded</td><td>K12898</td><td>HNRNPF_H; heterogeneous nuclear ribonucleoprotein</td></tr>
<tr><td>DLG4</td><td>193.0966</td><td>0.59588</td><td>0.195447805</td><td>3.048793</td><td>0.0022976</td><td>0.01979</td><td>UP</td><td>yes</td><td>gi|982956470|ref|XP_015314086.1|</td><td>4039</td><td>0</td><td>PREDICTED: disks large homolog 4 isoform X6 [Bos taurus]</td><td>gi|982956469|ref|XM_015458600.1|</td><td>3102</td><td>0</td><td>PREDICTED: Bos taurus discs large homolog 4 (DLG4), transcript variant X6, mRNA</td><td>P78352</td><td>1476</td><td>0</td><td>Disks large homolog 4</td><td>YDR454c</td><td>103</td><td>2.36e-24</td><td>COG0194</td><td>pfam00625</td><td>Guanylate_kin</td><td>Guanylate kinase.</td><td>GO:0097113|AMPA glutamate receptor clustering;GO:0007268|synaptic transmission;GO:0060997|dendritic spine morphogenesis;GO:0045184|establishment of protein localization;GO:0045197|establishment or maintenance of epithelial cell apical/basal polarity;GO:0007612|learning;GO:0035641|locomotory exploration behavior;GO:0000165|MAPK cascade;GO:0002091|negative regulation of receptor internalization;GO:0007399|nervous system development;GO:0050885|neuromuscular process controlling</td><td>GO:0032281|AMPA glutamate receptor complex;GO:0016323|basolateral plasma membrane;GO:0030054|cell junction;GO:0044300|cerebellar mossy</td><td>GO:0033130|acetylcholine receptor binding;GO:0031697|beta-1 adrenergic</td><td>K11828</td><td>DLG4, PSD95; discs large protein 4</td></tr>
<tr><td>SMG9</td><td>491.7423</td><td>0.452388</td><td>0.148415741</td><td>3.048111</td><td>0.0023028</td><td>0.01982</td><td>Not_Sig</td><td>no</td><td>gi|594048362|ref|XP_006048579.1|</td><td>2268</td><td>0</td><td>PREDICTED: protein SMG9 isoform X1 [Bubalus bubalis]</td><td>gi|594048361|ref|XM_006048517.1|</td><td>2167</td><td>0</td><td>PREDICTED: Bubalus bubalis SMG9 nonsense mediated mRNA decay factor (SMG9), transcript variant X1, mRNA</td><td>Q2YDD2</td><td>877</td><td>0</td><td>Protein SMG9</td><td>.</td><td>.</td><td>.</td><td>.</td><td>pfam10220</td><td>DUF2146</td><td>Uncharacterized conserved protein (DUF2146). This is a family of proteins conserved from plants to humans. In Dictyostelium it is</td><td>GO:0000184|nuclear-transcribed mRNA catabolic process, nonsense-mediated decay;</td><td>.</td><td>.</td><td>K18735</td><td>SMG9; protein SMG9</td></tr>
<tr><td>DUSP3</td><td>128.6565</td><td>0.570209</td><td>0.187107258</td><td>3.0475</td><td>0.0023075</td><td>0.019845</td><td>Not_Sig</td><td>no</td><td>gi|528996591|ref|XP_005220998.1|</td><td>1029</td><td>5e-138</td><td>PREDICTED: dual specificity protein phosphatase 3 isoform X2 [Bos taurus]</td><td>gi|965890157|ref|XM_012157361.2|</td><td>930</td><td>0</td><td>PREDICTED: Ovis aries musimon dual specificity phosphatase 3 (DUSP3), transcript variant X2, mRNA</td><td>Q5RD73</td><td>377</td><td>1e-130</td><td>Dual specificity protein phosphatase 3</td><td>SPBC17A3.06</td><td>77.8</td><td>2.24e-15</td><td>COG2453</td><td>pfam00782</td><td>DSPc</td><td>Dual specificity phosphatase, catalytic domain. Ser/Thr and Tyr protein phosphatases. The enzyme's tertiary fold is highly</td><td>GO:0016311|dephosphorylation;GO:0070373|negative regulation of ERK1 and ERK2 cascade;GO:0046329|negative regulation of JNK cascade;GO:0050868|negative regulation of T cell activation;GO:0050860|negative regulation of T cell receptor signaling pathway;GO:0045931|positive regulation of mitotic cell cycle;</td><td>GO:0005829|cytosol;GO:0070062|extracellular exosome;GO:0001772|immunological synapse;GO:0005654|nucleoplasm;</td><td>GO:0033549|MAP kinase phosphatase activity;GO:0016791|phosphatase</td><td>K17614</td><td>DUSP3, VHR; dual specificity phosphatase 3 [EC:3.1.3.16</td></tr>
<tr><td>TIMM8B</td><td>274.3819</td><td>-0.42794</td><td>0.140432349</td><td>-3.04729</td><td>0.0023092</td><td>0.019845</td><td>Not_Sig</td><td>no</td><td>gi|548494389|ref|XP_005689505.1|</td><td>498</td><td>7e-61</td><td>PREDICTED: mitochondrial import inner membrane translocase subunit Tim8 B [Capra hircus]</td><td>gi|942071769|ref|XM_005901527.2|</td><td>553</td><td>0</td><td>PREDICTED: Bos mutus translocase of inner mitochondrial membrane 8 homolog B (yeast) (TIMM8B), mRNA</td><td>Q3SZ93</td><td>173</td><td>4e-54</td><td>Mitochondrial import inner membrane translocase subunit Tim8 B</td><td>.</td><td>.</td><td>.</td><td>.</td><td>pfam02953</td><td>zf-Tim10_DDP</td><td>Tim10/DDP family zinc finger. Putative zinc binding domain with four conserved cysteine residues.</td><td>GO:0015031|protein transport;</td><td>GO:0005743|mitochondrial inner membrane;</td><td>GO:0046872|metal ion binding;</td><td>K17780</td><td>TIM8; mitochondrial import inner membrane translocase subunit</td></tr>
<tr><td>CKAP2L</td><td>459.5571</td><td>0.380268</td><td>0.124807813</td><td>3.046826</td><td>0.0023127</td><td>0.01986</td><td>Not_Sig</td><td>no</td><td>gi|742093594|ref|XP_010832417.1|</td><td>3818</td><td>0</td><td>PREDICTED: cytoskeleton-associated protein 2-like isoform X2 [Bison bison bison]</td><td>gi|555955784|ref|XM_005889986.1|</td><td>3057</td><td>0</td><td>PREDICTED: Bos mutus cytoskeleton associated protein 2-like (CKAP2L), mRNA</td><td>A5PK21</td><td>1446</td><td>0</td><td>Cytoskeleton-associated protein 2-like</td><td>.</td><td>.</td><td>.</td><td>.</td><td>pfam15297</td><td>CKAP2_C</td><td>This domain is found in the human Cytoskeleton-associated protein 2 C-terminus. This family includes the C-terminus of CKAP2 and CKAP2L. CKAP2 is a microtubule</td><td>.</td><td>GO:0005813|centrosome;GO:0005737|cytoplasm;GO:0000922|spindle pole;</td><td>.</td><td>K16769</td><td>CKAP2; cytoskeleton-associated protein 2</td></tr>
<tr><td>AASDHPPT</td><td>783.1047</td><td>-0.42281</td><td>0.138790042</td><td>-3.04637</td><td>0.0023162</td><td>0.019876</td><td>Not_Sig</td><td>no</td><td>gi|330417903|ref|NP_001193406.1|</td><td>1569</td><td>0</td><td>L-aminoadipate-semialdehyde dehydrogenase-phosphopantetheinyl transferase precursor [Bos taurus]</td><td>gi|330417902|ref|NM_001206477.1|</td><td>3250</td><td>0</td><td>Bos taurus aminoadipate-semialdehyde dehydrogenase-phosphopantetheinyl transferase (AASDHPPT), mRNA</td><td>Q9NRN7</td><td>573</td><td>0</td><td>L-aminoadipate-semialdehyde dehydrogenase-phosphopantetheinyl</td><td>BS_sfpm</td><td>86.7</td><td>4.04e-18</td><td>COG2091</td><td>pfam01648</td><td>ACPS</td><td>4'-phosphopantetheinyl transferase superfamily. Members of this family transfers the 4'-phosphopantetheine (4'-PP)</td><td>GO:0019878|lysine biosynthetic process via aminoadipic acid;GO:0015939|pantothenate metabolic process;</td><td>GO:0005829|cytosol;GO:0070062|extracellular exosome;</td><td>GO:0008897|holo-[acyl-carrier-protein] synthase activity;GO:0000287</td><td>K06133</td><td>LYS5, acpT; 4'-phosphopantetheinyl transferase [EC:2.7.8.-]</td></tr>
<tr><td>CDC34</td><td>592.9725</td><td>0.462273</td><td>0.151775133</td><td>3.045775</td><td>0.0023208</td><td>0.019887</td><td>Not_Sig</td><td>no</td><td>gi|594057222|ref|XP_006052791.1|</td><td>1295</td><td>1e-175</td><td>PREDICTED: ubiquitin-conjugating enzyme E2 R1 isoform X1 [Bubalus bubalis]</td><td>gi|741933475|ref|XM_005209318.2|</td><td>1209</td><td>0</td><td>PREDICTED: Bos taurus cell division cycle 34 (CDC34), transcript variant X1, mRNA</td><td>Q8CFI2</td><td>397</td><td>5e-136</td><td>Ubiquitin-conjugating enzyme E2 R1</td><td>SPBC1105.09</td><td>157</td><td>1.09e-45</td><td>COG5078</td><td>pfam00179</td><td>UQ_con</td><td>Ubiquitin-conjugating enzyme. Proteins destined for proteasome-mediated degradation may be ubiquitinated. Ubiquitination</td><td>GO:0007049|cell cycle;GO:0035458|cellular response to interferon-beta;GO:0043951|negative regulation of cAMP-mediated signaling;GO:0043161|proteasome-mediated ubiquitin-dependent protein catabolic process;GO:0070936|protein K48-linked ubiquitination;GO:0006513|protein monoubiquitination;GO:0000209|protein polyubiquitination;GO:0016567|protein ubiquitination;</td><td>GO:0005737|cytoplasm;GO:0005654|nucleoplasm;</td><td>GO:0005524|ATP binding;GO:0061631|ubiquitin conjugating enzyme</td><td>K02207</td><td>UBE2R, UBC3, CDC34; ubiquitin-conjugating enzyme E2 R [EC:2.3.2.23]</td></tr>
<tr><td>LMTK2</td><td>1445.455</td><td>0.322988</td><td>0.106045539</td><td>3.045746</td><td>0.002321</td><td>0.019887</td><td>Not_Sig</td><td>no</td><td>gi|440899915|gb|ELR51157.1|</td><td>6572</td><td>0</td><td>Serine/threonine-protein kinase LMTK2, partial [Bos mutus]</td><td>gi|983007451|ref|XM_015469435.1|</td><td>7821</td><td>0</td><td>PREDICTED: Bos taurus lemur tyrosine kinase 2 (LMTK2), transcript variant X1, mRNA</td><td>Q8IWU2</td><td>1854</td><td>0</td><td>Serine/threonine-protein kinase LMTK2</td><td>.</td><td>.</td><td>.</td><td>.</td><td>pfam07714</td><td>Pkinase_Tyr</td><td>Protein tyrosine kinase.</td><td>GO:0045022|early endosome to late endosome transport;GO:0032456|endocytic recycling;GO:0018105|peptidyl-serine phosphorylation;GO:0018107|peptidyl-threonine phosphorylation;GO:0046777|protein autophosphorylation;GO:0006468|protein phosphorylation;GO:0001881|receptor recycling;GO:0033572|transferrin transport;</td><td>GO:0005829|cytosol;GO:0005769|early endosome;GO:0005794|Golgi apparatus;GO:0016021|integral component of membrane;GO:0048471|perinuclear</td><td>GO:0005524|ATP binding;GO:0070853|myosin VI binding;GO:0004864</td><td>K08898</td><td>LMTK2; lemur tyrosine kinase 2 [EC:2.7.10.1]</td></tr>
<tr><td>ARF4</td><td>4037.967</td><td>-0.34059</td><td>0.111834293</td><td>-3.04547</td><td>0.0023232</td><td>0.019891</td><td>Not_Sig</td><td>no</td><td>gi|431899854|gb|ELK07801.1|</td><td>1049</td><td>1e-136</td><td>ADP-ribosylation factor 4 [Pteropus alecto]</td><td>gi|742170723|ref|XM_010851632.1|</td><td>1629</td><td>0</td><td>PREDICTED: Bison bison bison ADP-ribosylation factor 4 (ARF4), mRNA</td><td>Q3SZF2</td><td>372</td><td>4e-125</td><td>ADP-ribosylation factor 4</td><td>.</td><td>.</td><td>.</td><td>.</td><td>pfam00025</td><td>Arf</td><td>ADP-ribosylation factor family. Pfam combines a number of different Prosite families together</td><td>GO:0031584|activation of phospholipase D activity;GO:0045176|apical protein localization;GO:0016477|cell migration;GO:0060996|dendritic spine development;GO:0045197|establishment or maintenance of epithelial cell apical/basal polarity;GO:0007612|learning;GO:0043066|negative regulation of apoptotic process;GO:0045944|positive regulation of transcription from RNA polymerase II promoter;GO:0006471|protein ADP-ribosylation;GO:0061512|protein localization to cilium;GO:0015031|protein</td><td>GO:0005829|cytosol;GO:0043197|dendritic spine;GO:0070062|extracellular exosome;GO:0005794|Golgi apparatus;GO:0032587|ruffle membrane;</td><td>GO:0005525|GTP binding;</td><td>K07939</td><td>ARF4; ADP-ribosylation factor 4</td></tr>
<tr><td>TMEM129</td><td>356.8886</td><td>0.486202</td><td>0.159753884</td><td>3.043444</td><td>0.0023389</td><td>0.019995</td><td>Not_Sig</td><td>no</td><td>gi|742077315|ref|XP_010831599.1|</td><td>2223</td><td>0</td><td>PREDICTED: E3 ubiquitin-protein ligase TM129 [Bison bison bison]</td><td>gi|942056002|ref|XM_005896296.2|</td><td>2419</td><td>0</td><td>PREDICTED: Bos mutus transmembrane protein 129, E3 ubiquitin protein ligase (TMEM129), mRNA</td><td>Q08DK0</td><td>738</td><td>0</td><td>E3 ubiquitin-protein ligase TM129</td><td>.</td><td>.</td><td>.</td><td>.</td><td>pfam10272</td><td>Tmpp129</td><td>Putative transmembrane protein precursor. This is a family of proteins conserved from worms to humans. The proteins are</td><td>GO:0016567|protein ubiquitination;GO:0006986|response to unfolded protein;</td><td>GO:0005789|endoplasmic reticulum membrane;GO:0016021|integral component of membrane;</td><td>GO:0016874|ligase activity;GO:0046872|metal ion binding;</td><td>.</td><td>.</td></tr>
<tr><td>ITFG1</td><td>1045.336</td><td>-0.31499</td><td>0.103505067</td><td>-3.04327</td><td>0.0023402</td><td>0.019995</td><td>Not_Sig</td><td>no</td><td>gi|84370089|ref|NP_001033621.1|</td><td>3087</td><td>0</td><td>T-cell immunomodulatory protein precursor [Bos taurus]</td><td>gi|742140133|ref|XM_010843982.1|</td><td>3133</td><td>0</td><td>PREDICTED: Bison bison bison integrin alpha FG-GAP repeat containing 1 (ITFG1), mRNA</td><td>Q8TB96</td><td>1122</td><td>0</td><td>T-cell immunomodulatory protein</td><td>.</td><td>.</td><td>.</td><td>.</td><td>pfam13517</td><td>VCBS</td><td>Repeat domain in Vibrio, Colwellia, Bradyrhizobium and Shewanella. This domain of about 100 residues is found in multiple (up to 35)</td><td>.</td><td>GO:0070062|extracellular exosome;GO:0016021|integral component of membrane;</td><td>.</td><td>K17257</td><td>ITFG1; integrin alpha FG-GAP repeat containing protein 1</td></tr>
<tr><td>FOXP4</td><td>1653.77</td><td>0.497164</td><td>0.16336731</td><td>3.043228</td><td>0.0023405</td><td>0.019995</td><td>Not_Sig</td><td>no</td><td>gi|329664926|ref|NP_001192453.1|</td><td>2450</td><td>0</td><td>forkhead box protein P4 [Bos taurus]</td><td>gi|555968947|ref|XM_005896441.1|</td><td>3782</td><td>0</td><td>PREDICTED: Bos mutus forkhead box P4 (FOXP4), transcript variant X1, mRNA</td><td>Q8IVH2</td><td>893</td><td>0</td><td>Forkhead box protein P4</td><td>SPBC4C3.05</td><td>85.1</td><td>7.12e-16</td><td>COG5025</td><td>pfam00250</td><td>Fork_head</td><td>Fork head domain.</td><td>GO:0006351|transcription, DNA-templated;</td><td>GO:0005634|nucleus;</td><td>GO:0046872|metal ion binding;GO:0000981|RNA polymerase II</td><td>K09409</td><td>FOXP; forkhead box protein P</td></tr>
<tr><td>SMIM30</td><td>284.2568</td><td>-0.57032</td><td>0.187418732</td><td>-3.043</td><td>0.0023423</td><td>0.019995</td><td>Not_Sig</td><td>no</td><td>gi|51095128|gb|EAL24371.1|</td><td>220</td><td>3e-18</td><td>hypothetical gene supported by BX537645 [Homo sapiens]</td><td>gi|555950592|ref|XR_314739.1|</td><td>994</td><td>0</td><td>PREDICTED: Bos mutus uncharacterized LOC102273470 (LOC102273470), ncRNA</td><td>A4D0T7</td><td>67</td><td>9e-13</td><td>Putative transmembrane protein LINC00998</td><td>.</td><td>.</td><td>.</td><td>.</td><td>pfam05470</td><td>eIF-3c_N</td><td>Eukaryotic translation initiation factor 3 subunit 8 N-terminus. The largest of the mammalian translation initiation factors, eIF3,</td><td>.</td><td>GO:0016021|integral component of membrane;</td><td>.</td><td>.</td><td>.</td></tr>
<tr><td>INSIG2</td><td>472.768</td><td>-0.41583</td><td>0.136664609</td><td>-3.04272</td><td>0.0023445</td><td>0.019999</td><td>Not_Sig</td><td>no</td><td>gi|301775039|ref|XP_002922939.1|</td><td>1192</td><td>1e-154</td><td>PREDICTED: insulin-induced gene 2 protein isoform X2 [Ailuropoda melanoleuca]</td><td>gi|982977492|ref|XM_003581843.4|</td><td>2534</td><td>0</td><td>PREDICTED: Bos taurus insulin induced gene 2 (INSIG2), mRNA</td><td>Q9Y5U4</td><td>457</td><td>5e-154</td><td>Insulin-induced gene 2 protein</td><td>.</td><td>.</td><td>.</td><td>.</td><td>pfam07281</td><td>INSIG</td><td>Insulin-induced protein (INSIG). This family contains a number of eukaryotic Insulin-induced proteins (INSIG-1 and INSIG-2)</td><td>GO:0006695|cholesterol biosynthetic process;GO:0060363|cranial suture morphogenesis;GO:0042472|inner ear morphogenesis;GO:0042474|middle ear morphogenesis;GO:0045717|negative regulation of fatty acid biosynthetic process;GO:0010894|negative regulation of steroid biosynthetic process;GO:0060021|palate development;GO:0070542|response to fatty acid;GO:0032868|response to insulin;GO:0032933|SREBP signaling pathway;GO:0006641|triglyceride metabolic process;</td><td>GO:0005789|endoplasmic reticulum membrane;GO:0032937|SREBP-SCAP-Insig complex;</td><td>.</td><td>.</td><td>.</td></tr>
<tr><td>PDCD5</td><td>373.2238</td><td>-0.4765</td><td>0.156664421</td><td>-3.04151</td><td>0.0023539</td><td>0.020043</td><td>Not_Sig</td><td>no</td><td>gi|562880871|ref|XP_006168053.1|</td><td>659</td><td>9e-84</td><td>PREDICTED: programmed cell death protein 5 isoform X2 [Tupaia chinensis]</td><td>gi|942059627|ref|XM_005897753.2|</td><td>588</td><td>0</td><td>PREDICTED: Bos mutus programmed cell death 5 (PDCD5), transcript variant X2, mRNA</td><td>Q2HJH9</td><td>253</td><td>9e-84</td><td>Programmed cell death protein 5</td><td>SPAC23C4.09c</td><td>96.3</td><td>2.44e-24</td><td>COG2118</td><td>pfam01984</td><td>dsDNA_bind</td><td>Double-stranded DNA-binding domain. This domain is believed to bind double-stranded DNA of 20 bases length.</td><td>GO:0006915|apoptotic process;GO:0071560|cellular response to transforming growth factor beta stimulus;GO:0008285|negative regulation of cell proliferation;GO:1903645|negative regulation of chaperone-mediated protein folding;GO:0043280|positive regulation of cysteine-type endopeptidase activity involved in apoptotic process;GO:0010628|positive regulation of gene expression;GO:1903638|positive regulation of protein import into mitochondrial outer membrane;GO:0090200|positive regulation of release of cytochrome c from</td><td>GO:0005829|cytosol;GO:0070062|extracellular exosome;GO:0005634|nucleus;</td><td>GO:0010698|acetyltransferase activator activity;GO:0003677|DNA</td><td>K06875</td><td>PDCD5, TFAR19; programmed cell death protein 5</td></tr>
<tr><td>MMP28</td><td>46.44765</td><td>0.919878</td><td>0.30245109</td><td>3.041412</td><td>0.0023547</td><td>0.020043</td><td>UP</td><td>yes</td><td>gi|594064526|ref|XP_006056277.1|</td><td>2423</td><td>0</td><td>PREDICTED: matrix metalloproteinase-28 isoform X1 [Bubalus bubalis]</td><td>gi|983001319|ref|XM_015467950.1|</td><td>2377</td><td>0</td><td>PREDICTED: Bos taurus matrix metallopeptidase 28 (MMP28), transcript variant X2, mRNA</td><td>Q9H239</td><td>835</td><td>0</td><td>Matrix metalloproteinase-28</td><td>.</td><td>.</td><td>.</td><td>.</td><td>pfam00413</td><td>Peptidase_M10</td><td>Matrixin. The members of this family are enzymes that cleave peptides. These proteases require zinc for catalysis.</td><td>GO:0010760|negative regulation of macrophage chemotaxis;</td><td>GO:0005737|cytoplasm;GO:0005578|proteinaceous extracellular matrix;</td><td>GO:0005509|calcium ion binding;GO:0004222|metalloendopeptid</td><td>K08006</td><td>MMP28; matrix metalloproteinase-28 (epilysin) [EC:3.4.24.-]</td></tr>
<tr><td>SH3D21</td><td>79.67626</td><td>0.690917</td><td>0.227205592</td><td>3.040933</td><td>0.0023585</td><td>0.020043</td><td>UP</td><td>yes</td><td>gi|296488852|tpg|DAA30965.1|</td><td>2741</td><td>0</td><td>TPA: SH3 domain-containing protein C1orf113 homolog [Bos taurus]</td><td>gi|242117964|ref|NM_001162534.1|</td><td>2163</td><td>0</td><td>Bos taurus SH3 domain containing 21 (SH3D21), mRNA</td><td>A4FU49</td><td>326</td><td>3e-99</td><td>SH3 domain-containing protein 21</td><td>.</td><td>.</td><td>.</td><td>.</td><td>pfam14604</td><td>SH3_9</td><td>Variant SH3 domain.</td><td>.</td><td>GO:0070062|extracellular exosome;</td><td>.</td><td>.</td><td>.</td></tr>
<tr><td>LRFN3</td><td>91.59357</td><td>0.761064</td><td>0.250280913</td><td>3.040838</td><td>0.0023592</td><td>0.020043</td><td>UP</td><td>yes</td><td>gi|594090871|ref|XP_006068832.1|</td><td>2841</td><td>0</td><td>PREDICTED: leucine-rich repeat and fibronectin type-III domain-containing protein 3 isoform X1 [Bubalus bubalis]</td><td>gi|594090870|ref|XM_006068770.1|</td><td>3947</td><td>0</td><td>PREDICTED: Bubalus bubalis leucine rich repeat and fibronectin type III domain containing 3 (LRFN3), transcript variant X1, mRNA</td><td>Q1RMS4</td><td>1054</td><td>0</td><td>Leucine-rich repeat and fibronectin type-III domain-containing protein 3</td><td>alr0124_1</td><td>53.5</td><td>4.52e-06</td><td>COG4886</td><td>pfam13855</td><td>LRR_8</td><td>Leucine rich repeat.</td><td>GO:0007409|axonogenesis;GO:0007155|cell adhesion;</td><td>GO:0030424|axon;GO:0030054|cell junction;GO:0009986|cell surface;GO:0030425|dendrite;GO:0016021|integral component of</td><td>.</td><td>K16356</td><td>LRFN3, SALM4; leucine-rich repeat and fibronectin type-III domain-containing</td></tr>
<tr><td>TSKU</td><td>797.996</td><td>0.512084</td><td>0.168409205</td><td>3.040713</td><td>0.0023602</td><td>0.020043</td><td>Not_Sig</td><td>no</td><td>gi|555995124|ref|XP_005909307.1|</td><td>1762</td><td>0</td><td>PREDICTED: tsukushin [Bos mutus]</td><td>gi|982946510|ref|XM_010812724.2|</td><td>3086</td><td>0</td><td>PREDICTED: Bos taurus tsukushi, small leucine rich proteoglycan (TSKU), transcript variant X3, mRNA</td><td>Q8WUA8</td><td>580</td><td>0</td><td>Tsukushin</td><td>alr0124_1</td><td>64.3</td><td>8.83e-10</td><td>COG4886</td><td>pfam13855</td><td>LRR_8</td><td>Leucine rich repeat.</td><td>GO:0021960|anterior commissure morphogenesis;GO:0061073|ciliary body morphogenesis;GO:0021540|corpus callosum morphogenesis;GO:0021670|lateral ventricle development;GO:0030178|negative regulation of Wnt signaling pathway;GO:0010468|regulation of gene expression;</td><td>GO:0005615|extracellular space;</td><td>.</td><td>K08130</td><td>TSK, LRRC54; tsukushi (leucine-rich repeat-containing protein 54)</td></tr>
<tr><td>PACS2</td><td>1356.975</td><td>0.403999</td><td>0.132863274</td><td>3.040711</td><td>0.0023602</td><td>0.020043</td><td>Not_Sig</td><td>no</td><td>gi|955525752|ref|XP_014646077.1|</td><td>3851</td><td>0</td><td>PREDICTED: phosphofurin acidic cluster sorting protein 2 isoform X1 [Ceratotherium simum simum]</td><td>gi|983003940|ref|XM_015468569.1|</td><td>5584</td><td>0</td><td>PREDICTED: Bos taurus phosphofurin acidic cluster sorting protein 2 (PACS2), transcript variant X2, mRNA</td><td>Q86VP3</td><td>1417</td><td>0</td><td>Phosphofurin acidic cluster sorting protein 2</td><td>.</td><td>.</td><td>.</td><td>.</td><td>pfam10254</td><td>Pacs-1</td><td>PACS-1 cytosolic sorting protein. PACS-1 is a cytosolic sorting protein that directs the localisation of membrane proteins in the trans-</td><td>GO:0006915|apoptotic process;GO:0000045|autophagosome assembly;GO:0034497|protein localization to pre-autophagosomal structure;GO:0072661|protein targeting to plasma membrane;GO:0016032|viral process;</td><td>GO:0005783|endoplasmic reticulum;GO:0005788|endoplasmic reticulum lumen;GO:0005739|mitochondrion;</td><td>GO:0044325|ion channel binding;</td><td>.</td><td>.</td></tr>
<tr><td>EXOC5</td><td>1322.755</td><td>-0.43239</td><td>0.142289346</td><td>-3.03879</td><td>0.0023753</td><td>0.020147</td><td>Not_Sig</td><td>no</td><td>gi|300794420|ref|NP_001179640.1|</td><td>3667</td><td>0</td><td>exocyst complex component 5 [Bos taurus]</td><td>gi|742156617|ref|XM_010847691.1|</td><td>5487</td><td>0</td><td>PREDICTED: Bison bison bison exocyst complex component 5 (EXOC5), mRNA</td><td>O00471</td><td>1389</td><td>0</td><td>Exocyst complex component 5</td><td>.</td><td>.</td><td>.</td><td>.</td><td>pfam07393</td><td>Sec10</td><td>Exocyst complex component Sec10. This family contains the Sec10 component (approximately 650 residues long) of the</td><td>GO:0042384|cilium assembly;GO:0006887|exocytosis;GO:0006892|post-Golgi vesicle-mediated transport;GO:0015031|protein transport;GO:0048278|vesicle docking;</td><td>GO:0005737|cytoplasm;GO:0005829|cytosol;GO:0000145|exocyst;</td><td>GO:0017160|Ral GTPase binding;</td><td>.</td><td>.</td></tr>
<tr><td>DET1</td><td>227.1368</td><td>0.483235</td><td>0.159026521</td><td>3.038709</td><td>0.0023759</td><td>0.020147</td><td>Not_Sig</td><td>no</td><td>gi|410960574|ref|XP_003986864.1|</td><td>2997</td><td>0</td><td>PREDICTED: DET1 homolog isoform X2 [Felis catus]</td><td>gi|742081748|ref|XM_010856715.1|</td><td>2254</td><td>0</td><td>PREDICTED: Bison bison bison de-etiolated homolog 1 (Arabidopsis) (DET1), mRNA</td><td>Q7L5Y6</td><td>1140</td><td>0</td><td>DET1 homolog</td><td>.</td><td>.</td><td>.</td><td>.</td><td>pfam09737</td><td>Det1</td><td>De-etiolated protein 1 Det1. This is the C-terminal conserved 400 residues of Det1 proteins of approximately 550 amino acids.</td><td>.</td><td>GO:0005634|nucleus;</td><td>.</td><td>K10571</td><td>DET1; de-etiolated-1</td></tr>
<tr><td>MSMO1</td><td>2045.929</td><td>-0.44153</td><td>0.145318763</td><td>-3.03834</td><td>0.0023788</td><td>0.020157</td><td>Not_Sig</td><td>no</td><td>gi|149642567|ref|NP_001092333.1|</td><td>1434</td><td>0</td><td>methylsterol monooxygenase 1 [Bos taurus]</td><td>gi|149642566|ref|NM_001098863.1|</td><td>1785</td><td>0</td><td>Bos taurus methylsterol monooxygenase 1 (MSMO1), mRNA</td><td>Q5R574</td><td>543</td><td>0</td><td>Methylsterol monooxygenase 1</td><td>SPAC630.08c</td><td>181</td><td>2.59e-51</td><td>COG3000</td><td>pfam04116</td><td>FA_hydroxylase</td><td>Fatty acid hydroxylase superfamily. This superfamily includes fatty acid and carotene hydroxylases and sterol desaturases. Beta-carotene</td><td>GO:0006633|fatty acid biosynthetic process;GO:0016126|sterol biosynthetic process;</td><td>GO:0005789|endoplasmic reticulum membrane;GO:0016021|integral component of membrane;</td><td>GO:0000254|C-4 methylsterol oxidase activity;GO:0005506</td><td>K07750</td><td>E1.14.13.72, SC4MOL, ERG25; methylsterol monooxygenase</td></tr>
<tr><td>DAGLB</td><td>353.3585</td><td>0.430547</td><td>0.141730866</td><td>3.037775</td><td>0.0023833</td><td>0.02018</td><td>Not_Sig</td><td>no</td><td>gi|742149797|ref|XP_010844062.1|</td><td>3172</td><td>0</td><td>PREDICTED: sn1-specific diacylglycerol lipase beta [Bison bison bison]</td><td>gi|594039620|ref|XM_006044335.1|</td><td>3753</td><td>0</td><td>PREDICTED: Bubalus bubalis diacylglycerol lipase, beta (DAGLB), partial mRNA</td><td>Q8NCG7</td><td>1048</td><td>0</td><td>Sn1-specific diacylglycerol lipase beta</td><td>.</td><td>.</td><td>.</td><td>.</td><td>pfam01764</td><td>Lipase_3</td><td>Lipase (class 3).</td><td>GO:0019369|arachidonic acid metabolic process;GO:0016042|lipid catabolic process;GO:0007405|neuroblast proliferation;GO:0042136|neurotransmitter biosynthetic process;</td><td>GO:0016021|integral component of membrane;GO:0005765|lysosomal membrane;GO:0005886|plasma membrane;</td><td>GO:0047372|acylglycerol lipase activity;GO:0046872|metal ion binding;</td><td>K13806</td><td>DAGL; sn1-specific diacylglycerol lipase [EC:3.1.1.-]</td></tr>
<tr><td>PDCD4</td><td>422.2009</td><td>-0.39936</td><td>0.131494575</td><td>-3.03705</td><td>0.0023891</td><td>0.020214</td><td>Not_Sig</td><td>no</td><td>gi|139948333|ref|NP_001077116.1|</td><td>2148</td><td>0</td><td>programmed cell death protein 4 [Bos taurus]</td><td>gi|742200133|ref|XM_010859354.1|</td><td>2250</td><td>0</td><td>PREDICTED: Bison bison bison programmed cell death 4 (neoplastic transformation inhibitor) (PDCD4), transcript variant X1, mRNA</td><td>Q9JID1</td><td>811</td><td>0</td><td>Programmed cell death protein 4</td><td>.</td><td>.</td><td>.</td><td>.</td><td>pfam02847</td><td>MA3</td><td>MA3 domain. Domain in DAP-5, eIF4G, MA-3 and other proteins. Highly alpha-helical. May contain repeats and/or regions similar to</td><td>GO:0006915|apoptotic process;GO:0007569|cell aging;GO:0043066|negative regulation of apoptotic process;GO:0043508|negative regulation of JUN kinase activity;GO:0045892|negative regulation of transcription, DNA-templated;GO:0034393|positive regulation of smooth muscle cell apoptotic process;GO:0051246|regulation of protein metabolic process;</td><td>GO:0005737|cytoplasm;GO:0005829|cytosol;GO:0005654|nucleoplasm;GO:0005634|nucleus;</td><td>GO:0003723|RNA binding;</td><td>K16865</td><td>PDCD4; programmed cell death protein 4</td></tr>
<tr><td>KIFC1</td><td>521.7075</td><td>0.385032</td><td>0.126795974</td><td>3.036627</td><td>0.0023924</td><td>0.020227</td><td>Not_Sig</td><td>no</td><td>gi|741970005|ref|XP_010816335.1|</td><td>2884</td><td>0</td><td>PREDICTED: kinesin-like protein KIFC1 isoform X1 [Bos taurus]</td><td>gi|982962982|ref|XM_010818033.2|</td><td>2245</td><td>0</td><td>PREDICTED: Bos taurus kinesin family member C1 (KIFC1), transcript variant X1, mRNA</td><td>Q9BW19</td><td>973</td><td>0</td><td>Kinesin-like protein KIFC1</td><td>SPAC3A11.14c</td><td>332</td><td>2.69e-101</td><td>COG5059</td><td>pfam00225</td><td>Kinesin</td><td>Kinesin motor domain.</td><td>GO:0051301|cell division;GO:0007018|microtubule-based movement;GO:0007080|mitotic metaphase plate congression;GO:0000070|mitotic sister chromatid segregation;GO:0090307|mitotic spindle assembly;GO:0007283|spermatogenesis;</td><td>GO:0005769|early endosome;GO:0005871|kinesin complex;GO:0016020|membrane;GO:0005874|microtubule;GO:0005815|microtubule</td><td>GO:0005524|ATP binding;GO:0016887|ATPase activity;GO:0003777</td><td>K10405</td><td>KIFC1; kinesin family member C1</td></tr>
<tr><td>PM20D1</td><td>282.9363</td><td>0.417381</td><td>0.137519717</td><td>3.035062</td><td>0.0024049</td><td>0.020318</td><td>Not_Sig</td><td>no</td><td>gi|742179651|ref|XP_010852483.1|</td><td>2532</td><td>0</td><td>PREDICTED: probable carboxypeptidase PM20D1 [Bison bison bison]</td><td>gi|942096730|ref|XM_005909476.2|</td><td>2186</td><td>0</td><td>PREDICTED: Bos mutus peptidase M20 domain containing 1 (PM20D1), mRNA</td><td>Q2T9M7</td><td>969</td><td>0</td><td>N-fatty-acyl-amino acid synthase/hydrolase PM20D1</td><td>RSp0487</td><td>298</td><td>4.95e-92</td><td>COG0624</td><td>pfam01546</td><td>Peptidase_M20</td><td>Peptidase family M20/M25/M40. This family includes a range of zinc metallopeptidases belonging to several families in the peptidase</td><td>GO:1990845|adaptive thermogenesis;GO:0043604|amide biosynthetic process;GO:0043605|cellular amide catabolic process;GO:0006520|cellular amino acid metabolic process;GO:0044255|cellular lipid metabolic process;GO:0097009|energy homeostasis;GO:0043171|peptide catabolic process;GO:2000275|regulation of oxidative phosphorylation uncoupler activity;</td><td>GO:0005615|extracellular space;</td><td>GO:0016811|hydrolase activity, acting on carbon-nitrogen (but not peptide)</td><td>K13049</td><td>PM20D1; carboxypeptidase PM20D1 [EC:3.4.17.-]</td></tr>
<tr><td>CRTC2</td><td>501.8838</td><td>0.578595</td><td>0.190681361</td><td>3.034355</td><td>0.0024105</td><td>0.020328</td><td>Not_Sig</td><td>no</td><td>gi|555996703|ref|XP_005910086.1|</td><td>2533</td><td>0</td><td>PREDICTED: CREB-regulated transcription coactivator 2 [Bos mutus]</td><td>gi|555996702|ref|XM_005910024.1|</td><td>2634</td><td>0</td><td>PREDICTED: Bos mutus CREB regulated transcription coactivator 2 (CRTC2), mRNA</td><td>Q08E26</td><td>975</td><td>0</td><td>CREB-regulated transcription coactivator 2</td><td>.</td><td>.</td><td>.</td><td>.</td><td>pfam12885</td><td>TORC_M</td><td>Transducer of regulated CREB activity middle domain. This family includes the region between the N and C terminus of TORC proteins.</td><td>GO:0006094|gluconeogenesis;GO:0042593|glucose homeostasis;GO:0032793|positive regulation of CREB transcription factor activity;GO:0045944|positive regulation of transcription from RNA polymerase II promoter;GO:0051289|protein homotetramerization;GO:0006351|transcription, DNA-templated;</td><td>GO:0005737|cytoplasm;GO:0005634|nucleus;</td><td>GO:0008140|cAMP response element binding protein binding;</td><td>K16333</td><td>CRTC2, TORC2; CREB-regulated transcription coactivator 2</td></tr>
<tr><td>LIMS1</td><td>802.6701</td><td>-0.37668</td><td>0.124139954</td><td>-3.0343</td><td>0.0024109</td><td>0.020328</td><td>Not_Sig</td><td>no</td><td>gi|528968431|ref|XP_005212716.1|</td><td>1896</td><td>0</td><td>PREDICTED: LIM and senescent cell antigen-like-containing domain protein 1 isoform X1 [Bos taurus]</td><td>gi|803037317|ref|XM_012170483.1|</td><td>3474</td><td>0</td><td>PREDICTED: Ovis aries LIM and senescent cell antigen-like domains 1 (LIMS1), transcript variant X1, mRNA</td><td>Q99JW4</td><td>592</td><td>0</td><td>LIM and senescent cell antigen-like-containing domain protein 1</td><td>.</td><td>.</td><td>.</td><td>.</td><td>pfam00412</td><td>LIM</td><td>LIM domain. This family represents two copies of the LIM structural domain.</td><td>GO:0045216|cell-cell junction organization;GO:0007160|cell-matrix adhesion;GO:0043009|chordate embryonic development;GO:0045184|establishment of protein localization;GO:0007163|establishment or maintenance of cell polarity;GO:0043066|negative regulation of apoptotic process;GO:0050680|negative regulation of epithelial cell proliferation;GO:2000346|negative regulation of hepatocyte proliferation;GO:2000178|negative regulation of neural precursor cell</td><td>GO:0005911|cell-cell junction;GO:0005925|focal adhesion;GO:0048471|perinuclear region of cytoplasm;GO:0005886|plasma membrane;</td><td>GO:0019901|protein kinase binding;GO:0008270|zinc ion binding;</td><td>.</td><td>.</td></tr>
<tr><td>TRAPPC2</td><td>259.2628</td><td>-0.53659</td><td>0.176845564</td><td>-3.03424</td><td>0.0024114</td><td>0.020328</td><td>Not_Sig</td><td>no</td><td>gi|395753700|ref|XP_003779644.1|</td><td>776</td><td>3e-97</td><td>PREDICTED: trafficking protein particle complex subunit 2 isoform X1 [Pongo abelii]</td><td>gi|942037790|ref|XM_005889577.2|</td><td>1558</td><td>0</td><td>PREDICTED: Bos mutus trafficking protein particle complex 2 (TRAPPC2), mRNA</td><td>F1SRI0</td><td>291</td><td>7e-95</td><td>Trafficking protein particle complex subunit 2</td><td>SPBC11G11.04</td><td>147</td><td>2.27e-41</td><td>COG5603</td><td>pfam04628</td><td>Sedlin_N</td><td>Sedlin, N-terminal conserved region. Mutations in this protein are associated with the X-linked spondyloepiphyseal dysplasia</td><td>GO:0006888|ER to Golgi vesicle-mediated transport;GO:0006351|transcription, DNA-templated;</td><td>GO:0005783|endoplasmic reticulum;GO:0005794|Golgi apparatus;GO:0005634|nucleus;GO:0048471|perinuclear region of cytoplasm;</td><td>.</td><td>.</td><td>.</td></tr>
<tr><td>SNRPG</td><td>416.5641</td><td>-0.51464</td><td>0.169622946</td><td>-3.034</td><td>0.0024134</td><td>0.02033</td><td>Not_Sig</td><td>no</td><td>gi|4507133|ref|NP_003087.1|</td><td>394</td><td>1e-45</td><td>small nuclear ribonucleoprotein G isoform a [Homo sapiens]</td><td>gi|746816120|ref|XM_001040543.3|</td><td>650</td><td>0</td><td>Bos taurus small nuclear ribonucleoprotein polypeptide G (SNRPG), mRNA</td><td>P62309</td><td>156</td><td>4e-47</td><td>Small nuclear ribonucleoprotein G</td><td>.</td><td>.</td><td>.</td><td>.</td><td>pfam01423</td><td>LSM</td><td>LSM domain. The LSM domain contains Sm proteins as well as other related LSM (Like Sm) proteins. The U1, U2, U4/U6, and</td><td>GO:0000398|mRNA splicing, via spliceosome;GO:0000387|spliceosomal snRNP assembly;</td><td>GO:0071013|catalytic step 2 spliceosome;GO:0005829|cytosol;GO:0034709|methylosome;GO:0043186|P granule;GO:0071011|precatalytic</td><td>GO:0003723|RNA binding;</td><td>K11099</td><td>SNRPG, SMG; small nuclear ribonucleoprotein G</td></tr>
</table>

LOC102286668	150.2449	-0.65586	0.216233042	-3.03311	0.0024205	0.020374	DOWN	yes	gi|958720315|ref|XP_014703348.1|	781	6e-103	PREDICTED: 40S ribosomal protein S13 [Equus asinus]	gi|75832133|ref|NM_001025342.2|	510	0	Bos taurus ribosomal protein S13 (RPS13), mRNA	P62278	296	1e-101	40S ribosomal protein S13	YDR064w	243	4.22e-83	COG0184	pfam08069	Ribosomal_S13_N	Ribosomal S13/S15 N-terminal domain. This domain is found at the N-terminus of ribosomal S13 and S15 proteins. This domain is	GO:0033119|negative regulation of RNA splicing;GO:0006412|translation;	GO:0022627|cytosolic small ribosomal subunit;GO:0070062|extracellular exosome;GO:0005925|focal adhesion;GO:0016020|membrane;GO:0005	GO:0003729|mRNA binding;GO:0070181|small ribosomal subunit rRNA	K02953	RP-S13e, RPS13; small subunit ribosomal protein S13e
WDR7	391.3702	0.416522	0.137381267	3.031871	0.0024304	0.020443	Not_Sig	no	gi|741972817|ref|XP_010817213.1|	7378	0	PREDICTED: WD repeat-containing protein 7 isoform X3 [Bos taurus]	gi|982965571|ref|XM_010818911.2|	5222	0	PREDICTED: Bos taurus WD repeat domain 7 (WDR7), transcript variant X3, mRNA	Q9Y4E6	2727	0	WD repeat-containing protein 7	all0438_2	61.2	1.08e-08	COG2319	pfam00400	WD40	WD domain, G-beta repeat.	GO:0002244|hematopoietic progenitor cell differentiation;	GO:0005737|cytoplasm;GO:0008021|synaptic vesicle;	.	.	.
UBTF	925.1357	0.3299	0.108876458	3.030038	0.0024452	0.020553	Not_Sig	no	gi|297487171|ref|XP_002696088.1|	3178	0	PREDICTED: nucleolar transcription factor 1 isoform X1 [Bos taurus]	gi|742086748|ref|XM_010832458.1|	2813	0	PREDICTED: Bison bison bison upstream binding transcription factor, RNA polymerase I (UBTF), transcript variant X2, mRNA	P25977	1191	0	Nucleolar transcription factor 1	.	.	.	.	pfam14887	HMG_box_5	HMG (high mobility group) box 5. Nucleolar transcription factor/upstream binding factor contains six HMG box domains.	GO:0045943|positive regulation of transcription from RNA polymerase I promoter;GO:0006351|transcription, DNA-templated;	GO:0001650|fibrillar center;GO:0005730|nucleolus;GO:0005634|nucleus;	GO:0003677|DNA binding;	K09273	UBTF; upstream-binding transcription factor
BACH1	1401.757	-0.30842	0.101837702	-3.02858	0.002457	0.020629	Not_Sig	no	gi|742186141|ref|XP_010854120.1|	3062	0	PREDICTED: transcription regulator protein BACH1 [Bison bison bison]	gi|742186140|ref|XM_010855818.1|	1918	0	PREDICTED: Bison bison bison BTB and CNC homology 1, basic leucine zipper transcription factor 1 (BACH1), transcript variant X1, mRNA	O14867	931	0	Transcription regulator protein BACH1	.	.	.	.	pfam00651	BTB	BTB/POZ domain. The BTB (for BR-C, ttk and bab) or POZ (for Pox virus and Zinc finger) domain is present near the N-terminus of a	GO:0006310|DNA recombination;GO:0006281|DNA repair;GO:0006302|double-strand break repair;GO:0000122|negative regulation of transcription from RNA polymerase II promoter;GO:0016567|protein ubiquitination;GO:0061418|regulation of transcription from RNA polymerase II promoter in response to hypoxia;GO:0000083|regulation of transcription involved in G1/S transition of mitotic cell cycle;GO:0000117|regulation of transcription involved in G2/M transition of mitotic cell cycle;GO:0006355|regulation of transcription, DNA-	GO:0031463|Cul3-RING ubiquitin ligase complex;GO:0005737|cytoplasm;GO:0005634|nucleus;	GO:0020037|heme binding;GO:0000980|RNA polymerase II distal enhancer	K09042	BACH; transcription regulator BACH
LOC138991803	231.9411	0.586175	0.19355472	3.028472	0.0024579	0.020629	UP	yes	gi|694975020|ref|XP_009433825.1|	151	5e-08	PREDICTED: CAP-Gly domain-containing linker protein 3 [Pan troglodytes]	gi|850489142|gb|CP011899.1|	536	0	Ovis canadensis canadensis isolate 43U chromosome 14 sequence	Q96DZ5	60.5	1e-08	CAP-Gly domain-containing linker protein 3	.	.	.	.	pfam09879	DUF2106	Predicted membrane protein (DUF2106). This domain, found in various hypothetical archaeal proteins, has no known function.	GO:0072321|chaperone-mediated protein transport;GO:0045444|fat cell differentiation;GO:0044091|membrane biogenesis;GO:0031115|negative regulation of microtubule polymerization;GO:0018230|peptidyl-L-cysteine S-palmitoylation;GO:0043065|positive regulation of apoptotic process;GO:0045807|positive regulation of endocytosis;GO:0090004|positive regulation of establishment of protein localization to plasma membrane;GO:0010828|positive regulation of glucose	GO:0005829|cytosol;GO:0031901|early endosome membrane;GO:0005795|Golgi stack;GO:0045121|membrane raft;GO:0005886|plasma	GO:0035594|ganglioside binding;GO:0008017|microtubule	K10423	CLIP3_4; CAP-Gly domain-containing linker protein 3/4
IRAK4	255.2739	-0.46643	0.154028628	-3.02818	0.0024603	0.020634	Not_Sig	no	gi|555970461|ref|XP_005897252.1|	2369	0	PREDICTED: interleukin-1 receptor-associated kinase 4 [Bos mutus]	gi|982925339|ref|XM_005206426.3|	2623	0	PREDICTED: Bos taurus interleukin 1 receptor associated kinase 4 (IRAK4), transcript variant X1, mRNA	Q1RMT8	911	0	Interleukin-1 receptor-associated kinase 4	PA0074_1	112	1.18e-25	COG0515	pfam07714	Pkinase_Tyr	Protein tyrosine kinase.	GO:0001816|cytokine production;GO:0019221|cytokine-mediated signaling pathway;GO:0045087|innate immune response;GO:0035556|intracellular signal transduction;GO:0002446|neutrophil mediated immunity;GO:1990266|neutrophil migration;GO:0043123|positive regulation of I-kappaB kinase/NF-kappaB signaling;	GO:0005737|cytoplasm;GO:0005634|nucleus;	GO:0005524|ATP binding;GO:0000287|magnesium ion binding;GO:0004674	K04733	IRAK4; interleukin-1 receptor-associated kinase 4 [EC:2.7.11.1]
CDK16	1158.666	0.480854	0.158816016	3.027742	0.0024639	0.020649	Not_Sig	no	gi|529015155|ref|XP_005228149.1|	2523	0	PREDICTED: cyclin-dependent kinase 16 isoform X4 [Bos taurus]	gi|965893936|ref|XM_012158996.2|	2853	0	PREDICTED: Ovis aries musimon cyclin-dependent kinase 16 (CDK16), transcript variant X5, mRNA	Q00536	795	0	Cyclin-dependent kinase 16	.	.	.	.	pfam00069	Pkinase	Protein kinase domain.	GO:0006887|exocytosis;GO:0030252|growth hormone secretion;GO:0031175|neuron projection development;GO:0006468|protein phosphorylation;GO:0061178|regulation of insulin secretion involved in cellular response to glucose stimulus;GO:0007283|spermatogenesis;	GO:0030054|cell junction;GO:0005737|cytoplasm;GO:0031234|extrinsic component of cytoplasmic side of plasma	GO:0005524|ATP binding;GO:0004693|cyclin-dependent protein	K08820	CDK16, PCTK1; cyclin-dependent kinase 16 [EC:2.7.11.22]
ABCB8	522.0889	0.462602	0.1528641	3.02623	0.0024762	0.020738	Not_Sig	no	gi|803262129|ref|XP_011990767.1|	3143	0	PREDICTED: ATP-binding cassette sub-family B member 8, mitochondrial isoform X3 [Ovis aries musimon]	gi|982981222|ref|XM_015463180.1|	4117	0	PREDICTED: Bos taurus ATP binding cassette subfamily B member 8 (ABCB8), transcript variant X1, mRNA	Q5RFQ9	1010	0	ATP-binding cassette sub-family B member 8, mitochondrial	CC3274	344	2.09e-103	COG1132	pfam00005	ABC_tran	ABC transporter. ABC transporters for a large family of proteins responsible for translocation of a variety of compounds across	.	GO:0016021|integral component of membrane;GO:0005743|mitochondrial inner membrane;	GO:0005524|ATP binding;GO:0042626|ATPase activity, coupled to	K05655	ABCB8; ATP-binding cassette, subfamily B (MDR/TAP), member 8
SCAF4	618.6615	0.393971	0.130250025	3.02473	0.0024885	0.020812	Not_Sig	no	gi|296491707|tpg|DAA33740.1|	1538	4e-180	TPA: splicing factor, arginine/serine-rich 15-like [Bos taurus]	gi|742212292|ref|XM_010862310.1|	5443	0	PREDICTED: Bison bison bison SR-related CTD-associated factor 4 (SCAF4), transcript variant X2, mRNA	O95104	531	2e-160	Splicing factor, arginine/serine-rich 15	.	.	.	.	pfam04818	CTD_bind	RNA polymerase II-binding domain. This domain binds to the phosphorylated C-terminal domain (CTD) of RNA polymerase II.	.	GO:0005654|nucleoplasm;	GO:0000166|nucleotide binding;GO:0044822|poly(A) RNA	K13167	SFRS15, SCAF4; splicing factor, arginine/serine-rich 15
PIGQ	643.1729	0.497621	0.164529025	3.024516	0.0024903	0.020812	Not_Sig	no	gi|555949381|ref|XP_005886901.1|	2443	0	PREDICTED: phosphatidylinositol N-acetylglucosaminyltransferase subunit Q [Bos mutus]	gi|942029132|ref|XM_005886839.2|	2895	0	PREDICTED: Bos mutus phosphatidylinositol glycan anchor biosynthesis, class Q (PIGQ), mRNA	Q9QYT7	795	0	Phosphatidylinositol N-acetylglucosaminyltransferase subunit Q	.	.	.	.	pfam05024	Gpi1	N-acetylglucosaminyl transferase component (Gpi1). Glycosylphosphatidylinositol (GPI) represents an important anchoring	GO:0006506|GPI anchor biosynthetic process;	GO:0000506|glycosylphosphatidylinositol-N-acetylglucosaminyltransferase (GPI-GnT) complex;GO:0016021|integral component of membrane;	GO:0017176|phosphatidylinositol N-acetylglucosaminyltransferase activity;	K03860	PIGQ, GPI1; phosphatidylinositol glycan, class Q
TMX1	837.7254	-0.42236	0.139648535	-3.02448	0.0024906	0.020812	Not_Sig	no	gi|115494922|ref|NP_001068853.1|	1234	8e-161	thioredoxin-related transmembrane protein 1 precursor [Bos taurus]	gi|742134335|ref|XM_010842265.1|	2408	0	PREDICTED: Bison bison bison thioredoxin-related transmembrane protein 1 (TMX1), mRNA	Q0Z7W6	479	2e-162	Thioredoxin-related transmembrane protein 1	SPAC1F5.02	67.4	8.10e-11	COG0526	pfam00085	Thioredoxin	Thioredoxin. Thioredoxins are small enzymes that participate in redox reactions, via the reversible oxidation of an active centre	GO:0045454|cell redox homeostasis;GO:0006457|protein folding;GO:0034976|response to endoplasmic reticulum stress;	GO:0005783|endoplasmic reticulum;GO:0005789|endoplasmic reticulum membrane;GO:0016021|integral component of	GO:0015036|disulfide oxidoreductase activity;GO:0003756|protein disulfide	.	.
TMEM175	509.4431	0.431002	0.142520035	3.02415	0.0024933	0.02082	Not_Sig	no	gi|942037938|ref|XP_014331832.1|	2396	0	PREDICTED: endosomal/lysomomal potassium channel TMEM175 isoform X1 [Bos mutus]	gi|942037937|ref|XM_014476346.1|	2482	0	PREDICTED: Bos mutus transmembrane protein 175 (TMEM175), transcript variant X1, mRNA	Q32PG7	858	0	Endosomal/lysomomal potassium channel TMEM175	L85237	57.8	3.93e-08	COG3548	pfam00078	RVT_1	Reverse transcriptase (RNA-dependent DNA polymerase). A reverse transcriptase gene is usually indicative of a mobile	GO:0090385|phagosome-lysosome fusion;GO:0071805|potassium ion transmembrane transport;GO:0035751|regulation of lysosomal lumen pH;	GO:0005768|endosome;GO:0010008|endosome membrane;GO:0016021|integral component of membrane;GO:0005765|lysosomal	GO:0022841|potassium ion leak channel activity;	.	.
PFKFB3	1779.423	0.396809	0.131232817	3.023704	0.002497	0.020836	Not_Sig	no	gi|635051482|ref|XP_008000388.1|	2814	0	PREDICTED: 6-phosphofructo-2-kinase/fructose-2,6-bisphosphatase 3 isoform X2 [Chlorocebus sabaeus]	gi|982942168|ref|XM_005214135.3|	4275	0	PREDICTED: Bos taurus 6-phosphofructo-2-kinase/fructose-2,6-biphosphatase 3 (PFKFB3), transcript variant X2, mRNA	Q16875	1051	0	6-phosphofructo-2-kinase/fructose-2,6-bisphosphatase 3	YJL155c_2	239	5.01e-71	COG0406	pfam01591	6PF2K	6-phosphofructo-2-kinase. This enzyme occurs as a bifunctional enzyme with fructose-2,6-bisphosphatase. The bifunctional	GO:0007420|brain development;GO:0061621|canonical glycolysis;GO:0006003|fructose 2,6-bisphosphate metabolic process;GO:0006000|fructose metabolic process;	GO:0005829|cytosol;GO:0005654|nucleoplasm;	GO:0003873|6-phosphofructo-2-kinase activity;GO:0005524	K01103	PFKFB3; 6-phosphofructo-2-kinase / fructose-2,6-biphosphatase
ERBB2	780.0792	0.364717	0.12066104	3.022656	0.0025057	0.020893	Not_Sig	no	gi|747019242|ref|NP_001290558.1|	5902	0	receptor tyrosine-protein kinase erbB-2 precursor [Bos taurus]	gi|747019241|ref|NM_001303629.1|	4466	0	Bos taurus erb-b2 receptor tyrosine kinase 2 (ERBB2), mRNA	O18735	2122	0	Receptor tyrosine-protein kinase erbB-2	.	.	.	.	pfam07714	Pkinase_Tyr	Protein tyrosine kinase.	GO:0071364|cellular response to epidermal growth factor stimulus;GO:0071363|cellular response to growth factor stimulus;GO:0030307|positive regulation of cell growth;GO:0090314|positive regulation of protein targeting to membrane;GO:0045943|positive regulation of transcription from RNA polymerase I promoter;GO:0045945|positive regulation of transcription from RNA polymerase III promoter;GO:0045727|positive regulation of translation;GO:0070372|regulation of ERK1 and ERK2	GO:0016021|integral component of membrane;GO:0005634|nucleus;GO:0048471|perinuclear region of cytoplasm;GO:0005886|plasma membrane;	GO:0005524|ATP binding;GO:0004716|receptor signaling protein tyrosine	.	.
HIRIP3	212.8123	0.45473	0.150584919	3.019759	0.0025298	0.021078	Not_Sig	no	gi|440907048|gb|ELR57240.1|	2124	0	HIRA-interacting protein 3 [Bos mutus]	gi|742107188|ref|XM_010837298.1|	1958	0	PREDICTED: Bison bison bison HIRA interacting protein 3 (HIRIP3), mRNA	Q9BW71	526	2e-178	HIRA-interacting protein 3	.	.	.	.	pfam07218	RAP1	Rhoptry-associated protein 1 (RAP-1). This family consists of several rhoptry-associated protein 1 (RAP-1) sequences which appear to be	GO:0006333|chromatin assembly or disassembly;	GO:0005730|nucleolus;GO:0005634|nucleus;	.	.	.
MAP1LC3B	1355.387	-0.36154	0.119750361	-3.01911	0.0025352	0.021091	Not_Sig	no	gi|440895359|gb|ELR47567.1|	254	4e-19	Zinc finger protein 879, partial [Bos mutus]	gi|742167899|ref|XM_010850843.1|	1959	0	PREDICTED: Bison bison bison microtubule-associated protein 1 light chain 3 beta (MAP1LC3B), mRNA	Q9GZQ8	68.9	2e-11	Microtubule-associated proteins 1A/1B light chain 3B	.	.	.	.	pfam04484	DUF566	Family of unknown function (DUF566). Family of related proteins that is plant specific.	GO:0000045|autophagosome assembly;GO:0006995|cellular response to nitrogen starvation;GO:0016236|macroautophagy;GO:1903146|regulation of mitophagy;	GO:0005776|autophagosome;GO:0000421|autophagosome membrane;GO:0005930|axoneme;GO:0031410|cytoplasmic	GO:0008017|microtubule binding;GO:0031625|ubiquitin protein	K10435	MAP1LC; microtubule-associated protein 1 light chain
RPS6	7010.374	-0.35015	0.115978983	-3.01907	0.0025355	0.021091	Not_Sig	no	gi|755785705|ref|XP_003995546.2|	1144	5e-154	PREDICTED: 40S ribosomal protein S6 [Felis catus]	gi|371873165|ref|NM_001015548.2|	883	0	Bos taurus ribosomal protein S6 (RPS6), mRNA	Q5E995	428	7e-150	40S ribosomal protein S6	SPAC13G6.07c	300	4.17e-102	COG2125	pfam01092	Ribosomal_S6e	Ribosomal protein S6e.	GO:0042593|glucose homeostasis;GO:0042274|ribosomal small subunit biogenesis;GO:0006364|rRNA processing;GO:0006412|translation;	GO:0022627|cytosolic small ribosomal subunit;GO:0005634|nucleus;GO:0048471|perinuclear region of cytoplasm;	GO:0003735|structural constituent of ribosome;	K02991	RP-S6e, RPS6; small subunit ribosomal protein S6e
PLPPR2	148.8592	0.631201	0.209082159	3.018914	0.0025368	0.021091	UP	yes	gi|440910156|gb|ELR59982.1|	1822	0	Lipid phosphate phosphatase-related protein type 2 [Bos mutus]	gi|982930420|ref|XM_015472007.1|	3533	0	PREDICTED: Bos taurus phospholipid phosphatase related 2 (PLPPR2), transcript variant X3, mRNA	Q96GM1	561	0	Phospholipid phosphatase-related protein type 2	.	.	.	.	pfam01569	PAP2	PAP2 superfamily. This family includes the enzyme type 2 phosphatidic acid phosphatase (PAP2), Glucose-6-phosphatase	GO:0046839|phospholipid dephosphorylation;GO:0006644|phospholipid metabolic process;GO:0007165|signal transduction;	GO:0005887|integral component of plasma membrane;	GO:0042577|lipid phosphatase activity;GO:0008195|phosphatidate	K19581	LPPR1_2_5; lipid phosphate phosphatase-related protein type 1/2/5
EPHB4	375.0655	0.401702	0.133083295	3.018428	0.0025409	0.021102	Not_Sig	no	gi|555998276|ref|XP_005910851.1|	4935	0	PREDICTED: ephrin type-B receptor 4 isoform X1 [Bos mutus]	gi|942100449|ref|XM_005910789.2|	3921	0	PREDICTED: Bos mutus EPH receptor B4 (EPHB4), transcript variant X1, mRNA	P54760	1833	0	Ephrin type-B receptor 4	.	.	.	.	pfam07714	Pkinase_Tyr	Protein tyrosine kinase.	GO:0001525|angiogenesis;GO:0007155|cell adhesion;GO:0002042|cell migration involved in sprouting angiogenesis;GO:0048013|ephrin receptor signaling pathway;GO:0003007|heart morphogenesis;GO:0046777|protein autophosphorylation;	GO:0005829|cytosol;GO:0070062|extracellular exosome;GO:0005576|extracellular region;GO:0005887|integral component of plasma membrane;GO:0005886|plasma	GO:0005524|ATP binding;GO:0005003|ephrin receptor activity;GO:0004714	K05113	EPHB4, HTK; Eph receptor B4 [EC:2.7.10.1]
CSRP1	10635.08	0.448067	0.148449175	3.01832	0.0025418	0.021102	Not_Sig	no	gi|139948177|ref|NP_001077240.1|	785	1e-97	cysteine and glycine-rich protein 1 [Bos taurus]	gi|942067981|ref|XM_005900276.2|	1633	0	PREDICTED: Bos mutus cysteine and glycine-rich protein 1 (CSRP1), transcript variant X1, mRNA	Q3MHY1	306	3e-99	Cysteine and glycine-rich protein 1	.	.	.	.	pfam00412	LIM	LIM domain. This family represents two copies of the LIM structural domain.	GO:0070527|platelet aggregation;	GO:0070062|extracellular exosome;GO:0005925|focal adhesion;GO:0005634|nucleus;	GO:0044822|poly(A) RNA binding;GO:0008270|zinc ion binding;	K09377	CSRP; cysteine and glycine-rich protein
NUDCD2	474.4485	-0.41623	0.137926697	-3.01778	0.0025464	0.021104	Not_Sig	no	gi|556773874|ref|XP_005982083.1|	1048	1e-130	PREDICTED: nudC domain-containing protein 2 [Pantholops hodgsonii]	gi|942036702|ref|XM_005889207.2|	2262	0	PREDICTED: Bos mutus NudC domain containing 2 (NUDCD2), transcript variant X1, mRNA	Q9CQ48	325	6e-102	NudC domain-containing protein 2	.	.	.	.	pfam04969	CS	CS domain. The CS and CHORD (pfam04968) are fused into a single polypeptide chain in metazoans but are found in	GO:0032502|developmental process;GO:0006457|protein folding;	GO:0000777|condensed chromosome kinetochore;GO:0005737|cytoplasm;GO:0070062|extracellular exosome;GO:0005622|intracellular;GO:001	GO:0051082|unfolded protein binding;	.	.
UQCR11	465.9758	-0.46243	0.153239733	-3.01767	0.0025472	0.021104	Not_Sig	no	gi|27806355|ref|NP_776640.1|	290	3e-31	cytochrome b-c1 complex subunit 10 [Bos taurus]	gi|742142239|ref|XM_010844555.1|	411	0	PREDICTED: Bison bison bison cytochrome b-c1 complex subunit 10 (LOC104991884), mRNA	P07552	116	1e-32	Cytochrome b-c1 complex subunit 10	.	.	.	.	pfam08997	UCR_6-4kD	Ubiquinol-cytochrome C reductase complex, 6.4kD protein. The ubiquinol-cytochrome C reductase complex (cytochrome bc1	.	GO:0016021|integral component of membrane;GO:0005743|mitochondrial inner membrane;GO:0070469|respiratory chain;	GO:0009055|electron carrier activity;GO:0008121|ubiquinol-	K00420	QCR10, UQCR; ubiquinol-cytochrome c reductase subunit 10
SUMO1	1124.002	-0.40618	0.134600882	-3.01764	0.0025475	0.021104	Not_Sig	no	gi|594115840|ref|XP_006080600.1|	942	1e-122	PREDICTED: uncharacterized protein LOC102390768 [Bubalus bubalis]	gi|594115839|ref|XM_006080538.1|	1362	0	PREDICTED: Bubalus bubalis uncharacterized LOC102390768 (LOC102390768), mRNA	Q5I0H3	211	5e-65	Small ubiquitin-related modifier 1	SPBC365.06	98.6	4.45e-24	COG5227	pfam11976	Rad60-SLD	Ubiquitin-2 like Rad60 SUMO-like. The small ubiquitin-related modifier SUMO-1 is a Ub/Ubl family member, and although	GO:0045759|negative regulation of action potential;GO:1902260|negative regulation of delayed rectifier potassium channel activity;GO:0043392|negative regulation of DNA binding;GO:0043433|negative regulation of sequence-specific DNA binding transcription factor activity;GO:0045892|negative regulation of transcription, DNA-templated;GO:0060021|palate development;GO:0030578|PML body organization;GO:1901896|positive regulation of calcium-transporting ATPase activity;GO:0032436|positive regulation of proteasomal	GO:0005737|cytoplasm;GO:0030425|dendrite;GO:0001650|fibrillar center;GO:0031965|nuclear membrane;GO:0016607|nuclear	GO:0044822|poly(A) RNA binding;GO:0015459|potassium channel	K12160	SUMO, SMT3; small ubiquitin-related modifier
PRKACA	1089.583	0.358806	0.118923901	3.017103	0.002552	0.021126	Not_Sig	no	gi|795433196|ref|XP_011949526.1|	1831	0	PREDICTED: cAMP-dependent protein kinase catalytic subunit alpha [Cercocebus atys]	gi|926695198|ref|XM_013965083.1|	2003	0	PREDICTED: Capra hircus protein kinase, cAMP-dependent, catalytic, alpha (PRKACA), mRNA	P00517	709	0	cAMP-dependent protein kinase catalytic subunit alpha	.	.	.	.	pfam00069	Pkinase	Protein kinase domain.	GO:0071333|cellular response to glucose stimulus;GO:0071374|cellular response to parathyroid hormone stimulus;GO:0001707|mesoderm formation;GO:0050804|modulation of synaptic transmission;GO:0006397|mRNA processing;GO:1901621|negative regulation of smoothened signaling pathway involved in dorsal/ventral neural tube patterning;GO:0001843|neural tube closure;GO:0018105|peptidyl-serine phosphorylation;GO:0018107|peptidyl-threonine phosphorylation;GO:0071158|positive regulation of cell cycle arrest;GO:0046827|positive	GO:0005813|centrosome;GO:0097546|ciliary base;GO:0005737|cytoplasm;GO:0070062|extracellular	GO:0005524|ATP binding;GO:0004691|cAMP-dependent protein kinase	K04345	PKA; protein kinase A [EC:2.7.11.11]
GTPBP1	550.6559	0.37876	0.125576448	3.016168	0.0025599	0.021176	Not_Sig	no	gi|440903018|gb|ELR53732.1|	3039	0	GTP-binding protein 1, partial [Bos mutus]	gi|742144527|ref|XM_010845114.1|	3577	0	PREDICTED: Bison bison bison GTP binding protein 1 (GTPBP1), transcript variant X1, mRNA	Q58DC5	1171	0	GTP-binding protein 1	SSO2429	322	2.19e-97	COG5258	pfam00009	GTP_EFTU	Elongation factor Tu GTP binding domain. This domain contains a P-loop motif, also found in several other families such as pfam00071,	GO:0061014|positive regulation of mRNA catabolic process;GO:0006414|translational elongation;	GO:0000177|cytoplasmic exosome (RNase complex);GO:0005829|cytosol;GO:0005622|intracellular;GO:0016020|membrane;	GO:0005525|GTP binding;GO:0003924|GTPase activity;GO:0044822	.	.
FBXL3	605.5343	-0.38612	0.128082508	-3.01463	0.0025729	0.021268	Not_Sig	no	gi|466043804|ref|XP_004276893.1|	2233	0	PREDICTED: F-box/LRR-repeat protein 3 [Orcinus orca]	gi|982941417|ref|XM_005213875.3|	3407	0	PREDICTED: Bos taurus F-box and leucine-rich repeat protein 3 (FBXL3), transcript variant X1, mRNA	Q9UKT7	815	0	F-box/LRR-repeat protein 3	.	.	.	.	pfam12937	F-box-like	F-box-like. This is an F-box-like family.	GO:0043153|entrainment of circadian clock by photoperiod;GO:0031648|protein destabilization;GO:0016567|protein ubiquitination;GO:0042752|regulation of circadian rhythm;GO:0048511|rhythmic process;GO:0031146|SCF-dependent proteasomal ubiquitin-dependent protein catabolic process;	GO:0005829|cytosol;GO:0005634|nucleus;GO:0019005|SCF ubiquitin ligase complex;GO:0000151|ubiquitin ligase complex;	GO:0004842|ubiquitin-protein transferase activity;	K10269	FBXL3; F-box and leucine-rich repeat protein 3
TCEAL1	148.0425	-0.53312	0.176932989	-3.01314	0.0025856	0.021357	Not_Sig	no	gi|594047644|ref|XP_006048238.1|	554	4e-62	PREDICTED: transcription elongation factor A protein-like 1 isoform X1 [Bubalus bubalis]	gi|942054161|ref|XM_005895635.2|	1123	0	PREDICTED: Bos mutus transcription elongation factor A (SII)-like 1 (TCEAL1), transcript variant X1, mRNA	Q2KIJ9	169	2e-47	Transcription elongation factor A protein-like 1	.	.	.	.	pfam04538	BEX	Brain expressed X-linked like family. This is a family of transcription elongation factors which includes those referred to as	GO:0006357|regulation of transcription from RNA polymerase II promoter;GO:0006351|transcription, DNA-templated;	GO:0005654|nucleoplasm;GO:0005634|nucleus;	GO:0050699|WW domain binding;	.	.
ZMYND8	868.3158	0.37954	0.12600244	3.012163	0.0025939	0.021391	Not_Sig	no	gi|741947828|ref|XP_010809695.1|	5467	0	PREDICTED: protein kinase C-binding protein 1 isoform X1 [Bos taurus]	gi|982942924|ref|XM_010811393.2|	5223	0	PREDICTED: Bos taurus zinc finger, MYND-type containing 8 (ZMYND8), transcript variant X1, mRNA	Q9ULU4	1834	0	Protein kinase C-binding protein 1	SPAC1952.05	63.2	4.07e-09	COG5076	pfam12064	DUF3544	Domain of unknown function (DUF3544). This presumed domain is functionally uncharacterized.	GO:0098815|modulation of excitatory postsynaptic potential;GO:0060999|positive regulation of dendritic spine development;GO:1902952|positive regulation of dendritic spine maintenance;GO:0051491|positive regulation of filopodium assembly;GO:1902897|regulation of postsynaptic density protein 95 clustering;GO:0006355|regulation of transcription, DNA-templated;	GO:0005737|cytoplasm;GO:0043198|dendritic shaft;GO:0043197|dendritic spine;GO:0005634|nucleus;	GO:0003682|chromatin binding;GO:0042393|histone	.	.
ARHGEF17	1750.208	0.372422	0.123644141	3.012044	0.0025949	0.021391	Not_Sig	no	gi|742163046|ref|XP_010847829.1|	7818	0	PREDICTED: rho guanine nucleotide exchange factor 17 [Bison bison bison]	gi|594082273|ref|XM_006064677.1|	7536	0	PREDICTED: Bubalus bubalis Rho guanine nucleotide exchange factor (GEF) 17 (ARHGEF17), mRNA	Q96PE2	2731	0	Rho guanine nucleotide exchange factor 17	.	.	.	.	pfam00621	RhoGEF	This domain is found in RhoGEF domain. Guanine nucleotide exchange factor for Rho/Rac/Cdc42-like GTPases Also called Dbl-homologous (DH)	GO:0030036|actin cytoskeleton organization;GO:0043065|positive regulation of apoptotic process;GO:0035023|regulation of Rho protein signal transduction;GO:0051056|regulation of small GTPase mediated signal transduction;	GO:0005829|cytosol;	GO:0005085|guanyl-nucleotide exchange factor activity;GO:0005089	.	.
NAT10	703.0804	0.327476	0.108727272	3.011903	0.0025962	0.021391	Not_Sig	no	gi|594077456|ref|XP_006062447.1|	4723	0	PREDICTED: N-acetyltransferase 10 [Bubalus bubalis]	gi|742236977|ref|XM_010831837.1|	3894	0	PREDICTED: Bison bison bison N-acetyltransferase 10 (GCN5-related) (NAT10), mRNA	Q9H0A0	1759	0	RNA cytidine acetyltransferase	YNL132w	986	0	COG1444	pfam13718	GNAT_acetyltr_2	GNAT acetyltransferase 2. This domain has N-acetyltransferase activity. It has a GCN5-related N-acetyltransferase (GNAT) fold.	GO:0032211|negative regulation of telomere maintenance via telomerase;GO:0042274|ribosomal small subunit biogenesis;GO:0000154|rRNA modification;GO:0051391|tRNA acetylation;	GO:0016020|membrane;GO:0000784|nuclear chromosome, telomeric region;GO:0005730|nucleolus;GO:0005654|nucleoplasm;GO:0005634|nucleus;	GO:0005524|ATP binding;GO:0008080|N-acetyltransferase activity;GO:0044822	K14521	NAT10, KRE33; N-acetyltransferase 10 [EC:2.3.1.-]
SPEN	1050.811	0.391095	0.129858804	3.011697	0.0025979	0.021391	Not_Sig	no	gi|297484241|ref|XP_002694185.1|	15386	0	PREDICTED: msx2-interacting protein isoform X1 [Bos taurus]	gi|982996864|ref|XM_591419.8|	11960	0	PREDICTED: Bos taurus spen family transcriptional repressor (SPEN), transcript variant X1, mRNA	Q96T58	3754	0	Msx2-interacting protein	.	.	.	.	pfam07744	SPOC	SPOC domain. The SPOC (Spen paralogue and orthologue C-terminal) domain is involved in developmental signalling.	GO:0000398|mRNA splicing, via spliceosome;GO:0000122|negative regulation of transcription from RNA polymerase II promoter;GO:0045892|negative regulation of transcription, DNA-templated;GO:0007219|Notch signaling pathway;GO:0050769|positive regulation of neurogenesis;GO:0006351|transcription, DNA-templated;GO:0016032|viral process;	GO:0070062|extracellular exosome;GO:0005654|nucleoplasm;GO:0005634|nucleus;GO:0017053|transcriptional repressor complex;	GO:0003677|DNA binding;GO:0003676|nucleic acid binding;GO:0000166	.	.
BSCL2	437.6044	0.412791	0.137068283	3.011574	0.002599	0.021391	Not_Sig	no	gi|742179025|ref|XP_010852297.1|	2275	0	PREDICTED: seipin isoform X1 [Bison bison bison]	gi|594081972|ref|XM_006064535.1|	1957	0	PREDICTED: Bubalus bubalis Berardinelli-Seip congenital lipodystrophy 2 (seipin) (BSCL2), transcript variant X8, mRNA	Q5E9P6	739	0	Seipin	.	.	.	.	pfam06775	Seipin	Putative adipose-regulatory protein (Seipin). Seipin is a protein of approximately 400 residues, in humans, which is the product of a	GO:0045444|fat cell differentiation;GO:0016042|lipid catabolic process;GO:0034389|lipid particle organization;GO:0019915|lipid storage;GO:0050995|negative regulation of lipid catabolic process;	GO:0030176|integral component of endoplasmic reticulum membrane;	.	K19365	BSCL2; seipin
DAP	2313.016	0.34088	0.113244142	3.010131	0.0026113	0.021472	Not_Sig	no	gi|594071961|ref|XP_006059826.1|	463	2e-51	PREDICTED: death-associated protein 1 [Bubalus bubalis]	gi|402693023|ref|NM_001078058.2|	2115	0	Bos taurus death-associated protein (DAP), mRNA	Q5EAE6	182	8e-53	Death-associated protein 1	.	.	.	.	pfam15228	DAP	Death-associated protein.	GO:0097190|apoptotic signaling pathway;GO:0006914|autophagy;GO:0034198|cellular response to amino acid starvation;GO:0010507|negative regulation of autophagy;	.	GO:0070513|death domain binding;	.	.
CREB3L1	5728.252	0.456059	0.151520527	3.009883	0.0026135	0.021472	Not_Sig	no	gi|555973955|ref|XP_005898973.1|	2320	0	PREDICTED: cyclic AMP-responsive element-binding protein 3-like protein 1 [Bos mutus]	gi|965802035|ref|XM_012116870.2|	2458	0	PREDICTED: Ovis aries musimon cAMP responsive element binding protein 3-like 1 (CREB3L1), transcript variant X2, mRNA	Q96BA8	838	0	Cyclic AMP-responsive element-binding protein 3-like protein 1	.	.	.	.	pfam00170	bZIP_1	bZIP transcription factor. The Pfam entry includes the basic region and the leucine zipper region.	GO:0030968|endoplasmic reticulum unfolded protein response;GO:0070278|extracellular matrix constituent secretion;GO:0007275|multicellular organismal development;GO:1902236|negative regulation of endoplasmic reticulum stress-induced intrinsic apoptotic signaling pathway;GO:0001649|osteoblast differentiation;GO:1990440|positive regulation of transcription from RNA polymerase II promoter in response to endoplasmic reticulum stress;	GO:0005783|endoplasmic reticulum;GO:0005789|endoplasmic reticulum membrane;GO:0016021|integral component of	GO:0035497|cAMP response element binding;GO:0003682|chromatin	K09048	CREB3; cyclic AMP-responsive element-binding protein 3
SRP14	992.0054	-0.41094	0.136549674	-3.00947	0.0026171	0.021472	Not_Sig	no	gi|594112549|ref|XP_006079061.1|	538	2e-65	PREDICTED: signal recognition particle 14 kDa protein-like [Bubalus bubalis]	gi|942035910|ref|XM_005888932.2|	885	0	PREDICTED: Bos mutus signal recognition particle 14kDa (homologous Alu RNA binding protein) (SRP14), mRNA	A6QQL9	196	4e-61	Signal recognition particle 14 kDa protein	.	.	.	.	pfam02290	SRP14	Signal recognition particle 14kD protein. The signal recognition particle (SRP) is a multimeric protein involved in targeting	GO:0045047|protein targeting to ER;GO:0042493|response to drug;GO:0006614|SRP-dependent cotranslational protein targeting to membrane;	GO:0070062|extracellular exosome;GO:0005634|nucleus;GO:0005786|signal recognition particle, endoplasmic reticulum targeting;	GO:0008312|7S RNA binding;GO:0030942|endoplasmic	K03104	SRP14; signal recognition particle subunit SRP14
SYNGAP1	294.6949	0.480037	0.159526214	3.009143	0.0026199	0.021472	Not_Sig	no	gi|982963065|ref|XP_015315221.1|	5862	0	PREDICTED: ras/Rap GTPase-activating protein SynGAP isoform X3 [Bos taurus]	gi|982963064|ref|XM_015459735.1|	4794	0	PREDICTED: Bos taurus synaptic Ras GTPase activating protein 1 (SYNGAP1), transcript variant X3, mRNA	F6SEU4	2227	0	Ras/Rap GTPase-activating protein SynGAP	.	.	.	.	pfam12004	DUF3498	Domain of unknown function (DUF3498). This presumed domain is functionally uncharacterized.	GO:0016358|dendrite development;GO:0050771|negative regulation of axonogenesis;GO:0043524|negative regulation of neuron apoptotic process;GO:0046580|negative regulation of Ras protein signal transduction;GO:0007389|pattern specification process;GO:0007265|Ras protein signal transduction;GO:0043113|receptor clustering;GO:0048169|regulation of long-term neuronal synaptic plasticity;GO:0043408|regulation of MAPK cascade;GO:0050803|regulation of synapse structure or activity;GO:0048167|regulation of synaptic	GO:0030054|cell junction;GO:0005737|cytoplasm;GO:0043198|dendritic shaft;GO:0031235|intrinsic component of the cytoplasmic side of the	GO:0005096|GTPase activator activity;	K17631	SYNGAP, RASA5; Ras/Rap GTPase-activating protein SynGAP
MED21	173.1717	-0.48096	0.159834003	-3.00914	0.0026199	0.021472	Not_Sig	no	gi|440892234|gb|ELR45514.1|	1170	3e-144	Transposon TX1 uncharacterized 149 kDa protein, partial [Bos mutus]	gi|850480667|gb|CP011888.1|	1618	0	Ovis canadensis canadensis isolate 43U chromosome 3 sequence	Q2TBU8	291	7e-89	Mediator of RNA polymerase II transcription subunit 21	.	.	.	.	pfam11221	Med21	This domain is found in Subunit 21 of Mediator complex. Med21 has been known as Srb7 in yeasts, hSrb7 in humans and Trap 19 in Drosophila. The heterodimer	GO:0001824|blastocyst development;GO:0045944|positive regulation of transcription from RNA polymerase II promoter;GO:0019827|stem cell population maintenance;GO:0006351|transcription, DNA-templated;	GO:0016592|mediator complex;	GO:0001104|RNA polymerase II transcription cofactor	K15152	MED21, SRB7; mediator of RNA polymerase II transcription subunit
SIX5	200.2591	0.57067	0.189654766	3.008994	0.0026211	0.021472	Not_Sig	no	gi|329664814|ref|NP_001192695.1|	2506	0	homeobox protein SIX5 [Bos taurus]	gi|803247006|ref|XM_012128115.1|	3006	0	PREDICTED: Ovis aries musimon SIX homeobox 5 (SIX5), transcript variant X2, mRNA	Q8N196	899	0	Homeobox protein SIX5	.	.	.	.	pfam00046	Homeobox	Homeobox domain.	GO:0002088|lens development in camera-type eye;GO:1902723|negative regulation of skeletal muscle satellite cell proliferation;GO:0045892|negative regulation of transcription, DNA-templated;GO:0045944|positive regulation of transcription from RNA polymerase II promoter;GO:0007286|spermatid development;GO:0006351|transcription, DNA-templated;	GO:0005737|cytoplasm;GO:0005794|Golgi apparatus;GO:0043231|intracellular membrane-bounded organelle;GO:0005654|nucleoplasm;	GO:0000978|RNA polymerase II core promoter proximal region sequence-	K19474	SIX5; homeobox protein SIX5
AUTS2	160.6019	0.605598	0.201267733	3.008916	0.0026218	0.021472	UP	yes	gi|296473231|tpg|DAA15346.1|	3355	0	TPA: autism susceptibility candidate 2 [Bos taurus]	gi|694921463|ref|XM_519135.4|	3323	0	PREDICTED: Pan troglodytes autism susceptibility candidate 2 (AUTS2), transcript variant X3, mRNA	Q8WXX7	1201	0	Autism susceptibility gene 2 protein	.	.	.	.	pfam15336	Auts2	Autism susceptibility gene 2 protein. Auts2, or FBRSL2, Fibrosin-1-like protein 2, is a family of eukaryotic proteins associated	GO:0098582|innate vocalization behavior;GO:0051571|positive regulation of histone H3-K4 methylation;GO:2000620|positive regulation of histone H4-K16 acetylation;GO:0045944|positive regulation of transcription from RNA polymerase II promoter;GO:0060013|righting reflex;	GO:0005634|nucleus;	GO:0003682|chromatin binding;	.	.

BCAM	4606.52	0.39771	0.132209332	3.008186	0.0026281	0.021502	Not_Sig	no	gi|555976888|ref|XP_005900403.1|	2653	0	PREDICTED: basal cell adhesion molecule [Bos mutus]	gi|133778182|gb|BC123496.1|	2339	0	Bos taurus basal cell adhesion molecule (Lutheran blood group), mRNA (cDNA clone MGC:139914 IMAGE:8284754), complete cds	Q9MZ08	1021	0	Basal cell adhesion molecule	.	.	.	.	pfam08205	C2-set_2	CD80-like C2-set immunoglobulin domain. These domains belong to the immunoglobulin superfamily.	GO:0007160|cell-matrix adhesion;	GO:0009986|cell surface;GO:0070062|extracellular exosome;GO:0016021|integral component of membrane;GO:0005886|plasma	GO:0005055|laminin receptor activity;	K06578	LU; Lutheran blood group glycoprotein
ATP5F1E	754.6175	-0.52661	0.175065725	-3.00805	0.0026293	0.021502	Not_Sig	no	gi|296475007|tpg|DAA17122.1|	362	8e-42	TPA: ATP synthase, H+ transporting, mitochondrial F1 complex, epsilon subunit-like [Bos taurus]	gi|219522069|ref|NM_001143741.1|	443	0	Bos taurus ATP synthase, H+ transporting, mitochondrial F1 complex, epsilon subunit (ATP5E), mRNA	P05632	105	2e-28	ATP synthase subunit epsilon, mitochondrial	.	.	.	.	pfam04627	ATP-synt_Eps	Mitochondrial ATP synthase epsilon chain. This family constitutes the mitochondrial ATP synthase epsilon subunit. This is	GO:0015986|ATP synthesis coupled proton transport;	GO:0005743|mitochondrial inner membrane;GO:0005753|mitochondrial proton-transporting ATP synthase complex;GO:0000275|mitochondrial	GO:0046933|proton-transporting ATP synthase activity, rotational	K02135	ATPeF1E, ATP5E, ATP15; F-type H+-transporting ATPase subunit epsilon
LAMTOR5	329.4144	-0.49868	0.165810372	-3.00754	0.0026337	0.021523	Not_Sig	no	gi|926687870|ref|XP_005677982.2|	520	5e-64	PREDICTED: ragulator complex protein LAMTOR5 [Capra hircus]	gi|942042235|ref|XM_005891278.2|	608	0	PREDICTED: Bos mutus late endosomal/lysosomal adaptor, MAPK and MTOR activator 5 (LAMTOR5), transcript variant X2, mRNA	Q66X52	182	3e-57	Ragulator complex protein LAMTOR5	.	.	.	.	pfam14455	Metal_CEHH	Predicted metal binding domain. A predicted metal-binding domain that is found in gene-neighborhood associations with	GO:0071230|cellular response to amino acid stimulus;GO:0043154|negative regulation of cysteine-type endopeptidase activity involved in apoptotic process;GO:0043547|positive regulation of GTPase activity;GO:0032008|positive regulation of TOR signaling;GO:0061462|protein localization to lysosome;GO:0008361|regulation of cell size;GO:0019079|viral genome replication;	GO:0005764|lysosome;GO:0071986|Ragulator complex;	.	K16344	HBXIP; hepatitis B virus X-interacting protein
WSB1	9564.477	-0.26752	0.088964639	-3.00707	0.0026378	0.021528	Not_Sig	no	gi|358417242|ref|XP_885171.4|	2194	0	PREDICTED: WD repeat and SOCS box-containing protein 1 [Bos taurus]	gi|942056950|ref|XM_005896680.2|	2516	0	PREDICTED: Bos mutus WD repeat and SOCS box containing 1 (WSB1), transcript variant X1, mRNA	Q9Y6I7	838	0	WD repeat and SOCS box-containing protein 1	.	.	.	.	pfam07525	SOCS_box	SOCS box. The SOCS box acts as a bridge between specific substrate-binding domains and more generic proteins that comprise a large	GO:0035556|intracellular signal transduction;GO:0000209|protein polyubiquitination;	GO:0005829|cytosol;GO:0005622|intracellular;	GO:0004842|ubiquitin-protein transferase activity;	K10341	WSB1; WD repeat and SOCS box-containing protein 1
PCMTD1	835.3825	-0.32929	0.109505636	-3.00704	0.002638	0.021528	Not_Sig	no	gi|301768463|ref|XP_002919648.1|	1631	0	PREDICTED: protein-L-isoaspartate O-methyltransferase domain-containing protein 1 isoform X1 [Ailuropoda melanoleuca]	gi|528975769|ref|XM_005215410.1|	3884	0	PREDICTED: Bos taurus protein-L-isoaspartate (D-aspartate) O-methyltransferase domain containing 1 (PCMTD1), transcript variant X2, mRNA	A2VDP2	631	0	Protein-L-isoaspartate O-methyltransferase domain-containing protein 1	MTH827	89	6.01e-19	COG2518	pfam01135	PCMT	Protein-L-isoaspartate(D-aspartate) O-methyltransferase (PCMT).	.	GO:0005737|cytoplasm;GO:0016020|membrane;	GO:0004719|protein-L-isoaspartate (D-aspartate) O-methyltransferase	.	.
CRIP1	1386.494	-0.31816	0.105841217	-3.00597	0.0026474	0.021588	Not_Sig	no	gi|830027483|ref|XP_004681612.2|	431	2e-51	PREDICTED: cysteine-rich protein 1 [Condylura cristata]	gi|965907020|ref|XM_015090495.1|	422	0	PREDICTED: Ovis aries musimon cysteine-rich protein 1 (LOC105605869), transcript variant X2, mRNA	Q56K04	160	1e-49	Cysteine-rich protein 1	.	.	.	.	pfam00412	LIM	LIM domain. This family represents two copies of the LIM structural domain.	GO:0071236|cellular response to antibiotic;GO:0071493|cellular response to UV-B;GO:0008630|intrinsic apoptotic signaling pathway in response to DNA damage;GO:0010043|response to zinc ion;	GO:0005737|cytoplasm;	GO:0003680|AT DNA binding;GO:0008301|DNA binding,	.	.
SIGMAR1	912.4098	0.380633	0.126683532	3.004599	0.0026593	0.021671	Not_Sig	no	gi|803311411|ref|XP_012014033.1|	1006	3e-131	PREDICTED: sigma non-opioid intracellular receptor 1 isoform X1 [Ovis aries musimon]	gi|942076527|ref|XM_005903045.2|	1604	0	PREDICTED: Bos mutus sigma non-opioid intracellular receptor 1 (SIGMAR1), transcript variant X1, mRNA	Q58DH7	392	1e-132	Sigma non-opioid intracellular receptor 1	.	.	.	.	pfam04622	ERG2_Sigma1R	ERG2 and Sigma1 receptor like protein. This family consists of the fungal C-8 sterol isomerase and mammalian sigma1 receptor. C-8	GO:0006869|lipid transport;GO:0043523|regulation of neuron apoptotic process;	GO:0030054|cell junction;GO:0016023|cytoplasmic membrane-bounded vesicle;GO:0005789|endoplasmic reticulum	GO:0004872|receptor activity;	.	.
NT5DC2	1340.287	0.319331	0.106293984	3.004222	0.0026626	0.021682	Not_Sig	no	gi|359078268|ref|XP_003587683.1|	2576	0	PREDICTED: 5'-nucleotidase domain-containing protein 2 isoform X1 [Bos taurus]	gi|983004663|ref|XM_003583808.3|	1774	0	PREDICTED: Bos taurus 5'-nucleotidase domain containing 2 (NT5DC2), transcript variant X1, mRNA	Q6Q0N3	969	0	5'-nucleotidase domain-containing protein 2	.	.	.	.	pfam05761	5_nucleotid	5' nucleotidase family. This family of eukaryotic proteins includes 5' nucleotidase enzymes, such as purine 5'-nucleotidase EC:3.1.3.5.	.	.	GO:0008253|5'-nucleotidase activity;GO:0046872|metal ion binding;	.	.
PAPLN	50.14642	-0.89402	0.297961511	-3.00046	0.0026957	0.021936	DOWN	yes	gi|594062426|ref|XP_006055269.1|	6209	0	PREDICTED: papilin isoform X1 [Bubalus bubalis]	gi|982990057|ref|XM_015465221.1|	4295	0	PREDICTED: Bos taurus papilin, proteoglycan-like sulfated glycoprotein (PAPLN), transcript variant X2, mRNA	O95428	1916	0	Papilin	.	.	.	.	pfam05986	ADAM_spacer1	ADAM-TS Spacer 1. This family represents the Spacer-1 region from the ADAM-TS family of metalloproteinases.	.	GO:0005578|proteinaceous extracellular matrix;	GO:0004222|metalloendopeptidase activity;GO:0004867|serine-type	.	.
RAB2A	2958.305	-0.30244	0.100813688	-2.99998	0.0027	0.021955	Not_Sig	no	gi|528975876|ref|XP_005215510.1|	1013	7e-130	PREDICTED: ras-related protein Rab-2A isoform X4 [Bos taurus]	gi|528975873|ref|XM_005215452.1|	2043	0	PREDICTED: Bos taurus RAB2A, member RAS oncogene family (RAB2A), transcript variant X3, mRNA	Q5R6B6	383	6e-127	Ras-related protein Rab-2A	.	.	.	.	pfam00071	Ras	Ras family. Includes sub-families Ras, Rab, Rac, Ral, Ran, Rap Ypt1 and more. Shares P-loop motif with GTP_EFTU, arf and	GO:0007030|Golgi organization;GO:0015031|protein transport;GO:0007264|small GTPase mediated signal transduction;GO:0016192|vesicle-mediated transport;	GO:0005789|endoplasmic reticulum membrane;GO:0033116|endoplasmic reticulum-Golgi intermediate compartment membrane;GO:0070062|extracellular	GO:0019003|GDP binding;GO:0005525|GTP binding;GO:0003924	K07877	RAB2A; Ras-related protein Rab-2A
RAD23A	816.5258	0.38957	0.129897291	2.99906	0.0027081	0.022006	Not_Sig	no	gi|548471923|ref|XP_005682329.1|	1389	0	PREDICTED: UV excision repair protein RAD23 homolog A [Capra hircus]	gi|528954958|ref|XM_005208675.1|	1857	0	PREDICTED: Bos taurus RAD23 homolog A, nucleotide excision repair protein (RAD23A), transcript variant X1, mRNA	A3KMV2	532	0	UV excision repair protein RAD23 homolog A	SPBC2D10.12	121	3.40e-29	COG5272	pfam09280	XPC-binding	XPC-binding domain. Members of this family adopt a structure consisting of four alpha helices, arranged in an array. They bind	GO:0006289|nucleotide-excision repair;GO:0045070|positive regulation of viral genome replication;GO:0043161|proteasome-mediated ubiquitin-dependent protein catabolic process;GO:0032434|regulation of proteasomal ubiquitin-dependent protein catabolic process;	GO:0005634|nucleus;GO:0000502|proteasome complex;	GO:0003684|damaged DNA binding;	K10839	RAD23, HR23; UV excision repair protein RAD23
ZNF575	81.94198	0.719317	0.239939194	2.997914	0.0027183	0.022059	UP	yes	gi|528992080|ref|XP_005219241.1|	1314	2e-178	PREDICTED: zinc finger protein 575 isoform X1 [Bos taurus]	gi|982953380|ref|XM_005219185.3|	1253	0	PREDICTED: Bos taurus zinc finger protein 575 (ZNF575), transcript variant X2, mRNA	Q86XF7	363	5e-123	Zinc finger protein 575	.	.	.	.	pfam09770	PAT1	Topoisomerase II-associated protein PAT1. Members of this family are necessary for accurate chromosome transmission during	GO:0006355|regulation of transcription, DNA-templated;GO:0006351|transcription, DNA-templated;	GO:0005634|nucleus;	GO:0003677|DNA binding;GO:0046872|metal ion binding;	.	.
MEF2A	1141.871	-0.32193	0.107386571	-2.9979	0.0027185	0.022059	Not_Sig	no	gi|528998710|ref|XP_005221769.1|	2005	0	PREDICTED: myocyte-specific enhancer factor 2A isoform X1 [Bos taurus]	gi|982959831|ref|XM_005221712.3|	5548	0	PREDICTED: Bos taurus myocyte enhancer factor 2A (MEF2A), transcript variant X1, mRNA	A2VDZ3	769	0	Myocyte-specific enhancer factor 2A	YPL089c	92.4	6.95e-18	COG5068	pfam12347	HJURP_C	Holliday junction regulator protein family C-terminal repeat. Although this family is conserved in the Holliday junction regulator, HJURP,	GO:0006915|apoptotic process;GO:0061337|cardiac conduction;GO:0071277|cellular response to calcium ion;GO:0048813|dendrite morphogenesis;GO:0070375|ERK5 cascade;GO:0000165|MAPK cascade;GO:0000002|mitochondrial genome maintenance;GO:0048311|mitochondrion distribution;GO:0000122|negative regulation of transcription from RNA polymerase II promoter;GO:0045944|positive regulation of transcription from RNA polymerase II promoter;GO:0055005|ventricular cardiac myofibril	GO:0005737|cytoplasm;GO:0000790|nuclear chromatin;GO:0005654|nucleoplasm;GO:0005634|nucleus;GO:0005667|transcription	GO:0033613|activating transcription factor binding;GO:0003682	K09260	MEF2A; MADS-box transcription enhancer factor 2A
RPL37	3094.747	-0.43628	0.145559086	-2.99725	0.0027243	0.022075	Not_Sig	no	gi|594059539|ref|XP_006080949.1|	476	2e-58	PREDICTED: LOW QUALITY PROTEIN: ribosomal protein L37 [Bubalus bubalis]	gi|942038271|ref|XM_005889715.2|	490	0	PREDICTED: Bos mutus ribosomal protein L37 (RPL37), mRNA	P61928	177	4e-56	60S ribosomal protein L37	YLR185w	117	3.74e-34	COG2126	pfam01907	Ribosomal_L37e	Ribosomal protein L37e. This family includes ribosomal protein L37 from eukaryotes and archaebacteria. The family	GO:0006412|translation;	GO:0022625|cytosolic large ribosomal subunit;	GO:0019838|growth factor binding;GO:0046872|metal ion	K02922	RP-L37e, RPL37; large subunit ribosomal protein L37e
ALDH16A1	652.1539	0.370544	0.123629699	2.997212	0.0027246	0.022075	Not_Sig	no	gi|742163330|ref|XP_010847914.1|	3875	0	PREDICTED: LOW QUALITY PROTEIN: aldehyde dehydrogenase family 16 member A1 [Bison bison bison]	gi|742163329|ref|XM_010849612.1|	2877	0	PREDICTED: Bison bison bison aldehyde dehydrogenase 16 family, member A1 (ALDH16A1), mRNA	A6QR56	1479	0	Aldehyde dehydrogenase family 16 member A1	.	.	.	.	pfam00171	Aldedh	Aldehyde dehydrogenase family. This family of dehydrogenases act on aldehyde substrates. Members use NADP as a cofactor. The family	.	.	GO:0047105|4-trimethylammoniobutyraldehyde dehydrogenase	.	.
UBE3B	754.17	0.341929	0.114089535	2.997027	0.0027263	0.022075	Not_Sig	no	gi|329664448|ref|NP_001193161.1|	5624	0	ubiquitin-protein ligase E3B [Bos taurus]	gi|982950255|ref|XM_005217809.3|	5400	0	PREDICTED: Bos taurus ubiquitin protein ligase E3B (UBE3B), transcript variant X3, mRNA	Q7Z3V4	2095	0	Ubiquitin-protein ligase E3B	SPAC167.07c	302	2.56e-84	COG5021	pfam00632	HECT	HECT-domain (ubiquitin-transferase). The name HECT comes from Homologous to the E6-AP Carboxyl Terminus.	GO:0042787|protein ubiquitination involved in ubiquitin-dependent protein catabolic process;	GO:0005737|cytoplasm;GO:0005634|nucleus;	GO:0004842|ubiquitin-protein transferase activity;	K10588	UBE3B; ubiquitin-protein ligase E3 B [EC:2.3.2.26]
LOC138990481	120.8606	-0.65794	0.219687042	-2.99489	0.0027454	0.022198	DOWN	yes	.	.	.	.	gi|742094014|ref|XM_010834218.1|	412	0	PREDICTED: Bison bison bison McKusick-Kaufman syndrome (MKKS), transcript variant X2, mRNA	.	.	.	.	.	.	.	.	pfam09500	YiiD_Cterm	Putative thioesterase (yiiD_Cterm). This entry consists of a broadly distributed uncharacterized domain often found as a	.	.	.	.	.
WDR18	574.9162	0.413144	0.137949879	2.994884	0.0027455	0.022198	Not_Sig	no	gi|77736123|ref|NP_001029760.1|	2122	0	WD repeat-containing protein 18 [Bos taurus]	gi|742142361|ref|XM_010844585.1|	3595	0	PREDICTED: Bison bison bison WD repeat domain 18 (WDR18), mRNA	Q3SZD4	822	0	WD repeat-containing protein 18	SPAC13G7.08c	87	7.59e-17	COG2319	pfam14077	WD40_alt	Alternative WD40 repeat motif. WD repeats are short subdomains of about 40 amino acids and fold into 4 antiparallel beta hairpins.	GO:0007275|multicellular organismal development;	GO:0005737|cytoplasm;GO:0005654|nucleoplasm;	.	K14829	IPI3; pre-rRNA-processing protein IPI3
TRAPPC9	193.0942	0.512579	0.171162368	2.994692	0.0027472	0.022198	Not_Sig	no	gi|982944162|ref|XP_015329839.1|	5984	0	PREDICTED: trafficking protein particle complex subunit 9 isoform X5 [Bos taurus]	gi|982944161|ref|XM_015474353.1|	4122	0	PREDICTED: Bos taurus trafficking protein particle complex 9 (TRAPPC9), transcript variant X7, mRNA	Q32PH0	2262	0	Trafficking protein particle complex subunit 9	.	.	.	.	pfam08626	TRAPPC9-Trs120	Transport protein Trs120 or TRAPPC9, TRAPP II complex subunit. This region is found at the N terminal of Saccharomyces	GO:0030154|cell differentiation;	GO:0005783|endoplasmic reticulum;GO:0005802|trans-Golgi network;	.	.	.
GEMIN2	109.0157	-0.61072	0.204031568	-2.99325	0.0027602	0.022287	DOWN	yes	gi|358417992|ref|XP_003583807.1|	1450	0	PREDICTED: gem-associated protein 2 [Bos taurus]	gi|942074187|ref|XM_005902288.2|	1197	0	PREDICTED: Bos mutus gem (nuclear organelle) associated protein 2 (GEMIN2), transcript variant X1, mRNA	Q9CQQ4	523	0	Gem-associated protein 2	.	.	.	.	pfam04938	SIP1	Survival motor neuron (SMN) interacting protein 1 (SIP1). Survival motor neuron (SMN) interacting protein 1 (SIP1)	GO:0000245|spliceosomal complex assembly;GO:0000387|spliceosomal snRNP assembly;	GO:0005737|cytoplasm;GO:0005829|cytosol;GO:0097504|Gemini of coiled bodies;GO:0005622|intracellular;GO:0005730|nucleolus;GO:0005634|nucleus;GO:0032	.	K13130	SIP1, GEMIN2; survival of motor neuron protein-interacting protein 1
AZIN1	3202.969	-0.31906	0.106615396	-2.99263	0.0027659	0.022317	Not_Sig	no	gi|129277532|ref|NP_001076080.1|	2402	0	antizyme inhibitor 1 [Bos taurus]	gi|619328998|ref|NM_001291727.1|	4213	0	Bubalus bubalis antizyme inhibitor 1 (AZIN1), transcript variant 1, mRNA	O14977	885	0	Antizyme inhibitor 1	SPAC144.04c	298	7.28e-89	COG0019	pfam02784	Orn_Arg_deC_N	Pyridoxal-dependent decarboxylase, pyridoxal binding domain. These pyridoxal-dependent decarboxylases acting	GO:0042177|negative regulation of protein catabolic process;GO:1902269|positive regulation of polyamine transmembrane transport;GO:0033387|putrescine biosynthetic process from ornithine;GO:0006521|regulation of cellular amino acid metabolic process;	GO:0005737|cytoplasm;GO:0005829|cytosol;GO:0005634|nucleus;	GO:0003824|catalytic activity;GO:0042978|ornithine	.	.
PRKAB2	615.0784	-0.37768	0.126232239	-2.99195	0.002772	0.022335	Not_Sig	no	gi|332248219|ref|XP_003273262.1|	1260	3e-156	PREDICTED: 5'-AMP-activated protein kinase subunit beta-2 [Nomascus leucogenys]	gi|982916755|ref|XM_005203986.3|	5397	0	PREDICTED: Bos taurus protein kinase, AMP-activated, beta 2 non-catalytic subunit (PRKAB2), transcript variant X1, mRNA	O43741	488	4e-157	5'-AMP-activated protein kinase subunit beta-2	.	.	.	.	pfam04739	AMPKBI	5'-AMP-activated protein kinase beta subunit, interation domain. This region is found in the beta subunit of the 5'-AMP-activated	GO:0006853|carnitine shuttle;GO:0007050|cell cycle arrest;GO:0006633|fatty acid biosynthetic process;GO:0016236|macroautophagy;GO:0006468|protein phosphorylation;GO:0042304|regulation of fatty acid biosynthetic process;GO:0045859|regulation of protein kinase activity;GO:1901796|regulation of signal transduction by p53 class mediator;GO:0007165|signal transduction;	GO:0005829|cytosol;GO:0005654|nucleoplasm;GO:0031588|nucleotide-activated protein kinase complex;	.	K07199	PRKAB; 5'-AMP-activated protein kinase, regulatory beta subunit
ZER1	393.6965	0.500989	0.167445528	2.991953	0.002772	0.022335	Not_Sig	no	gi|440894679|gb|ELR47079.1|	3977	0	Protein zer-1-like protein, partial [Bos mutus]	gi|982939925|ref|XM_005213354.3|	4067	0	PREDICTED: Bos taurus zyg-11 related, cell cycle regulator (ZER1), transcript variant X1, mRNA	Q7Z7L7	1505	0	Protein zer-1 homolog	.	.	.	.	pfam09770	PAT1	Topoisomerase II-associated protein PAT1. Members of this family are necessary for accurate chromosome transmission during	GO:0051438|regulation of ubiquitin-protein transferase activity;	GO:0031462|Cul2-RING ubiquitin ligase complex;	GO:0004842|ubiquitin-protein transferase activity;	K10350	ZYG11; Zyg-11 protein homolog
NOP16	477.6499	-0.38097	0.127393575	-2.99052	0.002785	0.022412	Not_Sig	no	gi|62460412|ref|NP_001014856.1|	851	9e-113	nucleolar protein 16 [Bos taurus]	gi|742152692|ref|XM_010846592.1|	753	0	PREDICTED: Bison bison bison NOP16 nucleolar protein (NOP16), mRNA	Q5E996	332	3e-114	Nucleolar protein 16	.	.	.	.	pfam09420	Nop16	Ribosome biogenesis protein Nop16. Nop16 is a protein involved in ribosome biogenesis.	GO:0042273|ribosomal large subunit biogenesis;	GO:0005730|nucleolus;	GO:0044822|poly(A) RNA binding;	.	.
MDGA1	72.40759	0.754103	0.252168492	2.990473	0.0027855	0.022412	UP	yes	gi|300797902|ref|NP_001179811.1|	4946	0	MAM domain-containing glycosylphosphatidylinositol anchor protein 1 precursor [Bos taurus]	gi|594114319|ref|XM_006079824.1|	3612	0	PREDICTED: Bubalus bubalis MAM domain containing glycosylphosphatidylinositol anchor 1 (MDGA1), transcript variant X2, mRNA	Q8NFP4	1868	0	MAM domain-containing glycosylphosphatidylinositol anchor protein 1	.	.	.	.	pfam00629	MAM	MAM domain. An extracellular domain found in many receptors.	GO:0007420|brain development;GO:0001764|neuron migration;GO:0021527|spinal cord association neuron differentiation;	GO:0046658|anchored component of plasma membrane;GO:0005615|extracellular space;GO:0005794|Golgi	.	.	.
RCN2	784.5484	-0.31904	0.106697789	-2.99015	0.0027884	0.022415	Not_Sig	no	gi|115497628|ref|NP_001069047.1|	1542	0	reticulocalbin-2 precursor [Bos taurus]	gi|115497627|ref|NM_001075579.1|	1787	0	Bos taurus reticulocalbin 2 (RCN2), mRNA	Q62703	542	0	Reticulocalbin-2	.	.	.	.	pfam13499	EF-hand_7	EF-hand domain pair.	.	GO:0005788|endoplasmic reticulum lumen;GO:0005730|nucleolus;	GO:0005509|calcium ion binding;	.	.
DXO	200.6526	0.484201	0.161939701	2.990008	0.0027897	0.022415	Not_Sig	no	gi|742090415|ref|XP_010831649.1|	2043	0	PREDICTED: decapping and exoribonuclease protein [Bison bison bison]	gi|942049414|ref|XM_014477569.1|	1280	0	PREDICTED: Bos mutus decapping exoribonuclease (DXO), transcript variant X2, mRNA	Q5E9Y5	791	0	Decapping and exoribonuclease protein	.	.	.	.	pfam08652	RAI1	RAI1 like PD-(D/E)XK nuclease. RAI1 is homologous to Caenorhabditis elegans DOM-3 and human DOM3Z and binds to a	GO:0008152|metabolic process;GO:0006402|mRNA catabolic process;GO:0071028|nuclear mRNA surveillance;GO:0090305|nucleic acid phosphodiester bond hydrolysis;GO:0050779|RNA destabilization;	GO:0005634|nucleus;	GO:0008409|5'-3' exonuclease activity;GO:0000287|magnesium ion	K14845	RAI1, DOM3Z; RAT1-interacting protein
SSBP4	245.8778	0.487413	0.163025059	2.989803	0.0027916	0.022415	Not_Sig	no	gi|803248247|ref|XP_011984095.1|	1535	0	PREDICTED: single-stranded DNA-binding protein 4 isoform X3 [Ovis aries musimon]	gi|821403049|ref|XM_012535736.1|	899	0	PREDICTED: Orcinus orca single stranded DNA binding protein 4 (SSBP4), transcript variant X1, mRNA	Q9BWG4	530	0	Single-stranded DNA-binding protein 4	.	.	.	.	pfam04503	SSDP	Single-stranded DNA binding protein, SSDP. This is a family of eukaryotic single-stranded DNA binding proteins with specificity to	.	GO:0005634|nucleus;	GO:0003697|single-stranded DNA binding;	.	.
MED11	281.5314	-0.48056	0.160762762	-2.98925	0.0027966	0.022423	Not_Sig	no	gi|741962649|ref|XP_010814215.1|	649	7e-80	PREDICTED: mediator of RNA polymerase II transcription subunit 11 isoform X1 [Bos taurus]	gi|594072694|ref|XM_006060115.1|	1093	0	PREDICTED: Bubalus bubalis mediator complex subunit 11 (MED11), mRNA	Q32L03	228	2e-71	Mediator of RNA polymerase II transcription subunit 11	.	.	.	.	pfam10280	Med11	Mediator complex protein. Mediator is a large, modular protein complex that is conserved from yeast to human and conveys	GO:0006351|transcription, DNA-templated;	GO:0016592|mediator complex;	GO:0001104|RNA polymerase II transcription cofactor activity;	K15131	MED11; mediator of RNA polymerase II transcription subunit 11
LOC138986330	518.1355	-0.39384	0.13175052	-2.98925	0.0027966	0.022423	Not_Sig	no	gi|358420079|ref|XP_003584417.1|	2490	0	PREDICTED: eukaryotic translation initiation factor 2 subunit 3, Y-linked [Bos taurus]	gi|983012001|ref|XM_003584369.4|	1767	0	PREDICTED: Bos taurus eukaryotic translation initiation factor 2, subunit 3, structural gene Y-linked (EIF2S3Y), mRNA	P41091	931	0	Eukaryotic translation initiation factor 2 subunit 3	SPBC17G9.09	705	0	COG5257	pfam00009	GTP_EFTU	Elongation factor Tu GTP binding domain. This domain contains a P-loop motif, also found in several other families such as pfam00071,	GO:0001731|formation of translation preinitiation complex;GO:0006413|translational initiation;GO:0055085|transmembrane transport;	GO:0005913|cell-cell adherens junction;GO:0005737|cytoplasm;GO:0005829|cytosol;GO:0070062|extracellular exosome;	GO:0098641|cadherin binding involved in cell-cell adhesion;GO:00055	K03242	EIF2S3; translation initiation factor 2 subunit 3
LOC102273166	518.9063	0.440523	0.147402116	2.988582	0.0028027	0.022457	Not_Sig	no	gi|511910465|ref|XP_004774628.1|	1102	4e-137	PREDICTED: homeobox protein Hox-C6 isoform X1 [Mustela putorius furo]	gi|982981570|ref|XM_010827624.2|	3800	0	PREDICTED: Bos taurus homeobox C6 (HOXC6), transcript variant X1, mRNA	P10629	428	2e-138	Homeobox protein Hox-C6	.	.	.	.	pfam00046	Homeobox	Homeobox domain.	GO:0009952|anterior/posterior pattern specification;GO:0048706|embryonic skeletal system development;GO:0006351|transcription, DNA-templated;	GO:0005737|cytoplasm;GO:0005654|nucleoplasm;	GO:0043565|sequence-specific DNA binding;GO:0003700|transcription factor	K09306	HOX_6; homeobox protein HoxA/B/C6
PEDS1	842.5284	0.480426	0.160770246	2.988279	0.0028055	0.022464	Not_Sig	no	gi|594047988|ref|XP_006048400.1|	1407	0	PREDICTED: transmembrane protein 189 isoform X1 [Bubalus bubalis]	gi|742097783|ref|XM_010835122.1|	2031	0	PREDICTED: Bison bison bison transmembrane protein 189 (TMEM189), mRNA	A6QLM0	541	0	Transmembrane protein 189	.	.	.	.	pfam10520	Kua-UEV1_localn	Kua-ubiquitin conjugating enzyme hybrid localisation domain. This domain is part of the transcript of the fusion of two genes, the UEV1,	.	GO:0005737|cytoplasm;GO:0005789|endoplasmic reticulum membrane;GO:0016021|integral component of membrane;	GO:0061630|ubiquitin protein ligase activity;GO:0031625|ubiquitin protein	.	.
TBL1XR1	635.4405	-0.45919	0.153730415	-2.98695	0.0028177	0.022546	Not_Sig	no	gi|300795665|ref|NP_001179958.1|	2584	0	F-box-like/WD repeat-containing protein TBL1XR1 [Bos taurus]	gi|742149523|ref|XM_010845691.1|	6528	0	PREDICTED: Bison bison bison transducin (beta)-like 1 X-linked receptor 1 (TBL1XR1), transcript variant X2, mRNA	Q8BHJ5	994	0	F-box-like/WD repeat-containing protein TBL1XR1	YBR103w	162	7.94e-41	COG2319	pfam00400	WD40	WD domain, G-beta repeat.	GO:0060612|adipose tissue development;GO:0060070|canonical Wnt signaling pathway;GO:0060613|fat pad development;GO:0016575|histone deacetylation;GO:0016042|lipid catabolic process;GO:0035264|multicellular organism growth;GO:0000122|negative regulation of transcription from RNA polymerase II promoter;GO:0045892|negative regulation of transcription, DNA-templated;GO:0045944|positive regulation of transcription from RNA polymerase II promoter;GO:0045893|positive regulation of	GO:0000118|histone deacetylase complex;GO:0005654|nucleoplasm;GO:0005634|nucleus;GO:0005876|spindle microtubule;GO:0017053|transcriptional	GO:0008013|beta-catenin binding;GO:0003677|DNA	K04508	TBL1; transducin (beta)-like 1
SYDE1	1204.406	0.391485	0.1310862	2.986469	0.0028222	0.022566	Not_Sig	no	gi|742196031|ref|XP_010856659.1|	2813	0	PREDICTED: rho GTPase-activating protein SYDE1 isoform X1 [Bison bison bison]	gi|742196030|ref|XM_010858357.1|	2903	0	PREDICTED: Bison bison bison synapse defective 1, Rho GTPase, homolog 1 (C. elegans) (SYDE1), transcript variant X1, mRNA	Q9DBZ9	948	0	Rho GTPase-activating protein SYDE1	.	.	.	.	pfam00620	RhoGAP	RhoGAP domain. GTPase activator proteins towards Rho/Rac/Cdc42-like small GTPases.	GO:0090630|activation of GTPase activity;GO:0050806|positive regulation of synaptic transmission;GO:0007165|signal transduction;GO:0016081|synaptic vesicle docking;GO:0007130|synaptonemal complex assembly;	GO:0044300|cerebellar mossy fiber;GO:0005829|cytosol;GO:0098793|presynapse;GO:0097060|synaptic membrane;	GO:0005096|GTPase activator activity;GO:0032403|protein complex	.	.
CIAO1	218.8187	0.449344	0.15047161	2.986235	0.0028244	0.022567	Not_Sig	no	gi|555959117|ref|XP_005891669.1|	1686	0	PREDICTED: probable cytosolic iron-sulfur protein assembly protein CIAO1 [Bos mutus]	gi|942042969|ref|XM_005891607.2|	1217	0	PREDICTED: Bos mutus cytosolic iron-sulfur assembly component 1 (CIAO1), mRNA	Q32PJ6	652	0	Probable cytosolic iron-sulfur protein assembly protein CIAO1	.	.	.	.	pfam00400	WD40	WD domain, G-beta repeat.	GO:0007059|chromosome segregation;GO:0016226|iron-sulfur cluster assembly;	GO:0097361|CIA complex;GO:0071817|MMXD complex;	.	.	.
ZNF703	420.9014	0.448547	0.150236336	2.985607	0.0028302	0.022598	Not_Sig	no	gi|803237931|ref|XP_011979079.1|	1825	0	PREDICTED: zinc finger protein 703 [Ovis aries musimon]	gi|965816188|ref|XM_012123689.2|	2979	0	PREDICTED: Ovis aries musimon zinc finger protein 703 (LOC105607920), mRNA	Q9H7S9	665	0	Zinc finger protein 703	.	.	.	.	pfam12402	nlz1	NocA-like zinc-finger protein 1. This domain family is found in eukaryotes, and is typically between 42 and 57 amino acids in	GO:0034333|adherens junction assembly;GO:0071392|cellular response to estradiol stimulus;GO:0060644|mammary gland epithelial cell differentiation;GO:0034111|negative regulation of homotypic cell-cell adhesion;GO:0045892|negative regulation of transcription, DNA-templated;GO:0030335|positive regulation of cell migration;GO:0008284|positive regulation of cell proliferation;GO:0010718|positive regulation of epithelial to mesenchymal transition;GO:0033601|positive regulation of mammary gland epithelial cell	GO:0005737|cytoplasm;GO:0016363|nuclear matrix;GO:0005634|nucleus;GO:0043234|protein complex;	GO:0046872|metal ion binding;GO:0003676|nucleic acid	.	.
SINHCAF	772.5782	-0.36049	0.120792691	-2.98438	0.0028416	0.022673	Not_Sig	no	gi|859806213|ref|XP_012913297.1|	1088	2e-137	PREDICTED: protein FAM60A [Mustela putorius furo]	gi|982926413|ref|XM_005206844.3|	2690	0	PREDICTED: Bos taurus family with sequence similarity 60 member A (FAM60A), transcript variant X1, mRNA	Q9NP50	407	2e-133	Protein FAM60A	.	.	.	.	pfam15396	FAM60A	Protein Family FAM60A. This protein family, FAM60A is a family of proteins is found in eukaryotes. It is known to be a cell cycle	GO:0030336|negative regulation of cell migration;	GO:0016580|Sin3 complex;	.	.	.
CD44	8505.649	0.298301	0.099987386	2.983388	0.0028508	0.022731	Not_Sig	no	gi|594077402|ref|XP_006062420.1|	3113	0	PREDICTED: CD44 antigen isoform X1 [Bubalus bubalis]	gi|528978299|ref|XM_005216354.1|	2402	0	PREDICTED: Bos taurus CD44 molecule (Indian blood group) (CD44), transcript variant X6, mRNA	P16070	782	0	CD44 antigen	.	.	.	.	pfam00193	Xlink	Extracellular link domain.	GO:0051216|cartilage development;GO:0007160|cell-matrix adhesion;GO:0044344|cellular response to fibroblast growth factor stimulus;GO:0022617|extracellular matrix disassembly;GO:0030198|extracellular matrix organization;GO:0030214|hyaluronan catabolic process;GO:0060333|interferon-gamma-mediated signaling pathway;GO:0050900|leukocyte migration;GO:0070487|monocyte aggregation;GO:0043066|negative regulation of apoptotic process;GO:0043154|negative regulation of cysteine-type endopeptidase activity	GO:0009986|cell surface;GO:0005737|cytoplasm;GO:0070062|extracellular exosome;GO:0005925|focal adhesion;GO:0005794|Golgi	GO:0005518|collagen binding;GO:0005540|hyaluronic acid	K06256	CD44; CD44 antigen
UXT	104.7275	-0.61289	0.205453588	-2.98313	0.0028532	0.022734	DOWN	yes	gi|594053493|ref|XP_006051040.1|	957	9e-129	PREDICTED: ETS domain-containing protein Elk-1-like isoform X2 [Bubalus bubalis]	gi|942065125|ref|XM_005899362.2|	663	0	PREDICTED: Bos mutus ubiquitously-expressed, prefoldin-like chaperone (UXT), mRNA	Q32P97	291	5e-99	Protein UXT	.	.	.	.	pfam02996	Prefoldin	Prefoldin subunit. This family comprises of several prefoldin subunits. The biogenesis of the cytoskeletal proteins actin and	GO:0051297|centrosome organization;GO:0000122|negative regulation of transcription from RNA polymerase II promoter;GO:0006351|transcription, DNA-templated;	GO:0005813|centrosome;GO:0005737|cytoplasm;GO:0005634|nucleus;	GO:0003682|chromatin binding;GO:0001104|RNA polymerase II	.	.

NSMCE1	614.5473	-0.35212	0.11809027	-2.98179	0.0028657	0.022818	Not_Sig	no	gi|803239859|ref|XP_011980019.1|	1628	0	PREDICTED: non-structural maintenance of chromosomes element 1 homolog isoform X2 [Ovis aries musimon]	gi|742106818|ref|XM_010837224.1|	974	0	PREDICTED: Bison bison bison non-SMC element 1 homolog (S. cerevisiae) (NSMCE1), transcript variant X1, mRNA	Q3T0X7	547	0	Non-structural maintenance of chromosomes element 1 homolog	.	.	.	.	pfam07574	SMC_Nse1	Nse1 non-SMC component of SMC5-6 complex. S. cerevisiae Nse1 forms part of a complex with SMC5-SMC6 This non-structural	GO:0000724|double-strand break repair via homologous recombination;GO:0035556|intracellular signal transduction;GO:2001022|positive regulation of response to DNA damage stimulus;GO:0006301|postreplication repair;GO:0016567|protein ubiquitination;	GO:0000781|chromosome, telomeric region;GO:0005634|nucleus;GO:0030915|Smc5-Smc6 complex;	GO:0016874|ligase activity;GO:0004842|ubiquitin-protein transferase	.	.
B4GALT2	1191.745	0.420853	0.141177336	2.981026	0.0028728	0.022844	Not_Sig	no	gi|157279969|ref|NP_001098500.1|	1872	0	beta-1,4-galactosyltransferase 2 [Bos taurus]	gi|926688999|ref|XM_013962915.1|	2316	0	PREDICTED: Capra hircus UDP-Gal:betaGlcNAc beta 1,4- galactosyltransferase, polypeptide 2 (B4GALT2), partial mRNA	O60909	691	0	Beta-1,4-galactosyltransferase 2	.	.	.	.	pfam13733	Glyco_transf_7N	N-terminal region of glycosyl transferase group 7. This is the N-terminal half of a family of galactosyltransferases from a wide	GO:0021680|cerebellar Purkinje cell layer development;GO:0018146|keratan sulfate biosynthetic process;GO:0007626|locomotory behavior;GO:0007613|memory;GO:0006486|protein glycosylation;GO:0008542|visual learning;	GO:0032580|Golgi cisterna membrane;GO:0000139|Golgi membrane;GO:0016021|integral component of membrane;	GO:0003831|beta-N-acetylglucosaminylglycopeptide beta-	K07967	B4GALT2; beta-1,4-galactosyltransferase 2 [EC:2.4.1.22 2.4.1.90 2.4.1.38
NCSTN	2099.782	0.358328	0.120208189	2.980893	0.0028741	0.022844	Not_Sig	no	gi|555984107|ref|XP_005903947.1|	3706	0	PREDICTED: nicastrin isoform X1 [Bos mutus]	gi|742153824|ref|XM_010846885.1|	2278	0	PREDICTED: Bison bison bison nicastrin (NCSTN), transcript variant X1, mRNA	Q92542	1205	0	Nicastrin	.	.	.	.	pfam05450	Nicastrin	Nicastrin. Nicastrin and presenilin are two major components of the gamma-secretase complex, which executes the intramembrane	GO:0042987|amyloid precursor protein catabolic process;GO:0050435|beta-amyloid metabolic process;GO:0048013|ephrin receptor signaling pathway;GO:0050673|epithelial cell proliferation;GO:0006509|membrane protein ectodomain proteolysis;GO:0031293|membrane protein intracellular domain proteolysis;GO:0002262|myeloid cell homeostasis;GO:0007220|Notch receptor processing;GO:0007219|Notch signaling pathway;GO:0043065|positive regulation of apoptotic process;GO:0043085|positive regulation of catalytic activity;GO:0016485|protein	GO:0005783|endoplasmic reticulum;GO:0070062|extracellular exosome;GO:0005925|focal adhesion;GO:0005794|Golgi	GO:0004175|endopeptidase activity;	K06171	NCSTN; nicastrin
REEP4	402.1673	0.41942	0.14070674	2.980812	0.0028748	0.022844	Not_Sig	no	gi|982933867|ref|XP_015328069.1|	1470	0	PREDICTED: receptor expression-enhancing protein 4 isoform X3 [Bos taurus]	gi|982933866|ref|XM_015472583.1|	1518	0	PREDICTED: Bos taurus receptor accessory protein 4 (REEP4), transcript variant X3, mRNA	Q3ZCI8	362	8e-121	Receptor expression-enhancing protein 4	SPBC30D10.09c	66.6	9.20e-12	COG5052	pfam03134	TB2_DP1_HVA22	TB2/DP1, HVA22 family. This family includes members from a wide variety of eukaryotes. It includes the TB2/DP1 (deleted in	GO:0051301|cell division;GO:0007067|mitotic nuclear division;GO:0007084|mitotic nuclear envelope reassembly;GO:0006998|nuclear envelope organization;	GO:0005783|endoplasmic reticulum;GO:0005789|endoplasmic reticulum membrane;GO:0016021|integral component of	GO:0008017|microtubule binding;	K17338	REEP1_2_3_4; receptor expression-enhancing protein 1/2/3/4
ZNF628	150.009	0.559786	0.187829663	2.980288	0.0028798	0.022867	Not_Sig	no	gi|803321661|ref|XP_012019047.1|	3429	0	PREDICTED: zinc finger protein 628 [Ovis aries musimon]	gi|982955058|ref|XM_010815491.2|	3254	0	PREDICTED: Bos taurus zinc finger protein 628 (ZNF628), transcript variant X1, mRNA	Q5EBL2	791	0	Zinc finger protein 628	.	.	.	.	pfam13465	zf-H2C2_2	Zinc-finger double domain.	GO:0006355|regulation of transcription, DNA-templated;GO:0006351|transcription, DNA-templated;	GO:0005634|nucleus;	GO:0003677|DNA binding;GO:0046872|metal ion binding;GO:0003700	.	.
QKI	3065.211	-0.31652	0.106225735	-2.97973	0.002885	0.022893	Not_Sig	no	gi|7542357|gb|AAF63417.1|AF142422_1	1499	0	QUAKING isoform 3 [Homo sapiens]	gi|965823365|ref|XR_001029686.2|	6759	0	PREDICTED: Ovis aries musimon QKI, KH domain containing, RNA binding (QKI), transcript variant X12, misc_RNA	Q5W9D5	551	1e-176	Protein quaking	SPCC962.06c_1	107	4.48e-24	COG5176	pfam07777	MFMR	G-box binding protein MFMR. This region is found to the N-terminus of the pfam00170 transcription factor domain. It is between 150	GO:0030154|cell differentiation;GO:0006397|mRNA processing;GO:0051028|mRNA transport;GO:0007275|multicellular organismal development;GO:0006417|regulation of translation;GO:0008380|RNA splicing;	GO:0005737|cytoplasm;GO:0005634|nucleus;	GO:0003723|RNA binding;	K14945	QKI; protein quaking
DNAJC5	932.3767	0.33806	0.113466486	2.979379	0.0028883	0.022903	Not_Sig	no	gi|594674793|ref|XP_007185242.1|	908	7e-112	PREDICTED: LOW QUALITY PROTEIN: dnaJ homolog subfamily C member 5 [Balaenoptera acutorostrata scammoni]	gi|741947734|ref|XR_236996.2|	2749	0	PREDICTED: Bos taurus DnaJ heat shock protein family (Hsp40) member C5 (DNAJC5), transcript variant X2, misc_RNA	Q29455	351	2e-112	DnaJ homolog subfamily C member 5	PA4760	90.5	2.16e-18	COG0484	pfam00226	DnaJ	DnaJ domain. DnaJ domains (J-domains) are associated with hsp70 heat-shock system and it is thought that this domain mediates	GO:0043524|negative regulation of neuron apoptotic process;	GO:0070062|extracellular exosome;GO:0005765|lysosomal membrane;GO:0042470|melanosome;GO:0005739|mitochondrion;GO:0005886|plasm	.	K09525	DNAJC5; DnaJ homolog subfamily C member 5
TRIM8	786.2998	0.400176	0.134379629	2.977955	0.0029018	0.022994	Not_Sig	no	gi|148710059|gb|EDL42005.1|	2988	0	tripartite motif protein 8, isoform CRA_b, partial [Mus musculus]	gi|803229409|ref|XM_012119587.1|	3237	0	PREDICTED: Ovis aries musimon tripartite motif containing 8 (TRIM8), transcript variant X2, mRNA	Q9BZR9	1132	0	Probable E3 ubiquitin-protein ligase TRIM8	.	.	.	.	pfam05483	SCP-1	Synaptonemal complex protein 1 (SCP-1). Synaptonemal complex protein 1 (SCP-1) is the major component of the transverse	GO:0045087|innate immune response;GO:0060333|interferon-gamma-mediated signaling pathway;GO:0046597|negative regulation of viral entry into host cell;GO:1902187|negative regulation of viral release from host cell;GO:0032897|negative regulation of viral transcription;GO:0010508|positive regulation of autophagy;GO:0043123|positive regulation of I-kappaB kinase/NF-kappaB signaling;GO:0051092|positive regulation of NF-kappaB transcription factor activity;GO:1900182|positive regulation of protein localization to	GO:0005737|cytoplasm;GO:0005829|cytosol;GO:0043231|intracellular membrane-bounded organelle;GO:0005654|nucleoplasm;GO:001	GO:0016874|ligase activity;GO:0042803|protein homodimerization	K12001	TRIM8; tripartite motif-containing protein 8 [EC:2.3.2.27]
SEPTIN9	4083.162	0.354948	0.119212073	2.977449	0.0029066	0.023016	Not_Sig	no	gi|555992614|ref|XP_005908079.1|	2954	0	PREDICTED: septin-9 [Bos mutus]	gi|742187617|ref|XM_010856235.1|	4119	0	PREDICTED: Bison bison bison septin 9 (SEPT9), transcript variant X2, mRNA	Q9UHD8	1055	0	Septin-9	SPAC821.06	318	1.44e-97	COG5019	pfam00735	Septin	Septin. Members of this family include CDC3, CDC10, CDC11 and CDC12/Septin. Members of this family bind GTP. As regards the	GO:0007049|cell cycle;GO:0051301|cell division;GO:1902857|positive regulation of nonmotile primary cilium assembly;GO:0051291|protein heterooligomerization;	GO:0015629|actin cytoskeleton;GO:0005930|axoneme;GO:0005913|cell-cell adherens junction;GO:0005737|cytoplasm;GO:000587	GO:0098641|cadherin binding involved in cell-cell adhesion;GO:00055	K16938	SEPT3_9_12; septin 3/9/12
ABL1	6280.033	0.332148	0.111603356	2.97615	0.0029189	0.023098	Not_Sig	no	gi|332309186|ref|NP_001193789.1|	5317	0	tyrosine-protein kinase ABL1 [Bos taurus]	gi|332309185|ref|NM_001206860.1|	5451	0	Bos taurus ABL proto-oncogene 1, non-receptor tyrosine kinase (ABL1), mRNA	P00519	1780	0	Tyrosine-protein kinase ABL1	.	.	.	.	pfam07714	Pkinase_Tyr	Protein tyrosine kinase.	GO:0030036|actin cytoskeleton organization;GO:0090135|actin filament branching;GO:0030041|actin filament polymerization;GO:0050798|activated T cell proliferation;GO:1990051|activation of protein kinase C activity;GO:0046632|alpha-beta T cell differentiation;GO:0008306|associative learning;GO:0006914|autophagy;GO:0002322|B cell proliferation involved in immune response;GO:0050853|B cell receptor signaling pathway;GO:0001922|B-1 B cell homeostasis;GO:0060020|Bergmann glial cell	GO:0015629|actin cytoskeleton;GO:0005737|cytoplasm;GO:0005829|cytosol;GO:0005783|endoplasmic reticulum;GO:0031234|extrinsic component	GO:0003785|actin monomer binding;GO:0005524|ATP	K06619	ABL1; abelson tyrosine-protein kinase 1 [EC:2.7.10.2]
CFAP418	219.4668	-0.46618	0.156673702	-2.97545	0.0029256	0.023135	Not_Sig	no	gi|76684737|ref|XP_871425.1|	947	1e-115	PREDICTED: protein C8orf37 homolog [Bos taurus]	gi|742090970|ref|XM_010833483.1|	3740	0	PREDICTED: Bison bison bison chromosome unknown open reading frame, human C8orf37 (LOC104983938), mRNA	E1BC52	369	5e-117	Protein C8orf37 homolog	.	.	.	.	pfam14996	RMP	Retinal Maintenance. RMP is encoded for by a gene, C8orf37. Mutations in the gene cause two types of retinal dystrophies: cone-	.	GO:0030054|cell junction;GO:0005737|cytoplasm;GO:0005886|plasma membrane;	.	.	.
COX3	1554.854	-0.34111	0.114685231	-2.97433	0.0029363	0.023203	Not_Sig	no	gi|722490026|ref|YP_009107153.1|	1144	1e-155	cytochrome c oxidase subunit III (mitochondrion) [Bos mutus]	gi|698854195|gb|KM233417.1|	784	0	Bos mutus mitochondrion, complete genome	Q5Y4Q4	444	7e-157	Cytochrome c oxidase subunit 3	mlr7496	209	2.24e-66	COG1845	pfam00510	COX3	Cytochrome c oxidase subunit III.	GO:0019646|aerobic electron transport chain;GO:0008535|respiratory chain complex IV assembly;	GO:0016021|integral component of membrane;GO:0005743|mitochondrial inner membrane;GO:0045277|respiratory chain complex IV;	GO:0004129|cytochrome-c oxidase activity;	.	.
MRPL22	232.9049	-0.50923	0.171301016	-2.97273	0.0029516	0.023309	Not_Sig	no	gi|742226330|ref|XP_010827695.1|	1082	5e-147	PREDICTED: 39S ribosomal protein L22, mitochondrial isoform X1 [Bison bison bison]	gi|742226329|ref|XM_010829393.1|	795	0	PREDICTED: Bison bison bison mitochondrial ribosomal protein L22 (MRPL22), transcript variant X1, mRNA	Q3SZX5	402	3e-141	39S ribosomal protein L22, mitochondrial	SMc01304	67.4	2.12e-13	COG0091	pfam00237	Ribosomal_L22	Ribosomal protein L22p/L17e. This family includes L22 from prokaryotes and chloroplasts and L17 from eukaryotes.	GO:0070125|mitochondrial translational elongation;GO:0070124|mitochondrial translational initiation;	GO:0005743|mitochondrial inner membrane;GO:0005762|mitochondrial large ribosomal subunit;GO:0005739|mitochondrion;	GO:0044822|poly (A) RNA binding;GO:0003735|structural	K02890	RP-L22, MRPL22, rplV; large subunit ribosomal protein L22
TSNAX	459.1827	-0.45765	0.154002466	-2.97167	0.0029618	0.023366	Not_Sig	no	gi|164448725|ref|NP_001069474.2|	1554	0	translin-associated protein X isoform 1 [Bos taurus]	gi|942041910|ref|XM_005891159.2|	2440	0	PREDICTED: Bos mutus translin-associated factor X (TSNAX), mRNA	Q5RC21	588	0	Translin-associated protein X	SPCC736.09c	104	1.98e-24	COG2178	pfam01997	Translin	Translin family. Members of this family include Translin that interacts with DNA and forms a ring around the DNA. This family	GO:0030154|cell differentiation;GO:0007275|multicellular organismal development;GO:0007283|spermatogenesis;	GO:0005794|Golgi apparatus;GO:0005634|nucleus;GO:0048471|perinuclear region of cytoplasm;	GO:0046872|metal ion binding;GO:0043565|sequence-specific	.	.
KRR1	493.1277	-0.41271	0.138886756	-2.97156	0.0029629	0.023366	Not_Sig	no	gi|555995928|ref|XP_005909703.1|	1692	0	PREDICTED: KRR1 small subunit processome component homolog [Bos mutus]	gi|942097183|ref|XM_005909641.2|	2537	0	PREDICTED: Bos mutus KRR1, small subunit (SSU) processome component, homolog (yeast) (KRR1), mRNA	Q3B7L9	656	0	KRR1 small subunit processome component homolog	YCL059c	337	2.19e-108	COG1094	pfam00013	KH_1	KH domain. KH motifs bind RNA in vitro. Autoantibodies to Nova, a KH domain protein, cause paraneoplastic opsoclonus ataxia.	GO:0000462|maturation of SSU-rRNA from tricistronic rRNA transcript (SSU-rRNA, 5.8S rRNA, LSU-rRNA);	GO:0005737|cytoplasm;GO:0045171|intercellular bridge;GO:0016020|membrane;GO:0005730|nucleolus;GO:0032040|small-subunit	GO:0044822|poly (A) RNA binding;	K06961	KRR1; ribosomal RNA assembly protein
INTU	170.2895	-0.54163	0.182290519	-2.97124	0.002966	0.023374	Not_Sig	no	gi|528981595|ref|XP_005217612.1|	4656	0	PREDICTED: protein inturned isoform X1 [Bos taurus]	gi|742203883|ref|XM_010860259.1|	5689	0	PREDICTED: Bison bison bison inturned planar cell polarity protein (INTU), mRNA	F1MDL2	1724	0	Protein inturned	.	.	.	.	pfam00078	RVT_1	Reverse transcriptase (RNA-dependent DNA polymerase). A reverse transcriptase gene is usually indicative of a mobile	GO:0042384|cilium assembly;GO:0042733|embryonic digit morphogenesis;GO:0031069|hair follicle morphogenesis;GO:0030216|keratinocyte differentiation;GO:0060173|limb development;GO:1903887|motile primary cilium assembly;GO:0051782|negative regulation of cell division;GO:0010839|negative regulation of keratinocyte proliferation;GO:0007399|nervous system development;GO:0021915|neural tube development;GO:0045880|positive regulation of smoothened signaling	GO:0009986|cell surface;GO:0005737|cytoplasm;	.	.	.
SH3GLB2	515.9512	0.402307	0.135441524	2.970334	0.0029748	0.023427	Not_Sig	no	gi|528969867|ref|XP_005213253.1|	1973	0	PREDICTED: endophilin-B2 isoform X3 [Bos taurus]	gi|982939743|ref|XM_005213196.3|	1608	0	PREDICTED: Bos taurus SH3-domain GRB2-like endophilin B2 (SH3GLB2), transcript variant X3, mRNA	Q08DK5	639	0	Endophilin-B2	.	.	.	.	pfam03114	BAR	BAR domain. BAR domains are dimerization, lipid binding and curvature sensing modules found in many different protein families.	.	GO:0005737|cytoplasm;GO:0005634|nucleus;	.	K11248	SH3GLB; endophilin-B
SH3GL1	2604.407	0.410514	0.138225719	2.969878	0.0029792	0.023446	Not_Sig	no	gi|555967975|ref|XP_005896031.1|	1918	0	PREDICTED: endophilin-A2 isoform X1 [Bos mutus]	gi|942055057|ref|XM_005895969.2|	2547	0	PREDICTED: Bos mutus SH3-domain GRB2-like 1 (SH3GL1), transcript variant X1, mRNA	Q2KJA1	736	0	Endophilin-A2	.	.	.	.	pfam03114	BAR	BAR domain. BAR domains are dimerization, lipid binding and curvature sensing modules found in many different protein families.	GO:0006897|endocytosis;	GO:0030054|cell junction;GO:0042995|cell projection;GO:0031901|early endosome membrane;GO:0002102|podosome;	GO:0008289|lipid binding;	K11247	SH3GL; endophilin-A
MED25	900.1578	0.488978	0.164698482	2.968929	0.0029884	0.023485	Not_Sig	no	gi|594114833|ref|XP_006080132.1|	2884	0	PREDICTED: mediator of RNA polymerase II transcription subunit 25 isoform X1 [Bubalus bubalis]	gi|594114832|ref|XM_006080070.1|	3697	0	PREDICTED: Bubalus bubalis mediator complex subunit 25 (MED25), transcript variant X1, mRNA	A2VE44	940	0	Mediator of RNA polymerase II transcription subunit 25	.	.	.	.	pfam11265	Med25_VWA	Mediator complex subunit 25 von Willebrand factor type A. The overall function of the full-length Med25 is efficiently to coordinate	GO:0006355|regulation of transcription, DNA-templated;GO:0006351|transcription, DNA-templated;	GO:0005634|nucleus;	.	K15168	MED25; mediator of RNA polymerase II transcription subunit 25
RBPJ	1139.737	-0.36582	0.12322198	-2.96882	0.0029895	0.023485	Not_Sig	no	gi|465988250|ref|XP_004266261.1|	2475	0	PREDICTED: recombining binding protein suppressor of hairless isoform X3 [Orcinus orca]	gi|982928120|ref|XM_005207811.3|	5672	0	PREDICTED: Bos taurus recombination signal binding protein for immunoglobulin kappa J region (RBPJ), transcript variant X3, mRNA	Q5RFK6	952	0	Recombining binding protein suppressor of hairless	.	.	.	.	pfam09270	BTD	Beta-trefoil DNA-binding domain. Members of this family of DNA binding domains adopt a beta-trefoil fold, that is, a capped beta-	GO:0061419|positive regulation of transcription from RNA polymerase II promoter in response to hypoxia;GO:0007221|positive regulation of transcription of Notch receptor target;GO:0006351|transcription, DNA-templated;	GO:0005737|cytoplasm;GO:0005634|nucleus;	GO:0000978|RNA polymerase II core promoter proximal region sequence-	.	.
IPO13	784.1005	0.385032	0.129695475	2.968736	0.0029903	0.023485	Not_Sig	no	gi|39104529|dbj|BAC98010.2|	3793	0	mKIAA0724 protein [Mus musculus]	gi|982920071|ref|XM_015466307.1|	4058	0	PREDICTED: Bos taurus importin 13 (IPO13), transcript variant X2, mRNA	A7YWD2	1415	0	Importin-13	.	.	.	.	pfam08389	Xpo1	Exportin 1-like protein. The sequences featured in this family are similar to a region close to the N-terminus of yeast exportin 1	GO:0006606|protein import into nucleus;	GO:0005737|cytoplasm;GO:0031965|nuclear membrane;	GO:0008139|nuclear localization sequence binding;GO:0008565	.	.
CALHM2	256.1156	0.458874	0.154610343	2.967936	0.0029981	0.02353	Not_Sig	no	gi|555951808|ref|XP_005888099.1|	1675	0	PREDICTED: calcium homeostasis modulator protein 2 [Bos mutus]	gi|942033746|ref|XM_005888037.2|	2039	0	PREDICTED: Bos mutus calcium homeostasis modulator 2 (CALHM2), mRNA	Q2HJ63	645	0	Calcium homeostasis modulator protein 2	.	.	.	.	pfam14798	Ca_hom_mod	Calcium homeostasis modulator. This family of proteins control cytosolic calcium concentration. They are transmembrane proteins	GO:0034220|ion transmembrane transport;	GO:0005887|integral component of plasma membrane;	GO:0005261|cation channel activity;	.	.
PGM2L1	139.0199	-0.5669	0.191062754	-2.9671	0.0030062	0.023559	Not_Sig	no	gi|426245131|ref|XP_004016367.1|	3246	0	PREDICTED: glucose 1,6-bisphosphate synthase isoform X1 [Ovis aries]	gi|742160680|ref|XM_010848857.1|	7486	0	PREDICTED: Bison bison bison phosphoglucomutase 2-like 1 (PGM2L1), mRNA	Q5R979	1211	0	Glucose 1,6-bisphosphate synthase	SPCC1840.05c	416	4.47e-127	COG1109	pfam02878	PGM_PMM_I	Phosphoglucomutase/phosphomannomutase, alpha/beta/alpha domain I.	GO:0006006|glucose metabolic process;	.	GO:0047933|glucose-1,6-bisphosphate synthase activity;GO:0016868	K11809	PGM2L1; glucose-1,6-bisphosphate synthase [EC:2.7.1.106]
TRABD2B	106.2209	0.584059	0.196851941	2.966996	0.0030073	0.023559	Not_Sig	no	gi|826306838|ref|XP_012504392.1|	2080	0	PREDICTED: metalloprotease TIKI2 [Propithecus coquereli]	gi|982979465|ref|XM_001254585.5|	6883	0	PREDICTED: Bos taurus TraB domain containing 2B (TRABD2B), transcript variant X1, mRNA	B1ATG9	692	0	Metalloprotease TIKI2	BMEI0146	62	6.37e-09	COG3735	pfam01963	TraB	TraB family. pAD1 is a hemolysin/bacteriocin plasmid originally identified in Enterococcus faecalis DS16. It	GO:0030178|negative regulation of Wnt signaling pathway;GO:0032461|positive regulation of protein oligomerization;GO:0006508|proteolysis;GO:0016055|Wnt signaling pathway;	GO:0070062|extracellular exosome;GO:0031301|integral component of organelle membrane;GO:0005887|integral	GO:0046872|metal ion binding;GO:0004222|metalloendopeptid	.	.
PRRX1	3212.518	-0.32481	0.109479825	-2.96686	0.0030086	0.023559	Not_Sig	no	gi|126165288|ref|NP_001075208.1|	1236	5e-154	paired mesoderm homeobox protein 1 [Bos taurus]	gi|594073215|ref|XM_006060370.1|	4514	0	PREDICTED: Bubalus bubalis paired related homeobox 1 (PRRX1), transcript variant X1, mRNA	P63014	476	4e-154	Paired mesoderm homeobox protein 1	ECU03g1170	53.1	1.65e-06	COG5576	pfam00046	Homeobox	Homeobox domain.	GO:0048844|artery morphogenesis;GO:0051216|cartilage development;GO:0048701|embryonic cranial skeleton morphogenesis;GO:0030326|embryonic limb morphogenesis;GO:0042472|inner ear morphogenesis;GO:0042474|middle ear morphogenesis;GO:0000122|negative regulation of transcription from RNA polymerase II promoter;GO:0048664|neuron fate determination;GO:0097150|neuronal stem cell population maintenance;GO:0060021|palate development;GO:0002053|positive regulation of	GO:0005730|nucleolus;	GO:0001105|RNA polymerase II transcription coactivator	K09329	PRRX, PMX; paired mesoderm homeobox protein
GINM1	893.8022	-0.34203	0.115289076	-2.96672	0.0030099	0.023559	Not_Sig	no	gi|742213364|ref|XP_010860870.1|	1358	0	PREDICTED: glycoprotein integral membrane protein 1, partial [Bison bison bison]	gi|942092301|ref|XM_005908057.2|	1594	0	PREDICTED: Bos mutus glycoprotein integral membrane 1 (GINM1), transcript variant X1, mRNA	Q5RBQ2	473	2e-162	Glycoprotein integral membrane protein 1	.	.	.	.	pfam14887	HMG_box_5	HMG (high mobility group) box 5. Nucleolar transcription factor/upstream binding factor contains six HMG box domains.	.	GO:0016021|integral component of membrane;	.	.	.
FLNC	4432.115	0.355927	0.11999774	2.966117	0.0030159	0.023589	Not_Sig	no	gi|594102786|ref|XP_006074442.1|	13975	0	PREDICTED: filamin-C isoform X2 [Bubalus bubalis]	gi|942083142|ref|XM_005905254.2|	8754	0	PREDICTED: Bos mutus filamin C, gamma (FLNC), transcript variant X1, mRNA	Q14315	5310	0	Filamin-C	ECU09g0290	140	3.24e-33	COG5069	pfam00630	Filamin	Filamin/ABP280 repeat.	GO:0034329|cell junction assembly;GO:0048747|muscle fiber development;	GO:0043034|costamere;GO:0005737|cytoplasm;GO:0005856|cytoskeleton;GO:0005829|cytosol;GO:0005925|focal adhesion;GO:0005886|plasma	GO:0030506|ankyrin binding;GO:0008092|cytoskeletal	K04437	FLNA; filamin
SELENOK	410.3207	-0.3899	0.131524198	-2.9645	0.0030318	0.023697	Not_Sig	no	gi|144953867|ref|NP_001032566.2|	253	2e-25	selenoprotein K [Bos taurus]	gi|942056327|ref|XM_014478442.1|	398	0	PREDICTED: Bos mutus selenoprotein K (LOC102270591), transcript variant X1, mRNA	Q32PE3	102	8e-27	Selenoprotein K	.	.	.	.	pfam10961	DUF2763	Protein of unknown function (DUF2763). This eukaryotic family of proteins has no known function.	GO:0006816|calcium ion transport;GO:0032469|endoplasmic reticulum calcium ion homeostasis;GO:0002376|immune system process;	GO:0005789|endoplasmic reticulum membrane;GO:0005794|Golgi apparatus;GO:0016021|integral component of membrane;GO:0005886|plasma	.	.	.
RNF181	1088.163	-0.31866	0.107535478	-2.96329	0.0030437	0.023769	Not_Sig	no	gi|965790734|ref|XP_014962634.1|	871	8e-116	PREDICTED: E3 ubiquitin-protein ligase RNF181 isoform X3 [Ovis aries musimon]	gi|741943110|ref|XM_010810056.1|	652	0	PREDICTED: Bos taurus ring finger protein 181 (RNF181), transcript variant X1, mRNA	Q3T0W3	266	2e-89	E3 ubiquitin-protein ligase RNF181	.	.	.	.	pfam13639	zf-RING_2	Ring finger domain.	GO:0043161|proteasome-mediated ubiquitin-dependent protein catabolic process;GO:0051865|protein autoubiquitination;GO:0000209|protein polyubiquitination;GO:0042787|protein ubiquitination involved in ubiquitin-dependent protein catabolic process;	.	GO:0016874|ligase activity;GO:0061630|ubiquitin protein ligase	.	.
PPP1R13B	404.7812	0.464687	0.156823687	2.963116	0.0030454	0.023769	Not_Sig	no	gi|741898854|ref|XP_010823294.1|	5016	0	PREDICTED: apoptosis-stimulating of p53 protein 1 isoform X1 [Bos taurus]	gi|983004160|ref|XM_010824996.2|	4199	0	PREDICTED: Bos taurus protein phosphatase 1 regulatory subunit 13B (PPP1R13B), transcript variant X3, mRNA	Q62415	1685	0	Apoptosis-stimulating of p53 protein 1	all2748	53.9	2.38e-06	COG0666	pfam12796	Ank_2	Ankyrin repeats (3 copies).	GO:0097193|intrinsic apoptotic signaling pathway;GO:0072332|intrinsic apoptotic signaling pathway by p53 class mediator;GO:0045786|negative regulation of cell cycle;GO:1901216|positive regulation of neuron death;	GO:0005737|cytoplasm;GO:0005654|nucleoplasm;GO:0005634|nucleus;GO:0048471|perinuclear region of cytoplasm;GO:0005886|plasma membrane;	GO:0002039|p53 binding;	K17554	PPP1R13B, ASPP1; apoptosis-stimulating of p53 protein 1
LYPLA1	986.3023	-0.41755	0.140923964	-2.96295	0.0030471	0.023769	Not_Sig	no	gi|926708517|ref|XP_013824643.1|	1247	3e-161	PREDICTED: acyl-protein thioesterase 1 [Capra hircus]	gi|741949971|ref|XM_005215419.2|	2417	0	PREDICTED: Bos taurus lysophospholipase I (LYPLA1), transcript variant X1, mRNA	Q3MHR0	473	7e-160	Acyl-protein thioesterase 1	SPAC8E11.04c	153	1.83e-41	COG0400	pfam02230	Abhydrolase_2	Phospholipase/Carboxylesterase. This family consists of both phospholipases and carboxylesterases with broad	GO:0006631|fatty acid metabolic process;GO:0002084|protein depalmitoylation;	GO:0005737|cytoplasm;	GO:0052689|carboxylic ester hydrolase activity;GO:0008474|palmitoyl-(protein)	K06128	LYPLA1; lysophospholipase I [EC:3.1.1.5]
SMNDC1	349.6587	-0.42549	0.143617181	-2.96265	0.0030501	0.023774	Not_Sig	no	gi|395828102|ref|XP_003787225.1|	1097	3e-138	PREDICTED: survival of motor neuron-related-splicing factor 30 [Otolemur garnettii]	gi|965998450|ref|XM_012102912.2|	2657	0	PREDICTED: Ovis aries survival motor neuron domain containing 1 (SMNDC1), transcript variant X2, mRNA	Q5R591	425	3e-139	Survival of motor neuron-related-splicing factor 30	.	.	.	.	pfam06003	SMN	Survival motor neuron protein (SMN). This family consists of several eukaryotic survival motor neuron (SMN) proteins. The	GO:0006915|apoptotic process;GO:0006397|mRNA processing;GO:0008380|RNA splicing;	GO:0015030|Cajal body;GO:0005737|cytoplasm;GO:0016607|nuclear speck;GO:0005681|spliceosomal complex;	GO:0044822|poly (A) RNA binding;	K12839	SMNDC1, SPF30; survival of motor neuron-related-splicing factor 30
MSTO1	180.5182	0.508052	0.171497105	2.962455	0.003052	0.023774	Not_Sig	no	gi|742111394|ref|XP_010836570.1|	2775	0	PREDICTED: protein misato homolog 1 isoform X1 [Bison bison bison]	gi|742111393|ref|XM_010838268.1|	1813	0	PREDICTED: Bison bison bison misato 1, mitochondrial distribution and morphology regulator (MSTO1), transcript variant X1, mRNA	A5D9D4	1071	0	Protein misato homolog 1	.	.	.	.	pfam10644	Misat_Tub_SegII	Misato Segment II tubulin-like domain. The misato protein contains three distinct, conserved domains, segments I, II and III.	GO:0048311|mitochondrion distribution;GO:0007005|mitochondrion organization;	GO:0005741|mitochondrial outer membrane;	GO:0003924|GTPase activity;	.	.
RAB33B	377.1805	-0.4333	0.146304638	-2.96163	0.0030602	0.023806	Not_Sig	no	gi|742229951|ref|XP_010828590.1|	1461	0	PREDICTED: ras-related protein Rab-33B isoform X1 [Bison bison bison]	gi|942054628|ref|XM_005895822.2|	3400	0	PREDICTED: Bos mutus RAB33B, member RAS oncogene family (RAB33B), mRNA	Q5R615	428	7e-140	Ras-related protein Rab-33B	.	.	.	.	pfam00071	Ras	Ras family. Includes sub-families Ras, Rab, Rac, Ral, Ran, Rap Ypt1 and more. Shares P-loop motif with GTP_EFTU, arf and	GO:0006914|autophagy;GO:1903434|negative regulation of constitutive secretory pathway;GO:0000042|protein targeting to Golgi;GO:2000785|regulation of autophagosome assembly;GO:1903358|regulation of Golgi organization;GO:2000156|regulation of retrograde vesicle-mediated transport, Golgi to ER;GO:0048705|skeletal system morphogenesis;GO:0007264|small GTPase mediated signal transduction;	GO:0070062|extracellular exosome;GO:0005796|Golgi lumen;GO:0000139|Golgi membrane;	GO:0005525|GTP binding;GO:0003924|GTPase activity;	K07920	RAB33B; Ras-related protein Rab-33B
SOCS3	164.7716	0.52406	0.176950076	2.961624	0.0030602	0.023806	Not_Sig	no	gi|27806151|ref|NP_776891.1|	1045	1e-132	suppressor of cytokine signaling 3 [Bos taurus]	gi|982958517|ref|XM_005221139.3|	2274	0	PREDICTED: Bos taurus suppressor of cytokine signaling 3 (SOCS3), transcript variant X1, mRNA	Q9BEG9	407	4e-134	Suppressor of cytokine signaling 3	.	.	.	.	pfam00017	SH2	SH2 domain.	GO:0060670|branching involved in labyrinthine layer morphogenesis;GO:0019221|cytokine-mediated signaling pathway;GO:0007259|JAK-STAT cascade;GO:0043066|negative regulation of apoptotic process;GO:0050728|negative regulation of inflammatory response;GO:0046627|negative regulation of insulin receptor signaling pathway;GO:0046426|negative regulation of JAK-STAT cascade;GO:0006469|negative regulation of protein kinase activity;GO:0060674|placenta blood vessel development;GO:0045597|positive	GO:0005737|cytoplasm;	GO:0004860|protein kinase inhibitor activity;	K04696	SOCS3, CIS3; suppressor of cytokine signaling 3
PES1	1352.694	0.317163	0.107115337	2.960948	0.0030669	0.023829	Not_Sig	no	gi|594093082|ref|XP_006069869.1|	2709	0	PREDICTED: pescadillo homolog isoform X1 [Bubalus bubalis]	gi|594093081|ref|XM_006069807.1|	2178	0	PREDICTED: Bubalus bubalis pescadillo ribosomal biogenesis factor 1 (PES1), transcript variant X1, mRNA	O00541	936	0	Pescadillo homolog	SPBC19F5.05c	449	3.32e-148	COG5163	pfam06732	Pescadillo_N	Pescadillo N-terminus. This family represents the N-terminal region of Pescadillo. Pescadillo protein localizes to distinct substructures	GO:0008283|cell proliferation;GO:0000466|maturation of 5.8S rRNA from tricistronic rRNA transcript (SSU-rRNA, 5.8S rRNA, LSU-rRNA);GO:0000463|maturation of LSU-rRNA from tricistronic rRNA transcript (SSU-rRNA, 5.8S rRNA, LSU-rRNA);GO:0007000|nucleolus organization;GO:0033365|protein localization to organelle;GO:0051726|regulation of cell cycle;GO:0042273|ribosomal large subunit biogenesis;GO:0006364|rRNA processing;	GO:0000793|condensed chromosome;GO:0005737|cytoplasm;GO:0016020|membrane;GO:0005730|nucleolus;GO:0005654|nucleoplasm;GO:0005634|nucle	GO:0044822|poly (A) RNA binding;GO:0043021|ribonucleoprotein	K14843	PES1, NOP7; pescadillo
DGKH	621.8677	-0.43842	0.148068236	-2.96091	0.0030673	0.023829	Not_Sig	no	gi|965855163|ref|XP_014963595.1|	5560	0	PREDICTED: diacylglycerol kinase eta isoform X8 [Ovis aries musimon]	gi|982991869|ref|XM_015465681.1|	5192	0	PREDICTED: Bos taurus diacylglycerol kinase eta (DGKH), transcript variant X6, mRNA	Q86XP1	2028	0	Diacylglycerol kinase eta	.	.	.	.	pfam00609	DAGK_acc	Diacylglycerol kinase accessory domain. Diacylglycerol (DAG) is a second messenger that acts as a protein kinase C activator. This	GO:0035556|intracellular signal transduction;GO:0030168|platelet activation;GO:0007205|protein kinase C-activating G-protein coupled receptor signaling pathway;GO:0051259|protein oligomerization;	GO:0005737|cytoplasm;GO:0005768|endosome;GO:0031965|nuclear membrane;GO:0005634|nucleus;GO:0005886|plasma membrane;	GO:0005524|ATP binding;GO:0004143|diacylglycerol kinase	K00901	dgkA, DGK; diacylglycerol kinase (ATP) [EC:2.7.1.107]
LOC138985981	80.49835	-0.78047	0.263617596	-2.96061	0.0030703	0.023836	DOWN	yes	gi|611991798|ref|XP_001364419.2|	620	5e-77	PREDICTED: uncharacterized protein LOC100012315 [Monodelphis domestica]	gi|73587392|gb|BC103170.1|	665	0	Bos taurus eukaryotic translation initiation factor 1, mRNA (cDNA clone MGC:128453 IMAGE:7984251), complete cds	Q5RFF4	204	3e-65	Eukaryotic translation initiation factor 1	YNL244c	122	3.36e-35	COG0023	pfam01253	SUI1	Translation initiation factor SUI1.	.	.	GO:0003743|translation initiation factor activity;	K03113	EIF1, SUI1; translation initiation factor 1
RICTOR	394.6091	-0.44991	0.151982216	-2.96028	0.0030736	0.023846	Not_Sig	no	gi|742178049|ref|XP_010852047.1|	8266	0	PREDICTED: rapamycin-insensitive companion of mTOR isoform X1 [Bison bison bison]	gi|742178058|ref|XM_010853748.1|	9846	0	PREDICTED: Bison bison bison RPTOR independent companion of MTOR, complex 2 (RICTOR), transcript variant X4, mRNA	Q6R327	3075	0	Rapamycin-insensitive companion of mTOR	.	.	.	.	pfam14664	RICTOR_N	Rapamycin-insensitive companion of mTOR, N-term. Rictor appears to serve as a scaffolding protein that is important for maintaining	GO:0031532|actin cytoskeleton reorganization;GO:0009790|embryo development;GO:0030010|establishment of cell polarity;GO:0030950|establishment or maintenance of actin cytoskeleton polarity;GO:0018105|peptidyl-serine phosphorylation;GO:0048015|phosphatidylinositol-mediated signaling;GO:0030838|positive regulation of actin filament polymerization;GO:0001938|positive regulation of endothelial cell proliferation;GO:0050731|positive regulation of peptidyl-tyrosine	GO:0005829|cytosol;GO:0031932|TORC2 complex;	GO:0008047|enzyme activator activity;GO:0019901|protein kinase	K08267	RICTOR; rapamycin-insensitive companion of mTOR

PDE7A	88.66492	0.662212	0.223714485	2.960075	0.0030756	0.023846	UP	yes	gi|358415372|ref|XP_003583087.1|	2500	0	PREDICTED: high affinity cAMP-specific 3',5'-cyclic phosphodiesterase 7A isoform X1 [Bos taurus]	gi|942047016|ref|XM_005892949.2|	3111	0	PREDICTED: Bos mutus phosphodiesterase 7A (PDE7A), mRNA
LOC106701720	3910.896	-0.28717	0.097039656	-2.95928	0.0030836	0.023887	Not_Sig	no	gi|72536919|gb|AAZ73479.1|	1535	0	MHC class I antigen [Bos taurus]	gi|942074733|ref|XM_014480353.1|	1790	0	PREDICTED: Bos mutus BOLA class I histocompatibility antigen, alpha chain BL3-7 (LOC102268170), mRNA
GSTM3	457.3224	-0.39008	0.131823456	-2.95912	0.0030851	0.023887	Not_Sig	no	gi|114053087|ref|NP_001040025.1|	1218	4e-167	glutathione S-transferase Mu 3 [Bos taurus]	gi|942042299|ref|XM_005891293.2|	869	0	PREDICTED: Bos mutus glutathione S-transferase mu 3 (brain) (GSTM3), mRNA
GTF3A	396.703	0.44849	0.15158598	2.958649	0.0030899	0.023908	Not_Sig	no	gi|555980660|ref|XP_005902254.1|	2042	0	PREDICTED: transcription factor IIIA [Bos mutus]	gi|942073863|ref|XM_005902192.2|	1420	0	PREDICTED: Bos mutus general transcription factor IIIA (GTF3A), mRNA
SEC61G	1188.465	-1.12983	0.382170568	-2.95634	0.0031131	0.024059	DOWN	yes	gi|742133411|ref|XP_010840341.1|	400	8e-47	PREDICTED: protein transport protein Sec61 subunit gamma [Bison bison bison]	gi|742133410|ref|XM_010842039.1|	518	0	PREDICTED: Bison bison bison Sec61 gamma subunit (SEC61G), mRNA
PCIF1	288.1384	0.445812	0.150801501	2.956287	0.0031137	0.024059	Not_Sig	no	gi|625193988|ref|XP_003500578.2|	3539	0	PREDICTED: phosphorylated CTD-interacting factor 1 isoform X1 [Cricetulus griseus]	gi|982943161|ref|XM_005214797.3|	2658	0	PREDICTED: Bos taurus PDX1 C-terminal inhibiting factor 1 (PCIF1), transcript variant X1, mRNA
NSMF	481.0879	0.400591	0.13553565	2.955617	0.0031204	0.02408	Not_Sig	no	gi|594077843|ref|XP_006062634.1|	2567	0	PREDICTED: NMDA receptor synaptonuclear signaling and neuronal migration factor isoform X2 [Bubalus bubalis]	gi|594077842|ref|XM_006062572.1|	3040	0	PREDICTED: Bubalus bubalis NMDA receptor synaptonuclear signaling and neuronal migration factor (NSMF), transcript variant X2, mRNA
KDM7A	205.4511	-0.49808	0.168533334	-2.95538	0.0031229	0.02408	Not_Sig	no	gi|982981343|ref|XP_015318695.1|	4638	0	PREDICTED: LOW QUALITY PROTEIN: lysine-specific demethylase 7A isoform X2 [Bos taurus]	gi|982981342|ref|XM_015463209.1|	9227	0	PREDICTED: Bos taurus lysine (K)-specific demethylase 7A (KDM7A), transcript variant X2, mRNA
ZC3H3	143.0131	0.572827	0.19382875	2.955326	0.0031234	0.02408	Not_Sig	no	gi|982994146|ref|XP_015321754.1|	3362	0	PREDICTED: zinc finger CCCH domain-containing protein 3 isoform X2 [Bos taurus]	gi|982994145|ref|XM_015466268.1|	2919	0	PREDICTED: Bos taurus zinc finger CCCH-type containing 3 (ZC3H3), transcript variant X2, mRNA
LOC138990480	109.4468	-0.84173	0.284829659	-2.95519	0.0031247	0.02408	DOWN	yes	gi|10834786|gb|AAG23823.1|AF275813_1	218	7e-20	PNAS-117 [Homo sapiens]	gi|982942035|ref|XM_010811036.2|	393	0	PREDICTED: Bos taurus McKusick-Kaufman syndrome (MKKS), transcript variant X1, mRNA
NR3C1	963.6576	-0.33413	0.113088494	-2.95459	0.0031309	0.024111	Not_Sig	no	gi|528957121|ref|XP_005209585.1|	3840	0	PREDICTED: glucocorticoid receptor isoform X1 [Bos taurus]	gi|741933955|ref|XM_005209531.2|	6343	0	PREDICTED: Bos taurus nuclear receptor subfamily 3 group C member 1 (NR3C1), transcript variant X4, mRNA
SAMD1	368.7977	0.396429	0.134198247	2.954051	0.0031363	0.024128	Not_Sig	no	gi|555951778|ref|XP_005888084.1|	834	1e-101	PREDICTED: atherin, partial [Bos mutus]	gi|965934593|ref|XM_015095611.1|	1957	0	PREDICTED: Ovis aries sterile alpha motif domain containing 1 (SAMD1), transcript variant X1, mRNA
MAP1S	283.7895	0.47588	0.161105289	2.953843	0.0031384	0.024128	Not_Sig	no	gi|296486071|tpg|DAA28184.1|	4372	0	TPA: microtubule-associated protein 1S [Bos taurus]	gi|742149356|ref|XM_010845639.1|	4711	0	PREDICTED: Bison bison bison microtubule-associated protein 1S (MAP1S), mRNA
PPIP5K1	180.6083	0.470419	0.159261738	2.953746	0.0031394	0.024128	Not_Sig	no	gi|528999894|ref|XP_005222239.1|	7613	0	PREDICTED: inositol hexakisphosphate and diphosphoinositol-pentakisphosphate kinase 1 isoform X1 [Bos taurus]	gi|982960704|ref|XM_005222182.3|	5718	0	PREDICTED: Bos taurus diphosphoinositol pentakisphosphate kinase 1 (PPIP5K1), transcript variant X1, mRNA
UNC93B1	384.6638	0.488411	0.165374933	2.953355	0.0031434	0.024143	Not_Sig	no	gi|300798093|ref|NP_001180076.1|	2363	0	protein unc-93 homolog B1 [Bos taurus]	gi|300798092|ref|NM_001193147.1|	2228	0	Bos taurus unc-93 homolog B1 (C. elegans) (UNC93B1), mRNA
AACS	647.265	0.336646	0.113996713	2.953118	0.0031458	0.024145	Not_Sig	no	gi|555973771|ref|XP_005898881.1|	3607	0	PREDICTED: acetoacetyl-CoA synthetase [Bos mutus]	gi|942063410|ref|XM_005898819.2|	3206	0	PREDICTED: Bos mutus acetoacetyl-CoA synthetase (AACS), mRNA
TMEM201	106.7342	0.819965	0.277814302	2.951486	0.0031625	0.024245	UP	yes	gi|528979892|ref|XP_005217010.1|	2699	0	PREDICTED: transmembrane protein 201 isoform X1 [Bos taurus]	gi|982947899|ref|XM_005216953.3|	2176	0	PREDICTED: Bos taurus transmembrane protein 201 (TMEM201), transcript variant X1, mRNA
ARHGAP19	383.7871	-0.37627	0.127496904	-2.95125	0.003165	0.024245	Not_Sig	no	gi|982968268|ref|XP_015316192.1|	2397	0	PREDICTED: rho GTPase-activating protein 19 isoform X1 [Bos taurus]	gi|742089237|ref|XM_010833048.1|	2929	0	PREDICTED: Bison bison bison Rho GTPase activating protein 19 (ARHGAP19), transcript variant X1, mRNA
NAA40	487.8266	0.397852	0.134809375	2.951222	0.0031652	0.024245	Not_Sig	no	gi|926727470|ref|XP_005699898.2|	1245	3e-159	PREDICTED: N-alpha-acetyltransferase 40 [Capra hircus]	gi|555964256|ref|XM_005894136.1|	3100	0	PREDICTED: Bos mutus N(alpha)-acetyltransferase 40, NatD catalytic subunit (NAA40), transcript variant X1, mRNA
RAD9A	128.5861	0.577862	0.195858528	2.950405	0.0031736	0.024293	Not_Sig	no	gi|555983438|ref|XP_005903618.1|	1851	0	PREDICTED: cell cycle checkpoint control protein RAD9A isoform X1 [Bos mutus]	gi|942077932|ref|XM_005903556.2|	1855	0	PREDICTED: Bos mutus RAD9 checkpoint clamp component A (RAD9A), transcript variant X1, mRNA
TBCD	775.1978	0.327108	0.11089156	2.949804	0.0031798	0.024325	Not_Sig	no	gi|555951271|ref|XP_005887834.1|	5561	0	PREDICTED: tubulin-specific chaperone D isoform X1 [Bos mutus]	gi|942032832|ref|XM_005887772.2|	3875	0	PREDICTED: Bos mutus tubulin folding cofactor D (TBCD), transcript variant X1, mRNA
ERBIN	2617.038	-0.31497	0.106799666	-2.94917	0.0031863	0.024358	Not_Sig	no	gi|742148992|ref|XP_010843838.1|	7331	0	PREDICTED: protein LAP2 isoform X1 [Bison bison bison]	gi|982959267|ref|XM_005221451.3|	6713	0	PREDICTED: Bos taurus erbb2 interacting protein (ERBB2IP), transcript variant X1, mRNA
LOC102274319	2073.694	-0.32602	0.110591969	-2.94798	0.0031986	0.024436	Not_Sig	no	gi|77736600|ref|NP_001029984.1|	1112	8e-148	transmembrane emp24 domain-containing protein 9 precursor [Bos taurus]	gi|942081251|ref|XM_005904617.2|	1416	0	PREDICTED: Bos mutus transmembrane emp24 domain-containing protein 9 (LOC102274319), mRNA
COX7A2	1006.687	-0.90741	0.307882464	-2.94726	0.0032061	0.024472	DOWN	yes	gi|752423922|ref|XP_002925517.2|	452	4e-54	PREDICTED: LOW QUALITY PROTEIN: cytochrome c oxidase subunit 7A2, mitochondrial [Ailuropoda melanoleuca]	gi|942076001|ref|XM_005902812.2|	535	0	PREDICTED: Bos mutus cytochrome c oxidase subunit 7A2, mitochondrial (LOC102278351), mRNA
COX5A	570.7707	-0.40095	0.136047221	-2.94711	0.0032076	0.024472	Not_Sig	no	gi|965858675|ref|XP_014963830.1|	725	7e-94	PREDICTED: cytochrome c oxidase subunit 5A, mitochondrial [Ovis aries musimon]	gi|742137910|ref|XM_010843342.1|	711	0	PREDICTED: Bison bison bison cytochrome c oxidase subunit 5A, mitochondrial (LOC104990981), mRNA
ARHGEF18	422.3468	0.362246	0.122937728	2.94658	0.0032131	0.024498	Not_Sig	no	gi|982930577|ref|XP_015327516.1|	5791	0	PREDICTED: rho guanine nucleotide exchange factor 18 isoform X1 [Bos taurus]	gi|982984674|ref|XM_010798244.2|	6083	0	PREDICTED: Bos taurus Rho/Rac guanine nucleotide exchange factor 18 (ARHGEF18), transcript variant X2, mRNA
CMTR2	304.1941	-0.43443	0.147447438	-2.94633	0.0032157	0.024502	Not_Sig	no	gi|742168089|ref|XP_010849199.1|	4108	0	PREDICTED: cap-specific mRNA (nucleoside-2'-O-)-methyltransferase 2 [Bison bison bison]	gi|942065710|ref|XM_005899535.2|	3795	0	PREDICTED: Bos mutus cap methyltransferase 2 (CMTR2), mRNA
CYP51A1	2736.783	-0.30862	0.104765056	-2.94583	0.0032209	0.024525	Not_Sig	no	gi|440894334|gb|ELR46811.1|	2629	0	Lanosterol 14-alpha demethylase, partial [Bos mutus]	gi|555994253|ref|XM_005908819.1|	2398	0	PREDICTED: Bos mutus lanosterol 14-alpha demethylase (LOC102282237), mRNA
NIPA2	1377.341	-0.39139	0.132909103	-2.94481	0.0032315	0.024568	Not_Sig	no	gi|77735549|ref|NP_001029470.1|	1437	0	magnesium transporter NIPA2 [Bos taurus]	gi|742175337|ref|XM_010852980.1|	2284	0	PREDICTED: Bison bison bison non imprinted in Prader-Willi/Angelman syndrome 2 (NIPA2), transcript variant X10, mRNA
MBTPS1	2036.585	0.322167	0.109409267	2.944604	0.0032337	0.024568	Not_Sig	no	gi|166157453|ref|NP_001020501.2|	5355	0	membrane-bound transcription factor site-1 protease precursor [Bos taurus]	gi|742167793|ref|XM_010850812.1|	4187	0	PREDICTED: Bison bison bison membrane-bound transcription factor peptidase, site 1 (MBTPS1), transcript variant X1, mRNA
PPP1CB	3168.156	-0.38618	0.131158406	-2.94438	0.0032361	0.024568	Not_Sig	no	gi|50513466|pdb|1S70|A	1754	0	Chain A, Complex Between Protein Ser/thr Phosphatase-1 (delta) And The Myosin Phosphatase Targeting Subunit 1 (mypt1)	gi|942078841|ref|XM_005903859.2|	2765	0	PREDICTED: Bos mutus protein phosphatase 1, catalytic subunit, beta isozyme (PPP1CB), mRNA
LRIF1	300.4406	-0.43045	0.146201227	-2.94426	0.0032372	0.024568	Not_Sig	no	gi|742232210|ref|XP_010829159.1|	3642	0	PREDICTED: ligand-dependent nuclear receptor-interacting factor 1 isoform X1 [Bison bison bison]	gi|594073830|ref|XM_006060667.1|	4422	0	PREDICTED: Bubalus bubalis ligand dependent nuclear receptor interacting factor 1 (LRIF1), transcript variant X1, mRNA
DDI2	1985.845	-0.30802	0.104618161	-2.94425	0.0032374	0.024568	Not_Sig	no	gi|742164941|ref|XP_010848344.1|	4799	0	PREDICTED: regulatory solute carrier protein family 1 member 1 isoform X1 [Bison bison bison]	gi|742164940|ref|XM_010850042.1|	9288	0	PREDICTED: Bison bison bison protein DDI1 homolog 2 (LOC104995963), transcript variant X1, mRNA
RPL19	6431.515	-0.32569	0.110625273	-2.94406	0.0032394	0.024568	Not_Sig	no	gi|655871008|ref|XP_008269617.1|	674	7e-86	PREDICTED: LOW QUALITY PROTEIN: 60S ribosomal protein L19 [Oryctolagus cuniculus]	gi|942060445|ref|XM_005897992.2|	709	0	PREDICTED: Bos mutus ribosomal protein L19 (RPL19), transcript variant X1, mRNA
SPCS3	1716.835	-0.38617	0.131223813	-2.94286	0.0032519	0.024647	Not_Sig	no	gi|926725527|ref|XP_013830814.1|	1097	1e-135	PREDICTED: signal peptidase complex subunit 3 [Capra hircus]	gi|742228447|ref|XM_010829916.1|	3755	0	PREDICTED: Bison bison bison signal peptidase complex subunit 3 homolog (S. cerevisiae) (SPCS3), mRNA
DOLPP1	201.8366	0.515296	0.175122201	2.942495	0.0032558	0.024652	Not_Sig	no	gi|674038212|ref|XP_008824238.1|	1219	6e-160	PREDICTED: dolichyldiphosphatase 1 isoform X1 [Nannospalax galili]	gi|942094512|ref|XM_005908780.2|	2170	0	PREDICTED: Bos mutus dolichyldiphosphatase 1 (DOLPP1), transcript variant X1, mRNA
RBIS	240.685	-0.6391	0.217222096	-2.94217	0.0032592	0.024652	DOWN	yes	gi|158519815|ref|NP_001103538.1|	509	5e-63	uncharacterized protein C8orf59 homolog [Bos taurus]	gi|942095333|ref|XM_005909090.2|	483	0	PREDICTED: Bos mutus chromosome unknown open reading frame, human C8orf59 (LOC102281394), transcript variant X1, mRNA
CDK19	477.4174	-0.37784	0.128423186	-2.94216	0.0032593	0.024652	Not_Sig	no	gi|28972574|dbj|BAC65703.1|	2234	0	mKIAA1028 protein [Mus musculus]	gi|942039691|ref|XM_005890225.2|	5662	0	PREDICTED: Bos mutus cyclin-dependent kinase 19 (CDK19), mRNA
KANSL1	799.2591	0.418293	0.142180443	2.941984	0.0032612	0.024652	Not_Sig	no	gi|359077044|ref|XP_003587505.1|	5135	0	PREDICTED: KAT8 regulatory NSL complex subunit 1 isoform X1 [Bos taurus]	gi|742086391|ref|XM_010832380.1|	4987	0	PREDICTED: Bison bison bison KAT8 regulatory NSL complex subunit 1 (KANSL1), transcript variant X3, mRNA

PDE7A	Q13946	935	0	High affinity cAMP-specific 3',5'-cyclic phosphodiesterase 7A	.	.	.	.	pfam00233	PDEase_I	3'5'-cyclic nucleotide phosphodiesterase.	GO:0006198|cAMP catabolic process;GO:0007165|signal transduction;	GO:0005829|cytosol;	GO:0004115|3',5'-cyclic-AMP phosphodiesterase activity;GO:0046872	K18436	PDE7; high affinity cAMP-specific 3',5'-cyclic phosphodiesterase 7
LOC106701720	P13753	592	0	BOLA class I histocompatibility antigen, alpha chain BL3-7	.	.	.	.	pfam00129	MHC_I	Class I Histocompatibility antigen, domains alpha 1 and 2.	GO:0002474|antigen processing and presentation of peptide antigen via MHC class I;GO:0006955|immune response;	GO:0016021|integral component of membrane;GO:0042612|MHC class I protein complex;	.	.	.
GSTM3	Q9Z1B2	436	1e-153	Glutathione S-transferase Mu 5	.	.	.	.	pfam00043	GST_C	Glutathione S-transferase, C-terminal domain. GST conjugates reduced glutathione to a variety of targets including S-crystallin from	GO:0006749|glutathione metabolic process;	GO:0005737|cytoplasm;	GO:0004364|glutathione transferase activity;GO:0042803|protein	K00799	GST, gst; glutathione S-transferase [EC:2.5.1.18]
GTF3A	Q92664	556	0	Transcription factor IIIA	SPAC144.09c	156	6.25e-42	COG5048	pfam12756	zf-C2H2_2	C2H2 type zinc-finger (2 copies). This family contains two copies of a C2H2-like zinc finger domain.	GO:0006355|regulation of transcription, DNA-templated;GO:0009303|rRNA transcription;GO:0006383|transcription from RNA polymerase III promoter;	GO:0005654|nucleoplasm;GO:0005634|nucleus;	GO:0003677|DNA binding;GO:0046872|metal ion binding;GO:0003723	K09191	GTF3A; general transcription factor IIIA
SEC61G	P60060	81.3	9e-19	Protein transport protein Sec61 subunit gamma	.	.	.	.	pfam00584	SecE	SecE/Sec61-gamma subunits of protein translocation complex. SecE is part of the SecYEG complex in bacteria which translocates	GO:0006605|protein targeting;	GO:0005789|endoplasmic reticulum membrane;GO:0016021|integral component of membrane;GO:0016020|membrane;	GO:0015450|P-P-bond-hydrolysis-driven protein transmembrane	K07342	SEC61G, SSS1, secE; protein transport protein SEC61 subunit gamma and
PCIF1	Q9H4Z3	1299	0	Phosphorylated CTD-interacting factor 1	.	.	.	.	pfam12237	PCIF1_WW	Phosphorylated CTD interacting factor 1 WW domain. This domain family is found in bacteria and eukaryotes, and is approximately	GO:0010923|negative regulation of phosphatase activity;	GO:0045171|intercellular bridge;GO:0015630|microtubule cytoskeleton;GO:0005654|nucleoplasm;	.	K17584	PCIF1; phosphorylated CTD-interacting factor 1
NSMF	Q6X4W1	920	0	NMDA receptor synaptonuclear signaling and neuronal migration factor	.	.	.	.	pfam05934	MCLC	Mid-1-related chloride channel (MCLC). This family consists of several mid-1-related chloride channels. mid-1-related chloride	GO:0071230|cellular response to amino acid stimulus;GO:0071257|cellular response to electrical stimulus;GO:0071371|cellular response to gonadotropin stimulus;GO:2001224|positive regulation of neuron migration;GO:0035307|positive regulation of protein dephosphorylation;GO:0048814|regulation of dendrite morphogenesis;GO:0043523|regulation of neuron apoptotic process;GO:0048168|regulation of neuronal synaptic plasticity;	GO:0097440|apical dendrite;GO:0030054|cell junction;GO:0030863|cortical cytoskeleton;GO:0005737|cytoplasm;GO:00	GO:0048306|calcium-dependent protein binding;	.	.
KDM7A	Q6ZMT4	1689	0	Lysine-specific demethylase 7A	.	.	.	.	pfam02373	JmjC	JmjC domain, hydroxylase. The JmjC domain belongs to the Cupin superfamily. JmjC-domain proteins may be protein hydroxylases that	GO:0071557|histone H3-K27 demethylation;GO:0070544|histone H3-K36 demethylation;GO:0033169|histone H3-K9 demethylation;GO:0035574|histone H4-K20 demethylation;GO:0030901|midbrain development;GO:0045893|positive regulation of transcription, DNA-templated;GO:0006351|transcription, DNA-templated;	GO:0005730|nucleolus;GO:0005654|nucleoplasm;GO:0005634|nucleus;	GO:0032452|histone demethylase activity;GO:0071558|histone	K11445	KDM7A, JHDM1D; lysine-specific demethylase 7A [EC:1.14.11.-]
ZC3H3	Q8IXZ2	873	0	Zinc finger CCCH domain-containing protein 3	SPBC337.12	112	6.85e-26	COG5084	pfam00642	zf-CCCH	Zinc finger C-x8-C-x5-C-x3-H type (and similar).	GO:0006378|mRNA polyadenylation;GO:0016973|poly(A)+ mRNA export from nucleus;GO:0032927|positive regulation of activin receptor signaling pathway;GO:0010793|regulation of mRNA export from nucleus;	GO:0005847|mRNA cleavage and polyadenylation specificity factor complex;GO:0005634|nucleus;	GO:0003677|DNA binding;GO:0004521|endoribonuclease activity;GO:0046872	.	.
LOC138990480	.	.	.	.	.	.	.	.	.	.	.	.	.	.	.	.
NR3C1	Q9N1U3	1379	0	Glucocorticoid receptor	.	.	.	.	pfam02155	GCR	Glucocorticoid receptor.	GO:0071383|cellular response to steroid hormone stimulus;GO:0016569|covalent chromatin modification;GO:0045944|positive regulation of transcription from RNA polymerase II promoter;GO:0006351|transcription, DNA-templated;	GO:0005737|cytoplasm;GO:0005815|microtubule organizing center;GO:0005739|mitochondrion;GO:0005634|nucleus;GO:0005819|spindle;	GO:0038051|glucocorticoid-activated RNA polymerase II transcription factor	K05771	NR3C1, GR; glucocorticoid receptor
SAMD1	Q6SPF0	320	2e-97	Atherin	.	.	.	.	pfam00536	SAM_1	SAM domain (Sterile alpha motif). It has been suggested that SAM is an evolutionarily conserved protein binding domain that is	.	GO:0005737|cytoplasm;GO:0005576|extracellular region;	.	.	.
MAP1S	A6QQ70	1684	0	Microtubule-associated protein 1S	.	.	.	.	pfam15449	Retinal	Retinal protein. This family of proteins is found in the photoreceptor cells of the retina. Mutations of the gene encoding	GO:0006915|apoptotic process;GO:0000226|microtubule cytoskeleton organization;	GO:0005829|cytosol;GO:0005874|microtubule;GO:0005634|nucleus;GO:0005819|spindle;	GO:0003677|DNA binding;	K10429	MAP1; microtubule-associated protein 1
PPIP5K1	A7Z050	2082	0	Inositol hexakisphosphate and diphosphoinositol-pentakisphosphate kinase 1	.	.	.	.	pfam00328	His_Phos_2	Histidine phosphatase superfamily (branch 2). The histidine phosphatase superfamily is so named because catalysis centres	GO:0006020|inositol metabolic process;GO:0032958|inositol phosphate biosynthetic process;	GO:0005829|cytosol;GO:0005654|nucleoplasm;GO:0005886|plasma membrane;	GO:0003993|acid phosphatase activity;GO:0005524|ATP	K13024	PPIP5K, VIP; inositol-hexakisphosphate/diphosphoinositol-pentakisphosphate
UNC93B1	Q9H1C4	863	0	Protein unc-93 homolog B1	.	.	.	.	pfam05978	UNC-93	Ion channel regulatory protein UNC-93. This family of proteins is a component of a multi-subunit protein complex which is involved	GO:0002250|adaptive immune response;GO:0051607|defense response to virus;GO:0045087|innate immune response;GO:0006886|intracellular protein transport;GO:0034138|toll-like receptor 3 signaling pathway;GO:0034154|toll-like receptor 7 signaling pathway;GO:0034162|toll-like receptor 9 signaling pathway;GO:0002224|toll-like receptor signaling pathway;	GO:0032009|early phagosome;GO:0005783|endoplasmic reticulum;GO:0005789|endoplasmic reticulum	GO:0035325|Toll-like receptor binding;	.	.
AACS	Q9D2R0	1302	0	Acetoacetyl-CoA synthetase	PA1997	577	0	COG0365	pfam00501	AMP-binding	AMP-binding enzyme.	GO:0060612|adipose tissue development;GO:0071397|cellular response to cholesterol;GO:0071333|cellular response to glucose stimulus;GO:0071394|cellular response to testosterone stimulus;GO:0006631|fatty acid metabolic process;GO:0001889|liver development;GO:0032024|positive regulation of insulin secretion;GO:0042493|response to drug;GO:0045471|response to ethanol;GO:0007584|response to nutrient;GO:0034201|response to oleic acid;GO:0014074|response to purine-containing	GO:0005829|cytosol;	GO:0030729|acetoacetate-CoA ligase activity;GO:0005524|ATP	K01907	AACS, acsA; acetoacetyl-CoA synthetase [EC:6.2.1.16]
TMEM201	Q5SNT2	874	0	Transmembrane protein 201	.	.	.	.	pfam10476	DUF2448	Protein of unknown function C-terminus (DUF2448). The family DUF2349 is the N-terminal part of this family. This protein is found in	GO:0010761|fibroblast migration;GO:0030473|nuclear migration along microtubule;	GO:0016021|integral component of membrane;GO:0005637|nuclear inner membrane;GO:0031965|nuclear membrane;	GO:0051015|actin filament binding;GO:0005521|lamin binding;	.	.
ARHGAP19	Q14CB8	854	0	Rho GTPase-activating protein 19	.	.	.	.	pfam00620	RhoGAP	RhoGAP domain. GTPase activator proteins towards Rho/Rac/Cdc42-like small GTPases.	GO:0051056|regulation of small GTPase mediated signal transduction;GO:0007165|signal transduction;	GO:0005829|cytosol;GO:0005634|nucleus;	GO:0005096|GTPase activator activity;	.	.
NAA40	Q8VE10	424	3e-138	N-alpha-acetyltransferase 40	.	.	.	.	pfam00583	Acetyltransf_1	Acetyltransferase (GNAT) family. This family contains proteins with N-acetyltransferase functions such as Elp3-related proteins.	GO:0006629|lipid metabolic process;	.	GO:0008080|N-acetyltransferase activity;	.	.
RAD9A	Q99638	586	0	Cell cycle checkpoint control protein RAD9A	.	.	.	.	pfam04139	Rad9	Rad9. Rad9 is required for transient cell-cycle arrests and transcriptional induction of DNA repair in response to DNA damage.	GO:0006974|cellular response to DNA damage stimulus;GO:0071479|cellular response to ionizing radiation;GO:0000077|DNA damage checkpoint;GO:0006281|DNA repair;GO:0006260|DNA replication;GO:0000076|DNA replication checkpoint;GO:0031573|intra-S DNA damage checkpoint;GO:1902231|positive regulation of intrinsic apoptotic signaling pathway in response to DNA damage;GO:1901796|regulation of signal transduction by p53 class mediator;	GO:0030896|checkpoint clamp complex;GO:0005737|cytoplasm;GO:0005654|nucleoplasm;GO:0005634|nucleus;	GO:0008408|3'-5' exonuclease activity;GO:0019899|enzyme	K10994	RAD9A; cell cycle checkpoint control protein RAD9A [EC:3.1.11.2]
TBCD	Q28205	2141	0	Tubulin-specific chaperone D	SPBC11C11.04c	202	3.78e-52	COG5234	pfam12612	TFCD_C	Tubulin folding cofactor D C terminal. This domain family is found in eukaryotes, and is typically between 182 and 199	GO:0034333|adherens junction assembly;GO:0070830|bicellular tight junction assembly;GO:0010812|negative regulation of cell-substrate adhesion;GO:0031115|negative regulation of microtubule polymerization;GO:0007023|post-chaperonin tubulin folding pathway;GO:0006457|protein folding;GO:0007021|tubulin complex assembly;	GO:0005912|adherens junction;GO:0005923|bicellular tight junction;GO:0005829|cytosol;GO:0016328|lateral plasma	GO:0048487|beta-tubulin binding;GO:0005096|GTPase activator	.	.
ERBIN	Q96RT1	2496	0	Erbin	alr0124_1	177	1.05e-46	COG4886	pfam00595	PDZ	PDZ domain (Also known as DHR or GLGF). PDZ domains are found in diverse signaling proteins.	GO:0045175|basal protein localization;GO:0007155|cell adhesion;GO:0007049|cell cycle;GO:0016049|cell growth;GO:0071356|cellular response to tumor necrosis factor;GO:0007173|epidermal growth factor receptor signaling pathway;GO:0038128|ERBB2 signaling pathway;GO:0045197|establishment or maintenance of epithelial cell apical/basal polarity;GO:0007229|integrin-mediated signaling pathway;GO:0045104|intermediate filament cytoskeleton organization;GO:0071638|negative regulation of monocyte chemotactic protein-1	GO:0009925|basal plasma membrane;GO:0005604|basement membrane;GO:0016323|basolateral plasma membrane;GO:0030054|cell	GO:0005176|ErbB-2 class receptor binding;GO:0005178|integrin	K12796	ERBB2IP, ERBIN; erbb2-interacting protein
LOC102274319	Q3T133	432	3e-149	Transmembrane emp24 domain-containing protein 9	.	.	.	.	pfam01105	EMP24_GP25L	emp24/gp25L/p24 family/GOLD. Members of this family are implicated in bringing cargo forward from the ER and binding	GO:0048205|COPI coating of Golgi vesicle;GO:0007030|Golgi organization;GO:0010638|positive regulation of organelle organization;GO:0015031|protein transport;	GO:0005789|endoplasmic reticulum membrane;GO:0033116|endoplasmic reticulum-Golgi intermediate compartment membrane;GO:0070062|extracellular	.	.	.
COX7A2	P13184	170	4e-53	Cytochrome c oxidase subunit 7A2, mitochondrial	.	.	.	.	pfam02238	COX7a	Cytochrome c oxidase subunit VIIa. Cytochrome c oxidase, a 13 sub-unit complex, is the terminal oxidase in the mitochondrial	.	GO:0005746|mitochondrial respiratory chain;	GO:0004129|cytochrome-c oxidase activity;	K02270	COX7A; cytochrome c oxidase subunit 7a
COX5A	P00426	278	1e-93	Cytochrome c oxidase subunit 5A, mitochondrial	.	.	.	.	pfam02284	COX5A	Cytochrome c oxidase subunit Va. Cytochrome c oxidase, a 13 sub-unit complex, EC:1.9.3.1 is the terminal oxidase in the	GO:0006123|mitochondrial electron transport, cytochrome c to oxygen;	GO:0070062|extracellular exosome;GO:0005743|mitochondrial inner membrane;GO:0005751|mitochondrial respiratory chain complex	GO:0004129|cytochrome-c oxidase activity;GO:0046872|metal ion binding;	K02264	COX5A; cytochrome c oxidase subunit 5a
ARHGEF18	Q6ZSZ5	1126	0	Rho guanine nucleotide exchange factor 18	.	.	.	.	pfam00621	RhoGEF	RhoGEF domain. Guanine nucleotide exchange factor for Rho/Rac/Cdc42-like GTPases Also called Dbl-homologous (DH)	GO:0030036|actin cytoskeleton organization;GO:0043065|positive regulation of apoptotic process;GO:0008360|regulation of cell shape;GO:0035023|regulation of Rho protein signal transduction;GO:0051056|regulation of small GTPase mediated signal transduction;GO:0007264|small GTPase mediated signal transduction;GO:0007179|transforming growth factor beta receptor signaling pathway;	GO:0045177|apical part of cell;GO:0016324|apical plasma membrane;GO:0030054|cell junction;GO:0005737|cytoplasm;GO:000585	GO:0005085|guanyl-nucleotide exchange factor activity;GO:0005089	K16529	AKAP13; A-kinase anchor protein 13
CMTR2	Q8IYT2	1400	0	Cap-specific mRNA (nucleoside-2'-O-)-methyltransferase 2	.	.	.	.	pfam01728	FtsJ	FtsJ-like methyltransferase. This family consists of FtsJ from various bacterial and archaeal sources FtsJ is a methyltransferase, but actually	GO:0006370|7-methylguanosine mRNA capping;GO:0097310|cap2 mRNA methylation;	GO:0005737|cytoplasm;GO:0016021|integral component of membrane;GO:0005634|nucleus;	GO:0004483|mRNA (nucleoside-2'-O-)-methyltransferase activity;	K14590	CMTR2, FTSJD1, AFT; cap2 methyltransferase [EC:2.1.1.296]
CYP51A1	Q4PJW3	999	0	Lanosterol 14-alpha demethylase	SPAC13A11.02c	336	4.98e-106	COG2124	pfam00067	p450	Cytochrome P450. Cytochrome P450s are haem-thiolate proteins involved in the oxidative degradation of various	GO:0033488|cholesterol biosynthetic process via 24,25-dihydrolanosterol;GO:0006694|steroid biosynthetic process;	GO:0005783|endoplasmic reticulum;GO:0005789|endoplasmic reticulum membrane;GO:0016021|integral component of	GO:0020037|heme binding;GO:0005506|iron ion binding;GO:0008398	K05917	CYP51; sterol 14-demethylase [EC:1.14.13.70]
NIPA2	Q3SWX0	558	0	Magnesium transporter NIPA2	.	.	.	.	pfam05653	Mg_trans_NIPA	Magnesium transporter NIPA. NIPA (nonimprinted in Prader-Willi/Angelman syndrome) is a family of integral membrane	GO:0015693|magnesium ion transport;	GO:0005769|early endosome;GO:0016021|integral component of membrane;GO:0016020|membrane;GO:000	GO:0015095|magnesium ion transmembrane transporter activity;	.	.
MBTPS1	Q14703	1995	0	Membrane-bound transcription factor site-1 protease	BS_aprE	110	5.85e-25	COG1404	pfam00082	Peptidase_S8	Subtilase family. Subtilases are a family of serine proteases. They appear to have independently and convergently evolved an	GO:0036500|ATF6-mediated unfolded protein response;GO:0008203|cholesterol metabolic process;GO:0007040|lysosome organization;GO:0031293|membrane protein intracellular domain proteolysis;GO:0006508|proteolysis;GO:0042990|regulation of transcription factor import into nucleus;GO:0034976|response to endoplasmic reticulum stress;	GO:0005788|endoplasmic reticulum lumen;GO:0005789|endoplasmic reticulum membrane;GO:0000139|Golgi membrane;GO:0005795|Golgi	GO:0004252|serine-type endopeptidase activity;	K08653	MBTPS1; membrane-bound transcription factor site-1 protease
PPP1CB	P62142	677	0	Serine/threonine-protein phosphatase PP1-beta catalytic subunit	.	.	.	.	pfam00149	Metallophos	Calcineurin-like phosphoesterase. This family includes a diverse range of phosphoesterases, including protein phosphoserine	GO:0007049|cell cycle;GO:0051301|cell division;GO:0032922|circadian regulation of gene expression;GO:0043153|entrainment of circadian clock by photoperiod;GO:0005977|glycogen metabolic process;GO:0006470|protein dephosphorylation;GO:0030155|regulation of cell adhesion;GO:0042752|regulation of circadian rhythm;GO:0005979|regulation of glycogen biosynthetic process;GO:0005981|regulation of glycogen catabolic process;GO:0019433|triglyceride catabolic process;	GO:0005829|cytosol;GO:0070062|extracellular exosome;GO:0005925|focal adhesion;GO:0042587|glycogen granule;GO:0070688|MLL5-L	GO:0046872|metal ion binding;GO:0017018|myosin	K06269	PPP1C; serine/threonine-protein phosphatase PP1 catalytic subunit
LRIF1	Q5T3J3	1161	0	Ligand-dependent nuclear receptor-interacting factor 1	.	.	.	.	pfam00816	Histone_HNS	H-NS histone family.	GO:0006355|regulation of transcription, DNA-templated;GO:0006351|transcription, DNA-templated;	GO:0005815|microtubule organizing center;GO:0016363|nuclear matrix;GO:0005634|nucleus;	.	.	.
DDI2	Q29106	805	0	Regulatory solute carrier protein family 1 member 1	.	.	.	.	pfam09668	Asp_protease	Aspartyl protease. This family of eukaryotic aspartyl proteases have a fold similar to retroviral proteases which implies they	GO:0051051|negative regulation of transport;GO:0006355|regulation of transcription, DNA-templated;GO:0006351|transcription, DNA-templated;	GO:0005794|Golgi apparatus;GO:0005634|nucleus;GO:0005886|plasma membrane;	GO:0008200|ion channel inhibitor activity;	.	.
RPL19	P84100	264	3e-87	60S ribosomal protein L19	SPCC1682.14	186	5.34e-59	COG2147	pfam01280	Ribosomal_L19e	Ribosomal protein L19e.	GO:0097421|liver regeneration;GO:0006412|translation;	GO:0022625|cytosolic large ribosomal subunit;GO:0005925|focal adhesion;GO:0016020|membrane;GO:0005730|nucleolus;	GO:0070180|large ribosomal subunit rRNA binding;GO:0044822	K02885	RP-L19e, RPL19; large subunit ribosomal protein L19e
SPCS3	P61009	367	1e-116	Signal peptidase complex subunit 3	.	.	.	.	pfam04573	SPC22	Signal peptidase subunit. Translocation of polypeptide chains across the endoplasmic reticulum membrane is triggered	GO:0006465|signal peptide processing;	GO:0005783|endoplasmic reticulum;GO:0005789|endoplasmic reticulum membrane;GO:0016021|integral component of	GO:0008233|peptidase activity;	K12948	SPCS3, SPC3; signal peptidase complex subunit 3 [EC:3.4.-.-]
DOLPP1	Q86YN1	447	1e-151	Dolichyldiphosphatase 1	YGR036c	85.5	7.78e-18	COG0671	pfam01569	PAP2	PAP2 superfamily. This family includes the enzyme type 2 phosphatidic acid phosphatase (PAP2), Glucose-6-phosphatase	GO:0006489|dolichyl diphosphate biosynthetic process;GO:0008610|lipid biosynthetic process;GO:0006487|protein N-linked glycosylation;	GO:0005789|endoplasmic reticulum membrane;GO:0030176|integral component of endoplasmic reticulum membrane;	GO:0047874|dolichyldiphosphatase activity;	K07252	E3.6.1.43; dolichyldiphosphatase [EC:3.6.1.43]
RBIS	Q0VG62	154	4e-46	Uncharacterized protein C8orf59 homolog	.	.	.	.	pfam02384	N6_Mtase	N-6 DNA Methylase. Restriction-modification (R-M) systems protect a bacterial cell against invasion of foreign DNA by	GO:0098779|mitophagy in response to mitochondrial depolarization;GO:0002230|positive regulation of defense response to virus by host;GO:0098792|xenophagy;	.	.	.	.
CDK19	Q8BWD8	837	0	Cyclin-dependent kinase 19	.	.	.	.	pfam00069	Pkinase	Protein kinase domain.	GO:0071222|cellular response to lipopolysaccharide;GO:0043065|positive regulation of apoptotic process;GO:0050729|positive regulation of inflammatory response;	GO:0005829|cytosol;GO:0005634|nucleus;	GO:0005524|ATP binding;GO:0004693|cyclin-dependent protein	K02208	CDK8_11; cyclin-dependent kinase 8/11 [EC:2.7.11.22 2.7.11.23]
KANSL1	Q7Z3B3	1853	0	KAT8 regulatory NSL complex subunit 1	.	.	.	.	pfam15275	PEHE	PEHE domain. This domain was first identified in drosophila MSL1 (male-specific lethal 1). In drosophila it binds to the histone	GO:0043984|histone H4-K16 acetylation;GO:0043981|histone H4-K5 acetylation;GO:0043982|histone H4-K8 acetylation;	GO:0000777|condensed chromosome kinetochore;GO:0000123|histone acetyltransferase complex;GO:0071339|MLL1	GO:0001047|core promoter binding;GO:0035035|histone	K18400	KANSL1; KAT8 regulatory NSL complex subunit 1

EGR1	1650.388	0.474591	0.161340015	2.941561	0.0032656	0.024669	Not_Sig	no	gi|109658287|gb|AAI18329.1|	2058	0	Early growth response 1 [Bos taurus]	gi|109658286|gb|BC118328.1|	3052	0	Bos taurus early growth response 1, mRNA (cDNA clone MGC:139820 IMAGE:8283079), complete cds	Q29W20	780	0	Early growth response protein 1	.	.	.	.	pfam11914	DUF3432	Domain of unknown function (DUF3432). This presumed domain is functionally uncharacterized. This domain is found in	GO:0071504|cellular response to heparin;GO:0098759|cellular response to interleukin-8;GO:0071506|cellular response to mycophenolic acid;GO:0044849|estrous cycle;GO:0072110|glomerular mesangial cell proliferation;GO:0045080|positive regulation of chemokine biosynthetic process;GO:0072303|positive regulation of glomerular metanephric mesangial cell proliferation;GO:0046886|positive regulation of hormone biosynthetic process;GO:0050725|positive regulation of interleukin-1 beta biosynthetic process;GO:0045944|positive regulation of	GO:0005737|cytoplasm;GO:0005634|nucleus;	GO:0010385|double-stranded methylated DNA binding;GO:0044729	K09203	EGR1; early growth response protein 1
PDLIM2	481.246	0.421766	0.143443378	2.940297	0.003279	0.024753	Not_Sig	no	gi|742148779|ref|XP_010843781.1|	1532	0	PREDICTED: PDZ and LIM domain protein 2 [Bison bison bison]	gi|742148782|ref|XM_010845480.1|	1417	0	PREDICTED: Bison bison bison PDZ and LIM domain 2 (mystique) (PDLIM2), transcript variant X2, mRNA	Q3T0C8	591	0	PDZ and LIM domain protein 2	.	.	.	.	pfam00595	PDZ	PDZ domain (Also known as DHR or GLGF). PDZ domains are found in diverse signaling proteins.	.	GO:0015629|actin cytoskeleton;GO:0005737|cytoplasm;GO:0070062|extracellular exosome;GO:0005925|focal adhesion;	GO:0008270|zinc ion binding;	.	.
COMMD8	754.5685	-0.38232	0.130038046	-2.94006	0.0032815	0.024753	Not_Sig	no	gi|61821147|ref|XP_583879.1|	899	9e-111	PREDICTED: COMM domain-containing protein 8 [Bos taurus]	gi|942046962|ref|XM_005892917.2|	2919	0	PREDICTED: Bos mutus COMM domain containing 8 (COMMD8), mRNA	Q9NX08	300	2e-93	COMM domain-containing protein 8	.	.	.	.	pfam07258	HCaRG	HCaRG protein. This family consists of several mammalian HCaRG(hypertension-related, calcium-regulated gene) proteins.	GO:0006355|regulation of transcription, DNA-templated;GO:0006351|transcription, DNA-templated;	GO:0005737|cytoplasm;GO:0005634|nucleus;	.	.	.
ABCD1	171.5174	0.52558	0.178774609	2.939905	0.0032831	0.024753	Not_Sig	no	gi|555994750|ref|XP_005909123.1|	3801	0	PREDICTED: ATP-binding cassette sub-family D member 1 isoform X1 [Bos mutus]	gi|555994749|ref|XM_005909061.1|	3528	0	PREDICTED: Bos mutus ATP-binding cassette, sub-family D (ALD), member 1 (ABCD1), transcript variant X1, mRNA	P33897	1323	0	ATP-binding cassette sub-family D member 1	YPL147w	362	5.24e-108	COG4178	pfam06472	ABC_membrane_2	ABC transporter transmembrane region 2. This domain covers the transmembrane of a small family of ABC transporters and shares	GO:0036109|alpha-linolenic acid metabolic process;GO:0006635|fatty acid beta-oxidation;GO:0033540|fatty acid beta-oxidation using acyl-CoA oxidase;GO:0043651|linoleic acid metabolic process;GO:0042758|long-chain fatty acid catabolic process;GO:0015910|peroxisomal long-chain fatty acid import;GO:0015919|peroxisomal membrane transport;GO:0007031|peroxisome organization;GO:0055085|transmembrane transport;GO:0042760|very long-chain fatty acid catabolic process;	GO:0005737|cytoplasm;GO:0005829|cytosol;GO:0005779|integral component of peroxisomal membrane;GO:0016020|membrane;GO:000	GO:0005524|ATP binding;GO:0016887|ATPase activity;GO:0042626	K05675	ABCD1, ALD; ATP-binding cassette, subfamily D (ALD), member 1
KPNA5	38.68703	-0.99536	0.338664475	-2.93907	0.003292	0.024803	DOWN	yes	gi|548479479|ref|XP_005684588.1|	2687	0	PREDICTED: importin subunit alpha-6 isoform X1 [Capra hircus]	gi|742210322|ref|XM_010861813.1|	5091	0	PREDICTED: Bison bison bison karyopherin alpha 5 (importin alpha 6) (KPNA5), mRNA	O15131	983	0	Importin subunit alpha-6	SPCC962.03c	511	6.38e-162	COG5064	pfam01749	IBB	Importin beta binding domain. This family consists of the importin alpha (karyopherin alpha), importin beta (karyopherin beta)	GO:0075733|intracellular transport of virus;GO:0019054|modulation by virus of host process;GO:0006607|NLS-bearing protein import into nucleus;	GO:0005737|cytoplasm;GO:0005829|cytosol;GO:0005643|nuclear pore;GO:0005654|nucleoplasm;	GO:0008139|nuclear localization sequence binding;GO:0008565	.	.
YAP1	1841.033	-0.3323	0.113100012	-2.9381	0.0033023	0.024864	Not_Sig	no	gi|675695612|ref|XP_009005426.1|	2193	0	PREDICTED: yorkie homolog isoform X1 [Callithrix jacchus]	gi|982945328|ref|XM_015474584.1|	4553	0	PREDICTED: Bos taurus Yes associated protein 1 (YAP1), transcript variant X1, mRNA	P46937	826	0	Transcriptional coactivator YAP1	YER125w	85.9	6.41e-16	COG5021	pfam00397	WW	WW domain. The WW domain is a protein module with two highly conserved tryptophans that binds proline-rich peptide motifs in vitro.	GO:0060449|bud elongation involved in lung branching;GO:0000902|cell morphogenesis;GO:0008283|cell proliferation;GO:0006974|cellular response to DNA damage stimulus;GO:0071480|cellular response to gamma radiation;GO:0071300|cellular response to retinoic acid;GO:0060242|contact inhibition;GO:0003143|embryonic heart tube morphogenesis;GO:0035329|hippo signaling;GO:0030216|keratinocyte differentiation;GO:0048368|lateral mesoderm development;GO:0060487|lung epithelial cell	GO:0005737|cytoplasm;GO:0005829|cytosol;GO:0016020|membrane;GO:0005654|nucleoplasm;GO:0005634|nucleus;GO:0005667|transcription factor complex;	GO:0003682|chromatin binding;GO:0000979|RNA polymerase II	K16687	YAP1, Yki; protein yorkie
UROC1	63.35376	0.770958	0.262590777	2.935969	0.0033251	0.02502	UP	yes	gi|297488858|ref|XP_002697206.1|	3440	0	PREDICTED: urocanate hydratase isoform X4 [Bos taurus]	gi|942095395|ref|XM_005909104.2|	2655	0	PREDICTED: Bos mutus urocanate hydratase 1 (UROC1), mRNA	Q8VC12	1194	0	Urocanate hydratase	SPy2082	565	0	COG2987	pfam01175	Urocanase	Urocanase.	GO:0006548|histidine catabolic process;GO:0019556|histidine catabolic process to glutamate and formamide;GO:0019557|histidine catabolic process to glutamate and formate;	GO:0005829|cytosol;	GO:0016153|urocanate hydratase activity;	K01712	hutU, UROC1; urocanate hydratase [EC:4.2.1.49]
ZNF414	200.6309	0.603861	0.20574505	2.934999	0.0033355	0.025082	UP	yes	gi|296485756|tpg|DAA27871.1|	1272	7e-170	TPA: zinc finger protein 414 [Bos taurus]	gi|402746591|ref|NM_001038175.2|	1374	0	Bos taurus zinc finger protein 414 (ZNF414), mRNA	Q32KV8	490	1e-169	Zinc finger protein 414	.	.	.	.	pfam05616	Neisseria_TspB	Neisseria meningitidis TspB protein. This family consists of several Neisseria meningitidis TspB virulence factor proteins.	GO:0006355|regulation of transcription, DNA-templated;GO:0006351|transcription, DNA-templated;	GO:0005634|nucleus;	GO:0003677|DNA binding;GO:0046872|metal ion binding;	.	.
GABARAPL2	1148.295	-0.33744	0.114990658	-2.93446	0.0033412	0.025108	Not_Sig	no	gi|426382957|ref|XP_004058063.1|	690	6e-86	PREDICTED: uncharacterized protein LOC101141496 [Gorilla gorilla gorilla]	gi|73587000|gb|BC102902.1|	959	0	Bos taurus GABA(A) receptor-associated protein-like 2, mRNA (cDNA clone MGC:128308 IMAGE:7985087), complete cds	P60522	242	2e-78	Gamma-aminobutyric acid receptor-associated protein-like 2	.	.	.	.	pfam02991	Atg8	Autophagy protein Atg8 ubiquitin like. Light chain 3 is proposed to function primarily as a subunit of microtubule associated proteins	GO:0000045|autophagosome assembly;GO:0006995|cellular response to nitrogen starvation;GO:0000422|mitophagy;GO:0015031|protein transport;	GO:0005776|autophagosome;GO:0000421|autophagosome membrane;GO:0031410|cytoplasmic vesicle;GO:0005829|cytosol;GO:0005794|G	.	K08341	GABARAP, ATG8, LC3; GABA(A) receptor-associated protein
RNF146	831.1031	-0.3401	0.115907455	-2.93425	0.0033435	0.025109	Not_Sig	no	gi|116004181|ref|NP_001070448.1|	1442	0	E3 ubiquitin-protein ligase RNF146-B [Bos taurus]	gi|528960337|ref|XM_005210700.1|	1843	0	PREDICTED: Bos taurus ring finger protein 146 (RNF146), transcript variant X1, mRNA	Q3T139	560	0	E3 ubiquitin-protein ligase RNF146-B	.	.	.	.	pfam02825	WWE	WWE domain. The WWE domain is named after three of its conserved residues and is predicted to mediate specific protein- protein	GO:0090263|positive regulation of canonical Wnt signaling pathway;GO:0051865|protein autoubiquitination;GO:0070936|protein K48-linked ubiquitination;GO:0042787|protein ubiquitination involved in ubiquitin-dependent protein catabolic process;GO:0016055|Wnt signaling pathway;	GO:0005829|cytosol;	GO:0016874|ligase activity;GO:0072572|poly-ADP-D-ribose binding;GO:0061630	K15700	RNF146; E3 ubiquitin-protein ligase RNF146 [EC:2.3.2.27]
USP30	691.6034	0.451141	0.153865881	2.932037	0.0033675	0.025259	Not_Sig	no	gi|300798203|ref|NP_001178290.1|	2423	0	ubiquitin carboxyl-terminal hydrolase 30 [Bos taurus]	gi|742219872|ref|XM_010828670.1|	3604	0	PREDICTED: Bison bison bison ubiquitin specific peptidase 30 (USP30), transcript variant X1, mRNA	Q70CQ3	857	0	Ubiquitin carboxyl-terminal hydrolase 30	.	.	.	.	pfam00443	UCH	Ubiquitin carboxyl-terminal hydrolase.	GO:0008053|mitochondrial fusion;GO:0000422|mitophagy;GO:0016579|protein deubiquitination;GO:0035871|protein K11-linked deubiquitination;GO:0044313|protein K6-linked deubiquitination;GO:0006511|ubiquitin-dependent protein catabolic process;	GO:0016021|integral component of membrane;GO:0005741|mitochondrial outer membrane;GO:0005739|mitochondrion;	GO:0004197|cysteine-type endopeptidase activity;GO:0004843	K11851	USP30; ubiquitin carboxyl-terminal hydrolase 30 [EC:3.4.19.12]
DPP9	1540.721	0.391503	0.133529382	2.931964	0.0033683	0.025259	Not_Sig	no	gi|440894092|gb|ELR46642.1|	4681	0	Dipeptidyl peptidase 9, partial [Bos mutus]	gi|982984874|ref|XM_015464141.1|	4080	0	PREDICTED: Bos taurus dipeptidyl-peptidase 9 (DPP9), transcript variant X3, mRNA	Q86TI2	1618	0	Dipeptidyl peptidase 9	CC2154	270	7.50e-76	COG1506	pfam00930	DPPIV_N	Dipeptidyl peptidase IV (DPP IV) N-terminal region. This family is an alignment of the region to the N-terminal side of the active site. The	.	GO:0005737|cytoplasm;GO:0005829|cytosol;GO:0005634|nucleus;	GO:0004177|aminopeptidase activity;GO:0008236|serine-type	K08656	DPP9; dipeptidyl-peptidase 9 [EC:3.4.14.5]
ASCC2	659.2826	0.374641	0.127792124	2.931642	0.0033718	0.025259	Not_Sig	no	gi|62752025|ref|NP_001015524.1|	3692	0	activating signal cointegrator 1 complex subunit 2 [Bos taurus]	gi|982950723|ref|XM_005218035.3|	2690	0	PREDICTED: Bos taurus activating signal cointegrator 1 complex subunit 2 (ASCC2), transcript variant X1, mRNA	Q9H1I8	1280	0	Activating signal cointegrator 1 complex subunit 2	.	.	.	.	pfam02845	CUE	CUE domain. CUE domains have been shown to bind ubiquitin. It has been suggested that CUE domains are related to pfam00627	GO:0006307|DNA dealkylation involved in DNA repair;GO:0006355|regulation of transcription, DNA-templated;GO:0006351|transcription, DNA-templated;	GO:0005654|nucleoplasm;GO:0005634|nucleus;	.	K18667	ASCC2; activating signal cointegrator complex subunit 2
CHST12	478.7754	0.427223	0.145741529	2.931377	0.0033746	0.025259	Not_Sig	no	gi|555950355|ref|XP_005887380.1|	2122	0	PREDICTED: carbohydrate sulfotransferase 12 [Bos mutus]	gi|942031285|ref|XM_005887318.2|	1749	0	PREDICTED: Bos mutus carbohydrate (chondroitin 4) sulfotransferase 12 (CHST12), mRNA	Q99LL3	664	0	Carbohydrate sulfotransferase 12	.	.	.	.	pfam03567	Sulfotransfer_2	Sulfotransferase family. This family includes a variety of sulfotransferase enzymes. Chondroitin 6-sulfotransferase	GO:0016051|carbohydrate biosynthetic process;GO:0030206|chondroitin sulfate biosynthetic process;GO:0030208|dermatan sulfate biosynthetic process;	GO:0000139|Golgi membrane;GO:0016021|integral component of membrane;GO:0016020|membrane;	GO:0047756|chondroitin 4-sulfotransferase activity;	K04742	CHST12; chondroitin 4-sulfotransferase 12 [EC:2.8.2.5]
CCNC	500.8048	-0.44917	0.153229905	-2.93137	0.0033747	0.025259	Not_Sig	no	gi|593709138|ref|XP_007101178.1|	1591	0	PREDICTED: cyclin-C isoform X1 [Physeter catodon]	gi|982935054|ref|XM_005210847.3|	1004	0	PREDICTED: Bos taurus cyclin C (CCNC), transcript variant X1, mRNA	Q3ZCK5	535	0	Cyclin-C	YNL025c	124	4.49e-30	COG5333	pfam00134	Cyclin_N	Cyclin, N-terminal domain. Cyclins regulate cyclin dependent kinases (CDKs). Human cyclin-O is a Uracil-DNA glycosylase that is related to	GO:0045737|positive regulation of cyclin-dependent protein serine/threonine kinase activity;GO:1901409|positive regulation of phosphorylation of RNA polymerase II C-terminal domain;GO:0045944|positive regulation of transcription from RNA polymerase II promoter;GO:0006351|transcription, DNA-templated;	GO:0000307|cyclin-dependent protein kinase holoenzyme complex;GO:0016592|mediator complex;GO:0005634|nucleus;	GO:0016538|cyclin-dependent protein serine/threonine kinase regulator	K15161	CCNC, SSN8; cyclin C
RGS4	183.0589	-0.48197	0.164428011	-2.93119	0.0033766	0.025259	Not_Sig	no	gi|114050719|ref|NP_001040065.1|	988	3e-124	regulator of G-protein signaling 4 [Bos taurus]	gi|742181328|ref|XM_010854660.1|	2678	0	PREDICTED: Bison bison bison regulator of G-protein signaling 4 (RGS4), transcript variant X1, mRNA	Q29RM9	385	9e-126	Regulator of G-protein signaling 4	.	.	.	.	pfam00615	RGS	Regulator of G protein signaling domain. RGS family members are GTPase-activating proteins for heterotrimeric G-protein alpha-	GO:0009968|negative regulation of signal transduction;	GO:0005737|cytoplasm;GO:0005886|plasma membrane;	GO:0005096|GTPase activator activity;	K16449	RGS; regulator of G-protein signaling
TXNDC5	5479.935	0.300529	0.102558977	2.930305	0.0033863	0.025315	Not_Sig	no	gi|332078455|ref|NP_001193661.1|	1849	0	thioredoxin domain-containing protein 5 precursor [Bos taurus]	gi|332078454|ref|NM_001206732.1|	1474	0	Bos taurus thioredoxin domain containing 5 (endoplasmic reticulum) (TXNDC5), mRNA	Q8NBS9	657	0	Thioredoxin domain-containing protein 5	SPAC17H9.14c	127	9.85e-32	COG0526	pfam00085	Thioredoxin	Thioredoxin. Thioredoxins are small enzymes that participate in redox reactions, via the reversible oxidation of an active centre	GO:0043277|apoptotic cell clearance;GO:0045454|cell redox homeostasis;GO:0043066|negative regulation of apoptotic process;GO:0006457|protein folding;GO:0034976|response to endoplasmic reticulum stress;	GO:0005783|endoplasmic reticulum;GO:0005788|endoplasmic reticulum lumen;GO:0070062|extracellular exosome;GO:0043202|lysosomal lumen;	GO:0003756|protein disulfide isomerase activity;	K13984	TXNDC5, ERP46; thioredoxin domain-containing protein 5
TBC1D17	565.7348	0.474032	0.1618077	2.929602	0.003394	0.025349	Not_Sig	no	gi|296477465|tpg|DAA19580.1|	2848	0	TPA: TBC1 domain family, member 17 [Bos taurus]	gi|402692257|ref|NM_001102012.2|	2462	0	Bos taurus TBC1 domain family member 17 (TBC1D17), mRNA	Q9HA65	1038	0	TBC1 domain family member 17	SPAC630.05	180	1.46e-46	COG5210	pfam12068	DUF3548	Domain of unknown function (DUF3548). This presumed domain is functionally uncharacterized. This domain is found in	GO:0006914|autophagy;GO:0015031|protein transport;GO:0042147|retrograde transport, endosome to Golgi;	GO:0005776|autophagosome;GO:0031410|cytoplasmic vesicle;GO:0005829|cytosol;	GO:0005096|GTPase activator activity;	.	.
TM9SF4	1886.354	0.385955	0.131748593	2.929482	0.0033953	0.025349	Not_Sig	no	gi|296199873|ref|XP_002747493.1|	3135	0	PREDICTED: transmembrane 9 superfamily member 4 isoform X1 [Callithrix jacchus]	gi|166063944|ref|NM_001098077.2|	3838	0	Bos taurus transmembrane 9 superfamily protein member 4 (TM9SF4), mRNA	A5D7E2	1204	0	Transmembrane 9 superfamily member 4	.	.	.	.	pfam02990	EMP70	Endomembrane protein 70.	GO:0070863|positive regulation of protein exit from endoplasmic reticulum;GO:2000010|positive regulation of protein localization to cell surface;	GO:0005769|early endosome;GO:0005794|Golgi apparatus;GO:0016021|integral component of membrane;	.	K17086	TM9SF2_4; transmembrane 9 superfamily member 2/4
PGGT1B	467.5267	-0.37989	0.129692671	-2.92918	0.0033985	0.025357	Not_Sig	no	gi|555957585|ref|XP_005890922.1|	2039	0	PREDICTED: geranylgeranyl transferase type-1 subunit beta isoform X1 [Bos mutus]	gi|965906249|ref|XM_012165795.2|	2647	0	PREDICTED: Ovis aries musimon protein geranylgeranyltransferase type I, beta subunit (PGGT1B), mRNA	Q5EAD5	788	0	Geranylgeranyl transferase type-1 subunit beta	SPAC2E1P5.04c	155	1.17e-40	COG5029	pfam13249	Prenyltrans_2	Prenyltransferase-like.	GO:0018344|protein geranylgeranylation;	GO:0005953|CAAX-protein geranylgeranyltransferase complex;	GO:0004662|CAAX-protein geranylgeranyltransferase	K11713	PGTB1; geranylgeranyl transferase type-1 subunit beta
LOC138991446	60.82423	-0.83798	0.286421634	-2.92568	0.003437	0.025627	DOWN	yes	gi|742092097|ref|XP_010832059.1|	485	3e-60	PREDICTED: COMM domain-containing protein 6 [Bison bison bison]	gi|742092096|ref|XM_010833757.1|	425	0	PREDICTED: Bison bison bison COMM domain-containing protein 6 (LOC104984135), mRNA	Q2KIY0	177	1e-56	COMM domain-containing protein 6	.	.	.	.	pfam07258	HCaRG	HCaRG protein. This family consists of several mammalian HCaRG(hypertension-related, calcium-regulated gene) proteins.	GO:0006355|regulation of transcription, DNA-templated;GO:0006351|transcription, DNA-templated;	GO:0005737|cytoplasm;GO:0005634|nucleus;	.	.	.
TRAF4	260.3506	0.50552	0.172802452	2.925424	0.0034399	0.025632	Not_Sig	no	gi|155372275|ref|NP_001094750.1|	2391	0	TNF receptor-associated factor 4 [Bos taurus]	gi|155372274|ref|NM_001101280.1|	1974	0	Bos taurus TNF receptor associated factor 4 (TRAF4), mRNA	Q9BUZ4	912	0	TNF receptor-associated factor 4	.	.	.	.	pfam02176	zf-TRAF	TRAF-type zinc finger.	GO:0007250|activation of NF-kappaB-inducing kinase activity;GO:0006915|apoptotic process;GO:0046330|positive regulation of JNK cascade;GO:0090073|positive regulation of protein homodimerization activity;GO:0045860|positive regulation of protein kinase activity;GO:0042981|regulation of apoptotic process;GO:0007585|respiratory gaseous exchange;GO:0030323|respiratory tube development;GO:0007165|signal transduction;	GO:0005923|bicellular tight junction;GO:0005737|cytoplasm;GO:0005856|cytoskeleton;GO:0005634|nucleus;GO:0048471|perinuclear region of	GO:0003677|DNA binding;GO:0042802|identical protein binding;GO:0031996	K09848	TRAF4; TNF receptor-associated factor 4
ST3GAL3	194.8354	0.464348	0.158788313	2.924321	0.0034521	0.025706	Not_Sig	no	gi|742213490|ref|XP_010860902.1|	2059	0	PREDICTED: CMP-N-acetylneuraminate-beta-1,4-galactoside alpha-2,3-sialyltransferase isoform X1 [Bison bison bison]	gi|594040074|ref|XM_006044548.1|	2150	0	PREDICTED: Bubalus bubalis ST3 beta-galactoside alpha-2,3-sialyltransferase 3 (ST3GAL3), transcript variant X1, mRNA	P61132	663	0	CMP-N-acetylneuraminate-beta-1,4-galactoside alpha-2,3-sialyltransferase	.	.	.	.	pfam00777	Glyco_transf_29	Glycosyltransferase family 29 (sialyltransferase). Members of this family belong to glycosyltransferase family 29.	GO:0001574|ganglioside biosynthetic process;GO:0009311|oligosaccharide metabolic process;GO:0018279|protein N-linked glycosylation via asparagine;	GO:0005576|extracellular region;GO:0032580|Golgi cisterna membrane;GO:0030173|integral component of Golgi membrane;	GO:0003836|beta-galactoside (CMP) alpha-2,3-sialyltransferase	K00781	SIAT6; N-acetyllactosaminide alpha-2,3-sialyltransferase
ZBTB40	536.4584	0.415641	0.142199847	2.922933	0.0034675	0.025796	Not_Sig	no	gi|742228787|ref|XP_010828302.1|	6129	0	PREDICTED: zinc finger and BTB domain-containing protein 40 [Bison bison bison]	gi|742228810|ref|XM_010830007.1|	5525	0	PREDICTED: Bison bison bison zinc finger and BTB domain containing 40 (ZBTB40), transcript variant X7, mRNA	Q9NUA8	1979	0	Zinc finger and BTB domain-containing protein 40	SPAC144.09c	97.1	2.83e-20	COG5048	pfam00651	BTB	BTB/POZ domain. The BTB (for BR-C, ttk and bab) or POZ (for Pox virus and Zinc finger) domain is present near the N-terminus of a	GO:0030282|bone mineralization;GO:0006974|cellular response to DNA damage stimulus;GO:0006355|regulation of transcription, DNA-templated;GO:0006351|transcription, DNA-templated;	GO:0005634|nucleus;	GO:0003677|DNA binding;GO:0046872|metal ion binding;	K10512	ZBTB40; zinc finger and BTB domain-containing protein 40
RTKN	82.81438	0.638597	0.218485542	2.922835	0.0034686	0.025796	UP	yes	gi|741942328|ref|XP_010808136.1|	2714	0	PREDICTED: rhotekin isoform X2 [Bos taurus]	gi|982938382|ref|XM_010809834.2|	3628	0	PREDICTED: Bos taurus rhotekin (RTKN), transcript variant X2, mRNA	Q9BST9	870	0	Rhotekin	.	.	.	.	pfam08174	Anillin	Cell division protein anillin. Anillin is a protein involved in septin organisation during cell division. It is an actin binding protein that is	GO:0006915|apoptotic process;GO:0007266|Rho protein signal transduction;GO:0007165|signal transduction;	GO:0005829|cytosol;	GO:0005525|GTP binding;GO:0017049|GTP-Rho binding;GO:0005095	.	.
CEP85L	98.87283	-0.63405	0.216961187	-2.92239	0.0034735	0.025815	DOWN	yes	gi|528960475|ref|XP_002690127.3|	4035	0	PREDICTED: centrosomal protein of 85 kDa-like isoform X2 [Bos taurus]	gi|982987477|ref|XM_015464770.1|	6794	0	PREDICTED: Bos taurus centrosomal protein 85kDa-like (CEP85L), transcript variant X2, mRNA	Q5SZL2	1383	0	Centrosomal protein of 85 kDa-like	.	.	.	.	pfam05483	SCP-1	Synaptonemal complex protein 1 (SCP-1). Synaptonemal complex protein 1 (SCP-1) is the major component of the transverse	.	GO:0005813|centrosome;GO:0005737|cytoplasm;	.	K16766	CEP85; centrosomal protein CEP85
C1H21orf91	242.0161	-0.51891	0.17759368	-2.92192	0.0034788	0.025822	Not_Sig	no	gi|555972465|ref|XP_005898236.1|	1585	0	PREDICTED: protein EURL homolog isoform X1 [Bos mutus]	gi|742175273|ref|XM_010852959.1|	5562	0	PREDICTED: Bison bison bison chromosome unknown open reading frame, human C21orf91 (LOC104997979), transcript variant X2, mRNA	Q9NYK6	560	0	Protein EURL homolog	.	.	.	.	pfam06937	EURL	EURL protein. This family consists of several animal EURL proteins. EURL is preferentially expressed in chick retinal precursor cells as well	.	.	.	.	.
H3-3A	1753.557	-0.32127	0.109950425	-2.92192	0.0034788	0.025822	Not_Sig	no	gi|731286412|ref|XP_010611509.1|	788	2e-101	PREDICTED: LOW QUALITY PROTEIN: histone H3.3 [Fukomys damarensis]	gi|136256445|ref|NM_001014389.2|	1043	0	Bos taurus H3 histone, family 3A (H3F3A), mRNA	Q6P823	273	4e-90	Histone H3.3	.	.	.	.	pfam00125	Histone	Core histone H2A/H2B/H3/H4.	GO:0006334|nucleosome assembly;	GO:0000786|nucleosome;GO:0005634|nucleus;	GO:0031492|nucleosomal DNA binding;	K11253	H3; histone H3
GUCD1	341.431	0.479615	0.164191489	2.921069	0.0034883	0.025875	Not_Sig	no	gi|594047485|ref|XP_006048160.1|	1201	2e-154	PREDICTED: protein GUCD1 isoform X3 [Bubalus bubalis]	gi|147905337|ref|NM_001098044.1|	2706	0	Bos taurus guanylyl cyclase domain containing 1 (GUCD1), mRNA	Q96NT3	438	6e-145	Protein GUCD1	.	.	.	.	pfam09778	Guanylate_cyc_2	Guanylylate cyclase. Members of this family of proteins catalyse the conversion of guanosine triphosphate (GTP) to 3',5'-cyclic	.	.	.	.	.
GLRX	281.3893	-0.43801	0.149965166	-2.92075	0.0034919	0.025885	Not_Sig	no	gi|742237444|ref|XP_010830251.1|	685	3e-84	PREDICTED: glutaredoxin-1 isoform X1 [Bison bison bison]	gi|742237446|ref|XM_010831950.1|	1595	0	PREDICTED: Bison bison bison glutaredoxin (thioltransferase) (GLRX), transcript variant X2, mRNA	P10575	218	7e-67	Glutaredoxin-1	SPAC4F10.20	72.4	8.06e-15	COG0695	pfam00462	Glutaredoxin	Glutaredoxin.	GO:0045454|cell redox homeostasis;	GO:0005737|cytoplasm;	GO:0009055|electron carrier activity;GO:0015035|protein disulfide	K03676	grxC, GLRX, GLRX2; glutaredoxin 3
CD274	55.44856	-0.77744	0.266347284	-2.91889	0.0035128	0.026023	DOWN	yes	gi|742205832|ref|XP_010859028.1|	1615	0	PREDICTED: programmed cell death 1 ligand 1 isoform X1 [Bison bison bison]	gi|965920580|ref|XM_004004362.3|	1620	0	PREDICTED: Ovis aries CD274 molecule (CD274), transcript variant X4, mRNA	Q9NZQ7	434	3e-138	Programmed cell death 1 ligand 1	.	.	.	.	pfam08205	C2-set_2	CD80-like C2-set immunoglobulin domain. These domains belong to the immunoglobulin superfamily.	GO:0007166|cell surface receptor signaling pathway;GO:0006955|immune response;GO:0046007|negative regulation of activated T cell proliferation;GO:0032689|negative regulation of interferon-gamma production;GO:0032693|negative regulation of interleukin-10 production;GO:1903556|negative regulation of tumor necrosis factor superfamily cytokine production;GO:0030335|positive regulation of cell migration;GO:2001181|positive regulation of interleukin-10 secretion;GO:0042102|positive regulation of T cell	GO:0012505|endomembrane system;GO:0009897|external side of plasma membrane;GO:0070062|extracellular exosome;GO:0016021|integral component	.	K06745	PDCD1LG1, CD274; programmed cell death 1 ligand 1
MRPS24	209.4154	-0.46276	0.158564437	-2.91841	0.0035182	0.026046	Not_Sig	no	gi|594103682|ref|XP_006074844.1|	943	4e-124	PREDICTED: 28S ribosomal protein S24, mitochondrial [Bubalus bubalis]	gi|594103681|ref|XM_006074782.1|	783	0	PREDICTED: Bubalus bubalis mitochondrial ribosomal protein S24 (MRPS24), mRNA	Q2M2T7	347	6e-119	28S ribosomal protein S24, mitochondrial	.	.	.	.	pfam14955	MRP-S24	Mitochondrial ribosome subunit S24. This family of proteins corresponds to mitochondrial ribosomal subunit S24 in	GO:0032543|mitochondrial translation;GO:0070125|mitochondrial translational elongation;GO:0070124|mitochondrial translational initiation;	GO:0005743|mitochondrial inner membrane;GO:0005763|mitochondrial small ribosomal subunit;	GO:0044822|poly(A) RNA binding;GO:0003735|structural	K17403	MRPS24; small subunit ribosomal protein S24
TRAM2	2224.135	0.344435	0.118052235	2.917646	0.0035268	0.026094	Not_Sig	no	gi|300798154|ref|NP_001180079.1|	1713	0	translocating chain-associated membrane protein 2 [Bos taurus]	gi|982963549|ref|XM_015459816.1|	5635	0	PREDICTED: Bos taurus translocation associated membrane protein 2 (TRAM2), transcript variant X2, mRNA	Q15035	635	0	Translocating chain-associated membrane protein 2	.	.	.	.	pfam03798	TRAM_LAG1_CLN8	TLC domain.	GO:0032964|collagen biosynthetic process;GO:0006613|cotranslational protein targeting to membrane;	GO:0016021|integral component of membrane;GO:0005791|rough endoplasmic reticulum;	.	.	.
LOC106700684	112.9516	-0.57466	0.196979455	-2.91734	0.0035303	0.026102	Not_Sig	no	gi|528759995|gb|EPY79654.1|	186	1e-14	hypothetical protein CB1_000904002 [Camelus ferus]	gi|742183577|ref|XR_826622.1|	777	0	PREDICTED: Bison bison bison uncharacterized LOC104999609 (LOC104999609), transcript variant X3, ncRNA	Q32PD9	62	3e-10	Single-pass membrane and coiled-coil domain-containing protein 4	.	.	.	.	pfam15012	DUF4519	Domain of unknown function (DUF4519). This family of proteins is found in eukaryotes. Proteins in this family are typically between	.	GO:0016021|integral component of membrane;	.	.	.
RAB35	742.2378	0.365081	0.12526042	2.914576	0.0035617	0.026316	Not_Sig	no	gi|5803135|ref|NP_006852.1|	895	2e-110	ras-related protein Rab-35 isoform 1 [Homo sapiens]	gi|148229406|ref|NM_001098127.1|	2643	0	Bos taurus RAB35, member RAS oncogene family (RAB35), mRNA	Q5U316	349	7e-112	Ras-related protein Rab-35	.	.	.	.	pfam00071	Ras	Ras family. Includes sub-families Ras, Rab, Rac, Ral, Ran, Rap Ypt1 and more. Shares P-loop motif with GTP_EFTU, arf and	GO:0019882|antigen processing and presentation;GO:1990090|cellular response to nerve growth factor stimulus;GO:0000910|cytokinesis;GO:0032456|endocytic recycling;GO:0016197|endosomal transport;GO:0006888|ER to Golgi vesicle-mediated transport;GO:0031175|neuron projection development;GO:0048227|plasma membrane to endosome transport;GO:0036010|protein localization to endosome;GO:0015031|protein transport;GO:0007264|small GTPase mediated signal	GO:0031253|cell projection membrane;GO:0045334|clathrin-coated endocytic vesicle;GO:0005905|coated pit;GO:0010008|endosome	GO:0019003|GDP binding;GO:0005525|GTP binding;GO:0003924	K07876	RAB35, RAB1C; Ras-related protein Rab-35
LOC106700651	280.4492	0.545839	0.187290648	2.914393	0.0035638	0.026316	Not_Sig	no	gi|942042487|ref|XP_014332390.1|	1882	0	PREDICTED: uncharacterized protein LOC106700651 [Bos mutus]	gi|942042486|ref|XM_014476904.1|	1348	0	PREDICTED: Bos mutus uncharacterized LOC106700651 (LOC106700651), mRNA	Q15399	86.7	8e-16	Toll-like receptor 1	.	.	.	.	pfam13676	TIR_2	TIR domain. This is a family of bacterial Toll-like receptors.	GO:0071727|cellular response to triacyl bacterial lipopeptide;GO:0042742|defense response to bacterium;GO:0042495|detection of triacyl bacterial lipopeptide;GO:0006955|immune response;GO:0006954|inflammatory response;GO:0045087|innate immune response;GO:0042116|macrophage activation;GO:0002755|MyD88-dependent toll-like receptor signaling pathway;GO:0045410|positive regulation of interleukin-6 biosynthetic process;GO:0042535|positive regulation of tumor necrosis factor biosynthetic	GO:0005794|Golgi apparatus;GO:0005887|integral component of plasma membrane;GO:0016020|membrane;GO:004	GO:0071723|lipopeptide binding;GO:0046982|protein	K05398	TLR1; toll-like receptor 1
SPIN1	635.7131	-0.34744	0.119266159	-2.91318	0.0035777	0.026402	Not_Sig	no	gi|982934271|ref|XP_015326897.1|	1377	4e-175	PREDICTED: spindlin-1 isoform X1 [Bos taurus]	gi|982934270|ref|XM_015471411.1|	4440	0	PREDICTED: Bos taurus spindlin 1 (SPIN1), transcript variant X1, mRNA	Q5R997	475	3e-154	Spindlin-1	.	.	.	.	pfam02513	Spin-Ssty	Spin/Ssty Family. Spindlin (Spin) is a novel maternal transcript present in the unfertilised egg and early embryo. The Y-linked	GO:0016569|covalent chromatin modification;GO:0007276|gamete generation;GO:0051321|meiotic cell cycle;GO:0007275|multicellular organismal development;GO:0030177|positive regulation of Wnt signaling pathway;GO:0009303|rRNA transcription;GO:0016055|Wnt signaling pathway;	GO:0005730|nucleolus;GO:0005819|spindle;	GO:0003677|DNA binding;GO:0035064|methylated histone binding;	.	.
XIAP	288.3169	-0.45983	0.15788394	-2.91248	0.0035857	0.026443	Not_Sig	no	gi|555954410|ref|XP_005889372.1|	2642	0	PREDICTED: E3 ubiquitin-protein ligase XIAP [Bos mutus]	gi|594082945|ref|XM_006065002.1|	3462	0	PREDICTED: Bubalus bubalis X-linked inhibitor of apoptosis (XIAP), transcript variant X1, mRNA	P98170	915	0	E3 ubiquitin-protein ligase XIAP	.	.	.	.	pfam00653	BIR	Inhibitor of Apoptosis domain. BIR stands for 'Baculovirus Inhibitor of apoptosis protein Repeat'. It is found repeated in inhibitor of	GO:0006974|cellular response to DNA damage stimulus;GO:0055070|copper ion homeostasis;GO:1990001|inhibition of cysteine-type endopeptidase activity involved in apoptotic process;GO:0043066|negative regulation of apoptotic process;GO:0043154|negative regulation of cysteine-type endopeptidase activity involved in apoptotic process;GO:0090263|positive regulation of canonical Wnt signaling pathway;GO:1902530|positive regulation of protein linear polyubiquitination;GO:0031398|positive regulation of protein	GO:0005737|cytoplasm;GO:0005829|cytosol;GO:0005654|nucleoplasm;GO:0005634|nucleus;	GO:0043027|cysteine-type endopeptidase inhibitor activity	K04725	XIAP, BIRC4; E3 ubiquitin-protein ligase XIAP [EC:2.3.2.27]
FADD	670.4459	-0.35725	0.122681643	-2.912	0.0035913	0.026443	Not_Sig	no	gi|56119112|ref|NP_001007817.1|	928	1e-122	FAS-associated death domain protein [Bos taurus]	gi|742108885|ref|XM_010837648.1|	946	0	PREDICTED: Bison bison bison Fas (TNFRSF6)-associated via death domain (FADD), mRNA	Q645M6	362	3e-124	FAS-associated death domain protein	.	.	.	.	pfam00531	Death	Death domain.	GO:0097202|activation of cysteine-type endopeptidase activity;GO:0006915|apoptotic process;GO:0071260|cellular response to mechanical stimulus;GO:0051607|defense response to virus;GO:0097192|extrinsic apoptotic signaling pathway in absence of ligand;GO:0045087|innate immune response;GO:0048535|lymph node development;GO:0097049|motor neuron apoptotic process;GO:0097527|necroptotic signaling pathway;GO:0070236|negative regulation of activation-induced cell death of T cells;GO:0060546|negative regulation of necroptotic	GO:0031265|CD95 death-inducing signaling complex;GO:0097342|ripoptosome;	.	K02373	FADD; FAS-associated death domain protein

<table>
<tr><td>PRKCSH</td><td>2351.43</td><td>0.281833</td><td>0.096784757</td><td>2.911958</td><td>0.0035917</td><td>0.026443</td><td>Not_Sig</td><td>no</td><td>gi|942041227|ref|XP_005890830.2|</td><td>2549</td><td>0</td><td>PREDICTED: glucosidase 2 subunit beta [Bos mutus]</td><td>gi|942041226|ref|XM_005890768.2|</td><td>2162</td><td>0</td><td>PREDICTED: Bos mutus protein kinase C substrate 80K-H (PRKCSH), mRNA</td><td>Q28034</td><td>887</td><td>0</td><td>Glucosidase 2 subunit beta</td><td>.</td><td>.</td><td>.</td><td>.</td><td>pfam12999</td><td>PRKCSH-like</td><td>Glucosidase II beta subunit-like. The sequences found in this family are similar to a region found in the beta-subunit of glucosidase II,</td><td>GO:0001701|in utero embryonic development;GO:0001889|liver development;GO:0006491|N-glycan processing;GO:0010977|negative regulation of neuron projection development;GO:0006807|nitrogen compound metabolic process;GO:0072001|renal system development;</td><td>GO:0005783|endoplasmic reticulum;</td><td>GO:0005509|calcium ion binding;GO:0016787|hydrolase</td><td>K08288</td><td>PRKCSH; protein kinase C substrate 80K-H</td></tr>
<tr><td>SRRM2</td><td>4054.161</td><td>0.341487</td><td>0.11727329</td><td>2.911893</td><td>0.0035925</td><td>0.026443</td><td>Not_Sig</td><td>no</td><td>gi|440913489|gb|ELR62938.1|</td><td>2407</td><td>0</td><td>Serine/arginine repetitive matrix protein 2 [Bos mutus]</td><td>gi|742119958|ref|XM_010840332.1|</td><td>3625</td><td>0</td><td>PREDICTED: Bison bison bison serine/arginine repetitive matrix 2 (SRRM2), transcript variant X1, mRNA</td><td>Q9UQ35</td><td>631</td><td>0</td><td>Serine/arginine repetitive matrix protein 2</td><td>.</td><td>.</td><td>.</td><td>.</td><td>pfam08312</td><td>cwf21</td><td>cwf21 domain. The cwf21 family is involved in mRNA splicing. It has been isolated as a subcomplex of the splicosome in</td><td>GO:0000398|mRNA splicing, via spliceosome;</td><td>GO:0015030|Cajal body;GO:0071013|catalytic step 2 spliceosome;GO:0016607|nuclear speck;GO:0005654|nucleoplasm;</td><td>GO:0070742|C2H2 zinc finger domain binding;GO:0044822|poly(A) RNA</td><td>K13172</td><td>SRRM2, SRM300; serine/arginine repetitive matrix protein 2</td></tr>
<tr><td>RASSF5</td><td>160.2255</td><td>0.494849</td><td>0.169953006</td><td>2.911682</td><td>0.0035949</td><td>0.026444</td><td>Not_Sig</td><td>no</td><td>gi|166157482|ref|NP_001107232.1|</td><td>1774</td><td>0</td><td>ras association domain-containing protein 5 [Bos taurus]</td><td>gi|284813547|ref|NM_001113760.2|</td><td>3509</td><td>0</td><td>Bos taurus Ras association (RalGDS/AF-6) domain family member 5 (RASSF5), mRNA</td><td>Q8WWW0</td><td>596</td><td>0</td><td>Ras association domain-containing protein 5</td><td>.</td><td>.</td><td>.</td><td>.</td><td>pfam00788</td><td>RA</td><td>Ras association (RalGDS/AF-6) domain. RasGTP effectors (in cases of AF6, canoe and RalGDS); putative RasGTP effectors in other</td><td>GO:0006915|apoptotic process;GO:0035556|intracellular signal transduction;GO:0008285|negative regulation of cell proliferation;GO:0031398|positive regulation of protein ubiquitination;GO:0042981|regulation of apoptotic process;GO:1900180|regulation of protein localization to nucleus;</td><td>GO:0005737|cytoplasm;GO:0005874|microtubule;GO:0005634|nucleus;</td><td>GO:0046872|metal ion binding;</td><td>K08015</td><td>RASSF5, RAPL; Ras association domain-containing protein 5</td></tr>
<tr><td>DBI</td><td>1360.552</td><td>-0.48557</td><td>0.166811193</td><td>-2.91091</td><td>0.0036037</td><td>0.026477</td><td>Not_Sig</td><td>no</td><td>gi|594631605|ref|XP_007169332.1|</td><td>505</td><td>6e-61</td><td>PREDICTED: acyl-CoA-binding protein [Balaenoptera acutorostrata scammoni]</td><td>gi|742206085|ref|XM_010860785.1|</td><td>691</td><td>0</td><td>PREDICTED: Bison bison bison diazepam binding inhibitor (GABA receptor modulator, acyl-CoA binding protein) (DBI), mRNA</td><td>P07107</td><td>178</td><td>3e-55</td><td>Acyl-CoA-binding protein</td><td>SPBC1539.06</td><td>103</td><td>4.71e-28</td><td>COG4281</td><td>pfam00887</td><td>ACBP</td><td>Acyl CoA binding protein.</td><td>GO:0036151|phosphatidylcholine acyl-chain remodeling;GO:0006810|transport;</td><td>GO:0005783|endoplasmic reticulum;GO:0070062|extracellular exosome;GO:0005794|Golgi apparatus;</td><td>GO:0008289|lipid binding;GO:0036042|long-chain fatty acyl-CoA binding;</td><td>K08762</td><td>DBI, ACBP; diazepam-binding inhibitor (GABA receptor modulator,</td></tr>
<tr><td>RPL13A</td><td>7216.273</td><td>-0.28928</td><td>0.099379007</td><td>-2.91089</td><td>0.003604</td><td>0.026477</td><td>Not_Sig</td><td>no</td><td>gi|946794453|ref|XP_005879589.2|</td><td>1066</td><td>1e-144</td><td>PREDICTED: 60S ribosomal protein L13a [Myotis brandtii]</td><td>gi|942082539|ref|XM_005904989.2|</td><td>711</td><td>0</td><td>PREDICTED: Bos mutus ribosomal protein L13a (RPL13A), transcript variant X1, mRNA</td><td>Q3SZ90</td><td>384</td><td>2e-134</td><td>60S ribosomal protein L13a</td><td>SPBC839.13c</td><td>237</td><td>5.01e-79</td><td>COG0102</td><td>pfam00572</td><td>Ribosomal_L13</td><td>Ribosomal protein L13.</td><td>GO:1901194|negative regulation of formation of translation preinitiation complex;GO:0017148|negative regulation of translation;GO:0006412|translation;</td><td>GO:0022625|cytosolic large ribosomal subunit;GO:0097452|GAIT complex;</td><td>GO:0003729|mRNA binding;GO:0003735|structural constituent of</td><td>K02872</td><td>RP-L13Ae, RPL13A; large subunit ribosomal protein L13Ae</td></tr>
<tr><td>RASL10B</td><td>249.799</td><td>0.484858</td><td>0.166599352</td><td>2.910321</td><td>0.0036106</td><td>0.026501</td><td>Not_Sig</td><td>no</td><td>gi|694967496|ref|XP_009430660.1|</td><td>1228</td><td>3e-157</td><td>PREDICTED: ras-like protein family member 10B isoform X2 [Pan troglodytes]</td><td>gi|965797354|ref|XM_015090883.1|</td><td>2724</td><td>0</td><td>PREDICTED: Ovis aries musimon RAS-like family 10 member B (RASL10B), transcript variant X2, mRNA</td><td>Q96S79</td><td>421</td><td>4e-138</td><td>Ras-like protein family member 10B</td><td>.</td><td>.</td><td>.</td><td>.</td><td>pfam00071</td><td>Ras</td><td>Ras family. Includes sub-families Ras, Rab, Rac, Ral, Ran, Rap Ypt1 and more. Shares P-loop motif with GTP_EFTU, arf and</td><td>GO:0090277|positive regulation of peptide hormone secretion;GO:0003050|regulation of systemic arterial blood pressure by atrial natriuretic peptide;GO:0007264|small GTPase mediated signal transduction;</td><td>GO:0005622|intracellular;GO:0005886|plasma membrane;</td><td>GO:0005525|GTP binding;</td><td>K07851</td><td>RASL10B; Ras-like protein family member 10B</td></tr>
<tr><td>MAMSTR</td><td>38.13508</td><td>0.937031</td><td>0.321980494</td><td>2.910209</td><td>0.0036119</td><td>0.026501</td><td>UP</td><td>yes</td><td>gi|742163172|ref|XP_010847867.1|</td><td>1565</td><td>0</td><td>PREDICTED: MEF2-activating motif and SAP domain-containing transcriptional regulator isoform X2 [Bison bison bison]</td><td>gi|742163171|ref|XM_010849565.1|</td><td>3059</td><td>0</td><td>PREDICTED: Bison bison bison MEF2 activating motif and SAP domain containing transcriptional regulator (MAMSTR), transcript variant X2, mRNA</td><td>A7E346</td><td>425</td><td>2e-131</td><td>MEF2-activating motif and SAP domain-containing transcriptional regulator</td><td>.</td><td>.</td><td>.</td><td>.</td><td>pfam02037</td><td>SAP</td><td>SAP domain. The SAP (after SAF-A/B, Acinus and PIAS) motif is a putative DNA/RNA binding domain found in diverse nuclear and</td><td>GO:0010831|positive regulation of myotube differentiation;GO:0045944|positive regulation of transcription from RNA polymerase II promoter;GO:0006351|transcription, DNA-templated;</td><td>GO:0005634|nucleus;</td><td>GO:0001076|transcription factor activity, RNA polymerase II transcription factor</td><td>.</td><td>.</td></tr>
<tr><td>KIAA0930</td><td>1732.516</td><td>0.326478</td><td>0.112213637</td><td>2.90943</td><td>0.0036209</td><td>0.02655</td><td>Not_Sig</td><td>no</td><td>gi|926693271|ref|XP_013819861.1|</td><td>2045</td><td>0</td><td>PREDICTED: uncharacterized protein KIAA0930 homolog [Capra hircus]</td><td>gi|982982779|ref|XM_015463646.1|</td><td>5401</td><td>0</td><td>PREDICTED: Bos taurus KIAA0930 ortholog (KIAA0930), transcript variant X2, mRNA</td><td>Q6ICG6</td><td>765</td><td>0</td><td>Uncharacterized protein KIAA0930</td><td>.</td><td>.</td><td>.</td><td>.</td><td>pfam09741</td><td>DUF2045</td><td>Uncharacterized conserved protein (DUF2045). This entry is the conserved 250 residues of proteins of approximately 450 amino acids.</td><td>.</td><td>.</td><td>.</td><td>.</td><td>.</td></tr>
<tr><td>FERMT2</td><td>3497.814</td><td>-0.28758</td><td>0.098888394</td><td>-2.90817</td><td>0.0036355</td><td>0.026636</td><td>Not_Sig</td><td>no</td><td>gi|528966447|ref|XP_005211919.1|</td><td>3553</td><td>0</td><td>PREDICTED: fermitin family homolog 2 isoform X1 [Bos taurus]</td><td>gi|982937265|ref|XM_005211862.3|</td><td>3357</td><td>0</td><td>PREDICTED: Bos taurus fermitin family member 2 (FERMT2), transcript variant X1, mRNA</td><td>Q96AC1</td><td>1295</td><td>0</td><td>Fermitin family homolog 2</td><td>.</td><td>.</td><td>.</td><td>.</td><td>pfam00169</td><td>PH</td><td>PH domain. PH stands for pleckstrin homology.</td><td>GO:0034329|cell junction assembly;GO:0007160|cell-matrix adhesion;GO:0048041|focal adhesion assembly;GO:0033622|integrin activation;GO:0007229|integrin-mediated signaling pathway;GO:0072657|protein localization to membrane;GO:0008360|regulation of cell shape;GO:0034446|substrate adhesion-dependent cell spreading;GO:0007179|transforming growth factor beta receptor signaling pathway;GO:0016055|Wnt signaling pathway;</td><td>GO:0005938|cell cortex;GO:0009986|cell surface;GO:0005737|cytoplasm;GO:0005829|cytosol;GO:0031234|extrinsic component of cytoplasmic side of plasma</td><td>GO:0005547|phosphatidylinositol-3,4,5-trisphosphate binding;</td><td>K17083</td><td>FERMT2, KIND2; kindlin 2</td></tr>
<tr><td>KXD1</td><td>1183.222</td><td>0.332302</td><td>0.114270994</td><td>2.908017</td><td>0.0036373</td><td>0.026636</td><td>Not_Sig</td><td>no</td><td>gi|528954388|ref|XP_005208524.1|</td><td>776</td><td>4e-98</td><td>PREDICTED: kxDL motif-containing protein 1 isoform X1 [Bos taurus]</td><td>gi|942061169|ref|XM_005898312.2|</td><td>1397</td><td>0</td><td>PREDICTED: Bos mutus KxDL motif containing 1 (KXD1), transcript variant X2, mRNA</td><td>Q3SZV2</td><td>300</td><td>2e-98</td><td>KxDL motif-containing protein 1</td><td>.</td><td>.</td><td>.</td><td>.</td><td>pfam10241</td><td>KxDL</td><td>Uncharacterized conserved protein. This is a family of short proteins which are conserved over a region of 80 residues. There is a</td><td>GO:0032418|lysosome localization;GO:0016192|vesicle-mediated transport;</td><td>GO:0005765|lysosomal membrane;GO:0043234|protein complex;</td><td>.</td><td>.</td><td>.</td></tr>
<tr><td>CLASRP</td><td>313.4704</td><td>0.520682</td><td>0.179079821</td><td>2.907539</td><td>0.0036428</td><td>0.02666</td><td>Not_Sig</td><td>no</td><td>gi|556732158|ref|XP_005985944.1|</td><td>1058</td><td>4e-131</td><td>PREDICTED: LOW QUALITY PROTEIN: CLK4-associating serine/arginine rich protein [Pantholops hodgsonii]</td><td>gi|402693169|ref|NM_001077887.2|</td><td>2329</td><td>0</td><td>Bos taurus CLK4-associating serine/arginine rich protein (CLASRP), mRNA</td><td>A0JNI5</td><td>411</td><td>1e-130</td><td>CLK4-associating serine/arginine rich protein</td><td>.</td><td>.</td><td>.</td><td>.</td><td>pfam09750</td><td>DRY_EERY</td><td>Alternative splicing regulator. This entry represents the conserved N-terminal region of SWAP (suppressor-of-white-apricot</td><td>GO:0006397|mRNA processing;GO:0008380|RNA splicing;</td><td>GO:0005634|nucleus;</td><td>.</td><td>K13168</td><td>SFRS16; splicing factor, arginine/serine-rich 16</td></tr>
<tr><td>PPP1R10</td><td>931.8785</td><td>0.38642</td><td>0.132931137</td><td>2.906921</td><td>0.0036501</td><td>0.026696</td><td>Not_Sig</td><td>no</td><td>gi|219804658|ref|NP_001137335.1|</td><td>2355</td><td>0</td><td>serine/threonine-protein phosphatase 1 regulatory subunit 10 [Bos taurus]</td><td>gi|942088380|ref|XM_005911980.2|</td><td>4398</td><td>0</td><td>PREDICTED: Bos mutus protein phosphatase 1, regulatory subunit 10 (PPP1R10), mRNA</td><td>Q767K9</td><td>895</td><td>0</td><td>Serine/threonine-protein phosphatase 1 regulatory subunit 10</td><td>.</td><td>.</td><td>.</td><td>.</td><td>pfam08711</td><td>Med26</td><td>TFIIS helical bundle-like domain. Mediator is a large complex of up to 33 proteins that is conserved from plants to fungi to humans -</td><td>GO:0006351|transcription, DNA-templated;</td><td>GO:0000785|chromatin;GO:0000784|nuclear chromosome, telomeric region;GO:0005654|nucleoplasm;GO:0072357|PTW/PP1 phosphatase complex;</td><td>GO:0003677|DNA binding;GO:0046872|metal ion binding;GO:0044822</td><td>K17552</td><td>PPP1R10; protein phosphatase 1 regulatory subunit 10</td></tr>
<tr><td>LOC102268415</td><td>133.4626</td><td>0.655877</td><td>0.22566617</td><td>2.906404</td><td>0.0036561</td><td>0.02672</td><td>UP</td><td>yes</td><td>gi|548514121|ref|XP_005695466.1|</td><td>535</td><td>3e-64</td><td>PREDICTED: proline-rich receptor-like protein kinase PERK8 isoform X1 [Capra hircus]</td><td>gi|594112859|ref|XM_006079142.1|</td><td>524</td><td>0</td><td>PREDICTED: Bubalus bubalis uncharacterized LOC102406900 (LOC102406900), mRNA</td><td>.</td><td>.</td><td>.</td><td>.</td><td>.</td><td>.</td><td>.</td><td>.</td><td>pfam12238</td><td>MSA-2c</td><td>Merozoite surface antigen 2c. This family of proteins is found in eukaryotes. Proteins in this family are typically between 263 and 318</td><td>.</td><td>.</td><td>.</td><td>.</td><td>.</td></tr>
<tr><td>LAPTM4A</td><td>6880.485</td><td>-0.3035</td><td>0.104443082</td><td>-2.90593</td><td>0.0036616</td><td>0.02672</td><td>Not_Sig</td><td>no</td><td>gi|625267463|ref|XP_007625238.1|</td><td>1036</td><td>2e-135</td><td>PREDICTED: lysosomal-associated transmembrane protein 4A isoform X2 [Cricetulus griseus]</td><td>gi|942054252|ref|XM_005895648.2|</td><td>1399</td><td>0</td><td>PREDICTED: Bos mutus lysosomal protein transmembrane 4 alpha (LAPTM4A), mRNA</td><td>Q6QRN8</td><td>397</td><td>2e-135</td><td>Lysosomal-associated transmembrane protein 4A</td><td>.</td><td>.</td><td>.</td><td>.</td><td>pfam03821</td><td>Mtp</td><td>Golgi 4-transmembrane spanning transporter.</td><td>GO:0006810|transport;</td><td>GO:0005794|Golgi apparatus;GO:0016021|integral component of membrane;GO:0016020|membrane;</td><td>.</td><td>K12387</td><td>LAPTM; lysosomal-associated transmembrane protein</td></tr>
<tr><td>SEC31B</td><td>142.5708</td><td>0.515009</td><td>0.177231214</td><td>2.905857</td><td>0.0036625</td><td>0.02672</td><td>Not_Sig</td><td>no</td><td>gi|742200891|ref|XP_010857827.1|</td><td>5553</td><td>0</td><td>PREDICTED: protein transport protein Sec31B isoform X2 [Bison bison bison]</td><td>gi|742200890|ref|XM_010859525.1|</td><td>4998</td><td>0</td><td>PREDICTED: Bison bison bison SEC31 homolog B (S. cerevisiae) (SEC31B), transcript variant X2, mRNA</td><td>Q9NQW1</td><td>1711</td><td>0</td><td>Protein transport protein Sec31B</td><td>.</td><td>.</td><td>.</td><td>.</td><td>pfam03154</td><td>Atrophin-1</td><td>Atrophin-1 family. Atrophin-1 is the protein product of the dentatorubral-pallidoluysian atrophy (DRPLA) gene. DRPLA</td><td>GO:0015031|protein transport;GO:0016192|vesicle-mediated transport;</td><td>GO:0005789|endoplasmic reticulum membrane;GO:0012507|ER to Golgi transport vesicle membrane;GO:0030120|vesicle coat;</td><td>.</td><td>K14005</td><td>SEC31; protein transport protein SEC31</td></tr>
<tr><td>SEMA4B</td><td>76.33268</td><td>0.658498</td><td>0.22661222</td><td>2.905836</td><td>0.0036627</td><td>0.02672</td><td>UP</td><td>yes</td><td>gi|594068740|ref|XP_006058290.1|</td><td>4197</td><td>0</td><td>PREDICTED: semaphorin-4B isoform X1 [Bubalus bubalis]</td><td>gi|983003587|ref|XR_001498944.1|</td><td>4336</td><td>0</td><td>PREDICTED: Bos taurus uncharacterized LOC101908359 (LOC101908359), transcript variant X2, ncRNA</td><td>Q9NPR2</td><td>1296</td><td>0</td><td>Semaphorin-4B</td><td>SPAC9G1.03c</td><td>114</td><td>4.22e-28</td><td>COG1911</td><td>pfam01403</td><td>Sema</td><td>Sema domain. The Sema domain occurs in semaphorins, which are a large family of secreted and transmembrane proteins, some of</td><td>GO:0050919|negative chemotaxis;GO:0048843|negative regulation of axon extension involved in axon guidance;GO:0001755|neural crest cell migration;GO:0030335|positive regulation of cell migration;GO:0071526|semaphorin-plexin signaling pathway;</td><td>GO:0005615|extracellular space;GO:0016021|integral component of membrane;GO:0005886|plasma membrane;</td><td>GO:0045499|chemorepellent activity;GO:0030215|semaphorin</td><td>K06521</td><td>SEMA4; semaphorin 4</td></tr>
<tr><td>HAPSTR1</td><td>1005.665</td><td>-0.3069</td><td>0.105632514</td><td>-2.90536</td><td>0.0036683</td><td>0.026734</td><td>Not_Sig</td><td>no</td><td>gi|951062457|ref|XP_014466292.1|</td><td>1175</td><td>1e-148</td><td>PREDICTED: UPF0472 protein C16orf72 homolog [Alligator mississippiensis]</td><td>gi|528925652|ref|XM_863921.5|</td><td>3359</td><td>0</td><td>PREDICTED: Bos taurus chromosome 25 open reading frame, human C16orf72 (C25H16orf72), mRNA</td><td>Q28HY5</td><td>449</td><td>3e-147</td><td>UPF0472 protein C16orf72 homolog</td><td>.</td><td>.</td><td>.</td><td>.</td><td>pfam15251</td><td>DUF4588</td><td>Domain of unknown function (DUF4588). This family of proteins is found in eukaryotes. Proteins in this family are typically between</td><td>.</td><td>.</td><td>.</td><td>.</td><td>.</td></tr>
<tr><td>HCFC1R1</td><td>207.2728</td><td>0.498707</td><td>0.171654991</td><td>2.905286</td><td>0.0036692</td><td>0.026734</td><td>Not_Sig</td><td>no</td><td>gi|114051938|ref|NP_001039863.1|</td><td>513</td><td>4e-62</td><td>host cell factor C1 regulator 1 [Bos taurus]</td><td>gi|942028765|ref|XM_005886766.2|</td><td>827</td><td>0</td><td>PREDICTED: Bos mutus host cell factor C1 regulator 1 (XPO1 dependent) (HCFC1R1), transcript variant X4, mRNA</td><td>Q2M2S6</td><td>202</td><td>1e-63</td><td>Host cell factor C1 regulator 1</td><td>.</td><td>.</td><td>.</td><td>.</td><td>pfam15226</td><td>HPIP</td><td>HCF-1 beta-propeller-interacting protein family. HPIP is a small cellular polypeptide that binds to the beta-propeller domain of HCF-</td><td>.</td><td>GO:0005737|cytoplasm;GO:0005634|nucleus;</td><td>.</td><td>.</td><td>.</td></tr>
<tr><td>SLC17A9</td><td>1069.865</td><td>0.405911</td><td>0.139804367</td><td>2.903424</td><td>0.0036911</td><td>0.026876</td><td>Not_Sig</td><td>no</td><td>gi|154152059|ref|NP_001093848.1|</td><td>2045</td><td>0</td><td>solute carrier family 17 member 9 [Bos taurus]</td><td>gi|982943066|ref|XM_005214706.3|</td><td>2191</td><td>0</td><td>PREDICTED: Bos taurus solute carrier family 17 (vesicular nucleotide transporter), member 9 (SLC17A9), transcript variant X1, mRNA</td><td>Q9BYT1</td><td>665</td><td>0</td><td>Solute carrier family 17 member 9</td><td>.</td><td>.</td><td>.</td><td>.</td><td>pfam07690</td><td>MFS_1</td><td>Major Facilitator Superfamily.</td><td>GO:0006820|anion transport;GO:0006887|exocytosis;GO:1903955|positive regulation of protein targeting to mitochondrion;GO:1903146|regulation of mitophagy;GO:0055085|transmembrane transport;</td><td>GO:0016021|integral component of membrane;</td><td>GO:0022857|transmembrane transporter activity;</td><td>K12303</td><td>SLC17A9; MFS transporter, ACS family, solute carrier family 17 (sodium-</td></tr>
<tr><td>PICALM</td><td>6242.339</td><td>-0.30007</td><td>0.103363769</td><td>-2.90301</td><td>0.0036959</td><td>0.026894</td><td>Not_Sig</td><td>no</td><td>gi|803278515|ref|XP_011998932.1|</td><td>3665</td><td>0</td><td>PREDICTED: phosphatidylinositol-binding clathrin assembly protein isoform X2 [Ovis aries musimon]</td><td>gi|965858129|ref|XM_012143542.2|</td><td>3533</td><td>0</td><td>PREDICTED: Ovis aries musimon phosphatidylinositol binding clathrin assembly protein (PICALM), transcript variant X2, mRNA</td><td>Q7M6Y3</td><td>1282</td><td>0</td><td>Phosphatidylinositol-binding clathrin assembly protein</td><td>.</td><td>.</td><td>.</td><td>.</td><td>pfam07651</td><td>ANTH</td><td>ANTH domain. AP180 is an endocytotic accessory proteins that has been implicated in the formation of clathrin-coated pits.</td><td>GO:0007409|axonogenesis;GO:0035459|cargo loading into vesicle;GO:0008283|cell proliferation;GO:0048268|clathrin coat assembly;GO:0072583|clathrin-mediated endocytosis;GO:0048813|dendrite morphogenesis;GO:0016197|endosomal transport;GO:0030097|hemopoiesis;GO:0055072|iron ion homeostasis;GO:0097459|iron ion import into cell;GO:0010629|negative regulation of gene expression;GO:1902963|negative regulation of metalloendopeptidase activity involved in amyloid precursor protein catabolic</td><td>GO:0030122|AP-2 adaptor complex;GO:0005913|cell-cell adherens junction;GO:0005905|coated pit;GO:0005794|Golgi</td><td>GO:0005545|1-phosphatidylinositol binding;GO:0098641|cadherin binding</td><td>.</td><td>.</td></tr>
<tr><td>AAMDC</td><td>112.8668</td><td>-0.64159</td><td>0.221039383</td><td>-2.9026</td><td>0.0037008</td><td>0.026912</td><td>DOWN</td><td>yes</td><td>gi|555992712|ref|XP_005908127.1|</td><td>636</td><td>2e-81</td><td>PREDICTED: mth938 domain-containing protein [Bos mutus]</td><td>gi|982971158|ref|XM_010820831.2|</td><td>558</td><td>0</td><td>PREDICTED: Bos taurus adipogenesis associated, Mth938 domain containing (AAMDC), transcript variant X3, mRNA</td><td>Q32PA8</td><td>248</td><td>3e-82</td><td>Mth938 domain-containing protein</td><td>AF0029</td><td>53.9</td><td>7.43e-09</td><td>COG1504</td><td>pfam04430</td><td>DUF498</td><td>Protein of unknown function (DUF498/DUF598). This is a large family of uncharacterized proteins found in all domains of life. The</td><td>GO:0043066|negative regulation of apoptotic process;GO:0045600|positive regulation of fat cell differentiation;GO:0045944|positive regulation of transcription from RNA polymerase II promoter;</td><td>GO:0005737|cytoplasm;</td><td>.</td><td>.</td><td>.</td></tr>
<tr><td>AP1AR</td><td>236.9669</td><td>-0.44372</td><td>0.152913195</td><td>-2.90181</td><td>0.0037101</td><td>0.026964</td><td>Not_Sig</td><td>no</td><td>gi|125630699|ref|NP_001075001.1|</td><td>1288</td><td>2e-157</td><td>AP-1 complex-associated regulatory protein [Bos taurus]</td><td>gi|965857060|ref|XM_012143112.2|</td><td>3048</td><td>0</td><td>PREDICTED: Ovis aries musimon adaptor-related protein complex 1 associated regulatory protein (AP1AR), transcript variant X4, mRNA</td><td>Q63HQ0</td><td>463</td><td>8e-146</td><td>AP-1 complex-associated regulatory protein</td><td>.</td><td>.</td><td>.</td><td>.</td><td>pfam07946</td><td>DUF1682</td><td>Protein of unknown function (DUF1682). The members of this family are all hypothetical eukaryotic proteins of unknown</td><td>GO:0034613|cellular protein localization;GO:2000146|negative regulation of cell motility;GO:0001920|negative regulation of receptor recycling;GO:1900025|negative regulation of substrate adhesion-dependent cell spreading;GO:0015031|protein transport;GO:0034315|regulation of Arp2/3 complex-mediated actin nucleation;GO:0048203|vesicle targeting, trans-Golgi to endosome;</td><td>GO:0005829|cytosol;GO:0005769|early endosome;GO:0005794|Golgi apparatus;GO:0005770|late endosome;GO:0030133|transport vesicle;</td><td>GO:0035650|AP-1 adaptor complex binding;GO:0019894|kinesin binding;</td><td>.</td><td>.</td></tr>
<tr><td>EFR3A</td><td>2379.005</td><td>-0.34821</td><td>0.120032622</td><td>-2.90095</td><td>0.0037203</td><td>0.027021</td><td>Not_Sig</td><td>no</td><td>gi|594113340|ref|XP_006079422.1|</td><td>4357</td><td>0</td><td>PREDICTED: protein EFR3 homolog A isoform X1 [Bubalus bubalis]</td><td>gi|942069408|ref|XM_005900733.2|</td><td>5082</td><td>0</td><td>PREDICTED: Bos mutus EFR3 homolog A (EFR3A), mRNA</td><td>Q14156</td><td>1645</td><td>0</td><td>Protein EFR3 homolog A</td><td>.</td><td>.</td><td>.</td><td>.</td><td>pfam09602</td><td>PhaP_Bmeg</td><td>Polyhydroxyalkanoic acid inclusion protein (PhaP_Bmeg). This entry describes a protein found in polyhydroxyalkanoic acid (PHA)</td><td>GO:0090002|establishment of protein localization to plasma membrane;GO:0046854|phosphatidylinositol phosphorylation;</td><td>GO:0005829|cytosol;GO:0070062|extracellular exosome;GO:0005886|plasma membrane;</td><td>.</td><td>.</td><td>.</td></tr>
<tr><td>NXF1</td><td>403.2198</td><td>0.388327</td><td>0.133874411</td><td>2.900678</td><td>0.0037236</td><td>0.027027</td><td>Not_Sig</td><td>no</td><td>gi|115495603|ref|NP_001069023.1|</td><td>2939</td><td>0</td><td>nuclear RNA export factor 1 [Bos taurus]</td><td>gi|965863392|ref|XM_012146230.2|</td><td>3145</td><td>0</td><td>PREDICTED: Ovis aries musimon nuclear RNA export factor 1 (NXF1), mRNA</td><td>Q1RMS5</td><td>1136</td><td>0</td><td>Nuclear RNA export factor 1</td><td>.</td><td>.</td><td>.</td><td>.</td><td>pfam09162</td><td>Tap-RNA_bind</td><td>Tap, RNA-binding. Members of this family adopt a structure consisting of an alpha+beta sandwich with an antiparallel beta-sheet, arranged in</td><td>GO:0016973|poly(A)+ mRNA export from nucleus;</td><td>GO:0005737|cytoplasm;GO:0042405|nuclear inclusion body;GO:0005643|nuclear pore;GO:0016607|nuclear speck;GO:0005634|nucleus;GO:0000346|tr</td><td>GO:0003729|mRNA binding;GO:0005487|nucleocytoplasmic transporter</td><td>K14284</td><td>NXF, TAP, MEX67; nuclear RNA export factor</td></tr>
<tr><td>RAMP3</td><td>254.7273</td><td>0.486534</td><td>0.167747063</td><td>2.900405</td><td>0.0037268</td><td>0.027033</td><td>Not_Sig</td><td>no</td><td>gi|440909232|gb|ELR59163.1|</td><td>618</td><td>5e-74</td><td>Receptor activity-modifying protein 3, partial [Bos mutus]</td><td>gi|942043692|ref|XM_005891866.2|</td><td>1340</td><td>0</td><td>PREDICTED: Bos mutus receptor (G protein-coupled) activity modifying protein 3 (RAMP3), mRNA</td><td>Q7YS88</td><td>209</td><td>8e-63</td><td>Receptor activity-modifying protein 3</td><td>.</td><td>.</td><td>.</td><td>.</td><td>pfam04901</td><td>RAMP</td><td>Receptor activity modifying family. The calcitonin-receptor-like receptor can function as either a calcitonin-gene-related peptide or</td><td>GO:0071392|cellular response to estradiol stimulus;GO:0086103|G-protein coupled receptor signaling pathway involved in heart process;GO:0006886|intracellular protein transport;GO:0090004|positive regulation of establishment of protein localization to plasma membrane;GO:0008277|regulation of G-protein coupled receptor protein signaling pathway;</td><td>GO:0016021|integral component of membrane;GO:0005622|intracellular;GO:0005886|plasma membrane;</td><td>GO:0008565|protein transporter activity;</td><td>K08449</td><td>RAMP3; receptor activity modifying protein 3</td></tr>
<tr><td>OSBPL7</td><td>107.3135</td><td>0.622259</td><td>0.214591581</td><td>2.899736</td><td>0.0037348</td><td>0.027074</td><td>UP</td><td>yes</td><td>gi|942060323|ref|XP_014334370.1|</td><td>4260</td><td>0</td><td>PREDICTED: oxysterol-binding protein-related protein 7 isoform X2 [Bos mutus]</td><td>gi|594102654|ref|XM_006074323.1|</td><td>4294</td><td>0</td><td>PREDICTED: Bubalus bubalis oxysterol binding protein-like 7 (OSBPL7), transcript variant X3, mRNA</td><td>Q9BZF2</td><td>1537</td><td>0</td><td>Oxysterol-binding protein-related protein 7</td><td>.</td><td>.</td><td>.</td><td>.</td><td>pfam01237</td><td>Oxysterol_BP</td><td>Oxysterol-binding protein.</td><td>GO:0071397|cellular response to cholesterol;GO:0006869|lipid transport;GO:1901800|positive regulation of proteasomal protein catabolic process;GO:0010506|regulation of autophagy;</td><td>GO:0005776|autophagosome;GO:0005829|cytosol;GO:0005789|endoplasmic reticulum membrane;GO:0097038|perinuclear endoplasmic reticulum;GO:0005886|plasma</td><td>GO:0015485|cholesterol binding;</td><td>.</td><td>.</td></tr>
<tr><td>TMEM67</td><td>351.9703</td><td>-0.41276</td><td>0.142377725</td><td>-2.89907</td><td>0.0037427</td><td>0.027115</td><td>Not_Sig</td><td>no</td><td>gi|555967083|ref|XP_005895590.1|</td><td>5128</td><td>0</td><td>PREDICTED: meckelin [Bos mutus]</td><td>gi|942053905|ref|XM_005895528.2|</td><td>4124</td><td>0</td><td>PREDICTED: Bos mutus transmembrane protein 67 (TMEM67), mRNA</td><td>Q5HYA8</td><td>1788</td><td>0</td><td>Meckelin</td><td>.</td><td>.</td><td>.</td><td>.</td><td>pfam09773</td><td>Meckelin</td><td>Meckelin (Transmembrane protein 67). Members of this family are thought to be related to the ciliary basal body. Defects result in</td><td>GO:0042384|cilium assembly;GO:0060271|cilium morphogenesis;GO:0030433|ER-associated ubiquitin-dependent protein catabolic process;GO:0010826|negative regulation of centrosome duplication;</td><td>GO:0005813|centrosome;GO:0060170|ciliary membrane;GO:0035869|ciliary transition zone;GO:0030659|cytoplasmic vesicle membrane;GO:0005789|endoplasmic</td><td>GO:0031005|filamin binding;GO:0051082|unfolded protein binding;</td><td>K19348</td><td>TMEM67, MKS3; meckelin</td></tr>
<tr><td>BAX</td><td>774.3575</td><td>-0.36891</td><td>0.127280065</td><td>-2.89841</td><td>0.0037506</td><td>0.027155</td><td>Not_Sig</td><td>no</td><td>gi|926714829|ref|XP_005692753.2|</td><td>1006</td><td>5e-136</td><td>PREDICTED: apoptosis regulator BAX isoform X1 [Capra hircus]</td><td>gi|555966764|ref|XM_005895371.1|</td><td>688</td><td>0</td><td>PREDICTED: Bos mutus BCL2-associated X protein (BAX), transcript variant X1, mRNA</td><td>O02703</td><td>390</td><td>2e-137</td><td>Apoptosis regulator BAX</td><td>.</td><td>.</td><td>.</td><td>.</td><td>pfam00452</td><td>Bcl-2</td><td>Apoptosis regulator proteins, Bcl-2 family.</td><td>GO:0006919|activation of cysteine-type endopeptidase activity involved in apoptotic process;GO:0008635|activation of cysteine-type endopeptidase activity involved in apoptotic process by cytochrome c;GO:0097296|activation of cysteine-type endopeptidase activity involved in apoptotic signaling pathway;GO:0008637|apoptotic mitochondrial changes;GO:1902263|apoptotic process involved in embryonic digit morphogenesis;GO:1902262|apoptotic process involved in patterning of blood vessels;GO:0097190|apoptotic signaling</td><td>GO:0097144|BAX complex;GO:0097136|Bcl-2 family protein complex;GO:0005829|cytosol;GO:0005783|endoplasmic</td><td>GO:0051434|BH3 domain binding;GO:0015267|channel</td><td>K02159</td><td>BAX; apoptosis regulator BAX</td></tr>
<tr><td>PCBP1</td><td>3273.139</td><td>0.350035</td><td>0.120801104</td><td>2.897616</td><td>0.0037601</td><td>0.027206</td><td>Not_Sig</td><td>no</td><td>gi|6754994|ref|NP_035995.1|</td><td>1874</td><td>0</td><td>poly(rC)-binding protein 1 [Mus musculus]</td><td>gi|926704726|ref|XM_005686838.2|</td><td>1672</td><td>0</td><td>PREDICTED: Capra hircus poly(rC) binding protein 1 (PCBP1), mRNA</td><td>O19048</td><td>726</td><td>0</td><td>Poly(rC)-binding protein 1</td><td>.</td><td>.</td><td>.</td><td>.</td><td>pfam00013</td><td>KH_1</td><td>KH domain. KH motifs bind RNA in vitro. Autoantibodies to Nova, a KH domain protein, cause paraneoplastic opsoclonus ataxia.</td><td>GO:0045944|positive regulation of transcription from RNA polymerase II promoter;GO:0039694|viral RNA genome replication;</td><td>GO:0005737|cytoplasm;GO:0070062|extracellular exosome;GO:0030529|ribonucleoprotein complex;GO:0016020|membrane;GO:00056</td><td>GO:0003730|mRNA 3'-UTR binding;GO:0000981|RNA polymerase II</td><td>K12889</td><td>PCBP1; poly(rC)-binding protein 1</td></tr>
<tr><td>KIF5B</td><td>5350.609</td><td>-0.38919</td><td>0.134357828</td><td>-2.89669</td><td>0.0037712</td><td>0.02727</td><td>Not_Sig</td><td>no</td><td>gi|742202778|ref|XP_010858304.1|</td><td>4890</td><td>0</td><td>PREDICTED: kinesin-1 heavy chain isoform X2 [Bison bison bison]</td><td>gi|982942232|ref|XM_005214188.3|</td><td>5659</td><td>0</td><td>PREDICTED: Bos taurus kinesin family member 5B (KIF5B), transcript variant X1, mRNA</td><td>P33176</td><td>1863</td><td>0</td><td>Kinesin-1 heavy chain</td><td>SPAC1834.07</td><td>316</td><td>2.83e-93</td><td>COG5059</td><td>pfam00225</td><td>Kinesin</td><td>Kinesin motor domain.</td><td>GO:0007411|axon guidance;GO:0007028|cytoplasm organization;GO:0030705|cytoskeleton-dependent intracellular transport;GO:0007018|microtubule-based movement;GO:0072383|plus-end-directed vesicle transport along microtubule;GO:0090004|positive regulation of establishment of protein localization to plasma membrane;GO:0035774|positive regulation of insulin secretion involved in cellular response to glucose stimulus;GO:0090316|positive regulation of intracellular protein</td><td>GO:0044295|axonal growth cone;GO:0005913|cell-cell adherens junction;GO:0035253|ciliary rootlet;GO:0005737|cytoplasm;GO:0030139</td><td>GO:0005524|ATP binding;GO:0008574|ATP-dependent microtubule motor</td><td>K10396</td><td>KIF5; kinesin family member 5</td></tr>
<tr><td>LOC106700497</td><td>878.7871</td><td>-0.33377</td><td>0.11525247</td><td>-2.89602</td><td>0.0037793</td><td>0.027302</td><td>Not_Sig</td><td>no</td><td>gi|157785573|ref|NP_001099117.1|</td><td>673</td><td>2e-79</td><td>interferon alpha responsive isoform 2 [Bos taurus]</td><td>gi|982948240|ref|XM_010813375.2|</td><td>2766</td><td>0</td><td>PREDICTED: Bos taurus torsin A interacting protein 2 (TOR1AIP2), transcript variant X3, mRNA</td><td>Q9H496</td><td>263</td><td>8e-81</td><td>Torsin-1A-interacting protein 2, isoform IFRG15</td><td>.</td><td>.</td><td>.</td><td>.</td><td>pfam09231</td><td>RDV-p3</td><td>Rice dwarf virus p3. Members of this family are core structural proteins found in the double-stranded RNA virus Phytoreovirus.</td><td>.</td><td>.</td><td>.</td><td>.</td><td>.</td></tr>
<tr><td>OGG1</td><td>208.5344</td><td>0.459288</td><td>0.158598137</td><td>2.895925</td><td>0.0037804</td><td>0.027302</td><td>Not_Sig</td><td>no</td><td>gi|161760649|ref|NP_001073754.2|</td><td>1850</td><td>0</td><td>N-glycosylase/DNA lyase [Bos taurus]</td><td>gi|742133601|ref|XM_010842087.1|</td><td>1761</td><td>0</td><td>PREDICTED: Bison bison bison 8-oxoguanine DNA glycosylase (OGG1), mRNA</td><td>O15527</td><td>617</td><td>0</td><td>N-glycosylase/DNA lyase</td><td>YML060w</td><td>193</td><td>4.51e-55</td><td>COG0122</td><td>pfam07934</td><td>OGG_N</td><td>8-oxoguanine DNA glycosylase, N-terminal domain. The presence of 8-oxoguanine residues in DNA can give rise to G-C to T-A transversion</td><td>GO:0002526|acute inflammatory response;GO:0007568|aging;GO:0006284|base-excision repair;GO:0071276|cellular response to cadmium ion;GO:0006974|cellular response to DNA damage stimulus;GO:0045007|depurination;GO:0045008|depyrimidination;GO:0043066|negative regulation of apoptotic process;GO:1901291|negative regulation of double-strand break repair via single-strand annealing;GO:0033683|nucleotide-excision repair, DNA incision;GO:0033158|regulation of protein import into nucleus,</td><td>GO:0005739|mitochondrion;GO:0016363|nuclear matrix;GO:0016607|nuclear speck;GO:0005654|nucleoplasm;GO:0005634|nucleus;GO:0043234|protein complex;</td><td>GO:0034039|8-oxo-7,8-dihydroguanine DNA N-glycosylase activity;GO:0003684</td><td>K03660</td><td>OGG1; N-glycosylase/DNA lyase [EC:3.2.2.- 4.2.99.18]</td></tr>
<tr><td>PRC1</td><td>1159.346</td><td>0.345566</td><td>0.119356327</td><td>2.895246</td><td>0.0037886</td><td>0.027339</td><td>Not_Sig</td><td>no</td><td>gi|555966339|ref|XP_005895222.1|</td><td>2967</td><td>0</td><td>PREDICTED: protein regulator of cytokinesis 1 isoform X1 [Bos mutus]</td><td>gi|942053029|ref|XM_005895160.2|</td><td>2958</td><td>0</td><td>PREDICTED: Bos mutus protein regulator of cytokinesis 1 (PRC1), transcript variant X1, mRNA</td><td>O43663</td><td>1054</td><td>0</td><td>Protein regulator of cytokinesis 1</td><td>.</td><td>.</td><td>.</td><td>.</td><td>pfam03999</td><td>MAP65_ASE1</td><td>Microtubule associated protein (MAP65/ASE1 family).</td><td>GO:0000910|cytokinesis;GO:0001578|microtubule bundle formation;GO:0000022|mitotic spindle elongation;GO:0008284|positive regulation of cell proliferation;</td><td>GO:0070938|contractile ring;GO:0005737|cytoplasm;GO:0005829|cytosol;GO:0015630|microtubule cytoskeleton;GO:0030496|midbody;GO:000</td><td>GO:0042802|identical protein binding;GO:0019894|kinesin</td><td>K16732</td><td>PRC1; protein regulator of cytokinesis 1</td></tr>
<tr><td>LTBP1</td><td>7154.614</td><td>-0.25909</td><td>0.089492443</td><td>-2.8951</td><td>0.0037903</td><td>0.027339</td><td>Not_Sig</td><td>no</td><td>gi|440908298|gb|ELR58333.1|</td><td>8370</td><td>0</td><td>Latent-transforming growth factor beta-binding protein 1, partial [Bos mutus]</td><td>gi|926703801|ref|XM_013967526.1|</td><td>5759</td><td>0</td><td>PREDICTED: Capra hircus latent transforming growth factor beta binding protein 1 (LTBP1), mRNA</td><td>Q14766</td><td>3040</td><td>0</td><td>Latent-transforming growth factor beta-binding protein 1</td><td>.</td><td>.</td><td>.</td><td>.</td><td>pfam00683</td><td>TB</td><td>TB domain. This domain is also known as the 8 cysteine domain. This family includes the hybrid domains. This cysteine rich repeat</td><td>GO:0035904|aorta development;GO:0060976|coronary vasculature development;GO:0035583|sequestering of TGFbeta in extracellular matrix;GO:0003281|ventricular septum development;</td><td>GO:0031012|extracellular matrix;GO:0005576|extracellular region;GO:0001527|microfibril;GO:0043234|protein</td><td>GO:0005509|calcium ion binding;GO:0050436|microfibril</td><td>K19559</td><td>LTBP1; latent transforming growth factor beta binding protein 1</td></tr>
<tr><td>MBD3</td><td>1092.691</td><td>0.315982</td><td>0.109155022</td><td>2.894803</td><td>0.003794</td><td>0.027348</td><td>Not_Sig</td><td>no</td><td>gi|803222428|ref|XP_011971485.1|</td><td>1427</td><td>0</td><td>PREDICTED: methyl-CpG-binding domain protein 3 isoform X2 [Ovis aries musimon]</td><td>gi|742142255|ref|XM_010844557.1|</td><td>2061</td><td>0</td><td>PREDICTED: Bison bison bison methyl-CpG binding domain protein 3 (MBD3), transcript variant X2, mRNA</td><td>O95983</td><td>536</td><td>0</td><td>Methyl-CpG-binding domain protein 3</td><td>.</td><td>.</td><td>.</td><td>.</td><td>pfam14048</td><td>MBD_C</td><td>C-terminal domain of methyl-CpG binding protein 2 and 3. CpG-methylation is a frequently occurring epigenetic modification</td><td>GO:0007568|aging;GO:0043044|ATP-dependent chromatin remodeling;GO:0007420|brain development;GO:0048568|embryonic organ development;GO:0007507|heart development;GO:0016573|histone acetylation;GO:0001701|in utero embryonic development;GO:0006346|methylation-dependent chromatin silencing;GO:0000122|negative regulation of transcription from RNA polymerase II promoter;GO:0044030|regulation of DNA methylation;GO:1901796|regulation of signal transduction by p53 class</td><td>GO:0005737|cytoplasm;GO:0000792|heterochromatin;GO:0000790|nuclear chromatin;GO:0005654|nucleoplasm;GO:0016581|NuRD complex;GO:0043234|protein</td><td>GO:0003682|chromatin binding;GO:0003677|DNA</td><td>K11591</td><td>MBD3; methyl-CpG-binding domain protein 3</td></tr>
<tr><td>FOXJ2</td><td>1043.545</td><td>0.38838</td><td>0.134181053</td><td>2.894451</td><td>0.0037982</td><td>0.027352</td><td>Not_Sig</td><td>no</td><td>gi|521023772|gb|EPQ05560.1|</td><td>2437</td><td>0</td><td>Forkhead box protein J2 [Myotis brandtii]</td><td>gi|982926862|ref|XM_005207076.3|</td><td>4152</td><td>0</td><td>PREDICTED: Bos taurus forkhead box J2 (FOXJ2), transcript variant X1, mRNA</td><td>Q9P0K8</td><td>798</td><td>0</td><td>Forkhead box protein J2</td><td>ECU11g1330</td><td>108</td><td>1.94e-25</td><td>COG5025</td><td>pfam00250</td><td>Fork_head</td><td>Fork head domain.</td><td>GO:0045944|positive regulation of transcription from RNA polymerase II promoter;GO:0045893|positive regulation of transcription, DNA-templated;</td><td>GO:0005730|nucleolus;GO:0005634|nucleus;</td><td>GO:0000978|RNA polymerase II core promoter proximal region sequence-</td><td>K09403</td><td>FOXJ2_3; forkhead box protein J2/3</td></tr>
<tr><td>RTN4</td><td>7673.671</td><td>-0.24777</td><td>0.085608279</td><td>-2.89421</td><td>0.0038011</td><td>0.027352</td><td>Not_Sig</td><td>no</td><td>gi|742132031|ref|XP_010839998.1|</td><td>4800</td><td>0</td><td>PREDICTED: reticulon-4 [Bison bison bison]</td><td>gi|982938638|ref|XM_005212571.3|</td><td>4686</td><td>0</td><td>PREDICTED: Bos taurus reticulon 4 (RTN4), transcript variant X1, mRNA</td><td>Q9NQC3</td><td>1452</td><td>0</td><td>Reticulon-4</td><td>.</td><td>.</td><td>.</td><td>.</td><td>pfam02453</td><td>Reticulon</td><td>Reticulon. Reticulon, also know as neuroendocrine-specific protein (NSP), is a protein of unknown function which associates with the</td><td>GO:0006915|apoptotic process;GO:0007413|axonal fasciculation;GO:0060317|cardiac epithelial to mesenchymal transition;GO:0021801|cerebral cortex radial glia guided migration;GO:0071787|endoplasmic reticulum tubular network assembly;GO:0071786|endoplasmic reticulum tubular network organization;GO:0030517|negative regulation of axon extension;GO:0050771|negative regulation of axonogenesis;GO:0030308|negative regulation of cell growth;GO:0051292|nuclear pore</td><td>GO:0042995|cell projection;GO:0005913|cell-cell adherens junction;GO:0005783|endoplasmic reticulum;GO:0005789|endoplasmic</td><td>GO:0098641|cadherin binding involved in cell-cell adhesion;GO:00448</td><td>.</td><td>.</td></tr>
<tr><td>RIPOR1</td><td>1476.767</td><td>0.3953</td><td>0.13658485</td><td>2.894169</td><td>0.0038016</td><td>0.027352</td><td>Not_Sig</td><td>no</td><td>gi|742168586|ref|XP_010849341.1|</td><td>3036</td><td>0</td><td>PREDICTED: protein FAM65A isoform X1 [Bison bison bison]</td><td>gi|742168585|ref|XM_010851039.1|</td><td>5329</td><td>0</td><td>PREDICTED: Bison bison bison family with sequence similarity 65, member A (FAM65A), transcript variant X1, mRNA</td><td>Q6ZS17</td><td>931</td><td>0</td><td>Protein FAM65A</td><td>.</td><td>.</td><td>.</td><td>.</td><td>pfam13646</td><td>HEAT_2</td><td>HEAT repeats. This family includes multiple HEAT repeats.</td><td>.</td><td>GO:0005737|cytoplasm;GO:0070062|extracellular exosome;</td><td>.</td><td>.</td><td>.</td></tr>
<tr><td>HSPB6</td><td>626.8902</td><td>0.472151</td><td>0.163150817</td><td>2.893954</td><td>0.0038042</td><td>0.027353</td><td>Not_Sig</td><td>no</td><td>gi|803146703|ref|XP_012045587.1|</td><td>656</td><td>1e-79</td><td>PREDICTED: heat shock protein beta-6 isoform X1 [Ovis aries]</td><td>gi|742161325|ref|XM_010849030.1|</td><td>1422</td><td>0</td><td>PREDICTED: Bison bison bison heat shock protein, alpha-crystallin-related, B6 (HSPB6), mRNA</td><td>Q148F8</td><td>239</td><td>8e-75</td><td>Heat shock protein beta-6</td><td>.</td><td>.</td><td>.</td><td>.</td><td>pfam00011</td><td>HSP20</td><td>Hsp20/alpha crystallin family.</td><td>.</td><td>GO:0005737|cytoplasm;GO:0005634|nucleus;</td><td>GO:0042803|protein homodimerization activity;GO:0005212|structural</td><td>K09545</td><td>HSPB6; heat shock protein beta-6</td></tr>
</table>

NCOA6	566.2862	0.359495	0.124232937	2.893717	0.0038071	0.027357	Not_Sig	no	gi|440902205|gb|ELR53021.1|	8539	0	Nuclear receptor coactivator 6, partial [Bos mutus]	gi|982993625|ref|XM_015466156.1|	7017	0	PREDICTED: Bos taurus nuclear receptor coactivator 6 (NCOA6), transcript variant X2, mRNA	Q14686	1667	0	Nuclear receptor coactivator 6	.	.	.	.	pfam13820	Nucleic_acid_bd	Putative nucleic acid-binding region. This is a family of putative nucleic acid-binding proteins. Several members are annotated as	GO:0007420|brain development;GO:0044255|cellular lipid metabolic process;GO:0006974|cellular response to DNA damage stimulus;GO:0006310|DNA recombination;GO:0006281|DNA repair;GO:0006260|DNA replication;GO:0006352|DNA-templated transcription, initiation;GO:0042921|glucocorticoid receptor signaling pathway;GO:0007507|heart development;GO:0030520|intracellular estrogen receptor signaling pathway;GO:0030099|myeloid cell differentiation;GO:0045944|positive regulation of transcription from RNA polymerase II	GO:0035097|histone methyltransferase complex;GO:0043231|intracellular membrane-bounded organelle;GO:0005654|nucleoplasm;GO:000	GO:0003682|chromatin binding;GO:0019899|enzyme	K14971	NCOA6, ASC2; nuclear receptor coactivator 6
PTS	164.1789	-0.49364	0.170691758	-2.892	0.0038279	0.027472	Not_Sig	no	gi|550822062|ref|NP_001272622.1|	534	1e-97	p97Bcnt protein [Capra hircus]	gi|850489648|gb|CP011900.1|	2002	0	Ovis canadensis canadensis isolate 43U chromosome 15 sequence	O02751	209	7e-97	Craniofacial development protein 2	alr4554	67.4	5.14e-16	COG0720	pfam01242	PTPS	6-pyruvoyl tetrahydropterin synthase. 6-Pyruvoyl tetrahydrobiopterin synthase catalyses the conversion of	.	GO:0005737|cytoplasm;GO:0005634|nucleus;	.	.	.
SIN3B	889.8078	0.34394	0.11892958	2.891967	0.0038284	0.027472	Not_Sig	no	gi|359066803|ref|XP_003586291.1|	5646	0	PREDICTED: paired amphipathic helix protein Sin3b isoform X1 [Bos taurus]	gi|982984162|ref|XM_003582367.4|	4930	0	PREDICTED: Bos taurus SIN3 transcription regulator family member B (SIN3B), transcript variant X1, mRNA	O75182	2062	0	Paired amphipathic helix protein Sin3b	SPBC12C2.10c	285	2.60e-80	COG5602	pfam08295	Sin3_corepress	Sin3 family co-repressor. This domain is found on transcriptional regulators. It forms interactions with histone deacetylases.	GO:0048738|cardiac muscle tissue development;GO:0044255|cellular lipid metabolic process;GO:0016575|histone deacetylation;GO:0000122|negative regulation of transcription from RNA polymerase II promoter;GO:0007519|skeletal muscle tissue development;GO:0006351|transcription, DNA-templated;	GO:0030849|autosome;GO:0005737|cytoplasm;GO:0005654|nucleoplasm;GO:0005634|nucleus;GO:0016580|Sin3 complex;GO:0000805|X	GO:0003682|chromatin binding;GO:0001106|RNA polymerase II	.	.
ARRB2	111.5375	0.624747	0.216052676	2.891642	0.0038323	0.027472	UP	yes	gi|328550530|ref|NP_001192206.1|	1924	0	beta-arrestin-2 [Bos taurus]	gi|982956493|ref|XM_005220181.3|	1766	0	PREDICTED: Bos taurus arrestin, beta 2 (ARRB2), transcript variant X1, mRNA	P32120	743	0	Beta-arrestin-2	.	.	.	.	pfam00339	Arrestin_N	Arrestin (or S-antigen), N-terminal domain. Ig-like beta-sandwich fold. Scop reports duplication with C-terminal domain.	GO:0002029|desensitization of G-protein coupled receptor protein signaling pathway;GO:0002031|G-protein coupled receptor internalization;GO:0002092|positive regulation of receptor internalization;GO:0015031|protein transport;GO:0031623|receptor internalization;GO:0007165|signal transduction;	GO:0005905|coated pit;GO:0005737|cytoplasm;GO:0030139|endocytic vesicle;GO:0005634|nucleus;GO:0005886|p	GO:0000822|inositol hexakisphosphate binding;GO:0035091|phosphatidylinosit	.	.
RBM38	201.5737	0.654475	0.226342234	2.891527	0.0038337	0.027472	UP	yes	gi|329664824|ref|NP_001192952.1|	849	3e-105	RNA-binding protein 38 [Bos taurus]	gi|329664823|ref|NM_001206023.1|	2122	0	Bos taurus RNA binding motif protein 38 (RBM38), mRNA	Q9H0Z9	311	4e-99	RNA-binding protein 38	TP0356	61.2	9.70e-11	COG0724	pfam00076	RRM_1	RNA recognition motif. (a.k.a. RRM, RBD, or RNP domain). The RRM motif is probably diagnostic of an RNA binding protein. RRMs	GO:0070935|3'-UTR-mediated mRNA stabilization;GO:0007049|cell cycle;GO:0007050|cell cycle arrest;GO:0030154|cell differentiation;GO:0006977|DNA damage response, signal transduction by p53 class mediator resulting in cell cycle arrest;GO:0006978|DNA damage response, signal transduction by p53 class mediator resulting in transcription of p21 class mediator;GO:0006397|mRNA processing;GO:0008285|negative regulation of cell proliferation;GO:0010830|regulation of myotube differentiation;GO:0043484|regulation of	GO:0005829|cytosol;GO:0005634|nucleus;	GO:0003730|mRNA 3'-UTR binding;GO:0003729|mRNA	.	.
KIF22	464.8761	0.360128	0.124550692	2.891417	0.0038351	0.027472	Not_Sig	no	gi|803240217|ref|XP_011980205.1|	3018	0	PREDICTED: kinesin-like protein KIF22 [Ovis aries musimon]	gi|555953405|ref|XM_005888817.1|	2117	0	PREDICTED: Bos mutus kinesin family member 22 (KIF22), mRNA	A6QPL4	1144	0	Kinesin-like protein KIF22	ECU11g2020	223	9.15e-63	COG5059	pfam00225	Kinesin	Kinesin motor domain.	GO:0006281|DNA repair;GO:0051310|metaphase plate congression;GO:0007018|microtubule-based movement;GO:0007080|mitotic metaphase plate congression;GO:0007062|sister chromatid cohesion;	GO:0000785|chromatin;GO:0005737|cytoplasm;GO:0005925|focal adhesion;GO:0005871|kinesin complex;GO:0005874|microtubule;GO:0072	GO:0005524|ATP binding;GO:0016887|ATPase activity;GO:0003677	K10403	KIF22; kinesin family member 22
LOC106701260	348.8619	-0.39807	0.137696932	-2.89092	0.0038411	0.027498	Not_Sig	no	.	.	.	.	gi|982983545|ref|XR_231448.3|	1748	0	PREDICTED: Bos taurus uncharacterized LOC100847947 (LOC100847947), transcript variant X2, ncRNA	.	.	.	.	.	.	.	.	.	.	.	.	.	.	.	.
CEP290	176.3939	-0.5001	0.17301878	-2.89042	0.0038473	0.02751	Not_Sig	no	gi|555998557|ref|XP_005910988.1|	10382	0	PREDICTED: centrosomal protein of 290 kDa [Bos mutus]	gi|742237874|ref|XM_010832046.1|	6979	0	PREDICTED: Bison bison bison centrosomal protein 290kDa (CEP290), transcript variant X1, mRNA	O15078	3631	0	Centrosomal protein of 290 kDa	.	.	.	.	pfam07888	CALCOCO1	Calcium binding and coiled-coil domain (CALCOCO1) like. Proteins found in this family are similar to the coiled-coil transcriptional	GO:0042384|cilium assembly;GO:0060271|cilium morphogenesis;GO:0042462|eye photoreceptor cell development;GO:0000086|G2/M transition of mitotic cell cycle;GO:0030902|hindbrain development;GO:0030916|otic vesicle formation;GO:0090316|positive regulation of intracellular protein transport;GO:0045893|positive regulation of transcription, DNA-templated;GO:0048793|pronephros development;GO:0015031|protein transport;GO:0070201|regulation of establishment of protein localization;	GO:0034451|centriolar satellite;GO:0005814|centriole;GO:0005813|centrosome;GO:0036064|ciliary basal body;GO:0035869|ciliary transition	.	K16533	CEP290, NPHP6; centrosomal protein CEP290
LOC138986371	891.5371	-0.38042	0.131619668	-2.89027	0.0038491	0.02751	Not_Sig	no	gi|741983996|ref|XP_010820556.1|	2937	0	PREDICTED: ATP-dependent RNA helicase DDX3X-like isoform X2 [Bos taurus]	gi|983011902|ref|XM_015470631.1|	4331	0	PREDICTED: Bos taurus ATP-dependent RNA helicase DDX3X-like (LOC107131212), transcript variant X2, mRNA	O00571	1036	0	ATP-dependent RNA helicase DDX3X	.	.	.	.	pfam00270	DEAD	DEAD/DEAH box helicase. Members of this family include the DEAD and DEAH box helicases. Helicases are involved in	GO:0071243|cellular response to arsenic-containing substance;GO:0071470|cellular response to osmotic stress;GO:0007059|chromosome segregation;GO:0008625|extrinsic apoptotic signaling pathway via death domain receptors;GO:0045087|innate immune response;GO:0035556|intracellular signal transduction;GO:0097193|intrinsic apoptotic signaling pathway;GO:0042256|mature ribosome assembly;GO:0043066|negative regulation of apoptotic process;GO:0030308|negative regulation of cell growth;GO:0043154|negative	GO:0005913|cell-cell adherens junction;GO:0005737|cytoplasm;GO:0010494|cytoplasmic stress granule;GO:0070062|extracellular	GO:0005524|ATP binding;GO:0004003|ATP-dependent DNA helicase	K11594	DDX3X, bel; ATP-dependent RNA helicase DDX3X [EC:3.6.4.13]
INTS15	265.6987	0.54598	0.188920458	2.889998	0.0038524	0.02751	Not_Sig	no	gi|555982154|ref|XP_005902985.1|	2067	0	PREDICTED: uncharacterized protein C7orf26 homolog isoform X1 [Bos mutus]	gi|942076333|ref|XM_005902923.2|	1953	0	PREDICTED: Bos mutus chromosome unknown open reading frame, human C7orf26 (LOC102266491), transcript variant X1, mRNA	Q96N11	752	0	Uncharacterized protein C7orf26	.	.	.	.	pfam14964	DUF4507	Domain of unknown function (DUF4507). This family of proteins is found in eukaryotes. Proteins in this family are typically between	.	.	.	.	.
GRAMD1A	420.137	0.408212	0.14125891	2.889817	0.0038547	0.02751	Not_Sig	no	gi|440905811|gb|ELR56144.1|	2930	0	GRAM domain-containing protein 1A [Bos mutus]	gi|965809585|ref|XM_012120413.2|	2884	0	PREDICTED: Ovis aries musimon GRAM domain containing 1A (GRAMD1A), transcript variant X11, mRNA	Q96CP6	1089	0	GRAM domain-containing protein 1A	.	.	.	.	pfam02893	GRAM	GRAM domain. The GRAM domain is found in in glucosyltransferases, myotubularins and other putative membrane-associated proteins.	.	GO:0016021|integral component of membrane;	.	.	.
EDIL3	311.6688	-0.45154	0.156254205	-2.88981	0.0038548	0.02751	Not_Sig	no	gi|358413303|ref|XP_618255.4|	2605	0	PREDICTED: EGF-like repeat and discoidin I-like domain-containing protein 3 isoform X1 [Bos taurus]	gi|982985732|ref|XM_618255.8|	4746	0	PREDICTED: Bos taurus EGF like repeats and discoidin domains 3 (EDIL3), transcript variant X1, mRNA	O43854	981	0	EGF-like repeat and discoidin I-like domain-containing protein 3	.	.	.	.	pfam00754	F5_F8_type_C	F5/8 type C domain. This domain is also known as the discoidin (DS) domain family.	GO:0007155|cell adhesion;GO:0007275|multicellular organismal development;GO:0010811|positive regulation of cell-substrate adhesion;	GO:0070062|extracellular exosome;GO:0031012|extracellular matrix;GO:1903561|extracellular vesicle;	GO:0005509|calcium ion binding;GO:0005178|integrin binding;	.	.
BCL3	208.2949	0.654907	0.22669434	2.888941	0.0038654	0.027556	UP	yes	gi|329664582|ref|NP_001192922.1|	1699	0	B-cell lymphoma 3 protein [Bos taurus]	gi|965825325|ref|XM_012128205.2|	1815	0	PREDICTED: Ovis aries musimon B-cell CLL/lymphoma 3 (BCL3), transcript variant X2, mRNA	P20749	600	0	B-cell lymphoma 3 protein	all2748	79.7	5.41e-15	COG0666	pfam12796	Ank_2	Ankyrin repeats (3 copies).	GO:0019730|antimicrobial humoral response;GO:0006974|cellular response to DNA damage stimulus;GO:0042742|defense response to bacterium;GO:0042832|defense response to protozoan;GO:0030330|DNA damage response, signal transduction by p53 class mediator;GO:0030198|extracellular matrix organization;GO:0002268|follicular dendritic cell differentiation;GO:0002467|germinal center formation;GO:0002455|humoral immune response mediated by circulating immunoglobulin;GO:0007249|I-kappaB kinase/NF-kappaB	GO:0032996|Bcl3-Bcl10 complex;GO:0033257|Bcl3/NF-kappaB2 complex;GO:0005737|cytoplasm;GO:0045171|intercellular	GO:0003677|DNA binding;GO:0030674|protein binding, bridging;GO:000370	K09258	BCL3; B-cell CLL/lymphoma protein 3
MAML2	645.6782	0.338716	0.117248523	2.888871	0.0038663	0.027556	Not_Sig	no	gi|296480332|tpg|DAA22447.1|	2136	0	TPA: mastermind-like protein 2 [Bos taurus]	gi|594052643|ref|XM_006050571.1|	7192	0	PREDICTED: Bubalus bubalis mastermind-like 2 (Drosophila) (MAML2), mRNA	A5D7F6	827	0	Mastermind-like protein 2	.	.	.	.	pfam09596	MamL-1	MamL-1 domain. The MamL-1 domain is a polypeptide of up to 70 residues, numbers 15-67 of which adopt an elongated kinked	GO:0007221|positive regulation of transcription of Notch receptor target;GO:0006351|transcription, DNA-templated;	GO:0016607|nuclear speck;	GO:0003713|transcription coactivator activity;	K06061	MAML; mastermind
UBL5	764.0442	-0.43332	0.15000524	-2.8887	0.0038684	0.027556	Not_Sig	no	gi|593715849|ref|XP_007104382.1|	439	1e-50	PREDICTED: ubiquitin-like protein 5 isoform X1 [Physeter catodon]	gi|942100717|ref|XM_005910858.2|	532	0	PREDICTED: Bos mutus ubiquitin-like 5 (UBL5), transcript variant X2, mRNA	Q791B0	151	9e-46	Ubiquitin-like protein 5	.	.	.	.	pfam00240	ubiquitin	Ubiquitin family. This family contains a number of ubiquitin-like proteins: SUMO (smt3 homologue) Nedd8, Elongin B, Rub1, and	.	GO:0005737|cytoplasm;	.	.	.
SRSF10	1550.47	-0.39688	0.137469622	-2.88705	0.0038887	0.027684	Not_Sig	no	gi|829976393|ref|XP_012606119.1|	432	9e-45	PREDICTED: serine/arginine-rich splicing factor 10 isoform X3 [Microcebus murinus]	gi|982915643|ref|XM_005203271.3|	2627	0	PREDICTED: Bos taurus serine/arginine-rich splicing factor 10 (SRSF10), transcript variant X1, mRNA	Q9R0U0	139	1e-34	Serine/arginine-rich splicing factor 10	.	.	.	.	pfam13893	RRM_5	RNA recognition motif. (a.k.a. RRM, RBD, or RNP domain). The RRM motif is probably diagnostic of an RNA binding protein. RRMs	GO:0016482|cytoplasmic transport;GO:0006406|mRNA export from nucleus;GO:0006376|mRNA splice site selection;GO:0000398|mRNA splicing, via spliceosome;GO:0048025|negative regulation of mRNA splicing, via spliceosome;GO:0048024|regulation of mRNA splicing, via spliceosome;GO:0006355|regulation of transcription, DNA-templated;GO:0000375|RNA splicing, via transesterification reactions;	GO:0043679|axon terminus;GO:0005829|cytosol;GO:0030425|dendrite;GO:0043025|neuronal cell body;GO:0016607|nuclear	GO:0000166|nucleotide binding;GO:0044822|poly(A) RNA	K12900	FUSIP1; FUS-interacting serine-arginine-rich protein 1
SERPING1	576.2213	0.434342	0.150521598	2.885579	0.0039069	0.027781	Not_Sig	no	gi|440909830|gb|ELR59697.1|	2354	0	Factor XIIa inhibitor, partial [Bos mutus]	gi|942041874|ref|XM_005891140.2|	1750	0	PREDICTED: Bos mutus serpin peptidase inhibitor, clade G (C1 inhibitor), member 1 (SERPING1), mRNA	P50448	887	0	Factor XIIa inhibitor	MA3388	92.4	4.07e-19	COG4826	pfam00079	Serpin	Serpin (serine protease inhibitor). Structure is a multi-domain fold containing a bundle of helices and a beta sandwich.	GO:0007596|blood coagulation;GO:0042730|fibrinolysis;	GO:0005615|extracellular space;	GO:0004867|serine-type endopeptidase inhibitor activity;	K04001	SERPING1, C1INH; C1 inhibitor
TFIP11	601.8351	0.374536	0.129796861	2.885553	0.0039073	0.027781	Not_Sig	no	gi|594063185|ref|XP_006055630.1|	3964	0	PREDICTED: tuftelin-interacting protein 11 isoform X1 [Bubalus bubalis]	gi|165905562|ref|NM_001046030.2|	2580	0	Bos taurus tuftelin interacting protein 11 (TFIP11), mRNA	Q29RR5	1531	0	Tuftelin-interacting protein 11	.	.	.	.	pfam07842	GCFC	GC-rich sequence DNA-binding factor-like protein. Sequences found in this family are similar to a region of a human GC-rich	GO:0031214|biomineral tissue development;GO:0006355|regulation of transcription, DNA-templated;GO:0000390|spliceosomal complex disassembly;	GO:0005737|cytoplasm;GO:0005681|spliceosomal complex;GO:0071008|U2-type post-mRNA release spliceosomal complex;	GO:0003677|DNA binding;	K13103	TFIP11; tuftelin-interacting protein 11
ASB7	309.0945	-0.38469	0.13336583	-2.88446	0.0039208	0.027847	Not_Sig	no	gi|637346758|ref|XP_003226537.2|	1717	0	PREDICTED: ankyrin repeat and SOCS box protein 7 isoform X1 [Anolis carolinensis]	gi|742149971|ref|XM_010845813.1|	6065	0	PREDICTED: Bison bison bison ankyrin repeat and SOCS box containing 7 (ASB7), transcript variant X1, mRNA	Q9H672	662	0	Ankyrin repeat and SOCS box protein 7	all2748	95.1	2.69e-19	COG0666	pfam12796	Ank_2	Ankyrin repeats (3 copies).	GO:0035556|intracellular signal transduction;GO:0016567|protein ubiquitination;	GO:0005622|intracellular;	.	K10329	ASB7; ankyrin repeat and SOCS box protein 7
COG1	666.2919	0.385558	0.133669076	2.884424	0.0039213	0.027847	Not_Sig	no	gi|528997281|ref|XP_005221276.1|	4618	0	PREDICTED: conserved oligomeric Golgi complex subunit 1 isoform X1 [Bos taurus]	gi|982958673|ref|XM_005221219.3|	2996	0	PREDICTED: Bos taurus component of oligomeric golgi complex 1 (COG1), transcript variant X1, mRNA	Q8WTW3	1514	0	Conserved oligomeric Golgi complex subunit 1	.	.	.	.	pfam08700	Vps51	Vps51/Vps67. This family includes a presumed domain found in a number of components of vesicular transport. The VFT	GO:0006888|ER to Golgi vesicle-mediated transport;GO:0007030|Golgi organization;GO:0006891|intra-Golgi vesicle-mediated transport;GO:0015031|protein transport;GO:0000301|retrograde transport, vesicle recycling within Golgi;	GO:0005794|Golgi apparatus;GO:0000139|Golgi membrane;GO:0017119|Golgi transport complex;GO:0032588|trans-Golgi network	.	.	.
ARPC4	1997.019	-0.40991	0.142125266	-2.88416	0.0039246	0.027853	Not_Sig	no	gi|675730222|ref|XP_003734934.2|	1031	3e-135	PREDICTED: actin-related protein 2/3 complex subunit 4 isoform X1 [Callithrix jacchus]	gi|742133628|ref|XM_010842093.1|	1474	0	PREDICTED: Bison bison bison actin related protein 2/3 complex, subunit 4, 20kDa (ARPC4), mRNA	P59999	342	5e-114	Actin-related protein 2/3 complex subunit 4	.	.	.	.	pfam05856	ARPC4	ARP2/3 complex 20 kDa subunit (ARPC4). This family consists of several eukaryotic ARP2/3 complex 20 kDa subunit (P20-ARC)	GO:0030041|actin filament polymerization;GO:0034314|Arp2/3 complex-mediated actin nucleation;	GO:0005885|Arp2/3 protein complex;GO:0042995|cell projection;GO:0005737|cytoplasm;GO:0070062|extracellular exosome;	GO:0019899|enzyme binding;GO:0005200|structural	K05755	ARPC4; actin related protein 2/3 complex, subunit 4
TCN2	620.9128	0.452895	0.157065062	2.883487	0.003933	0.027895	Not_Sig	no	gi|99028973|ref|NP_776620.2|	2079	0	transcobalamin-2 precursor [Bos taurus]	gi|942102976|ref|XM_005911482.2|	2206	0	PREDICTED: Bos mutus transcobalamin II (TCN2), transcript variant X1, mRNA	Q9XSC9	781	0	Transcobalamin-2	.	.	.	.	pfam01122	Cobalamin_bind	Eukaryotic cobalamin-binding protein.	GO:0015889|cobalamin transport;GO:0006824|cobalt ion transport;	GO:0070062|extracellular exosome;	GO:0031419|cobalamin binding;GO:0046872|metal ion binding;	K14619	TCN2; transcobalamin-2
FRMD4A	369.8327	0.429721	0.149117461	2.881762	0.0039546	0.028031	Not_Sig	no	gi|594085065|ref|XP_006066076.1|	4707	0	PREDICTED: FERM domain-containing protein 4A isoform X1 [Bubalus bubalis]	gi|982942220|ref|XM_010811107.2|	3261	0	PREDICTED: Bos taurus FERM domain containing 4A (FRMD4A), transcript variant X1, mRNA	Q8BIE6	1644	0	FERM domain-containing protein 4A	.	.	.	.	pfam11819	DUF3338	Domain of unknown function (DUF3338). This family of proteins are functionally uncharacterized. This family is found in eukaryotes.	GO:0090162|establishment of epithelial cell polarity;	GO:0005923|bicellular tight junction;GO:0005737|cytoplasm;GO:0005856|cytoskeleton;	GO:0030674|protein binding, bridging;	.	.
RPS11	4066.185	-0.31309	0.108822171	-2.87709	0.0040136	0.028432	Not_Sig	no	gi|744603787|ref|XP_010991488.1|	878	2e-117	PREDICTED: 40S ribosomal protein S11 [Camelus dromedarius]	gi|742163356|ref|XM_010849620.1|	667	0	PREDICTED: Bison bison bison ribosomal protein S11 (RPS11), mRNA	P62282	325	3e-112	40S ribosomal protein S11	YDR025w	212	6.17e-70	COG0186	pfam00366	Ribosomal_S17	Ribosomal protein S17.	GO:0001649|osteoblast differentiation;GO:0006412|translation;	GO:0022627|cytosolic small ribosomal subunit;GO:0070062|extracellular exosome;GO:0005925|focal adhesion;GO:0016020|membrane;GO:0005	GO:0044822|poly(A) RNA binding;GO:0019843|rRNA	K02949	RP-S11e, RPS11; small subunit ribosomal protein S11e
SEC62	1785.838	-0.30585	0.106336007	-2.87625	0.0040243	0.028484	Not_Sig	no	gi|859809418|ref|XP_012913758.1|	819	7e-95	PREDICTED: translocation protein SEC62 isoform X1 [Mustela putorius furo]	gi|942092703|ref|XM_005908153.2|	3724	0	PREDICTED: Bos mutus SEC62 homolog, preprotein translocation factor (SEC62), transcript variant X1, mRNA	Q99442	316	4e-95	Translocation protein SEC62	SPAC17G6.09	88.2	3.82e-18	COG5232	pfam03839	Sec62	Translocation protein Sec62.	GO:0006613|cotranslational protein targeting to membrane;GO:0036498|IRE1-mediated unfolded protein response;GO:0006620|posttranslational protein targeting to membrane;	GO:0016235|aggresome;GO:0005829|cytosol;GO:0005783|endoplasmic reticulum;GO:0005789|endoplasmic reticulum membrane;GO:0016021|integral	GO:0008565|protein transporter activity;GO:0004872|receptor activity;	K12275	SEC62; translocation protein SEC62
PFDN6	271.4231	-0.4373	0.152043956	-2.87612	0.004026	0.028484	Not_Sig	no	gi|545838985|ref|XP_005665938.1|	633	4e-80	PREDICTED: prefoldin subunit 6 isoform X1 [Sus scrofa]	gi|982962976|ref|XM_005223224.3|	648	0	PREDICTED: Bos taurus prefoldin subunit 6 (PFDN6), transcript variant X2, mRNA	Q17Q89	230	2e-75	Prefoldin subunit 6	YLR200w	79.7	1.36e-18	COG1382	pfam01920	Prefoldin_2	Prefoldin subunit. This family includes prefoldin subunits that are not detected by pfam02996.	GO:0051086|chaperone mediated protein folding independent of cofactor;GO:0051131|chaperone-mediated protein complex assembly;GO:0006457|protein folding;	GO:0005737|cytoplasm;GO:0005829|cytosol;GO:0016272|prefoldin complex;	GO:0051087|chaperone binding;	K04798	pfdB, PFDN6; prefoldin beta subunit
NRIP1	1246.901	-0.29758	0.103480666	-2.87574	0.0040308	0.028484	Not_Sig	no	gi|742144590|ref|XP_010843431.1|	5975	0	PREDICTED: nuclear receptor-interacting protein 1 [Bison bison bison]	gi|742144617|ref|XM_010845135.1|	7442	0	PREDICTED: Bison bison bison nuclear receptor interacting protein 1 (NRIP1), transcript variant X7, mRNA	P48552	2015	0	Nuclear receptor-interacting protein 1	.	.	.	.	pfam08372	PRT_C	Plant phosphoribosyltransferase C-terminal. This domain is found at the C-terminus of phosphoribosyltransferases and	GO:0030521|androgen receptor signaling pathway;GO:0071392|cellular response to estradiol stimulus;GO:0032922|circadian regulation of gene expression;GO:0007623|circadian rhythm;GO:0019915|lipid storage;GO:0000122|negative regulation of transcription from RNA polymerase II promoter;GO:0001543|ovarian follicle rupture;GO:0045944|positive regulation of transcription from RNA polymerase II promoter;GO:0045893|positive regulation of transcription, DNA-templated;GO:0006351|transcription, DNA-templated;	GO:0030054|cell junction;GO:0000118|histone deacetylase complex;GO:0000790|nuclear chromatin;GO:0016607|nuclear	GO:0050681|androgen receptor binding;GO:0001046|core promoter	K17965	NRIP1; nuclear receptor-interacting protein 1
SETD1B	339.3887	0.495486	0.172308304	2.87558	0.0040329	0.028484	Not_Sig	no	gi|803210447|ref|XP_011965646.1|	2359	0	PREDICTED: histone-lysine N-methyltransferase SETD1B isoform X2 [Ovis aries musimon]	gi|965789189|ref|XM_012110256.2|	7798	0	PREDICTED: Ovis aries musimon SET domain containing 1B (SETD1B), transcript variant X2, mRNA	Q9UPS6	877	0	Histone-lysine N-methyltransferase SETD1B	SPCC306.04c	189	7.32e-48	COG2940	pfam11764	N-SET	COMPASS (Complex proteins associated with Set1p) component N. The n-SET or N-SET domain is a component of the COMPASS	GO:0051568|histone H3-K4 methylation;GO:0006355|regulation of transcription, DNA-templated;GO:0006351|transcription, DNA-templated;	GO:0005694|chromosome;GO:0005737|cytoplasm;GO:0035097|histone methyltransferase complex;GO:0016607|nuclear	GO:0018024|histone-lysine N-methyltransferase activity;GO:0000166	K11422	SETD1, SET1; histone-lysine N-methyltransferase SETD1 [EC:2.1.1.43]
RPA3	362.7773	-0.49406	0.171814074	-2.87554	0.0040333	0.028484	Not_Sig	no	gi|555973085|ref|XP_005898542.1|	640	1e-81	PREDICTED: replication protein A 14 kDa subunit isoform X1 [Bos mutus]	gi|942061763|ref|XM_005898480.2|	733	0	PREDICTED: Bos mutus replication protein A3, 14kDa (RPA3), transcript variant X1, mRNA	P35244	221	1e-71	Replication protein A 14 kDa subunit	.	.	.	.	pfam08661	Rep_fac-A_3	Replication factor A protein 3. Replication factor A is involved in eukaryotic DNA replication, recombination and repair.	GO:0006284|base-excision repair;GO:0042769|DNA damage response, detection of DNA damage;GO:0006260|DNA replication;GO:0000724|double-strand break repair via homologous recombination;GO:0070987|error-free translesion synthesis;GO:0042276|error-prone translesion synthesis;GO:0000082|G1/S transition of mitotic cell cycle;GO:0036297|interstrand cross-link repair;GO:0006298|mismatch repair;GO:0006289|nucleotide-excision repair;GO:0006297|nucleotide-excision repair, DNA gap	GO:0005662|DNA replication factor A complex;GO:0005654|nucleoplasm;	GO:0003684|damaged DNA binding;GO:0003697|single-stranded	K10740	RPA3; replication factor A3
HSBP1	1078.028	-0.37514	0.130513773	-2.87435	0.0040485	0.028574	Not_Sig	no	gi|795458397|ref|XP_011765639.1|	383	1e-40	PREDICTED: heat shock factor-binding protein 1 [Macaca nemestrina]	gi|594042539|ref|XM_006045741.1|	1708	0	PREDICTED: Bubalus bubalis heat shock factor binding protein 1 (HSBP1), mRNA	Q5RDI2	149	2e-41	Heat shock factor-binding protein 1	.	.	.	.	pfam06825	HSBP1	Heat shock factor binding protein 1. Heat shock factor binding protein 1 (HSBP1) appears to be a negative regulator of the heat	.	GO:0005634|nucleus;	GO:0003714|transcription corepressor activity;	.	.
PTPRF	5136.919	0.320711	0.111595371	2.873874	0.0040547	0.028599	Not_Sig	no	gi|594040047|ref|XP_006044596.1|	9888	0	PREDICTED: receptor-type tyrosine-protein phosphatase F isoform X1 [Bubalus bubalis]	gi|155371888|ref|NM_001101079.1|	7376	0	Bos taurus protein tyrosine phosphatase, receptor type F (PTPRF), mRNA	A7MBJ4	3785	0	Receptor-type tyrosine-protein phosphatase F	SPAC11E3.09	141	6.39e-36	COG5599	pfam00102	Y_phosphatase	Protein-tyrosine phosphatase.	GO:0007155|cell adhesion;GO:0016477|cell migration;GO:1900121|negative regulation of receptor binding;GO:0031102|neuron projection regeneration;GO:0035335|peptidyl-tyrosine dephosphorylation;GO:0048679|regulation of axon regeneration;	GO:0070062|extracellular exosome;GO:0016021|integral component of membrane;GO:0043005|neuron projection;GO:0043025|neuronal cell body;	GO:0035373|chondroitin sulfate proteoglycan binding;GO:0008201	K05695	PTPRF, LAR; receptor-type tyrosine-protein phosphatase F
TPX2	1215.371	0.324371	0.112876284	2.873686	0.0040571	0.028599	Not_Sig	no	gi|555951552|ref|XP_005887973.1|	3537	0	PREDICTED: targeting protein for Xklp2 isoform X1 [Bos mutus]	gi|982942965|ref|XM_005214602.3|	6061	0	PREDICTED: Bos taurus TPX2, microtubule-associated (TPX2), transcript variant X1, mRNA	A6H6Z7	1267	0	Targeting protein for Xklp2	.	.	.	.	pfam12214	TPX2_importin	Cell cycle regulated microtubule associated protein. This domain is found in eukaryotes. This domain is typically between 127 to 182	GO:0032147|activation of protein kinase activity;GO:0006915|apoptotic process;GO:0051301|cell division;GO:0090307|mitotic spindle assembly;GO:0060236|regulation of mitotic spindle organization;	GO:0005737|cytoplasm;GO:0005874|microtubule;GO:0005634|nucleus;GO:0000922|spindle pole;	GO:0061676|importin-alpha family protein binding;	K16812	TPX2; targeting protein for Xklp2
LOC102285922	3064.505	0.302119	0.10514986	2.873227	0.004063	0.028619	Not_Sig	no	gi|741921965|ref|XP_010801987.1|	2928	0	PREDICTED: elongation of very long chain fatty acids protein 1 isoform X1 [Bos taurus]	gi|982920161|ref|XM_010803685.2|	2307	0	PREDICTED: Bos taurus ELOVL fatty acid elongase 1 (ELOVL1), transcript variant X1, mRNA	Q9BW60	548	0	Elongation of very long chain fatty acids protein 1	.	.	.	.	pfam10232	Med8	Mediator of RNA polymerase II transcription complex subunit 8. Arc32, or Med8, is one of the subunits of the Mediator complex	GO:0036109|alpha-linolenic acid metabolic process;GO:0034625|fatty acid elongation, monounsaturated fatty acid;GO:0034626|fatty acid elongation, polyunsaturated fatty acid;GO:0019367|fatty acid elongation, saturated fatty acid;GO:0043651|linoleic acid metabolic process;GO:0035338|long-chain fatty-acyl-CoA biosynthetic process;GO:0030148|sphingolipid biosynthetic process;GO:0006636|unsaturated fatty acid biosynthetic process;GO:0042761|very long-chain fatty acid biosynthetic process;	GO:0005783|endoplasmic reticulum;GO:0005789|endoplasmic reticulum membrane;GO:0030176|integral component of endoplasmic reticulum	GO:0009922|fatty acid elongase activity;	K10247	ELOVL1; elongation of very long chain fatty acids protein 1 [EC:2.3.1.199]
FAM53B	408.4898	0.421551	0.146724598	2.873079	0.0040649	0.028619	Not_Sig	no	gi|741903267|ref|XP_010799424.1|	1705	0	PREDICTED: protein FAM53B isoform X1 [Bos taurus]	gi|742199318|ref|XM_010859171.1|	3968	0	PREDICTED: Bison bison bison family with sequence similarity 53, member B (FAM53B), transcript variant X3, mRNA	Q14153	510	7e-164	Protein FAM53B	.	.	.	.	pfam15242	FAM53	Family of FAM53. The FAM53 protein family refers to a family of proteins, which bind to a transcriptional regulator that	.	.	.	.	.
CTSA	1581.082	0.409522	0.142570073	2.872427	0.0040733	0.028643	Not_Sig	no	gi|942059193|ref|XP_014334240.1|	2535	0	PREDICTED: lysosomal protective protein [Bos mutus]	gi|742097448|ref|XM_010835040.1|	1859	0	PREDICTED: Bison bison bison cathepsin A (CTSA), mRNA	Q3MI05	968	0	Lysosomal protective protein	YBR139w	185	1.25e-50	COG2939	pfam00450	Peptidase_S10	Serine carboxypeptidase.	GO:0051603|proteolysis involved in cellular protein catabolic process;	GO:0005764|lysosome;	GO:0004185|serine-type carboxypeptidase activity;	K13289	CTSA; cathepsin A (carboxypeptidase C) [EC:3.4.16.5]
RPS6KB2	391.7956	0.405639	0.141218362	2.872426	0.0040733	0.028643	Not_Sig	no	gi|965994683|ref|XP_011957761.2|	2103	0	PREDICTED: ribosomal protein S6 kinase beta-2 isoform X1 [Ovis aries]	gi|742109135|ref|XM_010837704.1|	1717	0	PREDICTED: Bison bison bison ribosomal protein S6 kinase, 70kDa, polypeptide 2 (RPS6KB2), mRNA	Q9UBS0	776	0	Ribosomal protein S6 kinase beta-2	.	.	.	.	pfam00069	Pkinase	Protein kinase domain.	GO:0035556|intracellular signal transduction;GO:0045948|positive regulation of translational initiation;GO:0043491|protein kinase B signaling;GO:0007165|signal transduction;GO:0006412|translation;	GO:0005737|cytoplasm;GO:0005654|nucleoplasm;GO:0005634|nucleus;	GO:0005524|ATP binding;GO:0042277|peptide binding;GO:0004672	K04688	RPS6KB; p70 ribosomal S6 kinase [EC:2.7.11.1]
ENPP1	175.8126	-0.48851	0.170099986	-2.87189	0.0040802	0.028674	Not_Sig	no	gi|329664290|ref|NP_001193141.1|	4486	0	ectonucleotide pyrophosphatase/phosphodiesterase family member 1 [Bos taurus]	gi|982935234|ref|XM_010808573.2|	4139	0	PREDICTED: Bos taurus ectonucleotide pyrophosphatase/phosphodiesterase 1 (ENPP1), transcript variant X2, mRNA	P22413	1535	0	Ectonucleotide pyrophosphatase/phosphodiesterase family member 1	SPBC725.05c	229	4.10e-64	COG1524	pfam01663	Phosphodiest	Type I phosphodiesterase / nucleotide pyrophosphatase. This family consists of phosphodiesterases, including	GO:0050427|3'-phosphoadenosine 5'-phosphosulfate metabolic process;GO:0031214|biomineral tissue development;GO:0030643|cellular phosphate ion homeostasis;GO:0032869|cellular response to insulin stimulus;GO:0006091|generation of precursor metabolites and energy;GO:0006955|immune response;GO:0030505|inorganic diphosphate transport;GO:0030308|negative regulation of cell growth;GO:0045599|negative regulation of fat cell differentiation;GO:0046325|negative regulation of glucose import;GO:0045719|negative	GO:0016323|basolateral plasma membrane;GO:0009986|cell surface;GO:0005615|extracellular space;GO:0016021|integral component of	GO:0050656|3'-phosphoadenosine 5'-phosphosulfate binding;GO:0005524	K01513	ENPP1_3; ectonucleotide pyrophosphatase/phosphodiesterase
GLI2	296.5263	0.468481	0.163190622	2.870759	0.0040949	0.028759	Not_Sig	no	gi|982913961|ref|XP_015313566.1|	6921	0	PREDICTED: zinc finger protein GLI2 isoform X1 [Bos taurus]	gi|982913960|ref|XM_015458080.1|	6368	0	PREDICTED: Bos taurus GLI family zinc finger 2 (GLI2), transcript variant X1, mRNA	P10070	2229	0	Zinc finger protein GLI2	.	.	.	.	pfam09770	PAT1	Topoisomerase II-associated protein PAT1. Members of this family are necessary for accurate chromosome transmission during	GO:0009952|anterior/posterior pattern specification;GO:0007411|axon guidance;GO:0048754|branching morphogenesis of an epithelial tube;GO:0008283|cell proliferation;GO:0071407|cellular response to organic cyclic compound;GO:0098586|cellular response to virus;GO:0021696|cerebellar cortex morphogenesis;GO:0002062|chondrocyte differentiation;GO:0090103|cochlea morphogenesis;GO:0048589|developmental growth;GO:0048566|embryonic digestive tract development;GO:0042733|embryonic digit	GO:0005930|axoneme;GO:0097546|ciliary base;GO:0097542|ciliary tip;GO:0005829|cytosol;GO:0016020|membrane;GO:0031512|motile primary	GO:0003682|chromatin binding;GO:0000978|RNA polymerase II	K16798	GLI2; zinc finger protein GLI2
IQSEC1	248.3609	0.493643	0.172081609	2.868656	0.0041222	0.028933	Not_Sig	no	gi|332205875|ref|NP_001193743.1|	4380	0	IQ motif and SEC7 domain-containing protein 1 [Bos taurus]	gi|359751477|ref|NM_001206814.2|	3363	0	Bos taurus IQ motif and Sec7 domain 1 (IQSEC1), mRNA	Q8R0S2	1433	0	IQ motif and SEC7 domain-containing protein 1	YDR170c	137	8.77e-32	COG5307	pfam01369	Sec7	Sec7 domain. The Sec7 domain is a guanine-nucleotide-exchange-factor (GEF) for the pfam00025 family.	GO:0030036|actin cytoskeleton organization;GO:0032012|regulation of ARF protein signal transduction;	GO:0005737|cytoplasm;GO:0016020|membrane;GO:0005634|nucleus;	GO:0005086|ARF guanyl-nucleotide exchange factor activity;GO:0008289	K12495	IQSEC; IQ motif and SEC7 domain-containing protein

RPS18	4070.44	-0.3721	0.129729345	-2.8683	0.0041269	0.028948	Not_Sig	no	gi|602653259|ref|XP_007445325.1|	706	2e-91	PREDICTED: 40S ribosomal protein S18-like, partial [Python bivittatus]	gi|742159676|ref|XM_010848597.1|	566	0	PREDICTED: Bison bison bison ribosomal protein S18 (RPS18), mRNA	P62271	270	8e-91	40S ribosomal protein S18	SPCC1259.01c	204	6.48e-67	COG0099	pfam00416	Ribosomal_S13	Ribosomal protein S13/S18. This family includes ribosomal protein S13 from prokaryotes and S18 from eukaryotes.	GO:0042254|ribosome biogenesis;GO:0006412|translation;	GO:0022627|cytosolic small ribosomal subunit;GO:0070062|extracellular exosome;GO:0005925|focal adhesion;GO:0016020|membrane;GO:0005	GO:0044822|poly(A) RNA binding;GO:0019901|protein kinase	K02964	RP-S18e, RPS18; small subunit ribosomal protein S18e
BRAF	352.6474	-0.39066	0.136213418	-2.86798	0.004131	0.028952	Not_Sig	no	gi|926690766|ref|XP_013818995.1|	3546	0	PREDICTED: serine/threonine-protein kinase B-raf isoform X1 [Capra hircus]	gi|982981064|ref|XM_015463145.1|	9260	0	PREDICTED: Bos taurus B-Raf proto-oncogene, serine/threonine kinase (BRAF), transcript variant X1, mRNA	P15056	1318	0	Serine/threonine-protein kinase B-raf	YAR019c	117	2.61e-25	COG0515	pfam00069	Pkinase	Protein kinase domain.	GO:0009887|organ morphogenesis;GO:0043367|CD4-positive, alpha-beta T cell differentiation;GO:0071277|cellular response to calcium ion;GO:0035690|cellular response to drug;GO:0090150|establishment of protein localization to membrane;GO:0060324|face development;GO:0015758|glucose transport;GO:0060323|head morphogenesis;GO:0060291|long-term synaptic potentiation;GO:0000165|MAPK cascade;GO:0002318|myeloid progenitor cell differentiation;GO:0043066|negative regulation of apoptotic	GO:0044297|cell body;GO:0005737|cytoplasm;GO:0005829|cytosol;GO:0043231|intracellular membrane-bounded	GO:0005524|ATP binding;GO:0005509|calcium ion binding;GO:0042802	K04365	BRAF; B-Raf proto-oncogene serine/threonine-protein kinase
CBLN3	89.76469	0.630482	0.219843085	2.867874	0.0041324	0.028952	UP	yes	gi|440905360|gb|ELR55746.1|	658	3e-129	hypothetical protein M91_14620 [Bos mutus]	gi|742234369|ref|XM_010831366.1|	3970	0	PREDICTED: Bison bison bison cerebellin 3 precursor (CBLN3), mRNA	Q17QF9	289	8e-87	Cerebellin-3	.	.	.	.	pfam00386	C1q	C1q domain. C1q is a subunit of the C1 enzyme complex that activates the serum complement system.	.	GO:0030054|cell junction;GO:0005783|endoplasmic reticulum;GO:0005576|extracellular region;GO:0005794|Golgi	.	.	.
SNRPF	277.9528	-0.48126	0.167846694	-2.86725	0.0041405	0.028991	Not_Sig	no	gi|594053720|ref|XP_006051143.1|	637	5e-82	PREDICTED: small nuclear ribonucleoprotein F [Bubalus bubalis]	gi|742102731|ref|XM_010836291.1|	468	0	PREDICTED: Bison bison bison small nuclear ribonucleoprotein polypeptide F (SNRPF), mRNA	P62321	156	5e-48	Small nuclear ribonucleoprotein F	.	.	.	.	pfam01423	LSM	LSM domain. The LSM domain contains Sm proteins as well as other related LSM (Like Sm) proteins. The U1, U2, U4/U6, and	GO:0000387|spliceosomal snRNP assembly;	GO:0005829|cytosol;GO:0034709|methylosome;GO:0034715|pICln-Sm protein complex;GO:0005732|small nucleolar ribonucleoprotein	GO:0003723|RNA binding;	K11098	SNRPF, SMF; small nuclear ribonucleoprotein F
ECSIT	307.5893	0.445624	0.155445945	2.866745	0.0041472	0.02902	Not_Sig	no	gi|555957387|ref|XP_005890826.1|	1962	0	PREDICTED: evolutionarily conserved signaling intermediate in Toll pathway, mitochondrial isoform X1 [Bos mutus]	gi|942041278|ref|XM_005890764.2|	1513	0	PREDICTED: Bos mutus ECSIT signalling integrator (ECSIT), transcript variant X1, mRNA	Q3SX05	756	0	Evolutionarily conserved signaling intermediate in Toll pathway, mitochondrial	.	.	.	.	pfam06239	ECSIT	Evolutionarily conserved signalling intermediate in Toll pathway. Activation of NF-kappaB as a consequence of signaling through	GO:0045087|innate immune response;GO:0051341|regulation of oxidoreductase activity;	GO:0005737|cytoplasm;GO:0005739|mitochondrion;GO:0005654|nucleoplasm;GO:0005634|nucleus;	GO:0016651|oxidoreductase activity, acting on NAD(P)H;	K04405	ECSIT; evolutionarily conserved signaling intermediate in Toll pathway
TAB1	352.9345	0.404517	0.141132255	2.866224	0.004154	0.029047	Not_Sig	no	gi|555975055|ref|XP_005899512.1|	2470	0	PREDICTED: TGF-beta-activated kinase 1 and MAP3K7-binding protein 1 [Bos mutus]	gi|942065407|ref|XM_005899450.2|	3289	0	PREDICTED: Bos mutus TGF-beta activated kinase 1/MAP3K7 binding protein 1 (TAB1), mRNA	Q15750	939	0	TGF-beta-activated kinase 1 and MAP3K7-binding protein 1	.	.	.	.	pfam00481	PP2C	Protein phosphatase 2C. Protein phosphatase 2C is a Mn++ or Mg++ dependent protein serine/threonine phosphatase.	GO:0000187|activation of MAPK activity;GO:0000185|activation of MAPKKK activity;GO:0035904|aorta development;GO:0003279|cardiac septum development;GO:0060976|coronary vasculature development;GO:0038095|Fc-epsilon receptor signaling pathway;GO:0003007|heart morphogenesis;GO:0007249|I-kappaB kinase/NF-kappaB signaling;GO:0001701|in utero embryonic development;GO:0007254|JNK cascade;GO:0030324|lung development;GO:0002755|MyD88-dependent toll-like receptor signaling pathway;GO:0070423|nucleotide-binding	GO:0005737|cytoplasm;GO:0005829|cytosol;GO:0010008|endosome membrane;GO:0005654|nucleoplasm;GO:0043234|protein complex;	GO:0008047|enzyme activator activity;GO:0019209|kinase activator	K04403	MAP3K7IP1, TAB1; TAK1-binding protein 1
PVR	3890.951	0.355626	0.124081428	2.866068	0.004156	0.029047	Not_Sig	no	gi|942068074|ref|XP_014335114.1|	2005	0	PREDICTED: poliovirus receptor [Bos mutus]	gi|982953932|ref|XM_005219427.3|	3063	0	PREDICTED: Bos taurus poliovirus receptor (LOC526865), transcript variant X1, mRNA	P32506	371	3e-116	Poliovirus receptor homolog	.	.	.	.	pfam08205	C2-set_2	CD80-like C2-set immunoglobulin domain. These domains belong to the immunoglobulin superfamily.	GO:0007155|cell adhesion;GO:0016032|viral process;	GO:0005576|extracellular region;GO:0016021|integral component of membrane;GO:0005886|plasma membrane;	.	.	.
PFDN4	434.2886	-0.40069	0.139822269	-2.86574	0.0041604	0.029059	Not_Sig	no	gi|84370105|ref|NP_001033629.1|	636	6e-80	prefoldin subunit 4 [Bos taurus]	gi|803250234|ref|XM_012129686.1|	831	0	PREDICTED: Ovis aries musimon prefoldin subunit 4 (PFDN4), transcript variant X4, mRNA	Q2TBR6	249	2e-81	Prefoldin subunit 4	YNL153c	75.9	2.48e-16	COG1382	pfam01920	Prefoldin_2	Prefoldin subunit. This family includes prefoldin subunits that are not detected by pfam02996.	GO:0051086|chaperone mediated protein folding independent of cofactor;GO:0006457|protein folding;	GO:0005737|cytoplasm;GO:0005829|cytosol;GO:0005739|mitochondrion;GO:0005634|nucleus;GO:0016272|prefoldin complex;	GO:0051087|chaperone binding;	K09550	PFDN4; prefoldin subunit 4
DELE1	987.0789	0.340745	0.11896431	2.864258	0.0041799	0.029178	Not_Sig	no	gi|742166284|ref|XP_010848705.1|	2560	0	PREDICTED: death ligand signal enhancer isoform X1 [Bison bison bison]	gi|594078016|ref|XM_006062653.1|	1894	0	PREDICTED: Bubalus bubalis KIAA0141 ortholog (KIAA0141), transcript variant X1, mRNA	Q14154	652	0	Death ligand signal enhancer	STM1760	77.8	3.24e-14	COG0790	pfam08238	Sel1	Sel1 repeat. This short repeat is found in the Sel1 protein. It is related to TPR repeats.	GO:0008625|extrinsic apoptotic signaling pathway via death domain receptors;GO:0043281|regulation of cysteine-type endopeptidase activity involved in apoptotic process;	GO:0005739|mitochondrion;	.	.	.
PGGHG	452.3548	0.354044	0.123643482	2.863422	0.0041909	0.029237	Not_Sig	no	gi|942099220|ref|XP_014338689.1|	3447	0	PREDICTED: LOW QUALITY PROTEIN: acid trehalase-like protein 1 [Bos mutus]	gi|965912411|ref|XM_012172657.2|	3241	0	PREDICTED: Ovis aries musimon ATH1, acid trehalase-like 1 (yeast) (ATHL1), transcript variant X3, mRNA	Q32M88	1126	0	Acid trehalase-like protein 1	all4989	159	7.28e-39	COG1554	pfam03632	Glyco_hydro_65m	Glycosyl hydrolase family 65 central catalytic domain. This family of glycosyl hydrolases contains vacuolar acid trehalase	GO:0005975|carbohydrate metabolic process;	GO:0005618|cell wall;	GO:0016787|hydrolase activity;GO:0016798|hydrolase activity,	.	.
RNF123	679.7703	0.360822	0.126034433	2.862882	0.0041981	0.029252	Not_Sig	no	gi|300796384|ref|NP_001179993.1|	6477	0	E3 ubiquitin-protein ligase RNF123 [Bos taurus]	gi|982961943|ref|XM_005222792.3|	4268	0	PREDICTED: Bos taurus ring finger protein 123 (RNF123), transcript variant X2, mRNA	Q5XPI3	2332	0	E3 ubiquitin-protein ligase RNF123	.	.	.	.	pfam00622	SPRY	SPRY domain. SPRY Domain is named from SPla and the RYanodine Receptor. Domain of unknown function. Distant	GO:0016567|protein ubiquitination;	GO:0005737|cytoplasm;	GO:0016874|ligase activity;GO:0008270|zinc ion binding;	K12169	KPC1, RNF123; Kip1 ubiquitination-promoting complex protein 1
EVL	1860.755	0.417875	0.145965367	2.862839	0.0041986	0.029252	Not_Sig	no	gi|528999841|ref|XP_005222214.1|	1609	0	PREDICTED: ena/VASP-like protein isoform X4 [Bos taurus]	gi|300797265|ref|NM_001192084.1|	1790	0	Bos taurus Enah/Vasp-like (EVL), mRNA	Q9UI08	587	0	Ena/VASP-like protein	.	.	.	.	pfam00568	WH1	WH1 domain. WASp Homology domain 1 (WH1) domain. WASP is the protein that is defective in Wiskott-Aldrich syndrome (WAS).	GO:0007015|actin filament organization;GO:0008154|actin polymerization or depolymerization;GO:0009887|organ morphogenesis;GO:0007411|axon guidance;GO:0007166|cell surface receptor signaling pathway;GO:0010633|negative regulation of epithelial cell migration;GO:1900028|negative regulation of ruffle assembly;GO:0007399|nervous system development;GO:0030838|positive regulation of actin filament polymerization;GO:0051496|positive regulation of stress fiber assembly;GO:0051289|protein	GO:0005737|cytoplasm;GO:0005856|cytoskeleton;GO:0005829|cytosol;GO:0005925|focal adhesion;GO:0030027|lamellipodium;GO:00	GO:0005522|profilin binding;GO:0017124|SH3 domain	.	.
ASXL1	1263.819	0.349452	0.122071432	2.862687	0.0042006	0.029252	Not_Sig	no	gi|742132118|ref|XP_010840022.1|	7184	0	PREDICTED: putative Polycomb group protein ASXL1 isoform X2 [Bison bison bison]	gi|982993578|ref|XM_600364.7|	6410	0	PREDICTED: Bos taurus additional sex combs like 1, transcriptional regulator (ASXL1), transcript variant X1, mRNA	Q8IXJ9	2080	0	Putative Polycomb group protein ASXL1	.	.	.	.	pfam13919	ASXH	Asx homology domain. A conserved alpha helical domain with a characteristic LXXLL motif. The LXXLL motif is detected in	GO:0060348|bone development;GO:0003007|heart morphogenesis;GO:0060430|lung saccule development;GO:0035522|monoubiquitinated histone H2A deubiquitination;GO:0045599|negative regulation of fat cell differentiation;GO:0035359|negative regulation of peroxisome proliferator activated receptor signaling pathway;GO:0048387|negative regulation of retinoic acid receptor signaling pathway;GO:0000122|negative regulation of transcription from RNA polymerase II promoter;GO:0048386|positive regulation of retinoic acid	GO:0070062|extracellular exosome;GO:0000790|nuclear chromatin;GO:0035517|PR-DUB complex;	GO:0003677|DNA binding;GO:0046872|metal ion binding;GO:0042975	K11471	ASXL; additional sex combs-like protein
ZC3H18	777.0139	0.329123	0.115003095	2.861863	0.0042116	0.02931	Not_Sig	no	gi|528918819|ref|XP_005192716.1|	1460	4e-180	PREDICTED: zinc finger CCCH domain-containing protein 18 isoform X2 [Bos taurus]	gi|982999089|ref|XM_005192658.3|	3649	0	PREDICTED: Bos taurus zinc finger CCCH-type containing 18 (ZC3H18), transcript variant X1, mRNA	Q86VM9	428	9e-130	Zinc finger CCCH domain-containing protein 18	.	.	.	.	pfam03344	Daxx	Daxx Family. The Daxx protein (also known as the Fas-binding protein) is thought to play a role in apoptosis, but precise role played	.	GO:0005654|nucleoplasm;	GO:0046872|metal ion binding;GO:0044822|poly(A) RNA	K13092	ZC3H18; nuclear protein NHN1
OXA1L	1010.875	0.362654	0.126731889	2.861581	0.0042153	0.029319	Not_Sig	no	gi|742233840|ref|XP_010829542.1|	1969	0	PREDICTED: mitochondrial inner membrane protein OXA1L [Bison bison bison]	gi|555966952|ref|XM_005895463.1|	1863	0	PREDICTED: Bos mutus mitochondrial inner membrane protein OXA1L (LOC102283387), mRNA	Q3SYV3	758	0	Mitochondrial inner membrane protein OXA1L	SPBC1346.02c	170	4.75e-46	COG0706	pfam02096	60KD_IMP	60Kd inner membrane protein.	GO:0097031|mitochondrial respiratory chain complex I biogenesis;GO:0051205|protein insertion into membrane;GO:0051262|protein tetramerization;	GO:0016021|integral component of membrane;GO:0005743|mitochondrial inner membrane;GO:0031966|mitochondrial	GO:0097177|mitochondrial ribosome binding;GO:0042803|protein	K03217	yidC, spoIIIJ, OXA1; YidC/Oxa1 family membrane protein insertase
ZBED4	438.0482	0.368199	0.128706403	2.860764	0.0042262	0.029376	Not_Sig	no	gi|594085691|ref|XP_006066366.1|	5254	0	PREDICTED: LOW QUALITY PROTEIN: zinc finger BED domain-containing protein 4 [Bubalus bubalis]	gi|594085690|ref|XM_006066304.1|	4392	0	PREDICTED: Bubalus bubalis zinc finger, BED-type containing 4 (ZBED4), mRNA	Q80WQ9	1571	0	Zinc finger BED domain-containing protein 4	.	.	.	.	pfam02892	zf-BED	BED zinc finger.	GO:0006357|regulation of transcription from RNA polymerase II promoter;	GO:0005737|cytoplasm;GO:0005634|nucleus;	GO:0046872|metal ion binding;GO:0000977|RNA polymerase II	.	.
CNTNAP1	328.1816	0.452781	0.158304428	2.860189	0.0042339	0.029412	Not_Sig	no	gi|555974513|ref|XP_005899249.1|	6764	0	PREDICTED: contactin-associated protein 1 [Bos mutus]	gi|594063515|ref|XM_006055726.1|	5710	0	PREDICTED: Bubalus bubalis contactin associated protein 1 (CNTNAP1), transcript variant X1, mRNA	P78357	2545	0	Contactin-associated protein 1	.	.	.	.	pfam02210	Laminin_G_2	Laminin G domain. This family includes the Thrombospondin N-terminal-like domain, a Laminin G subfamily.	GO:0007155|cell adhesion;GO:0007010|cytoskeleton organization;GO:0050885|neuromuscular process controlling balance;GO:0050884|neuromuscular process controlling posture;GO:0048812|neuron projection morphogenesis;GO:0019227|neuronal action potential propagation;GO:0030913|paranodal junction assembly;GO:0002175|protein localization to paranode region of axon;GO:0007165|signal transduction;	GO:0016021|integral component of membrane;GO:0005887|integral component of plasma membrane;GO:0043209|myelin	GO:0004872|receptor activity;GO:0017124|SH3 domain	K07379	CNTNAP1; contactin associated protein 1
PIK3CA	1810.615	-0.35761	0.125087577	-2.8589	0.0042511	0.029509	Not_Sig	no	gi|835020386|pdb|4YKN|A	5783	0	Chain A, Pi3k Alpha Lipid Kinase With Active Site Inhibitor	gi|742185400|ref|XM_010855613.1|	9138	0	PREDICTED: Bison bison bison phosphatidylinositol-4,5-bisphosphate 3-kinase, catalytic subunit alpha (PIK3CA), transcript variant X1, mRNA	P32871	2227	0	Phosphatidylinositol 4,5-bisphosphate 3-kinase catalytic subunit alpha isoform	SPAC458.05	218	2.15e-57	COG5032	pfam00613	PI3Ka	Phosphoinositide 3-kinase family, accessory domain (PIK domain). PIK domain is conserved in all PI3 and PI4-kinases. Its role is unclear	GO:0060612|adipose tissue development;GO:0001525|angiogenesis;GO:0071333|cellular response to glucose stimulus;GO:0097009|energy homeostasis;GO:0006006|glucose metabolic process;GO:0044029|hypomethylation of CpG island;GO:0001889|liver development;GO:0098779|mitophagy in response to mitochondrial depolarization;GO:2000811|negative regulation of anoikis;GO:2000270|negative regulation of fibroblast apoptotic process;GO:0043524|negative regulation of neuron apoptotic	GO:0005622|intracellular;GO:0030027|lamellipodium;GO:0005942|phosphatidylinositol 3-kinase complex;GO:0005943|phosphatidylinositol	GO:0016303|1-phosphatidylinositol-3-kinase activity;GO:0035005	K00922	PIK3C; phosphatidylinositol-4,5-bisphosphate 3-kinase [EC:2.7.1.153]
CTC1	211.659	0.471714	0.165014717	2.858618	0.0042549	0.029509	Not_Sig	no	gi|742136977|ref|XP_010841345.1|	5893	0	PREDICTED: CST complex subunit CTC1 isoform X1 [Bison bison bison]	gi|742136976|ref|XM_010843043.1|	4072	0	PREDICTED: Bison bison bison CTS telomere maintenance complex component 1 (CTC1), transcript variant X1, mRNA	Q2NKJ3	1709	0	CST complex subunit CTC1	.	.	.	.	pfam15489	CTC1	CST, telomere maintenance, complex subunit CTC1. CTC1 is one of the three components of the CST complex that assists Shelterin	GO:0048539|bone marrow development;GO:0006974|cellular response to DNA damage stimulus;GO:0071425|hematopoietic stem cell proliferation;GO:0035264|multicellular organism growth;GO:0051974|negative regulation of telomerase activity;GO:0045740|positive regulation of DNA replication;GO:0048146|positive regulation of fibroblast proliferation;GO:0010389|regulation of G2/M transition of mitotic cell cycle;GO:1904356|regulation of telomere maintenance via telomere lengthening;GO:0090399|replicative senescence;GO:0048536|spleen	GO:0000784|nuclear chromosome, telomeric region;GO:0005634|nucleus;	GO:0098505|G-rich strand telomeric DNA binding;GO:0003697	.	.
LLGL1	841.5809	0.348273	0.121839875	2.858445	0.0042572	0.029509	Not_Sig	no	gi|528995297|ref|XP_005220468.1|	5321	0	PREDICTED: lethal(2) giant larvae protein homolog 1 isoform X1 [Bos taurus]	gi|982957335|ref|XM_005220411.3|	3693	0	PREDICTED: Bos taurus lethal giant larvae homolog 1 (Drosophila) (LLGL1), transcript variant X1, mRNA	Q15334	1845	0	Lethal(2) giant larvae protein homolog 1	.	.	.	.	pfam08366	LLGL	LLGL2. This domain is found in lethal giant larvae homolog 2 (LLGL2) proteins and syntaxin-binding proteins like tomosyn. It	GO:0007409|axonogenesis;GO:0030866|cortical actin cytoskeleton organization;GO:0051294|establishment of spindle orientation;GO:0006887|exocytosis;GO:0006893|Golgi to plasma membrane transport;GO:0022008|neurogenesis;GO:0006461|protein complex assembly;GO:0032878|regulation of establishment or maintenance of cell polarity;GO:0008593|regulation of Notch signaling pathway;GO:0050708|regulation of protein secretion;	GO:0030424|axon;GO:0030864|cortical actin cytoskeleton;GO:0005737|cytoplasm;GO:0005856|cytoskeleton;GO:0031901|early	GO:0005096|GTPase activator activity;GO:0019901|protein kinase	K06094	LLGL; lethal(2) giant larvae protein
INPP5E	104.11	0.589962	0.206397544	2.858378	0.0042581	0.029509	UP	yes	gi|555952786|ref|XP_005888574.1|	3091	0	PREDICTED: 72 kDa inositol polyphosphate 5-phosphatase [Bos mutus]	gi|982939755|ref|XM_005213206.3|	2888	0	PREDICTED: Bos taurus inositol polyphosphate-5-phosphatase E (INPP5E), transcript variant X2, mRNA	Q9JII1	877	0	72 kDa inositol polyphosphate 5-phosphatase	YNL106c_2	159	9.70e-40	COG5411	pfam03372	Exo_endo_phos	Endonuclease/Exonuclease/phosphatase family. This large family of proteins includes magnesium dependent endonucleases and a	GO:0046855|inositol phosphate dephosphorylation;GO:0046856|phosphatidylinositol dephosphorylation;GO:0046488|phosphatidylinositol metabolic process;	GO:0005930|axoneme;GO:0032580|Golgi cisterna membrane;GO:0000139|Golgi membrane;GO:0005886|plasma membrane;GO:0001726|ruffle;	GO:0004439|phosphatidylinositol-4,5-bisphosphate 5-phosphatase	.	.
ADAMTS7	956.0308	0.422375	0.147792974	2.857884	0.0042648	0.029533	Not_Sig	no	gi|594100487|ref|XP_006073345.1|	7356	0	PREDICTED: A disintegrin and metalloproteinase with thrombospondin motifs 7-like [Bubalus bubalis]	gi|594100486|ref|XM_006073283.1|	5115	0	PREDICTED: Bubalus bubalis ADAM metallopeptidase with thrombospondin type 1 motif, 7 (ADAMTS7), mRNA	Q9UKP4	2459	0	A disintegrin and metalloproteinase with thrombospondin motifs 7	.	.	.	.	pfam05986	ADAM_spacer1	ADAM-TS Spacer 1. This family represents the Spacer-1 region from the ADAM-TS family of metalloproteinases.	GO:0071773|cellular response to BMP stimulus;GO:0071347|cellular response to interleukin-1;GO:0071356|cellular response to tumor necrosis factor;GO:0032331|negative regulation of chondrocyte differentiation;GO:0036066|protein O-linked fucosylation;GO:0051603|proteolysis involved in cellular protein catabolic process;	GO:0009986|cell surface;GO:0005788|endoplasmic reticulum lumen;GO:0005578|proteinaceous extracellular matrix;	GO:0004222|metalloendopeptidase activity;GO:0008237|metallopeptidase	K08622	ADAMTS7; a disintegrin and metalloproteinase with
SLC35C1	112.4	0.560313	0.196068573	2.857741	0.0042667	0.029533	Not_Sig	no	gi|594035478|ref|XP_006042415.1|	1811	0	PREDICTED: GDP-fucose transporter 1 isoform X1 [Bubalus bubalis]	gi|942090756|ref|XM_005907598.2|	1610	0	PREDICTED: Bos mutus solute carrier family 35 (GDP-fucose transporter), member C1 (SLC35C1), mRNA	A6QM03	686	0	GDP-fucose transporter 1	.	.	.	.	pfam03151	TPT	Triose-phosphate Transporter family. This family includes transporters with a specificity for triose phosphate.	GO:0008643|carbohydrate transport;GO:0030259|lipid glycosylation;GO:0098779|mitophagy in response to mitochondrial depolarization;GO:0045746|negative regulation of Notch signaling pathway;GO:0002230|positive regulation of defense response to virus by host;GO:0098792|xenophagy;	GO:0000139|Golgi membrane;GO:0016021|integral component of membrane;	.	K15279	SLC35C1, FUCT1; solute carrier family 35 (GDP-fucose transporter),
CSDC2	179.7214	0.546353	0.191290762	2.856137	0.0042883	0.029664	Not_Sig	no	gi|528951599|ref|XP_005207457.1|	850	1e-103	PREDICTED: cold shock domain-containing protein C2 isoform X1 [Bos taurus]	gi|982927398|ref|XM_005207400.3|	2491	0	PREDICTED: Bos taurus cold shock domain containing C2, RNA binding (CSDC2), transcript variant X1, mRNA	Q9Y534	259	3e-79	Cold shock domain-containing protein C2	SMa0181	50.1	3.39e-07	COG1278	pfam00313	CSD	'Cold-shock' DNA-binding domain.	GO:0006397|mRNA processing;GO:0006355|regulation of transcription, DNA-templated;	GO:0005737|cytoplasm;GO:0005634|nucleus;	GO:0003677|DNA binding;GO:0003723|RNA binding;	.	.
SOX4	762.4615	-0.33982	0.118993814	-2.85579	0.004293	0.029679	Not_Sig	no	gi|296474047|tpg|DAA16162.1|	910	1e-106	TPA: SRY (sex determining region Y)-box 4 [Bos taurus]	gi|965884635|ref|XM_012154862.2|	4697	0	PREDICTED: Ovis aries musimon transcription factor SOX-4 (LOC105611687), mRNA	Q06945	298	7e-87	Transcription factor SOX-4	.	.	.	.	pfam00505	HMG_box	HMG (high mobility group) box.	GO:0035910|ascending aorta morphogenesis;GO:0003289|atrial septum primum morphogenesis;GO:0060070|canonical Wnt signaling pathway;GO:0003215|cardiac right ventricle morphogenesis;GO:0003211|cardiac ventricle formation;GO:0071333|cellular response to glucose stimulus;GO:0042769|DNA damage response, detection of DNA damage;GO:0006977|DNA damage response, signal transduction by p53 class mediator resulting in cell cycle arrest;GO:0031018|endocrine pancreas development;GO:0021782|glial cell	GO:0005737|cytoplasm;GO:0005739|mitochondrion;GO:0044798|nuclear transcription factor complex;GO:0005654|nucleoplasm;GO:000	GO:0001046|core promoter sequence-specific DNA binding;GO:0001071	K09268	SOX4_11_12; transcription factor SOX4/11/12 (SOX group C)
BAHCC1	367.1834	0.432666	0.151526689	2.855378	0.0042986	0.029696	Not_Sig	no	gi|803208637|ref|XP_011964824.1|	10333	0	PREDICTED: BAH and coiled-coil domain-containing protein 1 isoform X2 [Ovis aries musimon]	gi|965787383|ref|XM_012109434.2|	8904	0	PREDICTED: Ovis aries musimon BAH domain and coiled-coil containing 1 (BAHCC1), transcript variant X2, mRNA	Q9P281	3187	0	BAH and coiled-coil domain-containing protein 1	.	.	.	.	pfam01426	BAH	BAH domain. This domain has been called BAH (Bromo adjacent homology) domain and has also been called ELM1 and BAM	GO:0006342|chromatin silencing;GO:0031507|heterochromatin assembly;	GO:0000785|chromatin;GO:0005677|chromatin silencing complex;	GO:0003682|chromatin binding;GO:0000976|transcription	.	.
ARL6	128.5278	-0.57003	0.199644996	-2.85522	0.0043007	0.029696	Not_Sig	no	gi|115494910|ref|NP_001069250.1|	901	2e-109	ADP-ribosylation factor-like protein 6 [Bos taurus]	gi|742158968|ref|XM_010848387.1|	3652	0	PREDICTED: Bison bison bison ADP-ribosylation factor-like 6 (ARL6), mRNA	Q0IIM2	351	7e-111	ADP-ribosylation factor-like protein 6	YDL192w	147	1.42e-39	COG1100	pfam00025	Arf	ADP-ribosylation factor family. Pfam combines a number of different Prosite families together	GO:0007420|brain development;GO:0042384|cilium assembly;GO:0045444|fat cell differentiation;GO:0046909|intermembrane transport;GO:0097499|protein localization to nonmotile primary cilium;GO:0051258|protein polymerization;GO:0006612|protein targeting to membrane;GO:0008589|regulation of smoothened signaling pathway;GO:0010842|retina layer formation;GO:0007264|small GTPase mediated signal transduction;GO:0016055|Wnt signaling pathway;	GO:0005879|axonemal microtubule;GO:0005930|axoneme;GO:0060170|ciliary membrane;GO:0005829|cytosol;GO:007006	GO:0005525|GTP binding;GO:0005543|phospholipid binding;	K07951	ARL6, BBS3; ADP-ribosylation factor-like protein 6
CAMSAP1	886.6682	0.359154	0.125911234	2.852437	0.0043385	0.029925	Not_Sig	no	gi|741944288|ref|XP_010808697.1|	7647	0	PREDICTED: calmodulin-regulated spectrin-associated protein 1 isoform X1 [Bos taurus]	gi|982939756|ref|XM_010810395.2|	6221	0	PREDICTED: Bos taurus calmodulin regulated spectrin associated protein 1 (CAMSAP1), transcript variant X1, mRNA	Q5T5Y3	2279	0	Calmodulin-regulated spectrin-associated protein 1	.	.	.	.	pfam08683	CAMSAP_CKK	Microtubule-binding calmodulin-regulated spectrin-associated. This is the C-terminal domain of a family of eumetazoan proteins	GO:0007010|cytoskeleton organization;GO:0031175|neuron projection development;GO:0022604|regulation of cell morphogenesis;	GO:0005737|cytoplasm;GO:0005874|microtubule;	GO:0005516|calmodulin binding;GO:0008017|microtubule	K17493	CAMSAP; calmodulin-regulated spectrin-associated protein
SLC44A2	2433.108	0.310152	0.108733474	2.852409	0.0043389	0.029925	Not_Sig	no	gi|148223245|ref|NP_001091608.1|	3372	0	choline transporter-like protein 2 [Bos taurus]	gi|942041257|ref|XM_005890793.2|	3270	0	PREDICTED: Bos mutus solute carrier family 44 (choline transporter), member 2 (SLC44A2), transcript variant X2, mRNA	A5D7H3	1300	0	Choline transporter-like protein 2	.	.	.	.	pfam04515	Choline_transpo	Plasma-membrane choline transporter. This family represents a high-affinity plasma-membrane choline transporter in C.elegans	GO:0043123|positive regulation of I-kappaB kinase/NF-kappaB signaling;GO:0006810|transport;	GO:0070062|extracellular exosome;GO:0016021|integral component of membrane;GO:0005765|lysosomal membrane;	GO:0004871|signal transducer activity;	K15377	SLC44A2_4_5; solute carrier family 44 (choline transporter-like protein),
PET100	92.80769	-0.63438	0.222441375	-2.8519	0.0043459	0.029954	DOWN	yes	gi|426229047|ref|XP_004008605.1|	385	2e-45	PREDICTED: protein PET100 homolog, mitochondrial [Ovis aries]	gi|942041290|ref|XM_014476705.1|	394	0	PREDICTED: Bos mutus PET100 homolog (PET100), mRNA	E1BHC3	150	3e-46	Protein PET100 homolog, mitochondrial	.	.	.	.	pfam09803	DUF2346	Uncharacterized conserved protein (DUF2346). Members of this family of proteins have no known function.	GO:0033617|mitochondrial respiratory chain complex IV assembly;	GO:0016021|integral component of membrane;GO:0005739|mitochondrion;	.	K18186	PET100; protein PET100, animal type
CROT	627.9051	-0.35018	0.122798476	-2.85165	0.0043493	0.02996	Not_Sig	no	gi|742199155|ref|XP_010857434.1|	3295	0	PREDICTED: peroxisomal carnitine O-octanoyltransferase [Bison bison bison]	gi|942070253|ref|XM_005900998.2|	3152	0	PREDICTED: Bos mutus carnitine O-octanoyltransferase (CROT), mRNA	O19094	1272	0	Peroxisomal carnitine O-octanoyltransferase	.	.	.	.	pfam00755	Carn_acyltransf	Choline/Carnitine o-acyltransferase.	GO:0009437|carnitine metabolic process;GO:0015936|coenzyme A metabolic process;GO:0006635|fatty acid beta-oxidation;GO:0006631|fatty acid metabolic process;GO:0006091|generation of precursor metabolites and energy;GO:0051791|medium-chain fatty acid metabolic process;GO:0006810|transport;	GO:0005777|peroxisome;	GO:0008458|carnitine O-octanoyltransferase activity;	K05940	CROT; carnitine O-octanoyltransferase [EC:2.3.1.137]
DPY19L4	351.2134	-0.44468	0.155953585	-2.85134	0.0043536	0.029972	Not_Sig	no	gi|194673034|ref|XP_609930.4|	3474	0	PREDICTED: probable C-mannosyltransferase DPY19L4 isoform X1 [Bos taurus]	gi|982994732|ref|XM_609930.9|	4072	0	PREDICTED: Bos taurus dpy-19 like 4 (C. elegans) (DPY19L4), transcript variant X1, mRNA	Q5R8N9	1305	0	Probable C-mannosyltransferase DPY19L4	.	.	.	.	pfam10034	Dpy19	Q-cell neuroblast polarisation. Dyp-19, formerly known as DUF2211, is a transmembrane domain family that is required to	.	GO:0016021|integral component of membrane;	GO:0016757|transferase activity, transferring glycosyl groups;	.	.
RFTN2	39.05636	-0.86769	0.304332875	-2.85113	0.0043564	0.029973	DOWN	yes	gi|528939655|ref|XP_005202665.1|	2541	0	PREDICTED: raftlin-2 isoform X1 [Bos taurus]	gi|742206628|ref|XM_010860910.1|	2968	0	PREDICTED: Bison bison bison raftlin family member 2 (RFTN2), transcript variant X2, mRNA	Q52LD8	843	0	Raftlin-2	.	.	.	.	pfam15250	Raftlin	Raftlin. This family of proteins plays a role in the formation and/or maintenance of lipid rafts.	GO:0050851|antigen receptor-mediated signaling pathway;GO:0033227|dsRNA transport;GO:0032596|protein transport into membrane raft;GO:0043330|response to exogenous dsRNA;	GO:0005737|cytoplasm;GO:0045121|membrane raft;GO:0005886|plasma membrane;	.	.	.
TUG1	50.63854	-0.79898	0.280273651	-2.8507	0.0043623	0.029979	DOWN	yes	gi|149047521|gb|EDM00191.1|	181	1e-14	rCG36134 [Rattus norvegicus]	gi|594053630|ref|XR_326862.1|	704	0	PREDICTED: Bubalus bubalis uncharacterized LOC102411461 (LOC102411461), transcript variant X2, misc_RNA	.	.	.	.	.	.	.	.	pfam15394	DUF4616	Domain of unknown function (DUF4616). This protein family is a domain of unknown function found at the C-terminal domain of	.	.	.	.	.
MEMO1	614.6596	-0.3429	0.120293371	-2.85052	0.0043648	0.029979	Not_Sig	no	gi|918608340|ref|XP_013373062.1|	1673	0	PREDICTED: protein MEMO1 [Chinchilla lanigera]	gi|602731236|ref|XM_007450962.1|	1566	0	PREDICTED: Lipotes vexillifer mediator of cell motility 1 (MEMO1), transcript variant X1, mRNA	Q4R6D9	626	0	Protein MEMO1	SPAC4H3.04c	221	6.60e-67	COG1355	pfam01875	Memo	Memo-like protein. This family contains members from all branches of life. The molecular function of this protein is	GO:0032886|regulation of microtubule-based process;	.	.	K06990	K06990; uncharacterized protein
NOP2	808.7205	0.358494	0.125769421	2.850408	0.0043663	0.029979	Not_Sig	no	gi|555991476|ref|XP_005907522.1|	3759	0	PREDICTED: probable 28S rRNA (cytosine(4447)-C(5))-methyltransferase isoform X3 [Bos mutus]	gi|742143965|ref|XM_010844965.1|	2656	0	PREDICTED: Bison bison bison NOP2 nucleolar protein (NOP2), transcript variant X2, mRNA	P46087	1143	0	Probable 28S rRNA (cytosine(4447)-C(5))-methyltransferase	SPBP8B7.20c	508	1.36e-169	COG0144	pfam01189	Nol1_Nop2_Fmu	NOL1/NOP2/sun family.	GO:0000470|maturation of LSU-rRNA;GO:0008284|positive regulation of cell proliferation;GO:0070475|rRNA base methylation;	GO:0005730|nucleolus;	GO:0044822|poly(A) RNA binding;GO:0009383|rRNA (cytosine-	K14835	NOP2; ribosomal RNA methyltransferase Nop2 [EC:2.1.1.-]
SLC16A5	840.6846	0.373341	0.130983084	2.850302	0.0043678	0.029979	Not_Sig	no	gi|555992549|ref|XP_005908049.1|	2417	0	PREDICTED: monocarboxylate transporter 6 [Bos mutus]	gi|742187316|ref|XM_010856149.1|	2804	0	PREDICTED: Bison bison bison solute carrier family 16 (monocarboxylate transporter), member 5 (SLC16A5), transcript variant X9, mRNA	O15375	684	0	Monocarboxylate transporter 6	.	.	.	.	pfam07690	MFS_1	Major Facilitator Superfamily.	GO:0015718|monocarboxylic acid transport;GO:0035879|plasma membrane lactate transport;	GO:0005887|integral component of plasma membrane;GO:0016020|membrane;	GO:0015129|lactate transmembrane transporter activity;GO:0008028	K08182	SLC16A5; MFS transporter, MCP family, solute carrier family 16
LOC102276651	159.3243	-0.62225	0.218326337	-2.8501	0.0043705	0.029981	DOWN	yes	gi|34785465|gb|AAH57655.1|	1014	2e-131	Hnrpa3 protein, partial [Mus musculus]	gi|982913279|ref|XM_015474992.1|	1560	0	PREDICTED: Bos taurus heterogeneous nuclear ribonucleoprotein A3 (HNRNPA3), transcript variant X3, mRNA	P51991	384	1e-127	Heterogeneous nuclear ribonucleoprotein A3	.	.	.	.	pfam00076	RRM_1	RNA recognition motif. (a.k.a. RRM, RBD, or RNP domain). The RRM motif is probably diagnostic of an RNA binding protein. RRMs	GO:0010467|gene expression;GO:0000398|mRNA splicing, via spliceosome;GO:0051028|mRNA transport;	GO:0071013|catalytic step 2 spliceosome;GO:0005737|cytoplasm;GO:0030529|ribonucleoprotein complex;GO:1990124|messenger	GO:0003729|mRNA binding;GO:0000166|nucleotide binding;GO:0044822	K12741	HNRNPA1_3; heterogeneous nuclear ribonucleoprotein
SNX3	1619.016	-0.30098	0.105768114	-2.84569	0.0044315	0.030381	Not_Sig	no	gi|617628971|ref|XP_007529105.1|	872	5e-113	PREDICTED: sorting nexin-3 [Erinaceus europaeus]	gi|942060794|ref|XM_005898188.2|	1308	0	PREDICTED: Bos mutus sorting nexin 3 (SNX3), transcript variant X1, mRNA	Q5U211	336	3e-113	Sorting nexin-3	SPBC887.06c	132	2.72e-36	COG5391	pfam00787	PX	PX domain. PX domains bind to phosphoinositides.	GO:0006897|endocytosis;GO:0050765|negative regulation of phagocytosis;GO:0010976|positive regulation of neuron projection development;GO:0022615|protein to membrane docking;GO:0015031|protein transport;GO:0030111|regulation of Wnt signaling pathway;GO:0016050|vesicle organization;	GO:0030136|clathrin-coated vesicle;GO:0005769|early endosome;GO:0032009|early phagosome;GO:0005768|endosome;GO:001	GO:0035091|phosphatidylinositol binding;GO:0080025|phosphatidylinosit	K17918	SNX3_12; sorting nexin-3/12

CRTC1	221.4197	0.539156	0.189500158	2.845147	0.0044391	0.030398	Not_Sig	no	gi|528954340|ref|XP_005208500.1|	2892	0	PREDICTED: CREB-regulated transcription coactivator 1 isoform X1 [Bos taurus]	gi|982929473|ref|XM_005208443.3|	2475	0	PREDICTED: Bos taurus CREB regulated transcription coactivator 1 (CRTC1), transcript variant X1, mRNA	Q6UUV9	1012	0	CREB-regulated transcription coactivator 1	.	.	.	.	pfam12885	TORC_M	Transducer of regulated CREB activity middle domain. This family includes the region between the N and C terminus of TORC proteins.	GO:0043153|entrainment of circadian clock by photoperiod;GO:0060081|membrane hyperpolarization;GO:0007613|memory;GO:0032793|positive regulation of CREB transcription factor activity;GO:1900006|positive regulation of dendrite development;GO:1900273|positive regulation of long-term synaptic potentiation;GO:0045944|positive regulation of transcription from RNA polymerase II promoter;GO:0051289|protein homotetramerization;GO:0048511|rhythmic process;GO:0006351|transcription, DNA-	GO:0005737|cytoplasm;GO:0030425|dendrite;GO:0043025|neuronal cell body;GO:0005654|nucleoplasm;GO:0005634|nucleus;GO:0005886|plasma	GO:0008140|cAMP response element binding protein binding;	K15309	CRTC1, TORC1; CREB-regulated transcription coactivator 1
ACSF2	483.1773	0.441204	0.155073421	2.84513	0.0044393	0.030398	Not_Sig	no	gi|555956460|ref|XP_005890378.1|	3213	0	PREDICTED: acyl-CoA synthetase family member 2, mitochondrial isoform X1 [Bos mutus]	gi|942039912|ref|XM_005890316.2|	2062	0	PREDICTED: Bos mutus acyl-CoA synthetase family member 2 (ACSF2), transcript variant X1, mRNA	Q17QJ1	1216	0	Acyl-CoA synthetase family member 2, mitochondrial	RSc0442	542	0	COG0318	pfam00501	AMP-binding	AMP-binding enzyme.	GO:0006631|fatty acid metabolic process;	GO:0005739|mitochondrion;	GO:0005524|ATP binding;GO:0016874|ligase activity;	K00666	K00666; fatty-acyl-CoA synthase [EC:6.2.1.-]
MFSD13A	140.6321	0.558562	0.196335347	2.844941	0.004442	0.030398	Not_Sig	no	gi|555951842|ref|XP_005888116.1|	2016	0	PREDICTED: transmembrane protein 180 [Bos mutus]	gi|982968090|ref|XM_005225526.3|	2367	0	PREDICTED: Bos taurus major facilitator superfamily domain containing 13A (MFSD13A), transcript variant X2, mRNA	Q58CT4	778	0	Transmembrane protein 180	.	.	.	.	pfam13347	MFS_2	MFS/sugar transport protein. This family is part of the major facilitator superfamily of membrane transport proteins.	.	GO:0016021|integral component of membrane;	.	.	.
LAMTOR3	568.2013	-0.41485	0.145845363	-2.84446	0.0044487	0.030409	Not_Sig	no	gi|115496109|ref|NP_001069450.1|	645	4e-79	ragulator complex protein LAMTOR3 [Bos taurus]	gi|942098696|ref|XM_005910305.2|	1459	0	PREDICTED: Bos mutus late endosomal/lysosomal adaptor, MAPK and MTOR activator 3 (LAMTOR3), mRNA	Q17QQ1	253	1e-80	Ragulator complex protein LAMTOR3	.	.	.	.	pfam08923	MAPKK1_Int	Mitogen-activated protein kinase kinase 1 interacting. Mitogen-activated protein kinase kinase 1 interacting protein is a small	GO:0000186|activation of MAPKK activity;GO:0034613|cellular protein localization;GO:0071230|cellular response to amino acid stimulus;GO:0032008|positive regulation of TOR signaling;	GO:0070062|extracellular exosome;GO:0005925|focal adhesion;GO:0071986|Ragulator complex;	GO:0005085|guanyl-nucleotide exchange factor activity;GO:0019209	K04370	MP1, MAP2K1IP1; mitogen-activated protein kinase kinase 1 interacting protein
EPS8L2	481.8858	0.452121	0.158948937	2.844441	0.0044489	0.030409	Not_Sig	no	gi|594042629|ref|XP_006045845.1|	3432	0	PREDICTED: epidermal growth factor receptor kinase substrate 8-like protein 2 [Bubalus bubalis]	gi|803331739|ref|XM_012168594.1|	2421	0	PREDICTED: Ovis aries musimon EPS8-like 2 (EPS8L2), transcript variant X4, mRNA	Q5RC07	1155	0	Epidermal growth factor receptor kinase substrate 8-like protein 2	.	.	.	.	pfam08416	PTB	Phosphotyrosine-binding domain. The phosphotyrosine-binding domain (PTB, also phosphotyrosine-interaction or PI	.	GO:0005737|cytoplasm;	.	K17277	EPS8; epidermal growth factor receptor kinase substrate 8
DES	167.8063	-0.50008	0.175859408	-2.84363	0.0044603	0.030458	Not_Sig	no	gi|126158909|ref|NP_001075044.1|	1926	0	desmin [Bos taurus]	gi|126158908|ref|NM_001081575.1|	2222	0	Bos taurus desmin (DES), mRNA	O62654	746	0	Desmin	.	.	.	.	pfam00038	Filament	Intermediate filament protein.	GO:0045109|intermediate filament organization;	GO:0070062|extracellular exosome;GO:0005916|fascia adherens;GO:0014704|intercalated disc;GO:0005882|intermediate	GO:0005198|structural molecule activity;	K07610	DES; desmin
BCL7B	505.7593	0.359445	0.126406718	2.843558	0.0044613	0.030458	Not_Sig	no	gi|77736493|ref|NP_001029947.1|	871	1e-110	B-cell CLL/lymphoma 7 protein family member B [Bos taurus]	gi|594066385|ref|XM_006057103.1|	1634	0	PREDICTED: Bubalus bubalis B-cell CLL/lymphoma 7B (BCL7B), mRNA	Q3T0A6	340	4e-112	B-cell CLL/lymphoma 7 protein family member B	.	.	.	.	pfam04714	BCL_N	BCL7, N-terminal conserver region. Members of the BCL family have significant sequence similarity at their N-terminus, represented in	GO:0006915|apoptotic process;GO:0030154|cell differentiation;GO:0016055|Wnt signaling pathway;	.	.	.	.
SYCP3	66.81581	-0.7812	0.274743902	-2.84336	0.004464	0.030458	DOWN	yes	gi|440903711|gb|ELR54336.1|	1218	5e-160	Synaptonemal complex protein 3, partial [Bos mutus]	gi|594038061|ref|XM_006043591.1|	1970	0	PREDICTED: Bubalus bubalis synaptonemal complex protein 3 (SYCP3), transcript variant X1, mRNA	Q3T0E2	438	7e-148	Synaptonemal complex protein 3	.	.	.	.	pfam04803	Cor1	Cor1/Xlr/Xmr conserved region. Cor1 is a component of the chromosome core in the meiotic prophase chromosomes. Xlr is a	GO:0051301|cell division;GO:0051321|meiotic cell cycle;GO:0007286|spermatid development;	GO:0000775|chromosome, centromeric region;GO:0000800|lateral element;GO:0005634|nucleus;	GO:0003677|DNA binding;	K19528	SYCP3; synaptonemal complex protein 3
TRAPPC4	421.7889	-0.35314	0.124262122	-2.84188	0.0044848	0.030582	Not_Sig	no	gi|84370033|ref|NP_001033591.1|	1124	4e-151	trafficking protein particle complex subunit 4 [Bos taurus]	gi|742230545|ref|XM_010830444.1|	1200	0	PREDICTED: Bison bison bison trafficking protein particle complex 4 (TRAPPC4), mRNA	Q2TBL9	437	1e-152	Trafficking protein particle complex subunit 4	SPBC3B9.12	115	1.45e-30	COG5122	pfam04099	Sybindin	Sybindin-like family. Sybindin is a physiological syndecan-2 ligand on dendritic spines, the small protrusions on the surface of	GO:0016358|dendrite development;GO:0006888|ER to Golgi vesicle-mediated transport;	GO:0030425|dendrite;GO:0005783|endoplasmic reticulum;GO:0005795|Golgi stack;GO:0008021|synaptic vesicle;GO:0030008|TRAPP complex;	GO:0017112|Rab guanyl-nucleotide exchange factor activity;	.	.
GLA	2210.647	-0.31054	0.1093201	-2.84069	0.0045016	0.030678	Not_Sig	no	gi|555978281|ref|XP_005901085.1|	2359	0	PREDICTED: alpha-galactosidase A [Bos mutus]	gi|942070317|ref|XM_005901023.2|	1942	0	PREDICTED: Bos mutus galactosidase, alpha (GLA), mRNA	P06280	709	0	Alpha-galactosidase A	.	.	.	.	pfam00078	RVT_1	Reverse transcriptase (RNA-dependent DNA polymerase). A reverse transcriptase gene is usually indicative of a mobile	GO:0046479|glycosphingolipid catabolic process;GO:0006687|glycosphingolipid metabolic process;GO:0046477|glycosylceramide catabolic process;GO:0045019|negative regulation of nitric oxide biosynthetic process;GO:0051001|negative regulation of nitric-oxide synthase activity;GO:0009311|oligosaccharide metabolic process;	GO:0005737|cytoplasm;GO:0070062|extracellular exosome;GO:0005576|extracellular region;GO:0005794|Golgi apparatus;GO:0043202|lysosomal	GO:0004557|alpha-galactosidase activity;GO:0003824|catalytic	K01189	GLA; alpha-galactosidase [EC:3.2.1.22]
ACVR1B	465.0493	0.362341	0.127585255	2.839995	0.0045114	0.030713	Not_Sig	no	gi|743736574|ref|XP_010962622.1|	2434	0	PREDICTED: activin receptor type-1B [Camelus bactrianus]	gi|982981620|ref|XM_015463282.1|	4475	0	PREDICTED: Bos taurus activin A receptor type IB (ACVR1B), transcript variant X2, mRNA	P36896	938	0	Activin receptor type-1B	.	.	.	.	pfam00069	Pkinase	Protein kinase domain.	GO:0032924|activin receptor signaling pathway;GO:0007417|central nervous system development;GO:0046545|development of primary female sexual characteristics;GO:0097191|extrinsic apoptotic signaling pathway;GO:0000082|G1/S transition of mitotic cell cycle;GO:0001942|hair follicle development;GO:0001701|in utero embryonic development;GO:0030308|negative regulation of cell growth;GO:0010629|negative regulation of gene expression;GO:0038092|nodal signaling pathway;GO:0018107|peptidyl-threonine	GO:0048179|activin receptor complex;GO:0009986|cell surface;GO:0070062|extracellular exosome;GO:0005887|integral component	GO:0016361|activin receptor activity, type I;GO:0005524|ATP	K13567	ACVR1B, ALK4; activin receptor type-1B [EC:2.7.11.30]
SRSF11	2086.721	-0.29602	0.104233242	-2.83995	0.0045121	0.030713	Not_Sig	no	gi|955519742|ref|XP_014643842.1|	373	2e-34	PREDICTED: serine/arginine-rich splicing factor 11, partial [Ceratotherium simum simum]	gi|965850441|ref|XM_012139852.2|	2798	0	PREDICTED: Ovis aries musimon serine/arginine-rich splicing factor 11 (SRSF11), transcript variant X7, mRNA	Q05519	141	2e-33	Serine/arginine-rich splicing factor 11	.	.	.	.	pfam06738	DUF1212	Protein of unknown function (DUF1212). This family represents a conserved region within a number of hypothetical membrane	GO:0031124|mRNA 3'-end processing;GO:0006406|mRNA export from nucleus;GO:0006397|mRNA processing;GO:0000398|mRNA splicing, via spliceosome;GO:0006405|RNA export from nucleus;GO:0008380|RNA splicing;GO:0006369|termination of RNA polymerase II transcription;	GO:0005654|nucleoplasm;GO:0005634|nucleus;	GO:0000166|nucleotide binding;GO:0044822|poly(A) RNA	K12899	SFRS11; splicing factor, arginine/serine-rich 11
YIPF4	615.2271	-0.41194	0.145087	-2.83929	0.0045215	0.030759	Not_Sig	no	gi|426223849|ref|XP_004006086.1|	964	9e-123	PREDICTED: protein YIPF4 [Ovis aries]	gi|594115941|ref|XM_006080587.1|	1852	0	PREDICTED: Bubalus bubalis Yip1 domain family, member 4 (YIPF4), mRNA	Q9BSR8	362	5e-119	Protein YIPF4	.	.	.	.	pfam04893	Yip1	Yip1 domain. The Yip1 integral membrane domain contains four transmembrane alpha helices. The domain is characterized by the	.	GO:0005783|endoplasmic reticulum;GO:0005794|Golgi apparatus;GO:0016021|integral component of membrane;GO:0043231|intracellular	.	.	.
ZMPSTE24	555.9205	-0.40673	0.143273312	-2.83886	0.0045276	0.030782	Not_Sig	no	gi|300793653|ref|NP_001179857.1|	2278	0	CAAX prenyl protease 1 homolog [Bos taurus]	gi|982920128|ref|XM_015466442.1|	2753	0	PREDICTED: Bos taurus zinc metallopeptidase STE24 (ZMPSTE24), transcript variant X1, mRNA	O75844	852	0	CAAX prenyl protease 1 homolog	SPAC3H1.05	237	6.20e-68	COG0501	pfam01435	Peptidase_M48	Peptidase family M48.	GO:0071586|CAAX-box protein processing;GO:0006998|nuclear envelope organization;GO:0030327|prenylated protein catabolic process;GO:0006508|proteolysis;	GO:0005789|endoplasmic reticulum membrane;GO:0070062|extracellular exosome;GO:0016021|integral component of	GO:0046872|metal ion binding;GO:0004222|metalloendopeptid	K06013	STE24; STE24 endopeptidase [EC:3.4.24.84]
ADCY9	519.9886	0.341609	0.120355388	2.838333	0.004535	0.030807	Not_Sig	no	gi|529005897|ref|XP_005224538.1|	6564	0	PREDICTED: adenylate cyclase type 9 isoform X1 [Bos taurus]	gi|982966159|ref|XM_005224481.3|	7320	0	PREDICTED: Bos taurus adenylate cyclase 9 (ADCY9), transcript variant X1, mRNA	O60503	2303	0	Adenylate cyclase type 9	all0661	165	2.09e-42	COG2114	pfam00211	Guanylate_cyc	Adenylate and Guanylate cyclase catalytic domain.	GO:0007190|activation of adenylate cyclase activity;GO:0034199|activation of protein kinase A activity;GO:0071880|adenylate cyclase-activating adrenergic receptor signaling pathway;GO:0007189|adenylate cyclase-activating G-protein coupled receptor signaling pathway;GO:0007193|adenylate cyclase-inhibiting G-protein coupled receptor signaling pathway;GO:0006171|cAMP biosynthetic process;GO:0019933|cAMP-mediated signaling;GO:0071377|cellular response to glucagon stimulus;GO:0003091|renal water	GO:0030424|axon;GO:0030425|dendrite;GO:0016021|integral component of membrane;GO:0005887|integral component of plasma	GO:0004016|adenylate cyclase activity;GO:0005524|ATP	K08049	ADCY9; adenylate cyclase 9 [EC:4.6.1.1]
PITRM1	817.0045	0.333357	0.117452716	2.838219	0.0045366	0.030807	Not_Sig	no	gi|528973266|ref|XP_005214527.1|	5409	0	PREDICTED: presequence protease, mitochondrial isoform X1 [Bos taurus]	gi|982993062|ref|XM_590885.7|	3236	0	PREDICTED: Bos taurus pitrilysin metallopeptidase 1 (PITRM1), transcript variant X2, mRNA	Q5JRX3	1754	0	Presequence protease, mitochondrial	SPBC119.17	640	0	COG1026	pfam08367	M16C_assoc	Peptidase M16C associated. This domain appears in eukaryotes as well as bacteria and tends to be found near the C-terminus of the	GO:0016485|protein processing;GO:0006508|proteolysis;	GO:0005759|mitochondrial matrix;GO:0005739|mitochondrion;	GO:0008047|enzyme activator activity;GO:0004222|metalloendopeptid	K06972	K06972; uncharacterized protein
SMIM26	127.9854	-0.64978	0.228959268	-2.83799	0.0045399	0.030811	DOWN	yes	gi|942036124|ref|XP_014338748.1|	792	4e-105	PREDICTED: uncharacterized protein LOC106701607 [Bos mutus]	gi|942036123|ref|XM_014483262.1|	590	0	PREDICTED: Bos mutus uncharacterized LOC106701607 (LOC106701607), mRNA	.	.	.	.	.	.	.	.	pfam07520	SrfB	Virulence factor SrfB. This family includes homologues of SsrAB is a two-component regulatory system encoded within the Salmonella	.	.	.	.	.
IMPDH2	940.2381	0.317906	0.112035151	2.837553	0.0045461	0.030835	Not_Sig	no	gi|742171576|ref|XP_010850185.1|	2859	0	PREDICTED: inosine-5'-monophosphate dehydrogenase 2 isoform X1 [Bison bison bison]	gi|742171575|ref|XM_010851883.1|	1755	0	PREDICTED: Bison bison bison IMP (inosine 5'-monophosphate) dehydrogenase 2 (IMPDH2), transcript variant X1, mRNA	Q3SWY3	1038	0	Inosine-5'-monophosphate dehydrogenase 2	.	.	.	.	pfam00478	IMPDH	IMP dehydrogenase / GMP reductase domain. This family is involved in biosynthesis of guanosine nucleotide. Members of	GO:0071353|cellular response to interleukin-4;GO:0006177|GMP biosynthetic process;GO:0006183|GTP biosynthetic process;GO:0046651|lymphocyte proliferation;	GO:0005737|cytoplasm;GO:0070062|extracellular exosome;GO:0005634|nucleus;GO:0005778|peroxisomal membrane;	GO:0003677|DNA binding;GO:0003938|IMP dehydrogenase	K00088	guaB; IMP dehydrogenase [EC:1.1.1.205]
EDEM3	1374.479	-0.39118	0.137905882	-2.83657	0.0045601	0.03089	Not_Sig	no	gi|741955256|ref|XP_010811859.1|	4578	0	PREDICTED: ER degradation-enhancing alpha-mannosidase-like protein 3 isoform X1 [Bos taurus]	gi|555962130|ref|XM_005893097.1|	6633	0	PREDICTED: Bos mutus ER degradation enhancer, mannosidase alpha-like 3 (EDEM3), transcript variant X1, mRNA	Q9BZQ6	1687	0	ER degradation-enhancing alpha-mannosidase-like protein 3	.	.	.	.	pfam01532	Glyco_hydro_47	Glycosyl hydrolase family 47. Members of this family are alpha-mannosidases that catalyse the hydrolysis of the terminal 1,2-	GO:1904380|endoplasmic reticulum mannose trimming;GO:0030968|endoplasmic reticulum unfolded protein response;GO:0030433|ER-associated ubiquitin-dependent protein catabolic process;GO:1904382|mannose trimming involved in glycoprotein ERAD pathway;GO:0006491|N-glycan processing;	GO:0005783|endoplasmic reticulum;GO:0005788|endoplasmic reticulum lumen;GO:0044322|endoplasmic reticulum quality control	GO:0004559|alpha-mannosidase activity;GO:0005509|calcium ion	K10086	EDEM3; ER degradation enhancer, mannosidase alpha-
ATRIP	283.3605	0.42291	0.149096518	2.836486	0.0045613	0.03089	Not_Sig	no	gi|157074172|ref|NP_001096800.1|	3394	0	ATR-interacting protein [Bos taurus]	gi|942096294|ref|XM_005909340.2|	2490	0	PREDICTED: Bos mutus ATR interacting protein (ATRIP), mRNA	Q8WXE1	1005	0	ATR-interacting protein	.	.	.	.	pfam12718	Tropomyosin_1	Tropomyosin like. This family is a set of eukaryotic tropomyosins. Within the yeast Tmp1 and Tmp2, biochemical and sequence	GO:0000077|DNA damage checkpoint;GO:0006260|DNA replication;GO:0036297|interstrand cross-link repair;GO:1901796|regulation of signal transduction by p53 class mediator;	GO:0005654|nucleoplasm;	GO:0070530|K63-linked polyubiquitin binding;	K10905	ATRIP; ATR interacting protein
ITGB1	39911.62	-0.27821	0.0980905	-2.83625	0.0045647	0.03089	Not_Sig	no	gi|555956187|ref|XP_005890243.1|	4097	0	PREDICTED: integrin beta-1 isoform X1 [Bos mutus]	gi|982942190|ref|XM_005214148.3|	3694	0	PREDICTED: Bos taurus integrin subunit beta 1 (ITGB1), transcript variant X1, mRNA	P53712	1560	0	Integrin beta-1	.	.	.	.	pfam00362	Integrin_beta	Integrin, beta chain. Integrins have been found in animals and their homologues have also been found in cyanobacteria, probably due to	GO:0033627|cell adhesion mediated by integrin;GO:0007160|cell-matrix adhesion;GO:0071404|cellular response to low-density lipoprotein particle stimulus;GO:0007229|integrin-mediated signaling pathway;GO:0090004|positive regulation of establishment of protein localization to plasma membrane;GO:0031623|receptor internalization;GO:0010710|regulation of collagen catabolic process;	GO:0009986|cell surface;GO:0005925|focal adhesion;GO:0008305|integrin complex;GO:0071438|invadopodium membrane;GO:0030027|lamellipodium;GO:	GO:0046872|metal ion binding;GO:0046982|protein	K05719	ITGB1; integrin beta 1
NDUFA4	1249.413	-0.40488	0.142761555	-2.83608	0.0045671	0.03089	Not_Sig	no	gi|444707155|gb|ELW48447.1|	408	1e-47	NADH dehydrogenase [ubiquinone] 1 alpha subcomplex subunit 4 [Tupaia chinensis]	gi|942096071|ref|XM_005909285.2|	525	0	PREDICTED: Bos mutus NDUFA4, mitochondrial complex associated (NDUFA4), transcript variant X1, mRNA	Q01321	152	3e-46	Cytochrome c oxidase subunit NDUFA4	.	.	.	.	pfam06522	B12D	NADH-ubiquinone reductase complex 1 MLRQ subunit. The MLRQ subunit of mitochondrial NADH-ubiquinone reductase	.	GO:0070062|extracellular exosome;GO:0005747|mitochondrial respiratory chain complex I;GO:0005751|mitochondrial respiratory	GO:0004129|cytochrome-c oxidase activity;GO:0008137|NADH	K03948	NDUFA4; NADH dehydrogenase (ubiquinone) 1 alpha subcomplex subunit
STX17	287.3041	-0.42928	0.151371155	-2.83596	0.0045688	0.03089	Not_Sig	no	gi|602699395|ref|XP_007461706.1|	1274	9e-156	PREDICTED: syntaxin-17 [Lipotes vexillifer]	gi|982933780|ref|XM_005210212.3|	6962	0	PREDICTED: Bos taurus syntaxin 17 (STX17), transcript variant X3, mRNA	Q5E9Y2	492	3e-156	Syntaxin-17	.	.	.	.	pfam05739	SNARE	SNARE domain. Most if not all vesicular membrane fusion events in eukaryotic cells are believed to be mediated by a conserved fusion	GO:0097352|autophagosome maturation;GO:0097111|endoplasmic reticulum-Golgi intermediate compartment organization;GO:0006888|ER to Golgi vesicle-mediated transport;GO:0007030|Golgi organization;GO:0006886|intracellular protein transport;GO:0034497|protein localization to pre-autophagosomal structure;GO:0048278|vesicle docking;GO:0006906|vesicle fusion;	GO:0000421|autophagosome membrane;GO:0005829|cytosol;GO:0012505|endomembrane system;GO:0005789|endoplasmic reticulum	GO:0005484|SNAP receptor activity;GO:0000149|SNARE binding;	K08491	STX17; syntaxin 17
CACNA1H	743.2471	0.502506	0.177207477	2.835693	0.0045726	0.03089	Not_Sig	no	gi|983006648|ref|XP_015324707.1|	8135	0	PREDICTED: LOW QUALITY PROTEIN: voltage-dependent T-type calcium channel subunit alpha-1H isoform X2 [Bos taurus]	gi|983006647|ref|XM_015469221.1|	5851	0	PREDICTED: Bos taurus calcium channel, voltage-dependent, T type, alpha 1H subunit (CACNA1H), transcript variant X2, mRNA	O88427	2774	0	Voltage-dependent T-type calcium channel subunit alpha-1H	.	.	.	.	pfam00520	Ion_trans	Ion transport protein. This family contains Sodium, Potassium, Calcium ion channels. This family is 6 transmembrane helices in which	GO:0032342|aldosterone biosynthetic process;GO:0070509|calcium ion import;GO:0006816|calcium ion transport;GO:0034651|cortisol biosynthetic process;GO:0034220|ion transmembrane transport;GO:0086010|membrane depolarization during action potential;GO:2000344|positive regulation of acrosome reaction;GO:0045956|positive regulation of calcium ion-dependent exocytosis;GO:0042391|regulation of membrane potential;	GO:0005901|caveola;GO:0016021|integral component of membrane;GO:0005886|plasma membrane;GO:0042383|sarcolemma;GO:00	GO:0005261|cation channel activity;GO:0008332|low voltage-gated	.	.
MEIS3	364.7855	0.508097	0.179180662	2.835667	0.004573	0.03089	Not_Sig	no	gi|528992437|ref|XP_005219418.1|	1721	0	PREDICTED: homeobox protein Meis3 isoform X1 [Bos taurus]	gi|594088100|ref|XM_006067446.1|	1826	0	PREDICTED: Bubalus bubalis Meis homeobox 3 (MEIS3), transcript variant X1, mRNA	Q99687	629	0	Homeobox protein Meis3	.	.	.	.	pfam05920	Homeobox_KN	Homeobox KN domain. This is a homeobox transcription factor KN domain conserved from fungi to human and plants.	GO:2001234|negative regulation of apoptotic signaling pathway;GO:0051897|positive regulation of protein kinase B signaling;	GO:0005634|nucleus;	GO:0003682|chromatin binding;GO:0043565|sequence-specific	K16671	MEIS3; homeobox protein Meis3
MAP3K6	210.3018	0.462568	0.163142148	2.835371	0.0045773	0.030901	Not_Sig	no	gi|742229255|ref|XP_010828425.1|	6299	0	PREDICTED: mitogen-activated protein kinase kinase kinase 6 isoform X1 [Bison bison bison]	gi|742229254|ref|XM_010830123.1|	4511	0	PREDICTED: Bison bison bison mitogen-activated protein kinase kinase kinase 6 (MAP3K6), transcript variant X1, mRNA	O95382	1967	0	Mitogen-activated protein kinase kinase kinase 6	.	.	.	.	pfam13281	DUF4071	Domain of unknown function (DUF4071). This domain is found at the N-terminus of many serine-threonine kinase-like proteins.	GO:0007257|activation of JUN kinase activity;GO:0006468|protein phosphorylation;GO:0007165|signal transduction;	GO:0005622|intracellular;	GO:0005524|ATP binding;GO:0000287|magnesium ion binding;GO:0004709	K04425	MAP3K6, ASK2; mitogen-activated protein kinase kinase kinase 6
HDAC7	1209.56	0.384605	0.135660019	2.835066	0.0045816	0.030912	Not_Sig	no	gi|528949021|ref|XP_005206430.1|	4374	0	PREDICTED: histone deacetylase 7 isoform X4 [Bos taurus]	gi|742108260|ref|XM_010837513.1|	5745	0	PREDICTED: Bison bison bison histone deacetylase 7 (HDAC7), mRNA	Q8WUI4	1493	0	Histone deacetylase 7	YNL021w	241	5.03e-66	COG0123	pfam00850	Hist_deacetyl	Histone deacetylase domain. Histones can be reversibly acetylated on several lysine residues. Regulation of	GO:0007043|cell-cell junction assembly;GO:0032703|negative regulation of interleukin-2 production;GO:1901223|negative regulation of NIK/NF-kappaB signaling;GO:0045668|negative regulation of osteoblast differentiation;GO:0000122|negative regulation of transcription from RNA polymerase II promoter;GO:0090050|positive regulation of cell migration involved in sprouting angiogenesis;GO:0016925|protein sumoylation;GO:0006351|transcription, DNA-templated;GO:0001570|vasculogenesis;	GO:0005737|cytoplasm;GO:0000118|histone deacetylase complex;GO:0005654|nucleoplasm;GO:0005634|nucleus;	GO:0071889|14-3-3 protein binding;GO:0033613|activating	K11408	HDAC7; histone deacetylase 7 [EC:3.5.1.98]
RNPS1	1701.55	0.325349	0.114783375	2.834457	0.0045904	0.030953	Not_Sig	no	gi|947237821|ref|XP_014444163.1|	499	5e-54	PREDICTED: LOW QUALITY PROTEIN: RNA-binding protein with serine-rich domain 1-like [Tupaia chinensis]	gi|742079196|ref|XM_010844828.1|	2132	0	PREDICTED: Bison bison bison RNA binding protein S1, serine-rich domain (RNPS1), transcript variant X2, mRNA	Q5NVM8	181	1e-49	RNA-binding protein with serine-rich domain 1	.	.	.	.	pfam00076	RRM_1	RNA recognition motif. (a.k.a. RRM, RBD, or RNP domain). The RRM motif is probably diagnostic of an RNA binding protein. RRMs	GO:0006397|mRNA processing;GO:0048025|negative regulation of mRNA splicing, via spliceosome;GO:0000184|nuclear-transcribed mRNA catabolic process, nonsense-mediated decay;GO:0043065|positive regulation of apoptotic process;GO:0000381|regulation of alternative mRNA splicing, via spliceosome;GO:0008380|RNA splicing;	GO:0061574|ASAP complex;GO:0005737|cytoplasm;GO:0016607|nuclear speck;	GO:0000166|nucleotide binding;GO:0003723|RNA binding;	K14325	RNPS1; RNA-binding protein with serine-rich domain 1
MINDY3	850.194	-0.35452	0.125105386	-2.83375	0.0046006	0.031003	Not_Sig	no	gi|115497904|ref|NP_001069575.1|	2289	0	protein FAM188A [Bos taurus]	gi|742202642|ref|XM_010859969.1|	2374	0	PREDICTED: Bison bison bison family with sequence similarity 188, member A (FAM188A), mRNA	Q0IIH8	886	0	Ubiquitin carboxyl-terminal hydrolase FAM188A	.	.	.	.	pfam13898	DUF4205	Domain of unknown function (DUF4205). The proteins in this family are uncharacterized but often named FAM188B.	GO:0006915|apoptotic process;	GO:0031965|nuclear membrane;GO:0005654|nucleoplasm;	GO:0036459|ubiquitinyl hydrolase activity;	.	.
PFKL	6874.158	0.313816	0.110793415	2.832441	0.0046194	0.031082	Not_Sig	no	gi|122692503|ref|NP_001073713.1|	4092	0	ATP-dependent 6-phosphofructokinase, liver type [Bos taurus]	gi|402691653|ref|NM_001080244.2|	2717	0	Bos taurus phosphofructokinase, liver (PFKL), mRNA	A1A4J1	1580	0	ATP-dependent 6-phosphofructokinase, liver type	SPBC16H5.02	639	0	COG0205	pfam00365	PFK	Phosphofructokinase.	GO:0030388|fructose 1,6-bisphosphate metabolic process;GO:0006002|fructose 6-phosphate metabolic process;GO:0006096|glycolytic process;GO:0046676|negative regulation of insulin secretion;GO:0051259|protein oligomerization;GO:0009749|response to glucose;	GO:0005945|6-phosphofructokinase complex;GO:0070062|extracellular exosome;GO:0016020|membrane;	GO:0003872|6-phosphofructokinase activity;GO:0005524	K00850	pfkA, PFK; 6-phosphofructokinase 1 [EC:2.7.1.11]
DLD	746.2984	-0.35853	0.12659161	-2.83215	0.0046236	0.031082	Not_Sig	no	gi|440891370|gb|ELR45099.1|	2740	0	Dihydrolipoyl dehydrogenase, mitochondrial, partial [Bos mutus]	gi|742131064|ref|XM_010841478.1|	3396	0	PREDICTED: Bison bison bison dihydrolipoamide dehydrogenase (DLD), transcript variant X1, mRNA	P09622	1015	0	Dihydrolipoyl dehydrogenase, mitochondrial	SPAC1002.09c	559	0	COG1249	pfam07992	Pyr_redox_2	Pyridine nucleotide-disulphide oxidoreductase. This family includes both class I and class II oxidoreductases and also NADH	GO:0006103|2-oxoglutarate metabolic process;GO:0007568|aging;GO:0009083|branched-chain amino acid catabolic process;GO:0045454|cell redox homeostasis;GO:0051068|dihydrolipoamide metabolic process;GO:0007369|gastrulation;GO:0046487|glyoxylate metabolic process;GO:0009106|lipoate metabolic process;GO:0006554|lysine catabolic process;GO:0061732|mitochondrial acetyl-CoA biosynthetic process from pyruvate;GO:0006120|mitochondrial electron transport,	GO:0043159|acrosomal matrix;GO:0005929|cilium;GO:0005759|mitochondrial matrix;GO:0005739|mitochondrion;GO:004	GO:0004148|dihydrolipoyl dehydrogenase activity;GO:0050660	K00382	DLD, lpd, pdhD; dihydrolipoamide dehydrogenase [EC:1.8.1.4]
CBX2	133.6445	0.513677	0.181388007	2.831926	0.0046269	0.031082	Not_Sig	no	gi|329663474|ref|NP_001192522.1|	2141	0	chromobox protein homolog 2 [Bos taurus]	gi|982958570|ref|XM_010816519.2|	4577	0	PREDICTED: Bos taurus chromobox 2 (CBX2), transcript variant X1, mRNA	Q14781	711	0	Chromobox protein homolog 2	.	.	.	.	pfam00385	Chromo	Chromo (CHRomatin Organisation MOdifier) domain.	GO:0030154|cell differentiation;GO:0016569|covalent chromatin modification;GO:0045137|development of primary sexual characteristics;GO:0000122|negative regulation of transcription from RNA polymerase II promoter;GO:0016925|protein sumoylation;GO:0006351|transcription, DNA-templated;	GO:0000791|euchromatin;GO:0000792|heterochromatin;GO:0005654|nucleoplasm;GO:0005634|nucleus;GO:0031519|PcG protein complex;GO:0035102|PRC1 complex;	GO:0003682|chromatin binding;GO:0003677|DNA binding;	K11451	CBX2; chromobox protein 2
ASB14	409.7968	-0.39821	0.140615233	-2.83188	0.0046276	0.031082	Not_Sig	no	gi|282721055|ref|NP_001030242.2|	3028	0	dynein heavy chain 12, axonemal [Bos taurus]	gi|594097058|ref|XM_006071714.1|	3360	0	PREDICTED: Bubalus bubalis ankyrin repeat and SOCS box containing 14 (ASB14), transcript variant X1, mRNA	A6NK59	1023	0	Ankyrin repeat and SOCS box protein 14	all2748	133	2.52e-32	COG0666	pfam12796	Ank_2	Ankyrin repeats (3 copies).	GO:0035556|intracellular signal transduction;	GO:0005737|cytoplasm;GO:0005634|nucleus;GO:0000151|ubiquitin ligase complex;	GO:0031625|ubiquitin protein ligase binding;GO:0004842|ubiquitin-protein	K10336	ASB14; ankyrin repeat and SOCS box protein 14
PAK2	1870.915	-0.35645	0.125873723	-2.83184	0.004628	0.031082	Not_Sig	no	gi|332078552|ref|NP_001193656.1|	2600	0	serine/threonine-protein kinase PAK 2 [Bos taurus]	gi|742094515|ref|XM_010834347.1|	9825	0	PREDICTED: Bison bison bison p21 protein (Cdc42/Rac)-activated kinase 2 (PAK2), mRNA	Q64303	998	0	Serine/threonine-protein kinase PAK 2	.	.	.	.	pfam00069	Pkinase	Protein kinase domain.	GO:0003300|cardiac muscle hypertrophy;GO:0071560|cellular response to transforming growth factor beta stimulus;GO:0000278|mitotic cell cycle;GO:0042981|regulation of apoptotic process;	GO:0016020|membrane;GO:0005634|nucleus;GO:0048471|perinuclear region of cytoplasm;GO:0030141|secretory granule;	GO:0005524|ATP binding;GO:0004702|receptor signaling protein	K04410	PAK2; p21-activated kinase 2 [EC:2.7.11.1]
ERO1B	132.0817	-0.50684	0.178980107	-2.83182	0.0046285	0.031082	Not_Sig	no	gi|803240777|ref|XP_011980485.1|	2418	0	PREDICTED: ERO1-like protein beta isoform X4 [Ovis aries musimon]	gi|926726016|ref|XM_013975536.1|	1994	0	PREDICTED: Capra hircus endoplasmic reticulum oxidoreductase beta (ERO1B), mRNA	Q86YB8	877	0	ERO1-like protein beta	SPCC1450.14c	224	1.09e-63	COG5061	pfam04137	ERO1	Endoplasmic Reticulum Oxidoreductin 1 (ERO1). Members of this family are required for the formation of disulphide bonds in	GO:0019471|4-hydroxyproline metabolic process;GO:0045454|cell redox homeostasis;GO:0006464|cellular protein modification process;GO:0030198|extracellular matrix organization;GO:0042593|glucose homeostasis;GO:0030070|insulin processing;GO:0006457|protein folding;GO:0034975|protein folding in endoplasmic reticulum;GO:0022417|protein maturation by protein folding;	GO:0005783|endoplasmic reticulum;GO:0005789|endoplasmic reticulum membrane;	GO:0016491|oxidoreductase activity;GO:0016671|oxidoreductase	K10976	ERO1LB; ERO1-like protein beta [EC:1.8.4.-]
TRAM1	2486.395	-0.31169	0.110104034	-2.83083	0.0046428	0.03116	Not_Sig	no	gi|742154119|ref|XP_010845276.1|	1664	0	PREDICTED: translocating chain-associated membrane protein 1 [Bison bison bison]	gi|982944809|ref|XM_005215508.3|	2702	0	PREDICTED: Bos taurus translocation associated membrane protein 1 (TRAM1), transcript variant X1, mRNA	Q9GKZ4	643	0	Translocating chain-associated membrane protein 1	.	.	.	.	pfam03798	TRAM_LAG1_CLN8	TLC domain.	GO:0015031|protein transport;	GO:0005789|endoplasmic reticulum membrane;GO:0016021|integral component of membrane;GO:0005791|rough endoplasmic	.	K14010	TRAM1; translocating chain-associated membrane protein 1
MMD	1990.185	-0.29826	0.105422137	-2.8292	0.0046664	0.0313	Not_Sig	no	gi|742138859|ref|XP_010841929.1|	1072	1e-129	PREDICTED: monocyte to macrophage differentiation factor isoform X2 [Bison bison bison]	gi|850486717|gb|CP011896.1|	4324	0	Ovis canadensis canadensis isolate 43U chromosome 11 sequence	Q15546	342	1e-104	Monocyte to macrophage differentiation factor	YPO0900	60.8	1.05e-08	COG1272	pfam03006	HlyIII	Haemolysin-III related. Members of this family are integral membrane proteins. This family includes a protein with hemolytic	GO:0019835|cytolysis;GO:0045666|positive regulation of neuron differentiation;GO:0045860|positive regulation of protein kinase activity;GO:0032880|regulation of protein localization;	GO:0005794|Golgi apparatus;GO:0005887|integral component of plasma membrane;GO:0031902|late endosome	GO:0004672|protein kinase activity;GO:0004872|receptor activity;	K11064	MMD; monocyte to macrophage differentiation protein
TFAM	424.1459	-0.44495	0.157310564	-2.82848	0.0046769	0.031352	Not_Sig	no	gi|555989640|ref|XP_005906647.1|	1228	3e-156	PREDICTED: transcription factor A, mitochondrial isoform X1 [Bos mutus]	gi|942087442|ref|XM_005906585.2|	3572	0	PREDICTED: Bos mutus transcription factor A, mitochondrial (TFAM), transcript variant X1, mRNA	Q0II87	474	1e-156	Transcription factor A, mitochondrial	.	.	.	.	pfam00505	HMG_box	HMG (high mobility group) box.	GO:0045893|positive regulation of transcription, DNA-templated;GO:0006390|transcription from mitochondrial promoter;GO:0006391|transcription initiation from mitochondrial promoter;	GO:0042645|mitochondrial nucleoid;	GO:0003682|chromatin binding;GO:0008301|DNA binding,	K11830	TFAM, MTTFA; transcription factor A, mitochondrial
RAB1A	3649.02	-0.32417	0.114635081	-2.82784	0.0046863	0.031387	Not_Sig	no	gi|440907831|gb|ELR57927.1|	1087	9e-144	Ras-related protein Rab-1A, partial [Bos mutus]	gi|942048848|ref|XM_005893555.2|	1454	0	PREDICTED: Bos mutus RAB1A, member RAS oncogene family (RAB1A), transcript variant X1, mRNA	Q6NYB7	419	2e-144	Ras-related protein Rab-1A	.	.	.	.	pfam00071	Ras	Ras family. Includes sub-families Ras, Rab, Rac, Ral, Ran, Rap Ypt1 and more. Shares P-loop motif with GTP_EFTU, arf and	GO:0000045|autophagosome assembly;GO:0006914|autophagy;GO:0016477|cell migration;GO:0042742|defense response to bacterium;GO:0006897|endocytosis;GO:0006888|ER to Golgi vesicle-mediated transport;GO:0030252|growth hormone secretion;GO:0072606|interleukin-8 secretion;GO:0032482|Rab protein signal transduction;GO:0047496|vesicle transport along microtubule;	GO:0005829|cytosol;GO:0005769|early endosome;GO:0005789|endoplasmic reticulum membrane;GO:0000139|Golgi membrane;GO:0042470|melanosome;GO:0	GO:0005525|GTP binding;GO:0003924|GTPase activity;	K07874	RAB1A; Ras-related protein Rab-1A

PABPC1	9032.92	0.278538	0.098501644	2.827752	0.0046876	0.031387	Not_Sig	no	gi|926708692|ref|XP_005689254.2|	3265	0	PREDICTED: polyadenylate-binding protein 1 isoform X1 [Capra hircus]	gi|75832066|ref|NM_174568.2|	2826	0	Bos taurus poly(A) binding protein, cytoplasmic 1 (PABPC1), mRNA	P11940	1229	0	Polyadenylate-binding protein 1	.	.	.	.	pfam00658	PABP	Poly-adenylate binding protein, unique domain. The region featured in this family is found towards the C-terminus of poly(A)-	GO:0031047|gene silencing by RNA;GO:0006378|mRNA polyadenylation;GO:0000398|mRNA splicing, via spliceosome;GO:0048255|mRNA stabilization;GO:2000623|negative regulation of nuclear-transcribed mRNA catabolic process, nonsense-mediated decay;GO:0000184|nuclear-transcribed mRNA catabolic process, nonsense-mediated decay;GO:0000289|nuclear-transcribed mRNA poly(A) tail shortening;GO:1900153|positive regulation of nuclear-transcribed mRNA catabolic process, deadenylation-dependent	GO:0071013|catalytic step 2 spliceosome;GO:0005737|cytoplasm;GO:0036464|cytoplasmic ribonucleoprotein granule;GO:0010494|cytoplasmic stress	GO:0003730|mRNA 3'-UTR binding;GO:0000166|nucleotide	K13126	PABPC; polyadenylate-binding protein
STBD1	585.8382	-0.33128	0.117216867	-2.82622	0.0047101	0.031511	Not_Sig	no	gi|119894119|ref|XP_591045.3|	1468	0	PREDICTED: starch-binding domain-containing protein 1 [Bos taurus]	gi|965814561|ref|XM_012122854.2|	3002	0	PREDICTED: Ovis aries musimon starch binding domain 1 (STBD1), mRNA	O95210	371	1e-115	Starch-binding domain-containing protein 1	.	.	.	.	pfam00686	CBM_20	Starch binding domain.	GO:0005980|glycogen catabolic process;GO:0061723|glycophagy;GO:0006936|muscle contraction;	GO:0005789|endoplasmic reticulum membrane;GO:0005887|integral component of plasma membrane;GO:0016020|membrane;GO:004	GO:2001069|glycogen binding;GO:2001070|starch binding;	.	.
RPL10	10618.7	-0.30194	0.10684323	-2.826	0.0047133	0.031511	Not_Sig	no	gi|946668211|ref|XP_014419151.1|	1187	6e-163	PREDICTED: 60S ribosomal protein L10 isoform X1 [Camelus ferus]	gi|942098250|ref|XM_014483087.1|	777	0	PREDICTED: Bos mutus ribosomal protein L10 (RPL10), transcript variant X2, mRNA	A8D8X1	445	5e-158	60S ribosomal protein L10	SPAP7G5.05	312	9.62e-108	COG0197	pfam00252	Ribosomal_L16	Ribosomal protein L16p/L10e.	GO:0006412|translation;	GO:0005840|ribosome;	GO:0003735|structural constituent of ribosome;	K02866	RP-L10e, RPL10; large subunit ribosomal protein L10e
PYGO1	267.5261	-0.43336	0.153351648	-2.82593	0.0047143	0.031511	Not_Sig	no	gi|297479662|ref|XP_002690938.1|	1992	0	PREDICTED: pygopus homolog 1 isoform X1 [Bos taurus]	gi|742104720|ref|XM_010836783.1|	8155	0	PREDICTED: Bison bison bison pygopus family PHD finger 1 (PYGO1), mRNA	Q9Y3Y4	725	0	Pygopus homolog 1	.	.	.	.	pfam09770	PAT1	Topoisomerase II-associated protein PAT1. Members of this family are necessary for accurate chromosome transmission during	GO:0002244|hematopoietic progenitor cell differentiation;GO:0001822|kidney development;GO:0045944|positive regulation of transcription from RNA polymerase II promoter;GO:0009791|post-embryonic development;GO:0034504|protein localization to nucleus;GO:0007289|spermatid nucleus differentiation;GO:0016055|Wnt signaling pathway;	GO:0005654|nucleoplasm;	GO:0008270|zinc ion binding;	.	.
NAA80	106.3005	0.646458	0.228781433	2.825659	0.0047183	0.03152	UP	yes	gi|965990116|ref|XP_014957894.1|	1307	1e-176	PREDICTED: LOW QUALITY PROTEIN: N-acetyltransferase 6 isoform X1 [Ovis aries]	gi|594058394|ref|XM_006080881.1|	1183	0	PREDICTED: Bubalus bubalis N-acetyltransferase 6 (GCN5-related) (NAT6), mRNA	Q9R123	360	9e-121	N-acetyltransferase 6	.	.	.	.	pfam00583	Acetyltransf_1	Acetyltransferase (GNAT) family. This family contains proteins with N-acetyltransferase functions such as Elp3-related proteins.	.	GO:0005737|cytoplasm;	GO:0008080|N-acetyltransferase activity;	.	.
EPS15L1	510.7539	0.351138	0.124368464	2.823372	0.0047521	0.031727	Not_Sig	no	gi|742149200|ref|XP_010843897.1|	3809	0	PREDICTED: epidermal growth factor receptor substrate 15-like 1 isoform X2 [Bison bison bison]	gi|742149201|ref|XM_010845596.1|	2895	0	PREDICTED: Bison bison bison epidermal growth factor receptor pathway substrate 15-like 1 (EPS15L1), transcript variant X3, mRNA	Q9UBC2	1293	0	Epidermal growth factor receptor substrate 15-like 1	.	.	.	.	pfam12763	EF-hand_4	Cytoskeletal-regulatory complex EF hand. This is an efhand family from the N-terminal of actin cytoskeleton-regulatory complex	GO:0006897|endocytosis;GO:0042059|negative regulation of epidermal growth factor receptor signaling pathway;	GO:0005913|cell-cell adherens junction;GO:0030132|clathrin coat of coated pit;GO:0016020|membrane;GO:0005634|n	GO:0098641|cadherin binding involved in cell-cell adhesion;GO:00055	K12472	EPS15; epidermal growth factor receptor substrate 15
CERK	723.5007	0.368746	0.130667926	2.822012	0.0047723	0.031843	Not_Sig	no	gi|926730939|ref|XP_013832739.1|	2439	0	PREDICTED: ceramide kinase [Capra hircus]	gi|942060703|ref|XM_005898155.2|	2011	0	PREDICTED: Bos mutus ceramide kinase (CERK), mRNA	Q8TCT0	853	0	Ceramide kinase	SPAC4A8.07c	102	5.89e-22	COG1597	pfam00781	DAGK_cat	Diacylglycerol kinase catalytic domain. Diacylglycerol (DAG) is a second messenger that acts as a protein kinase C activator. The	GO:0006672|ceramide metabolic process;GO:0006687|glycosphingolipid metabolic process;	GO:0016021|integral component of membrane;GO:0005739|mitochondrion;GO:0005886|plasma membrane;	GO:0005524|ATP binding;GO:0001729|ceramide kinase activity;GO:0000287	K04715	E2.7.1.138, CERK; ceramide kinase [EC:2.7.1.138]
OSR2	242.3946	0.559325	0.198215701	2.821801	0.0047755	0.031846	Not_Sig	no	gi|859875901|ref|XP_012920244.1|	1741	0	PREDICTED: protein odd-skipped-related 2 isoform X1 [Mustela putorius furo]	gi|982945036|ref|XM_005215616.3|	1842	0	PREDICTED: Bos taurus odd-skipped related transciption factor 2 (OSR2), transcript variant X1, mRNA	Q8N2R0	639	0	Protein odd-skipped-related 2	.	.	.	.	pfam13465	zf-H2C2_2	Zinc-finger double domain.	GO:0060349|bone morphogenesis;GO:0030154|cell differentiation;GO:0002062|chondrocyte differentiation;GO:0009790|embryo development;GO:0042733|embryonic digit morphogenesis;GO:0035115|embryonic forelimb morphogenesis;GO:0035116|embryonic hindlimb morphogenesis;GO:0072498|embryonic skeletal joint development;GO:0060272|embryonic skeletal joint morphogenesis;GO:0036023|embryonic skeletal limb joint morphogenesis;GO:0048704|embryonic skeletal system	GO:0005634|nucleus;	GO:0046872|metal ion binding;GO:0000977|RNA polymerase II	K09215	OSR, ODD; odd-skipped
CLIP2	770.1449	0.342192	0.121308161	2.820851	0.0047897	0.031922	Not_Sig	no	gi|594066320|ref|XP_006057135.1|	4767	0	PREDICTED: CAP-Gly domain-containing linker protein 2 isoform X3 [Bubalus bubalis]	gi|982967188|ref|XM_005225046.3|	5396	0	PREDICTED: Bos taurus CAP-Gly domain containing linker protein 2 (CLIP2), transcript variant X1, mRNA	Q9UDT6	1695	0	CAP-Gly domain-containing linker protein 2	ECU06g0410	72	9.12e-13	COG5244	pfam01302	CAP_GLY	CAP-Gly domain. Cytoskeleton-associated proteins (CAPs) are involved in the organisation of microtubules and transportation of	.	GO:0005881|cytoplasmic microtubule;GO:0005875|microtubule associated complex;GO:0035371|microtubule plus-	.	K10422	CLIP2, CYLN2; CAP-Gly domain-containing linker protein 2
DNAJC11	893.6568	0.316549	0.112236343	2.820381	0.0047967	0.03195	Not_Sig	no	gi|114050887|ref|NP_001039458.1|	2867	0	dnaJ homolog subfamily C member 11 [Bos taurus]	gi|114050886|ref|NM_001045993.1|	2922	0	Bos taurus DnaJ heat shock protein family (Hsp40) member C11 (DNAJC11), mRNA	Q2NL21	1087	0	DnaJ homolog subfamily C member 11	.	.	.	.	pfam11875	DUF3395	Domain of unknown function (DUF3395). This domain is functionally uncharacterized. This domain is found in eukaryotes.	.	GO:0070062|extracellular exosome;GO:0005743|mitochondrial inner membrane;	.	K09531	DNAJC11; DnaJ homolog subfamily C member 11
CHAF1A	438.3253	0.376936	0.1337066	2.819126	0.0048155	0.032057	Not_Sig	no	gi|156120333|ref|NP_001095312.1|	2158	0	chromatin assembly factor 1 subunit A [Bos taurus]	gi|982930820|ref|XM_010806967.2|	3428	0	PREDICTED: Bos taurus chromatin assembly factor 1 subunit A (CHAF1A), transcript variant X1, mRNA	A6QLA6	835	0	Chromatin assembly factor 1 subunit A	.	.	.	.	pfam15539	CAF1-p150_C2	CAF1 complex subunit p150, region binding to CAF1-p60 at C-term. CAF1-p150_C2 is part of the binding region of the CAF1	GO:0007049|cell cycle;GO:0031497|chromatin assembly;GO:0006281|DNA repair;GO:0006260|DNA replication;GO:0006335|DNA replication-dependent nucleosome assembly;GO:0006334|nucleosome assembly;	GO:0033186|CAF-1 complex;GO:0000790|nuclear chromatin;GO:0005730|nucleolus;GO:0005634|nucleus;	.	K10750	CHAF1A; chromatin assembly factor 1 subunit A
HUWE1	3257.914	0.291083	0.103261547	2.818894	0.004819	0.032061	Not_Sig	no	gi|741983128|ref|XP_010820301.1|	11828	0	PREDICTED: E3 ubiquitin-protein ligase HUWE1 isoform X4 [Bos taurus]	gi|982974172|ref|XM_015461710.1|	14303	0	PREDICTED: Bos taurus HECT, UBA and WWE domain containing 1, E3 ubiquitin protein ligase (HUWE1), transcript variant X3, mRNA	Q7Z6Z7	4434	0	E3 ubiquitin-protein ligase HUWE1	SPAC19D5.04	453	1.78e-126	COG5021	pfam06025	DUF913	Domain of Unknown Function (DUF913). Members of this family are found in various ubiquitin protein ligases.	GO:0006284|base-excision repair;GO:0030154|cell differentiation;GO:0016574|histone ubiquitination;GO:1903955|positive regulation of protein targeting to mitochondrion;GO:0006513|protein monoubiquitination;GO:0000209|protein polyubiquitination;GO:0042787|protein ubiquitination involved in ubiquitin-dependent protein catabolic process;	GO:0005737|cytoplasm;GO:0005829|cytosol;GO:0070062|extracellular exosome;GO:0016020|membrane;GO:0005654|nucleoplasm;GO:0005634|nucleus;	GO:0003677|DNA binding;GO:0044822|poly(A) RNA binding;GO:0004842	K10592	HUWE1, MULE, ARF-BP1; E3 ubiquitin-protein ligase HUWE1 [EC:2.3.2.26]
SHROOM3	957.7425	0.348032	0.123630882	2.815087	0.0048764	0.032425	Not_Sig	no	gi|555974445|ref|XP_005899215.1|	9471	0	PREDICTED: protein Shroom3 [Bos mutus]	gi|942064498|ref|XM_005899153.2|	6563	0	PREDICTED: Bos mutus shroom family member 3 (SHROOM3), mRNA	Q8TF72	2479	0	Protein Shroom3	.	.	.	.	pfam08687	ASD2	Apx/Shroom domain ASD2. This region is found in the actin binding protein Shroom which mediates apical contriction in epithelial cells	GO:0045176|apical protein localization;GO:0000902|cell morphogenesis;GO:0043482|cellular pigment accumulation;GO:0002064|epithelial cell development;GO:0001843|neural tube closure;GO:0007389|pattern specification process;GO:0008360|regulation of cell shape;	GO:0005912|adherens junction;GO:0043296|apical junction complex;GO:0016324|apical plasma membrane;GO:0005737|cytoplasm;GO:000	.	K18625	SHROOM; protein Shroom
G3BP2	1845.883	-0.28624	0.101687323	-2.81488	0.0048795	0.032426	Not_Sig	no	gi|821000040|ref|XP_003265810.2|	1649	0	PREDICTED: ras GTPase-activating protein-binding protein 2 isoform X1 [Nomascus leucogenys]	gi|942095409|ref|XM_005909115.2|	4368	0	PREDICTED: Bos mutus GTPase activating protein (SH3 domain) binding protein 2 (G3BP2), transcript variant X1, mRNA	Q5R9L3	593	0	Ras GTPase-activating protein-binding protein 2	.	.	.	.	pfam02136	NTF2	Nuclear transport factor 2 (NTF2) domain. This family includes the NTF2-like Delta-5-3-ketosteroid isomerase proteins.	GO:0051028|mRNA transport;	GO:0005737|cytoplasm;	GO:0000166|nucleotide binding;GO:0044822|poly(A) RNA	.	.
CHMP4A	191.8891	-0.43547	0.154727642	-2.81441	0.0048868	0.032456	Not_Sig	no	gi|190360723|ref|NP_001121976.1|	695	6e-87	charged multivesicular body protein 4a [Bos taurus]	gi|942082207|ref|XM_005904916.2|	1036	0	PREDICTED: Bos mutus charged multivesicular body protein 4A (CHMP4A), mRNA	A2VDY3	272	2e-88	Charged multivesicular body protein 4a	.	.	.	.	pfam03357	Snf7	Snf7. This family of proteins are involved in protein sorting and transport from the endosome to the vacuole/lysosome in	GO:0000920|cell separation after cytokinesis;GO:0010324|membrane invagination;GO:0097320|membrane tubulation;GO:0007080|mitotic metaphase plate congression;GO:1902902|negative regulation of autophagosome assembly;GO:1901215|negative regulation of neuron death;GO:0006997|nucleus organization;GO:0006620|posttranslational protein targeting to membrane;GO:0051260|protein homooligomerization;GO:0051258|protein polymerization;GO:0007034|vacuolar transport;GO:0039702|viral budding via host ESCRT	GO:0009898|cytoplasmic side of plasma membrane;GO:0030659|cytoplasmic vesicle membrane;GO:0000815|ESCRT III complex;GO:0070062|extracellular	GO:0008289|lipid binding;	K12194	CHMP4, SNF7, VPS32; charged multivesicular body protein 4
SLC35A5	861.4703	-0.30459	0.10824178	-2.81395	0.0048936	0.032483	Not_Sig	no	gi|742174494|ref|XP_010851053.1|	2183	0	PREDICTED: probable UDP-sugar transporter protein SLC35A5 [Bison bison bison]	gi|942077339|ref|XM_005903364.2|	3094	0	PREDICTED: Bos mutus solute carrier family 35, member A5 (SLC35A5), mRNA	A6QPI1	838	0	Probable UDP-sugar transporter protein SLC35A5	SPCC1795.03	81.3	1.67e-15	COG0697	pfam04142	Nuc_sug_transp	Nucleotide-sugar transporter. This family of membrane proteins transport nucleotide sugars from the cytoplasm into golgi vesicles.	.	GO:0000139|Golgi membrane;GO:0016021|integral component of membrane;	GO:0005351|sugar: proton symporter activity;	K15274	SLC35A5; solute carrier family 35 (probable UDP-sugar transporter),
USP5	1170.79	0.348063	0.123713811	2.813451	0.0049013	0.032515	Not_Sig	no	gi|556724291|ref|XP_005957950.1|	4336	0	PREDICTED: ubiquitin carboxyl-terminal hydrolase 5 isoform X1 [Pantholops hodgsonii]	gi|300796696|ref|NM_001192056.1|	3182	0	Bos taurus ubiquitin specific peptidase 5 (isopeptidase T) (USP5), mRNA	P56399	1647	0	Ubiquitin carboxyl-terminal hydrolase 5	SPBC6B1.06c	397	2.75e-123	COG5207	pfam00443	UCH	Ubiquitin carboxyl-terminal hydrolase.	GO:0032436|positive regulation of proteasomal ubiquitin-dependent protein catabolic process;GO:0016579|protein deubiquitination;GO:0071108|protein K48-linked deubiquitination;GO:0006511|ubiquitin-dependent protein catabolic process;	.	GO:0004843|ubiquitin-specific protease activity;GO:0043130|ubiquitin	K11836	USP5_13, UBP14; ubiquitin carboxyl-terminal hydrolase 5/13 [EC:3.4.19.12]
SPTLC1	1297.077	-0.30578	0.108703381	-2.81295	0.004909	0.032547	Not_Sig	no	gi|77736443|ref|NP_001029921.1|	2150	0	serine palmitoyltransferase 1 [Bos taurus]	gi|742101377|ref|XM_010835958.1|	2684	0	PREDICTED: Bison bison bison serine palmitoyltransferase, long chain base subunit 1 (SPTLC1), mRNA	Q3MHG1	832	0	Serine palmitoyltransferase 1	SPBC18E5.02c	291	5.08e-88	COG0156	pfam00155	Aminotran_1_2	Aminotransferase class I and II.	GO:0046513|ceramide biosynthetic process;GO:1904504|positive regulation of lipophagy;GO:0046511|sphinganine biosynthetic process;GO:0006686|sphingomyelin biosynthetic process;GO:0046512|sphingosine biosynthetic process;	GO:0016021|integral component of membrane;GO:0035339|SPOTS complex;	GO:0030170|pyridoxal phosphate binding;GO:0004758|serine C-	K00654	SPT; serine palmitoyltransferase [EC:2.3.1.50]
PRR11	383.014	0.366536	0.130318141	2.812626	0.0049139	0.032554	Not_Sig	no	gi|555981859|ref|XP_005902842.1|	1308	2e-164	PREDICTED: proline-rich protein 11 [Bos mutus]	gi|742209544|ref|XM_010861621.1|	2088	0	PREDICTED: Bison bison bison proline rich 11 (PRR11), transcript variant X1, mRNA	Q96HE9	234	3e-66	Proline-rich protein 11	.	.	.	.	pfam08496	Peptidase_S49_N	Peptidase family S49 N-terminal. This domain is found to the N-terminus of bacterial signal peptidases of the S49 family	GO:0051726|regulation of cell cycle;	GO:0005737|cytoplasm;GO:0016020|membrane;GO:0005634|nucleus;	.	.	.
URI1	450.1793	-0.36133	0.128473973	-2.81246	0.0049164	0.032554	Not_Sig	no	gi|440902962|gb|ELR53687.1|	2489	0	Unconventional prefoldin RPB5 interactor, partial [Bos mutus]	gi|594073341|ref|XM_006060428.1|	3288	0	PREDICTED: Bubalus bubalis unconventional prefoldin RPB5 interactor-like (LOC102389550), mRNA	Q3B7M7	955	0	Unconventional prefoldin RPB5 interactor	.	.	.	.	pfam02996	Prefoldin	Prefoldin subunit. This family comprises of several prefoldin subunits. The biogenesis of the cytoskeletal proteins actin and	GO:0071363|cellular response to growth factor stimulus;GO:0071383|cellular response to steroid hormone stimulus;GO:2001243|negative regulation of intrinsic apoptotic signaling pathway;GO:0010923|negative regulation of phosphatase activity;GO:0000122|negative regulation of transcription from RNA polymerase II promoter;GO:0001558|regulation of cell growth;GO:0006357|regulation of transcription from RNA polymerase II promoter;GO:0009615|response to virus;GO:0006351|transcription, DNA-templated;	GO:0005737|cytoplasm;GO:0030425|dendrite;GO:0005665|DNA-directed RNA polymerase II, core complex;GO:0005739|mitochondrion;GO:00	GO:0003682|chromatin binding;GO:0019212|phosphatase	K17560	URI1; unconventional prefoldin RPB5 interactor 1
UBE2K	941.2733	-0.38611	0.137294413	-2.8123	0.0049188	0.032554	Not_Sig	no	gi|511888671|ref|XP_004764077.1|	1041	2e-130	PREDICTED: ubiquitin-conjugating enzyme E2 K [Mustela putorius furo]	gi|965941890|ref|XM_012180482.2|	2739	0	PREDICTED: Ovis aries ubiquitin conjugating enzyme E2K (UBE2K), transcript variant X1, mRNA	P61087	389	7e-127	Ubiquitin-conjugating enzyme E2 K	YDR177w	146	4.40e-39	COG5078	pfam00179	UQ_con	Ubiquitin-conjugating enzyme. Proteins destined for proteasome-mediated degradation may be ubiquitinated. Ubiquitination	GO:0035458|cellular response to interferon-beta;GO:0010994|free ubiquitin chain polymerization;GO:0070059|intrinsic apoptotic signaling pathway in response to endoplasmic reticulum stress;GO:0010800|positive regulation of peptidyl-threonine phosphorylation;GO:0060340|positive regulation of type I interferon-mediated signaling pathway;GO:0070936|protein K48-linked ubiquitination;GO:0000209|protein polyubiquitination;GO:0032434|regulation of proteasomal ubiquitin-dependent protein catabolic process;	GO:0005737|cytoplasm;GO:0070062|extracellular exosome;GO:0032433|filopodium tip;GO:0005634|nucleus;	GO:0005524|ATP binding;GO:0061631|ubiquitin conjugating enzyme	K04649	HIP2, UBC1; ubiquitin-conjugating enzyme (huntingtin
RGP1	1019.43	0.363642	0.129311568	2.812136	0.0049214	0.032554	Not_Sig	no	gi|440901934|gb|ELR52794.1|	1691	0	Retrograde Golgi transport protein RGP1-like protein, partial [Bos mutus]	gi|942069559|ref|XM_005900776.2|	3256	0	PREDICTED: Bos mutus RGP1 homolog, RAB6A GEF complex partner 1 (RGP1), mRNA	Q92546	635	0	RAB6A-GEF complex partner protein 2	.	.	.	.	pfam08737	Rgp1	Rgp1. Rgp1 forms heterodimer with Ric1 (pfam07064) which associates with Golgi membranes and functions as a guanyl-	GO:1903363|negative regulation of cellular protein catabolic process;GO:0043547|positive regulation of GTPase activity;GO:0042147|retrograde transport, endosome to Golgi;	GO:0005829|cytosol;GO:0000139|Golgi membrane;GO:0016020|membrane;GO:0043234|protein complex;GO:0034066|RIC1-RGP1 guanyl-nucleotide exchange factor	GO:0017137|Rab GTPase binding;GO:0017112|Rab guanyl-	.	.
DRG2	496.9796	0.340361	0.121084283	2.810944	0.0049396	0.032656	Not_Sig	no	gi|62460422|ref|NP_001014865.1|	1842	0	developmentally-regulated GTP-binding protein 2 [Bos taurus]	gi|742136509|ref|XM_010842903.1|	1821	0	PREDICTED: Bison bison bison developmentally regulated GTP binding protein 2 (DRG2), mRNA	Q58D56	714	0	Developmentally-regulated GTP-binding protein 2	SPBC354.01	461	6.04e-159	COG1163	pfam01926	MMR_HSR1	50S ribosome-binding GTPase. The full-length GTPase protein is required for the complete activity of the protein of interacting with	.	GO:0016020|membrane;GO:0005739|mitochondrion;GO:0005654|nucleoplasm;	GO:0005525|GTP binding;	K06944	K06944; uncharacterized protein
FRG1	292.0777	-0.39255	0.139678997	-2.8104	0.004948	0.032692	Not_Sig	no	gi|555950047|ref|XP_005887228.1|	1241	4e-169	PREDICTED: protein FRG1 isoform X1 [Bos mutus]	gi|942030545|ref|XM_005887166.2|	1031	0	PREDICTED: Bos mutus FSHD region gene 1 (FRG1), transcript variant X1, mRNA	Q14331	473	9e-167	Protein FRG1	.	.	.	.	pfam06229	FRG1	FRG1-like family. The human FRG1 gene maps to human chromosome 4q35 and has been identified as a candidate for facioscapulohumeral	GO:0000398|mRNA splicing, via spliceosome;GO:0007517|muscle organ development;GO:0006364|rRNA processing;	GO:0015030|Cajal body;GO:0071013|catalytic step 2 spliceosome;GO:0005730|nucleolus;GO:0005120|striated muscle dense	GO:0051015|actin filament binding;GO:0044822|poly(A) RNA	K13122	FRG1; protein FRG1
PATL1	802.5527	0.357208	0.12711582	2.810102	0.0049526	0.032704	Not_Sig	no	gi|300794107|ref|NP_001180136.1|	3110	0	protein PAT1 homolog 1 [Bos taurus]	gi|965979486|ref|XM_012096472.2|	2872	0	PREDICTED: Ovis aries protein associated with topoisomerase II homolog 1 (yeast) (PATL1), transcript variant X1, mRNA	Q5R8Q4	1161	0	Protein PAT1 homolog 1	.	.	.	.	pfam09770	PAT1	Topoisomerase II-associated protein PAT1. Members of this family are necessary for accurate chromosome transmission during	GO:0033962|cytoplasmic mRNA processing body assembly;GO:0000290|deadenylation-dependent decapping of nuclear-transcribed mRNA;	GO:0000932|cytoplasmic mRNA processing body;GO:0016607|nuclear speck;GO:0016605|PML body;	GO:0003723|RNA binding;	K12617	PATL1, PAT1; DNA topoisomerase 2-associated protein PAT1
KLF9	1109.077	-0.32509	0.115697677	-2.8098	0.0049572	0.032716	Not_Sig	no	gi|58332856|ref|NP_001011504.1|	1208	2e-150	Krueppel-like factor 9 [Sus scrofa]	gi|942059069|ref|XM_005897474.2|	4805	0	PREDICTED: Bos mutus Kruppel-like factor 9 (KLF9), mRNA	P79288	469	7e-152	Krueppel-like factor 9	.	.	.	.	pfam13465	zf-H2C2_2	Zinc-finger double domain.	GO:0071387|cellular response to cortisol stimulus;GO:0097067|cellular response to thyroid hormone stimulus;GO:0007623|circadian rhythm;GO:0010839|negative regulation of keratinocyte proliferation;GO:0006355|regulation of transcription, DNA-templated;GO:0006351|transcription, DNA-templated;	GO:0005737|cytoplasm;GO:0005654|nucleoplasm;GO:0005634|nucleus;GO:0005886|plasma membrane;	GO:0003677|DNA binding;GO:0046872|metal ion binding;	K09208	KLF9S, BTEB; krueppel-like factor 9/13/14/16
TBC1D1	2222.109	0.288195	0.102585113	2.809325	0.0049645	0.032745	Not_Sig	no	gi|742173328|ref|XP_010850717.1|	6229	0	PREDICTED: TBC1 domain family member 1 isoform X1 [Bison bison bison]	gi|742173327|ref|XM_010852415.1|	5642	0	PREDICTED: Bison bison bison TBC1 (tre-2/USP6, BUB2, cdc16) domain family, member 1 (TBC1D1), transcript variant X1, mRNA	Q60949	2093	0	TBC1 domain family member 1	.	.	.	.	pfam00566	RabGAP-TBC	Rab-GTPase-TBC domain. Identification of a TBC domain in GYP6_YEAST and GYP7_YEAST, which are GTPase activator	GO:0090630|activation of GTPase activity;GO:0006886|intracellular protein transport;GO:0061024|membrane organization;GO:0032880|regulation of protein localization;GO:0031338|regulation of vesicle fusion;	GO:0005829|cytosol;GO:0012505|endomembrane system;GO:0005622|intracellular;GO:0005634|nucleus;	GO:0005096|GTPase activator activity;GO:0017137|Rab GTPase	K18341	TBC1D1; TBC1 domain family member 1
GNB2	3302.84	0.331179	0.117902221	2.80893	0.0049707	0.032767	Not_Sig	no	gi|13937391|ref|NP_034442.1|	1720	0	guanine nucleotide-binding protein G(I)/G(S)/G(T) subunit beta-2 [Mus musculus]	gi|594044045|ref|XM_006046465.1|	1584	0	PREDICTED: Bubalus bubalis guanine nucleotide binding protein (G protein), beta polypeptide 2 (GNB2), mRNA	P54313	667	0	Guanine nucleotide-binding protein G(I)/G(S)/G(T) subunit beta-2	.	.	.	.	pfam00400	WD40	WD domain, G-beta repeat.	.	GO:0044297|cell body;GO:0070062|extracellular exosome;GO:0005615|extracellular space;GO:0005925|focal	GO:0005246|calcium channel regulator activity;GO:0004871|signal transducer	K04537	GNB2; guanine nucleotide-binding protein G(I)/G(S)/G(T)
STRADA	326.1192	0.375464	0.133776761	2.806644	0.0050061	0.032975	Not_Sig	no	gi|528996438|ref|XP_005220922.1|	1713	0	PREDICTED: STE20-related kinase adapter protein alpha isoform X3 [Bos taurus]	gi|742188292|ref|XM_010856435.1|	2302	0	PREDICTED: Bison bison bison STE20-related kinase adaptor alpha (STRADA), transcript variant X3, mRNA	Q5E9J9	661	0	STE20-related kinase adapter protein alpha	.	.	.	.	pfam00069	Pkinase	Protein kinase domain.	GO:0032147|activation of protein kinase activity;GO:0007049|cell cycle;GO:0006611|protein export from nucleus;	GO:0005737|cytoplasm;GO:0005634|nucleus;	GO:0005524|ATP binding;GO:0030295|protein kinase activator	K08271	STRADA, LYK5; STE20-related kinase adapter protein alpha
TOLLIP	1635.13	0.34098	0.121495347	2.806526	0.0050079	0.032975	Not_Sig	no	gi|122136310|sp|Q2LGB5.1|TOLIP_BOVIN	1113	1e-141	RecName: Full=Toll-interacting protein	gi|90403623|ref|NM_001039961.1|	2573	0	Bos taurus toll interacting protein (TOLLIP), mRNA	Q2LGB5	433	4e-143	Toll-interacting protein	.	.	.	.	pfam02845	CUE	CUE domain. CUE domains have been shown to bind ubiquitin. It has been suggested that CUE domains are related to pfam00627	GO:0006914|autophagy;GO:0030855|epithelial cell differentiation;GO:0006954|inflammatory response;GO:0045087|innate immune response;GO:0016310|phosphorylation;GO:0036010|protein localization to endosome;GO:0007165|signal transduction;	GO:0005737|cytoplasm;GO:0070062|extracellular exosome;	.	K05402	TOLLIP; toll-interacting protein
NADK2	188.6774	-0.51227	0.182577255	-2.80577	0.0050197	0.033018	Not_Sig	no	gi|594103877|ref|XP_006074937.1|	2209	0	PREDICTED: NAD kinase 2, mitochondrial isoform X1 [Bubalus bubalis]	gi|942093509|ref|XM_014482428.1|	2477	0	PREDICTED: Bos mutus NAD kinase 2, mitochondrial (NADK2), transcript variant X2, mRNA	Q8C5H8	796	0	NAD kinase 2, mitochondrial	.	.	.	.	pfam01513	NAD_kinase	ATP-NAD kinase. Members of this family include ATP-NAD kinases EC:2.7.1.23, which catalyses the phosphorylation of NAD to NADP	GO:0019674|NAD metabolic process;GO:0006741|NADP biosynthetic process;	GO:0005739|mitochondrion;	GO:0005524|ATP binding;GO:0003951|NAD+ kinase activity;GO:0042803	K00858	ppnK, NADK; NAD+ kinase [EC:2.7.1.23]
TMEM50B	559.8042	-0.39765	0.141727275	-2.80574	0.0050202	0.033018	Not_Sig	no	gi|8659559|ref|NP_006125.2|	836	5e-104	transmembrane protein 50B [Homo sapiens]	gi|742212176|ref|XM_010862286.1|	2229	0	PREDICTED: Bison bison bison transmembrane protein 50B (TMEM50B), transcript variant X4, mRNA	Q5R4C3	326	2e-105	Transmembrane protein 50B	.	.	.	.	pfam05255	UPF0220	Uncharacterized protein family (UPF0220). This family of proteins is functionally uncharacterized.	.	GO:0016021|integral component of membrane;	.	.	.
SLC7A2	282.2306	-0.41112	0.146550092	-2.80534	0.0050264	0.03304	Not_Sig	no	gi|594115005|ref|XP_006080214.1|	3298	0	PREDICTED: low affinity cationic amino acid transporter 2 isoform X1 [Bubalus bubalis]	gi|983008536|ref|XM_015469731.1|	4998	0	PREDICTED: Bos taurus solute carrier family 7 (cationic amino acid transporter, y+ system), member 2 (SLC7A2), transcript variant X1, mRNA	P52569	1164	0	Cationic amino acid transporter 2	XF2207	247	2.87e-70	COG0531	pfam13520	AA_permease_2	Amino acid permease.	GO:0006865|amino acid transport;GO:0015809|arginine transport;GO:0006520|cellular amino acid metabolic process;GO:0097638|L-arginine import across plasma membrane;GO:0097639|L-lysine import across plasma membrane;GO:0097640|L-ornithine import across plasma membrane;GO:0042116|macrophage activation;GO:0006809|nitric oxide biosynthetic process;GO:0002537|nitric oxide production involved in inflammatory response;GO:0050727|regulation of inflammatory response;GO:0043030|regulation of macrophage	GO:0005887|integral component of plasma membrane;GO:0016020|membrane;GO:0005886|plasma membrane;	GO:0015171|amino acid transmembrane transporter activity;GO:0015297	K13864	SLC7A2, ATRC2; solute carrier family 7 (cationic amino acid transporter),
CEP95	469.654	-0.37993	0.135461771	-2.80468	0.0050366	0.033082	Not_Sig	no	gi|742188173|ref|XP_010854708.1|	4596	0	PREDICTED: centrosomal protein of 95 kDa [Bison bison bison]	gi|742188172|ref|XM_010856406.1|	2858	0	PREDICTED: Bison bison bison centrosomal protein 95kDa (CEP95), transcript variant X1, mRNA	Q96GE4	1296	0	Centrosomal protein of 95 kDa	.	.	.	.	pfam12128	DUF3584	Protein of unknown function (DUF3584). This protein is found in bacteria and eukaryotes. Proteins in this family are typically between	.	GO:0005813|centrosome;GO:0005737|cytoplasm;GO:0000922|spindle pole;	.	K16544	CEP95; centrosomal protein CEP95
DAB2	1774.132	0.335857	0.119754023	2.804556	0.0050386	0.033082	Not_Sig	no	gi|555955227|ref|XP_005889772.1|	3334	0	PREDICTED: disabled homolog 2 [Bos mutus]	gi|555955226|ref|XM_005889710.1|	3276	0	PREDICTED: Bos mutus Dab, mitogen-responsive phosphoprotein, homolog 2 (Drosophila) (DAB2), mRNA	P98082	1092	0	Disabled homolog 2	.	.	.	.	pfam00640	PID	Phosphotyrosine interaction domain (PTB/PID).	GO:0006915|apoptotic process;GO:0008283|cell proliferation;GO:0035026|leading edge cell differentiation;GO:0007275|multicellular organismal development;GO:0060766|negative regulation of androgen receptor signaling pathway;GO:0043066|negative regulation of apoptotic process;GO:0090090|negative regulation of canonical Wnt signaling pathway;GO:0032091|negative regulation of protein binding;GO:1903077|negative regulation of protein localization to plasma membrane;GO:0045892|negative regulation of transcription, DNA-	GO:0005905|coated pit;GO:0030136|clathrin-coated vesicle;GO:0030665|clathrin-coated vesicle membrane;GO:0070062|extracellular	GO:0038024|cargo receptor activity;GO:0035615|clathrin adaptor	K12475	DAB2; disabled homolog 2
EIF3M	1679.454	-0.29235	0.104270234	-2.8038	0.0050504	0.033129	Not_Sig	no	gi|23397429|ref|NP_006351.2|	1885	0	eukaryotic translation initiation factor 3 subunit M isoform 1 [Homo sapiens]	gi|942041423|ref|XM_005890873.2|	1199	0	PREDICTED: Bos mutus eukaryotic translation initiation factor 3, subunit M (EIF3M), transcript variant X1, mRNA	Q7L2H7	730	0	Eukaryotic translation initiation factor 3 subunit M	.	.	.	.	pfam01399	PCI	PCI domain. This domain has also been called the PINT motif (Proteasome, Int-6, Nip-1 and TRIP-15).	GO:0002183|cytoplasmic translational initiation;GO:0001731|formation of translation preinitiation complex;GO:0006446|regulation of translational initiation;GO:0006413|translational initiation;	GO:0005829|cytosol;GO:0016282|eukaryotic 43S preinitiation complex;GO:0033290|eukaryotic 48S preinitiation	GO:0003743|translation initiation factor activity;	K15030	EIF3M; translation initiation factor 3 subunit M
RPS20	5694.867	-0.38746	0.138195928	-2.80373	0.0050515	0.033129	Not_Sig	no	gi|4506697|ref|NP_001014.1|	613	1e-78	40S ribosomal protein S20 isoform 2 [Homo sapiens]	gi|742114268|ref|XM_010838970.1|	554	0	PREDICTED: Bison bison bison ribosomal protein S20 (RPS20), mRNA	P60868	240	3e-80	40S ribosomal protein S20	SPCC576.09	171	5.27e-55	COG0051	pfam00338	Ribosomal_S10	Ribosomal protein S10p/S20e. This family includes small ribosomal subunit S10 from prokaryotes and S20 from eukaryotes.	GO:0006412|translation;	GO:0022627|cytosolic small ribosomal subunit;	GO:0003723|RNA binding;GO:0003735|structural constituent of	K02969	RP-S20e, RPS20; small subunit ribosomal protein S20e
PRDX4	3642.959	-0.31218	0.111372967	-2.80303	0.0050625	0.033182	Not_Sig	no	gi|594098157|ref|XP_006072251.1|	1226	2e-166	PREDICTED: peroxiredoxin-4 isoform X3 [Bubalus bubalis]	gi|982974539|ref|XM_005228351.2|	1030	0	PREDICTED: Bos taurus peroxiredoxin 4 (PRDX4), transcript variant X1, mRNA	Q9BGI2	465	1e-163	Peroxiredoxin-4	SPCC576.03c	253	1.91e-83	COG0450	pfam00578	AhpC-TSA	AhpC/TSA family. This family contains proteins related to alkyl hydroperoxide reductase (AhpC) and thiol specific antioxidant (TSA).	GO:0019471|4-hydroxyproline metabolic process;GO:0045454|cell redox homeostasis;GO:0030198|extracellular matrix organization;GO:0008584|male gonad development;GO:2000255|negative regulation of male germ cell proliferation;GO:0022417|protein maturation by protein folding;GO:0072593|reactive oxygen species metabolic process;GO:0007283|spermatogenesis;	GO:0005829|cytosol;GO:0005783|endoplasmic reticulum;GO:0070062|extracellular exosome;GO:0005739|mitochondrion;GO:0005634|nucleus;	GO:0004601|peroxidase activity;GO:0051920|peroxiredoxin	K03386	E1.11.1.15, PRDX, ahpC; peroxiredoxin (alkyl hydroperoxide reductase subunit C)
DBN1	2507.729	0.276609	0.09869769	2.802586	0.0050695	0.033209	Not_Sig	no	gi|114052300|ref|NP_001039382.1|	2503	0	drebrin [Bos taurus]	gi|742152555|ref|XM_010846547.1|	2584	0	PREDICTED: Bison bison bison drebrin 1 (DBN1), mRNA	Q16643	848	0	Drebrin	.	.	.	.	pfam00241	Cofilin_ADF	Cofilin/tropomyosin-type actin-binding protein. Severs actin filaments and binds to actin monomers.	GO:0007015|actin filament organization;GO:0010643|cell communication by chemical coupling;GO:0010644|cell communication by electrical coupling;GO:0032507|maintenance of protein location in cell;GO:0061351|neural precursor cell proliferation;GO:0050773|regulation of dendrite development;GO:0048168|regulation of neuronal synaptic plasticity;	GO:0015629|actin cytoskeleton;GO:0042641|actomyosin;GO:0005938|cell cortex;GO:0005913|cell-cell adherens	GO:0003779|actin binding;GO:0098641|cadherin binding involved in cell-cell	.	.

MAN1B1	1333.884	0.313283	0.111792724	2.802359	0.005073	0.033213	Not_Sig	no	gi|821423325|ref|XP_012394189.1|	2727	0	PREDICTED: endoplasmic reticulum mannosyl-oligosaccharide 1,2-alpha-mannosidase isoform X2 [Orcinus orca]	gi|982940269|ref|XM_015473736.1|	1704	0	PREDICTED: Bos taurus mannosidase, alpha, class 1B, member 1 (MAN1B1), transcript variant X1, mRNA	Q9UKM7	941	0	Endoplasmic reticulum mannosyl-oligosaccharide 1,2-alpha-mannosidase	.	.	.	.	pfam01532	Glyco_hydro_47	Glycosyl hydrolase family 47. Members of this family are alpha-mannosidases that catalyse the hydrolysis of the terminal 1,2-	GO:1904380|endoplasmic reticulum mannose trimming;GO:0030433|ER-associated ubiquitin-dependent protein catabolic process;GO:1904382|mannose trimming involved in glycoprotein ERAD pathway;GO:0006491|N-glycan processing;GO:0009311|oligosaccharide metabolic process;GO:0036508|protein alpha-1,2-demannosylation;GO:0036509|trimming of terminal mannose on B branch;	GO:0005783|endoplasmic reticulum;GO:0005789|endoplasmic reticulum membrane;GO:0044322|endoplasmic	GO:0004559|alpha-mannosidase activity;GO:0005509|calcium ion	K01230	MAN1; mannosyl-oligosaccharide alpha-1,2-mannosidase
LAPTM4B	620.6625	-0.31236	0.111491071	-2.80165	0.0050842	0.033267	Not_Sig	no	gi|926708788|ref|XP_013824738.1|	988	2e-128	PREDICTED: lysosomal-associated transmembrane protein 4B [Capra hircus]	gi|151554321|gb|BC149605.1|	1379	0	Bos taurus lysosomal protein transmembrane 4 beta, mRNA (cDNA clone MGC:159492 IMAGE:8404368), complete cds	Q71SV0	365	3e-123	Lysosomal-associated transmembrane protein 4B	.	.	.	.	pfam03821	Mtp	Golgi 4-transmembrane spanning transporter.	GO:0006810|transport;	GO:0012505|endomembrane system;GO:0016021|integral component of membrane;GO:0016020|membrane;	.	K12387	LAPTM; lysosomal-associated transmembrane protein
SDC3	955.6748	0.40315	0.14394925	2.800643	0.0051001	0.033352	Not_Sig	no	gi|942086095|ref|XP_014337093.1|	744	3e-84	PREDICTED: LOW QUALITY PROTEIN: syndecan-3 [Bos mutus]	gi|742105134|ref|XM_010836881.1|	4870	0	PREDICTED: Bison bison bison syndecan 3 (SDC3), mRNA	O75056	270	3e-77	Syndecan-3	.	.	.	.	pfam01034	Syndecan	Syndecan domain. Syndecans are transmembrane heparin sulfate proteoglycans which are implicated in the binding of	GO:0016477|cell migration;GO:0006024|glycosaminoglycan biosynthetic process;GO:0006027|glycosaminoglycan catabolic process;GO:0030203|glycosaminoglycan metabolic process;GO:0001523|retinoid metabolic process;	GO:0009986|cell surface;GO:0005796|Golgi lumen;GO:0016021|integral component of membrane;GO:0043202|lysosomal lumen;GO:0016020|membrane;GO:000588	.	K16337	SDC3; syndecan 3
NPC2	3407.589	-0.31774	0.113528525	-2.79878	0.0051297	0.033505	Not_Sig	no	gi|440901722|gb|ELR52611.1|	726	1e-91	Epididymal secretory protein E1, partial [Bos mutus]	gi|942070205|ref|XM_005900977.2|	1289	0	PREDICTED: Bos mutus Niemann-Pick disease, type C2 (NPC2), mRNA	P79345	280	2e-91	Epididymal secretory protein E1	.	.	.	.	pfam02221	E1_DerP2_DerF2	ML domain. ML domain - MD-2-related lipid recognition domain. This family consists of proteins from plants, animals and fungi,	GO:0033344|cholesterol efflux;GO:0042632|cholesterol homeostasis;GO:0008203|cholesterol metabolic process;GO:0030301|cholesterol transport;GO:0032367|intracellular cholesterol transport;GO:0009615|response to virus;	GO:0005783|endoplasmic reticulum;GO:0070062|extracellular exosome;GO:0005764|lysosome;	GO:0015485|cholesterol binding;	K13443	NPC2; Niemann-Pick C2 protein
PLIN3	515.1219	0.345118	0.123317264	2.798619	0.0051322	0.033505	Not_Sig	no	gi|528955688|ref|XP_005209028.1|	2147	0	PREDICTED: perilipin-3 isoform X2 [Bos taurus]	gi|982930906|ref|XM_005208971.3|	1867	0	PREDICTED: Bos taurus perilipin 3 (PLIN3), transcript variant X5, mRNA	Q5BLZ2	726	0	Perilipin-3	.	.	.	.	pfam03036	Perilipin	Perilipin family. The perilipin family includes lipid droplet-associated protein (perilipin) and adipose differentiation-related	GO:0006810|transport;	GO:0010008|endosome membrane;GO:0005811|lipid particle;	.	.	.
GOLM2	1582.663	-0.316	0.112916657	-2.79857	0.005133	0.033505	Not_Sig	no	gi|528967345|ref|XP_005212271.1|	2097	0	PREDICTED: protein CASC4 isoform X1 [Bos taurus]	gi|982937950|ref|XM_005212214.3|	3400	0	PREDICTED: Bos taurus cancer susceptibility candidate 4 (CASC4), transcript variant X1, mRNA	Q5R5X4	733	0	Protein CASC4	.	.	.	.	pfam05483	SCP-1	Synaptonemal complex protein 1 (SCP-1). Synaptonemal complex protein 1 (SCP-1) is the major component of the transverse	.	GO:0016021|integral component of membrane;	.	.	.
DSEL	1451.587	-0.3076	0.109919991	-2.79843	0.0051351	0.033505	Not_Sig	no	gi|742212323|ref|XP_010860619.1|	6063	0	PREDICTED: dermatan-sulfate epimerase-like protein [Bison bison bison]	gi|982965198|ref|XM_005224003.3|	9337	0	PREDICTED: Bos taurus dermatan sulfate epimerase-like (DSEL), transcript variant X2, mRNA	Q8IZU8	2125	0	Dermatan-sulfate epimerase-like protein	.	.	.	.	pfam00685	Sulfotransfer_1	Sulfotransferase domain.	GO:0030204|chondroitin sulfate metabolic process;GO:0030208|dermatan sulfate biosynthetic process;	GO:0000139|Golgi membrane;GO:0016021|integral component of membrane;	GO:0047757|chondroitin-glucuronate 5-epimerase activity;GO:0008146	.	.
AUP1	1196.813	0.349312	0.124832628	2.798243	0.0051381	0.033506	Not_Sig	no	gi|926703669|ref|XP_005686375.2|	2099	0	PREDICTED: ancient ubiquitous protein 1 [Capra hircus]	gi|942087772|ref|XM_005906735.2|	1563	0	PREDICTED: Bos mutus ancient ubiquitous protein 1 (AUP1), transcript variant X1, mRNA	A1L134	669	0	Ancient ubiquitous protein 1	.	.	.	.	pfam02845	CUE	CUE domain. CUE domains have been shown to bind ubiquitin. It has been suggested that CUE domains are related to pfam00627	GO:0030433|ER-associated ubiquitin-dependent protein catabolic process;GO:0030970|retrograde protein transport, ER to cytosol;	GO:0070062|extracellular exosome;GO:0000839|Hrd1p ubiquitin ligase ERAD-L complex;GO:0030176|integral component of endoplasmic reticulum	GO:0043130|ubiquitin binding;GO:0097027|ubiquitin-protein	.	.
MRPL13	278.4831	-0.40096	0.143418973	-2.79576	0.0051778	0.033746	Not_Sig	no	gi|77736592|ref|NP_001029977.1|	950	7e-127	39S ribosomal protein L13, mitochondrial [Bos taurus]	gi|742151284|ref|XM_010846185.1|	842	0	PREDICTED: Bison bison bison mitochondrial ribosomal protein L13 (MRPL13), mRNA	Q3SYS1	370	2e-128	39S ribosomal protein L13, mitochondrial	BH0168	102	4.67e-26	COG0102	pfam00572	Ribosomal_L13	Ribosomal protein L13.	GO:0070125|mitochondrial translational elongation;GO:0070124|mitochondrial translational initiation;GO:0006412|translation;	GO:0005743|mitochondrial inner membrane;GO:0005762|mitochondrial large ribosomal subunit;GO:0005761|mitochondrial	GO:0003729|mRNA binding;GO:0003735|structural constituent of	K02871	RP-L13, MRPL13, rplM; large subunit ribosomal protein L13
RANBP3	922.6398	0.310532	0.111081713	2.795529	0.0051815	0.03375	Not_Sig	no	gi|926695652|ref|XP_013820700.1|	2366	0	PREDICTED: ran-binding protein 3 [Capra hircus]	gi|982930665|ref|XM_010806912.2|	3031	0	PREDICTED: Bos taurus RAN binding protein 3 (RANBP3), transcript variant X4, mRNA	Q9H6Z4	794	0	Ran-binding protein 3	.	.	.	.	pfam00638	Ran_BP1	RanBP1 domain.	GO:0046907|intracellular transport;GO:0046827|positive regulation of protein export from nucleus;GO:0015031|protein transport;	GO:0005829|cytosol;GO:0005654|nucleoplasm;GO:0005634|nucleus;	GO:0070412|R-SMAD binding;GO:0008536|Ran GTPase	K15304	RANBP3; Ran-binding protein 3
LRRC42	270.2686	-0.43306	0.154931118	-2.79519	0.005187	0.033767	Not_Sig	no	gi|594087601|ref|XP_006067274.1|	2224	0	PREDICTED: leucine-rich repeat-containing protein 42 isoform X1 [Bubalus bubalis]	gi|742154697|ref|XM_010847145.1|	1691	0	PREDICTED: Bison bison bison leucine rich repeat containing 42 (LRRC42), mRNA	Q2HJ90	829	0	Leucine-rich repeat-containing protein 42	.	.	.	.	pfam15402	Spc7_N	N-terminus of kinetochore NMS complex subunit Spc7.	.	.	.	.	.
QPRT	130.1675	-0.49337	0.176592512	-2.79383	0.0052087	0.033889	Not_Sig	no	gi|742107289|ref|XP_010835620.1|	1202	2e-160	PREDICTED: nicotinate-nucleotide pyrophosphorylase [carboxylating] [Bison bison bison]	gi|926724211|ref|XM_013974887.1|	1259	0	PREDICTED: Capra hircus quinolinate phosphoribosyltransferase (QPRT), mRNA	Q3T063	464	1e-160	Nicotinate-nucleotide pyrophosphorylase [carboxylating]	YFR047c	213	7.59e-65	COG0157	pfam01729	QRPTase_C	Quinolinate phosphoribosyl transferase, C-terminal domain. Quinolinate phosphoribosyl transferase (QPRTase) or	GO:0009435|NAD biosynthetic process;GO:0034213|quinolinate catabolic process;	GO:0005737|cytoplasm;	GO:0004514|nicotinate-nucleotide diphosphorylase (carboxylating)	K00767	nadC, QPRT; nicotinate-nucleotide pyrophosphorylase
GTF3C6	372.9746	-0.39621	0.141827498	-2.79362	0.0052122	0.033893	Not_Sig	no	gi|742188938|ref|XP_010854916.1|	1157	9e-150	PREDICTED: general transcription factor 3C polypeptide 6 [Bison bison bison]	gi|982987578|ref|XM_596663.8|	2405	0	PREDICTED: Bos taurus general transcription factor IIIC subunit 6 (GTF3C6), mRNA	Q969F1	322	2e-102	General transcription factor 3C polypeptide 6	.	.	.	.	pfam10419	TFIIIC_sub6	TFIIIC subunit. This is a family of proteins subunits of TFIIIC. TFIIIC in yeast and humans is required for transcription of tRNA and 5 S RNA	GO:0042791|5S class rRNA transcription from RNA polymerase III type 1 promoter;GO:0006383|transcription from RNA polymerase III promoter;GO:0006351|transcription, DNA-templated;GO:0042797|tRNA transcription from RNA polymerase III promoter;	GO:0005654|nucleoplasm;GO:0000127|transcription factor TFIIIC complex;	GO:0003677|DNA binding;	K15203	GTF3C6; general transcription factor 3C polypeptide 6
PCBP2	4277.696	0.293724	0.105161596	2.793075	0.005221	0.03393	Not_Sig	no	gi|62087230|dbj|BAD92062.1|	1853	0	poly(rC)-binding protein 2 isoform b variant [Homo sapiens]	gi|742194275|ref|XM_010857935.1|	1563	0	PREDICTED: Bison bison bison poly(rC) binding protein 2 (PCBP2), transcript variant X2, mRNA	Q61990	704	0	Poly(rC)-binding protein 2	.	.	.	.	pfam00013	KH_1	KH domain. KH motifs bind RNA in vitro. Autoantibodies to Nova, a KH domain protein, cause paraneoplastic opsoclonus ataxia.	GO:0051607|defense response to virus;GO:0045087|innate immune response;GO:0075522|IRES-dependent viral translational initiation;GO:0050687|negative regulation of defense response to virus;GO:0043161|proteasome-mediated ubiquitin-dependent protein catabolic process;GO:0039694|viral RNA genome replication;	GO:0005737|cytoplasm;GO:0070062|extracellular exosome;GO:0005925|focal adhesion;GO:0030529|ribonucleoprotein complex;GO:0016020|membrane;GO:00056	GO:1990829|C-rich single-stranded DNA binding;GO:0003677|DNA	K13162	PCBP2_3_4; poly(rC)-binding protein 2/3/4
TRABD	648.0275	0.385481	0.13803925	2.792549	0.0052295	0.033932	Not_Sig	no	gi|77735851|ref|NP_001029624.1|	1673	0	traB domain-containing protein [Bos taurus]	gi|741928807|ref|XM_010805824.1|	2045	0	PREDICTED: Bos taurus TraB domain containing (TRABD), transcript variant X1, mRNA	Q58DF3	649	0	TraB domain-containing protein	BB0416	95.9	3.17e-20	COG1916	pfam01963	TraB	TraB family. pAD1 is a hemolysin/bacteriocin plasmid originally identified in Enterococcus faecalis DS16. It	.	.	.	.	.
GMIP	454.5823	0.414676	0.148500503	2.792421	0.0052315	0.033932	Not_Sig	no	gi|555997543|ref|XP_005910494.1|	4466	0	PREDICTED: GEM-interacting protein isoform X2 [Bos mutus]	gi|742155082|ref|XM_010847242.1|	3341	0	PREDICTED: Bison bison bison GEM interacting protein (GMIP), transcript variant X1, mRNA	Q9P107	1445	0	GEM-interacting protein	.	.	.	.	pfam00620	RhoGAP	RhoGAP domain. GTPase activator proteins towards Rho/Rac/Cdc42-like small GTPases.	GO:0035556|intracellular signal transduction;GO:0034260|negative regulation of GTPase activity;GO:0051056|regulation of small GTPase mediated signal transduction;	GO:0005829|cytosol;GO:0005622|intracellular;	GO:0005096|GTPase activator activity;GO:0046872|metal ion binding;	.	.
TMC6	67.71628	0.682878	0.244564706	2.792217	0.0052348	0.033932	UP	yes	gi|741964630|ref|XP_010814814.1|	3707	0	PREDICTED: transmembrane channel-like protein 6 isoform X1 [Bos taurus]	gi|982958554|ref|XM_010816514.2|	3288	0	PREDICTED: Bos taurus transmembrane channel like 6 (TMC6), transcript variant X2, mRNA	Q7Z403	1030	0	Transmembrane channel-like protein 6	.	.	.	.	pfam07810	TMC	TMC domain. These sequences are similar to a region conserved amongst various protein products of the transmembrane channel-like	GO:0006811|ion transport;	GO:0005737|cytoplasm;GO:0005783|endoplasmic reticulum;GO:0005789|endoplasmic reticulum membrane;GO:0070062|extracellular	.	.	.
DHRS7	417.8713	-0.34397	0.123195224	-2.79206	0.0052374	0.033932	Not_Sig	no	gi|555957220|ref|XP_005890744.1|	1650	0	PREDICTED: dehydrogenase/reductase SDR family member 7 [Bos mutus]	gi|942040995|ref|XM_005890682.2|	1298	0	PREDICTED: Bos mutus dehydrogenase/reductase (SDR family) member 7 (DHRS7), mRNA	Q9Y394	558	0	Dehydrogenase/reductase SDR family member 7	BS_yqjQ	134	1.21e-35	COG0300	pfam00106	adh_short	short chain dehydrogenase. This family contains a wide variety of dehydrogenases.	.	GO:0016020|membrane;	GO:0016491|oxidoreductase activity;	K11165	DHRS7; dehydrogenase/reductase SDR family member 7
ZNF526	145.1821	0.506602	0.18145414	2.791902	0.0052399	0.033932	Not_Sig	no	gi|156120641|ref|NP_001095467.1|	2470	0	zinc finger protein 526 [Bos taurus]	gi|156120640|ref|NM_001101997.1|	2778	0	Bos taurus zinc finger protein 526 (ZNF526), mRNA	A6QR00	956	0	Zinc finger protein 526	.	.	.	.	pfam13465	zf-H2C2_2	Zinc-finger double domain.	GO:0006355|regulation of transcription, DNA-templated;GO:0006351|transcription, DNA-templated;	GO:0005634|nucleus;	GO:0003677|DNA binding;GO:0046872|metal ion binding;	.	.
LY75	463.5098	-0.42809	0.153337665	-2.79178	0.0052418	0.033932	Not_Sig	no	gi|942058567|ref|XP_014334161.1|	10084	0	PREDICTED: LOW QUALITY PROTEIN: lymphocyte antigen 75 [Bos mutus]	gi|942058566|ref|XM_014478675.1|	8159	0	PREDICTED: Bos mutus lymphocyte antigen 75 (LOC102285069), mRNA	O60449	2826	0	Lymphocyte antigen 75	.	.	.	.	pfam00059	Lectin_C	Lectin C-type domain. This family includes both long and short form C-type	GO:0006897|endocytosis;GO:0006955|immune response;GO:0006954|inflammatory response;	GO:0070062|extracellular exosome;GO:0005887|integral component of plasma membrane;	GO:0030246|carbohydrate binding;GO:0004872|receptor	K06559	LY75; lymphocyte antigen 75
LOC138985537	270.8793	0.418192	0.149794016	2.791782	0.0052419	0.033932	Not_Sig	no	gi|556760195|ref|XP_005975418.1|	168	2e-10	PREDICTED: ly-6/neurotoxin-like protein 1-like [Pantholops hodgsonii]	gi|850494870|gb|CP011909.1|	896	0	Ovis canadensis canadensis isolate 43U chromosome 24 sequence	.	.	.	.	.	.	.	.	pfam15244	HSD3	Hydroxy-steroid dehydrogenase. This family also goes by the name of Spermatogenesis-associated protein 7 or SPAT7. It is an aldo-	.	.	.	.	.
AGPAT1	761.867	0.429633	0.153943315	2.790854	0.0052569	0.03401	Not_Sig	no	gi|426250489|ref|XP_004018969.1|	1379	0	PREDICTED: 1-acyl-sn-glycerol-3-phosphate acyltransferase alpha isoform X1 [Ovis aries]	gi|742091244|ref|XM_010833538.1|	2195	0	PREDICTED: Bison bison bison 1-acylglycerol-3-phosphate O-acyltransferase 1 (AGPAT1), mRNA	Q95JH2	535	0	1-acyl-sn-glycerol-3-phosphate acyltransferase alpha	YDL052c	141	1.17e-36	COG0204	pfam01553	Acyltransferase	Acyltransferase. This family contains acyltransferases involved in phospholipid biosynthesis and other proteins of unknown	GO:0016024|CDP-diacylglycerol biosynthetic process;GO:0046474|glycerophospholipid biosynthetic process;GO:0006654|phosphatidic acid biosynthetic process;GO:0001819|positive regulation of cytokine production;	GO:0005789|endoplasmic reticulum membrane;GO:0016021|integral component of membrane;	GO:0003841|1-acylglycerol-3-phosphate O-acyltransferase	K13509	AGPAT1_2; lysophosphatidate acyltransferase [EC:2.3.1.51]
MTA2	1461.044	0.294485	0.10553428	2.790421	0.005264	0.034036	Not_Sig	no	gi|803191098|ref|XP_011957421.1|	3320	0	PREDICTED: metastasis-associated protein MTA2 isoform X1, partial [Ovis aries]	gi|742179102|ref|XM_010854020.1|	3035	0	PREDICTED: Bison bison bison metastasis associated 1 family, member 2 (MTA2), mRNA	Q9R190	1252	0	Metastasis-associated protein MTA2	.	.	.	.	pfam01426	BAH	BAH domain. This domain has been called BAH (Bromo adjacent homology) domain and has also been called ELM1 and BAM	GO:0043044|ATP-dependent chromatin remodeling;GO:0006338|chromatin remodeling;GO:0006306|DNA methylation;GO:0006323|DNA packaging;GO:0000122|negative regulation of transcription from RNA polymerase II promoter;GO:0045892|negative regulation of transcription, DNA-templated;GO:0045944|positive regulation of transcription from RNA polymerase II promoter;GO:0010762|regulation of fibroblast migration;	GO:0000118|histone deacetylase complex;GO:0016020|membrane;GO:0000790|nuclear chromatin;GO:0005654|nucleoplasm;GO:00	GO:0003682|chromatin binding;GO:0004407|histone	K11660	MTA; metastasis-associated protein MTA
SNURF	527.0108	-0.37104	0.132995516	-2.78991	0.0052723	0.034072	Not_Sig	no	gi|594091032|ref|XP_006068908.1|	384	2e-40	PREDICTED: SNRPN upstream reading frame protein isoform X1 [Bubalus bubalis]	gi|942098129|ref|XM_014483070.1|	2359	0	PREDICTED: Bos mutus SNRPN upstream reading frame protein (LOC102267948), transcript variant X1, mRNA	Q9XS96	134	3e-35	SNRPN upstream reading frame protein	.	.	.	.	pfam07192	SNURF	SNURF/RPN4 protein. This family consists of several mammalian SNRPN upstream reading frame (SNURF) proteins. SNURF or RPF4	.	GO:0005634|nucleus;	.	.	.
GTF2A1	324.3818	-0.49632	0.177969101	-2.78882	0.00529	0.034167	Not_Sig	no	gi|507944738|ref|XP_004681706.1|	1057	5e-126	PREDICTED: transcription initiation factor IIA subunit 1 [Condylura cristata]	gi|965886731|ref|XM_012155887.2|	5613	0	PREDICTED: Ovis aries musimon general transcription factor IIA 1 (GTF2A1), transcript variant X1, mRNA	P52655	410	4e-127	Transcription initiation factor IIA subunit 1	YOR194c	63.9	9.54e-10	COG5149	pfam03153	TFIIA	Transcription factor IIA, alpha/beta subunit. Transcription initiation factor IIA (TFIIA) is a heterotrimer, the three subunits being known as	GO:0006355|regulation of transcription, DNA-templated;GO:0042795|snRNA transcription from RNA polymerase II promoter;GO:0006368|transcription elongation from RNA polymerase II promoter;GO:0006366|transcription from RNA polymerase II promoter;GO:0006367|transcription initiation from RNA polymerase II promoter;	GO:0005737|cytoplasm;GO:0005654|nucleoplasm;GO:0005672|transcription factor TFIIA complex;	GO:0003677|DNA binding;GO:0046982|protein heterodimerization	K03122	TFIIA1, GTF2A1, TOA1; transcription initiation factor TFIIA large subunit
MYO18A	705.5576	0.343105	0.12307949	2.787671	0.0053088	0.034268	Not_Sig	no	gi|982956348|ref|XP_015314065.1|	10135	0	PREDICTED: unconventional myosin-XVIIIa isoform X7 [Bos taurus]	gi|942033943|ref|XM_005888202.2|	7316	0	PREDICTED: Bos mutus myosin XVIIIA (MYO18A), mRNA	Q92614	3057	0	Unconventional myosin-XVIIIa	SPCC645.05c	362	4.45e-101	COG5022	pfam00063	Myosin_head	Myosin head (motor domain).	GO:0031032|actomyosin structure organization;GO:0090164|asymmetric Golgi ribbon formation;GO:0016477|cell migration;GO:0006259|DNA metabolic process;GO:0007030|Golgi organization;GO:0090161|Golgi ribbon formation;GO:0048194|Golgi vesicle budding;GO:0043066|negative regulation of apoptotic process;GO:0050714|positive regulation of protein secretion;	GO:0042641|actomyosin;GO:0005793|endoplasmic reticulum-Golgi intermediate compartment;GO:0005794|Golgi apparatus;GO:0000139|Golgi	GO:0051015|actin filament binding;GO:0043531|ADP	K10362	MYO18; myosin XVIII
UBA5	1286.468	-0.36175	0.129777352	-2.7875	0.0053117	0.034268	Not_Sig	no	gi|155371865|ref|NP_001094537.1|	1819	0	ubiquitin-like modifier-activating enzyme 5 [Bos taurus]	gi|742116701|ref|XM_010839576.1|	3372	0	PREDICTED: Bison bison bison ubiquitin-like modifier activating enzyme 5 (UBA5), mRNA	A7MAZ3	705	0	Ubiquitin-like modifier-activating enzyme 5	Ta0844	77.4	1.24e-14	COG0476	pfam00899	ThiF	ThiF family. This family contains a repeated domain in ubiquitin activating enzyme E1 and members of the bacterial	GO:0061504|cyclic threonylcarbamoyladenosine biosynthetic process;GO:1990592|protein K69-linked ufmylation;GO:0071569|protein ufmylation;GO:0033146|regulation of intracellular estrogen receptor signaling pathway;GO:0034976|response to endoplasmic reticulum stress;	GO:0005829|cytosol;GO:0005634|nucleus;	GO:0005524|ATP binding;GO:0046872|metal ion binding;GO:0061503	K12164	UBA5, UBE1DC1; ubiquitin-like modifier-activating enzyme 5
RHEB	1958.187	-0.36687	0.131628259	-2.78716	0.0053173	0.03427	Not_Sig	no	gi|528947692|ref|XP_005205918.1|	939	1e-119	PREDICTED: GTP-binding protein Rheb isoform X1 [Bos taurus]	gi|850481938|gb|CP011889.1|	1036	0	Ovis canadensis canadensis isolate 43U chromosome 4 sequence	Q62639	341	2e-111	GTP-binding protein Rheb	.	.	.	.	pfam00071	Ras	Ras family. Includes sub-families Ras, Rab, Rac, Ral, Ran, Rap Ypt1 and more. Shares P-loop motif with GTP_EFTU, arf and	GO:0032008|positive regulation of TOR signaling;GO:0048168|regulation of neuronal synaptic plasticity;GO:0007264|small GTPase mediated signal transduction;	GO:0005829|cytosol;GO:0030425|dendrite;GO:0005789|endoplasmic reticulum membrane;GO:0000139|Golgi membrane;GO:0043025|neuronal cell body;	GO:0019003|GDP binding;GO:0005525|GTP binding;GO:0003924	K07208	RHEB; Ras homolog enriched in brain
CEP76	440.1233	-0.37512	0.134596244	-2.787	0.0053198	0.03427	Not_Sig	no	gi|300798638|ref|NP_001179335.1|	3256	0	centrosomal protein of 76 kDa [Bos taurus]	gi|982965486|ref|XM_010818829.2|	3705	0	PREDICTED: Bos taurus centrosomal protein 76kDa (CEP76), transcript variant X1, mRNA	Q8TAP6	1216	0	Centrosomal protein of 76 kDa	.	.	.	.	pfam15627	CEP76-C2	CEP76 C2 domain. Many ciliary proteins are involved in ciliogenesis and implicated for ciliophathies. A recent study has	GO:0000086|G2/M transition of mitotic cell cycle;GO:0046599|regulation of centriole replication;	GO:0005814|centriole;GO:0005813|centrosome;GO:0005829|cytosol;GO:0043234|protein complex;	.	K16457	CEP76; centrosomal protein CEP76
SLC38A2	8437.741	-0.30711	0.110196437	-2.7869	0.0053215	0.03427	Not_Sig	no	gi|440899654|gb|ELR50925.1|	2166	0	Sodium-coupled neutral amino acid transporter 2, partial [Bos mutus]	gi|942077027|ref|XM_005903247.2|	4788	0	PREDICTED: Bos mutus solute carrier family 38, member 2 (SLC38A2), transcript variant X1, mRNA	A2VE31	816	0	Sodium-coupled neutral amino acid transporter 2	.	.	.	.	pfam01490	Aa_trans	Transmembrane amino acid transporter protein. This transmembrane region is found in many amino acid transporters	GO:0003333|amino acid transmembrane transport;GO:0006814|sodium ion transport;	GO:0005887|integral component of plasma membrane;	GO:0015171|amino acid transmembrane transporter activity;GO:0015293	K14207	SLC38A2, SNAT2; solute carrier family 38 (sodium-coupled neutral amino acid
APPL1	1174.619	-0.3393	0.1217593	-2.78668	0.0053252	0.03427	Not_Sig	no	gi|149642983|ref|NP_001092473.1|	3496	0	DCC-interacting protein 13-alpha [Bos taurus]	gi|742170790|ref|XM_010851653.1|	5882	0	PREDICTED: Bison bison bison adaptor protein, phosphotyrosine interaction, PH domain and leucine zipper containing 1 (APPL1), mRNA	Q9UKG1	1323	0	DCC-interacting protein 13-alpha	.	.	.	.	pfam00640	PID	Phosphotyrosine interaction domain (PTB/PID).	GO:0007049|cell cycle;GO:0008283|cell proliferation;GO:0097192|extrinsic apoptotic signaling pathway in absence of ligand;GO:0008286|insulin receptor signaling pathway;GO:0090003|regulation of establishment of protein localization to plasma membrane;GO:0046324|regulation of glucose import;GO:0007165|signal transduction;	GO:0005737|cytoplasm;GO:0005829|cytosol;GO:0031901|early endosome membrane;GO:0010008|endosome membrane;GO:0070062|extracellular	GO:0042802|identical protein binding;GO:0043422|protein kinase B	K08733	APPL, DIP13A; DCC-interacting protein 13 alpha
PLEKHM1	902.4349	0.375149	0.13463793	2.786352	0.0053305	0.03427	Not_Sig	no	gi|742086492|ref|XP_010830702.1|	5174	0	PREDICTED: pleckstrin homology domain-containing family M member 1 [Bison bison bison]	gi|594077154|ref|XM_006062241.1|	5066	0	PREDICTED: Bubalus bubalis pleckstrin homology domain containing, family M (with RUN domain) member 1 (PLEKHM1), transcript variant X1, mRNA	Q9Y4G2	1627	0	Pleckstrin homology domain-containing family M member 1	.	.	.	.	pfam13901	DUF4206	Domain of unknown function (DUF4206). This is a family of cysteine-rich proteins. Many members also carry a pleckstrin-	GO:0006914|autophagy;GO:0035556|intracellular signal transduction;GO:0015031|protein transport;	GO:0010008|endosome membrane;GO:0005765|lysosomal membrane;	GO:0046872|metal ion binding;	.	.
KDSR	748.9607	-0.30729	0.110283676	-2.78633	0.0053309	0.03427	Not_Sig	no	gi|114052280|ref|NP_001039384.1|	1661	0	3-ketodihydrosphingosine reductase precursor [Bos taurus]	gi|594082043|ref|XR_328078.1|	3668	0	PREDICTED: Bubalus bubalis uncharacterized LOC102402617 (LOC102402617), misc_RNA	Q2KIJ5	642	0	3-ketodihydrosphingosine reductase	.	.	.	.	pfam00106	adh_short	short chain dehydrogenase. This family contains a wide variety of dehydrogenases.	GO:0006666|3-keto-sphinganine metabolic process;	GO:0005783|endoplasmic reticulum;GO:0005789|endoplasmic reticulum membrane;GO:0016021|integral component of membrane;	GO:0047560|3-dehydrosphinganine reductase activity;	K04708	E1.1.1.102; 3-dehydrosphinganine reductase [EC:1.1.1.102]
THOC7	290.9963	-0.45482	0.16324108	-2.78621	0.0053329	0.03427	Not_Sig	no	gi|982997664|ref|XP_015322514.1|	1047	7e-140	PREDICTED: THO complex subunit 7 homolog isoform X2 [Bos taurus]	gi|982997663|ref|XM_015467028.1|	955	0	PREDICTED: Bos taurus THO complex subunit 7 homolog (LOC101907835), transcript variant X2, mRNA	Q6I9Y2	348	3e-118	THO complex subunit 7 homolog	.	.	.	.	pfam05615	THOC7	Tho complex subunit 7. The Tho complex is involved in transcription elongation and mRNA export from the nucleus.	GO:0031124|mRNA 3'-end processing;GO:0006406|mRNA export from nucleus;GO:0006405|RNA export from nucleus;GO:0008380|RNA splicing;GO:0006369|termination of RNA polymerase II transcription;GO:0006368|transcription elongation from RNA polymerase II promoter;GO:0046784|viral mRNA export from host cell nucleus;	GO:0005737|cytoplasm;GO:0016607|nuclear speck;GO:0005654|nucleoplasm;GO:0000446|nucleoplasmic THO	GO:0003676|nucleic acid binding;GO:0003723|RNA binding;	K13176	THOC7; THO complex subunit 7
GRK6	354.0773	0.407475	0.14628596	2.785471	0.005345	0.034329	Not_Sig	no	gi|329663681|ref|NP_001192319.1|	2815	0	G protein-coupled receptor kinase 6 isoform 2 [Bos taurus]	gi|982931461|ref|XM_010807152.2|	2872	0	PREDICTED: Bos taurus G protein-coupled receptor kinase 6 (GRK6), transcript variant X2, mRNA	P43250	1048	0	G protein-coupled receptor kinase 6	.	.	.	.	pfam00069	Pkinase	Protein kinase domain.	GO:0008277|regulation of G-protein coupled receptor protein signaling pathway;GO:0016055|Wnt signaling pathway;	GO:0016020|membrane;GO:0005886|plasma membrane;	GO:0005524|ATP binding;GO:0047696|beta-adrenergic receptor kinase	K08291	GRK4_5_6; G protein-coupled receptor kinase [EC:2.7.11.16]
ABHD12	893.2315	0.309206	0.111036494	2.78472	0.0053574	0.034376	Not_Sig	no	gi|926706286|ref|XP_013823851.1|	2066	0	PREDICTED: unconventional myosin-XVI isoform X3 [Capra hircus]	gi|118151299|ref|NM_001078116.1|	1837	0	Bos taurus abhydrolase domain containing 12 (ABHD12), mRNA	Q08DW9	786	0	Monoacylglycerol lipase ABHD12	Rv2307c	89	8.52e-19	COG1073	pfam12695	Abhydrolase_5	Alpha/beta hydrolase family. This family contains a diverse range of alpha/beta hydrolase enzymes.	GO:0046464|acylglycerol catabolic process;GO:0007628|adult walking behavior;GO:0006660|phosphatidylserine catabolic process;GO:0010996|response to auditory stimulus;	GO:0032281|AMPA glutamate receptor complex;GO:0016021|integral component of membrane;	GO:0047372|acylglycerol lipase activity;	K13704	ABHD12; abhydrolase domain-containing protein 12 [EC:3.1.1.23]
IL17RA	1740.347	0.321045	0.115290409	2.784665	0.0053583	0.034376	Not_Sig	no	gi|982982594|ref|XP_010826265.2|	3481	0	PREDICTED: interleukin-17 receptor A isoform X3 [Bos taurus]	gi|982982593|ref|XM_010827963.2|	3563	0	PREDICTED: Bos taurus interleukin 17 receptor A (IL17RA), transcript variant X3, mRNA	Q96F46	890	0	Interleukin-17 receptor A	.	.	.	.	pfam08357	SEFIR	SEFIR domain. This family comprises IL17 receptors (IL17Rs) and SEF proteins. The latter are feedback inhibitors of FGF	GO:0007166|cell surface receptor signaling pathway;GO:0050832|defense response to fungus;GO:0072537|fibroblast activation;GO:0071621|granulocyte chemotaxis;GO:1900017|positive regulation of cytokine production involved in inflammatory response;GO:2000667|positive regulation of interleukin-13 secretion;GO:0032747|positive regulation of interleukin-23 production;GO:2000664|positive regulation of interleukin-5 secretion;	GO:0005576|extracellular region;GO:0005887|integral component of plasma membrane;GO:0005886|plasma membrane;	GO:0030368|interleukin-17 receptor activity;	K05164	IL17R; interleukin 17 receptor
C1QTNF1	2866.923	0.351528	0.126248205	2.784422	0.0053623	0.034382	Not_Sig	no	gi|440892468|gb|ELR45647.1|	1348	8e-176	Complement C1q tumor necrosis factor-related protein 1, partial [Bos mutus]	gi|982958619|ref|XM_005221199.3|	2833	0	PREDICTED: Bos taurus C1q and tumor necrosis factor related protein 1 (C1QTNF1), transcript variant X2, mRNA	Q9BXJ1	443	2e-146	Complement C1q tumor necrosis factor-related protein 1	.	.	.	.	pfam00386	C1q	C1q domain. C1q is a subunit of the C1 enzyme complex that activates the serum complement system.	GO:0010544|negative regulation of platelet activation;GO:0090331|negative regulation of platelet aggregation;GO:2000860|positive regulation of aldosterone secretion;GO:0007204|positive regulation of cytosolic calcium ion concentration;GO:0010628|positive regulation of gene expression;GO:0043410|positive regulation of MAPK cascade;GO:0051897|positive regulation of protein kinase B signaling;GO:0070208|protein heterotrimerization;GO:0051260|protein homooligomerization;GO:0010906|regulation of glucose metabolic	GO:0005581|collagen trimer;GO:0005615|extracellular space;GO:0005887|integral component of plasma membrane;	GO:0005518|collagen binding;	.	.
NFYC	420.5438	0.399388	0.14346173	2.783937	0.0053703	0.034396	Not_Sig	no	gi|802977185|ref|XP_011962497.1|	1195	3e-155	PREDICTED: nuclear transcription factor Y subunit gamma isoform X1 [Ovis aries]	gi|982920117|ref|XM_010803662.2|	2072	0	PREDICTED: Bos taurus nuclear transcription factor Y subunit gamma (NFYC), transcript variant X8, mRNA	Q5E9X1	455	3e-153	Nuclear transcription factor Y subunit gamma	YOR358w	131	8.80e-34	COG5208	pfam00808	CBFD_NFYB_HMF	Histone-like transcription factor (CBF/NF-Y) and archaeal histone. This family includes archaebacterial histones and	GO:0006351|transcription, DNA-templated;	GO:0016602|CCAAT-binding factor complex;	GO:0003677|DNA binding;GO:0003700|transcription factor activity, sequence-	K08066	NFYC; nuclear transcription factor Y, gamma

HSPA13	1693.826	-0.33676	0.120965969	-2.78393	0.0053704	0.034396	Not_Sig	no	gi|84370043|ref|NP_001033594.1|	2386	0	heat shock 70 kDa protein 13 precursor [Bos taurus]	gi|742144687|ref|XM_010845141.1|	4015	0	PREDICTED: Bison bison bison heat shock protein 70kDa family, member 13 (HSPA13), mRNA	Q2TBX4	923	0	Heat shock 70 kDa protein 13	YER103w	300	2.20e-87	COG0443	pfam00012	HSP70	Hsp70 protein. Hsp70 chaperones help to fold many proteins. Hsp70 assisted folding involves repeated cycles of substrate binding and	.	GO:0005783|endoplasmic reticulum;GO:0070062|extracellular exosome;	GO:0005524|ATP binding;	K09491	STCH; stress 70 protein chaperone microsome-associated 60kDa
H2AX	308.5302	0.446584	0.16049195	2.782595	0.0053926	0.034504	Not_Sig	no	gi|545848861|ref|XP_003129998.3|	815	2e-100	PREDICTED: histone H2AX [Sus scrofa]	gi|594111869|ref|XM_006078682.1|	1608	0	PREDICTED: Bubalus bubalis histone H2AX-like (LOC102392520), mRNA	P16104	241	2e-74	Histone H2AX	YDR225w	181	1.65e-53	COG5262	pfam00125	Histone	Core histone H2A/H2B/H3/H4.	GO:0006974|cellular response to DNA damage stimulus;GO:0071480|cellular response to gamma radiation;GO:0090398|cellular senescence;GO:0021987|cerebral cortex development;GO:0006342|chromatin silencing;GO:0000077|DNA damage checkpoint;GO:0006302|double-strand break repair;GO:0000724|double-strand break repair via homologous recombination;GO:0006303|double-strand break repair via nonhomologous end joining;GO:0051321|meiotic cell	GO:0000794|condensed nuclear chromosome;GO:0070062|extracellular exosome;GO:0001673|male germ cell nucleus;GO:0000790|nuclear	GO:0003684|damaged DNA binding;GO:0003677|DNA	K11251	H2A; histone H2A
HDAC5	1601.857	0.331464	0.119122375	2.782552	0.0053933	0.034504	Not_Sig	no	gi|440895481|gb|ELR47654.1|	4700	0	Histone deacetylase 5, partial [Bos mutus]	gi|982957995|ref|XM_005220824.3|	3751	0	PREDICTED: Bos taurus histone deacetylase 5 (HDAC5), transcript variant X2, mRNA	Q80ZH1	1610	0	Histone deacetylase 5	SPBC800.03	239	7.43e-66	COG0123	pfam00850	Hist_deacetyl	Histone deacetylase domain. Histones can be reversibly acetylated on several lysine residues. Regulation of	GO:0040029|regulation of gene expression, epigenetic;GO:0010830|regulation of myotube differentiation;GO:0006355|regulation of transcription, DNA-templated;GO:0006351|transcription, DNA-templated;	GO:0005737|cytoplasm;GO:0000118|histone deacetylase complex;GO:0005634|nucleus;	GO:0046872|metal ion binding;GO:0032041|NAD-dependent	K11406	HDAC4_5; histone deacetylase 4/5 [EC:3.5.1.98]
UNK	208.4319	0.499153	0.179407455	2.782234	0.0053986	0.034519	Not_Sig	no	gi|440895198|gb|ELR47459.1|	3767	0	RING finger protein unkempt-like protein, partial [Bos mutus]	gi|555992488|ref|XM_005907958.1|	3697	0	PREDICTED: Bos mutus unkempt family zinc finger (UNK), mRNA	Q9C0B0	1373	0	RING finger protein unkempt homolog	.	.	.	.	pfam03962	Mnd1	Mnd1 family. This family of proteins includes MND1 from S. cerevisiae. The mnd1 protein forms a complex with hop2 to	GO:0048667|cell morphogenesis involved in neuron differentiation;GO:2000766|negative regulation of cytoplasmic translation;GO:0001764|neuron migration;	GO:0005737|cytoplasm;GO:0005844|polysome;	GO:0046872|metal ion binding;GO:1990715|mRNA CDS	.	.
ACTR2	5868.891	-0.32394	0.116558405	-2.77923	0.0054488	0.03482	Not_Sig	no	gi|5031571|ref|NP_005713.1|	2010	0	actin-related protein 2 isoform b [Homo sapiens]	gi|156121072|ref|NM_001102213.1|	2709	0	Bos taurus ARP2 actin-related protein 2 homolog (yeast) (ACTR2), mRNA	P61161	778	0	Actin-related protein 2	YDL029w	517	6.15e-176	COG5277	pfam00022	Actin	Actin.	GO:0030036|actin cytoskeleton organization;GO:0034314|Arp2/3 complex-mediated actin nucleation;GO:0008356|asymmetric cell division;GO:0042384|cilium assembly;GO:0016482|cytoplasmic transport;GO:0007163|establishment or maintenance of cell polarity;GO:0051321|meiotic cell cycle;GO:0016344|meiotic chromosome movement towards spindle pole;GO:0033206|meiotic cytokinesis;GO:0051653|spindle localization;	GO:0030478|actin cap;GO:0005885|Arp2/3 protein complex;GO:0005938|cell cortex;GO:0042995|cell projection;GO:0005737|cytoplasm;GO:0005	GO:0005524|ATP binding;GO:0005200|structural constituent of	K17260	ACTR2, ARP2; actin-related protein 2
NDST1	950.0986	0.338649	0.121879871	2.778546	0.0054603	0.034864	Not_Sig	no	gi|803216882|ref|XP_011968803.1|	4396	0	PREDICTED: bifunctional heparan sulfate N-deacetylase/N-sulfotransferase 1 isoform X1 [Ovis aries musimon]	gi|803216889|ref|XM_012113417.1|	6521	0	PREDICTED: Ovis aries musimon N-deacetylase/N-sulfotransferase (heparan glucosaminyl) 1 (NDST1), transcript variant X3, mRNA	P52848	1680	0	Bifunctional heparan sulfate N-deacetylase/N-sulfotransferase 1	.	.	.	.	pfam12062	HSNSD	heparan sulfate-N-deacetylase. This family of proteins is are heparan sulfate N-deacetylase enzymes. This protein is found in	GO:0035904|aorta development;GO:0003279|cardiac septum development;GO:0060976|coronary vasculature development;GO:0048702|embryonic neurocranium morphogenesis;GO:0048703|embryonic viscerocranium morphogenesis;GO:0008543|fibroblast growth factor receptor signaling pathway;GO:0030900|forebrain development;GO:0006024|glycosaminoglycan biosynthetic process;GO:0015012|heparan sulfate proteoglycan biosynthetic	GO:0000139|Golgi membrane;GO:0016021|integral component of membrane;	GO:0015016|[heparan sulfate]-glucosamine N-sulfotransferase	K02576	NDST1; heparan sulfate N-deacetylase/N-sulfotransferase
XDH	7997.374	0.23264	0.08373753	2.778206	0.005466	0.034864	Not_Sig	no	gi|742227999|ref|XP_010828106.1|	6761	0	PREDICTED: xanthine dehydrogenase/oxidase isoform X2 [Bison bison bison]	gi|742227998|ref|XM_010829804.1|	4587	0	PREDICTED: Bison bison bison xanthine dehydrogenase (XDH), transcript variant X2, mRNA	P80457	2609	0	Xanthine dehydrogenase/oxidase	PA1523	588	0	COG4631	pfam02738	Ald_Xan_dh_C2	Molybdopterin-binding domain of aldehyde dehydrogenase.	GO:0009115|xanthine catabolic process;	GO:0005829|cytosol;GO:0005615|extracellular space;GO:0005777|peroxisome;	GO:0051537|2 iron, 2 sulfur cluster binding;GO:0009055|electron carrier	K00106	XDH; xanthine dehydrogenase/oxidase [EC:1.17.1.4 1.17.3.2]
LRRC41	1252.488	0.339057	0.122045899	2.778107	0.0054677	0.034864	Not_Sig	no	gi|55731310|emb|CAH92369.1|	3098	0	hypothetical protein [Pongo abelii]	gi|555964430|ref|XM_005894222.1|	2720	0	PREDICTED: Bos mutus leucine rich repeat containing 41 (LRRC41), mRNA	Q29RR1	1197	0	Leucine-rich repeat-containing protein 41	.	.	.	.	pfam09770	PAT1	Topoisomerase II-associated protein PAT1. Members of this family are necessary for accurate chromosome transmission during	.	GO:0005737|cytoplasm;GO:0005634|nucleus;	GO:0042803|protein homodimerization activity;	K10347	LRRC41, MUF1; leucine-rich repeat-containing protein 41
RNF141	224.0819	-0.46638	0.167876536	-2.7781	0.0054679	0.034864	Not_Sig	no	gi|594043040|ref|XP_006046041.1|	1463	0	PREDICTED: RING finger protein 141 isoform X1 [Bubalus bubalis]	gi|742226091|ref|XM_010829331.1|	3637	0	PREDICTED: Bison bison bison ring finger protein 141 (RNF141), transcript variant X2, mRNA	Q32L15	482	2e-159	RING finger protein 141	.	.	.	.	pfam13639	zf-RING_2	Ring finger domain.	GO:0051865|protein autoubiquitination;GO:0006355|regulation of transcription, DNA-templated;	GO:0016020|membrane;	GO:0003677|DNA binding;GO:0004842|ubiquitin-protein transferase	.	.
C3H1orf52	197.306	-0.46785	0.168463411	-2.77715	0.0054837	0.034946	Not_Sig	no	gi|528943966|ref|XP_005204437.1|	755	7e-95	PREDICTED: UPF0690 protein C1orf52 homolog isoform X1 [Bos taurus]	gi|594115696|ref|XR_329519.1|	1047	0	PREDICTED: Bubalus bubalis chromosome unknown open reading frame, human C1orf52 (LOC102396984), transcript variant X3, misc_RNA	Q32LF5	262	2e-84	UPF0690 protein C1orf52 homolog	.	.	.	.	pfam15559	DUF4660	Domain of unknown function (DUF4660). This family of proteins is found in eukaryotes. Proteins in this family are typically between	.	GO:0005634|nucleus;	GO:0044822|poly(A) RNA binding;	.	.
SRRM1	1278.844	0.318412	0.114688854	2.776308	0.005498	0.035012	Not_Sig	no	gi|602694600|ref|XP_007459367.1|	318	1e-26	PREDICTED: serine/arginine repetitive matrix protein 1-like [Lipotes vexillifer]	gi|742229043|ref|XM_010830067.1|	3360	0	PREDICTED: Bison bison bison serine/arginine repetitive matrix 1 (SRRM1), transcript variant X3, mRNA	Q8IYB3	125	1e-27	Serine/arginine repetitive matrix protein 1	.	.	.	.	pfam01480	PWI	PWI domain.	GO:0031124|mRNA 3'-end processing;GO:0006406|mRNA export from nucleus;GO:0000398|mRNA splicing, via spliceosome;GO:0006405|RNA export from nucleus;GO:0008380|RNA splicing;GO:0000375|RNA splicing, via transesterification reactions;GO:0006369|termination of RNA polymerase II transcription;	GO:0071013|catalytic step 2 spliceosome;GO:0005829|cytosol;GO:0016363|nuclear matrix;GO:0016607|nuclear speck;GO:0005654|nucleoplasm;GO:000563	GO:0003677|DNA binding;GO:0044822|poly(A) RNA binding;	K13171	SRRM1, SRM160; serine/arginine repetitive matrix protein 1
SCRIB	641.7054	0.372004	0.133998486	2.776184	0.0055001	0.035012	Not_Sig	no	gi|741949528|ref|XP_010810182.1|	6357	0	PREDICTED: protein scribble homolog isoform X1 [Bos taurus]	gi|982944285|ref|XM_010811880.2|	4999	0	PREDICTED: Bos taurus scribbled planar cell polarity protein (SCRIB), transcript variant X1, mRNA	Q80U72	2082	0	Protein scribble homolog	alr0124_1	162	1.26e-41	COG4886	pfam00595	PDZ	PDZ domain (Also known as DHR or GLGF). PDZ domains are found in diverse signaling proteins.	GO:0090630|activation of GTPase activity;GO:0060561|apoptotic process involved in morphogenesis;GO:0043615|astrocyte cell migration;GO:0008105|asymmetric protein localization;GO:0002093|auditory receptor cell morphogenesis;GO:0060088|auditory receptor cell stereocilium organization;GO:0016477|cell migration;GO:0008283|cell proliferation;GO:0021747|cochlear nucleus development;GO:0035089|establishment of apical/basal cell polarity;GO:0060603|mammary gland duct	GO:0016323|basolateral plasma membrane;GO:0030054|cell junction;GO:0005913|cell-cell adherens junction;GO:0044291|cell-cell contact	GO:0098641|cadherin binding involved in cell-cell adhesion;	K16175	SCRIB; protein scribble
DPY19L3	272.1965	-0.38726	0.139539487	-2.77531	0.005515	0.035064	Not_Sig	no	gi|297485437|ref|XP_002694965.1|	3405	0	PREDICTED: probable C-mannosyltransferase DPY19L3 isoform X1 [Bos taurus]	gi|965809343|ref|XM_012120303.2|	4737	0	PREDICTED: Ovis aries musimon dpy-19-like 3 (C. elegans) (DPY19L3), transcript variant X1, mRNA	Q6ZPD9	1221	0	Probable C-mannosyltransferase DPY19L3	.	.	.	.	pfam10034	Dpy19	Q-cell neuroblast polarisation. Dyp-19, formerly known as DUF2211, is a transmembrane domain family that is required to	GO:0018406|protein C-linked glycosylation via 2'-alpha-mannosyl-L-tryptophan;	GO:0016021|integral component of membrane;GO:0005637|nuclear inner membrane;	GO:0000030|mannosyltransferase activity;	.	.
TSPAN31	851.4019	-0.29949	0.107928433	-2.77493	0.0055214	0.035064	Not_Sig	no	gi|759186298|ref|XP_011381197.1|	955	2e-124	PREDICTED: tetraspanin-31 [Pteropus vampyrus]	gi|942072145|ref|XM_005901665.2|	1219	0	PREDICTED: Bos mutus tetraspanin 31 (TSPAN31), transcript variant X1, mRNA	Q32KP1	356	8e-121	Tetraspanin-31	.	.	.	.	pfam00335	Tetraspannin	Tetraspanin family.	GO:0007166|cell surface receptor signaling pathway;	GO:0005887|integral component of plasma membrane;	.	K17356	TSPAN13_31; tetraspanin-13/31
FADS2	3740.532	0.299498	0.107930499	2.774911	0.0055217	0.035064	Not_Sig	no	gi|742179168|ref|XP_010852339.1|	2290	0	PREDICTED: fatty acid desaturase 2 [Bison bison bison]	gi|742179167|ref|XM_010854037.1|	3077	0	PREDICTED: Bison bison bison fatty acid desaturase 2 (FADS2), mRNA	A4FV48	882	0	Fatty acid desaturase 2	SPAC1F12.10	61.6	2.80e-10	COG5274	pfam00487	FA_desaturase	Fatty acid desaturase.	GO:0006636|unsaturated fatty acid biosynthetic process;	GO:0005789|endoplasmic reticulum membrane;GO:0016021|integral component of membrane;	GO:0004768|stearoyl-CoA 9-desaturase activity;	K10226	FADS2; fatty acid desaturase 2 (delta-6 desaturase) [EC:1.14.19.-]
SNAPC1	425.4328	-0.3947	0.142240045	-2.77488	0.0055223	0.035064	Not_Sig	no	gi|555957246|ref|XP_005890757.1|	1740	0	PREDICTED: snRNA-activating protein complex subunit 1 [Bos mutus]	gi|850484816|gb|CP011892.1|	4508	0	Ovis canadensis canadensis isolate 43U chromosome 7 sequence	Q4R6W9	565	0	snRNA-activating protein complex subunit 1	.	.	.	.	pfam09808	SNAPc_SNAP43	Small nuclear RNA activating complex (SNAPc), subunit SNAP43. Members of this family are part of the SNAPc complex required for	GO:0006355|regulation of transcription, DNA-templated;GO:0006351|transcription, DNA-templated;	GO:0005634|nucleus;	GO:0003677|DNA binding;	K15208	SNAPC1; snRNA-activating protein complex subunit 1
TSEN34	246.0245	0.412471	0.14865739	2.774642	0.0055262	0.035064	Not_Sig	no	gi|594666759|ref|XP_007181454.1|	1554	0	PREDICTED: LOW QUALITY PROTEIN: tRNA-splicing endonuclease subunit Sen34 [Balaenoptera acutorostrata scammoni]	gi|77735456|ref|NM_001034251.1|	1340	0	Bos taurus tRNA splicing endonuclease subunit 34 (TSEN34), mRNA	Q9BSV6	536	0	tRNA-splicing endonuclease subunit Sen34	YAR008w	79.3	9.72e-16	COG1676	pfam01974	tRNA_int_endo	tRNA intron endonuclease, catalytic C-terminal domain. Members of this family cleave pre tRNA at the 5' and 3' splice sites to	GO:0006397|mRNA processing;GO:0006388|tRNA splicing, via endonucleolytic cleavage and ligation;GO:0000379|tRNA-type intron splice site recognition and cleavage;	GO:0005730|nucleolus;GO:0005654|nucleoplasm;GO:0005634|nucleus;GO:0000214|tRNA-intron endonuclease complex;	GO:0016829|lyase activity;GO:0003676|nucleic acid binding;GO:0000213	K15323	TSEN34; tRNA-splicing endonuclease subunit Sen34
HNRNPA3	4441.282	-0.35493	0.127920444	-2.77462	0.0055266	0.035064	Not_Sig	no	gi|795174449|ref|XP_011799421.1|	1021	2e-122	PREDICTED: heterogeneous nuclear ribonucleoprotein A3 isoform X3 [Colobus angolensis palliatus]	gi|742201875|ref|XM_010859783.1|	5448	0	PREDICTED: Bison bison bison heterogeneous nuclear ribonucleoprotein A3 (HNRNPA3), transcript variant X1, mRNA	P51991	384	2e-118	Heterogeneous nuclear ribonucleoprotein A3	.	.	.	.	pfam00076	RRM_1	RNA recognition motif. (a.k.a. RRM, RBD, or RNP domain). The RRM motif is probably diagnostic of an RNA binding protein. RRMs	GO:0010467|gene expression;GO:0000398|mRNA splicing, via spliceosome;GO:0051028|mRNA transport;	GO:0071013|catalytic step 2 spliceosome;GO:0005737|cytoplasm;GO:0030529|ribonucleoprotein complex;GO:1990124|messenger	GO:0003729|mRNA binding;GO:0000166|nucleotide binding;GO:0044822	K12741	HNRNPA1_3; heterogeneous nuclear ribonucleoprotein
PRICKLE3	229.6102	0.488759	0.176225373	2.773489	0.0055459	0.035167	Not_Sig	no	gi|555951008|ref|XP_005887704.1|	2479	0	PREDICTED: prickle-like protein 3 isoform X1 [Bos mutus]	gi|942032442|ref|XM_005887642.2|	2202	0	PREDICTED: Bos mutus prickle homolog 3 (PRICKLE3), transcript variant X1, mRNA	O43900	786	0	Prickle-like protein 3	.	.	.	.	pfam06297	PET	PET Domain. This domain is suggested to be involved in protein-protein interactions. The family is found in conjunction with	.	.	GO:0008270|zinc ion binding;	.	.
AP5B1	208.8475	0.490764	0.176993304	2.772784	0.0055579	0.035205	Not_Sig	no	gi|555964057|ref|XP_005894100.1|	2851	0	PREDICTED: AP-5 complex subunit beta-1 [Bos mutus]	gi|594065032|ref|XM_006056454.1|	2850	0	PREDICTED: Bubalus bubalis adaptor-related protein complex 5, beta 1 subunit (AP5B1), mRNA	G3MZC5	1094	0	AP-5 complex subunit beta-1	.	.	.	.	pfam07793	DUF1631	Protein of unknown function (DUF1631). The members of this family are sequences derived from a group of hypothetical proteins	GO:0016197|endosomal transport;GO:0015031|protein transport;	GO:0030119|AP-type membrane coat adaptor complex;GO:0005765|lysosomal membrane;	.	K19022	AP5B1; AP-5 complex subunit beta-1
STX7	846.5956	-0.31526	0.113698942	-2.77278	0.005558	0.035205	Not_Sig	no	gi|118150840|ref|NP_001071332.1|	1160	5e-151	syntaxin-7 [Bos taurus]	gi|742117173|ref|XM_010839687.1|	2183	0	PREDICTED: Bison bison bison syntaxin 7 (STX7), mRNA	Q3ZBT5	451	2e-152	Syntaxin-7	SPBC31E1.04	78.2	7.32e-15	COG5325	pfam14523	Syntaxin_2	Syntaxin-like protein. This domain includes syntaxin-like domains including from the Vam3p protein.	GO:0006886|intracellular protein transport;GO:0070925|organelle assembly;GO:0051640|organelle localization;GO:1902685|positive regulation of receptor localization to synapse;GO:0001916|positive regulation of T cell mediated cytotoxicity;GO:1903076|regulation of protein localization to plasma membrane;GO:0048278|vesicle docking;GO:0006906|vesicle fusion;	GO:0042582|azurophil granule;GO:0031901|early endosome membrane;GO:0030139|endocytic vesicle;GO:0012505|endomembrane	GO:0019869|chloride channel inhibitor activity;GO:0005484|SNAP receptor	K08488	STX7; syntaxin 7
TTLL3	184.2774	0.455915	0.164443668	2.772469	0.0055633	0.035219	Not_Sig	no	gi|982961569|ref|XP_015314984.1|	3829	0	PREDICTED: LOW QUALITY PROTEIN: tubulin monoglycylase TTLL3 [Bos taurus]	gi|983004436|ref|XM_015468703.1|	2464	0	PREDICTED: Bos taurus tubulin tyrosine ligase-like family, member 3 (TTLL3), mRNA	A4Q9E5	1135	0	Tubulin monoglycylase TTLL3	.	.	.	.	pfam03133	TTL	Tubulin-tyrosine ligase family. Tubulins and microtubules are subjected to several post-translational modifications of	GO:0035082|axoneme assembly;GO:0042384|cilium assembly;GO:0018094|protein polyglycylation;	GO:0005930|axoneme;GO:0005929|cilium;GO:0005737|cytoplasm;GO:0005874|microtubule;GO:0015630|microtubule cytoskeleton;	GO:0005524|ATP binding;GO:0070735|protein-glycine ligase	K16608	TTLL3_8; tubulin monoglycylase TTLL3/8 [EC:6.3.2.-]
LOC106700841	151.4894	-0.5042	0.181871198	-2.77227	0.0055667	0.035221	Not_Sig	no	.	.	.	.	gi|308153303|ref|NR_036663.1|	726	0	Bos taurus small nucleolar RNA host gene 3 (non-protein coding) (SNHG3), long non-coding RNA	.	.	.	.	.	.	.	.	pfam00922	Phosphoprotein	Vesiculovirus phosphoprotein.	.	.	.	.	.
CNEP1R1	455.8293	-0.40672	0.146733322	-2.77184	0.0055741	0.035248	Not_Sig	no	gi|77735559|ref|NP_001029475.1|	671	1e-81	nuclear envelope phosphatase-regulatory subunit 1 [Bos taurus]	gi|555981584|ref|XM_005902645.1|	1809	0	PREDICTED: Bos mutus CTD nuclear envelope phosphatase 1 regulatory subunit 1 (CNEP1R1), transcript variant X2, mRNA	Q5R7J7	263	4e-83	Nuclear envelope phosphatase-regulatory subunit 1	.	.	.	.	pfam09771	Tmemb_18A	Transmembrane protein 188. The function of this family of transmembrane proteins has not, as yet, been determined.	GO:0006629|lipid metabolic process;GO:0035307|positive regulation of protein dephosphorylation;GO:0010867|positive regulation of triglyceride biosynthetic process;GO:0034504|protein localization to nucleus;	GO:0005737|cytoplasm;GO:0016021|integral component of membrane;GO:0071595|Nem1-Spo7 phosphatase complex;GO:0031965|nuclear	.	.	.
PLXNB1	516.8555	0.356456	0.128618776	2.771419	0.0055813	0.035274	Not_Sig	no	gi|741969482|ref|XP_010816169.1|	9924	0	PREDICTED: LOW QUALITY PROTEIN: plexin-B1 [Bos taurus]	gi|983004738|ref|XM_003583823.4|	7215	0	PREDICTED: Bos taurus plexin B1 (PLXNB1), mRNA	O43157	3489	0	Plexin-B1	.	.	.	.	pfam08337	Plexin_cytopl	Plexin cytoplasmic RasGAP domain. This family features the C-terminal regions of various plexins. Plexins are receptors for	GO:0048675|axon extension;GO:0016477|cell migration;GO:0035556|intracellular signal transduction;GO:0007162|negative regulation of cell adhesion;GO:0033689|negative regulation of osteoblast proliferation;GO:0048812|neuron projection morphogenesis;GO:0043931|ossification involved in bone maturation;GO:0050772|positive regulation of axonogenesis;GO:0043547|positive regulation of GTPase activity;GO:0014068|positive regulation of phosphatidylinositol 3-kinase signaling;GO:0030334|regulation of cell	GO:0005576|extracellular region;GO:0005887|integral component of plasma membrane;GO:0005622|intracellular;GO:00	GO:0032794|GTPase activating protein binding;GO:0005096|GTPase activator	K06821	PLXNB; plexin B
PIGF	1119.815	-0.32883	0.118664925	-2.77111	0.0055865	0.035288	Not_Sig	no	gi|803030612|ref|XP_012014542.1|	1041	8e-139	PREDICTED: phosphatidylinositol-glycan biosynthesis class F protein isoform X5 [Ovis aries]	gi|555967251|ref|XM_005895612.1|	983	0	PREDICTED: Bos mutus phosphatidylinositol glycan anchor biosynthesis, class F (PIGF), transcript variant X1, mRNA	Q07326	362	1e-123	Phosphatidylinositol-glycan biosynthesis class F protein	.	.	.	.	pfam06699	PIG-F	GPI biosynthesis protein family Pig-F. PIG-F is involved in glycosylphosphatidylinositol (GPI) anchor biosynthesis.	GO:0006506|GPI anchor biosynthetic process;GO:0016254|preassembly of GPI anchor in ER membrane;	GO:0005789|endoplasmic reticulum membrane;GO:0016021|integral component of membrane;	GO:0004307|ethanolaminephosphotransferase activity;GO:0016491	K05287	PIGF; phosphatidylinositol glycan, class F [EC:2.7.-.-]
LRRC40	287.1009	-0.44666	0.161198454	-2.7709	0.0055902	0.035292	Not_Sig	no	gi|375364744|ref|NP_001096732.2|	2917	0	leucine-rich repeat-containing protein 40 [Bos taurus]	gi|742160272|ref|XM_010848759.1|	4330	0	PREDICTED: Bison bison bison leucine rich repeat containing 40 (LRRC40), mRNA	Q9H9A6	1034	0	Leucine-rich repeat-containing protein 40	alr0124_1	112	4.78e-25	COG4886	pfam13855	LRR_8	Leucine rich repeat.	.	GO:0016020|membrane;	.	.	.
TEAD4	603.5486	0.340403	0.122870753	2.770419	0.0055984	0.035325	Not_Sig	no	gi|803272436|ref|XP_011995913.1|	2138	0	PREDICTED: transcriptional enhancer factor TEF-3 isoform X3 [Ovis aries musimon]	gi|965851937|ref|XM_012140523.2|	2185	0	PREDICTED: Ovis aries musimon TEA domain family member 4 (TEAD4), transcript variant X3, mRNA	Q15561	767	0	Transcriptional enhancer factor TEF-3	.	.	.	.	pfam01285	TEA	TEA/ATTS domain family.	GO:0035329|hippo signaling;GO:0007517|muscle organ development;GO:0006357|regulation of transcription from RNA polymerase II promoter;GO:0001501|skeletal system development;GO:0006367|transcription initiation from RNA polymerase II promoter;	GO:0005654|nucleoplasm;	GO:0003677|DNA binding;GO:0003700|transcription factor activity, sequence-	K09448	TEAD; transcriptional enhancer factor
RPL7A	7255.326	-0.28524	0.102990102	-2.76963	0.0056121	0.035391	Not_Sig	no	gi|94966839|ref|NP_001035610.1|	1173	2e-159	60S ribosomal protein L7a [Bos taurus]	gi|942058921|ref|XM_005897428.2|	881	0	PREDICTED: Bos mutus ribosomal protein L7a (RPL7A), mRNA	Q2TBQ5	456	5e-161	60S ribosomal protein L7a	SPBC29A3.04	284	1.03e-95	COG1358	pfam01248	Ribosomal_L7Ae	Ribosomal protein L7Ae/L30e/S12e/Gadd45 family. This family includes: Ribosomal L7A from metazoa, Ribosomal L8-A	GO:0000470|maturation of LSU-rRNA;GO:0006412|translation;	GO:0022625|cytosolic large ribosomal subunit;GO:0070062|extracellular exosome;GO:0005925|focal adhesion;GO:0016020|membrane;GO:0005	GO:0044822|poly(A) RNA binding;GO:0003723|RNA binding;	K02936	RP-L7Ae, RPL7A; large subunit ribosomal protein L7Ae
CBARP	110.6317	0.654327	0.236349664	2.768472	0.005632	0.035468	UP	yes	gi|803222546|ref|XP_011971547.1|	2330	0	PREDICTED: protein Dos isoform X3 [Ovis aries musimon]	gi|965800680|ref|XM_012116158.2|	2654	0	PREDICTED: Ovis aries musimon calcium channel, voltage-dependent, beta subunit associated regulatory protein (CBARP), transcript variant X5, mRNA	Q8N350	523	3e-170	Voltage-dependent calcium channel beta subunit-associated regulatory protein	.	.	.	.	pfam12705	PDDEXK_1	PD-(D/E)XK nuclease superfamily. Members of this family belong to the PD-(D/E)XK nuclease superfamily	GO:1903170|negative regulation of calcium ion transmembrane transport;GO:0045955|negative regulation of calcium ion-dependent exocytosis;GO:1901386|negative regulation of voltage-gated calcium channel activity;	GO:0030054|cell junction;GO:0030426|growth cone;GO:0016021|integral component of membrane;GO:0005886|plasma	GO:0044325|ion channel binding;	.	.
STXBP1	1424.796	0.349171	0.126125645	2.768438	0.0056326	0.035468	Not_Sig	no	gi|537149757|gb|ERE71210.1|	3057	0	syntaxin-binding protein 1 [Cricetulus griseus]	gi|982939711|ref|XM_005213174.3|	3902	0	PREDICTED: Bos taurus syntaxin binding protein 1 (STXBP1), transcript variant X1, mRNA	P61765	1107	0	Syntaxin-binding protein 1	SPCC584.05	234	3.26e-64	COG5158	pfam00995	Sec1	Sec1 family.	GO:0007412|axon target recognition;GO:0060292|long term synaptic depression;GO:0043524|negative regulation of neuron apoptotic process;GO:0031333|negative regulation of protein complex assembly;GO:0035544|negative regulation of SNARE complex assembly;GO:0032229|negative regulation of synaptic transmission, GABAergic;GO:0007274|neuromuscular synaptic transmission;GO:0007269|neurotransmitter secretion;GO:0070527|platelet aggregation;GO:0002576|platelet	GO:0005737|cytoplasm;GO:0005829|cytosol;GO:0070062|extracellular exosome;GO:0005739|mitochondrion;GO:0043209|myelin	GO:0042802|identical protein binding;GO:0044822|poly(A) RNA	K15292	STXBP1, MUNC18-1; syntaxin-binding protein 1
FES	294.0369	0.401464	0.145018979	2.768357	0.005634	0.035468	Not_Sig	no	gi|73853464|ref|NP_001027471.1|	3922	0	tyrosine-protein kinase Fes/Fps [Bos taurus]	gi|73853463|ref|NM_001032300.1|	2724	0	Bos taurus FES proto-oncogene, tyrosine kinase (FES), mRNA	P14238	1402	0	Tyrosine-protein kinase Fes/Fps	.	.	.	.	pfam07714	Pkinase_Tyr	Protein tyrosine kinase.	GO:0007155|cell adhesion;GO:0030154|cell differentiation;GO:0006935|chemotaxis;GO:0007173|epidermal growth factor receptor signaling pathway;GO:0045087|innate immune response;GO:0038083|peptidyl-tyrosine autophosphorylation;GO:0018108|peptidyl-tyrosine phosphorylation;GO:2000251|positive regulation of actin cytoskeleton reorganization;GO:0031116|positive regulation of microtubule polymerization;GO:0045639|positive regulation of myeloid cell differentiation;GO:0010976|positive regulation of neuron projection	GO:0005737|cytoplasm;GO:0016023|cytoplasmic membrane-bounded vesicle;GO:0005829|cytosol;GO:0031234|extrinsic component of cytoplasmic side of	GO:0005524|ATP binding;GO:0034987|immunoglobulin receptor	.	.
COASY	296.7282	0.426115	0.15393852	2.768086	0.0056387	0.035468	Not_Sig	no	gi|296476421|tpg|DAA18536.1|	2898	0	TPA: Coenzyme A synthase [Bos taurus]	gi|983002144|ref|XM_010800548.2|	2149	0	PREDICTED: Bos taurus Coenzyme A synthase (COASY), mRNA	Q8MIR4	1048	0	Bifunctional coenzyme A synthase	BH3150	116	5.54e-29	COG0237	pfam01121	CoaE	Dephospho-CoA kinase. This family catalyses the phosphorylation of the 3'-hydroxyl group of dephosphocoenzyme A to form	GO:0015937|coenzyme A biosynthetic process;	GO:0070062|extracellular exosome;GO:0005759|mitochondrial matrix;GO:0005654|nucleoplasm;	GO:0005524|ATP binding;GO:0004140|dephospho-CoA kinase	K02318	COASY; phosphopantetheine adenylyltransferase / dephospho-CoA
SDC2	512.3862	-0.32324	0.116779199	-2.76794	0.0056412	0.035468	Not_Sig	no	gi|742090946|ref|XP_010831778.1|	826	3e-103	PREDICTED: syndecan-2 [Bison bison bison]	gi|742090945|ref|XM_010833476.1|	1882	0	PREDICTED: Bison bison bison syndecan 2 (SDC2), mRNA	Q58DD4	321	3e-104	Syndecan-2	.	.	.	.	pfam01034	Syndecan	Syndecan domain. Syndecans are transmembrane heparin sulfate proteoglycans which are implicated in the binding of	GO:0016477|cell migration;GO:0048813|dendrite morphogenesis;GO:0048814|regulation of dendrite morphogenesis;	GO:0009986|cell surface;GO:0016021|integral component of membrane;	.	K16336	SDC2; syndecan 2
TUBB6	7354.075	0.336885	0.121715368	2.767812	0.0056434	0.035468	Not_Sig	no	gi|926722935|ref|XP_005709465.2|	2208	0	PREDICTED: LOW QUALITY PROTEIN: tubulin beta-6 chain [Capra hircus]	gi|114052147|ref|NM_001046373.1|	1611	0	Bos taurus tubulin beta 6 class V (TUBB6), mRNA	Q2HJ81	854	0	Tubulin beta-6 chain	ECU03g0820i	711	0	COG5023	pfam03953	Tubulin_C	Tubulin C-terminal domain. This family includes the tubulin alpha, beta and gamma chains. Members of this family are involved in	GO:0007017|microtubule-based process;	GO:0005737|cytoplasm;GO:0005874|microtubule;	GO:0005525|GTP binding;GO:0003924|GTPase activity;GO:0005200	K07375	TUBB; tubulin beta
SIX2	47.90614	0.763558	0.275884965	2.767666	0.0056459	0.035468	UP	yes	gi|511860269|ref|XP_004753047.1|	1251	1e-164	PREDICTED: homeobox protein SIX2 isoform X1 [Mustela putorius furo]	gi|982938778|ref|XM_005212654.3|	2029	0	PREDICTED: Bos taurus SIX homeobox 2 (SIX2), transcript variant X1, mRNA	Q62232	471	3e-160	Homeobox protein SIX2	.	.	.	.	pfam05920	Homeobox_KN	Homeobox KN domain. This is a homeobox transcription factor KN domain conserved from fungi to human and plants.	GO:0009948|anterior/posterior axis specification;GO:0016477|cell migration;GO:0008283|cell proliferation;GO:0002062|chondrocyte differentiation;GO:0072137|condensed mesenchymal cell proliferation;GO:0048701|embryonic cranial skeleton morphogenesis;GO:0048557|embryonic digestive tract morphogenesis;GO:0048704|embryonic skeletal system morphogenesis;GO:0001822|kidney development;GO:0072161|mesenchymal cell differentiation involved in kidney	GO:0005737|cytoplasm;GO:0031965|nuclear membrane;GO:0005654|nucleoplasm;GO:0005634|nucleus;GO:0005886|plasma	GO:0032403|protein complex binding;GO:0000978|RNA polymerase II	K19472	SIX2; homeobox protein SIX2
MTFP1	58.38891	0.699133	0.252801561	2.765541	0.0056828	0.035651	UP	yes	gi|115497128|ref|NP_001069331.1|	836	2e-109	mitochondrial fission process protein 1 [Bos taurus]	gi|594093096|ref|XM_006069814.1|	905	0	PREDICTED: Bubalus bubalis mitochondrial fission process 1 (MTFP1), transcript variant X1, mRNA	Q9UDX5	305	1e-102	Mitochondrial fission process protein 1	.	.	.	.	pfam10558	MTP18	Mitochondrial 18 KDa protein (MTP18). This family of proteins are mitochondrial 18KDa proteins that are often misannotated as	GO:0006915|apoptotic process;GO:0000266|mitochondrial fission;	GO:0016021|integral component of membrane;GO:0005743|mitochondrial inner membrane;	.	K17981	MTFP1, MTP18; mitochondrial fission process protein 1
LOC138987150	403.0745	-0.49177	0.17782366	-2.76549	0.0056838	0.035651	Not_Sig	no	gi|954470796|gb|KRZ01443.1|	795	2e-91	hypothetical protein T11_16386 [Trichinella zimbabwensis]	gi|151415227|emb|AL353644.34|	2161	0	Human DNA sequence from clone RP11-164K15 on chromosome 22, complete sequence	Q3E811	60.5	3e-09	Regulator of rDNA transcription protein 15	.	.	.	.	pfam05781	MRVI1	MRVI1 protein. This family consists of mammalian MRVI1 proteins which are related to the lymphoid-restricted membrane protein	GO:0006355|regulation of transcription, DNA-templated;GO:0006351|transcription, DNA-templated;	.	.	.	.
AKNA	250.0072	0.539412	0.195053724	2.765455	0.0056844	0.035651	Not_Sig	no	gi|555978414|ref|XP_005901149.1|	6261	0	PREDICTED: LOW QUALITY PROTEIN: AT-hook-containing transcription factor [Bos mutus]	gi|742097864|ref|XM_010835140.1|	5165	0	PREDICTED: Bison bison bison AT-hook transcription factor (AKNA), transcript variant X2, mRNA	Q7Z591	1557	0	AT-hook-containing transcription factor	.	.	.	.	pfam12443	AKNA	AT-hook-containing transcription factor. This domain family is found in eukaryotes, and is approximately 110 amino acids in	GO:0045944|positive regulation of transcription from RNA polymerase II promoter;	GO:0016020|membrane;GO:0005634|nucleus;	GO:0000978|RNA polymerase II core promoter proximal region sequence-	.	.

RPL31	4356.482	-0.33458	0.12101839	-2.76474	0.0056968	0.03571	Not_Sig	no	gi|148672373|gb|EDL04320.1|	553	1e-69	mCG49427 [Mus musculus]	gi|942069282|ref|XM_005900701.2|	451	0	PREDICTED: Bos mutus ribosomal protein L31 (RPL31), transcript variant X2, mRNA	P62902	203	4e-66	60S ribosomal protein L31	YLR406c	129	6.65e-39	COG2097	pfam01198	Ribosomal_L31e	Ribosomal protein L31e.	GO:0002181|cytoplasmic translation;	GO:0022625|cytosolic large ribosomal subunit;GO:0005730|nucleolus;GO:0005654|nucleoplasm;	GO:0003735|structural constituent of ribosome;	K02910	RP-L31e, RPL31; large subunit ribosomal protein L31e
CLIP3	554.5116	0.462861	0.167653144	2.760823	0.0057656	0.036121	Not_Sig	no	gi|958686651|ref|XP_014693788.1|	2581	0	PREDICTED: CAP-Gly domain-containing linker protein 3 isoform X1 [Equus asinus]	gi|594090858|ref|XM_006068764.1|	2589	0	PREDICTED: Bubalus bubalis CAP-GLY domain containing linker protein 3 (CLIP3), mRNA	Q96DZ5	936	0	CAP-Gly domain-containing linker protein 3	SPAC27D7.13c	74.7	6.72e-13	COG5244	pfam01302	CAP_GLY	CAP-Gly domain. Cytoskeleton-associated proteins (CAPs) are involved in the organisation of microtubules and transportation of	GO:0072321|chaperone-mediated protein transport;GO:0045444|fat cell differentiation;GO:0044091|membrane biogenesis;GO:0031115|negative regulation of microtubule polymerization;GO:0018230|peptidyl-L-cysteine S-palmitoylation;GO:0043065|positive regulation of apoptotic process;GO:0045807|positive regulation of endocytosis;GO:0090004|positive regulation of establishment of protein localization to plasma membrane;GO:0010828|positive regulation of glucose	GO:0005829|cytosol;GO:0031901|early endosome membrane;GO:0005795|Golgi stack;GO:0045121|membrane raft;GO:0005886|plasma	GO:0035594|ganglioside binding;GO:0008017|microtubule	K10423	CLIP3_4; CAP-Gly domain-containing linker protein 3/4
PIN4	95.97085	-0.69979	0.25354889	-2.75997	0.0057806	0.036196	DOWN	yes	gi|440898939|gb|ELR50334.1|	632	3e-81	Peptidyl-prolyl cis-trans isomerase NIMA-interacting 4, partial [Bos mutus]	gi|942079448|ref|XM_005904024.2|	516	0	PREDICTED: Bos mutus peptidylprolyl cis/trans isomerase, NIMA-interacting 4 (PIN4), mRNA	A6QPY8	214	6e-70	Peptidyl-prolyl cis-trans isomerase NIMA-interacting 4	SMc02451	75.9	3.66e-16	COG0760	pfam00639	Rotamase	PPIC-type PPIASE domain. Rotamases increase the rate of protein folding by catalyzing the interconversion of cis-proline and	.	GO:0005737|cytoplasm;GO:0070062|extracellular exosome;GO:0005730|nucleolus;GO:0005819|spindle;	GO:0003677|DNA binding;GO:0003755|peptidyl-prolyl cis-trans isomerase	K09579	PIN4; peptidyl-prolyl cis-trans isomerase NIMA-interacting 4 [EC:5.2.1.8]
NDUFB5	623.9734	-0.37667	0.136505977	-2.75935	0.0057917	0.036245	Not_Sig	no	gi|28603776|ref|NP_788829.1|	783	3e-100	NADH dehydrogenase [ubiquinone] 1 beta subcomplex subunit 5, mitochondrial precursor [Bos taurus]	gi|942045709|ref|XM_005892507.2|	1144	0	PREDICTED: Bos mutus NADH dehydrogenase (ubiquinone) 1 beta subcomplex, 5, 16kDa (NDUFB5), transcript variant X1, mRNA	Q02380	306	1e-101	NADH dehydrogenase [ubiquinone] 1 beta subcomplex subunit 5, mitochondrial	.	.	.	.	pfam09781	NDUF_B5	NADH:ubiquinone oxidoreductase, NDUFB5/SGDH subunit. Members of this family mediate the transfer of electrons from NADH to the	GO:0055114|oxidation-reduction process;	GO:0016021|integral component of membrane;GO:0005747|mitochondrial respiratory chain complex I;GO:0005654|nucleoplasm;	.	K03961	NDUFB5; NADH dehydrogenase (ubiquinone) 1 beta subcomplex subunit
KIF24	103.4514	0.548615	0.198867893	2.758691	0.0058033	0.036298	Not_Sig	no	gi|742119012|ref|XP_010838412.1|	6964	0	PREDICTED: kinesin-like protein KIF24 [Bison bison bison]	gi|982934027|ref|XM_005210319.3|	4528	0	PREDICTED: Bos taurus kinesin family member 24 (KIF24), transcript variant X1, mRNA	Q5T7B8	1934	0	Kinesin-like protein KIF24	ECU11g0470	262	1.10e-74	COG5059	pfam00225	Kinesin	Kinesin motor domain.	GO:0042384|cilium assembly;GO:0007019|microtubule depolymerization;GO:0007018|microtubule-based movement;	GO:0005814|centriole;GO:0005829|cytosol;GO:0005871|kinesin complex;GO:0005874|microtubule;GO:0043234|protein complex;	GO:0005524|ATP binding;GO:0016887|ATPase activity;GO:0003777	K10393	KIF2_24, MCAK; kinesin family member 2/24
ERCC2	346.666	0.370539	0.134376517	2.757469	0.0058251	0.036415	Not_Sig	no	gi|157168345|ref|NP_001096787.1|	3972	0	TFIIH basal transcription factor complex helicase XPD subunit [Bos taurus]	gi|742173955|ref|XM_010852594.1|	2833	0	PREDICTED: Bison bison bison excision repair cross-complementation group 2 (ERCC2), mRNA	A6QLJ0	1529	0	TFIIH basal transcription factor complex helicase XPD subunit	SPAC1D4.12	892	0	COG1199	pfam06777	DUF1227	Protein of unknown function (DUF1227). This family represents a conserved region within a number of eukaryotic DNA repair	GO:0007059|chromosome segregation;GO:0035315|hair cell differentiation;GO:0033683|nucleotide-excision repair, DNA incision;GO:0045944|positive regulation of transcription from RNA polymerase II promoter;GO:0045893|positive regulation of transcription, DNA-templated;GO:0006468|protein phosphorylation;GO:1901990|regulation of mitotic cell cycle phase transition;GO:0006979|response to oxidative stress;GO:0006366|transcription from RNA polymerase II promoter;	GO:0005737|cytoplasm;GO:0005675|holo TFIIH complex;GO:0071817|MMXD complex;GO:0005634|nucleus;GO:0005819|spindle;	GO:0051539|4 iron, 4 sulfur cluster binding;GO:0043139|5'-3' DNA helicase	K10844	ERCC2, XPD; DNA excision repair protein ERCC-2 [EC:3.6.4.12]
CD47	3946.243	-0.3434	0.124565343	-2.75679	0.0058371	0.036435	Not_Sig	no	gi|528936232|ref|XP_005201321.1|	1305	5e-163	PREDICTED: leukocyte surface antigen CD47 isoform X4 [Bos taurus]	gi|982909975|ref|XM_005201264.3|	4473	0	PREDICTED: Bos taurus CD47 molecule (CD47), transcript variant X4, mRNA	Q9N0K1	485	1e-156	Leukocyte surface antigen CD47	.	.	.	.	pfam04549	CD47	CD47 transmembrane region. This family represents the transmembrane region of CD47 leukocyte antigen.	GO:0007155|cell adhesion;	GO:0016021|integral component of membrane;GO:0005886|plasma membrane;	.	.	.
MRPL50	198.3943	-0.45994	0.166841498	-2.75677	0.0058375	0.036435	Not_Sig	no	gi|114051364|ref|NP_001039645.1|	775	1e-100	39S ribosomal protein L50, mitochondrial [Bos taurus]	gi|594088998|ref|XM_006067873.1|	857	0	PREDICTED: Bubalus bubalis mitochondrial ribosomal protein L50 (MRPL50), mRNA	Q2KI49	303	4e-102	39S ribosomal protein L50, mitochondrial	.	.	.	.	pfam10501	Ribosomal_L50	Ribosomal subunit 39S. The 39S ribosomal protein appears to be a subunit of one of the larger mitochondrial 66S or 70S units.	GO:0070125|mitochondrial translational elongation;GO:0070124|mitochondrial translational initiation;	GO:0005743|mitochondrial inner membrane;GO:0005840|ribosome;	.	K17431	MRPL50; large subunit ribosomal protein L50
ZBTB10	186.6422	-0.45759	0.165988619	-2.75676	0.0058378	0.036435	Not_Sig	no	gi|803291832|ref|XP_012005571.1|	3715	0	PREDICTED: zinc finger and BTB domain-containing protein 10 [Ovis aries musimon]	gi|965875048|ref|XM_012150181.2|	3690	0	PREDICTED: Ovis aries musimon zinc finger and BTB domain containing 10 (ZBTB10), mRNA	Q96DT7	1333	0	Zinc finger and BTB domain-containing protein 10	.	.	.	.	pfam00651	BTB	BTB/POZ domain. The BTB (for BR-C, ttk and bab) or POZ (for Pox virus and Zinc finger) domain is present near the N-terminus of a	GO:0006355|regulation of transcription, DNA-templated;GO:0006351|transcription, DNA-templated;	GO:0005654|nucleoplasm;	GO:0003677|DNA binding;GO:0046872|metal ion binding;	K10497	ZBTB10, RINZF; zinc finger and BTB domain-containing protein 10
PRXL2B	711.0938	-0.32788	0.118984262	-2.75566	0.0058574	0.036537	Not_Sig	no	gi|926711570|ref|XP_005690822.2|	978	5e-131	PREDICTED: prostamide/prostaglandin F synthase [Capra hircus]	gi|942035641|ref|XM_005888842.2|	815	0	PREDICTED: Bos mutus family with sequence similarity 213, member B (FAM213B), mRNA	Q58CY6	364	5e-126	Prostamide/prostaglandin F synthase	.	.	.	.	pfam13911	AhpC-TSA_2	AhpC/TSA antioxidant enzyme. This family contains proteins related to alkyl hydro-peroxide reductase (AhpC) and thiol specific	GO:0001516|prostaglandin biosynthetic process;	GO:0005737|cytoplasm;GO:0005829|cytosol;GO:0005783|endoplasmic reticulum;GO:0043209|myelin sheath;	GO:0016616|oxidoreductase activity, acting on the CH-OH group of donors,	K15717	K15717; prostamide/prostaglandin F2alpha synthase
INPP5K	451.113	0.361467	0.13119777	2.755132	0.0058668	0.036576	Not_Sig	no	gi|440912303|gb|ELR61887.1|	2279	0	Inositol polyphosphate 5-phosphatase K, partial [Bos mutus]	gi|742208374|ref|XM_010861346.1|	2859	0	PREDICTED: Bison bison bison inositol polyphosphate-5-phosphatase K (INPP5K), transcript variant X3, mRNA	Q9BT40	668	0	Inositol polyphosphate 5-phosphatase K	YIL002c_2	128	1.35e-30	COG5411	pfam03372	Exo_endo_phos	Endonuclease/Exonuclease/phosphatase family. This large family of proteins includes magnesium dependent endonucleases and a	GO:0030036|actin cytoskeleton organization;GO:0071320|cellular response to cAMP;GO:0071364|cellular response to epidermal growth factor stimulus;GO:0032870|cellular response to hormone stimulus;GO:0032869|cellular response to insulin stimulus;GO:0071356|cellular response to tumor necrosis factor;GO:0016311|dephosphorylation;GO:0007186|G-protein coupled receptor signaling pathway;GO:0042593|glucose homeostasis;GO:0001701|in utero embryonic development;GO:0046855|inositol phosphate	GO:0005737|cytoplasm;GO:0005829|cytosol;GO:0005783|endoplasmic reticulum;GO:0016020|membrane;GO:0043005|neuron	GO:0016312|inositol bisphosphate phosphatase activity;GO:0046030	K01106	E3.1.3.56; inositol-1,4,5-trisphosphate 5-phosphatase [EC:3.1.3.56]
FOXK2	1612.161	0.307162	0.111494923	2.75494	0.0058703	0.036578	Not_Sig	no	gi|803315419|ref|XP_012015994.1|	2792	0	PREDICTED: forkhead box protein K2 isoform X2 [Ovis aries musimon]	gi|742188118|ref|XM_010856390.1|	4518	0	PREDICTED: Bison bison bison forkhead box K2 (FOXK2), transcript variant X1, mRNA	Q01167	1026	0	Forkhead box protein K2	YNL068c	111	1.04e-23	COG5025	pfam00250	Fork_head	Fork head domain.	GO:0045944|positive regulation of transcription from RNA polymerase II promoter;GO:0006357|regulation of transcription from RNA polymerase II promoter;GO:0006355|regulation of transcription, DNA-templated;	GO:0043231|intracellular membrane-bounded organelle;GO:0005654|nucleoplasm;GO:0005634|nucleus;	GO:0000287|magnesium ion binding;GO:0000978|RNA polymerase II	K09404	FOXK; forkhead box protein K
NDUFC2	374.5425	-0.36481	0.132472854	-2.75382	0.0058905	0.036658	Not_Sig	no	gi|555992718|ref|XP_005908130.1|	633	3e-81	PREDICTED: NADH dehydrogenase [ubiquinone] 1 subunit C2 [Bos mutus]	gi|942092357|ref|XM_005908068.2|	635	0	PREDICTED: Bos mutus NADH dehydrogenase (ubiquinone) 1, subcomplex unknown, 2, 14.5kDa (NDUFC2), mRNA	Q02827	246	1e-81	NADH dehydrogenase [ubiquinone] 1 subunit C2	.	.	.	.	pfam06374	NDUF_C2	NADH-ubiquinone oxidoreductase subunit b14.5b (NDUFC2). This family consists of several NADH-ubiquinone oxidoreductase	GO:0006120|mitochondrial electron transport, NADH to ubiquinone;	GO:0016021|integral component of membrane;GO:0005747|mitochondrial respiratory chain complex I;	GO:0008137|NADH dehydrogenase (ubiquinone) activity;	K03968	NDUFC2; NADH dehydrogenase (ubiquinone) 1 subunit C2
PCMTD2	592.0283	-0.3355	0.121843897	-2.7535	0.0058961	0.036658	Not_Sig	no	gi|741947817|ref|XP_010809692.1|	1786	0	PREDICTED: protein-L-isoaspartate O-methyltransferase domain-containing protein 2 isoform X1 [Bos taurus]	gi|982942917|ref|XM_010811390.2|	3501	0	PREDICTED: Bos taurus protein-L-isoaspartate (D-aspartate) O-methyltransferase domain containing 2 (PCMTD2), transcript variant X1, mRNA	Q58CZ2	661	0	Protein-L-isoaspartate O-methyltransferase domain-containing protein 2	MTH827	88.6	3.19e-18	COG2518	pfam01135	PCMT	Protein-L-isoaspartate(D-aspartate) O-methyltransferase (PCMT).	.	GO:0005737|cytoplasm;	GO:0004719|protein-L-isoaspartate (D-aspartate) O-methyltransferase	.	.
LOC138992082	94.35433	0.711275	0.258336392	2.753288	0.0059	0.036658	UP	yes	gi|4239700|emb|CAA10770.1|	933	5e-111	reverse transcriptase-like [Bos taurus]	gi|315630352|gb|AC167875.4|	2448	0	Bos taurus Y Chr BAC CH240-202L22 (Children's Hospital Oakland Research Institute Bovine BAC Library (male)) complete sequence	.	.	.	.	.	.	.	.	pfam00078	RVT_1	Reverse transcriptase (RNA-dependent DNA polymerase). A reverse transcriptase gene is usually indicative of a mobile	.	.	.	.	.
TRAF3IP2	967.2514	0.35401	0.128590188	2.753006	0.0059051	0.036658	Not_Sig	no	gi|440910568|gb|ELR60355.1|	2367	0	Adapter protein CIKS [Bos mutus]	gi|942039680|ref|XM_005890215.2|	1847	0	PREDICTED: Bos mutus TRAF3 interacting protein 2 (TRAF3IP2), transcript variant X1, mRNA	O43734	701	0	Adapter protein CIKS	.	.	.	.	pfam08357	SEFIR	SEFIR domain. This family comprises IL17 receptors (IL17Rs) and SEF proteins. The latter are feedback inhibitors of FGF	GO:0001783|B cell apoptotic process;GO:0006959|humoral immune response;GO:0048305|immunoglobulin secretion;GO:0035556|intracellular signal transduction;GO:0002230|positive regulation of defense response to virus by host;GO:0043123|positive regulation of I-kappaB kinase/NF-kappaB signaling;	GO:0005622|intracellular;	.	.	.
TAOK1	906.5489	-0.33889	0.12310219	-2.75295	0.0059061	0.036658	Not_Sig	no	gi|507954339|ref|XP_004684892.1|	4918	0	PREDICTED: serine/threonine-protein kinase TAO1 [Condylura cristata]	gi|742208512|ref|XM_010861382.1|	11891	0	PREDICTED: Bison bison bison TAO kinase 1 (TAOK1), mRNA	Q5F2E8	1867	0	Serine/threonine-protein kinase TAO1	.	.	.	.	pfam00069	Pkinase	Protein kinase domain.	GO:0006974|cellular response to DNA damage stimulus;GO:0006281|DNA repair;GO:0097194|execution phase of apoptosis;GO:0031572|G2 DNA damage checkpoint;GO:0046330|positive regulation of JNK cascade;GO:0032874|positive regulation of stress-activated MAPK cascade;GO:0051493|regulation of cytoskeleton organization;	GO:0005737|cytoplasm;GO:0070062|extracellular exosome;	GO:0005524|ATP binding;GO:0004674|protein serine/threonine	K04429	TAO; thousand and one amino acid protein kinase [EC:2.7.11.1]
SNRNP27	299.4931	-0.45282	0.16448963	-2.75286	0.0059077	0.036658	Not_Sig	no	gi|795160303|ref|XP_011840432.1|	291	8e-29	PREDICTED: U4/U6.U5 small nuclear ribonucleoprotein 27 kDa protein [Mandrillus leucophaeus]	gi|742147439|ref|XM_010845300.1|	886	0	PREDICTED: Bison bison bison small nuclear ribonucleoprotein 27kDa (U4/U6.U5) (SNRNP27), mRNA	Q8K194	114	4e-29	U4/U6.U5 small nuclear ribonucleoprotein 27 kDa protein	.	.	.	.	pfam08648	DUF1777	Protein of unknown function (DUF1777). This is a family of eukaryotic proteins of unknown function. Some of the proteins in	GO:0006397|mRNA processing;GO:0008380|RNA splicing;	GO:0071011|precatalytic spliceosome;	.	K12846	SNRNP27; U4/U6.U5 tri-snRNP-associated protein 3
PHKB	414.9651	-0.34439	0.125105981	-2.75281	0.0059086	0.036658	Not_Sig	no	gi|742139835|ref|XP_010842203.1|	5623	0	PREDICTED: phosphorylase b kinase regulatory subunit beta isoform X1 [Bison bison bison]	gi|742139834|ref|XM_010843901.1|	4012	0	PREDICTED: Bison bison bison phosphorylase kinase, beta (PHKB), transcript variant X1, mRNA	Q7TSH2	2056	0	Phosphorylase b kinase regulatory subunit beta	.	.	.	.	pfam00723	Glyco_hydro_15	Glycosyl hydrolases family 15. In higher organisms this family is represented by phosphorylase kinase subunits.	GO:0005977|glycogen metabolic process;	GO:0005964|phosphorylase kinase complex;GO:0005886|plasma membrane;	GO:0004689|phosphorylase kinase activity;	K07190	PHKA_B; phosphorylase kinase alpha/beta subunit
CUL7	2558.297	0.291613	0.105933073	2.752806	0.0059087	0.036658	Not_Sig	no	gi|528924112|ref|XP_005193007.1|	8172	0	PREDICTED: cullin-7 [Bos taurus]	gi|983005172|ref|XM_005192950.3|	6093	0	PREDICTED: Bos taurus cullin 7 (CUL7), mRNA	Q14999	2741	0	Cullin-7	.	.	.	.	pfam00888	Cullin	Cullin family.	GO:0001837|epithelial to mesenchymal transition;GO:0007030|Golgi organization;GO:0036498|IRE1-mediated unfolded protein response;GO:0000226|microtubule cytoskeleton organization;GO:0000281|mitotic cytokinesis;GO:0001890|placenta development;GO:0050775|positive regulation of dendrite morphogenesis;GO:0016567|protein ubiquitination;GO:0006508|proteolysis;GO:0007088|regulation of mitotic nuclear division;GO:0006511|ubiquitin-dependent protein catabolic	GO:1990393|3M complex;GO:0005680|anaphase-promoting complex;GO:0005813|centrosome;GO:0031467|Cul7-RING ubiquitin ligase	.	K10613	CUL7; cullin 7
PDE4D	218.4985	-0.45776	0.166358768	-2.75165	0.0059295	0.036743	Not_Sig	no	gi|589937923|ref|XP_006981717.1|	3578	0	PREDICTED: cAMP-specific 3',5'-cyclic phosphodiesterase 4D-like isoform X2 [Peromyscus maniculatus bairdii]	gi|742177111|ref|XM_010853479.1|	7615	0	PREDICTED: Bison bison bison phosphodiesterase 4D, cAMP-specific (PDE4D), transcript variant X9, mRNA	Q08499	1299	0	cAMP-specific 3',5'-cyclic phosphodiesterase 4D	.	.	.	.	pfam00233	PDEase_I	3'5'-cyclic nucleotide phosphodiesterase.	GO:0071875|adrenergic receptor signaling pathway;GO:0086024|adrenergic receptor signaling pathway involved in positive regulation of heart rate;GO:0007568|aging;GO:0006198|cAMP catabolic process;GO:0019933|cAMP-mediated signaling;GO:0071320|cellular response to cAMP;GO:0071872|cellular response to epinephrine stimulus;GO:0071222|cellular response to lipopolysaccharide;GO:0061028|establishment of endothelial barrier;GO:0035264|multicellular organism growth;GO:0045822|negative regulation of heart contraction;GO:0033137|negative	GO:0016324|apical plasma membrane;GO:0034704|calcium channel complex;GO:0005813|centrosome;GO:0005829|cytosol;GO:0016020|membrane;GO:00	GO:0004115|3',5'-cyclic-AMP phosphodiesterase activity;GO:0004114	K13293	PDE4; cAMP-specific phosphodiesterase 4 [EC:3.1.4.53]
IL1R1	895.2763	0.296986	0.107930469	2.751644	0.0059297	0.036743	Not_Sig	no	gi|555960565|ref|XP_005892385.1|	3082	0	PREDICTED: interleukin-1 receptor type 1 isoform X1 [Bos mutus]	gi|742227385|ref|XM_010829661.1|	4074	0	PREDICTED: Bison bison bison interleukin 1 receptor, type I (IL1R1), transcript variant X4, mRNA	P14778	869	0	Interleukin-1 receptor type 1	.	.	.	.	pfam01582	TIR	TIR domain. The Toll/interleukin-1 receptor (TIR) homology domain is an intracellular signalling domain found in MyD88, interleukin 1	GO:0007166|cell surface receptor signaling pathway;GO:0006955|immune response;GO:0070498|interleukin-1-mediated signaling pathway;GO:2000661|positive regulation of interleukin-1-mediated signaling pathway;GO:2000391|positive regulation of neutrophil extravasation;GO:0050727|regulation of inflammatory response;GO:0070555|response to interleukin-1;	GO:0009986|cell surface;GO:0005576|extracellular region;GO:0005887|integral component of plasma	GO:0004908|interleukin-1 receptor activity;GO:0004909|interleukin-1, Type	K04386	IL1R1; interleukin 1 receptor type I
OAZ2	812.3452	-0.35415	0.12871239	-2.75145	0.0059332	0.036743	Not_Sig	no	gi|119598096|gb|EAW77690.1|	808	6e-101	ornithine decarboxylase antizyme 2, isoform CRA_a, partial [Homo sapiens]	gi|588480517|ref|NM_001290136.1|	1898	0	Bos mutus ornithine decarboxylase antizyme 2 (OAZ2), mRNA	Q5R680	276	5e-87	Ornithine decarboxylase antizyme 2	.	.	.	.	pfam02100	ODC_AZ	Ornithine decarboxylase antizyme. This family consists of ornithine decarboxylase antizyme proteins. The polyamine biosynthetic	GO:0006596|polyamine biosynthetic process;GO:0090316|positive regulation of intracellular protein transport;GO:0045732|positive regulation of protein catabolic process;	GO:0005829|cytosol;GO:0005634|nucleus;	GO:0008073|ornithine decarboxylase inhibitor activity;	K16613	OAZ2; ornithine decarboxylase antizyme 2
ZBTB33	389.2685	-0.4463	0.162211346	-2.75132	0.0059355	0.036743	Not_Sig	no	gi|147899495|ref|NP_001091626.1|	3376	0	transcriptional regulator Kaiso [Bos taurus]	gi|942042705|ref|XM_005891537.2|	5275	0	PREDICTED: Bos mutus zinc finger and BTB domain containing 33 (ZBTB33), mRNA	Q86T24	1208	0	Transcriptional regulator Kaiso	.	.	.	.	pfam00651	BTB	BTB/POZ domain. The BTB (for BR-C, ttk and bab) or POZ (for Pox virus and Zinc finger) domain is present near the N-terminus of a	GO:0035556|intracellular signal transduction;GO:0045892|negative regulation of transcription, DNA-templated;GO:0006351|transcription, DNA-templated;GO:0016055|Wnt signaling pathway;	GO:0005737|cytoplasm;GO:0005730|nucleolus;GO:0005654|nucleoplasm;GO:0005634|nucleus;GO:0005886|plasma membrane;	GO:0046872|metal ion binding;GO:0008327|methyl-CpG	K10507	ZBTB33, KAISO; zinc finger and BTB domain-containing protein 33
LOC102270894	821.0673	-0.31599	0.11486598	-2.75094	0.0059425	0.036743	Not_Sig	no	gi|594112638|ref|XP_006079103.1|	949	8e-121	PREDICTED: ras-related protein Rap-2c-like [Bubalus bubalis]	gi|594112637|ref|XM_006079041.1|	1255	0	PREDICTED: Bubalus bubalis ras-related protein Rap-2c-like (LOC102401189), mRNA	Q8BU31	174	7e-49	Ras-related protein Rap-2c	.	.	.	.	pfam00071	Ras	Ras family. Includes sub-families Ras, Rab, Rac, Ral, Ran, Rap Ypt1 and more. Shares P-loop motif with GTP_EFTU, arf and	GO:0090557|establishment of endothelial intestinal barrier;GO:0030336|negative regulation of cell migration;GO:0031954|positive regulation of protein autophosphorylation;GO:0032486|Rap protein signal transduction;GO:0061097|regulation of protein tyrosine kinase activity;	GO:0005923|bicellular tight junction;GO:0044291|cell-cell contact zone;GO:0005737|cytoplasm;GO:0005829|cytosol;GO:0070062|extracellular	GO:0019003|GDP binding;GO:0005525|GTP binding;GO:0003713	K07839	RAP2C; Ras-related protein Rap-2C
ELAC2	424.2885	0.338307	0.122983092	2.750838	0.0059443	0.036743	Not_Sig	no	gi|528995147|ref|XP_005220394.1|	4114	0	PREDICTED: zinc phosphodiesterase ELAC protein 2 isoform X1 [Bos taurus]	gi|982957156|ref|XM_005220337.3|	3138	0	PREDICTED: Bos taurus elaC ribonuclease Z 2 (ELAC2), transcript variant X1, mRNA	Q9GL73	1261	0	Zinc phosphodiesterase ELAC protein 2	SPAC1D4.10	286	7.15e-82	COG1234	pfam12706	Lactamase_B_2	Beta-lactamase superfamily domain. This family is part of the beta-lactamase superfamily and is related to pfam00753.	GO:0072684|mitochondrial tRNA 3'-trailer cleavage, endonucleolytic;	GO:0005739|mitochondrion;GO:0005634|nucleus;	GO:0004519|endonuclease activity;GO:0046872|metal ion binding;	.	.
ZNF341	140.5077	0.610666	0.221994454	2.750817	0.0059447	0.036743	UP	yes	gi|741948056|ref|XP_010809771.1|	4014	0	PREDICTED: zinc finger protein 341 isoform X1 [Bos taurus]	gi|982943078|ref|XM_010811469.2|	3271	0	PREDICTED: Bos taurus zinc finger protein 341 (ZNF341), transcript variant X1, mRNA	Q9BYN7	1449	0	Zinc finger protein 341	.	.	.	.	pfam13912	zf-C2H2_6	C2H2-type zinc finger.	GO:0006355|regulation of transcription, DNA-templated;GO:0006351|transcription, DNA-templated;	GO:0005634|nucleus;	GO:0003677|DNA binding;GO:0046872|metal ion binding;GO:0003700	.	.
FYCO1	1005.892	0.278815	0.101378261	2.750244	0.0059551	0.036786	Not_Sig	no	gi|942069917|ref|XP_014335321.1|	6522	0	PREDICTED: FYVE and coiled-coil domain-containing protein 1 [Bos mutus]	gi|742212696|ref|XM_010862402.1|	8161	0	PREDICTED: Bison bison bison FYVE and coiled-coil domain containing 1 (FYCO1), transcript variant X1, mRNA	Q9BQS8	1982	0	FYVE and coiled-coil domain-containing protein 1	.	.	.	.	pfam01363	FYVE	FYVE zinc finger. The FYVE zinc finger is named after four proteins that it has been found in: Fab1, YOTB/ZK632.12, Vac1, and EEA1.	GO:0072383|plus-end-directed vesicle transport along microtubule;GO:1901098|positive regulation of autophagosome maturation;	GO:0005776|autophagosome;GO:0031410|cytoplasmic vesicle;GO:0016021|integral component of membrane;GO:0043231|intracellular	GO:0046872|metal ion binding;	.	.
RHBDD3	405.71	0.421745	0.153365862	2.749931	0.0059608	0.036786	Not_Sig	no	gi|742220148|ref|XP_010827030.1|	932	1e-115	PREDICTED: rhomboid domain-containing protein 3 isoform X1 [Bison bison bison]	gi|742220174|ref|XM_010828731.1|	1859	0	PREDICTED: Bison bison bison rhomboid domain containing 3 (RHBDD3), transcript variant X4, mRNA	Q9Y3P4	247	2e-73	Rhomboid domain-containing protein 3	.	.	.	.	pfam04388	Hamartin	Hamartin protein. This family includes the hamartin protein which is thought to function as a tumor suppressor. The hamartin	GO:0001889|liver development;GO:0000165|MAPK cascade;GO:0032815|negative regulation of natural killer cell activation;GO:0045732|positive regulation of protein catabolic process;GO:0002673|regulation of acute inflammatory response;GO:0050708|regulation of protein secretion;GO:0009410|response to xenobiotic stimulus;	GO:0016021|integral component of membrane;GO:0005622|intracellular;	GO:0004252|serine-type endopeptidase activity;	.	.
LOC102287414	1129.055	-0.34675	0.126094289	-2.7499	0.0059613	0.036786	Not_Sig	no	gi|1181671|gb|AAC50363.1|	1807	0	GTP-binding protein alpha q subunit [Homo sapiens]	gi|942047113|ref|XM_005892987.2|	6196	0	PREDICTED: Bos mutus guanine nucleotide binding protein (G protein), q polypeptide (GNAQ), mRNA	Q2PKF4	700	0	Guanine nucleotide-binding protein G(q) subunit alpha	.	.	.	.	pfam00503	G-alpha	G-protein alpha subunit. G proteins couple receptors of extracellular signals to intracellular signaling pathways. The G protein	GO:0001508|action potential;GO:0007189|adenylate cyclase-activating G-protein coupled receptor signaling pathway;GO:0009649|entrainment of circadian clock;GO:0007213|G-protein coupled acetylcholine receptor signaling pathway;GO:0007215|glutamate receptor signaling pathway;GO:0060158|phospholipase C-activating dopamine receptor signaling pathway;GO:0007603|phototransduction, visible light;	GO:0016020|membrane;GO:0031965|nuclear membrane;GO:0001750|photoreceptor outer segment;	GO:0005525|GTP binding;GO:0005096|GTPase activator activity;GO:0003924	K04634	GNAQ; guanine nucleotide-binding protein G(q) subunit alpha
GLI3	576.4938	0.309033	0.112406163	2.749253	0.0059731	0.036839	Not_Sig	no	gi|926690388|ref|XP_013818855.1|	7419	0	PREDICTED: transcriptional activator GLI3 [Capra hircus]	gi|742225104|ref|XM_010829084.1|	7855	0	PREDICTED: Bison bison bison GLI family zinc finger 3 (GLI3), transcript variant X5, mRNA	Q5IS56	2450	0	Transcriptional activator GLI3	.	.	.	.	pfam13465	zf-H2C2_2	Zinc-finger double domain.	GO:0009952|anterior/posterior pattern specification;GO:0009953|dorsal/ventral pattern formation;GO:0048568|embryonic organ development;GO:0045893|positive regulation of transcription, DNA-templated;GO:0007224|smoothened signaling pathway;GO:0006351|transcription, DNA-templated;	GO:0005929|cilium;GO:0005737|cytoplasm;GO:0005634|nucleus;	GO:0003677|DNA binding;GO:0046872|metal ion binding;GO:0003700	K06230	GLI3; zinc finger protein GLI3
MGAT4B	1866.035	0.385271	0.140146561	2.749061	0.0059766	0.036841	Not_Sig	no	gi|803306447|ref|XP_012011647.1|	2805	0	PREDICTED: alpha-1,3-mannosyl-glycoprotein 4-beta-N-acetylglucosaminyltransferase B isoform X2 [Ovis aries musimon]	gi|965887474|ref|XM_012156257.2|	2017	0	PREDICTED: Ovis aries musimon mannosyl (alpha-1,3-)-glycoprotein beta-1,4-N-acetylglucosaminyltransferase, isozyme B (MGAT4B), transcript variant X2, mRNA	Q812F8	1059	0	Alpha-1,3-mannosyl-glycoprotein 4-beta-N-acetylglucosaminyltransferase B	.	.	.	.	pfam04666	Glyco_transf_54	N-Acetylglucosaminyltransferase-IV (GnT-IV) conserved region. The complex-type of oligosaccharides are synthesized through	GO:0006491|N-glycan processing;GO:0006487|protein N-linked glycosylation;	GO:0000139|Golgi membrane;GO:0016021|integral component of membrane;	GO:0008375|acetylglucosaminyltransferase activity;GO:0008454	K00738	MGAT4A_B; alpha-1,3-mannosylglycoprotein beta-1,4-N-
EVI2A	414.5835	-0.34541	0.125687671	-2.74818	0.0059927	0.036864	Not_Sig	no	gi|555969429|ref|XP_005896739.1|	1132	1e-148	PREDICTED: protein EVI2A [Bos mutus]	gi|942056948|ref|XM_005896677.2|	1557	0	PREDICTED: Bos mutus ecotropic viral integration site 2A (EVI2A), mRNA	P22794	333	3e-108	Protein EVI2A	.	.	.	.	pfam05399	EVI2A	Ectropic viral integration site 2A protein (EVI2A). This family contains several mammalian ectropic viral integration site 2A	.	GO:0016021|integral component of membrane;	GO:0004888|transmembrane signaling receptor activity;	.	.
PLK2	993.2274	-0.29127	0.105990963	-2.74807	0.0059948	0.036864	Not_Sig	no	gi|594082640|ref|XP_006064914.1|	3299	0	PREDICTED: LOW QUALITY PROTEIN: serine/threonine-protein kinase PLK2 [Bubalus bubalis]	gi|300795346|ref|NM_001192245.1|	2730	0	Bos taurus polo like kinase 2 (PLK2), mRNA	Q5R4L1	1257	0	Serine/threonine-protein kinase PLK2	.	.	.	.	pfam00069	Pkinase	Protein kinase domain.	GO:0000082|G1/S transition of mitotic cell cycle;GO:0060292|long term synaptic depression;GO:0060291|long-term synaptic potentiation;GO:0007613|memory;GO:0007093|mitotic cell cycle checkpoint;GO:0007052|mitotic spindle organization;GO:0043066|negative regulation of apoptotic process;GO:0018105|peptidyl-serine phosphorylation;GO:0010508|positive regulation of autophagy;GO:0043123|positive regulation of I-kappaB kinase/NF-kappaB signaling;GO:0045732|positive regulation of protein catabolic	GO:0005814|centriole;GO:0005813|centrosome;GO:0000785|chromatin;GO:0030425|dendrite;GO:0005622|intracellular;	GO:0005524|ATP binding;GO:0004674|protein serine/threonine	.	.
HACE1	376.9446	-0.35457	0.129028168	-2.74802	0.0059957	0.036864	Not_Sig	no	gi|742189209|ref|XP_010854984.1|	4920	0	PREDICTED: E3 ubiquitin-protein ligase HACE1 isoform X1 [Bison bison bison]	gi|742189208|ref|XM_010856682.1|	3667	0	PREDICTED: Bison bison bison HECT domain and ankyrin repeat containing E3 ubiquitin protein ligase 1 (HACE1), transcript variant X1, mRNA	F1N6G5	1898	0	E3 ubiquitin-protein ligase HACE1	SPAC19D5.04	328	1.97e-91	COG5021	pfam00632	HECT	HECT-domain (ubiquitin-transferase). The name HECT comes from Homologous to the E6-AP Carboxyl Terminus.	GO:0007049|cell cycle;GO:0007030|Golgi organization;GO:0061025|membrane fusion;GO:0070936|protein K48-linked ubiquitination;GO:0016567|protein ubiquitination;GO:0042787|protein ubiquitination involved in ubiquitin-dependent protein catabolic process;GO:0030334|regulation of cell migration;GO:0006355|regulation of transcription, DNA-templated;GO:0006351|transcription, DNA-templated;	GO:0005737|cytoplasm;GO:0005783|endoplasmic reticulum;GO:0032580|Golgi cisterna membrane;GO:0000139|Golgi membrane;GO:0005634|nucleus;	GO:0017137|Rab GTPase binding;GO:0004842|ubiquitin-protein	K12166	HACE1; E3 ubiquitin-protein ligase HACE1 [EC:2.3.2.26]
SRP19	520.6812	-0.40555	0.147587116	-2.7479	0.0059978	0.036864	Not_Sig	no	gi|77735907|ref|NP_001029650.1|	711	3e-91	signal recognition particle 19 kDa protein [Bos taurus]	gi|942075706|ref|XM_005902716.2|	916	0	PREDICTED: Bos mutus signal recognition particle 19kDa (SRP19), transcript variant X1, mRNA	Q3ZBG7	278	1e-92	Signal recognition particle 19 kDa protein	MK1551	55.8	2.29e-09	COG1400	pfam01922	SRP19	SRP19 protein. The signal recognition particle (SRP) binds to the signal peptide of proteins as they are being translated. The	GO:0006614|SRP-dependent cotranslational protein targeting to membrane;	GO:0005786|signal recognition particle, endoplasmic reticulum targeting;	GO:0008312|7S RNA binding;	K03105	SRP19; signal recognition particle subunit SRP19
EIF3D	2382.378	0.28503	0.103734413	2.747687	0.0060017	0.036864	Not_Sig	no	gi|926722265|ref|XP_005696886.2|	2926	0	PREDICTED: protein DEK isoform X1 [Capra hircus]	gi|942031829|ref|XM_005887463.2|	1919	0	PREDICTED: Bos mutus eukaryotic translation initiation factor 3, subunit D (EIF3D), mRNA	Q3T122	1115	0	Eukaryotic translation initiation factor 3 subunit D	.	.	.	.	pfam05091	eIF-3_zeta	Eukaryotic translation initiation factor 3 subunit 7 (eIF-3). This family is made up of eukaryotic translation initiation factor 3	GO:0001732|formation of cytoplasmic translation initiation complex;GO:0001731|formation of translation preinitiation complex;GO:0075522|IRES-dependent viral translational initiation;GO:0006446|regulation of translational initiation;GO:0006413|translational initiation;GO:0075525|viral translational termination-reinitiation;	GO:0016282|eukaryotic 43S preinitiation complex;GO:0033290|eukaryotic 48S preinitiation complex;GO:0005852|eukaryotic translation	GO:0044822|poly(A) RNA binding;GO:0003743|translation	K03251	EIF3D; translation initiation factor 3 subunit D
DENR	584.2981	-0.39015	0.141990963	-2.74769	0.0060018	0.036864	Not_Sig	no	gi|114051872|ref|NP_001039993.1|	830	5e-106	density-regulated protein [Bos taurus]	gi|742130368|ref|XM_010841311.1|	1348	0	PREDICTED: Bison bison bison density-regulated protein (DENR), mRNA	Q2HJ47	324	1e-107	Density-regulated protein	SPBC16C6.05	94.7	7.35e-22	COG0023	pfam01253	SUI1	Translation initiation factor SUI1.	.	.	GO:0003743|translation initiation factor activity;	.	.
CSNK1G3	441.2338	-0.37024	0.134753079	-2.74752	0.0060048	0.036864	Not_Sig	no	gi|548473329|ref|XP_005682756.1|	2334	0	PREDICTED: casein kinase I isoform gamma-3 isoform X1 [Capra hircus]	gi|742151684|ref|XM_010846300.1|	4368	0	PREDICTED: Bison bison bison casein kinase 1, gamma 3 (CSNK1G3), transcript variant X2, mRNA	Q5R4V3	889	0	Casein kinase I isoform gamma-3	.	.	.	.	pfam00069	Pkinase	Protein kinase domain.	GO:0016055|Wnt signaling pathway;	GO:0005737|cytoplasm;	GO:0005524|ATP binding;GO:0004674|protein serine/threonine	K08958	CSNK1G; casein kinase 1, gamma [EC:2.7.11.1]

TTYH2	550.2935	0.384104	0.13980359	2.747455	0.006006	0.036864	Not_Sig	no	gi|157785621|ref|NP_001099096.1|	1935	0	protein tweety homolog 2 [Bos taurus]	gi|742187159|ref|XM_010856098.1|	3268	0	PREDICTED: Bison bison bison tweety family member 2 (TTYH2), mRNA	Q3TH73	597	0	Protein tweety homolog 2	.	.	.	.	pfam04906	Tweety	Tweety. The tweety (tty) gene has not been characterized at the protein level. However, it is thought to form a membrane	.	GO:0034707|chloride channel complex;GO:0016021|integral component of membrane;GO:0005886|plasma membrane;	GO:0005254|chloride channel activity;	.	.
TMEM70	403.3484	-0.36543	0.13304319	-2.74673	0.0060193	0.036925	Not_Sig	no	gi|742154003|ref|XP_010845242.1|	1290	9e-175	PREDICTED: transmembrane protein 70, mitochondrial isoform X1 [Bison bison bison]	gi|742154006|ref|XM_010846941.1|	1167	0	PREDICTED: Bison bison bison transmembrane protein 70 (TMEM70), transcript variant X2, mRNA	A6H773	452	2e-157	Transmembrane protein 70, mitochondrial	.	.	.	.	pfam06979	DUF1301	Protein of unknown function (DUF1301). This family contains a number of eukaryotic proteins of unknown function that are	GO:0033615|mitochondrial proton-transporting ATP synthase complex assembly;	GO:0032592|integral component of mitochondrial membrane;GO:0005743|mitochondrial inner membrane;GO:0005654|nucleoplasm;	.	K17966	TMEM70; transmembrane protein 70, mitochondrial
LOC102283293	235.2943	-0.4395	0.160033101	-2.74633	0.0060267	0.036951	Not_Sig	no	gi|431896900|gb|ELK06164.1|	210	7e-25	Non-specific lipid-transfer protein [Pteropus alecto]	gi|850474981|gb|CP011886.1|	2507	0	Ovis canadensis canadensis isolate 43U chromosome 1 sequence	P07857	83.2	7e-27	Non-specific lipid-transfer protein	.	.	.	.	pfam02036	SCP2	SCP-2 sterol transfer family. This domain is involved in binding sterols. It is found in the SCP2 protein, as well as the C terminus	GO:1901373|lipid hydroperoxide transport;GO:0015914|phospholipid transport;GO:0032385|positive regulation of intracellular cholesterol transport;GO:0006694|steroid biosynthetic process;	GO:0005739|mitochondrion;GO:0005777|peroxisome;	GO:0033814|propanoyl-CoA C-acyltransferase activity;GO:0032934	K08764	SCP2, SCPX; sterol carrier protein 2 [EC:2.3.1.176]
BCAP31	2198.359	-0.31583	0.115066355	-2.7448	0.0060548	0.037104	Not_Sig	no	gi|440894044|gb|ELR46609.1|	1163	2e-156	B-cell receptor-associated protein 31, partial [Bos mutus]	gi|982973089|ref|XM_005227692.3|	1176	0	PREDICTED: Bos taurus B-cell receptor-associated protein 31 (BCAP31), transcript variant X1, mRNA	Q5R8H3	384	6e-131	B-cell receptor-associated protein 31	.	.	.	.	pfam05529	Bap31	B-cell receptor-associated protein 31-like. Bap31 is a polytopic integral protein of the endoplasmic reticulum membrane	GO:0006915|apoptotic process;GO:0006886|intracellular protein transport;GO:0016192|vesicle-mediated transport;	GO:0005789|endoplasmic reticulum membrane;GO:0033116|endoplasmic reticulum-Golgi intermediate compartment membrane;GO:0016021|integral	.	K14009	BCAP31, BAP31; B-cell receptor-associated protein 31
CPEB4	609.7737	-0.36222	0.131986849	-2.74436	0.0060629	0.03712	Not_Sig	no	gi|594062811|ref|XP_006055450.1|	3341	0	PREDICTED: cytoplasmic polyadenylation element-binding protein 4 isoform X1 [Bubalus bubalis]	gi|983002946|ref|XM_003583671.4|	7714	0	PREDICTED: Bos taurus cytoplasmic polyadenylation element binding protein 4 (CPEB4), transcript variant X1, mRNA	Q17RY0	1285	0	Cytoplasmic polyadenylation element-binding protein 4	.	.	.	.	pfam00076	RRM_1	RNA recognition motif. (a.k.a. RRM, RBD, or RNP domain). The RRM motif is probably diagnostic of an RNA binding protein. RRMs	GO:0071230|cellular response to amino acid stimulus;GO:0036294|cellular response to decreased oxygen levels;GO:0042149|cellular response to glucose starvation;GO:0035235|ionotropic glutamate receptor signaling pathway;GO:2000766|negative regulation of cytoplasmic translation;GO:0043524|negative regulation of neuron apoptotic process;GO:0002931|response to ischemia;	GO:0005737|cytoplasm;GO:0030425|dendrite;GO:0005783|endoplasmic reticulum;GO:1990124|messenger ribonucleoprotein	GO:0003730|mRNA 3'-UTR binding;GO:0000166|nucleotide	K02602	CPEB, ORB; cytoplasmic polyadenylation element-binding
VHL	163.0157	-0.47092	0.171597511	-2.74431	0.0060638	0.03712	Not_Sig	no	gi|158517959|ref|NP_001103489.1|	886	7e-110	von Hippel-Lindau disease tumor suppressor [Bos taurus]	gi|742133712|ref|XM_010842116.1|	2536	0	PREDICTED: Bison bison bison von Hippel-Lindau tumor suppressor, E3 ubiquitin protein ligase (VHL), mRNA	Q5Q9Z2	315	2e-99	Von Hippel-Lindau disease tumor suppressor	.	.	.	.	pfam01847	VHL	von Hippel-Lindau disease tumor suppressor protein. VHL forms a ternary complex with the elonginB and elonginC proteins. This	GO:0016567|protein ubiquitination;	GO:0016020|membrane;GO:0005634|nucleus;GO:0030891|VCB complex;	GO:0061630|ubiquitin protein ligase activity;	K03871	VHL; von Hippel-Lindau disease tumor supressor
LOC102282467	182.7163	-0.49623	0.180894736	-2.74322	0.0060839	0.037223	Not_Sig	no	gi|157279963|ref|NP_001098497.1|	441	1e-61	uncharacterized protein LOC790886 precursor [Bos taurus]	gi|157279962|ref|NM_001105027.1|	418	0	Bos taurus uncharacterized LOC790886 (LOC790886), mRNA	Q28085	173	7e-63	Complement factor H	.	.	.	.	pfam00084	Sushi	Sushi domain (SCR repeat).	GO:0006957|complement activation, alternative pathway;	GO:0005576|extracellular region;	.	K04004	HF1; complement factor H
FAM171B	249.9708	-0.43792	0.159661977	-2.74279	0.0060919	0.037239	Not_Sig	no	gi|742201684|ref|XP_010858033.1|	3806	0	PREDICTED: protein FAM171B [Bison bison bison]	gi|742201683|ref|XM_010859731.1|	5695	0	PREDICTED: Bison bison bison family with sequence similarity 171, member B (FAM171B), mRNA	Q6P995	1337	0	Protein FAM171B	.	.	.	.	pfam10577	UPF0560	Uncharacterized protein family UPF0560. This family of proteins has no known function.	.	GO:0016021|integral component of membrane;	.	.	.
SLMAP	3239.88	-0.32993	0.120293373	-2.74273	0.0060931	0.037239	Not_Sig	no	gi|594044449|ref|XP_006046720.1|	4139	0	PREDICTED: sarcolemmal membrane-associated protein isoform X3 [Bubalus bubalis]	gi|742170673|ref|XM_010851615.1|	4423	0	PREDICTED: Bison bison bison sarcolemma associated protein (SLMAP), transcript variant X3, mRNA	Q14BN4	1469	0	Sarcolemmal membrane-associated protein	SPBC3H7.13	92	3.58e-19	COG1716	pfam00498	FHA	FHA domain. The FHA (Forkhead-associated) domain is a phosphopeptide binding motif.	GO:0006936|muscle contraction;	GO:0005887|integral component of plasma membrane;GO:0005815|microtubule organizing center;GO:0042383|sarcolemma;GO:00057	.	.	.
RAB11B	568.4748	0.42982	0.156828184	2.740707	0.0061307	0.037449	Not_Sig	no	gi|395841772|ref|XP_003793707.1|	1299	1e-178	PREDICTED: ras-related protein Rab-11B [Otolemur garnettii]	gi|742218278|ref|XM_010828266.1|	916	0	PREDICTED: Bison bison bison RAB11B, member RAS oncogene family (RAB11B), mRNA	O35509	451	1e-159	Ras-related protein Rab-11B	SPAC18G6.03	304	7.15e-104	COG1100	pfam00071	Ras	Ras family. Includes sub-families Ras, Rab, Rac, Ral, Ran, Rap Ypt1 and more. Shares P-loop motif with GTP_EFTU, arf and	GO:0071468|cellular response to acidic pH;GO:0045054|constitutive secretory pathway;GO:0090150|establishment of protein localization to membrane;GO:0035773|insulin secretion involved in cellular response to glucose stimulus;GO:0032402|melanosome transport;GO:0001881|receptor recycling;GO:0045055|regulated secretory pathway;GO:0044070|regulation of anion transport;GO:2001135|regulation of endocytic recycling;GO:2000008|regulation of protein localization to cell surface;GO:1990126|retrograde	GO:0016235|aggresome;GO:0030054|cell junction;GO:0016023|cytoplasmic membrane-bounded vesicle;GO:0070062|extracellular	GO:0019003|GDP binding;GO:0005525|GTP binding;GO:0003924	K07905	RAB11B; Ras-related protein Rab-11B
VPS52	442.6083	0.390767	0.142606322	2.740179	0.0061406	0.037475	Not_Sig	no	gi|586544662|ref|XP_006907447.1|	3409	0	PREDICTED: vacuolar protein sorting-associated protein 52 homolog isoform X1 [Pteropus alecto]	gi|942042378|ref|XM_005891391.2|	2929	0	PREDICTED: Bos mutus vacuolar protein sorting 52 homolog (S. cerevisiae) (VPS52), transcript variant X1, mRNA	O55166	1161	0	Vacuolar protein sorting-associated protein 52 homolog	.	.	.	.	pfam04129	Vps52	Vps52 / Sac2 family. Vps52 complexes with Vps53 and Vps54 to form a multi- subunit complex involved in regulating membrane	GO:0010668|ectodermal cell differentiation;GO:0048611|embryonic ectodermal digestive tract development;GO:0032456|endocytic recycling;GO:0007041|lysosomal transport;GO:0015031|protein transport;	GO:1990745|EARP complex;GO:0010008|endosome membrane;GO:0000938|GARP complex;GO:0048471|perinuclear region of	.	.	.
COX14	517.0868	-0.3913	0.142804537	-2.74013	0.0061415	0.037475	Not_Sig	no	gi|953856580|ref|XP_014596382.1|	338	7e-41	PREDICTED: uncharacterized protein LOC106783320 isoform X2 [Equus caballus]	gi|982925083|ref|XM_010804994.2|	2211	0	PREDICTED: Bos taurus COX14 cytochrome c oxidase assembly homolog (S. cerevisiae) (COX14), transcript variant X5, mRNA	Q1RMH3	119	2e-30	Cytochrome c oxidase assembly protein COX14	.	.	.	.	pfam14880	COX14	Cytochrome oxidase c assembly. COX14 plays an essential role in cytochrome oxidase assembly. The COX14 product is a low-molecular	GO:0033617|mitochondrial respiratory chain complex IV assembly;	GO:0016021|integral component of membrane;GO:0031966|mitochondrial membrane;GO:0005739|mitochondrion;	.	K18181	COX14; cytochrome c oxidase assembly factor 14
MFAP3	601.7525	-0.34533	0.126062347	-2.73938	0.0061556	0.037541	Not_Sig	no	gi|262073102|ref|NP_001159991.1|	1803	0	microfibril-associated glycoprotein 3 precursor [Bos taurus]	gi|594099784|ref|XM_006072949.1|	4353	0	PREDICTED: Bubalus bubalis microfibrillar-associated protein 3 (MFAP3), transcript variant X1, mRNA	Q5R9E4	604	0	Microfibril-associated glycoprotein 3	.	.	.	.	pfam13927	Ig_3	Immunoglobulin domain. This family contains immunoglobulin-like domains.	.	GO:0016021|integral component of membrane;GO:0005886|plasma membrane;	.	.	.
ADAM33	244.8734	0.471502	0.172209573	2.737957	0.0061822	0.037672	Not_Sig	no	gi|942049825|ref|XP_014333121.1|	3739	0	PREDICTED: disintegrin and metalloproteinase domain-containing protein 33 [Bos mutus]	gi|942049824|ref|XM_014477635.1|	3738	0	PREDICTED: Bos mutus ADAM metallopeptidase domain 33 (ADAM33), mRNA	Q9BZ11	961	0	Disintegrin and metalloproteinase domain-containing protein 33	.	.	.	.	pfam01421	Reprolysin	Reprolysin (M12B) family zinc metalloprotease. The members of this family are enzymes that cleave peptides. These proteases require	GO:0006508|proteolysis;	GO:0016021|integral component of membrane;	GO:0004222|metalloendopeptidase activity;GO:0008270|zinc ion binding;	K08616	ADAM33; disintegrin and metalloproteinase domain-containing
TTLL4	196.0561	0.461927	0.16871679	2.737884	0.0061836	0.037672	Not_Sig	no	gi|555955131|ref|XP_005889725.1|	5456	0	PREDICTED: tubulin polyglutamylase TTLL4 [Bos mutus]	gi|742207828|ref|XM_010861208.1|	4188	0	PREDICTED: Bison bison bison tubulin tyrosine ligase-like family, member 4 (TTLL4), mRNA	Q14679	1671	0	Tubulin polyglutamylase TTLL4	.	.	.	.	pfam03133	TTL	Tubulin-tyrosine ligase family. Tubulins and microtubules are subjected to several post-translational modifications of	GO:0018200|peptidyl-glutamic acid modification;GO:0018095|protein polyglutamylation;	GO:0005929|cilium;GO:0005737|cytoplasm;GO:0005874|microtubule;	GO:0005524|ATP binding;GO:0070739|protein-glutamic acid ligase	K16601	TTLL4; tubulin polyglutamylase TTLL4 [EC:6.-.-.-]
FLOT2	1143.115	0.35828	0.130966977	2.735649	0.0062257	0.037851	Not_Sig	no	gi|594035093|ref|XP_006042231.1|	2102	0	PREDICTED: flotillin-2 isoform X2 [Bubalus bubalis]	gi|151553622|gb|BC148058.1|	2618	0	Bos taurus flotillin 2, mRNA (cDNA clone MGC:142810 IMAGE:8279239), complete cds	A6QLR4	794	0	Flotillin-2	BS_yuaG	244	7.39e-71	COG2268	pfam01145	Band_7	SPFH domain / Band 7 family. This family has been called SPFH, Band 7 or PHB domain. Recent phylogenetic analysis has shown	GO:0007155|cell adhesion;	GO:0005901|caveola;GO:0030139|endocytic vesicle;GO:0005768|endosome;	.	K07192	FLOT; flotillin
MRPL47	272.2864	-0.46573	0.170261258	-2.73541	0.0062303	0.037851	Not_Sig	no	gi|555960933|ref|XP_005892566.1|	1325	0	PREDICTED: 39S ribosomal protein L47, mitochondrial isoform X1 [Bos mutus]	gi|942045597|ref|XM_005892504.2|	1002	0	PREDICTED: Bos mutus mitochondrial ribosomal protein L47 (MRPL47), transcript variant X1, mRNA	Q08DT6	513	0	39S ribosomal protein L47, mitochondrial	.	.	.	.	pfam06984	MRP-L47	Mitochondrial 39-S ribosomal protein L47 (MRP-L47). This family represents the N-terminal region (approximately 8 residues) of the	GO:0032543|mitochondrial translation;GO:0070125|mitochondrial translational elongation;GO:0070124|mitochondrial translational initiation;	GO:0005743|mitochondrial inner membrane;GO:0005762|mitochondrial large ribosomal subunit;	GO:0003735|structural constituent of ribosome;	K17428	MRPL47, NCM1; large subunit ribosomal protein L47
LOC106701310	83.63009	-0.60652	0.221733475	-2.73533	0.0062317	0.037851	DOWN	yes	gi|281348834|gb|EFB24418.1|	805	4e-106	hypothetical protein PANDA_001311, partial [Ailuropoda melanoleuca]	gi|942083170|ref|XM_014481293.1|	762	0	PREDICTED: Bos mutus coatomer subunit gamma-2-like (LOC106701310), mRNA	A2VE21	327	6e-104	Coatomer subunit gamma-2	YNL287w	57.8	9.90e-09	COG5240	pfam08752	COP-gamma_platf	Coatomer gamma subunit appendage platform subdomain. COPI-coated vesicles function in retrograde transport from the	GO:0006891|intra-Golgi vesicle-mediated transport;GO:0006886|intracellular protein transport;	GO:0030126|COPI vesicle coat;GO:0005829|cytosol;GO:0000139|Golgi membrane;	GO:0005198|structural molecule activity;	K17267	COPG; coatomer protein complex, subunit gamma
SMPD4	953.4472	0.349144	0.127650799	2.735152	0.0062352	0.037851	Not_Sig	no	gi|329663533|ref|NP_001192531.1|	3807	0	sphingomyelin phosphodiesterase 4 [Bos taurus]	gi|329663532|ref|NM_001205602.1|	3260	0	Bos taurus sphingomyelin phosphodiesterase 4, neutral membrane (neutral sphingomyelinase-3) (SMPD4), mRNA	Q9NXE4	1273	0	Sphingomyelin phosphodiesterase 4	.	.	.	.	pfam14724	mit_SMPDase	Mitochondrial-associated sphingomyelin phosphodiesterase. The GO annotation for this family indicates that it is a single-pass	GO:0071356|cellular response to tumor necrosis factor;GO:0046513|ceramide biosynthetic process;GO:0046475|glycerophospholipid catabolic process;GO:0006687|glycosphingolipid metabolic process;GO:0006685|sphingomyelin catabolic process;	GO:0005783|endoplasmic reticulum;GO:0005789|endoplasmic reticulum membrane;GO:0005794|Golgi apparatus;GO:0000139|Golgi	GO:0046872|metal ion binding;GO:0004767|sphingomyelin	K12353	SMPD4; sphingomyelin phosphodiesterase 4 [EC:3.1.4.12]
ZNF629	231.9224	0.41877	0.153107475	2.73514	0.0062354	0.037851	Not_Sig	no	gi|926724347|ref|XP_005697772.2|	4401	0	PREDICTED: zinc finger protein 629 [Capra hircus]	gi|742107412|ref|XM_010837340.1|	7198	0	PREDICTED: Bison bison bison zinc finger protein 629 (ZNF629), mRNA	Q9UEG4	1533	0	Zinc finger protein 629	.	.	.	.	pfam13465	zf-H2C2_2	Zinc-finger double domain.	GO:0006355|regulation of transcription, DNA-templated;GO:0006351|transcription, DNA-templated;	GO:0005634|nucleus;	GO:0003677|DNA binding;GO:0046872|metal ion binding;GO:0003700	.	.
S100A2	717.7557	-0.37138	0.13578139	-2.73513	0.0062356	0.037851	Not_Sig	no	gi|594032707|ref|XP_006041113.1|	572	4e-72	PREDICTED: protein S100-A2 isoform X2 [Bubalus bubalis]	gi|742111989|ref|XM_010838424.1|	468	0	PREDICTED: Bison bison bison S100 calcium binding protein A2 (S100A2), transcript variant X6, mRNA	P10462	198	1e-63	Protein S100-A2	.	.	.	.	pfam01023	S_100	S-100/ICaBP type calcium binding domain. The S-100 domain is a subfamily of the EF-hand calcium binding proteins.	GO:0043542|endothelial cell migration;	.	GO:0005509|calcium ion binding;	.	.
LOC138989864	106.7136	-0.7796	0.285034424	-2.73511	0.006236	0.037851	DOWN	yes	gi|620981096|ref|XP_007671083.1|	1556	0	PREDICTED: E3 ubiquitin-protein ligase KCMF1 isoform X1 [Ornithorhynchus anatinus]	gi|115495508|ref|NM_001076275.1|	1587	0	Bos taurus potassium channel modulatory factor 1 (KCMF1), mRNA	Q9P0J7	601	0	E3 ubiquitin-protein ligase KCMF1	.	.	.	.	pfam05605	zf-Di19	Drought induced 19 protein (Di19), zinc-binding. This family consists of several drought induced 19 (Di19) like proteins. Di19 has been found	.	.	GO:0016874|ligase activity;GO:0008270|zinc ion binding;	.	.
LGALS1	7572.085	-0.29756	0.108845574	-2.73378	0.0062612	0.03798	Not_Sig	no	gi|28461189|ref|NP_786976.1|	713	3e-93	galectin-1 [Bos taurus]	gi|742144406|ref|XM_010845078.1|	555	0	PREDICTED: Bison bison bison lectin, galactoside-binding, soluble, 1 (LGALS1), mRNA	P11116	279	1e-94	Galectin-1	.	.	.	.	pfam00337	Gal-bind_lectin	Galactoside-binding lectin. This family contains galactoside binding lectins. The family also includes enzymes such as human eosinophil	GO:0006915|apoptotic process;GO:0045445|myoblast differentiation;GO:0002317|plasma cell differentiation;GO:0043123|positive regulation of I-kappaB kinase/NF-kappaB signaling;GO:0046598|positive regulation of viral entry into host cell;GO:0031295|T cell costimulation;	GO:0070062|extracellular exosome;GO:0005615|extracellular space;GO:0005622|intracellular;GO:0005578|proteinaceous extracellular matrix;	GO:0030246|carbohydrate binding;GO:0044822|poly(A) RNA	K06830	LGALS1; galectin-1
RDX	1123.487	-0.36267	0.132667999	-2.73364	0.0062639	0.03798	Not_Sig	no	gi|655601716|ref|XP_002708517.2|	2471	0	PREDICTED: LOW QUALITY PROTEIN: radixin [Oryctolagus cuniculus]	gi|115496124|ref|NM_001075749.1|	4219	0	Bos taurus radixin (RDX), mRNA	Q32LP2	954	0	Radixin	.	.	.	.	pfam00769	ERM	Ezrin/radixin/moesin family. This family of proteins contain a band 4.1 domain (pfam00373), at their amino terminus. This family	GO:0051693|actin filament capping;	GO:0032154|cleavage furrow;GO:0005737|cytoplasm;GO:0005856|cytoskeleton;GO:0019898|extrinsic component of	.	K05762	RDX; radixin
SIPA1L1	498.3977	0.385168	0.140935254	2.732942	0.0062771	0.03804	Not_Sig	no	gi|742152909|ref|XP_010844954.1|	8384	0	PREDICTED: signal-induced proliferation-associated 1-like protein 1 isoform X1 [Bison bison bison]	gi|742152962|ref|XM_010846667.1|	7666	0	PREDICTED: Bison bison bison signal-induced proliferation-associated 1 like 1 (SIPA1L1), transcript variant X15, mRNA	O43166	3161	0	Signal-induced proliferation-associated 1-like protein 1	.	.	.	.	pfam11881	DUF3401	Domain of unknown function (DUF3401). This domain is functionally uncharacterized. This domain is found in eukaryotes.	GO:0031532|actin cytoskeleton reorganization;GO:0090630|activation of GTPase activity;GO:0048013|ephrin receptor signaling pathway;GO:0050770|regulation of axonogenesis;GO:0061001|regulation of dendritic spine morphogenesis;GO:0043087|regulation of GTPase activity;GO:0051056|regulation of small GTPase mediated signal transduction;GO:0048167|regulation of synaptic plasticity;	GO:0030054|cell junction;GO:0005737|cytoplasm;GO:0005856|cytoskeleton;GO:0043197|dendritic spine;GO:0014069|postsynaptic	GO:0005096|GTPase activator activity;	K17701	SIPA1L1, E6TP1; signal-induced proliferation-associated 1 like
RELT	72.05397	0.680273	0.248971288	2.732336	0.0062887	0.038089	UP	yes	gi|296479820|tpg|DAA21935.1|	1593	0	TPA: RELT tumor necrosis factor receptor [Bos taurus]	gi|982995530|ref|XM_005194658.2|	2575	0	PREDICTED: Bos taurus RELT tumor necrosis factor receptor (RELT), transcript variant X2, mRNA	Q9N092	512	8e-172	Tumor necrosis factor receptor superfamily member 19L	.	.	.	.	pfam12606	RELT	tumor necrosis factor receptor superfamily member 19. This family of proteins is found in bacteria and eukaryotes. Proteins	.	GO:0005737|cytoplasm;GO:0016021|integral component of membrane;GO:0005886|plasma membrane;	.	K05156	TNFRSF19L, RELT; tumor necrosis factor receptor superfamily member
SGCB	1015.643	-0.35259	0.129052497	-2.73218	0.0062917	0.038089	Not_Sig	no	gi|742201622|ref|XP_010858016.1|	1576	0	PREDICTED: beta-sarcoglycan [Bison bison bison]	gi|942080262|ref|XM_005904286.2|	4282	0	PREDICTED: Bos mutus sarcoglycan, beta (43kDa dystrophin-associated glycoprotein) (SGCB), mRNA	A6QP70	604	0	Beta-sarcoglycan	.	.	.	.	pfam04790	Sarcoglycan_1	Sarcoglycan complex subunit protein. The dystrophin glycoprotein complex (DGC) is a membrane-spanning complex that	GO:0055013|cardiac muscle cell development;GO:0061024|membrane organization;GO:0048747|muscle fiber development;GO:0007517|muscle organ development;GO:0097084|vascular smooth muscle cell development;	GO:0005737|cytoplasm;GO:0005856|cytoskeleton;GO:0016021|integral component of membrane;GO:0016012|sarcoglycan complex;GO:0042383|sarcolemma;	.	K12566	SGCB; beta-sarcoglycan
LOC102270678	67.30274	-3.07975	1.127430501	-2.73165	0.0063017	0.038108	DOWN	yes	gi|281350307|gb|EFB25891.1|	780	3e-103	hypothetical protein PANDA_004337, partial [Ailuropoda melanoleuca]	gi|742216631|ref|XM_010863373.1|	504	0	PREDICTED: Bison bison bison ribosomal protein S23 (RPS23), mRNA	P62268	285	9e-98	40S ribosomal protein S23	YPR132w	227	9.20e-77	COG0048	pfam00164	Ribosom_S12_S23	Ribosomal protein S12/S23. This protein is known as S12 in bacteria and archaea and S23 in eukaryotes.	GO:0006412|translation;	GO:0022627|cytosolic small ribosomal subunit;GO:0005840|ribosome;GO:0015935|small ribosomal subunit;	GO:0003735|structural constituent of ribosome;GO:0045182|translation	K02973	RP-S23e, RPS23; small subunit ribosomal protein S23e
NCOA5	466.8141	0.322429	0.118039186	2.731543	0.0063039	0.038108	Not_Sig	no	gi|594054339|ref|XP_006051436.1|	2277	0	PREDICTED: nuclear receptor coactivator 5 isoform X1 [Bubalus bubalis]	gi|594054340|ref|XM_006051375.1|	2138	0	PREDICTED: Bubalus bubalis nuclear receptor coactivator 5 (NCOA5), transcript variant X2, mRNA	Q9HCD5	822	0	Nuclear receptor coactivator 5	.	.	.	.	pfam14431	YwqJ-deaminase	YwqJ-like deaminase. A member of the nucleic acid/nucleotide deaminase superfamily prototyped by Bacillus YwqJ. Members of this	GO:0042593|glucose homeostasis;GO:0046627|negative regulation of insulin receptor signaling pathway;GO:0006355|regulation of transcription, DNA-templated;GO:0007165|signal transduction;GO:0006351|transcription, DNA-templated;	GO:0015629|actin cytoskeleton;GO:0005615|extracellular space;GO:0005634|nucleus;	GO:0003682|chromatin binding;GO:0044822|poly(A) RNA	.	.
GPR180	361.1742	-0.41784	0.152970638	-2.73149	0.006305	0.038108	Not_Sig	no	gi|555995103|ref|XP_005909298.1|	2282	0	PREDICTED: integral membrane protein GPR180 [Bos mutus]	gi|594115382|ref|XM_006080332.1|	3381	0	PREDICTED: Bubalus bubalis G protein-coupled receptor 180 (GPR180), mRNA	Q86V85	764	0	Integral membrane protein GPR180	.	.	.	.	pfam10192	GpcrRhopsn4	Rhodopsin-like GPCR transmembrane domain. This region of 270 amino acids is the seven transmembrane alpha-	GO:0007186|G-protein coupled receptor signaling pathway;GO:0019236|response to pheromone;	GO:0070062|extracellular exosome;GO:0016021|integral component of membrane;	.	.	.
YBX3	6456.657	0.281434	0.103042856	2.731237	0.0063097	0.038117	Not_Sig	no	gi|148235020|ref|NP_001091543.1|	842	1e-102	Y-box-binding protein 3 [Bos taurus]	gi|594107152|ref|XM_006076424.1|	1947	0	PREDICTED: Bubalus bubalis Y box binding protein 3 (YBX3), transcript variant X1, mRNA	Q62764	307	4e-96	Y-box-binding protein 3	BU489	67.8	1.78e-13	COG1278	pfam00313	CSD	'Cold-shock' DNA-binding domain.	GO:0031100|organ regeneration;GO:0000122|negative regulation of transcription from RNA polymerase II promoter;GO:0007283|spermatogenesis;GO:0006351|transcription, DNA-templated;	GO:0005737|cytoplasm;GO:0005921|gap junction;GO:0005634|nucleus;	GO:0003697|single-stranded DNA binding;	K06099	CSDA, ZONAB; cold shock domain protein A
APH1B	248.6158	-0.43902	0.1608555	-2.72929	0.0063472	0.038312	Not_Sig	no	gi|528966036|ref|XP_005211751.1|	1144	4e-142	PREDICTED: gamma-secretase subunit APH-1B isoform X1 [Bos taurus]	gi|982936931|ref|XM_005211694.3|	4004	0	PREDICTED: Bos taurus aph-1 homolog B, gamma secretase subunit (APH1B), transcript variant X1, mRNA	Q5RDM3	378	6e-119	Gamma-secretase subunit APH-1B	.	.	.	.	pfam06105	Aph-1	Aph-1 protein. This family consists of several eukaryotic Aph-1 proteins.Gamma-secretase catalyses the intramembrane	GO:0007219|Notch signaling pathway;GO:0043085|positive regulation of catalytic activity;GO:0016485|protein processing;	GO:0016021|integral component of membrane;GO:0030133|transport vesicle;	GO:0008233|peptidase activity;	K06172	APH1; anterior pharynx defective 1
PGRMC1	1902.585	-0.25719	0.094242249	-2.729	0.0063527	0.038312	Not_Sig	no	gi|555950129|ref|XP_005887269.1|	798	2e-99	PREDICTED: membrane-associated progesterone receptor component 1 isoform X1 [Bos mutus]	gi|942030727|ref|XM_005887207.2|	1863	0	PREDICTED: Bos mutus progesterone receptor membrane component 1 (PGRMC1), transcript variant X1, mRNA	Q17QC0	310	3e-100	Membrane-associated progesterone receptor component 1	.	.	.	.	pfam00173	Cyt-b5	Cytochrome b5-like Heme/Steroid binding domain. This family includes heme binding domains from a diverse range of proteins.	GO:1903537|meiotic cell cycle process involved in oocyte maturation;	GO:0000775|chromosome, centromeric region;GO:0012505|endomembrane system;GO:0005783|endoplasmic reticulum;GO:0005789|endoplasmic	GO:0020037|heme binding;GO:0005496|steroid binding;	K17278	PGRMC1_2; membrane-associated progesterone
CYLD	712.81	-0.35747	0.130996559	-2.72883	0.006356	0.038312	Not_Sig	no	gi|742081230|ref|XP_010853467.1|	5094	0	PREDICTED: ubiquitin carboxyl-terminal hydrolase CYLD isoform X1 [Bison bison bison]	gi|741958393|ref|XM_005218684.2|	7922	0	PREDICTED: Bos taurus cylindromatosis (turban tumor syndrome) (CYLD), transcript variant X1, mRNA	Q1RMU2	1965	0	Ubiquitin carboxyl-terminal hydrolase CYLD	.	.	.	.	pfam01302	CAP_GLY	CAP-Gly domain. Cytoskeleton-associated proteins (CAPs) are involved in the organisation of microtubules and transportation of	GO:0070266|necroptotic process;GO:0090090|negative regulation of canonical Wnt signaling pathway;GO:0042347|negative regulation of NF-kappaB import into nucleus;GO:0032088|negative regulation of NF-kappaB transcription factor activity;GO:2001238|positive regulation of extrinsic apoptotic signaling pathway;GO:0070536|protein K63-linked deubiquitination;GO:1902017|regulation of cilium assembly;GO:2001242|regulation of intrinsic apoptotic signaling pathway;GO:0070507|regulation of microtubule cytoskeleton	GO:0005813|centrosome;GO:0036064|ciliary basal body;GO:0097542|ciliary tip;GO:0005881|cytoplasmic microtubule;GO:0005829|cytosol;GO:00312	GO:0061578|Lys63-specific deubiquitinase activity;GO:0004843	K08601	CYLD, USLP2; ubiquitin thioesterase CYLD [EC:3.4.19.12]
AK6	107.6726	-0.69014	0.252908935	-2.7288	0.0063564	0.038312	DOWN	yes	gi|555954496|ref|XP_005889415.1|	911	4e-122	PREDICTED: adenylate kinase isoenzyme 6 [Bos mutus]	gi|942037060|ref|XM_005889353.2|	722	0	PREDICTED: Bos mutus adenylate kinase 6 (AK6), mRNA	A5PJA1	355	2e-123	Adenylate kinase isoenzyme 6	SPCC830.11c	156	1.85e-47	COG1936	pfam13238	AAA_18	AAA domain.	.	GO:0015030|Cajal body;GO:0005654|nucleoplasm;	GO:0004017|adenylate kinase activity;GO:0005524|ATP	K18532	AK6, FAP7; adenylate kinase [EC:2.7.4.3]
SUCLA2	811.0753	-0.38575	0.14136874	-2.72869	0.0063586	0.038312	Not_Sig	no	gi|440895016|gb|ELR47317.1|	2395	0	Succinyl-CoA ligase [ADP-forming] subunit beta, mitochondrial, partial [Bos mutus]	gi|942092662|ref|XM_005908144.2|	2297	0	PREDICTED: Bos mutus succinate-CoA ligase, ADP-forming, beta subunit (SUCLA2), mRNA	Q148D5	924	0	Succinate--CoA ligase [ADP-forming] subunit beta, mitochondrial	SPCC1620.08	464	5.20e-157	COG0045	pfam08442	ATP-grasp_2	ATP-grasp domain.	GO:0006099|tricarboxylic acid cycle;	GO:0005739|mitochondrion;	GO:0005524|ATP binding;GO:0046872|metal ion binding;GO:0004775	K01900	LSC2; succinyl-CoA synthetase beta subunit [EC:6.2.1.4 6.2.1.5]
SUB1	1332.841	-0.45527	0.166930127	-2.72732	0.0063851	0.038451	Not_Sig	no	gi|545873280|ref|XP_005671677.1|	456	8e-49	PREDICTED: activated RNA polymerase II transcriptional coactivator p15 [Sus scrofa]	gi|742178382|ref|XM_010853836.1|	3384	0	PREDICTED: Bison bison bison SUB1 homolog (S. cerevisiae) (SUB1), mRNA	Q5R6D0	176	5e-49	Activated RNA polymerase II transcriptional coactivator p15	.	.	.	.	pfam02229	PC4	Transcriptional Coactivator p15 (PC4). p15 has a bipartite structure composed of an amino-terminal regulatory domain and a carboxy-	GO:0006355|regulation of transcription, DNA-templated;GO:0006351|transcription, DNA-templated;	GO:0005634|nucleus;	GO:0003677|DNA binding;GO:0003713|transcription coactivator activity;	.	.
CAPN1	1658.659	0.316763	0.116162698	2.726888	0.0063935	0.038472	Not_Sig	no	gi|555964101|ref|XP_005894122.1|	3790	0	PREDICTED: calpain-1 catalytic subunit [Bos mutus]	gi|942050061|ref|XM_005894060.2|	2965	0	PREDICTED: Bos mutus calpain 1, (mu/I) large subunit (CAPN1), transcript variant X2, mRNA	Q27970	1461	0	Calpain-1 catalytic subunit	.	.	.	.	pfam00648	Peptidase_C2	Calpain family cysteine protease.	GO:0060056|mammary gland involution;GO:0006508|proteolysis;GO:0032801|receptor catabolic process;	GO:0005737|cytoplasm;GO:0005829|cytosol;GO:0070062|extracellular exosome;GO:0005925|focal adhesion;GO:0005764|lysosome;GO:00057	GO:0005509|calcium ion binding;GO:0004198|calcium-dependent	K01367	CAPN1; calpain-1 [EC:3.4.22.52]
RAB2B	483.5299	-0.33429	0.122608013	-2.72649	0.0064012	0.038472	Not_Sig	no	gi|300798691|ref|NP_001179594.1|	1166	5e-152	ras-related protein Rab-2B [Bos taurus]	gi|942068858|ref|XM_005900552.2|	2352	0	PREDICTED: Bos mutus RAB2B, member RAS oncogene family (RAB2B), transcript variant X1, mRNA	Q8WUD1	438	2e-147	Ras-related protein Rab-2B	.	.	.	.	pfam00071	Ras	Ras family. Includes sub-families Ras, Rab, Rac, Ral, Ran, Rap Ypt1 and more. Shares P-loop motif with GTP_EFTU, arf and	GO:0045921|positive regulation of exocytosis;GO:0015031|protein transport;GO:0007264|small GTPase mediated signal transduction;GO:0016192|vesicle-mediated transport;	GO:0005789|endoplasmic reticulum membrane;GO:0070062|extracellular exosome;GO:0000139|Golgi membrane;GO:0005886|plasma membrane;	GO:0005525|GTP binding;	K07878	RAB2B; Ras-related protein Rab-2B

<table>
<tr><td>SLC25A22</td><td>646.5875</td><td>0.387122</td><td>0.141999929</td><td>2.726212</td><td>0.0064066</td><td>0.038472</td><td>Not_Sig</td><td>no</td><td>gi|555966240|ref|XP_005895173.1|</td><td>1508</td><td>0</td><td>PREDICTED: mitochondrial glutamate carrier 1 [Bos mutus]</td><td>gi|982972255|ref|XM_005227301.3|</td><td>2466</td><td>0</td><td>PREDICTED: Bos taurus solute carrier family 25 (mitochondrial carrier: glutamate), member 22 (SLC25A22), transcript variant X1, mRNA</td><td>Q08DK4</td><td>584</td><td>0</td><td>Mitochondrial glutamate carrier 1</td><td>.</td><td>.</td><td>.</td><td>.</td><td>pfam00153</td><td>Mito_carr</td><td>Mitochondrial carrier protein.</td><td>GO:0006412|translation;</td><td>GO:0016021|integral component of membrane;GO:0005743|mitochondrial inner membrane;GO:0005654|nucleoplasm;</td><td>GO:0005313|L-glutamate transmembrane transporter</td><td>K15107</td><td>SLC25A18_22, GC; solute carrier family 25 (mitochondrial glutamate</td></tr>
<tr><td>TMEM222</td><td>473.2346</td><td>0.41288</td><td>0.151451207</td><td>2.726155</td><td>0.0064077</td><td>0.038472</td><td>Not_Sig</td><td>no</td><td>gi|555967831|ref|XP_005895960.1|</td><td>1045</td><td>4e-137</td><td>PREDICTED: transmembrane protein 222 [Bos mutus]</td><td>gi|942054794|ref|XM_005895898.2|</td><td>1623</td><td>0</td><td>PREDICTED: Bos mutus transmembrane protein 222 (TMEM222), mRNA</td><td>Q9H0R3</td><td>387</td><td>4e-131</td><td>Transmembrane protein 222</td><td>.</td><td>.</td><td>.</td><td>.</td><td>pfam05608</td><td>DUF778</td><td>Protein of unknown function (DUF778). This family consists of several eukaryotic proteins of unknown function.</td><td>.</td><td>GO:0016021|integral component of membrane;</td><td>.</td><td>.</td><td>.</td></tr>
<tr><td>PIK3C3</td><td>338.2282</td><td>-0.35611</td><td>0.130627801</td><td>-2.72612</td><td>0.0064084</td><td>0.038472</td><td>Not_Sig</td><td>no</td><td>gi|742152262|ref|XP_010844759.1|</td><td>4286</td><td>0</td><td>PREDICTED: phosphatidylinositol 3-kinase catalytic subunit type 3 isoform X2 [Bison bison bison]</td><td>gi|742152261|ref|XM_010846457.1|</td><td>5242</td><td>0</td><td>PREDICTED: Bison bison bison phosphatidylinositol 3-kinase, catalytic subunit type 3 (PIK3C3), transcript variant X2, mRNA</td><td>Q8NEB9</td><td>1624</td><td>0</td><td>Phosphatidylinositol 3-kinase catalytic subunit type 3</td><td>YLR240w</td><td>432</td><td>1.97e-130</td><td>COG5032</td><td>pfam00454</td><td>PI3_PI4_kinase</td><td>Phosphatidylinositol 3- and 4-kinase. Some members of this family probably do not have lipid kinase activity and are protein</td><td>GO:0006914|autophagy;GO:0042149|cellular response to glucose starvation;GO:0000910|cytokinesis;GO:0045022|early endosome to late endosome transport;GO:0007032|endosome organization;GO:0016236|macroautophagy;GO:0006661|phosphatidylinositol biosynthetic process;GO:0048015|phosphatidylinositol-mediated signaling;GO:0006497|protein lipidation;GO:0034497|protein localization to pre-autophagosomal structure;GO:0016485|protein processing;GO:0032465|regulation of cytokinesis;GO:0050708|regulation of protein</td><td>GO:0044754|autolysosome;GO:0005930|axoneme;GO:0005829|cytosol;GO:0005770|late endosome;GO:0016020|membrane;GO:003</td><td>GO:0016303|1-phosphatidylinositol-3-kinase activity;GO:0005524</td><td>K00914</td><td>PIK3C3, VPS34; phosphatidylinositol 3-kinase [EC:2.7.1.137]</td></tr>
<tr><td>N4BP2</td><td>387.1895</td><td>-0.43573</td><td>0.159836572</td><td>-2.72611</td><td>0.0064086</td><td>0.038472</td><td>Not_Sig</td><td>no</td><td>gi|555980245|ref|XP_005902050.1|</td><td>8388</td><td>0</td><td>PREDICTED: NEDD4-binding protein 2 [Bos mutus]</td><td>gi|942073242|ref|XM_014480205.1|</td><td>5351</td><td>0</td><td>PREDICTED: Bos mutus NEDD4 binding protein 2 (N4BP2), transcript variant X2, mRNA</td><td>Q86UW6</td><td>2536</td><td>0</td><td>NEDD4-binding protein 2</td><td>.</td><td>.</td><td>.</td><td>.</td><td>pfam08590</td><td>DUF1771</td><td>Domain of unknown function (DUF1771). This domain is always found adjacent to pfam01713.</td><td>.</td><td>GO:0005829|cytosol;</td><td>GO:0005524|ATP binding;GO:0046404|ATP-dependent polydeoxyribonucle</td><td>K15720</td><td>N4BP2; NEDD4-binding protein 2 [EC:3.-.-.-]</td></tr>
<tr><td>TYMS</td><td>534.2944</td><td>0.321296</td><td>0.117869713</td><td>2.725861</td><td>0.0064134</td><td>0.03848</td><td>Not_Sig</td><td>no</td><td>gi|594113010|ref|XP_006079272.1|</td><td>1560</td><td>0</td><td>PREDICTED: thymidylate synthase [Bubalus bubalis]</td><td>gi|741972600|ref|XM_010818818.1|</td><td>1477</td><td>0</td><td>PREDICTED: Bos taurus thymidylate synthetase (TYMS), transcript variant X1, mRNA</td><td>Q2TA32</td><td>584</td><td>0</td><td>Thymidylate synthase</td><td>ECU08g0090</td><td>360</td><td>6.03e-122</td><td>COG0207</td><td>pfam00303</td><td>Thymidylat_synt</td><td>Thymidylate synthase.</td><td>GO:0006231|dTMP biosynthetic process;GO:0006235|dTTP biosynthetic process;</td><td>GO:0005737|cytoplasm;GO:0005743|mitochondrial inner membrane;GO:0005759|mitochondrial matrix;GO:0005739|mitochondrion;GO:000</td><td>GO:0004799|thymidylate synthase activity;</td><td>K00560</td><td>thyA, TYMS; thymidylate synthase [EC:2.1.1.45]</td></tr>
<tr><td>PLBD2</td><td>8229.115</td><td>0.342819</td><td>0.125833984</td><td>2.724379</td><td>0.0064423</td><td>0.038633</td><td>Not_Sig</td><td>no</td><td>gi|594057830|ref|XP_006053085.1|</td><td>2901</td><td>0</td><td>PREDICTED: putative phospholipase B-like 2 isoform X1 [Bubalus bubalis]</td><td>gi|982950268|ref|XM_005217818.3|</td><td>2457</td><td>0</td><td>PREDICTED: Bos taurus phospholipase B domain containing 2 (PLBD2), transcript variant X3, mRNA</td><td>Q2KIY5</td><td>1093</td><td>0</td><td>Putative phospholipase B-like 2</td><td>.</td><td>.</td><td>.</td><td>.</td><td>pfam04916</td><td>Phospholip_B</td><td>Phospholipase B. Phospholipase B (PLB) catalyses the hydrolytic cleavage of both acylester bonds of glycerophospholipids. This</td><td>GO:0016042|lipid catabolic process;</td><td>GO:0043202|lysosomal lumen;</td><td>GO:0016787|hydrolase activity;</td><td>.</td><td>.</td></tr>
<tr><td>DUSP1</td><td>4822.511</td><td>0.313054</td><td>0.114920646</td><td>2.724087</td><td>0.006448</td><td>0.038647</td><td>Not_Sig</td><td>no</td><td>gi|803301768|ref|XP_012009397.1|</td><td>1949</td><td>0</td><td>PREDICTED: dual specificity protein phosphatase 1 [Ovis aries musimon]</td><td>gi|942072579|ref|XM_005901795.2|</td><td>2013</td><td>0</td><td>PREDICTED: Bos mutus dual specificity phosphatase 1 (DUSP1), transcript variant X1, mRNA</td><td>P28562</td><td>726</td><td>0</td><td>Dual specificity protein phosphatase 1</td><td>SPBC17A3.06</td><td>74.3</td><td>1.40e-13</td><td>COG2453</td><td>pfam00782</td><td>DSPc</td><td>Dual specificity phosphatase, catalytic domain. Ser/Thr and Tyr protein phosphatases. The enzyme's tertiary fold is highly</td><td>GO:0032870|cellular response to hormone stimulus;GO:0001706|endoderm formation;GO:0000188|inactivation of MAPK activity;GO:0035556|intracellular signal transduction;GO:0071850|mitotic cell cycle arrest;GO:0043066|negative regulation of apoptotic process;GO:2000279|negative regulation of DNA biosynthetic process;GO:0070373|negative regulation of ERK1 and ERK2 cascade;GO:0043407|negative regulation of MAP kinase activity;GO:0043409|negative regulation of MAPK cascade;GO:0051447|negative</td><td>GO:0005737|cytoplasm;GO:0005634|nucleus;</td><td>GO:0017017|MAP kinase tyrosine/serine/threonine phosphatase</td><td>K04459</td><td>DUSP, MKP; dual specificity MAP kinase phosphatase [EC:3.1.3.16</td></tr>
<tr><td>LOC102271224</td><td>1493.902</td><td>-0.3076</td><td>0.112928913</td><td>-2.72386</td><td>0.0064523</td><td>0.038653</td><td>Not_Sig</td><td>no</td><td>gi|655864474|ref|XP_008267717.1|</td><td>2843</td><td>0</td><td>PREDICTED: signal recognition particle 54 kDa protein isoform X3 [Oryctolagus cuniculus]</td><td>gi|982960504|ref|XM_005222057.3|</td><td>2309</td><td>0</td><td>PREDICTED: Bos taurus signal recognition particle 54kDa (SRP54), transcript variant X2, mRNA</td><td>Q5R4R6</td><td>979</td><td>0</td><td>Signal recognition particle 54 kDa protein</td><td>SPCC188.06c</td><td>528</td><td>7.28e-180</td><td>COG0541</td><td>pfam00448</td><td>SRP54</td><td>SRP54-type protein, GTPase domain. This family includes relatives of the G-domain of the SRP54 family of proteins.</td><td>GO:0045047|protein targeting to ER;GO:0042493|response to drug;GO:0006614|SRP-dependent cotranslational protein targeting to membrane;</td><td>GO:0016607|nuclear speck;GO:0005634|nucleus;GO:0005786|signal recognition particle, endoplasmic reticulum targeting;</td><td>GO:0008312|7S RNA binding;GO:0008144|drug</td><td>K03106</td><td>SRP54, ffh; signal recognition particle subunit SRP54</td></tr>
<tr><td>GLS</td><td>2823.419</td><td>-0.38611</td><td>0.141844278</td><td>-2.72208</td><td>0.0064872</td><td>0.038823</td><td>Not_Sig</td><td>no</td><td>gi|594044155|ref|XP_006046578.1|</td><td>2928</td><td>0</td><td>PREDICTED: glutaminase kidney isoform, mitochondrial isoform X2 [Bubalus bubalis]</td><td>gi|850477813|gb|CP011887.1|</td><td>9439</td><td>0</td><td>Ovis canadensis canadensis isolate 43U chromosome 2 sequence</td><td>D3Z7P3</td><td>971</td><td>0</td><td>Glutaminase kidney isoform, mitochondrial</td><td>all2934</td><td>262</td><td>4.32e-77</td><td>COG2066</td><td>pfam04960</td><td>Glutaminase</td><td>Glutaminase. This family of enzymes deaminates glutamine to glutamate EC:3.5.1.2.</td><td>GO:0007268|synaptic transmission;GO:0006537|glutamate biosynthetic process;GO:0006543|glutamine catabolic process;GO:0051289|protein homotetramerization;GO:0002087|regulation of respiratory gaseous exchange by neurological system process;GO:0001967|suckling behavior;</td><td>GO:0005829|cytosol;GO:0005759|mitochondrial matrix;GO:0005739|mitochondrion;</td><td>GO:0004359|glutaminase activity;</td><td>K01425</td><td>glsA, GLS; glutaminase [EC:3.5.1.2]</td></tr>
<tr><td>RPL35</td><td>5214.343</td><td>-0.35795</td><td>0.131499186</td><td>-2.72207</td><td>0.0064874</td><td>0.038823</td><td>Not_Sig</td><td>no</td><td>gi|555962089|ref|XP_005893138.1|</td><td>583</td><td>2e-73</td><td>PREDICTED: 60S ribosomal protein L35 [Bos mutus]</td><td>gi|742140373|ref|XM_010844043.1|</td><td>553</td><td>0</td><td>PREDICTED: Bison bison bison ribosomal protein L35 (RPL35), mRNA</td><td>Q3MHM7</td><td>201</td><td>1e-64</td><td>60S ribosomal protein L35</td><td>SPCC613.05c</td><td>108</td><td>2.88e-30</td><td>COG0255</td><td>pfam00831</td><td>Ribosomal_L29</td><td>Ribosomal L29 protein.</td><td>GO:0000463|maturation of LSU-rRNA from tricistronic rRNA transcript (SSU-rRNA, 5.8S rRNA, LSU-rRNA);GO:0006412|translation;</td><td>GO:0022625|cytosolic large ribosomal subunit;</td><td>GO:0003729|mRNA binding;GO:0003735|structural constituent of</td><td>K02918</td><td>RP-L35e, RPL35; large subunit ribosomal protein L35e</td></tr>
<tr><td>ZNF205</td><td>300.692</td><td>0.387274</td><td>0.142285302</td><td>2.721817</td><td>0.0064924</td><td>0.038833</td><td>Not_Sig</td><td>no</td><td>gi|529005816|ref|XP_005224498.1|</td><td>2780</td><td>0</td><td>PREDICTED: zinc finger protein 205 isoform X1 [Bos taurus]</td><td>gi|555949202|ref|XM_005886754.1|</td><td>2241</td><td>0</td><td>PREDICTED: Bos mutus zinc finger protein 205 (ZNF205), transcript variant X1, mRNA</td><td>Q58DK7</td><td>1066</td><td>0</td><td>Zinc finger protein 205</td><td>.</td><td>.</td><td>.</td><td>.</td><td>pfam01352</td><td>KRAB</td><td>KRAB box. The KRAB domain (or Kruppel-associated box) is present in about a third of zinc finger proteins containing C2H2 fingers.</td><td>GO:0000122|negative regulation of transcription from RNA polymerase II promoter;GO:0006351|transcription, DNA-templated;</td><td>GO:0005634|nucleus;</td><td>GO:0046872|metal ion binding;GO:0043565|sequence-specific</td><td>K09228</td><td>KRAB; KRAB domain-containing zinc finger protein</td></tr>
<tr><td>POLK</td><td>303.5968</td><td>-0.39036</td><td>0.143505843</td><td>-2.72018</td><td>0.0065246</td><td>0.03898</td><td>Not_Sig</td><td>no</td><td>gi|742156303|ref|XP_010845902.1|</td><td>4765</td><td>0</td><td>PREDICTED: DNA polymerase kappa [Bison bison bison]</td><td>gi|742156302|ref|XM_010847600.1|</td><td>4191</td><td>0</td><td>PREDICTED: Bison bison bison polymerase (DNA directed) kappa (POLK), mRNA</td><td>Q9UBT6</td><td>1448</td><td>0</td><td>DNA polymerase kappa</td><td>SPCC553.07c</td><td>167</td><td>4.47e-43</td><td>COG0389</td><td>pfam00817</td><td>IMS</td><td>impB/mucB/samB family. These proteins are involved in UV protection.</td><td>GO:0006281|DNA repair;GO:0006260|DNA replication;GO:0042276|error-prone translesion synthesis;GO:0006297|nucleotide-excision repair, DNA gap filling;GO:0033683|nucleotide-excision repair, DNA incision;GO:0006296|nucleotide-excision repair, DNA incision, 5'-to lesion;GO:0006283|transcription-coupled nucleotide-excision repair;GO:0019985|translesion synthesis;</td><td>GO:0043231|intracellular membrane-bounded organelle;GO:0005654|nucleoplasm;GO:0005634|nucleus;</td><td>GO:0003684|damaged DNA binding;GO:0003887|DNA-directed DNA</td><td>K03511</td><td>POLK; DNA polymerase kappa [EC:2.7.7.7]</td></tr>
<tr><td>SIRT2</td><td>1343.979</td><td>0.341073</td><td>0.125402007</td><td>2.719837</td><td>0.0065314</td><td>0.03898</td><td>Not_Sig</td><td>no</td><td>gi|594090707|ref|XP_006068754.1|</td><td>1896</td><td>0</td><td>PREDICTED: NAD-dependent protein deacetylase sirtuin-2 [Bubalus bubalis]</td><td>gi|555956929|ref|XM_005890541.1|</td><td>1828</td><td>0</td><td>PREDICTED: Bos mutus sirtuin 2 (SIRT2), mRNA</td><td>Q8VDQ8</td><td>657</td><td>0</td><td>NAD-dependent protein deacetylase sirtuin-2</td><td>SPCC132.02</td><td>268</td><td>2.74e-84</td><td>COG0846</td><td>pfam02146</td><td>SIR2</td><td>Sir2 family. This region is characteristic of Silent information regulator 2 (Sir2) proteins, or sirtuins. These are protein</td><td>GO:0006914|autophagy;GO:0051301|cell division;GO:0044242|cellular lipid catabolic process;GO:0061433|cellular response to caloric restriction;GO:0071872|cellular response to epinephrine stimulus;GO:0035729|cellular response to hepatocyte growth factor stimulus;GO:0071456|cellular response to hypoxia;GO:0071219|cellular response to molecule of bacterial origin;GO:0034599|cellular response to oxidative stress;GO:0048012|hepatocyte growth factor receptor signaling pathway;GO:0016575|histone</td><td>GO:0005814|centriole;GO:0005813|centrosome;GO:0005694|chromosome;GO:0005737|cytoplasm;GO:0005829|cytosol;GO:0097386|glial cell</td><td>GO:0048487|beta-tubulin binding;GO:0003682|chromatin</td><td>K11412</td><td>SIRT2, SIR2L2; NAD-dependent deacetylase sirtuin 2 [EC:3.5.1.-]</td></tr>
<tr><td>TSHZ3</td><td>441.4604</td><td>0.393394</td><td>0.144640642</td><td>2.719805</td><td>0.006532</td><td>0.03898</td><td>Not_Sig</td><td>no</td><td>gi|296478175|tpg|DAA20290.1|</td><td>4817</td><td>0</td><td>TPA: hypothetical protein BOS_17333 [Bos taurus]</td><td>gi|965809328|ref|XM_012120300.2|</td><td>4400</td><td>0</td><td>PREDICTED: Ovis aries musimon teashirt zinc finger homeobox 3 (TSHZ3), transcript variant X2, mRNA</td><td>Q63HK5</td><td>1790</td><td>0</td><td>Teashirt homolog 3</td><td>.</td><td>.</td><td>.</td><td>.</td><td>pfam09770</td><td>PAT1</td><td>Topoisomerase II-associated protein PAT1. Members of this family are necessary for accurate chromosome transmission during</td><td>GO:0007275|multicellular organismal development;GO:0045892|negative regulation of transcription, DNA-templated;GO:0002087|regulation of respiratory gaseous exchange by neurological system process;GO:0006357|regulation of transcription from RNA polymerase II promoter;GO:0006351|transcription, DNA-templated;</td><td>GO:0030426|growth cone;GO:0005654|nucleoplasm;GO:0005634|nucleus;</td><td>GO:0003682|chromatin binding;GO:0003677|DNA</td><td>K09236</td><td>TSHZ; teashirt</td></tr>
<tr><td>CHFR</td><td>745.8882</td><td>0.309438</td><td>0.113773351</td><td>2.719775</td><td>0.0065326</td><td>0.03898</td><td>Not_Sig</td><td>no</td><td>gi|742173499|ref|XP_010850763.1|</td><td>3142</td><td>0</td><td>PREDICTED: E3 ubiquitin-protein ligase CHFR [Bison bison bison]</td><td>gi|742173498|ref|XM_010852461.1|</td><td>3107</td><td>0</td><td>PREDICTED: Bison bison bison checkpoint with forkhead and ring finger domains, E3 ubiquitin protein ligase (CHFR), mRNA</td><td>Q96EP1</td><td>1016</td><td>0</td><td>E3 ubiquitin-protein ligase CHFR</td><td>.</td><td>.</td><td>.</td><td>.</td><td>pfam00498</td><td>FHA</td><td>FHA domain. The FHA (Forkhead-associated) domain is a phosphopeptide binding motif.</td><td>GO:0051301|cell division;GO:0007093|mitotic cell cycle checkpoint;GO:0007067|mitotic nuclear division;GO:0019941|modification-dependent protein catabolic process;GO:0032436|positive regulation of proteasomal ubiquitin-dependent protein catabolic process;GO:0031398|positive regulation of protein ubiquitination;GO:0031648|protein destabilization;GO:0000209|protein polyubiquitination;GO:0006511|ubiquitin-dependent protein catabolic process;</td><td>GO:0005634|nucleus;GO:0016605|PML body;</td><td>GO:0016874|ligase activity;GO:0000166|nucleotide binding;GO:0061630</td><td>K10644</td><td>CHFR; E3 ubiquitin-protein ligase CHFR [EC:2.3.2.27]</td></tr>
<tr><td>PTK2</td><td>1597.479</td><td>0.266593</td><td>0.098022665</td><td>2.719709</td><td>0.0065339</td><td>0.03898</td><td>Not_Sig</td><td>no</td><td>gi|803267610|ref|XP_011993551.1|</td><td>5588</td><td>0</td><td>PREDICTED: focal adhesion kinase 1 isoform X6 [Ovis aries musimon]</td><td>gi|742096474|ref|XM_010834797.1|</td><td>4186</td><td>0</td><td>PREDICTED: Bison bison bison protein tyrosine kinase 2 (PTK2), transcript variant X4, mRNA</td><td>P34152</td><td>2049</td><td>0</td><td>Focal adhesion kinase 1</td><td>.</td><td>.</td><td>.</td><td>.</td><td>pfam07714</td><td>Pkinase_Tyr</td><td>Protein tyrosine kinase.</td><td>GO:0001525|angiogenesis;GO:0007411|axon guidance;GO:0001568|blood vessel development;GO:0007155|cell adhesion;GO:0071560|cellular response to transforming growth factor beta stimulus;GO:0021955|central nervous system neuron axonogenesis;GO:0043542|endothelial cell migration;GO:0048013|ephrin receptor signaling pathway;GO:0007173|epidermal growth factor receptor signaling pathway;GO:0040023|establishment of nucleus localization;GO:0030198|extracellular matrix</td><td>GO:0016324|apical plasma membrane;GO:0005737|cytoplasm;GO:0005829|cytosol;GO:0031234|extrinsic component of cytoplasmic side of plasma</td><td>GO:0003779|actin binding;GO:0005524|ATP binding;GO:0008432</td><td>K05725</td><td>PTK2, FAK; focal adhesion kinase 1 [EC:2.7.10.2]</td></tr>
<tr><td>BNIP2</td><td>1367.672</td><td>-0.35115</td><td>0.129156432</td><td>-2.71883</td><td>0.0065513</td><td>0.039049</td><td>Not_Sig</td><td>no</td><td>gi|942078808|ref|XP_014336322.1|</td><td>1981</td><td>0</td><td>PREDICTED: BCL2/adenovirus E1B 19 kDa protein-interacting protein 2 [Bos mutus]</td><td>gi|742104967|ref|XM_010836844.1|</td><td>4553</td><td>0</td><td>PREDICTED: Bison bison bison BCL2/adenovirus E1B 19kDa interacting protein 2 (BNIP2), partial mRNA</td><td>Q12982</td><td>637</td><td>0</td><td>BCL2/adenovirus E1B 19 kDa protein-interacting protein 2</td><td>.</td><td>.</td><td>.</td><td>.</td><td>pfam12496</td><td>BNIP2</td><td>Bcl2-/adenovirus E1B nineteen kDa-interacting protein 2. This domain family is found in eukaryotes, and is typically</td><td>GO:0006915|apoptotic process;GO:0043066|negative regulation of apoptotic process;GO:0051149|positive regulation of muscle cell differentiation;</td><td>GO:0005737|cytoplasm;GO:0005829|cytosol;GO:0043231|intracellular membrane-bounded organelle;GO:0005635|nuclear envelope;GO:0048471|perinuclear region of</td><td>GO:0005509|calcium ion binding;GO:0005096|GTPase activator</td><td>K18448</td><td>BNIP2; BCL2/adenovirus E1B 19 kDa protein-interacting protein 2</td></tr>
<tr><td>WWP2</td><td>667.2953</td><td>0.379993</td><td>0.139765805</td><td>2.718781</td><td>0.0065523</td><td>0.039049</td><td>Not_Sig</td><td>no</td><td>gi|528990432|ref|XP_005218597.1|</td><td>4396</td><td>0</td><td>PREDICTED: NEDD4-like E3 ubiquitin-protein ligase WWP2 isoform X1 [Bos taurus]</td><td>gi|942056697|ref|XM_005896501.2|</td><td>4485</td><td>0</td><td>PREDICTED: Bos mutus WW domain containing E3 ubiquitin protein ligase 2 (WWP2), mRNA</td><td>Q9DBH0</td><td>1634</td><td>0</td><td>NEDD4-like E3 ubiquitin-protein ligase WWP2</td><td>YER125w</td><td>548</td><td>1.15e-175</td><td>COG5021</td><td>pfam00632</td><td>HECT</td><td>HECT-domain (ubiquitin-transferase). The name HECT comes from Homologous to the E6-AP Carboxyl Terminus.</td><td>GO:0010629|negative regulation of gene expression;GO:0051224|negative regulation of protein transport;GO:0043433|negative regulation of sequence-specific DNA binding transcription factor activity;GO:0000122|negative regulation of transcription from RNA polymerase II promoter;GO:0045892|negative regulation of transcription, DNA-templated;GO:0032410|negative regulation of transporter activity;GO:0045944|positive regulation of transcription from RNA polymerase II promoter;GO:0043161|proteasome-mediated ubiquitin-</td><td>GO:0005737|cytoplasm;GO:0070062|extracellular exosome;GO:0016020|membrane;GO:0005634|nucleus;</td><td>GO:0001085|RNA polymerase II transcription factor binding;GO:0008134</td><td>K05630</td><td>WWP2, AIP2; atrophin-1 interacting protein 2 (WW domain</td></tr>
<tr><td>BROX</td><td>785.1904</td><td>-0.38664</td><td>0.142250419</td><td>-2.71804</td><td>0.006567</td><td>0.039116</td><td>Not_Sig</td><td>no</td><td>gi|926710921|ref|XP_005690553.2|</td><td>2058</td><td>0</td><td>PREDICTED: BRO1 domain-containing protein BROX isoform X2 [Capra hircus]</td><td>gi|942078459|ref|XM_005903734.2|</td><td>3865</td><td>0</td><td>PREDICTED: Bos mutus BRO1 domain and CAAX motif containing (BROX), transcript variant X1, mRNA</td><td>Q5RDD7</td><td>777</td><td>0</td><td>BRO1 domain-containing protein BROX</td><td>.</td><td>.</td><td>.</td><td>.</td><td>pfam03097</td><td>BRO1</td><td>BRO1-like domain. This domain is found in a number proteins including Rhophilin and BRO1. It is known to have a role in endosomal</td><td>.</td><td>GO:0016020|membrane;</td><td>.</td><td>.</td><td>.</td></tr>
<tr><td>B4GALT4</td><td>808.8294</td><td>-0.28794</td><td>0.105965894</td><td>-2.71728</td><td>0.0065821</td><td>0.039186</td><td>Not_Sig</td><td>no</td><td>gi|555987307|ref|XP_005905506.1|</td><td>1632</td><td>0</td><td>PREDICTED: beta-1,4-galactosyltransferase 4 [Bos mutus]</td><td>gi|982910038|ref|XM_005201290.3|</td><td>2075</td><td>0</td><td>PREDICTED: Bos taurus UDP-Gal:betaGlcNAc beta 1,4- galactosyltransferase, polypeptide 4 (B4GALT4), transcript variant X1, mRNA</td><td>O60513</td><td>539</td><td>0</td><td>Beta-1,4-galactosyltransferase 4</td><td>.</td><td>.</td><td>.</td><td>.</td><td>pfam13733</td><td>Glyco_transf_7N</td><td>N-terminal region of glycosyl transferase group 7. This is the N-terminal half of a family of galactosyltransferases from a wide</td><td>GO:0018146|keratan sulfate biosynthetic process;GO:0006643|membrane lipid metabolic process;GO:0006486|protein glycosylation;</td><td>GO:0032580|Golgi cisterna membrane;GO:0000139|Golgi membrane;GO:0016021|integral component of membrane;</td><td>GO:0003831|beta-N-acetylglucosaminylglycopeptide beta-</td><td>K07969</td><td>B4GALT4; beta-1,4-galactosyltransferase 4 [EC:2.4.1.90 2.4.1.275 2.4.1.-]</td></tr>
<tr><td>IPO4</td><td>609.2989</td><td>0.336263</td><td>0.12375963</td><td>2.717067</td><td>0.0065863</td><td>0.039191</td><td>Not_Sig</td><td>no</td><td>gi|555986221|ref|XP_005904975.1|</td><td>4750</td><td>0</td><td>PREDICTED: importin-4 isoform X2 [Bos mutus]</td><td>gi|555986220|ref|XM_005904913.1|</td><td>3745</td><td>0</td><td>PREDICTED: Bos mutus importin 4 (IPO4), transcript variant X2, mRNA</td><td>Q8TEX9</td><td>1633</td><td>0</td><td>Importin-4</td><td>.</td><td>.</td><td>.</td><td>.</td><td>pfam13513</td><td>HEAT_EZ</td><td>HEAT-like repeat. The HEAT repeat family is related to armadillo/beta-catenin-like repeats (see pfam00514). These EZ repeats are</td><td>GO:0006335|DNA replication-dependent nucleosome assembly;GO:0006336|DNA replication-independent nucleosome assembly;GO:0006607|NLS-bearing protein import into nucleus;GO:0000059|protein import into nucleus, docking;GO:0000060|protein import into nucleus, translocation;GO:0015031|protein transport;GO:0006610|ribosomal protein import into nucleus;</td><td>GO:0005737|cytoplasm;GO:0016020|membrane;GO:0000790|nuclear chromatin;GO:0031965|nuclear membrane;GO:0034399|nuclear</td><td>GO:0008139|nuclear localization sequence binding;GO:0008565</td><td>.</td><td>.</td></tr>
<tr><td>SBF1</td><td>1225.16</td><td>0.328834</td><td>0.121043108</td><td>2.716671</td><td>0.0065942</td><td>0.039217</td><td>Not_Sig</td><td>no</td><td>gi|803209777|ref|XP_011965346.1|</td><td>8933</td><td>0</td><td>PREDICTED: myotubularin-related protein 5 isoform X7 [Ovis aries musimon]</td><td>gi|594085769|ref|XM_006066342.1|</td><td>3633</td><td>0</td><td>PREDICTED: Bubalus bubalis SET binding factor 1 (SBF1), transcript variant X5, mRNA</td><td>O95248</td><td>3284</td><td>0</td><td>Myotubularin-related protein 5</td><td>.</td><td>.</td><td>.</td><td>.</td><td>pfam06602</td><td>Myotub-related</td><td>Myotubularin-like phosphatase domain. This family represents the phosphatase domain within eukaryotic myotubularin-related</td><td>GO:0006470|protein dephosphorylation;GO:0007283|spermatogenesis;</td><td>GO:0016021|integral component of membrane;GO:0005634|nucleus;</td><td>GO:0019208|phosphatase regulator activity;GO:0008138|protein</td><td>K18061</td><td>SBF1_2, MTMR5_13; myotubularin-related protein 5/13</td></tr>
<tr><td>PAIP2</td><td>857.0331</td><td>-0.29155</td><td>0.107331533</td><td>-2.71632</td><td>0.0066012</td><td>0.039233</td><td>Not_Sig</td><td>no</td><td>gi|148664717|gb|EDK97133.1|</td><td>443</td><td>2e-49</td><td>polyadenylate-binding protein-interacting protein 2, isoform CRA_b, partial [Mus musculus]</td><td>gi|742165940|ref|XM_010850321.1|</td><td>1413</td><td>0</td><td>PREDICTED: Bison bison bison poly(A) binding protein interacting protein 2 (PAIP2), mRNA</td><td>Q9BPZ3</td><td>173</td><td>4e-50</td><td>Polyadenylate-binding protein-interacting protein 2</td><td>.</td><td>.</td><td>.</td><td>.</td><td>pfam07145</td><td>PAM2</td><td>Ataxin-2 C-terminal region. The PABP-interacting motif PAM2 has been identified in various eukaryotic proteins as an</td><td>GO:0007613|memory;GO:0045947|negative regulation of translational initiation;GO:1900271|regulation of long-term synaptic potentiation;GO:0007283|spermatogenesis;</td><td>GO:0005737|cytoplasm;</td><td>GO:0003729|mRNA binding;GO:0030371|translation repressor activity;</td><td>.</td><td>.</td></tr>
<tr><td>HS1BP3</td><td>518.1063</td><td>0.403565</td><td>0.148577055</td><td>2.716197</td><td>0.0066037</td><td>0.039233</td><td>Not_Sig</td><td>no</td><td>gi|375364520|ref|NP_001039745.2|</td><td>1592</td><td>0</td><td>HCLS1-binding protein 3 [Bos taurus]</td><td>gi|375364519|ref|NM_001046280.2|</td><td>3109</td><td>0</td><td>Bos taurus HCLS1 binding protein 3 (HS1BP3), mRNA</td><td>Q53T59</td><td>462</td><td>2e-150</td><td>HCLS1-binding protein 3</td><td>.</td><td>.</td><td>.</td><td>.</td><td>pfam00787</td><td>PX</td><td>PX domain. PX domains bind to phosphoinositides.</td><td>GO:0042981|regulation of apoptotic process;</td><td>GO:0005783|endoplasmic reticulum;GO:0005739|mitochondrion;</td><td>GO:0035091|phosphatidylinositol binding;</td><td>.</td><td>.</td></tr>
<tr><td>ARHGAP1</td><td>1890.358</td><td>0.411312</td><td>0.151479831</td><td>2.715291</td><td>0.0066218</td><td>0.03932</td><td>Not_Sig</td><td>no</td><td>gi|594035570|ref|XP_006042461.1|</td><td>2216</td><td>0</td><td>PREDICTED: rho GTPase-activating protein 1 isoform X1 [Bubalus bubalis]</td><td>gi|982946893|ref|XM_005216451.3|</td><td>2903</td><td>0</td><td>PREDICTED: Bos taurus Rho GTPase activating protein 1 (ARHGAP1), transcript variant X1, mRNA</td><td>Q07960</td><td>785</td><td>0</td><td>Rho GTPase-activating protein 1</td><td>.</td><td>.</td><td>.</td><td>.</td><td>pfam00620</td><td>RhoGAP</td><td>RhoGAP domain. GTPase activator proteins towards Rho/Rac/Cdc42-like small GTPases.</td><td>GO:0051056|regulation of small GTPase mediated signal transduction;GO:0007266|Rho protein signal transduction;GO:0007264|small GTPase mediated signal transduction;</td><td>GO:0005913|cell-cell adherens junction;GO:0005737|cytoplasm;GO:0005829|cytosol;GO:0070062|extracellular exosome;GO:0048471|perinuclear region of</td><td>GO:0098641|cadherin binding involved in cell-cell adhesion;GO:00050</td><td>K18470</td><td>ARHGAP1, CDC42GAP; Rho GTPase-activating protein 1</td></tr>
<tr><td>RBM18</td><td>495.2845</td><td>-0.34313</td><td>0.126386251</td><td>-2.71495</td><td>0.0066287</td><td>0.039331</td><td>Not_Sig</td><td>no</td><td>gi|555966165|ref|XP_005895138.1|</td><td>1017</td><td>3e-129</td><td>PREDICTED: probable RNA-binding protein 18 [Bos mutus]</td><td>gi|942052762|ref|XM_005895076.2|</td><td>2585</td><td>0</td><td>PREDICTED: Bos mutus RNA binding motif protein 18 (RBM18), mRNA</td><td>Q3ZCC5</td><td>394</td><td>4e-130</td><td>Probable RNA-binding protein 18</td><td>.</td><td>.</td><td>.</td><td>.</td><td>pfam00076</td><td>RRM_1</td><td>RNA recognition motif. (a.k.a. RRM, RBD, or RNP domain). The RRM motif is probably diagnostic of an RNA binding protein. RRMs</td><td>GO:0002230|positive regulation of defense response to virus by host;GO:0098792|xenophagy;</td><td>.</td><td>GO:0000166|nucleotide binding;GO:0003723|RNA binding;</td><td>.</td><td>.</td></tr>
<tr><td>ACTR1A</td><td>3542.134</td><td>0.247323</td><td>0.091099876</td><td>2.714856</td><td>0.0066305</td><td>0.039331</td><td>Not_Sig</td><td>no</td><td>gi|431895484|gb|ELK05000.1|</td><td>2139</td><td>0</td><td>Alpha-centractin [Pteropus alecto]</td><td>gi|555951835|ref|XM_005888051.1|</td><td>2899</td><td>0</td><td>PREDICTED: Bos mutus ARP1 actin-related protein 1 homolog A, centractin alpha (yeast) (ACTR1A), transcript variant X1, mRNA</td><td>P85515</td><td>776</td><td>0</td><td>Alpha-centractin</td><td>ECU01g0460</td><td>441</td><td>8.37e-146</td><td>COG5277</td><td>pfam00022</td><td>Actin</td><td>Actin.</td><td>.</td><td>GO:0005813|centrosome;GO:0005737|cytoplasm;GO:0005869|dynactin complex;GO:0070062|extracellular exosome;GO:1990752|microtubule</td><td>GO:0005524|ATP binding;</td><td>K16575</td><td>ACTR1, ARP1; centractin</td></tr>
<tr><td>TUBGCP2</td><td>1079.058</td><td>0.272046</td><td>0.100250527</td><td>2.713665</td><td>0.0066543</td><td>0.039447</td><td>Not_Sig</td><td>no</td><td>gi|148235721|ref|NP_001091618.1|</td><td>4457</td><td>0</td><td>gamma-tubulin complex component 2 [Bos taurus]</td><td>gi|942042477|ref|XM_005891443.2|</td><td>2843</td><td>0</td><td>PREDICTED: Bos mutus tubulin, gamma complex associated protein 2 (TUBGCP2), transcript variant X2, mRNA</td><td>Q921G8</td><td>1594</td><td>0</td><td>Gamma-tubulin complex component 2</td><td>.</td><td>.</td><td>.</td><td>.</td><td>pfam04130</td><td>Spc97_Spc98</td><td>Spc97 / Spc98 family. The spindle pole body (SPB) functions as the microtubule-organizing centre in yeast. Members of this family are</td><td>GO:0051298|centrosome duplication;GO:0031122|cytoplasmic microtubule organization;GO:0051415|interphase microtubule nucleation by interphase microtubule organizing center;GO:0007126|meiotic nuclear division;GO:0090307|mitotic spindle assembly;</td><td>GO:0005813|centrosome;GO:0000923|equatorial microtubule organizing center;GO:0008275|gamma-tubulin small complex;GO:0016020|membrane;GO:00058</td><td>GO:0043015|gamma-tubulin binding;GO:0005200|structural</td><td>K16569</td><td>TUBGCP2, GCP2; gamma-tubulin complex component 2</td></tr>
<tr><td>RPRD1A</td><td>229.6097</td><td>-0.40943</td><td>0.150883908</td><td>-2.71354</td><td>0.0066569</td><td>0.039447</td><td>Not_Sig</td><td>no</td><td>gi|148664582|gb|EDK96998.1|</td><td>1599</td><td>0</td><td>cDNA sequence BC021395, isoform CRA_a, partial [Mus musculus]</td><td>gi|942036795|ref|XM_005889225.2|</td><td>4388</td><td>0</td><td>PREDICTED: Bos mutus regulation of nuclear pre-mRNA domain containing 1A (RPRD1A), mRNA</td><td>Q5R8Y3</td><td>605</td><td>0</td><td>Regulation of nuclear pre-mRNA domain-containing protein 1A</td><td>.</td><td>.</td><td>.</td><td>.</td><td>pfam04818</td><td>CTD_bind</td><td>RNA polymerase II-binding domain. This domain binds to the phosphorylated C-terminal domain (CTD) of RNA polymerase II.</td><td>GO:0070940|dephosphorylation of RNA polymerase II C-terminal domain;</td><td>GO:0016591|DNA-directed RNA polymerase II, holoenzyme;</td><td>.</td><td>.</td><td>.</td></tr>
<tr><td>ZNF219</td><td>329.3896</td><td>0.409211</td><td>0.150825347</td><td>2.713148</td><td>0.0066647</td><td>0.039453</td><td>Not_Sig</td><td>no</td><td>gi|741884236|ref|XP_010808621.1|</td><td>2199</td><td>0</td><td>PREDICTED: zinc finger protein 219 [Bos taurus]</td><td>gi|942068900|ref|XM_005900560.2|</td><td>2671</td><td>0</td><td>PREDICTED: Bos mutus zinc finger protein 219 (ZNF219), transcript variant X2, mRNA</td><td>Q9P2Y4</td><td>803</td><td>0</td><td>Zinc finger protein 219</td><td>.</td><td>.</td><td>.</td><td>.</td><td>pfam13465</td><td>zf-H2C2_2</td><td>Zinc-finger double domain.</td><td>GO:0007275|multicellular organismal development;GO:0000122|negative regulation of transcription from RNA polymerase II promoter;GO:0045892|negative regulation of transcription, DNA-templated;GO:0001505|regulation of neurotransmitter levels;GO:0006355|regulation of transcription, DNA-templated;GO:0007165|signal transduction;GO:0006351|transcription, DNA-templated;</td><td>GO:0016021|integral component of membrane;GO:0005634|nucleus;</td><td>GO:0003677|DNA binding;GO:0004969|histamine receptor activity;GO:0046872</td><td>.</td><td>.</td></tr>
<tr><td>PKN1</td><td>1362.652</td><td>0.315949</td><td>0.116451175</td><td>2.713148</td><td>0.0066647</td><td>0.039453</td><td>Not_Sig</td><td>no</td><td>gi|122692517|ref|NP_001073715.1|</td><td>4012</td><td>0</td><td>serine/threonine-protein kinase N1 [Bos taurus]</td><td>gi|122692516|ref|NM_001080246.1|</td><td>2888</td><td>0</td><td>Bos taurus protein kinase N1 (PKN1), mRNA</td><td>A1A4I4</td><td>1550</td><td>0</td><td>Serine/threonine-protein kinase N1</td><td>.</td><td>.</td><td>.</td><td>.</td><td>pfam00069</td><td>Pkinase</td><td>Protein kinase domain.</td><td>GO:0010631|epithelial cell migration;GO:0035407|histone H3-T11 phosphorylation;GO:0035556|intracellular signal transduction;GO:0018105|peptidyl-serine phosphorylation;GO:0006468|protein phosphorylation;GO:2000145|regulation of cell motility;GO:0006357|regulation of transcription from RNA polymerase II promoter;GO:0006351|transcription, DNA-templated;</td><td>GO:0032154|cleavage furrow;GO:0005737|cytoplasm;GO:0005768|endosome;GO:0005622|intracellular;GO:0030496|midbody;GO:0005634|nucleus;GO:</td><td>GO:0050681|androgen receptor binding;GO:0005524|ATP</td><td>K06071</td><td>PKN; protein kinase N [EC:2.7.11.13]</td></tr>
<tr><td>TMEM64</td><td>159.1646</td><td>-0.48131</td><td>0.177420087</td><td>-2.71281</td><td>0.0066716</td><td>0.039457</td><td>Not_Sig</td><td>no</td><td>gi|803268759|ref|XP_011994107.1|</td><td>1472</td><td>0</td><td>PREDICTED: transmembrane protein 64 isoform X1 [Ovis aries musimon]</td><td>gi|982994771|ref|XM_015466401.1|</td><td>5019</td><td>0</td><td>PREDICTED: Bos taurus transmembrane protein 64 (TMEM64), transcript variant X2, mRNA</td><td>Q6YI46</td><td>410</td><td>1e-127</td><td>Transmembrane protein 64</td><td>slr0305</td><td>60.8</td><td>3.25e-09</td><td>COG0398</td><td>pfam09335</td><td>SNARE_assoc</td><td>SNARE associated Golgi protein. This is a family of SNARE associated Golgi proteins. The yeast member of this family</td><td>GO:0044339|canonical Wnt signaling pathway involved in osteoblast differentiation;GO:0090090|negative regulation of canonical Wnt signaling pathway;GO:0045668|negative regulation of osteoblast differentiation;GO:0045780|positive regulation of bone resorption;GO:0045600|positive regulation of fat cell differentiation;GO:0045672|positive regulation of osteoclast differentiation;GO:0043462|regulation of ATPase activity;GO:0051480|cytosolic calcium ion homeostasis;</td><td>GO:0005783|endoplasmic reticulum;GO:0016021|integral component of membrane;</td><td>.</td><td>.</td><td>.</td></tr>
<tr><td>C1D</td><td>664.9495</td><td>-0.49455</td><td>0.182303109</td><td>-2.71277</td><td>0.0066723</td><td>0.039457</td><td>Not_Sig</td><td>no</td><td>gi|594109700|ref|XP_006077700.1|</td><td>761</td><td>9e-93</td><td>PREDICTED: nuclear nucleic acid-binding protein C1D isoform X1 [Bubalus bubalis]</td><td>gi|942048838|ref|XM_005893569.2|</td><td>2181</td><td>0</td><td>PREDICTED: Bos mutus C1D nuclear receptor corepressor (C1D), mRNA</td><td>Q32PE4</td><td>291</td><td>1e-92</td><td>Nuclear nucleic acid-binding protein C1D</td><td>.</td><td>.</td><td>.</td><td>.</td><td>pfam04000</td><td>Sas10_Utp3</td><td>Sas10/Utp3/C1D family. This family contains Utp3 and LCP5 which are components of the U3 ribonucleoprotein complex. It also</td><td>GO:0006915|apoptotic process;GO:0000460|maturation of 5.8S rRNA;GO:0045892|negative regulation of transcription, DNA-templated;GO:0006351|transcription, DNA-templated;</td><td>GO:0005737|cytoplasm;GO:0005730|nucleolus;GO:0017053|transcriptional repressor complex;</td><td>GO:0003677|DNA binding;GO:0003723|RNA binding;GO:0003714</td><td>K12592</td><td>C1D, LRP1; exosome complex protein LRP1</td></tr>
<tr><td>RIMKLB</td><td>468.4124</td><td>-0.34445</td><td>0.12699654</td><td>-2.71227</td><td>0.0066825</td><td>0.039497</td><td>Not_Sig</td><td>no</td><td>gi|926692761|ref|XP_013819674.1|</td><td>1905</td><td>0</td><td>PREDICTED: beta-citrylglutamate synthase B isoform X1 [Capra hircus]</td><td>gi|982926843|ref|XM_015471271.1|</td><td>8291</td><td>0</td><td>PREDICTED: Bos taurus ribosomal modification protein rimK-like family member B (RIMKLB), transcript variant X1, mRNA</td><td>Q0VCE9</td><td>704</td><td>0</td><td>Beta-citrylglutamate synthase B</td><td>MA1361</td><td>114</td><td>1.52e-26</td><td>COG0189</td><td>pfam08443</td><td>RimK</td><td>RimK-like ATP-grasp domain. This ATP-grasp domain is found in the ribosomal S6 modification enzyme RimK.</td><td>GO:0006464|cellular protein modification process;</td><td>GO:0005737|cytoplasm;</td><td>GO:0005524|ATP binding;GO:0072591|citrate-L-glutamate ligase</td><td>K18310</td><td>RIMKLB, NAAGS-I; beta-citrylglutamate/N-acetylaspartylglutam</td></tr>
<tr><td>SF3B6</td><td>677.5208</td><td>-0.34269</td><td>0.126369395</td><td>-2.71178</td><td>0.0066923</td><td>0.039528</td><td>Not_Sig</td><td>no</td><td>gi|7706326|ref|NP_057131.1|</td><td>588</td><td>2e-73</td><td>splicing factor 3B subunit 6 [Homo sapiens]</td><td>gi|942054383|ref|XM_005895666.2|</td><td>789</td><td>0</td><td>PREDICTED: Bos mutus splicing factor 3b, subunit 6, 14kDa (SF3B6), mRNA</td><td>P59708</td><td>231</td><td>6e-75</td><td>Splicing factor 3B subunit 6</td><td>.</td><td>.</td><td>.</td><td>.</td><td>pfam00076</td><td>RRM_1</td><td>RNA recognition motif. (a.k.a. RRM, RBD, or RNP domain). The RRM motif is probably diagnostic of an RNA binding protein. RRMs</td><td>GO:0001825|blastocyst formation;GO:0000398|mRNA splicing, via spliceosome;</td><td>GO:0071013|catalytic step 2 spliceosome;GO:0005654|nucleoplasm;GO:0005634|nucleus;GO:0071011|precatalytic spliceosome;GO:0005689|U12-type</td><td>GO:0000166|nucleotide binding;GO:0044822|poly(A) RNA</td><td>K12833</td><td>SF3B14; pre-mRNA branch site protein p14</td></tr>
<tr><td>VMP1</td><td>2931.195</td><td>-0.25097</td><td>0.092553094</td><td>-2.71166</td><td>0.0066947</td><td>0.039528</td><td>Not_Sig</td><td>no</td><td>gi|115496266|ref|NP_001068836.1|</td><td>1914</td><td>0</td><td>vacuole membrane protein 1 [Bos taurus]</td><td>gi|982955891|ref|XM_005219885.3|</td><td>3248</td><td>0</td><td>PREDICTED: Bos taurus vacuole membrane protein 1 (VMP1), transcript variant X2, mRNA</td><td>Q0VCK9</td><td>741</td><td>0</td><td>Vacuole membrane protein 1</td><td>.</td><td>.</td><td>.</td><td>.</td><td>pfam09335</td><td>SNARE_assoc</td><td>SNARE associated Golgi protein. This is a family of SNARE associated Golgi proteins. The yeast member of this family</td><td>GO:0006914|autophagy;GO:0034329|cell junction assembly;GO:0007566|embryo implantation;GO:0007029|endoplasmic reticulum organization;GO:0006887|exocytosis;GO:0007030|Golgi organization;GO:0010506|regulation of autophagy;GO:0016337|single organismal cell-cell adhesion;</td><td>GO:0000421|autophagosome membrane;GO:0005783|endoplasmic reticulum;GO:0033116|endoplasmic reticulum-Golgi intermediate compartment</td><td>.</td><td>.</td><td>.</td></tr>
<tr><td>PUM2</td><td>1786.832</td><td>-0.30901</td><td>0.113975545</td><td>-2.71116</td><td>0.0067048</td><td>0.039565</td><td>Not_Sig</td><td>no</td><td>gi|555967333|ref|XP_005895714.1|</td><td>4139</td><td>0</td><td>PREDICTED: pumilio homolog 2 isoform X3 [Bos mutus]</td><td>gi|742120777|ref|XM_010840532.1|</td><td>5942</td><td>0</td><td>PREDICTED: Bison bison bison pumilio RNA-binding family member 2 (PUM2), transcript variant X8, mRNA</td><td>Q8TB72</td><td>794</td><td>0</td><td>Pumilio homolog 2</td><td>SPAC1687.22c</td><td>369</td><td>2.94e-113</td><td>COG5099</td><td>pfam00806</td><td>PUF</td><td>Pumilio-family RNA binding repeat. Puf repeats (aka PUM-HD, Pumilio homology domain) are necessary and sufficient for sequence specific</td><td>GO:2000637|positive regulation of gene silencing by miRNA;GO:1900246|positive regulation of RIG-I signaling pathway;GO:0010608|posttranscriptional regulation of gene expression;GO:0051983|regulation of chromosome segregation;GO:0060964|regulation of gene silencing by miRNA;GO:0043488|regulation of mRNA stability;GO:0034063|stress granule assembly;</td><td>GO:0005737|cytoplasm;GO:0010494|cytoplasmic stress granule;GO:0031965|nuclear membrane;GO:0048471|perinuclear region of cytoplasm;</td><td>GO:0003730|mRNA 3'-UTR binding;GO:0044822|poly(A) RNA</td><td>K17943</td><td>PUM; pumilio RNA-binding family</td></tr>
<tr><td>CAPN5</td><td>732.345</td><td>0.32349</td><td>0.119324533</td><td>2.711013</td><td>0.0067078</td><td>0.039565</td><td>Not_Sig</td><td>no</td><td>gi|440910241|gb|ELR60056.1|</td><td>3490</td><td>0</td><td>Calpain-5, partial [Bos mutus]</td><td>gi|982946524|ref|XM_005216288.3|</td><td>4388</td><td>0</td><td>PREDICTED: Bos taurus calpain 5 (CAPN5), transcript variant X1, mRNA</td><td>O15484</td><td>1263</td><td>0</td><td>Calpain-5</td><td>.</td><td>.</td><td>.</td><td>.</td><td>pfam00648</td><td>Peptidase_C2</td><td>Calpain family cysteine protease.</td><td>GO:0007165|signal transduction;</td><td>GO:0009986|cell surface;GO:0005737|cytoplasm;GO:0070062|extracellular exosome;GO:0005925|focal adhesion;</td><td>GO:0004198|calcium-dependent cysteine-type endopeptidase</td><td>K08574</td><td>CAPN5; calpain-5 [EC:3.4.22.-]</td></tr>
<tr><td>BZW1</td><td>4922.891</td><td>-0.29552</td><td>0.109029873</td><td>-2.71047</td><td>0.0067188</td><td>0.03961</td><td>Not_Sig</td><td>no</td><td>gi|62898051|dbj|BAD96965.1|</td><td>2186</td><td>0</td><td>BZW1 protein variant [Homo sapiens]</td><td>gi|331028746|ref|NM_001206613.1|</td><td>5004</td><td>0</td><td>Bos taurus basic leucine zipper and W2 domains 1 (BZW1), mRNA</td><td>Q6P7P5</td><td>835</td><td>0</td><td>Basic leucine zipper and W2 domain-containing protein 1</td><td>.</td><td>.</td><td>.</td><td>.</td><td>pfam02020</td><td>W2</td><td>eIF4-gamma/eIF5/eIF2-epsilon. This domain of unknown function is found at the C-terminus of several translation initiation</td><td>GO:0006355|regulation of transcription, DNA-templated;GO:0006351|transcription, DNA-templated;</td><td>GO:0005737|cytoplasm;GO:0016020|membrane;</td><td>GO:0044822|poly(A) RNA binding;</td><td>.</td><td>.</td></tr>
</table>

BLOC1S2	390.9658	-0.3744	0.13818884	-2.70933	0.006742	0.039726	Not_Sig	no	gi|803229704|ref|XP_011975118.1|	556	2e-67	PREDICTED: biogenesis of lysosome-related organelles complex 1 subunit 2 [Ovis aries musimon]	gi|742200908|ref|XM_010859528.1|	924	0	PREDICTED: Bison bison bison biogenesis of lysosomal organelles complex-1, subunit 2 (BLOC1S2), mRNA	Q32WR5	206	2e-64	Biogenesis of lysosome-related organelles complex-1 subunit 2	.	.	.	.	pfam10046	BLOC1_2	Biogenesis of lysosome-related organelles complex-1 subunit 2. Members of this family of proteins play a role in cellular proliferation,	GO:0008089|anterograde axon cargo transport;GO:0048490|anterograde synaptic vesicle transport;GO:0008625|extrinsic apoptotic signaling pathway via death domain receptors;GO:0032418|lysosome localization;GO:0097345|mitochondrial outer membrane permeabilization;GO:0031175|neuron projection development;GO:0045944|positive regulation of transcription from RNA polymerase II promoter;	GO:1904115|axon cytoplasm;GO:0031083|BLOC-1 complex;GO:0005813|centrosome;GO:0005737|cytoplasm;GO:0005765|lysosomal	GO:0008022|protein C-terminus binding;	K16750	BLOC1S2; biogenesis of lysosome-related organelles complex 1 subunit 2
NAA35	412.6586	-0.35096	0.12955822	-2.70893	0.0067501	0.039753	Not_Sig	no	gi|803287555|ref|XP_012003439.1|	3901	0	PREDICTED: N-alpha-acetyltransferase 35, NatC auxiliary subunit isoform X3 [Ovis aries musimon]	gi|982934108|ref|XM_010808132.2|	2724	0	PREDICTED: Bos taurus N(alpha)-acetyltransferase 35, NatC auxiliary subunit (NAA35), transcript variant X2, mRNA	Q5RBT3	1486	0	N-alpha-acetyltransferase 35, NatC auxiliary subunit	.	.	.	.	pfam04112	Mak10	Mak10 subunit, NatC N(alpha)-terminal acetyltransferase. NatC N(alpha)-terminal acetyltransferases contains	GO:0043066|negative regulation of apoptotic process;GO:0048659|smooth muscle cell proliferation;	GO:0005737|cytoplasm;GO:0031417|NatC complex;	.	.	.
PAF1	752.3338	0.285526	0.105467477	2.707243	0.0067845	0.039935	Not_Sig	no	gi|731289453|ref|XP_010612826.1|	2039	0	PREDICTED: RNA polymerase II-associated factor 1 homolog [Fukomys damarensis]	gi|942040620|ref|XM_005890549.2|	2245	0	PREDICTED: Bos mutus PAF1 homolog, Paf1/RNA polymerase II complex component (PAF1), transcript variant X1, mRNA	Q8K2T8	789	0	RNA polymerase II-associated factor 1 homolog	.	.	.	.	pfam03985	Paf1	Paf1. Members of this family are components of the RNA polymerase II associated Paf1 complex. The Paf1 complex	GO:0071222|cellular response to lipopolysaccharide;GO:0001711|endodermal cell fate commitment;GO:0033523|histone H2B ubiquitination;GO:0010390|histone monoubiquitination;GO:0006378|mRNA polyadenylation;GO:0045638|negative regulation of myeloid cell differentiation;GO:0000122|negative regulation of transcription from RNA polymerase II promoter;GO:0016584|nucleosome positioning;GO:0031062|positive regulation of histone methylation;GO:0031442|positive regulation of mRNA 3'-end	GO:0016593|Cdc73/Paf1 complex;GO:0030054|cell junction;GO:0005737|cytoplasm;GO:0016020|membrane;GO:0005654|nucleoplasm;	GO:0003682|chromatin binding;GO:0000993|RNA polymerase II	K15174	PAF1; RNA polymerase II-associated factor 1
H3-3B	4334.646	-0.28477	0.105203748	-2.70685	0.0067925	0.039962	Not_Sig	no	gi|602731422|ref|XP_007451114.1|	765	5e-92	PREDICTED: histone H3.3-like [Lipotes vexillifer]	gi|742187422|ref|XM_010856181.1|	2720	0	PREDICTED: Bison bison bison H3 histone, family 3B (H3.3B) (H3F3B), mRNA	Q6P823	273	2e-84	Histone H3.3	.	.	.	.	pfam00125	Histone	Core histone H2A/H2B/H3/H4.	GO:0006334|nucleosome assembly;	GO:0000786|nucleosome;GO:0005634|nucleus;	GO:0031492|nucleosomal DNA binding;	K11253	H3; histone H3
ZC3H12A	420.4322	0.364549	0.134704196	2.70629	0.006804	0.040005	Not_Sig	no	gi|440908122|gb|ELR58180.1|	2676	0	Ribonuclease ZC3H12A, partial [Bos mutus]	gi|555962454|ref|XM_005893255.1|	2547	0	PREDICTED: Bos mutus zinc finger CCCH-type containing 12A (ZC3H12A), mRNA	A6QQJ8	1003	0	Ribonuclease ZC3H12A	.	.	.	.	pfam11977	RNase_Zc3h12a	Zc3h12a-like Ribonuclease NYN domain. This domain is found in the Zc3h12a protein which has shown to be a ribonuclease that	GO:0061158|3'-UTR-mediated mRNA destabilization;GO:0001525|angiogenesis;GO:0006915|apoptotic process;GO:0030154|cell differentiation;GO:1990869|cellular response to chemokine;GO:0042149|cellular response to glucose starvation;GO:0071347|cellular response to interleukin-1;GO:1904637|cellular response to ionomycin;GO:0071222|cellular response to lipopolysaccharide;GO:0034599|cellular response to oxidative stress;GO:1904628|cellular response to phorbol 13-acetate 12-	GO:0005737|cytoplasm;GO:0000932|cytoplasmic mRNA processing body;GO:0005856|cytoskeleton;GO:0042406|extrinsic component of endoplasmic	GO:0003682|chromatin binding;GO:0003677|DNA	K18668	ZC3H12, MCPIP; ribonuclease ZC3H12 [EC:3.1.-.-]
NPC1	3574.901	0.298398	0.110266539	2.706156	0.0068067	0.040005	Not_Sig	no	gi|440903483|gb|ELR54134.1|	6727	0	Niemann-Pick C1 protein, partial [Bos mutus]	gi|555973882|ref|XM_005898875.1|	4658	0	PREDICTED: Bos mutus Niemann-Pick disease, type C1 (NPC1), transcript variant X1, mRNA	P56941	2377	0	Niemann-Pick C1 protein	.	.	.	.	pfam02460	Patched	Patched family. The transmembrane protein Patched is a receptor for the morphogene Sonic Hedgehog. This protein	GO:0008206|bile acid metabolic process;GO:0042632|cholesterol homeostasis;GO:0008203|cholesterol metabolic process;GO:0030301|cholesterol transport;GO:0007041|lysosomal transport;GO:0006486|protein glycosylation;	GO:0005783|endoplasmic reticulum;GO:0005576|extracellular region;GO:0005887|integral component of plasma membrane;GO:0031902|late	GO:0015485|cholesterol binding;GO:0005319|lipid transporter	K12385	NPC1; Niemann-Pick C1 protein
RBM10	1091.932	0.322047	0.119026564	2.705677	0.0068165	0.040042	Not_Sig	no	gi|594069876|ref|XP_006058831.1|	4551	0	PREDICTED: RNA-binding protein 10 isoform X1 [Bubalus bubalis]	gi|594069875|ref|XM_006058769.1|	3485	0	PREDICTED: Bubalus bubalis RNA binding motif protein 10 (RBM10), transcript variant X1, mRNA	P98175	1494	0	RNA-binding protein 10	.	.	.	.	pfam01585	G-patch	G-patch domain. This domain is found in a number of RNA binding proteins, and is also found in proteins that contain RNA binding	GO:0006397|mRNA processing;GO:0000381|regulation of alternative mRNA splicing, via spliceosome;GO:0042981|regulation of apoptotic process;GO:0008380|RNA splicing;	GO:0005654|nucleoplasm;GO:0005634|nucleus;	GO:0000166|nucleotide binding;GO:0044822|poly(A) RNA	K13094	RBM5_10; RNA-binding protein 5/10
TEAD3	1863.96	0.405038	0.149710188	2.705483	0.0068205	0.040045	Not_Sig	no	gi|970711297|ref|XP_015092824.1|	1897	0	PREDICTED: transcriptional enhancer factor TEF-5 [Vicugna pacos]	gi|942101181|ref|XM_014483422.1|	2648	0	PREDICTED: Bos mutus TEA domain family member 3 (TEAD3), mRNA	Q99594	694	0	Transcriptional enhancer factor TEF-5	.	.	.	.	pfam01285	TEA	TEA/ATTS domain family.	GO:0055059|asymmetric neuroblast division;GO:0007565|female pregnancy;GO:0035329|hippo signaling;GO:0045944|positive regulation of transcription from RNA polymerase II promoter;GO:0006357|regulation of transcription from RNA polymerase II promoter;GO:0006367|transcription initiation from RNA polymerase II promoter;	GO:0005654|nucleoplasm;	GO:0003677|DNA binding;GO:0001085|RNA polymerase II transcription factor	K09448	TEAD; transcriptional enhancer factor
ZMIZ1	1499.17	0.385681	0.142567436	2.705255	0.0068252	0.040052	Not_Sig	no	gi|664713949|ref|XP_008513944.1|	4467	0	PREDICTED: zinc finger MIZ domain-containing protein 1 isoform X1 [Equus przewalskii]	gi|742093815|ref|XM_010834169.1|	6796	0	PREDICTED: Bison bison bison zinc finger, MIZ-type containing 1 (ZMIZ1), transcript variant X3, mRNA	Q6P1E1	1670	0	Zinc finger MIZ domain-containing protein 1	.	.	.	.	pfam02891	zf-MIZ	MIZ/SP-RING zinc finger. This domain has SUMO (small ubiquitin-like modifier) ligase activity and is involved in DNA	GO:0048844|artery morphogenesis;GO:0007569|cell aging;GO:0048589|developmental growth;GO:0003007|heart morphogenesis;GO:0001701|in utero embryonic development;GO:0048146|positive regulation of fibroblast proliferation;GO:0045944|positive regulation of transcription from RNA polymerase II promoter;GO:0006351|transcription, DNA-templated;GO:0001570|vasculogenesis;GO:0007296|vitellogenesis;	GO:0005737|cytoplasm;GO:0043231|intracellular membrane-bounded organelle;GO:0016607|nuclear speck;GO:0005654|nucleoplasm;	GO:0008270|zinc ion binding;	.	.
AP3D1	3342.847	0.255731	0.094544699	2.704872	0.0068331	0.040066	Not_Sig	no	gi|254281264|ref|NP_776423.3|	5556	0	AP-3 complex subunit delta-1 [Bos taurus]	gi|254281263|ref|NM_173998.4|	4576	0	Bos taurus adaptor related protein complex 3 delta 1 subunit (AP3D1), mRNA	Q865S1	2144	0	AP-3 complex subunit delta-1	.	.	.	.	pfam01602	Adaptin_N	Adaptin N terminal region. This family consists of the N terminal region of various alpha, beta and gamma subunits of the AP-1, AP-2	GO:0008089|anterograde axon cargo transport;GO:0048490|anterograde synaptic vesicle transport;GO:0048007|antigen processing and presentation, exogenous lipid antigen via MHC class Ib;GO:0035646|endosome to melanosome transport;GO:0006886|intracellular protein transport;GO:0051138|positive regulation of NK T cell differentiation;GO:0072657|protein localization to membrane;GO:0033365|protein localization to organelle;GO:0061088|regulation of sequestering of zinc ion;GO:0048499|synaptic vesicle membrane organization;	GO:0030123|AP-3 adaptor complex;GO:1904115|axon cytoplasm;GO:0010008|endosome membrane;GO:0000139|Golgi	.	K12396	AP3D1; AP-3 complex subunit delta-1
PGM3	758.6037	-0.30891	0.11420888	-2.7048	0.0068346	0.040066	Not_Sig	no	gi|440899586|gb|ELR50870.1|	2911	0	Phosphoacetylglucosamine mutase, partial [Bos mutus]	gi|942077217|ref|XM_014480641.1|	2084	0	PREDICTED: Bos mutus phosphoglucomutase 3 (PGM3), transcript variant X1, mRNA	O95394	1006	0	Phosphoacetylglucosamine mutase	YEL058w	412	1.58e-135	COG1109	pfam02878	PGM_PMM_I	Phosphoglucomutase/phosphomannomutase, alpha/beta/alpha domain I.	GO:0006041|glucosamine metabolic process;GO:0019255|glucose 1-phosphate metabolic process;GO:0030097|hemopoiesis;GO:0006487|protein N-linked glycosylation;GO:0006493|protein O-linked glycosylation;GO:0007283|spermatogenesis;GO:0006048|UDP-N-acetylglucosamine biosynthetic process;	GO:0005829|cytosol;	GO:0000287|magnesium ion binding;GO:0004610|phosphoacetylgluc	K01836	E5.4.2.3; phosphoacetylglucosamine mutase [EC:5.4.2.3]
PI4KB	961.6289	0.341188	0.126183599	2.703899	0.0068531	0.040154	Not_Sig	no	gi|982916570|ref|XP_015318084.1|	4355	0	PREDICTED: phosphatidylinositol 4-kinase beta isoform X4 [Bos taurus]	gi|742192633|ref|XM_010857516.1|	3338	0	PREDICTED: Bison bison bison phosphatidylinositol 4-kinase, catalytic, beta (PI4KB), mRNA	Q9UBF8	1662	0	Phosphatidylinositol 4-kinase beta	.	.	.	.	pfam00454	PI3_PI4_kinase	Phosphatidylinositol 3- and 4-kinase. Some members of this family probably do not have lipid kinase activity and are protein	GO:0006661|phosphatidylinositol biosynthetic process;GO:0048015|phosphatidylinositol-mediated signaling;GO:0006898|receptor-mediated endocytosis;GO:0007165|signal transduction;	GO:0005737|cytoplasm;GO:0005829|cytosol;GO:0005768|endosome;GO:0000139|Golgi membrane;GO:0005741|mitochondrial outer membrane;GO:0048471|perinuclear	GO:0004430|1-phosphatidylinositol 4-kinase activity;GO:0071889	.	.
UBE2O	729.9554	0.318781	0.117927566	2.703195	0.0068677	0.040197	Not_Sig	no	gi|297487400|ref|XP_002696213.1|	5621	0	PREDICTED: (E3-independent) E2 ubiquitin-conjugating enzyme isoform X1 [Bos taurus]	gi|983002694|ref|XM_015468273.1|	5231	0	PREDICTED: Bos taurus ubiquitin conjugating enzyme E2O (UBE2O), transcript variant X1, mRNA	Q9C0C9	2048	0	(E3-independent) E2 ubiquitin-conjugating enzyme	.	.	.	.	pfam00179	UQ_con	Ubiquitin-conjugating enzyme. Proteins destined for proteasome-mediated degradation may be ubiquitinated. Ubiquitination	GO:0030513|positive regulation of BMP signaling pathway;GO:0070534|protein K63-linked ubiquitination;GO:0006513|protein monoubiquitination;GO:0042147|retrograde transport, endosome to Golgi;	GO:0005737|cytoplasm;GO:0005829|cytosol;GO:0005634|nucleus;	GO:0005524|ATP binding;GO:0044822|poly(A) RNA binding;GO:0061631	K10581	UBE2O; ubiquitin-conjugating enzyme E2 O [EC:2.3.2.24]
LOC138986233	159.4338	-0.5095	0.188486623	-2.70309	0.0068698	0.040197	Not_Sig	no	.	.	.	.	gi|983009294|ref|XR_001499839.1|	1163	0	PREDICTED: Bos taurus uncharacterized LOC107131948 (LOC107131948), transcript variant X1, ncRNA	.	.	.	.	.	.	.	.	.	.	.	.	.	.	.	.
COX7C	829.897	-0.38116	0.14101107	-2.70304	0.0068708	0.040197	Not_Sig	no	gi|556765364|ref|XP_005977945.1|	369	7e-41	PREDICTED: keratin, type I cytoskeletal 9-like [Pantholops hodgsonii]	gi|942084889|ref|XM_005905820.2|	490	0	PREDICTED: Bos mutus cytochrome c oxidase subunit 7C, mitochondrial (LOC102264900), transcript variant X1, mRNA	P00430	132	2e-38	Cytochrome c oxidase subunit 7C, mitochondrial	.	.	.	.	pfam02935	COX7C	Cytochrome c oxidase subunit VIIc. Cytochrome c oxidase, a 13 sub-unit complex, EC:1.9.3.1 is the terminal oxidase in the	.	GO:0016021|integral component of membrane;GO:0005743|mitochondrial inner membrane;GO:0045277|respiratory chain complex IV;	GO:0004129|cytochrome-c oxidase activity;	K02272	COX7C; cytochrome c oxidase subunit 7c
SHOC2	667.8241	-0.32505	0.120270206	-2.7027	0.0068779	0.040204	Not_Sig	no	gi|156120535|ref|NP_001095413.1|	2463	0	leucine-rich repeat protein SHOC-2 [Bos taurus]	gi|742200169|ref|XM_010859363.1|	4042	0	PREDICTED: Bison bison bison soc-2 suppressor of clear homolog (C. elegans) (SHOC2), transcript variant X3, mRNA	A6QLV3	953	0	Leucine-rich repeat protein SHOC-2	alr0124_1	150	9.87e-38	COG4886	pfam13855	LRR_8	Leucine rich repeat.	GO:0046579|positive regulation of Ras protein signal transduction;	GO:0005737|cytoplasm;GO:0005654|nucleoplasm;GO:0005634|nucleus;GO:0000164|protein phosphatase type 1 complex;	GO:0019903|protein phosphatase binding;	K19613	SHOC2, SUR8; leucine-rich repeat protein SHOC2
KIF3A	544.5494	-0.38614	0.142876326	-2.70264	0.0068791	0.040204	Not_Sig	no	gi|675644673|ref|XP_002744651.2|	3260	0	PREDICTED: kinesin-like protein KIF3A isoform X1 [Callithrix jacchus]	gi|742217487|ref|XM_010863602.1|	4652	0	PREDICTED: Bison bison bison kinesin family member 3A (KIF3A), transcript variant X1, mRNA	Q4R628	773	0	Kinesin-like protein KIF3A	SPAC1834.07	272	2.78e-78	COG5059	pfam00225	Kinesin	Kinesin motor domain.	GO:0042384|cilium assembly;GO:0007018|microtubule-based movement;	GO:0005737|cytoplasm;GO:0005874|microtubule;GO:0072372|primary cilium;	GO:0005524|ATP binding;GO:0003777|microtubule motor activity;	K10394	KIF3_17; kinesin family member 3/17
CACNA2D1	757.1844	-0.40583	0.150216387	-2.70162	0.0069002	0.040307	Not_Sig	no	gi|594092269|ref|XP_006069493.1|	5823	0	PREDICTED: voltage-dependent calcium channel subunit alpha-2/delta-1 isoform X1 [Bubalus bubalis]	gi|742158067|ref|XM_010848131.1|	7572	0	PREDICTED: Bison bison bison calcium channel, voltage-dependent, alpha 2/delta subunit 1 (CACNA2D1), transcript variant X1, mRNA	P54289	2185	0	Voltage-dependent calcium channel subunit alpha-2/delta-1	sll0103	52.8	9.13e-06	COG2304	pfam08473	VGCC_alpha2	Neuronal voltage-dependent calcium channel alpha 2acd. This eukaryotic domain has been found in the neuronal voltage-dependent	GO:0061577|generation of L-type calcium current;GO:0006816|calcium ion transport;GO:0060402|calcium ion transport into cytosol;GO:0061337|cardiac conduction;GO:0086002|cardiac muscle cell action potential involved in contraction;GO:0086012|membrane depolarization during cardiac muscle cell action potential;GO:1901843|positive regulation of high voltage-gated calcium channel activity;GO:0051924|regulation of calcium ion transport;GO:0086091|regulation of heart rate by cardiac conduction;GO:0098903|regulation	GO:0070062|extracellular exosome;GO:1990454|L-type voltage-gated calcium channel complex;GO:0005886|plasma	GO:0046872|metal ion binding;GO:0005245|voltage-gated	K04858	CACNA2D1; voltage-dependent calcium channel alpha-2/delta-1
CALM3	1915.854	0.261986	0.096981513	2.701398	0.0069049	0.040313	Not_Sig	no	gi|744616901|ref|XP_010996006.1|	794	2e-97	PREDICTED: calmodulin [Camelus dromedarius]	gi|402693480|ref|NM_001046249.2|	2035	0	Bos taurus calmodulin 3 (phosphorylase kinase, delta) (CALM3), mRNA	P62155	299	9e-96	Calmodulin	SPAC3A12.14	233	1.63e-72	COG5126	pfam13499	EF-hand_7	EF-hand domain pair.	.	.	GO:0005509|calcium ion binding;GO:0005102|receptor binding;	K02183	CALM; calmodulin
OARD1	382.0266	-0.3793	0.140423776	-2.70114	0.0069102	0.040324	Not_Sig	no	gi|116004445|ref|NP_001070581.1|	815	2e-105	O-acetyl-ADP-ribose deacetylase 1 [Bos taurus]	gi|982963563|ref|XM_005223511.2|	1160	0	PREDICTED: Bos taurus O-acyl-ADP-ribose deacylase 1 (OARD1), transcript variant X4, mRNA	Q1LZ74	318	6e-107	O-acetyl-ADP-ribose deacetylase 1	mll3540	49.7	1.25e-06	COG2110	pfam01661	Macro	Macro domain. This domain is an ADP-ribose binding module. It is found in a number of otherwise unrelated proteins. It is found at	GO:0042278|purine nucleoside metabolic process;	.	GO:0019213|deacetylase activity;GO:0001883|purine nucleoside	.	.
ATP5PF	1248.951	-0.3886	0.143926234	-2.70002	0.0069334	0.040439	Not_Sig	no	gi|982909511|ref|XP_015322134.1|	321	2e-32	PREDICTED: ATP synthase-coupling factor 6, mitochondrial isoform X1 [Bos taurus]	gi|982975194|ref|XM_015461927.1|	543	0	PREDICTED: Bos taurus ATP synthase, H+ transporting, mitochondrial Fo complex subunit F6 (ATP5J), transcript variant X1, mRNA	.	.	.	.	.	.	.	.	pfam05109	Herpes_BLLF1	Herpes virus major outer envelope glycoprotein (BLLF1). This family consists of the BLLF1 viral late glycoprotein, also termed	.	.	.	.	.
LTBP2	14242.65	0.309704	0.114725627	2.699516	0.006944	0.040467	Not_Sig	no	gi|296482939|tpg|DAA25054.1|	9306	0	TPA: latent-transforming growth factor beta-binding protein 2 [Bos taurus]	gi|982937452|ref|XM_005211929.3|	7143	0	PREDICTED: Bos taurus latent transforming growth factor beta binding protein 2 (LTBP2), transcript variant X1, mRNA	Q28019	3415	0	Latent-transforming growth factor beta-binding protein 2	.	.	.	.	pfam00683	TB	TB domain. This domain is also known as the 8 cysteine domain. This family includes the hybrid domains. This cysteine rich repeat	.	GO:0071953|elastic fiber;GO:0001527|microfibril;	GO:0005509|calcium ion binding;GO:0008201|heparin	K08023	LTBP2_3_4; latent transforming growth factor beta binding protein 2/3/4
MARCHF5	653.7768	-0.4091	0.151548915	-2.69946	0.0069453	0.040467	Not_Sig	no	gi|8923415|ref|NP_060294.1|	1488	0	E3 ubiquitin-protein ligase MARCH5 [Homo sapiens]	gi|742134111|ref|XM_010842201.1|	3882	0	PREDICTED: Bison bison bison membrane-associated ring finger (C3HC4) 5 (MARCH5), mRNA	Q3KNM2	577	0	E3 ubiquitin-protein ligase MARCH5	.	.	.	.	pfam12906	RINGv	RING-variant domain.	GO:0090344|negative regulation of cell aging;GO:0090141|positive regulation of mitochondrial fission;GO:0051865|protein autoubiquitination;GO:0070585|protein localization to mitochondrion;GO:0000209|protein polyubiquitination;GO:0090140|regulation of mitochondrial fission;	GO:0005783|endoplasmic reticulum;GO:0005789|endoplasmic reticulum membrane;GO:0016021|integral component of	GO:0051020|GTPase binding;GO:0016874|ligase	K10660	MARCH5; E3 ubiquitin-protein ligase MARCH5 [EC:2.3.2.27]
TIMM9	236.6096	-0.45809	0.169714893	-2.69918	0.0069511	0.040473	Not_Sig	no	gi|148704607|gb|EDL36554.1|	476	1e-56	translocase of inner mitochondrial membrane 9 homolog (yeast), isoform CRA_a, partial [Mus musculus]	gi|942069777|ref|XM_005900859.2|	900	0	PREDICTED: Bos mutus translocase of inner mitochondrial membrane 9 homolog (yeast) (TIMM9), transcript variant X1, mRNA	Q9Y5J7	186	1e-57	Mitochondrial import inner membrane translocase subunit Tim9	.	.	.	.	pfam02953	zf-Tim10_DDP	Tim10/DDP family zinc finger. Putative zinc binding domain with four conserved cysteine residues. This domain is found in the human	GO:0072321|chaperone-mediated protein transport;GO:0045039|protein import into mitochondrial inner membrane;GO:0006626|protein targeting to mitochondrion;GO:0007605|sensory perception of sound;	GO:0005743|mitochondrial inner membrane;GO:0042721|mitochondrial inner membrane protein insertion complex;GO:0005758|mitochondrial	GO:0051087|chaperone binding;GO:0042803|protein	K17777	TIM9; mitochondrial import inner membrane translocase subunit
SGK3	200.7258	-0.4449	0.164833871	-2.69907	0.0069533	0.040473	Not_Sig	no	gi|440908352|gb|ELR58376.1|	2639	0	Serine/threonine-protein kinase Sgk3, partial [Bos mutus]	gi|965848495|ref|XM_012138897.2|	3447	0	PREDICTED: Ovis aries musimon serum/glucocorticoid regulated kinase family member 3 (SGK3), transcript variant X2, mRNA	Q5R7A7	1004	0	Serine/threonine-protein kinase Sgk3	.	.	.	.	pfam00069	Pkinase	Protein kinase domain.	.	GO:0016023|cytoplasmic membrane-bounded vesicle;GO:0005769|early endosome;GO:0055037|recycling endosome;	GO:0005524|ATP binding;GO:0035091|phosphatidylinositol	K13304	SGK3; serum/glucocorticoid-regulated kinase 3 [EC:2.7.11.1]
TNXB	62.4545	0.698293	0.258759047	2.698621	0.0069627	0.040507	UP	yes	gi|942071983|ref|XP_014335563.1|	13401	0	PREDICTED: LOW QUALITY PROTEIN: tenascin-X, partial [Bos mutus]	gi|31340694|ref|NM_174703.2|	9251	0	Bos taurus tenascin XB (TNXB), mRNA	P22105	1995	0	Tenascin-X	.	.	.	.	pfam00147	Fibrinogen_C	Fibrinogen beta and gamma chains, C-terminal globular domain.	GO:0030036|actin cytoskeleton organization;GO:0007155|cell adhesion;GO:0030199|collagen fibril organization;GO:0032963|collagen metabolic process;GO:0048251|elastic fiber assembly;	GO:0070062|extracellular exosome;GO:0005615|extracellular space;GO:0005622|intracellular;GO:0005578|proteinaceous extracellular matrix;	GO:0008201|heparin binding;GO:0005178|integrin binding;	K06252	TN; tenascin
MRPS21	233.6245	-0.52446	0.194402648	-2.69783	0.0069794	0.040561	Not_Sig	no	gi|826334825|ref|XP_012514295.1|	405	6e-47	PREDICTED: 28S ribosomal protein S21, mitochondrial isoform X3 [Propithecus coquereli]	gi|942052461|ref|XM_014477983.1|	550	0	PREDICTED: Bos mutus mitochondrial ribosomal protein S21 (MRPS21), transcript variant X1, mRNA	P82920	146	9e-44	28S ribosomal protein S21, mitochondrial	.	.	.	.	pfam01165	Ribosomal_S21	Ribosomal protein S21.	GO:0032543|mitochondrial translation;GO:0070125|mitochondrial translational elongation;GO:0070124|mitochondrial translational initiation;	GO:0005743|mitochondrial inner membrane;GO:0005763|mitochondrial small ribosomal subunit;	GO:0003735|structural constituent of ribosome;	K02970	RP-S21, MRPS21, rpsU; small subunit ribosomal protein S21
COLGALT1	2017.076	0.286641	0.106254114	2.697691	0.0069822	0.040561	Not_Sig	no	gi|149944687|ref|NP_001092425.1|	3142	0	procollagen galactosyltransferase 1 precursor [Bos taurus]	gi|149944686|ref|NM_001098955.1|	3134	0	Bos taurus collagen beta(1-O)galactosyltransferase 1 (COLGALT1), mRNA	A5PK45	1216	0	Procollagen galactosyltransferase 1	.	.	.	.	pfam01755	Glyco_transf_25	Glycosyltransferase family 25 (LPS biosynthesis protein). Members of this family belong to Glycosyltransferase family 25 This	.	GO:0005788|endoplasmic reticulum lumen;GO:0016020|membrane;	GO:0050211|procollagen galactosyltransferase activity;	K11703	GLT25D; collagen beta-1,O-galactosyltransferase [EC:2.4.1.50]
CCNG2	186.0158	-0.42727	0.158384651	-2.69768	0.0069825	0.040561	Not_Sig	no	gi|594072346|ref|XP_006060007.1|	1575	0	PREDICTED: cyclin-G2 isoform X1 [Bubalus bubalis]	gi|742110795|ref|XM_010838121.1|	2628	0	PREDICTED: Bison bison bison cyclin G2 (CCNG2), transcript variant X2, mRNA	Q16589	559	0	Cyclin-G2	ECU08g0260	59.7	1.19e-08	COG5024	pfam00134	Cyclin_N	Cyclin, N-terminal domain. Cyclins regulate cyclin dependent kinases (CDKs). Human cyclin-O is a Uracil-DNA glycosylase that is related to	GO:0000075|cell cycle checkpoint;GO:0051301|cell division;GO:0007067|mitotic nuclear division;GO:0051726|regulation of cell cycle;	GO:0005737|cytoplasm;	.	K10146	CCNG2; cyclin G2
TRIM28	2601.533	0.307407	0.113962746	2.697431	0.0069877	0.04057	Not_Sig	no	gi|742193062|ref|XP_010855930.1|	3708	0	PREDICTED: transcription intermediary factor 1-beta isoform X2 [Bison bison bison]	gi|742193061|ref|XM_010857628.1|	2678	0	PREDICTED: Bison bison bison tripartite motif containing 28 (TRIM28), transcript variant X2, mRNA	Q13263	1375	0	Transcription intermediary factor 1-beta	.	.	.	.	pfam00628	PHD	PHD-finger. PHD folds into an interleaved type of Zn-finger chelating 2 Zn ions in a similar manner to that of the RING and	GO:0016569|covalent chromatin modification;GO:0060028|convergent extension involved in axis elongation;GO:0043045|DNA methylation involved in embryo development;GO:0006281|DNA repair;GO:0007566|embryo implantation;GO:0060669|embryonic placenta morphogenesis;GO:0001837|epithelial to mesenchymal transition;GO:0045087|innate immune response;GO:1901536|negative regulation of DNA demethylation;GO:0045869|negative regulation of single stranded viral RNA replication via double stranded DNA	GO:0005719|nuclear euchromatin;GO:0005720|nuclear heterochromatin;GO:0005654|nucleoplasm;GO:0005634|nucleus;GO:0090575|RNA	GO:0003682|chromatin binding;GO:0070087|chromo shadow	K08882	TRIM28, TIF1B; tripartite motif-containing protein 28 [EC:2.3.2.27]
REEP5	2877.817	-0.25695	0.095304602	-2.69609	0.0070159	0.040689	Not_Sig	no	gi|594065891|ref|XP_006056929.1|	1004	3e-126	PREDICTED: receptor expression-enhancing protein 5 [Bubalus bubalis]	gi|114053300|ref|NM_001046605.1|	2839	0	Bos taurus receptor accessory protein 5 (REEP5), mRNA	Q29RM3	389	5e-127	Receptor expression-enhancing protein 5	SPCC830.08c	114	2.26e-28	COG5052	pfam03134	TB2_DP1_HVA22	TB2/DP1, HVA22 family. This family includes members from a wide variety of eukaryotes. It includes the TB2/DP1 (deleted in	.	GO:0071782|endoplasmic reticulum tubular network;GO:0016021|integral component of membrane;	.	K17279	REEP5_6; receptor expression-enhancing protein 5/6
PPP2CB	1000.858	-0.31111	0.115399412	-2.69598	0.0070183	0.040689	Not_Sig	no	gi|512987159|ref|XP_004855515.1|	1685	0	PREDICTED: serine/threonine-protein phosphatase 2A catalytic subunit beta isoform [Heterocephalus glaber]	gi|115495140|ref|NM_001075857.1|	1524	0	Bos taurus protein phosphatase 2, catalytic subunit, beta isozyme (PPP2CB), mRNA	Q0P594	650	0	Serine/threonine-protein phosphatase 2A catalytic subunit beta isoform	SPBC16H5.07c	531	0	COG0639	pfam00149	Metallophos	Calcineurin-like phosphoesterase. This family includes a diverse range of phosphoesterases, including protein phosphoserine	GO:0008637|apoptotic mitochondrial changes;GO:0043161|proteasome-mediated ubiquitin-dependent protein catabolic process;GO:0010468|regulation of gene expression;GO:0046677|response to antibiotic;GO:0034976|response to endoplasmic reticulum stress;GO:0042542|response to hydrogen peroxide;	GO:0000775|chromosome, centromeric region;GO:0005737|cytoplasm;GO:0070062|extracellular exosome;GO:0005634|nucleus;GO:0000159	GO:0046872|metal ion binding;GO:0004721|phosphoprotein	K04382	PPP2C; serine/threonine-protein phosphatase 2A catalytic subunit
VRK2	526.1477	-0.33647	0.12480593	-2.69592	0.0070194	0.040689	Not_Sig	no	gi|742214865|ref|XP_010861244.1|	2914	0	PREDICTED: serine/threonine-protein kinase VRK2 isoform X1 [Bison bison bison]	gi|742214864|ref|XM_010862942.1|	1913	0	PREDICTED: Bison bison bison vaccinia related kinase 2 (VRK2), transcript variant X1, mRNA	Q86Y07	833	0	Serine/threonine-protein kinase VRK2	.	.	.	.	pfam00069	Pkinase	Protein kinase domain.	GO:0034599|cellular response to oxidative stress;GO:0018105|peptidyl-serine phosphorylation;GO:0046777|protein autophosphorylation;GO:0006468|protein phosphorylation;GO:0008360|regulation of cell shape;GO:2000659|regulation of interleukin-1-mediated signaling pathway;GO:0043408|regulation of MAPK cascade;GO:0016032|viral process;	GO:0005737|cytoplasm;GO:0005783|endoplasmic reticulum;GO:0005789|endoplasmic reticulum membrane;GO:0016021|integral component of	GO:0005524|ATP binding;GO:0019904|protein domain specific	K08816	VRK; vaccinia related kinase [EC:2.7.11.1]
YTHDF1	575.4241	0.3576	0.132651869	2.695781	0.0070224	0.040689	Not_Sig	no	gi|528973973|ref|XP_005214870.1|	2363	0	PREDICTED: YTH domain-containing family protein 1 isoform X1 [Bos taurus]	gi|982943173|ref|XM_005214813.3|	2935	0	PREDICTED: Bos taurus YTH N(6)-methyladenosine RNA binding protein 1 (YTHDF1), transcript variant X1, mRNA	P59326	841	0	YTH domain-containing family protein 1	.	.	.	.	pfam04146	YTH	YT521-B-like domain. A protein of the YTH family has been shown to selectively remove transcripts of meiosis-specific genes expressed in	GO:0045948|positive regulation of translational initiation;	GO:0005737|cytoplasm;	GO:1990247|N6-methyladenosine-containing RNA binding;GO:0044822	.	.
SLC4A5	38.74914	-0.83581	0.31009064	-2.69537	0.0070311	0.040719	DOWN	yes	gi|742228285|ref|XP_010828175.1|	5866	0	PREDICTED: electrogenic sodium bicarbonate cotransporter 4 [Bison bison bison]	gi|742228284|ref|XM_010829873.1|	5601	0	PREDICTED: Bison bison bison solute carrier family 4 (sodium bicarbonate cotransporter), member 5 (SLC4A5), mRNA	Q6RI88	1773	0	Electrogenic sodium bicarbonate cotransporter 4	.	.	.	.	pfam00955	HCO3_cotransp	HCO3- transporter family. This family contains Band 3 anion exchange proteins that exchange CL-/HCO3-. This family also	GO:0006820|anion transport;GO:0015701|bicarbonate transport;GO:0033326|cerebrospinal fluid secretion;GO:0002064|epithelial cell development;GO:0048311|mitochondrion distribution;GO:0010468|regulation of gene expression;GO:0051453|regulation of intracellular pH;GO:0006885|regulation of pH;GO:0003073|regulation of systemic arterial blood pressure;GO:0003014|renal system process;GO:0060041|retina development in camera-type eye;GO:0006814|sodium ion transport;	GO:0016323|basolateral plasma membrane;GO:0016021|integral component of membrane;GO:0005887|integral	GO:0015301|anion:anion antiporter activity;GO:0005452|inorganic anion	K13857	SLC4A5, NBC4; solute carrier family 4 (sodium bicarbonate
FLT1	426.4118	0.34831	0.129277405	2.694283	0.007054	0.040832	Not_Sig	no	gi|555980618|ref|XP_005902233.1|	6854	0	PREDICTED: vascular endothelial growth factor receptor 1 [Bos mutus]	gi|555980617|ref|XM_005902171.1|	6535	0	PREDICTED: Bos mutus fms-related tyrosine kinase 1 (FLT1), mRNA	P17948	2265	0	Vascular endothelial growth factor receptor 1	.	.	.	.	pfam07714	Pkinase_Tyr	Protein tyrosine kinase.	GO:0001525|angiogenesis;GO:0048514|blood vessel morphogenesis;GO:0030154|cell differentiation;GO:0016477|cell migration;GO:0035924|cellular response to vascular endothelial growth factor stimulus;GO:0048598|embryonic morphogenesis;GO:0002548|monocyte chemotaxis;GO:0018108|peptidyl-tyrosine phosphorylation;GO:0045766|positive regulation of angiogenesis;GO:0030335|positive regulation of cell migration;GO:0008284|positive regulation of cell proliferation;GO:0043406|positive	GO:0005768|endosome;GO:0005615|extracellular space;GO:0005925|focal adhesion;GO:0005887|integral component of plasma membrane;GO:0005886|plasma	GO:0005524|ATP binding;GO:0019838|growth factor binding;GO:0036332	K05096	FLT1, VEGFR1; FMS-like tyrosine kinase 1 [EC:2.7.10.1]
ISG15	161.9243	-0.55253	0.205188985	-2.69279	0.0070856	0.040994	Not_Sig	no	gi|555953513|ref|XP_005888931.1|	792	3e-104	PREDICTED: ubiquitin-like protein ISG15 [Bos mutus]	gi|742164865|ref|XM_010850022.1|	615	0	PREDICTED: Bison bison bison ISG15 ubiquitin-like modifier (ISG15), mRNA	O02741	304	5e-104	Ubiquitin-like protein ISG15	.	.	.	.	pfam00240	ubiquitin	Ubiquitin family. This family contains a number of ubiquitin-like proteins: SUMO (smt3 homologue) Nedd8, Elongin B, Rub1, and	GO:0042742|defense response to bacterium;GO:0051607|defense response to virus;GO:0032020|ISG15-protein conjugation;GO:0019941|modification-dependent protein catabolic process;GO:0031397|negative regulation of protein ubiquitination;GO:0045071|negative regulation of viral genome replication;GO:0045648|positive regulation of erythrocyte differentiation;GO:0032649|regulation of interferon-gamma production;GO:0034340|response to type I interferon;	GO:0005737|cytoplasm;GO:0005576|extracellular region;	GO:0031386|protein tag;	K12159	ISG15; ubiquitin cross-reactive protein
BAP1	1146.777	0.303889	0.112881077	2.69212	0.0070999	0.041056	Not_Sig	no	gi|555972308|ref|XP_005898160.1|	3305	0	PREDICTED: ubiquitin carboxyl-terminal hydrolase BAP1 isoform X1 [Bos mutus]	gi|942060537|ref|XM_005898098.2|	3487	0	PREDICTED: Bos mutus BRCA1 associated protein-1 (ubiquitin carboxy-terminal hydrolase) (BAP1), transcript variant X1, mRNA	Q92560	1232	0	Ubiquitin carboxyl-terminal hydrolase BAP1	.	.	.	.	pfam01088	Peptidase_C12	Ubiquitin carboxyl-terminal hydrolase, family 1.	GO:0006464|cellular protein modification process;GO:0035522|monoubiquitinated histone H2A deubiquitination;GO:0035520|monoubiquitinated protein deubiquitination;GO:0008285|negative regulation of cell proliferation;GO:1903955|positive regulation of protein targeting to mitochondrion;GO:0016579|protein deubiquitination;GO:0071108|protein K48-linked deubiquitination;GO:0051726|regulation of cell cycle;GO:0001558|regulation of cell	GO:0005737|cytoplasm;GO:0005654|nucleoplasm;GO:0005634|nucleus;GO:0035517|PR-DUB complex;	GO:0003682|chromatin binding;GO:0008233|peptidase	K08588	BAP1, UCHL2; ubiquitin carboxyl-terminal hydrolase BAP1 [EC:3.4.19.12]
DVL1	705.174	0.34522	0.128249836	2.691776	0.0071073	0.041078	Not_Sig	no	gi|594087026|ref|XP_006067003.1|	2306	0	PREDICTED: segment polarity protein dishevelled homolog DVL-1 isoform X9 [Bubalus bubalis]	gi|926711342|ref|XM_013970217.1|	3071	0	PREDICTED: Capra hircus dishevelled segment polarity protein 1 (DVL1), mRNA	P51141	788	0	Segment polarity protein dishevelled homolog DVL-1	.	.	.	.	pfam12316	Dsh_C	Segment polarity protein dishevelled (Dsh) C terminal. This domain family is found in eukaryotes, and is typically	GO:0048675|axon extension;GO:0007411|axon guidance;GO:0007409|axonogenesis;GO:0060070|canonical Wnt signaling pathway;GO:0090103|cochlea morphogenesis;GO:0048668|collateral sprouting;GO:0022007|convergent extension involved in neural plate elongation;GO:0060029|convergent extension involved in organogenesis;GO:0031122|cytoplasmic microtubule organization;GO:0048813|dendrite morphogenesis;GO:0060997|dendritic spine morphogenesis;GO:0035556|intracellular signal	GO:0030424|axon;GO:0030136|clathrin-coated vesicle;GO:0031410|cytoplasmic vesicle;GO:0016023|cytoplasmic membrane-bounded	GO:0008013|beta-catenin binding;GO:0019899|enzyme	K02353	DVL; segment polarity protein dishevelled

AMOTL2	1539.082	0.26625	0.098922431	2.691498	0.0071132	0.041091	Not_Sig	no	gi|555992256|ref|XP_005907904.1|	2750	0	PREDICTED: angiomotin-like protein 2 isoform X2 [Bos mutus]	gi|942091646|ref|XM_005907842.2|	4176	0	PREDICTED: Bos mutus angiomotin like 2 (AMOTL2), transcript variant X2, mRNA	F1MRK3	1057	0	Angiomotin-like protein 2	.	.	.	.	pfam12240	Angiomotin_C	Angiomotin C terminal. This domain family is found in eukaryotes, and is typically between 197 and 211 amino acids	GO:0016055|Wnt signaling pathway;	GO:0055037|recycling endosome;	.	.	.
MAFB	193.8808	0.447234	0.166227724	2.690489	0.0071347	0.041195	Not_Sig	no	gi|829998796|ref|XP_012610703.1|	1189	3e-150	PREDICTED: transcription factor MafB [Microcebus murinus]	gi|982993782|ref|XM_610891.8|	3223	0	PREDICTED: Bos taurus v-maf avian musculoaponeurotic fibrosarcoma oncogene homolog B (MAFB), mRNA	P54842	455	6e-149	Transcription factor MafB	.	.	.	.	pfam03131	bZIP_Maf	bZIP Maf transcription factor. Maf transcription factors contain a conserved basic region leucine zipper (bZIP) domain, which	GO:0035284|brain segmentation;GO:0042472|inner ear morphogenesis;GO:0045647|negative regulation of erythrocyte differentiation;GO:0045671|negative regulation of osteoclast differentiation;GO:0007399|nervous system development;GO:0006357|regulation of transcription from RNA polymerase II promoter;GO:0007585|respiratory gaseous exchange;GO:0021571|rhombomere 5 development;GO:0021572|rhombomere 6 development;GO:0007379|segment	GO:0005634|nucleus;GO:0005667|transcription factor complex;	GO:0000978|RNA polymerase II core promoter proximal region sequence-	K09036	MAFB; transcription factor MAFB
ITGB3BP	130.4825	-0.51852	0.192743995	-2.69019	0.0071411	0.041211	Not_Sig	no	gi|742092680|ref|XP_010832199.1|	981	5e-120	PREDICTED: centromere protein R isoform X1 [Bison bison bison]	gi|850474981|gb|CP011886.1|	2828	0	Ovis canadensis canadensis isolate 43U chromosome 1 sequence	Q13352	214	2e-61	Centromere protein R	.	.	.	.	pfam06729	CENP-R	Kinetochore component, CENP-R. This family consists of mammalian kinetochore sub-complex proteins CENP-R, also referred to as nuclear	GO:0006915|apoptotic process;GO:0007155|cell adhesion;GO:0051301|cell division;GO:0034080|CENP-A containing nucleosome assembly;GO:0007067|mitotic nuclear division;GO:0043065|positive regulation of apoptotic process;GO:0006355|regulation of transcription, DNA-templated;GO:0007165|signal transduction;GO:0007062|sister chromatid cohesion;GO:0006351|transcription, DNA-templated;	GO:0000777|condensed chromosome kinetochore;GO:0005737|cytoplasm;GO:0005829|cytosol;GO:0016020|membrane;GO:0005654|nucleoplasm;GO:0005634|nucleus	GO:0008022|protein C-terminus binding;GO:0004871|signal transducer	K11510	ITGB3BP, CENPR; centromere protein R
MTERF1	146.6027	-0.47566	0.176826396	-2.69	0.0071452	0.041214	Not_Sig	no	gi|440894333|gb|ELR46810.1|	2115	0	Transcription termination factor, mitochondrial, partial [Bos mutus]	gi|942094590|ref|XM_014482590.1|	2806	0	PREDICTED: Bos mutus mitochondrial transcription termination factor 1 (MTERF1), transcript variant X2, mRNA	Q99551	590	0	Transcription termination factor 1, mitochondrial	.	.	.	.	pfam02536	mTERF	mTERF. This family contains one sequence of known function Human mitochondrial transcription termination factor	GO:0032392|DNA geometric change;GO:0006353|DNA-templated transcription, termination;GO:0007005|mitochondrion organization;GO:0006355|regulation of transcription, DNA-templated;GO:0006393|termination of mitochondrial transcription;	GO:0005759|mitochondrial matrix;GO:0042645|mitochondrial nucleoid;GO:0005739|mitochondrion;	GO:0003690|double-stranded DNA binding;GO:0044822|poly(A) RNA	K15031	MTERF; transcription termination factor, mitochondrial
TMEM168	526.042	-0.32503	0.120849859	-2.68954	0.0071551	0.04125	Not_Sig	no	gi|118151154|ref|NP_001071499.1|	3343	0	transmembrane protein 168 [Bos taurus]	gi|742233619|ref|XM_010831186.1|	3837	0	PREDICTED: Bison bison bison transmembrane protein 168 (TMEM168), transcript variant X3, mRNA	A0JNG0	1292	0	Transmembrane protein 168	.	.	.	.	pfam09586	YfhO	Bacterial membrane protein YfhO. This protein is a conserved membrane protein. The yfhO gene is transcribed in Difco sporulation	.	GO:0016021|integral component of membrane;GO:0030133|transport vesicle;	.	.	.
ELL2	1186.623	-0.28149	0.104676713	-2.68912	0.0071641	0.041282	Not_Sig	no	gi|329755297|ref|NP_001178075.1|	2932	0	RNA polymerase II elongation factor ELL2 [Bos taurus]	gi|742237677|ref|XM_010832000.1|	5519	0	PREDICTED: Bison bison bison elongation factor, RNA polymerase II, 2 (ELL2), mRNA	O00472	1063	0	RNA polymerase II elongation factor ELL2	.	.	.	.	pfam10390	ELL	RNA polymerase II elongation factor ELL. ELL is a family of RNA polymerase II elongation factors. It is bound stably to elongation-	GO:0006355|regulation of transcription, DNA-templated;GO:0042795|snRNA transcription from RNA polymerase II promoter;GO:0006368|transcription elongation from RNA polymerase II promoter;	GO:0005813|centrosome;GO:0005737|cytoplasm;GO:0005654|nucleoplasm;GO:0008023|transcription elongation factor complex;	.	K15183	ELL; RNA polymerase II elongation factor ELL
ZNF532	797.076	0.348346	0.129556265	2.688764	0.0071717	0.041305	Not_Sig	no	gi|440892054|gb|ELR45423.1|	5963	0	Zinc finger protein 532, partial [Bos mutus]	gi|942099620|ref|XM_005910575.2|	5444	0	PREDICTED: Bos mutus zinc finger protein 532 (ZNF532), mRNA	Q9HCE3	2117	0	Zinc finger protein 532	.	.	.	.	pfam02591	DUF164	Putative zinc ribbon domain. Structural modelling suggests this domain may bind nucleic acids.	GO:0006351|transcription, DNA-templated;	GO:0005634|nucleus;	GO:0046872|metal ion binding;GO:0043565|sequence-specific	.	.
TP53INP2	1088.4	0.310003	0.115315334	2.688309	0.0071815	0.04134	Not_Sig	no	gi|594047441|ref|XP_006048138.1|	706	2e-81	PREDICTED: tumor protein p53-inducible nuclear protein 2 isoform X1 [Bubalus bubalis]	gi|982993642|ref|XM_010824093.2|	4123	0	PREDICTED: Bos taurus tumor protein p53 inducible nuclear protein 2 (TP53INP2), transcript variant X2, mRNA	Q8CFU8	231	5e-67	Tumor protein p53-inducible nuclear protein 2	.	.	.	.	pfam14839	DOR	DOR family. This family of proteins regulate autophagy and gene transcription.	GO:0000045|autophagosome assembly;GO:0016236|macroautophagy;GO:1903828|negative regulation of cellular protein localization;GO:0001649|osteoblast differentiation;GO:0010508|positive regulation of autophagy;GO:0045893|positive regulation of transcription, DNA-templated;GO:0001894|tissue homeostasis;GO:0006351|transcription, DNA-templated;GO:0006511|ubiquitin-dependent protein catabolic process;	GO:0005776|autophagosome;GO:0031410|cytoplasmic vesicle;GO:0005829|cytosol;GO:0005634|nucleus;GO:0016605|PML body;	GO:0043130|ubiquitin binding;	.	.
ARMCX3	1401.335	-0.3204	0.119219089	-2.68751	0.0071986	0.041418	Not_Sig	no	gi|300794378|ref|NP_001179382.1|	1711	0	armadillo repeat-containing X-linked protein 3 [Bos taurus]	gi|982973271|ref|XM_005227788.3|	6630	0	PREDICTED: Bos taurus armadillo repeat containing, X-linked 3 (ARMCX3), transcript variant X1, mRNA	Q5R9J3	624	0	Armadillo repeat-containing X-linked protein 3	.	.	.	.	pfam04826	Arm_2	Armadillo-like. This domain contains armadillo-like repeats. Proteins containing this domain interact with numerous other	GO:0034613|cellular protein localization;GO:0045944|positive regulation of transcription from RNA polymerase II promoter;	GO:0031307|integral component of mitochondrial outer membrane;	.	.	.
SLC25A6	9000.845	0.289459	0.107718531	2.687182	0.0072058	0.041439	Not_Sig	no	gi|32189336|ref|NP_777085.1|	1480	0	ADP/ATP translocase 3 [Bos taurus]	gi|151557096|gb|BC150034.1|	1368	0	Bos taurus solute carrier family 25 (mitochondrial carrier; adenine nucleotide translocator), member 6, mRNA (cDNA clone MGC:166054 IMAGE:8634856), complete cds	P32007	574	0	ADP/ATP translocase 3	.	.	.	.	pfam00153	Mito_carr	Mitochondrial carrier protein.	GO:0006915|apoptotic process;GO:0006412|translation;GO:0055085|transmembrane transport;	GO:0016021|integral component of membrane;GO:0005743|mitochondrial inner membrane;GO:0005634|nucleus;	GO:0003735|structural constituent of ribosome;GO:0005215|transporter	K05863	SLC25A4S, ANT; solute carrier family 25 (mitochondrial adenine nucleotide
CLCC1	880.4257	-0.32162	0.119705081	-2.68675	0.007215	0.041467	Not_Sig	no	gi|555958548|ref|XP_005891392.1|	2625	0	PREDICTED: chloride channel CLIC-like protein 1 isoform X1 [Bos mutus]	gi|982916975|ref|XM_010803167.2|	3768	0	PREDICTED: Bos taurus chloride channel CLIC like 1 (CLCC1), transcript variant X1, mRNA	Q1LZF8	1012	0	Chloride channel CLIC-like protein 1	.	.	.	.	pfam05934	MCLC	Mid-1-related chloride channel (MCLC). This family consists of several mid-1-related chloride channels. mid-1-related chloride	.	GO:0034707|chloride channel complex;GO:0005783|endoplasmic reticulum;GO:0005794|Golgi apparatus;GO:0005634|nucleus;	GO:0005254|chloride channel activity;	.	.
LOC102265605	188.3066	-0.49551	0.184443143	-2.68651	0.0072202	0.041467	Not_Sig	no	gi|743742979|ref|XP_010966045.1|	802	2e-102	PREDICTED: calmodulin [Camelus bactrianus]	gi|942055428|ref|XM_005896088.2|	1107	0	PREDICTED: Bos mutus calmodulin-alpha (LOC102265605), mRNA	P62155	282	4e-93	Calmodulin	SPAC3A12.14	221	4.65e-71	COG5126	pfam13499	EF-hand_7	EF-hand domain pair.	.	.	GO:0005509|calcium ion binding;GO:0005102|receptor binding;	K02183	CALM; calmodulin
PRCC	707.822	0.326277	0.121452702	2.686452	0.0072215	0.041467	Not_Sig	no	gi|440903656|gb|ELR54293.1|	1399	0	Proline-rich protein PRCC [Bos mutus]	gi|942063163|ref|XM_005898748.2|	2094	0	PREDICTED: Bos mutus papillary renal cell carcinoma (translocation-associated) (PRCC), mRNA	Q92733	532	0	Proline-rich protein PRCC	.	.	.	.	pfam10253	PRCC	Mitotic checkpoint regulator, MAD2B-interacting. This family constitutes the major, conserved, portion of PRCC proteins. In	GO:0007093|mitotic cell cycle checkpoint;	GO:0005634|nucleus;GO:0005681|spliceosomal complex;	.	K13105	PRCC; proline-rich protein PRCC
TMEM88	158.6823	-0.49701	0.185050135	-2.68584	0.0072348	0.041522	Not_Sig	no	gi|767992608|ref|XP_011522339.1|	276	9e-39	PREDICTED: N(alpha)-acetyltransferase 38, NatC auxiliary subunit isoform X3 [Homo sapiens]	gi|942052095|ref|XM_005894808.2|	893	0	PREDICTED: Bos mutus transmembrane protein 88 (TMEM88), mRNA	A5D7M7	130	5e-33	Transmembrane protein 88	.	.	.	.	pfam01423	LSM	LSM domain. The LSM domain contains Sm proteins as well as other related LSM (Like Sm) proteins. The U1, U2, U4/U6, and	GO:0007275|multicellular organismal development;GO:0090090|negative regulation of canonical Wnt signaling pathway;GO:0072659|protein localization to plasma membrane;GO:0050821|protein stabilization;GO:0016055|Wnt signaling pathway;	GO:0005829|cytosol;GO:0016021|integral component of membrane;GO:0005886|plasma membrane;	.	.	.
MTMR14	343.2097	0.366199	0.1364	2.684745	0.0072585	0.041638	Not_Sig	no	gi|742133540|ref|XP_010840376.1|	2592	0	PREDICTED: myotubularin-related protein 14 isoform X4 [Bison bison bison]	gi|742133539|ref|XM_010842074.1|	4020	0	PREDICTED: Bison bison bison myotubularin related protein 14 (MTMR14), transcript variant X4, mRNA	Q8NCE2	941	0	Myotubularin-related protein 14	.	.	.	.	pfam06602	Myotub-related	Myotubularin-like phosphatase domain. This family represents the phosphatase domain within eukaryotic myotubularin-related	GO:0016236|macroautophagy;GO:0006661|phosphatidylinositol biosynthetic process;	GO:0005829|cytosol;GO:0048471|perinuclear region of cytoplasm;GO:0001726|ruffle;	GO:0052629|phosphatidylinositol-3,5-bisphosphate 3-phosphatase	K18086	MTMR14; myotubularin-related protein 14 [EC:3.1.3.64
LIMS2	1841.477	0.412641	0.153718412	2.684397	0.0072661	0.04166	Not_Sig	no	gi|528938581|ref|XP_005202254.1|	1815	0	PREDICTED: LIM and senescent cell antigen-like-containing domain protein 2 isoform X1 [Bos taurus]	gi|982912834|ref|XM_005202197.3|	1766	0	PREDICTED: Bos taurus LIM-type zinc finger domains 2 (LIMS2), transcript variant X1, mRNA	Q2KJ33	645	0	LIM and senescent cell antigen-like-containing domain protein 2	.	.	.	.	pfam00412	LIM	LIM domain. This family represents two copies of the LIM structural domain.	GO:0045216|cell-cell junction organization;GO:0043066|negative regulation of apoptotic process;GO:2000346|negative regulation of hepatocyte proliferation;GO:2000178|negative regulation of neural precursor cell proliferation;GO:2001046|positive regulation of integrin-mediated signaling pathway;GO:0016337|single organismal cell-cell adhesion;	GO:0005925|focal adhesion;GO:0005886|plasma membrane;	GO:0008270|zinc ion binding;	.	.
SEMA4G	167.3868	0.508763	0.189584708	2.683565	0.0072842	0.041743	Not_Sig	no	gi|741975979|ref|XP_010818201.1|	3930	0	PREDICTED: semaphorin-4G isoform X1 [Bos taurus]	gi|982968123|ref|XM_010819899.2|	4318	0	PREDICTED: Bos taurus semaphorin 4G (SEMA4G), transcript variant X1, mRNA	Q9NTN9	1383	0	Semaphorin-4G	.	.	.	.	pfam01403	Sema	Sema domain. The Sema domain occurs in semaphorins, which are a large family of secreted and transmembrane proteins, some of	GO:0050919|negative chemotaxis;GO:0048843|negative regulation of axon extension involved in axon guidance;GO:0001755|neural crest cell migration;GO:0030335|positive regulation of cell migration;GO:0071526|semaphorin-plexin signaling pathway;	GO:0005615|extracellular space;GO:0016021|integral component of membrane;GO:0005886|plasma membrane;	GO:0045499|chemorepellent activity;GO:0030215|semaphorin	K06521	SEMA4; semaphorin 4
PLSCR3	615.5943	0.358632	0.133669504	2.682972	0.0072971	0.041788	Not_Sig	no	gi|555955699|ref|XP_005890005.1|	1275	2e-163	PREDICTED: phospholipid scramblase 3 isoform X1 [Bos mutus]	gi|742155389|ref|XM_010847326.1|	2880	0	PREDICTED: Bison bison bison phospholipid scramblase 3 (PLSCR3), transcript variant X2, mRNA	Q9NRY6	443	4e-146	Phospholipid scramblase 3	.	.	.	.	pfam03803	Scramblase	Scramblase. Scramblase is palmitoylated and contains a potential protein kinase C phosphorylation site. Scramblase	GO:0006915|apoptotic process;GO:0071222|cellular response to lipopolysaccharide;GO:0042632|cholesterol homeostasis;GO:0042593|glucose homeostasis;GO:0017121|phospholipid scrambling;	GO:0016021|integral component of membrane;GO:0031966|mitochondrial membrane;GO:0005886|plasma membrane;	GO:0005509|calcium ion binding;GO:0048306|calcium-dependent	.	.
RPL22	1414.107	-0.86055	0.320757066	-2.68288	0.0072992	0.041788	DOWN	yes	gi|635112121|ref|XP_007978994.1|	426	5e-50	PREDICTED: 60S ribosomal protein L22 isoform X2 [Chlorocebus sabaeus]	gi|742164624|ref|XM_010849955.1|	479	0	PREDICTED: Bison bison bison ribosomal protein L22 (RPL22), mRNA	P67985	166	2e-51	60S ribosomal protein L22	.	.	.	.	pfam01776	Ribosomal_L22e	Ribosomal L22e protein family.	GO:0002181|cytoplasmic translation;	GO:0022625|cytosolic large ribosomal subunit;	GO:0008201|heparin binding;GO:0003723|RNA	K02891	RP-L22e, RPL22; large subunit ribosomal protein L22e
FAM114A1	1488.325	-0.26	0.096924578	-2.68248	0.0073078	0.041816	Not_Sig	no	gi|555970769|ref|XP_005897403.1|	2541	0	PREDICTED: protein NOXP20 [Bos mutus]	gi|942058745|ref|XM_005897341.2|	4299	0	PREDICTED: Bos mutus family with sequence similarity 114, member A1 (FAM114A1), mRNA	Q8IWE2	778	0	Protein NOXP20	.	.	.	.	pfam05334	DUF719	Protein of unknown function (DUF719). This family consists of several eukaryotic proteins of unknown function.	.	GO:0005737|cytoplasm;	.	.	.
PSMA3	1064.42	-0.40388	0.150584793	-2.68211	0.007316	0.041842	Not_Sig	no	gi|655865215|ref|XP_002718317.2|	1312	6e-179	PREDICTED: proteasome subunit alpha type-3 [Oryctolagus cuniculus]	gi|942069768|ref|XM_005900863.2|	964	0	PREDICTED: Bos mutus proteasome subunit alpha 3 (PSMA3), transcript variant X1, mRNA	Q58DU5	493	5e-175	Proteasome subunit alpha type-3	.	.	.	.	pfam00227	Proteasome	Proteasome subunit. The proteasome is a multisubunit structure that degrades proteins. Protein degradation is an essential	GO:0052548|regulation of endopeptidase activity;GO:0006511|ubiquitin-dependent protein catabolic process;	GO:0005737|cytoplasm;GO:0070062|extracellular exosome;GO:0005634|nucleus;GO:0005839|proteasome core	GO:0004298|threonine-type endopeptidase activity;	K02727	PSMA3; 20S proteasome subunit alpha 7 [EC:3.4.25.1]
BLOC1S1	296.1107	-0.4154	0.154963587	-2.68061	0.0073488	0.042009	Not_Sig	no	gi|594662823|ref|XP_007179596.1|	602	1e-75	PREDICTED: biogenesis of lysosome-related organelles complex 1 subunit 1 [Balaenoptera acutorostrata scammoni]	gi|742115082|ref|XM_010839176.1|	575	0	PREDICTED: Bison bison bison biogenesis of lysosomal organelles complex-1, subunit 1 (BLOC1S1), mRNA	Q5R7L8	225	2e-73	Biogenesis of lysosome-related organelles complex 1 subunit 1	.	.	.	.	pfam06320	GCN5L1	GCN5-like protein 1 (GCN5L1). This family consists of several eukaryotic GCN5-like protein 1 (GCN5L1) sequences. The function	GO:0009060|aerobic respiration;GO:0008089|anterograde axon cargo transport;GO:0048490|anterograde synaptic vesicle transport;GO:0032418|lysosome localization;GO:0098779|mitophagy in response to mitochondrial depolarization;GO:0031175|neuron projection development;GO:0018394|peptidyl-lysine acetylation;	GO:1904115|axon cytoplasm;GO:0031083|BLOC-1 complex;GO:0005829|cytosol;GO:0005769|early endosome;GO:0070062|extracellular	.	.	.
RIDA	326.0485	-0.43557	0.162559564	-2.67942	0.0073749	0.042122	Not_Sig	no	gi|555985123|ref|XP_005904442.1|	703	3e-89	PREDICTED: ribonuclease UK114 [Bos mutus]	gi|742217257|ref|XM_010863539.1|	1068	0	PREDICTED: Bison bison bison heat-responsive protein 12 (HRSP12), mRNA	Q3T114	275	1e-90	Ribonuclease UK114	lin0837	130	1.63e-36	COG0251	pfam01042	Ribonuc_L-PSP	Endoribonuclease L-PSP. Endoribonuclease active on single-stranded mRNA. Inhibits protein synthesis by cleavage of mRNA.	.	GO:0070062|extracellular exosome;GO:0005739|mitochondrion;	GO:0004519|endonuclease activity;GO:0044822|poly(A) RNA	.	.
TCIRG1	655.3666	0.357701	0.133501785	2.679374	0.007376	0.042122	Not_Sig	no	gi|115497292|ref|NP_001069102.1|	3446	0	V-type proton ATPase 116 kDa subunit a isoform 3 [Bos taurus]	gi|742109003|ref|XM_010837670.1|	2760	0	PREDICTED: Bison bison bison T-cell, immune regulator 1, ATPase, H+ transporting, lysosomal V0 subunit A3 (TCIRG1), transcript variant X2, mRNA	Q13488	1165	0	V-type proton ATPase 116 kDa subunit a isoform 3	YOR270c	383	1.84e-118	COG1269	pfam01496	V_ATPase_I	V-type ATPase 116kDa subunit family. This family consists of the 116kDa V-type ATPase (vacuolar (H+)-ATPases) subunits, as well as	GO:0015991|ATP hydrolysis coupled proton transport;GO:0015986|ATP synthesis coupled proton transport;GO:0006968|cellular defense response;GO:0008286|insulin receptor signaling pathway;GO:0034220|ion transmembrane transport;GO:0016236|macroautophagy;GO:0090383|phagosome acidification;GO:0008284|positive regulation of cell proliferation;GO:0007039|protein catabolic process in the vacuole;GO:0015992|proton transport;GO:0033572|transferrin	GO:0016324|apical plasma membrane;GO:0010008|endosome membrane;GO:0005887|integral component of plasma	GO:0051117|ATPase binding;GO:0046961|proton-	K02154	ATPeV0A, ATP6N; V-type H+-transporting ATPase subunit a
FAM98B	477.2413	-0.37723	0.140825362	-2.67872	0.0073903	0.042182	Not_Sig	no	gi|359069460|ref|XP_002690863.2|	1526	0	PREDICTED: protein FAM98B [Bos taurus]	gi|982989150|ref|XM_015465035.1|	1624	0	PREDICTED: Bos taurus family with sequence similarity 98 member B (FAM98B), transcript variant X2, mRNA	Q80VD1	542	0	Protein FAM98B	.	.	.	.	pfam10239	DUF2465	Protein of unknown function (DUF2465). FAM98A and B proteins are found from worms to humans but their function is	.	GO:0005737|cytoplasm;GO:0005634|nucleus;GO:0072669|tRNA-splicing ligase complex;	GO:0044822|poly(A) RNA binding;	K15434	FAM98B; protein FAM98B
RABL2B	342.1106	0.357558	0.133488428	2.67857	0.0073937	0.042182	Not_Sig	no	gi|77735963|ref|NP_001029680.1|	1115	2e-150	rab-like protein 2B [Bos taurus]	gi|942077487|ref|XM_005903411.2|	1028	0	PREDICTED: Bos mutus RAB, member of RAS oncogene family-like 2B (RABL2B), transcript variant X1, mRNA	Q9UBK7	390	1e-134	Rab-like protein 2A	.	.	.	.	pfam00071	Ras	Ras family. Includes sub-families Ras, Rab, Rac, Ral, Ran, Rap Ypt1 and more. Shares P-loop motif with GTP_EFTU, arf and	GO:0007264|small GTPase mediated signal transduction;	GO:0005622|intracellular;	GO:0005525|GTP binding;GO:0003924|GTPase activity;	K07931	RABL2; Rab-like protein 2
GEM	1278.177	-0.30201	0.112764817	-2.67819	0.007402	0.042208	Not_Sig	no	gi|555967089|ref|XP_005895593.1|	1587	0	PREDICTED: GTP-binding protein GEM [Bos mutus]	gi|555967088|ref|XM_005895531.1|	2365	0	PREDICTED: Bos mutus GTP binding protein overexpressed in skeletal muscle (GEM), mRNA	P55040	553	0	GTP-binding protein GEM	.	.	.	.	pfam00071	Ras	Ras family. Includes sub-families Ras, Rab, Rac, Ral, Ran, Rap Ypt1 and more. Shares P-loop motif with GTP_EFTU, arf and	GO:0007166|cell surface receptor signaling pathway;GO:0051276|chromosome organization;GO:0006955|immune response;GO:0051310|metaphase plate congression;GO:0007067|mitotic nuclear division;GO:0007165|signal transduction;GO:0007264|small GTPase mediated signal transduction;	GO:0009898|cytoplasmic side of plasma membrane;GO:0030496|midbody;GO:0072686|mitotic spindle;GO:0005634|nucleus;GO:0051233|s	GO:0019003|GDP binding;GO:0005525|GTP binding;GO:0003924	K07846	GEM; GTP binding protein overexpressed in skeletal muscle
ADORA2B	150.036	0.474835	0.177327194	2.677734	0.0074122	0.042245	Not_Sig	no	gi|594113621|ref|XP_006079556.1|	1796	0	PREDICTED: adenosine receptor A2b isoform X1 [Bubalus bubalis]	gi|742136642|ref|XM_010842943.1|	1753	0	PREDICTED: Bison bison bison adenosine A2b receptor (ADORA2B), mRNA	Q1LZD0	641	0	Adenosine receptor A2b	.	.	.	.	pfam00001	7tm_1	7 transmembrane receptor (rhodopsin family). This family contains, amongst other G-protein-coupled receptors	GO:0007189|adenylate cyclase-activating G-protein coupled receptor signaling pathway;GO:0031668|cellular response to extracellular stimulus;GO:0030819|positive regulation of cAMP biosynthetic process;GO:0032722|positive regulation of chemokine production;GO:0002882|positive regulation of chronic inflammatory response to non-antigenic stimulus;GO:0031284|positive regulation of guanylate cyclase activity;GO:0032755|positive regulation of interleukin-6 production;GO:0043306|positive regulation of mast cell	GO:0016021|integral component of membrane;GO:0005886|plasma membrane;	GO:0001609|G-protein coupled adenosine receptor activity;	K04267	ADORA2B; adenosine receptor A2b
RABGGTB	691.0356	-0.39041	0.145821299	-2.6773	0.0074218	0.042271	Not_Sig	no	gi|926688391|ref|XP_005678294.2|	1785	0	PREDICTED: geranylgeranyl transferase type-2 subunit beta isoform X1 [Capra hircus]	gi|151556391|gb|BC147953.1|	1447	0	Bos taurus Rab geranylgeranyltransferase, beta subunit, mRNA (cDNA clone MGC:166017 IMAGE:8250966), complete cds	Q5E9B3	686	0	Geranylgeranyl transferase type-2 subunit beta	YPR176c	344	2.21e-115	COG5029	pfam13249	Prenyltrans_2	Prenyltransferase-like.	GO:0018344|protein geranylgeranylation;	GO:0005968|Rab-protein geranylgeranyltransferase complex;	GO:0004663|Rab geranylgeranyltransferase activity;GO:0017137	K05956	RABGGTB; geranylgeranyl transferase type-2 subunit beta
CPSF1	933.1462	0.307034	0.114690264	2.677067	0.007427	0.042271	Not_Sig	no	gi|27807297|ref|NP_777145.1|	7323	0	cleavage and polyadenylation specificity factor subunit 1 [Bos taurus]	gi|402743951|ref|NM_174720.3|	4379	0	Bos taurus cleavage and polyadenylation specific factor 1, 160kDa (CPSF1), mRNA	Q10569	2825	0	Cleavage and polyadenylation specificity factor subunit 1	SPBC1709.08	454	8.64e-135	COG5161	pfam03178	CPSF_A	CPSF A subunit region. This family includes a region that lies towards the C-terminus of the cleavage and polyadenylation specificity factor	GO:0006379|mRNA cleavage;GO:0006378|mRNA polyadenylation;	GO:0005847|mRNA cleavage and polyadenylation specificity factor complex;	GO:0035925|mRNA 3'-UTR AU-rich region binding;GO:0003729	K14401	CPSF1, CFT1; cleavage and polyadenylation specificity factor
SHMT2	4134.908	0.268717	0.100380626	2.676976	0.007429	0.042271	Not_Sig	no	gi|77735519|ref|NP_001029454.1|	2593	0	serine hydroxymethyltransferase, mitochondrial precursor [Bos taurus]	gi|742115647|ref|XM_010839317.1|	2084	0	PREDICTED: Bison bison bison serine hydroxymethyltransferase 2 (mitochondrial) (SHMT2), transcript variant X3, mRNA	Q3SZ20	1003	0	Serine hydroxymethyltransferase, mitochondrial	SPAC24C9.12c	547	0	COG0112	pfam00464	SHMT	Serine hydroxymethyltransferase.	GO:0006544|glycine metabolic process;GO:0006563|L-serine metabolic process;GO:0070536|protein K63-linked deubiquitination;GO:0051262|protein tetramerization;GO:0034340|response to type I interferon;GO:0035999|tetrahydrofolate interconversion;GO:0046653|tetrahydrofolate metabolic process;	GO:0070552|BRISC complex;GO:0005737|cytoplasm;GO:0070062|extracellular exosome;GO:0015630|microtubule	GO:0003682|chromatin binding;GO:0004372|glycine	K00600	glyA, SHMT; glycine hydroxymethyltransferase [EC:2.1.2.1]
ZBTB7A	884.4535	0.393713	0.147079551	2.676869	0.0074314	0.042271	Not_Sig	no	gi|594035842|ref|XP_006042593.1|	2439	0	PREDICTED: LOW QUALITY PROTEIN: zinc finger and BTB domain-containing protein 7A [Bubalus bubalis]	gi|965849765|ref|XM_012139519.2|	4433	0	PREDICTED: Ovis aries musimon zinc finger and BTB domain-containing protein 7A (LOC105609837), transcript variant X2, mRNA	O95365	840	0	Zinc finger and BTB domain-containing protein 7A	.	.	.	.	pfam00651	BTB	BTB/POZ domain. The BTB (for BR-C, ttk and bab) or POZ (for Pox virus and Zinc finger) domain is present near the N-terminus of a	GO:0030154|cell differentiation;GO:0007275|multicellular organismal development;GO:0045892|negative regulation of transcription, DNA-templated;GO:0006351|transcription, DNA-templated;	GO:0005634|nucleus;	GO:0003677|DNA binding;GO:0035035|histone acetyltransferase	K10494	ZBTB7; zinc finger and BTB domain-containing protein 7
PKN2	1134.464	-0.34952	0.130590847	-2.67648	0.00744	0.042299	Not_Sig	no	gi|300794792|ref|NP_001180171.1|	4643	0	serine/threonine-protein kinase N2 [Bos taurus]	gi|742204254|ref|XM_010860346.1|	6867	0	PREDICTED: Bison bison bison protein kinase N2 (PKN2), transcript variant X1, mRNA	Q16513	1734	0	Serine/threonine-protein kinase N2	.	.	.	.	pfam00069	Pkinase	Protein kinase domain.	GO:0043297|apical junction assembly;GO:0006915|apoptotic process;GO:0007049|cell cycle;GO:0051301|cell division;GO:0010631|epithelial cell migration;GO:0035556|intracellular signal transduction;GO:0018105|peptidyl-serine phosphorylation;GO:0032467|positive regulation of cytokinesis;GO:0045931|positive regulation of mitotic cell cycle;GO:0045070|positive regulation of viral genome replication;GO:0006468|protein phosphorylation;GO:2000145|regulation of cell motility;GO:0006355|regulation of transcription, DNA-	GO:0043296|apical junction complex;GO:0030054|cell junction;GO:0005913|cell-cell adherens junction;GO:0005813|centrosome;GO:0032	GO:0005524|ATP binding;GO:0098641|cadherin binding involved in cell-cell	K06071	PKN; protein kinase N [EC:2.7.11.13]
FBXO33	439.4544	-0.36154	0.135125494	-2.67555	0.0074606	0.042386	Not_Sig	no	gi|329664676|ref|NP_001192678.1|	2415	0	F-box only protein 33 [Bos taurus]	gi|965907572|ref|XM_012167299.2|	3422	0	PREDICTED: Ovis aries musimon F-box protein 33 (FBXO33), mRNA	Q7Z6M2	918	0	F-box only protein 33	.	.	.	.	pfam12937	F-box-like	F-box-like. This is an F-box-like family.	GO:0016567|protein ubiquitination;	.	.	K10310	FBXO33; F-box protein 33
LSM3	339.6602	-0.43473	0.162489686	-2.6754	0.0074639	0.042386	Not_Sig	no	gi|7657315|ref|NP_055278.1|	407	9e-48	U6 snRNA-associated Sm-like protein LSm3 [Homo sapiens]	gi|942063350|ref|XM_005898785.2|	560	0	PREDICTED: Bos mutus LSM3 homolog, U6 small nuclear RNA and mRNA degradation associated (LSM3), mRNA	P62311	161	3e-49	U6 snRNA-associated Sm-like protein LSm3	.	.	.	.	pfam01423	LSM	LSM domain. The LSM domain contains Sm proteins as well as other related LSM (Like Sm) proteins. The U1, U2, U4/U6, and	GO:0033962|cytoplasmic mRNA processing body assembly;GO:0000398|mRNA splicing, via spliceosome;	GO:0071013|catalytic step 2 spliceosome;GO:0000932|cytoplasmic mRNA processing body;GO:1990726|Lsm1-7-Pat1 complex;GO:0071011|precatalytic	GO:0044822|poly(A) RNA binding;GO:0003723|RNA	K12622	LSM3; U6 snRNA-associated Sm-like protein LSm3
PRPF19	1100.982	0.299325	0.111885027	2.675291	0.0074665	0.042386	Not_Sig	no	gi|148709445|gb|EDL41391.1|	2626	0	PRP19/PSO4 pre-mRNA processing factor 19 homolog (S. cerevisiae), isoform CRA_a, partial [Mus musculus]	gi|742116917|ref|XM_010839634.1|	2084	0	PREDICTED: Bison bison bison pre-mRNA processing factor 19 (PRPF19), mRNA	Q08E38	1016	0	Pre-mRNA-processing factor 19	.	.	.	.	pfam08606	Prp19	Prp19/Pso4-like. This regions is found specifically in PRP19-like protein. The region represented by this family covers the sequence	GO:0034613|cellular protein localization;GO:0006303|double-strand break repair via nonhomologous end joining;GO:0001833|inner cell mass cell proliferation;GO:0008610|lipid biosynthetic process;GO:0000398|mRNA splicing, via spliceosome;GO:0045665|negative regulation of neuron differentiation;GO:0048711|positive regulation of astrocyte differentiation;GO:0048026|positive regulation of mRNA splicing, via spliceosome;GO:0010498|proteasomal protein catabolic process;GO:0070534|protein K63-linked ubiquitination;GO:0072422|signal	GO:0071013|catalytic step 2 spliceosome;GO:0005737|cytoplasm;GO:0005662|DNA replication factor A complex;GO:0005811|lipid	GO:0016874|ligase activity;GO:0061630|ubiquitin protein ligase	K10599	PRPF19, PRP19; pre-mRNA-processing factor 19 [EC:2.3.2.27]
ZNF644	583.8377	-0.35088	0.131212199	-2.67412	0.0074925	0.042513	Not_Sig	no	gi|555997852|ref|XP_005910644.1|	6799	0	PREDICTED: zinc finger protein 644 isoform X2 [Bos mutus]	gi|742204446|ref|XM_010860395.1|	6013	0	PREDICTED: Bison bison bison zinc finger protein 644 (ZNF644), transcript variant X10, mRNA	Q9H582	2458	0	Zinc finger protein 644	.	.	.	.	pfam00078	RVT_1	Reverse transcriptase (RNA-dependent DNA polymerase). A reverse transcriptase gene is usually indicative of a mobile	GO:0006355|regulation of transcription, DNA-templated;GO:0006351|transcription, DNA-templated;	GO:0005634|nucleus;	GO:0003677|DNA binding;GO:0046872|metal ion binding;	.	.
EMC7	1047.508	-0.29762	0.111314213	-2.67366	0.0075029	0.042551	Not_Sig	no	gi|149642739|ref|NP_001092613.1|	827	1e-101	ER membrane protein complex subunit 7 precursor [Bos taurus]	gi|965945743|ref|XM_004010419.3|	1727	0	PREDICTED: Ovis aries ER membrane protein complex subunit 7 (EMC7), mRNA	A5PJA8	323	4e-103	ER membrane protein complex subunit 7	.	.	.	.	pfam09430	DUF2012	Protein of unknown function (DUF2012). This is a eukaryotic family of uncharacterized proteins.	.	GO:0072546|ER membrane protein complex;GO:0016021|integral component of membrane;	GO:0030246|carbohydrate binding;	.	.
RPS6KA1	239.5587	0.397193	0.148574696	2.673354	0.0075097	0.042557	Not_Sig	no	gi|918615518|ref|XP_013375124.1|	3944	0	PREDICTED: ribosomal protein S6 kinase alpha-1 isoform X2 [Chinchilla lanigera]	gi|139948214|ref|NM_001083722.1|	3082	0	Bos taurus ribosomal protein S6 kinase, 90kDa, polypeptide 1 (RPS6KA1), mRNA	Q15418	1508	0	Ribosomal protein S6 kinase alpha-1	.	.	.	.	pfam00069	Pkinase	Protein kinase domain.	GO:0007049|cell cycle;GO:0072574|hepatocyte proliferation;GO:0035556|intracellular signal transduction;GO:0043066|negative regulation of apoptotic process;GO:0043154|negative regulation of cysteine-type endopeptidase activity involved in apoptotic process;GO:0045597|positive regulation of cell differentiation;GO:0030307|positive regulation of cell growth;GO:2000491|positive regulation of hepatic stellate cell activation;GO:0045944|positive regulation of transcription from RNA polymerase II promoter;GO:0043620|regulation of	GO:0005737|cytoplasm;GO:0005829|cytosol;GO:0005654|nucleoplasm;GO:0005634|nucleus;	GO:0005524|ATP binding;GO:0043027|cysteine-type endopeptidase	K04373	RPS6KA, RSK2; p90 ribosomal S6 kinase [EC:2.7.11.1]

SPRED1	574.1232	-0.38591	0.14435798	-2.67328	0.0075113	0.042557	Not_Sig	no	gi|300795641|ref|NP_001179445.1|	2119	0	sprouty-related, EVH1 domain-containing protein 1 [Bos taurus]	gi|942035879|ref|XM_005888927.2|	4098	0	PREDICTED: Bos mutus sprouty-related, EVH1 domain containing 1 (SPRED1), mRNA	Q7Z699	750	0	Sprouty-related, EVH1 domain-containing protein 1	.	.	.	.	pfam05210	Sprouty	Sprouty protein (Spry). This family consists of eukaryotic Sprouty protein homologues. Sprouty proteins have been revealed as	GO:0008543|fibroblast growth factor receptor signaling pathway;GO:0000188|inactivation of MAPK activity;GO:0000165|MAPK cascade;GO:0007275|multicellular organismal development;GO:0010801|negative regulation of peptidyl-threonine phosphorylation;GO:0010923|negative regulation of phosphatase activity;GO:0043517|positive regulation of DNA damage response, signal transduction by p53 class mediator;GO:0090311|regulation of protein deacetylation;	GO:0005901|caveola;GO:0005737|cytoplasm;GO:0005829|cytosol;GO:0005654|nucleoplasm;GO:0005886|plasma membrane;	GO:0019902|phosphatase binding;GO:0019901|protein kinase	K04703	SPRED; sprouty-related, EVH1 domain-containing protein
CASC3	1044.65	0.292792	0.109569502	2.672204	0.0075355	0.04266	Not_Sig	no	gi|803275088|ref|XP_011997220.1|	2352	0	PREDICTED: protein CASC3 isoform X2 [Ovis aries musimon]	gi|742135475|ref|XM_010842616.1|	3922	0	PREDICTED: Bison bison bison cancer susceptibility candidate 3 (CASC3), mRNA	A5D7H5	909	0	Protein CASC3	.	.	.	.	pfam09405	Btz	CASC3/Barentsz eIF4AIII binding. This domain is found on CASC3 (cancer susceptibility candidate gene 3 protein) which is also	GO:0008298|intracellular mRNA localization;GO:0006397|mRNA processing;GO:0051028|mRNA transport;GO:0000184|nuclear-transcribed mRNA catabolic process, nonsense-mediated decay;GO:0006417|regulation of translation;GO:0016070|RNA metabolic process;GO:0008380|RNA splicing;	GO:0035145|exon-exon junction complex;GO:0030529|ribonucleoprotein complex;GO:0016607|nuclear speck;GO:0048471|perinuclear region of	GO:0044822|poly (A) RNA binding;	K14323	CASC3, MLN51; protein CASC3
ARHGAP39	195.7344	0.511844	0.191548139	2.672141	0.0075369	0.04266	Not_Sig	no	gi|803324838|ref|XP_012020599.1|	5199	0	PREDICTED: rho GTPase-activating protein 39 isoform X2 [Ovis aries musimon]	gi|742108522|ref|XM_010837565.1|	3892	0	PREDICTED: Bison bison bison Rho GTPase activating protein 39 (ARHGAP39), transcript variant X2, mRNA	Q9C0H5	1678	0	Rho GTPase-activating protein 39	.	.	.	.	pfam00620	RhoGAP	RhoGAP domain. GTPase activator proteins towards Rho/Rac/Cdc42-like small GTPases.	GO:0051056|regulation of small GTPase mediated signal transduction;GO:0007165|signal transduction;	GO:0005856|cytoskeleton;GO:0005829|cytosol;GO:0005634|nucleus;	GO:0005096|GTPase activator activity;	.	.
PPP3CA	684.5444	-0.30393	0.113752587	-2.67189	0.0075426	0.042671	Not_Sig	no	gi|926693529|ref|XP_013819948.1|	2979	0	PREDICTED: serine/threonine-protein phosphatase 2B catalytic subunit alpha isoform [Capra hircus]	gi|942059552|ref|XM_005897690.2|	4190	0	PREDICTED: Bos mutus protein phosphatase 3, catalytic subunit, alpha isozyme (PPP3CA), transcript variant X1, mRNA	P48452	1060	0	Serine/threonine-protein phosphatase 2B catalytic subunit alpha isoform	SPBC1346.01c	653	0	COG0639	pfam00149	Metallophos	Calcineurin-like phosphoesterase. This family includes a diverse range of phosphoesterases, including protein phosphoserine	GO:0033173|calcineurin-NFAT signaling cascade;GO:0006816|calcium ion transport;GO:0014898|cardiac muscle hypertrophy in response to stress;GO:0035690|cellular response to drug;GO:0016311|dephosphorylation;GO:0060079|excitatory postsynaptic potential;GO:0000082|G1/S transition of mitotic cell cycle;GO:0050804|modulation of synaptic transmission;GO:0033555|multicellular organismal response to stress;GO:0035562|negative regulation of chromatin binding;GO:0050774|negative regulation of dendrite	GO:0005955|calcineurin complex;GO:0005829|cytosol;GO:0005739|mitochondrion;GO:0005654|nucleoplasm;GO:0098794|postsynapse;GO:0042383|sarco	GO:0033192|calmodulin-dependent protein phosphatase activity;GO:0008144	K04348	PPP3C, CNA; serine/threonine-protein phosphatase 2B catalytic subunit
RRM2B	168.9378	-0.42854	0.160416864	-2.6714	0.0075537	0.042692	Not_Sig	no	gi|556717468|ref|XP_005954644.1|	1875	0	PREDICTED: ribonucleoside-diphosphate reductase subunit M2 B isoform X1 [Pantholops hodgsonii]	gi|742217112|ref|XM_010863500.1|	2246	0	PREDICTED: Bison bison bison ribonucleotide reductase M2 B (TP53 inducible) (RRM2B), transcript variant X1, mRNA	Q5R9G0	670	0	Ribonucleoside-diphosphate reductase subunit M2 B	YJL026w	462	1.02e-147	COG0208	pfam00268	Ribonuc_red_sm	Ribonucleotide reductase, small chain.	GO:0009186|deoxyribonucleoside diphosphate metabolic process;GO:0009200|deoxyribonucleoside triphosphate metabolic process;GO:0009263|deoxyribonucleotide biosynthetic process;GO:0006281|DNA repair;GO:0001822|kidney development;GO:0006264|mitochondrial DNA replication;GO:1902254|negative regulation of intrinsic apoptotic signaling pathway by p53 class mediator;GO:0003014|renal system process;GO:0006979|response to oxidative stress;	GO:0070062|extracellular exosome;GO:0005739|mitochondrion;GO:0005654|nucleoplasm;GO:0005971|ribonucleoside-diphosphate reductase complex;	GO:0046872|metal ion binding;GO:0004748|ribonucleoside-	K10808	RRM2; ribonucleoside-diphosphate reductase subunit
FJX1	1075.882	0.368017	0.13776231	2.671391	0.0075538	0.042692	Not_Sig	no	gi|829920954|ref|XP_012594216.1|	1802	0	PREDICTED: LOW QUALITY PROTEIN: four-jointed box protein 1 [Microcebus murinus]	gi|982995629|ref|XM_003584883.3|	2352	0	PREDICTED: Bos taurus four jointed box 1 (FJX1), transcript variant X2, mRNA	Q86VR8	621	0	Four-jointed box protein 1	.	.	.	.	pfam06702	DUF1193	Protein of unknown function (DUF1193). This family represents the C-terminus of several hypothetical eukaryotic proteins of	GO:0007267|cell-cell signaling;GO:0010842|retina layer formation;	GO:0005615|extracellular space;	.	K16674	FJX1; four-jointed box protein 1
ANAPC10	65.57511	-0.64968	0.243280454	-2.67051	0.0075736	0.042783	DOWN	yes	gi|982949679|ref|XP_010812025.2|	1098	4e-133	PREDICTED: anaphase-promoting complex subunit 10 isoform X2 [Bos taurus]	gi|850491151|gb|CP011902.1|	2539	0	Ovis canadensis canadensis isolate 43U chromosome 17 sequence	Q9UM13	232	1e-67	Anaphase-promoting complex subunit 10	SPBC1A4.01	92.8	1.74e-20	COG5156	pfam03256	APC10	Anaphase-promoting complex, subunit 10 (APC10).	GO:0031145|anaphase-promoting complex-dependent proteasomal ubiquitin-dependent protein catabolic process;GO:0051301|cell division;GO:0007067|mitotic nuclear division;GO:0008054|negative regulation of cyclin-dependent protein serine/threonine kinase by cyclin degradation;GO:0051436|negative regulation of ubiquitin-protein ligase activity involved in mitotic cell cycle;GO:0051437|positive regulation of ubiquitin-protein ligase activity involved in regulation of mitotic cell cycle transition;GO:0043161|proteasome-mediated ubiquitin-	GO:0005680|anaphase-promoting complex;GO:0005829|cytosol;GO:0005654|nucleoplasm;	.	K03357	APC10, DOC1; anaphase-promoting complex subunit 10
PDK2	194.7471	0.486083	0.182043994	2.670141	0.0075819	0.042809	Not_Sig	no	gi|440910496|gb|ELR60290.1|	2157	0	[Pyruvate dehydrogenase [lipoamide]] kinase isozyme 2, mitochondrial, partial [Bos mutus]	gi|742136069|ref|XM_010842781.1|	2269	0	PREDICTED: Bison bison bison pyruvate dehydrogenase kinase, isozyme 2 (PDK2), transcript variant X4, mRNA	Q15119	796	0	[Pyruvate dehydrogenase (acetyl-transferring)] kinase isozyme 2, mitochondrial	SPAC644.11c	184	2.91e-50	COG0642	pfam10436	BCDHK_Adom3	Mitochondrial branched-chain alpha-ketoacid dehydrogenase kinase. Catabolism and synthesis of leucine, isoleucine and valine	GO:0031670|cellular response to nutrient;GO:0034614|cellular response to reactive oxygen species;GO:0042593|glucose homeostasis;GO:0006006|glucose metabolic process;GO:0008286|insulin receptor signaling pathway;GO:0072332|intrinsic apoptotic signaling pathway by p53 class mediator;GO:0010510|regulation of acetyl-CoA biosynthetic process from pyruvate;GO:0010565|regulation of cellular ketone metabolic process;GO:0006111|regulation of gluconeogenesis;GO:0010906|regulation of glucose metabolic	GO:0005737|cytoplasm;GO:0005759|mitochondrial matrix;GO:0005967|mitochondrial pyruvate dehydrogenase complex;GO:0005739|mitochondrion;GO:00	GO:0005524|ATP binding;GO:0042803|protein homodimerization	K00898	PDK2_3_4; pyruvate dehydrogenase kinase 2/3/4 [EC:2.7.11.2]
ZBTB7B	1198.497	0.317827	0.119044968	2.669803	0.0075896	0.042831	Not_Sig	no	gi|440903602|gb|ELR54239.1|	2147	0	Zinc finger and BTB domain-containing protein 7B, partial [Bos mutus]	gi|926687150|ref|XM_013976897.1|	3762	0	PREDICTED: Capra hircus zinc finger and BTB domain containing 7B (ZBTB7B), mRNA	O15156	739	0	Zinc finger and BTB domain-containing protein 7B	.	.	.	.	pfam00651	BTB	BTB/POZ domain. The BTB (for BR-C, ttk and bab) or POZ (for Pox virus and Zinc finger) domain is present near the N-terminus of a	GO:0030154|cell differentiation;GO:0007398|ectoderm development;GO:0007275|multicellular organismal development;GO:0010628|positive regulation of gene expression;GO:0043370|regulation of CD4-positive, alpha-beta T cell differentiation;GO:0043376|regulation of CD8-positive, alpha-beta T cell differentiation;GO:0006366|transcription from RNA polymerase II promoter;	GO:0005654|nucleoplasm;GO:0005634|nucleus;	GO:0046872|metal ion binding;GO:0000978|RNA polymerase II	K10494	ZBTB7; zinc finger and BTB domain-containing protein 7
VPS26A	1538.701	-0.37549	0.140656826	-2.66956	0.007595	0.042841	Not_Sig	no	gi|115495819|ref|NP_001068923.1|	1736	0	vacuolar protein sorting-associated protein 26A [Bos taurus]	gi|982970228|ref|XM_010820572.2|	2754	0	PREDICTED: Bos taurus VPS26 retromer complex component A (VPS26A), transcript variant X1, mRNA	Q0VD53	673	0	Vacuolar protein sorting-associated protein 26A	.	.	.	.	pfam03643	Vps26	Vacuolar protein sorting-associated protein 26. Vacuolar protein sorting-associated protein (Vps) 26 is one of around 50	GO:0006886|intracellular protein transport;GO:0042147|retrograde transport, endosome to Golgi;GO:1990126|retrograde transport, endosome to plasma membrane;	GO:0005829|cytosol;GO:0005769|early endosome;GO:0005768|endosome;GO:0010008|endosome membrane;GO:0070062|extracellular	GO:0008565|protein transporter activity;	K18466	VPS26; vacuolar protein sorting-associated protein 26
MAPKAPK2	1199.78	0.331663	0.124274268	2.668797	0.0076123	0.042917	Not_Sig	no	gi|982995961|ref|XP_015322163.1|	1847	0	PREDICTED: MAP kinase-activated protein kinase 2 isoform X2 [Bos taurus]	gi|982995960|ref|XM_015466677.1|	2794	0	PREDICTED: Bos taurus mitogen-activated protein kinase-activated protein kinase 2 (MAPKAPK2), transcript variant X2, mRNA	P49137	704	0	MAP kinase-activated protein kinase 2	.	.	.	.	pfam00069	Pkinase	Protein kinase domain.	GO:0070935|3'-UTR-mediated mRNA stabilization;GO:0000187|activation of MAPK activity;GO:0006974|cellular response to DNA damage stimulus;GO:0035924|cellular response to vascular endothelial growth factor stimulus;GO:0031572|G2 DNA damage checkpoint;GO:0006954|inflammatory response;GO:0048839|inner ear development;GO:0006691|leukotriene metabolic process;GO:0044351|macropinocytosis;GO:0000165|MAPK cascade;GO:0038066|p38MAPK cascade;GO:0018105|peptidyl-serine	GO:0005813|centrosome;GO:0005737|cytoplasm;GO:0005829|cytosol;GO:0070062|extracellular exosome;GO:0005654|nucleoplasm;GO:000	GO:0005524|ATP binding;GO:0009931|calcium-dependent protein	K04443	MAPKAPK2; mitogen-activated protein kinase-activated protein
TBRG4	630.7259	0.335806	0.125892345	2.667403	0.007644	0.043075	Not_Sig	no	gi|555959640|ref|XP_005891929.1|	3238	0	PREDICTED: protein TBRG4 isoform X1 [Bos mutus]	gi|742224885|ref|XM_010829026.1|	2230	0	PREDICTED: Bison bison bison transforming growth factor beta regulator 4 (TBRG4), transcript variant X2, mRNA	Q3SZK4	1239	0	Protein TBRG4	.	.	.	.	pfam08368	FAST_2	FAST kinase-like protein, subdomain 2. This family represents a conserved region of eukaryotic Fas-activated	GO:0045333|cellular respiration;	GO:0005739|mitochondrion;	GO:0004672|protein kinase activity;	.	.
WASF2	2741.953	0.294089	0.11027319	2.666917	0.0076551	0.043116	Not_Sig	no	gi|440905981|gb|ELR56297.1|	1371	3e-169	Wiskott-Aldrich syndrome protein family member 2, partial [Bos mutus]	gi|942054743|ref|XM_005895903.2|	4883	0	PREDICTED: Bos mutus WAS protein family, member 2 (WASF2), transcript variant X1, mRNA	A2VDK6	525	6e-168	Wiskott-Aldrich syndrome protein family member 2	.	.	.	.	pfam02205	WH2	WH2 motif. The WH2 motif (for Wiskott Aldrich syndrome homology region 2) has been shown in WASP and Scar1	GO:0030036|actin cytoskeleton organization;GO:0030048|actin filament-based movement;GO:0001667|ameboidal-type cell migration;GO:0001525|angiogenesis;GO:0006897|endocytosis;GO:0030032|lamellipodium assembly;GO:0072673|lamellipodium morphogenesis;GO:0035855|megakaryocyte development;GO:0051497|negative regulation of stress fiber assembly;GO:0010592|positive regulation of lamellipodium assembly;GO:0016601|Rac protein signal transduction;	GO:0005911|cell-cell junction;GO:0005856|cytoskeleton;GO:0005769|early endosome;GO:0070062|extracellular	.	K05748	WASF2; WAS protein family, member 2
LEPROTL1	825.3946	-0.38256	0.143471916	-2.66643	0.0076661	0.043155	Not_Sig	no	gi|82697353|ref|NP_001032539.1|	670	9e-86	leptin receptor overlapping transcript-like 1 [Bos taurus]	gi|965790526|ref|XM_012110853.2|	735	0	PREDICTED: Ovis aries musimon leptin receptor overlapping transcript-like 1 (LEPROTL1), mRNA	Q32PD8	262	3e-87	Leptin receptor overlapping transcript-like 1	.	.	.	.	pfam04133	Vps55	Vacuolar protein sorting 55. Vps55 is involved in the secretion of the Golgi form of the soluble vacuolar carboxypeptidase Y, but not the	.	GO:0016021|integral component of membrane;	.	.	.
LOC102273302	250.7979	-0.47515	0.178208687	-2.66628	0.0076695	0.043155	Not_Sig	no	gi|744598167|ref|XP_010989591.1|	1057	4e-143	PREDICTED: 40S ribosomal protein S4, X isoform [Camelus dromedarius]	gi|402744101|ref|NM_001035445.2|	616	0	Bos taurus ribosomal protein S4, X-linked (RPS4X), mRNA	P79103	402	3e-141	40S ribosomal protein S4	SPAC959.07	316	1.27e-109	COG1471	pfam00900	Ribosomal_S4e	Ribosomal family S4e.	GO:0006412|translation;	GO:0022627|cytosolic small ribosomal subunit;	GO:0019843|rRNA binding;GO:0003735|structural constituent of	K02987	RP-S4e, RPS4; small subunit ribosomal protein S4e
DENND1A	243.8959	0.495185	0.185742043	2.665983	0.0076764	0.043172	Not_Sig	no	gi|528969684|ref|XP_005213183.1|	4663	0	PREDICTED: DENN domain-containing protein 1A isoform X1 [Bos taurus]	gi|594098183|ref|XM_006072202.1|	4871	0	PREDICTED: Bubalus bubalis DENN/MADD domain containing 1A (DENND1A), transcript variant X1, mRNA	Q8TEH3	1546	0	DENN domain-containing protein 1A	.	.	.	.	pfam02141	DENN	DENN (AEX-3) domain. DENN (after differentially expressed in neoplastic vs normal cells) is a domain which occurs in several	GO:0032456|endocytic recycling;GO:0006897|endocytosis;GO:0015031|protein transport;GO:0032483|regulation of Rab protein signal transduction;GO:0048488|synaptic vesicle endocytosis;	GO:0030054|cell junction;GO:0030136|clathrin-coated vesicle;GO:0030665|clathrin-coated vesicle membrane;GO:0030425|dendrite;GO:00430	GO:1901981|phosphatidylinositol phosphate binding;GO:0032266	.	.
TEPSIN	218.2888	0.52921	0.198704022	2.663308	0.0077376	0.043496	Not_Sig	no	gi|741964429|ref|XP_010814766.1|	2397	0	PREDICTED: AP-4 complex accessory subunit tepsin isoform X2 [Bos taurus]	gi|742187864|ref|XM_010856310.1|	3020	0	PREDICTED: Bison bison bison ENTH domain containing 2 (ENTHD2), transcript variant X3, mRNA	Q3U3N6	547	1e-180	AP-4 complex accessory subunit tepsin	.	.	.	.	pfam01417	ENTH	ENTH domain. The ENTH (Epsin N-terminal homology) domain is found in proteins involved in endocytosis and cytoskeletal	.	GO:0016023|cytoplasmic membrane-bounded vesicle;GO:0005829|cytosol;	.	.	.
SLC38A7	208.7063	0.41559	0.156069067	2.66286	0.007748	0.043529	Not_Sig	no	gi|440902648|gb|ELR53418.1|	2231	0	Putative sodium-coupled neutral amino acid transporter 7, partial [Bos mutus]	gi|982951610|ref|XM_015475733.1|	2245	0	PREDICTED: Bos taurus solute carrier family 38 member 7 (SLC38A7), transcript variant X2, mRNA	A7E3U5	833	0	Putative sodium-coupled neutral amino acid transporter 7	YER119c	105	3.58e-23	COG0814	pfam01490	Aa_trans	Transmembrane amino acid transporter protein. This transmembrane region is found in many amino acid transporters	GO:0006867|asparagine transport;GO:0015810|aspartate transport;GO:0015808|L-alanine transport;GO:0015825|L-serine transport;GO:0006814|sodium ion transport;	GO:0030424|axon;GO:0016021|integral component of membrane;GO:0043025|neuronal cell body;	GO:0015180|L-alanine transmembrane transporter	K14994	SLC38A7_8; solute carrier family 38 (sodium-coupled neutral amino acid
PAWR	1022.536	-0.37745	0.141752863	-2.66272	0.0077511	0.043529	Not_Sig	no	gi|528948229|ref|XP_002687221.2|	1158	6e-140	PREDICTED: PRKC apoptosis WT1 regulator protein isoform X1 [Bos taurus]	gi|982981477|ref|XM_003582181.4|	8362	0	PREDICTED: Bos taurus PRKC, apoptosis, WT1, regulator (PAWR), transcript variant X2, partial mRNA	Q96IZ0	402	7e-124	PRKC apoptosis WT1 regulator protein	.	.	.	.	pfam05911	DUF869	Plant protein of unknown function (DUF869). This family consists of a number of sequences found in Arabidopsis thaliana, Oryza sativa	GO:0051017|actin filament bundle assembly;GO:0006915|apoptotic process;GO:0097190|apoptotic signaling pathway;GO:0042094|interleukin-2 biosynthetic process;GO:0030889|negative regulation of B cell proliferation;GO:0048147|negative regulation of fibroblast proliferation;GO:0042130|negative regulation of T cell proliferation;GO:0050860|negative regulation of T cell receptor signaling pathway;GO:0000122|negative regulation of transcription from RNA polymerase II promoter;GO:0042986|positive regulation of	GO:0015629|actin cytoskeleton;GO:0005884|actin filament;GO:0005737|cytoplasm;GO:0000790|nuclear	GO:0003779|actin binding;GO:0019899|enzyme binding;GO:0043522	.	.
STK40	697.684	0.40566	0.152409557	2.661644	0.007776	0.043637	Not_Sig	no	gi|115496398|ref|NP_001069195.1|	2154	0	serine/threonine-protein kinase 40 [Bos taurus]	gi|742124798|ref|XM_010841161.1|	3934	0	PREDICTED: Bison bison bison serine/threonine kinase 40 (STK40), transcript variant X2, mRNA	Q17QV9	834	0	Serine/threonine-protein kinase 40	.	.	.	.	pfam00069	Pkinase	Protein kinase domain.	GO:0005977|glycogen metabolic process;GO:0048286|lung alveolus development;GO:0060425|lung morphogenesis;GO:0035264|multicellular organism growth;GO:0043066|negative regulation of apoptotic process;GO:0010468|regulation of gene expression;GO:0043408|regulation of MAPK cascade;GO:0003016|respiratory system process;	GO:0005737|cytoplasm;GO:0005654|nucleoplasm;	GO:0005524|ATP binding;GO:0004674|protein serine/threonine	K16312	STK40, SHIK; serine/threonine-protein kinase 40 [EC:2.7.11.1]
PFDN2	299.7757	-0.42151	0.158374341	-2.66146	0.0077802	0.043637	Not_Sig	no	gi|731294180|ref|XP_010614926.1|	634	4e-80	PREDICTED: prefoldin subunit 2 [Fukomys damarensis]	gi|942095798|ref|XM_005909195.2|	638	0	PREDICTED: Bos mutus prefoldin subunit 2 (PFDN2), mRNA	Q9UHV9	239	7e-79	Prefoldin subunit 2	.	.	.	.	pfam01920	Prefoldin_2	Prefoldin subunit. This family includes prefoldin subunits that are not detected by pfam02996.	GO:0051495|positive regulation of cytoskeleton organization;GO:0006457|protein folding;	GO:0005737|cytoplasm;GO:0070062|extracellular exosome;GO:0005739|mitochondrion;GO:0005730|nucleolus;GO:0005654|nucleoplas	GO:0044183|protein binding involved in protein folding;GO:0051082	K09549	PFDN2; prefoldin subunit 2
PSME1	1335.795	-0.3314	0.124520371	-2.66139	0.0077818	0.043637	Not_Sig	no	gi|671032402|ref|XP_008707006.1|	1120	1e-149	PREDICTED: proteasome activator complex subunit 1 [Ursus maritimus]	gi|942082184|ref|XM_005904906.2|	1005	0	PREDICTED: Bos mutus proteasome activator subunit 1 (PSME1), transcript variant X1, mRNA	Q4U5R3	428	1e-149	Proteasome activator complex subunit 1	.	.	.	.	pfam02252	PA28_beta	Proteasome activator pa28 beta subunit. PA28 activator complex (also known as 11s regulator of 20S proteasome) is a ring shaped	GO:0010950|positive regulation of endopeptidase activity;GO:2000045|regulation of G1/S transition of mitotic cell cycle;GO:0061136|regulation of proteasomal protein catabolic process;	GO:0005737|cytoplasm;GO:0008537|proteasome activator complex;GO:0000502|proteasome complex;	GO:0061133|endopeptidase activator activity;	.	.
WBP11	1113.245	0.291701	0.109615748	2.661119	0.0077881	0.043652	Not_Sig	no	gi|426225460|ref|XP_004006884.1|	1436	0	PREDICTED: WW domain-binding protein 11 [Ovis aries]	gi|942078151|ref|XM_014480768.1|	2823	0	PREDICTED: Bos mutus WW domain binding protein 11 (WBP11), transcript variant X3, mRNA	Q9Y2W2	534	2e-176	WW domain-binding protein 11	.	.	.	.	pfam09429	Wbp11	WW domain binding protein 11. The WW domain is a small protein module with a triple-stranded beta-sheet fold. This is a family of	GO:0045292|mRNA cis splicing, via spliceosome;GO:1903955|positive regulation of protein targeting to mitochondrion;GO:1903146|regulation of mitophagy;GO:0006364|rRNA processing;	GO:0005737|cytoplasm;GO:0043231|intracellular membrane-bounded organelle;GO:0016607|nuclear speck;GO:0005654|nucleoplasm;GO:000563	GO:0044822|poly (A) RNA binding;GO:0008599|protein	K12866	WBP11, NPWBP; WW domain-binding protein 11
GNB1	6911.454	0.281182	0.105701832	2.660146	0.0078107	0.043757	Not_Sig	no	gi|664769486|ref|XP_008542291.1|	1762	0	PREDICTED: guanine nucleotide-binding protein G(I)/G(S)/G(T) subunit beta-1 isoform X1 [Equus przewalskii]	gi|528980063|ref|XM_005217017.1|	2972	0	PREDICTED: Bos taurus guanine nucleotide binding protein (G protein), beta polypeptide 1 (GNB1), transcript variant X1, mRNA	P54311	670	0	Guanine nucleotide-binding protein G(I)/G(S)/G(T) subunit beta-1	.	.	.	.	pfam00400	WD40	WD domain, G-beta repeat.	GO:0010659|cardiac muscle cell apoptotic process;GO:0008283|cell proliferation;GO:0071456|cellular response to hypoxia;GO:0007186|G-protein coupled receptor signaling pathway;GO:0007200|phospholipase C-activating G-protein coupled receptor signaling pathway;GO:0007204|positive regulation of cytosolic calcium ion concentration;GO:0070208|protein heterotrimerization;GO:0060041|retina development in camera-type eye;GO:0050909|sensory perception of taste;	GO:0044297|cell body;GO:0030425|dendrite;GO:0070062|extracellular exosome;GO:0005834|heterotrimeric G-	GO:0047391|alkylglycerophosphoethanolamine phosphodiesterase	K04536	GNB1; guanine nucleotide-binding protein G(I)/G(S)/G(T)
LIX1L	778.8605	-0.29659	0.111500764	-2.65997	0.0078149	0.043759	Not_Sig	no	gi|470621507|ref|XP_004318804.1|	1870	0	PREDICTED: LOW QUALITY PROTEIN: LIX1-like protein [Tursiops truncatus]	gi|594032422|ref|XM_006040915.1|	1000	0	PREDICTED: Bubalus bubalis Lix1 homolog (mouse)-like (LIX1L), mRNA	Q8BQ89	496	3e-171	LIX1-like protein	.	.	.	.	pfam14954	LIX1	Limb expression 1. This entry represents the limb expression 1 (LIX1) family.	GO:0097352|autophagosome maturation;	GO:0005737|cytoplasm;	.	K16673	LIX1L; LIX1-like protein
TIA1	666.8436	-0.32763	0.123179419	-2.65977	0.0078193	0.043762	Not_Sig	no	gi|741943388|ref|XP_010808429.1|	1740	0	PREDICTED: nucleolysin TIA-1 isoform p40 isoform X6 [Bos taurus]	gi|982939169|ref|XM_010810127.2|	4653	0	PREDICTED: Bos taurus TIA1 cytotoxic granule-associated RNA binding protein (TIA1), transcript variant X6, mRNA	P31483	652	0	Nucleolysin TIA-1 isoform p40	.	.	.	.	pfam00076	RRM_1	RNA recognition motif. (a.k.a. RRM, RBD, or RNP domain). The RRM motif is probably diagnostic of an RNA binding protein. RRMs	GO:0006915|apoptotic process;GO:0008543|fibroblast growth factor receptor signaling pathway;GO:0042036|negative regulation of cytokine biosynthetic process;GO:0017148|negative regulation of translation;GO:1904037|positive regulation of epithelial cell apoptotic process;GO:1903608|protein localization to cytoplasmic stress granule;GO:0048024|regulation of mRNA splicing, via spliceosome;	GO:0005737|cytoplasm;GO:0010494|cytoplasmic stress granule;GO:0097165|nuclear stress granule;GO:0005654|nucleoplasm;	GO:0017091|AU-rich element binding;GO:0000166|nucleotide	K13201	TIA1, TIAL1; nucleolysin TIA-1/TIAR
MAP7D1	2232.3	0.276904	0.104135503	2.659077	0.0078355	0.043832	Not_Sig	no	gi|741875358|ref|XP_010825422.1|	1626	0	PREDICTED: MAP7 domain-containing protein 1 isoform X1 [Bos taurus]	gi|982979713|ref|XM_010827121.2|	3477	0	PREDICTED: Bos taurus MAP7 domain containing 1 (MAP7D1), transcript variant X2, mRNA	Q3KQU3	503	3e-159	MAP7 domain-containing protein 1	.	.	.	.	pfam05672	MAP7	MAP7 (E-MAP-115) family. The organisation of microtubules varies with the cell type and is presumably controlled by tissue-	GO:0000226|microtubule cytoskeleton organization;	GO:0005737|cytoplasm;GO:0015630|microtubule cytoskeleton;GO:0005819|spindle;	GO:0005198|structural molecule activity;	K16806	MAP7D1; MAP7 domain-containing protein 1
PLD2	536.3878	0.32681	0.122915753	2.658813	0.0078417	0.043838	Not_Sig	no	gi|555988551|ref|XP_005906114.1|	4910	0	PREDICTED: phospholipase D2 [Bos mutus]	gi|115496503|ref|NM_001075827.1|	3408	0	Bos taurus phospholipase D2 (PLD2), mRNA	Q0V8L6	1893	0	Phospholipase D2	SPAC2F7.16c	427	4.32e-128	COG1502	pfam00787	PX	PX domain. PX domains bind to phosphoinositides.	GO:0048870|cell motility;GO:0048017|inositol lipid-mediated signaling;GO:0016042|lipid catabolic process;GO:0006654|phosphatidic acid biosynthetic process;GO:0036465|synaptic vesicle recycling;	GO:0005794|Golgi apparatus;GO:0016020|membrane;GO:0098793|presynapse;	GO:0070290|N-acylphosphatidylethanolamine-specific phospholipase D	K01115	PLD1_2; phospholipase D1/2 [EC:3.1.4.4]
MPP1	635.0722	0.336008	0.126380783	2.658696	0.0078444	0.043838	Not_Sig	no	gi|594074230|ref|XP_006060919.1|	2360	0	PREDICTED: 55 kDa erythrocyte membrane protein isoform X1 [Bubalus bubalis]	gi|115497507|ref|NM_001075484.1|	1982	0	Bos taurus membrane protein, palmitoylated 1 (MPP1), mRNA	Q17QN6	909	0	55 kDa erythrocyte membrane protein	SPBC1198.05	95.5	1.14e-21	COG0194	pfam00625	Guanylate_kin	Guanylate kinase.	GO:0090022|regulation of neutrophil chemotaxis;	GO:0030863|cortical cytoskeleton;GO:0016020|membrane;GO:0032420|stereocilium;	.	.	.
SLC12A4	2797.576	0.337734	0.127045343	2.658373	0.0078519	0.043859	Not_Sig	no	gi|291490697|ref|NP_001167562.1|	5211	0	solute carrier family 12 member 4 [Bos taurus]	gi|291490696|ref|NM_001174091.1|	3745	0	Bos taurus solute carrier family 12 (potassium/chloride transporter), member 4 (SLC12A4), mRNA	Q28677	1965	0	Solute carrier family 12 member 4	SPBC18H10.16	158	1.92e-38	COG0531	pfam00324	AA_permease	Amino acid permease.	GO:0006813|potassium ion transport;	GO:0016021|integral component of membrane;	GO:0015379|potassium:chloride symporter activity;	K14427	SLC12A4_5_6, KCC; solute carrier family 12 (potassium/chloride
STK11	835.3931	0.329755	0.1240514	2.658214	0.0078556	0.043859	Not_Sig	no	gi|585202045|ref|XP_006751885.1|	1887	0	PREDICTED: serine/threonine-protein kinase STK11-like [Leptonychotes weddellii]	gi|594052503|ref|XM_006050503.1|	3281	0	PREDICTED: Bubalus bubalis serine/threonine kinase 11 (STK11), transcript variant X3, mRNA	Q15831	660	0	Serine/threonine-protein kinase STK11	.	.	.	.	pfam00069	Pkinase	Protein kinase domain.	GO:0032147|activation of protein kinase activity;GO:0043276|anoikis;GO:0006914|autophagy;GO:0007409|axonogenesis;GO:0060070|canonical Wnt signaling pathway;GO:0007050|cell cycle arrest;GO:0006974|cellular response to DNA damage stimulus;GO:0071493|cellular response to UV-B;GO:0097484|dendrite extension;GO:0030010|establishment of cell polarity;GO:0042593|glucose homeostasis;GO:0051645|Golgi	GO:0005737|cytoplasm;GO:0005829|cytosol;GO:0070062|extracellular exosome;GO:0016020|membrane;GO:0005739|mitochondrion;GO:0005654|nucleopla	GO:0005524|ATP binding;GO:0000287|magnesium ion binding;GO:0002039	K07298	STK11, LKB1; serine/threonine-protein kinase 11
YLPM1	1118.392	0.295533	0.111184804	2.658031	0.0078599	0.043861	Not_Sig	no	gi|440901727|gb|ELR52616.1|	5839	0	YLP motif-containing protein 1, partial [Bos mutus]	gi|555978196|ref|XM_005900981.1|	7495	0	PREDICTED: Bos mutus YLP motif containing 1 (YLPM1), mRNA	P49750	1226	0	YLP motif-containing protein 1	.	.	.	.	pfam13671	AAA_33	AAA domain. This family of domains contain only a P-loop motif, that is characteristic of the AAA superfamily. Many of the	GO:0006355|regulation of transcription, DNA-templated;GO:0006351|transcription, DNA-templated;	GO:0005737|cytoplasm;GO:0016607|nuclear speck;GO:0005654|nucleoplasm;GO:0005634|nucleus;	GO:0044822|poly (A) RNA binding;	K17602	YLPM1; YLP motif-containing protein 1
CD59	192.2488	-0.43768	0.164697909	-2.65746	0.0078732	0.043914	Not_Sig	no	gi|555957639|ref|XP_005890947.1|	581	2e-68	PREDICTED: CD59 glycoprotein [Bos mutus]	gi|982946716|ref|XM_005216374.3|	1724	0	PREDICTED: Bos taurus CD59 molecule (CD59), transcript variant X1, mRNA	O62680	137	1e-36	CD59 glycoprotein	.	.	.	.	pfam00021	UPAR_LY6	u-PAR/Ly-6 domain. This extracellular disulphide bond rich domain is related to pfam00087.	GO:0001775|cell activation;GO:0007166|cell surface receptor signaling pathway;GO:0045087|innate immune response;GO:0001971|negative regulation of activation of membrane attack complex;GO:0030449|regulation of complement activation;	GO:0031362|anchored component of external side of plasma membrane;GO:0009986|cell surface;GO:0070062|extracellular	GO:0001848|complement binding;	K04008	CD59; CD59 antigen
TMEM127	798.6656	0.397398	0.149586004	2.656655	0.007892	0.043998	Not_Sig	no	gi|795319418|ref|XP_011825076.1|	994	7e-124	PREDICTED: transmembrane protein 127, partial [Mandrillus leucophaeus]	gi|742227069|ref|XM_010829583.1|	2937	0	PREDICTED: Bison bison bison transmembrane protein 127 (TMEM127), mRNA	O75204	387	4e-125	Transmembrane protein 127	.	.	.	.	pfam13903	Claudin_2	PMP-22/EMP/MP20/Claudin tight junction. Members of this family are claudins, that form tight junctions between cells.	GO:0007032|endosome organization;GO:0008285|negative regulation of cell proliferation;GO:0032007|negative regulation of TOR signaling;	GO:0005737|cytoplasm;GO:0005769|early endosome;GO:0016021|integral component of membrane;GO:0005886|plasma membrane;	GO:0017137|Rab GTPase binding;	.	.
BAHD1	488.5242	0.347374	0.130776506	2.656241	0.0079017	0.04403	Not_Sig	no	gi|440911673|gb|ELR61314.1|	3309	0	Bromo adjacent-like protein domain-containing 1 protein, partial [Bos mutus]	gi|982936778|ref|XM_015473124.1|	4292	0	PREDICTED: Bos taurus bromo adjacent homology domain containing 1 (BAHD1), transcript variant X1, mRNA	Q8TBE0	1142	0	Bromo adjacent homology domain-containing 1 protein	.	.	.	.	pfam01426	BAH	BAH domain. This domain has been called BAH (Bromo adjacent homology) domain and has also been called ELM1 and BAM	GO:0016569|covalent chromatin modification;GO:0006342|chromatin silencing;GO:0031507|heterochromatin assembly;GO:0045892|negative regulation of transcription, DNA-templated;GO:0006351|transcription, DNA-templated;	GO:0000785|chromatin;GO:0005677|chromatin silencing complex;GO:0005654|nucleoplasm;	GO:0003682|chromatin binding;GO:0000976|transcription	.	.
LOC106700981	897.7146	-0.91734	0.345417305	-2.65574	0.0079136	0.044074	DOWN	yes	gi|556765364|ref|XP_005977945.1|	370	2e-41	PREDICTED: keratin, type I cytoskeletal 9-like [Pantholops hodgsonii]	gi|742157753|ref|XM_010848034.1|	419	0	PREDICTED: Bison bison bison cytochrome c oxidase subunit 7C, mitochondrial (LOC104994451), mRNA	P00430	132	4e-39	Cytochrome c oxidase subunit 7C, mitochondrial	.	.	.	.	pfam02935	COX7C	Cytochrome c oxidase subunit VIIc. Cytochrome c oxidase, a 13 sub-unit complex, EC:1.9.3.1 is the terminal oxidase in the	.	GO:0016021|integral component of membrane;GO:0005743|mitochondrial inner membrane;GO:0045277|respiratory chain complex IV;	GO:0004129|cytochrome-c oxidase activity;	K02272	COX7C; cytochrome c oxidase subunit 7c
NFKBIE	625.9687	0.336298	0.126638455	2.655579	0.0079172	0.044074	Not_Sig	no	gi|440902434|gb|ELR53226.1|	1986	0	NF-kappa-B inhibitor epsilon, partial [Bos mutus]	gi|942067522|ref|XM_005900083.2|	2279	0	PREDICTED: Bos mutus nuclear factor of kappa light polypeptide gene enhancer in B-cells inhibitor, epsilon (NFKBIE), mRNA	O00221	575	0	NF-kappa-B inhibitor epsilon	RSc1788	75.1	7.57e-14	COG0666	pfam12796	Ank_2	Ankyrin repeats (3 copies).	GO:0042994|cytoplasmic sequestering of transcription factor;GO:0042942|D-serine transport;	GO:0005737|cytoplasm;GO:0005829|cytosol;GO:0005794|Golgi apparatus;GO:0048471|perinuclear region of cytoplasm;	.	K05872	NFKBIE; NF-kappa-B inhibitor epsilon
FBXO42	889.3312	0.310483	0.116936819	2.655136	0.0079276	0.044111	Not_Sig	no	gi|555988466|ref|XP_005906072.1|	3500	0	PREDICTED: F-box only protein 42 [Bos mutus]	gi|982978072|ref|XM_003581908.4|	5659	0	PREDICTED: Bos taurus F-box protein 42 (FBXO42), transcript variant X1, mRNA	Q6P3S6	1301	0	F-box only protein 42	.	.	.	.	pfam13415	Kelch_3	Galactose oxidase, central domain.	.	.	.	K10317	FBXO42; F-box protein 42
RAMAC	225.3317	-0.46034	0.173388081	-2.65495	0.0079321	0.044114	Not_Sig	no	gi|594105438|ref|XP_006075674.1|	539	7e-64	PREDICTED: RNMT-activating mini protein-like isoform X1 [Bubalus bubalis]	gi|982960090|ref|XM_005221844.3|	1318	0	PREDICTED: Bos taurus family with sequence similarity 103 member A1 (FAM103A1), transcript variant X1, mRNA	Q5R9Q6	195	6e-59	RNMT-activating mini protein	.	.	.	.	pfam15320	RAM	mRNA cap methylation, RNMT-activating mini protein. This family of proteins is found in eukaryotes. Proteins in this family are typically	GO:0006370|7-methylguanosine mRNA capping;GO:0032259|methylation;GO:0036031|recruitment of mRNA capping enzyme to RNA polymerase II holoenzyme complex;	GO:0005845|mRNA cap binding complex;GO:0005634|nucleus;	GO:0044822|poly (A) RNA binding;GO:0003723|RNA binding;	K18708	FAM103A1; RNMT-activating mini protein

UBE2G1	931.6685	-0.29929	0.112787274	-2.65358	0.0079642	0.044271	Not_Sig	no	gi|655869905|ref|XP_008269303.1|	964	3e-116	PREDICTED: ubiquitin-conjugating enzyme E2 G1 [Oryctolagus cuniculus]	gi|594114010|ref|XM_006079676.1|	3634	0	PREDICTED: Bubalus bubalis ubiquitin-conjugating enzyme E2G 1 (UBE2G1), mRNA	P62255	322	3e-100	Ubiquitin-conjugating enzyme E2 G1	SPBC1105.09	205	3.00e-60	COG5078	pfam00179	UQ_con	Ubiquitin-conjugating enzyme. Proteins destined for proteasome-mediated degradation may be ubiquitinated. Ubiquitination	GO:0070936|protein K48-linked ubiquitination;GO:0070534|protein K63-linked ubiquitination;GO:0000209|protein polyubiquitination;GO:0016567|protein ubiquitination;GO:0006511|ubiquitin-dependent protein catabolic process;	GO:0005737|cytoplasm;GO:0070062|extracellular exosome;	GO:0005524|ATP binding;GO:0061631|ubiquitin conjugating enzyme	K10575	UBE2G1, UBC7; ubiquitin-conjugating enzyme E2 G1 [EC:2.3.2.23]
RTEL1	378.4315	0.352356	0.132802423	2.653238	0.0079724	0.044295	Not_Sig	no	gi|742103883|ref|XP_010834873.1|	6635	0	PREDICTED: regulator of telomere elongation helicase 1 isoform X1 [Bison bison bison]	gi|742103882|ref|XM_010836571.1|	4603	0	PREDICTED: Bison bison bison regulator of telomere elongation helicase 1 (RTEL1), transcript variant X1, mRNA	A4K436	2393	0	Regulator of telomere elongation helicase 1	YER171w	232	8.98e-63	COG1199	pfam06733	DEAD_2	DEAD_2. This represents a conserved region within a number of RAD3-like DNA-binding helicases that are seemingly	GO:0006310|DNA recombination;GO:0006281|DNA repair;GO:0006260|DNA replication;GO:0010569|regulation of double-strand break repair via homologous recombination;GO:0000723|telomere maintenance;	GO:0005634|nucleus;	GO:0051539|4 iron, 4 sulfur cluster binding;GO:0005524|ATP	K11136	RTEL1; regulator of telomere elongation helicase 1 [EC:3.6.4.12]
ZDHHC17	541.6413	-0.36065	0.135948504	-2.65287	0.0079811	0.044309	Not_Sig	no	gi|817344043|ref|XP_012297503.1|	2735	0	PREDICTED: palmitoyltransferase ZDHHC17 [Aotus nancymaae]	gi|942049011|ref|XM_005893634.2|	4284	0	PREDICTED: Bos mutus zinc finger, DHHC-type containing 17 (ZDHHC17), mRNA	Q8IUH5	1048	0	Palmitoyltransferase ZDHHC17	SPAC2F7.10_1	104	3.04e-23	COG0666	pfam01529	zf-DHHC	DHHC palmitoyltransferase. This family includes the well known DHHC zinc binding domain as well as three of the four conserved	GO:0042953|lipoprotein transport;GO:0043123|positive regulation of I-kappaB kinase/NF-kappaB signaling;GO:0018345|protein palmitoylation;	GO:0030054|cell junction;GO:0005737|cytoplasm;GO:0030659|cytoplasmic vesicle membrane;GO:0005794|Golgi	GO:0042802|identical protein binding;GO:0015095|magnesium ion	.	.
DCP1B	108.541	0.590781	0.222713992	2.652645	0.0079864	0.044309	UP	yes	gi|296487051|tpg|DAA29164.1|	2505	0	TPA: mRNA-decapping enzyme 1B [Bos taurus]	gi|402744158|ref|NM_001034467.2|	1864	0	Bos taurus decapping mRNA 1B (DCP1B), mRNA	Q3SZL6	968	0	mRNA-decapping enzyme 1B	.	.	.	.	pfam06058	DCP1	Dcp1-like decapping family. An essential step in mRNA turnover is decapping. In yeast, two proteins have been identified that are	GO:0000290|deadenylation-dependent decapping of nuclear-transcribed mRNA;GO:0031087|deadenylation-independent decapping of nuclear-transcribed mRNA;GO:0000184|nuclear-transcribed mRNA catabolic process, nonsense-mediated decay;	GO:0000932|cytoplasmic mRNA processing body;GO:0005634|nucleus;	GO:0008047|enzyme activator activity;GO:0030234|enzyme regulator	K12611	DCP1B; mRNA-decapping enzyme 1B [EC:3.-.-.-]
MRPL23	354.7111	-0.34745	0.130988337	-2.65256	0.0079883	0.044309	Not_Sig	no	gi|296483789|tpg|DAA25904.1|	789	1e-104	TPA: 39S ribosomal protein L23, mitochondrial-like [Bos taurus]	gi|118150875|ref|NM_001077884.1|	555	0	Bos taurus mitochondrial ribosomal protein L23 (MRPL23), mRNA	Q16540	264	7e-89	39S ribosomal protein L23, mitochondrial	PM1413	52	2.06e-08	COG0089	pfam00276	Ribosomal_L23	Ribosomal protein L23.	GO:0032543|mitochondrial translation;GO:0070125|mitochondrial translational elongation;GO:0070126|mitochondrial translational termination;GO:0006412|translation;	GO:0005743|mitochondrial inner membrane;GO:0005762|mitochondrial large ribosomal subunit;GO:0005739|mitochondrion;GO:00	GO:0000166|nucleotide binding;GO:0003723|RNA	K02892	RP-L23, MRPL23, rplW; large subunit ribosomal protein L23
CCNG1	3719.773	-0.29307	0.110497925	-2.65227	0.0079953	0.044309	Not_Sig	no	gi|61656171|ref|NP_001013382.1|	1493	0	cyclin-G1 [Bos taurus]	gi|151557068|gb|BC150004.1|	2309	0	Bos taurus cyclin G1, mRNA (cDNA clone MGC:165783 IMAGE:8417722), complete cds	Q5E9I1	579	0	Cyclin-G1	YLR210w	51.2	7.85e-06	COG5024	pfam00134	Cyclin_N	Cyclin, N-terminal domain. Cyclins regulate cyclin dependent kinases (CDKs). Human cyclin-O is a Uracil-DNA glycosylase that is related to	GO:0051301|cell division;GO:0007067|mitotic nuclear division;GO:0051726|regulation of cell cycle;	GO:0005634|nucleus;	.	K10145	CCNG1; cyclin G1
LSM1	349.058	-0.4217	0.15900434	-2.65215	0.007998	0.044309	Not_Sig	no	gi|731489888|ref|XP_003412581.2|	693	8e-87	PREDICTED: U6 snRNA-associated Sm-like protein LSm1 [Loxodonta africana]	gi|742113409|ref|XM_010838777.1|	1001	0	PREDICTED: Bison bison bison LSM1, U6 small nuclear RNA associated (LSM1), mRNA	Q5E9Z8	265	3e-87	U6 snRNA-associated Sm-like protein LSm1	.	.	.	.	pfam01423	LSM	LSM domain. The LSM domain contains Sm proteins as well as other related LSM (Like Sm) proteins. The U1, U2, U4/U6, and	GO:0000290|deadenylation-dependent decapping of nuclear-transcribed mRNA;GO:0071044|histone mRNA catabolic process;GO:0006397|mRNA processing;GO:0045665|negative regulation of neuron differentiation;GO:0008380|RNA splicing;GO:0019827|stem cell population maintenance;	GO:0000932|cytoplasmic mRNA processing body;GO:0005634|nucleus;	GO:0044822|poly (A) RNA binding;GO:0000339|RNA cap binding;	K12620	LSM1; U6 snRNA-associated Sm-like protein LSm1
UQCC2	166.2339	-0.56736	0.213925468	-2.65215	0.007998	0.044309	Not_Sig	no	gi|965991489|ref|XP_014958058.1|	735	1e-96	PREDICTED: ubiquinol-cytochrome-c reductase complex assembly factor 2 isoform X1 [Ovis aries]	gi|742159759|ref|XM_010848619.1|	489	0	PREDICTED: Bison bison bison ubiquinol-cytochrome-c reductase complex assembly factor 2 (LOC104994909), mRNA	Q3SZ13	281	4e-96	Ubiquinol-cytochrome-c reductase complex assembly factor 2	.	.	.	.	pfam01044	Vinculin	Vinculin family.	GO:0034551|mitochondrial respiratory chain complex III assembly;GO:1903364|positive regulation of cellular protein catabolic process;GO:0070131|positive regulation of mitochondrial translation;GO:0050796|regulation of insulin secretion;GO:0002082|regulation of oxidative phosphorylation;GO:2001014|regulation of skeletal muscle cell differentiation;	GO:0005743|mitochondrial inner membrane;GO:0005758|mitochondrial intermembrane space;GO:0005759|mitochondrial	.	K17682	MNF1; mitochondrial nucleoid factor 1
DPY19L1	1578.038	-0.29385	0.110818046	-2.65165	0.0080099	0.044354	Not_Sig	no	gi|594095655|ref|XP_006071107.1|	3332	0	PREDICTED: probable C-mannosyltransferase DPY19L1, partial [Bubalus bubalis]	gi|982980602|ref|XM_010827329.2|	4901	0	PREDICTED: Bos taurus dpy-19 like 1 (C. elegans) (DPY19L1), transcript variant X1, mRNA	Q2PZI1	1262	0	Probable C-mannosyltransferase DPY19L1	.	.	.	.	pfam10034	Dpy19	Q-cell neuroblast polarisation. Dyp-19, formerly known as DUF2211, is a transmembrane domain family that is required to	GO:0018406|protein C-linked glycosylation via 2'-alpha-mannosyl-L-tryptophan;	GO:0016021|integral component of membrane;GO:0016020|membrane;GO:0005637|nuclear inner membrane;	GO:0000030|mannosyltransferase activity;	.	.
MICOS13	482.9632	-0.34728	0.130984741	-2.65133	0.0080176	0.044375	Not_Sig	no	gi|61887001|ref|XP_613885.1|	607	5e-76	PREDICTED: MICOS complex subunit MIC13 isoform X2 [Bos taurus]	gi|942072336|ref|XM_005901723.2|	770	0	PREDICTED: Bos mutus chromosome unknown open reading frame, human C19orf70 (LOC102269931), transcript variant X2, mRNA	A1XQR7	211	6e-67	MICOS complex subunit MIC13	.	.	.	.	pfam06674	DUF1176	Protein of unknown function (DUF1176). This family consists of several hypothetical bacterial proteins of around 340 residues in	GO:0042407|cristae formation;	GO:0061617|MICOS complex;GO:0044284|mitochondrial crista junction;GO:0005743|mitochondrial inner membrane;	.	.	.
LOC102285446	174.592	-0.45094	0.170119246	-2.65074	0.0080315	0.0444	Not_Sig	no	gi|803171524|ref|XP_011952916.1|	633	6e-76	PREDICTED: centrin-4 isoform X1 [Ovis aries]	gi|942066252|ref|XM_005899732.2|	1064	0	PREDICTED: Bos mutus centrin-4 (LOC102285446), mRNA	Q8K4K1	204	7e-61	Centrin-4	SPCC1682.04	120	3.50e-31	COG5126	pfam13499	EF-hand_7	EF-hand domain pair.	.	GO:0005814|centriole;GO:0036064|ciliary basal body;	GO:0005509|calcium ion binding;GO:0031683|G-protein	.	.
COX4I1	2176.857	-0.27285	0.102934468	-2.65068	0.0080329	0.0444	Not_Sig	no	gi|47824862|ref|NP_001001439.1|	897	7e-120	cytochrome c oxidase subunit 4 isoform 1, mitochondrial precursor [Bos taurus]	gi|528990224|ref|XM_005218438.1|	748	0	PREDICTED: Bos taurus cytochrome c oxidase subunit IV isoform 1 (COX4I1), transcript variant X1, mRNA	P00423	350	2e-121	Cytochrome c oxidase subunit 4 isoform 1, mitochondrial	.	.	.	.	pfam02936	COX4	Cytochrome c oxidase subunit IV. Cytochrome c oxidase, a 13 sub-unit complex, EC:1.9.3.1 is the terminal oxidase in the	GO:0006123|mitochondrial electron transport, cytochrome c to oxygen;	GO:0070062|extracellular exosome;GO:0016021|integral component of membrane;GO:0005751|mitochondrial respiratory chain complex	GO:0004129|cytochrome-c oxidase activity;	K02263	COX4; cytochrome c oxidase subunit 4
CIBAR1	437.6134	-0.32279	0.121777287	-2.65065	0.0080337	0.0444	Not_Sig	no	gi|78369336|ref|NP_001030519.1|	1437	0	protein FAM92A1 [Bos taurus]	gi|926708720|ref|XM_013969259.1|	965	0	PREDICTED: Capra hircus family with sequence similarity 92, member A1 (FAM92A1), mRNA	Q3SZG6	558	0	Protein FAM92A1	.	.	.	.	pfam06730	FAM92	FAM92 protein. This family of proteins has a role in embryogenesis. During embryogenesis it is essential for	.	.	.	.	.
CENPT	190.327	0.431744	0.162902882	2.650313	0.0080417	0.04441	Not_Sig	no	gi|742168506|ref|XP_010849319.1|	2324	0	PREDICTED: centromere protein T isoform X2 [Bison bison bison]	gi|594097767|ref|XM_006072000.1|	2021	0	PREDICTED: Bubalus bubalis centromere protein T (CENPT), transcript variant X3, mRNA	Q4R5U8	571	0	Centromere protein T	SPBC800.13_2	51.2	4.35e-07	COG2036	pfam15511	CENP-T	Centromere kinetochore component CENP-T. CENP-T is a family of vertebral kinetochore proteins that associates directly	GO:0051301|cell division;GO:0051276|chromosome organization;GO:0007059|chromosome segregation;GO:0051382|kinetochore assembly;GO:0000278|mitotic cell cycle;GO:0007067|mitotic nuclear division;	GO:0000775|chromosome, centromeric region;GO:0000777|condensed chromosome kinetochore;GO:0005634|nucleus;	GO:0003677|DNA binding;	K11512	CENPT; centromere protein T
CHM	234.0973	-0.40272	0.15195663	-2.65025	0.0080433	0.04441	Not_Sig	no	gi|555980989|ref|XP_005902417.1|	3230	0	PREDICTED: rab proteins geranylgeranyltransferase component A 1 [Bos mutus]	gi|942074417|ref|XM_005902355.2|	5177	0	PREDICTED: Bos mutus choroideremia (Rab escort protein 1) (CHM), mRNA	P24386	1106	0	Rab proteins geranylgeranyltransferase component A 1	YER136w	132	1.48e-31	COG5044	pfam00996	GDI	GDP dissociation inhibitor.	GO:0018344|protein geranylgeranylation;GO:0006612|protein targeting to membrane;GO:0042981|regulation of apoptotic process;GO:0007264|small GTPase mediated signal transduction;GO:0007601|visual perception;	GO:0005829|cytosol;GO:0005968|Rab-protein geranylgeranyltransferase complex;	GO:0005092|GDP-dissociation inhibitor activity;GO:0005096	.	.
TNS1	14173.46	0.353028	0.13323556	2.649654	0.0080574	0.044467	Not_Sig	no	gi|594043873|ref|XP_006046443.1|	8290	0	PREDICTED: tensin-1 isoform X1 [Bubalus bubalis]	gi|594043884|ref|XM_006046387.1|	9489	0	PREDICTED: Bubalus bubalis tensin 1 (TNS1), transcript variant X7, mRNA	Q9GLM4	3035	0	Tensin-1	SPBC609.02	72.4	6.23e-12	COG2453	pfam08416	PTB	Phosphotyrosine-binding domain. The phosphotyrosine-binding domain (PTB, also phosphotyrosine-interaction or PI	.	GO:0009986|cell surface;GO:0005737|cytoplasm;GO:0005856|cytoskeleton;GO:0005925|focal adhesion;	.	K18080	TNS; tensin
MAD1L1	161.8346	0.4525	0.170808695	2.649163	0.0080691	0.04449	Not_Sig	no	gi|156120735|ref|NP_001095514.1|	3072	0	mitotic spindle assembly checkpoint protein MAD1 [Bos taurus]	gi|965834045|ref|XM_012132643.2|	3135	0	PREDICTED: Ovis aries musimon mitotic spindle assembly checkpoint protein MAD1-like (LOC105605963), transcript variant X4, mRNA	Q9Y6D9	1007	0	Mitotic spindle assembly checkpoint protein MAD1	.	.	.	.	pfam05557	MAD	Mitotic checkpoint protein. This family consists of several eukaryotic mitotic checkpoint (Mitotic arrest deficient or MAD)	GO:0051315|attachment of mitotic spindle microtubules to kinetochore;GO:0051301|cell division;GO:0007093|mitotic cell cycle checkpoint;GO:0007094|mitotic spindle assembly checkpoint;GO:0042130|negative regulation of T cell proliferation;GO:0090235|regulation of metaphase plate congression;GO:0007062|sister chromatid cohesion;GO:0048538|thymus development;	GO:0015629|actin cytoskeleton;GO:0005813|centrosome;GO:0000777|condensed chromosome kinetochore;GO:0005737|cytoplasm;GO:00	.	K06638	MAD1L; mitotic spindle assembly checkpoint protein MAD1
RPL27	4044.329	-0.35032	0.132238092	-2.64915	0.0080694	0.04449	Not_Sig	no	gi|528768520|gb|EPY88179.1|	644	5e-83	60S ribosomal protein L27-like protein [Camelus ferus]	gi|742135020|ref|XM_010842477.1|	518	0	PREDICTED: Bison bison bison ribosomal protein L27 (RPL27), mRNA	P61354	249	1e-83	60S ribosomal protein L27	YDR471w	149	4.25e-46	COG2163	pfam01777	Ribosomal_L27e	Ribosomal L27e protein family. The N-terminal region of the eukaryotic ribosomal L27 has the KOW motif. C-terminal region is	GO:1904044|response to aldosterone;GO:0006412|translation;	GO:0022625|cytosolic large ribosomal subunit;GO:0005840|ribosome;	GO:0003735|structural constituent of ribosome;	K02901	RP-L27e, RPL27; large subunit ribosomal protein L27e
SMC5	602.6829	-0.3685	0.139149504	-2.64825	0.0080909	0.044587	Not_Sig	no	gi|555971036|ref|XP_005897535.1|	5532	0	PREDICTED: structural maintenance of chromosomes protein 5 [Bos mutus]	gi|942059071|ref|XM_005897473.2|	5569	0	PREDICTED: Bos mutus structural maintenance of chromosomes 5 (SMC5), mRNA	Q8IY18	1971	0	Structural maintenance of chromosomes protein 5	YOL034w	313	2.20e-87	COG1196	pfam02463	SMC_N	RecF/RecN/SMC N terminal domain. This domain is found at the N terminus of SMC proteins. The SMC (structural maintenance	GO:0051301|cell division;GO:0006974|cellular response to DNA damage stimulus;GO:0090398|cellular senescence;GO:0000724|double-strand break repair via homologous recombination;GO:0007067|mitotic nuclear division;GO:0051984|positive regulation of chromosome segregation;GO:0034184|positive regulation of maintenance of mitotic sister chromatid cohesion;GO:0016925|protein sumoylation;GO:0071139|resolution of recombination intermediates;GO:0007062|sister chromatid cohesion;GO:0000722|telomere	GO:0030054|cell junction;GO:0000781|chromosome, telomeric region;GO:0035061|interchromatin	GO:0005524|ATP binding;	.	.
RANBP6	440.905	-0.37039	0.139882424	-2.64785	0.0081006	0.044619	Not_Sig	no	gi|296484804|tpg|DAA26919.1|	5796	0	TPA: karyopherin beta 3-like [Bos taurus]	gi|555958593|ref|XM_005891352.1|	4612	0	PREDICTED: Bos mutus RAN binding protein 6 (RANBP6), transcript variant X1, mRNA	O60518	2191	0	Ran-binding protein 6	.	.	.	.	pfam13646	HEAT_2	HEAT repeats. This family includes multiple HEAT repeats.	GO:0006607|NLS-bearing protein import into nucleus;GO:0000059|protein import into nucleus, docking;GO:0000060|protein import into nucleus, translocation;GO:0006610|ribosomal protein import into nucleus;	GO:0005737|cytoplasm;GO:0031965|nuclear membrane;GO:0034399|nuclear periphery;	GO:0008139|nuclear localization sequence binding;GO:0008565	.	.
DCAKD	186.7982	0.435319	0.164420121	2.647599	0.0081066	0.044629	Not_Sig	no	gi|556777345|ref|XP_005983762.1|	1442	0	PREDICTED: dephospho-CoA kinase domain-containing protein isoform X1 [Pantholops hodgsonii]	gi|742086573|ref|XM_010832419.1|	1590	0	PREDICTED: Bison bison bison dephospho-CoA kinase domain containing (DCAKD), mRNA	Q3ZBS0	470	4e-162	Dephospho-CoA kinase domain-containing protein	BS_ytaG	181	1.33e-52	COG0237	pfam01121	CoaE	Dephospho-CoA kinase. This family catalyses the phosphorylation of the 3'-hydroxyl group of dephosphocoenzyme A to form	GO:0015937|coenzyme A biosynthetic process;	GO:0016020|membrane;GO:0005739|mitochondrion;	GO:0005524|ATP binding;GO:0004140|dephospho-CoA kinase activity;	.	.
FBXO28	816.1161	-0.35055	0.132412443	-2.64738	0.0081119	0.044629	Not_Sig	no	gi|114051750|ref|NP_001039890.1|	1420	9e-178	F-box only protein 28 [Bos taurus]	gi|742174843|ref|XM_010852845.1|	5317	0	PREDICTED: Bison bison bison F-box protein 28 (FBXO28), mRNA	Q2NL16	551	3e-179	F-box only protein 28	.	.	.	.	pfam00646	F-box	F-box domain. This domain is approximately 50 amino acids long, and is usually found in the N-terminal half of a variety of	.	GO:0000777|condensed chromosome kinetochore;GO:0000776|kinetochore;	.	K10306	FBXO28; F-box protein 28
SMARCD2	704.8367	0.296923	0.112161386	2.647286	0.0081141	0.044629	Not_Sig	no	gi|291406363|ref|XP_002719524.1|	2262	0	PREDICTED: SWI/SNF-related matrix-associated actin-dependent regulator of chromatin subfamily D member 2 [Oryctolagus cuniculus]	gi|999808230|ref|NM_001205462.2|	2761	0	Bos taurus SWI/SNF related, matrix associated, actin dependent regulator of chromatin, subfamily d, member 2 (SMARCD2), mRNA	E1BJD1	873	0	SWI/SNF-related matrix-associated actin-dependent regulator of chromatin subfamily D	SPAC23G3.10c	222	2.53e-63	COG5531	pfam02201	SWIB	SWIB/MDM2 domain. This family includes the SWIB domain and the MDM2 domain. The p53-associated protein (MDM2) is an	GO:0043044|ATP-dependent chromatin remodeling;GO:0016569|covalent chromatin modification;GO:0006337|nucleosome disassembly;GO:0006355|regulation of transcription, DNA-templated;GO:0006351|transcription, DNA-templated;	GO:0000790|nuclear chromatin;GO:0005654|nucleoplasm;GO:0016514|SWI/SNF complex;	GO:0000978|RNA polymerase II core promoter proximal region sequence-	K11650	SMARCD; SWI/SNF-related matrix-associated actin-dependent regulator
MFSD1	822.4302	-0.3056	0.115448979	-2.64704	0.0081201	0.044641	Not_Sig	no	gi|440896920|gb|ELR48713.1|	2598	0	Major facilitator superfamily domain-containing protein 1, partial [Bos mutus]	gi|942086201|ref|XM_005906242.2|	2220	0	PREDICTED: Bos mutus major facilitator superfamily domain containing 1 (MFSD1), transcript variant X1, mRNA	Q1JQC1	926	0	Major facilitator superfamily domain-containing protein 1	ECU10g1410	92	1.18e-18	COG0477	pfam07690	MFS_1	Major Facilitator Superfamily.	GO:0055085|transmembrane transport;	GO:0016021|integral component of membrane;	.	.	.
SLC41A2	325.1145	-0.42755	0.161556211	-2.64644	0.0081344	0.044697	Not_Sig	no	gi|555974271|ref|XP_005899129.1|	2699	0	PREDICTED: solute carrier family 41 member 2 [Bos mutus]	gi|742176818|ref|XM_010853400.1|	5185	0	PREDICTED: Bison bison bison solute carrier family 41 (magnesium transporter), member 2 (SLC41A2), transcript variant X5, mRNA	Q4R335	1003	0	Solute carrier family 41 member 2	MA1437	74.7	5.42e-14	COG1824	pfam01769	MgtE	Divalent cation transporter. This region is the integral membrane part of the eubacterial MgtE family of magnesium transporters.	.	GO:0016021|integral component of membrane;	GO:0008324|cation transmembrane transporter activity;	K15122	SLC41A; solute carrier family 41
SERPINE1	100733.1	0.298625	0.112850622	2.646201	0.0081401	0.044697	Not_Sig	no	gi|555961536|ref|XP_005892862.1|	2086	0	PREDICTED: plasminogen activator inhibitor 1 isoform X2 [Bos mutus]	gi|742087314|ref|XM_010832592.1|	1511	0	PREDICTED: Bison bison bison serpin peptidase inhibitor, clade E (nexin, plasminogen activator inhibitor type 1), member 1 (SERPINE1), mRNA	P13909	805	0	Plasminogen activator inhibitor 1	MA2246	166	2.65e-45	COG4826	pfam00079	Serpin	Serpin (serine protease inhibitor). Structure is a multi-domain fold containing a bundle of helices and a beta sandwich.	GO:0001525|angiogenesis;GO:0071222|cellular response to lipopolysaccharide;GO:0001300|chronological cell aging;GO:0050829|defense response to Gram-negative bacterium;GO:0033629|negative regulation of cell adhesion mediated by integrin;GO:2000352|negative regulation of endothelial cell apoptotic process;GO:1902042|negative regulation of extrinsic apoptotic signaling pathway via death domain receptors;GO:0051918|negative regulation of fibrinolysis;GO:0010757|negative regulation of plasminogen activation;GO:0014912|negative	GO:0070062|extracellular exosome;GO:0031012|extracellular matrix;GO:0005615|extracellular space;	GO:0004857|enzyme inhibitor activity;GO:0004867|serine-type	K03982	SERPINE1, PAI1; plasminogen activator inhibitor-1
DHX37	427.2735	0.384708	0.145389556	2.646049	0.0081438	0.044697	Not_Sig	no	gi|329664328|ref|NP_001192890.1|	5154	0	probable ATP-dependent RNA helicase DHX37 [Bos taurus]	gi|942063412|ref|XM_005898818.2|	3922	0	PREDICTED: Bos mutus DEAH (Asp-Glu-Ala-His) box polypeptide 37 (DHX37), mRNA	Q8IY37	1555	0	Probable ATP-dependent RNA helicase DHX37	YMR128w	327	5.48e-92	COG1643	pfam07717	OB_NTP_bind	Oligonucleotide/oligosaccharide-binding (OB)-fold. This family is found towards the C-terminus of the DEAD-box helicases	GO:0006396|RNA processing;GO:0006364|rRNA processing;	GO:0005737|cytoplasm;GO:0005730|nucleolus;GO:0005654|nucleoplasm;	GO:0005524|ATP binding;GO:0004004|ATP-dependent RNA helicase	K14780	DHX37, DHR1; ATP-dependent RNA helicase DHX37/DHR1
DAD1	1270.487	-0.2781	0.105102951	-2.64597	0.0081458	0.044697	Not_Sig	no	gi|528767963|gb|EPY87622.1|	537	1e-64	hypothetical protein CB1_000233026 [Camelus ferus]	gi|942053737|ref|XM_005895466.2|	724	0	PREDICTED: Bos mutus defender against cell death 1 (DAD1), transcript variant X2, mRNA	Q5E9C2	198	1e-62	Dolichyl-diphosphooligosaccharide--protein glycosyltransferase	.	.	.	.	pfam02109	DAD	DAD family. Members of this family are thought to be integral membrane proteins. Some members of this family have been	GO:0006915|apoptotic process;GO:0001824|blastocyst development;GO:0043066|negative regulation of apoptotic process;GO:0006486|protein glycosylation;	GO:0070062|extracellular exosome;GO:0016021|integral component of membrane;GO:0008250|oligosaccharyltrans	GO:0004579|dolichyl-diphosphooligosaccharide-protein	K12668	OST2, DAD1; oligosaccharyltransferase complex subunit epsilon
SLC29A1	14057.32	0.332871	0.125833037	2.645343	0.0081608	0.044758	Not_Sig	no	gi|346716283|ref|NP_001029570.2|	2386	0	equilibrative nucleoside transporter 1 [Bos taurus]	gi|741970463|ref|XM_010818176.1|	2122	0	PREDICTED: Bos taurus solute carrier family 29 (equilibrative nucleoside transporter), member 1 (SLC29A1), transcript variant X2, mRNA	Q99808	679	0	Equilibrative nucleoside transporter 1	.	.	.	.	pfam01733	Nucleoside_tran	Nucleoside transporter. This is a family of nucleoside transporters. In mammalian cells nucleoside transporters transport nucleoside	GO:0071333|cellular response to glucose stimulus;GO:0071456|cellular response to hypoxia;GO:0060079|excitatory postsynaptic potential;GO:0007595|lactation;GO:0006139|nucleobase-containing compound metabolic process;GO:0015858|nucleoside transport;GO:0030431|sleep;GO:0015862|uridine transport;	GO:0016324|apical plasma membrane;GO:0016323|basolateral plasma membrane;GO:0005887|integral component of plasma	GO:0005337|nucleoside transmembrane transporter activity;	K15014	SLC29A1_2_3, ENT1_2_3; solute carrier family 29 (equilibrative
PHF6	811.6548	-0.42688	0.161469931	-2.64369	0.0082008	0.044921	Not_Sig	no	gi|655878008|ref|XP_008271449.1|	1603	0	PREDICTED: PHD finger protein 6 isoform X1 [Oryctolagus cuniculus]	gi|555999065|ref|XM_005911177.1|	4760	0	PREDICTED: Bos mutus PHD finger protein 6 (PHF6), transcript variant X3, mRNA	Q08DR0	615	0	PHD finger protein 6	.	.	.	.	pfam13771	zf-HC5HC2H	PHD-like zinc-binding domain. The members of this family are annotated as containing PHD domain, but the zinc-binding	GO:0006355|regulation of transcription, DNA-templated;GO:0006351|transcription, DNA-templated;	GO:0000777|condensed chromosome kinetochore;GO:0005730|nucleolus;	GO:0003677|DNA binding;GO:0008270|zinc ion binding;	.	.
ATP5PO	1225.446	-0.32073	0.121319906	-2.64368	0.008201	0.044921	Not_Sig	no	gi|555962652|ref|XP_005893414.1|	1104	2e-150	PREDICTED: ATP synthase subunit O, mitochondrial isoform X1 [Bos mutus]	gi|942048226|ref|XM_005893352.2|	778	0	PREDICTED: Bos mutus ATP synthase, H+ transporting, mitochondrial F1 complex, O subunit (ATP5O), transcript variant X1, mRNA	P13621	425	4e-150	ATP synthase subunit O, mitochondrial	SPCC1840.06	122	1.44e-33	COG0712	pfam00213	OSCP	ATP synthase delta (OSCP) subunit. The ATP D subunit from Escherichia coli is the same as the OSCP subunit which is this family.	GO:0015986|ATP synthesis coupled proton transport;	GO:0005753|mitochondrial proton-transporting ATP synthase complex;GO:0043209|myelin sheath;	GO:0046933|proton-transporting ATP synthase activity, rotational	K02137	ATPeF0O, ATP5O, ATP5; F-type H+-transporting ATPase subunit O
TC2N	2146.912	-0.30675	0.116035377	-2.64362	0.0082024	0.044921	Not_Sig	no	gi|555997171|ref|XP_005910313.1|	2426	0	PREDICTED: tandem C2 domains nuclear protein isoform X2 [Bos mutus]	gi|742102314|ref|XM_010836183.1|	3982	0	PREDICTED: Bison bison bison tandem C2 domains, nuclear (TC2N), transcript variant X1, mRNA	Q8N9U0	862	0	Tandem C2 domains nuclear protein	.	.	.	.	pfam00168	C2	C2 domain.	GO:0030193|regulation of blood coagulation;GO:0010468|regulation of gene expression;	GO:0005634|nucleus;GO:0005886|plasma membrane;	GO:0005509|calcium ion binding;	K17287	TC2N; tandem C2 domains nuclear protein
NUDT17	288.6045	0.409171	0.154800212	2.643219	0.0082122	0.044924	Not_Sig	no	gi|556724622|ref|XP_005958107.1|	1353	0	PREDICTED: nucleoside diphosphate-linked moiety X motif 17 [Pantholops hodgsonii]	gi|942061866|ref|XM_005898513.2|	1029	0	PREDICTED: Bos mutus nudix (nucleoside diphosphate linked moiety X)-type motif 17 (NUDT17), mRNA	A4FUG7	516	0	Nucleoside diphosphate-linked moiety X motif 17	.	.	.	.	pfam00293	NUDIX	NUDIX domain.	.	.	GO:0016787|hydrolase activity;GO:0046872|metal ion binding;	.	.
TMEM176B	537.802	-0.32147	0.121620734	-2.64321	0.0082123	0.044924	Not_Sig	no	gi|982922318|ref|XP_010802885.2|	1382	0	PREDICTED: transmembrane protein 176B isoform X1 [Bos taurus]	gi|742191503|ref|XM_010857254.1|	1251	0	PREDICTED: Bison bison bison transmembrane protein 176B (TMEM176B), transcript variant X2, mRNA	Q5R8D6	301	1e-94	Transmembrane protein 176B	.	.	.	.	pfam04103	CD20	CD20-like family. This family includes the CD20 protein and the beta subunit of the high affinity receptor for IgE Fc. The high	GO:0030154|cell differentiation;	GO:0016021|integral component of membrane;GO:0031965|nuclear membrane;	.	.	.
HSD17B11	98.49972	-0.55467	0.209854644	-2.64312	0.0082145	0.044924	Not_Sig	no	gi|555985317|ref|XP_005904537.1|	1563	0	PREDICTED: estradiol 17-beta-dehydrogenase 11 isoform X1 [Bos mutus]	gi|942080852|ref|XM_005904475.2|	1670	0	PREDICTED: Bos mutus hydroxysteroid (17-beta) dehydrogenase 11 (HSD17B11), transcript variant X1, mRNA	Q5NVG2	520	0	Estradiol 17-beta-dehydrogenase 11	.	.	.	.	pfam00106	adh_short	short chain dehydrogenase. This family contains a wide variety of dehydrogenases.	GO:0006694|steroid biosynthetic process;	GO:0005576|extracellular region;	GO:0004303|estradiol 17-beta-dehydrogenase activity;	.	.
ARL15	149.7839	-0.50041	0.189428287	-2.6417	0.0082491	0.045092	Not_Sig	no	gi|148686435|gb|EDL18382.1|	1103	6e-139	ADP-ribosylation factor-like 15, isoform CRA_a, partial [Mus musculus]	gi|742191161|ref|XM_010857165.1|	3255	0	PREDICTED: Bison bison bison ADP-ribosylation factor-like 15 (ARL15), mRNA	Q9NXU5	416	5e-136	ADP-ribosylation factor-like protein 15	YBR164c	105	4.33e-25	COG1100	pfam00025	Arf	ADP-ribosylation factor family. Pfam combines a number of different Prosite families together	GO:0007264|small GTPase mediated signal transduction;	GO:0070062|extracellular exosome;GO:0005622|intracellular;	GO:0005525|GTP binding;	K17201	ARL15; ADP-ribosylation factor-like protein 15
SLC35A1	328.5363	-0.36434	0.137941491	-2.64126	0.0082597	0.045128	Not_Sig	no	gi|743707771|ref|XP_010947256.1|	1769	0	PREDICTED: CMP-sialic acid transporter isoform X1 [Camelus bactrianus]	gi|555982086|ref|XM_005902890.1|	1773	0	PREDICTED: Bos mutus solute carrier family 35 (CMP-sialic acid transporter), member A1 (SLC35A1), transcript variant X1, mRNA	P78382	646	0	CMP-sialic acid transporter	SPCC1795.03	215	1.19e-63	COG0697	pfam04142	Nuc_sug_transp	Nucleotide-sugar transporter. This family of membrane proteins transport nucleotide sugars from the cytoplasm into golgi vesicles.	GO:0005975|carbohydrate metabolic process;GO:0006464|cellular protein modification process;GO:0015782|CMP-N-acetylneuraminate transport;	GO:0005794|Golgi apparatus;GO:0000139|Golgi membrane;GO:0005887|integral component of plasma membrane;	GO:0005456|CMP-N-acetylneuraminate transmembrane	K15272	SLC35A1_2_3; solute carrier family 35 (UDP-sugar transporter),
TRAPPC14	257.9979	0.513902	0.194592007	2.64092	0.0082681	0.045152	Not_Sig	no	gi|926724353|ref|XP_005698142.2|	2418	0	PREDICTED: uncharacterized protein C7orf43 homolog [Capra hircus]	gi|983007287|ref|XM_589329.9|	2535	0	PREDICTED: Bos taurus chromosome 25 open reading frame, human C7orf43 (C25H7orf43), mRNA	Q3UTZ3	932	0	Uncharacterized protein C7orf43 homolog	.	.	.	.	pfam07919	Gryzun	Gryzun, putative trafficking through Golgi. The proteins featured in this family are all eukaryotic, and many of them are	.	.	.	.	.
KANK1	991.6566	0.275009	0.104144172	2.640659	0.0082745	0.045166	Not_Sig	no	gi|296484796|tpg|DAA26911.1|	6468	0	TPA: KIAA0172-like [Bos taurus]	gi|742205645|ref|XM_010860683.1|	5379	0	PREDICTED: Bison bison bison KN motif and ankyrin repeat domains 1 (KANK1), transcript variant X4, mRNA	Q14678	2143	0	KN motif and ankyrin repeat domain-containing protein 1	all2748	85.9	2.30e-16	COG0666	pfam12075	KN_motif	KN motif. This small motif is found at the N-terminus of Kank proteins and has been called the KN (for Kank N-terminal) motif. This	GO:0030837|negative regulation of actin filament polymerization;GO:0030336|negative regulation of cell migration;GO:0046627|negative regulation of insulin receptor signaling pathway;GO:2000393|negative regulation of lamellipodium morphogenesis;GO:0010977|negative regulation of neuron projection development;GO:0035024|negative regulation of Rho protein signal transduction;GO:1900028|negative regulation of ruffle assembly;GO:1900025|negative regulation of substrate adhesion-dependent cell spreading;GO:0035413|positive	GO:0005737|cytoplasm;GO:0005634|nucleus;GO:0005886|plasma membrane;GO:0032587|ruffle membrane;	GO:0008013|beta-catenin binding;	.	.

LOC106701537	38.92139	-0.85404	0.323534252	-2.63972	0.0082975	0.04527	DOWN	yes	gi|958720315|ref|XP_014703348.1|	775	4e-102	PREDICTED: 40S ribosomal protein S13 [Equus asinus]	gi|982976425|ref|XM_010822974.2|	503	0	PREDICTED: Bos taurus 40S ribosomal protein S13-like (LOC504858), mRNA	P62278	295	3e-101	40S ribosomal protein S13	YDR064w	241	2.14e-82	COG0184	pfam08069	Ribosomal_S13_N	Ribosomal S13/S15 N-terminal domain. This domain is found at the N-terminus of ribosomal S13 and S15 proteins. This domain is	GO:0033119|negative regulation of RNA splicing;GO:0006412|translation;	GO:0022627|cytosolic small ribosomal subunit;GO:0070062|extracellular exosome;GO:0005925|focal adhesion;GO:0016020|membrane;GO:0005	GO:0003729|mRNA binding;GO:0070181|small ribosomal subunit rRNA	K02953	RP-S13e, RPS13; small subunit ribosomal protein S13e
AGPAT2	371.486	0.418922	0.158726751	2.639267	0.0083086	0.045309	Not_Sig	no	gi|122692545|ref|NP_001073733.1|	1295	1e-174	1-acyl-sn-glycerol-3-phosphate acyltransferase beta precursor [Bos taurus]	gi|122692544|ref|NM_001080264.1|	1387	0	Bos taurus 1-acylglycerol-3-phosphate O-acyltransferase 2 (AGPAT2), mRNA	O15120	415	1e-141	1-acyl-sn-glycerol-3-phosphate acyltransferase beta	SPAC1851.02	116	1.15e-28	COG0204	pfam01553	Acyltransferase	Acyltransferase. This family contains acyltransferases involved in phospholipid biosynthesis and other proteins of unknown	GO:0016024|CDP-diacylglycerol biosynthetic process;GO:0008544|epidermis development;GO:0006654|phosphatidic acid biosynthetic process;GO:0006644|phospholipid metabolic process;GO:0001819|positive regulation of cytokine production;GO:0001961|positive regulation of cytokine-mediated signaling pathway;GO:0019432|triglyceride biosynthetic process;	GO:0005783|endoplasmic reticulum;GO:0005789|endoplasmic reticulum membrane;GO:0016021|integral component of membrane;	GO:0003841|1-acylglycerol-3-phosphate O-acyltransferase	K13509	AGPAT1_2; lysophosphatidate acyltransferase [EC:2.3.1.51]
TNS4	96.1842	0.547728	0.20756853	2.638782	0.0083204	0.045352	Not_Sig	no	gi|555972191|ref|XP_005898103.1|	3225	0	PREDICTED: tensin-4 [Bos mutus]	gi|942060425|ref|XM_005898041.2|	3686	0	PREDICTED: Bos mutus tensin 4 (TNS4), mRNA	Q32PJ7	1234	0	Tensin-4	.	.	.	.	pfam08416	PTB	Phosphotyrosine-binding domain. The phosphotyrosine-binding domain (PTB, also phosphotyrosine-interaction or PI	GO:0006915|apoptotic process;	GO:0005737|cytoplasm;GO:0005856|cytoskeleton;GO:0005925|focal adhesion;	.	K18080	TNS; tensin
RC3H1	712.6487	-0.33958	0.128709896	-2.63831	0.0083321	0.045394	Not_Sig	no	gi|555957521|ref|XP_005890891.1|	5026	0	PREDICTED: roquin-1 isoform X1 [Bos mutus]	gi|742165108|ref|XM_010850082.1|	10957	0	PREDICTED: Bison bison bison ring finger and CCCH-type domains 1 (RC3H1), transcript variant X2, mRNA	Q5TC82	1888	0	Roquin-1	.	.	.	.	pfam13445	zf-RING_UBOX	RING-type zinc-finger. This zinc-finger is a typical RING-type of plant ubiquitin ligases.	GO:0061158|3'-UTR-mediated mRNA destabilization;GO:0001782|B cell homeostasis;GO:0071347|cellular response to interleukin-1;GO:0033962|cytoplasmic mRNA processing body assembly;GO:0048535|lymph node development;GO:0046007|negative regulation of activated T cell proliferation;GO:0030889|negative regulation of B cell proliferation;GO:0002635|negative regulation of germinal center formation;GO:0045623|negative regulation of T-helper cell differentiation;GO:0000956|nuclear-transcribed mRNA catabolic	GO:0000932|cytoplasmic mRNA processing body;GO:0010494|cytoplasmic stress granule;	GO:0003730|mRNA 3'-UTR binding;GO:0044822|poly(A) RNA	K15690	RC3H; RING finger and CCCH-type zinc finger domain-containing protein
TMEM150C	176.7232	-0.42056	0.159432497	-2.63788	0.0083426	0.04543	Not_Sig	no	gi|742150380|ref|XP_010844222.1|	1139	2e-144	PREDICTED: transmembrane protein 150C isoform X1 [Bison bison bison]	gi|942080422|ref|XM_005904343.2|	3028	0	PREDICTED: Bos mutus transmembrane protein 150C (TMEM150C), mRNA	A5D7C9	431	5e-142	Transmembrane protein 150C	.	.	.	.	pfam10277	Frag1	Frag1/DRAM/Sfk1 family. This family includes Frag1, DRAM and Sfk1 proteins. Frag1 (FGF receptor activating protein 1) is a protein	.	GO:0005887|integral component of plasma membrane;GO:0005765|lysosomal membrane;GO:0005764|lysosome;	.	.	.
SCAMP3	1112.65	0.320185	0.121405499	2.637315	0.0083565	0.045464	Not_Sig	no	gi|71649517|sp|Q58DR5.1|SCAM3_BOVIN	1240	5e-165	RecName: Full=Secretory carrier-associated membrane protein 3; Short=Secretory carrier membrane protein 3	gi|742111479|ref|XM_010838292.1|	1521	0	PREDICTED: Bison bison bison secretory carrier membrane protein 3 (SCAMP3), mRNA	Q58DR5	482	2e-166	Secretory carrier-associated membrane protein 3	.	.	.	.	pfam04144	SCAMP	SCAMP family. In vertebrates, secretory carrier membrane proteins (SCAMPs) 1-3 constitute a family of putative membrane-	GO:0015031|protein transport;	GO:0016021|integral component of membrane;	.	.	.
VAMP8	398.067	-0.33877	0.128455382	-2.63729	0.008357	0.045464	Not_Sig	no	gi|426223517|ref|XP_004005921.1|	402	5e-46	PREDICTED: vesicle-associated membrane protein 8 [Ovis aries]	gi|942043167|ref|XM_005891673.2|	745	0	PREDICTED: Bos mutus vesicle-associated membrane protein 8 (VAMP8), mRNA	Q3T0Y8	157	1e-46	Vesicle-associated membrane protein 8	SPAC6G9.11	52.8	3.06e-08	COG5143	pfam00957	Synaptobrevin	Synaptobrevin.	GO:0097352|autophagosome maturation;GO:0006887|exocytosis;GO:0070254|mucus secretion;GO:1903531|negative regulation of secretion by cell;GO:1903595|positive regulation of histamine secretion by mast cell;GO:0015031|protein transport;GO:1903076|regulation of protein localization to plasma membrane;GO:0006906|vesicle fusion;GO:0046718|viral entry into host cell;	GO:0035577|azurophil granule membrane;GO:0005829|cytosol;GO:0031901|early endosome membrane;GO:0070062|extracellular	GO:0019869|chloride channel inhibitor activity;GO:0005484|SNAP receptor	K08512	VAMP8; vesicle-associated membrane protein 8
PUS1	560.4812	0.365022	0.138415776	2.637142	0.0083608	0.045464	Not_Sig	no	gi|76638649|ref|XP_593895.2|	2163	0	PREDICTED: tRNA pseudouridine synthase A, mitochondrial isoform X1 [Bos taurus]	gi|942042038|ref|XM_005891210.2|	1711	0	PREDICTED: Bos mutus pseudouridylate synthase 1 (PUS1), mRNA	Q9Y606	679	0	tRNA pseudouridine synthase A, mitochondrial	YGL063w	143	4.04e-37	COG0101	pfam01416	PseudoU_synth_1	tRNA pseudouridine synthase. Involved in the formation of pseudouridine at the anticodon stem and loop of transfer-RNAs	GO:0070902|mitochondrial tRNA pseudouridine synthesis;	GO:0005759|mitochondrial matrix;GO:0005739|mitochondrion;GO:0005634|nucleus;	GO:0044822|poly(A) RNA binding;GO:0009982|pseudouridine	K06173	truA, PUS1; tRNA pseudouridine38-40 synthase [EC:5.4.99.12]
[truncated: 8,550,072 more chars]
